# Repeated and Widespread Evolution of Bioluminescence in Marine Fishes

**DOI:** 10.1371/journal.pone.0155154

**Published:** 2016-06-08

**Authors:** Matthew P. Davis, John S. Sparks, W. Leo Smith

**Affiliations:** 1 St. Cloud State University, St. Cloud, MN 56301, United States of America; 2 American Museum of Natural History, New York, NY 10024, United States of America; 3 University of Kansas, Lawrence, KS 66045, United States of America; The Evergreen State College, UNITED STATES

## Abstract

Bioluminescence is primarily a marine phenomenon with 80% of metazoan bioluminescent genera occurring in the world’s oceans. Here we show that bioluminescence has evolved repeatedly and is phylogenetically widespread across ray-finned fishes. We recover 27 independent evolutionary events of bioluminescence, all among marine fish lineages. This finding indicates that bioluminescence has evolved many more times than previously hypothesized across fishes and the tree of life. Our exploration of the macroevolutionary patterns of bioluminescent lineages indicates that the present day diversity of some inshore and deep-sea bioluminescent fish lineages that use bioluminescence for communication, feeding, and reproduction exhibit exceptional species richness given clade age. We show that exceptional species richness occurs particularly in deep-sea fishes with intrinsic bioluminescent systems and both shallow water and deep-sea lineages with luminescent systems used for communication.

## Introduction

Bioluminescence, the production and emission of light from a living organism, is a fascinating phenomenon that is documented in over 700 genera of metazoans across the tree of life, with the vast majority living in the ocean [1–3]. Among vertebrates, bioluminescence has evolved in cartilaginous (Chondrichthyes) [1–4] and ray-finned fishes (Actinopterygii) [1–3], and it is not observed in any lobe-finned fishes or tetrapods (Sarcopterygii). Previous survey studies [1–2] have identified bioluminescence in 11 orders of marine fishes; however, the phylogeny and classification of fishes has changed considerably since these previous studies, and the authors of these earlier studies did not investigate this phenomenon in a phylogenetic framework, identify independent evolutionary events of bioluminescence, or explore macroevolutionary patterns of bioluminescent lineages. Broad studies of bioluminescence have typically counted fishes as a single evolutionary event among the 40 independent higher-level evolutionary events of bioluminescence documented across the tree of life [2–3]; therefore, a focused study of the bioluminescent ray-finned fishes is critical to determine the number and identity of bioluminescent fish clades.

Bioluminescence is produced in living organisms following a chemical reaction between a substrate (luciferin) and an enzyme (luciferase) that results in a visible photon [2–3]. Among fishes, bioluminescence is generated intrinsically (e.g., stomiiform dragonfish barbels and photophores) [1–3, 5] or through bacterially mediated symbiosis (e.g., leiognathid [ponyfish] esophageal pouches, anomalopid [flashlightfish] subocular organs) [6–7]. The functions of bioluminescence are diverse and engrossing, exemplified by remarkable morphological specializations that range from anatomically complex species-specific luminescent structures to variation in the biochemistry of the bioluminescent systems themselves [1–13]. In ray-finned fishes, bioluminescent structures are variously used for camouflage, defense, predation, and communication [1–4, 7–11].

Here we present the first investigation of the evolution and distribution of bioluminescence across ray-finned fishes in a phylogenetic context. Recent work indicates that bioluminescence evolved once or twice within chondrichthyans (e.g., Etmopteridae and Dalatiidae) [4, 14]; however, the phenomenon is considerably more widespread, anatomically variable and complex, and biochemically diverse in ray-finned fishes [1–13]. Our objectives in this study were to determine the number of independent evolutionary origins of bioluminescence in ray-finned fishes, infer the ages of the phenomenon across this assemblage, and investigate patterns of diversification in bioluminescent lineages. Previous studies have suggested that bioluminescence may play a role in diversification within marine environments, particularly in deep-sea lineages, and specifically among taxa that are hypothesized to use bioluminescence for communication [8]. We further examine whether any bioluminescent lineages of ray-finned fishes exhibit exceptional species richness given their clade age for taxa living both in the deep sea, where there are few obvious physical barriers to reproduction, and shallow water habitats, to provide a roadmap for future macroevolutionary work.

## Materials and Methods

To investigate the evolution of bioluminescence across ray-finned fishes, we inferred a phylogeny from ten nuclear (enc1, Glyt, myh6, plagl2, Ptr, rag1, SH3PX3, sreb2, tbr1, zic1) and one mitochondrial (COI) gene fragments. Taxonomic sampling includes 301 taxa (297 genera, S1 Table). The data matrix is 80% complete and includes 274 newly collected gene fragments (S1 Table, GenBank KX227793-KX228066, with sequences aligned with MAFFT [15]). The previously published nuclear genes were obtained from a diversity of studies, as described in the data accessibility section. GenBank accession information is available for mitochondrial gene fragment cytochrome oxidase I in S2 Table, as data for this gene fragment were taken from various sources.

Evolutionary relationships were inferred using maximum likelihood in GARLI v2.01 [16] with 33 partitions (one for each codon position in each gene). Bootstrap values supporting clades are indicated in S1 Fig following the recommendation of Wiley et al. [17]. Codon positions were assigned models of nucleotide substitution from Akaike information criterion tests. Models of molecular evolution were identified by the program jModelTest v.2.1 [18] with the best fitting model under the Akaike information criterion (AIC): cytochrome oxidase I (GTR+Γ, GTR+I+Γ, GTR+Γ), ectodermal-neural cortex 1-like gene (GTR+Γ, GTR+I+Γ, GTR+I+Γ), glycosyltransferase (GTR+I+Γ, HKY+I+Γ, GTR+I+Γ), myosin heavy chain 6 alpha (GTR+I+Γ, GTR+I+Γ, GTR+Γ), pleiomorphic adenoma gene-like 2 gene (GTR+I+Γ, GTR+Γ, GTR+I+Γ), ptr hypothetical protein (GTR+I+Γ, GTR+I+Γ, GTR+I+Γ), recombination activating gene 1 (GTR+I+Γ, GTR+I+Γ, GTR+I+Γ), SH3 and PX3 domain-containing 3-like protein gene (GTR+I+Γ, GTR+I+Γ, GTR+I+Γ), brain super conserved receptor gene (GTR+I+Γ, GTR+I+Γ, GTR+I+Γ), T-box brain 1 gene (GTR+Γ, GTR+I+Γ, GTR+I+Γ), and zic family member protein (GTR+I+Γ, GTR+Γ, GTR+I+Γ).

**Fig 1:**
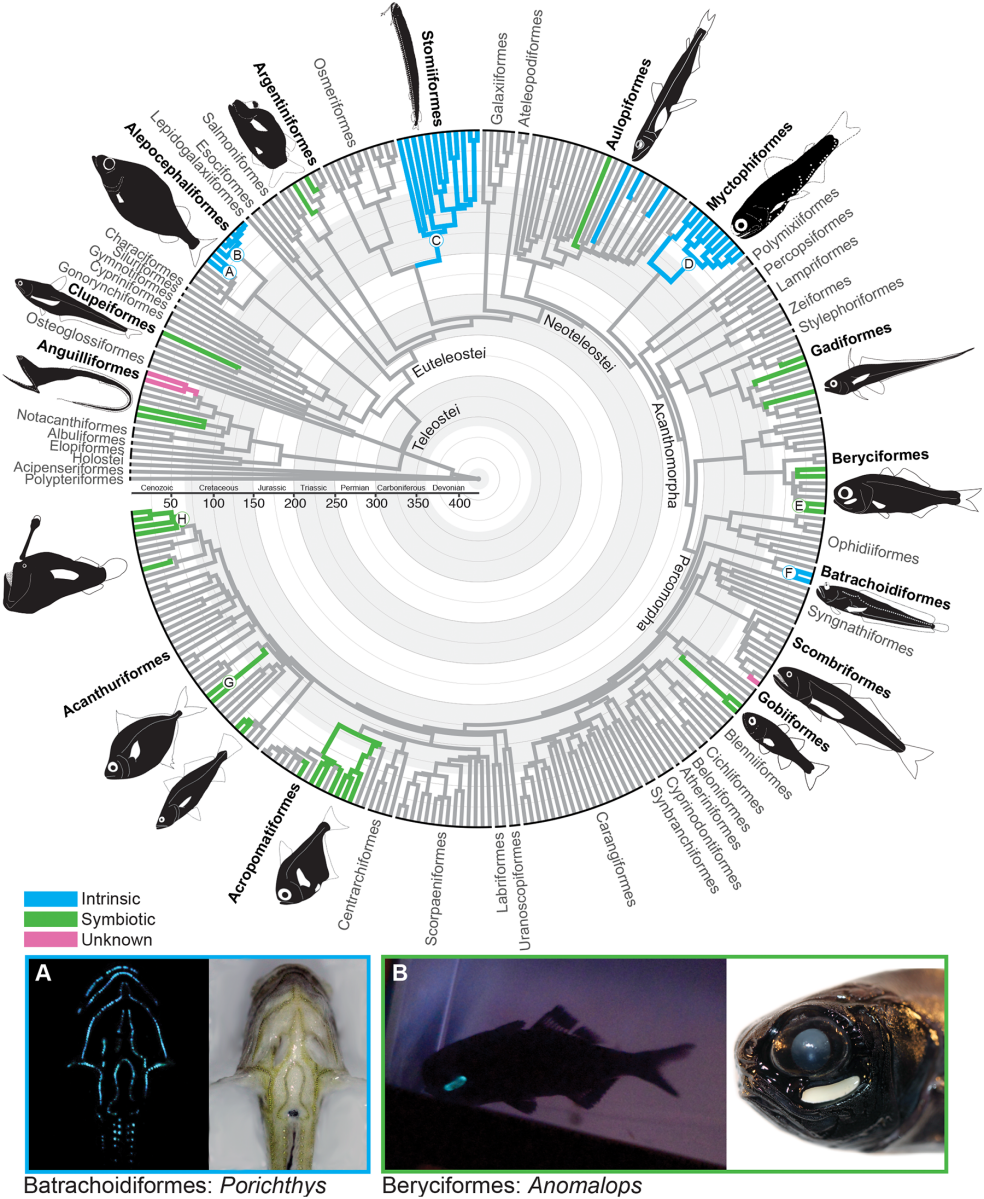
Evolution of Bioluminescence across Ray-Finned Fishes. Evolutionary relationships and divergence times of ray-finned fishes inferred from 11 gene fragments. Letters at nodes correspond to clades indicated in Fig 4. Branch colors indicate the presence of bioluminescence and whether the mechanism of bioluminescence is intrinsic, bacterially mediated, or unknown. Examples of bioluminescent ray-finned fishes include the A: midshipman (*Porichthys*: intrinsic), and B: flashlight fish (*Anomalops*: bacterially mediated).

**Fig 2:**
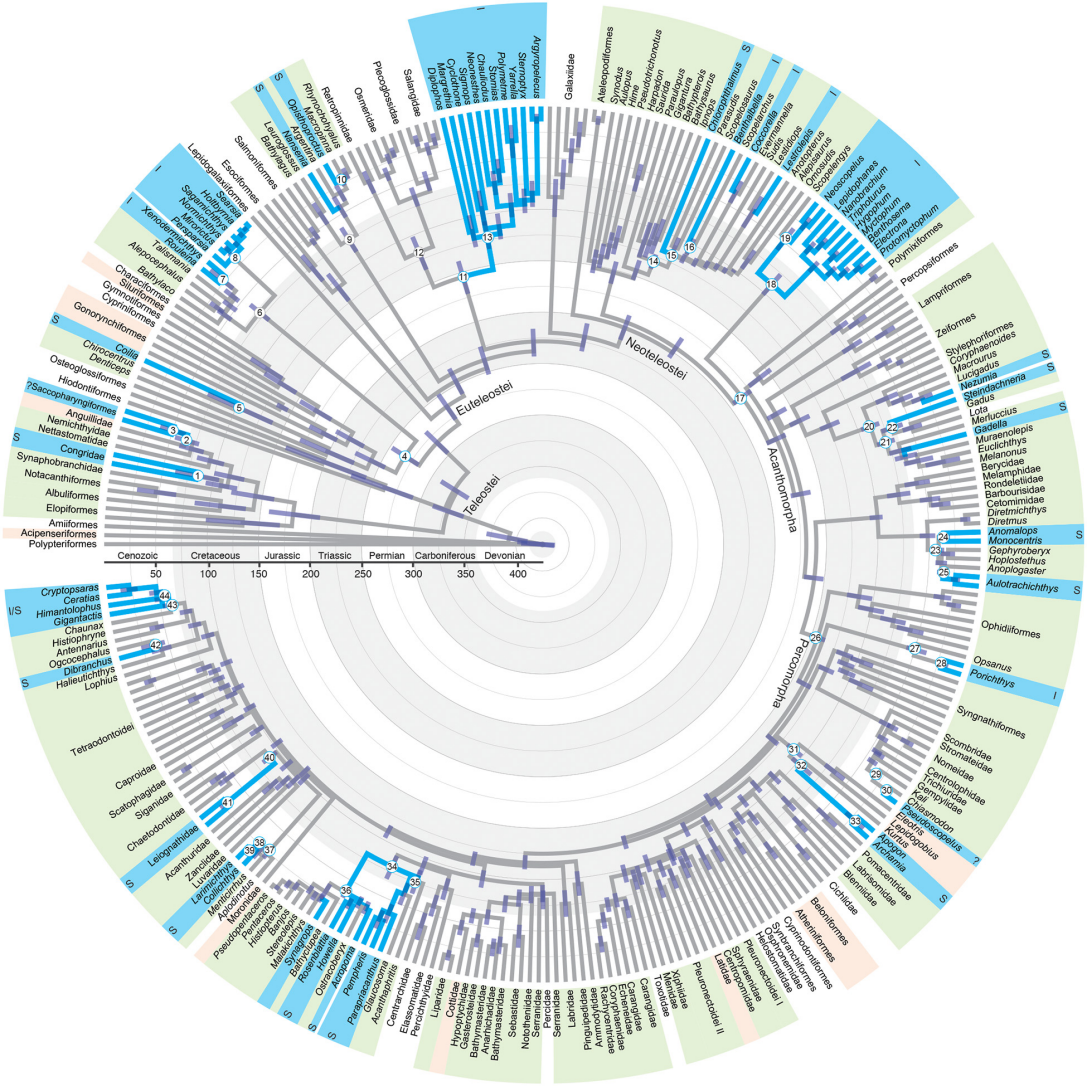
Evolutionary Relationships and Divergence Times of Ray-Finned Fishes Inferred from Eleven Gene Fragments. Numbers at nodes correspond to ancestral-character-state-reconstruction distributions of the evolution of bioluminescence indicated in Fig 3. Blue branches and taxa labels indicate the presence of bioluminescence, all of which occur in marine habitats. Green taxa labels indicate additional marine taxa. Pink labels indicate lineages with marine and freshwater taxa, and white labels indicate lineages that are predominantly found in freshwater habitats.

Five independent likelihood analyses were conducted, and the tree with the maximum likelihood score was stored and used as a fixed-topology prior to generate a distribution of temporal (ultrametric) trees for character evolution analyses in BEAST v.1.8 [19]. The relative divergence times of representative fishes were estimated by incorporating 21 previously published fossil calibrations [20–21] with lognormal priors (S1 Text, S1 Fig) and builds heavily on previous phylogenetic work [20]. Parameters and tree topologies from BEAST analyses converged on a stationary distribution. A 50% maximum clade credibility (mean heights) tree was generated from the posterior tree distribution and was subsampled down from 45,000 to 5,000 trees (Figs 1 and 2).

**Fig 3:**
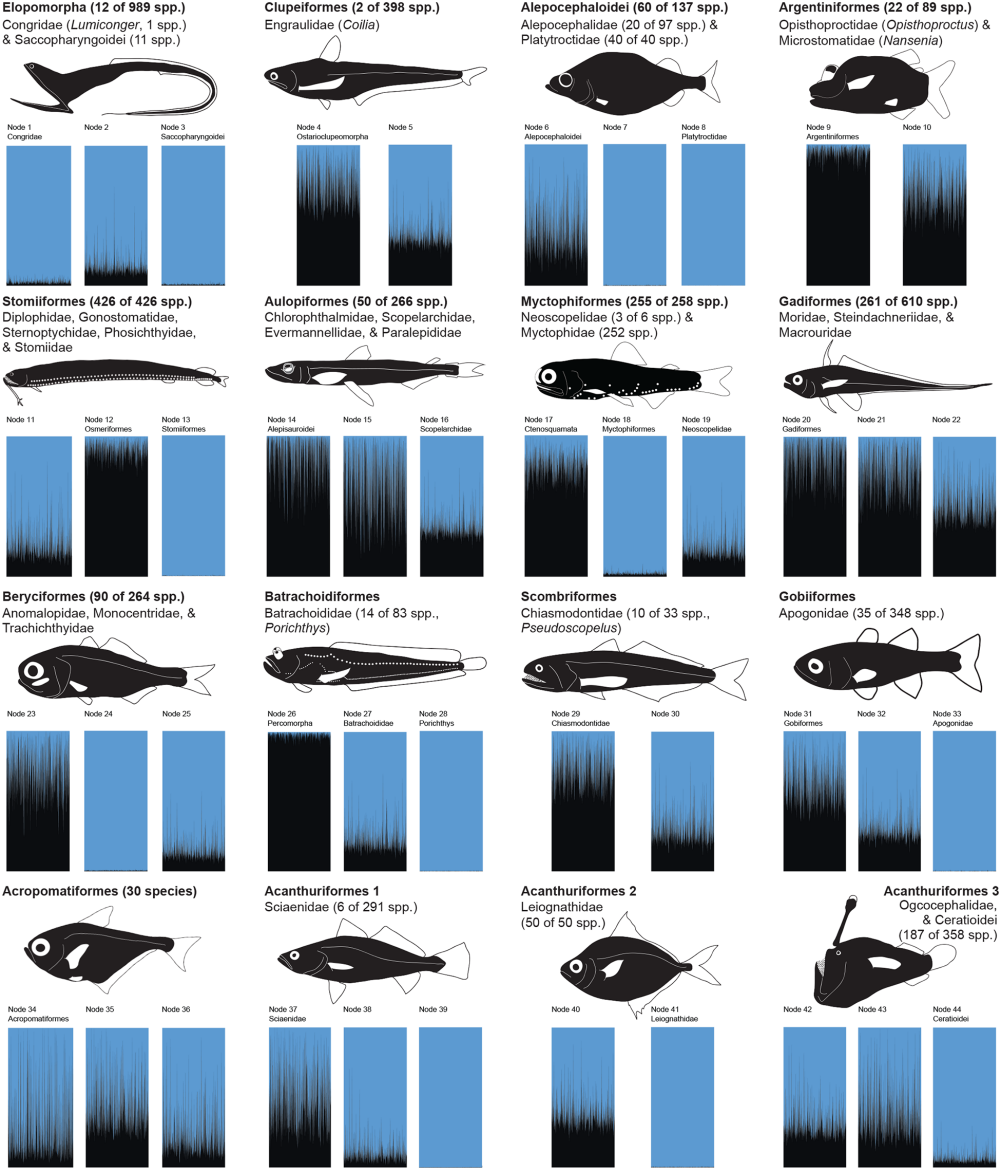
Ancestral-Character Evolution of Bioluminescence in Sixteen Major Lineages of Fishes. Bayesian ancestral-character-states reconstruction of bioluminescence across a distribution of 500 trees that resulted from the Bayesian inference of divergence times. Each rectangle includes 500 individual reconstructions across this distribution of 500 trees. Blue indicates the presence of bioluminescence and black indicates absence.

**Fig 4:**
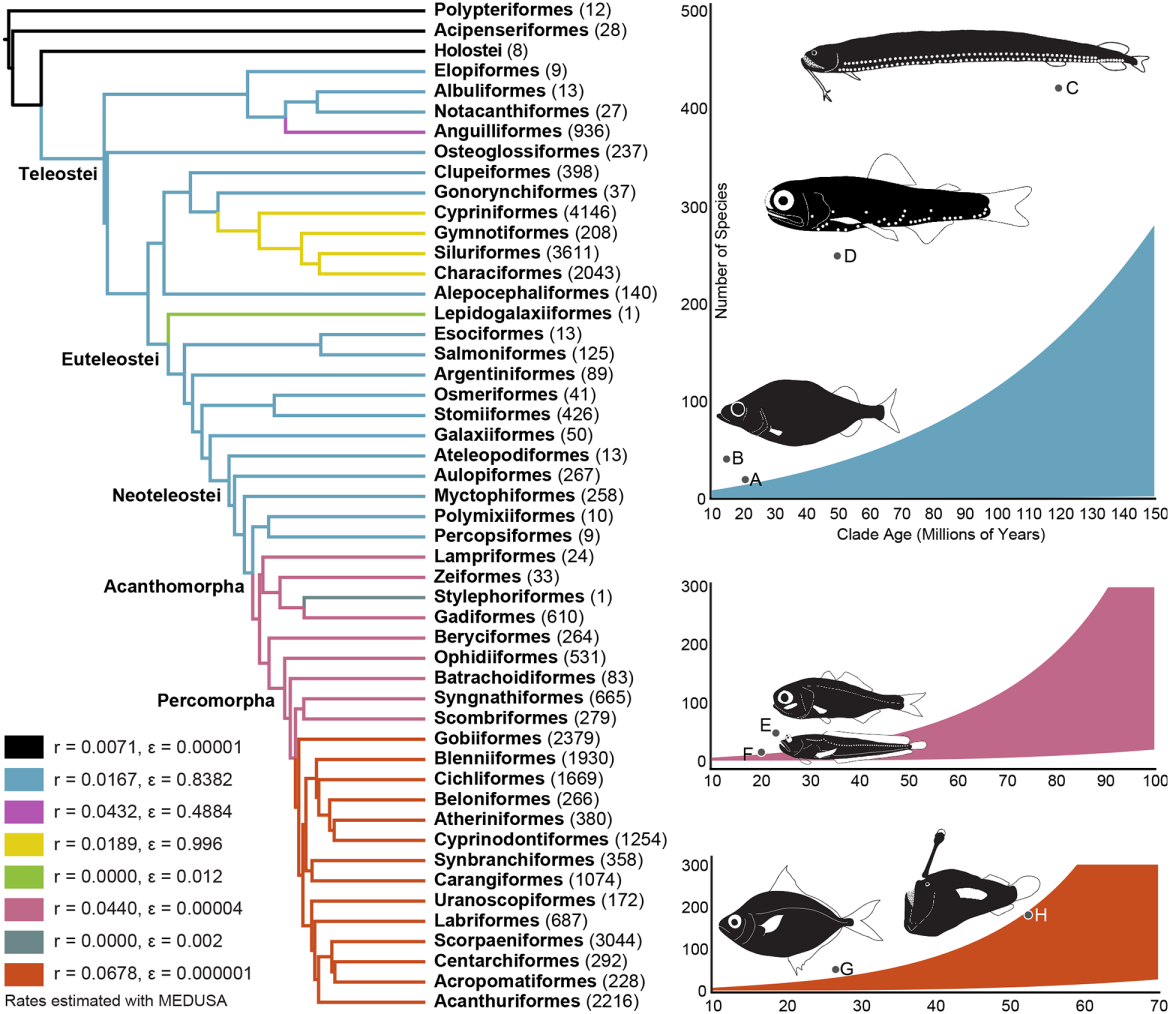
Patterns of Diversification across Ray-Finned Fishes and Bioluminescent Lineages. Temporal hypothesis of the relationships of ray-finned fishes with net diversification rates and relative rates of extinction estimated by MEDUSA. Species richness curves indicate the 95 percent confidence interval for the expected number of species given clade age given a net diversification rate and relative rate of extinction. Letters indicate bioluminescent lineages of fishes in Fig 1.

Bayesian ancestral-character-state reconstructions for the evolution of bioluminescence (0:Absent; 1:Present), coded from known and previously published occurrences in ray-finned fishes [1–3, 5–7], were performed in BayesTraits v2.0 MultiState [22] using Markov chain Monte Carlo (MCMC) approaches to infer ancestral states at nodes in the phylogeny across a distribution of topologies (500 trees subsampled from 5,000 post burn-in trees) where the branches have varying lengths relative to time (Fig 3). Each transformation from absence to presence in the Bayesian ancestral-states reconstruction was counted as an independent evolution of bioluminescence among ray-finned fishes. The GEIGER module in R [23] was used to calculate a 95% confidence interval of the expected number of species within a clade given a net diversification rate (r), a relative extinction rate, and crown clade age [24]. Rates for net diversification and relative extinction were estimated with MEDUSA [25] (Fig 4), with species richness [i.e., the number of currently valid described species for each clade (S3 Table)] generated from the *Catalog of Fishes* [26]. Following its use in recent studies [7–8], we identify and highlight lineages as having exceptional species richness if their known species diversity, given hypothesized clade age, lie outside the upper confidence interval bounds of expected species richness.

## Results

Bioluminescence is inferred to have evolved independently at least 27 times among 14 major fish clades (Figs 1 and 2, S1 Fig). Intrinsic bioluminescence, in which a fish produces and emits light without the aid of bacterial symbiosis, evolved eight times (Figs 1 and 2). Of the approximately 1,510 species of known bioluminescent fishes, more than half (~785 species) exhibit intrinsic bioluminescence (Figs 1 and 2, S1 Fig). Bacterially mediated bioluminescence through symbiosis has evolved at least 17 times (Figs 1 and 2, S1 Fig), representing approximately 48% of all bioluminescent fishes (~725 species, Fig 4). All occurrences of bioluminescence across ray-finned fishes evolved from the Early Cretaceous (150 Ma) through the Cenozoic (Fig 1), with the oldest occurrence in Stomiiformes (Fig 1). Six orders (Alepocephaliformes, Myctophiformes, Stomiiformes, Batrachoidiformes, Beryciformes, and Acanthuriformes), representing 57% of all bioluminescent fishes (~814 species), include lineages that exhibit exceptional species richness given clade age (Fig 4).

## Discussion

Bioluminescence is widespread across ray-finned fishes that occupy marine environments, and 27 independent evolutionary events of bioluminescence are identified (Figs 1 and 2). These 27 groups are distributed across 14 major lineages of ray-finned fishes (Figs 1 and 2, S1 Fig) that occupy deep-sea (e.g., lanternfishes, anglerfishes), inshore (e.g., ponyfishes, croakers), and coral reef (e.g., cardinalfishes, pineconefishes) habitats. Our findings demonstrate that the number of independent evolutionary events of bioluminescence across the tree of life is significantly higher than previous summaries suggest (40) [2–3] and highlight the need to explore the evolution of this phenomenon phylogenetically in bioluminescent lineages across Metazoa. By combining our findings with the inference that squaliform sharks have evolved bioluminescence once or twice [1, 4, 14, 27], we can infer that bioluminescence has evolved at least 29 times in vertebrates alone. This significant increase in the number of independent origins of bioluminescence in vertebrates is found exclusively among fishes living in marine environments. At present, the only known terrestrial animals capable of bioluminescence are arthropods (e.g., fireflies, millipedes) [1]; whereas in marine environments, bioluminescence has evolved across the tree of life from bacteria to vertebrates (e.g., Ctenophora, Mollusca, Crustacea, Tunicata, Vertebrata) [1–3].

Of the 27 evolutionary events of bioluminescence in ray-finned fishes, bacterially mediated symbiosis has evolved 17 times (Figs 1 and 2), particularly among acanthomorph (spiny-rayed) lineages. All bioluminescent bacteria that are symbiotic with fishes are vibrionaceans [28], and there is little to no host specificity between species of bioluminescent bacteria and fishes, which acquire bacteria from their local environment [6–7]. Fishes that live in symbiosis with bioluminescent bacteria exhibit a vast array of anatomical structures to focus, broadcast, or even restrict the light these bacteria produce [7, 10]. Multiple fish orders with bioluminescent bacteria contain lineages that exhibit exceptional species richness given clade age (Fig 4), including Beryciformes (flashlightfishes) and Acanthuriformes (ponyfishes), with Ceratioidei (deep-sea anglerfishes) exhibiting exceptional species richness in the younger range of its estimated age of divergence. Ponyfishes (Leiognathidae) have evolved a complex array of sexually dimorphic muscular shutters and species-specific translucent windows to control the light emitted by the symbiotic bacteria living in a specialized pouch derived from esophageal tissue [7, 10], and deep-sea anglerfishes have evolved complex, species-specific bioluminescent dorsal-fin escas (lures) that are presumably used for communication and prey attraction [29]. It is likely that the number of independent symbiotic relationships between fishes and bioluminescent bacteria could be higher than those estimated herein, given more fine scale species-level sampling of some lineages. For example, a densely sampled phylogeny of the diverse order Gobiiformes [30] suggests that bacterial bioluminescence may have independently evolved more than once among the cardinalfishes (Apogonidae), although bioluminescence was not explicitly optimized in the gobiiform study.

Across ray-finned fishes, intrinsic bioluminescence evolved at least eight times (Figs 1 and 2, S1 Fig) in some of the most species-rich lineages of deep-sea fishes (Figs 3 and 4), including dragonfishes (Stomiiformes, 426 species) and lanternfishes (Myctophiformes, 256 species). One genus of anglerfishes, the netdevils (*Linophyrne*), has even evolved an intrinsic bioluminescent chin barbel to complement their bacterially illuminated escal lure [29]. Despite evolving less frequently than bacterially mediated bioluminescence, intrinsic bioluminescence notably accounts for more than half of all known bioluminescent fish species and nearly 90 percent of bioluminescent species that exhibit exceptional species richness given their clade age (Fig 4). A recent study hypothesized that bioluminescence functions as a species-specific communication/identification system among species-rich lineages (lanternfishes, dragonfishes) and that this system has played a significant role in their diversification in the deep sea, a region devoid of obvious physical barriers to reproduction [8]. The current study corroborates those findings and also indicates that other lineages with intrinsic bioluminescence and the potential for bioluminescent communication (as opposed to camouflage) have increased rates of diversification, including both inshore and deep-sea bioluminescent lineages that have more recently evolved (Batrachoidiformes and Alepocephaliformes, respectively, Fig 4). It is still unclear how most fishes with intrinsic bioluminescence obtain the necessary substrates to produce light. For at least one lineage of fishes (*Porichthys*, midshipmen), luciferin is obtained from their diet [2–3].

We show that bioluminescence has repeatedly evolved in ray-finned fishes at varying times in Earth’s history (Figs 1 and 2), spanning the Mesozoic (150 to 65 Ma) and Cenozoic (65 Ma to present day). This suggests bioluminescence was present in Cretaceous seas and may have played an early role in the diversification of some deep-sea lineages that are exceptionally species rich given their clade age (lanternfishes and dragonfishes). Notably, none of the bioluminescent ray-finned fish lineages that possess exceptional present day species richness are thought to use bioluminescence exclusively for camouflage, with many of these lineages possessing species-specific anatomical structures that are thought to aid in communication, predation, and reproduction [7–8]. This pattern is also observed in squaliform sharks, where the two deep-sea bioluminescent lineages, Etmopteridae and Dalatiidae, are hypothesized to have also evolved during the Cretaceous and exhibit elevated rates of diversification [27]. As observed in the species-rich lanternfishes and dragonfishes [8], these sharks have species-specific bioluminescent structures and patterns [31]. Recent studies have shown that luminescent systems other than bioluminescence, such as biofluorescence, have repeatedly evolved and are phylogenetically widespread throughout the evolution of marine fishes [32]. Biofluorescence, like bioluminescence, may have a signaling function in marine fishes [32–33]. Our findings, and these additional studies investigating the evolution and function of bioluminescence and biofluorescence in marine systems, highlight how much remains to be discovered regarding the potential impacts of bioluminescence, and luminescent signaling in general, on the evolutionary history and ecology of marine fishes.

## Supporting Information

S1 Fig Maximum Likelihood Topology of the Evolutionary Relationships of Ray-Finned Fishes. Numbers at nodes indicate fossil calibrations. Black dots indicate bootstrap support value less than 60. All other nodes have bootstrap support values greater than 60.
(PDF)

S1 Table GenBank Accession Numbers for Newly Collected Sequences. (PDF)

S2 Table GenBank Accession Numbers for COI Sequences. (PDF)

S3 Table Classification of Vertebrates. Classification of vertebrates (www.classification.fish) with known species diversity [26].
(PDF)

S1 Text Fossil Calibrations for Divergence Time Analyses. (PDF)
