# Supplementary figures and images for: Repeated and Widespread Evolution of Bioluminescence in Marine Fishes
Source: PLoS One. 2016 Jun 8;11(6):e0155154. doi: 10.1371/journal.pone.0155154 (PMC4898709; doi:10.1371/journal.pone.0155154)

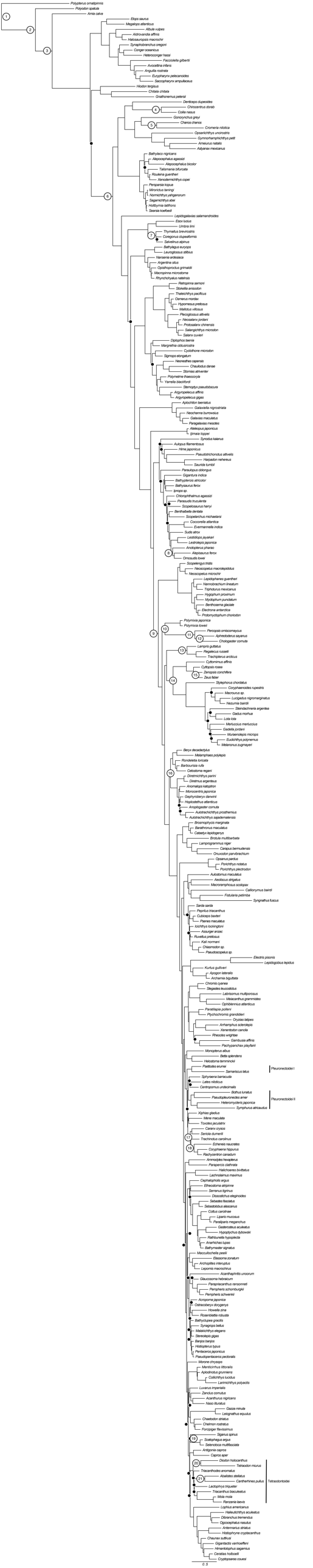

Supplement: S1 Fig — Numbers at nodes indicate fossil calibrations. Black dots indicate bootstrap support value less than 60. All other nodes have bootstrap support values greater than 60. (PDF) [file pone.0155154.s001.pdf]
