# Supplementary material for: Repeated and Widespread Evolution of Bioluminescence in Marine Fishes
Source: PLoS One. 2016 Jun 8;11(6):e0155154. doi: 10.1371/journal.pone.0155154 (PMC4898709; doi:10.1371/journal.pone.0155154)
Supplement: S1 Table — (PDF) [file pone.0155154.s002.pdf]

Supplement Table 1: Genbank accession numbers for newly collected sequences

	COI	ENC1	GLYT	MYH6	PLAG	PTR	RAG1	SH3	SREB	TBR	ZIC1
Alepocephalus bicolor	KJ190022	KX227793	KX227812	NA	KX227893	NA	NA	KX227950	NA	KX227989	KJ190108
Argyropelecus affinis	KJ190035	KX227794	NA	KX227850	KX227894	KX227913	KX228066	KX227951	NA	KX227990	KJ190121
Aulopus filamentosus	EU366546	EU366593	KX227813	KX227851	EU366642	NA	EU366688	NA	NA	NA	EU366733
Avocettina infans	KX228016	NA	KX227814	KX227852	KX227895	KX227914	NA	KX227952	NA	KX227991	NA
Bathyclupea gracilis	NA	NA	KX227815	KX227853	NA	KX227915	NA	KX227953	KX227980	KX227992	KX228050
Bathysaurus ferox	EU366547	EU366594	KX227816	KX227854	EU366643	KX227916	EU366689	NA	NA	NA	EU366734
Benthosema glaciale	KF768167	NA	KX227817	NA	NA	KX227917	KF768155	KX227954	NA	KX227993	KF768160
Chauliodus danae	KX228017	KX227795	KX227818	KX227855	NA	KX227918	NA	KX227955	NA	KX227994	KX228049
Coccorella atlantica	EU366554	EU366601	KX227819	KX227856	EU366649	KX227919	EU366696	NA	NA	NA	EU366741
Diplophos taenia	KJ190028	KX227796	KX227820	KX227857	KX227896	KX227920	KJ190079	KX227956	KX227981	NA	KJ190114
Evermannella indica	EU366555	EU366602	KX227821	KX227858	EU366650	NA	EU366697	NA	NA	NA	EU366742
Facciolella gilbertii	KF768170	KX227797	NA	KX227859	KX227897	KX227921	NA	KX227957	KX227982	KX227995	KF768162
Gadella jordani	KX228018	KX227798	KX227822	KX227860	KX227898	NA	NA	KX227958	NA	NA	NA
Gigantura indica	EU366557	EU366604	KX227823	KX227861	EU366652	KX227922	EU366699	NA	NA	NA	EU366744
Harpadon nehereus	EU366558	EU366605	KX227824	KX227862	EU366653	KX227923	EU366700	NA	NA	NA	EU366745
Hime japonicus	EU366545	EU366592	KX227825	KX227863	EU366641	KX227924	EU366687	NA	NA	NA	EU366732
Holtbyrnia latifrons	KJ190023	NA	KX227826	KX227864	KX227899	KX227925	NA	KX227959	NA	KX227996	KJ190109
Ipnops sp	EU366560	EU366607	KX227827	KX227865	EU366655	KX227926	EU366702	NA	NA	NA	EU366747
Lepidophanes guentheri	KJ190062	NA	NA	NA	KX227900	KX227927	KJ190094	KX227960	NA	NA	KJ190147
Leuroglossus stilbius	KX228019	KX227799	KX227828	KX227866	KX227901	KX227928	KX228065	KX227961	NA	KX227997	KX228048
Lucigadus nigromarginatus	NA	KX227800	KX227829	KX227867	NA	KX227929	KX228064	KX227962	NA	KX227998	KX228047
Margrethia obtusirostra	KJ190029	KX227801	NA	KX227868	NA	KX227930	KJ190080	KX227963	NA	KX227999	NA
Miroríctus taningi	KX228020	NA	KX227830	KX227869	KX227902	NA	KX228063	NA	NA	NA	KX228046
Myctophum punctatum	KJ190065	NA	KX227831	KX227870	KX227903	KX227931	KX228062	KX227964	NA	KX228000	NA
Nannobrachium lineatum	KX228021	NA	KX227832	NA	NA	KX227932	KX228061	KX227965	NA	NA	KX228045
Neoscopelus macrolepidotus	EU366587	EU366632	NA	KX227871	EU366678	KX227933	EU366727	KX227966	KX227983	KX228001	EU366771
Normichthys yahganorum	NA	NA	KX227833	KX227872	KX227904	KX227934	NA	KX227967	NA	KX228002	KX228044
Opisthoproctus grimaldii	NA	KX227802	NA	KX227873	NA	NA	KX228060	NA	NA	KX228003	KX228043
Parasudis truculenta	EU366569	EU366616	KX227834	KX227874	NA	KX227935	EU366710	NA	NA	NA	EU366753
Paraulopus oblongus	EU366568	EU366615	KX227835	KX227875	EU366664	KX227936	EU366709	NA	NA	NA	EU366752
Persparsia kopua	KX228022	NA	NA	KX227876	KX227905	KX227937	NA	KX227968	NA	KX228004	KX228042
Plecoglossus altivelis	KJ190025	KX227803	KX227836	KX227877	NA	NA	KJ190076	KX227969	KX227984	KX228005	KX228041
Polymetme thaeocoryla	KJ190039	KX227804	NA	KX227878	NA	KX227938	KX228059	KX227970	KX227985	KX228006	KX228040
Pseudoscopelus sp	KX228023	KX227805	KX227837	KX227879	KX227906	KX227939	KX228058	KX227971	KX227986	KX228007	KX228039
Pseudotrichonotus altivelis	EU366570	EU366617	KX227838	KX227880	NA	KX227940	EU366711	NA	NA	NA	EU366754
Rhynchohyalus natalensis	KX228024	KX227806	KX227839	KX227881	NA	NA	KX228057	KX227972	NA	KX228008	KX228038
Rosenblattia robusta	KX228025	KX227807	KX227840	KX227882	KX227907	KX227941	KX228056	KX227973	NA	KX228009	KX228037
Rouleina guentheri	NA	NA	KX227841	KX227883	NA	KX227942	NA	KX227974	NA	KX228010	KX228036
Saurida tumbil	KF768174	KX227808	KX227842	KX227884	NA	KX227943	KX228055	NA	NA	NA	KX228035
Scopelosaurus harryi	EU366572	EU366619	KX227843	KX227885	EU366666	KX227944	EU366713	NA	NA	NA	EU366756
Sigmops elongatum	KX228026	KX227809	KX227844	KX227886	KX227908	NA	NA	NA	NA	NA	KX228034
Sternoptyx pseudobscura	KF768176	NA	NA	KX227887	KX227909	NA	KX228054	KX227975	KX227987	KX228011	KX228033
Stomias atriventer	KF768177	KX227810	NA	KX227888	KX227910	KX227945	KX228053	KX227976	NA	KX228012	KF768166
Synaphobranchus oregoni	KX228027	NA	KX227845	KX227889	KX227911	KX227946	NA	KX227977	NA	KX228013	KX228032
Synodus kaianus	EU366578	EU366625	KX227846	KX227890	EU366672	KX227947	EU366719	NA	NA	NA	EU366761
Talismania bifurcata	KX228028	KX227811	KX227847	NA	KX227912	NA	KX228052	NA	NA	NA	KX228031
Thaleichthys pacificus	KX228029	EU366635	KX227848	KX227891	EU366681	KX227948	AY380537	KX227978	KX227988	KX228014	EU366774
Triphoturus mexicanus	KJ190074	NA	KX227849	KX227892	NA	KX227949	KX228051	KX227979	NA	KX228015	KX228030
