# Supplementary material for: Repeated and Widespread Evolution of Bioluminescence in Marine Fishes
Source: PLoS One. 2016 Jun 8;11(6):e0155154. doi: 10.1371/journal.pone.0155154 (PMC4898709; doi:10.1371/journal.pone.0155154)
Supplement: S2 Table — (PDF) [file pone.0155154.s003.pdf]

Supplement Table 2: Genbank accession numbers for COI sequences

	COI
Abalistes stellatus	JF492756
Acanthochaenus luetkenii	EU148068
Acanthurus nigricans	EF648267
Acropoma japonica	HQ945845
Albula vulpes	GU225131
Aldrovandia affinis	AP002974
Alepisaurus ferox	EU366542
Alepocephalus agassizi	NC13564
Alepocephalus bicolor	KJ190022
Ameiurus natalis	EU524425
Amia calva	AB042952
Ammodytes hexapterus	JQ353965
Anguilla rostrata	EU524441
Anomalops katoprion	FJ58285
Anoplogaster cornuta	FJ164283
Anotopterus pharo	GU440224
Antennarius striatus	GU188497
Antigonia capros	AP002943
Aphredoderus sayanus	JN024806
Aplochiton taeniatus	HQ540333
Aplodinotus grunniens	EU523923
Archoplites interuptus	JN024809
Argyropelecus affinis	KJ190035
Argyropelecus gigas	EU148084
Assurger anzac	JN990845
Astyanax mexicanus	HQ557152
Ateleopus japonicus	AP002916
Aulopus filamentosus	EU366546
Aulostomus maculatus	JQ842017
Aulotrachichthys prosthemius	DQ648438
Avocettina infans	KX228016
Barbourisia rufa	JQ354000
Bathylaco nigricans	AP009494
Bathymaster signatus	HQ712316
Bathysaurus ferox	EU366547
Benthalbella dentata	EU366552
Benthosema glaciale	KF768167
Beryx decadactylus	JF492956
Betta splendens	JQ667506
Bothus lunatus	JQ842025
Brosmophycis marginata	JQ354026
Brotula multibarbata	JQ431501
Callionymus bairdi	JQ840435
Cantherhines pullus	JQ841489
Capros aper	JQ774992
Caranx crysos	GU702378
Carapus bermudensis	JQ841493
Centroberyx druzhinini	HQ945941
Centropomus undecimalis	JQ365276
Cephalopholis argus	JX674947
Ceratias holboelli	GU440269
Cetostoma regani	AP010882
Chaetodon striatus	JQ840451
Chanos chanos	HQ654700
Chauliodus danae	KX228017
Chelmon rostratus	FJ583134
Chiasmodon sp	JQ354040
Chirocentrus dorab	JF493145
Chitala chitala	FJ918897
Chlorophthalmus agassizi	EU366553
Chologaster cornuta	HQ557553
Chromis cyanea	JQ840457
Coccorella atlantica	EU366554
Coilia nasus	AP009135
Collichthys lucidus	HM180544
Coregonus clupeaformis	EU523959
Coryphaena hippurus	JQ839745
Cottus carolinae	JN025051
Cromeria nilotica	AP007275
Cryptopsaras couesi	EU403054
Cyclothone microdon	EU148134.
Cyttopsis rosea	JQ774524
Denticeps clupeoides	AP007276
Dibranchus tremendus	KC015319
Diodon holocanthus	GU225199
Diplophos taenia	KJ190028
Diretmus argenteus	EU148155
Dissostichus eleginoides	JN640627
Echeneis naucrates	JQ841117
Elassoma zonatum	JN025308
Electrona antarctica	EU326342
Eleotris pisonis	AY722157
Elops saurus	AP004807
Esox lucius	EU524591
Etheostoma atripinne	HQ557533
Eurypharynx pelecanoides	AB046473
Evermanella indica	EU366555
Facciolella gilberti	KF768170
Fistularia petimba	JQ365356
Forcipiger flavissimus	JF434972
Gadella jordani	KX228018
Gadus morhua	KP975727
Galaxias maculatus	AP004104
Galaxiella nigrostriata	AP006853
Gambusia affinis	JQ842475
Gasterosteus aculeatus	JQ354102
Gazza minuta	DQ028007
Gephyroberyx darwinii	HQ945918
Gigantactis vanhoeffeni	EU148172
Gigantura indica	EU366557
Glaucosoma hebraicum	EF609357
Gnathonemus petersii	HM880237
Gonorynchus greyi	AB054134
Gymnorhamphichthys petit	JN988878
Gyrinomimus sp	FJ164638
Halichoeres bivittatus	JQ840524
Halosauropsis macrochir	EU869816
Harpadon neherus	EU366558
Heteroconger hassi	JQ350055
Heteromycteris japonica	ABFJ254
Himantolophus sagamius	GU440342
Hime japonicus	EU366545
Hiodon tergisus	EU524659
Histiophryne cryptacanthus	GU188513
Histiopterus typus	HQ945817
Holtbyrnia latifrons	KJ190023
Hoplostethus atlanticus	JN580187
Hygophum proximum	KJ555400
Hypomesus pretiosus	JQ354137
Hypoptychus dybowski	NC4400
Ipnops sp	EU366560
Kali normani	GU440362
Kurtus gulliveri	EU381031
Lachnolaimus maximus	JQ841240
Lactophrys triqueter	JQ861018
Lampris guttatus	DQ885096
Lamprogrammus niger	JQ354156
Larimichthys polyactis	HM068248
Lates niloticus	GU324190
Leiognathus equulus	DQ028017
Lepidogalaxias salamandroides	FJ918925
Lepidophanes guentheri	KJ190062
Lepomis macrochirus	EU524741
Lestidiops jayakari	EU366562
Lestrolepis japonica	KJ190055
Leuroglossus stilbius	KX228019
Liparis mucosus	JQ354182
Lophius americanus	EU660715
Lota lota	HQ961084
Luvarus imperialis	AP009161
Maccullochella peelii	DQ107940
Macropinna microstoma	EU869816
Macroramphosus scolopax	JQ775070
Macrourus sp	EU074455
Mallotus villosus	HQ712650
Margrethia obtusirostra	KJ190029
Megalops atlanticus	AP004808
Melanonus zugmayeri	EU148250
Mene maculata	GU805056
Merluccius merluccius	KC500913.
Mirorictus taningi	KX228020
Mola mola	JX438518
Monocentris japonica	DQ648452
Monopterus albus	AP002945
Morone chrysops	EU524141
Myctophum punctatum	KJ190065
Myripristis murdjan	JF493941
Myripristis violacea	HM034239
Nannobrachium lineatum	KX228021
Nansenia ardesiaca	AP004106
Naso lituratus	HM034244
Neonesthes capensis	KF768171
Neosalanx jordani	HM151574
Neoscopelus macrolepidotus	EU366587
Neoscopelus microchir	KF768172
Omosudis lowei	EU366566
Onuxodon parvibrachium	JF494022
Ophioblennius atlanticus	FJ583755
Opsariichthys uncirostris	AB218897
Oryzias latipes	AP004421
Osmerus mordax	JQ354253
Parapercis clathrata	JQ844110
Parapriacanthus ransonneti	GU805108
Paratilapia polleni	AY263886
Paraulopus oblongus	EU366568
Pempheris schwenkii	HQ561454
Percopsis omiscomaycus	EU524269
Persparsia kopua	KX228022
Plecoglossus altivelis	KJ190025
Polymetme thaeocoryla	KJ190039
Polymixia japonica	AB034826
Polymixia loweii	AP002927
Porichthys notatus	JQ354298
Poromitra crassiceps	FJ165052
Protomyctophum choriodon	EU326418
Protosalanx chinensis	JN242658
Psettodes erumei	EF609580
Pseudopentaceros pectoralis	GU440483
Pseudopleuronectes americanus	EU752158
Pseudoscopelus sp	KX228023
Pseudotrichonotus altivelis	EU366570
Ptychochromis grandidieri	AY263878
Rachycentron canadum	JQ841349
Ranzania laevis	HQ167729
Rathbunella hypoplecta	GU440493
Retropinna semoni	FJ918924
Rheocles wrightae	AY290803
Rhynochohyalus natelnsis	KX228024
Rondeletia loricata	EU148304
Rosenblattia robusta	KX228025
Saccopharynx ampullaceus	EU148306
Sagamichthys abei	KF930364
Salangichthys microdon	FJ205609
Salanx cuvieri	HM151589
Salvelinus alpinus	JX960951
Sarda sarda	JQ623978
Sargocentron cornutum	FJ237590
Sargocentron vexillarium	JQ840676
Saurida tumbil	KF768174
Scatophagus argus	JN021250
Scopelarchus michaelarsi	KM983051
Scopelengys tristis	KF768175
Scopeloberyx sp	EU148313
Scopelogadus beanii	EU148316
Scopelosaurus harryi	EU366572
Searsia koefoedi	EU148320
Sebastolobus alascanus	JQ354498
Selenotoca multifasciata	DQ107759
Seriola dumerili	JX261426
Serranus tigrinus	JQ843057
Siganus spinus	JQ432159
Sigmops elongatum	KX228026
Sphyraena barracuda	JQ842711
Stegastes leucostictus	JQ841016
Stereolepis gigas	GU440534
Sternoptyx pseudobscura	KF768176
Stomias atriventer	KF768177
Stylephorus chordatus	AB280689
Sudis atrox	EU366576
Symphurus atricaudus	GU440541
Synaphobranchus oregoni	KX228027
Syngnathus fuscus	JN312447
Synodus kaianus	EU366578
Talismania bifurcata	KX228028
Tetraodon miurus	NC15361
Thaleichthys pacificus	KX228029
Thymallus brevirostris	JX960972
Toxotes jaculatrix	HQ654759
Trachinotus carolinus	JX034020
Triacanthodes anomalus	AP009172
Triacanthus biaculeatus	JF952881
Triphoturus mexicanus	KJ190074
Umbra limi	EU522452
Xenentodon cancila	FJ459539
Xenodermichthys copei	FJ918920
Yarrella blackfordi	FJ918935
Zanclus cornuta	JQ350417
Zeus faber	HQ945864
