# Supplementary material for: Repeated and Widespread Evolution of Bioluminescence in Marine Fishes
Source: PLoS One. 2016 Jun 8;11(6):e0155154. doi: 10.1371/journal.pone.0155154 (PMC4898709; doi:10.1371/journal.pone.0155154)
Supplement: S3 Table — Classification of vertebrates (www.classification.fish) with known species diversity [26]. (PDF) [file pone.0155154.s004.pdf]

Supplement Table 3: Classification of vertebrates (www.classification.fish) with known species diversity (based on Catalog of Fishes; Eschmeyer, 2014).

Higher Categories	Order	Family	Species in Family	Species in Order (MEDUSA CLADES)
Infraphylum Vertebrata				
Superclass Petromyzontomorphi				
	Petromyzontiformes			46
		Petromyzontidae	42	
		Geotriidae	1	
		Mordaciidae	3	
Superclass Gnathostomata				
Class Chondrichthyes				
Subclass Holocephali				
	Chimaeriformes			49
		Callorhinchidae	3	
		Chimaeridae	38	
		Rhinochimaeridae	8	
Subclass Elasmobranchii				
Subdivision Selachii				
Superorder Squalomorphi				
	Hexanchiformes			6
		Hexanchidae	4	
		Chlamydoselachidae	2	
	Squaliformes			123
		Dalatiidae	9	
		Etmopteridae	46	
		Somniosidae	17	
		Oxynotidae	5	
		Centrophoridae	15	
		Squalidae	29	
		Echinorhinidae	2	
	Pristiophoriformes			8
		Pristiophoridae	8	
	Squatiniformes			21
		Squatinidae	21	
Superorder Galeomorphi				
	Heterodontiformes			9
		Heterodontidae	9	
	Lamniformes			15
		Odontaspididae	3	
		Mitsukurinidae	1	
		Pseudocarchariidae	1	
		Lamnidae	5	
		Megachasmidae	1	
		Cetorhinidae	1	
		Alopiidae	3	
	Orectolobiformes			43
		Rhincodontidae	1	
		Parascylliidae	8	
		Brachaeluridae	2	
		Orectolobidae	12	
		Hemiscylliidae	16	
		Stegostomatidae	1	
		Ginglymostomatidae	3	
	Carcharhiniformes			285
		Pentanchidae	86	
		Scyliorhinidae	63	
		Proscylliidae	7	
		Pseudotriakidae	4	
		Leptochariidae	1	
		Triakidae	47	
		Hemigaleidae	8	
		Carcharhinidae	59	
		Sphyrnidae	10	
Subdivision Batoidea				
	Rajiformes			299
		Arhynchobatidae	107	
		Rajidae	180	
		Anacanthobatidae	12	
	Platyrhiniformes			4
		Platyrhinidae	4	
	Torpediniformes			67
		Narkidae	12	
		Narcinidae	31	
		Hypnidae	1	
		Torpedinidae	23	
	Rhinobatiformes			56
		Rhinobatidae	56	
	Pristiformes			7
		Pristidae	7	
	Myliobatiformes			228
		Zanobatidae	2	
		Plesiobatidae	1	
		Hexatrygonidae	1	
		Dasyatidae	91	
		Gymnuridae	14	
		Myliobatidae	44	
		Urolophidae	30	
		Potamotrygonidae	28	
		Urotrygonidae	17	
Class Sarcopterygii				
Subclass Coelacanthimorpha				
	Coelacanthiformes			2
		Latimeriidae	2	
Subclass Dipnoi				
Superorder Ceratodontimorpha				
	Ceratodontiformes			1
		Neoceratodontidae	1	
	Lepidosireniformes			5
		Lepidosirenidae	1	
		Protopteridae	4	
[unranked] Tetrapodomorpha				
Subclass Tetrapoda [estimated 28,000 species]				
Class Actinopterygii				
Subclass Cladistia				
	Polypteriformes			12
		Polypteridae	12	
Subclass Chondrostei				
	Acipenseriformes			28
		Acipenseridae	26	
		Polyodontidae	2	
Subclass Neopterygii				
Division Holostei	Holostei			8
		Lepisosteidae	7	
		Amiidae	1	
Division Teleostei				
Subdivision Elopomorpha				
	Elopiformes			9
		Elopidae	7	
		Megalopidae	2	
	Albuliformes			13
		Albulidae	13	
	Notacanthiformes			27
		Halosauridae	16	
		Notacanthidae	11	
	Anguilliformes			936
		Protanguillidae	1	
		Synaphobranchidae	38	
		Anguillidae	18	
		Cyematidae	2	
		Eurypharyngidae	1	
		Monognathidae	15	
		Moringuidae	15	
		Nemichthyidae	9	
		Saccopharyngidae	10	
		Serrivomeridae	10	
		Chlopsidae	22	
		Colocongridae	10	
		Congridae	196	
		Derichthyidae	3	
		Muraenesocidae	15	
		Nettastomatidae	43	
		Ophichthidae	317	
		Heterenchelyidae	8	
		Muraenidae	198	
		Myrocongridae	5	
Subdivision Osteoglossomorpha				
	Osteoglossiformes			237
		Osteoglossidae	6	
		Arapaimidae	6	
		Pantodontidae	1	
		Hiodontidae	2	
		Notopteridae	10	
		Mormyridae	211	
		Gymnarchidae	1	
Subdivision Otocephala				
Superorder Clupeomorpha				
	Clupeiformes			398
		Denticipitidae	1	
		Clupeidae	250	
		Engraulidae	147	
Superorder Alepocephalomorpha				
	Alepocephaliformes			140
		Alepocephalidae	100	
		Platytroctidae	40	
Superorder Ostariophysi				
Series Anotophysi				
	Gonorynchiformes			37
		Chanidae	1	
		Gonorynchidae	5	
		Kneriidae	30	
		Phractolaemidae	1	
Series Otophysi				
	Cypriniformes			4146
		Psilorhynchidae	23	
		Cyprinidae	2938	
		Botiidae	56	
		Cobitidae	187	
		Balitoridae	230	
		Nemacheilidae	623	
		Vaillantellidae	3	
		Gyrinocheilidae	3	
		Catostomidae	83	
	Characiformes			2043
		Citharinidae	8	
		Distichodontidae	98	
		Alestidae	117	
		Hepsetidae	4	
		Hemiodontidae	31	
		Parodontidae	32	
		Curimatidae	103	
		Prochilodontidae	21	
		Anostomidae	145	
		Chilodontidae	8	
		Erythrinidae	16	
		Lebiasinidae	74	
		Gasteropelecidae	9	
		Ctenoluciidae	7	
		Acestrorhynchidae	26	
		Cynodontidae	8	
		Serrasalmidae	87	
		Characidae	1059	
		Bryconidae	49	
		Triportheidae	21	
		Iguanodectidae	30	
		Chalceidae	5	
		Crenuchidae	85	
	Siluriformes			3611
		Diplomystidae	6	
		Lacantuniidae	1	
		Ictaluridae	51	
		Horabagridae	10	
		Bagridae	210	
		Cranoglanididae	5	
		Austroglanididae	3	
		Siluridae	101	
		Schilbeidae	60	
		Pangasiidae	28	
		Amblycipitidae	35	
		Amphiliidae	93	
		Akysidae	57	
		Sisoridae	210	
		Erethistidae	41	
		Clariidae	118	
		Heteropneustidae	4	
		Claroteidae	92	
		Chacidae	4	
		Olyridae	6	
		Malapteruridae	19	
		Ariidae	149	
		Anchariidae	6	
		Plotosidae	40	
		Mochokidae	209	
		Doradidae	92	
		Auchenipteridae	111	
		Pimelodidae	109	
		Pseudopimelodidae	39	
		Heptapteridae	207	
		Cetopsidae	42	
		Aspredinidae	40	
		Nematogenyidae	1	
		Trichomycteridae	273	
		Callichthyidae	200	
		Loricariidae	872	
		Scoloplacidae	6	
		Astroblepidae	61	
	Gymnotiformes			208
		Sternopygidae	35	
		Apteronotidae	90	
		Rhamphichthyidae	15	
		Hypopomidae	29	
		Gymnotidae	39	
Subdivision Euteleostei				
[unranked] Lepidogalaxi				
	Lepidogalaxiiformes			1
		Lepidogalaxiidae	1	
[unranked] Neognathi				
Superorder Protacanthopterygii				
	Argentiniformes			89
		Argentinidae	28	
		Opisthoproctidae	19	
		Microstomatidae	19	
		Bathylagidae	23	
	Esociformes			13
		Esocidae	6	
		Umbridae	7	
	Salmoniformes			125
		Salmonidae	125	
[unranked] Neoteleostei				
Superorder Stenopterygii				
	Stomiiformes			426
		Diplophidae	9	
		Gonostomatidae	24	
		Sternoptychidae	75	
		Phosichthyidae	24	
		Stomiidae	294	
	Osmeriformes			41
		Osmeridae	14	
		Plecoglossidae	1	
		Salangidae	20	
		Retropinnidae	6	
[unranked][unnamed]				
	Galaxiiformes			50
		Galaxiidae	50	
[unranked] [unnamed]				
Superorder Ateleopodomorpha				
	Ateleopodiformes			13
		Ateleopodidae	13	
Superorder Ctenosquamata				
[unranked] Eurypterygii				
	Aulopiformes			267
		Aulopidae	12	
		Synodontidae	72	
		Pseudotrichonotidae	2	
		Paraulopidae	14	
		Giganturidae	2	
		Bathysauridae	2	
		Bathysauroididae	1	
		Bathysauropsidae	2	
		Ipnopidae	31	
		Chlorophthalmidae	21	
		Notosudidae	17	
		Scopelarchidae	18	
		Evermannellidae	8	
		Sudidae	2	
		Alepisauridae	3	
		Anotopteridae	3	
		Lestidiidae	46	.
		Paralepididae	11	
Superorder Scopelomorpha				
[unranked] Ctenosquamata				
	Myctophiformes			258
		Neoscopelidae	6	
		Myctophidae	252	
[unranked] Acanthomorpha				
[unranked][unnamed]				
Superorder Polymixiomorpha				
	Polymixiiformes			10
		Polymixiidae	10	
Superorder Paracanthopterygii				
	Percopsiformes			9
		Percopsidae	2	
		Aphredoderidae	1	
		Amblyopsidae	6	
	Zeiformes			33
		Cyttidae	3	
		Oreosomatidae	10	
		Parazenidae	4	
		Zenionidae	7	
		Grammicolepididae	3	
		Zeidae	6	
	Stylephoriformes			1
		Stylephoridae	1	
	Gadiformes			610
		Muraenolepididae	8	
		Bregmacerotidae	14	
		Euclichthyidae	1	
		Macrouridae	400	
		Moridae	107	
		Melanonidae	2	
		Gadidae	23	
		Lotidae	22	
		Phycidae	11	
		Merlucciidae	22	
Superorder Acanthopterygii				
	Lampriformes			24
		Lampridae	2	
		Veliferidae	2	
		Lophotidae	5	
		Radiicephalidae	1	
		Trachipteridae	11	
		Regalecidae	3	
Series Berycimorpha				
	Beryciformes			264
		Monocentridae	4	
		Trachichthyidae	48	
		Anomalopidae	9	
		Diretmidae	4	
		Anoplogastridae	2	
		Holocentridae	84	
		Stephanoberycidae	4	
		Hispidoberycidae	1	
		Melamphaidae	63	
		Gibberichthyidae	2	
		Berycidae	10	
		Rondeletiidae	2	
		Barbourisiidae	1	
		Cetomimidae	30	
Series Percomorpha				
	Ophidiiformes			531
		Ophidiidae	261	
		Carapidae	35	
		Bythitidae	209	
		Aphyonidae	23	
		Parabrotulidae	3	
	Batrachoidiformes			83
		Batrachoididae	83	
	Gobiiformes			2379
		Apogonidae	348	
		Kurtidae	2	
		Rhyacichthyidae	3	
		Odontobutidae	22	
		Eleotridae	145	
		Thalasseleotrididae	3	
		Butidae	45	
		Gobiidae	1811	
	Scombriformes			279
		Gempylidae	25	
		Trichiuridae	46	
		Scombridae	53	
		Scombrolabracidae	1	
		Amarsipidae	1	
		Centrolophidae	31	
		Nomeidae	17	
		Ariommatidae	8	
		Tetragonuridae	3	
		Stromateidae	17	
		Pomatomidae	1	
		Bramidae	20	
		Caristiidae	19	
		Icosteidae	1	
		Arripidae	4	
		Chiasmodontidae	32	
	Syngnathiformes			665
		Aulostomidae	3	
		Fistulariidae	4	
		Centriscidae	12	
		Solenostomidae	6	
		Syngnathidae	339	
		Dactylopteridae	7	
		Pegasidae	5	
		Mullidae	85	
		Callionymidae	190	
		Draconettidae	14	
	Synbranchiformes			358
		Nandidae	33	
		Anabantidae	31	
		Osphronemidae	132	
		Helostomatidae	1	
		Channidae	37	
		Synbranchidae	23	
		Mastacembelidae	88	
		Chaudhuriidae	10	
		Indostomidae	3	
	Carangiformes			1074
		Centropomidae	12	
		Latidae	13	
		Lactariidae	1	
		Rachycentridae	1	
		Echeneidae	8	
		Carangidae	149	
		Nematistiidae	1	
		Coryphaenidae	2	
		Menidae	1	
		Leptobramidae	1	
		Xiphiidae	1	
		Istiophoridae	10	
		Polynemidae	42	
		Sphyraenidae	29	
		Toxotidae	7	
		Psettodidae	3	
		Citharidae	6	
		Scophthalmidae	9	
		Paralichthyidae	110	
		Bothidae	166	
		Achiropsettidae	4	
		Pleuronectidae	106	
		Samaridae	27	
		Achiridae	36	
		Soleidae	183	
		Cynoglossidae	146	
	Atheriniformes			380
		Atherinidae	71	
		Bedotiidae	16	
		Melanotaeniidae	119	
		Pseudomugilidae	36	
		Atherinopsidae	109	
		Notocheiridae	1	
		Isonidae	5	
		Phallostethidae	23	
	Cyprinodontiformes			1254
		Aplocheilidae	14	
		Nothobranchiidae	258	
		Rivulidae	382	
		Profundulidae	8	
		Fundulidae	41	
		Valenciidae	2	
		Goodeidae	48	
		Poeciliidae	357	
		Cyprinodontidae	126	
		Anablepidae	18	
	Beloniformes			266
		Scomberesocidae	5	
		Belonidae	40	
		Hemiramphidae	61	
		Zenarchopteridae	58	
		Exocoetidae	66	
		Adrianichthyidae	36	
	Blenniiformes			1930
		Gobiesocidae	162	
		Tripterygiidae	175	
		Labrisomidae	119	
		Clinidae	87	
		Chaenopsidae	97	
		Dactyloscopidae	48	
		Blenniidae	405	
		Opistognathidae	82	
		Grammatidae	13	
		Plesiopidae	50	
		Pseudochromidae	128	
		Congrogadidae	24	
		Pomacentridae	393	
		Embiotocidae	23	
		Ambassidae	51	
		Mugilidae	73	
	Cichliformes			1669
		Polycentridae	4	
		Pholidichthyidae	2	
		Cichlidae	1663	
	Scorpaeniformes			3044
		Serranidae	87	
		Epinephelidae	236	
		Anthiidae	207	
		Niphonidae	11	
		Trachinidae	9	
		Bembropidae	24	
		Percophidae	1	
		Bovichtidae	11	
		Pseudaphritidae	1	
		Eleginopsidae	1	
		Nototheniidae	59	
		Harpagiferidae	12	
		Artedidraconidae	34	
		Bathydraconidae	17	
		Channichthyidae	26	
		Percidae	235	
		Hoplichthyidae	13	
		Platycephalidae	82	
		Bembridae	13	
		Triglidae	171	
		Congiopodidae	8	
		Neosebastidae	18	
		Apistidae	3	
		Synanceiidae	128	
		Scorpaenidae	362	
		Normanichthyidae	1	
		Hypoptychidae	2	
		Gasterosteidae	18	
		Aulorhynchidae	1	
		Anoplopomatidae	2	
		Bathymasteridae	7	
		Stichaeidae	70	
		Cryptacanthodidae	4	
		Pholidae	16	
		Ptilichthyidae	1	
		Zaproridae	1	
		Scytalinidae	1	
		Anarhichadidae	5	
		Zoarcidae	303	
		Zaniolepididae	3	
		Hexagrammidae	9	
		Trichodontidae	2	
		Cyclopteridae	27	
		Liparidae	415	
		Rhamphocottidae	4	
		Scorpaenichthyidae	1	
		Jordaniidae	2	
		Agonidae	60	
		Cottidae	106	
		Psychrolutidae	214	
	Acropomatiformes			228
		Epigonidae	43	
		Banjosidae	1	
		Pentacerotidae	13	
		Polyprionidae	4	
		Acropomatidae	31	
		Howellidae	9	
		Pempheridae	30	
		Glaucosomatidae	4	
		Ostracoberycidae	3	
		Lateolabracidae	2	
		Bathyclupeidae	7	
		Dinolestidae	1	
		Hemerocoetidae	24	
		Trichonotidae	10	
		Creediidae	18	
		Champsodontidae	13	
		Scombropidae	3	
		Symphysanodontidae	12	
	Labriformes			687
		Gerreidae	55	
		Labridae	632	
	Uranoscopiformes			172
		Centrogenyidae	1	
		Leptoscopidae	5	
		Pinguipedidae	84	
		Cheimarrichthyidae	1	
		Ammodytidae	28	
		Uranoscopidae	53	
	Centrarchiformes			292
		Centrarchidae	45	
		Sinipercidae	13	
		Cirrhitidae	35	
		Chironemidae	6	
		Aplodactylidae	5	
		Cheilodactylidae	27	
		Latridae	7	
		Percichthyidae	24	
		Perciliidae	2	
		Enoplosidae	1	
		Terapontidae	54	
		Kuhliidae	13	
		Kyphosidae	50	
		Parascorpididae	1	
		Oplegnathidae	7	
		Dichistiidae	2	
	Acanthuriformes			2216
		Callanthiidae	14	
		Sillaginidae	34	
		Dinopercidae	2	
		Lethrinidae	41	
		Nemipteridae	69	
		Sparidae	139	
		Centracanthidae	9	
		Emmelichthyidae	16	
		Luvaridae	1	
		Zanclidae	1	
		Acanthuridae	85	
		Malacanthidae	45	
		Hapalogenyidae	8	
		Haemulidae	132	
		Lutjanidae	132	
		Sciaenidae	291	
		Leiognathidae	50	
		Chaetodontidae	131	
		Pomacanthidae	88	
		Monodactylidae	6	
		Drepaneidae	3	
		Ephippidae	15	
		Lobotidae	2	
		Datnioididae	5	
		Moronidae	6	
		Siganidae	32	
		Cepolidae	23	
		Scatophagidae	4	
		Priacanthidae	19	
		Caproidae	18	
		Lophiidae	28	
		Antennariidae	49	
		Tetrabrachiidae	2	
		Lophichthyidae	1	
		Brachionichthyidae	14	
		Chaunacidae	22	
		Ogcocephalidae	75	
		Caulophrynidae	5	
		Neoceratiidae	1	
		Melanocetidae	6	
		Himantolophidae	22	
		Diceratiidae	6	
		Oneirodidae	64	
		Thaumatichthyidae	8	
		Centrophrynidae	1	
		Ceratiidae	4	
		Gigantactinidae	23	
		Linophrynidae	27	
		Triacanthodidae	23	
		Triacanthidae	7	
		Balistidae	42	
		Monacanthidae	109	
		Aracanidae	13	
		Ostraciidae	27	
		Triodontidae	1	
		Tetraodontidae	193	
		Diodontidae	18	
		Molidae	4	
