# Supplementary material for: Repeated and Widespread Evolution of Bioluminescence in Marine Fishes
Source: PLoS One. 2016 Jun 8;11(6):e0155154. doi: 10.1371/journal.pone.0155154 (PMC4898709; doi:10.1371/journal.pone.0155154)
Supplement: S1 Text — (PDF) [file pone.0155154.s005.pdf]

Supplementary Information

Fossil calibrations.—For further information and literature regarding the fossil calibrations, please refer to the relevant citations associated with each previously published calibration. All calibrations use soft upper bounds that represent conservative estimates based on the age of known fossil lineages that are hypothesized to represent a more stem lineage in the evolutionary history of ray-finned fishes. As a result of using these soft upper bounds for the lognormal priors, the parameters associated with each calibration have been slightly modified from their original source.

Calibration 1 (Modified from Near et al., 2012; calibration 1). Node: Crown-group Actinopterygii. First occurrence: †*Mimipiscis toombsi* and †*Moythomasia durgaringa*. Stratigraphy: lower Frasnian. Absolute age estimate: 382.5 Ma. The upper bound is based on the age of stem chondrichthyian †*Elegestolepis conica*.

Absolute age offset = 382.5 Ma

95% soft upper bound = 438 Ma

Log(Mean) = 2.17

Log(StDev) = 0.8

Calibration 2 (Modified from Near et al., 2012; calibration 2). Node: Crown-group Actinopteri. First occurrence: †*Cosmoptychius striatus*. Stratigraphy: Asbian regional

stage, but often correlated with the upper Viséan. The upper bound is based on the age of †*Mimipiscis toombsi* and †*Moythomasia durgaringa*.

Absolute age offset = 325.5 Ma

95% soft upper bound = 382.5 Ma

Log(Mean) = 2.475

Log(StDev) = 0.8

Calibration 3 (Modified from Arratia 2013). Node: Stem lineage Teleostei, dating the MRCA of Teleostei and *Amia*. Occurrence: †*Prohalecites*, the oldest stem teleostomorpha. Stratigraphy: Middle/Upper Triassic, Ladinian-Carnian boundary. The soft upper bound is based on the age of †*Cosmoptychius striatus*.

Absolute age offset = 237 Ma

95% soft upper bound = 325.5 Ma

Log(Mean) = 2.915

Log(StDev) = 0.8

Citation: Arratia G (2013) Morphology, taxonomy, and phylogeny of Triassic pholidophorid fishes (Actinopterygii, Teleostei). Jour Vert Paleo 33(6): 1–138.

Calibration 4 (Modified from Near et al., 2012; calibration 5). Node: Stem lineage Notopteridae, dating the MRCA of *Gymnarchus*, *Gnathonemus*, *Xenomystus*, and *Chitala*. First occurrence: †*Palaeonotopterus greenwoodi*. Stratigraphy: uppermost

Albian- lowermost Cenomanian. The upper bound is based on the age of stem teleost †*Pholidophorus*.

Absolute age offset = 99.6 Ma

95% soft upper bound = 220 Ma

Log(Mean) = 3.223

Log(StDev) = 0.8

Calibration 5 (Modified from Near et al., 2012; calibration 6). Node: Stem lineage Chanidae, dating the MRCA of *Chanos* and *Cromeria*. First occurrence: †*Rubiesichthys gregalis*. Stratigraphy: Berriasian-Valanginian. The upper bound is based on the Tithonian stem-lineage ostariophysian †*Tischlingerichthys viohi*.

Absolute age offset = 133.9 Ma

95% soft upper bound = 150 Ma

Log(Mean) = 1.26

Log(StDev) = 0.8

Calibration 6 (Modified from Davis and Fielitz, 2010). Node: Stem Lineage Euteleostei, dating the MRCA of Euteleostei, Alepocephaloidei, Clupeiformes, and Ostariophysi. First occurrence: †*Leptolepides sprattiformis*. Stratigraphy: Late Jurassic, Early Tithonian (Malm Z2) deposits. The upper bound is based on the age of stem teleost †*Pholidophorus*.

Absolute age offset = 150 Ma

95% soft upper bound = 220 Ma

Log(Mean) = 2.345

Log(StDev) = 0.8

Calibration 7 (Modified from Near et al., 2012; calibration 10). Node: Stem lineage Salmoninae, dating the MRCA of *Thymallus*, *Salvelinus*, and *Coregonus*. First occurrence: †*Eosalmo driftwoodensis*. Stratigraphy: middle Ypresian, U-Pb zircon dated to approximately 51.8 Ma. The upper bound is based on the Tithonian stem-lineage ostariophysian †*Tischlingerichthys viohi*.

Absolute age offset = 51.8 Ma

95% soft upper bound = 150 Ma

Log(Mean) = 3.019

Log(StDev) = 0.8

Calibration 8 (Modified from Davis and Fielitz, 2010). Node: Stem lineage Alepisauridae, dating the MRCA of *Anotopterus*, *Alepisaurus*, and *Omosudis*. First occurrence: †*Enchodus brevis* and †*Saurorhamphus freyeri*. Stratigraphy: Upper Cenomanian. The upper bound is based on the stem-lineage euteleost †*Leptolepides sprattiformis*.

Absolute age offset = 100 Ma

95% soft upper bound = 150 Ma

Log(Mean) = 2.344

Log(StDev) = 0.8

Calibration 9 (Modified from Davis and Fielitz, 2010). Node: Stem lineage Myctophiformes, dating the MRCA of Myctophiformes and Acanthomorpha. First Occurrence: †*Sardinoides.* Stratigraphy: Middle Cenomainian of Hakel and Middle Cenomainian of Hajula deposits. The upper bound is based on the stem-lineage euteleost †*Leptolepides sprattiformis*.

Absolute age offset = 96 Ma

95% soft upper bound = 150 Ma

Log(Mean) = 2.421

Log(StDev) = 0.8

Calibration 10 (Modified from Near et al., 2012; calibration 11). Node: Stem lineage Polymixiiformes, dating the MRCA of *Polymixia* and Percopsiformes (*Percopsis*, *Aphredoderus*, and *Chologaster*). First occurrence: †*Homonotichthys dorsalis*. Stratigraphy: middle-upper Cenomanian. The conservative upper bound is based on the minimal age of stem teleost †*Pholidophorus*.

Absolute age offset = 93.6 Ma

95% soft upper bound = 220 Ma

Log(Mean) = 1.88

Log(StDev) = 0.8

Calibration 11 (Modified from Near et al., 2012; calibration 12). Node: Stem lineage Percopsidae, dating the MRCA of Percopsiformes (*Percopsis*, *Aphredoderus*, and *Chologaster*). First occurrence: †*Massamorichthys wilsoni*. Stratigraphy: Thanetian, or middle Tiffanian NALMA. The upper bound is based on the minimal age of †*Homonotichthys dorsalis*.

Absolute age offset = 57 Ma

95% soft upper bound = 93.6 Ma

Log(Mean) = 2.032

Log(StDev) = 0.8

Calibration 12 (Modified from Near et al., 2012; calibration 13). Node: Stem lineage Aphredoderidae, dating the MRCA of *Aphredoderus* and *Chologaster*. First occurrence: †*Trichophanes foliarum*. Stratigraphy: upper Priabonian, dated to approximately 34.1 Ma. The upper bound is based on the minimal age of †*Massamorichthys wilsoni*.

Absolute age offset = 34.1 Ma

95% soft upper bound = 57 Ma

Log(Mean) = 1.563

Log(StDev) = 0.8

Calibration 13 (Modified from Near et al., 2012; calibration 16). Node: Stem lineage Lampridae dating the MRCA of *Lampris*, *Regalecus*, and *Trachipterus*. First occurrence: †*Turkmene finitimus*. Stratigraphy: uppermost Thanetian-lowermost Ypresian. The conservative upper bound is based on the minimal age of the oldest aulopiform †*Atolvorator longipectoralis*.

Absolute age offset = 55.8 Ma

95% soft upper bound = 125 Ma

Log(Mean) = 2.669

Log(StDev) = 0.8

Calibration 14 (Modified from Near et al., 2012; calibration 14). Node: Stem lineage Zeiformes, dating the MRCA of Zeiformes (*Cyttopsis*, *Zenopsis*, and *Zeus*), Gadiformes (*Lota* and *Coryphaenoides*), and *Stylephorus chordatus*. First occurrence: †*Cretazeus rinaldii.* Stratigraphy: latest Campanian-earliest Maastrichtian. The conservative upper bound is based on the minimal age of the oldest aulopiform †*Atolvorator longipectoralis*.

Absolute age offset = 70.6 Ma

95% soft upper bound = 125 Ma

Log(Mean) = 2.4278

Log(StDev) = 0.8

Calibration 15 (Modified from Near et al., 2012; calibration 15). Node: Stem lineage *Zenopsis*, dating the MRCA of *Zenopsis* and *Zeus*. First occurrence: †*Zenopsis clarus*, †*Zenopsis tyleri*, and †*Zenopsis hoernesi*. Stratigraphy: lower Rupelian [P18], lower Khadumian regional stage. The soft upper bound is based on the minimal age of †*Cretazeus rinaldii.*

Absolute age offset = 32 Ma

95% soft upper bound = 70.6 Ma

Log(Mean) = 2.0852

Log(StDev) = 0.8

Calibration 16 (Modified from Near et al., 2012; calibration 17). Node: Stem lineage Trachichthyoidei dating the MRCA of Beryciformes. First occurrence: †*Hoplopteryx lewesiensis* and †*Hoplopteryx simus*. Stratigraphy: middle-upper Cenomanian, zone of Holoaster subglobosus. The conservative upper bound is based on the minimal age of the oldest aulopiform †*Atolvorator longipectoralis*.

Absolute age offset = 93.6 Ma

95% soft upper bound = 125 Ma

Log(Mean) = 1.88

Log(StDev) = 0.8

Calibration 17 (Modified from Near et al., 2012; calibration 23). Node: Stem lineage Carangidae, dating the MRCA of Carangidae (*Caranx*, *Seriola*, and *Trachinotus*), *Echeneis*, *Coryphaena*, and *Rachycentron*. First occurrence: †*Archaeus oblongus*. Stratigraphy: uppermost Thanetian-lowermost Ypresian. The conservative upper bound is based on the minimal age of the oldest aulopiform †*Atolvorator longipectoralis*.

Absolute age offset = 55.8 Ma

95% soft upper bound = 125 Ma

Log(Mean) = 2.669

Log(StDev) = 0.8

Calibration 18 (Modified from Near et al., 2012; calibration 24). Node: Stem lineage Echeneidae, dating the MRCA of *Echeneis*, *Coryphaena*, and *Rachycentron*. First occurrence: †*Opisthomyzon glaronensis* and unnamed echeneid cf. *Echeneis*. Stratigraphy: Engi slates: Rupelian. The conservative upper bound is based on the minimal age of the oldest aulopiform †*Atolvorator longipectoralis*.

Absolute age offset = 30.1 Ma

95% soft upper bound = 125 Ma

Log(Mean) = 2.985

Log(StDev) = 0.8

Calibration 19 (Modified from Near et al., 2012; calibration 26). Node: Stem lineage Siganidae, dating the MRCA of *Siganus* and Scatophagidae (*Scatophagus* and *Selenotoca*). First occurrence: †*Siganopygaeus rarus*. Stratigraphy: uppermost Thanetian- lowermost Ypresian. The conservative upper bound is based on the minimal age of the oldest aulopiform †*Atolvorator longipectoralis*.

Absolute age offset = 55.8 Ma

95% soft upper bound = 125 Ma

Log(Mean) = 2.669

Log(StDev) = 0.8

Calibration 20 (Modified from Near et al., 2012; calibration 33). Node: Stem lineage Diodontidae, dating the MRCA of Diodontidae (*Diodon* and *Chilomycterus*) and *Tetraodon*. Stratigraphy: upper Ypresian. The conservative upper bound is based on the minimal age of the oldest aulopiform †*Atolvorator longipectoralis*.

Absolute age offset = 50 Ma

95% soft upper bound = 125 Ma

Log(Mean) = 2.75

Log(StDev) = 0.8

Calibration 21 (Modified from Near et al., 2012; calibration 35). Node: Stem lineage of Balistidae, dating the MRCA of *Abalistes* and *Cantherhines*. First occurrence:

†*Gornylistes prodigiosus*. Stratigraphy: Bartonian [NP17] Kumian regional stage. The conservative upper bound is based on the minimal age of the oldest aulopiform †*Atolvorator longipectoralis*.

Absolute age offset = 37.2 Ma

95% soft upper bound = 125 Ma

Log(Mean) = 2.907

Log(StDev) = 0.8

Alignment in Fasta Format

```
>Polypterus ornatipinnis
AGCCTCCTAATTCGCGCAGAACTAGGCCAACCAGGAGCCCTAATAGGAGA
TGACCAAATTTATAATGTTGTCGTCACTGCACATGCATTTGTAATAATTT
TCTTTATAGTAATGCCAATCATAATCGGAGGATTCGGTAACTGACTTGTA
CCACTAATAATTGGAGCCCCAGATATGGCCTTCCCACGAATAAATAACAT
AAGTTTCTGATTACTTCCACCATCGCTTCTCCTGCTACTAACCTCTTCTG
CAGTAGAGGCCGGTGTCGGAACAGGATGAACTGTATATCCCCCATTAGCC
GGGAATCTAGCACATGCAGGGGCATCAGTTGATTTAGCAATTTTCTCACT
CCATTTAGCCGGTGTTTCCTCAATTCTTGGGGCAATTAACTTCATTACTA
CAATTATTAATATGAAACCGCCATCCACCTCACAATATCAAACCCCCCTA
TTCGTATGATCAGTATTAGTCACTGCAGTCCTCTTACTCCTATCCCTTCC
AGTTTTAGCCGCCGGAATTACAATACTACTAACAGATCGAAATTTAAACA
CTACCTTCTTTGACCCTGCTGGCGGAGGAGATCCAATTCTTTACCAACAT
TTATTCTGATTTTTTGGCCATCCAGAAGTATACATTTTAATTCTTCCAGG
ATTTGGTATAGTCTCTCATATCGTAGCTTACTATTCAGGTAAAAATGAAC
CTTTCGGTTATATAGGAATAGTATGAGCAATAATAGCGATTGGACTCCTA
GGATTTATCGTATGAGCCCACCACATATTTACAGTTGGTATAGACGTTGA
TACTCGAGNNNN------------------------------------------
--------------------------------------------------
--------------------------------------------------
--------------------------------------------------
--------------------------------------------------
--------------------------------------------------
--------------------------------------------------
--------------------------------------------------
--------------------------------------------------
--------------------------------------------------
--------------------------------------------------
--------------------------------------------------
--------------------------------------------------
```


---------------------GGAAGAGTACATTGTGGTTTTCAGCCGCA
CCATCAACAGACTCATTCTCAACGAGGCCGAGCTCATTTTGGCACTTGCT
CAAGAGTTTCAGATGAAAACAGTCACCGTGTCATTGGACGACCACTCTTT
TGCTGACGTAGTGCAGATCATTAGCAAGGCTTCCATGCTGATTAGCATGC
ACGGTGCCCAACTCATTTCGTCTATCTTCCTCCCCAGAGGAGCTGTCGTC
GTGGAGCTTTTCCCCTATGCAGTGAATCCAGATCATTACACACCTTATAA
AACTTTGGCCTCTCTGCCTGGAATGGATCTCCAGTACGTTGCATGGCAAA
ACACTATAGAAGAAAATTCTGTGGCTTTCCCGGACAGGCCCTGGGATCAA
GGGGGCATAGGTCATTTGGAAAAAGAAGAGCAGATACGCATTATGAAAAG
TGAAGAAGTCCCTCGGCACCTCTGTTGCCGTAACCCCGAATGGCTCTATC
GCATTTATCAGGATACAAAAGTGGACATACCCTCTCTGATCGAAACCATC
CG---TCAGAGCATTAAA---ACAAAGCCTAGTGTGAAAAA---GGTAAA
GATGGCCAGTGTTGTTCACCCCGGGAAAGTTAGGGAGGCCAAATGCCAAG
CTTCCGTCCATGGTACCAATGAAGCCAAGCTGACTGTTTCCTGGCAGATT
CCTTGGAATNNNNNNNNNNNNNNNNNNNNNNNNNNNNNNNNNNNNNNNNNNNN-------------
----

----NNNNNNNNNNNNNNNTTCAAATGTGAAGAGTGCGGAAAGAATTACAATACCAAGATGGGAT
ACAAGCGGCATATCGCCATGCATGCGGCCACCAGTGGGGACCTCACCTGT
AAGGTTTGCATGCAGAGTTATGACAGCACTCCTGTCCTTCTGGAGCACTT
GAAGAGCCACTCTGGGAAGTCTTCAGGCAGCGTAAAAGAGAAAAAGCACC
CCTGTGATCACTGCGATCGCCACTTCTATACCCGGAAAGATGTTCGCCGT
CACATGGTGGTTCACACAGGTCGCAAAGACTTTCTCTGCCAGTACTGTGC
TCAGCGCTTTGGTCGGAAAGACCACTTGACTCGCCACGTGAAGAAGAGCC
ACTCACAGGAGTTACTCAAAATCAAAACAGAGCCACAGGATATGCTAGGA
ATTCTGAATTGTGGTTCCTCTGTTT---CAGTAAAGGAGGAACTCACTCC
CATGATGTG---CATGGCATCTTCTAAGGACTCTATGATGGGAAAATCCT
TTTCCAATA------CCTTTTCTGTGGGCATGTACAACCCGCAC------
---TTGCCAACCATGTCCAATTCTGGGATGTCT---------------CA
TACTTTAGTACCCAGTTCATTG---
GCTNNNNNNNNNNNNNNNNNNNNNNNNNNNNNNNNNNNNNNNNNNNNNNNNNNNNNNNNNNNNNNTTAC
AAATAAGTGATGGATCTA
ACATTGTCAATCTTTTAGCCAGTGACTCACCCAGTGTCTCCTATGCACTA
ACACAGCAAAAATACTTTAGCAACTACAGCCCCGTCATTGGGTTCTATAT
ATATGAGCCTATTGAGTACTGGAACAGCACAGTACAAGAACATCTAAAAA
CATTAGGCCATGGGTTTCATAAAATAGCTTGGATTGACCATTACTTTCAG
TATTTAAAAGTTATTAATATCAGTGCAGCAACCAAAACTGATTTTATCAC
AGTTCTTCAGAATTCATTCTTGAAGAAACCTGAATTTCAGCACTTTAGGG
AAGACATCATTTTTTCTAAGA---CGGGGG------------ATGAAATA

AACATTATTGCATCACGGATGTACATGGTTGCCAGAACTAGTGTGAATAC
AAGGGAAGAAGTTGTGGAGCTCCTGGAGGGACTGAGGCCGCTGTCCCTCA
TAAACAGCATCAAGTTCATTGTCTTCAACCCAACTTTTGTCTTCATGGAT
CGCTACAGTTCTTCCATCATATCTCCTATCCTGACCTCTGGATTTAGCAT
CTTGATTGTGCTAATACTGACAGTCTTCCTAGTGATCCATCCACTTGGCA
ACTTTTGGTTGATTCTAACAGTTACCTCTNNNNNNNNNNNNNNNNNNNNNNNNNNNNNNNNNNNNNNNNN
NNNNNNNNNNNNNNNNNNNNNNNNNNNNNNNNNNNNNNNNNNNNNNNNNNNNNNNNNNNNNNNNNNNNNN
NNNNNNNNNNNNNNNNNNNNNNNNNNNNNNNNNNNNNNNNNNNNNNNNNNNNNNNNNNNNNNNNNNNNNN
NNNNNNNNNNNNNNNNNNNNNNNNNNNNNNNNNNNNNNNNNNNNNNNNNNCATGAGTTGGATGATTCC--
-
TCCACCGAAATTTTTACTGTGACAGTAAAAGAGTCATGTGATGGAATGGG
AGATGTAAGTGAAAAACACGGCGGTGGTCCAGCACTACCAGAAAAAGCTG
TGCGCTACTCTTTCACGATTATGTCAATTGCGACCGAGATGAAAGAGGGC
AAC---------------------------------AACGTTATGATCTT
TCAAGAGTCCAAGCCTAATTCTGAACTCTGCTGCAAGCCACTCTGCCTGA
TGTTCGCTGATGAATCTGATCATGAGACATTAACAGCCATCTTAGGCCCT
GTGGTGGCCGAAAGAGAAGCTATGAAGGAGAGTCGATTAATTTTAGACAT
TGGTGGACTTTCTCGGTGTTTTCGCTTTATTTTCCGAGGAACAGGCTACG
ATGAAAAACTGGTAAGAGAATTAGAGGGATTAGAAGCCTCTGGGTCGACG
TATATATGCACACTTTGTGACTCTACCAGGGTTGAAGCATCCAGAAACAT
GATTCTCCACGCCATTACTAGAAGTCACGAAGAGAACTTGGAACGTTACG
AGATCTGGAGGTCCAACCCATTCTCAGAGTCTGCTGATGACCTCCGGGAC
AGAGTCAAAGGAGTTTCAGCCAAACCTTTCATGGAGACCCACCCTTCCAT
TGATGCACTCCACTGTGACATTGGAAATGCGGCTGAGTTTTACAAGATTT
TCCAGGATGAAATTGGGGAAGTCTTCCAAAATCC---CAAC---CCTACA
AAAGAGGAAAGAAAAAGATGGCAATCGGCTCTTGACAAGCAACTGAGAAA
AAAGATGAACTTAAAACCAATTATGAGAATGAACGGAAATTTTGCTCGCA
AACTCATAACCAGTGAAGCAGTGGAAGCAATTTGTGAACTCGTTCCCTCA
GAGGAGCGCAAAGAAGCTCTTCGAGAACTCGTGAACCTCTACCTTCAAAT
GAAACCTGTCTGGCGTTCAACTTGTCCACTTAAAGAATGTCCCGACCTGT
TATGTCGATATAGTTTTAACTCGCAGCGCTTTGCGGAGCTTCTCACCTCC
ACCTTCAGTTATCGCTATGATGGAAAGATTACCNNNNNNNNNNNNNNNNNNNNNNNNNNNNNNNNNNNNN
NNNNNNNNNNNNNNNNNNNNNNNNNNNNNNNNNNNNNNNNNNNNNNNNNNNNNNNNNNNNNN--------
----

--------------------------------------CCTCTCACGACA
TTCCTCAAATTGACCTCCCTGGGCTTCATCATTGGGGTCGGCGTGGTTGG
TAACCTCTTGATCTCCATCCTGCTGGTGAAAGACAAAACCCTGCACAGAG
CCCCCTACTACTTTCTGTTCGACCTGTGCGTCTCGGACATACTGCGATCT
GCCATCTGTTTTCCGTTCGTGTTCACCTCTGTCAAAAACGGCTCCTCGTG
GACGTATGGGACGCTCACCTGTAAAGTGATTGCCTTCCTAGGAGTGTTGT

CTTGCTTTCACACGGCTTTTATGCTGTTTTGCATTAGTGTCACCCGATAC
CTCGCCATAGCCCATCACCGCTTTTACACGAAAAGATTAACCTTCTGGAC
CTGCCTGGCTGTGATCTGTATGGTTTGGACGCTCTCGGTCGCTATGGCTT
TCCCGCCTGTACTGGATGTGGGGACCTACTCATTTATCCGGGAGGAAGAT
CAGTGTACCTTTCAGCATCGCTCATTCAGGGCTAACGATTCGCTGGGATT
TATGCTGCTCTTGGCCCTCATCCTACTAGCCACCCAGCTGGTCTACCTCA
AGCTCATCTTTTTTGTGCACGACCGGAGGAAGATGAAACCCGTTCAGTTT
GTGCCAGCTGTCAGCCAGAACTGGACCTTTCATGGTCCGGGGGCTAGCGG
CCAGGCGGCGGCCAACTGGTTAGCTGGCTTCGGCAGAGGCCCTACACCGC
CTACTCTTCTGGGAATAAGACAAAACACCAACACCACGGGCAGAAGAAGA
CTGCTCGTCCTGGATGAGTTTAAAACTGAAAAGCGAATAAGCAGGATGTT
CTACATCATGACGTTCTTCTTTTTGACCCTGTGGGGCCCCTATCTTGTTG
CCTGCTACTGGAGAGTGTTTGCTAGGGGGCCGGCGGTGCCAGGGGGTTAC
CTGACGGCCGCCGTCTGGATGAGTTTTGCCCAGGCTGGTGTCAATCCCTT
TATCTGCATCTTTTCCAACAGGGAG-------------------------

----NNNNNNNNNGTCACCGACAGAGAAGTGGGGTTGGGGATAAATCCATTTGCTGATG
GGATGGGCGCTTTTAAAATCAACCACAGTACCCACGATCTTGCATCTGG-
--TCAAACGGCTTTTTCATCCCAGGCG---CCGGGGTAC---GCAGCGGC
AGCGCTGGGG---CATCATCA------CCACCCCTCTCATGTCAGCTCT-
--TATTCCACCGCAGCTTTCAATTCCACTCGGGACTTTCTTTTCCGAAAT
CGGGGATTTGGTGAAGCAGCCAG------------CGCCCAACATAGCCT
GTTTGCCTCTGC---CGCGGGAAGTTT---T------GCGGGGCCCCATG
GACACACAGATGCTGCTGGACATTTGCTTTTCCCGGGACTTCACGAG---
CAGGCGACAACTCATGCGTCTCCCAACGTTGTTAACAGTCAAATGCGATT
AGGCTTTTCCGGAGACATGTATGGCAGAGCAGACCAGTACAGCCAGGTAA
CAAGCCCCAGAT---CAGACCATTATGCTTCGACCCAGCTACACAGCTAC
GGACCCATGAACATGAATATGGCTGCA---CACCATGGGGCAGGGGCCTT
TTTTCGTTATATGAGGCAGCCAATAAAGCAAGAACTTATCTGTAAATGGA
TAGAACCTGAACAGTTGACAAACCCGAAAAAGTCTTGTAACAAAACTTTT
AGCACAATGCACGAACTAGTAACTCACCTCACAGTGGAACATGTGGGGGG
ACCAGAGCAAGCCAACCATATCTGCTTTTGGGAAGAATGCGCACGAGAAG
GAAAGCCATTTAAAGCCAAGTATAAGCTTGTAAATCACATACGAGTCCAC
ACAGGTGAAAAACCTTTTCCTTGTCCANNNNNNNNNNNNNNNNNN
>Polyodon spatula
AGTCTACTGATTCGTGCCGAACTAAGCCAACCCGGTGCCTTACTTGGCGA
TGATCAAATCTATAATGTTATCGTCACAGCCCACGCCTTTGTCATGATTT
TCTTTATAGTAATACCCATCATAATCGGCGGATTCGGAAATTGACTGGTC
CCCCTGATAATCGGAGCTCCAGATATAGCCTTCCCTCGCATGAATAATAT
GAGTTTCTGGCTCCTACCCCCATCCTTCCTTCTCCTTTTGGCCTCCTCTG

GGGTAGAGGCCGGAGCCGGCACCGGGTGAACTGTCTACCCTCCTCTGGCA
GGAAACCTAGCCCACGCAGGAGCCTCTGTAGACCTTACCATCTTCTCCCT
ACACTTGGCTGGGGTGTCGTCTATCCTAGGGGCTATTAATTTTATCACTA
CGATTATCAACATAAAACCTCCCGCAGTATCTCAGTATCAAACCCCCCTA
TTCGTGTGATCTGTACTAGTTACAGCTGTACTCCTCCTCCTATCACTACC
AGTTCTAGCTGCAGGAATTACAATACTGCTGACAGATCGAAATTTAAATA
CCACATTCTTTGACCCCGCCGGAGGGGGAGACCCCATCCTGTATCAGCAC
CTGNNNNNNNNNNNNNNNNNNNNNNNNNNNNNNNNNNNNNNNNNNNNNNNNNNNNNNNNNNNNNNNNNNN
NNNNNNNNNNNNNNNNNNNNNNNNNNNNNNNNNNNNNNNNNNNNNNNNNNNNNNNNNNNNNNNNNNNNNN
NNNNNNNNNNNNNNNNNNNNNNNNNNNNNNNNNNNNNNNNNNNNNNNNNNNNNNNNNNNNNNNNNNNNNN
NNTTCCTGGAGAAGAACCTGCATCCCACCAACTGCCTGGGCATGCTGCT
TTTCTCGGATGCCCATCAGTGCACCAGGCTGTTCGAGCTCTCCTGGGGGA
TGTGCCTGAGCAACTTCCCAGCCATCAGCAAGTCTGAGGACTTCCTGCAG
CTGCCCAAAGACATGCTGGTGCAGCTGCTCTCCAATGAGGAGCTGGAGAC
AGAGGACGAAAGGGTTGTTTACGATTCTGCCCTCAACTGGGTGAACTACG
ACCTGAAGAAGAGGCACTGCTACCTCCCAGAGCTGCTGCGCACAGTCAGG
CTGGCCCTCCTACCTGCCATCTTTCTGATGGAGAATGTCTCCACAGAGGA
GCTCATCAACAAGCAGGCCCAGAGCAAGGCGCTGGTGGAGGAGGCTATCC
TCTGTAAGCTAAAGATCTTGCAGAATGACGGGGTGGTCACTAGCTTGTGT
GCCCGTCCCCGCAAGACCAGTCATGCCCTGTTCCTGTTGGGAGGCCAGAC
GTTCATGTGTGACAAACTCTACCTGGTGGACCAGAAAGCAAAAGAGATCA
TCCCCAAGACAGACATCCCCAGCCCTCGCAAGGAGTTCAGTGCCTGTGCC
ATCGAGTGCAAGGTCTACATCACTGGGGGCCGGGGCTC---AGAAAATGG
GGTGTCAAAAGATGTTTGGGTCTACGACACCCTGCATGAGGAGTGGTCCA
AGGCAGCCCCCATGCTTATCGCCAGGTTTGGCCATGGCTCTGCAGAGCTT
AAGCATTGTCTGTATGTAGTAGGTGGACACACAGCAGCCACAGGGTGCCT
CCCAGCATCTCCATCA----------------------------------

---CCC
AACAAGGAGGCCTTCAAGTGTGAGGAGTGCGGCAAGAACTACAACACCAA
GCTGGGCTACAAGCGCCACGTGGCCATGCATGCTGCCACCAACGGAGACC
TGACCTGCAAGGTGTGCCTGCAGAGCTACGAGAGTACGCCCGTGCTACTG
GAGCACCTCAAGAGCCACTCTGGTAAGTCCTCGGGCGGCGTGAAGGAAAA
GAAGCATCCCTGCGACCACTGCGACCGCCGCTTCTACACCCGGAAGGATG
TGCGCCGCCACATGGTGGTCCACACGGGCCGCAAGGACTTCCTATGCCAG
TACTGTGCCCAGCGCTTTGGCCGCAAGGACCACCTGACCAGGCACGTCAA
GAAGAGCCACTCCCAGGAGCTGCTAAAGATCAAGACAGAGCATCCCGACA
TGCTGGGGCTGCTCCACTCCAGCTCGCCCATCT---CTGTGAAGGAGGAG
CTCAGCCCCATGATGTG---CATGCCCCCTTGCAAAGACTCCATGATGGG
CAAGCCCTTTGCCAGCT------CTTTCCCCATGAGCATGTACAACCAGC
AC---------CTGCAGTCCATGTCCAACTCAGGGGTCTCG---------
------CACACCTTGGTGCCCAGTTCGCTG---GCCACCATGGGGATGGG
TTGCCCCATGGAGTATCTAATTTATGCATCATTTTCTTTCATGGGGTGTT
TGCAAATTAGTGATGGATCAAACATAGTCAACCTCTTACCTAGTGACTCG
CCAAGTGTCTCCTATGCACTGACCCAGCAAAAATACTTCAGTAACTACAG
TCCTGTAATTGGGTTCTATATTTATGAGCCGATAGAGTACTGGAACACAA
CCGTACAAGAACATTTAAATACATTAGGTCATGGGTTTAATAGAATATCA
TGGATTGATCATTACTTTCAGCATTTAAAAGTCATCAATATTACTACTCC
AACCAAAAGCGATTTTATAACTGTTCTGCAAAATTCATTCTTGATAAAAA
AAGAATTTCAGCACTTTAAAGATGACATCATTTTTTCCAAAA---CAGGG
G------------ATGAAGTTGATATCATTGCATCCAGGATGTATATGGT
TGCCAGAACCAGCGAGAACACAAGGGAAGAAGTCGTTGAATTGCTGGAAA
GGCTAAGGCCATTGTCTCTTATCAACAGCATCAAATTTATTGTGTTTAAT
CCTACCTTTGTGTTCATGGATCGCTACAGCGCCTCCATCATATCACCCAT
TGTGACGTCTGGATTTAGTGTCCTGATTGCCTTAGTCCTGACCGTCTTCC
TAGTTATTAATCCACTTGGTAACTTTTGGTTGATAATTACAGTCACCTCA
GTAGAGTTAGGCGTTCTTGGCTTGATGGGATACCACCCGTTTGAGTGGCA
GCCAGCCCTGAAGAATGTCTCTAGCAGCACAGATGTGGGGATTATTGATG
GGTTGTCAGGCTGGACAGTTTCTGTGGAGGACTCTCAAATGGACACTATT
GCTCGCAGGTTCCGCTATGATGCTGCCTTGGTTTCAGCCTTAAAAGACCT
AGAGGAGGACATCCTGGAAGGCATTAATGAGCAAGGACTGGACGAATTC-
--TCCACAGAGGTCTTCTCAGTGGTGATCAAGGAATCATGTGATGGAATG
GGCGATGTGAGTGAGAAACACGGAGGTGGACCATCGCTCCCAGAGAAAGC
TATCCGCTTCTCCTTCACCATCATGTCTATTGCCACCAAGAACGAAGATG
GAGAA-----------------------------------AACATAAATGTC
TTCCAGGAACACAAGCCAAACTCAGAGCTCTGCTGCAAACCCATGTGTTT
GATGTTTGCAGATGAATCTGACCATGAGACACTGACAGCTATTCTGGGTC
CAGTGCTGGCCGAACGGGAAGCCATGAAAGAGAGTCGCCTGATCCTGGAG
ATTGGTGGCCTGTCTAGGTCTTTTCGATTTATCTTTAGGGGCACAGGGTA
TGACGAGAAGCTGGTGAGGGATGTTGAAGGATTGGAGGCCTCAGGGTCAA
CCTATATCTGTACCCTTTGTGATGCCACCAGAGCCGAAGCTGCCCAGAAT
ATGGTTCTTCACTCTGTCACCAGGAACCATGAGGAAAACCTAGATCGCTA
TGAGATATGGCGGTCCAATCCCTACTCAGAGTCAGTGGATGAACTCCGTG
ACAGAGTGAAGGGAGTTTCAGCTAAGCCCTTTATGGAAACCCAGCCTTCC
ATTGATGCCCTGCACTGTGACATTGGCAATGCCACAGAGTTCTACAAAAT
ATTCCAGGATGAAATAGGGGAAGTCTACCAGAATTA---CAAC---CCTA
CCCACGAGGAGAGGAAAAGGTGGCAATCTGCCCTAGATAAACAGCTGAGG
AAGAAGATGAACCTTAGACCTGTCATGAGGATGAATGGCAATTTTGCCCG
CAAGTTGATGACTAAGGAGACAGTAGAAGCTGTTTGTGAGCTTGTCCCAT
CTCAAGAACGAAGAGAGGCCCTCAGGGAGCTTGTGCACCTGTACCTCCAA
ATGAAGCCTGTCTGGCGTGCCAACTGCCCAGCCAAAGAGTGCCCAGACTT

```
GCTCTGCCGTTACAGCTTCAACTCACAGCGCTTTGCAGAGCTCCTCTCCA
CTACCTTCAAGTACAGATATGATGGAAAGATCACCAACTACCTCCACAAG
ACCTTGGCCCATGTGCCTGAGATCATTGAAAGGGAGGGTTCCATAGGCGC
CTGGGCCAGTGAGGGCAATGAGTCCGGGAACAAGACATACGCCATTGAGA
TGGGCACCAGAGGACCCCAGTGGAGTGCCAACCCAAGGCCCTTTGTCTGC
TMCATCGAGGAGCCGATCAAGCAGACCAAATTCAAGGGCATCAAGACCTA
CATCTCCTACCGGGTGTCCCCCAGCCACACAGGCAGYCCTGTCTACCGSC
GCTACAAGCACTTCGACTGGCTCTACAACCGGCTGCTGCACAAGTTCACC
GTCATCTCAGTACCYCACCTGCCAGAGAAGCAGGCCACTGGCCGATTCGA
GGAGGACTTCATCGAGAAGCGCAAAAGGAGGCTGATCCTCTGGATGGACC
ACATGACCAGCCACCCTGTTCTCTCCCAGTATGAGGGCTTCGAACACTTC
CTCATGTGTGSCGATGACAAGCAGTGGAAGCTGGGCAAGCGGCGGGCAGA
GAAGGATGAGATGGTGGGCGCCAGCTTTCTGCTCACCTTCCAGATCCCCA
GTGAACACCAGGACCTGCAGGATGTGGAGGACCGGGTTGACACCTTCAAG
GCCTTCASTAAGAAGATGGACGACAGCGTGTTGCAGCTCACAAACGTGAC
CACCGAGCTGGTACGCAAGCAYGTGGGAGGCTTCCGCAAGGAGTTCCAGA
AGCTGGGCAGCGCATTCCAGTCAGTCAGCCAGGCCTTCACCCTAGACCCA
CCCTACAGCTTCGATGCCCTCAACAACGCCATCTCCCA-CCTCTTACGAC
TTTTCTCAAACTGACTTCCCTTGGCTTCATCATTGGGGTTGGCATGGTTG
GTAACCTCCTGATATCCATCTTATTGGTCAAAGACAAGACCTTGCACAGA
GCTCCCTACTACYTTTTACTTGATCTTTGTGGTTCAGATATACTCAGATC
CGCAATTTGTTTCCCATTTGTGTTCACTTCAGTTAAAAATGGTTCATCGT
GGACATATGGAACACTTACATGTAAAGTGATTGCCTTCCTGGGWGTTTTG
TCTTGCTTTCATACTGCCTTTATGCTGTTTTGCATCAGTGTGACTCGATA
YCTAGCCATAGCTCATCACCGTTTCTATAYGAAAAGGCTGACGTTTTGGA
CCTGCTTGGCTGTCATTTGTATGGTGTGGACTCTTTCCGTGGCTATGGCK
TTTCCACCAGTCTTGGACGTGGGTACCTACTCCTTTATCAGAGAGGAAGA
CCAGTGCACTTTCCAACATCGCTCCTTCAGAGCCAACGACTCACTGGGAT
TTATGCTACTTCTAGCCCTAATACTTCTCGCCACCCAGCTTGTCTACCTC
AAGCTTATTTTTTTTGTCCATGATCGCAGGAAGATGAAACCAGTCCAGTT
TGTACCAGCCGTCAGCCAAAACTGGACTTTTCATGGTCCAGGRGCCAGTG
GCCAGGCGGCTGCAAACTGGTTAGCTGGCTTTGGAAGAGGCCCTACTCCA
CCGACCCTGCTGGGAATAAGGCAAAACACCAATACAACAGGGAGAAGACG
GCTCCTCATTTTGGATGAATTTAAAACTGAAAAAAGGGTTAGCAGGATGT
TCTACATTATGACGTTTTTTTTTCTTACTTTGTGGGGCCCCTATCTTGTA
GCATGCTATTGGAGAGTGTTTGCCAGGGGGCCCGTGGTGCCAGGGGGGTA
TCTGACAGCTGCAGTCTGGATGAGTTTTGCCCAGGCTGGAGTCAATCCAT
TTATCTGCATTTTNNNNNNNNNNNNN-------------------------------------
--------------------------------------------------
--------------------------------------------------
--------------------------------------------------
--------------------------------------------------
--------------------------------------------------
--------------------------------------------------
--------------------------------------------------
--------------------------------------------------
--------------------------------------------------
--------------------------------------------------
--------------------------------------------------
--------------------------------------------------
--------------------------------------------------
------------------------------------------ACAGGCGA
TGTCACCGACAGAGAAGTGGGGTTGGGAATAAACCCGTTTGCAGATGGGA
```

TGGGGGCTTTTAAAATCAACCACAGTACTCATGATCTTGCMTCTGG---C
CAAACTGCATTTGCATCCCAAGCG---CCCGGGTAT---GCTGCGGCGGC
TCTGGGG---CATCATCA------CCATCCCACCCATGTCAGCTCG---T
ATTCCACTGCTGCTTTCAATTCAACCCGGGACTTTCTGTTTCGAAACCGG
GGGTTTGGAGAAGCGGCTAG------------CGCACAGCACAGCCTCTT
TGCCTCTGC---TGCAGGAACTTT---T------GCGGGGCCTCACGGGC
ACACAGATGCTGCAGGACACCTACTTTTCCCCGGACTTCACGAA---CAG
GCGGCCACTCATGCCTCTCCAAATGTTGTTAACAGCCAAATGCGCTTAGG
CTTTTCTGGGGACATGTATGGCAGGGCAGACCAGTATAGCCAGGTTACTA
GTCCCAGAT---CMGATCACTATGCATCGACCCAGCTGCACAGTTACGGT
GCCATGAACATGAACATGGCCGCC---CACCACGGTGCRGGGGCCTTYTT
TCGTTACATGAGGCAACCCATCAAACAAGAACTTATTGCAAATGGATTG
AACCSGARCAATTGGCAAACCCCAAAAAGGCTTGCAACAAAACTTTTAGC
ACAATGCACGAGCTCGTTACTCACCTCACAGTAGAGCACGTTGGGGGACC
CGAGCAAGCGAATCATATCTGTGTTTGGGAAGAGTGTCCCAGAGAAGGAA
AACCCTTCAAAGCCAAGTACAAACTTGTAAATCATATCCGAGTACACACC
GGCGAGAAACCCTTCCCNNNNNNNNNNNNNNNNNNNNNNNNN
>Amia calva
AGTCTCCTAATTCGAGCAGAACTAAGTCAACCAGGAGCCTTGCTAGGTGA
CGACCAGATCTATAATGTAATTGTTACAGCACATGCCTTTGTAATAATTT
TCTTTATAGTAATGCCCGTTATAATTGGTGGGTTTGGCAACTGGCTAGTC
CCCCTAATAATTGGAGCCCCGGACATAGCTTTCCCCCGAATAAACAACAT
GAGCTTCTGACTGCTGCCCCCATCCTTCCTACTTCTTCTTGCCTCCTCAG
GCGTAGAAGCGGGAGCCGGGACCGGCTGAACTGTATACCCCCCTCTGGCA
AGCAACCTTGCACACGCAGGCGCATCAGTAGATTTAACCATCTTCTCCCT
ACACTTAGCTGGAGTTTCATCCATTCTTGGGGCAATTAACTTTATCACAA
CCATTATCAACATAAAACCCCCAGCAGCCTCTCAATACCAAACACCTCTG
TTCGTATGATCCGTCCTAATTACTGCTGTTCTACTCCTCCTATCCCTGCC
CGTCTTGGCCGCAGGCATCACAATGCTACTAACTGACCGAAATCTCAATA
CCACATTCTTTGACCCTGCCGGAGGGGGCGACCCCATCTTATACCAACAC
CTTTTCTGATTCTTCGGACACCCAGAAGTATACATTTTAATTCTCCCAGG
GTTCGGAATAGTTTCCCATATTGTAGCATACTACGCAGGTAAAAAAGAAC
CATTCGGCTATATGGGAATAGTATGAGCCATAATGGCCATCGGCCTATTA
GGATTCATCGTATGAGCTCACCACATGTTTACAGTTGGAATAGACGTGGA
CACTCGAGCTTATTCCTAGAGAGAAACCTTCATCCATCCAACTGCCTTGG
CATGCTGCTGCTGTCTGATGCCCACCAGTGCACCAAGCTGTCAGAGCTCT
CATGGGGCATGTGCCTCAGCAACTTTCCCGCTATTTGCAAGACCGAGGAC
TTCCTCCAACTGCCCAAAGATATGGTGGTCCAGCTTTTGTCACATGAGGA
GCTGGAGACAGAGGATGAGAGACTGGTTTATGAAGCTGCCCTTAACTGGA
TCAACTATGACCTGGAAAAGAGACACTGTAACCTTCCAGAGCTCCTGAGA
ACAGTGCGTCTGGCCCTGCTGCCTGCCATCTTTCTCATGGAGAATGTATC
TACCGAAGAGCTGATCAACGCCCAGGCCAAGAGCAAGGAGCTGGTGGATG
AAGCTATCCGGTGTAAGCTGAAGATCCTGCAGAATGATGGCGTCGTTAAC
AGCCCGTGTGCTAGACCAAGAAAAACCAGCCATGCCCTCTTTCTTCTGGG
AGGGCAGACTTTCATGTGTGACAAGTTGTACCTGGTGGACCAGAAAGCCA
AAGAGATCATCCCCAAAGCTGACATCCCCAGCCCAAGGAAAGAGTTCAGC
GCCTGCGCCATCGGCTGTAAGGTGTACATCACTGGCGGCA--GAGGCTC-
AGAGAATGGTGTTTCCAAAGATGTATGGGTCTATGACACCGTCCATGAGG
AATGGTCAAAGGCGGCACCAATGCTCATTGCCAGGTTTGGCCATGGCTCT
GCTGAGCTGAAGCACTGCCTCTACGTGGTAGGAGGTCACACCGCAGCGAC
TGGCTGCCTCCCGGCCTCTCCATCTTGAGGAGTACATTGTGGTCTTTAGC
CGTAGCATCAACAGGCTCATCCTTAATGAAGCCGAACTAATTTTGGCTCT
GGCTCAGGAGTTTCAGATGAAGACGATCACGGTGTCTTTAGATGAGCACT

CCTTCGCAGATATCGTACAAATAATCAGTAAGGCGTCAATGCTTGTCAGT
ATGCACGGGGCTCAGCTGGTGACCTCACTCTTCCTGCCCCGCGGCGCTAT
AGTGGTAGAGCTTTTCCCATATGCTGTGAACCCTGAGCACTACACGCCCT
ACAAAACTATAGCCTCCTTGCCTGGCATGGATCTTCAGTATGTGGCCTGG
CAAAACACCAAAGAAGAGAACTCCATTGCGTTCCCAGACAGGCCCTGGGA
TCAAGGCGGAATAGCCCATCTAGAAAAGGAGGAGCAGGATCGCATCTTAA
AAAGCAAGGAGGTGCCTCGGCACTTGTGTTGCCGTAACCCGGAGTGGCTT
TTCCGCATTTACCAGGACACTAGTGTGGACATCCCCTCTCTTCTTGAAGT
ACTGAG---GCAGACGGTGAAG---TYTAAGCCCAATCTGAAAAA---GT
TTAAGCCGGCCAGCACAATTCACCCCGGTAAAGTGCGGGAACCCAAATGC
CAGGCTTCTGTACAAGGAACCAACGAGGCGAAGCTGACAGTGTCGTGGCA
GATCCCTTGGAATCTGAAGTATTTGAAAGTCAGGGAGGTCAAGTATGAAG
TGTGGATACAGAAGAAAGACACAGGCAAAGGGACACTGGAAGATCAAATC
ATCCAGGCCAACCCTGCTCTGGAAGCTTTTGGCAATGCCAAAACACTGCG
AAATGATAACTCCTCGCGTTTTGGCAAATTCATCCGTATTCATTTCGGAG
CCAGTGGGAAGCTGTCCTCTGCAGACATAGAGACTTACCTTCTTGAAAAA
TCCCGTGTCACCTTTCAGCTCAAAGCAGAAAGAAACTACCACATATTTTA
CCAGATATTATCTAATCAAAAGCCCGAGCTGCTGGACATGCTTTTAATCA
CAAACAATCCGTACGACTACTCCTATGTGTCCCAAGGAGAAGTCACAGTT
GCATCTATAGATGATGCTGAAGAACTGATCGCCACGGACAGTGCCTTTGA
TGTGCTTGGCTTCACCACAGAAGAAAAAATGGGGGTCTATAAACTAACAG
GTGCCATCATGCATTATGGGAACATGAGGTTCAAGCAAAAGCAGCGTGAA
GAGCAGGCAGAGCCCGATGGCAGTGAGTCTGCTGACAAGTCAGCATACCT
GATGGGACTCAACTCAGCTGACCTCTTGAAAGGACTTTGCCACCCCAGGG
TGAAAGTCGGCAATGAGTATGTAACCAAGGGGCAAAGTGTTGAACAGGTT
TACTATCCGAACAAGGAGGCTTTCAAGTGCGAGGAGTGTGGCAAGAACTA
CAACACCAAGCTGGGCTACAAGCGGCACGTGGCCATGCACGCTGCCACCA
GTGGCGACCTCACCTGCAAAGTGTGCCTGCAGACGTACGAGAGCACGCCT
GTGCTGCTGGAGCACCTCAAGAGCCACTCGGGCAAGTCCTCCGGCGGCAC
CAAGGAGAAGAAGCACCCCTGCGACCACTGTGACCGCCGCTTCTACACCC
GCAAGGATGTGCGGCGCCACATGGTTGTACACACGGGGCGCAAGGACTTC
CTGTGCCAGTACTGTGCCCAGCGCTTTGGCCGCAAGGACCACCTGACCCG
GCACGTCAAGAAGAGCCACTCTCAGGAGCTCCTGAAGATCAAATCGGAGC
CACCCGACATGCTAGGCCTCCTCAACTCAGGCTCACCCATCT---CTGTG
AAGGAGGAGCTCAGCCCCATGATGTG---CATGGCTGCCTCCAAGGACAA
CATGATGGGCAAGCCCTTCAGTAACA------GCTTCCCCATGGGCATGT
ACAACCCACAC---------CTCCAGGTCATGTCCAACTCAGGAGTGTCA
CAC---------TCGCACACCTTGGTGCCGAACTCACTG---GCCGCYAT
GGGAATGGGCTGCCCCAT----TACCTAATTTATGCATCGTTTTCTTTTA
TGGGATGTTTACAAATTAGTGATGGGTCAAACATCGTCAACCTCCTAGCC
AGTGATTCACCGAGCGTATCCTATGCTCTCACACAGCAAAAATACTTTAG
CAACTACAGTCCTGTCATTGGATTCTACATTTATGAGCCGATAGATTATT
GGAATACAACTGTTCAGGACCACCTGAAGACCTTGGGTCATGGGTTCAAT
AAAATATCATGGATTGATCATTACTTTCAGTATCTAAAGGTAGTAAACAT
CAGTGCATCGACCAAAAGTGATTTCATAACTGTCCTCCAGAGTTCCTTCT
TAAGGAAGCCAGAATTTCAACACTTTAAGGAAGACATTATTTTTTCCAAA
A---CCGGGG------------ATGACATTGATATAATTGCGTCCAGAAT
GTACATGGTGGCAAGAACAACGGAGAAAACAAGAGAGGAAGTCGTGGAAT
TGTTGGATAGGCTTAGGCCCCTTTCTCTCATCAACAGCATCAAATTTATA
GTGTTTAACCCCACCTTTGTGTTCATGGATCGTTACAGCTCCTCTGTCAT
TTCACCGATCCTGACCTCTGGATTCAGTGTCTTGATTGTTTTAATTCTGA
CATTCTTCCTAGTCATTCATCCTCTTGGCAACTTTTGGTTGATACTAACA
GTTACATCTGTAGAGCTAGGNNNNNNNNNNNNNNNN------------------------------

```
-------------------ACACAGAGGTGGGGATTATAGATGGGCTGTC
TGGCTGGGTGGCTTCTGTGGATGACCAACAGATCAACACCATCTCCCGCA
GGTTTCGCTATGATGTGGCTCTTGCCGCTGCTTTGAAGGACTTTGAAGAG
GACATTGTGGAAGGCATGAGGGAGCAGCAGCTGGATGACAGC---TTGAC
TGAKAGTTTCACTGTGATCATCAAGGAGGCTTGCGATGGCATGGGAGACG
TCAGCGAGAAACATGGGAATGGCCCAGCTCTTCCTGAGAAAGCTGTCCGC
TTCTCCTTCACCATCATGTCTATTTCCGCCCACAGCGAAGATGGG-----
----------------------------AAAAATGTCAAGATCTTCCAGG
AGCTCAAACCTAACTCAGAGCTCTGCTGCAAGCCCCTGTGCCTTATGTTC
GCGGACGAATCTGACCATGAAACGCTGACTGCTATATTGGGGCCTGTAGT
GGCTGAGCGGGAAGCCATGAAAGAAAGCCGCTTGATCCTTTCAATTGGGG
GCATGCTGAGGTCCTTCCGGTTTGTGTTTAGGGGCACAGGCTATGATGAG
AAATTGGTCAGAGATGTGGAAGGACTTGAGGCTTCAGGGTCTACTTACAT
CTGCACCCTTTGTGATTCCACCAGAGCAGAAGCTTCTCAGAATATGGTGC
TGCACACAATCACCAGGAGCCACGAGGAGAACCTAGAACGCTATGAGATC
TGGAGGACCAACCCATACTCAGAGTCGGCCGATGAGCTGCGTGATCGGGT
CAAAGGGGTTTCAGCAAAACCGTTCTTGGAAACGTTGCCCTCCATCGATG
CGCTGCACTGTGACATCGGGAATGCCACCGAGTTTTACAAGATCTTCCAG
GATGAAATTGGAGAGCTCTACATCAAAAG---CAAC---CCCACCCGGGA
GGAACGGAAGCGGTGGCAGTCTGCCCTAGACAAGCAGCTGAGGAAGAAGA
TGAACCTTAAGCCCATCATGAGAATGAATGGCAATTTTGCCCGCAAACTG
ATGACCAAGGAGACGGTGGATGCTGTGTGTGAGCTGGTTCCTTCTGAGGA
GCGCCGAGAAGCTCTCAAGGAGCTGATGAATCTCTACCTCCAAATGAAAC
CTGTTTGGCGCTCCACCTGCCCAGCCAAAGAGTGTCCTGACTTGCTGTGC
CGCTATAGCTTCAACTCGCAACGTTTTGCCGAGCTCCTATCTTCCACCTT
CAAATACCGCTACGATGGCAAGATCACCAATTACCTGCACAAGACCCTCG
CTCACGT-------------------------------------------
---------------------------ACTTACCAAGTCGAGATGGGTTC
CAGAGGCCCACAGTGGAAAGGCAACCCCAGGCCTTTTTCTTGTTCCATTG
AGGATCCCACTAAACAGACCAAATTCAAGGGCATCAAGACCTACATTTCC
TATCGGGTGACACCCAGCCACACAGGCAGGCCTGTCTACCGCCGCTACAA
GCACTTTGACTGGCTCTACAACCGGCTACTGCACAAGTTCACAGTTATCT
CCGTGCCCCACCTGCCGGAGAAGCAGGCCACTGGGCGCTTCGAAGAGGAC
TTCATTGAGAAGCGGAAGCGGAGGCTAATCCTGTGGATGGACCACATGAC
CAGCCACCCAGTGCTCTCCCAGTATGAAGGCTTCGAGCACTTCCTCATGT
GTGCAGATGACAAACAATGGAAACTGGGTAAGCGGCGGGCCGAGAAGGAC
GAGATGGTGGGTGCCAGCTTCCTCCTCACATTTCAGATTCCCAATGAGCA
CCAGGACTTGCAGGATGTGGAGGAGCGGGTAGACTCCTTCAAGGCCTTCA
CCAAGAAGATGGATGATAGTGTAATGCAGCTCACCCATGTGGCCTCCGAG
CTTGTGCGCAAACACTTGGGTGGATTCCGCAAGGAGTTCCAGAAGCTGGG
CAATGCCTTCCAGTCTGTCAGCCAGGCTTTTGCCTTAGATCCCCCATACA
CCTCAGAGGCCCTCAACAAGCAAATCTCCCANNNNNNNNNNNNNNNNNNNNNNNTGACGTCCCTCGGTTT
TAT
CATTGGAGTCGGTGTGGTTGGCAATCTCCTGATCTCTATCCTGCTCGTCA
AAGACAAAACCTTGCACAGGGCACCCTACTACTTCTTACTGGATCTGTGT
GGCTCAGACATCCTGAGGTCTGCGATTTGTTTCCCCTTTGTGTTCACTTC
GGTCAAAAATGGGTCAACGTGGACATATGGTACACTTACATGCAAAGTAA
TTGCCTTCCTAGGAGTTTTATCTTGCTTTCACACTGCCTTTATGTTATTT
TGCATCAGTGTGACACGGTATCTCGCCATAGCCCACCACCGCTTCTATAC
AAAAAGGCTGACGTTCTGGACTTGTCTGGCTGTCATCTGCATGGTGTGGA
CGTTGTCAGTGGCTATGGCTTTCCCCCCAGTCTTGGACGTGGGGACCTAT
TCTTTCATCAGGGAAGAAGATCAATGCACCTTCCAGCACCGATCCTTCAG
AGCCAACGATTCCCTGGGATTTATGCTCCTCCTAGCCCTCATCCTTCTGG
```

CCACTCAGCTTGTCTACCTCAAGCTAATCTTTTTCGTCCATGACCGCAGA
AAAATGAAACCAGTCCAGTTTGTGCCGGCTGTGAGCCAGAACTGGACATT
TCACGGTCCAGGAGCCAGTGGTCAGGCGGCTGCCAACTGGCTAGCTGGCT
TCGGAAGAGGCCCAACCCCACCAACCCTGTTAGGAATAAGGCAGAATACC
AATACGGCAGGCAGGAGAAGGCTTCTGGTGTTGGACGAGTTTAAGACTGA
AAAGAGGATAAGCAGGATGTTCTACATAATGACGTTCTTCTTTCTCACCT
TGTGGGGCCCCTATCTGATAGCTTGCTATTGGAGAGTGTTTGCCAGGGGG
CCAGTTGTGCCAGGAGGGTACCTGACAGCTGCAGTCTGGATGAGTTTTGC
CCAGGCAGGAGTCAATCCATTTNNNNNNNNNNNNNNNNNNNNNNNN------------------------
----

-ACAGGCGATGTCACCGACAGAGAAGTGGGGTTGGGGATAAATCCGTTTG
CTGATGGGATGGGCGCTTTTAAAATCAACCACAGTACCCACGATCTAGCT
TCTGG---CCAAACGGCTTTTTCGTCCCAAGCG---CCTGGTTAT---GC
GGCCGCAGCTCTGGGG---CATCATCA------CCACCCCTCCCATGTAA
GCTCC---TATTCYACCGCGGCTTTCAATTCTACACGGGACTTTCTCTTT
CGAAATCGGGGTTTCGGAGAAGCGGCCAG------------CGCTCAGCA
CAGCCTCTTTGCGTCTGC---AGCAGGAAGTTT---T------GCGGGGC
CACATGGACACACAGATGCCACAGGACACTTACTTTTCCCCGGACTTCAC
GAG---CAGGCGACCACTCACGGGTCTCCCAATGTTGTAAATAGCCAGAT
GCGCTTGGGCTTTTCTGGGGACATGTACGGCAGAGCCGAGCAATACAGCC
AGGTAACAAGCCCCAGAT---CAGATCACTATGCGTCGACCCAGTTACAC
AGTTATGGCCCTATGAACATGAACATGGCTGCA---CACCATGGGGCAGG
GGCCTTTTTTCGGTATATGAGGCAACCTATAAAACAAGAACTTATCTGCA
AATGGATTGAACCGGAGCAGTTGAGTAACCCGAAAAAGTCTTGTAATAAA
ACTTTTAGCACAATGCATGAGCTCGTCACTCACCTCACAGTGGAGCATGT
TGGGGGACCAGAACAGTCGAATCATATCTGTTTCTGGGAAGAGTGTTCCA
GGGAAGGGAAACCGTTTAAAGCCAAGTACAAACTGGTAAATCACATCCGA
GTGCACACAGGCGAGAAACCGTTTCCATGTCCGTTCCCTGGCTGTGGCAA
A
>Abalistes stellatus
AGCTTGCTAATCCGAGCAGAATTAAGCCAACCCGGCGCTCTCTTAGGCGA
CGATCAAATTTATAATGTCATCGTCACAGCACATGCTTTCGTAATAATTT
TCTTTATAGTAATACCAATTATGATTGGAGGCTTTGGAAATTGATTAATT
CCATTAATGATTGGAGCCCCTGACATAGCATTTCCCCGAATAAACAACAT
GAGCTTTTGACTTTTACCCCCCTCCCTCCTTCTCCTACTCGCTTCTTCAA
GCGTAGAGGCCGGGGCCGGAACTGGGTGAACAGTGTACCCCCCTCTTGCC
GGCAATCTAGCCCATGCAGGGGCTTCCGTAGACCTGACTATTTTTTCTCT
TCACCTAGCAGGAATTTCGTCAATCCTTGGAGCAATTAATTTTATTACAA
CAATTATTAATATGAAACCCCCTGCCATCTCCCAATATCAAACACCACTA

TTCGTCTGAGCAGTCCTAATCACAGCCGTTCTCCTTCTCCTCTCCCTTCC
TGTCCTAGCCGCCGGAATCACGATACTACTTACCGACCGAAATTTAAATA
CCACATTTTTTGACCCCGCCGGAGGTGGGGACCCAATCCTCTATCAGCA-

------------NNNNNNNNGAGAAACCTTCACCCATCTAACTGCCTTGGCATGCTGC
TGCTGTCTGACGCCCACCAGTGCACCAAGCTGTCCGAGCTGTCCTGGGGC
ATGTGCCTCAGCAACTTCCCGGCCATTTGCAAGACGGAGGACTTCCTGCA
ACTGCCCAAAGATATGGTGGTGCAGCTTTTGTCGCACGAGGAGCTAGAGA
CCGAAGATGAGAGACTGGTTTATGAAGCGGCCCTGAACTGGATCAACTAC
GACCTGGAAAAGAGGCACTGCCACCTTCCCGAGCTCCTGAGAACGGTCCG
YCTGGCACTTCTGCCCGCCATCTTTCTCATGGAGAATGTCTCTACAGAAG
AGCTGATCAACGCCCAGGCCAAGAGCAAGGAGCTGGTGGACGAAGCTATC
CGATGCAAGCTGAAGATCCTGCAGAATGACGGCGTTGTTAACAGTCCGTG
TGCTCGGCCGAGAAAAACCAGCCACGCTCTCTTTCTTCTGGGTGGGCAAA
CGTTCATGTGTGACAAGTTGTATCTGGTGGACCAGAAGGCCAAAGAGATC
ATCCCCAAGGCGGACATTCCTAGCCCTAGGAAGGAGTTTAGTGCCTGTGC
CATTGGCTGCAAGGTGTACATCACTGGAGGAA--GGGGCTC-TGAGAACG
GTGTGTCCAAAGATGTGTGGGTCTACGACACAGTCCACGAGGAATGGTCT
AAAGCAGCGCCCATGCTTATCGCCAGGTTTGGCCACGGCTCTGCAGAGCT
GAAACACTGCCTCTACGTGGTAGGANNNNNNNNNNNNNNNNNNNNNNNNNNNNNNNNNNNNNNGGA
TGAATATATTGTTGTGTTCAGT
CGCTCAACAACGAGGCTGATACTGAATGAAGCCGAGCTAATTATGGCGCT
GGCGCAGGAGTTTCAGATGAGAGTGGTTACGGTGTCACTGGAAGAGCAGT
CTTTCCCCAGTATCATCCAGGTGATCAGCGGTGCTACCATGTTAGTCAGC
ATGCATGGAGCTCAGCTCATCACCTCACTCTTCCTGCCCAGAGGAGCTGC
TGTGGTTGAGCTGTTTCCCTTTGCAGTGAACCCGGAGCAATACACCCCAT
ATAAAACCCTTACATCCCTTCCAGGTATGGACCTTCACTATATTTCCTGG
AGAAACACGAAGGAGGAGAACACCATCACCCATCCGGACAGACCCTGGGA
ACAAGGGGGCATCGCTCACTTAGAGAAGGAGGAGCAAGAGAGGATACTGG
CGAGCAAAGATGTCCCCAGGCACCTGTGCTGCCGCAATCCAGAGTGGCTT
TTCCGGATCTACCAGGACACCCTGGTGGACATTCCGTCCTTCTTGGAAGT
CCTCAA---GGAGGGCTTAAAA---ACAAAGCCCACTTTAAAAAA---GT
CAAAGCCAGCCAGCTCGCTGCACCCAGGCCGGGTCAGAGAACCTCAGTGT
CAGACCTCAGTACAGACCAGTAATGAGGCTAAACTCACAGTCTCCTGGCA
GATCCCATGGAATCTGAAATACCTGAAGGTGAGAGAGGTGAAATNNNNNNNNNNNNNNNNNNAAAAAG
GACACCAGCAAGGGGACCCTTGAGGATCAAATCATTCAGGCCAACCCCGC
CCTGGAGGCCTTCGGAAACGCCAAAACGGTGAGAAACGACAACTCATCTC
GGTTTGGAAAATTCATCCGGATTCACTTTGGTCCGAGCGGCAAGCTGTCA
TCAGCTGACATTGAGACATACCTGCTGGAGAAGTCACGTGTCACCTTTCA
GCTCAAGTCTGAGAGGAATTACCACATCTTCTACCAGATTCTGTCCAATC
AGAAGCCAGAACTGCTGGACATGCTGCTGATCACCAACAACCCCTACGAC
TACTCCTACATCTCCCAGGGAGAGGTAACAGTGGCCTCCATCAACGATTC
CGAGGAGTTGATGGCCACCGACAGCGCCTTCGATGTGCTGGGCTTCACGG
TCGAGGAGAAGATGGGCGTCTACAAACTGACTGGCGCCATCATGCACTAC
GGCAACATGAAGTTCAAACAAAAGCAGCGCGAGGAGCAGGCCGAACCCGA
TGGGACAGAAGCTGCCGATAAATCGGCTTACCTCATGGGCCTGAACTCTG
CTGACCTCATCAAAGGGCTGTGCCACCCCCGAGTCAAGGTGGGCAATGAA
TATGTCACCAAAGGCCAGAGCGTAGACCAAGTCTACTACCCTAACAAAGA
GGCCTTCAAGTGTGAGGAGTGTGGGAAGCACTACAACACCAAGTTGGGAT
ATAAGCGCCACGTGGCCATGCACTCTGCCACAGCAGGTGATCTCACCTGT

AAAGTTTGCATGCAGACTTATGAGAGCACACCTGTGCTCTTGGAGCATCT
CAAGAGCCACTCCGGAAAGTCTTCCGGGGGCACCAAGGAGAAGAAACACC
CATGTGACCACTGTGATCGCCGTTTCTACACACGAAAGGATGTGAGACGA
CACATGGTCGTCCACACCGGTCGAAAGGACTTCCTGTGCCAGTACTGTGC
CCAGCGTTTTGGCAGGAAGGACCATCTGACGCGACATGTCAAGAAGAGTC
ACTCACAGGAGCTGCTGAAAATCAAGACCGAGCCTCCTGATATGTTAGGT
CTTTTAGCCTCTGGGTCACCACCGTGCTCCGTGAAAGAGGAGCTCAGCCC
CATGATGTGCGGCATGGGTTCCAACAAAGATCCCATGATGGGCAAA---T
TCCCTAGCGGAGCCCCTTTTCCGATGAGCATGTACAACCCCCACCAT---
---CTCCAGGCCATGTCTAATTCTGGAGTTGGTCACCCA------CACCC
ATCCTTAATGCCCACTTCCTTGTCTGCAGCTGTGAGCATGGGCTGTCACA
TGGAATATCTGATCTACGCATCTTTCTCGTTCATGGGATGTTTACAAATC
AGCGATGGTTCTAATATTGTGAACCTGTTGTCCAGTAACTCTCCGAGTGT
TTCATATGCTTTGACCCAGCAAAAGTACTTCAGTAACTATAGTCCTGTTA
TTGGCTTTTACATTTATGAGCCTATTGAGTACTGGAATTCAACAGTGCAG
GAACACCTTAAGACTCTGAGTCATGGCTTCAATAAGATCTCCTGGATGGA
CAACTTTTTCCACTACCTGAGGGTAGTGAACGTGAGTGCATCAACCAAGA
GTGACTTCATCACTATCCTCAAGGGCTCCTTCTTACGCAGCCCAGAGTAC
CAACACTTCACTGAGGACATTATATTCTCTAAGA---ACCGGGACACTG-
-----ATGAATATGATATCATTGCCTCACGGATGTACTTGGTGGCTCGGA
CAACTGAGAAGAAGCGGGAAGAGGTGGTTGAGCTCCTCGAGAAGCTTCGT
CCGTTGATGCTGATCAACAGCATCAAGTTCATTGCCTTCAACCCTACATT
CGTCTTTATGGACCGCTACAGCTCCTCTGTCATCTCGCCCATCCTGACCT
CAGGATTCAGTGTACTAACAATCCTTATTCTCACTTTCTTCCTGGTCATC
AATCCCTTGGGAAACTTTTGGCTCATTCTGACGGTAACATCTGTGGAGCT
AGGCGTCCTGNNNNNNNNNNNNNNNNNNNNNNNNNNNNNNNNNNNNNNNNNNNNATGTGTCCACATCT
TGCAACGTTGGAATCATTAATGGGCT
CGCTGGATGGTCTTCTACTGTGGATGACTCCCCCGCTGACACCATCACTC
GACGGTTTCGATACGACGTGGCTCTGGTGTCAGCTTTAAAGGACTTGGAG
GAGGACATCATGGACGGGCTGAGAGAGAATAGAATGGATGACAGCACTTG
CACCTCAGGCTTCAGTGTCTTGATCAAAGAATCTTGTGATGGCATGGGGG
ATGTCAGCGAGAAGCACGGTGGAGGACCACTTGTTCCTGAAAAGGTTGTG
CGTTTCTCTTTCACAGTGATGTCTGTCTCTATTCTGGCAGACGGGGCAGA
G-------------------------------GGTAAAGTCACCATCTACA
CAGAGCCAAAGCCAAACTCAGAGCTGTCCTGTAAGCCCCTTAGCCTGATG
TCTGTTGATGAGTCCGACCATGAGACACTCACCGCCATTCTGAGGCCTAT
AATCGCTGAGCGTAACGCAATGAAAGAGAGCAGACTTATACTATCCATGG
GTGGACTCCCACGCTCCTTCCGCTTTCATTTAGAAGCACAGGATACGAT
GAGAAGATGGTGCGCGAGATAGAGGGTCTTGAGGCCTCTGGGTCCACCTA
CATTTGCACTCTTTGCGACTCCAGTCGTGCAGAGGCCTCTCAAAACATGG
TGCTGCATTCCATCACCCGCAGTCATGAAGAGAACCTGGAACGTTATGAA
ATATGGAGAACCAACCCTTTTTCTGAGTCTGTAGAGGAGCTGCGAGACAG
AGTCAAAGGAGTCTCCGCAAAGCCCTTCATGGAAACGCATCCCACGCTCG
ATGCGCTACACTGTGACATCGGCAATGCCACTGAGTTTTACAAAATCTTC
CAGGATGAAATCGGAGAGATGTACAAAAAGGT---CAGC---CCCAGCCG
GGAGGAGCGGCGCAGCTGGAGGGCAGCCCTAGATAAACAGCTGAGGAAGA
AGATGAAGCTCAAACCAATAATGAGGATGAATGGGAACTATGCCCGTCGG
TTAATGACCCTGGAGGCTGTGGAAGTGGTGTGTGAGTTGGTGCCCTCAGA
AGAGAGGAGGGAGGCCCTCAGGGAGCTCATGAGGCTCTATCTTCAGATGA
AGCCTGTGTGGCGAGCCACCTGCCCGGCCAAGGAGTGCCCTGACCAGCTG
TGCCGCTACAGCTTTAACTCCCAGCGCTTTGCCGACCTCCTCTCCTCTAC
CTTCAAATATAGGTACAATGGGAAGATAACCAATTACCTGCACAAGACCC
TGGCTCACGTGCCAGAANNNNNNNNNNNNNNNNNNNNNNNNNNNNNNNNNNNNNNNNNNNNNNNNNNN

NNNNNNNNNNTCTTACACAATTGAGATGGGTCCTCTGGGGCCC
CAATGGAAGGAGAGCCCACAGCCCTTCTCCTGCTCCATCGAAGACCCAAC
AAAGCAGACAAAGTTCAAGGGCATTAAGACGTACATATCGTACCGGGTGA
CGCCGAGCCACACTGGGCACCCTGTCTACCGGCGCTACAAACACTTTGAC
TGGCTGTACAACCGCCTACTGCACAAGTTTACTGTGATCTCTGTGCCTCA
CCTGCCCGAGAAACAGGCCACGGGGCGATTTGAGGAAGACTTCATCGAGA
AGCGCAAGAGGCGACTGATCCTGTGGATGAACCACATGACCAGTCACCCC
GTCCTCTCCCAGTACGAAGGCTTTGAACACTTTCTGATGTGCGCCGATGA
CAAGCAGTGGAAACTGGGCAAGAGGCGGGCGGAGAAGGACGAGATGGTGG
GTGCGCATTTCATGCTGACCCTCCAGATCCCAAACGAGCACCAGGACCTG
CAGGACGTTGAGGAGCGTGTTGACTCCTTCAAGGCCTTCGCCAAAAAGAT
GGATGACAGCGTGATGCAGCTCACGCACGTTGCCTCGGAGCTGGTGCGCA
AACACCTGGGTGGATTCAGGAAGGAGTTCCAGCGGCTGGGGAATGCCTTC
CAGTCAATCAGCCAGGCGTTCATGCTGGACCCTCCCCACAGCTCAGAGAC
CTTGAACAACGCCATCTCCCATNNNNNNNNNNNNNTTCCTCAAACTGACCTCTCTTGGCTTCA
TCATCGGTGTCGGCGTGGTCGGAAACCTCCTGATCTCCATCCTGCTGGTC
AAAGATAAGAGCCTGCACCGAGCGCCCTACTATTTCCTGCTGGACCTGTG
CGCCTCGGACATTCTTCGATCCGCCATCTGCTTCCCGTTTGTCTTCACCT
CCGTCAAGAATGGATCCGCGTGGACGTACGGCACGCTGACCTGCAAGGTG
ATCGCCTTCCTGGGCGTGCTCTCCTGTTTCCACACGGCCTTTATGCTGTT
CTGCGTTAGTGTCACGCGCTATTTGGCCATAGCGCACCACCGTTTCTACA
CCAAGAGGCTGACCTTCTGGACCTGCCTGGCCGTCATCTGCATGGTTTGG
ACGTTGTCGGTGGCGATGGCGTTCCCGCCGGTGCTCGACGTGGGGACGTA
CTCTTTTATCCGGGAGGAGGACCAGTGCACATTCCAGCACCGCTCCTTCA
GGGCGAATGACTCCTTGGGCTTCATGCTCCTCCTGGCTCTCATCCTCCTC
GCCACACAGCTGGTTTACCTCAAGCTCATCTTTTTCGTTCACGACCGCAG
GAAGATGAAGCCTGTCCAGTTTGTGCCTGCTGTTAGCCAAAACTGGACAT
TCCACGGGCCGGGCGCCAGCGGGCAGGCCGCCGCCAACTGGCTGGCTGGA
TTCGGGCGAGGCCCCACCCCACCGACTCTGCTGGGTATCCGGCAGAACAG
CAACGCGGCGGGCCGCAGGCGTCTTCTGGTACTGGATGAGTTCAAAACAG
AGAAGAGGATTAGTAGGATGTTCTACATCATGACGTTTTTCTTCCTGGCA
CTGTGGGGGCCCTATTTGGTCGCCTGCTACTGGCGGGTATTTGCGAGGGG
CCCCGTGGTCCCCGGAGGCTACCTGACAGCAGCCGTGTGGATGAGCTTTG
CCCNNNNNNNNNNNNNNNNNNNNNNNNNNNNNNNNNNNNNNNNNNNNNNNGCCAAATCTCGCTTTCACCCTGG
CATGGGGACTGGTCCTGGCACGGA
GC---GCAGCGTCCCACTCGGCAACAGCTTGCTGTCCCCGCAGCAAACCG
AGGAGCCCACTGTTGCCACCCCCCCGCAGCGATGGTTTGTCACCC---CT
GCCAACAACCGACTGGACTTTGCTGCCTCGGCATACGACGCGGCC-----
----GATTTCGCCGGTAACGCGGCCACCTTGCTGTCCTACGCAGCGGCCG
GAGTGAAGGCTC------TCCCCCTGCCGACTGCGGGCTGCTCCAACCGG
CCTCTTGGTTATTACGCAGACCCGTCGG---GCTGG---GGAGGACGCAC
GCCGCCGCAGTACTGTGGCGTCAACAGCAAGTCCAGCTCGGTCTTCTCCT
GCTGGCCTTCCAACTCCATCGCGGGCAGAGCGGGCG---CC---AACTAC
CTGG---------CCGAGGA---GGGC---GACCC---CGTGCCCACGGA
GAGGTCGCCG---AT---CGGCGGCGCCGAGGAG---ACCAAACCGAAGG
ACATAAG---CTCGGA---GTCGAACTGGATAGAG---ACGCCGTCCTCC
ATCAAGTCCATAGATTCGAGCGACTCTGGGATCTTTG---AACAGGCCAA
ACGGAGGCGGATCTCACCTTCTGCCACGCCG---------GTGTCAGAGG
CAGTGTCCCCGCTGAAGTCTGAGCATCACTCAACAGGCGAAGTCACGGAG
AGAGAAGTGGCGCTGGGGATAAATCCGTTCGCGGACGGGATGGGCGCCTT
CAAGATAAACCACAGCTCCCACGACATTGGCTCCGG---GCAGACGGCGT
TTTCCTCTCAGGCG---CCCGGCTACGCGGCGGCGGCAGCCCTGGGA---
CACCATCA------CCACCCGACCCACGTTGGCTCT---TACTCCACGGC

AGCTTTCAACTCCACCAGGGACTTTCTCTTCAGAAATCGGGGATTCGGGG
ACGCCGCCGG------------CGCGCAGCACAGTTTGTTCGCCTC----
-----CGGAAGTTT---C------GCAGGGCCACATGGACACTCAGATGC
AGCGGGGCACCTGCTCTTCCCGGGGCTCCACGAG---CAAGCGGCGAGCC
ACGCGTCTTCCAATGTGGTTAACAGTCAGATGCGGCTGGGCTTCTCGGGG
GACATGTACGGACGGGCCGACCAGTACGGCCATGTTACGAGCCCGCGGT-
--CCGACCACTACGCGTCGACCCAGCTGCACGGCTACGGCCCCATGAACA
TGAATATGGCCGCA---CACCACGGAGCGGGGGCCTTCTTTCGGTACATG
CGGCAGCCGATCAAGCAAGAGCTCATCTGCAAGTGGATCGAGCCGGAGCA
GCTGACAAATCCCAAAAAGTCGTGCAACAAAACTTTTAGCACGATGCACG
AGCTGGTGACCCATCTGACGGTGGAGCATGTGGGGGGACCGGAGCAGACC
AACCACGTCTGCTTCTGGGAGGACTGCTCCAGGGAAGGAAAGCCATTCAA
AGCCAAATACAAACTTGTGAATCATATCAGAGTACACACTGGAGAGAAGC
CCTTCCCGTGTCCGTTCCCCGGCTGTGGCAAA
>Acanthaphritis unoorum

------------NNNNNNNNNNGAAACCTTCACCCATCCAACTGCCTGGGGATGTTGTTG
CTGTCAGATGCTCACCAGTGCACCAAGCTGTCAGAGCTCTCCTGGGGCAT
GTGCCTCAGCAACTTTCCCGCTATTTGCAAGACAGAGGATTTCCTCCAAC
TGCCCAAAGATATGGTAGTGCAGCTTTTATCACACGAAGAGCTTGAGACA
GAAGATGAAAGGCTAGTTTACGAAGCTGCACTTAACTGGATCAACTATGA
CCTGGAAAAGAGGCACTGCCACCTACCAGAGCTTCTGAGAACGGTCCGTC
TGGCCCTGCTTCCTGCTATCTTTCTGATGGAGAATGTTTCAACAGAAGAG
CTGATTAATGCCCAGGCAAAGAGCAAGGAGCTGGTGGATGAAGCTATTCG
TTGCAAGCTGAAGATTCTGCAGAATGATGGAGTCGTGAACAGCCCATGTG
CCCGACCAAGAAAAACCAGTCATGCACTCTTTCTCCTTGGAGGGCAAACC
TTTATGTGTGATAAGCTGTACCTTGTGGACCAGAAAGCCAAAGAGATCAT
CCCCAAAGCCGACATCCCCAGCCCCAGAAAGGAGTTCAGTGCTTGCGCTA
TTGGCTGCAAGGTCTACATCACCGGAGGGA--GAGGCTC-GGAGAATGGC
GTGTCCAAAGATGTGTGGGTCTATGACACCGTCCATGAGGAATGGTCCAA
GGCTGCACCCATGCTCATCGCCAGGTTTGGTCATGGTTCCGCAGAGCTGA
AACACTGCCTCTACGTGGTNNNNNNNNNNNNNNNNNNNNNNNNNNNNNNNNNNNNNNNNNNNNNNNN-----
----

-----AAGAAAGACTCCAGTAAGGGGACCCTGGAGGATCAGATCATCCAG
GCCAACCCAGCGCTTGAGGCCTTTGGGAACGCCAAGACGCTCAGGAACGA
CAACTCGTCTCGTTTTGGAAAGTTCATACGGATCCACTTTGGAACGAGCG
GGAAGCTGTCGTCCGCCGACATCGAGACGTACCTGCTGGAGAAGTCCCGC
GTCACCTTTCAGCTCAAAGCTGAGAGGAACTACCACATCTTCTACCAGAT
CTTGTCCAATCAGAAGCCGGAGCTGCTGGACATGCTTTTGATCACCAACA
ACCCGTACGACTACTCCTACATCTCGCAGGGAGAGGTAACAGTGGCCTCC
ATCAACGACGCAGACGAGCTGATGGCCACCGACAGTGCCTTCGACGTGCT
TGGCTTCACACCCGACGAGAAGATGGGCGTGTACAAGCTGACGGGCGCCA
TCATGCACTACGGAAATATGAAGTTTAAGCAGAAGCAGCGTGAGGAGCAG
GCGGAGTCAGACGGGACTGAAGCGGCGGATAAATCGGCGTACCTCATGGG
GCTGAACTCCGCCGACCTCATCAAGGGGCTGTGCCATCCCAGGGTGAAGG
TGGGGAACGAATACGTGACCAAAGGACAGAGTGTGGACCAAGTCTACTAC
NNNNNNNNNNNNNNNNNNNNNNNNNNNNNNNNGGGAAGCACTACAACACCAAGCTGGGATACAAGCGCC
ATGTGGCCATGCA
CTCGGCCACAGCGGGGGATCTCACCTGTAAAGTGTGCATGCAGACCTACG
AGAGCACGCCGGTTCTCCTAGAGCACCTCAAGAGTCACTCCGGGAAGTCT
TCAGGCGGCACCAAGGAGAAAAAACACCCCTGCGATCACTGCGACCGTCG
ATTCTACACGCGGAAGGATGTGAGACGACACATGGTGGTTCACACGGGCA
GAAAGGACTTCCTGTGTCAGTACTGCGCCCAGCGCTTCGGCAGGAAGGAC
CACCTGACGCGTCACGTGAAGAAGAGCCACTCGCAGGAGCTGCTGAAGAT
CAAGACGGAGCCTCCTGACATGTTGGGTCTGTTGGCGTCGGGGTCGCCCC
CCTGCTCTGTGAAGGAGGAGCTCAGCCCCATGATGTGCGGTATGGGTCCT
AACAAAGACCCCATGATGGGCAAACCCTTCCCCAGCGGGGCTCCTTTCCC
GATGGGCATGTACAACCCCCACCAC------CTCCAGGCCATGTCTAATT
CAGGGGTGGGTCACCCT------CACCCGTCCCTGGTGCCCACCTCCTTG
TCTGCAGCTATGGGCATGGGCTGCCANNNNNNNNNNNNNNNNNNNNNNNNNNNNNNNNNNNNNNNNNNN
NNNNNNNNNNNNNNNNNNNNNNNNTGTGAACCTGCTGGCTAGTAACTC
TCCAAGTGTTTCCTTTGCTATGACCCAGCAGAAATACTTCAGTAACTACA
GTCCAGTGATTGGGTTTTACATTTACGAGCCCATTGAATATTGGAACTCA
ACGGTACAGGAGCACCTGAAGACTCTGAGTCATGGCTTCAACAAGATCTC
CTGGATGGACAACTTTTTCCACTACCTGAGGATGGTGAATGTGAGTGCGT
CAACCAAGAGTGACTTCATCAGCATCCTTAAGGGCTCCTTCCTGCGCAGC
CCGGAGTACCAGCACTTCACTGAGGACATCATCTTTACAAAGA---ACCG
CGACACTG------ATGAGTATGACATTATTGCCTCGCGGATGTATTTGG
TGGCGAGGACGACAGAGAAAAAGCGCGAGGAGGTGGTGGAGCTTTTGGAG
AAGCTTCGTCCATTGATGCTGATCAACAGCATCAAATTCATTGCCTTCAA
TCCTACGTTTGTGTTCATGGACCGCTACAGCTCCTCTGTCATCTCACCCA
TTCTGACCTCAGGCTTCAGCGTGCTCACAATCCTCATCCTCACTTTCTTC
CTGGTCATCAACCCCTTGGAAAACTTCTGNNNNNNNNNNNNNNNNNNNNNNNNNNNNNNNNNNNNNNNN
NNNNNNNNNNNNNNNNNNNNNNNNNNNNNNNNNNNNNNNNNNNNNNNGTGTCTACTTCTTGCAGTGTG
GGCATTATTAATGGGCTGTCTGGATGGGTTTCCTCGGTGGATGACTCCCC
AGCTGACACTATCACTCGACGCTTTCGCTATGATGTGGCACTGGTGTCAG
CGATAAAGGATCTGGAGGAGGACATCATGGAGGGGCTGAGAGAGAGCGGG

TTGGAAGACAGTGCATGCAACGCAGGCTTCAGTGTCATGATCAAAGAATC
CTGTGATGGTATGGGCGATGTCAGTGAGAAGCACGGCGGAGGACCGGCTG
TTCCTGAGAAGGCTGTGCGTTTTTCTTTCACTGTTATGTCTGTCTCTGTC
CTGGCAGACGGCAAGAAG------------------------------GA
AGAAGTTACCATCTTCACCGAGCCGAAGCCAAACTCAGAGCTGTCCTGTA
AGCCACTTTGCTTAATGTTTGTGGATGAGTCAGACCATGAGACTCTCACA
GCCATCCTTGGGCCTATAATTGCAGAGCGTGATGCGATGCAGCAGAGCCG
ACTCATCCTGTCCATGGGTGGACTACCTCGATCTTTTCGCTTTCACTTTA
GAGGCACGGGATATGACGAGAAGATGGTGCGTGAGATGGAGGGCCTGGAG
GCCTCGGGATCATCCTACATTTGCACTCTTTGTGACTCCAGTCGTGTAAA
TGCCTCCGAAAACATGGTGCTACACTGCATAACTCGCAGTCATGAGGAGA
ACCTAGAGCGTTATGAATTATGGAGAACCAACCCCTTTTCTGAGTCTGCA
GATGAGCTGCGAGATAGAGTCAAAGGGCTCTCTGCCAAGCCCTTCATGGA
GACCCATCCCACAATGGATGCATTACACTGTGACATCGGCAATGCCACAG
AGTTCTACAAAATCTTCCAGGACGAGATCGGGGAAGTGTACCAAAAGGT-
--CAAC---CCAAGCCGGGAGGAACGGCGCAGCTGGAGGGCCGCCCTAGA
TAAACAGCTGAGGAAGAAGATAAAGCTTAAACCCGTAATGAGGATGAATG
GGAACTTTGCCCGCAAGCTAATGACCCAGGAGGCTGTGGAGGTGGTGTGT
GAGCTAGTGCCTTCGGAGGAGCGCAGACAGGCCCTGAGGGAGCTTATGAG
ACTCTATCTCCAGATGAAGCCAGTGTGGCGCGCCACCTGTCCGGCCAAAG
AGTGCCCAGACCAGCTGTGCCGCTACAGCTTCAACTCTCAGAGCTTTGCC
GACCTCCTCTCCTCTACCTTCAAATATAGATACAAAGGGAAGATAACCAA
TTACCTGCACAAGACCCTGGCCCATGTGCCTGAAATCATAGNNNNNNNNNNNNNNNNNNNNNNNNNNNNNN
NNNNNNNNNNNNNNNNNNNNNNNNNNTCGTACACC
ATCGAAATGAGCCCCCTTGGTCCGGGGTGGAAGGAGAGTCCACAGCCGTT
CTCCTGCTCCATCGAAGACCCGACAAAACAGACCAAGTTCAAAGGCATCA
AGACGTACATCTCGTACCGGGTGACGCCGAGCCACACCGGGCGTCCCGTC
TACAGGCGATACAAACACTTTGACTGGCTTTACAACCGCCTACTGCACAA
GTTCACTGTGATCTCCGTGCCCCACCTGCCYGAGAAGCAGGCCACGGGCC
GCTTTGAGGARGACTTCATCGAGAAACGCAAACGGCGACTGATCTTGTGG
ATGAACCATATGACCAGTCACCCTGTCCTCTCGCAGTACGAAGGCTTCGA
GCACTTTCTTATGTGCGCCGACGACAAGCAGTGGAAGCTTGGAAAGAGGC
GGGCGGAGAAGGACGAGATGGTTGGCGCCCATTTCATGCTGACCCTTCAG
ATCCCCAATGAGCACCAGGACCTTCAGGACGTGGAGGAGCGCATAGACAC
TTTCAAGTCCTTTGCAAAGAAAATGGACGACAGCGTGATGCAGCTTACGC
ACGTGGCCTCAGAGCTGGTTCGCAAACACCTGGGCGGATTCAGGAAGGAG
TTCCAACGGTTAGGAAACTCCTTCCAGTCTATCAGCCAGGCTTTCATGCT
GGATCCACCGCACAGCTCCGAGACCTTAAACAACGCCTTTTCCCACNNNNNNNNNNNNNNNNNNNNNNN
NNNNNNNNNGGTT
TTATCATTGGGGTTGGCGTGGTCGGAAACCTCCTGATTTCCATCCTACTG
GTCAAAGACAAGAGCCTGCACCGAGCGCCCTACTATTTCCTGCTGGACCT
GTGCGCCTCCGACATCCTGCGATCCGCCATCTGCTTCCCCTTTGTCTTCA
CGTCGGTTAAAAACGGTTCGGCCTGGACGTATGGTACACTGACCTGCAAA
GTGATCGCCTTCCTCGGGGTGCTATCCTGTTTCCACACGGCGTTTATGCT
CTTCTGTGTTAGCGTTACCCGCTACCTGGCCATCGCACACCACCGCTTCT
ACACCAAGAGGCTGACCTTCTGGACCTGCCTCGCCGTCATCTGCATGGTG
TGGACGTTGTCGGTGGCTATGGCCTTTCCGCCGGTGCTAGATGTAGGGAC
TTACTCTTTTATCCGCGAGGAGGACCAGTGCACGTTCCAGCACCGCTCCT
TCCGGGCGAACGATTCTCTGGGCTTCATGCTCCTGCTGGCGCTCATCCTC
TTGGCCACACAGCTGGTTTACCTCAAGCTCATCTTCTTCGTCCACGACCG
GCGGAAGATGAAGCCTGTCCAGTTTGTGCCTGCTGTCAGCCAAAACTGGA
CCTTCCACGGACCGGGGGCCAGCGGGCAGGCGGCGGCCAACTGGCTGGCT
GGATTCGGTCGAGGCCCCACCCCGCCTACTTTACTGGGAATCCGGCAGAA

CAGCAATGCAGCGGGCCGCAGGCGTCTCCTGGTGTTGGATGAATTCAAAA
CAGAGAAGAGGATTAGTAGGATGTTCTACATTATGACGTTTTTCTTCCTG
GCTCTCTGGGGGCCCTATCTGGTCGCCTGCTACTGGCGGGTGTTTGCGAG
GGGCCCCGTGGTCCCTGGAGGCTACCTGACGGCAGCCGTGTGGATGAGCT
TTGCNNNNNNNNNNNNNNNNNNNNNNNNNNNNNNNNNNNNNNNNNNNNNNGCCAAATCTCGCTTTCACCC
TGGCGTGGGGACTGGTCCTGGCATGG
AGC---GCAGCGTCCCACTCGGCAACAGCTTGCTGTCCCCGCAGCAAACC
GACGAGTCCACTGTTGCCACCCCCCCGCAGCGATGGTTTGTCACCC---C
TGCCAACAACCGACTGGACTTTGCTGCCTCGGCATACGACGCCGCT----
-----GATTTCGCCGGTAACGCGGCCACCTTGCTGTCCTACGCAGCGGCC
GGAGTGAAGGCTC------TTCCCCTGCCGACTGCAGGCTGCTCCAACCG
GCCTCTTGGCTATTACGCAGACCCGTCCG---GCTGG---GGAGGACGCA
CACCGCCGCAGTACTGTGGTGTAAACAGCAAATCCAGCTCGGTCTTTTCC
TGCTGGCCCGCTAACTCTATCGGTGGCAGAGCGGGCA---CT---AACTA
CCTGG---------CCGAGGA---GGGA---GACTC---CATCACGACGG
AGAGGTCGCCG---CT---CGGCGGCTCGGACGAG---ACCAAACCCAAA
GACATGAC---ATCCGA---GTCGAGCTGGATAGAG---ACGCCGTCCTC
CATTAAGTCAATTGATTCGAGCGATTCTGGTATCTTTG---AACAGGCCA
AAAGGAGAAGAATCTCACCTTCTGCCACGCCA---------GTTTCAGAG
ACAGTGTCCCCGTTAAAATCTGAG--------------------------

>Acanthurus nigricans
AGCCTCCTAATCCGAGCAGAATTAAGCCAACCAGGCGCCCTCCTCGGGGA
TGATCAAATTTATAATGTAATTGTTACAGCACACGCATTCGTAATAATTT
TCTTTATAGTAATACCAATTATGATTGGTGGATTTGGAAATTGATTAATT
CCACTAATGATTGGAGCTCCTGACATAGCATTCCCACGAATAAATAATAT
GAGCTTTTGGCTCCTACCCCCATCCTTCCTGCTTCTACTAGCATCTTCTG
CAGTAGAGTCTGGTGCTGGCACAGGGTGAACAGTATACCCTCCTCTAGCC
GGTAATTTAGCACATGCAGGAGCATCTGTAGACCTAACCATTTTCTCCCT
CCACCTCGCAGGTATTTCTTCAATTCTTGGAGCTATTAATTTTATTACAA
CAATTATTAATATGAAACCTCCTGCTATTTCTCAATATCAAACCCCCCTA
TTTGTATGAGCCGTACTAATTACTGCTGTCCTACTCCTTCTCTCACTTCC
CGTTCTCGCCGCCGGAATTACAATGCTACTAACAGACCGTAATCTAAACA
CTACCTTCTTTGATCCGGCAGGGGGAGGAGACCCCATCCTATACCAACAT
TTATTCTGATTTTTTGGTC-------------------------------

------------TTCCTAGAGAGAAACCTTCATCCATCTAACTGCCTTGG
CATGCTGTTACTGTCTGACGCCCACCAGTGCACCAAGCTCTCYGAGCTCT
CCTGGGGCATGTGCCTGAGCAACTTTCCAGCTATTTGCAAGACTGAGGAC
TTCCTCCAGCTACCCAAAGATATGGTAGTGCAGCTTTTGTCACATGAGGA
RCTAGAGACAGAAGATGAGAGGCTGGTTTATGAGGCTGCCCTCAACTGGA
TCAACTATGACCTGGAAAAGAGGCACTGCCACCTTCCAGAGCTCCTGAGA
ACAGTCCGCCTGGCTCTGCTGCCTGCCATCTTTCTCATGGAGAACGTTTC
TACTGAAGAGTTGATCAACGCCCAGGCCAAGAGCAAAGAGCTGGTGGATG
AAGCCATCCGTTGTAAGCTGAAGATCCTGCAGAATGATGGCGTCGTCAAC
AGCCCGTGTGCTCGACCAAGAAAAACCAGCCATGCTCTCTTCCTTCTGGG
AGGGCAGACCTTTATGTGTGACAAGTTGTATCTGGTGGACCAGAAAGCCA
AAGAGATCATCCCCAAGGCCGACATTCCCAGTCCCAGGAAGGAGTTCAGC
GCCTGCGCCATTGGCTGTAAGGTGTACATCACTGGTGGAA--GAGGCTC-
AGAGAACGGTGTGTCCAAAGATGTATGGGTTTACGATACCGTCCACGAGG
AATGGTCGAAGGCTGCGCCCATGCTTATCGCTAGGTTCGGCCATGGCTCT
GCAGAGCTGAAACACTGTCTCTATGTGGTGGGAGGTCACACTGCTGCGAC
TGGCTGTCTCCCGGCCTCTCCCTCCGGACGAGTACATTGTTGTGTTTAGT
CGGTCTACAACAAGACTCATACTGAATGAAGCCGAGCTCATCATGGCACT
CGCCCAGGAGTTCCAGATGAGAGTGGTCACAGTTTCCCTGGAGGAACAGT
CTTTTCCCAGTATCGTCCAGGTGATCAGCAGTGCTACCATGTTAGTCAGT
ATGCATGGAGCTCAGCTTATCACCTCGCTCTTCCTTCCCAGAGGAGCTGT
TGTGGTGGAGCTGTTCCCCTTTGCTGTGAACCCAGAGCAGTACACCCCAT
ATAAAACCCTAGCTTCTCTTCCAGGTATTGACCTTCACTATATCTCCTGG
AGAAACACCAAGGAGGAGAACACAGTCACTCACCCAGACAGACCCTGGGA
ACAAGGCGGCATCACTCACTTGGAAAAGGAGGAGCAGGAGAGAATACTGG
GGAGCAAAGATGTCCCTAGACACCTGTGCTGCCGCAACCCAGAGTGGCTC
TTCAGGATCTACCAGGACACCTTGGTGGACATCCCCTCCTTTCTGGAAGT
TCTCAA---AGAGGGCATGAAG---ACAAAGCCCAGCCTGAAGAA---GT
CCAAACCGGCCAGCACTGTCCACCCGGGCCGTGTCAGAGAACCCCAGTGT
CAGACTTCAGTACAAACAACTAATGAGGCTAAACTCACAGTCTCCTGGCA
GATTCCATGGAATCTGAAATACCTGAAGGTCAGAGAGGTGAAATACGAGG
TGTGGATCCAGAAAAAAGACACCAGCAAGGGGACTCTGGAGGATCAAATC
ATCCAGGCGAACCCTGCACTGGAGGCCTTCGGCAACGCCAAGACACTAAG
AAACGACAACTCGTCTCGCTTTGGAAAATTTATCAGAATTCACTTTGGTA
CGAGTGGSAAGCTGTCGTCTGCTGACATCGAGACGTACCTGCTGGAGAAG
TCACGTGTCACCTTTCAGCTCAAGGCTGAGAGGAACTATCACATTTTCTA
CCAGATCCTGTCCAATCAGAAGCCAGAGCTGCTCGACATGCTGCTGATCA
CCAACAACCCCTACGACTACTCCTACATCTCCCAAGGAGAGGTGACGGTC
GCCTCCATCAACGACTCGGAGGAGCTGATGGCCACAGACAGCGCCTTCGA
TGTGCTCGGCTTCACCGCAGATGAGAAGATGGGTGTGTACAAGCTGACGG
GCGCCATCATGCACTACGGTAACATGAAGTTCAAACAGAAGCAGCGTGAG
GAGCAGGCTGAGCCGGACGGACGGAGGCTGCTGATAAATCAGCTTACCT
GATGGGGCTGAACTCTGCCGACCTCATCAAAGGGCTTTGCCATCCCAGAG
TCAAGGTGGGAAATGAATATGTCACCAAAGGCCAAAGTGTAGACCAAGTC
TACTACCCCAACAAGGAGGCCTTCAAGTGTGAGGAGTGTGGAAAACATTA
CAACACCAAGCTGGGATATAAGCGCCATGTGGCCATGCACTCTGCCACAG
CGGGGGATCTCACCTGCAAAGTGTGCATGCAGACCTACGAGAGCACACCT
GTGCTCTTGGAGCACCTCAAGAGCCACTCTGGGAAGTCTTCGGGTGGCAC
CAAGGAGAAAAAGCACCCTTGCGACCACTGTGACCGTCGTTTCTACACTC
GTAAGGATGTGAGAAGGCACATGGTGGTCCACACGGGCCGAAAGGACTTC
CTGTGCCAGTACTGTGCCCAACGCTTTGGCAGGAAGGACCACCTGACACG

CCATGTGAAGAAAAGCCACTCACAAGAGCTGCTGAAGATCAAGACGGAGC
CCCCCGATATGTTAGGTCTTTTAGCTTCTGGGTCACCACCTTGTTCTGTG
AAGGAGGAGCTCAGCCCCATGATGTGCGGCATGGGTCCCAACAAAGACCC
CATGATGGGCAAACCGTTCCCCAGTGGGGCCCCTTTCCCGATGGGTATGT
ACAACCCCCACCAT------CTCCAGGCCATGTCTAATTCTGGGGTGGGT
CACCCA------CACCCGTCCCTGATGCCCAGTTCCTTGTCTGCAGCTAT
GGGCATGGGCTGTCATATGGAATATCTCATCTACGCCTCTTTCTCATTCA
TGGGATGTTTACAAATTAGTGATGGATCAAATATCGTGAACCTGCTGGCA
AGTAACTCTCCGAGTGTTTCCTACGCTCTGACCCAGCAAAAATACTTTAG
CAACTACAGTCCCGTGATTGGGTTTTACATTTATGAGCCCATTGAGTACT
GGAACTCCACGGTGCAGGAGCACCTGAAGACTCTAAGYCATGGCTTCAAC
AAGATATCCTGGATGGACAACTTTTTCCACTACCTGCGGGTGGTGAATGT
AAGTGCATCGACCAAGAGCGACTTCATCACCATCCTCAAAGGCTCCTTCT
TGCGCAGCCCGGAGTACCAGCACTTCACTGAGGACATCATATTCTCCAAG
G---ACCGTGAAACTG------GTGAGTATGACATTATTGCCTCACGGAT
GTACTTGGTGGCACGGACGACAGAGAAGAAGCGCGAAGAGGTGGTGGAGC
TTCTGGAAAAGCTGCGTCCATTGATGCTGATCAACAGCATCAAGTTCATT
GCCTTCAATCCCACGTTTGTGTTCATGGACCGTTACAGCTCCTCTGTCAT
CTCGCCCATCCTGACCTCAGGATTCAGCGTACTCACAATCCTCATCCTTA
CTTTCTTCCTGGTCATCAATCCCTTGGGTAACTTCTGGCTCATCCTCACT
GTCACATCTGTGGAGCTTGGCGTCCTGGGTTTGATGGGCTTTCACCAGTT
TGAATGGCAGCCAGCTCTCAAGAATGTGTCTACATCTTGCAATGTTGGCA
TTATTAATGGGCTCTCTGGAAGGGCTTCCTCAGTGGATGACTCCCCGGCT
GACACCATCACTCGACGCTTTCGCTATGATGTGGCACTTGTGTCAGCATT
AAAGGATATGGAGGAGGACATCATGGAGGGGCTGAGAGAGAGTGGGATGG
AAGACAGTGCCTGCACCTCGGGCTTCAGTGTCATGATCAAAGAATCTTGT
GATGGCATGGGCGATGTCAGCGAAAAGCACGGCGGAGGACCAGTTGTTCC
TGAGAAGGCTGTACGTTTTTCTTTCACCATAATGTCTGTTTCTGTCCTGG
CAGACGGTGAGGAG------------------------------GAAGAG
GTTACCATCTTCACCGAGCCAAAGCCAAACTCAGAGCTGTCTTGTAAGCC
TCTTTGCCTGACATTTGTGGATGAGTCAGACCATGAGACACTCACAGCCA
TCCTGTCACCTATAGTTGCAGAGCGTGACGCAATGAAAGAAAGCAGGCTG
ATCCTATCCATYGGTGGTCTATCTCGTGCCTTCCGCTTTCACTTCAGAGG
CACKGGATACGAYGAGAAGATGGTACGTGAGATGGAGGGCCTCGAGGCTT
CAGGGTCCTCATATATCTGCACTCTTTGTGACTCTGGTCGGGCAGAGGCC
TCTCAGAACATGGTGCTACACTCCATCACCCGCAGCCATGAAGAGAACCT
CGAACGTTATGAAGTATGGAGAACCAACCCCTTTTCAGAATCTGTGGAAG
AGCTCAGAGACAGAGTCAAAGGGGTCTCTGCCAAACCCTTTTTGGAGACC
CATCCTACGCTAGATGCATTACACTGTGACATTGGCAATGCCACTGAGTT
CTACAAAATCTTCCAGGACGAGATCGGAGAGGTGTTCCAAAATGC---CA
AC---CCCAGCCGGGAGGAACGGCGCAGCTGGAGGGCAGCCCTCGATAAA
CAGCTGAGGAAGAAGTTAAAGCTTAAACCGGTAATGAGGATGAATGGGAA
CTATGCCCGCCGGCTAATGACTCTTGAGACCGTAGAGGCCGTGTGCGAGC
TGGTGCCCTCAGAAGAGAGGAGAGAGGCCCTGAGGGAGCTTGTGAGGCTC
TACCTCCAGATGAAGCCTGTATGGCGTGCCACCTGCCCAGCCAAGGAGTG
CCCCGATCAGCTGTGCCGCTACAGCTTCAACTCTCAGCGCTTCGCCGAAC
TCTTATCTTCTACCTACAAATATAGGTATAAGGGAAAGATACCCAATTAC
CTGCACAAGACCCTGGCCCATGTCCCTGAAATCATAGAGAGAGAGATGGATC
CATAGGAGCCTGGGCCAGCGAGGGGAACGAATCAGCAAACAAATCGTACA
CCATTGATATGGGGCCCCTGGGCCCCAGGTGGAAGGAGAATCCACAGCCT
TTCTCCTGCTCCATTGAAGACCCCACAAAACAGACAAAGTTTAAAGGCAT
CAAGACGTACATTTCGTACCGSGTCACGCCGAGCCACACGGGGCATCCTG
TCTACAGGCGCTACAAACACTTTGACTGGCTGTACAACCGCTTGCTGCAC

AAGTTCACTGTGATCTCAGTGCCTCACCTGCCTGAGAAGCAGGCCACTGG
GCGATTTGAGGAAGACTTCATCGAGAAGCGCAAGAGGCGGCTGATACTGT
GGATGAACCACATGACCAGTCACCCAGTCCTCTCCCAGTATGAAGGCTTC
GAGCACTTYCTCATGTGTGCCGATGACAAGCAGTGGAAACTTGGCAAGAG
ACGGGCGGAGAAGGACGAGATGGTGGGCGCCCATTTCATGCTCACCCTCC
AGATCCCCAATGAGCACCAGGACCTTCAGGATGTAGAGGAACGGATCGAC
ACCTTCAAATCCTTYGCTAAGAAAATGGATGACAGTGTGATGCAGCTCAC
ACACGTCGCCTCGGAGCTGGTGCGGAAACACCTGGGTGGGTTCAGGAAGG
AGTTCCAGCGGCTGGGAAACGCCTTCCAGTCCATCAGCCAGGCGTTCATG
CTGGACCCTCCTCACAGCTCAGAGGCCCTCAACAANNNNNNNNNNNNNNNNNNNNNNNNGTTCCTCAAAC
TGAC
CTCTCTGGGTTTCATCATCGGAGTCGGTGTGGTTGGGAACCTCCTGATCT
CCATCCTGCTGGTCAAAGACAAGAGTCTGCACCGAGCGCCCTACTACTTC
CTGCTCGACCTGTGCGCCTCCGATATCCTGCGCTCTGCCATCTGCTTCCC
CTTTGTCTTCACCTCGGTCAAGAATGGATCTGCCTGGACGTACGGCACGC
TGACCTGCAAAGTGATCGCCTTCCTGGGTGTGCTTTCCTGTTTCCACACG
GCGTTTATGCTGTTCTGTGTCAGCGTCACACGCTACCTGGCCATCGCACA
TCACCGTTTCTACACCAAGAGGTTGACCTTCTGGACCTGTCTAGCTGTGA
TCTGCATGGTGTGGACGTTGTCAGTGGCTATGGCGTTTCCGCCGGTGCTA
GATGTAGGGACGTACTCTTTTATCCGAGAGGAGGACCAGTGCACATTCCA
GCACCGCTCCTTCAGGGCCAATGATTCGCTGGGCTTCATGCTCCTGCTGG
CRCTCATYCTCCTGGCCACACAGCTGGTTTACCTCAAGCTCATCTTCTTC
GTCCACGACCGTCGAAAGATGAAGCCCGTCCAGTTCGTGCCTGCTGTCAG
CCAGAACTGGACCTTCCATGGGCCAGGTGCCAGCGGGCAGGCGGCAGCCA
ACTGGCTGGCCGGGTTTGGTCGCGGCCCCACCCCACCAACTTTGCTGGGC
ATCCGGCAGAACAGCAACGCAGCGGGCCGCAGGCGTCTACTGGTATTGGA
TGAATTCAAAACAGAGAAGAGGATTAGTAGGATGTTCTACATCATGACGT
TTTTCTTCCTTGCACTGTGGGGGCCCTATCTGGTCGCCTGCTACTGGCGA
GTGTTTGCAAGGGGCCCAGTGGTCCCTGGGGGTTACCTGACAGCAGCCGT
GTGGATGAGCTTTGCCCAGGCTGGGGTCAATCCTTTCATCTGCATCTTCT
CCAACCAGNNNGCCAAATCTCGCTTTCACCCTGGCATGGGGACTGGTCCTGGC
ACGGAGC---GCAGCGTCCCACTCGGCAACAGCTTGCTATCCCCGCAGCA
AAGCGAGGAGCCCACTGTTGCCACCCCCCCGCAGCGATGGTTTGTCACCC
---CTGCCAACAACCGACTGGACTTTGCTGCCTCGGCATACGACGCCGCT
---------GATTTCGCCGGTAACGCGGCCACCTTGCTGTCCTACGCAGC
GGCCGGAGTGAAGGCTC------TTCCCCTGCCGACTGCAGGCTGCTCCA
ATCGGCCTCTTGGCTATTACGCAGACCCGTCAG---GCTGG---GGAGGA
CGCACGCCGCCGCAGTACTGTGGTGTAAATAGCAAATCCAGCTCGGTGTT
TTCCTGCTGGCCCACTAACTCTATCGGWGGCAGGGCGGGCA---GC---A
ATTACCTGG---------CGGAAGA---GGGA---GACCC---CATCCCG
ACAGAGAGGTCACCG---AT---CGGCGGCTCGGAGGAG---ACCAAACC
CAAAGACATGAC---ATCAGA---GTCGAACTGGATAGAG---ACGCCGT
CCTCCATTAAGTCCATCGATTCAAGCGATTCTGGTATCTTTG---AACAG
GCCAAAAGGAGAAGGATCTCACCGTCTGCAACGCCA---------GTTTC
AGAGACTGTGTCCCCGTTAAAATCTGAGCATCACTCAACAGGCGAAGTCA
CAGAGAGAGAAGTGGCGTTGGGGATAAATCCGTTCGCGGATGGATGGGC
GCCTTCAAAATAAACCACAGCTCCCACGATATTGGTTCCGG---ACAAAC
GGCTTTTTCCTCCCAGGCG---CCCGGCTAT---GCAGCAGCTGCCCTGG
GA---CACCATCA------CCACCCGACCCACGTTGGCTCT---TACTCC
ACGGCGGCTTTCAACTCCACCAGGGACTTTCTCTTCAGAAATCGGGGATT
CGGGGACGCCACCAG------------CGCGCAGCACAGTTTGTTCGCCT
C---------CGGAAGTTT---C------GCAGGGCCACATGGACACTCA
GATGCAGCGGGGCACCTGCTCTTCCCGGGGCTCCATGAA---CAAGCGGC

GAGCCATGCGTCTTCCAACGTGGTCAACAGCCAGATGCGGCTGGGCTTCT
CGGGGGACATGTACGGACGGGCCGACCAGTATGGCCACGTTACGAGCCCG
CGGT---CCGACCACTATGCTTCGACCCAGCTGCACGGCTATGGCCCCAT
GAACATGAATATGGCCGCA---CACCACGGAGCAGGGGCCTTCTTTCGAT
ACATGAGGCAGCCGATCAAGCAAGAGCTCATCTGCAAGTGGATCGAGCCG
GAGCAGCTGACAAATCCCAAAAAGTCGTGCAACAAAACTTTTAGCACGAT
GCACGAGCTCGTGACCCATCTGACGGTGGAGCATGTTGGGGGACCGGAGC
AGACCAACCACGTCTGCTTCTGGGAGGATTGCGCCAGAGAAGGAAAGCCA
TTCAAAGCCAAATACAAACTTGTAAATCATATCAGAGTGCACACCGGAGA
AAAGCCCTTTCCGTGTCCGTTCCCGGGCTGTGGCAAA
>Acropoma japonica
AGCCTGCTAATTCGAGCAGAACTGTGCCAGCCGGGCTCTCTTCTGGGAGA
CGATCAAATCTATAATGTAATTGTTACAGCCCATGCCTTTGTGATAATCT
TCTTTATAGTAATACCCATCATGATCGGAGGCTTCGGAAACTGGCTCATT
CCCCTAATAATTGCTGCCCCCGACATAGCATTCCCTCGGATAAATAATAT
GAGCTTTTGACTCCTACCCCCTTCCTTCCTCTTGCTCCTTGCCTCCTCTG
GAGTAGAAGCAGGAGCCGGCACCGGATGAACAGTTTATCCCCCTCTCTCT
AGCAACCTGGCCCACGCAGGAGCGTCCGTAGACCTCGCAATCTTCTCCCT
CCACTTAGCAGGAATTTCATCAATTCTAGGGGCCATTAATTTTATTACAA
CCATTATTAACATAAAACCTCCTGCTATTTCACAGTACCAAACACCCCTC
TTTGTGTGGTCCGTACTAATTACCGCCGTACTCCTCCTTCTCTCCCTCCC
CGTGCTTGCCGCAGGCATTACAATGCTTCTCACAGACCGAAACCTAAATA
CCACCTTCTTCGACCCTGCCGGCGGCGGAGACCCCATCCTCTATCAACAC
CTC---

------------NNNNNNNNNNNNAACCTTCACCCATCTAACTGCCTTGGCATGCTGTTGCT
GTCTGATGCCCACCAGTGCACCAAGCTGTCAGAGCTCTCCTGGGGCATGT
GCCTCAGCAACTTTCCCGCTATTTGTAAGACGGAGGACTTCCTCCAACTG
CCCAAAGATATGGTGGTGCAGCTTTTGTCACATGAGGAGCTAGAGACAGA
AGATGAGAGACTGGTTTATGAAGCTGCCCTTAACTGGATCAACTATGACC
TGGAAAAGAGGCACTGCAACCTTCCAGAGCTCCTGAGAACGGTGCGTCTG
GCCCTGCTGCCTGCCATCTTTCTCATGGAGAACGTTTCTACAGAAGAGCT
GATCAACGCCCAGGCCAAGAGCAAGGAGTTGGTGGATGAAGCTATCCGCT
GTAAGCTGAAGATCCTGCAAAATGATGGCGTCGTTAACAGCCCGTGTGCT
CGACCAAGAAAAACCAGCCATGCCCTCTTTCTTCTGGGAGGGCAGACTTT
CATGTGTGACAAGTTGTACCTGGTGGACCAGAAAGCCAAAGAGATCATCC
CCAAAGCTGACATTCCCAGCCCCAGGAAGGAGTTCAGCGCCTGCGCCATC
GGCTGTAAGGTGTACATCACTGGTGGGA--GAGGCTC-AGAAAATGGTGT
GTCCAAAGATGTGTGGGTCTACGACACCGTCCACGAGGAATGGTCGAAGG
CGGCGCCCATGCTCATTGCCAGGTTTGGCCATGGCTCTGCGGAGCTGAAA
CACTGCCTCTACGTGGTAGGAGNNNNNNNNNNNNNNNNNNNNNNNNNNNNNNNNNNNNNNNNNGGATGAG
TACATTGTTGTGTTCAGTCGT
TCAACGACGAGGCTGATATTGAATGAAGCTGAGCTAATCATGGTGCTGGC
CCAGGAGTTCCAGATGAGAGTGATCACGGTATCCCTGGAGGAACAGTCTT
TCCCCAGTATCGTCCAGGTGATCAGCGGTGCTTCCATGTTAGTCAGTATG
CATGGAGCTCAGCTTATCACCTCACTCTTCCTCCCCAGAGGAGCTGCTGT
GGTGGAGCTGTTCCCCTTTGCTGTGAACCCAGAGCAGTACACCCCGTATA
AAACTCTTGCCTCCCTTCCAGGCATGGACCTTCACTATGTCTCCTGGAGG
AACGCTAAGGAGGAGAACACTATCACCCACCCAGACAGACCCTGGGAGCA
AGGGGGCATTGCTCACTTGGAGAAGGAGGAGCAAGAGCGAATACTGGCGA
GCAAAGATGTCCCCAGGCACCTGTGCTGCCGCAACCCAGAATGGCTCTTC

CGGATCTACCAGGACACATTGGTCGACATCCCTTCCTTCCTGGAAGTCCT
CAA---AGAGGGCATGAAG---ACAAAGCCCAGCTTGAAGAA---GTCAA
AGCCGGCCAGCACAGTCCACCCGGGCCGGGTCAGAGAACCTCAGTGTCAG
ACCTCAGTACAAACCACTAATGAGGCTAAACTCACAGTCTCCTGGCAGAT
CCCGTGGAATCTGAAATTCCTAAAGGTGAGAGAGGTCAAATATGAAGTG-
---------AAGAAAGACACCAGTAAGGGGACATTGGAGGATCAAATCATC
CAGGCGAACCCTGCGCTGGAGGCCTTCGGCAATGCCAAAACATTGAGAAA
TGACAACTCGTCTCGTTTTGGAAAATTCATCAGAATTCACTTTGGTACGA
GCGGCAAGCTGTCATCTGCTGACATCGAGACGTACCTGCTGGAGAAGTCA
CGTGTCACCTTTCAGCTCAAAGCTGAGAGGAACTACCACATCTTCTACCA
GATTCTGTCCAATCAGAAGCCAGAGCTCCTGGACATGCTGTTGATCACCA
ACAACCCGTATGACTACTCCTACATCTCCCAAGGAGAGCTAACGGTTGCT
TCCATCAATGACTCAGAGGAGCTGATGGCCACTGACAGCGCCTTCGATGT
GCTCGGCTTCACTACAGACGAGAAGATGGGCGTCTATAAACTGACTGGCG
CCATCATGCACTACGGCAACATGAAGTTCAAACAGAAGCAGCGTGAGGAG
CAGGCAGAGCCGGATGGGACAGAGGCTGCTGATAAATCAGCTTACCTAAT
GGGGCTGAACTCTGCTGACCTCATCAAAGGGCTGTGCCATCCCAGAGTCA
AGGTAGGAAATGAATACGTCACCAAAGGCCAAAGTGTGGACCAAGTCTAC
TACCCCAACAAGGAGGCCTTCAAGTGTGAGGAGTGTGGGAAGCACTACAA
CACCAAGCTGGGATATAAGCGCCATGTGGCCATGCACTCTGCCACAGCAG
GGGATCTCACCTGTAAAGTGTGCATGCAGACCTACGAGAGCACACCTGTG
CTCTTGGAGCACCTCAAGAGCCACTCTGGGAAGTCTTCGGGTGGCACCAA
GGAGAAAAAACACCCGTGCGATCACTGTGACCGTCGTTTCTACACACGGA
AGGATGTGAGACGGCACATGGTGGTCCACACGGGCCGAAAGGACTTCCTG
TGCCAGTACTGTGCCCAGCGCTTCGGCAGGAAGGACCACCTGACGCGCCA
TGTGAAGAAGAGCCACTCTCAGGAGCTGCTGAAGATCAAGACAGAGCCTC
CTGATATGTTAGGTCTTTTAGCGTCGGGGTCACCACCCTGCTCTGTGAAG
GAGGAGCTCAGCCCCATGATGTGTGGCATGGGACCCAACAAAGACCCCAT
GATGGGCAAACCGTTCCCCAGTGGGGCCCCTTTCCCGATGGGCATGTACA
ACCCCCACCAT------CTCCAGGCCATGTCTAATTCTGGGGTGGGTCAC
CCA------CACCCGTCCCTAATGCCTAGTTCCTTGTCTGCAGCTATGGG
CATGGGCTGTCACATGGAATATCTCATCTACGCCTCTTTCTCATTCATGG
GATGTTTACAAATCAGTGATGGCTCAAATATCGTTAACCTGCTGGCTAGT
AACTCTCCGAGTGTTTCGTATGCTCTGACCCAGCAGAAATACTTCAGTAA
CTACAGTCCTGTAATTGGGTTTTACATTTATGAGCCCATAGAGTACTGGA
ACTCAACGGTGCAGGAGCACCTGAAGACTCTGAGTCATGGCTTCAACAAG
ATCTCCTGGATGGACAACTTTTTCCACTACCTGCGGGTGGTGAATGTGAG
TGCATCAACCAAGAGCGACTTCATCACCATCCTCAAGGGCTCCTTCCTGC
GCAGCCCGGAGTACCAGCACTTCACTGAGGACATCATATTCTCCAAGA--
-ACCGCGAGACTG------ATGAGTACGACATTATCGCCTCACGGATGTA
CTTGGTGGCACGGACGACAGAGAAGAAGCGTGAAGAGGTGGTAGAGCTTC
TGGAGAAGCTTCGTCCGTTGATGCTGATCAACAGCATCAAGTTCATTGCC
TTCAATCCTACATTTGTCTTCATGGACCGCTACAGCTCCTCTGTCATCTC
GCCCATCCTGACCTCAGGCTTCAGCGTACTCACAATCCTCATCCTCACTT
TCTTCCTGGTCATAAACCCCTTGGGGAACTTCTGGCTCATCCTCACTGTA
ACGTCCGTGGAGCTGGGCGTCTTGGGTTTGATGGGCTTTCACCAGTTTGA
ATGGCAGCCAGCTCTCAGGAATGTGTCTACATCTTGCAATGTTGGCATTA
TTAATGGGCTCTCTGGATGGGCTTCCTCGGTGGATGATTCCCCAGCTGAC
ACCATCACTCGACGGTTTCGCTATGATGTCGCGCTGGCATCAGCAATAAA
GGATCTGGAGGAGGACATAATGGAGGGACTGAGAGAGAGTGGGATGGAAG
ACAGTGTGTGCACCTCAGGCTTCAGTGTCATGATCAAGGAATCCTGTGAT
GGCATGGGCGATGTCAGCGAGAAGCACGGTGGAGGACCAGTTGTTCCTGA
GAAGGCTGTACGTTTCTCTTTCACTATTATGTCTGTCTCGGTCTTGGCAG

ACGATGAGGGG-------------------------------AAAGAGGTT
ACCATCTTCACCGAGCCAAAGCCAAACTCAGAACTGTCCTGTAAGCCTCT
TTGCCTGACGTTTGTGGATGAGTCAGACCATGAGACACTCACAGCCATCC
TAGGGCCTATAGTTGCAGAGCGTACTGCGATGAAAGAAAGTAGGCTCATC
CTATCCATGGGTGGACTACCTCGTTCCTTTCGCTTTCACTTCAGAGGCAC
AGGATACGATGAGAAGATGGTACGTGAGATGGAGGGCCTCGAGGCCTCAG
GGTCCTCCTATATCTGCACGCTTTGTGACTCCAGTCGGACAGAAGCCTCT
CAAAACATGGTGCTACACTCCGTCACTCGGAGTCATGACGAGAACCTAGA
ACGTTATGAAATATGGAGAACCAACCCCTTTTCTGAGTCTGTAGATGAGC
TGAGAGACAGAGTCAAAGGGGTATCTGCCAAGCCTTTCATGGAGACCCAT
CCCACGCTGGATGCATTACACTGTGACATTGGCAATGCCACTGAGTTCTA
CAAAATCTTCCAGGACGAGATCGGAGAGGTGTACCAAAAGGT---CAAC-
--CCCAGCCGGGAGGAACGGCGCAGCTGGAGGGCAGCCCTAGATAAACAG
CTGAGGAAGAAGATAAAGCTTAAACCGGTAATGAGGATGAATGGGAACTA
TGCACGCAGGCTAATGACCCAGGAGGCTGTGGATGTGGTGTGTGAGCTGG
TGCCCTCAGAAGGGAGGAGGGAGGCCCTGAGGGAGCTTATGAGGCTCTAC
CTCCAGATGAAGCCTGTGTGGCGCGCCACCTGCCCAGCCAAGGAGTGCCC
CGACCAGCTGTGCCGCTACAGCTTTAACTCCCAGCGCTTTGCCGACCTCC
TCTCCTCTACCTTCAAATATAGGTACAATGGAAAGATAACCAATTACCTG
CACAAGACCCTGGCCCATGTGCCTGAAATCATAGAGAGAGATGGATCCAT
AGGAGCCTGGGCCAGCGAGGGGAACGAGTCGGCAAACAAATCGTACACCA
TCGAGATGTGCCCCTTGGGGCCCGGGTGGAAGGAGAGCCCACAGCCTTTC
TCCTGCTCCATTGAAGACCCCACGAAACAGACAAAGTTCAAGGGCATCAA
GACATACATTTCGTACCGGGTCACGCCGAGCCACACAGGGCGTCCCGTCT
ACAGGCGCTACAAACACTTTGACTGGCTGTACAACCGCTTACTGCACAAG
TTCACTGTGATCTCCGTGCCGCACCTGCCTGAGAAGCAGGCCACGGGGCG
ATTTGAGGAAGACTTCATCGAGAAGCGTAAGAGGCGACTGATACTGTGGA
TGAACCACATGACCAGTCACCCAGTCCTCTCCCAGTATGAAGGCTTTGAG
CACTTTCTGATGTGCGCTGACGACAAGCAGTGGAAACTGGGCAAGAGACG
GGCGGAGAAGGACGAGATGGTGGGCGCCCATTTCATGCTGACCCTCCAGA
TCCCTAACGAGCACCAGGACCTTCAGGACGTAGAGGAACGGATCGACTCC
TTCAAGTCCTTTGCTAAGAAAATGGATGACAGCGTGATGCAGCTCACGCA
TGTTGCCTCAGAGCTGGTGCGTAAGCACCTGGGTGGATTCAGGAAGGAGT
TCCAGCGGCTGGGAAATGCCTTCCAGTCTATCAGCCAGGCGTTCATGCTG
GACCCTCCGCACAGCTCAGAGACCTTCAACAACGCCATCTCCCATCCTCT
CACCACGTTCCTCAAACTGACCTCTCTGGGTTTCATCATTGGAGTCGGTG
TGGTTGGAAACCTCCTGATCTCCATCCTGCTGGTCAAAGACAAGAGCCTG
CACCGGGCGCCCTACTATTTCCTGCTGGACCTGTGCGCCTCTGATATCCT
GCGCTCCGCCATCTGCTTCCCCTTTGTTTTCACCTCGGTCAAGAATGGAT
CTATCTGGACCTATGGCACGCTGACCTGCAAAGTGATCGCCTTCCTGGGG
GTGCTCTCCTGTTTCCACACGGCGTTTATGCTGTTCTGTGTCAGCGTCAC
GCGCTACCTGGCCATCGCACATCACCGTTTCTACACCAAGAGGCTGACCT
TCTGGACCTGTCTAGCTGTCATSTGCATGGTGTGGACGTTGTCAGTGGCT
ATGGCGTTCCCGCCGGTGTTAGACGTAGGGACGTACTCTTTTATCCAGGA
GGAGGACCAGTGCACCTTCCAGCACCGTTCCTTCAGGGCGAATGATTCGC
TGGGCTTCATGCTCCTGCTGGCGCTCATCCTCCTGGCCACACAGCTGGTT
TACCTCAAGCTCATCTTCTTCGTCCACGACCGTCGAAAGATGAAGCCTGT
CCAGTTCGTGCCTGCTGTCAGCCAGAACTGGACCTTCCACGGGCCAGGCG
CCAGCGGGCAGGCGGCGGCCAACTGGTTGGCCGGATTTGGTCGAGGTCCC
ACCCCGCCTACTTTGCTGGGCATCCGGCAGAACAGCAACGCAGCGGGCCG
CAGGCGTCTACTGGTATTGGATGAATTCAAAACAGAGAAGAGGATTAGTA
GGATGTTCTACATCATGACGTTTTTCTTCCTGGCACTGTGGGGGCCCTAT
CTGGTCGCCTGCTACTGGCGGGTGTTTGCAAGGGGCCCTTTGGTCCCTGG

GGGCTACCTGACGGCAGCCGTGTGGATGAGCTTTGCCCAGGCTGGGGTCA
ATCCTTNNNNNNNNNNNNNNNNNNNNNNNNNNNNNNNNNNNNNNNNTCACCCTGGCATGGGGACTGGTCC
TGGCACGGAGC---GCAGCG
TCCCACTCGGCAACAGCTTGCTATCCCCGCAGCAAACCGAGGAGCCCACT
GTTGCCACCCCCCCGCAGCGATGGTTTGTCACCC---CTGCCAACAACCG
ACTGGACTTTGCTGCCTCGGCATACGACGCCGCT---------GATTTCG
CCGGTAACGCGGCCACCTTGCTGTCCTACGCAGCGGCCGGAGTGAAGGCT
C------TTCCCCTGCCGACTGCAGGCTGCTCCAACCGGCCTCTTGGCTA
TTACGCAGACCCATCAG---GCTGG---GGAGGACGCACGCCGCCGCAGT
ACTGTGGTGTAAATAGCAAATCCAGCTCGGTCTTTTCCTGCTGGCCCGCT
AACTCTATCGGTGGCAGAGCAGGCA---CC---AACTACCTGG-------
--CGGAGGA---GGGA---GACTC---CATCCCGACGGAGAGGTCACCG-
--AT---CGGCGGCTCGGAGGAG---ACCAAACCCAAAGACCTGAC---A
TCAGA---GTCCAGCTGGATAGAG---ACGCCCTCCTCCATTAAGTCCAT
TGATTCAAGCGATTCTGGTATCTTTG---AACAGGCCAAAAGGAGGAGAA
TCTCACCTTCTGCCACGCCG---------GTTTCAGAGACTGTGTCCCAC
CTAAANNNNNNNNNNNNNNNNNNNNNNNNAGTCACAGAGAGAGAAGTGGCGTTGGGGATAAATCCGTTCG
CGGA
TGGGATGGGCGCCTTCAAAATAAACCACAGCTCCCATGATATTGGCTCCG
G---ACAAACGGCGTTTTCCTCCCAGGCG---CCCGGCTAC---GCAGCT
GCCGCCCTGGGA---CACCATCA------CCACCCGACCCACGTTGGCTC
T---TACTCCACGGCGGCTTTCAACTCCACCAGGGACTTTCTGTTCAGAA
ATCGGGGTTTCGGGGACGCCGCCGG------------GGCGCAGCACAGT
TTGTTCGCCTC---------CGGAAGTTT---C------GCAGGGCCACA
TGGACACTCAGATGCAGCGGGGCACCTGCTCTTCCCGGGGCTCCACGAG-
--CAAGCGGCGAGCCATGCGTCTTCCAACGTGGTCAACAGCCAGATGCGG
CTGGGCTTCTCGGGGGACATGTACGGACGGGCCGATCAGTACGGCCACGT
TACGAGCCCGCGGT---CCGACCACTATGCTTCGACCCAGCTGCACGGCT
ATGGCCCCATGAACATGAATATGGCCGCA---CACCACGGAGCAGGGGCC
TTCTTTCGATACATGAGGCAGCCGATCAAGCAAGAGCTCATCTGCAAGTG
GATCGAGCCGGAGCAGCTGACGAATCCCAAAAAGTCGTGCAACAAAACTT
TTAGCACGATGCACGAGCTCGTGACCCATCTGACGGTGGAGCATGTGGGG
GGACCAGAGCAGACCAACCACATCTGCTTCTGGGAGGANNNNNNNNNNNNNNNNNNNNNNNNNNNNNNNN
NNNNNNNNNNNNNNNNNNNNNNNNNNNNNNNNNNNNNNNNNNNNNNNNNNNNNNNNNNNNNNNNNNNNNN
NNNNNNN
>Aeoliscus strigatus

------------TTCCTGGAACGAAACCTTCACCCATCTAACTGCCTTGG

CATGCTGTTGCTCTCTGATGCCCACCAGTGTACCAAGCTGTCCGAGCTCT
CCTGGGGCATGTGTCTCAGCAACTTCCCTGCTATTTGCAAGACCGAGGAC
TTCCTCCAGCTACCGAAAGATATGGTAGTTCAGCTTCTGTCACATGAGGA
GCTGGAGACGGAAGATGAGCGTCTGGTTTATGAAGCTGCCCTGAACTGGA
TCAACTACGACCTGGAAAGGAGGCACTGCCACCTTCCAGAACTCCTCAGA
ACAGTCCGTCTGGCCCTTTTGCCCGCTATCTTTCTAATGGAGAATGTCTC
AATGGAAGAGTTGATCAATTCCCAGGCCAAGAGCAAGGAGCTGGTGGATG
AGGCTATCCGTTGTAAGCTGAAGATTCTGCAGAACGACGGTGTCGTCAAC
AGCCAGTGTGCCCGCCCAAGAAAAACCAGCCATGCCCTCTTTTTACTGGG
AGGGCAGACGTTCATGTGTGACAAGTTGTACCTGGTTGACCAGAAGGCCA
AAGAGATCATTCCCAAAGCAGACATTCCCAGTCCCAGGAAGGAATTCAGC
GCCTGCGCCATTGGCTGTAAGGTGTATATCACAGGTGGAA--GAGGATC-
GGAGAACGGCGTATCCAAAGATGTATGGGTCTACGACACCGTCCACGAAG
AATGGTCGAAAGCTGCGCCCATGCTGATAGCCAGATTTGGTCATGGCTCT
GCTGAGCTGAAACACTGCCTGTATGTGGTGGGAGGTCACACTGCCGCAAC
GGGCTGCCTACCCGCATCTCCGTCG-------------------------

-----------AAAAAAGATGCCAGCAAGGGGACACTGGAGGATCAAATC
ATCCAGGCCAATCCTGCGCTTGAGGCCTTCGGAAATGCCAAGACGGTGAG
AAACGACAACTCATCCCGCTTTGGAAAATTCATCCGAATTCACTTTGGAA
CCAGTGGCAAGCTTTCATCTGCTGACATCGAGACGTACCTGTTGGAGAAG
TCACGTGTCACCTTTCAGCTCAAGGCTGAGAGGAACTACCACATCTTCTA
CCAGATCCTGTCCAACCAGAAGCCAGAGCTACTGGACATGTTGTTAATCA
CCAACAACCCGTACGACTACTCCTACATCTCCCAGGGAGAAGTAACTGTC
GCTTCTATCAATGACTCTGAGGAGCTGATGGCCACCGACAGCGCCTTTGA
CGTGCTCGGATTCACTCCAGAGGAGAAGATGGGCGTCTACAAGCTGACAG
GTGCCATAATGCACTATGGCAACATGAAGTTCAAACAGAAGCAGCGTGAG
GAGCAGGCTGAGCCCGACGGAACAGAGGCTGCTGATAAGTCAGCGTACCT
GATGGGNCTTAACTCTGCCGACCTGATCAAAGGTCTGTGCCATCCCAGAGT
CAAGGTGGGGAACGAGTATGTCACCAAGGGCCAGAGTGTGGACCAGGTCT
ACTAC---


```
------------------------------------------------------------
------------------------------------------------------------
------------------------------------------------------------
-------------------------TATCTCATTTACGCTTCCTTTTCGTTCAT
GGGATGTTTACAAATCAGTGACGGTTCAAATATTGTAAACCTCTTGGCGA
GCAATTCGCCAAGTGTTTCATACGCTCTGACCCAGCAGAAATACTTTAGC
AACTACAGTCCCGTCATTGGCTTTTACATTTACGAACCCATCGAGTACTG
GAACTCCACGGTGCAGGAGCACCTGAAGACTTTGAGTCACGGCTTCAACA
AGATCTCCTGGATGGACAACTTCTTCCACTACCTGCGGGTGGTGAACGTG
AGTGCATCGACCAAGGGTGACTTCATCTCCAACCTCAAAGGCTCCTTCCT
GCGGAGCCCTGAGTACCAGCACTTCACAGAGGACATAATATTCACCAAGA
---ACCGCGAGACAG------ATGAATACGACATTATCGCCTCACGGATG
TACCTGGTGGCACGGACCACAGAGAAGAAGCGTGAGGAGGTGGTGGAGCT
TCTCGAGAAGCTTCGCCCTCTGATGCTGATCAACAGCATCAAGTTCATTG
CCTTCAATCCCACTTTTGTGTTCATGGACCGCTACAGCTCCTCCGTTATC
TCACCCATCCTGACCTCAGGCTTCAGCGTGCTCACCATACTTATTCTCAC
TTTCTTCCTGGTCATCAACCCCTTGGGGAACTTCTGGCTCATCCTCACGG
TCACATCCGTGGAGCTAGGAGTCTTGGGTTTGATGGGTTTTCACCATTTT
GAATGGCAGCCAGCGCTAAAAAATGTTTCCGCATCTTCAAATGTAGGCAT
TATGAATGGGCTCTCTGGATGGGTGTCCTCACTGGACGACTCACCAGGTG
ATACCATCACCCGGCGATTTCGCTATGATGTGGCTCTCGTGTCAGCACTA
AAGGATCTGGAAGAGGACATCGTGGAGGGGCTGAGAGAGAACAGGATGGA
AGACAGTGCTTGCACTTCAGGCTTCAGTGTCATGATCAAAGAATCTTGTG
ATGGTATGGGTGATGTTAGTGAAAAGCATGGTGGAGGGCCAGCTGTCCCA
GAGAAGGCTGTGCGTTTCTCTTTCACTCTTATGTCCATCTCTGTCCTGCC
AGATGGTGACACT------------------------------GAGGCTG
TAACCATCTTTACTGAGCCAAAGCCAAACTCGGAACTGTCCTGCAAGCCC
CTTTGTCTCATGTTTGTTGATGAATCAGACCATGAGACACTCACAGCTGT
CCTGGGGCCTATAGTTGCAGAACGAAATGCAATGAAGGAAAGCAGACTCA
TCCTATCCATCGGTGGCCTACTTCGCTCCTTCCGATTCCACTTCAGGGGT
ACAGGATATGATGAGAAGATGGTGCGTGAAATGGAGGGTCTGGAGGCCTC
GGGGTCCACCTATATCTGCACTCTGTGTGACACCAGTCGTGCTGAGGCCT
CTGAAAACATGGTACTACACTCTATTACACGTAGCCATGATGAGAACCTA
GAACGTTATGAAATATGGAGAACAAATCCCTTTTCTGAGCCTATAGACGA
GCTGCGGGACAGAGTGAAAGGAGTTTCTGCCAAGCCATTCCTGGAGACTC
AGTCCACACTAGATGCATTACACTGTGACATTGGCAATGCCACTGAGTTC
TACAAAATTTTCCAGGATGAAATTGGTGAAGTGCACCAAAAGGT---CAA
T---CCCAGCCGCGAAGAACGGCGCAGCTGGAGGGCTGCCCTTGATAAAG
AGTTAAGGAGGAAGCTGAAGCTTAAACCAGTRATGAGGATGAATGGTAAC
TATGCCCGCAAGCTAATGACACTAGAGGCTGTGGAGGTGGTGTGTGAGCT
GGTGCCCTCGGAGGAGAGGAGGGAGGCCTTGAGGGAGCTTATGAGGCTCT
ACCTCCAGATGAAGCCCGTGTGGCGCACCACCTGCCCAGCCAAGGAGTGC
CCTGACCAGCTGTGCCGCTACAGCTTTAACTCCCAACGCTTTGCTGACCT
CCTCTCCTCTACCTTCAAATATAGGTACAATGGAAAGATAACCAATTACC
TGCACAAGACCCTTGCCCATGTCCCTGAAATCATAGAGAGAGATGGATCT
ATCGGTGCCTGGGCTAGTGAGGGAAATGAGTCGGCAAACAAGTCATACAC
CATCGAGATGGGCCCTGTGGGGCCCCTGTGGAAAGAGAGCCCACAGCCTT
TCTCATGCTCCATTGAAGACCCTACCAAACAGACAAAGTTTAAAGGCATC
AAAACCTACATCTCATACCGTGTCACACCGAGCCACACAGGTCATCCTGT
ATACAGGCGCTACAAACACTTTGACTGGCTGTACAACCGCCTTCTGCACA
AGTTCACTGTGATCTCTGTGCCTCACCTGCCTGAGAAGCAGGCCACGGGG
CGATTTGAGGAAGACTTTATTGAGAAGCGCAAGAGACGACTGATACTGTG
GATGAACCACATGACCAGTCACCCAGTTCTTTCCCAATATGAGGGCTTTG
```

AGCACTTTCTGATGTGTGCGGATGACAAGCAGTGGAAACTGGGTAAGAGA
CGAGCTGAGAAGGATGAGATGGTGGGTGCCCACTTCATGCTGACCCTCCA
GATCCCTCAAGAGCACCAGGATCTTCAGGATGTAGAGGAGAGAGTTGACT
CCTTTAAGGCCTTTGCAAAGAAAATGGATGACAGTGTCATGCAGCTCACT
CACGTTGCCTCAGAGCTGGTCCGCAAGCACCTGGGTGGATTCAGGAAGGA
ATTCCAGAGACTGGGAAATTCTTTCCAGTCCATCAGCCAGTCATTTATGC
TGGATCCTCCCCACAGCTCAGAGGCCCTCAACAAGCCNNNNNNNNNNNNNNNNNNNNCGTTCCTCAAACT
GACCTCTCTGGGTTTTATCATTGGAGTYGGTGTGGTTGGGAACCTCCTGA
TCTCCATCCTGCTGGTCAAAGACAAGAGCCTGCACCGAGCACCCTACTAT
TTCCTGCTGGACCTGTGCGCCTCCGACATCCTGCGCTCAGCCATCTGCTT
CCCTTTCGTCTTCACCTCCGTCAAGAATGGCTCTGCTTGGACTTACGGCA
CGCTGACCTGCAAAGTGATCGCCTTCCTGGGGGTGTTGTCCTGTTTCCAC
ACGGCGTTCATGCTCTTCTGCGTCAGCGTCACCCGCTAYTTGGCCATCGC
ACATCACCGTTTCTACACCAAGCGGCTCACCTTCTGGACCTGCCTGGCCG
TCATCTGCATGGTGTGGACTCTGTCGGTGGCTATGGCGTTCCCGCCGGTG
TTGGATGTCGGGACGTATTCTTTTATTCAGGAGGAGGACCAGTGCACGTT
CCAGCACCGTTCCTTCAGGGCGAATGATTCACTGGGCTTCATGCTGCTGC
TGGCGCTCATCCTCCTGGCCACACAGCTGGTTTACCTCAAGCTCATCTTC
TTTGTCCACGACCGCCGAAAGATGAAGCCCGTCCAGTTCGTGCCTGCTGT
CAGCCAGAACTGGACATTCCACGGGCCGGGGGCCAGCGGGCAGGCAGCGG
CAAACTGGTTAGCTGGATTTGGTAGAGGCCCCACGCCGCCGACTCTGCTG
GGCATCCGGCAGAACAGCAACGCAGCGGGCCGCAGGCGTCTGCTGGTGTT
GGATGAGTTCAAAACAGAGAAGAGGATTAGTAGGATGTTCTACATCATGA
CGTTTTTCTTCCTGGCGCTGTGGGGGCCCTATCTGGTGGCCTGCTACTGG
CGGGTGTTTGCGAGGGGCCCCGTGGTCCCTGCGGGCTACCTTACGGCGGC
CGTGTGGATGAGCTTTGCCCAGGCTGGGGTCAATCCTTTCATCTGCNNNNNNNNNNNNNNNNNN----
----

----------------NNNNNNNNNNNNNGCGAAGTCACAGAGAGAGAAGTGGCGCTGGGGAT
AAATCCGTTTGCAGATGGGATGGGCGCTTTCAAAATCAACCACAGCTCCC
ACGACCTGGGCTCCGG---ACAGACGGCGTTCTCCTCCCAGGCG---CCC
GGCTAC---GCAGCAGCCGCCTTAGGACACCACCACCA------CCACCC
GACCCACGTTGGCTCT---TACTCCACGGCGGCTTTCAACTCCACCAGGG
ACTTTCTGTTCAGAAATCGGGGTTTCGGGGACGCCACCGG----------
--CGCTCAGCACAGCTTGTTTGCCTC---------CGGAAGTTT---C--
----GCAGGGCCACACGGACACTCGGAGGCGGCGGGGCACCTGCTCTTCC
CGGGGCTCCACGAA---CAGGCGGCGAGTCATGCGTCGTCCAACGTGGTC
AACAGCCAGATGCGTCTGGGCTTCTCGGGGGACATGTACGGACGGCCCGA
CCAGTACGGCCACGTCACGAGCCCACGGT---CCGA---CTATGCTTCGA
CTCAGCTGCACGGCTACGGCCCCATGAACATGAATATGGCCGCG---CAC
CACGGAGCGGGGGCCTTCTTTCGGTACATGCGGCAGCCGATCAAGCAAGA

GCTCATCTGCAAGTGGGTCGAGCCGGAGCAGCTGACGAATCCCAAAAAGT
CGTGCAACAAAACTTTCAGCACGATGCACGAGCTGGTGACCCATCTGACG
GTGGAGCATGTGGGGGGGCCGGAGCAGACCAACCACATCTGCTTCTGGGA
GGACTGCTCCAGAGAGGGGAAGCCGTTCAAAGCCAAATACAAACTTGTAA
ATCACATCCGAGTACACACCGGAGAAAAACCCTTTCCGTGCCCGTTCCCA
GGCTGCGGCAAA
>Albula vulpes
AGTCTTCTAATCCGGGCGGAGTTAAGTCAGCCTGGGGCTTTATTGGGAGA
TGATCAAATTTACAATGTTATTGTTACAGCACATGCTTTTGTAATGATTT
TCTTTATAGTGATGCCCATTTTAATTGGTGGGTTCGGTAACTGGCTTGTT
CCTCTTATGATTGGGGCTCCGGATATGGCGTTTCCTCGTATGAATAACAT
GAGCTTTTGGCTCCTTCCCCCTTCTTTCCTGCTTCTTTTAGCTTCTTCTG
GGGTGGAAGCAGGGGCTGGGACGGGATGGACGGTTTATCCTCCTCTTGCT
GGTAACTTAGCTCACGCTGGGGCGTCGGTAGACTTAACCATTTTTTCTCT
CCATCTCGCAGGTGTCTCTTCAATTCTTGGGGCTATTAATTTCATTACCA
CAATCATTAATATGAAACCTCCTGCTATTTCCCAGTATCAGACTCCTCTG
TTTGTCTGATCTGTCTTAGTTACTGCGGTTCTTCTCCTGTTGTCGTTGCC
CGTGTTAGCCGCTGGCATTACAATGCTTCTTACTGACCGTAATCTTAATA
CTACTTTCTTTGATCCAGCGGGGGGTGGTGATCCCATTCTTTATCAGCAT
TTANNNNNNNNNNNNNNNNNNNNNNNNNNNNNNNNNNNNNNNNNNNNNNNNNNNNNNNNNNNNNNNNNNN
NNNNNNNNNNNNNNNNNNNNNNNNNNNNNNNNNNNNNNNNNNNNNNNNNNNNNNNNNNNNNNNNNNNNNN
NNNNNNNNNNNNNNNNNNNNNNNNNNNNNNNNNNNNNNNNNNNNNNNNNNNNNNNNNNNNNNNNNNNNNN
NNTTTCTCGAGAGGAACCTGCACCCCACCAACTGTCTGGGCATGCTGCT
GCTGTCGGACGCCCATCAGTGCGCCAAGCTGTCAGAGCTCTCCTGGGGAA
TGTGCCTGAGCAACTTCCCCACCATCTGCAAGACGGAGGACTTCCTGCAG
CTTCCCAAAGACATGCTGGTGCAGCTGCTGTCCCACGAGGAGTTGGAGAC
GGAGGATGAGAGGCTGGTCTACGAGTCGGCCCTCAACTGGGTCAACTACG
ACCTGGAGAGGAGGCACTGCCACCTGCCGGAGTTGCTTCGCACAGTGCGT
CTGGCTCTCCTTCCCGCCATCTTCCTCATGGAGAATGTCTCCACTGAGGA
GCTCATCAACAAACAGGCAAAAAGCAAAGAGCTGGTGGACGAAGCCATCC
GCTGTAAGTTAAAGATATTGCAGAATGATGGGGTGGTCACCAGCTTGTGT
GCCCGGCCAAGGAAGACCAGCCACGCCCTCTTCCTCTTAGGAGGCCAGAC
CTTCATGTGCGACAAGCTGTACCTGGTGGACCAGAAGGCTAAAGAGATCA
TCCCCAAGGCCGACATCCCCAGCCCCCGCAAGGAGTTTAGTGCCTGCGCC
ATTGGCTGCAAGGTCTATGTTACGGGTGGCC--GAGGCTC-AGAGAATGG
AGTCTCTAAGGACGTTTGGGTCTATGACACTCTGCATGAAGAATGGTGCA
AAGCCGCCCCCATGCTTATCGCCCGGTTTGGTCACGGCTCAGCTGAGCTC
AGGCACTGCCTGTATGTGGTGGGTGGACACACGGCCGCCACAGGCTGCCT
CCCCGCCTCCCCATCA----------------------------------


```
--AAGAAGGATACCAGCAAGGGAACGCTGGAGGATCAAATCATCCAAGCT
AACCCAGCTTTAGAGGCTTTTGGCAATGCCAAAACTGTGAGGAACGACAA
CTCCTCACGTTTCGGGAAATTCATCCGCATTCATTTTGGAGTAAGTGGCA
AACTGTCCTCTGCTGACATTGAAACGTACCTGCTTGAGAAATCTCGTGTC
ACTTTTCAGCTCAAAGCTGAGAGGAACTACCACATCTTTTTCCAGATTTT
GTCCAACAAAAAGCCAGAACTACTGGACATGCTGTTGATCACTAACAACC
CATATGATTATGCATATGTCTCCCAAGGAGAGGTGTCGGTTGCTTCAATT
GATGACTCAGAGGAACTGATTGCAACCGACAGTGCCTTTGATGTGTTGGG
CTTCACAGCAGRTGAGAAGATGGGTGTTTACAAGTTGACAGGCGCCATCA
TGCACTACGGCAATATGAAGTTCAAGCAGAAGCAGCGCGAGGAGCAGGCA
GAGCCTGACGGCACCGAGTCAGCTGACAAATCAGCCTACCTAATGGGGCT
CAACTCGGCTGACCTCCTTAAGGGACTATGCCATCCAAGGGTTAAAGTCG
GCAATGAATATGTCACTAAAGGCCAAAATGTAGATCAAGTCTACTAC---
--------------------------------------------------
--------------------------------------------------
--------------------------------------------------
--------------------------------------------------
--------------------------------------------------
--------------------------------------------------
--------------------------------------------------
--------------------------------------------------
--------------------------------------------------
--------------------------------------------------
--------------------------------------------------
--------------------------------------------------
--------------------------------------------------
-------------TACCTAATTTATGCATCTTTTTCCTTCATGGGATGTT
TACAAATCAGTGATGGATCTAATATTGTCAACCTCTTAGCCAGCAACTCC
CCAAGCGTCTCCTATGCTCTGACACAGCAGAAATATTTCAGCAATTACAG
TCCGGTGATTGGATTCTACATATATGAACCTATAGAGTACTGGAATTCCA
CAGTTCAAGAACACCTGAAGACCCTGGGCAATGGATTCAACAAGATTTCA
TGGATCGACAACTTCTTTCAGTATCTGAAGGTAGTGAATGTCAGTGCGTC
AACCAAAAGTGAATTCATAACCATCCTGCAGACTTCCTTCTTGAGGAGCC
CAGAGTACCAGCACTTCAAAGATGACATCATTTTCTATAAAA---TGAGG
G------------ATGAAACTGAGATCATTGCATCCCGGATGTACTTGGT
GGCCAGAACCACAGAGAAGACGAGGGAGGAGGTGGTGGAATTACTGGAGA
GACTGAGACCTCTGTCCCTCATCAACAGCATAAAATTCATAGTCTTCAAC
CCCACATTTGTGTTCATGGATCGATACAGCTCCTCTGTCATTTCTCCAAT
CCTGACCTCTGGATTCAGCGTCCTGATTGTCTTGATCCTGACATTCTTTC
TGGTCATCAACCCCCTCGGAAACTTCTGGTTGATTCTGACTGTCACCTCA
GTGGAGCTGGGAGTTCTTGGCCTAATG-----------------------
--------------------------------------------------
--------------------------------------------------
--------------------------------------------------
--------------------------------------------------
--------------------------------------------------
---------AGCGAGAAGCATGGGGGTGGTCCAGCCCTCCCAGAGAAGGC
AGTGAGGTTCTCCTTCACCGTGATGTCGGTGTCTGTCAGGCCTGAGGGGG
GTGAA---------------------------------GGGGAAGTGACTCTC
TTCTGCGAACCCAAGCCAAACTCAGAGCTCAGCTGCAAGCCGCTGTGCCT
GATGTTTGTGGATGAGTCTGACCACGAGACGCTGACTGCCATTTTGGGGC
CTGTAGTGGCTGAACGCAACGCCATGAAAGAAAGCAGGCTCATTCTGTCT
GTCGGTGGCCTGCTGCGATCTTTCCGCTTCCACTTCCGGGGCACGGGGTA
```

CGATGAGAAGATGGTGCGAGAGATGGAGGGCCTGGAGGCCTCAGGGTCGA
CCTACATCTGCACCCTGTGCGACTCCACCCGCACCCAGGCCTCCCAGAAC
ATGGTGCTGCACTCGGTCACCCGGAGCCATGAGGAGAACGGTGAGCGCTA
CGAGGTCTGGCGCACCAACCCATATTCAGAATCTGCCGACGAACTCCGTG
ACCGTGTCAAAGGTGTCTCCGCCAAGCCCTTCATGGAGACCTTACCCACT
ATCGACGCCCTCCACTGCGACATCGGCAACGCTGCCGAGTTCTACAAGCT
GTTCCAGGATGAAATTGGAGAGGTCCACGCCGGCGA---AGCC---CCTA
GCCGCGAGGAGCGAAGACGATGGAGGTCTGCTCTTGACAAGCAGCTGAGG
AAGAAGCTAAAGCTGAAGCCAGTGATGAGGATGAACGG------------

--------------------------NNNNNNNNNNNNNNNNNNNCTGGCGCAGGGACCGGGTCTGGCA
CAGACC---GCAGCGTCCCACTTAGCAACAGCCTGCTAGCCCCACAACAA
GCCGAGGAGGTTGCAGCGG---CTTCCCCGCCGCGATGGTTCGTCACCT-
--CCGCCAACAACCGATTGGACTTTGCCGCCTCGGCTTACGACGCTGCC-

--GCCGCTGATTTTGCTGGAAACGCGGCCACCTTGCTGTCATACGCAGCG
GCGGGAGTGAAGGCAT------TACCGTTGCCTACAGCAGGGTGCTCGAG
CAGACCTCTGGGTTACTATGCAGACTCTTCGG---GATGG---AGCACAC
GGACTCCTTCCCAGTATTGC---------AGCAAACCTAGCTCTGTTCTT
TCCTGCTGGCCCTCCAATTCTTCTGGGAACAGGACTGGCA---CCTCTAC
CTATCCTG---------CGGATGA---GAGT---GATAC---TATCCCAA
ATGAGAGGTCACCC---CT---CGGTGGGTCTGAGGAT---GTAAAACCT
AAAGACTT------GTCGGA---ATCCAACTGGATAGAA---ACGCCATC
GTCCATAAAATCGATTGATTCAAGCGATTCTGGGATTTTTG---AGCAGG
CCAAACGGAGACGTATGTCTCCCTCCGCAACGCCG---------GTTTCT
GAAACTACCTCCCCATCTAAGAGCGAA---------ACCGGAGAAGTCAC
AGACAGAGAAGTGGCTTTGGGGATAAATCCCTTTGCTGATGGGATGGGCG
CTTTTAAAATCAACCACAGCACCCACGACCTGGGCTCTGG---ACAAACG
GCATTTTCTTCGCAAGCA---CCAGGCTAC------GCCGCTGCCCTGGG
A---CACCATCA------CCACCCCACTCATGTCAGCTCC---TATTCCA
CCGCGGCTTTCAATTCCACTCGGGACTTTCTGTTTCGAAACCGGGGCTTT
GGAGACGCGACGAG------------TGCACAGCACAGCCTGTTCGCCTC
CGC---AGCAGGAAGTTT---T------GCAGGGCCACATGGACACTCGG
ATGCCACCGGGCACCTGCTTTTTCCGGGACTTCATGAG---CAAGCGGCT
ACTCACGCGTCTTCCAATGTTGTAAATAGTCAGATGCGTCTGGGCTTTTC
GGGGGACATGTACGGTCGAGCCGAGCAGTATGGCCATGTAACCAGCCCCA
GGT---CTGATCATTACGCTTCGACCCAGTTGCACGGATATGGCCCTATG
AACATGAACATGGCTGCA---CACCATGGGGCTGGGGCCTTCTTTCGGTA
CATGAGGCAACCGATCAAGCAGGAACTTATCTGCAAATGGATCGAACCGG
AACAGTTGACAAACCCGAAAAAGTCCTGCAACAAAACTTTCAGTACCATG
CACGAGCTCGTTACACACCTCACAGTGGAACATGTCGGAGGACCAGAACA
ATCGAATCATATCTGCTTTTGGGAAGAGTGTCCGAGGGAAGGGAAACCTT
TCAAAGCAAAGTACAAACTTGTAAATCACATTAGAGTACACACCGGCGAA
AAACCCTTCCCATGTCCGTTTCCCGGCTGTGGGAAA
>Aldrovandia affinis
AGCCTTTTAATTCGGGCAGAACTAAGCCAACCCGGAGCCCTTTTAGGAGA
TGACCAAATTTATAATGTTATCGTTACAGCACACGCTTTCGTAATGATCT
TCTTTATGGTTATACCCGTAATAATTGGAGGCTTCGGAAACTGACTCGTT
CCCCTAATGATCGGCGCCCCCGACATAGCATTCCCGCGAATAAACAACAT
AAGCTTCTGACTCCTACCCCCGTCATTTCTCCTTCTACTCTCTTCCTCTG
GGGTTGAGGCAGGGGCAGGAACAGGCTGAACAGTATACCCCCCACTTGCT
AGCAACCTAGCACATGCCGGGGCATCCGTCGACCTAACCATCTTCTCCCT
ACACCTTGCAGGTATCTCATCAATTCTTGGGGCTATTAACTTCATTACCA
CAATCATTAACATGAAACCCCCAGCAATCTCACAATATCAAACACCCCTA
TTTGTATGATCAGTACTTGTCACTGCAGTCTTGCTACTCCTATCTCTACC
AGTCCTAGCCGCAGGCATTACCATGCTACTTACGGATCGAAATCTTAATA
CAACATTCTTTGACCCGGCAGGAGGGGGAGACCCAATTCTGTACCAACAT
CTGTTCTGATTCTTCGGCCACCCCGAGGTGTATATCCTAATCCTCCCTGG
GTTCGGAATAATCTCGCACATCGTCGCATATTATGCCGGCAAAAAAGAAC
CGTTTGGCTACATAGGAATAGTGTGAGCTATAATGGCCATCGGCCTTCTA
GGGTTCATTGTCTGAGCACACCACATATTTACAGTAGGCATAGATGTAGA
CACACGAGNNNN--


```
--------------------------------------------------
--------------------------------------------------
--------------------------------------------------
--------------------------------------------------
--------------------------------------------------
--------------------------------------------------
--------------------------------------------------
--------------------------------------------------
--------------------------------------------------
--------------------------------------------------
--------------------------------------------------
--------------------------------------------------
--------------------------------------------------
--------------------------------------------------
--------------------------------------------------
--------------------------------------------------
--------------------------------------------------
--------------------------------------------------
--------------------------------------------------
--------------------------------------------------
--------------------------------------------------
--------------------------------------------------
--------------------------------------------------
--------------------------------------------------
-------AAGAAGGATACCAGCAAGGGCACACTCGAGGATCAAATCATCC
AGGCTAACCCAGCGCTGGAAGCTTTTGGCAATGCTAAGACTGCGAGGAAC
GACAACTCCTCGCGTTTTGGGAAATTCATCCGCATTCATTTTGGAGTAAG
TGGCAAGCTTTCGTCCGCTGACATCGAAACTTACCTGCTTGAGAAATCTC
GCGTCACCTTTCAGCTCAAAGCCGAGAGGAACTATCACATCTTTTACCAG
ATTATGTCCAACAAAAAGCCTGAATTGYTGGACATGATGTTGATCACTAA
CAACCCATACGATTATGCTTACGTCTCCCAAGGAGAGGTGACAGTCGCGT
CAATTGATGACTCTGATGAACTAATTGCCACAGACAGTGCTTTTGATGTG
CTGGGATTCACAGCTGAGGAAAAGATGGGTGTCTACAAGCTGACAGGTGC
CATCATGCATTACGGAAATATGAAATTCAAGCAGAGGCAGCGTGAGGAGC
AGGCTGAGCCTGATGGCACTGAGTCTGCTGACAAGTCAGCCTACCTGATG
GGGTTGAACTCAGCCGACCTTCTCAAAGGACTATGCCACCCAAGGGTTAA
AGTCGGCAATGAGTATGTCACAAAGGGCCAAGGTGTTGATCAAGTCTACT
ATACCCAACAAGAGGCCTTCAAGTGCGAGGAGTGTGGCAAGAACTACAAC
ACCAAGTTAGGCTACAAGCGTCACGTGGCCATGCACGCGGCCACCAGCGG
CGACCTCACCTGCAAGGTGTGCCTGCAGAGCTATGAGAGCACGCCCGTGC
TGCTGGAGCACCTCAAGAGTCACTCGGGGAAGTCCTCGGGTGGGGCCAAG
GAGAAGAAGCACCCCTGTGACCACTGCGACCGGCGCTTCTACACGCGCAA
GGACGTGCGGCGCCACATGGTGGTGCACACAGGCCGCAAGGACTTCCTGT
GCCAGTACTGCGCCCAGCGCTTTGGCCGCAAGGACCACCTGACCCGGCAT
GTGAAGAAGAGCCACTCACAGGAGCTGCTGAAGATCAAGACGGAGCCACC
GGACATGTTGGGCCTGCTGGGCTCCGGCTCCCCGCCTTGCTCCGTCAAGG
AGGAGCTCAGCCCCATGATGTG---TATGGGGCCCTCCAAGGACTCCTTG
ATGGGCAAGCCCTTCCCTGGTG------CCTTCCCCATGGCATGTACAA
CCCCCAC---------CTGCAGGCTATGTCCAACTCTGGGGTATCC----
--------CACCACTCACTGGTGTCCGGGTCTCTGTCTGCAGCTATGGGG
ATGGGCTGCCACATGGAGTACCTTATTTATGCATCGTTTTCCTTCATGGG
ATGTTTACAAATCAGCGACGGATCGAATATAGTCAACCTTTTAGCCAGCA
ACTCCCCGAGTGTCTCGTATGCTCTGACCCAGCAGAAATATTTCAGCAAT
```

```
TACAGTCCTGTGATTGGGTTCTACATCTATGAACCGATAGAGTACTGGAA
TTCCACAGTTCAAGAACACCTGAAGACGCTGGGCCACGGATTCAATAAGA
TTTCGTGGATCGACAACTTCTTTCAGTATCTGAAGGTAGTGAATATCAGT
GCGTCAACCAAAAGTGAATTCATAACCATCCTCCAGACTTCCTTCTTAAG
GAGCCCAGACTACCAGCACTTCAAGGATGACATCATTTTTTACAAAA---
TGAGGG------------ATGAAACTGAAATCATTGCGTCCCGCATGTAC
CTGGTGGCCAGAACCACGGAGAAGACCAGGGAGGAGGTGGTGGAATTACT
GGAGAGACTGAGACCTCTGTCTCTCATCAACAGCATCAAATTCATTGTCT
TCAACCCCACCTTTGTGTTCATGGATCGATATAGCTCCTCAGTCATTTCT
CCGATCCTCACCTCTGGATTCAGTGTCCTGATTGTCTTGATCCTGACGTT
CTTTCTGGTCATTAACCCCCTCGGAAACTTCTGGTTGATACTGACGGTCA
CCTCAGTGGAGCTGGGAGTTCTTGGCCNNNNN-----------------------
--------------------------------------------------
--------------------------------------------------
--------------------------------------------------
--------------------------------------------------
--------------------------------------------------
--------------------------------------------------
--------------------------------------------------
--------------------------------------------------
--------------------------------------------------
--------------------------------------------------
--------------------------------------------------
--------------------------------------------------
--------------------------------------------------
--------------------------------------------------
--------------------------------------------------
--------------------------------------------------
--------------------------------------------------
--------------------------------------------------
--------------------------------------------------
--------------------------------------------------
--------------------------------------------------
--------------------------------------------------
--------------------------------------------------
--------------------------------------------------
--------------------------------------------------
--------------------------------------------------
--------------------------------------------------
----------------------------------TCATACACCATCGAGA
TGGGCCCCTGGGGGCCCCAGTGGAAGGAGAGCCCCCAGCCCTTCTCCTGT
TCCATCGAGGATCCCACCAAACAGACCAAGTTCAAGGGCATAAAGACGTA
CATCTCGTACCGCGTGACGCCCAGCCACACGTCTCAGCCCGTGTATCGTC
GCTACAAGCACTTTGACTGGCTTTACAACCGGCTCCTGCACAAGTTCACA
GTCATCTCTGTGCCACACCTGCCTGAGAAGCAGGCCACGGGCCGCTTCGA
GGAAGACTTCATCGAGAAGCGCAAGAGGCGGCTCATCCTGTGGATGGATC
ACATGACCAGCCACCCGGTGCTGTCGCAGTACGAGGGCTTCGAGCACTTC
CTCATGTGTGCCGACGACAAGCAGTGGAAGCTGGGCAAGCGGCGGGCGGA
GAAGGACGAGATGGTGGGTGCGCACTTCATGCTCACCTTCCAGATCCCCA
GCGAGCACCAGGACCTGCAGGACGTGGAGGAGCGCGTGGACTCCTTCAAG
GCCTTCGCCAAGAAGATGGACGACAGCGTGCTGCAGCTCACACACGTGGC
GTCCGAGCTGGTTCGGAAGCACCTGGGCGGCTTCCGGAGGGAGTTCCAGC
GGCTGGGGAACGCCTTCCAGTCCATCAGCCAAGCCTTCACCCTGGACCCT
```

CCCTACATGTCCGACGCCTTCAACAANNNNNNNNNNNNN------------------------
----

-------------GCCAAATCTCGCTTCCACCCTGGCGCTGGAACCGGGT
CTGGCACAGACC---GCAGCGTCCCACTTGCCAACGGCTTGCTATCCCC-
--ACAAGCTGAGGAGGTTGCAGCGG---CTTCCCCTTCACGATGGTTTGT
CGCCT---CTGCCAACAATCGACTGGACTTTGCCGCCTCGGCGTACGATG
CGGCC---GCTGCTGATTTCGCCGGTAACGCTGCCACCTTGCTGTCTTAC
GCAGCTGCTGGAGTCAAGGCAC------TTCCACTGCCTCCTGCGGGGTG
CTCGGGTCGAGCCCTCGGTTATTACGCCGACTCTTCTG---GGTGG---G
GAACGCGCACCCCTTCCCCGTATTGC---------AGTAAATCAGGCTCC
GTTCTTTCCTGCTGGCCCTCCAATTCTTGTGGAAGCAGGACTGCCA---C
CTCGAACTATGTGG---------TGGATGA---GGGG---GACAC---CA
TCTTAACGGAGAGGTCACCA---CT---CGGTGGGTCCGAGGAT---ACC
AAACCAAAAGATCT------GTCGGA---ATCTAGTTGGATAGAG---AC
GCCTTCCTCAATAAAATCAATTGATTCCAGCGATTCTGGGATTTTTG---
AGCAGGCCAAACGGAGACGAATATCACCTTCCGCCACACCG---------
GTTTCGGAAAGTACCTCCCCGTCGNNNNNNNNN---------ACCGGAGAAGTCACAGA
CAGAGACGTGGCTTTGGGGATAAATCCCTTCGCTGATGGGATGGGCGCTT
TTAAAATCAACCACAGCACCCACGACCTGGGCTCTGG---ACAAACGGCA
TTTTCCTCGCAAGCG---CCAGGCTAC------GCCGCTGCCCTGGGA--
-CACCATCA------CCACCCCACCCATGTCAGCTCC---TATTCCACCG
CGGCGTTCAATTCCACTCGGGACTTTCTGTTTCGAAATCGGGGCTTCGGC
GACGCGACGAG------------CGCACAGCACAGTCTGTTCGCCTCTGC
---CGCGGGAAGTTT---T------GCAGGACCACATGGACACTCGGATG
CCACTGGGCACCTGCTTTTTCCGGGACTTCACGAG---CAAGCGGCCACT
CACGCGTCTTCCAATGTTGTAAATAGTCAGATGCGCCTGGGCTTTTCGGG
TGACATGTACGGAAGAGCCGAGCAGTACGGCCATGTAACGAGCCCCAGGT
---CCGATCATTACGCGTCGACCCAGTTGCATGGATATGGCCCCATGAAC
ATGAACATGGCTGCA---CACCATGGGGCAGGGGCCTTCTTTCGGTACAT
GAGGCAACCGATAAAGCAGGAACTTATCTGCAAATGGATCGAACCGGAAC
AATTGACAAATCCGAAAAAGTCCTGCAACAAAACTTTCAGCACCATGCAC
GAGCTCGTTACACATCTCACAGTGGAACATGTCGGGGGACCAGAACAATC
GAATCATGTCTGCTTTTGGGAAGAGTGTCCGAGGGAAGGAAAACCTTTTA
AAGCAAAGTACAAACTTGTAAATCACATCAGAGTACACACCGGTGAAAAA
CCCTTCCCATGCCCGTTTCCCGGCTGTGGGAAA

>Alepisaurus ferox
AGTCTCCTTATCCGAGCCGAGCTAAGCCAACCCGGAGCCCTCCTGGGAGA
TGACCAAATCTATAACGTAATCGTAACCGCCCACGCCTTCGTAATAATCT
TCTTCATAGTAATACCCGTTATAATCGGCGGGTTCGGAAACTGACTTATC
CCCTTGATAATCGGGGCCCCTGACATAGCCTTTCCGCGAATGAACAACAT
GAGCTTCTGGCTACTTCCTCCATCCTTCCTCCTCCTCCTCTCCTCTTCAG
CTGTTGAAGCTGGGGCCGGAACAGGGTGAACAGTCTACCCTCCTCTAGCT
AGCAACTTAGCCCACGCCGGAGCCTCGGTCGACCTGACCATCTTCTCTCT
CCATCTGGCGGGTATTTCTTCTATCCTGGGGGCTATTAACTTCATCACAA
CAATTATTAACATGAAACCCCCGGCCATCACCCAATACCAAACTCCCCTG
TTTGTTTGAGCCGTTCTAATTACCGCAGTCCTTCTCCTACTCTCCCTCCC
CGTCCTAGCGGCAGGCATCACTATGCTCCTTACAGATCGAAACCTAAACA
CAACCTTCTTTGACCCAGCCGGCGGGGGGGATCCAATTCTTTACCAACAC
CTCTTCTGGTTCTTTGGCCACCCAGAAGTTTACATCTTAATTCTTCCCGG
CTTCGGAATGATCTCCCACATTGTTGCCTACTATTCAGGTAAAAAAGAAC
CCTTCGGCTACATGGGCATGGTCTGAGCAATGATAGCAATCGGACTTCTA
GGTTTCATTGTCTGAGCACCTCCCATGTTTACAGTCGGAATGGACGTAGA
CACACGTGCCTATTTTTAGAGAAAAATCTGCATCCCTCAAATTGTCTTGG
AATGTTCCTTCTCTCCGACGCTCATCAGTGCACGAAGCTCAACGAACTTT
CCTGGAGCATGTGTCTCAGTCATTTCCCTACGATATGCAAGACTGAGGAC
TTTTTGCAGTTGCCCAAAGACATGGCAACCCAGTTGCTATCCCACGAGGA
GCTGGAGACGGAGGATGAGAGGCTGGTCTATGAGGCGGCCCTCAGCTGGG
TTAACTATGACCTGGAGAGGAGGCACTGCCACCTGACAGAGCTTCTGAGA
ACAGTCCGGCTGGCCCTTCTACCGGCCATCTTCCTCATGGAGAATGTATC
CACAGAGGAGCTAATTAATTCCCAGACCAAGAGCAAGGAACTTGTAGACG
AGGCCATACGCTGCAAGCTGAGAATACTTCAGAACGATGGTGTGGTCAAC
AGCCCATTGGCAAGACCCAGAAAGACCAGCCATGCCTTGTTCCTGCTCGG
TGGCCAGACCTTCATGTGTGATAAGCTTTACCTGGTAGACCAGAAGGTCA
AGGAGATCATTCCCAAGGCTGACATACCCAGCCCCAGGAAGGAGTTCAGC
GCCTGCGCCATCGGCTGTAAGGTGTACATCACCGGTGGGA--GAGGCTC-
AGAGAACGGTGTGTCCAAAGATGTGTGGGTCTACGACACCGTCCACGAGG
AATGGTCCAAGGCGGCGCCCATGCTCATTGCCAGGTTTGGCCATGGCTCT
GCAGAGCTGAAACACTGCCTCTATNNNNNNNNNNNNNNNNNNNNNNNNNNNNNNNNNNNNNNNNNNNNNN
NNNNNGGATGAGTACATTGTGGTGTTCAGTC
GTTCCACAACTAGGCTGATTCTGAATGAAGCAGAGCTGATACTGGCACTG
GCCCAAGAGTTTCAGATGAGGGTGGTCACTGTGTCCCTGGAGGAACAGTC
TTTCCCCAGCATCGTACAGGTCATCAGTGGTGCCTCCATGTTGGTCAGTA
TGCACGGAGCTCAGCTCGTCACCTCACTCTTCCTCCCTAGAGGATCTGCT
GTTGTAGAGCTCTTCCCCTATGCCGTGAACCCAGAGCAGTACACTCCGTA
CAAAACCCTCGCCTCCCTACCGGGCATGGACCTTCAATACGTTTCCTGGA
GGAACACTGTTGAGGAGAACACCGTCTCCCACCCAGACAGACCCTGGGAG
CAAGGAGGCATTGCCCATTTGGAGAAAGAAGAGCAGGAGAGAATCCTAGC
CAGCACGGATGTCCCCAGGCACCTATGCTGCCGCAACCCAGAGTGGCTCT
TCAGGATCTACCAGGACACTCTGGTAGACATCCCCTCCTTCTTAGACGTC
CTCAA---GGAGGGCCTGAAG---ACGAGGCCAAGCTTGAAGAA---GTC
CAAGCCGGCCAGCACGGTTCACCCGGGCCGGGTGCGAGAACCCCAGTGCC
AGACTTCAGTCCAAGCCACCAACGAGGCTAAACTCACAGTGTCCTGGCAG
ATCCCGTGGAACCTTAAGTACCTGAAGGTGCGAGAGGTGAA---------
----------AAGAAGGACACAAGCAAGGGAACCCTGGAGGATCAAATCA
TTCAGGCAAACCCCGCACTGGAGGCTTTCGGTAATGCCAAAACATTGAGG
AACGATAATTCTTCCCGTTTTGGAAAATTCATCAGAATCCACTTTGGAAA
CAGTGGTAAGCTGTCTTCTGCGGACATTGAGACCTACCTGCTGGAGAAGT
CACGGGTCACCTTTCAGCTTAAGGCAGAGAGGAACTACCACATATTCTTC

CAGATCTTGTCCAATCAAAAGCCAGAGCTGCTGGACATGCTGTTGATCAC
CAACAACCCTTATGATTACTGCTACATCTCCCAAGGAGAAGTAACTGTAG
CGTCCATCAATGATTCAGAAGAGCTGATGGCCACTGACAGTGCCTTCGAT
GTGCTTGGCTTCACGCAAGAGGAGAAGCTGGGAGTCTACAAGTTGATAGG
GGCCATTATGCACTATGGCAACATGAGGTTCAAGCAGAAGCAGCGTGAGG
AACAGGCAGAGCCTGATGGTACAGAGGCTGCTGATAAGTCCGCTTATCTA
ATGGGGCTGAATTCAGCAGACCTAATCAAGGGGCTCTGCCATCCCAGAGT
CAAGGTAGGGAATGAGTACGTCACCAAAGGCCAGGGTGTAGATCAAGTCT
ACTACCCCAACAAGGAGGCCTTCAAGTGTGAAGAGTGTGGCAAGCACTAC
AACACCAAGCTGGGATACAAGCGCCATGTGGCCATGCACTCGGCCACGGC
GGGGGACCTCACGTGCAAGGTGTGCATGCAGAGCTACGAGAGCACCCCGG
TGCTCCTGGAGCACCTCAAGAGCCACTCGGGGAAGTCCTCGGGCGGCGCC
AAGGAGAAGAAGCACCCGTGCGACCACTGCGACCGCCGCTTCTACACGCG
GAAGGACGTCAGGCGGCACATGGTCGTCCACACGGGCCGGAAGGACTTCC
TGTGCCAGTACTGTGCCCAGCGCTTTGGCCGGAAGGACCACCTGACGCGG
CACGTGAAGAAGAGCCACTCGCAGGAGCTGCTGAAGATCAAGACGGAGCC
TCCGGACATGCTGGGCCTCCTGGGCTCCGGCTCGCCGCCTTGCTCCGTCA
AGGAGGAGCTGAGTCCCATGATGTGCAGCATGGGTCCCAATAAAGACCCC
ATGATGGGCAAACCCTTCCCCAGCGGGACCCCCTTCCCCATGGGCATGTA
CAACCCCCACCAC------CTCCAGGCCATGTCTAATCCCNNNNNNNNNNNNNNN------
NNNNNNNNNNNNNNNNNNNNNNNNNNNNNNNNNNNNNNNNNNNNNNNNNNNNNNNNNNNNNNTATC
TCATCTATGCCTCCTTCTCCTTCATGGGATGTTTACAAATCAGCGATGGG
TCAAACGTGGTGAATTTGCTGGCCAGTAACTCTCCGAGCGTCTCGTATGC
TCTGACACAGCAGAAGTACTTCAGTAACTATAGCCCTGTGATCGGGTTTT
ACATCTACGAGCCCATCGAGTACTGGAACTCCACGGTGCAGGAGCACCTG
AAGACACTCAGCCACGGCTTCAACAAGATCTCCTGGGTGGACAATTTTGC
TCACTACCTGCGGGCGGTGAACCTGAGCGCGTCTACCAAGGCCGACTTTG
TCGCCGTCCTCAAGGGCTCCTTCCTGCGGAGCCCAGTGTACCAGCACTTC
ACCGAGGACATCATCTTCTCCAAGA---------AGAACA------ACGA
GTATGACATCATCGCCTCGCGCATGTACCTGGTGGCGCGCACCACAGAGA
AGCGGCGCGAGGATGTCGTGGAGCTGCTGGAGAAGCTCCGGCCGCTGATG
CTGATCAACAGCATCAAGTTCATTGCCTTCAATCCCACCTTCGTCTTCAT
GGACCGCTACAGCTCCTCGGTCGTCTCGCCCATCCTCACCTCGGGCTTCA
GCGTGCTCGCCATCCTCATCCTCACCTTCTTCCTCGTCATCAACCCGCTG
GGAAACCTGTGGCTCATCCTGACAGTCACATCCGTGGAGCTGGGCGTACT
GGGCCTGATGNNNNNNNNNNNNNNNNNNNNNNNNNNNNNNNNNNNNNNNNNNNNNNNNNNNNNNNN
NNNATCATCAATGGGCTTTCCGGGTGGGCAGCCTCGGTGGATG
ACGTCCCCGCTGACATTATCACCCGTCGGTTTCGCTATGACGTGGCGCTT
GTATCGGCCCTGAAGGACTTAGAGGAGGACATCATGGCAGGACTGAGGGT
GCATGGGCTGGAAGACAGTGCTTGTACCTCAGGCTTCAGTGTTATGATCA
AGGAGTCCTGTGATGGCATGGGAGATGTCAGCGAGAAGCATGGCGGGGGG
CCACCGGTGCCTGAGAAGGCTGTGCGTTTCTCTTTCACCGTCATGTCTGT
CTCTGTCCAGGCCGACGGAGAGGAA-------------------------
-----GAGGCGGTTGTTGTCTTCAGGGAGCCAAAACCCAACTCTGAACTG
TCCTGTAAGCCCATGTGTCTGATGTTTGTGGATGAGTCTGACCATGAGAC
ACTCACTGCTGTCCTTGGGCCTTTGGTAGCAGAGAGGAATGCAATGAAGC
AAAGCCAACTCATCCTCTCAATGGGAGACCTCCCCCGATCCTTCCGCTTC
CACTTCAGAGGCACGGGCTACGATGAGAAGATGGTGCGAGAGATGGAAGG
CCTGGAGGCCTCAGGCTCCACCTATATCTGCACTCTTTGTGACTCCACTC
GGGCAGAGGCCTCTCACAACATGGTGCTCCACTCTATCACGCGCACCCAC
GACGAGAACCTGGAGCGCTACGAGATATGGAGGACCAACCCCTTCTCGGA
GTCTGCCATGGAGCTGCGAGATCGGGTCAAAGGCGTCTCCGCCAAACCCT
TTATCGAGACTCATCCGACTATGGATGCATTACACTGTGACATAGGTAAC

```
GCCACTGAATTCTACAAAATCTTCCAGGATGAGATCGGGGAGGTGTTCCA
GAAGGC---CAAC---CCCAGCAGGGAGGAGCGGCGCAGCTGGAGGGCAG
CCCTCGATAAACAGCTGAGGAAGAAGTTGAAGCTGAAACCGGTGATGCGG
ATGAATGGGAACTTTGCCCGAAGGCTGATGACTGAGGAGGCTGTAGAGGT
GGTGTGTGAATTGGTGCCCTCGGACGAGAGACGGCAGGCCCTCAGGGAGC
TGATGGGGCTCTATATCCAGATGAAGCCTGTATGGCGCGCCACCTGCCCA
GCCAAGGAGTGCCCTGATCAGCTGTGCCGCTACAGCTTTAACTCTCAGCG
CTTTGCTGATCTCCTCTCATCCACCTTCAAATACAGGTATGATGGGAAAA
TCACCAACTACCTGCACAAGACTCTGGCCCACGTCCCTGAAATCATAGAG
AAGGATGGCTCCATCGGAGCCTGGGCTCATCTGAGCTGCAGTCAGTACCA
TAAG----------------------------------------------
--------------------------------------------------
--------------------------------------------------
--------------------------------------------------
--------------------------------------------------
--------------------------------------------------
--------------------------------------------------
--------------------------------------------------
--------------------------------------------------
--------------------------------------------------
--------------------------------------------------
--------------------------------------------------
--------------------------------------------------
--------------------------------------------------
--------------------------------------------------
--------------------------------------------------
--------------------------------------------------
--------------------------------------------------
--------------------------------------------------
--------------------------------------------------
--------------------------------------------------
--------------------------------------------------
--------------------------------------------------
--------------------------------------------------
--------------------------------------------------
--------------------------------------------------
--------------------------------------------------
--------------------------------------------------
--------------------------------------------------
--------------------------------------------------
--------------------------------------------------
--------------------------------------------------
--------------------------------------------------
--------------------------------------------------
--------------------------------------------------
--------------------------------------------------
--------------------------------------------------
--------------------------------------------------
--------------------------------------------------
--------------------------------------------------
--------------------------------------------------
--------------------------------------------------
--------------------------------------------------
```


-------------------------ACAGGCGAAGTCACAGAAAGAGAAG
TGGCTTTGGGGATAAACCCGTTCGCGGACGGCATGGGCGCCTTCAAAATA
AACCACAGCTCCCACGACATCGGCTCCGG---GCAAACGGCGTTTTCCTC
CCAGGCG---CCCGGCTAC---GCGGCGGCCGCCCTGGGA---CACCACC
A------CCATCCGACCCACGTCAGCTCC---TATTCCACGGCAGCCTTC
AATTCCACGCGGGACTTTCTCTTCAGAAACCGGGGCTTCGGGGACGCCAC
CAG------------CGCGCAGCACAGTCTGTTCGCCTCCGC---CGCCG
GAAGTTT---C------GCAGGGCCACACGGACACTCAGATGCCGCGGGC
CACCTGCTCTTCCCCGGACTCCACGAG---CAAGCTGCGAGCCACGCGTC
GTCTAATGTCGTGAACAGCCAGATGAGACTGGGCTTTTCCGGAGACATGT
ACGGGCGGGCCGACCAGTATGGCCACGTTACGAGCCCGAGAT---CCGAC
CACTACGCTTCGACCCAACTGCACGGCTATGGCCCCATGAACATGAATAT
GGCCGCT---CACCACGGCGCGGGGGCCTTCTTTCGGTACATGCGGCAGC
CGATCAAACAAGAGCTGATCTGCAAGTGGATCGAACCGGAGCAACTGTCG
AACCCCAAAAAGTCGTGCAACAAAACTTTCAGCACGATGCACGAGCTCGT
GACCCACCTGACGGTGGAGCATGTGGGAGGACCCGAGCAGTCGAACCACA
TTTGCTTCTGGGAAGATTGCGCCAGGGAAGGGAAACCGTTCAAAGCCAAA
TACAAACTCGTAAATCATATCAGAGTTCACACCGGAGAAAAACCCTTCCC
GTGTCCGTTCCCCGGCT--------
>Alepocephalus agassizii
AGCCTCTTAATTCGAGCCGAGCTTAGCCAGCCCGGCGCCCTCCTCGGCGA
TGACCAAATTTATAATGTCATCGTCACAGCACATGCCTTCGTAATGATTT
TCTTTATAGTAATACCAATCATGATCGGAGGCTTCGGAAACTGGCTACTC
CCCCTAATACTAGGGGCCCCTGACATAGCATTCCCCCGAATGAACAACAT
GAGTTTCTGACTTCTACCCCCCTCACTCCTCCTCCTCCTTTCATCCTCAG
GGGTTGAAGCTGGGGTGGGGACAGGGTGGACCGTCTATCCCCCCTTGGCC
GGCAACTTAGCCCACGCCGGTGCCTCTGTAGACCTCGCCATCTTCTCTTT
ACACCTGGCAGGTGTTTCCTCAATTCTGGGATCAATTAATTTTATTACCA
CAATTACCAACATGAAGCCCCCAGCCATCTCCCAATACCAGACACCCCTG
TTCGTGTGATCCCTTCTCGTCACAACTGTCCTACTTCTCCTGTCTCTCCC
AGTTCTAGCCGCCGCTATCACCATGCTCCTCACAGACCGTAATCTTAACA
CAACATTCTTTGACCCAGCCGGAGGAGGAGACCCCATCCTATATCAACAC
CTATTCTGATTCTTCGGCCACCCAGAGGTTTACATTCTAATCCTGCCCGG
ATTTGGCATCGTCTCACACGTCGTCGCCTACTACGCCGGTAAAAAAGAAC
CATTCGGTTATATGGGCATAGTTTGGGCTATAATAGCTATCGGACTCCTA
GGATTTATTGTTTGAGCCCACCACATGTTTACAGTGGGAATGGACGTAGA
CACTCGTGCCTA--------------------------------------


```
--------------------------------------------------
--------------------------------------------------
--------------------------------------------------
--------------------------------------------------
-------------------------GGATGAGTACATCGTGGTCTTCAGC
CGCTCCGTCAACCGGCTCATCCTCAACGAGGCTGAGTTGATCCTGGCTCT
GGCGCAAGAGTTCCAGATGAGGGCCATCACAGTGTCCCTGGAGGAGCAGT
CGTTTGCCAGCATCGTTCAGTTGATCAGTGGGGCGTCCATGCTGGTCAGC
ATCCACGGAGCCCAGCTGATCAGCTCCCTGTTCCTGCCACGCGGCGCGGC
CGTGGTGGAGCTCTTCCCCTACGCCATCAACCCAGAGCAGTACACGCCCT
ACAAAACTCTGGCTTTGTTGCCAGGCATGGACCTCCAGTATGTGGCTTGG
AGGAACACCATGGAGCAGAACTCGGTGGCTTACCCTGAGCGTGCCTGGGA
CCAGGGTGGCATCGCCCACCTGGAGATGGAGGAACAGGAGCGCATCCTGG
CGAGCGATGAGGTGCCGCGGCACCTATGCTGCCGCAACCCAGAGTGGCTC
TTTCGGATTTACCAGGACACCCAGGTGGACGTCCCCTCCTTGCTGGAGGT
CCTCAG---AGAAAATCTGAAG---GCCAAGCCTAACCTGAGAAA---GG
CCAAGGCAGCTAGTACAGTACACCCCGGTAGAGTCAGGGAGCCCAAGTGC
CAGACTTCGGTCCAGGCCACCAACGAGGCCAAGCTGACAGTGTCGTGGCA
GATCCCCTGGAACCTCAAATACCTGAAGGTCAGGGAGGTCAAATATGAAG
TATGGATACAGAAGAAGGATGCCAGCAAAGGGACCTTGGAGGATCAAATC
ATTCAAGCCAACCCAGCACTGGAGGCTTTTGGCAACGCCAAAACCTCAAG
AAACGACAACTCCTCACGCTTTGGGAAGTTCATTCGTATTCATTTTGGAA
CGAGCGGCAAACTCTCCTCTGCTGACATAGAAACTTACTTGCTTGAGAAA
TCCCGCGTGACCTTTCAGCTCAAATCGGAGAGGAATTACCACGTCTTCTA
CCAGATATTGTCCAATGAAAAGCCAGAGCTGCTGGACATGCTGTTGATTA
CCAACAACCCTTATGATTATTGCTACATCTCCCAAGGAGAAGTAACAGTT
AAGTCCATCAATGACAATGAAGAGTTGCTTGCCACTGACAGTGCCTTTGA
TGTGCTTGGCTTCACTCCAGAGGAGAAGATGGGGATCTATAAGTTGACAG
GTGCCATTATGCACTATGGCAACATGAAGTTCAAGCAGAAGCAGCGTGAG
GAGCAGGCAGAGCCTGATGGCACTGAGGCAGCTGACAAGTCAGCCTACCT
GATGGGGCTTAACTCTGCTGATCTTGTGAAGGGACTCTGCCATCCCAGGG
TCAAAGTTGGCAATGAGTATGTAACAAAGGGACAAGGTGTAGATCAAGTC
TATTAC--------------------------------------------
--------------------------------------------------
--------------------------------------------------
--------------------------------------------------
--------------------------------------------------
--------------------------------------------------
--------------------------------------------------
--------------------------------------------------
--------------------------------------------------
--------------------------------------------------
--------------------------------------------------
--------------------------------------------------
--------------------------------------------------
----------------------TACCTGATCTACGCTTCCTTCTCGTTCA
TGGGATGTTTACAAATCACAGACGGATCCAACATAGTCAACCTTTTGGCC
AGCGACTCGCCAAGCGTGTCGTACGCTCTGACCCAGCGGAAGTATTTCAG
CAACTACAGCCCAGTGATAGGGTTCTACATCTACGAGCCCATTGACTACT
GGAACTCCACGGTGCAGGAGCACCTCAGGACACTGGGCCAGGGATTCAAT
ACCATATCGTGGATCGATAATTACTTTCAGTATCTGAAGGTGACGAACGT
CAGCGCGTCGACCAAAAGCGACTTCATCGCCGTCCTCAAGACTTCGTTCC
TGAGGAGTCCCGAGTATCAGCACTTCACGGACGACATCATCTTTACCAAA
```

```
A---CGGGCG------------ACGACTTCAACATCATCGCGTCCAGGTT
GTACTTGGTGGCGCGGACCACGGAGAAGACGCGGGAGGAGGTGGTGGAGC
TGCTGGAGAGGCTCCGCCCGCTCTCGCTCATCAACAGCATCAAGTTCATC
GTGTTCAACCCCACCTTCGTGTTCACGGACCGCTACAGCTCCTCGGTCGT
CTCCCCCATCATGACGTCCGGATTCAGCGTCCTGACCGTGCTCGTGCTCA
CGTTCTTCCTCGTCGTCAACCCACTGGGCAACTTCTGGCTGATACTGACC
GTCACCTCCGTGGAGCTGGGCGTCCTGGGCCTGATG--------------
--------------------------------------------------
--------------------------------------------------
--------------------------------------------------
--------------------------------------------------
--------------------------------------------------
--------------------------------------------------
--------------------------------------------------
--------------------------------------------------
--------------------------------------------------
--------------------------------------------------
--------------------------------------------------
--------------------------------------------------
--------------------------------------------------
--------------------------------------------------
--------------------------------------------------
--------------------------------------------------
--------------------------------------------------
--------------------------------------------------
--------------------------------------------------
--------------------------------------------------
--------------------------------------------------
--------------------------------------------------
--------------------------------------------------
--------------------------------------------------
--------------------------------------------------
--------------------------------------------------
--------------------------------------------------
-------------------------------------------TCCTACA
CCATCGAGATGGGCGCAAGAGGGCCTCAGTGGAAGGAGAGCCCCCAGCCT
TTCGCTTGCTCCATTGAGGACCCCACTAAGCAAACCAAGTTCAAGGGGAT
TAAGACGTATATATCCTACCGCGTGACCCCCAGCCACGTTGGCCGGCCTG
TGTACCGTCGCTACAAGCACTTTGACTGGCTGTATAACAGGTTGCTGCAC
AAGTTCACGGTCATCTCTGTGCCCCACCTGCCTGAGAAGCAGGCGACTGG
GCGTTTTGAGGAGGACTTCATTGAAAAGCGTAAGAGGCGGCTCATCCTCT
GGATGGACCACATGACCAGTCACCCTGTCCTCTCGCAGTACGAGGGCTTT
GAACACTTCTTCATGTGCGGTGACGATAAACAGTGGAAGCTGGGCAAGCG
GAGGGCGGAGAAGGATGAGATGGTGGGAGCCCACTTCATGCTCACCTTCC
AGATCCCCAACGAGCACCAGGACCTGCAGGACGTGGAGGAGCGGGTGGAC
TCCTTTAAGGCCTTTGCCAAGAAGATGGACGACAGYGTCATGCAGTTAAC
GCACGTGGCCTCCGAGCTGGTGCGCAAGCACCTGGGAGGGTTCCGCAAAG
AGTTCCAAAGGCTGGGGAACTCCTTCCAGTCCATCAGCCAGGCTTTCATG
CTGGACCCCCCCTACAGCTCTGATGCCCTGAATAATGCCATCTCCCAC--
--------------------------------------------------
--------------------------------------------------
--------------------------------------------------
--------------------------------------------------
```


-----------------------------------GCCAAATCCCGCTTT
CACCATGGCGTCGGCACGGGGCCTGGCACGGACC---GCAGCGTCCCACT
C---AACAGCTTGCTATCCCCGCAACAAACCGATGAGACCGCAGTGG---
CCTCCCCGCAGCGATGGTTTGTCACCC---CTGCCAACAACCGACTGGAC
TTTGCCGCCTCGGCATACGATGCCGCCGCTGCAGCTGATTTTGCCGGTAA
CGCGGCCACCCTTCTGTCGTACGCAGCTGCCGGAGTGAAAGCGC------
TCCCACTGCCCACTGCAGGCTGCTCCAACAGAGCCTTGGGCTATTACGCG
GAGCCGCCAG---GGTGG---GGCACACGCACCCCACCGCAGTACTGT--
-------AGTAAATCAAGCGCGGTTCTCTCATGCTGGCCCGCCAATTCCG
TTGGGAGCAGAACATCCA---CCTCCAATTATCTGG---TTGGCTTGGAC
GA---CGGG---GACGC---TATCGCACCTGAGAGGTCACCT---CT---
CGGGGGGGCAGACGAA---GCCAAGCCAAAAGACCT------GTCGGA--
-ATCAAGCTGGATAGAG---ACCCCGTCTTCAATTAAGTCAATCGACTCA
AGTGATTCTGGGATCTTTG---AGCAGGCCAAACGGAGGAGAATTTCGCC
ATCTGCTACACCG---------GTTTCAGAGACGTCGTCCCCATTAAAAT
CAGAA--TGGGG
ATAAATCCGTTCGCCGACGGTATGGGAGCGTTCAAAATCAACCACAGCTC
CCACGATTTTGGCTCTGG---GCAAACGGCGTTTGCCTCGCAGGCG---C
CCGGCTAC---GCAGCCGCTGCCCTGGGA---CACCATCA------CCAC
CCAACCCATGTCAGCTCC---TACTCCACCGCGGCGTTCAACTCCACCCG
GGACTTTCTCTTTCGGAATCGGGGATTCGGAGACGCCACTAG--------
----CGCGCAGCACAGTCTCTTCGCCTCAGC---TGCAGGAAGTTT---C
------GCAGGGCCACATGGACACACCGATGCCACGGGACACCTGCTCTT
CTCGGGACTGCACGAG---CAAGCGGCGACGCACGCGTCTTCGAACGCGG
TGAACAGTCAGATGCGCCTGGGCTTTTCGGGGGACATGTACGGCAGAGCC
GAGCAGTACGGTCACGTAACGAGCCCCCGGT---CCGAGCACTACGCTTC
GACTCAGTTGCACGGCTATGGCCCCATGAACATGAATATGGCTGCC---C
ACCACGGGGCAGGGGCCTTCTTCCGTTACATGAGGCAGCCGATCAAACAA
GAGCTCATCTGCAAGTGGGTTGAACCAGAGCAGCTGTCGAATCCGAAAAA
GTCCTGCAACAAAACTTTCAGCACGATGCATGAGCTCGTGACCCACCTCA
CAGTGGAACATGTCGGGGGACCGGAACAGTCGAATCACATTTGCTTTTGG
GAAGAGTGTCCGCGAGAAGGGAAACCATTTAAAGCCAAGTACAAACTTGT
AAATCATATCAGAGTGCAC-------------------------------

>Alepocephalus bicolor
AGCCTCTTAATCCGAGCCGAGCTTAGCCAGCCCGGCGCCCTCCTGGGCGA
TGACCAAATTTATAATGTCATCGTCACAGCACATGCCTTCGTAATGATTT
TCTTTATAGTAATACCAATCATGATCGGGGGCTTTGGAAACTGGCTACTC

```
CCCCTAATACTAGGAGCCCCTGACATAGCATTCCCTCGAATAAATAACAT
GAGTTTCTGGCTCCTTCCACCTTCACTACTCCTCCTCCTTTCATCCTCAG
GGGTTGAAGCCGGAGTAGGTACAGGATGAACCGTCTACCCCCCTTTAGCC
GGCAACCTAGCCCACGCCGGTGCTTCTGTAGACCTCGCCATCTTCTCTCT
ACACCTGGCAGGTGTCTCCTCAATTCTCGGCTCAATCAATTTTATTACTA
CTATTATCAACATGAAACCCCCAGCCATCTCCCAATACCAAACACCCTTG
TTCGTATGGTCCGTCCTCGTCACAACTGTCCTACTTCTCCTCTCTCTCCC
AGTCCTAGCTGCAGCCATTACCATGCTCCTTACAGACCGCAATCTTAACA
CAACATTCTTTGACCCGGCAGGAGGAGGAGACCCCATCTTGTATCAACAC
CTATTCTGATTCTTTGGCCACCCAGAAGTTTACATTTTAATCCTACCCGG
GTTTGGTATCATCTCACACGTCGTCGCCTACTATGCCGGCAAAAAAGAGC
CATTCGGTTACATGGGTATGGTCTGAGCTATAATAGCCATCGGACTCCTA
GGGTTTATTGTTTGAGCCCACCACATGTTTACAGTGGGAATGGATGTAGA
CACCCGTGCCTATTCCTGGAGAGAAA-CTTCACCCGTCCAACTGCCTCGG
CATGCTGCTGCTGTCTGATGCCCATCAGTGCACCAAGCTCTCTGAGCTCT
CCTGGGGCATGTGCCTCAGCAACTTTCCCGCTATTTGCAAGACTGAGGAA
TTCCTCCAACTGCCCAAAGACATCGTGTTGCAACTGCTGTCTCACGAGGA
GCTGGAAACTGAAGATGAGAGGCTGGTCTATGAAGCTGCCCTCAACTGGA
TCAACTATGACTTGGAAAAACGGCACTGTTACCTGCCAGAGCTCCTGAGG
ACTGTCCGCCTAGCCCTTCTGCCTGCCATCTTCCTAATGGAGAACGTTTC
TACAGAAGAGCTGATCAATGCCCAGGCCAAAAGCAAGGAACTGGTGGATG
AAGCTATCCGCTGCAAGCTGAGGATCCTGCAGAACGATGGTGTTGTCAAC
AGCCCGTGTGCGCGGCCTAGAAAGACGAGCCATGCTCTCTTCCTCCTAGG
TGGCCAGACCTTCATGTGTGACAAGTTGTACCTGGTGGACCAGAAAGCCA
AAGAGATCATCCCCAAGGCTGACATTCCCAGTCCGAGGAAGGAGTTCAGT
GCCTGCGCTATTGGCTGCAAAGTGTACATCACAGGCGGGA--GGGGCTC-
GGAGAATGGGGTCTCCAAAGATGTGTGGGTCTATGACACCGTCCATGAGG
AGTGGTCAAAGGCAGCACCCATGCTGATCGCTAGGTTTGGCCATGGTTCA
GCTGAGCTGAAACACTGCCTATACGTAGTTGGAGGTCACACTGCAGCGAC
TGGTTGCCTCCCAGCGTCTCCGTCTCGATGAGTACATCGTGGTCTTCAGC
CGCTCCGTCAACCGGCTCATCCTCAACGAGGCTGAGTTGATCCTGGCTCT
GGCGCAAGAGTTCCAGATGAGGGCCATCACAGTGTCCCTGGAGGAGCAGT
CGTTTGCCAGCATCATTCAGTTGATCAGCGGGGCGTCCATGCTGGTCAGC
ATCCACGGAGCCCAGCTGATCAGCTCCCTGTTCCTGCCACGCGGCGCAGC
CGTGGTGGAGCTCTTCCCCTACGCCGTCAACCCAGAGCAGTACACTCCCT
ACAAAACTCTGGCTTTGTTGCCAGGCATGGACCTCCAGTATGTGGCTTGG
AGGAACACCATAGAGCAGAACTCGGTGGCTTACCCTGAGCGTGCCTGGGA
CCAGGGTGGCATCGCCCACCTGGAGAAGGAGGAACAGGAGCGTATCCTGG
CGAGCGATGAGGTGCCGCGGCACCTGTGCTGCCGCAACCCAGAGTGGCTT
TTTCGGATTTACCAGGACACCCAGGTGGACGTCCCCTCCTTGCTGGAGGT
CCTCCG---AGAAAATCTGAAG---GCCAAGCCTAACCTGAGAAA---GG
CCAAGGCGGCTAGTACAGTACACCCCGGTAGAGTCAGGGAGCCCAAGTGC
CAGACCTCGGTCCAGGCCACCAACGAGGCCAAGCTGACCGTGTCGTGGCA
GATCCCCTGGAACCTCAAATACCTGAAGGTCAGGGAGGTCAAATATGAAG
TATGGATACAG---------------------------------------
--------------------------------------------------
--------------------------------------------------
--------------------------------------------------
--------------------------------------------------
--------------------------------------------------
--------------------------------------------------
--------------------------------------------------
--------------------------------------------------
```

```
--------------------------------------------------
--------------------------------------------------
--------------------------------------------------
--------------------------------------------------
--------------GGAGGCCTTCAAGTGTGAGGAGTGCGGGAAGCACTA
CAACACCAAGCTGGGATATAAGCGCCATGTGGCCATGCACTCTGCTACAG
CAGGGGATCTCACCTGCAAAGTGTGCATGCAGAGCTATGAGAGCACACCC
GTGCTCTTGGAGCACCTCAAGAGCCACTCCGGGAAGTCTTCGGGTGGCGC
CAAGGAGAAAAAACACCCRTGCGACCACTGTGACCGTCGTTTCTACACAC
GGAAGGATGTGAGACGGCACATGGTGGTCCACACAGGCCGAAAGGACTTC
CTATGCCAGTACTGTGCCCAGCGCTTTGGCAGGAAGGACCATCTGACACG
CCACGTGAAGAAGAGCCACTCGCAGGAGCTCCTGAGGATCAAGACGGAGC
CTCCTGATATGTTAGGTCTTTTAGCTTCTGGGTCGCCACCTTGCTCTGTG
AAGGAGGAGCTCAGCCCCATGATGTGCGGCATGGGTCCCAACAAAGACCC
CATGATGGGCAAACCGTTCCCAAGTGGGGCCCCTTTTCCGATGGGCATGT
ACAACCCCCACCAT------CTCCAGGCCATGTCTARTTCTGGGGTGGGT
CACCCA------CACCCGTCCCTGATGCCTAGTCCCTTGTCTGCTGCTAT
GGGCATGGGCTGTCACATGGAA----------------------------
--------------------------------------------------
--------------------------------------------------
--------------------------------------------------
--------------------------------------------------
--------------------------------------------------
--------------------------------------------------
--------------------------------------------------
--------------------------------------------------
--------------------------------------------------
--------------------------------------------------
--------------------------------------------------
--------------------------------------------------
--------------------------------------------------
--------------------------------------------------
--------------------------------------------------
--------------------------------------------------
--------------------------------------------------
--------------------------------------------------
--------------------------------------------------
--------------------------------------------------
--------------------------------------------------
--------------------------------------------------
--------------------------------------------------
--------------------------------------------------
--------------------------------------------------
--------------------------------------------------
--------------------------------------------------
--------------------------------------------------
--------------------------------------------------
--------------------------------------------------
--------------------------------------------------
--------------------------------------------------
--------------------------------------------------
--------------------------------------------------
--------------------------------------------------
```

```
--------------------------------------------------
--------------------------------------------------
--------------------------------------------------
--------------------------------------------------
--------------------------------------------------
--------------------------------------------------
--------------------------------------------------
------------------------------GGGAGGAGAGCCCGCAGCCT
TTCTTTTGTTCCATGGAAGWYCCCACGAAACAGACAWAGTTCAAGGGCAT
CAAGACCTACATTTCGTACCGGGTGACGCCSAGTCACACGGGGGACCCCG
TGTACMGGCGCTACAAACACTTTGACTGGTTGTACAACCGCSTGCTGCAC
AAGTTTACTGTGATATCCGTGCCCCACCTGCCWGAGAAACAGGCCACGGG
GCGCTTCGAGGAAGACTTCATCGAGAAGCGCAAGAGACGGCTGATACTGT
GGATGAACCACATGACCAGTCACCCAGTCCTCTCCCAGTACGAAGGCTTC
GAGCACTTTCTGATGTGCGCCGACGACAAGCAGTGGAAACTGGGGAAGAG
GCGGGCGGAGAAGGAMGAGATGGTGGGAGCCCACTTCATGCTGACTCTKC
AGATCCCCACCGAGCACCAGGACCTTCAGGACGTGGAGGAGCGCATKGAM
MCCTTCAAGTSCTTNGCCAAAAAGATGGACGACAGCSTGATGCAGTTKACG
CACGTGGCCTCCGAGCTGGTGCGCAAACACCTGGGGGGGTTCAGGAAGGA
GTTCCARCGGSTGGGAAATGCCTTCCAGTCCATCAGCCAGGGCTTCATGG
TGGA----------------------------------------------
--------------------------------------------------
--------------------------------------------------
--------------------------------------------------
--------------------------------------------------
--------------------------------------------------
--------------------------------------------------
--------------------------------------------------
--------------------------------------------------
--------------------------------------------------
--------------------------------------------------
--------------------------------------------------
--------------------------------------------------
--------------------------------------------------
--------------------------------------------------
--------------------------------------------------
--------------------------------------------------
--------------------------------------------------
--------------------------------------------------
--------------------------------------------------
----------------------------------GCCAAATCCCGCTTTC
ACCATGGCGTCGGCACGGGGCCTGGCACGGACC---GCAGCGTCCCACTC
---AACAGCTTGCTATCCCCGCAACAAACCGATGAGACCGCAGTGG---C
CTCCCCGCAGCGATGGTTTGTCACCC---CTGCCAACAACCGACTGGACT
TTGCCGCCTCGGCATACGATGCCGCCGCTGCAGCTGATTTTGCCGGTAAC
GCGGCCACCCTTCTGTCGTACGCAGCTGCCGGAGTGAAAGCGC------T
CCCACTGCCCACTGCAGGCTGCTCCAACAGAGCCTTGGGCTATTACGCGG
AGCCGCCAG---GGTGG---GGCACACGCACTCCGCCGCAGTACTGT---
------AGTAAATCAAGCGCGGTTCTCTCATGCTGGCCCGCCAATTCCGT
TGGGAGCAGAACATCCA---CCTCCAATTACCTGG---TTGGCTTGGACG
A---CGGG---GACGC---TATCGCACCTGAGAGGTCACCT---CT---C
GGGGGGGCAGACGAA---GCCAAGCCAAAAGACCT------GTCGGA---
ATCAAGCTGGATAGAG---ACCCCGTCTTCAATAAAGTCAATCGACTCAA
```

GTGATTCTGGGATCTTTG---AGCAGGCCAAACGGAGGAGAATTTCGCCA
TGTGCTACACCG---------GTTTCAGAGACGTCGTCCCCATTAGAATC
AGAA---------ACAGGCGAAGTCACAGACAGAGAAGTGGCTTTGGGGA
TAAATCCGTTCGCCGACGGGATGGGAGCGTTCAAAATCAACCACAGCTCC
CACGATCTTGGTTCTGG---GCAAACGGCGTTTGCCTCGCAGGCG---CC
CGGTTAC---GCAGCCGCTGCCCTGGGA---CACCATCA------CCACC
CAACCCATGTCAGCTCC---TACTCCACCGCGGCGTTCAACTCCACCCGG
GACTTTCTCTTTCGGAATCGGGGATTCGGAGACGCCACTAG---------
---CGCGCAGCACAGTCTCTTCGCCTCAGC---TGCAGGAAGTTT---C-
-----GCAGGGCCACATGGACACACCGATGCCACGGGACACCTGCTCTTC
TCGGGACTGCACGAG---CAAGCGGCGACGCACGCGTCTTCGAACGTGGT
GAACAGTCAGATGCGCCTGGGCTTTTCGGGGGACATGTACGGCAGAGCCG
AGCAGTACGGTCACGTAACGAGCCCCCGGT---CCGAGCACTACGCTTCG
ACTCAGTTGCACGGCTATGGCCCCATGAACATGAATATGGCTGCC---CA
CCACGGGGCAGGGGCCTTCTTCCGTTACATGAGGCAGCCGATCAAACAAG
AGCTCATCTGCAAGTGGGTCGAACCAGAGCAGCTGTCGAATCCGAAAAAG
TCCTGCAACAAAACTTTCAGCACGATGCATGAGCTCGTGACCCACCTCAC
AGTGGAACATGTCGGGGGACCGGAACAGTCAAATCACATTTGCTTTTGGG
AAGAGTGTCCGCGAGAAGGGAAACCATTTAAAGCCAAGTACAAACTTGTA
AATCATATCAGAGTGCACACTGGAGAAAAACCGTTTCCGTGTCCATTCCC
CGGCTGTGGCAAA
>Ameiurus natalis
AGCCTGCTTATTCGGGCAGAGCTAGCCCAACCTGGTGCCCTCCTAGGCGA
TGATCAAATTTACAATGTTATTGTTACTGCCCACGCCTTTGTAATAATTT
TCTTTATAGTAATGCCAATTATAATCGGGGGATTTGGAAACTGACTTGTA
CCCCTTATGATTGGAGCCCCCGATATAGCTTTCCCCCGAATAAATAACAT
AAGCTTCTGACTCCTCCCCCCTTCTCTTCTCCTTCTACTAGCCTCCTCCG
GAGTTGAAGCAGGAGCAGGAACAGGATGAACTGTTTACCCTCCACTTGCT
GGTAACCTTGCACATGCGGGGGCCTCTGTAGACTTAACTATCTTTTCACT
TCACCTTGCAGGGGTTTCATCTATTCTAGGGGCCATTAATTTTATTACAA
CTATTATCAACATAAAACCCCCCGCAATTTCACAATATCAGACGCCCCTA
TTTGTTTGAGCCGTTCTTATCACAGCCGTCCTCCTATTACTGTCCCTCCC
AGTCTTAGCAGCTGGTATTACAATACTTCTTACTGATCGAAATTTAAACA
CCACATTCTTTGATCCAGCAGGAGGAGGGGACCCTATCTTATATCAACAT
CTT---

------------NNNNNNNNNNNNNNNNNTCATCCATCCAACTGCCTCGGCATGCTGCTTTTGTCTG
ATGCCCACCACTGCACCCAGCTGTCCCAGCTTTCCTGGAACATGTGTCTC
AGTAATTTCCCAGCTATCTGCAAGACAGAGGAATTTCTGCAACTTCCCAA
GGACATGATAGTCCAGTTGCTCTCACACGAAGAGCTGGAGACAGAGGATG
AGCGGTTAGTCTATGAATCTGCACTTAACTGGGTGAACTATGACTTGGAA
CGGCGACATTTCGACTTACCAGAGCTGTTGCGCACTGTACGTCTAGCCCT
CTTGCCAGCCATCTTCCTCATGGAGAATGTCTCCACAGAGGAGCTTATCA
ACTCACAAGCCAAAAGCAAAGAGTTGGTGGATGAAGCAATCCGCTGCAAG
CTACGTATACTGCAGAATGACGGCGTGGTCAACAGTCCTTGTGCCAGGCC
CCGCAAGACTAGCCATGCCCTTTTCCTGCTAGGTGGTCCTACCTTTATGT
GTGACAAGCTGTATTTAGTGGACCAGAAGGCCAAAGAAATAATACCAAAA
GCAGATATTCCCAGTCCACGAAAGGAGTTCAGTGCTTGTGCTATTGGTTG
CAAAGTCTATGTGACAGGTGGTC--GGGGCTC-AGAGAACGGTGTGTCCA
AGGATGTTTGGGTGTACGATACCTTGCATGAAGAGTGGTCAAAAGCAGCT
CCCATGCTAATAGCTCGATTTGGGCATGGTTCAGCTGAANNNNNNNNNNNNNNNNNNNNNNNNNNNNNN

NNNNNNNNNNNNNNNNNNNNNNNNNNNNNNNNNNNNNNNTGATGATTATA
TAGTAGTGTTTAGTAGAACTTCTAACAGGTTGATACTCAATGAGGCAGAG
TTGCTTCTTTCTTTGGCTCAAGAGTACAAAATGAGAACAGTCACCGTGTC
CTTGGAGGAGCAAACATTTGCAAGTATAGTCCAGCTTATCAGTGCGGCAT
CCATGCTGGTCAGCATGCATGGTGCTCAACTGATCACGTCCATGTTCCTA
CCACGAGGGGCTGCTGTCATCGAGCTTTTTCCATACGCTGTCAACCCAGA
ACAGTATACTCCATATAAAACTCTGGCCTCCTTGCCAGGAATGGACTTGC
AGTACATAGCATGGAGAAACACCATTGAAGAGAATTCTGTAACCTATCCT
GACCGCCCTTGGGACCAAGGAGGTATCACTCACCTGGAAAAAGAGGAACA
GGAACGTATCCTAGCTAGTAAGGAGGTGCAGAGACACCTCTGCTGTCGCA
ACCCAGAGTGGCTTTTCCGGATTTATCAAGACACCATTGTGGACATTCCT
TCCTTCCTGGAAGTCCTTCG---AGACGGTCTAAAA---ATTAAGCCTAA
TCTGAAAAA---GAGCAAACCTGTCAGTGTAGTCCATCCTGGTCGAGTCA
GACATCCCAAGTGCCAGACATCAGTGCAGGCTGCCCATGAAGCAAAGCTG
TCTGTGTCCTGGCAGATTCCTTGGAACCTCAAGTACTTGAAGGTAAGAGA
GGTGAAATATGAAGTGTGGATCCAGAAGAAAGATTCTAACAAAGGGACAC
TGGAAGATCAAATCATCCAGGCCAACCCTGCACTAGAGGCTTTTGGCAAT
GCCAAAACAGTGAGAAATGACAACTCATCTCGCTTTGGAAAGTTCATTCG
CATTCATTTTGGAACAAGTGGTAAACTATCTTCAGCTGACATTGAAACAT
ACCTGCTGGAAAAATCACGAGTGACCTTTCAGCTAAAGGCAGAAAGAAAC
TATCACATCTTTTACCAGATACTGACCAATGCAAAGCCTGAGCTGTTGGA
TATGCTGCTGATTACAAATAATCCGTACGATTACTCTTACATCTCCCAAG
GAGAAATATCTGTTCAATCCATCAATGATAGTGAGGAGTTAATGGCCACT
GACAATGCATTTGATGTGCTAGGCTTCACCGCAGATGAGAAGACGGGAGT
CTACAAACTGACGGGAGCCATCATGCATTATGGCAACATGAAATTCAAGC
AGAAACAGCGAGAAGAACAGGCAGAACCAGATGGTACAGAAGCTGCTGAC
AAGTCAGCCTACCTTATGGGGCTGAACTCTGCTGATCTCCTGAAAGGACT
CTGTCATCCACGGGTTAAGGTAGGAAATGAATATGTCACAAAAGGCCAGG
GTGTGGATCAAGTATACTACCCCAACAAAGAGGCTTTTAAGTGTGAGGAG
TGTGGCAAGCACTATAACACAAAGCTAGGCTACAAACGGCATGTGGCCAT
GCACTCTGCCACAGCAGGAGACCTGACCTGCAAGGTATGTTTGCAGAGCT
ACGAGAGCACTCCTGCCCTCTTGGATCACCTCAAGAGCCATTCTGGCAAG
TCTTCAGGTGGTACCAAGGAGAAGAAACACCCATGCGATCACTGTGACCG
TCGCTTCTACACCCGCAAGGATGTTCGGCGACACATGGTAGTGCACACGG
GTCGCAAAGATTTCCTGTGCCAGTACTGTGCCCAGCGCTTTGGAAGAAAG
GACCACCTCACACGACACGTGAAGAAGAGCCACTCACAGGAGCTGCTGAA
GATCAAGACAGAGCCACCAGATATGCTGACTCTACTTGGTTCTGGCTCTC
CAACCTGTGCTGTCAAGGAAGAGCTCAGTCCTATGATGTGTAGCATGGGC
CCCAACAAGGACTCCATGATGACCAAACCTTTCCGTAGTGGTACCCCTTT
CTCTATGGGCATGTACAACCCACACCAC------CTCCAGGCCATGTCCA
GCCCAGGATTGGGCCAC---------CATCACTCCTTGGTTCCTGGGTCA
CTGTCCGCTGCTATGGGTATGGGTTGCTCCATGGAGNNNNNNNNNNNNNNNNNNNNNNNNNNNNNNN
NNNNNNNNNNNNNNNNNNNNNNNNNNNNNAGTGAATTTACTCG
CCAGCAACTCACCCAGTGTGTCGTTTGCCCTGACCCAGCAGAAACTGTTC
AGCAACTATAGCCCGGTGATAGGATTCTACATCTATGAGCCTATTGAATA
CTGGAACTCCACAGTACAGGAGCACCTCAAGACACTAGGGCAGGGTTTCA
ATAAAATTTCCTGGATTGATAATTACTTTAATTATTTGAAGGTCACAAAT
GTCAGCGCATCAACCAAAAGCGATTTCATCAATATTCTCAAGACGTCGTT
TCTTAGAAGACCGGAGTATCAGCACTTCACGGAAGACATCATTTTCTCAA
AAA---ATGGCG------------ATGAGTATGACATCATTGCATCCAGG
ATGTACTTGGTAGCGAGAACCACTGAGAAGACAAGGGAGGAGGTGGTGGA
GCTGCTGGAGAGACTCAGACCCCTCTCTCTTATAAACAGCATCAAGTTCA
TCGTCTTCAACCCCACCTTTGTGTTCATGGACCGCTACAGTTCCTCAGTT

GTCTCCCCGATTCTAACATCAGGTTTCAGTGTCCTGACCATCCTCATCCT
CACGTTCTTCCTCGTCATTAACCCCTTAGGGAACTTCTGGTTGATTTTGA
CCGTCACCTCTGTGGAGCTGGGTGTCCTAGGCCTCATGGGCTTCCATCAA
TTTGAGTGGCAACCAGCTCTCAAAAATGTCTCAAACTCTTGCAATACTGG
GATCATCGATGGGCTTTCTGGGTGGACTGCCTCTGTAGATGATGTACCTG
CAGAGACCATTTCTAGAAGATTCCGTTATGATGTGGCGCTGGTATCAGCG
TTAAAGGACTTGGAGGAGGACATCATGGAGGGACTTAGAGAAAAAGGATT
GGATGACAGCACCTGCACCTCAGGTTTCACTGTGGTGATTAAGGAGTCAT
GTGATGGTATGGGAGATGTCAGTGAGAAGCATGGAGGAGGACCAGCTGTC
CCAGAAAAGGCAGTGAGATTTTCTATTACAGTGATGTCTATCACTATTCA
AGCTGAAGGTGAAGAG------------------------------GAAG
CAGTCACAATCTTCCAGGAAAAAAAGCCTAACTCTGAGCTTTCCTGTAGG
CCACTGTGCCTCATGTTTGTGGATGAGTCTGATCATGAGATACTGACGGC
CATCTTGGGACCTGTGGTGGCAGAGCGTAAAGCTATGAAGGAGAGTCGCC
TCATTCTCTCTATAGGTGGGCTCCAACGCTCCTTCCGCTTTTATTTTAGG
AGCACAGGCTATGATGAAAAAATGGTGAGAGACATGGAAGGTTTGGAGGC
TTCAGGCTCCACTTACATCTGCACCCTCTGTGACTCAACACGAGCTGAAG
CCTCTCAGAATATGGTACTTCACTCCATCACTAGAAGCCATGATGAAAAT
CTTGAGCGCTATGAGATATGGAGAACTAACCCTTTTTCTGAGTCTGCAGA
GGAGCTTCGGGATCGAGTCAAAGGGGTCTCAGCCAAGCCTTTCATGGAGA
CACAACCCACATTAGATGCCTTGCACTGTGACATTGGCAATGCTACTGAA
TTCTACAAGATCTTCCAAGATGAAATTGCTGAAGTGTACTTGAAAAA---
CAAC---CCAACTCGAGAAGAACGCAGACGCTGGCGGTCAGCCCTTGACA
AGCAGTTGAGAAAGAAGATGAAGCTAAAACCAGTGATGAGAATGAATGGG
AACTATGCCCGGCGACTTATGACCCGTGAGGCTGTTGAAGTGGTGTGTGA
GCTAGTTCCTTCAGAGGAGCGCCGTGAGGCTTTGAAAGAACTAATGGAGC
TGTATCTCCAGATGAAACCTGTGTGGCGTTCTACTTGTCCAGCTCGAGAG
TGTCCTGATCAGCTTTGCCGCTATAGCTTCAACTCTCAACGTTTTGCAGA
GATCCTCTCCACCACATTCAAATATCGCTATGATGGCAAAATTACTAACT
ACCTCCACAAGACTTTAGCCCATGTACCTGAAATTGTTGAGCGGGATGGC
TCAATTGGTGCCTGGGCCAGCGAAGGGAATGAATCTGGGAACAAANNNNNNNNNNNNNNNNNNNNNN
NNNNNNNTCAGT
GGAAGGAGAGTCCTCAGCCTTTCATCTGCTCCATCGAGGATCCCACCAAA
CAGACCAAGTTCAAGGGCATTAAGAGTTACATTTCCTACAGAGTCACCCC
CAGCCACACTAGCAGGCCTGTGTATCGGCGCTATAAGCACTTTGACTGGC
TGTACAATCGCCTGTTGCACAAATTTACCGTCATTTCCGTACCCCACCTC
CCGGAGAAGCAAGCCACCGGGCGNTTCGAGGAAGACTTCATCGAAAAGAGA
AAGCGGCGGCTCATCCTATGGATGGATCACATGACCAGTCACCCGGTTCT
GTCTCAGTATGAGGGTTTCGAACACTTCCTCATGTGTGTGGATGACAAGC
AGTGGAAACTGGGAAAGCGTCGTGCTGAGAAGGATGAGATGGTGGGAGCT
CATTTTATGCTCACTCTCCACATCCCTAAAGAGCATCAGGACCTGCAAGA
CGTCGAGGAGCGCATCGATACCTTCAAGGCCTTCGCCAAGAAAATGGACG
ACAGCGTCATGCAGCTGACGCACGTCGCGTCCGAACTCGTCCGCAAGCAT
CTCGGCGGCTTTCGCAAGGAGTTTCAGCGGCTGGGTAATGCTTTCCAGTC
CATCAGCCAGGCGTTCATGCTGGACCCTCCGTACAGNNNNNNNNNNNNNNNNNNNNNNNNNNNNNNCCC
CTCGCCACATT
CCTCAAACTAACCTCCCTGGGCTTCATCATCGGCGTCGGTGTTGTGGGAA
ACCTCCTGATCTCCATCCTGCTGGTCAAAGACAAGAGCCTGCACCGTGCA
CCCTATTACTTCTTGTTGGACCTGTGCGCCTCTGACATCCTGCGCTCAGC
CATCTGCTTCCCCTTTGTATTCACCTCGGTGAAGAACGGCTCGGCGTGGA
CGTACGGCACCCTCACGTGCAAAGTGATCGCCTTCCTGGGTGTGCTCTCC
TGCTTCCACACAGCCTTCATGCTGTTCTGTGTCAGCGTGACACGTTACTT
AGCTATCGCACACCACCGCTTCTATACCAAACGGCTGACCCTGTGGACGT

GCTTGGCTGTGGTGTGTATGGTGTGGACACTGTCAGTGGCCATGGCCTTT
CCGCCTGTGCTGGACGTGGGCACATACTCATTCATCCGTGAGGAGGACCA
GTGCACGTTTCAGCACCGCTCATTCCGAGCCAATGACTCGCTGGGCTTCA
TGCTGCTGCTCGCCCTGATCCTCCTGGCCACCCAGCTTGTCTACCTCAAG
CTCATATTCTTCGTTCACGACCGGCGCAAGATGAAACCGGTCCAGTTCGT
GCCTGCTGTCAGCCAAAACTGGACCTTCCACGGGCCAGGAGCCAGCGGGC
AAGCAGCGGCTAACTGGCTGGCTGGTTTCGGCCGAGGCCCGACACCTCCA
ACCTTGCTGGGCATCCGGCAGAACGGCAATGCGGCAGGCCGGCGGCGCCT
CCTGGTGTTGGATGAGTTTAAGACTGAGAAGAGGATAAGCAGAATGTTCT
ACGTCATGACTTTCTTCTTCCTTAGTCTGTGGGGGCCTTACCTAGTGGCA
TGCTACTGGAGAGTGTTCGCCCGTGGCCCTGCCGTGCCAGGGGGCTATCT
CACAGCAGCTGTATGGATGAGCTTCGCTCAGGCTGCCGTCAATCCCTTCA
TTTGCATCTTCTCCAACCGTGAGNNNNNNNNNNNNNNNNNNNNNNNNNNNNNNNNNNNNNNNNNNNNNNNNNNNNNACGCG--
------------CCCACTTA
GTAACAGCTTGCCCTCCCCGCAACAAACCGAGGACACCACGGTGG---CC
TCCCCGCAGCGATGGTTTGTCACCC---CTGCCAACAACCGACTGGACTT
TGCCGCCTCGGCATACGATGCTGCCGCGGCTGCTGATTTCGCCGGTAACG
CGGCCACCCTTCTGTCGTACGCAGCTGCCGGAGTTAAAGCGC------TG
CCCCTGCCCGGGGCCGGGTGCTCCAACAGACCCCTGGGGTATTACGGCGA
GCCGCCGG---GGTGG---GGCACCCGCACACCACCGCAATATTGC----
-----AGCAAGTCCAGCTCTGTCCTGTCGTGCTGGCCGTCCAACTCCGTA
GGGAGCAGAACGAGCT---CCTCCAGTTACCTGGTCCCTGGCCTCGACGA
---CGGA---GACGC---CATCGCGCCCGAGAGGTCACCG---CT---AG
GCGGGGCGGATGAG---TCCAAACCCAAAGACCT------GTCCGA---A
TCCAGCTGGATAGAA---ACCCCGTCCTCAATTAAGTCGATCGATTCGAG
CGATTCTGGGATTTTTG---AGCAAGCCAAGCGGAGGAGGATTTCACCAT
CTGCCACAAGG---------NNNNNNNNNNNNNNNNNNNNNNNNNNNNNNNNNNNNNNNNNNNN--------------------
----

>Ammodytes hexapterus
AGCCTGCTCATCCGAGCAGAACTTAGCCAACCCGGCGCCCTCCTAGGAGA
TGATCAAATCTATAACGTAATTGTTACCGCTCATGCATTCGTAATGATTT
TCTTTATAGTAATACCAATTATGATTGGTGGTTTTGGAAACTGACTAATC
CCCCTAATAATTGGTGCCCCTGATATAGCATTCCCTCGAATAAATAACAT
GAGCTTTTGACTCCTCCCACCCTCCCTCCTTCTTCTTTTAGCCTCTTCAG
GCGTAGAAGCTGGAGCTGGTACCGGTTGAACTGTATACCCTCCCCTGGCC

GGAAATCTAGCCCACGCAGGTGCATCTGTTGACTTAACAATCTTCTCTCT
GCATTTAGCCGGGATCTCTTCGATTCTTGGAGCAATCAACTTCATCACCA
CAATTATTAACATGAAACCTCCCGCTATCTCTCAGTATCAGACACCCTTA
TTTGTGTGAGCTGTGCTGATTACAGCCGTCCTTCTCCTCCTCTCCCTCCC
CGTCCTTGCTGCCGGCATTACAATGCTTCTTACAGACCGCAACTTAAATA
CCACCTTCTTTGACCCTGCAGGAGGGGGAGACCCTATTCTGTACCAACAC
CTGTTCTGATTCTTCGG---------------------------------

NNNNNNNNNNNNNNNNNNNNNNNNNCTAATTGCCTTGGCATGGCGTTGTTGTCTGACGCTCAC
CACTGCATCAAGCTGTCAGAGCTGTCCTGGGGCATGTGCCTCAGCAACTT
TCCCACTATTTGCAAGACAGAGGACTTCCTCCATCTGCCCAAAGATATGG
TGGTGCAGCTTTTGTCACATGAGGAGCTGGAGACAGAAGATGAGAGACTG
GTTTATGAAGNTGCCCTGAACTGGATCAACTATGACCTGGAAAGGAGGCAC
TGCCACCTTCCAGAGCTCCTGAGAACGGTCCGTCTTGCCCTGCTGCCCCC
CATCTTTCTGATGGAGAATGTCTCGACAGAAGAGGTGATCAACGCCCAGG
CCAAGAGCAAGGAGCTGGTGGATGAGGCTATCCGCTGTAAGCTGAAGATC
CTTCAGAATGACGGCGTTGTTAACAGCCCGTGTGCTCGACCAAGAAAAAC
CAGCCATGCTCTCTTTTTCTTGGGAGGGCAGACTTTCATGTGTGACAAGT
TGTACCTGGTGGACCAGAAGGCCAAAGAGATCATCCCGAAGGCGGACATT
CCCAGCCCAAGGAAGGAGTTCAGCGCCTGCGCCATCGGCTGTAAGGTGTA
CATCACAGGTGGGA--GAGGCTC-AGAGAATGGNGTGTCCAAAGATGTATG
GGTCTATGACACCGTCCAGGAGGAATGGTCCAAAGCGGCGCCCATTATCA
TGGCCAGGTTTGGNNNNNNNNNNNNNNNNNNNNNNNNNNNNNNNNNNNNNNNNNNNNNNNNNNNNN
NNNNNNNNNNNNNNNNNNNNNNNNNNNNGGATGAATACATTGTTGTGTTTAGTCGTTCAGCAACA
AGGCTTATTTTGAATGAAGTGGAGCTCATTATGGCACTGGCCCAGGAGTT
CCAGATGAGAGTGGTCACAGTGACCCTGGAGGAACAGTCATTCCCCAGTA
TCATCCAGGTCATTAGCAGTGCTTCCATGTTAGTTAGTATGCATGGAGCT
CAGCTTATCACCTCACTCTTTCTCCCCAGAGGAGCTGCTGTTGTGGAGCT
GTTTCCCTTTGCTGTGAACCCAGAACAGTACACCCCATATAAAACCCTGG
CCTCCCTTCCTGGCATGGACCTTCACTATGTCTCCTGGAGGAATACTAAA
GAGGAGAACACCATCACCCACCCAGACAGAGCCTGGGAACAAGGAGGCAT
CACTCACTTGGATAAGGAGGAGCAAGAGCGAATACTGGCCAGCAAAGATG
TTCCCAGGCACCTCTGTTGTCGCAACCCAGAGTGGCTCTTCCGGATCTAC
CAGGACACTATGGTGGACATCCCTTCCTTCCTGGGAGTCCTGAG---AGA
CGGCATAAAG---ACAAAGCCTGGCTTAAAGAA---GTCAAAGCCAGCCA
GCACAGTTCACCCAGGCCGGGTCAGACAACCCCACTGTCAGACCTCAGTA
CAAACCACTAGTGAGGCTAAACTCACTGTCTCCTGGCAGATCCCATGGAA
TCTGAAATACCTAAAGGTGAGAGAGGTCAAGTATGAGGNNNNNNNNNNNN-----------
----

----------------------------------CCCAACAAGGAGGCCTT
CAAGTGTGAGGAGTGTGGGAAGCACTACAACACCAAGCTGGGATATAAGC
GCCACGTGGCCATGCACTCTGCCACTGCGGGAGATCTCACCTGTAAAGTG
TGCATGCAGACCTACGAGAGCACGCCCGTTCTCCTGGAGCACCTCAAGAG
CCACTCTGGGAAGTCTTCGGGTGGCACCAAGGAGAAGAAACACCCGTGTG
ACCACTGCGACCGCCGCTTCTACACCCGCAAGGATGTGAGACGGCACATG
GTGGTCCACACGGGCCGCAAGGACTTCCTGTGCCAGTACTGTGCCCAGCG
CTTCGGCAGGAAGGACCACCTGACACGCCACGTGAAGAAGAGCCACTCAC
AGGAGCTCCTGAAGATCAAGACGGAGCCTCCTGACATGCTAGGTCTTTTA
GCTTCGGGCTCCTCGCCTTGCTCCGTGAAGGAAGAGCTCAGCCCCATGAT
GTGCAGCATGGGTCCCAATAAAGACCCCATGATGGGCAAACCATTCTCCA
GTGGGGCCCCCTTCTCCATGGGCATGTACAACCCGCACCAT------CTC
CAGGCCATGTCTAATACTGGGGTGGGTCACCCA------CACCCGTCCCT
GATGCCCGCTTCCTTGTCTTCTGGCATGGGCATGGGCTGTCACATGGAAT
ATCTCATATACGCTTCTTTCTCATTCATGGGATGTTTACAAATCAGTGAT
GGATCGAATGTTGTAAATCTGTTGGCTAGTAACTCTCCGAGTGTCTCGTA
TGCTATGACCCAGCAGAAATACTTTAGTAACTACAGTCCTGTGATTGGGT
TTTACATCTACGAGCCCATCGAGTACTGGAACTCCACGGTGCAGGAGCAT
CTGAAGACTCTGAGTCACGGTTTCAACAAGATCTCCTGGATGGACAACTT
TTTCCACTACCTGCGGGTGGTGAATGTGAGTGCGTCAACAAAGGGCGACT
TCATCAGCATCCTCAAGGGCTCCTTCCTGCGCAGCCCGGAGTACCAGCAC
TTCACAGAGGACATCATATTCTCCAAGA---ACCGTGAGACCG------A
TGAGTATGACATTATCGCCTCGCGGATGTACTTAGTTGCACGGACNACGGA
GAAGAAGCGCGAGGAGGTGGTGGAGCTTCTGGAAAAGCTCCGTCCGTTGA
TGCTGATCAACAGCATCAAATTCATTGCCTTCAATCCCACGTTTGTGTTC
ATGGACCGCTACAGCTCCTCTGTCATCTCGCCCATCCTGACCTCAGGCTT
CAGCGTACTCACAATCCTCATCCTCACATTCTTCCTGGTCATCAACCCCT
TGGGGAACTTCTGGCTCATCCTCACCGTGACGTCGGTGGAGCTGGGAGTG
TTGGGTCNNNNNNNNNNNNNNNNNNNNNNNNNNNNNNNNNNNNNNNNNNNNNNNNNNNNNNNNNNNNNN
NNNNNNNNNTTAATGGGCTCTCTGGATGGGCTTCCTCGGTGGATGACTGCCC
TGCTGACACCATCACTAGGCGCTATCGCTATGATGTGGCACTGGTGTCAG
CATTAAAAGACCTGGAGGAGGACATCATGGTGGGGCTGAGAGAGAGCGGG
ATGGAAGACAGCGCATGCACCTCAGGCTTCAGTGTCAAGATCAAAGAATC
TTGTGATGGCATGGGCGATATCAGTGAGAAGCACGGTGGAGGACCAGCCA
TTCCTGAGAAAGCTGTACGTTTCTCTTTGACTGTTATGTCAGTCTCTGTC
CTGGCAGAGGACAAGGAG------------------------------GA
AGAGATGACCATCTTTACTGAGCCAAAGCCAAACTCTGAACTGTCTTGCA
AGCCTCTTTGCCTTATGTTTGTGGATGAGTCAGACCACGAGACAATCACA
GCCATCCTGGGGCCTATAGTCGCAGAGCGTAATGCCATGAAAGAGAGTAG
GCTCATCTTATCCATGGGTGGACTACCTCGCTCCTTCCGCTTCCACTTCA
GAGGCACGGGATACGATGAGAAAATGGTGCGTGAGATGGAGGGCTTGGAG
GCCTCAGGGTCCACATACATCTGCACTCTTTGTGACTCCAGTCGGGCAGA
GGCATCTGAAAACATGGTGCTACACTCTGTCACGCGCAATCATGACGAGA
ACCTAGAGCGTTACGAAATATGGAGAACCAATCCCTTTTCTGAGTCTGTG
GATCAGCTGCGAGACAGAGTCAAAGGGGTGTCTGCCAAGCCCTTTATGGA
GACCCACCCCACTCTGGATGCATTACACTGCGACATTGGCAACGCAACCG
AGTTCTACAAAATCTTCCAGGATGAGATCGGGGAGGTGTTCCAAAGGGT-
--CAAC---CTCAGTCGGGAGGAACGGCGTAGCTGGAGAGCAGCGCTAGA
TAAACAGCTGAGGACGAAAATGAAGCTTAAACCAGTAATGAGGATGAATG
GGAACTTTGCCCGCAGGCTAATGACCCTGGAGGCTGCAGAGGTGGTGTGT
GAGTTGGTGCCCACAGAGGAGAGAAGGGAGGCCCTGAGGGAGCTCATGAG
GCTGTACATCCAGATGAAGCCAGTATGGCGTGCCACCTGCCCAGCCAAGG
AGTGCCCTGACCAGCTGTGCCGCTACAGCTTTAACTCCCAGCGCTTTGCC

GACCTTCTCTCCTCTACCTTCAAATATAGGTACAATGGGAAGATAACCAA
TTACCTGCACAAGACCCTGGCCCATGTTCCCGAAANNNNNNNNNNNNNNNNNNNNNNNNNNNNNNNNNN
NNNNNNNNNNNNNNNNNNNNNNNNNNNNNTCGTACACCATCGAA
ATGGGTCCCYTGGGTCCCGGTTGGAAGGAGAGCCCGCAGCCTTTCTCTTG
CTCCATCGAAGACCCCACCAAACAGACAAAGTTTAAGGGCATCAAGACGT
ACATCTCGTACCGGGTGACGCCGAGCCACACGGCGCATCCCGTCTACCGG
CGCTACAAACACTTTGACTGGCTGTACAACCGCCTGCTGCACAAGTTCAC
TGTGATCTCCGTGCCCCACCTGCCCGAGAAGCAGGCCACGGGGCGCTTCG
AGGAGGACTTCATCGAGAAGCGCAAGAGGCGACTGGTCCTGTGGATGAAC
CACATGACCAGTCACCCGGTCCTCTCCCAGTACGAGGGCTTCGAGCACTT
TCTGATGTGCGCCGACGACAAGCAGTGGAAACTGGGCAAGAGGCGGGCGG
AGAAGGACGAGATGGTGGGCGCCCATTTCATGCTGACGCTGCAGATCCCC
AACGAGCACCAGGACCTCCAGGACGTGGAGGAACGGGTGGACACCTTCAA
GGCCTTCGCTAAGAAGATGGACGACAGCGTTTTGCAGCTCACACACGTGG
CCTCGGAGCTGGTGAGGAAGCACCTGGGCGGCTTCAGGAAGGAGTTCCAG
AGGCTGGGGAACGCCTTCCAGTCTGTCAGCCAGGCCTTCATGCTGGACCC
TCCACACAGNNNNNNNNNNNNNNNNNNNNNNNNNNNNNNNNNNNNNNNNNNNNNNNNNNNNNCTGACCTCT
CTGGGTTTCNTCATTGGAGTCGGTGTGGTTGGA
AACCTCCTGATMTCCATCCTGCTGGTCAAAGACAAGAGCCTGCACCGAGC
ACCCTACTATTTCCTGCTGGACCTGTGCGCCTCCGATATCCTCCGCTCTG
CCATCTGCTTCCCCTTTGTCTTCACCTCAGTCAAGAATGGATCTGCCTGG
ACCTATGGCACGCTGACYTGCAAAGTGATCGCCTTCCTGGGTGTGCTCTC
CTGTTTCCACACGGCGTTCATGCTATTCTGYGTGAGCGTCACCCGCTACC
TGGCCATYGCACATCATCGTTTCTACACCAAGAGRCTGACCTTCTGGACC
TGTCTGGCTGTCATCTGCATGGTGTGGACGTTGTCAGTWGCTATGGCGTT
CCCGCCGGTGCTAGACGTRGGGACATACTCTTTTATCCGGGAGGAGGACC
AGTGCACATTCCAGCACCGCTCCTTCAGGGCRAATGATTCGTTGGGCTTC
ATGCTCCTGCTGGCRCTCATTCTCCTGGCCACACAGCTGGTTTACCTCAA
GCTCATCTTCTTCGTCCACGACCGYCGAAAGATGAAGCCTGTCCAGTTCG
TGCCTGCTGTCAGCCAGAACTGGACCTTCCACGGGCCAGGCGCCAGCGGG
CAGGCGGCGGCYAACTGGCTGGCTGGATTTGGTAGAGGCCCCACCCCGCC
TACTTTRCTGGGCATCCGGCAGAACAGCAACGCAGCGGGCCGCAGGCGTC
TGCTGGTATTGGATGAATTCAAAACAGAGAAGAGGATTAGTAGGATGTTC
TACATCATGACGTTTTTCTTCCTGGCACTGTGGGGTCCCTATCTGGTAGC
CTGCTACTGGCGGGTGTTTGCAAGGGGCCCTGTAGTCCCTGGGGGCTACC
TGACGGCAGCCGTGTGGATGAGCTTTGCCCARGCTGGGGTCAATCCTTTC
ATCTNNNNNNNNNNNNNNNNNNNNNGCCAAATCTCGCTTTCACCCTGGCGTGGGGACCGGTCCTGGCACGG
AGC---GCAGCGTCCCACTCGGCAACAGCTTGCTCTCCCCGCAGCAAAGC
GAGGAGCCCACTGTTGCCACCCCCCCGCAGCGATGGTTTGTCACCC---C
TGCCAACAACCGACTGGACTTTGCTGCCTCGGCATACGACGCCGCT----
-----GATTTCGCCGGTAACGCGGCCACCTTGCTATCCTACGCAGCGGCC
GGAGTGAAAGCTC------TTCCGCTGCCGACTGCAGCCTGCTCCAACCG
GCCTCTTGGCTATTACGCAGACCCGTCGG---GCTGG---GGAGGACGCA
CGCCGCCGCAGTACTGTGGCGTCAACAGCAAACCCAGCTCGGTCTTTTCC
TGCTGGCCCGCCAACTCTATCGGTGGCCGAGCGGGCA---CCATGAACTA
CCTGG---------CCGAGGA---GGTA---GACTC---CATACCGACGG
AGAGGTCACCC---ATAGGCGGCGGCTCGGAGGAG---GCCAAACCCAAG
GACATGAC---GTCAGA---GTCGAGCTGGATAGAGACTACGCCGTCCTC
CATAAAGTCAATCGACTCCAGCGACTCTGGCATCTTTG---AACAGGCAA
AACGGAGAAGAATCTCACCTTCTGCCACGCCA---------GTTTCAGAG
ACAGTGTCCCCGTTAAAGTCGGAGNNNNNNNNNNACAGGCGAAGTCACGGAGAGAGAAGT
GGCGCTGGGGATAAATCCGTTCGCTGATGGGATGGGAGCCTTCAAAATAA
ACCACAGCTCCCACGATATTGGCTCCGG---ACAAACGGCGTTCTCCTCC

CAGGCG---CCCGGCTAC---GCAGCAGCCGCCCTGGGA---CACCACCA
------CCACCCGACCCACGTTGGCTCT---TACTCTACGGCGGCGTTCA
ACTCCACCAGGGACTTTCTCTTCAGAAATCGGGGTTTCGGGGATGCCACC
GG------------GGCGCAGCACAGTCTGTTCGCCTC---------CGG
AAGTTT---C------GCGGGGCCACATGGACACTCGGATGCGGCAGGGC
ACCTGCTCTTCCCGGGGCTCCACGAG---CAAGCGGCGAGCCATGCGTCT
TCCAACGTGGTCAACAGCCAGATGCGGCTGGGCTTCTCGGGGGACATGTA
CGGACGGGCCGACCAGTACGGCCACGTTACGAGCCCGCGCT---CCGACC
ACTACGCCTCGAGCCAGCTACACGGCTACGGCCCCATGAATATGAATATG
GCCGCA---CACCACGGAGCAGGGGCCTTCTTCCGATACATGAGGCAGCC
GATCAAGCAAGAGCTCATCTGCAAGTGGATCGAGCCGGAGCAGCTGTCGA
ATCCCAAAAAGTCGTGCAACAAAACTTTCAGCACGATGCACGAGCTCGTG
ACCCATCTGACGGTGGAGCATGTGGGGGACCGGAGCAGACAAACCACAT
CTGCTTCTGGGAGGAATGCGCCAGAGAAGGAAAGCCATTCCAAGCCAAAT
ACAAACTTGTAAATCATATCAGAGTGCACACCGGAGAGAAGCCCTTTCCA
TGTCCGTTCCCCGGCTGTGGNNNN
>Anarhichas lupas

------------TTCCTAGAGAGAAACCTTCACCCGTCTAATTGCCTTGG
CATGCTCCTGCTGTCTGATGCCCACCAGTGCACCAAGCTGTCAGAGCTCT
CCTGGGGCATGTGCCTCAGCAATTTCCCCGCTATTTGCAAGACAGAAGAG
TTCCTCCAACTGCCCAAAGATATGGTGGTGCAGCTTTTGTCACACGAGGA
GCTAGAGACAGAAGATGAGAAACTGGTTTATGAAGCTGCCCTTAACTGGA
TCAACTATGACCTGGAAAAGAGACACTGCCACCTTCCGGAGCTCCTGAGG
ACTGTCCGTCTGGCCCTGTTGCCTGCCATCTTTCTCATGGAGAATGTTTC
GACAGAAGAGCTGATCAATTCCCAGTCCAAGAGCAAGGAGCTGGTGGATG
AAGCTATCCGATGTAAGCTGAAGATCCTGCAGAATGACGGTGTAGTTAAC
AGCCCGTGTGCCCGACCAAGAAAGACCAGCCATGCCCTATTTCTTCTGGG
AGGGCAGACTTTCATGTGTGACAAGTTGTACCTTGTGGACCAGAAAGCCA
AAGAGATCATCCCCAAGGCTGACATTCCCAGCCCCAGGAAGGAGTTCAGT
GCCTGCGCCATCGGCTGTAAGGTGTACATCACTGGTGGGA--GAGGCTC-
AGAGAATGGTGTGTCCAAAGATGTGTGGGTCTACGACACCGTCCATGAGG
AATGGTCGAAGGCAGCGCCCATGCTCATTGCCAGGTTCGGCCATGGCTCT
GCAGAGCTGAAACACTGCCTCTACGTGGTAGGAGGTCACACGGCAGCAAC
TGGCTGCCTCCCAGCTTCTCCATCTGGATGATTACATCGTCGTGTTCAGT
CGCTCAACAACAAGGCTGATACTGAATGAGGCGGAGCTAATCATGGCGCT
GGCCCAGGAGTTCCAGATGAGAGTGGTCACAGTGTCCCTAGAGGAACAGT
CTTTCCCCAGTATCGTCCAGGTGATCAGCGGCGCTTCTGTGTTGGTCAGT

ATGCATGGCGCTCAGCTCATTACCTCACTCTTCCTCCCCAGAGGAGCTGC
TGTGGTGGAGCTGTTCCCCTTTGCTGTGAACCCAGAGCAGTACACCCCGT
ATAAAACCCTCGCCACCCTTCCAGGCATGGACCTTCACTATATCTCCTGG
CGGAACACTAAGGAGGAGAACACCATCACCCACCCAGACAGACCCTGGGA
GCAAGGGGGCATCACTCACTTGGAGAAGGAGGAGCAGGAGCGTATACTGG
AGAGCAGAGATGTCCCCAGGCACCTGTGCTGCCGCAACCCAGAGTGGCTC
TTCCGAATCTACCAGGACACTTTGGTGGACATCCCTTCCTTCCTGGAAGC
CCTTAA---AGAGGGCATGAAG---ACAAAGCCCAGCGTGAAGAA---GT
CAAAGCCGGCCAGCACAGTCCACCCGGGCCGGGTCAGACAACCCCAGTGT
CAGACCTCGGTACAAACCACCAACGAGGCTAAACTCACAGTCTCCTGGCA
GATCCCATGGAATCTGAAATACTTGAAGGTGAGAGAGGTGAAGTACGAAG
TG---------AAAAGAGACACTAGCAAGGGGACGCTGGAGGACCAGATC
ATCCAGGCCAACCCGGCGCTGGAGGCTTTCGGCAACGCCAAAACGCTGAG
AAACGACAACTCGTCTCGTTTTGGAAAATTTATCCGGATTCACTTCGGCA
TGAGCGGAAAGCTGTCGTCTGCCGACGTCGAGACTTACCTGCTGGAGAAG
TCCCGGTGCACCTTTCAGCTCAAGGCTGAGAGGAACTACCACATCTTCTA
CCAGATCCTGTCCAATCAGAAGCCAGAGCTGCTGGACATGCTGCTGATCA
CCAACAACCCTTACGATTACTCCTACATCTCCCAAGGGGAGGTAACGGTC
GCCTCCATCAACGACTCGGAGGAACTGCTGGCCACCGACAGTGCCTTCGA
TGTGCTCGGCTTCACTCAGGAGGAGAAGATGGGCGTCTACAAGCTAACTG
GTGCCATCATGCACTACGGCAACATGAAGTTCAAGCAGAAGCAGCGCGAG
GAGCAGGCCGAGTCGGACGGGACGGAGGCTGCCGATAAAACCGCATACCT
AATGGGGCTGAATTCTGCCGACGTTATCAAGGGGCTGTGTCATCCCAGAG
TCAAGGTCGGAAATGAATTCGTCACCAAAGGACAAAGTGTGGACCAAGTC
TACTACCCCAACAAGGAGGCCTTCAAGTGTGAGGAGTGCGGGAAGCACTA
CAACACCAAGCTGGGATATAAGCGCCATGTGGCCATGCACTCTGCCACGT
CAGGGGATCTCACCTGCAAAGTGTGCATGCAGAGCTATGAGAGCACACCC
GTGCTCTTGGAGCACCTCAAGACCCACTCGGGGAAGTCTTCGGGCGGCAC
CAAGGAGAAAAAGCACCCGTGCGACCACTGTGACCGTCGTTTCTACACAC
GCAAGGATGTGAGACGGCACATGGTGGTCCACACGGGTCGAAAGGACTTC
CTCTGCCAGTACTGTGCCCAGCGCTTTGGCAGGAAGGACCATCTGACACG
TCACGTGAAGAAGAGTCACTCGCAGGAGCTACTGAGGATCAAGACGGAGC
CTCCGGATATGTTGGGTCTTTTAGCGTCCGGGTCACCACCTTGCTCTGTG
AAGGAGGAGCTCAGCCCCATGATGTGCGGCATGGGTCCCAACAAAGACCC
CATGATGGGCAAACCGTTCCCCAGCGGGGCCCCTTTTCCGATGGGCATGT
ACAACCCCCACCAT------CTCCAGGCCATGTCTAATTCTGGGGTGGGT
CACCCG------CACCCGTCCCTGATGCCCAGTCCCTTGTCTGCAGCTAT
GGGCATGGGCTGCCACATGGAATATCTCATCTACGCCTCTTTTTCATTCA
TGGGATGTTTACAGATCAGTGATGGATCAAATATAGTGAACCTGCTGGCT
AGTAACTCTCCGAGTGTTTCCTACGCTCTGACCCAGCAGAAATACTTCAG
TAACTACAGTCCTGTGATTGGCTTCTACATTTATGAGCCCATCGAGTACT
GGAACTCCACGGTGCAGGAGCACCTGAAGACCCTGAGTCATGGCTTCAAC
AAGATCTCTTGGATGGACACCTTTTTCCACTACCTGCGTGTGGTGAATGT
GAGCGCGTCAACCAAGAGCGACTTCATCGCAATCCTTAAGGGCTCCTTCC
TTCGCAGCCCGGAGTACCAGCACTTCACTGAGGACATCATTTTCTCCATG
A---ACCGCGATACTG------ACGAGTACGACATTATCGCCTCTCGGAT
GTACCTGGTGGCACGGACGACAGAGAAGAAGCGAGAGGAGGTGGTGGAGC
TTCTGGAAAAGCTTCGTCCGTTGATGCTGATCAACAGCATCAAATGCATT
GCCTTCAATCCCACGTTTGTGTTTATGGACCGCTACAGCTCCTCTGTCAT
CTCGCCCATCCTGACCTCAGGCTTTAGCGTACTCACAATCCTCATCCTCA
CTTTCTTCCTGGTCATCAACCCATTGGGGAACTTCTGGCTCATTCTCACG
GTGACGTCCGTGGAGCTGGGCGTCTTGGGTTTGATGGGTTTTCACCAGTT
TGAGTGGCAGCCAGCTCTCAGGAATGTGTCTACATCTTGCAATGTTGGCA

TTATTAATGGGCTCTCTGGATGGGCTTCCTCGGTGGATGACATCCCAGCT
GACACCGTCACTCGACGGTTTCGCTACGATGTGGCTCTGGTGTCAGCATT
AAAGGATCTGGAGGAGGACATCATGGAGGGGCTGAGAGAGAGTGGGATGG
AAGACAGTGCTTGCACCGCAGGCTTCAGTGTCATGATCAAGGAATCCTGT
GATGGCATGGGCGATGTCAGCGAGAAGCACGGTGGAGGACCAGCTGTTCC
TGAGAAGGCTGTGCGTTTTTCTTTCACTGTTATGTCTGTCTCTGTCCTGG
CAGACGAGGGGGAG------------------------------GACAAG
GTTACCATCTTTACTGAGCCAAAGCCAAACTCAGAACTGTCCTGTAAGCC
CCTTTGCCTGATGTTTGTGGATGAGTCAGACCACGAGACACTCACAGCTG
TCCTGGGGCCTGTAGTTGCAGAGCGTAATGCAATGACAGAGAGCAGGCTC
ATTCTGTCCATGGGTGGACTACCTTGCTCCTTCCGCTTTCACTTCAGAGG
CACGGGATATGATGAGAAGATGGTGCGCGAGGTAGAGGGCCTGGAGGCCT
CAGGGTCCACCTACGTCTGCACTCTTTGTGACTCCACTCGGGCAGAGGCC
TCTCAAAACATGGTGCTACACTCCATCACTCGCAGCCATGAACAGAACCT
AGATCGTTACGAAATATGGAGAACCAACCCCTTTTCTGAGTCTGTAGATG
AGCTGCGGGACAGAGTCAAAGGGATCTCTGCCAAGCCCTTCATGGAGACC
CAGCCCACAATGGATGCACTACACTGTGACATTGGCAATGCCACTGAGTT
CTACAAAATCTTCCAGGACGAGATCGGGGAGGTGTACAAAAAGGT---CA
AA---CCCAGCCGGGAGGAACGGCGCAGCTGGAGGGCAGCCCTGGATAAA
CAGCTGAGGAAGACGGTGAAGCTTAAACCGGTGATGAGGATGAATGGGAA
CTATGCCCGCAGGCTAATGACCCAAGAGGCTGTCGAGGTTGTCTGTGAGC
TGGTGCCCTCAGAGGAGAGGAGGGAGGCCCTGAGGGAGCTGATGAGGATC
TACATCCAGATGAAGCCTGTGTGGCGAGCCACCTGCCCTGCCAAGGAGTG
CCCTGACCAGCTGTGCCGCTACAGCTTTAACTCCCAGCGCTTTGCCGACC
TTCTCTCCTCTACCTTCAAATACAGGTACAATGGAAAGATAACTAATTAC
CTGCACAAGACCCTGGCCCATGTGCCTGAAATCATAGAGAGAGATGGATC
CATAGGAGCCTGGGCCAGCGAGGGGAACGAGTCGGCAAACAAGTCGTACA
CCATCGAGATGGGCCCCGTATGGCCCCTGTGGAAGGAGAGCCCACAGCCT
TTCTCCTGCTCCATCGAGGACCCCACAAAACAGACAAAGTTCAAGGGCAT
CAAGACGTACATTTCGTACCGGGTCACGCCGAGCCACACGGGGCATCCCG
TCTACAGGCGCTACAAACACTTTGACTGGCTGTACAACCGCTTACTGCAC
AAGTTCACTGTGATCTCCGTGCCTCACCTGCCTGAGAAGCAGGCCACGGG
GCGATTTGAGGAGGACTTCATCGAGAAGCGCAAGAGACGGCTGATACTGT
GGATGAACCACATGACCAGCCACCCGGTCCTCTCCCAGTACGAGGGCTTC
GAGCACTTTCTGATGTGCGCTGACGACAAGCAGTGGAAACTGGGCAAGAG
GCGGGCGGAGAAGGACGAGATGGTGGGTGCCCATTTCATGCTGACCCTGC
AGATCCCCAACGAGCACCAGGACCTTCAGGATGTCGAGGAGCGGATCGAC
ACCTTCAAGGCCTTCGCCAAGAAAATGGACGACAGCGTGATGCAGCTCAC
GCACGTTGCCTCGGAGCTAGTGCGCAAGCACCTGGGCGGGTTCAGGAAGG
AGTTCCAGCGGCTGGGAAATTCTTTCCAGTCCATCAGCCAGGCATTCATG
CTGGACCCTCCCCACAGCTCGGAGGCCCTCAACAAAGCCATCTCCCATNNNNNNNNNNNNNNNNNNNNNN
NNNNNNNNNNNGG
TTTTATCATTGGAGTCGGTGTGGTTGGAAACCTCCTAATCTCCATCCTGC
TGGTCAAAGACAAGAGTCTGCACCGAGCGCCCTACTATTTCCTGTTGGAC
CTGTGCGCCTCTGACATCCTGCGCTCTGCCATCTGCTTCCCCTTTGTCTT
CACCTCGGTCAAGAATGGATCCACCTGGACCTATGGCACGCTGACCTGTA
AAGTGATCGCCTTCCTGGGTGTGCTCTCCTGTTTCCACACAGCGTTTATG
CTGTTCTGTGTCAGCGTCACACGCTACCTGGCCATCGCACACCACCGTTT
CTACACCAAGAGGCTGACCTTCTGGACCTGTCTAGCTGTCATCTGCATGG
TGTGGACGTTGTCGGTGGCTATGGCATTCCCGCCGGTGCTAGATGTAGGG
ACGTACTCTTTTATCCGGGAGGAGGACCAGTGCACATTCCAGCACCGTTC
CTTTAGGGCGAATGATTCGCTGGGCTTCATGCTCCTGCTGGCGCTCATCC
TCCTGGCCACGCAGCTGGTTTACCTCAAGCTCATCTTCTTCGTCCACGAC

CGTCGAAAGATGAAGCCTGTCCAGTTTGTGCCTGCTGTCAGCCAGAACTG
GACCTTCCACGGGCCTGGCGCCAGCGGGCAGGCGGCGGCCAACTGGCTGG
CCGGATTCGGTCGAGGCCCCACCCCGCCTACCTTGCTGGGCATCCGGCAG
AACAGCAACGCAGCGGGCCGCAGGCGTCTACTGGTGTTGGATGAATTCAA
AACAGAGAAGAGGATTAGCAGGATGTTCTACATCATGACGTTTTTCTTCC
TGGCGCTGTGGGGGCCCTATCTGGTCGCCTGCTACTGGCGGGTGTTTGCA
AGGGGCCCTGTGGTCCCTGGGGGCTACCTGACGGCCGCCGTGTGGATGAG
CTTTGCCCAGGCTGGGGTCAATCCTTTCATCTGCATCTTCTCCAACAGGNNN-

-------------------CATCACTCAACAGGCGAAGTCACAGAGAGAG
AAGTAGCGTTGGGGATCAATCCGTTCGCGGATGGGATGGGCGCCTTCAAA
ATAAACCACAGCTCCCACGATATCGGCTCCGG---ACAAACGGCGTTTTC
CTCCCAGGCG---CCCGGCTAC---GCGGCAGCCGCACTGGGA---CACC
ATCA------CCACCCGACCCACGTTGGCTCT---TACTCCACGGCGGCT
TTCAACTCCACCAGGGACTTTCTCTTCAGAAATCGGGGTTTCGGGGACGC
CACCGG------------GGCGCAGCACAGTTTGTTCGCCTC--------
-CGGAAGTTT---C------GCAGGGCCACATGGACACTCGGATGCAGCG
GGGCACCTGCTCTTCCCGGGGCTCCACGAG---CAAGCAGCGAGCCATGC
GTCTTCCAACGTGGTCAACAGCCAGATGCGGCTGGGCTTCTCGGGGGACA
TGTATGGACGGGCCGACCAGTACGGCCACGTTACGAGCCCACGGT---CC
GACCACTACGCGTCGACCCAGCTGCACGGCTATGGCCCCATGAACATGAA
TATGGCCGCG---CACCACGGAGCAGGGGCCTTCTTTCGATACATGAGGC
AGCCGATCAAGCAAGAGCTCATCTGCAAGTGGATCGAGCCGGAGCAGCTG
ACGAATCCCAAAAAGTCGTGCAACAAAACTTTTAGCACGATGCACGAGCT
CGTGACCCATCTGACGGTGGAGCATGTGGGGGGACCAGAGCAGACCAACC
ACATCTGCTTCTGGGAGGAATGCGTCAGAGAAGGAAAGCCATTCAAAGCC
AAATACAAACTTGTAAATCATATCAGAGTACACACCGGAGAAAAGCCCTT
TCCGTGTCCGTTCCCCGGCTGTGGCAAA
>Anguilla rostrata
AGCCTTCTAATCCGTGCCGAATTAAGTCAACCAGGCGCCCTTCTTGGAGA
TGACCAAATTTACAATGTCATCGTCACAGCGCATGCCTTTGTAATGATTT
TCTTTATAGTAATGCCAGTAATAATCGGAGGATTTGGCAACTGACTTGTG
CCATTAATAATCGGCGCTCCAGACATAGCATTCCCCCGAATAAATAATAT
AAGCTTCTGACTTTTACCCCCATCATTTCTTCTACTACTAGCCTCCTCTG
GAGTAGAGGCTGGGGCTGGTACAGGCTGAACTGTATATCCGCCCCTGGCT
GGAAACTTAGCCCACGCCGGAGCATCTGTTGACCTGACAATTTTCTCACT
TCACCTTGCAGGTATCTCATCAATTCTAGGGGCCATTAATTTTATTACTA
CAATTATTAACATGAAACCGCCTGCAATTACACAATACCAAACTCCCCTG
TTCGTATGAGCTGTATTAGTAACCGCTGTTCTGCTACTCCTCTCCCTGCC
AGTCCTAGCCGCAGGCATTACAATACTTCTAACTGACCGAAATCTAAATA

CAACCTTCTTTGACCCTGCAGGGGGTGGGGACCCAATCCTCTACCAACAC
TTANNNNNNNNNNNNNNNNNNNNNNNNNNNNNNNNNNNNNNNNNNNNNNNNNNNNNNNNNNNNNNNNNNN
NNNNNNNNNNNNNNNNNNNNNNNNNNNNNNNNNNNNNNNNNNNNNNNNNNNNNNNNNNNNNNNNNNNNNN
NNNNNNNNNNNNNNNNNNNNNNNNNNNNNNNNNNNNNNNNNNNNNNNNNNNNNNNNNNNNNNNNNNNNNN
NNTTACTGGAGAGGAACCTGCACCCCACCAACTGCCTGGGCATGCTGCT
GCTGTCGGACGCCCACCAGTGCGCCAAGCTGTCGGAGCTCTCCTGGGGCA
TGTGCCTGAGCAACTTCCCCACCATCTGCAAGACGGAGGACTTCCTGCAG
CTGCCCAAAGACATCCTGGTCCAGCTGCTTTCCCACGAGGAGCTGGAGAC
GGAGGACGAGAGGCTGGTGTACGAATCGGCCCTCAACTGGGTCAACTACG
ACCTGGAGAGGAGGTACTGCGACCTCCCCGAGCTGCTACGCACTGTCCGA
CTGGCCCTGCTGCCCGCCATCTTCCTCATGGAGAACGTCTCCACCGAGGA
GCTCATCAACAAGCAGGTGAAGAGCAAAGAGCTGGTGGACGAGGCCATCC
GGTGCAAGCTGAAGATCCTGCAGAACGACGGTGTGGTGACCAGCTTGTGC
GCCCGGCCCAGGAAGACCAGCCACGCCCTCTTCCTCTTGGGCGGTCAGAC
CTTCATGTGCGACAAGCTGTACCTGGTGGACCAGAAGGCCAAAGAGATCA
TCCCCAAGGCCGACATCCCCAGCCCTCGCAAGGAGTTCAGCGCCTGCGCC
ATCGGCTGCAAGGTGTACGTGACGGGGGGGC--GGGGCTC-CGAGAACGG
GGTGTCCAAAGACGTTTGGGTCTATGACACCCTGCACGAGGAGTGGTCCA
AGGCCGCGCCCATGCTCATCGCCAGGTTCGGACACGGCTCGGCCGAGCTC
AAGCACTGCCTCTACGTGGTGGGCGGGCACACGGCCGCCACCGGCTGCCT
GCCGGCCTCCCCGTCAGGACGAGTATATTGTGGTCTTCAGCCGCTCTCTT
AACAGGCTGATCCTGAACGAGGCGGAGCTGATCCTGGCACTAGCGCAGGA
GTTCCAGATGAAGGTCGTCACCGTGTCCCTGGAGGAGCAGTCCTTTGCGG
ACATCGTCCGAGTCCTCAGCAGGGCGTCCATGCTGGTCAGTATGCATGGG
GCCCAGCTGGTCACCTCTCTCTTCCTTCCTCGTGGCGCCGCTGTGGTGGA
GCTGTATCCATATGCCGTCAACCCAGAGCATTATGCTCCCTACAGGACAC
TGACCTCGCTGCCAGGTATGGACCTGCAGTATGTGGCCTGGAGGAACACC
AAGGAGGAGAACTCTGTGACCTTCCCAGAGCGTGCCTGGGATCAGGGTGG
CATTGCACACTTGGAGAAGGAGGAGCAGGAACGTATCATGAAGAGCAAGG
AGGTGCCGCGACACCTGTGCTGTCGGAACCCAGAGTGGCTTTTCCGCATC
TACCAGGATACCAAGGTGGACATTGCCTCCCTTCTGGATGCCCTGCA---
CCAGGGACTGGCC---TCCAGGCCAGGGCCCAAGAG---GGCTAGGCCTG
CCAGCACAGTCCACCCAGGCAGGGTGAGGGAGCCCAAGTGCCAGACCTCC
GTCCAGGCGACCAACGAGGCCAAGCTGACCGTGTCCTGGCAGATCCCCTG
GAACCTCAAGTACCTGAAGGTGAGGGAGGTGAAGTATGAGGTATGGATTC
AGAAGAGGGATACCAGCAAGGGAACACTGGAGGATCAAATTATCCAGGCA
AACCCAGCCCTGGAGGCTTTTGGCAATGCCAAGACTGTGAGAAATGACAA
CTCCTCACGTTTTGGGAAATTCATTCGCATTCATTTCGGAGTAAGTGGCA
AGCTGTCCTCTGCTGACATAGAAACCTACCTACTCGAGAAATCCCGTGTC
ACCTTTCAGCTCAAATCTGAGAGGAACTATCAYATTTTCTACCAGATCAC
ATCCAACAAAAAGCCAGAATTGCTGGACATGATGTTAATCACCAACAACC
CGTACGATTATGCTTATGTCTCCCAAGGAGAGGTGACGGTCGCATCCATC
GATGACTCAGAGGAACTGATCGCCACAGACAGTGCCTTTGATGTGCTGGG
CTTCACGCCCGAGGAGAAGATGGGCGTCTATAAGCTGACGGGTGCCATCA
TGCATTACGGAAACATGAAATTCAAGCAGAAGCAGCGTGAGGAGCAGGGC
GAGCCTGATGGCACTGAGTCTGCTGACAAGTCAGCCTACCTGATGGGGCT
GAACTCTGCTGACCTTCTCAAGGGACTCTGCCATCCACGGGTTAAAGTCG
GAAATGAGTATGTCACCAAAGGTCAAAATGTGGATCAAGTCTACTAT---

-------------TACCTGATCTACGCTTCGTTTTCGTTCATGGGATGTT
TACAAATCAGCGACGGGTCGAACATAGTCAACCTCCTGGCCAGCAACTCC
CCGAGCGCCTCCTACGCTCTGACCCAGCAGAAGTACTTCAGCAACTACAG
TCCGGTCATCGGGTTCTACATCTACGAACCCATCGAGTACTGGAACGCCA
CGGTTCAAGAGCACCTGAAGACGCTGGGCCACGGGTTCAATAAGATCTCG
TGGATTGACAACTTCTTTCAGTATCTGAAGGTGGTGAACGTGAGCGCGTC
GACCAAAAGCGAATTCATAACCATCCTCCAAACTTCTTTCTTAAGGAGCC
CAGAGTACCAGCACTTCAAGGACGACATCATATTTTACAAAA---TGAGG
G------------ACGAGACGGAGATAATCGCGTCCCGGATGTACCTGGT
GGCCCGGACCACGGAGAAGACGCGAGAGGAGGTGGTGGAGCTCCTGGAGA
GACTGAGRCCGCTCTCTCTCATCAACAGCATCAAATTCATCGTCTTCAAC
CCCACCTTYGTCTTCATGGATCGGTATAGCTCGTCGGTCATCTCCCCCAT
CCTCACCTCGGGCTTCAGCGTCCTGATCGTCCTGATCCTGACCTTCTTCC
TGGTCATCAACCCCCTGGGAAACTTCTGGCTGATACTGACGGTCACCTCG
GTGGAGCTGGGGGTCCTGGGCCTAATG-----------------------

----------------------------------TCCTACACTATCGAAA
TGGGGTCCTGGGGGCCCCAGTGGAAGGAGAGCCCCCAGCCGTTCTCCTGC
TCCATAGAGGATCCCACCAAGCAGACCAAGTTCAAGGGCATAAAGACCTA

```
CATCTCATATCGCGTGACGCCCAGCCACACAGGCCGGCCTGTGTACCGCC
GCTACAAGCATTTTGACTGGCTGTACAACCGACTGCTGCACAAGTTCACG
GTCATCTCTGTCCCCCACCTGCCCGAGAAGCAGGCCACAGGTCGCTTCGA
GGAGGACTTCATTGAGAAGCGCAAGCGGCGGCTCATCCTATGGATGGACC
ACATGACCAGCCATCCGGTGCTCTCGCAGTATGAGGGCTTTGAGCACTTC
TTGATGTGCGCCGACGACAAGCAGTGGAAGCTGGGAAAGCGGCGGGCAGA
GAAGGACGAGATGGTGGGCGCGCACTTCATGCTCACCTTCCAGATTCCCA
ATGAGCACCAGGACCTGCAGGACGTAGAGGAGAGGGTGGACTCCTTCAAG
GCCTTCGCCAAAAAGATGGACGACAGCGTGCTGCAGCTCACGCACGTGGC
CTCCGAGCTGGTGCGCAAGCATTTGGGAGGCTTCCGGAAGGAGTTCCAGA
AGCTGGGGAACGCCTTCCAGTCCATCAGCCAGGCATTCACTCTGGATCCT
CCCCACTGCTCTGATGCCCTCAACACCGCCATATCGCAC-----------
--------------------------------------------------
--------------------------------------------------
--------------------------------------------------
--------------------------------------------------
--------------------------------------------------
--------------------------------------------------
--------------------------------------------------
--------------------------------------------------
--------------------------------------------------
--------------------------------------------------
--------------------------------------------------
--------------------------------------------------
--------------------------------------------------
--------------------------------------------------
--------------------------------------------------
--------------------------------------------------
--------------------------------------------------
--------------------------------------------------
--------------------------------------------------
--------------------------------------------------
--------------------------------------------------
--------------------------------------------------
--------------------------------------------------
--------------------------------------------------
--------------------------------------------------
--------------------------------------------------
--------------------------------------------------
--------------------------------------------------
--------------------------------------------------
--------------------------------------------------
--------------------------------------------------
--------------------------------------------------
--------------------------------------------------
--------------------------------------------------
---------------------------------------GGTTAAATCCG
TTTGCTGACGGGATGGGAGCTTTCAAAATCAACCATGGTACTCACGAACT
TGGTTCTGG---ACAAACAGCTTTCTCTTCGCAGACT---CCGAGCTAC-
--GCAGCAGCCGCTTTGGGG---CATCACCA------CCACCCTGCTCAT
GTAAGCTCC---TACTCAACTGCAGCGTTCAACTCCACCAGGGACTTTCT
GTTCCGAAATCGGGGATTCGGTGACGCAGCAAG------------CGCAC
AACACAGTCTTTTTGCCTCAGC---GGCAGGAACTTT---C------GCA
```

GGGGCTCATGGACACTCAGATGCCACCGGACACCTTCTATTCCCGGGACT
GCACGAG---CAGGCAGCCACACATGGGTCTCCGAACGTTGTAAATAGTC
AGATGCGTCTGGGGTTTACCGGGGACTTGTACGGCAGAGCACAACACTAT
GGGCAGGCGACCAGTCCCAGGT---CTGAGCACTATGCATCGACACAGTT
GCACGGTTATGGTGCTATGAATATGAACATGGCTGCA---CACCACGGGG
CTGGGGCCTTTTTTCGATACATGAGGCAACCGATTAAACAAGAGCTTATC
TGTAAGTGGATTGAACCGGACCAGCTGTCGAATCCCAAAAAGTCTTGTAA
CAAAACTTTCAGCACCATGCACGAGCTCGTGACTCACCTCACAGTTGAGC
ATGTTGGGGGCCCAGAACAGTCAAATCACATCTGCTTTTGGGAAGAATGT
CCCCGGGAAGGCAAGCCATTTAAAGCTAAATACAAGCTAG----------

>Anomalops katoptron
AGCCTTCTCATCCGAGCTGAGCTTAGTCAACCTGGGGCACTCTTAGGAGA
TGACCAGATTTACAATGTAATTGTTACAGCACATGCCTTCGTAATAATTT
TCTTTATAGTAATGCCAATCATGATTGGTGGCTTTGGCAACTGATTGGTA
CCCCTAATGATTGGGGCTCCAGACATGGCATTCCCACGAATAAATAACAT
AAGCTTCTGACTTCTTCCCCCCTCTTTCCTTCTCCTTCTAGCCTCTTCCG
GAGTTGAAGCAGGTGCCGGAACCGGATGAACCGTCTACCCACCCCTTGCG
GGGAACCTCGCCCACGCAGGGGCCTCCGTAGACTTAACCATCTTCTCCCT
ACATCTTGCAGGTGTCTCTTCTATTCTGGGTGCCATTAATTTCATTACAA
CCATTATTAACATGAAGCCCCCTGCCATCTCCCAATACCAAACCCCACTA
TTTGTGTGAGCCGTATTAATTACAGCAGTCCTGCTTCTACTATCCCTTCC
AGTCCTTGCAGCCGGCATCACAATACTCCTCACAGACCGAAACTTAAATA
CAACCTTCTTTGACCCTGCAGGGGGAGGCGACCCCATTCTGTACCAACAC
CTG---

------------TTTCTAGAGAGAAACCTTCACCCATCTAACTGCCTGGG
GATGCTGTTGCTGTCTGATGCACACCAGTGTACCAAGCTATCAGAGCTCT
CCTGGGGCATGTGCCTCAGCAACTTTCCAGCTATTTGCAAGACCGAGGAC
TTCCTCCAGCTGCCCAAAGACATGGTGGTGCAGCTTCTATCCCATGAGGA
GCTGGAGACAGAAGATGARAGATTGGTTTATGAAGCTGCCCTTAACTGGG
TCAACTATGACCTGGAAAGGAGGCACTGCCACCTGCCAGAGCTGCTGAGA
ACGGTTCGCTTGGCCCTGCTGCCTGCCATCTTCCTCATGGAGAATGTCTC
TACAGAAGAGCTGATCAATGCCCAGGCCAAGAGCAAGGAGCTGGTGGATG
AGGCCATTCGGTGCAAGCTGAAGATCTTGCAGAATGACGGCGTGGTTAAC
AGCCCCTGTGCTCGGCCAAGAAAAACCAGCCATGCCCTTTTCCTATTGGG
AGGGCAGACCTTCATGTGTGACAAGTTGTATCTGGTGGACCAGAAGGCAA
AAGAGATCATCCCCAAGGCTGACATCCCCAGCCCTAGGAAGGAGTTCAGT
GCCTGCGCCATTGGCTGCAAGGTCTACATCACAGGTGGCA--GAGGCTC-
AGAAAATGGTGTATCTAAAGATGTATGGGTCTATGACACCATCCATGAGG
AATGGTCCAAGGCAGCACCCATGCTCATCGCCAGATTTGGTCATGGCTCT
GCTGAGCTGAAACACTGCCTTTACGTAGTAGGAGGTCACACAGCAGCAAC
TGGTTGCCTCCCAGCCTCTCCCTCCGGATGAATACATTGTAGTCTTCAGC
CGTTCAACAACAAGGCTGATTCTGAACGAAGCAGAGCTAATCATGACGCT
GGCCCAGGAATTTCAGATGAGAGTGGTTACGATCTCCCTGGAGGAACAAA
CTTTCCCCAGCATTGCCCAGGTAATCAGTGGGGCTTCCATGTTAGTCAGT
ATGCATGGAGCTCAGCTTGTCACTTCACTCTTCCTCCCCAGAGGAGCTGC
TGTGGTGGAGCTGTTCCCCTATGCTGTCAACCCAGAACAGTATACCCCAT
ATAAAACCCTCGCCTCCCTACCAGGCATGGACCTTCAATATGTTTCCTGG
AGGAACACTATAGAGGAGAACACTGTCACCCACCCAGACAGACCCTGGGA

ACAAGGAGGCATTGCCCATTTGGAAAAGGATGAGCAAGAGCGAATACTGG
CCAGCAATGATGTCCCCAGACACCTGTGCTGCCGCAATCCAGAGTGGCTC
TTCCGAATCTACCAGGACACTTTGGTGGACATCCCTTCATTCCTGGAGGT
CCTCAA---AGAGGGCCTGAAG---ACCAGGCCCAGCTTGAAGAA---GA
CCAAGCTGGCCAGCACGGTTCACCCAGGCCGGGTCAGAGAACCCCAGTGC
CACACCTCAGTCCAAGCCACCAATGAGGCTAAACTCACGGTTTCCTGGCA
GATCCCATGGAATCTGAAATACCTGAAGGTGAGAGAAGTGAAGTACGAGG
TG---------AAGAAAGATACCAGCAAAGGAACACTGGAGGATCAAATC
ATCCAGGCAAACCCTGCACTGGAGGCTTTTGGTAACGCCAAAACAGTGAG
GAATGACAATTCATCCCGTTTTGGAAAATTCATCCGAATTCACTTTGGAA
CCAGTGGCAAGCTGTCCTCTGCTGATATTGAAACTTACCTATTGGAGAAG
TCACGTGTCACCTTTCAACTCAAGGCTGAGAGGAACTACCACATCTTCTT
CCAGATATTGTCCAATCAGAAGCCAGAGCTGTTGGACATGATGTTGATCA
CTAACAACCCATATGACTACTCCTACATCTCCCAAGGAGAGGTAACAGTA
GCATCCATCAACGACTCAGAGGAGCTGATGGCCACTGACAGCGCTTTTGA
CGTCCTTGGTTTCACTCAAGAGGAGAAGATGGGAGTATATAAGCTGATTG
GTGCAGTTATGCACTATGGCAACATGAAGTTTAAGCAGAAGCAGCGTGAG
GAGCAGGCTGAGCCTGACGGAACAGAGGCTGCTGATAAGTCAGCTTACCT
TATGGGACTGAACTCGGCAGACCTCATCAAAGGGCTATGCCATCCCAGAG
TCAAGGTAGGAAATGAATATGTCACCAAAGGCCAAGGTGTAGACCAAGTC
TACTACCCAAACAAGGAGGCCTTCAAGTGCGAGGAGTGTGGCAAGCACTA
CAACACCAAGCTGGGATACAAACGCCATGTGGCCATGCACTCTGCCACAG
CAGGGGACCTTACCTGTAAAGTGTGCATGCAGAGCTATGAGAGCACGCCG
GTGCTCCTGGAGCACCTTAAGAGCCACTCGGGGAAGTCCTCGGGTGGCGC
CAAGGAGAAAAAACACCCATGCGACCACTGCGACCGCCGTTTCTACACAC
GAAAGGATGTGAGAAGGCACATGGTGGTCCACACGGGCCGAAAGGACTTC
CTGTGCCAGTACTGTGCCCAGCGCTTTGGCAGGAAGGATCACCTGACACG
CCACGTAAAGAAGAGCCACTCGCAGGAGCTGCTGAAGATCAAGACGGAGC
CTCCAGATATGTTAGGTCTTTTAGGTTCTGGCTCACCGCCATGCTCTGTC
AAGGAGGAGCTTAGCCCTATGATGTGCAGCATGGGTCCCAACAAAGACCC
CATGATGGGTAAACCCTTCCCCAGCGGGACCCCCTTCCCCATGGGTATGT
ACAACCCCCACCAC------CTCCAGGCCATGTCCAATTCTGGGGTGGGT
CACCCC------CACCCCTCCCTGATGCCTAGCTCCCTCTCTGCAGCTAT
GGGCATGGGCTGTCACATGGAATATCTCATCTATGCTTCTTTCTCATTCA
TGGGATGTTTACAAATCAGCGATGGCTCAAACATTGTGAACTTGCTGGCT
AGTAACTCTCCGAGTGTTTCATATGCTCTGACCCAGCAGAAATACTTTAG
TAATTACAGTCCTGTGATTGGATTTTACATTTACGAGCCCATTGAGTACT
GGAACTCCACAGTGCAGGAGCATCTGAAGACACTGAGTCATGGCTTCAAC
AAGATCTCCTGGATGGACAACTTCTTCCACTACCTGCGGGTGGTGAACGT
GAGCGCGTCGACCAAGAATGACTTTATCAGCATCCTAAAGGGCTCTTTCC
TGCGTAGCCCTGAGTACCAGCACTTCACTGAGGACATCATCTTCTCCAAG
A---ACCGTGAGAGTG------ATGAGTATGACATTATTGCCTCGCGCAT
GTACCTAGTGGCGCGGACCACAGAGAAGAAGCGCGAGGAGGTGGTGGAGC
TTCTGGAGAAACTGCGTCCACTGATGCTGATCAATAGCATCAAGTTCATC
GCCTTCAACCCCACCTTTGTTTTCATGGACCGCTACAGCTCCTCAGTCAT
CTCACCCATCCTGACCTCAGGCTTCAGCGTGCTCACCATCCTCATCCTCA
CTTTCTTCCTGGTCATCAACCCCTTGGGGAACTTCTGGCTCATCCTCACA
GTTACTTCTGTGGAGCTGGGTGTCTTGGGTTTAATGGGCTATCATGTATT
CGAATGGCAGCCGGCCCTCAAGAATGTGTCTGCATCCTGCCATGTGGGCA
TTATTAATGGTCTCTCTGGATGGGCTGCCTTGGTGGATGATACCCCAGTT
GACACCATTACTCGGCGGTTTCGCTACGATGTGGCCCTGGTATCAGCCTT
AAAGGATCTGGAGGAAGACATCATGGAGGGACTGAGAGAGAGTGGGCTTG
AAGACAATGCTTGCACCTTGGGCTTCAGTGTTATGATCAAGGAATCTTGT

GATGGCATGGGAGATGTCAGCGAGAAGCACGGGGGAGGGCCAATGGTCCC
CGAGAAGGCTGTACGCTTCTCTTTTACTGTTATGTCTGTCTCTGTCCTGG
CAGATGGAGAGGAA--------------------------------AAGGCA
GTTACCATCTTTAGCGAGCCAAAGCCCAACTCAGAACTGTCCTGCAAGCC
CCTAAGCCTGATATTTGTAGATGAGTCAGACCATGAGACTGTCACTGCTG
TCCTGGGACCTTTAGTTGCAGAGCGTAATGCAATGAAGCAGAGTCAACTC
ATCCTATCTATGGGTGGCCTCCCTCGCTCCTTCAGCTTCCACTTCAGAGC
CACAGGATATGATGAGAAGATGGTGCGTGAGATGGAGGGCCTGGAGGCCT
CAGGTTCCACCTATGTCTGCACTCTGTGTGATTCCAGTCGAGCAGAGGCC
TCTCACAACATGCTTCTACACTCCATCACCCGCAGCCACGATGAGAACCT
GGAGCGATATGAAATATGGAGGACCAATCCCTTTTCTGAGTCTGTTGAGG
AGCTGCGGGACCGGGTCAAAGGGATCTCTGCCAAGCCCTTTATGGAGACC
CAGCCCACTATGGATGCATTACACTGTGACATTGGTAATGCCACAGAGTT
CTACAAAATCTTCCAGGATGAGATTGGGGAAGTGTACCAGAAAGC---AA
AC---CCCACCCGGGAGGAACGGCGAAGCTGGCGAGCAGCCCTAGACAAG
CACCTGAGGAAGAAGATGAAGCTTAAACCAGTGATGAGGATGAATGGGAA
CTATGCCCGGAAGCTAATGACCCAGGAGGCTGTGGAGGTGGTGTGTGAGC
TGGTACCCTCAGAAGAGAGGCGAGAGGCCCTGAAGGAACTTATGGGGCTC
TACATCCAGATGAAGCCTGGGTGGCGCGCTACCTGCCCAGCCAAAGAATG
CCCTGATGAGCTGGGCCGCTACAGCTTTAACTCCCAGCGCTTTGCGAATC
TCCTTTCCACTACCTTCAAATATAGGTACAATGGAAGAATCACCAATTAC
CTGCANNNNNNNNNNNNNNNNNNNNNNNNNNNNNNNNNNNNNNNNNNNNNNNNNNNNNNNNNNNNN
NNNNNNNNNNNNNNNNNNNNNNNTCGTACACCATTGAGATGGCTCCAAAGGGGCCCCAATGGAAGGAG
AGTCCTCAGCCTTTCTCCTGCTCCATTGAAGACCCCACAAAACAGACCAA
GTTCAAAGGCATCAAGACTTACATTTCTTACCGGGTCACGCCGAGCCACA
CGGGGCGTCCTGTTTACAGACGCTACAAACACTTTGACTGGCTGTACAAC
CGCTTGTTGCACAAGTTCACTGTGATCTCGGTGCCCCACCTGCCTGAGAA
GCAGGCCACGGGGCGCTTCGAGGAGGACTTCATTGAGAAGCGTAAAAGAC
GACTGATTCTATGGATGAATCACATGACCAGTCACCCAGTCCTCTCCCAG
TATGAGGGGTTTGAGCACTTCCTTATGTGTGCTGACGACAAGCAATGGAA
GCTGGGTAAGAGGCGGGCGGAGAAGGACGAGATGGTGGGCGCCCACTTCA
TGCTGACCTTCCAGATTCCTAACGAGCATCAGGATCTCCAGGATGTGGAG
GAGCGGGTCGACTCCTTCAAGTCCTTTGCTAAGAAAATGGACGACAGCGT
CATGCAGCTCACACATGTTGCCTCGGAGCTGGTGCGTAAGCACCTGGGTG
GATTCAGGAAGGAGTTCCAGCGGCTGGGAAATGCCTTCCAGTCTATCAGC
CAGGCATTCATGCTGGAACCTCCCCACAGCTCCGATGCCCTCAACAACGC
CATCTCCCACCTCTCTCGCACGTTCCTCAAACTGACCTCTCTGGGTTTCA
TCATTGGAGTCGGTGTGGTCGGGAACCTCCTGATCTCCATCCTGCTGGTC
AAAGACAAGAGCCTGCACCGCGCACCCTACTACTTCCTGCTAGACCTGTG
CGCCTCCGACATCCTGCGCTCTGCCATCTGCTTCCCCTTCGTCTTCACCT
CTGTCAAGAATGGATCCGCCTGGACCTATGGCACGCTGACCTGCAAAGTG
ATCGCCTTCCTGGGTGTGCTCTCCTGTTTCCACACGGCGTTCATGCTATT
CTGTGTCAGTGTCACTCGCTACCTGGCTATTGCGCATCACCGCTTCTACA
CCAAGAGGCTGACCTTCTGGACCTGTCTAGCTGTCATCTGTATGGTTTGG
ACGCTGTCAGTAGCCATGGCTTTTCCCCCAGTGCTGGACGTAGGGACGTA
CTCCTTCATCCGGGAGGAGGACCAGTGCACATTCCAGCACCGTTCTTTCA
GGGCCAATGACTCACTGGGCTTCATGCTCCTGCTGGCACTCATCCTCCTG
GCCACACAGCTGGTTTACCTCAAGCTCATCTTCTTTGTCCACGACCGTCG
GAAGATGAAGCCCGTCCAGTTCGTGCCTGCCGTCAGCCAGAACTGGACCT
TCCATGGGCCAGGCGCCAGTGGGCAGGCGGCGGCTAACTGGCTCGCTGGA
TTTGGGAGAGGTCCTACCCCGCCTACCTTGCTGGGCATCCGGCAGAACAG
CAACGCGGCGGGCCGCAGGCGTCTGCTGGTGCTGGATGAGTTCAAAACAG
AGAAGAGGATTAGTAGGATGTTCTACATCATGACGTTTTTCTTCCTGGCG

TTGTGGGGGCCCTATCTGGTAGCCTGCTACTGGCGGGTGTTTGCAAGGGG
CCCCGTAGTCCCCGGGGGCTACCTGACGGCAGCCGTGTGGATGAGCTTTG
CCCAGGCTGGGGTCAACCCCTTCATCTGCATCTTCTCTAACAGGGAGGCC
AAAACTCGCTTTCACCCTGGCGTGGGGACTGGGCCTGGCACGGAGC---G
CAGCGTCCCACTCGGCAACAGCTTGCTATCCCCGCAGCAAACCGAGGAGC
CCACTGTTGCCACCCCCCCGCAGCGATGGTTTGTCACCC---CTGCCAAC
AACCGACTGGACTTTGCTGCCTCGGCATACGACGCGGCC---------GA
TTTTGCCGGTAACGCGGCCACCTTGCTGTCCTACGCAGCGGCCGGAGTGA
AGGCTC------TTCCTTTGCCGGCTGCAGGCTGCTCCAACCGGCCTCTT
GGCTATTACGCAGACCCGTCGG---GCTGG---GGCGGACGCACGCCGCC
GCAGTACTGTAGCGTAAACAGCAAACCGAGCTCGGTCCTTTCCTGCTGGC
CTTCTAATTCCGTCGGCGGCAGAACAGGCG---CC---AACTACCTGG--
-------CCGAGGA---CGGA---GACGC---CATCCCGACGGAGAGATC
CCCG---AT---CGGTGGCTCCGAGGAG---ACGAAACCCAAGGACCT--
----GTCGGA---GTCGAGCTGGATAGAG---ACGCCGTCCTCCATTAAG
TCAATTGATTCAAGCGATTCTGGTATCTTCG---AACAGGCCAAGAGGAG
AAGAATCTCGCCCTCTGCCACGCCG---------GTTTCAGAAACTGTGT
CCCCGTTAAAATCTNNNCATCACTCAACAGGCGAAGTCACAGAGAGAGAAGTG
GCGTTGGGGATAAATCCGTTCGCGGACGGAATGGGCGCCTTCAAAATCAA
CCACAGCTCCCACGATATTGGCTCCGG---ACAGACGGCGTTTTCCTCCC
AGGCG---CCCGGCTAC---GCAGCGGCCGCCCTGGGA---CACCACCA-
-----CCACCCGACCCACGTTGGCTCT---TACTCCACGGCAGCTTTCAA
CTCCACCCGGGACTTTCTCTTCAGAAATCGGGGTTTCGGAGACGCCACCG
G------------GGCGCAGCACAGTTTGTTCGCCTC---------CGGA
AGTTT---C------GCAGGGCCACATGGACACTCAGATGCCGCGGGACA
CCTGCTCTTCCCAGGACTCCACGAG---CAAGCAGCGAGCCACGCGTCTT
CTAATGTGGTCAACAGTCAGATGCGATTGGGCTTCTCGGGGGACATGTAC
GGTCGGGCAGACCAGTATGGCCACGTTACAAGCCCAAGGT---CCGACCA
CTATGCTTCGACCCAGTTGCACGGCTATGGCCCCATGAACATGAATATGG
CCGCA---CACCACGGAGCAGGGGCCTTCTTTAGATACATGAGGCAGCCG
ATCAAACAAGAGCTCATCTGCAAGTGGATCGAGCCGGAGCAACTGACGAA
TCCCAAAAAGTCGTGCAACAAAACTTTTAGTACGATGCATGAGCTGGTGA
CCCATCTGACGGTGGAGCATGTGGGGGGACCAGAGCAGTCCAACCACATC
TGCTTCTGGGAAGACTGCGCCAGAGAAGGGAAACCATTCAAAGCCAAATA
CAAACTTGTGAATCATATCAGAGTACACACCGGAGAGAAACCCTTCCCAT
GTCCGTTTCCCGGGTGTGGCAAA
>Anoplogaster cornuta
AGTTTGCTCATCCGTGCCGAACTTAGCCAACCCGGGGCACTCCTCGGGGA
CGACCAGATTTATAATGTTATTGTTACAGCACATGCCTTTGTAATAATTT
TCTTTATAGTAATACCAGTTATAATCGGAGGCTTTGGGAACTGACTTATT
CCCCTAATGATCGGGGCCCCTGACATAGCATTTCCTCGAATAAATAATAT
GAGTTTCTGACTCCTTCCCCCCTCCTTCCTACTTCTGCTGTCCTCCTCCG
GAGTTGAAGCAGGGGCCGGAACCGGGTGAACCGTCTACCCGCCCCTCGCA
GGAAACCTTGCCCACGCAGGAGCTTCCGTAGACCTAACCATCTTCTCCCT
TCACTTAGCGGGTATCTCCTCCATTCTAGGGGCCATTAACTTTATTACAA
CTATTATTAATATGAAACCCCCAGCCATCTCCCAATACCAAACCCCTTTA
TTTGTGTGGTCCGTTCTTATTACAGCAGTCCTCCTTCTCCTCTCCCTCCC
CGTCCTTGCAGCCGGCATCACCATGCTTCTGACAGATCGTAACCTTAATA
CAACCTTCTTTGACCCCGCAGGAGGGGGTGACCCCATCCTATATCAACAC
CTG---

------------TTTCTAGAGAGAAACCTTCATCCATCTAACTGCCTGGG
CATGCTGTTGCTGTCTGATGCACACCAGTGTACCAAGCTATCAGAGCTCT
CCTGGGGCATGTGCCTCAGCAACTTTCCAGCTATTTGCAAGACAGAGGAC
TTCCTCCAGCTGCCCAAAGACATGGTGGTGCAGCTTCTATCCCACGAGGA
GCTGGAGACAGAAGATGAGAGACTGGTTTATGAAGCTACCCTTAACTGGG
TCAACTATGACCTGGAAAGGAGGCACTGCCATCTGCCAGAGCTGCTGAGA
ACGGTTCGCCTGGCCCTGCTACCTGCCATCTTCCTCATGGAGAATGTCTC
TACAGAAGAACTGATCAATGCCCAGGCCAAGAGCAAGGAGCTGGTGGATG
AGGCCATTCGCTGCAAGCTGAAGATCTTGCAGAATGATGGTGTGGTTAAC
AGCCCCTGTGCTCGGCCGAGAAAAACCAGCCATGCCCTTTTTCTGTTGGG
AGGGCAGACCTTCATGTGTGACAAGCTGTACCTGGTGGACCAGAAGGCAA
AAGAGATCATCCCCAAGGCTGACATCCCCAGCCCTAGGAAGGAGTTCAGT
GCCTGCGCCATCGGCTGTAAGGTCTACATCACCGGTGGCA--GAGGCTC-
AGAAAACGGTGTGTCTAAAGATGTATGGGTCTATGACACCGTCCATGAGG
AATGGTCCAAGGCGGCACCTATGCTCATTGCCAGATTTGGCCACGGCTCT
GCTGAGCTGAAACACTGCCTTTACGTAGTAGGAGGTCACACGGCAGCAAC
TGGCTGCCTCCCAGCCTCTCCCTCCGGATGAATACATTGTAGTGTTCAGC
CGTTCAACAACAAGGCTGATTCTGAACGAAGCGGAGCTAATCATGAGGCT
GGCCCAGGAATTTCAGATGAGAGTGGTTACGGTCTCCCTGGAGGAACAAA
CTTTCCCCAGCATCGTCCAGATGATCAGTGGGGCCTCCRTGTTAGTCAGC
ATGCATGGAGCTCAGCTTGTCACCTCACTCTTCCTCCCCAGAGGAGCTGC
TGTAGTGGAGCTGTTCCCTTATGCTGTCAACCCAGAACAGTACACCCCAT
ATAAAACCCTCGCCTCCCTACCAGGCATGGACCTTCATTATGTTTCCTGG
AGGAACACTATAGAGGAGAACACTGTCACCCACCCAGACAGACCCTGGGA
AGAAGGAGGCATCGCCCATTTGGAAAAGGACGAGCAAGAGCGAATACTGG
CCAGCAAGGATGTCCCCAGACACCTGTGCTGCCGCAACCCAGAGTGGCTC
TTCCGAATCTACCAGGACACTTTGGTGGACATCCCTTCATTSCTGGAGGT
CCTCAA---AGAGGGCCTGAAG---ACCAGGCCCAGCTTGAAGAA---GG
CCAAGCTGGCCAGCACGGTTCATCCAGGCCGGGTCAGAGAACCCCAGTGC
CACACCTCAGTCCAAGCCACCAACGAGGCTAAACTCCTGGTTTTCTGGCA
GATACCATGGAATCTGAAATACCTGAAGGTGAGAGAAGTGAAGTACGAGG
TG---------AAGAAGGATACCAGCAAGGGGACACTGGAGGATCAAATC
ATCCAGGCAAACCCTGCACTGGAGGCTTTTGGTAACGCCAAAACACTGAG
GAATGACAACTCATCCCGTTTTGGAAAATTCATCCGAATTCACTTCGGAA
CCAGTGGCAAGCTGTCCTCTGCTGATATTGAAACGTACCTATTGGAGAAG
TCACGTGTCACCTTTCAGCTCAAGGCTGAGAGGAACTATCACATCTTCTT
CCAGATATTGTCCAATCACAAGCCAGAGCTGTTGGACATGATGTTGCTCA
CTAACAACCCATATGACTACTCCTTCATCTCCCAAGGAGAGGTAACAGTA
GCATCCATCAATGACGCAGAGGAACTGATAGCCACTGACAGCGCCTTTGA
CGTGCTTGGCTTTACTCAAGAGGAGAAGATGGGAGTATATAAGCTGATTG
GTGCAATTATGCACTATGGCAACATGAGGTTTAAGCAGAAGCAGCGTGAG
GAGCAGGCTGAGCCTGACGGAACAGAGGCTGCTGATAAGTCAGCTTACCT
TATGGGGCTGAACTCTGCAGACCTCATCAAAGGGCTATGCCATCCCAGAG
TCAAGGTAGGAAATGAATATGTCACCAAAGGCCAAGGTGTAGACCAAGTC
TACTACCCCAACAAGGAGGCCTTCAAGTGCGAGGAGTGTGGCAAGCACTA
CAACACCAAGCTGGGATACAAACGCCATGTGGCCATGCACTCTGCCACCG
CGGGCGACCTCACCTGCAAAGTGTGCATGCAGAGCTACGAGAGCACGCCG
GTGCTSCTGGAGCACCTCAAGAGCCACTCGGGGAAGTCCTCNGGCGGCGCC
AAGGAGAAGAAACACCCGTGCGACCACTGCGACCGCCGCTTCTACACGCG
GAAGGACGTGAGGCGGCACATGGTGGTCCACACGGGCCGGAAGGACTTCC
TGTGCCAGTACTGTGCCCAGCGCTTCGGCAGGAAGGACCACCTGACGCGC
CACGTGAAGAAGAGCCACTCGCAGGAGCTGCTGAAGATCAAGACGGAGCC
TCCGGACATGTTGGGGCTTTTAGGTTCTGGCTCGCCGCCGTGCTCTGTCA

AGGAGGAGCTCAGCCCCATGATGTGCAGCATGGGTCCCAACAAAGACCCC
ATGATGGGCAAACCCTTCCMCAGYGGGACCCCCTTCCCCATGGGCATGTA
CAACCCCCACCAC------CTCCAGGCCATGTCCAACTCTGGGGTGGGTC
ACCCC------CACCCCTCCCTGATGCCCAGCTCCCTGTCTGCAGCTATG
GGCATGGGCTGCCACATGGAGNNNNNNNNNNNNNNNNNNNNNNNNNNNNNNNNNTGTTTACAGATCAGTG
ATGGCTCAAACAT
TGTGAACTTGCTGGCTAGTAACTCTCCGAGCGTTTCATATGCTCTGACCC
AGCAGAAATACTTCAGTAACTACAGTCCCGTGATTGGRTTTTACATTTAC
GAGCCCATTGAGTACTGGAACTCCACGGTGCAGGAGCACCTGAAGACACT
GAGTCATGGCTTCAACAAGATCTCCTGGATGGACAACTTCTTCCACTACC
TGCGGGTGGTGAACGTGAGCGCGTCGACCAAGAGCGACTTCATCACCATC
CTCAAGGGCTCCTTCCTGCGCAGCCCAGAGTACCAGCACTTCACTGAGGA
CATCATCTTCTCCAAGA---ACCGTGAGAGTG------ATGAGTACGACA
TTATYGCCTCGCGCATGTACCTGGTGGCGCGGACCACAGAGAAGAAGCGC
GAGGAGGTGGTGGAGCTTCTGGAGAAGCTGCGTCCGCTGATGCTGATCAA
CAGCATCAAGTTCATCGCCTTCAACCCCACCTTTGTTTTCATGGACCGCT
ACAGCTCCTCGGTCATCTCACCCATCCTGACCTCAGGCTTCAGCGTGCTC
ACCATCCTCATCCTCACTTTCTTCCTGGTCATCAACCCCTTGGNNNNNNNNNNNNNNNNNNNNNNNNNNN
NNNNNNNNNNNNNNNNNNNNNNNNNNNNNNNNNNNNNNNNNNNNNNNNNNNNNNNNNNNNNNNNNNNNNN
NNNNNNNNNNNNNTGCCATG
TGGGCATTATTAATGGGCTCTCTGGGTGGGCTGCCTTGGTGGATGACTCC
CCGGCTGACACCATTGCTCGACGGTTTCGCTACGACGTGGCCCTGGTATC
AGCATTAAAGGATCTGGAGGAGCACATAATGGAGGGACTGAAAGACTGTG
GGCTGGAAGACAGCTCTTGCACCTCAGGCTTCAGTGTTATGATCAAGGAA
TCTTGTGACGGCATGGGAGATGTCAGCGAGAAGCACGGGGGAGGGCCGGT
GGTCCCCGAGAAGGCTGTACGCTTCTCTATTACTGTTATGTCTGTCTCTG
TCCTGGCAGATGGAGAGGAA------------------------------
GAGGCGGTTACCATCTTCAGCGAGCCAAAGCCCAACTCAGAACTGTCCTG
TAAGCCCCTAAGCCTGATGTTTGTGGATGAGTCAGACCATGAGACAGTCA
CTGCTATCCTGGGACCTTTAGTTGCAGAGCGTAACGCAATGAAGCAGAGC
CAACTCATCCTATCTATGGGTGGCCTCCCTCGCTCCTTCCGCTTCCACTT
CAGAGCCACGGGATWTGATGAGAAGATGGTGCGTGACATGGAGGGCCTGG
AGGCCTCAGGTTCCGCCTATGTCTGCACTCTGTGTGATTCCAGTAGAGCA
GAAGCCTCTCACAACATGACGCTGCACTCCATCACACGCAACCACGATGA
GAACCTGGAGCGTTATGAAATATGGAGGAGCAATCCCTTTTCTGAGTCTG
TTGAGGAGCTGCGAGACCGGGTCAAAGGGATCTCTGCCAAGCCCTTTATG
GAGACCCAGCCCACTATCGATGCATTACACTGTGACATTGGTAATGCCAC
AGAGTTCTAYAAAATCTTCCAGGATGAGATTGGGGAGGTGTACCGGAAGG
C---CAAC---CCCACCCGGGAGGAACGGCGGAGCTGGCGAGCAGCCCTG
GACAAGCACCTGAGGAAGAAGATGAAGCTTAAACCAGTGATGAGGATGAA
TGGGAACTATGCCCGGAGGCTAATGACCCAGGAGGCTGTGGAGGTGGTGT
GTGAGCTGGTACCCTCAGAAGAGAGGCGAAAGGCCCTGACGGAGCTTATG
GGGCTCTACATCCAGNNNNNNNNNNNNNNNNNNNNNNNNNNNNNNNNNNNNNNNNNNNNNNNNNNNNNN
NNNNNNNNNNNNNNNNNNNNNNNNNNNNNNNNNNNNNNNNNNNNNNNNNNNNNNNNNNNNNNNNNNNNNN
NNNNNNNNNNNNNNNNNNNNNNNNNNNNNNNNNNNNNNNNNNNNNNNNNNNNNNNNNNNNNNNNNNNNNN
NNNNNNNNNNNNNNNNNNNNNNNNNNNNNNNNNNNNNNNTCGTACACCATTGAGATGGCTCCAAAGGGGC
CCCA
GTGGAAGGAGAGTCCTCAGCCTTTCTCCTGCTCCATTGAAGACCCCACAA
AACAGACCAAGTTCAAAGGCATCAAGACCTACATTTCGTACCGGGTCACA
CCGAGCCACACAGGGCGTCCGGTCTACAGACGCTACAAACACTTTGACTG
GCTGTACAACCGCTTGCTGCACAAGTTCACTGTGATCTCGGTGCCCCACC
TGCCGGAGAAGCAGGCCACGGGGCGCTTCGAGGAGGACTTCATCGAGAAG
CGCAAAAGACGWCTGATYCTGTGGATGAACCACATGACCAGTCACCCGGT

CCTCTCCCAGTACGAGGGATTTGAGCACTTCCTTATGTGTGCTGACGACA
AGCAGTGGAAGCTGGGCAAGAGGCGGGCGGAGAAGGACGAGATGGTGGGC
GCCCACTTCATGCTGACCTTCCAGATTCCTAACGAGCATCAGGACCTCCA
GGATGTGGAGGAGCGGGTTGACTCCTTCAAGTCCTTTGCTAAGAAAATGG
ACGACAGCGTCATGCAGCTCACACATGTTGCCTCGGAGCTGGTGCGTAAG
CACCTGGGTGGATTCAGGAAGGAGTTCCAGCGGCTAGGAAACGCCTTCCA
GTCCATCAGCCAGGCATTCATGCTGGAACCTCCCCACAGCTCCGATGCCC
TCAACAACGCCATCTCCCACNTCTCTCGCACGTTCCTCAAACTGACCTCGC
TGGGTTTCATCATTGGCGTCGGCGTGGTCGGGAACCTCCTGATCTCCATC
CTGCTGGTCAAAGACAAGAGCCTGCACCGTGTGCCCTACTACTTCCTGCT
GGACCTGTGCGCCTCTGACATCCTGCGCTCTGCCATCTGCTTCCCCTTTG
TCTTCACCTCTGTCAAGAATGGATCCGCCTGGACCTATGGCACGCTCACC
TGCAAAGTGATCGCCTTCCTCGGCGTGCTCTCCTGTTTCCACACGGCGTT
CATGCTATTCTGTGTCAGTGTCACTCGCTACCTGGCGATTGCGCATCACC
GCTTCTACACCAAGAGGCTGACCTTCTGGACCTGTCTAGCTGTCATCTGC
ATGGTGTGGACGTTGTCAGTAGCCATGGCTTTCCCCCCTGTGCTGGACGT
AGGGACGTACTCCTTCATCCGGGAGGAGGACCAGTGCACATTCCAGCACC
GTTCTTTCAGGGCCAATGACTCACTGGGCTTCATGCTCCTGCTGGCACTC
ATCCTCCTGGCCACACAGCTGGTTTACCTCAAGCTCATCTTCTTTGTCCA
CGACCGTCGGAAGATGAAGCCCGTCCAGTTCGTGCCCGCCGTCAGCCAGA
ACTGGACCTTCCATGGGCCGGGAGCCAGTGGGCAGGCGGCGGCTAACTGG
CTCGCTGGATTTGGGAGAGGCCCCACCCCGCCTACCTTGCTGGGCATCCG
GCAGAACAGCAACGCGGCAGGCCGCAGGCGTCTGCTGGTGCTGGATGAGT
TCAAAACAGAGAAGCGGATTAGTAGGATGTTCTACATCATGACGTTTTTC
TTCCTGGCGTTGTGGGGGCCCTATCTGGTAGCCTGCTACTGGCGGGTGTT
CGCAAGGGGCCCCGTAGTCCCCGGGGGCTACCTGACGGCAGCCGTGTGGA
TGAGCTTTGCCCAGGCTGGGGTCAATCCCTTCATCTGCATCTTCTCGAAC
AGGGAG--

NNNNNNNNNNNNNNNNNNNNNNNNNNNNNNNNNNNNNNNNNNNNNNNNNNNNNNNNNNNNNNNNNNNNNN
NNNNNNNNNNNNNNNNNNNNNNNNNNNNNNNNNNNNNNNNNNN---NNNGACGGCGTTCTCCTCGCAGGC
T---CCCGGCTAC---GCAGCGGCCGCCCTGGA---CACCATCA------
-CCACCCGACCCACGTCGGCTCTGCCTACTCCACGGCGGCTTTCAACTCC
ACGCGGGACTTTCTCTTCCGAAATCGGGGTTTCGGAGACGCCGCCGG---
---------GGCGCAGCACAGCTTGTTCGCCTC---------CGGGAGTT
T---C------GCAGGGCCACATGGGCACTCTGATGCGGCGGGGCACCTG
CTCTTCCCGGGGCTCCACGAG---CAAGCGGCGAGCCACGCGTCTTCCAA
CGTGGTCAACAGCCAGATGCGACTGGGCTTCTCGGGGGACATGTACGGTC
GGGCAGATCAGTACGGCCACGTTACAAGCCCGAGGT---CCGACCACTAT
GCTTCGACCCAGTTGCACGGCTATGGTCCCATGAACATGAATATGGCCGC

A---CACCATGGAGCAGGGGCCTTCTTTCGATACATGAGGCAGCCGATCA
AGCAAGAGCTCATCTGCAAGTGGATCGAGCCGGAGCAGCTGTCGAACCCC
AAAAAGTCGTGCAACAAAACTTTTAGCACGATGCACGAGCTGGTGACCCA
TCTGACGGTGGAGCATGTGGGGGGGCCAGAGCAGTCCAACCACATCTGCT
TCTGGGAAGACTGCGCCAGAGAAGGGAAGCCGTTCAAAGCCAAATACAAA
CTTGTGAATCACATCAGGGTACACACCGGAGAAAAGCCCTTCCCGTGTCG
GTTTCCCGGNNNNNNNNNN
>Anotopterus pharao
AGCCTTTTAATCCGGGCAGAACTTAGCCAACCCGGGGCCCTTTTAGGGGA
TGATCAAATTTATAATGTAATCGTCACTGCCCACGCCTTCGTAATAATCT
TCTTTATAGTAATGCCTATTATAATCGGCGGTTTCGGAAATTGATTAGTT
CCATTAATAATCGGAGCCCCTGATATAGCCTTCCCCCGAATAAATAATAT
AAGCTTCTGACTACTCCCCCCATCTTTTCTTCTTTTATTATCCTCCTCAG
CAGTAGAAGCAGGGGCCGGCACGGGTTGAACTGTTTATCCTCCCCTTGCT
AGTAACCTAGCACATGCTGGAGCTTCAGTTGACTTAACTATTTTTTCTCT
TCACTTGGCAGGAATCTCTTCTATTTTAGGGGCCATTAACTTTATTACAA
CTATTATCAATATAAAACCTCCAGCTATTACACAGTATCAAACCCCTTTA
TTCGTCTGATCAGTCTTAATTACTGCAGTACTTCTACTTCTTGCCCTCCC
TGTTTTAGCAGCAGGAATTACTATACTCCTGACAGACCGAAATTTAAATA
CCACCTTTTTTGACCCAGCAGGTGGAGGAGACCCGATTCTTTATCAACAC
TTANNNNNNNNNNNNNNNNNNNNNNNNNNNNNNNNNNNNNNNNNNNNNNNNNNNNNNNNNNNNNNNN
NNNNNNNNNNNNNNNNNNNNNNNNNNNNNNNNNNNNNNNNNNNNNNNNNNNNNNNNNNNNNNNNNNN
NNNNNNNNNNNNNNNNNNNNNNNNNNNNNNNNNNNNNNNNNNNNNNNNNNNNNNNNNNNNNNNNNNN
NNNNNNNNNNNNNNNNNNNNNTCCGTCCAACTGCCTTGGCATGCTGCTGCTGTCGGATGCCCACCAGT
GCACCAAGCTGTCAGAGCTGTCCTGGGGTATGTGCCTCAGCAACTTCCCT
GCCATCTGCAAGACGGAGGACTTCCTCCAGCTGCCCAAAGACATGGTGGT
GCAGCTCCTGTCCCATGAAGAGCTGGAGACGGAAGATGAGCGACTGGTTT
ATGAGGCTGCCCTTAACTGGGTCAACTATGACCTGGAGAGGAGACACTGC
CACCTACCAGAGCTGCTGAGAACCGTACGCCTGGCCTTGCTTCCCGCCAT
CTTCCTCATGGAGAACGTCTCCACGGAGGAGCTGATCAATGCCCAGGCAA
AGAGCAAGGAATTGGTGGACGAGGCCATCCGCTGCAAGCTGAAGATCCTG
CAGAACGACGGCGTGGTCAACAGTCCCTGTGCCCGGCCCAGAAAGACCAG
CCATGCCCTTTTCCTGCTGGGAGGGCAGACCTTCATGTGTGACAAGCTGT
ACTTGGTAGACCAGAAGGCCAAAGAGATCATCCCCAAGGCAGACATCCCC
AGCCCCAGGAAGGAGTTCAGCGCCTGCGCCATTGGCTGCAAGGTCTACGT
CACGGGCGGGA--GAGGCTC-TGAGAACGGCGTGTCTAAAGACGTGTGGG
TCTATGACACCGTCCACGAGGAGTGGTCCAAGGCGGCGCCCATGCTCATC
GCCAGGTTTGGCCACGGCTCCGCCGAGCTCAAACACTGCCTCTATGTGGT
CGGAGGACACACGGCAGCCACCGGCTGCCTCCCGGCCTCTCCGTCCGGAC
GAGTACATCGTGGTGTTCAGTCGGTCCACGACGCGGCTGATTCTGAACGA
AGCAGAGCTGATCCTGGCGCTGGCCCAGGAGTTCCAGATGAGGGTGGTCA
CCGTGTCCCTGGAGGAGCAGTCCTTCCTCGGCATTGTGCAGGCCATCAGC
GGGGCCTCCATGTTGGTCAGCATGCACGGAGCTCAGCTCGTCACCTCGCT
CTTCCTCCCCAGAGGAGCTGCCGTGGTGGAGCTCTTCCCCTATGCCGTGA
ACCCGGAGCAGTACACCCCGTACAAAACCCTTGCCTCCCTGCCAGGCATG
GACCTCCAGTACGTCTCCTGGAGGAACACTATTGAGGAGAACACCGTCTC
CCACCCGGACAGACCCTGGGACCAAGGAGGCATCGCCCACTTGGAGAAAG
ACGAGCAGGAGAGAATCCTAGCCAGCGAGGACGTCCCCAGGCACCTGTGC
TGCCGCAACCCAGAGTGGCTCTTCAGGATCTACCAGGACACGCTGGTGGA
CATCCCCTCCTTCCTGGACATCCTCAA---GGAGGGCCTGAAG---ACAA
GGCCAACCTTGAAGAA---GTCCAAGCCGGCCAGCACGGTTCACCCGGGC
CGGGTCCGGGAACCCCAGTGCCAGACTTCGGTTCAAGCCACCAACGAGGC
TAAGCTCACAGTGTCCTGGCAGATCCCGTGGAACCTCAAGTACCTGAAGG

TGCGAGA-------------------------AAAAGGGACGCGAGCAAG
GGAACCCTGGAGGATCAAATCATTCAGGCAAACCCCGCACTGGAGGCTTT
CGGTAATGCCAAAACATTGAGGAACGATAATTCCTCCCGTTTTGGAAAAT
TCATCCGAATCCACTTTGGAACCAGTGGTAAGCTGTCTTCTGCGGACATT
GAGACCTACCTGCTGGAGAAGTCGCGGGTCACCTTTCAGCTTAAGGCGGA
GAGGAATTACCACATCTTCTTCCAGATTTTGTCCAATCAAAAGCCGGAGC
TGCTGGACATGCTGTTGATCACCAACAACCCCTATGACTACTGCTACATC
TGCCAAGGAGAAGTAACCGTAGCATCCATCAACGATTCAGAAGAGCTGAT
GGCTACCGACAGTGCCTTCGACGTTCTTGGCTTCACGCAAGAGGAGAAAC
TGGGAGTCTACAAGTTGATAGGGGCCATTATGCACTATGGCAACATGAGG
TTCAAGCAAAAGCAGCGCGAGGAACAGGCTGAGTCAGACGGTACAGAGGC
AGCTGATAAGTCAGCTTATCTAATGGGGCTGAATTCAGCAGACCTAATCA
AGGGACTCTGCCATCCCAGAGTCAAGGTAGGGAATGAGTATGTCACCAAA
GGCCAGGGTGTAGATCAAGTCTATTACCCCAACAAGGAGGCCTTCAAGTG
TGAAGAGTGCGGCAAGCACTACAACACCAAGCTGGGCTACAAGCGCCATG
TGGCCATGCACTCGGCCACGGCGGGGGACCTCACCTGCAAGGTGTGCATG
CAGAGCTACGAGAGCACGCCGGTGCTCCTGGAGCACCTCAAGAGCCACTC
CGGGAAGTCCTCGGGCGGCGCCAAGGAGAAGAAGCATCCATGCGACCACT
GCGACCGCCGCTTCTACACGCGGAAGGACGTCAGGCGGCACATGGTCGTC
CACACAGGCCGAAAGGACTTCCTGTGCCAGTACTGTGCCCAGCGCTTCGG
CAGGAAGGATCACCTGACGCGGCACGTGAAGAAGAGCCACTCGCAGGAGC
TGCTGAAGATCAAGACGGAGCCTCCGGATCTGTTGGGCCTCCTGGGCTCC
GGCTCGCCGCCTTGCTCTGTCAAGGAGGAGCTAAGCCCCATGATGTGCAG
CATGGGTCCCAATAAAGACCCCATGATGGGCAAACCCTTCCCCAGCGGGA
CCCCCTTCCCCATGGGCATGTACAACCCCCACCAC------CTCCAGACC
ATGTCTAATCCCGGGGTGGGCCACCCC------CACCCCTCCCTTATGCC
CGGTTCCCTGTCTGCAGCTATGGGCATGGGCTGTCATATGGAATACCT-A
TCTACGCTTCCTTCTCCTTCATGGGGTGTTTACAGATCAGCGACGGGTCG
AACGTGGTGAACCTGCTGGCCAGCAACTCTCCGAGCGTCTCGTACGCGCT
CACGCAGCAGAAGTACTTCAGCAACTACAGCCCCGTGATCGGCTTCTACA
TCTACGAGCCCATCGAGTACTGGAACTCCACGGTGCAGGAGCACCTGCGG
ACGCTGAGCCACGGCTTCAACAAGATCTCCTGGGTGGACAACTTCGCCCA
ATACCTGCGGGCGGTGAACCTGAGCGCCTCCACCAAGGCCGACTTCGTCG
CCGTCCTCAAGGGCTCCTTCCTGCGGAGCCCTGTATACCAGCACTTCACC
GAGGACATCATCTTCTCCAAGA---GCCACGAGAACA------ACGAGTA
CGACATCATCGCCTCGCGGATGTACCTGGTGGCACGCACCACCGAGAAGC
GGCGAGAGGACGTGGTGGAGCTGCTGGAGAAGCTCCGGCCGCTGATGCTG
ATCAACAGCATCAAGTTCATCGCCTTCAACCCCACGTTCGTCTTCATGGA
CCGCTACAGCTCCTCGGTCATCTCGCCCATCCTCACGTCGGGCTTCAGCG
TGCTCACCATCCTCATCCTCACCTTCTTCCTGGTCGTCAACCCGCTGGGG
AACCTGTGGCTCATCCTGACCGTCACGTCGGTGGAGCTCGGCGTTCTGGG
TCTGATGGGCTACCACACGTTCGAGTGGCAGCCTGCCCTCAAGAATGTGT
CTCCATCCTGCCATGTGGGTATCATCAATGGGCTTGCGGGGTGGGCCGCC
TCGGTGGATGACGTCCCTGCTGACACCATCACCCGTCGGTTTCGTTATGA
CGTGGCGCTGGTATCGGCCCTGAAGGACCTAGAGGAGGACATCATGGAAG
GACTGAGTGAGCATGGGCTGGAAGACAGCGCTTGCACCTCAGGCTTCAGT
GTTATGATCAAGGAATCCTGTGACGGCATGGGAGATGTCAGCGAGAAGCA
CGGTGGGGGGCCAGCGATGCCTGAGAAGGCTGTGCGTTTCTCTTTCACCG
TCATGTCCATCTCTGTCCAGGCCGACGGAGAGGAC---------------
---------------GAGGCGTTCGTTGTTTCAGGGAGCCAAAGCCCAA
CTCGGAACTGTCCTGTAAGCCCCTGTGTCTGATGTTTGTGGACGAGTCAG
ACCACGAGACACTCACTGCTGTCCTTGGGCCTTTGGTAGCCGAGAGGAAT
GCAATGAAGCAAAGCCGACTCATCCTCCCAGTGGGAGACCTCCCCCGCTC

CTTCCGCTTCCACTTCAGAGGCACGGGCTACGATGAGAAGATGGTGCGAG
AGATAGAAGGCCTGGAGGCTGCAGGCTCCACCTATATCTGCACTCTTTGT
GACTCCACCCGGGCAGAAGCCTCTCACAACATGGTGCTCCACTCTGTCAC
GCGCAGCCACGACGAGAACCTGGAGCGCTATGAGATATGGAGGACCAACC
CCTTCTCGGAGTCTGCCATGGAGCTGCGAGATCGGGTCAAAGGCGTCTCT
GCCAAACCATTTATCGAGACCCATCCGACTATGGATGCATTACATTGTGA
CATAGGTAATGCCACTGAATTCTACAAAATCTTCCAGGATGAGATTGGGG
AGGTGTTCCAGAAGGC---GAAC---CCTAGCCGGGAGGAACGGCGCAGC
TGGAGGGCAGCCCTCGATAAACAGCTGAGAAAGAAGTTGAAGCTGAAACC
AGTGATGCGGATGAATGGGAACTTTGCCCGAAGGCTGATGACTGAGGAGA
CTATAGAGGTGGTGTGTGAACTGGTGCCCTCAGAGGAGAGACGGGAGGCA
CTGAGGGAGCTGATGGGGCTCTACATCCAGATGAAGCCGGTGTGGCGCGC
TACCTGCCCAGCCAAGGAGTGCCCTGACCAACTGTGCCGCTACAGCTTTA
ACTCTCAGCGTTTTGCCGATCTCCTCTCCTCCACCTTCAAATACAGGTAT
GACGGGAAGATCACCAACTACCTGCACAAGACCCTGGCTCATGTCCCCGA
AATCATAGAGAGAGACGGCTCCATCGGAGCCTGGGCCAGCGAGGGGAATG
AGTCTGCGAACAAA------------------------------------

>Antennarius striatus
AGCCTACTAATCCGCGCAGAACTAAGCCAACCAGGCGCACTCTTAGGTGA
TGATCAAATTTACAATGTTATCGTTACAGCACATGCTTTCGTTATAATTT
TCTTTATAGTCATACCAATTATGATCGGAGGGTTCGGCAACTGATTAATT
CCACTAATAATTGGCGCCCCTGACATAGCATTCCCTCGAATAAACAATAT
AAGCTTCTGACTCTTACCCCCATCATTTCTTCTTTTATTAGCCTCATCAG
GAGTAGAAGCTGGAGCAGGCACAGGATGAACGGTTTACCCACCTCTTGCG
GGCAACCTAGCCCATGCCGGAGCATCTGTTGATTTAACTATTTTCTCACT
CCACCTTGCAGGTGTATCATCCATCCTAGGGGCTATTAATTTTATTACAA
CTATTATTAATATAAAACCACCAGCTCTTTCACAATACCAAACACCTTTA
TTTGTATGGGCTGTATTAGTCACTGCTGTACTTCTCCTCCTTTCCCTTCC
TGTTCTTGCTGCAGGGATTACAATATTATTAACTGATCGAAACCTTAATA
CAACTTTCTTTGACCCCACTGGCGGAGGAGACCCCATTCTATACCAACAC
TTATTCTGGTTTTTTGGCCA------------------------------

------------TTCCTAGAGAGAAACCTTCACCCAACCAACTGCCTCGG
GATGCTGTTGCTGTCTGATGCGCACCAGTGCACCAAGCTGTCCGAGCTGT
CCTGGGGCATGTGCCTCAGCAACTTTCCCGCTATTTGCAAGACTGAGGAC
TTTCTCCAACTGCCAAAAGATATGGTTGTGCAGCTTTTGTCACACGAAGA
GTTAGAAACTGAAGATGAGAGACTTGTTTATGAAGCTGCCCTCAATTGGA
TCAACTATGACCTGGAAAAGAGGCACAGCCACCTTCCAGAACTCTTGAGA

ACGGTCCGACTGGCCCTGCTGCCTGCCATCTTTCTGATGGAGAACGTCTC
TACAGAAGAGCTGATTAATGCCCAGCCCAAGAGCAAGGAACTGGTGGATG
AAGCTATCCGTTGTAAGCTGAAGATCTTGCAGAATGATGGTGTTGTTAAC
AGCCCATGCGCTCGACCAAGAAAAACCAGCCATGCTCTTTTTCTTCTGGG
TGGTCAGACATTCATGTGTGATAAGTTGTATCTGGTAGACCAGAAAGCCA
AAGAGATCATCCCCAAAGCTGACATTCCCAGCCCCAGGAAGGAGTTCAGC
GCCTGCGCCATCGGCTGTAAGGTGTACATCACTGGAGGAA--GAGGTTC-
CGAGAACGGAGTGTCCAAAGATGTATGGGTCTACGATACAGTTCATGAGG
AATGGTCCAAGGCGGCGCCCATGCTCATTGCCAGGTTTGGCCATGGCTCT
GCAGAGCTTAAACACTGCCTCTATGTGGTAGGAGGTCACACTGCAGCAAC
CGGCTGCCTCCCAGCNNNNNNNNNNGGATGAGTACATTGTTGTGTTCAGTCGGTCAACAA
CAAGACTGATTCTGAATGAAGCAGAGCTAATTATGGCATTGGCCCAGGAG
TTTCAAATGAGAGTGGTGACATTGTCCCTGGAGGAACAGTCTTTCTCTAG
TATTGTCCAGATGATCAGCAGTGCTACCATATTAGTGAGTATGCATGGAG
CTCAACTCATTACCTCACTCTTCCTACCTAGAGGATCTGTTGTGGTGGAG
TTGTTCCCTTATGCTGTGAACCCAGAACAGTACACCCCATATAAAACCCT
TGCCTCCCTTCCAGGCATGGACCTTCATTATATTTCATGGAGGAACAATA
ACGAGGACAACAGCATCACCCACCCAGACAGACCCTGGGAACAAGGGGGT
ATTGCTCATTTGGAAAAAGAGGAGCAAGAACGTATAAAGATGAGYAAAGA
CGTCCCCAGGCATCTATGCTGCCGCAACCCAGAGTGGCTCTTCAGGATCT
ACCAGGACACTTTGGTGGACATCCCCTCCTTCCTGGAAGTTCTCAA---A
GATGGCATGAAG---ACAAAGCCCAGCCTGAGAAA---GTCAAAGCCGTC
TGGTACACTCCACCCGGGCAGGGTCCGAGAACCTCAGTGTCAAACTTCAG
TTCAAACCAGCAATGAGGCTAAACTCATAGTCTCCTGGCAGATACCATGG
AATCTGAAGTACCTGAAGGTCAGAGAGGTGAAATACGAGGTGTNNNNNNNNAGGAAGG
ATGCCAACAAGGGGACCCTGGAGGATCAAATCATTCAGGCCAACCCTGCG
TTGGAGGCCTTTGGCAACGCAAAAACGTTGAGGAACGACAACTCATCCCG
TTTTGGCAAGTTCATCAGAATTCATTTTGGGACAAGCGGCAAGTTGTCTT
CTGCGGACATTGAGACGTACCTGCTGGAGAAGTCTCGCGTCACTTTTCAG
CTCAAGGCTGAGAGAAACTATCACATCTTCTACCAGATCCTGTCCAATCA
GAAGCCAGAGCTGCTGGACATGCTGCTGGTCACCAACAACCCCTATGACT
ACTCCTACATCTCCCAAGGAGAGGTATCCGTCCCTTCCATTAACGACTCG
GAGGAGCTGATGGCCACCGATAGTGCCTTTGACGTACTGGGCTTCACGGC
AGATGAGAAGATGGGTGTCTACAAGCTCACAGGAGCCATCATGCATTACG
GCAACATGAAGTTTAAACAGAAGCAGCGTGAAGAGCAGGCAGAACCCGAC
GGAACGGAGGCGGCGGATAAATCCGCCTACCTGATGGGGCTGAACTCCGC
TGACCTCATCAAAGGGTTGTGTCACCCCAGGGTGAAGGTAGGAAACGAAT
ACGTCACCAAAGGCCAAAGTGTGGATCAAGTCTACTACCCCAACAAGGAG
GCCTTCAAATGTGACGAGTGTGGGAAGCACTACAACACCAAGCTGGGATT
TAAGCGCCATGTCGCCATGCACTCTGCCACGGCTGGGGACCTCACATGTA
AAGTGTGCATGCAGACCTACGATAGCACGCCTGTGCTGCTGGAGCACCTG
AAGAGCCACTCTGGGAAGTCCTCTGGTGGCACCAAGGAGAAGAAACACCA
GTGCGACCACTGTGATCGGCGTTTCTACACAAGGAAGGACGTGAGACGCC
ACATGGTGGTCCACACCGGCAGGAAGGACTTCCTTTGCCAGTACTGTGCC
CAACGCTTTGGCAGGAAGGACCACCTGACGCGCCACGTGAAGAAGAGCCA
CTCGCAGGAGCTGCTGAAGATCAAGACGGAGCCTCCTGACATGTTGGGTC
TCTTGGCTTCCGGGTCGCCGCCGTGCTCTGTGAAGGAGGAGCTCAGCCCC
ATGATGTGTGGGATGGGTCCCAACAAGGACCCCATCATGAGCAAACCCTT
TCCTAGTGGGGCCCCTTTTCCCATGGGCATGTACAACCCTCATCAC----
--CTCCAGGCCATGTCTAATTCAGGGGTCGGTCACTCA------CACCCG
TCCCTGATGCCCGGCTCCCTGTCCGCGGCCATGGGTATGGGCTGTCACAT
GGAATACCTCATCTACGCCTCTTTTTCCTTCATGGGATGTTTACAAATCA
GTGATGGATCAAATATTGTGAACCTGCTGGCAAGTAACTCTCCAAGTGTT

TCATATGCTCTAACCCAGCAAAAATACTTCAGCAACTATAGTCCAGTGAT
TGGGTTTTACATTTATGAGCCCATTGAGTACTGGAACTCCACAGTGCAGG
AGCACCTCAGGACTCTGAGCCAAGGCTTCATCAAGATCTCCTGGATGGAC
AATTTTTTCCAATACCTGAAGGTGGTAAATGTGAGTGCATCAACCAAGAG
CGACTTTATCAGCATCCTGAAGGGCTCCTTCCTGCGCAGCCCAGAGTACC
AGCACTTCACTGAGGACATCATTTTCTCAAAGA---ACCGTGAGACTG--
----ATGAGTACGACATTATCGCCTCTCGGATGTACTTGGTAGCACGGAC
TACAGACAAGAAACGTGAAGAAGTGATGGTGCTTCTGGAAAAACTTCGTC
CCCTGATGTTGATCAACAGCATCAAATTCATAGCCTTCAATCCTACATTT
GTGTTCATGGACCGCTACAGCTCCTCGGTCATCTCACCCATCCTGACCTC
AGGATTCAGCGTACTCACAATCCTCATCCTCACTTTCTTCCTGGTCATCA
ACCCCTTGGGTAATTTTTGGCTCATTCTCACTGTAACATCTGTGGAGCTG
GGAGTGCTGGGNNNNNNNGGCTTTCACCAGTTTGAATGGCAGCCAGCTCTCAAGAAT
GTGTCAACATCTTGCAGTGTTGGCATTATTAATGGTCTCTCTGGATGGGA
TTCCTCAGTGGATGAGTCCCCAGTTGACACCATCACTCGTCGGTTTCGCT
ATGATGTGGCACTGGTATCAGCATTAAAGGATCTGGAGGAGGACATCATA
GAGGGACTGAGAGAGAATGGAATGGAAGACAGCACTTGCC------ACTT
TACTGTCATGATCAAAGAGTCCTGTGATGGTATGGGTGATGTCAGTGAAA
AACATGGCGGAGGACCGGCTGTCCCTGAGAAGGCTGTGCGTTTCTCTTTC
ACCGTCATGTCTGTGTCTTTCAGAAAAGACAGCAAGGAG-----------
------------------GAAGAGGTCACCATCTTCACAGAACCAAAGC
CAAATTCAGAACTGTCCTGTAAGCCCCTTTGCCTGATGTTTGTGGATGAA
TCAGACCATGAGACACTCACAGCAATCCTGCATCCTATAATTGCAGAGCG
TAATGCAATGAAAGAGAGCAGATTAATCCTATCCATTGGTGGACTTCCAC
GATCCTTCAGCTTTCATTTTAGGGGCACGGGATATGATGAGAAGATGGTT
CGTGAGATGGAAGGCCTTGAGGCCTCTGGGTCTGCCTACATTTGCACCCT
CTGTGATGCCAGTCGTGCAGAGGCCTCTGAAAACATGGTGTTACATTGTA
TCACCCGCAGTCATGAAGAGAATCTTGAGTGTTATGAAATTTGGAGAACC
AATCCATTTTCTGAGTCTGTAGAGGAGTTGCGAGATAGAGTCAAAGGGGT
CTCTGCCAAGCCCTTCATGGAGACCCATCCCACGATGGATGCATTACACT
GTGACATTGGCAATGCCACTGAGTTCTACAAAATATTCCAAGATGAGATT
GGGGAGGTGTACCAAAAGGT---CAAT---CCCAGCCGGGATGAGAGGCG
TAGCTGGAGGGCAGCCCTAGATAAACAGCTGAGAAAGAAGATGAAGCTTA
AACCAGTAATGAGGATGAATGGGAACTATGCCCGCAGGCTAATGACCCTG
GAGGCTTTGGAGGTGGTGTGTGAGCTGGTGCCCTCAGAGGAGAGAAGGGA
GGCCCTTAGGGAACTAATGAGACTCTACATTCAGATGAAGCCCGTGTGGC
GCGCCACCTGCCCAGCCAAAGAGTGCCCTGACCAGCTGTGCCGATACAGC
TTCAACTCCCAGAGCTTTGCAGAGGTCCTCTCCTCTAAGTTCAAATATAG
GTACAATGGGAAGATAACCAATTACTTGCACAAGACTTTGGCTCATGTGC
CTGAAATCATAGACAGAGATGGATCCATAGGGGCTTGGGCCAGTGAAGGG
AATGAGTCAGCAAACAAATCCTACACCATTGAGATGGGTCCGCTGGGCCC
CGGATGGAAGGAGAGCCCACAGCCGTTCTCCTGCTCCATCGAAGACCCCA
CCAAACAGACGAAGTTCAAGGGCATCAAGACGTACATCTCGTACCGCGTG
ACGCCGAGCCACACCGGGCGTCCCGTCTACCGGCGCTACAAACACTTCGA
CTGGCTGTACAACCGCCTCCTGCACAAGTTCACTGTGATCTCCGTGCCCC
ACCTGCCTGAGAAGCAGGCCACGGGCCGATTCGAGGAAGACTTCATCGAG
AAACGCAAGAGACGACTGATCCTGTGGATGAACCACATGACCAGCCACCC
CGTCCTCTCCCAGTACGAAGGTTTCGAACACTTTCTGATGTGCGCCGACG
ACAAGCAGTGGAAACTGGGGAAGAGACGGGCCGAAAAAGATGAGATGGTG
GGCGCCCACTTCATGCTGACCCTCCAGATCCCCAACGAGCACCAGGACCT
TCAGGACGTGGAGGAGCGGATCGACACCTTCAAGTCCTTCGCCAAGAAGA
TGGACGACAGCGTGATGCAGCTCACGCATGTTGCGTCGGAGTTGGTGCGG
AAACACCTGGGCGGGTTCAGGAAGGAGTTCCAGCGTTTGGGGAACTCCTT

CCAGTCCATCAGCCAGGCCTTCATGCTGGACCCTCCCCACAGCTCTGACA
CCCTGAATAACGCCNNNNNNNNN------------------------------------

-GCCAAATCTCGCTTTCACCCTGGCATGGGGACTGGTCCTGGCACGGAGC
---GCAGCGTCCCACTCGGCAACAGCTTGCTATCCCCGCAGCAAACCGAG
GAGCCCACTGTTGCCACCCCCCCGCAGCGATGGTTTGTCACCC---CTGC
CAACAACCGACTGGACTTTGCTGCTTCGGCATACGAAGCCGCC-------
--GATTTCGCCGGTAACGCGGCCACCTTGCTGTCCTACGCAGCGGCCGGA
GTAAAGGCTC------TTCCCCTGCCGACTGCGGGCTGCTCCAATCGGCC
TCTTGGCTATTACGCGGACCCTTCAG---GCTGG---GGGGGACGCACAC
CGCCGCAGTATTGTGGCGTGAATAGCAAATCCAGCTCGGTCTTTTCCTGC
TGGCCCTCTAACACTATCGGAGGCAGAGTGGGCA---CC---AGTTACCT
GG---------CCGAGGA---GGGA---GACTC---TATCCCTACAGAGA
GGTCACCG---AT---CGGCGCGTCCGAGGAG---GCCAAACCCAAAGAC
ATCAC---GTCAGA---GTCGAACTGGATTGAG---ACGCCGTCCTCTAT
TAAGTCAATCGATTCAAGTGATTCTGGGATCTTTG---AACAGGCCAAAA
GGAGAAGGATCTCACCTTATGCCACGCCA---------GTTTCAGAGACA
GAGTCCNNNNNNNNNNNNNNNCATCACTCAACAGGCGAAGTCACAGAGAGAGAAGTGGCGTTGGG
GATAAATCCGTTCGCTGATGGGATGGGCGCCTTCAAAATAAACCACAGCT
CCCACGATATTGGCTCCGG---ACAAACGGCGTTTTCCTCCCAGACA---
CCCGGTTAC---GCAGCGGCCGCCCTGGGA---CACCACCA------CCA
CCCGACCCACGTTGGCTCC---TACTCCACAGCTGCTTTCAACTCCACCA
GGGACTTTCTCTTCAGAAATAGAGGATTCGGGGATGCCACCGG-------
-----AGCGCAGCACAGTTTGTTCGCCTC---------CGGGAGTTT---
C------GCAGGGCCACACGGACACTCAGATGCGGCGGGGCACCTGCTCT
TCCCGGGGCTCCACGAG---CAAGCGGCGACCCACGCGTCTTCCAACGTG
GTCAACAGCCAGATGCGGCTGGGCTTCTCGGGGGACATGTACGGACGAGC
AGACCAGTACGGCCACGTTACAAGCCCGCGGT---CCGATCATTACGCTT
CGACCCAGTTGCACGGCTACGGCCCTATGAACATGAATATGGCCGCA---
CACCACGGAGCAGGGGCCTTCTTTCGATACATGAGGCAGCCGATCAAACA
AGAGCTCATCTGCAAGTGGATCGAGCCGGAGCAGCTGACAAATCCCAAAA
AGTCGTGCAACAAAACTTTCAGCACGATGCATGAGCTGGTCACCCATCTG
ACGGTGGAGCACGTCGGTGGACCGGAGCAGACGAACCACGTCTGCGTGTG
GGAGGACTGCTCCAGAGAAGGGAAGCCATTCAAAGCCAAATACAAACTTG
TAAATCATATCAGAGTACACACCGGAGAAAAGCCCTTTCCGTGTCCCTTC

CCCGGCTGTGGCAAA
>Antigonia capros
AGCTTACTTATCCGAGCTGAACTAAGCCAACCTGGGGCACTCCTAGGAGA
TGACCAAATCTACAATGTTGTAGTTACAGCACATGCCTTTGTAATAATTT
TCTTTATAGTAATACCAATTATAATTGGAGGATTTGGAAACTGACTAATT
CCTTTAATGATTGGAGCCCCCGATATAGCATTCCCCCGAATGAACAATAT
GAGCTTCTGACTACTTCCACCCTCTTTTTACTTCTCCTTGCCTCTTCTA
TAGTAGAAGCAGGGGCGGGCACTGGATGAACAGTTTACCCCCCTCTAGCT
GGGAACCTGGCCCATGCCGGGGCATCAGTTGACTTAACAATTTTTCTCT
CCACTTAGCAGGGATTTCCTCAATCCTTGGGGCCATCAACTTTATCACAA
CTATTATTAATATGAAACCTCCCGCTATTTCCCAGTACCAAACTCCCCTG
TTTGTTTGAGCAGTACTAATTACTGCAGTTCTTCTTCTCCTCTCCCTTCC
CGTCCTTGCTGCCGGAATTACAATACTTCTTACAGACCGAAACTTGAACA
CCACCTTCTTTGACCCAGCCGGAGGAGGAGACCCGATTCTTTATCAACAT
CTATTCTGATTTTTTGGGCACCCTGAAGTTTACATCCTGATTCTTCCGGG
CTTTGGAATAATTTCTCATATTGTTGCCTACTACTCAGGTAAAAAGAAC
CATTCGGCTATATAGGCATAGTTTGAGCCATAATGGCTATTGGACTCTTG
GGCTTCATCGTGTGAGCCCATCATATGTTTACTGTAGGAATGGACGTAGA
CACACGAGCTTATTCCTAGAGAGAAACCTTCACCCATCTAATTGCCTTGG
CATGCTTTTGCTGTCCGATGCCCACCAGTGCACCAAGCTGTCAGAGCTCT
CCTGGGGCATGTGCCTCAGCAACTTTCCCGCTATTTGCAAGACAGAGGAC
TTCCTCCAACTGCCCAAAGATATGGTGGTGCAGCTTTTGTCACACGAGGA
GCTAGAGACTGAAGATGAGAGACTGGTTTATGAAGCTGCCCTCAATTGGA
TCAACTATGATCTGGAAAAGAGGCACTGCCACCTTCCAGAACTCCTGAGA
ACGGTCCGTCTGGCCCTGCTGCCTGCCATCTTCCTCATGGAGAATGTTTC
TACAGAAGAGCTGATCAATGCCCAGGCGAAGAGCAAGGAGCTGGTGGATG
AAGCTATCCGCTGCAAGCTGAAGATCCTGCAGAATGATGGTGTCGTTAAC
AGCCCATGTGCTCGACCAAGAAAAACCAGCCATGCCCTCTTTCTGCTGGG
TGGGCAGACTTTCATGTGTGACAAATTGTACCTGGTGGACCAGAAGGCCA
AAGAAATCATCCCCAAGGCTGACATTCCCAGCCCCAGGAAGGAGTTCAGC
GCCTGTGCCATCGGCTGTAAGGTGTATATCACTGGTGGGA--GAGGCTC-
AGAGAATGGGGTGTCTAAAGATGTATGGGTCTACGACACCGTCCACGAGG
AATGGTCAAAGGCGGCACCCATGATCATTGCCAGGTTTGACCTAGGCTCT
GCNNNNNNNNNNNNNNNNNNNNNNNNNNNNNNNNNNNNNNNNNNNNNNNNNNNNNNNNNNNNNNNNNNNN
NNNNNGGATGAATATATTGTTGTGTTTAGTCGTTCAACAACGAGACTGATACT
GAATGAAGCGGAGTTCATCATGGCGCTGGCTCAGGAGTTCCAGATGAGAG
TGGTCACAGTATCCCTGGAGGAGCAGTCTTTCCCCAGTATCGTCCAGGTG
ATCAGCGGTGCGACCATGTTAGTCAGTATGCATGGAGCTCAGCTCATCAC
CTCACTCTTCCTGCCCAGGGGAGCTGCTGTGGTGGAGCTGTTCCCCTTTG
CTGTGAACCCAGAGCAGTACACCCCATATAAGACTCTCGCCTCCCTTCCA
GGAATGGACCTTCACTATATCTCCTGGAGGAACACCAAGGAGGAGAACAC
CATCACATACCCAGACAGACCCTGGGAACAAGGGGGTATTGCTCACTTGG
ATAAGGAGGAGCAAGAGCGAATACTGGCGAGCAAAGATGTCCCCAGGCAT
CTGTGCTGCCGCAACCCGGAGTGGCTCTTCCGGATCTACCAGGACACTTT
GGTGGATATCCCCTCTTTCCTGGAAGTCCTCAA---AGAGGGCATGAAG-
--ACAAAGCCCGGCTTGAAGAA---GTCTAAGCCGGCCAGCACGCTCCAC
CCGGGCCGGGTCAGAGAACCTCAGTGTCAGACCTCAGTACAAACCACTAA
CGAGGCTAAACTCACAGTCTCTTGGCAGATCCCGTGGAATCTGAAATACC
TGAAGGTCAGAGAGGTCAAATACGAGGTG---------AAAAGGGACACC
AGCAAGGGGACACTGGAGGATCAAATCATCCAGGCGAACCCTGCGCTGGA
GGCCTTCGGTAACGCCAAAACACTGAGAAATGACAACTCGTCTCGCTTTG
GAAAATTCATCCGAATTCACTTTGGTACGAGCGGGAAGCTGTCATCCGCT
GACATTGAAACGTACCTGTTGGAGAAGTCACGCGTCACCTTTCAGCTGAA

GTCTGAGAGAAACTACCACATCTTCTTCCAGATCCTGTCCAACCAGAAGC
CAGAGCTGCTGGACATGCTGCTGATCACCAACAACCCATACGACTACTCC
TACATCTCCCAAGGAGAGGTAACGGTCGCTTCCATCAACGACTCCGAGGA
GCTGATGGCCACGGACAGCGCCTTTGATGTGCTCGGCTTCACTGCAGACG
AGAAGATGGGCGTCTATAAACTGACAGGCGCCATCATGCACTACGGCAAC
ATGAGGTTCAAACAGAAGCAGCGCGAGGAGCAGGCGGAGCCGGATGGGAC
GGAGGCTGCTGATAAATCAGCATACCTAATGGGGCTGAACTCTGCTGACC
TCATCAAAGGGCTATGCCATCCCAGAGTCAAGGTAGGAAATGAATACGTC
ACCAAAGGCCAAAGTGTGGACCAAGTCTACTATCCCAACAAGGAGGCCTT
CAAGTGTGAGGAGTGTGGGAAGCACTACAACACCAAGCTGGGTTACAAGC
GCCATGTGGCCATGCACTCGGCCACGGCAGGGGATCTCACCTGTAAAGTG
TGCATGCAGACCTATGAGAGCACGCCTGTGCTCTTGGAGCACCTCAAGAG
CCATTCCGGGAAGTCTTCAGGTGGCACCAAGGAGAAAAAGCACCCGTGCG
ACCACTGTGACCGTCGTTTCTACACAAGGAAGGATGTGAGACGGCACATG
GTCGTCCACACGGGCCGAAAGGACTTCCTGTGCCAGTACTGTGCCCAACG
CTTTGGCAGGAAGGACCACCTGACACGCCATGTGAAGAAGAGCCACTCAC
AGGAGCTGCTGAAAATCAAGACGGAGCCGCCTGATATGTTAGGTCTTTTA
GCTTCTGGGTCACCACCTTGCTCTGTGAAGGAAGAGCTCAGCCCCATGAT
GTGCGGCATGGGTCCCAACAAAGACCCCATGATGGGCAAACCGTTCCCCA
GCGGTGCCCCTTTTCCAATGGGCATGTACAACCCCCACCAT------CTC
CAGGCCATGTCTAATTCTGGGGTGGGTCACCCA------CACCCGTCCCT
GATGCCCAGTTCCTTGTCTGCAGCTATGGGCATGGGCTGTCACATGGAAT
ATCTCATCTATGCCTCTTTCTCATTCATGGGATGTTTACAAATCAGTGAT
GGATCAAATATCGTGAACCTGCTGACTAGTAACTCTCCAAGTGTTTCGTA
TGCTCTTACCCAACAAAAATACTTCAGTAACTACAGTCCAGTGATTGGGT
TCTACATTTATGAGCCCATCGAGTACTGGAACTCCACAGTGCAGGAGCAC
CTGAAGACTCTGAGTCATGGCTTCAACAAGATCTCCTGGATGGACAACTT
CTTCCACTACCTGCGGGTGGTGAATGTGAGTGCGTCAACCAAGAGCGACT
TCATCACCATCCTCAAGGCCTCCTTCCTGCACAGCCCGGAGTACCAGCAC
TTCACTGAGGACATCATATTCACAAAGA---ACCGTGACACTG------A
TGAGTATGACATTATCGCCTCACGGATGTACTTGGTGGCACGGACGACAG
AGAAGAAGCGCGAAGAGGTGGTGGAGCTTCTGGAAAAGCTTCGTCCGTTG
ATGCTGATCAACAGCATCAAGTTCATTGCCTTCAATCCTACGTTTGTGTT
CATGGACCGCTACAGTTCCTCTGTCATCTCGCCCATCCTGACCTCAGGAT
TCAGCGTACTCACAATCCTCATCCTCACTTTTTTCCTGGTCATCAACCCC
TTGGGTAACTTCTGGCTCATCCTCACTGTAACGTCCGTGGAGCTGGGCGT
CTTGGGGTTGANNNNNNNNNNNNNNNNNNNNNNNNNNNNNNNNNNNNNNNNNNNNNNNNNNNNNNNNNNT
GTTGGCATTATTAACGGACTCTCTGGATGGGCTTCCTC
AGTGGATGACTCCCCAGCTGACACCATCACTCGACGGTTTCGCTATGATG
TGGCACTGGTGTCAGCATTAAAGGATCTGGAGGAGGACATCATGGAGGGG
CTGAGAGAGAGTGGGCTGGAAGACAGCGCTTGCACCTCGGGCTTCAGTGT
CATGATCAAGGAATCTTGTGATGGCATGGGTGATGTCAGTGAGAAGCATG
GTGGAGGACCAGCTGTTCCTGAGAAGGCTGTACGTTTCTCTTTCACTGTT
ATGTCTGTTTCTGTCCTGGCAGACGATGAGGAG-----------------
-------------GAACGGGTTACCATCTTCACAGAGGCAAAGCCAAACT
CAGAGCTGTCCTGTAAGCCCCTATGCCTGATGTTTGTGGATGAGTCAGAT
CATGAGACACTGACAGCCATCCTGTGGCCTGTAATTGCAGAACGTCAAGC
AATGAAAGACAGCAGGCTAATTTTATCCATCGGTGGACTACCTCGCTCCT
TCCGGTTTCACTTCAGAGGCACGGGATACGATGAGAAGATGGTGCGTGAG
ATGGAGGGGCTTGAGGCCTCTGGGTCCTCCTATATCTGCACTCTTTGTGA
CTCCAGTCGAGCAGAGGCCTGTCAAAACATGGTGCTACACTCCGTCACCC
GGAGTCATGAAGAAAATCTAGAACGCTATGAAATATGGAGAACCAACCCC
TTTTCTGAGTCTGTAGATGAGCTGCGAGACAGAGTCAAAGGGGTCTCTGC

CAAGGCCTTCATGGAGACCCATCCCACGCTGGATGCATTACACTGTGACA
TCGGCAATGCCACTGAGTTCTACAAAATCTTCCAGGACGAGATCGGGGAG
GTGTACCAAAAGGT---CAAC---CCCAGCCGGGAGGAGCGGCGCAGCTG
GAGGGCAGCCCTAGATAAACAGCTGAGGAAGAAGATGAAGCTCAAACCGA
TAATGAGGATGAATGGGAACTATGCCCGCAGGCTAATGACCCAGGAGGCT
GTGGAGGTGGTGTGTGAGCTTGTGCCCTCTGAGGAGAGGAGGGAGGCCCT
GAGGGAGCTTATGAGGCTCTACATCCAGATGAAGCCCGTGTGGCGTGCCA
CCTGCCCAGCCAAGGAGTGCCCCGACCAGCTGTGCCGCTACAGCTTTAAC
TCCCAACGCTTTGCTGACCTCCTCTCCTCTACCTTCAAATATAGGTACAA
TGGAAAGATAACCAATTACCTGCACAAGACCCTGGCTCATGTGCCTGAAA
TCATAGAAAGAGATGGATCCATAGGAGCTTGGGCCAGCGAAGGNNNNNNNNNNNNNNNNNNNTCTTACA
CCATCGAGATGGGCCCCATGGGGCCCCAATGGAAGGAGAGCCCACAGCCG
TTCTCCTGTTCTATTGAAGACCCAACAAAACAAACAAAGTTCAAGGGCAT
CAAGACGTACATTTCGTACCGGGTCACGCCGAGCCACACGGGGCATCCCG
TCTACAGGCGCTACAAACACTTTGACTGGCTGTACAACCGCTTACTGCAC
AAGTTCACTGTGATCTCCGTGCCTCACCTGCCCGAGAAGCAGGCCACGGG
GCGATTTGAGGAAGACTTCATCGAGAAGCGCAAGAGGCGACTGATACTGT
GGATGAACCATATGACCAGTCACCCGGTCCTCTCCCAGTACGAAGGCTTC
GAGCACTTTCTGATGTGTGCTGACGACAAGCAGTGGAAACTAGGCAAGAG
GCGGGCAGAGAAGGACGAGATGGTGGGGGCCCATTTCATGCTGACCCTCC
AGATCCCTAACGAGCACCAAGACCTTCAGGATGTTGAGGAGCGGATTGAC
TCCTTCAAGTCCTTCGCTAAGAAAATGGATGACAGCGTGATGCAGCTCAC
ACATGTTGCCTCGGAGCTGGTGCGCAAGCACCTGGGCGGGTTCAGGAAGG
AGTTCCAGCGGCTGGGAAACGCCTTCCAGTCTATCAGCCAGGCGTTCATG
CTGGACCCACCCCACAGCTCCGAGACCCTAAACAGNNNNNNNNNNNNN-
NNNNNNNNNNNNNNNNNNNNNNNNNNNNNNNNNNNNNNNNNNNNNNNNNNNNNNNNNNNNNNNNNNNNNN
NNNNNNNNNNNNNNNNNNNNNNNNNNNNNNNNNNNNNNNNNNNNNNNNNNNNNNNNNNNNNNNNNNNNNN
NNNNNNNNNNNNNNNNNNNNNNNNNNNNNNNNNNNNNNNNNNNNNNNNNNNNNNNNNNNNNNNNNNNNNN
NNNNNNNNNNNNNNNNNNNNNNNNNNNNNNNNNNNNNNNNNNNNNNNNNNNNNNNNNNNNNNNNNNNNNN
NNNNNNCTTCTGTGTCAGTG
TCACGCGCTATCTGGCCATCGCACATCACCGTTTCTACACCAAGAGGCTG
ACCTTCTGGACCTGTCTAGCTGTCATCTGCATGGTGTGGACGTTGTCAGT
GGCTATGGCGTTCCCGCCAGTGCTAGACGTAGGGACGTACTCTTTTATCC
GGGAGGAGGACCAGTGCACATTCCAGCACCGTTCCTTCAGGGCGAATGAT
TCGCTGGGCTTCATGCTCCTGCTGGCGCTCATCCTCCTGGCCACACAGCT
GGTTTACCTCAAGCTCATCTTCTTCGTCCACGACCGTCGAAAAATGAAGC
CTGTCCAGTTCGTGCCTGCCGTCAGCCAGAACTGGACCTTCCATGGACCA
GGCGCCAGCGGGCAGGCGGCAGCCAACTGACTGGCAGGATTCGGTCGACG
CCCCACCCCGCCTACTCTGCTGGGCATCCGGCATAACAGCAACGCACCAG
GTCGCAAGCATCTACTGGTATTGGATGAGTTCAAAACAAAGAACAGGATT
AGTACGATGTTCTACATCATGACTTTTTTCTTCCTGGCACTGTGGGGGCC
CTACCTGGTCGCCTGCTACTGGCGCGTGTTCGCAGGGGCCCCCGTGACCG
CTGGGGGCTACCTGACGTCAGCCGTGTGGATGAGCTTTGCCCAGGCTGGG
GTCAATCCTTTCATCTGCATCTTCTCCAGCNNNNNNGCCAAATCTCGCTTTCACCC
TGGCATGGGGACTGGTCCTGGCACGGAGC---GCAGCGTCCCACTCGGCA
ACAGCTTGCTATCCCCGCAGCAAACCGAGGAGCCCACTGTTGCCACCCCC
CCGCAGCGATGGTTTGTCACCC---CTGCCAACAACCGACTGGACTTTGC
TGCCTCGGCATACGACGCCGCT---------GATTTCGCCGGTAACGCGG
CCACCTTGCTGTCCTACGCAGCGGCCGGAGTGAAAGCTC------TTCCC
CTGCCGACTGCAGGCTGCTCCAACCGGCCTCTTGGCTATTACGCAGACCC
GTCAG---GCTGG---GGAGGACGCACGCCGCCGCAGTACTGTGGTGTGA
ACAGCAAACCCAGCTCGGTCTTTTCCTGCTGGCCCTCTAACTCTATCGGA
GGCAGAGCGGGCA---CC---AACTACCTGG---------CCGAGGA---

GGGA---GACTC---CATCCCGACAGAGAGATCACCG---AT---CGGCG
GCTCCGAGGAG---ACCAAACCCAAAGACATAAC---GTCAGA---GTCG
AACTGGATAGAG---ACGCCGTCCTCCATTAAGTCCATCGATTCAAGCGA
TTCTGGTATCTTTG---AACAGGCCAAAAGGAGAAGGATCTCACCTTCTG
CCACGCCG---------
GTTTCAGAGACAGTGTCCCCGTTAAANNNNNNNNNNNNNNNNNNNNNNGAAGTCA
CAGAGAGAGAAGTGGCGTTGGGGATAAATCCGTTCGCGGATGGGATGGGC
GCCTTCAAAATAAACCACAGCTCCCACGATATTGGCTCCGG---ACAAAC
AGCGTTTTCCTCCCAGGCG---CCCGGCTAC---GCAGCAGCCGCCCTGG
GA---CACCATCA------CCACCCGACCCACGTTGGCTCT---TACTCC
ACGGCGGCTTTCAACTCCACCAGGGACTTTCTCTTCAGAAATCGGGGATT
CGGGGACGCCACCGG------------CGCGCAGCACAGTTTGTTCGCCT
C---------CGGAAGTTT---C------GCAGGGCCACATGGACACTCA
GATGCAGCGGGGCACCTGCTCTTCCCGGGGCTCCACGAG---CAAGCGGC
GAGCCATGCGTCCTCCAACGTGGTCAACAGCCAGATGCGGCTGGGCTTCT
CGGGGGACATGTACGGACGGGCCGACCAGTATGGCCACGTTACGAGCCCG
CGGT---CCGACCACTATGCTTCGACCCAGTTGCACGGCTACGGCCCCAT
GAACATGAATATGGCCGCA---CACCACGGAGCTGGGGCCTTCTTTCGAT
ACATGAGGCAGCCGATCAAACAAGAGCTCATCTGCAAGTGGATCGAGCCG
GAGCAGCTGACAAATCCCAAAAAGTCGTGCAACAAAACTTTTAGCACGAT
GCACGAGCTCGTGACCCACCTGACGGTGGAGCATGTGGGGGGACCGGAGC
AGACCAACCTCATCTGCTTCTGGGAGGACTGCCCNNNNNNNNNNNNNNNNNNNNNNNNNNNNNNNNNNNN
NNNNNNNNNNNNNNNNNNNNNNNNNNNNNNNNNNNNNNNNNNNNNNNNNNNNNNNNNNNNNNNNNNN
>Aphredoderus sayanus
AGCCTCCTAATTCGCGCGGAACTAAGCCAGCCCGGAGCCCTTTTAGGAGA
CGATCAAATTTATAATGTTATCGTAACAGCCCACGCCTTTGTAATAATCT
TCTTCATAGTAATGCCTGTAATGATCGGAGGATTTGGCAACTGATTGGTG
CCACTAATAATTGGTGCCCCAGACATAGCTTTCCCCCGAATAAACAACAT
AAGTTTCTGACTTCTCCCCCCATCTTTCCTTCTCCTATTAGCCTCCTCTG
GAGTAGAAGCAGGAGCTGGCACTGGTTGAACGGTATACCCCCCTTTAGCA
GGCAACCTGGCCCACGCAGGGGCCTCCGTCGACTTAACAATTTTCTCCCT
CCACCTCGCAGGAGTATCATCTATCCTCGGGGCAATTAACTTTATCACAA
CCATTATTAACATGAAACCCCCAGCAATTTCGCAGTACCAAACCCCACTA
TTTGTGTGGTCTGTATTAATCACTGCCGTCCTCTTACTACTATCCCTACC
CGTTCTCGCAGCAGGAATCACAATACTACTAACAGACCGGAATCTAAACA
CCACATTCTTTGACCCTGCCGGCGGAGGTGACCCAATCTTATACCAACAC
CTC---

------------TTCCTGGAGCGCAACCTGCACCCGTCCAACTGCCTGGG
CATGCTGCTGCTCTCGGACGCCCACCAGTGCACCAAGCTGTCGGAGCTCT
CCTGGGGCATGTGCCTGAGCAACTTCCCCGCCATCTGCAAGACGGAGGAC
TTCCTCCAGCTGCCCAAGGACATGGTGGTGCAGCTGCTGTCCCACGAGGA
GCTGGAGACGGAGGACGAGCGGCTGGTGTACGAGGCGGCCCTCAGCTGGC
TGAGCTACGACCTGGAGGGGCGACACCGCCACCTGCCCGCGCTCCTGCGT
ACGGTGCGCCTGGCGCTGCTGCCCGCCGTCTTCCTGATGGAGAACGTGTC
CACGGAGGAGCTGATCAACGCCCAGGCCGAGGGCAAGGAGCTGGTGGACG
AGGCCATCCGCTGCAAGCTCCAGATCCTGCAGAACGACGGCGTGGTGACC
AGCGCGTGCGCCCGGCCGCGCAAGACCAGCCACGCCCTGTTCCTGCTGGG
CGGCCAGACCTTCATGTGCGACAAGCTGTACCTGGTGGACCAGAAGGCCA
AGGAGATCATCCCCAAGGCGGACATCCCCAGCCCCCGCAAGGAGTTCAGC
GCGTGCGCCATCGGCTGCCGGGTGTACATCACCGGCGGGC--GCGGCTC-

GGAGAACGGCGTCTCCAAGGACGTGTGGGTGTACGACACGGTGCACGAGG
AGTGGTCCAAGGCGGCGCCCATGCTCATCGCCAGGTTCGGCCACGGCTCG
GCCGAGCTGAAGCACTGCCTCTACGCGGTGGGCGGCCACACCGCCGCCAC
GGGCTGCCTCCCGGCGTCGCCCTCGGGATGAATACATTGTGGTGTTCAGT
CGTTCAACAACAAGGCTGATTCTCAATGAAGCGGAGCTAATTATGGCACT
CGCACAAGAGTTCCAGATGAGAGTGGTCACCGTCTCCCTGGAGGAACAAC
CTTTTGCCAGTATCGTGCAGGTGATTAGCGGAGCCAGCATGTTGGTAAGC
ATGCATGGAGCTCAGCTGGTCGCATCACTCTTCTTGCCCAGAGGAGCAGC
CGTGGTGGAGCTCTTCCCTTATGCTGTGAACCCAGAGCAGTACACGCCAT
ATAGAACACTGGCCTCCCTACCAGGCATGGACCTTCAGTATGTTTCATGG
AGGAACACTATGGAGGAGAACACCGTCACCCACCCAGACAGACCTTGGGA
CCAAGGAGGCATTGCCCACTTGGAAAAGGAGGAACAAGAGCGGATACTGT
CCAGTAAGGATGTCCCCAGGCATTTATGTTGCCGCAACCCAGAGTGGCTT
TTCCGAATCTATCAGGACACTTTGGTAGACATCCCTTCAATCCTAGAGGT
CCTGAA---AGAGGGCCTGAAG---ACGAGGCCTAGCTTAAAGAA---AG
CCAGGCCGTCTAGCACCGTTCATCCTGGACGGGTCAGGGAAGTTCAGTGT
CAGACGTCCGTCCAAGCTGCTAATGAGGCTAAACTCACAGTCTCCTGGCA
GATCCCATGGAATCTCAAATACCTGAAGGTGAGAGAAGTGAAGTACNNNNNNNNNNNNNNNNAAGA
AGGACACCAGCAAGGGAACTCTAGAGGATCAAATCATCCAGGCCAACCCG
GCACTGGAGGCCTTCGGCAATGCCAAGACCGCCAGGAATGACAACTCCTC
CCGTTTTGGAAAATTCATTCGGATCCACTTTGGAACAAGCGGGAAGCTGT
CATCTGCTGACATTGAAACATATCTTCTGGAAAAGTCCCGGGTCACCTTT
CAGCTCAAGGCTGAGAGGAATTACCACATCTTCTTCCAGATCCTGTCCAA
TCAGAAACCAGAGCTCCTGGAGATGCTGCTGATCACCAATAACCCGTACG
ACTACTCCTACATTTCCCAGGGAGAAGTGGCCGTCCTCTCAATCAACGAT
TCAGAGGAGCTGATGGCCACAGACAGCGCCTTCGACGTGCTTGGGTTCAC
CGCAGAGGAGAAGATGGGCGTGTACAAGCTGACAGGCGCCATCATGCACT
ATGGCAACATGAAGTTTAAACAAAAACAGCGCGAGGAGCAGGCGGAGCCG
GACGGCACCGAGGCAGCGGACAAGTCGGCATACCTCATGGGTCTGAACTC
TGCGGACCTCATCAAAGGCCTGTGCCATCCCAGGGTCAAGGTGGGAAATG
AGTATGTCACCAAAGGACAGGGTGTAGACCAAGTCTACTACNCCAACAAGG
AGGCCTTCAAGTGCGACGAGTGTGGCAAGCACTACAACACCAAGCTGGGA
TACAAGCGCCATGTGGCCATGCACTCCGCCACGGCAGGGGACCTCACCTG
TAAGGTGTGCATGCAGAGCTACGAGAGCACCCCCGTTCTCCTGGAGCACC
TCAAGAGCCACTCGGGCAAGTCCTCAGGCGGCGCCAAGGAGAAGAAGCAC
CCGTGCGACCACTGCGACCGCCGCTTCTACACCCGCAAGGACGTCCGGCG
GCACATGGTGGTGCACACCGGCCGAAAAGACTTCCTGTGTCAGTACTGCG
CCCAGCGCTTTGGCAGGAAGGACCACCTGACGCGCCACGTGAAGAAGAGC
CACTCGCAGGAGCTGCTGAAGATCAAGACGGAGCCCCCAGACATGCTGGG
CCTGCTGGGCTCTGGCTCCCCCCCGTGCTCCGTCAAGGAGGAGCTGAGTC
CCATGATGTGCAGCATGGCCCCCAATAAAGACCCAATGATGGGCAAGCCC
TTCCCCAGCGGGACCCCCTTCCCCATGGGCATGTACAACCCCCAC-----
----CTCCAGGCCATATCCAGCTCAGGGGTGGCCCACCCT------CACC
CCTCCCTGATGCCTNNNNNNNNNNNNNNNNNNNNNNNNNNNNNNNNNNNNNNNNNNNNTATCTCATCTATGC
TTCCTTCTCATTCATGGGATGT
TTACAAATCAGCGACGGATCGAACATTGTGAATTTGCTGGCCAGTAACTC
TCCAAGCGTTTCCTACGCTCTGACCCAGCAGAAGTACTTCAGCAACTACA
GTCCGGTGATCGGGTTCTACATTTACGAACCCATYGAGTACTGGAACTCT
ACAGTGCAGGAGCACTTGAAGACGCTGAGTCACGGCTTCAACAAAATCTC
CTGGATGGACAACTTCTTCCACTACCTGCGGGTGGTGAACGTGAGTGCGT
CGACGAAGAACGACTTCATCAACGTACTCAAGGGCTCCTTCCTGCGCAGT
CCGGAGTACCAGCACTTCACAGAGGACATCATCTTCTCCAAGA---ACCG
CGAAAGCG------ACGAGTACGACATCATCGCCTCGCGGATGTATCTGG

```
TGGCGCGCACCACCGAGAAGAAACGCGAGGAGGTGGTGGAACTTCTGGAA
AAGCTGCGTCCACTAATGCTGATCAACAGCATTAAATTCATTGCCTTCAA
CCCCACTTTTGTGTTCATGGACCGTTACAGCTCTTCGGTAATCTCACCCA
TACTGACTTCAGGCTTTAGTGTGTTAACCATCCTAATCCTTACCTTCTTC
CTGGTCATCAACCCCTTGGGAAATCTATGGCTCATTTTGACAGTCACCTC
TGTGGAGTTAGGGGTTCTGGGTTTGATGGGCTACCACCCTTTTGAGTGGC
AGCCGGCACTTAAGAACGTGTCCGCCTCCTGCCACGTGGGCATCATCAAC
GGGCTCTCTGGGTGGGCCGCGTCCGTAGACGACGTTCCCATGGAGACCAT
CACTCGACGGTTTCGTTACGACGTAGCCCTGGCGTCGGCCTTGAAGGACC
TGGAGGAGGACATCATGGAGGGCTTGTCAGAGCACCTGCTGGACGACAGC
TCTTGCAACACAGGCTTCAACGTTATGATAAAGGAGTCCTGTGATGGCAT
GGGGGATGTCAGTGAGAAGCATGGTGGGGGGCCCGCGATTCCAGAGAAGG
CCGTACGATTCTCTTTCACCATTATGTCGGTCTCTGTGCTGCCTGAGGGA
AGGGAA------------------------------GAGGCGGTTACCAT
TTTCACAGAGCCCAAACCCAACTCTGAATTGTCCTGCAAGCCCCTGTGCC
TGATGTTTGTGGACGAGTCGGACCATGAGATGCTCACTGCTGTCCTGGAT
CCTTTGCTGGCCGAGCGGAATGCCATGAAACACAGCCGACTCATCCTATC
TATAGGCGGCCTGCCTCGCTCTTTCCGCTTCCACTTCAGAGGCACTGGCT
ACGATGAGAAGATGGTGCGCGAGATGGAGGGCCTAGAGGCCTCTGGCTCC
ACCTACATCTGCACCCTGTGTGACTCCACCAGAGCAGAGGCCTCCCAAAA
CATGGTGCTCCACTCCATCACCCGCAACCACGACGAGAATCTGGAGCGCT
ACGAAATCTGGCGGACCAATCCCTTCTCGGAGTCCGCGGAAGAGCTGCGG
GACCGGGTGAAAGGGGTTTCCGCCAAGCCATTCATGGAGACCCATCCCAC
TTTAGATGCGCTACACTGCGACATCGGCAACGCCACCGAGTTTTACAAAA
TCTTCCAGGATGAGATTGGGGAGGTTTTCCAGAAGCC---CAAC---CCC
ACTCGGGAGGAACGTCGGAGCTGGCGGGCGTCGCTGGACAAGCAGCTGAG
GAAGAAGATGAAGCTCAAGCCGGTGATGAAGATGAACGGGAACTACGCGC
GGAAACTAATGACCGAGGAAGCTGTGGAGGTCGTGTGTGAGCTGGTGCCC
TCTGAAGAACGCCGGCAAGCTCTTAGAGAGCTCATGGGTCTCTACCTACA
AATGAAACCCGTCTGGCGTGCCACCTGCCCTGCCAAGGAGTGCCCGGAAC
AACTGGGCCGCTACAGTTTCACCTCGCAACGTTTTGCCGAAATCCTCTCA
TCCACTTTCAGGTACGGGTACAATGGAAAGATTACCAATTACCTGCACAA
AACCCTGGCTCACGTACCTGAAATCATAAAGAGAAAAGGCTCGATCGGAG
CCTGGGCCAGCGAGGGCAATGAGTCGGCAAACAAA---------------
--------------------------------------------------
--------------------------------------------------
--------------------------------------------------
--------------------------------------------------
--------------------------------------------------
--------------------------------------------------
--------------------------------------------------
--------------------------------------------------
--------------------------------------------------
--------------------------------------------------
--------------------------------------------------
--------------------------------------------------
--------------------------------------------------
--------------------------------------------------
--------------------------------------------------
--------------------------------------------------
--------------------------------------------------
--------------------------------------------------
--------------------------------------------------
```


---------------------------GCCAAATCTCGATTTCACCCTGG
CGTAGGGACTGGTCCTGGCACTGAGC---GCAGCGTCCCACTCAGCAACA
GCTTGCTGTCTCCGCAACAAACCGAGGAGCCCACTGTTG---CCTCCCCA
CAGCGATGGTTTGTCACCC---CTGCCAACAACCGACTGGACTTCGCTGC
TTCGGCATACGATGCCGCT---------GATTTCGCCGGCAACGCGGCCA
CGTTGCTGTCCTACGCAGCGGCTGGAGTGAAGGCGC------TTCCCTTG
CCGGCTGCAGGTTGCTCCAACAGGCCTCTTGGGTATTACGCAGACCCATC
TG---GCTGG---GGCGCCCGCACTCCGCCACAGTACTGTACC------A
GTAAACCTAGCACCGTGCTCTCGTGCTGGCCT---AACTCTGTTGGCAGC
AGAGCGGGCT------CAAACTACCTGG---------CTGAGGA---CGG
A---GATGC---CATCCCGACAGAGAGGTCCCCG---AT---TGGTGGGA
CMGAGGAG---ACAAAACCCAAAGACCT------GTCGGA---ATCCAGC
TGGATAGAG---ACGCCGTCTTCGATCAAATCAATTGATTCAAGTGATTC
TGGGATCTTTG---AACAAGCTAAACGGAGAAGAATTTCTCCGTCAGCCA
CACCG---------GTTGCAGAAACTGTGTCCCCGTTAAAATCTGAACAT
CACTCAACAGGCGAAGTAACAGAACGAGAAGTCGCGTTGGGGATAAATCC
GTTCGCCGACGGGATGGGCGCCTTCAAGATCAACCACAGTTCCCATGACA
TCGGCTCCGG---ACAGACAGCGTTTTCCTCCCAGGCG---CCCGGGTAC
---GCAGCGGCGGCGCTGGGA---CATCATCA------CCACCCATCACA
CGTCAGCTCC---TACTCCACGGCTGCGTTCAACTCCACCCGGGACTTTC
TCTTTAGAAATCGTGGCTTCGGAGAAGCTGCGAG------------CGCG
CAGCACAGCTTGTTTGCATCCGC---CGCGGGAAGTTT---C------GC
GGGGCCACATGGACACTCAGATGCCGCGGGGCACCTGCTCTTCCCGGGGC
TCCATGAG---CAAGCCGCTACGCACGCGTCTTCGAACGTGGTCAACAGC
CAGATGAGGCTAGGCTTTTCCGGGGACATGTACGGCCGGGCTGACCAGTA
CGGCCACGTTACCAGCCCGAGGT---CCGACCACTACGCTTCGACGCAGT
TGCACGGCTATGGACCCATGAATATGAACATGGCCGCG---CACCACGGC
GCTGGGGCCTTCTTTCGGTACATGAGGCAGCCGATAAAACAAGAGCTCAT
CTGCAAGTGGATCGAACCGGAACAATTGACGAACCCCAAAAAGTCGTGTA
ACAAAACTTTCAGCACAATGCATGAGCTGGTGACCCACCTGACTGTGGAG
CATGTGGGGGGCCCAGAACAGTCGAACCACGTTTGTTTCTGGGAAGATTG
CTCTAGAGAAGGAAAACCGTTCAAAGCAAAATACAAACTAGTCAATCACA
TCCGAGTGCACACCGGAGAGAAACCATTTCCGTGCCCGTTCCCCGGCTGT
GGCAAA
>Aplochiton taeniatus
NNNNNNNNNNNNNNNNNNNNNNNNNNNNNNNNNNNNNNNNNNNNNNNNNNNNNCCAGATTTATAATGTTA
TCGTCACGGCACACGCTTTCGTGATAATCTTTT
TTATAGTTATGCCAATTATGATTGGGGGCTTTGGGAACTGATTAATTCCA
CTTATGATCGGGGCCCCCGATATGGCATTCCCTCGGATAAACAATATGAG

CTTCTGGCTTCTTCCCCCCTCTTTCCTCCTCTTGCTTGCCTCTTCTGGGG
TTGAAGCAGGGGCTGGAACAGGGTGAACGGTATACCCCCCACTGGCGGGC
AACCTAGCGCACGCTGGCGCCTCTGTGGATCTCACTATTTTTTCTCTTCA
CTTAGCCGGCATTTCTTCTATTCTAGGGGCCATTAACTTTATCACGACCA
TTATTAACATGAAACCCCCCGCAATTTCCCAGTACCAGACACCCCTATTT
GTGTGGGCCGTGCTAATTACAGCCGTGCTCCTGCTCTTGTCCCTCCCAGT
TCTTGCTGCCGGAATCACTATGCTACTTACGGACCGAAACCTAAATACGA
CTTTCTTTGACCCTG-----------------------------------

NNNNNNNNNNNNNNNNNNNNNNNNNNNNNNNNNNNNNNGGCTCATCCTCAACGAGGCCGAGGTGAT
CCTGGCGCTGGCCCAGGAGTTCCAGATGCGGGTGGTGACGGTGTCCCTGG
AGGACCAGTCCTTCCCCGGCATCGTCCAGGTCCTCAGCCAAGCTTCCATG
CTGGTGAGCATGCACGGCGCCCAGCTCATCACCTCCCTCTTCCTCCCCCG
CGGGGCCGTGGTGGTGGAGCTCTTCCCTTACGCCGTCAACCCGGAGCAGT
ACACGCCTTACAAGACCCTGGCCTCCCTGCCAGGCATGGACCTTCACTAC
GCCGCCTGGAGAAACACCATGAAGGAGAACACGGTGGCCCACCCGGAGCG
ACCCTGGGATCAGGGCGGCATCGCTCACCTGGAGAAGGAGGAGCAGGAGC
GCATCCTAGCCAGCGAGGACGTCCCCAGGCACCTGTGCTGCCGCAACCCC
GAGTGGCTGTTCCGCATCTACCAGGACACCAGGGTGGACATTCCGTCCCT
CCTGGAGCTCCTCCG---GGAGAACCTGAAG---ACCCGGCCCAGTCTGA
AGAA---GGCGCGGSCCGTCAGCACCGTCCACCCGGGACGGGTCCGCGAG
GCCCAGTGCCTGACGTCCGTCCAGGCCGCGAATGAGGCGAAGCTGACGGT
GTCGTGGCAGATCCCCTGGAACCTGAAGTACCTGAAGGTCCGGGAGGTGA
NNNNNNNNNNNNNNNNNNNNAAGAAGGATACAAGCAAGGGGACCCTGGAGGATCAAATCATTCAGGCAAA
TCCTGCACTGGAGGCTTTTGGGAATGCCAAAACGTTGAGAAACGACAACT
CTTCCCGTTTTGGTAAATTCATCCGAATTCACTTTGGAACAACTGGAAAA
TTATCCTCTGCTGACATAGAGACTTACCTTCTTGAGAAATCGCGTGTTAC
CTTTCAGCTAAAGTCAGAGAGGAACTACCATATCTTTTTCCAGATCTTGT
CCAATCAAAAGCCAGAGCTGTTGGACATGCTGCTAATCACCAACAATCCC
TATGACTACTCCTACATCTCCCAGGGAGAGGTGACCGTCGCGTCAATTAA
TGATTCCGAAGAGTTGATGGCCACTGACAGTGCCTTTGATGTGCTAGGCT
TTACTCAGGAAGAAAAAATGGGTGTCTACAAGTTGACAGGTGCCATCATG
CACTACGGCAACATGAGGTTCAAGCAAAAGCAGCGAGAGGAGCAGGCCGA

GCCAGATGGTACAGAAGCTGCTGACAAATCAGCTTACTTGATGGGCCTCA
ACTCTGCAGACCTTATTAAAGGGCTTTGTCACCCCAGAGTGAAAGTAGGC
AATGAGTATGTTACCAAAGGGCAAGGTGTAGATCAAGTCTACTAC-----

-------------------------GGCTACCACACCTTCGAGTGGCAGC
CGGCCCTGAAGAACGTGTCCCAGCACTGCCAGGKGGGCATCATCGACGGC
CTCTCTGGCTGGGCGGCCTCCGTGGACGACACCCCGGCGGACACCATCAC
CCGCCGGTTCCGCTAYGACGTGGCACTGGCCTCCGCCCTGATGGACCTGG
AGGAGGACATCCTGGAGGGCCTGAGGGAGCGCGGCCTGGAGGACAGCGCC
------GCGGGCTTCAACGTCATGGTCAAGGAGTCCTGCGACGGCATGGG
GGACGTGAGCGAGAAGCACGGCGGCGGGCCGGCCATCCCCGAGAAAGCCG
TGCGCTTCTCCTTCACCGTCATGTCCGTCTCCGTCCGGGCCGAGGGGCGG
GGC------------------------------GAGGCCGTGACGGTGTT
CCGGGAGGCCAAGCCCAACTCGGAGCTCTCCTGCAGGCCCCTGAGTCTGA
TGTTCGTGGACGAGTCGGACCACGAGACGCTCACCGCCATCCTGGGGCCG
GTGGTGGGCGAGCGGGACGCCATGAAGCAGAGCCGGCTCCTCCTGACGCT
GGGGGGCCTCCAGCGCTCCTTCCGCTTTCACTTCCGGGGCACCGGTTACG
ACGAGAAGATGGTGCGCGAGCTGGAGGGCCTGGAGGCCTCGGGCTCCACC
TACGTCTGCACCCTGTGCGACTCCACGCGGGCCGAGGCCTCGTCCAACAT
GGTGCTCCACTCCATCACCCGCAGCCACGACGAGAACCTGGAGCGCTACG
AGGTGTGGCGGACCAACCCGTTCTCCGAGTCGGCCGAGGAGCTGCGGGAC
CGGGTGAAGGGGGTCTCCGCCAAGCCCTTCCTGGAGACCCAGCCCACCCT
GGACGCGCTGCACTGCGACATCGGCAACGCCACCGAGTTCTACAAGATCT
TCCAGGACGAGATCGGGGAGGTGTTCCGGAAGGC---CAAC---CCGAGT
CGGCAGGAGAGGCGCGGCTGGCGGGCGGCCCTGGACAAACAGCTCCGGAA
GAAGCTGAAGCTGAAGCCAGTCATGCGCATGAACGGGAACTACGCCCGGC
GCCTGATGACCCAGGAGGCAGTGGAGGTGGTGTGCGAGCTGGTGCCCTCC

GAGGAGAGGCGACGGGCCCTCAGTGAGCTGATGGGCCTCTACATCCAGAT
GAAGCCGGTGTGGCGCGCCTCCTGCCCGGCCAARGAGTGCCCCGACCAGC
TGTGCCGCTACAGCTTCAACTCCCAGCGCTTCGCCGAGCTCCTCTCCACC
ACCTTCAAGTACAGGTACGACGGCAAGATCACCAACTACCTCCACAAGAC
GCTGGCCCACGTGCCCGAGATCATAGAGAGGGACGGGTCCATTGGAGCGT
GGGCCAGCGAGGGCAATGAGTCGGGTAACAAG------------------

------------------------GCCAAATCTCGATTTCACCCTGGCGT
AGGGACTGGTCCTGGCACAGACC---GCAGCGTCCCACTTAGCAACAGCT
TGCTATCTCCGCAACAAACCGAAGAGCCCACAGTTG---CTTCCCCACAG
CGTTGGTTTGTCACCC---CTGCCAATAACCGACTGGACTTTGCCGCCTC
GGCATACGATGCCGCTGCTGCTGCTGATTTTGCCGGTAACGCAGCCACCT
TGCTATCCTACGCAGCGGCTGGAGTAAAGGCAT------TGCCCCTGCCC
ACAGCAGGTTGCTCAAACAGGCCACTTGGTTATTATGCCGATCCATCAG-
--GGTGG---GGCGCCCGGACTCCACCCCAATACTGT---------AGTA
AATCTAGTTCAGTACTCTCTTGCTGGCCTAGTAATTCGGTTGGTGGCAGA
ACAACTA---------ACTACCTTA---------CGGAGGA---TGTA--
-GACGC---CCTCCCCACAGAGAGGTCGCCC---AT---TGGCGGCTCCG
AGGAA---ACAAAACCTAAAGACTT------GTCAGA---ATCAAGCTGG
ATAGAG---ACACCATCTTCAATAAAGTCAATTGATTCAAGTGATTCTGG
AATCTTTG---AGCAAGCCAAACGGAGAAGAATTTCACCGTCTGCCACTC

CG---------GTTTCAGAAACTGTGTCCCCGTTGAAATCTGAA------

>Aplodinotus grunniens
AGCCTTCTTATCCGAGCAGAGCTAAGTCAACCTGGCTCACTCCTTGGAGA
TGACCAGATCTATAACGTAATTGTTACAGCACACGCCTTCGTTATAATTT
TCTTTATAGTAATACCCGTTATGATCGGAGGCTTCGGAAACTGGCTCGTA
CCATTAATGATTGGAGCCCCTGATATGGCATTCCCTCGAATAAATAACAT
GAGCTTCTGACTTCTCCCCCCTTCCTTCCTCCTGCTCTTAACCTCCTCAG
GGGTGGAAGCAGGGGCCGGGACAGGATGAACAGTCTACCCCCCACTTGCT
GGAAATCTGGCACACGCAGGTGCTTCTGTTGACTTAGCTATCTTTTCCCT
ACATCTCGCAGGTGTCTCATCAATTCTAGGGGCTATCAACTTTATCACAA
CAATTATAAACATAAAACCGCCCGCTATTTCCCAATATCAAACACCCTTG
TTCGTATGGGCCGTTTTAATTACAGCTGTTCTCCTACTACTTTCACTTCC
CGTACTAGCTGCTGGCATTACAATACTTCTGACAGACCGCAATCTTAACA
CAACCTTCTTCGACCCAGCAGGAGGGGGCGATCCAATTCTTTACCAACAC
TTA---

------------TTCCTAGAGCGAAACCTTCACCCTTCTAACTGCCTTGG
CATGTTGTTGCTGTCTGACGCCCACCAGTGCACCAAGCTGTCAGAGCTCT
CCTGGGGCATGTGCCTCAGCAACTTCCCCGCCATCTGCAAGACAGAGGAC
TTCCTCCAACTGCCCAAAGATATGGTGGTGCAGCTTTTGTCACATGAGGA
GCTAGAGACAGAAGATGAGAGACTGGTTTATGAAGCTGCCCTCAACTGGA
TCAACTATGACTTGGAAAAGAGGCACTGTCACCTGCCAGAGCTCCTGAGA
ACGGTCCGTCTGGCCTTGCTGCCTGCCATCTTTCTCATGGAGAACGTATC
TACGGAAGAGCTGATCAATGCCCAGGCCAAGAGCAAGGAGCTAGTGGACG
AAGCTATCCGCTGTAAGCTGAAGATCCTGCAGAATGATGGTGTTGTTAAC
AGCCCATGTGCTCGACCAAGAAAAACCAGCCATGCGCTCTTTCTTCTGGG
AGGGCAGACTTTCATGTGTGACAAGTTGTACCTGGTGGACCAGAAAGCCA
AAGAGATCATCCCCAAAGCTGACATTCCCAGCCCCAGGAAGGAGTTCAGC
GCCTGCGCCATCGGCTGTAAAGTGTACATCACTGGTGGAA--GAGGCTC-
AGAGAACGGTGTGTCCAAAGATGTATGGGTCTATGACACTGTCCACGAGG
AATGGTCGAAGGCGGCACCTATGCTCATCGCCAGGTTCGGCCATGGCTCT
GCAGAGCTGAAACACTGCCTCTACGTGGTAGGAGGTCACACCGCAGCAAC

TGGCTGCCTCCCAGCCTCTCCATCTGGATGAATACATTGTTGTGTTTAGT
CGTTCAACAACAAGGCTGATACTGAATGAAGCAGAGTTAATCATGGCACT
GGCCCAGGAGTTCCAGATGAGAGTGGTCACAGTATCGCTGGAGGAACAGT
CTTTCCCCAGTATCGTCCAGGTGATCAGCAGGGCTACCATGCTAGTCAGT
ATGCATGGAGCTCAGTTGATCACCTCACTGTTCCTTCCCAGAGGAGCTGT
TGTGGTGGAGCTGTTCCCCTTTGCTGTGAACCCAGAACAGTACACCCCGT
ATAAAACCCTTGCCTCCCTTCCAGGCATGGACCTTCATTATATCTCCTGG
AGGAACACGAAGGAGGAGAACACCATCACCCACCCAGACAGACCCTGGGA
ACAAGGGGGCATCGCTCACTTGGAGAAGGAGGAGCAAGAGCGAATACTGT
CGAGCAAAGATGTCCCAAGGCACCTCTGCTGCCGCAACCCTGAGTGGCTC
TTCCGGATCTACCAGGACACCTTGGTGGACATCCCTTCTTTTCTGGAAGT
CCTGAA---AGAGGGCATGAAG---ACAAAGCCCAGCTTTAAGAA---GT
CAAAGCCGGCCAGTACAGTCCATCCAGGCCGGGTCAGAGAACCCCAGTGT
CAGACCTCAGTACAAACCACTAATGAAGCTAAACTCACAGTATCCTGGCA
GATCCCATGGAATCTGAAATACCTGAAGGTCAGAGAGGTGAAGNNNNNNNNNNNNNNNNNNNAAAAAAG
ACACCAGCAAGGGGACACTGGAGGATCAAATCATCCAGGCCAACCCGGCG
CTGGAGGCATTCGGCAATGCCAAAACACTGAGAAACGACAACTCGTCTCG
TTTTGGAAAATTCATCCGAATTCACTTTGGTACGAGCGGAAAGCTGTCGT
CTGCTGACATCGAGACGTACCTGCTGGAGAAGTCACGCGTCACCTTTCAG
CTCAAAGCTGAAAGGAACTACCACATCTTCTACCAGATCCTGTCCAATCA
GAAGCCGGAGCTGCTGGACATGCTGCTCATTACCAACAACCCATACGACT
ACTCCTACATCTCCCAAGGAGAGGTGACGGTCGCCTCCATCAATGACTCC
GAGGAGCTGATGGCCACCGACAGCGCCTTTGATGTGCTCGGCTTCACTCC
AGATGAGAAGATGGGCGTCTATAAATTGACGGGCGCCATCATGCACTATG
GCAACATGAAGTTCAAACAGAAGCAGCGTGAGGAGCAGGCTGAGCCGGAC
GGCACGGAGGCTGCCGATAAATCTGCTTATCTAATGGGGCTGAACTCTGC
TGACCTCATCAAAGGGCTGTGCCATCCCAGAGTCAAGGTAGGAAATGAAT
ACGTGACAAAAGGCCAAAGTGNGGATCAGGTCTACTACCCCAACAAGGAGG
CCTTCAAGTGTGAAGAGTGTGGGAAGCACTACAACACCAAGCTGGGATAT
AAGCGCCATGTAGCCATGCACTCTGCAACGGCAGGGGATCTCACCTGTAA
AGTGTGCATGCAGACCTACGAGAGCACGCCTGTGCTGTTGGAGCACCTCA
AGAGCCACTCTGGGAAGTCTTCAGGTGGCACCAAGGAGAAGAAACACCCA
TGTGACCACTGTGACCGTCGTTTCTACACACGAAAGGATGTGAGACGGCA
CATGGTGGTCCACACGGGCCGAAAGGACTTCCTGTGCCAGTACTGTGCCC
AACGCTTCGGCAGGAAGGACCATCTGACACGCCATGTGAAGAAGAGCCAC
TCACAGGAGCTGCTAAAGATCAAGACGGAGCCTCCTGATATGTTAGGTCT
TTTAGCTTCCGGGTCACCACCCTGCTCTGTGAAGGAGGAGCTCAGCCCCA
TGATGTGCGGTATGGGTCCCAACAAAGACCCCATGATGGGAAAACCATTT
CCCAGTGGGGCCCCTTTTCCAATGGGCATGTACAACCCCCACCAT-----
-CTCCAGGCCATGTCCAATTCTGGGGTGGGTCACCCA------CACCCTT
CCCTGATGCCCAGTTCCTTGTCTGCAGCTATGGGTATGGGCTGTCACATG
GAATATCTCATCTACGCCTCTTTTTCATTCATGGGATGTTTACAAATTAG
TGAYGGATCAAATATYGTGAATCTGCTGGCTAGTAACTCTCCGAGTGTTT
CCTATGCTCTGACCCAGCAAAAATACTTCAGTAACTACAGTCCCGTGATT
GGGTTTTACATTTATGAGCCCATCGAGTACTGGAACTCAACGGTGCAGGA
GCACNNNAAGACTNTGAGTCATGGCTTCAACAAGATCTCCTGGATGGACAACTT
TTTCCACTATTTGCGGGTGGTGAATGTGAGTGCGTCAACCAAGGGTGACT
TCATCACCATCCTCAAGGGCTCTTTCCTGCGCAGCCCGGAGTACCAGCAC
TTCACTGAGGACATCATATTCTCCAAGA---ACCGTGAGACTG------A
TGAGTATGACATTATCGCCTCACGGATGTACTTAGTGGCACGGACGACAG
AGAAGAAGCGCGAAGAGGTGGTGGAGCTTCTAGAAAAGCTTCGTCCGTTG
ATGCTAATCAACAGCATCAAATTCATTGCCTTCAATCCTACGTTTGTGTT
CATGGACCGCTACAGCTCTTCTGTCATCTCNCCCATCCTGACCTCAGGATT

CAGCGTACTCACAATCCTCATCCTCACTTTCTTCTTGGTCATCAACCCCT
TGGGTAACTTCTGGCTCATTCTCACTGTAACGTCCGTGGAGCTGGGCGTC
TTGGGTTNNNNNNNNNNNNNNNNNNNNNNNNTGGCAGCCAGCTCTCAAGAATGTGTCTACATCTTGCAATG
TTG
GCATTATTAATGGGCTCGCTGGATGGGCTTCCTCAGTGGATGATTCCCCA
GCTGACACCATCACTCGGCGGTTTCGCTATGATGTGGCACTTGTGTCAGC
TTTAAAGGACCTGGAGGAGGACATCATGGAGGGGCTGAAAGAGAGTGGGA
TGGAAGACAGTGCTTGCACCTCAGGCTTCAGTGTCATGATCAAAGAATCT
TGTGATGGCATGGGCGATGTCAGTGAGAAGCACGGTGGAGGACCAGTTGT
TCCCGAGAAGGCTGTACGTTTCTCTTTCACTATTATGTCTGTCTCTGTCC
TAGCAGACGAGGAGGAG-------------------------------GAA
GAGGTTACCATCTTCACAGAGCCAAAGCCAAACTCAGAACTCTCCTGTAA
GCCCCTTTGCCTGACATTTGTAGATGAGTCAGACCATGAGACACTCACAG
CCATCCTGTGGCCTATCGTTGCAGAGCGTAACGCAATGAAAGAGAGTAGG
CTTATCCTACCCATAGGTGGACTACCTCGCTCTTTCCGCTTTCACTTCAG
AGGCACAGGATATGATGAGAAGATGGTACGTGAGATGGAGGGCCTCGAAG
CCTCAGGGTCCACCTACATCTGCACTCTTTGCGACTCCAGTCGGGCAGAG
GCCTCTCAAAATATGGTGCTACACTCCGTCACCCGCAGTCATGAAGAGAA
CTTAGAACGTTATGAAATATGGCGAACCAACCCCTTTTCTGAGTCTGTAG
AGGAGCTGCGAGAGAGAGTCAAAGGGGTTTCTGCCAAGCCTTTCATGGAA
ACACACCCCACACTGGATGCATTACACTGTGACATAGGCAATGCCACTGA
GTTCTACAAAATCTTCCAGGATGAGATCGGGGAGGTGTATGAAAAGGT--
-CAAC---CCCAGCCGGGAGGAGCGACGCAGTTGGAGGGCAGCCCTAGAT
AAACAGCTGAGAAACAAGATGAAGCTTAAACCGGTAATGAGGATGAATGG
GAACTACGCCCGCCGGCTAATGACCATGGAGGCTGTGGAGATAGTGTGTG
AGCTCGTGCCCTCAGAGGAGAGGAGGGAGGCCCTGAGGGAGCTTATGAGG
CTCTACTTGCAGATGAAGCCTGTGTGGCGCGCCACCTGCCCAGCCAAGGA
GTGCCCTGACCAGCTGTGCCGCTACAGTTTTAACTCCCAGCGCTTTGCCG
ACCTCCTCTCCTCTGCCTTTAAATATAGGTACAATGGAAAGATAACCAAT
TACCTGCACAAGACCCTGGCCCATGTGCCTGAAATCATAGAGAGAGATGG
ATCTATAGGAGCCTGGGCCAGCGAGGGGAATGAGTCTGCAAACAAATCGT
ACACCATTGAGATGGGTCCCATGGGGCCCAGGTGGAAGGAGAGCCCACAA
CCTTTCTCCTGCTCCATCGAAGACCCAACGAAACAGACAAAGTTCAAGGG
CATCAAGACGTACATATCGTACCGCGTCACACCAAGCCACACAGCGCATC
CTGTCTACAGGCGCTACAAACACTTTGACTGGCTGTACAACCGCTTACTG
CACAAGTTCACTGTGATCTCTGTGCCTCACCTGCCTGAGAAGCAGGCCAC
GGGGCGATTTGAGGAAGACTTCATCGAGAAGCGCAAGAGACGACTTATAC
TGTGGATGAACCACATGACCAGTCACCCGGTCCTCTCCCAGTATGAAGGC
TTCGAGCACTTTCTGATGTGTGCTGATGACAAGCAGTGGAAACTGGGCAA
GAGACGGGCRGAGAAGGACGAGATGGTGGGCGCCCATTTCATGCTGACCC
TGCAGATCCCTAACGAGCACCAGGACCTTCAGGATGTAGAGGAGCGGATC
GACTCCTTCAAGTCCTTTGCTAAGAAGATGGATGACAGCGTGATGCAGCT
CACACATGTTGCCTCAGAGCTTGTGCGTAAGCACCTGGGTGGGTTCAGGA
AGGAGTTCCAGCGGCTGGGGAATGCCTTCCAGTCTGTAAGCCAGGCGTTC
ATGCTGGACCCACCCCACAGCTCAGAGACCTTCAACAACGCCATCTCCCA
TNNNNNNNNNNNNNNNNCTCAAACTGACCTCTCTGGGTTTCATCATTGGAGTCGGTGTGGTTGGAA
ACCTCCTGATCTCCATCCTGCTGGTCAAAGACAAGAGCCTGCACAGAGCA
CCCTACTATTTCCTGCTGGACCTGTGCGCCTCTGATATCCTGCGCTCAGC
CATCTGCTTCCCCTTTGTCTTCACCTCGGTCAARAATGGATCTGCCTGGA
CCTATGGCACGCTGACCTGCAAAGTGATCGCCTTTCTCGGTGTGCTCTCC
TGTTTCCACACAGCGTTTATGCTCTTCTGTGTCAGTGTCACACGCTACCT
GGCCATCGCACATCATCGTTTCTACACCAAGAGGCTGACCTTCTGGACCT
GTCTAGCTGTCATCTGCATGGTGTGGACGTTGTCAGTAGCTATGGCATTC

CCGCCGGTGCTGGACGTAGGGACGTACTCTTTTATCCGGGAGGAGGACCA
GTGCACATTCCAGCACCGTTCCTTCAGGGCGAATGATTCGCTGGGCTTCA
TGCTCCTGCTGGCGCTCATCCTCCTGGCCACACAGCTGGTTTACCTCAAG
CTCATCTTCTTCGTCCACGACCGTCGAAAGATGAAGCCTGTCCAGTTCGT
GCCTGCTGTCAGCCAGAACTGGACCTTTCACGGGCCAGGCGCCAGCGGGC
AGGCGGCGGCCAACTGGCTGGCTGGATTTGGTCGAGGCCCCACCCCGCCT
ACTTTGCTGGGCATCCGGCAGAACAGCAACGCAGCAGGCCGCAGGCGTCT
ACTGGTGTTGGATGAATTCAAAACAGAGAAGAGGATTAGTAGGATGTTCT
ACATCATGACGTTTTTCTTCCTGGCACTCTGGGGGCCATATCTGGTCGCC
TGCTACTGGCGGGTGTTTGCAAGGGGCCCGTTGGTCCCTGGGGGCTACCT
GACGGCAGCCGTGTGGATGAACTTTGCCCAGGCTGGGGTCAATCCTTTCA
TCTNNNNNNNNNNNNNNNNNNNNNGCCAAATCTCGCTTTCACCCTGGCATGGGGACTGGTCCTGGCACGGA
GC---GCAGCGTCCCACTCGGCAACAGCTTGCTATCCCCGCAGCAAACCG
AGGAGCCCACTGTTGCCACCCCCCCGCAGCGATGGTTTGTCACCC---CT
GCCAACAACCGACTGGACTTTGCTGCCTCGGCATACGACGCCGCT-----
----GATTTCGCCGGTAACGCGGCCACCTTGCTGTCCTACGCAGCGGCCG
GAGTGAAGGCTC------TTCCCCTGCCGACTGCAGGCTGCTCCAACCGG
CCTCTTGGCTATTACGCAGACCCGTCAG---GCTGG---GGAGGACGCAC
GCCGCCGCAGTACTGTGGTGTAAATAGCAAACCCAGCTCGGTCTTTTCCT
GCTGGCCCACTAACTCTATCGGTGGCAGAGCTAGTA---CC---AACTAC
CTGG---------CCGAGGA---GGGA---GACTC---CATCCCGACAGA
GAGATCACCG---AT---CGGCGGCACAGAGGAG---ACCAAACCCAAAG
ACATGAC---ATCAGA---GTCGAACTGGATAGAG---ACGCCGTCCTCC
ATTAAGTCCATTGATTCAAGCGATTCTGGTATCTTTG---AACAGGCCAA
AAGGAGAAGGATCTCACCTTCTGCCACGCCG---------GTGTCAGAGA
CAGTGTCCCCGTTAAAATNNNNNCATCACTCAACAGGCGAAGTCACAGAGAGAGA
AGTGGCGTTGGGGATAAATCCGTTCGCGGATGGGATGGGCGCCTTCAAAA
TAAACCACAGCTCCCATGATATTGGCTCCGG---ACAAACGGCGTTTTCC
TCCCAGGCG---CCCGGCTAC---GCGGCAGCCGCCCTGGGA---CACCA
TCA------CCACCCGACCCACGTTGGCTCT---TACTCCACGGCGGCTT
TCAACTCCACCAGGGACTTTCTCTTCAGAAATCGGGGATTCGGGGACGCC
ACCGG------------CGCGCAGCACAGTTTGTTCGCCTC---------
CGGAAGTTT---C------GCAGGGCCACATGGACACTCAGATGCAGCGG
GGCACCTGCTCTTCCCGGGGCTCCACGAG---CAAGCGGCGAGCCATGCG
TCTTCCAATGTGGTCAACAGCCAGATGCGGCTGGGCTTCTCGGGGGACAT
GTACGGACGGGCYGACCAGTACGGCCACGTTACAAGCCCGCGGT---CCG
ACCACTATGCTTCGACCCAGCTGCACGGCTACGGCCCCATGAACATGAAT
ATGGCCGCA---CATCATGGAGCAGGGGCCTTCTTTCGATACATGAGGCA
GCCGATCAAACAAGAGCTCATCTGCAAGTGGGTCGAGCCGGAGCAGCTGA
CGAATCCCAAAAAGTCGTGCAACAAAACTTTTAGCACGATGCACGAGCTC
GTGACCCATCTGACGGTGGAGCATGTGGGAGGACCGGAGCAAACGAACCA
CATCTGCTTCTGGGAGGAATGCGCCAGAGAAGGAAAGCCATTCAAAGCCA
AATACAAACTTGTAAATCATATCAGAGTACACACCGGAGAAAAGCCGTTT
CCGTGTCCGTTCCCGGGCTGTGGCAAA
>Apogon lateralis


```
--------------------------------------------------------
--------------------------------------------------------
--------------------------------------------------------
--------------------------------------------------------
--------------------------------------------------------
--------------------------------------------------------
--------------------------------------------------------
--------------------------------------------------------
------------TTTCTTGAAAGAAACCTCCACCCATCTAACTGTCTAGG
TATGCTGTTGCTCTCTGATGCCCACCAGTGTACCAAGCTGTCAGAGCTCT
CCTGGGGCATGTGCCTCAGCAACTTCCCTGCTATTTGCAAGACAGAGGAC
TTCCTCCAACTGCCCAAAGACATGGTWGTGCAGCTTTTGTCACATGAGGA
GCTAGAGACAGAAGATGAGAGGCTGGTTTATGAAGCTGCTCTCAACTGGA
TCAACTATGATCTGGAAAAGCGGCATTGCCACCTCCCAGAGCTCCTGAGA
ACGGTCCGCCTTGCCCTGCTGCCYGCTATTTTTCTGATGGAAAACGTCTC
AACAGAGGAGTTGATCAATGCCCAGGCCAAGAGCAAGGAGCTGGTGGACG
AGGCAATTAGGTGTAAACTGAAGATCCTGCAGAATGATGGYGTCGTTAAC
AGTCCATGYGCTCGACCACGAAAAACTAGCCATGCACTCTTTCTTCTTGG
AGGGCAGACGTTCATGTGTGACAAGTTGTACTTGGTGGACCAGAAGGCCA
AAGAGATAATCCCAAAGGCAGACATCCCCAGTCCCAGAAAGGAGTTCAGT
GCCTGCGCCATTGGCTGTAAGGTGTATATAACAGGAGGAA--GAGGCTC-
TGAGAATGGTGTGTCCAAAGATGTATGGGTCTATGACACAGTTCATGAAG
AATGGTCCAAGGCGGCACCCATGCTCATCGCCAGATTTGGCCATGGTTCT
GCAGAGCTCAAACACTGCCTCTACGTGGTGGGAGGTCACACTGCAGCAAC
TGGATGCCTCCCAGCCTCCCCGTCAGGACGAATACATCGTTGTCCTTAGT
CGATCAACAACAAGGCTCATCCTCAACGAAGCAGAGCTGATTATGGCGCT
GGCTCAGGAATTCCAGATGAGGGTAGTCACAGTGTCCCTGGAGGAGCAGT
CTTTCCCCAGTATCGTCCAGGTGATCAGTGGGTGTTCTATGTTGGTCAGT
ATGCACGGAGCCCAGCTCATCACCTCACTCTTCCTCCCCAGAGGCGCCGT
TGTGGTTGAGCTGTTCCCATTTGCTGTGAACCCTGAACGGTACACACCTT
ATAAAACCTTAGCATCCCTTCCTGGCATTGATCTTCACTATATTTCATGG
AGAAACACTGAGGAGGAAGATACCGTTACCCACCCAGACCGGCCCTGGGA
ACAAGGAGGCATCAGCCACTTGGAAAAAGATGAGCAGGAGCGAATTCTGG
CCAGCAAAGACGTCCCGAGGCACCTGTGCTGCCGCAACCCGGAGTGGCTT
TTCCGGATCTACCAGGACACTTTAGTAGATATACCTTCATTCTTGGACGC
CCTCAA---AGAGGGCATGAAG---ACCAAGCCTGTCTTGAAGAA---GT
CCAAGCCGGCCAGCACGGTCCATCCAGGCCGGGTCAGAGAGCCTCAGTGT
CAGACGTCGGTACAAACAACCAGCGAGGCCAAGCTCACAGTGTCCTGGCA
GATCCCGTGGAATCTGAAATACCTGNNNNNNNNNNNNNNNNNNNNNNNNNNNNNNNNNNNNNAAAAAAGAT
GCGAGTAAGGGAACAC
TAGAGGATCAAATCATCCAAGCAAACCCTGCATTGGAGGCATTTGGCAAT
GCCAAAACAGTAAGAAATGACAACTCATCTCGTTTTGGAAAATTCATCCG
CATTCATTTCGGAACAAGCGGCAAGTTGTCATCAGCTGACATTGAGACTT
ACTTGCTGGAGAAGTCACGTGTCACCTTTCAGNTAAAATCTGAGAGGAATT
ATCATATCTTYTTCCAGATCCTGTCCAATCAGAAGCCAGAGCTGTTGGAC
ATGTTGCTGATCACCAACAACCCATATGACTACTCCTACATCTCTCAAGG
AGAGGTGACAGTTGCTTCCATCAATGATTCGGAGGAGCTAATGGCCACCG
ACAGTGCCTTTGATGTGCTTGGCTTCACATCAGAGGAGAAGACGGGTGTG
TACAAACTGACCGGTGCCATCATGCACTATGGAAACATGAAGTTCAAACA
GAAGCAGCGTGAAGAGCAGGCCGAGCCTGACGGAACAGAGGCGGCTGATA
AATCAGCTTACCTAATGGGACTGAACTCTGCTGACCTCATTAAAGGGCTG
TGCCACCCCAGGGTCAAAGTAGGAAATGAATATGTCACCAAAGGTCAGAG
TGTGGACCAGGTCTACTACCCCAACAAGGAGGCCTTCAAGTGTGAGGAGT
```

GTGGGAAGCACTACAACACCAAGCTGGGATATAAGCGCCATGTGGCCATG
CACTCTGCCACTGCAGGGGATCTCACTTGTAAAGTGTGCATGCAGAGCTA
CGAGAGCACACCTGTGCTCCTGGAGCACCTCAAGAGCCACTCGGGGAAAT
CCTCAGGTGGCGCCAAGGAGAAAAAACACCCATGTGACCACTGTGACCGC
CGATTCTACACGAGGAAGGATGTGAGGCGGCACATGGTGGTCCACACGGG
CAGGAAGGACTTTCTGTGCCAGTACTGTGCCCAGCGCTTCGGCAGGAAGG
ACCACCTGACACGCCACGTAAAGAAGAGCCACTCACAGGAGCTGCTGAAG
ATCAAAACAGAGCCTCCAGATATGTTAGGTCTATTAGCGTCTGGGTCACC
TCCTTGCTCTGTGAAGGAGGAGCTTAGTCCCATGATGTGTGGCATGGGTC
CCAACAAAGACCCTATAATGGGAAAACCGTTCCCCAGTGGAGCCCCCTTT
CCTATGGGCATGTACAACCCCCACCAT------ATCCAGGCCATGTCTAA
TTCTGGTGTGGGTCACCCA------CACCCTTCCCTAATGCCCAGTTCCT
TGTCTGCAGCTATGGGCATGGGTTGTCATGTGGATNNNNNNNNNNNTGCATCTTTTTCATT
CATGGGATGTTTACAAATTAGTGATGGATCCAATATTGTGAACCTGCTTG
CTAGTAACTCTCCGAGTGTTTCATATGCTCTGACCCAACAGAAATACTTC
AGTAACTACAGTCCTGTGATTGGGTTTTATATTTATGAACCCATCGAATA
CTGGAACTCAACAGTGCAAGAGCACCTAAAGACACTGAGTCATGGTTTCA
ACAAGATCTCCTGGATGGACAACTTTTTCCACTACCTGCGGGTGGTGAAT
GTGAGCGCATCAACCAAAAGCGACTTCATCACTATCCTCAAGGGCTCCTT
TCTGCGCAGCCAGGAGTACCAGCACTTCACTGAGGACATCATATTCTCCA
AGA---ACCGTGAGACCA------ACGAGTATGACATTATTGCCTCACGC
ATGTACTTAGTGGCCCGAACCACCGAGAAGAAGCGTGAGGAGGTTGTGGA
GCTTTTGGAAAAGCTTCGCCCATTGATGTTGATCAACAGCATCAAGTTCA
TTGCCTTCAATCCCACGTTTGTGTTCATGGACCGCTACAGTTCCTCTGTC
ATCTCTCCCATTCTGACCTCAGGCTTCAGTGTGCTCACCATCCTCATCCT
CACTTTCTTCCTGGTCATCAACCCCCTGGGAAACTTCTGGCTCATCCTGA
CGGTAACATCCGTCGANNNNNNNNNNNNNNNNNNNNNNGGCTTTCACCAGTTTGAATGGCAGCCAGCTCT
CA
AGAATGTGTCTGCATCTTGCAATGTTGGCATTATTAATGGGCTTTCTGGA
TGGACTTCTACAGTGGACGAGTCCCCGGCTGACACAATCACACGGCGCTT
TCGCTATGATGTGGCACTGGTATCAGCATTAAAGGATCTGGAGGAGGACA
TCATGGAAGGGCTGCAAGAGAGTGGGATGGAAGACAGTGCATGCACCTCG
GGCTTCAGTGTTATGATCAAAGAATCGTGTGATGGCATGGGTGATGTCAG
CGAGAAGCATGGCGCAGGACCTGTTGTTCCCGAGAAGGCTGTTCGTTTCT
CTTTCACGATTATGTCTGTGTCTGTCAAGGCAGAAGGAGAGGAG------
---------------------------GAGGTTACCATCTTCACAGAGCC
GAAGCCAAACTCAGAACTGTCTTGTAAGCCCCTCTGCCTCACATTTGTGG
ATGAATCAGACCACGAGACGCTCACAGCTGTCTTGGGGCCGATAGTTGCA
GAGCGCACTGCAATGAAAGAGAGCAGACTCATCGTATCCATAGGTGGCCT
CCCTCGATCCTTCCGCTTYCACTTCCGAGGCACAGGATATGATGAGAAGA
TGGTCCGTGAGATGGAGGGCCTTGAGGCTTCGGGTTCCTCTTACGTCTGC
ACGCTGTGTGACACCAGTCGAGCAGAGGCCTCTCAGAATATGGTGCTGCA
CTCAGTCACCCGCAGTCATGATGAAAACCTAGAACGCTATGAAATTTGGA
GAACCAACCCTTTTTCAGAGTCTGCAGATGAACTGAGAGACAGAGTCAAA
GGGGTCTCCGCTAAACCTTTCATGGAGACCCAGCCTACAATAGATGCATT
GCACTGTGACATTGGTAATGCCATTGAGTTCTACAAAATCTTTCAGGATG
AAATAGGTGAGATGTATCAAAAGGT---CAAC---CCCAGCCGGGAGGAA
CGACGCAGCTGGAGGGCAGCTTTAGATAAACAGCTGAGGAAGAAGATGAA
GCTTAAACCAGTAATGAGGATGAATGGGAACTATGCCCGCAAGCTAATGA
CCCAGGAGACTGTGGAGGTGGTGTGTGAGTTGGTGCCCTCTGAGGAAAGG
AGGGAGGCCCTGAGGGAGCTGATGAGGCTCTACCTGCAGATGAAACCTGT
GTGGCGTGCCACCTGCCCTGCAAAAGAATGCCCTGACCAACTGTGCCGCT
ACAGCTACAACTCCCAGAGCTTTGCTGACCTTCTTTCCTCTACATTCAAA

TACAGGTACAATGGAAAGATAACCAACTACCTGCACAAGACCTTGGCTCA
CGTTCCTGAAATCATAGAGAGAGATGGATCCATAGGAGCCTGGGCCAGTG
AGGGAAACGAGTCAGCAAACAAATCATATACTATAGACATGGGCCCCTTG
GGACCCGGCTGGAAGGAGAGCCCACAGCCTTTCTGCTGCTCCATTGAAGA
CCCCACAAAACAAACTAAGTTCAAAGGCATCAAGACGTACATTTCGTACC
GTGTGACGCCGAGCCACACAGGGCGTCCCGTCTACAGACGCTACAAACAC
TTTGACTGGCTGTACAACCGCCTGCTGCACAAATTTACTGTGATCTCTGT
TCCTCACCTGCCTGAGAAGCAGGCCACCGGGCGATTTGAGGAAGACTTCA
TTGAAAAGCGCAAAAGACGACTGATACTATGGATGAACCACATGACCAGT
CACCCAGTACTCTCCCAGTATGAAGGCTTTGAGCACTTTCTGATGTGTGC
TGACGACAAGCAATGGAAACTGGGCAAGAGACGAGCCGAGAAGGACGAGA
TGGTGGGCGCTCACTTCATGCTGACGCTTCAGATTCCGAACGAGCACCAG
GACCTTCAGGATGTAGAGGAGCGTGTGGACTCCTTCAAGTCCTTTGCTAA
GAAAATGGACGACAGTGTGATGCAGCTCACACATGTTGCATCAGAGCTGG
TGCGTAAACATTTGGGTGGATTCAGGAAGGAGTTCCAGCGGCTGGGAAAT
GCCTTCCAGTCCATCAGCCAGGCCTTTATGCTTGACCCTCCTCATAGCTC
TGAGGCCCTCAACAACGCCATCTCCCACNNNNNNNNNNNNNTTCCTCAAACTGACCTCTCTGG
GTTTCATCATTGGAGTCGGTGTGGTTGGGAACCTCCTGATCTCCATCCTG
CTGGTTAAAGACAAGAGCCTGCACAGAGCGCCCTACTATTTCCTGCTGGA
CCTGTGCGCCTCTGATATCCTGCGTTCTGCCATCTGCTTCCCCTTTGTCT
TCACCTCRGTTAAGAATGGATCTGCCTGGACCTACGGCACGCTGACCTGT
AAAGTGATTGCCTTCCTGGGGGTGCTCTCCTGTTTCCATACGGCATTCAT
GCTATTCTGTGTCAGTGTTACCCGCTACCTGGCCATAGCACATCATCGTT
TCTACACCAAGAGGCTAACCTTCTGGACCTGTCTGGCTGTCATCTGTATG
GTCTGGACGTTGTCTGTTGCTATGGCATTCCCGCCGGTGCTAGATGTAGG
GACGTACTCTTTTATACGGGAGGAGGACCAATGCACATTCCAGCACCGCT
CCTTTAGGGCGAATGATTCGCTGGGCTTCATGCTCCTGCTGGCGCTTATC
CTCCTGGCCACACAGCTGGTTTACCTCAAGCTCATCTTCTTCGTCCACGA
TCGTCGAAAGATGAAACCAGTCCAGTTCGTGCCTGCAGTCAGCCAGAACT
GGACCTTCCACGGGCCAGGGGCCAGCGGGCAGGCAGCGGCTAACTGGCTG
GCTGGATTTGGTCGAGGCCCCACCCCGCCTACTTTGCTGGGCATCAGGCA
GAACAGCAACGCAGCGGGCCGCAGGCGTCTACTGGTGTTGGATGAGTTCA
AAACGGAGAAGAGAATTAGTAGGATGTTCTACATCATGACGTTTTTCTTC
CTGGCTCTGTGGGGGCCCTATCTGGTAGCCTGTTACTGGAGGGTGTTTGC
AAGGGGCCCCGTTGTCCCTGGGGGCTACCTGACAGCGGCCGTGTGGATGA
GCTTTGCCCAGGCTGGGGTCAATCCTTTCATCTGCNNNNNNNNNNNNNNNNNNNGCCAAATCTCGCTTT
CACCCTGGCGTGGGGACTGGTCCTGGCACGGAGC---GCAGCGTCCCACT
CGGCAACAGCTTGCTATCCCCGCAGCAAACCGAGGAGCCCACTGTTGCCA
CCCCCCCGCAGCGATGGTTTGTCACCC---CTGCCAACAACCGACTGGAC
TTTGCTGCCTCGGCATACGACGCCGCT---------GATTTCGCCGGTAA
CGCGGCCACCTTGCTGTCCTACGCAGCGGCCGGAGTGAAGGCTC------
TTCCCCTGCCGACTGCAGGCTGCTCCAACCGGCCTCTTGGCTATTACGCA
GACCCGTCAG---GCTGG---GGAGGACGCACGACGCCGCAGTACTGTGG
CGTAAATACCAAATCCAGCTCGGTCTTTTCGTGCTGGCCCGCTAACTCTC
TCGGCGGCAGAGCAGGCC---CC---AACTACCTGG---------CCGAG
GA---GGGA---GACTC---GATCCCGACGGAGAGGTCACCG---AT---
CGGCGGCTCGGAGGAG---ACCAAACCCAAGGACAT------GTCAGA--
-ATCCAGCTGGATAGAG---ACACCGTCTTCTATTAAGTCCATTGATTCG
AGTGATTCTGGGATCTTTG---AACAGGCCAAAAGGAGAAGGATCTCACC
TTCTGCCACACCA---------GTTTCAGAGACAGTGTCCCCATTAAAAT
CGGAGNNNNNNNNNNNNNNNNNNNNNNACAGAGAGAGAAGTGGCGTTGGGGATAAATCCATTCGCGGATGG
G
ATGGGCGCCTTCAAAATAAACCACAGCTCCCACGATATTGGCTCCGG---

ACAGACGGCGTTTTCCTCCCAGGCG---CCCGGTTAC---GCAGCAGCCG
CCCTGGGA---CACCACCA------CCACCCGACCCACGTTGGCTCT---
TACTCCACAGCGGCTTTCAACTCCACCAGGGACTTTCTGTTCAGAAATCG
GGGTTTCGGGGACGCCACCGG------------GGCTCAGCACAGTTTGT
TCGCCTC---------CGGAAGTTT---C------GCAGGGCCACATGGA
CACTCAGATGCAGCGGGGCACCTGCTCTTCCCGGGGCTCCACGAG---CA
AGCGGCGAGCCACGCGTCTTCCAACGTGGTCAACAGCCAGATGCGGCTGG
GCTTCTCGGGGGACATGTACGGACGAGCCGACCAGTATGGCCACGTTACA
AGCCCACGCT---CCGACCACTACGCCTCGACCCAGCTCCACGGCTACGG
CCCCATGAACATGAATATGGCCGCG---CACCACGGAGCAGGGGCCTTCT
TTCGATACATGAGGCAGCCCATCAAACAAGAGCTCATCTGCAAGTGGATC
GAACCGGAGCAGTTGACAAATCCTAAAAAGTCTTGTAACAAAACTTTTAG
CACCATGCACGAGCTGGTGACCCATCTGACGGTGGAGCATGTGGGGGGAC
CAGAGCAGACGAACCACATCTGCTTCTGGGAGGACTGTGCCAGAGAAGGA
AAGCCATTCAAAGCCAAATACAAACTTGTAAATCATATCAGAGTACACAC
CGGAGAAAAGCCCTTTCCCTGTCCGTTCCCCGGCTGTGGCAAA
>Archamia biguttata

-----------AGACTGGTNTCTTGCAAGGGAACACTGGAGGATCAAATCA
TCCAGGCAAACCCTGCGTTGGAGGCATTTGGCAATGCCAAAACAGTGAGA
AATGACAACTCATCTCGTTTTGGAAAATTCATCCGCATTCATTTCGGAAC
AAGCGGCAAGTTGTCATCAGCTGACATTGAGACTTACTTGCTGGAGAAGT
CACGTGTCACCTTTCAGCTAAAATCTGAGAGGAATTATCATATCTTCTTC
CAGATCCTGTCCAATCAGAAGCCAGAGCTGTTGGATATGTTGCTGATCAC
CAACAACCCATATGACTACTCCTACATCTCTCAAGGAGAGGTGACAGTTG
CCTCCATCAATGACTCGGAGGAGCTAATGGCCACTGACAGTGCCTTTGAT
GTGCTTGGCTTCACATCAGAGGAGAAGACGGGTGTGTACAAACTGACTGG
TGCCATCATGCACTATGGAAACATGAAGTTCAAACAGAAGCAGCGTGAAG
AGCAGGCCGAGCCTGACGGGACAGAGGCGGCCGATAAATCAGCTTACCTA
ATGGGGCTGAACTCTGCTGACCTCATTAAAGGGCTGTGCCACCCCAGGGT
CAAAGTAGGAAATGAATATGTCACCAAAGGTCAGAGTGTGGACCCAGTCT
ACTACCCCAACAAGGAGGCCTTCAAGTGTGAGGAGTGTGGGAAGCACTAC
AACACCAAACTGGGATATAAGCGCCATGTGGCCATGCACTCTGCCACTGC
AGGGGATCTCACTTGTAAAGTGTGCATGCAGAGCTACGAGAGCACACCTG
TGCTCCTGGAGCACCTCAAGAGCCACTCGGGGAAATCCTCAGGTGGCGCC
AAGGAGAAAAAACACCCATGTGACCACTGTGACCGTCGATTCTACACGAG
GAAGGATGTGAGGCGGCACATGGTGGTCCACACGGGCAGGAAGGACTTTC
TGTGCCAGTACTGTGCCCAGCGCTTCGGCAGGAAGGACCACCTGACGCGC
CACGTGAAGAAGAGCCACTCACAGGAGCTGCTGAAGATCAAAACAGAGCC
TCCAGATATGTTAGGTCTATTAGCTTCTGGTTCACCTCCTTGCTCTGTGA
AGGAGGAGCTTAGTCCCATGATGTGTGGCATGGGTCCCAACAAAGACCCT
ATAATGGGAAAACCGTTCCCCAGTGGAGCCCCCTTTCCTATGGGCATGTA
CAACCCCCACCAT------ATCCAGGCCATGTCTAATTCTGGTGTGGGTC
ACCCA------CACCCTTCCCTGATGCCCAGTTCCTTGTCTGCAGCTATG
GGCATGGGTTGTCATGTGGAT-----------------------------


```
--------------------------------------------------
--------------------------------------------------
--------------------------------------------------
--------------------------------------------------
--------------------------------------------------
--------------------------------------------------
--------------------------------------------------
--------------------------------------------------
--------------------------------------------------
--------------------------------------------------
--------------------------------------------------
--------------------------------------------------
--------------------------------------------------
--------------------------------------------------
--------------------------------------------------
--------------------------------------------------
--------------------------------------------------
--------------------------------------------------
--------------------------------------------------
--------------------------------------------------
--------------------------------------------------
--------------------------------------------------
--------------------------------------------------
--------------------------------------------------
--------------------------------------------------
--------------------------------------------------
--------------------------------------------------
--------------------------------------------------
--------------------------------------------------
--------------------------------------------------
--------------------------------------------------
--------------------------------------------------
--------------------------------------------------
--------------------------------------------------
--------------------------------------------------
--------------------------------------------------
--------------------------------------------------
--------------------------------------------------
--------------------------------------------------
--------------------------------------------------
--------------------------------------------------
--------------------------------------------------
--------------------------------------------------
--------------------------------------------------
--------------------------------------------------
--------------------------------------------------
--------------------------------------------------
--------------------------------------------------
--------------------------------------------------
--------------------------------------------------
--------------------------------------------------
--------------------------------------------------
--------------------------------------------------
--------------------------------------------------
--------------------------------------------------
--------------------------------------------------
--------------------------------------------------
--------------------------------------------------
--------------------------------------------------
```

```
--------------------------------------------------
--------------------------------------------------
--------------------------------------------------
--------------------------------------------------
--------------------------------------------------
--------------------------------------------------
--------------------------------------------------
--------------------------------------------------
--------------------------------------------------
--------------------------------------------------
--------------------------------------------------
--------------------------------------------------
--------------------------------------------------
--------------------------------------------------
--------------------------------------------------
--------------------------------------------------
--------------------------------------------------
--------------------------------------------------
--------------------------------------------------
--------------------------------------------------
--------------------------------------------------
--------------------------------------------------
--------------------------------------------------
------------CGGATGGGATGGGCGCCTTCAAAATAAACCACAGCTCC
CACGATATTGGCTCCGG---ACAGACGGCGTTTTCCTCCCAGGCG---CC
CGGTTAC---GCAGCAGCCGCCCTGGGA---CACCACCA------CCACC
CGACCCACGTTGGCTCT---TACTCCACAGCGGCTTTCAACTCCACCAGG
GACTTTCTGTTCAGAAATCGGGGTTTCGGGGACGCCACCGG---------
---GGCTCAGCACAGTTTGTTCGCCTC---------CGGAAGTTT---C-
-----GCAGGGCCACATGGACACTCAGATGCAGCGGGGCACCTGCTCTTC
CCGGGGCTCCACGAG---CAAGCGGCGAGCCACGCGTCTTCGAACGTGGT
CAACAGCCAGATGCGGCTGGGCTTCTCGGGGGACATGTACGGACGAGCCG
ACCAGTACGGCCACGTTACGAGCCCACGCT---CCGACCACTACGCCTCG
ACCCAGCTCCACGGCTACGGCCCCATGAACATGAATATGGCCGCG---CA
CCACGGAGCAGGGGCCTTCTTCCGATACATGAGGCAGCCGATCAAACAAG
AGCTCATCTGCAAGTGGATCGAACCGGAGCAGTTGACAAATCCTAAAAAG
TCTTGTAACAAAACTTTTAGCACCATGCACGAGCTGGTGACCCATCTGAC
GGTGGAGCATGTGGGGGGACCGGAGCAGACCAACCACATCT---------
--------------------------------------------------
--------------------------------------------------
-------------
>Archoplites interuptus
AGCCTACTGATTCGAGCAGAGCTCAGCCAACCGGGCGCCCTCCTCGGGGA
CGACCAGATTTATAATGTAATTGTAACAGCACATGCATTTGTAATAATTT
TCTTTATAGTAATACCAATTATGATTGGGGGCTTTGGCAACTGGTTAGTC
CCCCTAATGATTGGCGCACCCGATATAGCCTTCCCCCGAATAAATAATAT
GAGTTTTTGACTCCTCCCCCCTTCTTTCCTTCTCCTCCTTGCCTCCTCTG
GGGTTGAAGCCGGGGCAGGAACTGGGTGGACCGTTTACCCCCCTCTGTCC
GGCAATCTAGCCCACGCAGGAGCATCCGTTGACCTAACTATCTTCTCCCT
CCACCTCGCCGGGGTCTCTTCCATCCTGGGGGCCATTAACTTTATTACCA
CAATTATTAACATAAAGCCCCCCACTATTTCTCAGTACCAGACACCGTTG
TTTGTATGGTCCGTTCTAATTACTGCCGTCCTCCTTCTCCTCTCCCTCCC
AGTTCTTGCTGCAGGCATCACAATACTCCTTACAGACCGGAATCTTAATA
```

CCACATTCTTTGACCC------------------------------------

------------TTCCTAGAGAGGAATCTTCACCCATCCAACTGCCTTGG
CATGCTGTTGCTGTCTGACGCCCACCAGTGCACCAAGCTGTCAGAGCTCT
CCTGGGGCATGTGCCTCAGCAACTTTCCCGCTATTTGCAAGACCGAGGAT
TTCCTCCAACTGCCCAAAGATATGGTGGTGCAGCTTTTGTCACATGAGGA
GCTAGAGACAGAAGATGAGAGGCTGGTTTATGAAGCTGCCCTCAACTGGA
TCAACTATGACCTGGAAAAGAGACACTGTAACCTTCCAGAGCTCCTGAGA
ACAGTCCGTCTGGCCCTGCTGCCTGCCATCTTTCTCATGGAGAATGTATC
TACAGAAGAGCTGATCAACGCACAGGCCAAGAGCAAGGAGCTGGTGGATG
AAGCTATCCGGTGTAAGCTGAAGATCCTGCAGAATGATGGCGTCGTTAAC
AGCCCATGTGCTCGACCAAGAAAAACCAGCCATGCCCTCTTTCTTCTGGG
AGGGCAGACTTTCATGTGTGACAAGTTGTACCTGGTGGACCAGAAAGCCA
AAGAGATCATCCCCAAAGCTGACATCCCTAGCCCAAGGAAAGAGTTCAGC
GCCTGCGCCATCGGCTGTAAGGTGTACATCACTGGCGGGA--GAGGCTC-
AGAGAATGGCGTTTCCAAAGATGTATGGGTCTATGACACCGTCCATGAGG
ATTGGTCAAAGGCGGCACCAATGCTCATCGCCAGGTTTGGCCATGGCTCT
GCAGAGCTGAAACACTGCCTCTACGTGGTAGGAGGTCACACCGCAGCGAC
TGGCTGCCTCCCGGCCTCTCCGTCTGGATGAATACATTGTTGTGTTTAGT
CGTTCAGCGACGAGGCTGATACTGAATGAAGCAGAGCTAATCATGGCGCT
GGCCCAGGAGTTCCAGATGAGAGTGGTCACGGTATCACTGGAGGAACAGT
CCTTCACCAGCATCGTCCAGGTGATCAGCGGTGCTTCCATGTTAGTCAGT
ATGCATGGAGCTCAGCTTATCACCTCCCTCTTCCTCCCCAGAGGAGCTGC
TGTGGTGGAGCTGTACCCTTTTGCTGTTAACCCAGAGCAATACACCCCAT
ATAAAACCCTTGCGTCCCTTCCAGGTATGGACCTTCACTATATCTCCTGG
AGGAACACTAAGGAGGAGAACACCATCACCCACCCAGACAGACCCTGGGA
ACAAGGGGGCATTGCTCACTTGGAGAAGGAGGAGCAGGAGCGAATACTGG
CTAGCAAAGATGTCCCCAGGCACTTGTGCTGCCGCAACCCAGAGTGGCTC
TTCCGGATCTACCAGGACACTTTGGTCGACATCCCTTCCTTCCTGGAAGT
CCTCAA---AGAGGGCATGAAG---ACAAAGCCCAGCTTAAAGAA---GT
CAAAGCCGGCCAGCACAGTCCATCCAGGCCGGGTCAGAGAACCCCAGTGT
CAGACCTCAGTACAAACCACTAATGAGGCTAAACTCACAGTCTCCTGGCA
GATCCCATGGAATCTGAAATACCTGAAGGTGAGAGAGGTGAAGTACGAGG
TGTGGATCCAGAAAAGAGACACCAGCAAGGGGACACTGGAGGATCAAATC
ATCCAGGCAAACCCCGCGCTGGAGGCCTTCGGCAACGCCAAAACACTGAG
AAACGACAACTCGTCTCGTTTTGGTAAATTCATAAGAATTCACTTCGGTA
CGAGCGGCAAGCTGTCGTCTGCCGACATCGAGACGTACCTGCTGGAGAAG
TCACGTGTCACCTTTCAGCTGAAGGCTGAGAGGAACTACCACATATTCTA
CCAGATCCTGTCCAATCAGAAGCCAGAGCTGCTGGACATGCTGCTGATCA
CCAACAACCCGTACGACTACTCCTACATCTCCCAAGGAGAGGTAACAGTC
GCCTCCATCAACGACTCAGAGGAGCTGATGGCCACCGACAGCGCCTTCGA
TGTACTCGGCTTCACTGCAGACGAGAAGATGGGCGTCTATAAACTGACAG
GCGCCATCATGCACTACGGTAACATGAAGTTCAAACAGAAGCAGCGTGAG
GAGCAGGCGGAGCCGGACGGACGGAGGCTGCTGATAAATCGGCTTACCT
AATGGGGCTGAACTCTGCTGACCTCATCAAAGGGCTGTGCCACCCCAGAG
TCAAGGTAGGAAACGAATACGTCACCAAAGGACAAAGTGTGGACCAAGTC
AACTACCCTAACAAGGAGGCCTTCAAGTGTGAGGAGTGTGGGAAGCACTA
CAACACCAAGCTGGGATATAAGCGCCATGTGGCCATGCACTCTGCCACAG
CAGGGGATCTCACCTGTAAAGTTTGCATGCAGACCTATGAGAGCACACCT
GTACTCCTGGAGCACCTCAAGAGCCACTCTGGGAAGTCTTCTGGTGGCGC

CAAGGAGAAAAAACACCCATGCGACCACTGTGATCGGCGTTTCTATACGC
GGAAGGATGTGAGACGGCACATGGTGGTCCACACAGGCCGAAAGGACTTC
CTGTGCCAGTACTGTGCCCAGCGCTTTGGCAGGAAGGACCATCTGACACG
TCACGTGAAGAAGAGCCACTCGCAGGAGCTGCTGAAGATCAAGACGGAAC
CTCCTGATATGTTAGGTCTTTTAGCCTCAGGGTCACCACCTTGCTCTGTG
AAGGAGGAGCTCAGTCCCATGATGTGCGGAATGGGTCCCAACAAAGACCC
AATGATGGGCAAACCATTCCCCAGTGGGGCCCCTTTTTCGATGGGCATGT
ACAACCCCCACCAT------CTGCAGGCCATGTCTAATTCTGGGGTGGGT
CACCCA------CACCCGTCCCTGATGCCCAGTTCCTTGTCTGCAGCTAT
GGGCATAGGCTGTCACATGGAATATCTCATCTACGCCTCTTTCTCATTCA
TGGGATGTTTACAAATCAGTGACGGATCAAATATTGTGAACCTGCTGGCA
AGTAACTCTCCAAGTGTTTCTTACGCTCTGACCCAGCAGAAATACTTCAG
TAACTACAGTCCCGTAATTGGTTTTTACATTTACGAGCCCATCGAGTACT
GGAACTCCACGGTGCAGGAGCACCTGAAGACTCTGAGTCATGGCTTCAAC
AAGATCTCCTGGATGGACAACTTTTTCCATTACCTGCGAGTGGTGAATGT
GAGTGCATCAACCAAAAGCGACTTCATCACCATCCTGAAGGGCTCCTTCC
TGCGCAGCCCAGAGTACCAGCACTTCACTGAGGACATCATATTTTCCAAA
A---ACCGTGAGACTG------ATGAGTACGACATTATCGCCTCACGCAT
GTACTTGGTGGCAAGGACAACCGAGAAGAAGCGTGAAGAGGTGGTGGAGC
TTCTGGAAAAGCTTCGCCCGTTGATGCTGATTAACAGTATCAAGTTCATT
GCCTTCAATCCTACGTTTGTGTTCATGGACCGCTACAGCTCCTCTGTCAT
CTCGCCCATCCTGACCTCAGGCTTCAGCGTACTCACAATCCTCATCCTCA
CTTTCTTCCTGGTCATCAACCCCTTGGGAAACTTCTGGCTCATCCTCACG
GTAACTTCTGTGGAGCTGGGCGTCTTGGGTTTGATGNNCTTTCACCAGTTTG
AATGGCAGCCAGCTCTTAAGAATGTGTCTACATCGTGCAATGTTGGCATT
ATTAATGGGCTTTCTGGATGGGCTTCCTCGGTGGATGACGCTCCAGCTGA
CACTATCACTCGGCGGTTTCGCTATGATGTGGCACTGGTGTCAGCATTAA
AGGATCTGGAGGAGGACATCATGGAGGGGCTGAGAGAGAGTGGGATGGAA
GACAGTGCTTGCACTTCAGGCTTCAATGTCATGATCAAGGAATCTTGTGA
TGGCATGGGTGATGTCAGCGAGAAGCACGGTGGAGGACCAGTTGTTCCTG
AGAAGGCTGTACGTTACTCTTTTACTGTTATGAGTGTCTCTGTCCTGGAA
GACGGTGAGGAG------------------------------GAAGAGGT
TACCATCTTCACCGAGCCAAAGCCAAACTCAGAACTGTCCTGTAAGCCCC
TTTGCCTGACCTTTGTGGATGAGTCCGACCATGAGACACTCACAGCTGTC
CTGAGCCCTATAATTGCAGAACGTAAGGCAATGAAAGAGAGCAGGCTCAT
CCTGTCCATGGGTGGACTAGCTCGCTCCTTCCGCTTCCACTTCAGAGCCA
CAGGATACGATGAGAAGATGGTGCGTGAGATGGAGGGCCTCGAGGCCTCA
GGGTCCACCTATATCTGCACTCTTTGTGACTCCAGTCGGGCAGAGGCCTC
TCAAAACATGGTGCTACACTCTGTCACCCGCAGTCATGAAGAGAACCTAG
AACGCTATGAAATATGGAGAACCAACCCCTTTTCTGAGTCTGCGGATGAG
CTGCGAGACAGAGTCAAAGGTGTCTCTGCCAAGCCCTTCATGGAGACCCA
GCCCACACTGGATGCATTACACTGTGACATCGGAAATGCCACTGAGTTTT
ACAAAATCTTCCAGGACGAGATTGGGGAGGTGTACCAAAAGGT---CAAC
---CCCAGCCGTGAGGAACGGCGCAGCTGGAGGGCAGCCCTAGATAAACA
GCTGAGGAAGAAGATGAAGCTTAAACCGGTGATGAGGATGAATGGGAACT
ATGCCCGCAGGCTAATGACCCAGGAGGCTGTGGAGGCGGTGTGTGAGCTG
GTGCCCTCAGAGGAGAGGAGGGAGGCCCTGAGGGAGCTTGTGAGGCTCTA
CCTCCAGATGAAGCCTGTGTGGCGTGCCACCTGCCCAGCCAAAGAGTGCC
CTGACCAGCTGTGCCGCTACAGCTTTAACTCCCAGAGCTTTGCCGACCTC
CTCTCCTCTACTTTCAAATACAGGTACAATGGAAAGATAACCAATTACCT
CCACAAGACCCTGGCCCATGTGCCTGAAATCATAGAGAGAGATGGATCCA
TAGGAGCCTGGGCTAGCGAAGGGAACGAGTCAGCAAACAANTCATACACCA
TCGATATGGGTCCCTTGGGTCCCCGGTGGAAGGAGAACCCACAGCCTTTT

TCCTGCTCCGTTGAAGACCCCACAAAACAGACAAAGTTCAAGGGCATCAA
GACTTACATTTCGTACCGGGTGACGCCGAGCCACACAGGGGATCCCGTCT
ACAGGCGGTACAAACACTTTGACTGGCTATACAACCGCTTACTGCACAAG
TTCACTGTGATCTCTGTGCCTCACCTGCCTGAGAAGCAGGCYACGGGGCG
ATTTGAGGAAGATTTCATCGAGAAGCGTAAGAGACGACTGATTCTGTGGA
TGAACCACATGACCAGTCACCCAGTCCTCTCCCAGTATGAAGGCTTTGAG
CACTTTCTCATGTGTGCTGATGACAAGCAGTGGAAACTGGGCAAGAGACG
GGCGGAGAAGGACGAGATGGTGGGTGCCCATTTCATGCTGACCCTCCAGA
TCCCTAACGAGCACCAGGACCTTCAGGATGTAGAGGAGCGGATCGACTCC
TTCAAGGCCTTTGCTAAGAAAATGGATGACAGCGTGATGCAGCTCACACA
TGTTGCCTCTGAGCTGGTGCGTAAGCACCTGGGTGGGTTCAGGAAGGAGT
TCCAGCGCCTGGGAAATGCCTTCCAGTCTATCAGCCAGGCGTTCATGCTG
GACCCTCCCCACAGTTCAGAAACCTTCAACAACGCCATCTCCCATCCTCT
CGCCACGTTCCTCAAACTGACCTCTCTGGGTTTCATCATTGGAGTAGGTG
TGGTTGGAAACCTCCTGATCTCCATCCTGCTGGTCAAAGACAAGAGCCTG
CACCGAGCGCCCTACTACTTCCTGTTGGACCTGTGTGCCTCTGATATCCT
GCGCTCCGCCATCTGCTTCCCCTTTGTCTTCACCTCTGTCAAGAATGGAT
CTGTCTGGACCTATGGCACGCTGACCTGCAAAGTGATCGCCTTCCTGGGT
GTGCTCTCCTGTTTCCACACAGCGTTTATGCTGTTCTGTGTCAGTGTCAC
GCGCTACCTGGCCATCGCACATCACCGTTTCTACACCAAGAGGCTGACCT
TCTGGACCTGTCTAGCTGTCATCTGCATGGTGTGGACATTGTCAGTGGCT
ATGGCGTTCCCGCCGGTGCTAGACGTAGGGACGTACTCTTTTATCCGGGA
GGAGGACCAGTGCACATTCCAGCACCGTTCCTTCAGGGCGAATGATTCGC
TGGGCTTCATGCTCCTGCTGGCGCTCATCCTCCTGGCCACACAGCTGGTT
TACCTCAAGCTCATCTTCTTCGTCCACGACCGTCGAAAGATGAAGCCTGT
CCAGTTCGTGCCTGCTGTCAGCCAGAACTGGACCTTCCACGGGCCAGGCG
CCAGCGGCCAGGCGGCGGCCAACTGGCTAGCCGGATTTGGTCGAGGCCCC
ACCCCGCCTACTTTGCTGGGCATCCGGCAGAACAGCAACGCAGCGGGCCG
CAGGCGTCTATTGGTATTGGATGAATTCAAAACAGAGAAGAGGATTAGTA
GGATGTTCTACATCATGACGTTTTTCTTCCTGGCACTGTGGGGGCCCTAT
CTGGTCGCCTGCTACTGGCGGGTGTTTGCAAGGGGCCCTGTGGTCCCTGG
AGGCTACCTGACGGCAGCTGTGTGGATGAGCTTTGCCCAGGCTGGGGTCA
ATCCTTTCATCTGCATCTTCTCCAACAGGGAGGCCAAATCTCGCTTTCAC
CCTGGCATGGGGACTGGTCCTGGCACGGAGC---GCAGCGTCCCACTCGG
CAACAGCTTGCTATCCCCGCAGCAAAGCGAGGAGCCCACTGTTGCCACCC
CCCCGCAGCGATGGTTTGTCACCC---CTGCCAACAACCGACTGGACTTT
GCTGCCTCGGCATACGACGCCGCT---------GATTTGCCGGTAACGC
GGCCACCTTGCTGTCCTACGCAGCCGCCGGAGTGAAGGCTC------TTC
CCCTGCCGACTGCAGGCTGCTCCAACCGGCCTCTTGGCTATTACGCAGAC
CCGTCAG---GCTGG---GGAGGACGCACGCCGCCGCAGTACTGTGGCGT
AAATAGCAAATCCAGCTCGGTCTTTTCCTGCTGGCCCGCTAACTCTATCG
GTGGCAGAGCGGGCA---CT---AACTACCTGG---------CTGAGGA-
--GGGA---GACTC---CATCCCGACAGAGAGGTCACCG---AT---CGG
CGGCTCGGAGGAG---ACCAAACCCAAAGACATAAC---GTCAGA---GT
CGAGCTGGATAGAG---ACGCCGTCCTCCATAAAATCCATTGATTCGAGC
GATTCTGGTATCTTTG---AACAGGCCAAAAGGAGAAGGATCTCACCTTC
TGCCACGCCG---------GTTTCAGAGACAGTGTCCCCGTTAAAATCTG
AGCATCACTCAACAGGCGAAGTCACAGAGAGAGAGAAGTGGCGTTGGGGATA
AATCCGTTCGCAGATGGGATGGGCGCCTTCAAAATAAACCACAGCTCCCA
CGATATTGGCTCCGG---ACAAACAGCGTTTTCCTCCCAGGCG---CCCG
GCTAC---GCAGCAGCCGCTCTGGGA---CACCATCA------CCACCCG
ACCCACGTTGGCTCT---TACTCCACGGCGGCTTTCAACTCCACCAGGGA
CTTTCTCTTCCGAAATCGGGGCTTCGGGGACGCCACTGG-----------

```
-GGCGCAGCACAGTTTGTTCGCCTC---------CGGAAGTTT---C---
---GCAGGGCCACATGGACACTCGGATGCAGCGGGGCACCTGCTCTTCCC
AGGGCTCCACGAG---CAAGCAGCGAGCCATGCGTCTTCCAACGTGGTCA
ACAGCCAGATGCGATTGGGCTTCTCGGGGGACATGTACGGACGCGCCGAC
CAGTATGGCCACGTTACAAGCCCGCGGT---CCGACCACTACGCTTCGAC
CCAGCTGCACGGCTATGGTCCCATGAACATGAATATGGCCGCA---CACC
ACGGAGCAGGGGCCTTCTTTCGATACATGAGGCAGCCCATCAAACAAGAG
CTCATCTGCAAGTGGGTCGAGCCGGAGCAGCTGACGAATCCCAAAAAGGC
GTGCAACAAAACTTTTAGCACGATGCACGAGCTTGTGACCCATCTGACGG
TGGAGCATGTGGGGGGACCGGAGCAGACCAACCACATCTGCTTCTGGGAG
GAATGCGCCAGAGAAGGAAAACCATTCAAAGCCAAATACAAACTTGTAAA
TCATATCAGAGTACACACCGGAGAAAAGCCCTTTCCGTGTCCGTTCCCCG
GCTGTGGCAAA
>Argentina silus
--------------------------------------------------
--------------------------------------------------
--------------------------------------------------
--------------------------------------------------
--------------------------------------------------
--------------------------------------------------
--------------------------------------------------
--------------------------------------------------
--------------------------------------------------
--------------------------------------------------
--------------------------------------------------
--------------------------------------------------
--------------------------------------------------
--------------------------------------------------
--------------------------------------------------
--------------------------------------------------
------------TTTCTGGAGAGAAACCTGCATCCGTCCAACTGCCTGGG
GATGCTGCTGCTGTCGGATGCCCACACGTGCACCAAGCTCTCCGAGCTCT
CCTGGGGCATGTGCCTCAGCAACTTCCCTGCCATCTGCAAGACGGAGGAC
TTCCTGCAGCTGCCCAAGGACATGGTGGTTCAGCTCCTGTCCCACGAGGA
GCTGGAGACTGAGGACGAGAGGCTGGTCTACGAGGCCGCTCTTAACTGGG
TCAACTACGACCTGGAGAGGAGGCACTGCCACCTGCCGGAGATGCTGAGG
ACCGTCCGCCTGGCCCTGCTGCCCGCCATCTTCCTCATGGAGAACGTGTC
CACGGAGGAGCTGATCAACGCCCAGGTCAAGAGCAAGGAGCTGGTGGACG
AGGCCGTCCGCTGCAAGCTGAAGATCCTGCAGAACGACGGCGTGGTCAAC
AGCCTGTGCGCCCGGCCCAGGAAGACCAGCCACGCCCTCTTCCTGTTGGG
CGGCCAGACTTTCATGTGCGACAAGCTGTACCTGGTGGACCAGAAGGCCA
AGGAGATTATCCCCAAGGCAGACATCCCAAGCCCCAGGAAGGAGTTCAGC
GCCTGTGCCATCGGATGTAAGGTCTACGTCACGGGGGGAA--GGGGCTC-
CGAGAACGGCGTGTCCAAAGACGTGTGGGTCTATGACACCATCCAGGAGG
AGTGGTCCAAGGCAGCGCCCATGCTCATCGCCCGGTTTGGCCATGGCTCA
GCAGAGCTGAAACACTGCCTCTACGTGGTGGGGGGTCACACAGCTGCCAC
AGGCTGTCTGCCCGCCTCGCCATCTGGAGGAGTACATAGTGGTGTTCAGC
CGTTCCATTAACAGGCTGATTCTTAATGAAGCAGAGCTGATTCTGGCCCT
AGCACAGGAGTTCCAGATGAAGGTTGTAACTGTGTCTCTGGAGGAGCAGT
CCTTTGCCAGTATAGTGAAGATGGTCAGTGAGGCCTCCATGTTAGTCAGT
ATGCACGGAGCCCAACTGGTGTCCTCTCTCTTCCTGCCCAGGGGGGCCGC
AGTGGTGGAACTCTTCCCCTATGCAGTGAACCCAGAGCAGTACGCTCCCT
ACAAAACCTTGGCCTCACTCCCTGGCATGGACCTGCAGTATGTGGCCTGG
```

AGGAACATGATAGAAGAGAACTCAGTGGCCTACGCTGAGAGGCCTTGGGA
CCAGGGAGGCATCGACCATTTGGAGAARGACGAGCAAGACCGCATCCTGG
CCAGCAAAGAGGTACCAAGGCACCTGTGCTGCCGCAACCCAGAGTGGCTC
TACCGTATCTATCAGGACACCATTGTGGACATCCCCTCTTTACTGGAGAC
CCTCAG---AGAGACCCTGAAA---ACCCGGCCAAACCYGAAGAA---GG
CCAAGCCTGCCAGCACGGTCCACCCGGGCCGAGTGAGAGAGCCCCAGTGC
CAGACCTCGGTCCAGGCCACCAACGAGGCCAAGCTCACCGTGTCCTGGCA
GATCCCCTGGAACCTGAAGTACCTGAAGGTCAGGGAGGTTAAATACGAAG
TGTGGATACAGAAGAGGGACCCAAGCAAGGGTACCCTAGAGGATCAAATC
ATCCAGGCTAACCCTGCACTGGAGGCTTTCGGCAATGCCAAAACATTAAG
GAATGATAATTCATCACGTTTTGGCAAATTCATCCGTATTCATTTCGGAC
AAAGTGGCAAATTGTCCTCTGCTGACATAGAGACTTACCTGCTTGAGAAG
TCACGTTGCACCTTTCAGCTGAAAGCGGAGAGGAACTACCACATCTTCTT
CCAGATCTTGTCCAATGAAAAGCCAGAGCTGTTGGACATGCTGTTGATCA
CCAACAATCCATATGACTACGCCTACATCTCCCAAGGAGAGGTGACAGTA
GCATCCATCAATGATTCTGAGGAGCTGATTGCCACTGACAGTGCCTTTGA
TGTGCTGGGCTTTACTGCAGAGGAGAAAATGGGGGTCTACAAGTTGATAG
GTGCCATCATGCACTATGGCAACATGAAGTTCAAGAACAAGCAGCGGGAG
GAGCAGGCAGAGCCTGATGGTACAGAGGCTGCTGACAAGTCAGCTTATCT
AATGGGACTGAACTCTGCAGATCTTATTAAAGGACTGTGCCATCCGAGAG
TCAAGGTTGGCAATGAGTATGTCACCAAAGGTCAGAGTGTAGATCAAGTC
TACTAC--

----------------------NNNNNNNNNNACGCCTCCTTCTCCTTCATGGGATGTTT
ACAAATCAGTGACGGATCCAACATTGTCAACCTGCTGGCCAGTAACTCCC
CCAGTGTGTCGTTCGCGATGACCCAGCAGAAGTACTTCAGTAACTACAGT
CCGGTTATTGGGTTCTATATCTACGAGCCCATCGAGTACTGGAACTCGAC
GGTACAGGAACACCTCAGAACTCTGGGTCACGGCTTCAACAAGATCTCCT
GGATGGATAACTACTTTCACTATCTGAAGGTGGCGAACGTGAGCGCATCG
ACCAAGAGCGACTTCATCTCCATCCTCAAGGGCTCGTTCCTGAAGAGCCC
GGAGTATCAGCACTTTACGGAGGACATCATCTTCTCCAAGGACGAGAACG
------------AGGAGTACAACATCATCGCGTCGCGGATGTACCNGGTGG
CGCGCACCACGGAGAAGACGNGGGAGGAGGTGGTGGAGCTGCTGGAGAGGC
TCCGCCCCCTGTCCCTGATCAACAGCATCAAGTTCATCAGCTTCAACCCG
ACCTTCGTCTTCATGGACCGCTACAGTTCCTCCGTCGTCTCGCCCCTCCT
CACCTCGGCCTTCAGCGTGCTCACCATCCTCATCCTCACCTTCTTCCTGG
TGGTCAACCCGCTGGGGAACTTCTGGCTGATCCTGACGGTGACGTCCGTG
GAGCTCGGGGTCCTGGNNNNNNNN----------------------------------

------AGGTGGATGATCAAGGAGTCCTGCGATGGCATGGGGGACATCAG
TAGGAAGCAAGGTGGAGGGCCGCCCATCCCTGGAAAGGCTGTTCGCTTCT
CTTTTACCATCATTCCGTTTCTTGTCCAGGCTGAAGGAGAGGAG------
------------------------GAGGTGGTCACCATCTTCCGGGAGCC
CAAGCCGAACTTTGAACTTTCCTGTAAGCCACTGTGCCTGATGCTTGTGG
ACGAGTCGGACCACGAGACACTCACGGCAGTCCTAGGCCCTGTGGTGGCC
GAACGGAACGCCATGAAGCACAGCCGGCTCATTCTGTCCCTGGGTGGGCT
CCCTCGCTCCTTCCGCTTCCACTTCAGAGGAACGGGTTTTGATGAGAAGA
TGGTGCGTGAGATGGAGGGCCTGGAGGCCTCTGGGTCCACCTACATTTGC
ACCTTGTGTGACTCCACCAGGGCAGAGGCCTCCCACAACATGGTGCTCCA
CTCCATCACCCGCAGCCATGACGAGAACCTGGAGCGCTATGAGTTGTGGA
GGACCAACCCCTTCTCCGAGTCCGCAGATGAGCTGCGGGACCGGGTCAAA
GGTGTCTCTGCCAAGCCCTTCATGGAGACCCAGCCCACACTGGATGCCCT
GCACTGCGACATCGGCAACGCCACTGAGTTTTACAAGATCTTCCAGGATG
AGATCGGGGAGGTGTACCGCAACCC---CAAC---CCCAGACGAGAGGAG
CGGCGGAGCTGGAGGGCTGCTCTGGACAAGCAGCTGAGGAAGAAGATGAA
GTTGAGGCCGGTGATGCGTATGAACGGGAACTACGCACGGCGGCTGATGA
CCCAGGAGGCAATAGAGGTAGTGTGTGAGCTGGTGCCATCAGAGGAGAGG
CGAGAGGTCCTAAGGGAGCTGATAGGGCTCTACCTCCAGATGAAGCCAGT
GTGGCGCTCCTCCTGCCCAGCCAAGGAGTGCCCAGACCAGCTGTGTCGCT
ACAGCTTCAACTCCCAGCGCTTCGCAGAGCTCCTCTCCACCTCCTTTAAG
TACAGGTATTGT--------------------------------------

NNNNNNNNNNNNNNNNNNNNNNNNAGGTCCTCAATGGAAAGAGAGTCCCCA
GCCTTTCTCTTGCTCCATCGAAGACCCCACCAAGCAGACAAAGTTTAAAG
GCATCAAGACCTACATATCGTACCGGGTAACCCCTAGCCACATGGGGCGG
CCTGTGTACCGGCGCTACAAGCACTTTGACTGGCTGTACAGTCGCCTACT
GCACAAGTTTACGGTCATATCTGTGCCCCACCTGCCTGAGAAACAGGCCA
CGGGGCGTTTCGAGGAGGATTTCATTGAGAAGCGTAAAAGGCGGCTGGTC
CTCTGGATAAACCACATGACCAGCCACCCAGTCCTGTCTCAGTACGAAGG
CTTCGAGCACTTCCTGATGTGTGCCGATGACAAGCAGTGGAAGCTGGGTA
AGCGGCGGGCGGAGAAGGACGAGATGGTGGGCGCTCATTTCATGCTCACC
TTCCAGATCCCCACGGAGCACCAAGACCTTCAGGATGTGGAGGAGCGYGT
GGACAACTTCAAGTCCTTCGCCAAGAAAATGGATGACAGTGTCATGCAGC
TGACGCATGTGGCTTCAGAACTGGTGCGGAAGCACCTCGGAGGATTCCGG
AAGGAGTTTCAGCGGCTGGGGAACGCATTCCAGTCCGTCAGTCAGGCATT
CATGCTGGACCCTCCTCACAGCTCAGACGCCCTCAACAATGCCATCTCTC
AC--

--GCCAAATCTCG
CTTTCACCCTGGCGTAGGGACTGGTCCTGGCACAGACC---GCAGCGTCC
CACTTAGTAACAGCTTGCTATCCCCGCAACAAACCGAAGAGCACACAGTT
G---CTTCCCCGCAGCGATGGTTTGTCACCC---CTGCCAATAACCGACT
GGACTTTGCCGCCTCGGCATACGATGCCGCCGCGGCTGCTGATTTTGCCG
GTAACGCGGCCACCTTGCTGTCCTACGCAGCGGCTGGAGTAAAAGCAC--
----TTCCCCTGCCCACTGCTGGGTGTTCAAATAGACCTTTGGGTTATTA
TGCCGACCCATCAG---GCTGG---GGCACCCGCACACCACCTCAGTACT
GT---------AGTAAATCCAGCTCAGTGCTCTCGTGCTGGCCCACAAAT
GCTGTTGGAAGCAGAACAGGCA---CGTCCAATTACCTGA---------C
GGAGGA---TGGG---GACAC---CATCCCTACGGAGAGGTCTCCA---A
T---CGGAGTGCCAGAGGAG---ACAAAACCGAAAGACTT------GTCC
GA---ATCCAGCTGGATAGAG---ACGCCGTCTTCAATAAAGTCGATAGA
TTCAAGTGATTCTGGAATCTTTG---AGCAGGCCAAACGGAGAAGAATTT
CACCGTCTGCCACACCG---------GTTTCGGAGACAGTGTCCCCGCTG
AAATCAGAG---------ACAGGCGAAGTCACAGACAGAGAAGTGGCTTT
GGGGATAAATCCGTTCGCAGACGGGATGGGCGCTTTTAAAATCAACCACA
GCTCTCATGATCTTGGCTCCGG---GCAAACGGCGTTTTCCTCCCAAGCG
---CCCGGCTAC---GCAGCCGCTGCCTTGGGA---CATCATCA------
CCATCCGACCCATGTCAGCTCC---TACTCCACCGCCGCTTTCAATTCCA
CCCGGGACTTTCTTTTTCGAAATCGGGGCTTCGGAGACGCGACCAG----
--------CGCCCAACACAGTCTCTTCGCCTCCGC---AGCGGGAAGTTT
---T------GCAGGGCCACATGGACACTCAGATGCCGCGGGACACCTGC
TCTTCCCAGGACTTCACGAG---CAAGCCGCGAGCCATGCGTCCTCCAAT
GTTGTAAACAGTCAGATGCGATTGGGTTTTTCGGGGGACATGTACGGGCG
GGCTGACCAATATGGCCATGTTACGAGCCCACGTT---CCGACCACTACG
CTTCGACCCAGTTGCATGGCTATGGCCCCATGAACATGAATATGGCCGCA
---CACCACGGAGCAGGGGCCTTCTTCCGTTACATGAGGCAGCCGATCAA
ACAAGAGCTCATCTGCAAGTGGGTCGAGCCGGAGCAGTTGTCGAACCCGA
AAAAGTCGTGCAACAAAACTTTCAGCACGATGCACGAGCTCGTGACCCAC
CTGACAGTGGAACATGTGGGGGGACCAGAGCAGTCGAACCATATTTGCTT
TTGGGAAGAGTGTTGCCGAGAAGGAAAACCATTCAAAGCTAAATACAAAC
TTGTAAATCATATCAGAGTACACACCGGAGAAAAACCGTTCCCATGTCCA
TTCCCCGGCTGTGGCAAA
>Argyropelecus affinis
AGCTTACTCATCCGGGCAGAACTGAGTCAACCTGGCGCCCTCATGGGCGA
TGACCAAATCTACAATGTCCTAGTTACAGCACATGCTTTTGTAATAATCT
TCTTCATAGTAATACCCATCATAATTGGAGGTTTCGGAAACTGGTTAGTC
CCACTTATAATTGGTGCCCCAGATATGGCGTTTCCCCGAATGAATAACAT
AAGCTTTTGACTCCTTCCTCCCTCCTTTCTTCTTCTACTTGCCTCTTCGG
GTGTTGAAGCTGGGGCCGGAACCGGGTGGACTGTTTATCCCCCTCTTTCC
AGCAACTTAGCTCATGCAGGCGCCTCCGTTGATTTGACCATCTTCTCCCT
TCACCTCGCCGGTATCTCCTCTATCCTTGGGGCCATTAACTTTATTACCA
CAATTATTAATATAAAACCCCCAGCCATCTCTCAATACCAAACGCCCCTT
TTCGTTTGATCTGTTCTTGTCACCGCCGTACTTCTTCTTCTCTCCTTGCC
CGTCCTAGCCGCTGGTATTACAATACTTTTAACAGATCGGAACCTAAACA
CAACCTTTTTTGACCCGGCAGGAGGGGGAGATCCCATTCTCTACCAACAC
CTTTTCTGATTCTTTGGACACCCAGAAGTATATATCCTTATTCTCCCAGG
CTTTGGTATAATTTCCCACATTGTTGCATATTACTCAGGGAAAAAAGAAC

CCTTTGGATATATAGGAATAGTCTGAGCTATAATAGCCATTGGTCTCCTA
GGTTTTATTGTCTGAGCCCATCACATATTTACAGTTGGAATGGATGTGGA
CACCCGAGCTTNTTCCTGGAGAGGAACCTGCACTCGTCCAACTGTCTGGGA
ATGCTGCTTCTCTCAGACGCCCATCAGTGTACCAAGCTGTCTGAGCTGTC
CTGGGGCATGTGCCTCAGCAACTTTCCCGCCATTTGCAAGACCGAGGACT
TCCTGCAGTTGCCCAAAGACATGGCGGTCCAGCTGCTGTCTCACGAGGAG
CTGGAGACGGAGGACGAGAGGCTGGTCTACGAGGCCGCCCTTAACTGGGT
CAACTACGACCTGGAGAGGCGGCACTGCCACCTGCCGGAGCTGCTGAGAA
CTGTTCGTCTGGCCTTGCTGCCTGCCATCTTCCTCATGGAGAATGTCTCC
ACAGAGGAGCTGATCAATGCCCAGACCAAGAGCAAGGAGTTGGTGGACGA
GGCCATCCGCTGCAAGCTGAGGATCCTGCAGAACGAGGGCGTGGTCAACA
GCCCGCTGGCCCGGCCCAGGAAGACCAGCCACGCTCTCTTCCTGCTGGGT
GGGCAGACCTTCATGTGTGATAAACTCTACCTGGTGGACCAGAAGGCCAA
GGAGATCATCCCCAAGGCAGACATCCCTAGCCCCAGGAAGGAGTTCAGCG
CCTGCGCAATCGGCTGCAAGGTCTACATCACCGGAGGCA--GGGGCTC-T
GAGAACGGGGTCTCCAAAGATGTTTGGGTCTACGATACGTCCCATGAGGA
GTGGTCGAAGGCGGCTCCCATGCTCATCGCCCGATTCGGCCATGGTTCYG
CAGAACTCAAACACTGCCTCTACGTGGTGGGCGGACACACGGCCGGCACA
GGCTGCCTCCCCGCGTCCCCCTCG--------------------------

----------AAGAGGGATCCCAGCAAGGGAACCTTGGAGGATCAAATCA
TCCAAGCCAACCCTGCCCTGGAGGCTTTCGGTAACGCCAAAACATTGAGA
AATGACAACTCATCACGCTTTGGCAAATTCATTCGCATTCATTTCGGAAC
CAGTGGCAAGTTATCCTCTGCAGACATAGAGACTTACCTTCTGGAAAAGT
CACGTGTCACCTTTCAGCTCAAGTCAGAGAGGAACTATCACATCTTCTTC
CAGATCTTGTCCAATCAAAAGCCAGAGCTGTTGGACATGCTTTTAATCAC
CAACAATCCCTACGACTACTCCTTCATCTCCCAAGGAGAGGTAACAGTAG
CGTCCATCAATGATTCAGAGGAGTTGATAGCCACTGACAGTGCATTTGAC
GTGCTCGGCTTTACTCAAGAGGAGAAAATGGGGGTCTACAAGTTGACAGG
TGCGATCATGCATTATGGCAACATGAAGTTCAAGCAAAAGCAGCGCGAGG
AGCAGGCAGAACCTGATGGCACTGAGGCTGCTGACAAGGTAGCTTACCTA
ATGGGGCTGAACTCTGCAGATCTAGTGAAAGGACTCTGTCATCCCAGGGT
GAAGGTCGGTAATGAGTTTGTCACTAAAGGGCAGAGTGTAGACCAAGTCA
ACTACCCCAACAAGGAGGCCTTCAAGTGTGAGGAGTGTGGCAAGCACTAC
AACACCAAGCTGGGCTACAAGCGCCATGTGGCCATGCACTCGGCCACGGC
GGGCGACCTCACCTGCAAGGTGTGCCTGCAGAGCTACGAGAGCACACCCG
CCCTGCTGGAGCATCTGAAGAGCCACTCCGGGAAGTCGTCGGGCGGGGCC
AAGGAGAAGAAGCACCCGTGCGACCACTGCGACCGCCGTTTCTACACACG
CAAGGACGTCAGGCGCCACATGGTGGTGCACACCGGCCGCAAAGACTTCC
TGTGCCAGTACTGCGCCCAGCGCTTTGGCAGGAAGGACCATCTGACACGG

CACGTGAAGAAGAGCCACTCGCAGGAGCTGCTGAAGATCAAAGCGGAGCC
TCCGGATATGCTGGGGCTGCTGGGGTCTGGCTCGCCGCCCTGCTCTGTCA
AAGAGGAGCTCAGCCCCATGATGTGCAGCATGGGTCCCTCCAAGGACCCC
CTGATGGCCAAGCCTTTCCCCAGCGGGACCCCCTTCCCCATGGGCATGTA
CAACCCCCACCAC------CTGCAGGCCATGTCCGGCCCTGGGGGGGGCC
AC---------CACCCCTCCCTGATGCCCGGCTCCCTGTCTGCGGCTATG
GGCATGGGCTGCCACATGGAC-TACTGATCTACGCCTCCTTCTCCTTCAT
GGGCTGCTTGCAGATCAGCGACGGGTCCAACGTGGTCAACCTGTTAGCCA
GCAACTCCCCGAGTGTGTCCTACGCGCTGACCCAACAGAAGTACTTCAGC
AACTACAGCCCTGTGATCGGGTTCTACATCTACGAGCCCATAGAGTACTG
GAACTCCACGGTGCAGGAGCACCTGAGGACACTGGGCCACGGCTTCAACA
AGATCTCCTGGATGGACAACTACTTTCACTACCTGAAGGCGATGAACGCG
ACCGCCTCCACGAAGGCCGACTTCATCAGCATCCTCAAGGGTTCGTTCCT
GAGGAGCCCGGAGTACCAGCACTTCACGGAGGACATCATCTTCTCGAAGA
---ACGGCG------------AGGAGTACGACATCATCGCGTCCCGGATG
TACCTGGTGGCGCGCACCACAGAGAAGACCCGGGAGGAGGTGGTGGAGCT
GCTGGAGAGGCTCCGCCCGCTTTCCCTCATCAACAGCATCAAGTTCATCG
TCTTCAACCCCACCTTCGTGTTCATGGACCGCTACAGCTCCTCGGTCATT
TCACCCATCCTCACGTCAGGCTTCAGTGTCCTCACCATCCTCATCCTCAC
CTTCTTCCTCGTCATCAACCCCCTCGGGAACTTCTGGTTGATCCTGACGG
TGACCTCGGTGGAACTGGGCGTCCTGGGCCTGATGGGCTACCACCCCTTT
GAGTGGCAGCCGGCCCTCAAGAATGTGTCCACGTCCTGCCAGGTGGGCAT
CATCAACGGGCTGTCAGGGTGGGCTGCCTCTGTAGATGACTCCCTTGTGG
AAACCGTCACCCGGCGCTTCCGCTACGACGTGGCGCTGGTGTCAGCTCTG
AAGGACCTAGAGGAGGACATCATGGAGGGGCTGCGGGAGCGCGGCCTGGA
GGACAGCGCCTGCACCTCAGGCTTCAGCGTGATGATCAAAGAGTCCTGCG
ACGGCATGGGGGACGTCAGTGAGAAGCACGGCGGGGGGCCGGCGGTGCCT
GAAAAGGCTGTGCGGTTCTCTTTCACCATCATGTCTGTGTCTGTCCAGGC
TGAGGATGAGGAT------------------------------GAGGCTG
TTACTGTCTTCAGGGAGCCCAAGCCCAACTCTGAGCTGTCCTGCAAGCCT
CTCTGTCTGATGTTTGTGGACGAGTCTGATCATGAGACGCTGACAAGCAT
CCTTTGGCCTGTGGTGGCTGAGAGGAACGCCATGAAGAACAGCCGCCTCA
TTCTGTCTCTGGGAGGTCTCCCTCGATCCTTCCGCTTCCACTTCAGGGGT
ACAGGCTATGACGAAAAGATGGTGCGTGAGATGGAGGGCCTGGAGGCCTC
AGGTTCCACATACATCTGCACTCTCTGTGACTCCACCCGGGCAGAGGCCT
CCCACAACATGGTGCTTCACTCCATCACTCGCAGCCACAACGAGAACCTG
GAGCGCTATGAGATCTGGAGGACCAACCCCTACTCTGAGTCCGCAGATGA
GCTTCGGGATCGGGTGAAAGGAGTCTCTGCCAAACCCTTCATGGAGACCC
AGCCCACCCTGGATGCTCTGCACTGTGACATTGGTAACGCCACTGAGTTC
TACAAGATATTTCAGGATGAGATCGGGGAGGTGTACCGGAGGCC---GAA
C---CCAAGCAGGGAGGAGCGCCGGAGCTGGCGGGCTGCTCTGGACAAGC
AGYTGAGGAGGAAGATGAAGCTGAGGCCAGTGATGAGGATGAATGGGAAC
TATGCCCGGCGGCTGATGACCAGCGAGGGGGTGGAGGTGGTGTGTGAGCT
GGTACCCTCAGAACAGCGGCGGGAGGCCCTCAGGGAACTGATGGGGCT--

--TCCTACAC
AATCGAAATGGGCCCCAAAGGGCCTCAGTGGAAGGAGAGCCCTCAGCCGT
TCTCTTGTTCTGTTGAGGACCCTACCAAACAGACCAAGTTCAAAGGCATC
AAGACCTACATATCTTACCGGGTCACCCCAAGCCACACCGGGCGACCTGT
ATACCGTCGGTACAAGCATTTTGATTGGCTATACAACCGCMTGCTGCATA

AGTTCACTGTCATCTCTGTGCCTCACCTGCCGGAGAAGCAAGCCACAGGG
CGTTTCGAGGAGGACTTCATCGAAAAGCGCAAAAGGAGGCTGATCCTCTG
GATGGACCACATGACCAGTCACCCAGTCCTCTCCCAATATGAGGGCTTTG
AGCACTTCCTCATGTGCGCTGATGACAAACAGTGGAAGCTGGGCAAGCGG
CGAGCGGAGAAGGATGAAATGGTGGGCGCCCACTTCATGCTGACCTTTCA
GATTCCCAAAGAGCACCAGGACCTGCAGGATGTGGAGGAGAGAGTGGACT
CCTTCAAGTCTTTTGCTAAGAAAATGGATGATAGTGTCATGCAGCTGACT
CACGTGACCTCAGAACTGGTGCGGAAACATCTAGGGGGTTTCAGAAAGGA
ATTCCAGCGTCTGGGGAACGCTTTCCAGTCCATTAGTCAGGCATTCACGC
TGGACCCTCCTCACTGCTCTGAAGCCCTCAACAACGCCTTCTCACAC---

----------------------------------GCCAAGTCTCGCTTTC
ACCCTGGCGTAGGGACTGCCCCTGGCACGGACC---GCAGCATCCCACTT
AGTAACAGCTTGCTATCGCCCCAACAAACCGAAGAGCCCACGGTTG---C
TTCCCCGCAGCGATGGTTTGTCAGCC---CTGCCAACAACAGACTGGACT
TTGCCGCCTCGGCATACGATGCTGCTGCTGCTGCAGATTTTGCAGGAAAC
GCAGCCACCTTGTTGTCCTACGCAGCAGCTGGAGTGAAGGCAC------T
CCCCCTGTCCACGGCTGGTTGCTCAAACAGACCTCTTGGGTACTATACTG
ACCCATCAG---GCTGG---GGCGCCCGCACACCACCACAGTACTGC---
------AGCAAGTCCAGCTCTGTTCTCTCTTGCTGGCCCACAAATACTGT
TGGGGGCAGAACTGGCA---CCTCCAATTACCTGG---------CTGAGG
A---TGGG---GATGC---CCTCCCCACAGAGAGGTCTCCA---AT---A
GGCACGTCAGACGAA---GCGAAACCAAAAGACCT------GTCGGA---
ACCCAACTGGATAGAG---ACGCCGTCCTCAATCAAGTCGATTGATTCAA
GTGATTCTGGTATCTTTG---AGCAGGCAAAACGGAGAAGAATCTCTCCC
TCTGCCACACCA---------GTTTCAGA---TGTTTCCCCGTTGAAATC
CGAG--

>Argyropelecus gigas
AGCCTACTTATCCGGGCAGAACTAAGCCAACCCGGCACCCTCATAGGTGA
TGACCAAATCTATAATGTTCTCGTTACAGCACATGCTTTCGTAATGATTT
TCTTCATGGTAATACCTATTATAATTGGGGGCTTCGGAAACTGACTAGTC
CCATTAATAATTGGCGCCCCTGACATAGCATTTCCACGAATAAACAATAT
AAGCTTCTGACTCCTCCCCCCATCTTTCCTTCTTCTCCTTGCCTCCTCAG
GCGTTGAAGCTGGGGCCGGGACAGGGTGAACTGTTTACCCCCCTCTTTCC
AGCAACTTAGCACATGCAGGCGCCTCAGTAGACTTAACTATCTTCTCCCT
ACATCTCGCAGGCATCTCTTCCATTCTAGGGGCAATCAACTTTATTACTA
CAATTATCAACATAAAACCTCCAGCCATCTCTCAATACCAAACACCTCTT
TTCGTGTGATCAGTTCTTGTAACTGCTGTACTTCTTCTCCTTTCCTTACC
AGTCCTAGCTGCCGGTATTACAATACTTTTAACTGACCGAAACCTAAACA
CAACATTCTTTGACCCCGCAGGAGGAGGAGACCCCATCTTATACCAACAC
CT--

------------TTCCTGGAGAGGAACCTGCACTCGTCCAACTGTCTGGG
AATGCTGCTTCTCTCAGACGCCCATCAGTGTACCAAGCTGTCTGAGCTGT
CCTGGGGCATGTGTCTCAGCAACTTTCCCGCCATTTGCAAGACCGAGGAC
TTCCTGCAGTTGCCCAAAGACATGGCGGTCCAGCTGCTGTCTCACGAGGA
GCTGGAGACGGAGGACGAGAGGCTGGTCTACGAGGCTGCCCTTAACTGGG
TCAACTACGACCTGGAGAGGCGGCACTGCCACCTGCCGGAGCTGCTGAGA
ACTGTTCGTCTGGCCTTGCTGCCCGCCATCTTCCTCATGGAGAATGTCTC
CACAGAGGAGCTGATCAATGCCCAGACCAAGAGCAAGGAGTTGGTGGACG
AGGCCATCCGCTGCAAGCTGAGGATCCTGCAGAACGAGGGCGTGGTCAAC
AGCCCGCTGGCCCGGCCCAGGAAGACCAGCCACGCTCTCTTCCTGCTGGG
TGGGCAGACCTTCATGTGTGATAAACTCTACCTGGTGGACCAGAAGGCCA
AGGAGATCATCCCCAAGGCAGACATCCCTAGCCCCAGGAAGGAGTTCAGC
GCCTGCGCCATCGGCTGCAAGGTCTACATCACCGGAGGCA--GGGGCTC-
TGAGAACGGCGTGTCCAAAGATGTTTGGGTGTACGATACCTCCCATGAGG
AGTGGTCGAAGGCGGCTCCCATGCTCATCGCTCGATTCGGCCATGGTTCC
GCTGAACTCAAACACTGCCTCTAYGTGGTGGGAGGACAGACTGCCGCCAC
NNNNNNNNNNNNNNNNNNNNNNNNN---

------------------------------------AAGAGGGATCCCAG
CAAGGGAACCTTGGAGGATCAAATCATCCAAGCCAACCCTGCCCTGGAGG
CTTTCGGTAACGCCAAAACATTGAGAAATGACAACTCATCACGCTTTGGC
AAATTCATTCGCATTCACTTCGGAACCAATGGCAAGTTATCCTCTGCTGA
CATAGAGACTTACCTTCTGGAAAAGTCACGTGTCACCTTTCAGCTCAAGT
CAGAGAGGAACTACCATATCTTCTTCCAGATCTTGTCCAATCAAAAGCCA
GAGCTGTTGGACATGCTTTTAATCACCAACAATCCCTACGACTACTCCTT
CATCTCCCAAGGAGAGGTAACAGTAGCGTCCATCAATGATTCAGAGGAGT
TGATAGCCACTGACAGTGCATTTGACGTGCTCGGCTTTACTCAAGAGGAG
AAAATGGGGGTCTACAAGTTGACAGGTGCGATCATGCATTATGGCAACAT
GAAGTTCAAGCAAAAGCAGCGCGAGGAGCAGGCAGAACCTGATGGCACTG
AGGCTGCTGACAAGGCAGCTTACCTAATGGGGCTGAACTCTGCAGATCTA
GTGAAAGGACTATGTCATCCCAGGGTGAAGGTCGGTAATGAGTTTGTCAC
TAAGGGGCAGGGTGTAGACCAAGTCAACTACCCCAACAAGGAGGCCTTCA
AGTGCGAGGAGTGTGGCAAGCACTACAACACCAAGCTGGGCTACAAGCGC
CATGTGGCCATGCACTCGGCCACGGCGGGCGACCTCACTTGCAAGGTGTG
CCTGCAGAGCTACGAGAGCACACCCGCCCTGCTGGAGCACCTGAAGAGCC
ACTCCGGGAAGTCGTCGGGCGGGGCCAAGGAGAAGAAGCACCCGTGCGAC
CACTGCGACCGCCGTTTCTACACACGCAAGGACGTCAGGCGCCACATGGT
GGTGCACACCGGCCGCAAGGACTTCCTGTGCCAGTACTGCGCCCAGCGCT
TTGGCAGGAAGGACCATCTGACACGGCACGTGAAGAAGAGCCACTCGCAG
GAGCTGCTGAAGATCAAAGCGGAGCCTCCGGATATGCTGGGGCTGCTGGG
GTCCGGCTCGCCACCCTGCTCTGTCAAAGAGGAGCTCAGCCCCATGATGT
GCAGCATGGGTCCCTCCAAGGACCCCCTGATGGCCAAGCCTTTCCCCAGC
GGGACCCCCTTCCCCATGGGCATGTACAACCCCCACCAC------CTGCA
GGCCATGTCCGGCCCTGGGGGGGGCCAC---------CACCCCTCCCTGA
TGCCCGGCTCCCTGTCTGCGGCTATGGGCATGGGCTGCCACATGGACNNNNNNNNNNNNNNNNNNTTC
TCCTTCATGGGCTGCTTGCAGATCAGCGACGGGTCCAACGTGGTCAACCT
GTTAGCCAGCAACTCCCCGAGCGTGTCCTACGCGCTGACCCAACAGAAGT
ACTTCAGCAACTACAGCCCTGTGATCGGGTTCTACATCTACGAGCCCATT
GAGTACTGGAACTCCACGGTGCAGGAGCACCTAAGGACACTGGGCCACGG
CTTCAACAAGATCTCCTGGATGGACAACTACTTTCACTACCTGAAGGCGA
TGAACGCGACCGCCTCCACGAAGGCCGACTTCATCAGCATCCTCAAGGGT
TCTTTCCTGAGGAGCCCGGAGTACCAGCACTTCACGGAGGACATAATCTT
CTCGAAGA---ACGGCG------------AGGAGTACGACATCATCGCGT
CCCGGATGTACCTGGTGGCGCGCACCACAGAGAAGACCCGGGAGGAGGTG
GTGGAGCTGCTGGAGAGGCTCCGCCCGCTTTCCCTCATCAACAGCATCAA
GTTCATCGTCTTCAACCCCACCTTCGTGTTCATGGACCGCTACAGCTCCT
CGGTCATTTCACCCATCCTCACGTCAGGCTTCAGTGTCCTCACCATCCTC
ATCCTCACCTTCTTCCTCGTCATCAACCCCCTCGGGAACTTCTGGTTGAT
CCTGACCGTGACNNNNNNNNNNNNNNNNNNNNNNNNNNNNNNN---------------------------
----

---CAGCGAG
AAGCACGGCGGGGGGCCAGCGGTGCCTGAAAAGGCTGTGCGGTTCTCCTT

CACCATCATGTCTGTGTCTGTCCAGGCTGAGGATGAGGAT----------
--------------------GAGGCTGTTACTGTCTTCAGGGAGCCCAAG
CCCAACTCTGAGCTGTCGTGCAAGCCTCTCTGTCTGATGTTTGTGGACGA
GTCTGATCATGAGACGCTGACAGGCATCCTTGGGCCTGTGGTGGCTGAGA
GGAACGCCATGAAGAACAGCCGCCTCATTCTGTCTCTGGGAGGTCTCCCT
CGATCCTTCCGCTTCCACTTCAGGGGTACAGGCTATGACGAAAAGATGGT
GCGTGAGATGGAGGGCCTGGAGGCCTCAGGTTCCACATACATCTGCACTC
TCTGTGACTCCACCCGGGCAGAGGCCTCCCACAACATGGTGCTTCACTCC
ATCACTCGCAGCCACGACGAGAACCTGGAGCGCTATGAGATCTGGAGGAC
CAACCCCTACTCTGAGTCCGCAGATGAGCTTCGGGATCGGGTGAAAGGAG
TCTCTGCCAAACCCTTCATGGAGACCCAGCCCACCCTGGATGCTCTGCAC
TGTGACATCGGTAACGCCACTGAGTTCTACAAGATATTTCAGGATGAGAT
CGGGGAGGTGTACCGGAGGCC---GAAC---CCAAGCAGGGAGGAGCGCC
GGAGCTGGAGGGCTGCTCTGGACAAGCAGCTGAGGAGGAAGATGAAGCTG
AGGCCAGTGATGAGGATG--------------------------------

-------------------TCCTACACAATCGAAATGGGCCCCAAAGGGC
CTCAGTGGAAGGAGAGCCCTCAGCCGTTCTCTTGTTCTGTTGAGGACCCT
ACCAAACAGACCAAGTTCAAAGGCATCAAGACCTACATATCTTACCGGGT
CACCCCAAGCCACACTGGGCGACCTGTATACCGTCGGTACAAGCATTTTG
ATTGGCTATACAACCGCCTGCTGCATAAGTTCACTGTCATCTCTGTGCCT
CACCTGCCGGAGAAGCAGGCCACAGGGCGTTTCGAGGAGGACTTCATCGA
AAAGCGCAAAAGGAGGCTGATCCTCTGGATGGACCACATGACCAGTCACC
CAGTCCTCTCCCAATATGAGGGCTTTGAGCACTTCCTCATGTGCGCTGAT
GACAAACAGTGGAAGCTGGGCAAGCGGCGAGCGGAGAAGGATGAAATGGT
GGGCGCCCACTTCATGCTGACCTTTCAGATTCCCAAAGAGCACCAGGACC
TGCAGGATGTGGAGGAGAGAGTGGACTCCTTCAAGTCTTTTGCTAAGAAA
ATGGATGATAGTGTCATGCAGCTGACTCACGTGACCTCAGAACTGGTGCG
GAAACATCTAGGGGGGTTTCAGAAAGGAATTCCAGCGTCTGGGGAACGCTT
TCCAGTCCATTAGTCAGGCATTCACGCTGGACCCTCCTCACTGCTCTGAA
GCCCTCAACAACGCCATCTCACAC--------------------------

-----------GCCAAGTCTCGCTTTCACCCTGGCGTAGGGACTGCCCCT
GGCACGGACC---GCAGCATCCCACTTAGTAACAGCTTGCTATCGCCCCA
ACAAACCGAAGAGCCCACGGTTG---CTTCCCCGCAGCGATGGTTTGTCA
GCC---CTGCCAACAACAGACTGGACTTTGCCGCCTCGGCATACGATGCT
GCTGCTGCTGCAGATTTTGCAGGAAACGCAGCCACCTTGTTGTCCTACGC
AGCAGCTGGAGTGAAGGCAC------TCCCCCTGTCCACAGCTGGTTGCT
CAAACAGACCTCTTGGGTACTATGCTGACCCATCAG---GCTGG---GGC
GCCCGCACTCCACCACAGTACTGC---------AGCAAGTCCAGCTCTGT
TCTCTCTTGCTGGCCCACAAATACTGTTGGGGGCAGAACTGGCA---CCT
CCAATTACCTGG---------CTGAGGA---TGGG---GATGC---CCTC
CCCACAGAGAGGTCTCCA---AT---AGGCACGTCAGACGAA---GCGAA
ACCAAAAGACCT------GTCGGA---ACCCAACTGGATAGAG---ACGC
CGTCCTCAATCAAGTCAATTGATTCAAGTGATTCTGGTATCTTTG---AG
CAGGCAAAACGGAGAAGAATCTCTCCCTCTGCCACACCA---------GT
TTCAGA---TGCTTCCCCATTGAAATCCGAG------------------G
TCACAGAGAGAGAGGTGGCGCTGGGGATAAATCCGTTCGCAGACGGGATG
GGCGCTTTCAAAATCAACCACAGCTCGCACGACCTGGGCTCCGG---GCA
GACTGCCTTCTCCTCCCAGGCA---CCCGGCTAC---GCAGCAGCTGCTC
TGGGA---CACCACCA------CCACCCCACACACGTCAGCTCT---TAC
TCCACCGCGGCGTTCAACTCCACCAGGGACTTCCTGTTCAGAAACCGGGG
CTTCGGAGACGCCACCAG------------TGCCCAGCACAGTCTCTTCG
CCTCCGC----A--GGAAGTTT---C------GCAGGGCCACATGGACAC
TCAGATGC---------CTTGCTCTTCCCAGGACTCCACGAG---CAAGC
AGCCAGCCATGCGTCCTCAAATGTTGTTAACAGTCAGATGCGCTTGGGCT
TTTCTGGGGACATGTACGGGCGGGCTGACCAGTATGGCCACGTTACCAGC
CCCCGGT---CCGAGCACTATGCTTCGACCCAGCTGCATGGCTATGGCCC
TATGAACATGAATATGGCCGCA---CACCATGGAGCAGGGGCCTTCTTCC
GTTACATGAGGCAGCCGATCAAACAAGAGCTGATCTGCAAGTGGATTGAA
CCAGAGCAACTGACGAATCCGAAAAAGTCGTGCAACAAAACTTTTAGCAC
GATGCACGAGCTAGTGACCCATCTGACGGTGGAACATGTCGGTGGACCGG
AGCAGTCCAACCATATTTGCTTCTGGGAAGAGTGCGCCCGAGAAGGGAAA
CCRTTCAAAGCCAAATACAAACTTGTCAACCACATCAGAG----------

>Arrhamphus sclerolepis

------------TTCCTAGAGAGAAACCTTCACCCGTCCAACTGCCTTGG

CATGCTGTTGCTGTCTGACGCCCACCAGTGTACCAAGCTGTCAGAGCTCT
CCTGGGGCATGTGTCTAAGCAACTTCCCTGCTATTTGCAAGACAGAAGAT
TTCCTCCAACTGCCAAAAGATATGGTGGTGCAGCTTTTGTCACACGAGGA
GCTGGAGACAGAAGATGAGAGACTGGTTTATGAAGCTGCACTGAATTGGA
TCAACTATGACCTGGAAAGGAGGCACTGCCATCTTCCAGAGCTCCTGAGA
ACGGTCCGCCTCGCCCTGCTTCCTGCCATCTTTTTAATGGAGAACGTTTC
GACAGAAGAACTGATCAATGCCCAGGCAAAGAGCAAGGAACTGGTGGACG
AAGCTATTCGCTGTAAGCTGAAGATCTTGCAGAATGATGGCGTCGTTAAC
AGCCCTTGTGCTCGGCCAAGAAAAACCAGCCATGCCCTCTTTCTTCTTGG
CGGGCAGACTTTCATGTGCGACAAGTTGTACCTGGTGGACCAGAAGGCCA
AAGAGATAATTCCCAAAGCGGACATTCCCAGTCCCAGGAAGGAGTTCAGC
GCCTGTGCCATTGGTTGTAAGGTGTACATCACTGGCGGAA--GAGGATC-
GGAGAATGGTGTGTCCAAAGATGTGTGGGTCTACGATACGGTCCATGAAG
AATGGTCAAAGGCTGCACCCATGCTCATCGCCAGGTTTGGCCATGGATCT
GCAGAGCTAAAGCACTGCCTCTACGTCGTGGGTGGACACACTGCGGCAAC
TGGCTGCCTCCCGGCTTCTCCGTCG-------------------------

-----------AAAAAAGACACCAGCAAGGGAACACTGGAAGATCAAATC
ATCCAGGCGAACCCAGCACTGGAGGCCTTCGGCAACGCCAAGACGGTGAG
AAACGACAATTCATCTCGCTTCGGAAAGTTCATCCGAATTCACTTTGGGA
CAAGCGGGAAGCTGTCATCAGCTGATATTGAGACGTACCTGCTTGAGAAG
TCCCGTGTGACCTTTCAGCTCAAGGCTGAGAGGAACTACCACATCTTCTA
TCAGATCCTGTCCAATGAGAAGCCGGAGCTCCTGGACATGCTGCTCATCA
CCAACAATCCATACGACTACTCCTACATCTCCCAAGGAGAGGTAACGGTT
GCCTCCATCAATGACTCAGAGGAGCTGATAGCCACCGACAGCGCCTTTGA
CGTGCTTGGCTTCACCGCTGATGAGAAGATGGGTGTCTACAAACTGACTG
GTGCCATCATGCACTACGGCAACATGAAGTTCAAACAGAAGCAGCGCGAA
GAGCAGGCTGAGCCTGATGGGACTGAGGCTGCCGATAAATCGGCTTACCT
GATGGGACTGAACTCCGCAGACCTCATCAAAGGCCTCTGTCATCCCAGAG
TCAAGGTGGGAAATGAGTATGTGACCAAGGGCCAAAGTGTGGATCAAGTC
TACTATCCGAACAAGGAGGCCTTCAAGTGTGACGAGTGCGGGAAGCACTA
CAACACCAAGCTGGGATACAAGCGCCATGTGGCCATGCACTCTGCCACTG
CCGGGGATCTAACCTGTAAGGTGTGCATGCAGAGCTACGAGAGCACGCCA
GTCCTCCTGGAGCACCTGAAGAGTCACTCGGGGAAATCTTCGGGCGGAGC
CAAGGAGAAGAAGCACCCTTGTGACCACTGTGACCGACGTTTCTACACCC
GGAAGGACGTGAGACGGCACATGGTGGTGCACACAGGTCGTAAGGATTTC
CTGTGCCAGTACTGCGCCCAGCGCTTCGGCAGGAAAGACCACCTGACGCG
GCACGTGAAGAAGAGCCACTCTCAGGAGCTCCTGAAGATCAAGACAGAGC
CTCCAGACATGTTGGGTCTGTTGGCGTCAGGGTCTTCTCCATGTCCTGTC
AAGGAGGAGCTCAGCCCCATGATGTGTGCCATGGCGCCCAACAAAGACCC

CATGATGGGCAAGTCCTTCCCCAGCGGGGCGCCATTCCCCATGAGCATGT
ATAACCCCCATCCCCACCCGCTCCAGGCCATGTCCAGCCC---------C
CACCCT------CACCCCTCCCTGATGCCCGGGTCACTGTCTGCAGCGAT
GGGCATGGGCTGCCACATGGAGTATCTCATCTATGCCTCTTTCTCATTTA
TGGGATGTTTACAAATCAGTGATGGATCAAATATCGTGAACTTGCTGGCT
AGCAACTCCCCAAGCGTTTCGTATGCTCTGACCCAGCAGAAATACTTCAG
TAACTATAGTCCTGTGATTGGTTTTTACATTTATGAGCCCATCGAGTACT
GGAACTCCACAGTGCAGGAGCACCTCAAAACTCTGAGTCATGGCTTCAAC
AAGATCTCCTGGATGGACAACTTTTTTCACTACCTACGGGTAGTAAATGT
GAGCGCGTCCACCAAGAGTGACTTCATCAATATCCTAAAGGCTTCCTTCC
TACGCAGCCCGGAGTACCAGCATTTCACCGAGGATATCATATTCACCAAG
A---ACCGCGAGACTG------ACGAGTATGACATTATTGCCTCACGGAT
GTACTTGGTAGCACGGACCACAGAGAAGAAGCGAGAGGAGGTGGTGGAGC
TCTTGGAGAAGCTCCGCCCCTTGATGCTTATCAACAGCATCAGATTCATT
GCCTTCAATCCCACATTTGTTTTCATGGACCGCTACAGCTCCTCAGTCAT
CTCGCCCATCCTGACTTCAGGCTTCAGTGTACTCACCATCCTCATCCTCA
CTTTCTTCCTGGTCATCAATCCACTGGGGAACTTCTGGCTCATTCTCACA
GTAACGTCCGTGGAGCTGGGTGTCCTGGGTTTGATGNNNNNNNNNNNNNNNNNNNNNNNNNNNNNNNNN
NNNNNNNNNNNNNNNNNNNNNNNNNNNNNNNNNNNNNNNNNNNNNNNNNNNNNNNNNNNNNNNNNNNNN
NNNNNNNNNNNNNNNNNNNNNNNNNNNNNNNNNNNNNGAAGTTGCACTGGT
GGCAGCATTAAAGGATCTGGAGGAGGACATCATAGAGGGGCTGAGGCAGA
ATGAGTTGGAGGACAGCACTTGCACTTCAGGCTTTAGTGTCATGATCAAG
GAATGCTGTGATGGCATGGGTGATGTCAGCGAGAAACATGGTGGGGGACC
AGCTCTTCCTGAGAAAGCTGTCCGCTTCTCTTTCACTGTCATGTCTATCT
CTGTCCAGGAAGAAGGTGAGAAT---------------------------
---GAAGCGGTTACAGTTTTCACTGAAGCAAAGCCAAACTCAGAGCTGTC
ATGTAAGCCACTTTGTCTGATGTTTGTGGACGAGTCAGACCACGAGACAC
TCACAGCTGTCCTGGGGCCTATAATTGCTGAGCGGAATGCAATGAAAGAG
AGCCGGCTAATCCTATCCATAGGAGGCCTGCCACGCTCCTTTCGCTTCCA
CTTCAGGGGTACGGGATATGATGAAAAGATGGTGCGTGAGATGGAGGGCC
TGGAGGCCTCCGGGTCCACTTACGTCTGCACATTATGCGACTCAAGTCGT
GCAGAGGCTGCTCAAAACATGGTGTTACATTCAGTCACCCGCAGCCATGA
TGAGAATCTTGAACGTTATGAAATATGGAGAAGCAACCCCTTTTCTGAGT
CTGTGGAAGAGCTACGAGACAGAGTCAAAGGAGTCTCTGCCAAGCCATTC
ATGGAGACACAGCCCACACTAGATGCATTGCACTGTGACATTGGAAATGC
CGCTGAATTCTACAAAATCTTCCAGGATGAGATCGGTGAAGTGTTCAAAA
AAGC---CAAC---CCCAGCCGCGAGGAACGGCGCAGCTGGAGGGCAGCG
TTAGATAAACAGCTAAGGAAGGGGATGAAGCTTAAACCAGTAATGAGGAT
GAATGGGAACTACGCCCGCAAACTAATGACTCTGGAGGCTGTGGAGGTGG
TGTGTGAGCTAGTGCCCTCAGAGGAGAGAAGAGAGGTCCTGAGAGAGCTT
ATGAGGCTGTACCTCCAGATGAAGCCTGTGTGGCGCTCCACCTGTCCAGC
CAAAGAGTGTCCTGACCAGCTGTGCCGCTACAGTTTTAACTCACAGCGTT
TTGCTGACCTGCTCTCCTCTGCCTTCAAATATAGGTACAATGGAAAGATA
ACCAATTACCTGCACAAGACCCTGGCTCANNNNNNNNNNNNNNNNNNNNNNNNNNNNNNNNNNNNNNNN
NNNNNNNNNNNNNNNNNNNNNNNNNNNNNNNTCTTACACTATTGAGATGGGT
CCTATGGGTCCTCTGTGGAAGGATAACCCACAGCCTTTCACCTGCTCCAT
TGAAGACCCCACCAAACAGACTAAGTTCAAGGGTATCAAGACCTACATAT
CGTACCGGGTGACACCAAGCCACACTGGGCGTCCTGTCTACCGACGCTAC
AAACACTTTGACTGGCTGTACAATCGATTGCTGCACAAGTTCACTGTGAT
ATCTGTGCCTCACCTGCCCGAGAAGCAGGCCACAGGGCGATTCGAGGAAG
ACTTCATTGAGAAACGGAAGAGGCGTCTGATACTGTGGATGAACCACATG
ACCAGTCACCCAGTCCTCTCCCAGTATGAAGGCTTTGAGCACTTTCTGAT
GTGTGCAGATGATAAGCAGTGGAAGCTGGGCAAGAGGCGGGCAGAGAAGG

ATGAGATGGTGGGTGCCCATTTCATGCTGACCCTCCAAATCCCCAATGAG
CACCAAGATCTTCAGGATGTTGAGGAGAGAGTTGACAACTTCAAGGTCTT
TGCCAAGAAAATGGATGACAGTGTGATGCAGCTTACTCATGTTGCCTCGG
AGCTGGTGCGTAAACATCTGGGTGGATTTAGGAAGGAGTTCCAGCGGCTG
GGAAATGCCTTCCAGTCTATAAGTCAGGCATTCCAACTGGACCCTCCACA
TAGGTCAGACACCCTCAACAATGCCATNNNNNNNNNNNNNNNNNCGTTCCTCAAACTGACCTCTCTG
GGTTTCATCATTGGAGTCGGTGTGGTAGGGAACCTCCTGATCTCCATCTT
GCTGGTCAAAGACAAGAGCCTGCACCGAGCACCCTACTATTTTCTGTTGG
ATCTGTGTGCGTCTGACATCCTGCGCTCTGCCATCTGCTTCCCCTTTGTC
TTCACTTCAGTAAAGAATGGATCTGCCTGGACCTACGGCACGCTGACCTG
CAAAGTGATTGCCTTCCTGGGTGTGCTTTCCTGTTTCCACACAGCATTCA
TGCTGTTTTGTGTCAGTGTCACCCGCTACCTGGCCATCGCTCATCACCGT
TTCTACACCAAGAGGCTGACTTTTTGGACGTGTCTAGCCGTCATCTGCAT
GGTGTGGACATTGTCAGTGGCTATGGCATTCCCACCGGTGCTCGACGTAG
GGACGTACTCTTTTATCCGGGAGGAGGACCAGTGCACATTTCAGCACCGT
TCCTTCAGGGCGAATGATTCACTGGGCTTCATGCTCCTGCTGGCGCTCAT
CCTCTTGGCCACACAGCTGGTTTACCTCAAACTCATTTTCTTCGTCCATG
ACCGTCGAAAGATGAAGCCTGTCCAGTTCGTGCCTGCCGTCAGCCAGAAC
TGGACCTTCCATGGGCCGGGTGCCAGCGGGCAGGCAGCAGCCAACTGGCT
GGCTGGATTTGGTCGAGGCCCCACCCCTCCTACTTTGCTGGGGATCCGAC
AGAACAGCAACGCAGCGGGCCGCAGACGTCTACTTGTATTGGATGAATTC
AAAACAGAGAAGAGGATTAGTAGGATGTTCTACATCATGACGTTTTTCTT
CCTGGCTCTGTGGGGGCCCTACCTGGTTGCCTGCTACTGGCGAGTGTTTG
CAAGGGGCCCCGTGGTCCCTGGAGGCTACTTGACAGCAGCTGTGTGGATG
AGCTTTGCCCAGGCTGGGGTCAACCCTTTCATCTGCATCTTCTCCAACNNNNNNGC
CAAATCTCGCTTTCACCCTGGCATGGGGACTGGTCCTGGCACGGAGC---
GCAGCGTCCCACTCGGCAACAGCTTGCTGTCCCCGCAGCAAACCGAGGAG
CCCACTGTTGCCACCCCCCCGCAGCGATGGTTTGTCACCC---CTGCCAA
CAACCGACTGGACTTTGCTGCCTCGGCATACGACGCCGCT---------G
ATTTCGCCGGTAACGCGGCCACCTTGCTGTCCTACGCAGCGGCCGGAGTG
AAGGCTC------TCCCCCTGCCGACTGCGGGCTGCTCCAACCGGCCTCT
TGGCTATTACGCAGACCCGTCTG---GCTGG---GGAGGACGCACGCCGC
CGCAGTACTGCGGCGTAAACACTAAATCCAGCTCGGTGTTTTCCTGCTGG
CCCGCCAACTCCATCGGTGGGAGAGCGGGCA---CC---AACTACCTGT-
---------CCGAGGA---GGGA---GACTC---CCTCGCCACGGAGAGGT
CGCCC---AT---CGGTGGCTCGGAGGAG---ACGAAACCCAAAGACATG
AC---ATCCGA---GTCGAGCTGGATAGAG---ACGCCATCCTCCATTAA
ATCCATCGACTCGAGCGATTCTGGGATCTTTG---AACAAGCCAAACGGA
GGAGGATCTCACCTTCTGCCACACCG---------GTTTCAGAGGCAGTG
TCCCCGCTAAAATCTGAGNNNNNNNNNNNNNGGGGAAGTCACAGAGAGAGAAGTGGCGCTGGG
GATAAACCCGTTCGCGGATGGGATGGGCGCCTTCAAAATAAACCACAGTT
CCCACGACATCGGCTCCGG---ACAGACGGCGTTCTCCTCCCAGGCG---
CCCGGCTAC---GCGGCGGCTGCCCTGGGA---CACCACCA------CCA
CCCGACCCACGTTGGCTCC---TACTCCACGGCGGCGTTCAACTCCACCA
GGGACTTTCTTTTCAGAAACAGGGGCTTCGGAGATGCCGCCGG-------
-----GGCGCAGCACAGCCTGTTCGCCTC---------CGGAAGTTT---
C------GCAGGGCCACATGGACACTCAGATGCAGCGGGGCACCTGCTCT
TCCCGGGGCTGCACGAG---CAAGCGGCCAGCCACGCGTCTTCCAACGTG
GTCAACGGCCAGATGAGGCTGGGCTTCTCGGGGGACATGTACGGACGGGC
CGAGCAGTACGGCCACGTTACGAGCCCACGAT---CCGACCACTACGCCT
CCACTCAGCTGCACGGCTACGGCCCCATGAACATGAATATGGCCGCG---
CACCACGGAGCGGGGGCCTTCTTCAGATACATGAGGCAGCCCATCAAACA
AGAGCTCATCTGCAAGTGGATCGAGCCGGAGCAGCTGACGAACCCCAAAA

AGTCGTGCAACAAAACTTTCAGCACGATGCACGAGCTGGTGACCCACCTG
ACTGTGGAGCATGTGGGGGGACCGGAGCAGACCAACCACGTCTGCTTCTG
GGAGGACTGCTCCAGAGAAGGGAAGCCCTTCAAAGCCAAATACAAACTTG
TGAATCATATCAGAGTACACACCGGAGAAAAACCCTTTCCGTGTCCGTTC
CCTGGTTGTGGCAAA
>Assurger anzac
AGCCTCCTCATCCGAGCTGAACTGAGCCAACCAGGCTCTCTTCTCGGAGA
CGACCAGATTTATAATGTGATTGTTACGGCGCACGCGTTCGTAATAATTT
TTTTCATAGTAATACCCGTTATGATTGGAGGGTTCGGAAACTGACTCATC
CCTCTAATGATTGGGGCCCCAGACATGGCCTTCCCACGAATGAACAACAT
GAGTTTCTGGCTTCTGCCCCCTTCTTTCCTCCTCCTCCTGGCCTCCTCCG
GGGTAGAAGCTGGGGCTGGTACCGGTTGAACCGTGTACCCTCCCTTGGCA
AGCAACCTTGCCCACGCAGGGGCTTCCGTTGATTTAACTATTTTCTCCCT
TCACCTCGCAGGGATCTCCTCGATCCTTGGGGCCATTAACTTTATTACAA
CAATTGTTAACATAAAACCCGCAGCTATTACGCAATTCCAAACCCCGCTA
TTTGTGTGATCCGTCCTAATTACAGCTGTGCTCCTACTACTATCTCTGCC
GGTTCTTGCAGCCGGAATTACAATACTCCTCACAGACCGCAACCTTAATA
CAACCTTCTTTGACCCAGCAGGAGGGGGCGATCCCATCCTCTACCAACAC
TTG---

------------TTCTTAGAGAGAAACCTTCACCCGACTAACTGCCTTGG
CATGCTGTTGCTGTCTGACGCCCACCAGTGCACCAAGCTGTCAGAGCTTT
CTTGGGGCATGTGCCTCAGCAACTTCCCCGCTATTTGCAAGACAGAGGAC
TTCCTCCAACTGCCCAAAGACATGGTGGTGCAGCTTTTGTCACATGAGGA
GCTGGAGACAGAAGATGAGAGACTGGTTTATGACGCAGCCCTGAACTGGA
TCAACTATGACCTGGAAAGGAGGCACTGTCACCTTCCAGAGCTCCTGAGA
ACGGTCCGTCTTGCCCTGCTGCCCGCCATCTTTCTAATGGAGAATGTCTC
AACAGAAGAGCTTATCAATGCCCAGGCCAAGAGCAAGGAGTTGGTGGATG
AGGCTATCCGCTGTAAGCTGAAGATCCTGCAGAATGACGGCGTCGTTAAC
AGTCCATGTGCTCGGCCAAGAAAAACCAGCCATGCTCTCTTTCTCCTGGG
AGGGCAGACTTTCATGTGCGACAAGTTGTACCTGGTGGACCAGAAGGCCA
AAGAGATCATCCCAAAGGCGGACATTCCCAGTCCCAGGAAGGAGTTCAGC
GCCTGTGCCATTGGCTGTAAGGTGTACATCACAGGTGGGA--GAGGCTC-
AGAGAATGGCGTGTCCAAAGACGTATGGGTCTATGACACCGTCCATGAGG
AATGGTCCAAAGCGGCGCCCATGCTCATCGCCAGGTTTGGTCATGGCTCT
GCTGAGCTGAAACACTGCCTGTATGTGGTAGGAGGTCACACTGCGGCAAC
TGGCTGCCTCCCCGCTTCTCCATCCAGATGAATACGTTGTTGTGTTCAGT
CGTTCAACGACAAGGCTGATACTGAACGAAGCCGAGCTAATCATGGCTCT
GGCCCAGGAGTTCCAGATGAGAGTGGTCACAGTTTCCCTAGAGGAACAGT
CTTTCTCCAGCATTGTCCAGGTGATCAGCGGTGCTTCCATGTTAGTCAGC
ATGCATGGAGCTCAGCTTATCGCCTCACTCTTCCTCCCCAGAGGAGCTAC
TGTAGTCGAGCTGTTCCCCTTTGCTGTGAACTCAGAACAGTACACCCCAT
ATAAAACCCTTGCCTCTCTTCCAGGCATGGACCTTCATTATGTTTCCTGG
AGGAACACTCAGGAGGAGAACACCGTCACCCATCCAGACAGACCCTGGGA
ACAAGGWGGCATCACTCACTTGGAGAAGGAGGAGCAGGAGCGCATACTGT
CCAGCAAAGACGTCCCCAGGCACCTGTGCTGCCGCAACCCAGAGTGGCTC
TTCCGGATCTACCAGGACACTTTGGTAGACATACCTTCCTTCTTGGAAGT
TCTCAA---GGAGGGAATGAAG---ACCAAGCCAAGTTTGAAGAA---GT
CCAAACCAGCCAGTACAGTCCATCCGGGCCGGGTCAGAGAAGCCCAGTGT
CAGACCTCAGTACAAACCACCAACGAGGCTAAACTCACAGTCTCCTGGCA
GATCCCGTGGAATCTGAAATACCTGAAAGTAAGAGAGGTGAAGTATGAGG

TGTGGATCCAGAAAAGAGACACCAGCAAGGGAACTCTGGAGGATCAAATC
ATCCAGGCAAACCCAGCGCTGGAGGCCTTTGGAAATGCCAAAACGTTGAG
AAACGACAACTCATCTCGGTTTGGAAAATTCATCCGAATTCACTTTGGAA
CTAGCGGCAAGCTCTCGTCCGCTGATATCGAGACATACCTGCTGGAGAAG
TCTCGTGTCACCTATCAGCTAAAGGCTGAGAGGGACTACCACATCTTCTA
CCAGATCCTGTCCAATCAGAAGCCAGAGCTGCTGGACTTGCTGTTAATCA
CTAACAACCCATACGACTACTCGTACATCTCCCAAGGAGAGGTGACAGTC
GCCTCCATCAATGACTCGGAGGAGCTGATGGCCACTGACAGCGCCTTCGA
TGTGCTCGGATTCACTAATGAGGAGAAGATGGGCGTCTATAAACTGACCG
GTGCCATCATGCACTATGGCAACATGAAGTTCAAGCAGAAGCAGCGGGAG
GAGCAGGCCGAGTCTGATGGGACGGAGGCTGCTGATAAATCAGCTTACCT
GATGGGGCTGAACTCTGCTGACCTCATCAAAGGGCTGTGCCATCCCAGAG
TCAAGGTAGGAAATGAGTACGTCACCAAAGGCCAGAATGTGGACCAAGTC
TACTATCCCAACAAGGAGGCCTTCAAGTGTGAGGAGTGTGGGAAGCACTA
TAACACCAAGCTGGGATATAAGCGCCATGTGGCCATGCACTCTGCCACAG
CGGGGGATCTCACCTGTAAAGTGTGCATGCAGAGCTACGAGAGCACACCT
GTTCTTCTGGAGCACCTTAAGAGCCACTCGGGGAAGTCCTCTGGCGGAGC
CAAGGAGAAAAAGCACCCATGCGATCACTGCGACCGCCGTTTCTACACGC
GGAAGGATGTGAGACGGCACATGGTGGTCCACACGGGCCGAAAGGACTTC
CTGTGCCAATACTGTGCCCAGCGCTTTGGCAGGAAGGACCACCTGACCCG
CCACGTGAAGAAGAGCCACTCGCAGGAGCTGCTGAAGATCAAGACGGAGC
CTCCTGATATGCTAGGTCTTTTAGCTTCGGGGTCACCACCTTGCTCTGTG
AAGGAGGAGCTTAGCCCCATGATGTGCGGCATGGGTCCCAATAAAGACCC
CATGATGGGCAAACCATTTCCCAGTGGCGCCCCTTTTCCGATGGGCATGT
ACAACCCCCACCAC------CTTCAGGCCATGTCTAATTCTGGGGTGGGT
CATCCA------CACCCGTCCCTGATGCCCAGCTCCCTGTCTTCAGCTAT
GGGCATGGGCTGTCACATGGAANNNNNNNNNNNNNNNNNNNNNNNNNNNNNNNNNNNTTTACAGATCAGT
GATGGATCAAACATT
GTGAATCTTCTGGCTAGTAACTCTCCGAGTGTTTCGTATGCTGTGACCCA
GCAGAAATACTTCAGTAACTACAGTCCCGTGATTGGGTTTTACATTTACG
AGCCCATCGAGTACTGGAACTCCACGGTGCAGGAGCACCTGAAGACTCTG
AGTCACGGCTTCAACAAGATCTCCTGGATGGACAACTTCTTCCACTATCT
GCGGGTGGTGAATGTGAGCGCCTCGACCAAGAGCGACTTCATCACCATCC
TCAAGGGCTCCTTCCTGCGTAGCCCAGAGTACCAGCACTTCACTGAGGAC
ATCATATTCTCCAAGA---ACCGTGAGACTG------ATGAATACGACAT
TATTGCCTCACGCATGTATCTGGTGGCTCGGACCACAGAGAAGAAGCGAG
AGGAGGTGGTAGAGCTTCTGGAGAAGCTTCGCCCGTTGATGCTGATCAAC
AGCATCAAGTTCATTGCCTTCAATCCCACATTTGTGTTCATGGACCGCTA
CAGCTCCTCCGTCATCTCGCCCATCCTGACCTCAGGCTTCAGCGTGCTCA
CCATCCTCATCCTCACTTTCTTCCTGGTCATCAACCCCTTGGAAAACTTT
TGGCTCATCCTCACGGTTACGTCTGTGGAGCTGGTCGTCTTGGGGTTGAT
GNNNNNNNNNNNNNNNNNNNNNNNNNNNNNNNNNNNNNNNGTGTCTGCCTCTTGCAATGTTGGCATTATT
AATGGGCTCTCTGGATGGC
CTTCCTCGGTGGATGACTCCCCAGCTGACACCATCACTCGGCGGTTCCGC
TATGATGTGGCACTAGTGTCAGCGTTAAAGGATCTGGAGGAGGACATCAT
GGAAGGGCTGAGAGATAGTGGGATGGAAGACAGTGCTTGCACATCAGGCT
TCAGTGTTATGATCAAGGAATCTTGTGATGGCATGGGCGATGTCAGCGAA
AAGCACGGTGGAGGACCAGTTATTCCTGAGAAGGTTGTACGTTTCTCTAT
CACTATTATGTCTGTCTCTGTCCAGGCAGATGATGAGGAG----------
--------------------GARGCGGTTACTATCTTCACAGAACCAAAG
CCAAACTCAGAACTGTCCTGTAAGCCCCTTTGCCTGATGTTTGTGGATGA
ATCAGACCATGAGACACTCACAGCTCTCCTGGGGCCTGTAGTTGCAGAGC
GTAATGCAATGAAAGAGAGCAGGCTCATCCTTTCCATCGGCGGCCTGCCT

CGCTCCTTCCGCTTCCACTTCAGAGGCACAGGATATGATGAGAAGATGGT
GCGAGAGATGGAGGGCCTGGAGGCCTCAGGGTCCAGCTATGTCTGCACTC
TGTGTGATTCCAGTCGGGCAGAGGCATCTCAAAACATGGTGCTACACTCA
ATCACCCGCAGCCACGAAGACAACCTTGAACGTTACGAAATATGGAGAAC
AAACCCCTTTTCTGAGACTGGAGAAGCGCTGCGGAGCAGAGTCAAAGGGG
TCTCTGCCAAGCCCTTCATGGAGACCCATTGCACACTGGATGCATTACAT
TGTGACATTGGCAATGCCATTGAATTCTACAAAATCTTCCAGGACGAAAT
TGGAGAGCTGTACCAAAAGGT---GAAT---CCCAGCCGGGAGGAACGAC
GCAGCTGGAGGGCAGCCCTAGATAAACAGCTGAGGAAGAAGTTGAAGCTT
AAACCGGTAATGAGGATGAATGGGAACTATGCACGCAGGCTAATGACCCA
GGAGACTGTGGAGGTGGTGTGTGAGCTGGTGCCCTCAGAGGAGAGGAGGG
AGGCCCTGAGGGAGCTTATGAGGCTATACCTCCAGATGAAGCCTGTGTGG
CGTGCCACCTGCCCAGCCAAGGAGTGCCCTGACCAGCTGTGCCGCTATAG
CTTTAACTCCCAGAGCTTTGCTGACCTCCTCTCCTCTACCTTCAAATATA
GGTACAATGGAAAGATAACCAATTACCTGCACAAGACCCTGGCCCATGTC
CCTGAAATCATAGAGAGAGAGNNNNNNNNNNNNNNNNNNNNNNNNNNNNNNNNNNNNNNNNNNNNNNT
CATACACCATCGACATGTGTCCCCTGGG
ACCCCAGTGGAAGGAGAGCCCACAGCCTTTCTCCTGCTCCGTCGAAGACC
CCACAAAACAGACAAAGTTCAAAGGCATCAAGACGTACATTTCGTACCGG
GTCACGCCGAGCCACACCGGGCATCCCGTCTACAGGCGCTACAAACACTT
TGACTGGCTGTACAACCGCCTACTGCACAAGTTCACTGTGATCTCCGTGC
CTCACCTGCCTGAGAAGCAGGCCACGGGGAGATTTGAGGAAGACTTTATA
GAGAAGCGAAAGARGCGACTGATACTGTGGATGAACCACATGACCAGTCA
CCCTGTCCTCTCCCAGTATGAAGGCTTTGAGCACTTTCTGATGTGTGCCG
ACGACAAGCAATGGAAGCTGGGCAAGAGACGAGCTGAGAAGGACGAGATG
GTGGGCGCCCATTTCATGCTGACCCTCCAGATCCCTAACGAGCACCAAGA
CCTTCAGGATGTTGAGGAGAGAATCGACACCTTCAAGGCCTTTGCTAAGA
AAATGGACGACAGCGTCATGCAGCTCACACATGTTGCCTCGGAGCTCGTG
CGTAAGCACCTGGGTGGATTCCGGAAGGAGTTCCAGCGGCTGGGATATGC
CTTCCAGTCTATTAGTCAGGCATTCATGCTGGACCCTCCCCATAGCTCAG
AAACTCTCAACAACGCCATCTCCCATNNNNNNNNNNNNNNNNNNNNNNNGACCTCTCTGGGTTTTATCAT
TGG
AGTCGGTGTGGTTGGAAACCTCCTGATCTCCATCCTGCTGGTCAAAGACA
AGAGCCTGCACCGAGCGCCCTACTACTTCCTGCTGGACCTGTGCGCCTCA
GACATCCTTCGCTCCGCCATCTGCTTCCCCTTCGTCTTCACCTCGGTCAA
GAATGGATCTGCCTGGACCTATGGCACGCTGACCTGCAAAGTGATCGCCT
TCCTGGGTGTGCTCTCCTGTTTCCACACGGCGTTCATGCTATTCTGCGTG
AGCGTCACCCGCTACCTGGCCATCGCGCATCACCGTTTCTACACCAAGAG
GCTGACCTTCTGGACCTGTCTGGCTGTCATCTGCATGGTGTGGACGTTGT
CGGTGGCTATGGCGTTCCCGCCGGTGCTAGACGTAGGGACGTACTCTTTT
ATCCGGGAGGAGGACCAGTGCACATTCCAGCACCGCTCCTTCAGGGCGAA
TGATTCGCTGGGCTTCATGCTCCTGCTGGCGCTCATTCTCCTGGCCACAC
AGCTGGTTTACCTCAAGCTCATCTTCTTTGTCCACGACCGTCGAAAGATG
AAGCCTGTCCAGTTCGTGCCTGCTGTCAGCCAGAACTGGACCTTCCACGG
GCCAGGCGCCAGCGGGCAGGCGGCGGCCAACTGGCTGGCTGGATTTGGTA
GAGGCCCCACCCCGCCTACTTTGCTGGGCATCCGGCAGAACAGCAACGCA
GCGGGCCGCAGGCGTCTACTGGTATTGGATGAATTCAAAACAGAGAAGAG
GATTAGTAGGATGTTCTACATCATGACATTTTTCTTCCTGGCACTGTGGG
GGCCCTATCTGGTAGCCTGTTACTGGCGGGTGTTTGCAAGGGGCCCTGTA
GTCCCTGGGGGCTACCTGACGGCAGCCGTGTGGATGAGCTTTGCCCAGGC
TGGGGTCAATCCTTTCATCTGNNNNNNNNNNNNNNNNNNNNNNNNNNNNNNNNNTCACCCTGGCGTGGGG
ACTGGTCCTGGCA
CGGAGC---GCAGCGTCCCACTCGGCAACAGCTTGCTGTCCCCGCAGCAA

AACGAGGAGCCCACTGTTGCCACCCCCCCGCAGCGCTGGTTTGTCACCC-
--CTGCCAACAACCGACTGGACTTTGCTGCCTCGGCATACGACGCCGCT-
--------GATTTCGCCGGTAACGCGGCCACCTTGCTGTCCTACGCAGCG
GCCGGAGTGAAGGCTC------TTCCCCTGCCGACTGCAGGCTGCTCCAA
CCGGCCTCTTGGCTATTACGCAGACCCGTCCG---GCTGG---GGAGGAC
GCACGCCGCCGCAGTACTGCGGCGTGAACAGCAAATCCAGCTCGGTATTT
TCCTGCTGGCCCGCAAACTCTATCAGCGGCAGAGCGGGCG---GC---AA
CTACCTGT---------CCGAGGA---GGGA---GACTC---CATCCCCA
CGGAGAGGTCGCCC---AT---C---GGCTCCGAGGAG---ACCAAACCC
AAAGACATGAG---CTCAGA---GTCCAGCTGGATAGAG---ACGCCGTC
ATCCATCAAGTCCATCGATTCCAGTGACTCTGGAATCTTCG---AGCAGG
CCAAGAGGAGGAGAATCTCTCCTTCTGCCACCCCG---------GTGTCA
GAGACAGTGTCCCCGNNNNNNNNNNNNNNNNNNNNNNNNNNNNNNNNNNNNNNNNNNNNNNNNNNNNNNN
NNNNNNNNNNNNNNNNNNNNNNNNNNNNNNNNNNNNNNNNNNNNNNCAGCTCCCACGACATTGGCTCCGG
---ACAAACGG
CGTTTTCCTCCCAGGCA---CCCGGCTAC---GCAGCTGCCGCCCTGGGA
---CATCACCA------CCACCCGACCCACGTTGGCTCT---TACTCCAC
GGCGGCTTTCAACTCCACCAGGGACTTTCTCTTCAGAAATCGGGGTTTCG
GGGACGCCACCGG------------GGCGCAGCACAGTTTGTTCGCCTC-
--------CGGAAGTTT---C------GCAGGGCCACATGGACACTCGGA
TGCAGCGGGGCACCTGCTCTTCCCGGGGCTCCACGAG---CAAGCGGCGA
GCCATGCCTCTTCCAACGTGGTCAACAGCCAGATGCGGCTGGGCTTCTCG
GGGGACATGTACGGACGGGCTGACCAGTACGGCCACGTTACGAGCCCGCG
GT---CCGACCACTATGCTTCGACCCAGTTGCACGGCTACGGCCCCATGA
ACATGAATATGGCCGCG---CACCACGGAGCAGGGGCCTTCTTTCGATAC
ATGAGGCAGCCGATCAAACAAGAGCTCATCTGCAAGTGGGTCGAGCCGGA
GCAGCTGACGAATCCCAAAAAGTCGTGCAACAAAACTTTTAGCACGATGC
ACGAGCTGGTGACCCACCTGACGGTGGAGCATGTGGGGGGACCGGAGCAG
ACCAACCACATCTGCGTCTGGGAAGAGTGCACCAGAGAAGGGAAGCCATT
CAAAGCCAAATACAAACTTGTAAATCATATCAGAGTACACACCGGAGAAA
AGCCCTTCCCGTGTCNNNNNNNNNNNNNNNNNNNN
>Astyanax mexicanus
AGCCTGTTAATTCGGGCAGAGCTCGGCCAACCGGGATCCCTTCTAGGTGA
TGACCAGGTATATAATGTTCTGGTAACTGCACATGCTTTCGTTATAATTT
TCTTTATAGTTATACCCGTTATAATTGGAGGCTTTGGAAATTGACTAATC
CCACTTATAATTGGCGCCCCAGACATAGCCTTCCCTCGAATAAATAACAT
AAGTTTTTGACTTCTTCCACCATCTTTTCTTCTCCTTCTAGCATCCTCTG
GGGTCGAGGCTGGAGCAGGGACAGGATGAACTGTATACCCCCCCCTTGCT
GGCAATCTTGCACATGCAGGAGCTTCCGTTGACCTGACCATTTTCTCACT
CCACTTAGCTGGTGTCTCCTCAATTTTAGGAGCAATCAATTTTATTACAA
CTATTATTAATATAAAACCCCCAGCTATTTCGCAATACCAAACACCCTTG
TTTGTCTGAGCTGTCTTAGTAACGGCTGTCCTCCTTCTTCTTTCACTCCC
TGTCTTAGCAGCTGGAATCACAATACTTCTTACAGATCGTAACCTTAATA
CTTCATTTTTTGACCCAGCGGGAGGTGGAGATCCTATCCTTTATCAACAC
CTCNNNNNNNNNNNNNNNNNNNNNNNNNNNNNNNNNNNNNNNNNNNNNNNNNNNNNNNNNNNNNNNNNNN
NNNNNNNNNNNNNNNNNNNNNNNNNNNNNNNNNNNNNNNNNNNNNNNNNNNNNNNNNNNNNNNNNNNNNN
NNNNNNNNNNNNNNNNNNNNNNNNNNNNNNNNNNNNNNNNNNNNNNNNNNNNNNNNNNNNNNNNNNNNNN
NN--

----------------CGATGATTATATTGTGGTTTTTAGTCGTGCGTCT
AACAGACTCATACTCAATGAAGCCGAGTTGATTCTAGCTTTGGCTCAGGA
GTTCAAGATGAGGACAGTCACTGTGTCCTTAGACGAGCAGCCATTCGATA
GCATAGTCCGGGTCATCAGTGGGGCATCCATGTTGGTCAGCATGCACGGA
GCCCAGCTGATCACCTCCATGTTTCTTCCGCGAGGAGCTGCTGTGATCGA
ACTCTTCCCGTATGCAGTCAACCCARAACAGTACACTCCGTACAAAACCC
TGGCCTCTTTACCAGGCATGGATCTCCAGTATATATCATGGAGAAACAAC
ATTGAGGAGAATTCCGTGACCTATCCTGACCGCCCTTGGGATCAGGGAGG
CATCACACACTTGGAGAAAGAGGAGCAGGAGCGTATCCTCACTAGCAAGG
AGGTACAAAGGCACCTCTGCTGCCGCAACCCTGAGTGGCTTTTCCGGATT
TATCAAGACACCATTGTGGACGTTCCGTCCTTTTTGGAAGTCCTTAA---
AGAAGGTCTAAAA---GTTAAGCCTAATCTGAAAAA---GTCTAAGCCTG
CCAGCACAGTACATCCCGGTCGAGTCCGAGAACCCAAGTGTCAGACATCG
GTTCAAGCTNNNNNNNNNNNNNNNNNNNNNNNNNNNNNNNNNNNNNNNNNNNNNNNNNNNNNNNNNNNNN
NNNNNNNNNNNNNNNNNNNNNNNNNNNNNNNNAAGAAGGACGCAAACAAGGGCACCCTGGAGGATCAAAT
CAT
CCAGGCAAATCCTGCACTTGAGGCCTTTGGCAACGCCAAAACAGTAAGAA
ATGACAACTCCTCTCGTTTTGGAAAGTTTATCCGCATTCATTTTGGAACA
AGTGGCAAACTTTCTTCAGCTGACATCGAAACATATCTGCTGGAAAAGTC
CCGAGTTACCTTTCAGCTAAAATCAGAAAGAAACTACCACATCTTCTTTC
AGATCTTATCCAATGCCAAGCCAGAGCTGCTGGATATGCTTCTGATTACA
AACAATCCCTACGATTACTCTTACATCTCCCAAGGAGAAGTAACTGTTGC
AAGTATCAATGACAGCGAGGAGTTGTTAGCCACTGACAGTGCCTTTGATG
TGCTTGGTTTCACCCCAGATGAGAAGATAGGAGTGTACAAATTGACTGGT
GCCATCATGCATTATGGGAACATGAAGTTCAAGCAAAAGCAGCGTGAGGA
ACAGGCTGAACCTGATGGTAGCGAAGCTGCTGATAAATCTGCCTACCTAA
TGGGGCTAAACAGTGCAGATCTCCTGAAGGGACTCTGCCATCCGAGGGTC
AAGGTAGGAAATGAATATGTCACAAAAGGTCAAGGTGTGGATCAAGTATA
CTACCCCAACAAAGAGGCCTTCAAGTGTGAGGAGTGTGGCAAGCACTATA
ACACAAAGTTAGGTTATAAGCGCCACGTGGCCATGCACTCTGCCACAGCG
GGCGACCTGACCTGCAAGGTTTGTCTTCAGAGCTACGAGAGCACTCCTGC
CCTCCTGGAACACCTCAAGAGCCACTCTGGCAAATCTTCGGGCGGCACCA
AGGAGAAGAAACACCCTTGCGACCACTGCGACCGCCGCTTCTACACCCGC
AAGGATGTTCGCCGGCACATGGTAGTCCACACAGGCCGCAAAGATTTCTT
GTGCCAGTACTGTGCCCAGCGCTTTGGAAGGAAGGACCACCTCACGCGGC
ACGTTAAGAAGAGCCACTCTCAAGAGCTGCTTAAGATCAAGACAGAACCT
CCGGATATGTTGGGCTTGCTCGGGACTGGTTCTCCACCTTGTGCCGTGAA
GGAAGAGCTCAGTCCTATAATGTGTGGTATGGGCCCGAACAAGGACTCAA
TGATGACCAAGCCTTTCCGCAGTGGCACCCCTTTCCCAATGGGCATGTAC
ACTCCTCACCAC------CTCCCGGCCATGTCCAACCCAGGGATGGTCA
C---------CACCCGTCTTTGGTTCCTGGATCATTGTCTGCTGCTATGG
GTATGGGATGCCCCATGGAGTACTTGATTTACGCCTCTTTTTCTTTTATG
GGATGTTTACAAATCAGCGATGGCTACAATATAGTAAACCTATTAGCCAG

CAATTCACCCAGTGTGTCATTTGCCCTGACCCAGCAGAAACTGTTCAGCA
ACTACAGCCCTGTGATAGGCTTCTACATTTACGAACCCATTGAATACTGG
AACTCTACAGTGCAGGAGCACCTCAAGACTCTCGGCCAGGGTTTCAATAA
GATCTCGTGGATTGATAACTACTTCAACTATCTAAAAGTTGCCAATGTCA
GTGCATCAACCAAAAGTGATTTTATAAACATTCTCAAGAATTCCTTCCTG
AAGAGCCCGGAGTATCAGCACTTCACGGAGGACATCATTTTCTCGAAGA-
--ACGGGG------------AGGAGTACGACATCATCGCATCTAGGATGT
ACCTGGTAGCTAGAACCACAGAGAAGACCAGGGAGGAGGTGGTGGAACTG
CTGGAGAGACTCAGACCACTCTCCCTCATTAACAGCATCAAGTTCATCGT
CTTCAACCCTACCTTTGTGTTCATGGACCGCTACAGTTCCTCGGTCGTTT
CGCCAATCCTCACATCGGGTTTCAGCGTCCTGACCGTCCTCATCCTGACT
TTCTTCCTCGTCATCAACCCCCTGGGGAACTTCTGGTTGATCCTGACCGT
CACTTCTGTGGAACTGGGCGTCTTAGGCCTCATG----------------
-----------------------GTGTCCAGCTCTTGGAACATAGGCATC
ATAGATGGGCTCTCTGGCTGGACGGTGTCTGTAGAGGACGTGCCAGCAGA
CACCATCTCTAGACGATTCCGCTATGATGTTGCGCTGGTATCAGCTTTGA
AAGACCTGGAGGAGGACATCATGGAGGGTCTGAGAGAGAGAGGGTTGGAT
GACAGCACCTGCACGTCAGGTTTCACTGTGGTCATCAAGGAGTCGTGTGA
TGGCATGGGGGATGTCAGCGAAAAGCACGGAGGTGGACCGGCTGTTCCTG
AGAAGGCTGTGAGATTCTCTTTTACAGTGATGTCCATCACCATTCAAGCG
GAAGGTGAGGAA------------------------------GAAGCAGT
CACGATATTCCAGGAGCAAAAGCCCAACTCAGAACTGTCCTGCCGACCAC
TGTGCCTCATGTTTGTGGACGAGTCCGATCATGAGATGCTGACCGCCATC
CTGGGTCCTGTAGTGGCAGAGCGTAGAGCGATGAAGGAGAGTCGCCTCAT
CCTTTCCATAGGTGGGCTCCTTCGCTCCTTCCGCTTCTTCTTCCGAGGCA
CAGGCTATGACGAGAAGATGGTGCGAGAAATGGAAGGGCTGGAGGCTTCA
GGATCCACTTACGTCTGCACCTTGTGTGACTCAACACGAGCTGAAGCCTC
TCAAAACATGGTGCTGCATTCTATCACTCGAAGCCATGAGGAAAACCTTG
AGAGATATGAGATTTGGAGAACCAACCCTTTCTCAGAGACTGCAGATGAA
CTGCGGGATCGAGTCAAAGGGGTCTCCGCTAAGCCTTTCATGGAGACGCA
GCCCACGCTAGATGCCTTGCACTGTGACATTGGCAATGCTACAGAATTCT
ACAAGATATTCCAGGATGAGATTGGTGAAATGTACCAGAAAGT---CAAC
---CCCGCTCGAGGAGAGACGGCGCTGGCGATCAGCTCTTGACAAGCA
GTTGAGGATGAAGATGAAGCTGAAGCCGGTGATGAGGATGAACGGGAACT
ATGCCCGGAAGTTGATGACCAGTGAGGCTGTGGAGGTGGTGTGTGAGCTG
GTTCCTTCAGAGGAGCGCCGCAAGGCTCTGAGAGAGCTGATGGAGTTGTA
CCTCCAGATGAAGCCGGTCTGGCGCTCTACCTGCCCAGCCAGAGACTGCC
CTGATCAACTCTGCCGTTATAGCTTCAACTCACAACGCTTTGCTGAACTC
TTGTCCAGTACGTTCAAATACCGCTACGATGGCAAGATTACCAACTACCT
CCACAAGACTCTGGCCCATGTGCC--------------------------

>Ateleopus japonicus
AGCTTACTAATTCGAGCTGAATTAAACCAACCCGGTGCTCTTCTGGGAGA
CGACCAGATTTATAATGTAATTGTTACAGCACATGCCTTTGTAATAATCT
TCTTTATAGTCATACCTATCATAATTGGAGGTTTCGGCAACTGACTAATC
CCACTAATGATTGGTGCTCCTGACATAGCATTTCCCCGAATAAACAACAT
AAGTTTCTGACTTCTACCCCCTTCATTTCTGCTACTATTAGCTTCTTCTG
GGGTTGAAGCAGGGGCTGGAACAGGCTGAACTGTTTACCCCCCTTTAGCC
GGTAACTTAGCCCATGCTGGTGCCTCTGTAGATTTAACAATCTTCTCACT
ACACTTAGCAGGGGTCTCCTCTATCTTGGGTGCCATTAATTTCATTACTA
CTATTATTAATATAAAACCCCCAGCCGTCTCACAATATCAAACTCCTTTA
TTTGTCTGATCTGTACTTATTACAGCTGTACTACTTCTTCTCTCTCTCCC
TGTGCTTGCTGCTGGTATTACAATATTACTCACAGACCGAAATTTAAATA
CGACATTCTTTGACCCTGCAGGAGGAGGAGACCCAATCTTATACCAACAC
TTATTCTGGTTCTTCGGTCACCCCGAAGTTTATATTCTTATTCTCCCTGG
GTTCGGCATGATCTCACACATTGTAGCCTACTACTCCGGCAAAAAAGAAC
CTTTCGGATATATGGGCATGGTCTGAGCTATAATAGCCATCGGCCTCCTT
GGGTTTATCGTATGGGCTCACCATATGTTTACAGTAGGGATGGACGTAGA
CACACGAGNNNNTTCCTAGAAAGGAACCTGCACCCATCCAACTGCCTGGGCATG
CTGCTGTTGTCAGACGCTCACCAGTGCACCAAGCTGTCAGAGCTGTCCTG
GGGCATGTGCCTCAGCAACTTCCCTGCCATCTGCAAGACGGAGGAATTCC
TCCAACTGCCCAAAGACATGGTGGTGCAACTATTGTCGCACGAGGAGCTA
GAGACCGAAGATGAGAGACTAGTGTACGAGACTGCCCTTAACTGGATCAA
CTACGACCTTGATAGGAGACACTGCCATCTGCCAGAGCTGCTGAGAACGG
TTCGGCTGGCCCTGCTTCCTGCCATCTTCCTCATGGAAAATGTCTCCACG
GAAGAGCTGATCAACGCCCAGGCCAAGAGCAAGGAACTTGTCGACGAAGC
CATCCGCTGCAAGCTGAAGATTCTGCAGAACGACGGCGTGGTAAACAGTC
CCTGTGCCCGGCCCAGGAAGACCAGCCATGCCCTCTTCCTTCTGGGAGGG
CAGACTTTCATGTGCGATAAGCTTTACTTGGTGGACCAGAAGGCCAAAGA
GATCATCCCCAAGGCTGACATCCCCAGCCCGAGGAAGGAGTTCAGTGCCT
GCGCCATCGGCTGTAAGGTCTATGTCACAGGTGGGA--GGGGCTC-AGAG
AACGGCGTTTCCAAAGATGTTTGGGTCTATGACACCGTTCACGAGGAGTG
GTCTAAGGCAGCCCCCATGCTGATTGCCAGGTTCGGCCATGGCTCTGCCG
AACTGAAACACTGCCTCTACGTGGTGGGAGGACACACGGCAGCCACTGGC
TGCCTCCCAGCCTCCCCCTCTGGACGAGTACATAGTGGTGTTCAGTCGCT
CCTCTACGAGGCTGATTCTGAACGAGCCAGAGCTAATCATGGTGTTGGCG
CAGGAGTTTCAGATGAGGGTGGTCACGGTGTCTCTGGAAGAGCAATCCTT
CCCCAGCATCATCCAGGCGATCAGCGGCGCCTCCATGCTGGTCAGTATGC
ACGGAGCTCAGCTTGTTACCTCCCTCTTCCTCCCCAGAGGGGCTGCCATT
GTGGAGCTCTTCCCCTATGCTGTGAACCCAGAGCAGTATACTCCATACAA
AACCCTGGCGTCCCTCCCAGGCATGGATCTTCAATACGTTTCCTGGAGAA
ACACAATCGAAGAGAACTCRGTTAGCCACCCCGATAGGTCCTGGGACCAA
GGAGGCATTGCTCACCTGGACAAGGAAGAGCAGGAGAGAATTCTCGCCAG
CAAGGAGGTCCCCAGGCACCTGTGCTGCCGCAACCCCGAGTGGCTCTTCC
GAATCTACCAGGACACTCTGGTGGACATCCCCTCATTCCTCAAGACCCTC
AA---GGAGGGCCTAAAG---ACCAGGCCCAGCTTGAAGAA---GTCCAA
GTCGGCCAACACKGTTCACCCGGGTCGGGTCAGGGAAGCCCAGTGCCAGA
CCTCGGTCCAGGACACCAACGAGGCTAAACTCACTGTCTCCTGGCAGATC
CCGTGGAATCTCAAGTACCTGAAGGTACGAGAGGTTAAGTACGAGGTCTG
GATCCAGAAGAAAGATTCAAGCAAAGGTACACTGGAGGATCAAATCATTC
AGGCAAACCCTGCACTGGAGGCTTTTGGTAATGCCAAAACAGTGAGGAAC
GATAACTCATCCCGCTTTGGGAAATTCATCCGAATTCACTTTGGAACCAG

TGGCAAATTGTCCTCTGCCGACATTGAGACTTATCTCCTGGAGAAGTCAC
GTGTCACCTTTCAGCTCAAGGCTGAGAGGAACTATCACATCTTCTTCCAG
ATCCTGTCCAATCAAAAGCCAGAGCTGTTGGACATGATGTTGGTCACCAA
CAACCCATATGACTACTCCTTCATCTCCCAAGGAGAGGTAACAGTAGCAT
CCATCAATGATGCAGAGGAGCTGATTGCCACTGACAGTGCCTTTGATGTC
CTCGGCTTCACCCAGGAGGAGAAGCTGGGAGTTTACAAGTTGACAGGTGC
GATCATGCACTATGGCAACATGAAGTTCAAGCAAAAGCAGCGTGAAGAGC
AGGCTGAGTCTGATGGCACAGAGGCTGCTGATAAGTCAGCTTATCTAATG
GGGCTGAATTCTGCCGACCTCATTAAAGGGCTTTGCCATCCCAGAGTCAA
AGTAGGAAATGAGTACGTCACCAAAGGCCAAGGTGTAGATCAGGTCTACT
ACCCCAACAAGGAGGCCTTCAAGTGTGAGGAGTGTGGTAAGCACTATAAC
ACCAAGCTGGGATACAAGCGCCATGTGGCCATGCACTCGGCCACGGCGGG
CGACCTCACCTGTAAAGTGTGTATGCAGAGCTACGAGAGCACGCCGGTCC
TCCTGGAACACCTCAAGAGCCACTCTGGGAAATCTTCTGGTGGAGCCAAG
GAGAAAAAGCACCCATGTGATCACTGTGACCGTCGGTTCTACACCCGGAA
GGACGTGAGGCGGCACATGGTAGTCCACACGGGCCGAAAGGACTTCCTGT
GTCAATACTGCGCCCAGCGCTTTGGCCGGAAGGATCACCTGACGCGACAC
GTGAAGAAGAGCCACTCGCAGGAGTTGCTGAAGATAAAGTCAGAGCCTCC
GGATATGCTGGGCCTCCTGGGCTCCGGCTCGCCGCCTTGTGCAGTTAAGG
AGGAGCTTAGCCCCATGATGTGCAGCGTTGGTCCCAACAAAGACCCCATG
ATGGGGAAACCCTTCCCCAGCGGACCCCTTTCCCTATGGGTATGTATAA
CCCGCACCAC------CTCCAGGCCATATCCAGCCCAGGGGTGGCCACC
CC------CACCCCTCCCTGATGCCTAGTTCCCTGTCAGCGGCTATGGGC
ATGGGCTGTCATATGGAATATCTCATTTACGCTTCCTTCTCCTTCATGGG
ATGTTTACAAATCACTGATGGGTCGAACATAGTGAATTTATTAGCTAGTA
ATTCTCTGAGCGTCTCATATGCTCTGACCCAGCAAAAGTATTTCAGTAAC
TATAGTCCCGTCATTGGGTTTTACATCTACGAGCCTATTGAGTACTGGAA
CTCAACAGTGCAGGAGCACCTGAGGACACTGAGCGATGGTTTCAACAAGA
TCTCTTGGATGGACCACTTCTTCCACTACCTGCAGGTGGTGAATGTGAGC
GCGTCAACCAAGAATGACTTCATCGGCATCCTGAAAGGCTCCTTCTTGAG
GAACCCGGAGTACCAGCACTTCATGGAGGACATCATCTTCTCCAAGA---
ACCGGGAGAGCG------ATGAGTACGACATCATCGCCTCACGCATGTAC
CTGGTGGCGCGCACCACGGAGAAGAGGCGCGAGGAGGTGGTTGAACTTCT
GGAGAAGTTGCGACCGCTGATGCTCATAAACAGCATCAAGTTCATTGCTT
TCAACCCCACCTTTGTCTTCATGGACCGCTACAGCTCCTCYGTCATCTCA
CCCATCCTCACCTCTGGCTTCAGCGTCCTCACCATCCTCATCCTCACTTT
CTTCCTGGTCATAAACCCGTTGGGGAACTTTTGGCTCATCCTGACGGTCA
CATCTGTGGAGCTGGGAGTTTTGGGTTTAATGGGTTTTCACCAGTTTGAG
TGGCAGCCTGCCCTCCAGAATGTGTCCTCATCTTGCCACGTGGGCATCAT
CAACGGACTCTCCGGCTGGGCCACCTCTGTAGATGACTCACCAGCCGACA
CGATCTCCCGGCGGTATCGCTACGATGTGGCCCTGGCATCTGCCCTTAAG
GACCTGGAGGAAGACATCGTGGAGGGACTGGTAGAACACGGACTGCAGGA
CAGCGTTCTGACCTCGGAGTTCAGCGTTGTGATCAAGGAGTCCTGCGACG
GCATGGGTGACGTTAGCGAGAAGCACGGAGGAGGGCCGGCGATTCCCGAG
AAGGCGGTCCGCTTCTCCTTCACCGTTATGTCAGTCTCTGTCCGGGTCGA
AGGAACGGAA------------------------------GAGGCGGTCA
CCGTCTTCAGGGAGCCGAAGCCCAACTCTGAATTGTCCTGTAAACCCCTC
TGTCTGATGTTTGTGGATGAGTCTGACCACGAGACCCTGACAGCCATTCT
GGGGCCTCTGGTGGCCGAGCGGAATGCGATGAAGCAGAGCCGCCTGATCC
TGTCATTGGGCGGCCTCCCGCGCTCTTTCTCCTTCGAGTTCCGAGGCACG
GGCTACGACGAGAAGATGGTGCGAGAGTTGGAGGGCATGGAGGCCTCTGG
TTCCACCTACGTCTGCACCCTGTGCGACTCCACCCGGGCGGAGGCCTCTC
GAAACATGGTGCTCCACTCCATCACCCGGAGCCACGGCGAGAACCTGGAG

CGGTACGAGATGTGGAGGAGCAACCCTTTCTCGGAGTCGGCCGAGGAGCT
GCGGGAGCGGGTCAAGGGCATCTCCGCCAAACCCTTCCTGGAGACCCAGC
CCACTTTGGATGCATTGCACTGCGACATTGGCAACGCCACCGAGTTTTAT
AAGATCTTCCAGGATGAAATCGGGGAGGTGTTCCGGGGGAC---CAAC--
-CCCAGCAGAGAAGAGCGGAGGAGCTGGAGGACRGCGCTGGACAAGCAAC
TGAGGAAGAGGATGAAGCTGAAGCCTGTGATGAGGATGAATGGTAACTAC
GCCCGCCGGCTGATGACCCATGACACTGTGGAGGTGGTGTGCGAGCTGGT
GCCCTCGGAGGAGCGGCGGGAGGCGCTGAGGGAGTTGATGGGCCTCTACC
TCGAGATGAAGCCGGTGTGGCGCGCCACGTGTCCAGCTGAGGAGTGCCCG
GACCAGCTGTGCCGCTATAGCTTCAACTCCAAGCGCTTCGCAGATGTCCT
TGCCACTGCCTTCAAGTACAGGTACGACGGGAAGATCACCAACTACCTGC
ACAAGACGCTGGCCCACGTCCCTGAGATCATTGAGAGGGACGGCTCCATC
GGAGCTTGGGCCAGCGAGGGAAACGAGTC---------------------

------------------AGTCACAGAGAGAGAGGTAGCTTTGGGGATAA
ATCCATTCGCAGACGGGATGGGCGCCTTTAAGATCAACCACACTTCTCAC
GACATCGGCTCCGG---GCAGACGGCGTTTTCTTCCCAGGCT---CCCGG
GTACGCTGCCGCGGCCGCCCTGGGGCACCACCACCA------CCACCCGA
CCCATGTCAGCTCG---TACTCCACGGCGGCTTTCAACTCCACTCGGGAC
TTTCTGTTCAGAAACCGGGGCTTCGGAGACGCCACCGG------------
CGCGCAGCACAGCTTGTTCGCCTC---------GGGAGGTTT---T----
--GCGGGGCCACATGGACACTCGGACGCCGCGGGACACCTGCTCTTCCCG
GGGCTCCACGAG---CAAGCCGCGGGCCACGCGTCGTCCAATGTGGTGAA
CAGCCAGATGCGCTTGGGTTTTTCAGGGGACGTGTACGGGAGGGCCGAAC
AGTACGGCCACGTTACGAGCCCGAGGTCAGCAGAGCACTATGCCTCGACC
CAGTTGCACGGCTACGGCCCCATGAACATGAACATGGCCGCG---CACCA
CGGAGCGGGGGCCTTCTTCCGATACATGCGCCAGCCGATCAAGCAAGAGC
TCATCTGCAAGTGGATCGAACCGGAGCAGCTGACCAACCCCAAAAAGTCG
TGCAACAAAACTTTCAGCACCATGCACGAGCTGGTCACCCACCTGACGGT
GGAGCATGTGGGGGGGCCGGAGCAGTCTAACCACATTTGCTTCTGGGAGG
ACTGCTCCCGGGAAGGGAAACCGTTCAAAGCCAAATACAAACTTGTGAAY
CACATCA---

>Aulopus filamentosus
AGTCTCCTGATTCGGGCAGAACTCAGTCAACCTGGGGCCCTCCTAGGGGA
CGACCAGATTTACAATGTAATTGTAACTGCACACGCTTTCGTTATAATTT
TCTTTATAGTAATACCAATCATGATTGGTGGTTTCGGAAATTGACTTATT
CCACTTATAATCGGGGCCCCCGACATAGCATTCCCCCGAATAAATAACAT
AAGTTTCTGACTTCTCCCCCCATCCTTCCTCCTCCTCCTTGCCTCATCCG
GCGTCGAAGCTGGGGCTGGTACCGGTTGGACTGTTTACCCCCCTCTCGCC
GGCAACCTGGCGCATGCCGGGGCCTCCGTTGATTTAACTATTTTTTCCCT
TCATTTGGCTGGTATTTCTTCAATTTTAGGTGCTATTAATTTTATTACAA
CAATTATTAATATGAAGCCCCCAGCTATCTCTCAATATCAAACACCACTT
TTTGTGTGGGCTGTTTTAATTACAGCTGTCCTCCTACTTCTTTCCCTTCC
TGTCCTTGCAGCTGGGATTACGATACTTCTAACAGACCGAAACCTCAATA
CAACCTTCTTCGACCCCGCAGGAGGAGGGGACCCAATTCTGTACCAACAC
TTATTCTGATTCTTTGGTCACCCCGAGGTATATATTTTGATTCTTCCAGG
CTTTGGGATAATTTCTCACATTGTTGCCTACTATGCCGGCAAAAAAGAAC
CATTCGGCTATATGGGCATGGTTTGAGCCATGATGGCAATTGGTCTTCTA
GGATTCATCGTATGGGCCCACCACATGTTTACAGTAGGAATGGACGTTGA
CACACGAGCTTATTCTTCGAGAGGAACCTGCACCCATCCAACTGCCTTGG
CATGCTGCTCTTGTCTGATGCTCACCAGTGCACCAAGCTGTCAGAGCTGT
CCTGGGGTATGTGTCTCAGCAATTTCCCTGCCATCTGCAAGACAGAGGAC
TTCCTCCAGTTGCCCAAAGACATGGTGGTGCAGCTTCTGTCCCATGAGGA
ACTGGAGACAGAAGATGAGAGACTGGTTTACGAGGCTGCCCTCAACTGGG
TCAACTATGACCTGGAGAGGAGACACTGCCACCTGCCAGAGCTGCTGAGA
ACTGTGCGCCTGGCCTTGCTTCCGGCCATCTTCCTCATGGAGAACGTCTC
CACAGAGGAGCTGATCAATGCCCAAGCCAAGAGCAAGGAGCTGGTGGACG
AGGCCATCCGCTGCAAACTGAAGATCCTGCAGAATGACGGAGTGGTCAAC

AGTCCCTGTGCCCGGCCCAGAAAGACCAGCCATGCCCTCTTCCTGCTGGG
AGGACAGACCTTCATGTGTGATAAGCTGTACTTGGTGGACCAGAAGGCCA
AAGAGATCATCCCCAAGGCTGACATCCCCAGCCCCAGGAAGGAGTTCAGT
GCCTGTGCCATCGGCTGTAAGGTCTACGTCACAGGTGGGA--GGGGCTC-
AGAGAACGGTGTCTCTAAAGACGTCTGGGTCTATGATACCGTCCATGAGG
AGTGGTCCAAGGCAGCACCCATGCTCATTGCTAGGTTTGGCCATGGCTCT
GCTGAGCTCAAACACTGCCTCTATGTGGTGGGAGGACACACAGCAGCCAC
TGGCTGCCTCCCAGCCTCTCCGTCTGGATGAGTACATTGTTGTGTTCAGT
CGTTCAACAACAAGGCTGATTCTGAACGAAGCGGAGCTGATACTGGCACT
GGCCCAAGAGTTTCAGATGAGGGTGGTTACAGTGTCCCTGGAGGAACAGT
CTTTCCCCAGCATCGTACAGGCCATCAGTGGGGCCTCCATGTTGGTTAGT
ATGCATGGAGCTCAGCTCGTCACCTCACTCTTCCTCCCGAGAGGAGCTGC
TGTAGTAGAGCTCTTCCCCTATGCTGTGAATCCGGAACAGTACACCCCAT
ACAAAACCCTAGCCTCCCTACCAGGCATGGACCTTCAATACGTTTCCTGG
AGGAACACTATTGAGGAGAACACTGTCGCCCACCCAGACAGACCCTGGGA
CCAAGGAGGCATTGCCCATTTGGAGAAAGAAGAGCAGGAGAGAATCCTAG
CCAGCAAGGATGTCCCTAGGCACCTATGCTGCCGCAACCCAGAGTGGCTC
TTCAGGATCTACCAGGACACTATGGTAGACATCCCCTCCTTGCTGGAAGT
CCTCAA---GGAGGGCCTGAAG---ACCAGGCCGAGCTTGAAGAA---GT
CCAAGCCGGCCAGCACGGTTCACCCGGGCCGGGTCCGAGAACCCCAGTGC
CAGACTTCAGTCCAAGCCACCAACGAGGCTAAACTCACAGTATCCTGGCA
GATCCCATGGAACCTTAAGTACCTGAAGGTGCGAGAGGTCAAGTACGAGG
TGTGGATCCGGAAGAGGGATGCAAGCAAGGGAACCCTGGAGGATCAAATC
ATTCAGGCAAACCCTGCACTGGAGGCTTTTGGTAATGCTAAAACACTGAG
GAATGACAACTCCTCTCGCTTTGGGAAATTCATCCGAATTCACTTTGGAA
CCAGTGGCAAGCTGTCCTCTGCAGACATTGAGACTTACCTGCTGGAGAAA
TCACGAGTCACCTTTCAGCTTAAGTCAGAGAGGAACTACCATATCTTCTT
CCAGATTTTGTCCAATCAAAAGCCAGAGCTGTTGGACATGTTGCTGATCA
CCAACAACCCCTACGACTACTCCTACATCTCCCAAGGAGAGGTAACTGTA
GCATCCATCAATGATTCGGAGGAGTTGATGGCCACGGACAGTGCATTTGA
TGTGCTTGGCTTCACTCAAGAGGAGAAGATGGGCGTCTACAAGCTGACAG
GGGCTATTATGCACTATGGCAACATGAAGTTCAAGCAAAAGCAACGTGAG
GAGCAGGCTGAGCCTGATGGTACAGAGGCTGCTGATAAGTCAGCTTACCT
AATGGGGCTGAATTCAGCTGACCTCATCAAAGGACTCTGCCATCCCAGAG
TCAAGGTAGGAAATGAATATGTCACCAAAGGCCAGGGTGTAGATCAAGTC
TACTACCCCAACAAGGAGGCCTTCAAGTGTGATGAGTGTGGGAAGCACTA
CAACACCAAGCTGGGATACAAGCGCCATGTGGCCATGCACTCCGCTACCG
CCGGGGATTTAACCTGCAAGGTGTGCATGCAGAGTTACGAGAGCACGCCG
GTTCTCTTGGAGCACCTCAAGAGCCACTCGGGGAAGTCTTCGGGTGGGGC
CAAAGAGAAAAAGCACCCTTGTGACCACTGCGACCGACGTTTCTACACTC
GGAAGGATGTGAGACGGCATATGGTGGTCCACACAGGCCGCAAGGACTTT
CTCTGCCAGTACTGTGCCCAGCGGTTTGGCAGGAAGGACCACCTGACACG
GCATGTGAAGAAGAGCCACTCGCAGGAGCTCCTAAAGATCAAGACAGAGC
CTCCAGATATGTTGGGCTTGCTAACGTCAGGGTCCCCGCCATGCCCTGTC
AAGGAAGAACTCAGCCCCATGATGTGTGGCATGGCACCCAACAAAGACCC
CATGATGGGTAAATCCTTCCCCAGCGGGGCACCATTTCCCATGAGCATGT
ACAACCCCCACCAT------CTCCAGGCCATGTCCAATTCTGGGGTGGCT
CACCCG------CACCCCTCCCTGATGCCCAGTTCCCTGTCTGCAGCTAT
GGGCATGGGCTGCCACATGGAA----------------------------


```
--------------------------------------------------
--------------------------------------------------
--------------------------------------------------
--------------------------------------------------
--------------------------------------------------
--------------------------------------------------
--------------------------------------------------
--------------------------------------------------
--------------------------------------------------
------------------------------------GGCTACCACCCGTT
TGAGTGGCAGCCAGCCCTAAAGAACGTGTCCCCATCCTGCCATGTGGGTA
TCATCAATGGGCTCTCCGGGTGGGCTGCCTCAGTGGACGATGCCCCTGCT
GACACCATCACCCGTCGGTTTCGCTACGACGTTGCCCTGGTGTCAGCCCT
GAAGGACCTAGAGGAGGACATCATGAAGGGACTGAAAGAGCGTGGGCTGG
AAGACTGTGCTTGCACCTCAGGCTTCAATGTTATGATCAAGGAATCCTGT
GATGGCATGGGAGATGTCAACGAGAAGCACGGTGGAGGACCAGCGATACC
TGAGAAGGCTGTGCGTTTCTCTATCACCGTCATGTCTGTCTCTGTCCAGG
CCGACGGAGAGGAG------------------------------GAGGCG
GTTGTCATCTTCAGGGAGTCAAAGCCCAACTCTGAACTGTCCTGTAAGCC
CCTGTGCCTAATGTTTGTGGATGAGTCTGACCACGAGACACTCACCGCTG
TCCTGGGGCCTTTGGTAGCAGAGAGGAATGCAATGAAGCAAAGCCGACTC
ATCCTTTCAGTGGGTGGCCTCCCCCGCTCTTTCCGCTTCCATTTCAGAGG
CACAGGCTATGATGAGAAGATGGTGCGAGAGATGGAGGGCCTGGAGGCCT
CAGGCTCCACCTATATCTGCACTCTTTGTGACTCCACTCGGGCAGAAGCC
GCTCACAACATGGTGCTCCACTCTGTCACCCGCAGCCACGATGAGAACCT
GGAGCGCTACGAGATATGGAGGACCAACCCCTTCTCCGAGTCTGTCGTGG
AGCTGCGAGACCGGGTCAAAGGAGTCTCTGCCAAACCATTTATGGAGACA
CACCCAACTCTGGATGCATTACACTGTGACATAGGTAACGCCACTGAATT
CTACAAAATCTTCCAGGATGAGATTGGGGAGGTGTTCCGGAAGGC---CA
AC---CCTAGCCGGGAGGAACGGCGCAGCTGGAGGGCAGCCCTCGATAAG
CAGCTGAGGAAGAAATTGAAGCTGAAACCAGTGATGCGGATGAATGGGAA
CTATGCCCGAAGGTTGATGACCCAGGAGTCTGTGGAGGTGGTGTGTGAAC
TGGTGCCCTCAGAAGAGAGGCAGGAGGCCCTGAGGGAGCTGATGGGGCTC
TATATCCAGATGAAGCCTGTGTGGCGTGCTACCTGCCCAGCCAAGGAGTG
CCCTGACCAGCTGTGCCGCTACAGCTTTAACTCTCAGCGCTTTGCTGATC
TCCTCTCCTCCGCCTTTAAATACAGGTATGATGGAAAGATCACCAACTAC
CTGCACAAGACCTTGGCCCATGTCCCTGAAATTATAGAGAGAGATGGCTC
CATTGGAGCCTGGGCCAGCGAGGGGAATGAGTTTGCGAACAAG-------
--------------------------------------------------
--------------------------------------------------
--------------------------------------------------
--------------------------------------------------
--------------------------------------------------
--------------------------------------------------
--------------------------------------------------
--------------------------------------------------
--------------------------------------------------
--------------------------------------------------
--------------------------------------------------
--------------------------------------------------
--------------------------------------------------
--------------------------------------------------
--------------------------------------------------
```


NNNNNNNNNNNNNNNNNNNNNNNNNNNNNNNNNNTGGCTTTGGGGATAAATCCGTTCGCAGACGGGATGG
GCGCCTTCA
AAATAAACCACAGCTCTCATGACATTGGCTCCGG---GCAAACGGCGTTT
TCCTCCCAGGCG---CCCGGCTAT---GCAGCGGCTGCCCTGGGA---CA
CCACCA------CCACCCGACCCACGTTAGCTCT---TACTCCACGGCAG
CTTTCAATTCCACTCGGGACTTTCTCTTCAGAAATCGGGGCTTCGGAGAC
GCCACCAG------------CGCACAGCACAGTCTGTTCGCCTCCGC---
CGCGGGAAGTTT---T------GCTGGGCCACATGGACACTCAGATGCCG
CGGGACACCTGCTCTTTCCGGGGCTTCACGAG---CAAGCCGCGAGCCAC
GCGTCATCTAACGTTGTAAACAGCCAGATGCGGTTGGGCTTTTCGGGGGA
CATGTACGGGCGGGCCGAGCAGTATGGCCACGTTACAAGCCCGCGGT---
CCGACCACTATGCTTCGACCCAGTTGCACGGCTATGGCCCTATGAATATG
AATATGACCGCA---CACCACGGAGCCGGGGCCTTCTTTCGGTACATGAG
GCAGCCGATCAAACAAGAGCTCATCTGCAAGTGGATCGAACCGGAGCAAC
TGACGAATCCCAAAAAGTCGTGCAACAAAACTTTCAGCACGATGCACGAG
CTCGTGACCCATCTGACGGTGGAGCATGTGGGGGGACCGGAGCAGTCGAA
CCACATTTGCTTCTGGGAGGACTGTGCCCGAGAAGGGAAACCGTTCAAAG
CCAAATACAAACTTGTGAATCACATCAGAGTTCACACCGGCGAGANNNNNNNNNNNNNNNNNNNNNNNNN
NN-----

>Aulostomus maculatus
AGTCTTATCATCCGGGCCGAGCTTAGCCAACCGGGGGGTCTCCTGGGTAA
CGATCAACTTTACAATGTTGTTGTAACAGCCCACGCCTTCGTTATAATCT
TCTTTATAGTAATACCAATTATAATTGGAGGCTTCGGAAATTGATTAATT
CCCTTAATGATCGGAGCCCCCGACATGGCCTTCCCCCGAATAAATAACAT
GAGCTTCTGGCTCCTGCCCCCCTCCTTCCTCCTCCTCTTAACCTCCTCTG
CGGTAGAGGCCGGGTCCGGGACCGGATGGACGGTTTACCCGCCCCTGGCT
GGGAACCTGGCCCACGCCGGAGCGTCCGTAGACCTGACCATCTTCTCGCT
TCACCTCGCCGGAATTTCCTCCATCCTAGGAGCAATTAACTTTATTACAA
CCATTATTAATATAAAACCCCCTGCCACCTCCCCATACCAGCTCCCCCTG
TTCGTATGAGCTGTCCTGGTTACTGCTGTGCTTCTCCTCCTTTCTCTCCC
AGTCCTAGCAGCTGGCATTACAATGCTGTTAACTGACCGAAACCTGAACA
CCACCTTCTTCGACCCTGCAGGGGGAGGCGATCCTATTCTATATCAGCAC

------------TTCCTGGAGAGAAACCTCCACCCTTCTAACTGCCTTGG
CATGTTGTTGCTCTCTGATGCCCACCAGTGTACCAAGTTGTCAGAGCTCT
CCTGGGGCATGTGCCTCAGCAACTTCCCCGCTATTTGCAAGACGGAGGAC
TTCCTTCAACTACCCAAAGATATGGTGGTGCAGCTGTTGTCACACGAAGA
GCTAGAAACCGAAGATGAAAGGTTAGTTTATGAGGCGGCCCTGAACTGGA
TCAACTACGACCTAGATAGGAGGCACTGTCACCTCCCGGAGCTCCTCAGA
ACCGTGCGTCTAGCACTTCTGCCTGCCATCTTTCTAATGGAGAATGTCTC
GACTGAAGAGCTGATCAACGCCCAGGCCAAGAGCAAGGAGCTGGTGGATG
AGGCTATCCGGTGCAAGCTGAAGATCCTTCAAAATGACGGTGTCGTGAAC
AGCCAGTGTGCTCGACCAAGAAAAACCAGCCATGCCCTCTTTCTTCTGGG
AGGACAGACTTTTATGTGTGACAAGTTGTACCTGGTGGATCAGAAGGCCA
AAGAGATCATACCAAAGGCTGACATTCCCAGCCCCAGGAAGGAATTCAGC
GCCTGTGCCATCGGTTGTAAGGTCTACATCACAGGAGGAA--GAGGCTC-
AGAGAATGGTGTGTCCAAAGATGTATGGGTCTATGACACCGTCCATGAAG
AATGGTCCAAAGCAGCACCCATGCTCATTGCCAGATTTGGTCATGGCTCT
GCGGAGCTGAAACACTGCCTGTACGTGGTAGGAGGTCACACCGCAGCGAC
TGGCTGCCTCCCAGCTTCTCCATCTGGACGAATACATCGTTGTGGTCAGC
CGATCAACAACAAGGCTGATACTGAATGAAGCCGAGCTCATCATGGCGCT
GGCCCAGGAGTTCAGGATGAGGGTGGTCACGGTTTCCCTGGAGGAACAGT
CTTTCCCCAGCATCGCTCAGGTGGTCAGTGGTGCTTCCATGTTAGTCAGC
ATGCATGGAGCTCAACTCATCACCTCGCTCTTCCTCCCCAGAGGGGCTGC
TGTGGTGGAATTGTTCCCATTTGCTGTGAACCCAGAACAGTATACTCCAT
ACAAAACCTTGGTCACCCTTCAAGGCATGGACCTTCACTATGTGTCCTGG
AGAAACACAAAGGAGGAGAACACTGTTACCCACCCAGACAGACCGTGGGA
ACAAGGAGGCATTGTTCACCTGGAGAAGGAGGAGCAGGAGCGAATACTGG
CAAGCAAAGATGTCCCCAGACACCTGTGCTGCCGCAACCCGGAGTGGCTC
TTCAGAATCTACCAGGACACACTGGTAGATATCCCTTCCTTCCTGGAAGT
TCTGAG---AGAAGGCATGAAG---ACCAAGCCCAGCTTGAAGAA---GG
CCAAGCCAGCCAGCGTGGTCCATCCAGGCCGGGTCAGAGAACCTCAGTGT
CAGACCTCAGTACAAACCACCAATGAGGCCAAACTCTCAGTTTCCTGGCA
GATCCCATGGAATCTGAAATACCTGAAAGTAAGAGAGGTGAAATATGAGG
TGTGGATCCAGAAAAAGGACACCAGTAAAGGGACTCTGGAGGATCAGATT
ATCCAGGCGAATCCTGCGCTGGAGGCCTTTGGGAATGCCAAAACCTTGAG
AAATGACAACTCCTCTCGTTTTGGAAAATTTATCCGAATTCATTTTGGAA
CAAGTGGGAAGCTGTCATCTGCTGACATCGAGACGTACCTGCTGGAAAAG

TCTCGTGTCACCTTTCAGCTCAAGGCTGAAAGGAACTACCACATCTTCTA
TCAGATCCTGTCCAATCAGAAGCCAGAGCTGCTGGACATGCTGCTAATCA
CGAACAACCCGTACNACTACTCCTATATCTCGCAAGGAGAGGTGACAGTAG
CTTCCATTAATGACTCAAAGGAGCTGATGGCCACAGACAGTGCCTTCGAT
GTGCTCGGCTTTACTTCAAAGGAGAAAATGGGTGTCTACAAACTGACCGG
CGCCATAATGCACTATGGTAACATGAAGTTCAAGCAGAAGCAGCGTGAGG
AGCAGGCTGAGCCTGACGGGACAGAGGCTGCTGATAAGTCAGCTTATTTG
ATGGGGCTGAACTCTGCTGACCTCATCAAAGGGCTGTGTCATCCCAAAGT
TAAGGTAGGAAACGAGTACGTCACCAAAGGCCAGAGCGTGGACCAAGTCT
ACTACCCAAACAAGGAGGCCTTCAAGTGTGAGGAGTGCGGGAAGCACTAC
AACACCAAGCTGGGATATAAACGCCATGTGGCCATGCACTCTGCCACAGC
AGGAGATCTCACCTGTAAAGTGTGCATGCAGAGCTATGAGAGCACACCAG
TTCTCCTGGAGCACCTCAAGAGCCACTCTGGGAAGTCCTCAGGCGGAGCT
AAGGAGAAAAAACACCCATGTGACCACTGTGATCGCCGTTTCTATACACG
GAAGGATGTGAGGCGGCACATGGTGGTCCACACAGGCCGTAAAGACTTCC
TGTGCCAGTACTGTGCCCAGCGCTTTGGCAGGAAGGACCACCTAACTCGC
CATGTGAAGAAGAGCCACTCACAGGAGCTGCTGAAGATCAAGACGGAGCC
TCCGGATATGTTGGGTCTGTTAGCTTCTGGGTCACCTCCTTGCTCTGTGA
AGGAGGAGCTCAGTCCCATGATGTGTGGCATGGGTCCCAACAAAGACCCC
ATGATGGGCAAACCGTTCCCCAGTGGGGCCCCTTTTCCAATGGGCATGTA
CAACCCCCACCAT------CTTCAGGCCATGTCTAATTCTGGGGTGGGTC
ATCCA------CACCCGTCCCTGATGCCCAGTTCTCTGTCTGCAGCTATG
GGCATGGGTTGTCACATGGAANNNNNNNNNNNNNNNNNCTTCTCATTCATGGGATGTTTACAAATCA
GTGATGGTTCAAACATTGTAAACCTTCTGGCTAGTAACTCTCCAAGTGTT
TCATATGCTCTGACTCAGCAGAAATACTTCAGCAACTACAGCCCAGTAAT
TGGTTTTTACATTTATGAGCCTATTGAGTACTGGAACTCCACAGTGCAGG
AGCACCTCAAGACTCTGAGTCATGGTTTCAATAAGATCTCCTGGATGGAC
AACTTTTTCCACTACCTGCGGGTGGTGAATGTCAGTGCATCCACCAAGGG
TGACTTCATCACCATCCTCAAGGGCTCTTTCCTGCGGAGTCCTGAGTACC
AGCACTTCACTGAGGACATCATATTCTCCAAGA---ACCGCGAGACAG--
----ACGAATATGACATTATCGCCTCACGCATGTATTTAGTGGCACGGAC
CACAGAGAAGAAGCGTGAGGAGGTGGTGGAGCTTCTAGAGAAGCTTCGCC
CTTTGATGCTGATCAACAGCATCAAGTTCATTGCCTTCAATCCCACATTT
GTGTTCATGGACCGCTACAGCTCCTCTGTCATATCGCCCATACTGACCTC
AGGCTTCAGTGTACTCACCATCCTTATACTCACTTTCTTCCTGGTCATCA
ACCCCTTGGGGAATTTCTGGCTCATCCTCACAGTAACATCCGTGGAGCTG
GGTGTTCTGGGTTTGATGGGTTTCACCAGTTTGAATGGCAGCCAGCTCT
CAAGAATGTGTCAGCATCTTTCAATGTTGGCATTATTAATGGCCTCTCTG
GATGGGATTCGTCCGTGGATGACTCACCACCTGACACCATTACTCGGCGA
TTTCGCTATGATGTGGCTCTAGTATCAGCATTAAAGGATCTGGAAGAGGA
CATAATGGAAGGGCTCAGAGAGAGGGGGATGGAAGACAGTGCTTGCACCT
CAGGCTTCAGTGTTATGATCAAAGAGTCCTGTGATGGCATGGGCGATGTC
AGCGAGAAGCATGGTGGAGGACCAGTTGTTCCTGAGAAAGCTGTTCGTTT
CTCTTTCACAATTATGTCTATCTCTATCCTGGGTGATGATGATGAA----
--------------------------GATAAGGTTACTATATTCACTGAG
CCAAAGCCAAACTCAGAACTGTCCTGTAAGCCCCTTTGTCTGATGTTTGT
GGATGAATCTGATCATGAGACCCTCACAGCTGTACTGGGGCCTATTTTTA
CGGAGCGTAATGCAATGAAGGAAAGTAGACTGATCCTCTCCATTGGTGGT
CTGCCTCGCTCTTTCCGTTTCCACTTTAGAGGCACGGGATATGATGAGAA
GATGGTGCGAGAGATGGAGGGCCTGGAGGCCTCAGGCTCCACCTATATCT
GCACTCTGTGTGACTCAGGTCGGATGGAGGCCTCTAAAAACATGGTACTA
CATGCCATCACACGCAGCCACGATGAAAACCTAGAACGTTATGAAGTATG
GGAGGACCACCCCTTTTTTGAGTCTGTAGATGAGCTACGGACAGAGTCA

AAGGGGTCTCTGCCAAGCCATTTGCTGAGACCCATCCCACACTGGATGCA
TTACATTGTGACATCGGCAATGCCACTGAATTCTACAAAATCTTTCAGGA
TGAAATTGGGGAGGTGTACCAGAAGGT---CAAT---CCCTGCCGGGAGG
AGCGACGCAGCTGGAGGGCAGCTCTAGATAAGGAGCTGAGGAGGAAGATG
AAGCTTAAACCAGTAATGAGAATGAATGGCAACTATGCGCGGAAGCTAAT
GACCCTTGAGGCTGTGGAGGTTGTGTGTGAGCTGGTGCCATCTGAGGAGA
GGAGGGAGGCCTTGAGGGAGCTTATGAGGCTCTACCTTGAGATGAAGCCT
GTATGGCGTGCCACCTGCCCAGCAAAGGAGTGCCCTGACCAGTTGCACCG
CTACAGCTTCAACTCCCACCGTTTTTCTGACCTCCTCTCCTCCACCTTCA
AATACAGGTACAATGGCAAGATAACCAATTACCTGCACAAGACTTTGGCC
CATGTCCCTGAGATCATTGAGAGAGACGGATCTATTGGAGCCTGGGCTAG
TGAGGGGAAGGAGTCAGCAAACAAANNNNNNNNNNNNNNNNNNNNNNCTCTCGGGCCCCAGTGGAAGGAG
AG
CCCACAGCCTTTCTCCTGCTCCATTGAAGATCCCACAAAACAGACAAAAT
TCAAAGGCATCAAGACATACATTTCATACCGGGTCACGCCGTCTCACACG
GGCCGTCCCGTGTACAGACGCTACAAACACTTCGACTGGCTGTACAACCG
GCTGCTGCACAAGTTCACTGTGATCTCCGTGCCGCACCTGCCTGAGAAGC
AGGCCACGGGGCGGTTCGAGGAAGACTTTATCGAGAAGCGTAAGAGACGA
CTGATACTGTGGATGAATCACATGACCAGTCACCCAGTCCTCTCCCAGTA
TGAAGGCTTTGAGCACTTTCTGATGTGTGCTGACGACAAGCAGTGGAAGC
TGGGCAAGAGACGGGCGGAGAANGANGAGATGGTGGGTGCCCACTTCATGCT
GACCCTCCAGATCCCCAACGAGCACCAGGACCTTCAGGATGTAGAGGAGA
GGGTGGACTCCTTTAAGGCCTTTGCCAAGAAAATGGACGACAGTGTCATG
CAGCTCACACATGTTGCCTCTGAGCTGGTGCGTAAACACCTGGGTGGGTT
CAGGAAGGAATTCCAGCGACTGGGAAATGCCTTCCAGTCCATCAGCCAGT
CATTCATGCTGGACCCCCCCCACAGTTCAGAGACCCTCAACAACGCCATC
TCCCATNNNNNNNNNNNNNNNNNNNNNACTGACCTCTCTGGGTTTATTATTGGTGTTGGTGTGGTTGGGA
ACCTCCTGATTTCTATCCTGCTGGTCAAAGACAAGAGCCTGCACCGAGCA
CCCTACTATTTCCTGCTGGACCTGTGCGCCTCTGATATCCTGCGCTCAGC
CATCTGCTTCCCCTTTGTCTTCACCTCAGTCAAGAATGGCTCCGCCTGGA
CATACGGCACGTTAACCTGCAAAGTGATCGCCTTCCTGGGTGTGCTGTCC
TGTTTCCACACTGCATTCATGCTATTCTGTGTTAGTGTCACACGCTACTT
GGCCATTGCACATCACCGCTTCTATACTAAGAGGTTGACCTTCTGGACCT
GTCTGGCTGTCATCTGCATGGTGTGGACATTGTCAGTCGCTATGGCATTC
CCACCAGTGCTAGACGTAGGGACGTATTCTTTTATCCAAGAGGAGGATCA
GTGCACATTCCAGCACCGCTCCTTCAGGGCAAATGATTCACTGGGCTTCA
TGCTCCTGCTGGCTCTCATTCTCCTGGCCACACAGCTGGTTTACCTCAAG
CTCATCTTCTTCGTCCACGACCGCCGAAAGATGAAGCCCGTCCAGTTTGT
GCCTGCTGTCAGCCAGAACTGGACTTTCCATGGTCCAGGTGCCAGCGGGC
AGGCAGCAGCCAACTGGCTAGCCGGATTTGGTCGAGGCCCCACCCCACCT
ACTTTGCTGGGAATCCGACAGAACAGCAATGCAGCGGGCCGCCGTCGCCT
ACTGGTATTGGATGAGTTCAAAACAGAGAAGAGGATTAGTAGGATGTTCT
ACATCATGACGTTTTTCTTCCTGGCTCTGTGGGGGCCCTATCTGGTAGCC
TGCTACTGGCGGGTGTTTGCAAGGGGCCCCGTGGTTCCTGCAGGCTACCT
CACAGCAGCCGTGTGGATGAGTTTTGCCCAGGCTGGGGTCAATCCTTTCA
TCTGCATNNNNNNNNNNNNNNNNNNNNNNNNNNNNNNNNNNNNNGGCGTGGGGACTGGTCCTGGCACAGA
GC---GCAGCGTACCAC
TCGGCAACAGCTTGCTATCCCCGCAGCAAACCGAGGAGCCCACTGTTGCC
ACCCCCCCGCAGCGATGGTTTGTCACCC---CTGCCAACAACCGACTGGA
CTTTGCAGCCTCGGCATACGAGGCCGCT---------GATTTCGCCGGTA
ACGCGGCCACCTTGCTGTCCTACGCAGCGGCCGGAGTGAAAGCTC-----
-TTCCCCTGCCGACTGCAGGCTGCTCCAACCGGCCTCTTGGCTATTACGC
AGATCCGTCAG---GTTGG---GGAGGACGCACGCCGCCGCAGTACTGTG

GCGTCAATAGCAAATCAAGCTCGGTGTTTTCCTGCTGGCCCGCAAACTCT
ATCGGCGGCAGAGCAGGCA---CC---AACTGCCTGG---------CGGA
GGA---GGGA---GACTC---CATCCCGACAGAGAGGTCACCG---AT--
-C---GGATCAGAGGAG---ACCAAATCTAAAGACATGAC---GTCAGA-
--GTCGAGCTGGATAGAG---ACGCCGTCCTCCATTAAGTCCATCGATTC
AAGCGACTCTGGGATCTTTG---AACAGGCCAAACGGAGGAGAATCTCCC
CGTCTGCAACGCCG---------GTACCAGAGACAGTGTCCCCGTTAAAA
TCCGAGATCACTCAACAGGGCGAAGTCACAGAGAGAGAAGCGGCGTTGGG
GATAAATCCGTTTGCAGATGGGATGGGCGCCTTCAAAATAAACCACAGCT
CCCACGATATTGGCTCCGG---ACAAACGGCGTTTTCCTCCCAGGCG---
CCCGGCTAC---GCAGCAGCCGCCCTGGGGCACCACCACCA------CCA
CCCGACCCACGTTGGCTCT---TACTCCACGGCGGCTTTTAACTCCACGA
GGGACTTTCTCTTCAGAAATCGGGGTTTCGGGGACGCCACCGG-------
-----GGCGCAGCACAGTTTGTTCGCCTC---------CGGAAGTTT---
C------GCAGGGCCACATGGACACTCAGATGCAGCGGGGCACCTGCTCT
TCCCGGGGCTCCACGAG---CAGGCGGCGAGCCATGCGTCTTCCAACGTG
GTCAACAGCCAGATGCGACTGGGCTTCTCGGGGGACATGTACGGAAGGGC
CGACCAGTACGGCCACGTTACGAGCCCACGCT---CCGA---TTATGCCT
CGACCCAGTTGCATGGCTACGGCCCCATGAACATGAATATGGCCGCG---
CACCACGGAGCAGGGGCCTTCTTTCGATACATGAGGCAACCCATCAAACA
AGAGCTTATCTGCAAGTGGGTCGAACCGGAGCAGCTGACGAATCCCAAAA
AGTCATGCAACAAAACTTTTAGTACAATGCACGAGCTGGTGACCCATCTG
ACGGTGGAGCATGTGGGGGGACCGGAGCAGACCAACCACATCTGCTTCTG
GGAGGACTGCTCCAGAGAAGGGAAACCCTTCCAAGCCAAATACAAACTTG
TGAATCATATCCGAGTTCACACCGGAGAAAAGCCCTTTCCGTGCCCGTTC
CCCGGCTGTGGCAAA
>Aulotrachichthys prosthemius
AGCCTACTCATCCGAGCAGAGCTAAGCCAACCTGGGGCCCTCCTAGGTGA
TGATCAAATTTATAACGTTATTGTTACAGCGCATGCCTTTGTAATAATTT
TCTTTATAGTAATACCAATCATAATTGGTGGCTTCGGAAACTGACTTATT
CCCCTAATGATCGGAGCCCCTGATATAGCATTTCCTCGAATAAATAATAT
AAGCTTCTGACTTCTCCCACCATCATTCCTCCTCCTCCTCGCTTCTTCCG
GAGTTGAAGCAGGGGCCGGGACAGGATGAACAGTATACCCGCCCCTTGCA
GGAAACCTAGCTCACGCAGGAGCTTCCGTAGATCTAACCATTTTCTCCCT
ACATTTAGCAGGTGTTTCCTCCATCCTGGGGGCCATCAATTTTATTACAA
CCATTATTAATATGAAACCCCCTGCTATTTCTCAGTACCAAACCCCCTTA
TTTGTCTGAGCCGTTTTAATTACAGCAGTTCTTCTTTTACTCTCCCTTCC
CGTTCTCGCAGCCGGCATTACTATGCTCCTCACAGATCGAAACTTAAATA
CTACCTTCTTTGACCCAGCAGGAGGAGGAGACCCAATTCTTTATCAACAC
TTATTC--

------------NNNNNNNNNNNGAACCTTCACCCATCTAACTGCCTGGGCATGCTGTTGC
TGTCCGATGCACACCAGTGTACCAAGCTATCAGAGCTCTCCTGGGGCATG
TGCCTCAGCAACTTTCCGGCCATTTGCAAGACAGAGGACTTCCTCCAGCT
GCCCAAAGACATGGTGGTGCAGCTTCTATCCCACGAGGAGCTGGAGACAG
AAGATGAGAGACTGGTTTATGAAGCTGCCCTTAACTGGGTCAATTATGAC
CTGGAAAGGAGGCACTGCCATCTGCCAGAGCTGCTGAGAACAGTTCGCCT
GGCCCTGCTACCTGCCATCTTCCTCATGGAGAATGTCTCCACAGAAGAGC
TGATCAATGCCCAGGCCAAGAGCAAGGAGCTGGTAGATGAGGCCATCCGC
TGCAAGCTAAAGATCTTGCAGAATGACGGCGTGGTTAACAGCCCCTGCGC
TCGGCCGAGAAAAACCAGCCACGCCCTTTTTCTGTTGGGAGGGCAGACCT

TCATGTGTGACAAGCTGTATCTGGTGGACCAGAAAGCGAAAGAGATCATC
CCCAAGGCGGACATCCCCAGCCCTAGGAAGGAGTTCAGTGCCTGCGCCAT
CGGCTGTAAGGTCTACATCACAGGCGGGA--GAGGCTC-AGAAAACGGCG
TGTCTAAAGACGTATGGGTCTATGACACCGTCCACGAGGAATGGTCCAAG
GCAGCACCCATGCTCATTGCCAGATTTGGTCACGGCTCTGCTGAGCTGAA
ACACTGCCTTTACGTNNNNNNNNNNNNNNNNNNNNNNNNNNNNNNNNNNNNNNNNNNNNNNNNNNGGATGA
CTACGTTGTAGTGTTCAGCCGTTCAACAA
CAAGGCTGATTCTGAACGAAGCGGAGCTCATCATGACGTTGGCCCAGGAA
TTTCAGATGAGAGTGGTTACAGTCTCCCTAGAGGAGCAAACCTTCCCCAG
CATCGCCCAGGTGATCAGTGGTGCCTCCATGTTAGTCAGCATGCATGGAG
CTCAGCTTGTCACCTCACTCTTCCTGCCCAGAGGAGCTGCTGTGGTGGAG
CTGTTCCCCTATGCTGTCAACCCAGAACAGTACACCCCATATAAAACCCT
CGCCTCCTTACCAGGCATGGATCTTCAATATGTTTCCTGGAGGAACACTA
TAGAGGATAACACTGTTACCCACCCAGACAGACCCTGGGAAGAAGGAGGC
ATCGCCCATTTGGAAAAGGACGAGCAGGAGCGAATACTGGCCAGCAAGGA
TGTCCCCAGACACCTGTGCTGCCGCAACCCAGAGTGGCTCTTCCGAATCT
ATCAGGACACTTTGGTCGACATCCCTTCATTCGTGGAAGTCCTCAA---A
GAGGGCCTGAAG---ACCAGGCCCAGCTTGAAGAA---GGCCAAGCTGGC
CAGCACGGTACATCCAGGCCGGGTCAGAGAACCCCAGTGCCACACCTCGG
TCCAAGCCACCAACGAGGCTAAACTCACGGTTTCCTGGCAGATCCCATGG
AATCTGAAATACCTGAAGGTGCGAGAAGTGAAGTACGAGGTGTGGATCCG
GAAGAAGGATACCAGCAAGGGGACACTGGAGGATCAAATCATCCAGGCAA
ACCCAGCGCTGGAGGCTTTTGGTAATGCCAAAACAGTGAGGAATGACAAC
TCATCTCGTTTTGGAAAATTCATCCGAATTCACTTTGGAACCAGTGGCAA
GCTGTCCTCTGCTGATGTTGAAACCTACCTATTGGAGAAGTCACGTGTCA
CCTTTCAACTCAAGGCTGAAAGGAACTACCACATCTTCTTCCAGATATTG
TCCAATCAGAAGCCGGAACTGTTGGACATGCTGTTGATCACCAACAATCC
ATATGACTACTCCTACATCTCCCAAGGAGAGGTAACAGTAGCATCCATCA
ACGACTCAGAGGAGCTGATGGCCACTGACAGCGCCTTTGACGTGCTTGGC
TTTACTCTTGACGAGAAGATGGGAGTATATAAACTGATTGGTGCCATTAT
GCACTATGGCAACATGAGATTTAAGCAGAAGCAGCGTGAAGAGCAGGCTG
AGCCTGACGGAACAGAGGCTGCTGATAAGTCAGCTTACCTTATGGGGCTG
AACTCTGCAGACCTCATCAAAGGGCTATGCCATCCCAGAGTCAAGGTAGG
AAATGAATATGTCACCAAAGGCCAAGGTGTAGACCAAGTCTACTACCCCA
ACAAGGAGGCCTTCAAGTGCGAGGAGTGTGGCAAGCACTACAACACCAAG
CTGGGATACAAGCGCCATGTGGCCATGCACTCTGCCACGGCGGGGGATCT
CACCTGCAAAGTGTGCATGCAGACCTATGAGAGCACGCCGGTGCTCCTGG
AGCACCTTAAGAGTCACTCGGGGAAGTCCTCGGGTGGCACCAAGGAGAAG
AAACACCCGTGCGACCACTGTGACCGCCGCTTCTACACGCGCAAGGATGT
GAGACGGCACATGGTGGTCCACACGGGCCGGAAGGACTTCCTGTGCCAGT
ACTGTGCCCAGCGCTTCGGCAGGAAGGACCACCTGACGCGCCACGTGAAG
AAGAGCCACTCGCAGGAGCTGCTGAAGATCAAGACAGAGCCTCCAGATAT
GTTAGGTCTTTTAGCTTCTGGCTCACCACCCTGCTCTGTGAAGGAGGAGC
TCAGCCCCATGATGTGCGGCATGGGTCCCAACAAAGACCCCATGATGGGC
AAACCCTTCCCCAGCGGACCCCTTTCCCCATGGGCATGTACAACCCCCA
CCAT------CTCCAGGCCATGTCTAATTCTGGGGTGGGTCACCCA----
--CACCCATCCCTGATGCCCAGTTCCTTGTCTGCAGCTATGGGCATGGCC
TGTCACATGGAG--------------------------------------

NNNNNNNNNNNNNNNNNNNNNNNNNNNNNNNNNNNNNNNNNNNNNNNNNNNNNNNNNNNNNNNNNNNNNN
NNNNNNNNNNNNNNNNNNNNNNNNNNNNNNNNNNNNNNNNNNNNNNNNNNNNNNNNNNNNNNNNNNNNNN
NNNNNNNNNNNNNNNNNNNNNNNNNNNNNNNNNNNNNNNNNNNNNNNNNNNNNNNNNNNNNNNNNNNNNN
NNNNNNNNNNNNNNNNNNNNNNNNNNNNNNNNNNNNNNNNNNNNNNNNNNNNNNNNNNNNNNNNNNNNNN
NNNNNNNNNNNNNNNNNNNNNNNNNNNNNNNNNNNNNNNNNNNNNNNNNNNNNNNNNNNNNNNNNNNNNN
NNNNNNNNNNNNNNNNNNNNNNNNNNNN------------------------
------NNNNNNNNN---
NNNNNNNNNNNNNNNNNNNNNNNNNNNNNNNNNNNNNNNNNNNNNNNNNNNNNNNNNNNNNNNNNNNNNN
NNNNNNNNNNNNNNNNNNNNNNNNNNNNNNNNNNNNNNNNNNNNNNNNNNNNNNNNNNNNNNNNNNNNNN
NNNNNNNNNNNNNNNNNNNNNNNNNNNNNNNNNNNNNNNNNNNNNNNNNNNNNNNNNNNNNNNNNNNNNN
NNNNNNNNNNNNNNNNNNNNNNNNCTGGAGGCCTCAGGTTCCACGTACATCTGCACGCTTTGCGA
TTCCAGCCGTGCAGAAGCTTCTGAAAACATGGTGCTGCATTCCGTCACCC
GCAGTCATGACGAGAACTTAGAGCGTTATGAAATATGGCGAACCAACCCC
TTTTCTGAGTCTGTGGATGAGCTGCGNGACAGAGTCAAAGGGGTCTCTGCC
AAGGCATTTATGGAGACCCATCCCACTCTGGATGCGCTGCACTGTGAGAT
AGGAATTGCCACTGAATTTTACAAAATCTTCCAGGATGAGATTGGGGAGG
TGTACCAAAGGCT---AAAT---CCTAGTCGGGAGGAGCGGCGCGGGTGG
AGGGCAGCCCTGGACAAACAGCTGAGGGAGTCCATGAAACTTAAACCAGT
AATGAGGATGAATGGGAACTACGCACGCCGGCTCATGAGCCTGGAGGCTG
TGGAGTTGGTGTGCCAGTTAGTGCCCACAGAAGGTAGGAGGGAAGCCCTG
AGGGAGCTCATGAGGCTCTACCTCCAGATGAAGCCTGTATGGCGTGCCAC
CTGCCCCGCCAAGGAGTGCCCCGACCAGCTGTGTCGCTATAGCTTCAACT
CTCAGCGCTTTGCAGAGCTCCTCTCCTCCTCATTCAAATATAGGTACAAT
AAGAAAATCACCAATTACCTCCACAAAACCCTCGCCCACGTACCCGAAAT
CATAGAGAGAGATGGATCTATAGGAGCATGGGCCAGCGAAGNNNNNNNNNNNNNNNNNNNN---------
----

--NNNNNNNNNNNNTTCC
TCAAACTGACCTCTCTGGGTTTCATCATTGGAGTCGGTGTGGTTGGGAAC
CTCCTGATCTCCATCCTGCTGGTCAAAGACAAGAGCCTGCACCGAGCGCC
CTACTACTTCCTGCTCGACCTGTGCGCCTCCGACATCCTGCGCTCTGCCA
TCTGCTTCCCCTTTGTCTTCACCTCTGTCAAGAATGGATCCGCCTGGACC
TATGGCACGCTCACCTGCAAAGTGATCGCCTTCCTCGGCGTGCTCTCCTG

TTTCCACACGGCGTTCATGCTATTCTGTGTCAGCGTCACTCGCTACCTGG
CTATTGCGCATCACCGCTTCTACACCAAGAGGCTGACCTTCTGGACCTGT
CTAGCCGTCATCTGCATGGTGTGGACGTTGTCAGTAGCCATGGCTTTTCC
CCCGGTGCTGGACGTAGGGACGTACTCCTTCATCCGGGAGGAGGACCAGT
GCACATTCCAGCACCGTTCTTTCAGGGCCAATGACTCACTGGGCTTCATG
CTCCTGCTGGCACTCATCCTCCTGGCCACACAGCTGGTTTACCTCAAGCT
CATCTTCTTTGTCCACGACCGTCGGAAGATGAAGCCCGTCCAGTTCGTGC
CCGCCGTCAGCCAGAACTGGACCTTCCATGGGCCAGGCGCCAGTGGGCAG
GCGGCGGCTAACTGGCTCGCTGGATTTGGGAGAGGCCCCACCCCGCCTAC
CTTGCTGGGCATCCGGCAGAACAGCAACGCGGCGGGCCGCAGGCGTCTGC
TGGTGCTGGATGAGTTCAAAACAGAGAAGAGGATTAGTAGGATGTTCTAC
ATCATGACGTTTTTCTTCCTGGCGTTGTGGGGGCCCTATCTGGTAGCCTG
CTACTGGCGGGTGTTTGCAAGGGGCCCCGTAGTCCCTGGGGGCTACCTGA
CGGCAGCCGTGTGGATGAACTTTGCCCAGGCTGGGGTCAATCCCTTCATC
TGCATCTTNNNNNNNNNNNNNNGCCAAATCTCGCTTTCACCCTGGCGTGGGGACTGGTCCTGGC
ACGGAGC---GCAGCGTCCCACTCGGCAACAGCTTGCTATCCCCGCAGCA
AACCGAGGAGCCCACTGTTGCCACCCCCCCGCAGCGATGGTTTGTCACCC
---CTGCCAACAACCGACTGGACTTTGCTGCCTCGGCATACGACGCTGCC
---------GATTTCGCCGGTAACGCGGCCACCTTGCTGTCCTACGCAGC
GGCCGGAGTGAAGGCTC------TTCCTCTGCCGGCTGCAGGCTGCTCCA
ACCGGCCGCTTGGCTATTACGCAGACCCGTCGG---GCTGG---GGCGGA
CGCACGCCGCCGCAGTACTGTAGCGTAAACAGCAAACCGAGCTCGGTCCT
TTCCTGTTGGCCCTCCAACTCTGTCGGCGGCAGAGCGGGCC---CC---A
ACTACCTGG---------CCGAGGA---CGGA---GACGC---CATCCCG
ACGGAGAGATCCCCG---AT---CGGTGGCTCCGAGGAG---ACGAAACC
CAAGGACCT------GTCGGA---GTCGAGCTGGATAGAG---ACGCCGT
CCTCCATAAAGTCAATCGATTCAAGCGATTCTGGGATCTTCG---AACAG
GCCAAAAGGAGGAGAATCTCGCCCTCTGCCACGCCG---------GTTTC
AGAAACTGTGTCCCCGTTAAAATCTGAGCATCACTCAACAGGCGAAGTCA
CAGAGAGAGAAGTGGCGTTGGGGATAAATCCGTTCGCGGACGGGATGGGC
GCCTTCAAAATCAACCACAGCTCCCACGATATTGGCTCCGG---ACAGAC
GGCGTTTTCCTCCCAGGCG---CCCGGCTAC---GCAGCGGCCGCCCTGG
GA---CACCATCA------CCACCCGACCCACGTTGGCTCT---TACTCC
ACGGCGGCTTTCAACTCCACCCGGGACTTTCTCTTTAGAAATCGGGGCTT
CGGAGACGCCACTGG------------GGCGCAGCACAGTTTGTTCGCCT
C---------CGGAAGTTT---C------GCAGGGCCACATGGACACTCA
GATGCCGCGGGACACCTGCTCTTCCCAGGACTCCACGAG---CAAGCAGC
GAGCCACGCGTCTTCTAATGTGGTCAACAGCCAGATGCGACTGGGCTTCT
CGGGGGACATGTACGGTCGGGCCGACCAGTATGGCCACGTTACAAGCCCA
AGGT---CCGACCACTATGCTTCGACCCAGTTGCACGGCTATGGCCCCAT
GAACATGAATATGGCCGCA---CACCACGGAGCAGGGGCCTTCTTTCGAT
ACATGAGGCAGCCGATCAAACAAGAGCTCATCTGCAAGTGGATCGAGCCG
GAGCAACTGACGAATCCCAAAAAGTCGTGCAACAAAACTTTTAGTACGAT
GCATGAGCTGGTGACCCATCTGACGGTGGAGCATGTGGGGGGACCAGAGC
AGTCCAACCACATCTGCTTCTGGGAAGACTGCGCCAGAGAAGGGAAACCA
TTCAAAGCCAAATACAAACTTGTGAATCATATCAGAGTACACACCGGAGA
GAAACACTTTCCATGTCCGTTTCCCGGCTGTGGCAAA
>Aulotrachichthys sajademalensis

------------TTTCTAGAGAGAAACCTTCATCCATCTAACTGCCTGGG
CATGCTGTTGCTGTCCGATGCACACCAGTGTACCAAGCTATCAGAGCTCT
CCTGGGGCATGTGCCTCAGCAACTTTCCGGCCATTTGCAAGACAGAGGAC
TTCCTCCAGCTGCCCAAAGACATGGTGGTGCAGCTTCTATCCCACGAGGA
GCTGGAGACAGAAGATGAGAGACTGGTTTATGAAGCTGCCCTTAACTGGG
TCAATTATGACCTGGAAAGGAGGCACTGCCATCTGCCAGAGCTGCTGAGA
ACAGTTCGCCTGGCCCTGCTACCTGCCATCTTCCTCATGGAGAATGTCTC
TACAGAAGAGCTAATCAATGCCCAGGCCAAGAGCAAGGAGCTGGTAGATG
AGGCCATCCGCTGCAAGCTAAAGATCTTACAGAATGACGGTGTGGTTAAC
AGCCCCTGCGCTCGGCCGAGAAAAACCAGCCACGCCCTTTTTCTGTTGGG
AGGGCAGACCTTCATGTGTGACAAGCTGTATCTGGTGGACCAGAAAGCGA
AAGAGATCATCCCCAAGGCTGACATCCCCAGCCCTAGGAAGGAGTTCAGT
GCCTGCGCCATCGGCTGTAAGGTCTACATCACAGGCGGGA--GAGGCTC-
AGAAAACGGCGTGTCTAAAGACGTATGGGTCTATGACACCGTCCAYGAGG
AATGGTCCAAGGCAGCACCCATGCTCATTGCCAGATTTGGTCACGGCTCT
GCTGAGCTGAAACACTGCCTGTACGTAGTAGGAGGTCACACGGCAGCAAC
TGGCTGCCTCCCAGCCTCTCCCTCCGGATGACTACATTGTAGTGTTCAGC
CGTTCAGCAACAAGGCTGATTCTGAACGAAGCGGAGCTCATCATGACGCT
GGCCCAGGAATTTCAGATGAGAGTGGTTACAGTCTCCCTAGAGGAACAAA
CCTTCCCCAGCATCGCCCAGGTGATCAGTGGTGCCTCCATGTTAGTCAGC
ATGCATGGAGCTCAGCTTGTCACCTCACTCTTCCTGCCCAGAGGAGCTGC
TGTGGTGGAGCTGTTCCCCTATGCTGTCAACCCAGAACAGTACACCCCAT
ATAAAACCCTCGCCTCCTTACCAGGCATGGATCTTCAATATGTTTCCTGG
AGGAACACTATAGAGGATAACACTGTTACCCACCCAGACAGACCCTGGGA
AGAAGGAGGCATCGCCCATTTGGAAAAGGACGAGCAGGAGCGAATACTGG
CCAGCAAGGATGTCCCCAGACACCTGTGCTGCCGCAACCCAGAGTGGCTC
TTCCGAATCTATCAGGACACTTTGGTGGACATCCCTTCATTCGTGGAAGT
CCTCAA---AGAGGGCCTGAAG---ACCAGGCCCAGCTTGAAGAA---GG
CCAAGCTGGCCAGCACGGTACATCCAGGCCGGGTCAGAGAACCCCAGTGC
CACACCTCGGTCCAGGCCACCAACGAGGCTAAACTCACGGTTTCCTGGCA
GATCCCATGGAATCTGAAATACCTGAAGGTGCGAGAAGTGAAGTACGAGG
TGTGGATCCAGAAGAAGGATACCAGCAAGGGGACACTGGAGGATCAAATC
ATCCAGGCAAACCCAGCGCTGGAGGCTTTTGGTAACGCCAAAACAGTGAG
GAATGACAACTCATCTCGTTTTGGAAAATTCATCCGAATTCACTTCGGAA
CCAGTGGCAAGCTGTCCTCTGCTGATGTTGAAACATACCTATTGGAGAAG
TCACGTGTCACCTTTCAACTCAAAGCTGAAAGGAACTACCACATCTTCTT
CCAGATATTGTCCAATCAGAAGCCGGAACTGTTGGACATGCTGTTGATCA
CCAACAATCCATATGACTACTCCTACATCTCCCAAGGAGAGGTAACAGTA
GCATCCATCAACGACTCAGAGGAGCTGATGGCCACTGACAGCGCCTTTGA
CGTGCTTGGTTTTACTCTTGACGAGAAGATGGGAGTATATAAACTGATTG
GTGCCATTATGCACTATGGCAACATGAGATTTAAGCAGAAGCAGCGTGAA
GAGCAGGCTGAGCCTGACGGAACAGAGGCTGCTGATAAGTCAGCTTACCT

TATGGGGCTGAACTCTGCAGACCTCATCAAAGGGCTATGCCATCCCAGAG
TCAAGGTAGGAAATGAATATGTCACCAAAGGCCAAGGTGTAGACCAAGTC
TACTACCCAAACAAGGAGGCCTTCAAGTGCGAGGAGTGTGGCAAGCACTA
CAACACCAAGCTGGGATACAAGCGCCATGTGGCCATGCACTCTGCCACGG
CGGGGGACCTCACCTGCAAAGTGTGCATGCAGAGCTATGAGAGCACGCCG
GTGCTCCTGGAGCACCTTAAGAGTCACTCGGGGAAGTCCTCGGGCGGCGC
CAAGGAGAAGAAACACCCGTGCGACCACTGCGACCGCCGCTTCTACACGC
GCAAGGATGTGAGGCGGCACATGGTGGTCCACACGGGCCGGAAGGACTTC
CTGTGCCAGTACTGCGCCCAGCGCTTCGGCAGGAAGGACCACCTGACGCG
CCACGTGAAGAAGAGCCACTCGCAGGAGCTGCTGAAGATCAAGACAGAGC
CTCCAGATATGTTAGGTCTTTTAGGTTCTGGCTCACCGCCATGCTCTGTC
AAGGAAGAGCTTAGCCCTATGATGTGCAGCATGGGTGCCAACAAAGACCC
CATGATGGGCAAACCCTTCCCCAGCGGGACCCCCTTCCCCATGGGCATGT
ACAACCCCCACCAC------CTCCAGGCCATGTCCAATTCC------CCC
CACCCC------CATCCCTCTCTGATGCCTGGCTCCCTGTCTGCAGCTAT
GGGCATGGGCTGCCACATGGAGTATCTCATCTATGCCTCTTTCTCATTCA
TGGGATGTTTACAAATCAGTGATGGCTCAAACATTGTGAACTTGCTGGCT
AGTAATTCTCCGAGTGTTTCATATGCTCTGACCCAGCAGAAATACTTCAG
TAACTACAGTCCTGTGATTGGATTTTACATTTACGAGCCCATTGAGTACT
GGAACTCCACAGTGCAGGAGCACCTGAAGACACTGAGTCATGGCTTCAAC
AAGATCTCCTGGATGGACAACTTCTTTCACTACCTGCGGGTGGTGAACGT
GAGTGCGTCAACCAAGAATGACTTTATCAGCATTCTCAAGGGCTCTTTCC
TGCGTAGCCCGGAGTACCAGCACTTCACTGAGGACATCATCTTCTCCAAG
A---ACCGCGAGAGTG------ATGAGTATGACATTATTGCCTCGCGCAT
GTACCTAGTGGCGCGGACCACAGAGAAGAAGCGCGAGGAGGTGGTGGAGC
TTCTGGAGAAACTGCGTCCACTGATGCTGATCAATAGCATCAAGTTCATC
GCCTTCAACCCCACCTTTGTTTTCATGGACCGCTACAGCTCCTCAGTCAT
CTCGCCCATCCTGACCTCAGGCTTCAGCGTGCTCACCATCCTCATCCTCA
CTTTCTTCCTGGTCATCAACCCCTTGGGGAACTTCTGGCTCATCCTCACA
GTTACTTCTGTGGAGCTGGGTGTCTTGGGTTTAATGNNNNNNNNNNNNNNNNNNNNNNNNNNNNNNNNN
NNNNNNNNNNNNNNNNNTGCCATGTGGGCAT
TATTAATGGGCTCTCTGGATGGGCTTCCTTGGTGGATGACTCTCCAGCTG
ACACCATTACTCGACGGTATCGCTACGACGTGGCCCTGGTATCAGCCTTA
AAGGATCTGGAGGAGCACATCATGGAGGGACTGAAAGAGTGTGGGCTGGA
AGACAGCGCTTGCACCTCAGGCTTCAGTGTTATGATCAAGGAATCTTGTG
ATGGCATGGGAGATGTCAGCGAGAAGCACGGGGGAGGACCAGCGGTCCCC
GAGAAGGCTGTACGCTTCTCTATTACTGTTATGTCTGTCTCTGTCGTGGC
AGATGGAGAGGAA------------------------------GAGGCGG
TTACCATCTTTCTTGAGCCAAAGCCCAACTCAGAACTGTCCTGTAAGCCC
GTAAGCCTGATGTTTGTGGATGAGTCAGACCATGAGACACTCACTGCTGT
CCTGGGACCTTTAGTGGCAGAGCGTAATGCAATGAAACAGAGTCGACTCA
TCCTATCTATGGGTGGCCTCCCTCGTTCCTTCCGCTTCCACTTCAGAGCC
ACAGGATATGATGAGAAGATGGTGCGTGACATGGAGGGCCTGGAGGCCTC
AGGTTCCACCTACGTCTGCACTCTGTGTGATTCCAGTCGAGCAGAAGCGT
CTCACAACATGGTGCTACACTCCATCACCCGCAACCATGATGAGAACATG
GAGCGTTATGAAATATGGAGGACCAATCCCTTTTCTGAGTCTGTTGAAGA
GCTGCGAGACCGGGTCAAAGGAGTCTCTGCTAAGCCGTTTATGGAGACCC
AGCCCACTATTGATGCATTGCATTGTGACATTGGTAATGCCACAGAGTTC
TACAAAATCTTCCAGGATGAGATCGGGGAGGTGTACCAGAGGGC---CAA
C---CCCACCCGGGAGGAACGGCGAAGGTGGCGAGCAGCCCTAGATAAGC
AACTGAGGAAGAAGATGAAGCTTAAACCAGTGATGAGGATGAATGGGAAC
TATGCCCGGAGGCTCATGACCCTGGAGGCCGTGGAGGTGGTTTGTGAGCT
GGTACCCTCAGAAGAAAGGCGAAAGGCCCTGAGGGAGCTTATGGGGCTCT

ATATTCAGATGAAGCCTGTGTGGCGCTCGACCTGCCCAGCCAAGGAATGC
CCTGATGAGCTGTGCCGCTACAGCTTTAACTCCCAGCACTTTGCCGATCT
CCTCTCCACTACCTTCAAATATAGGTACAATGGAAAGATCTCCAATTACC
TGCATAAGACCCTCGCCCATGTCCCTGAAATCATTGAAAGAGATGGCTCT
ATAGGAGCCTGGGCCAGCGAGGNNNNNNNNNNNNNNNNNNNNTCGTACACCATTGAGATGGCTCCGAAGG
GCCCCCAGTGGAAGGAGAGTCCTCAGCCTTTCTCCTGCTCCATTGAAGAC
CCCACCAAGCAGACCAAGTTCAAAGGCATCAAGACCTACATTTCCTACCG
GGTCACGCCGAGCCACACGGGCCGCCCCGTCTACCGGCGCTACAAACACT
TTGACTGGCTGTACAACCGCTTGCTGCACAAGTTCACTGTGATCTCGGTG
CCCCACCTGCCGGAGAAGCAGGCCACGGGGCGCTTCGAGGAGGACTTCAT
CGAGAAGCGCAAAAGACGGCTGATCCTGTGGATGAACCACATGACCAGCC
ACCCGGTCCTCTCCCAGTACGAGGGGTTCGAGCACTTCCTGATGTGCGCC
GACGACAAGCAGTGGAAGCTGGGCAAGAGGCGGGCGGAGAAGGACGAGAT
GGTGGGCGCCCACTTCATGCTGACCTTCCAGATTCCCAACGAGCACCAGG
ACCTCCAGGACGTGGAGGAGCGGGTGGACTCCTTCAAGTCCTTCGCCAAG
AAGATGGACGACAGCGTCATGCAGCTCACGCACGTCGCCTCGGAGCTGGT
CCGCAAGCACCTGGGCGGCTTCCGGAAGGAGTTCCAGCGGCTCGGGAACG
CCTTCCAGTCCATCAGCCAGGCCTTCATGCTGGAACCCCCCCACAGCTCC
GACGGCCTCAACAACGCCATCTCCCACNTCTCTCGCACGTTCCTCAAACTG
ACCTCTCTGGGTTTCATCATTGGAGTCGGTGTGGTCGGGAACCTCCTGAT
CTCCATCCTGCTGGTCAAAGACAAGAGCCTGCACCGCGCGCCCTACTACT
TCCTGCTCGACCTGTGCGCCTCCGACATCCTGCGCTCTGCCATCTGCTTC
CCCTTTGTCTTCACCTCTGTCAAGAATGGATCCGCCTGGACCTATGGCAC
GCTCACCTGCAAAGTGATCGCCTTCCTCGGCGTGCTCTCCTGTTTCCACA
CGGCGTTCATGCTATTCTGTGTCAGCGTCACTCGCTACCTGGCTATTGCG
CATCACCGCTTCTACACCAAGAGGCTGACCTTCTGGACCTGTCTAGCCGT
CATCTGCATGGTGTGGACGTTGTCAGTAGCCATGGCTTTTCCCCCGGTGC
TGGACGTAGGGACGTACTCCTTCATCCGGGAGGAGGACCAGTGCACATTC
CAGCACCGTTCTTTCAGGGCCAATGACTCACTGGGCTTCATGCTCCTGCT
GGCACTCATCCTCCTGGCCACACAGCTGGTTTACCTCAAGCTCATCTTCT
TTGTCCACGACCGTCGGAAGATGAAGCCCGTCCAGTTCGTGCCCGCCGTC
AGCCAGAACTGGACCTTCCATGGGCCAGGCGCCAGTGGGCAGGCGGCGGC
TAACTGGCTCGCTGGATTTGGGAGAGGCCCCACCCCGCCTACCTTGCTGG
GCATCCGGCAGAACAGCAACGCGGCGGGCCGCAGGCGTCTGCTGGTGCTG
GATGAGTTCAAAACAGAGAAGAGGATTAGTAGGATGTTCTACATCATGAC
GTTTTTCTTCCTGGCGTTGTGGGGGCCCTATCTGGTAGCCTGCTACTGGC
GGGTGTTTGCAAGGGGNCCCGTAGTCCCTGGGGGCTACCTGACGGCAGCCG
TGTGGATGAGCTTTGCCCAGGCTGGGGTCAATCCCTTCATCTGCATCTTC
TCTAACAGGGAGGCCAAATCTCGCTTTCACCCTGGCGTGGGGACTGGTCC
TGGCACGGAGC---GCAGCGTCCCACTCGGCAACAGCTTGCTATCCCCGC
AGCAAACCGAGGAGCCCACTGTTGCCACCCCCCCGCAGCGATGGTTTGTC
ACCC---CTGCCAACAACCGACTGGACTTTGCTGCCTCGGCATACGACGC
TGCC---------GATTTCGCCGGTAACGCGGCCACCTTGCTGTCCTACG
CAGCGGCCGGAGTGAAGGCTC------TTCCTCTGCCGGCTGCAGGCTGC
TCCAACCGGCCGCTTGGCTATTACGCAGACCCGTCGG---GCTGG---GG
CGGACGCACGCCGCCGCAGTACTGTAGCGTAAACAGCAAACCGAGCTCGG
TCCTTTCCTGCTGGCCCTCTAACTCTGTCGGCGGCAGAGCGGGCC---CC
---AACTACCTGG---------CCGAGGA---CGGA---GACGC---CAT
CCCGACGGAGAGATCCCCG---AT---CGGTGGCTCCGAGGAG---ACGA
AACCCAAGGACCT------GTCGGA---GTCGAGCTGGATAGAG---ACG
CCGTCCTCCATAAAGTCAATCGATTCAAGCGATTCTGGGATCTTCG---A
ACAGGCCAAAAGGAGGAGAATCTCGCCCTCTGCCACGCCG---------G
TTTCAGAAACTGTGTCCCCGTTAAAATCTNNNCATCACTCAACAGGCGAAGTC

ACAGAGAGAGAAGTGGCGTTGGGGATAAATCCGTTCGCGGACGGGATGGG
CGCCTTCAAAATCAACCACAGCTCCCACGATATTGGCTCCGG---ACAGA
CGGCGTTTTCCTCCCAGGCG---CCCGGCTAC---GCAGCGGCCGCCCTG
GGA---CACCATCA------CCACCCGACCCACGTTGGCTCT---TACTC
CACGGCGGCTTTCAACTCCACCCGGGACTTTCTCTTTAGAAATCGGGGCT
TCGGAGACGCCACTGG------------GGCGCAGCACAGTTTGTTCGCC
TC---------CGGAAGTTT---C------GCAGGGCCACATGGACACTC
AGATGCCGCGGGACACCTGCTCTTCCCAGGACTCCACGAG---CAAGCAG
CGAGCCACGCGTCTTCTAATGTGGTCAACAGCCAGATGCGACTGGGCTTC
TCGGGGGACATGTACGGTCGGGCCGACCAGTATGGCCACGTTACAAGCCC
AAGGT---CCGACCACTATGCTTCGACCCAGTTGCACGGCTATGGCCCCA
TGAACATGAATATGGCCGCA---CACCACGGAGCAGGGGCCTTCTTTCGA
TACATGAGGCAGCCGATCAAACAAGAGCTCATCTGCAAGTGGATCGAGCC
GGAGCAACTGACGAATCCCAAAAAGTCGTGCAACAAAACTTTTAGTACGA
TGCATGAGCTGGTGACCCATCTGACGGTGGAGCATGTGGGGGGACCAGAG
CAGTCCAACCACATCTGCTTCTGGGAAGACTGCGCCAGAGAAGGGAAACC
ATTCAAAGCCAAATACAAACTTGTGAATCATATCAGAGTACACACCGGAG
AGAAACCCTTTCCATGTCCGTTCCCCGGCTGTGGANNN
>Avocettina infans
AGCCTGCTAATCCGGGCGGAGCTGAGTCAACCCGGAGCCTTTTTGGGAGA
CGACCAAATTTATAACGTCATCGTTACAGCACACGCTTTCGTGATAATTT
TCTTTATGGTAATACCAATCATGATTGGAGGCTTTGGAAACTGGCTAGTT
CCCCTAATGATCGGAGCCCCAGACATGGCCTTCCCACGAATAAACAACAT
AAGCTTTTGGTTGTTACCCCCTTCATTTCTTCTATTGTTATCCTCCTCGG
GAGTTGAAGCCGGGGCCGGAACAGGATGAACGGTCTACCCCCCTCTTGCT
GGCAATTTAGCTCATGCCGGAGCATCAGTAGACCTAACCATCTTTTCTCT
ACACTTAGCAGGTGTCTCATCAATTCTTGGGGCAATCAACTTTATTACCA
CAATTATTAACATGAAACCACCAGCCATCTCACAATACCAAACCCCCCTG
TTTGTGTGATCAGTGCTTATCACTGCAGTGCTATTATTATTATCACTCCC
AGTTTAGCTGCGGGCATTACCATGCTCCTTACAGATCGAAACTTAAATA
CAACCTTCTTTGACCCGGCAGGAGGAGGGGACCCAATTTTATATCAACAT
TTATTTTGGTTCTTCGGCCACCCCGAAGTATATATTCTTATTCTCCCAGG
GTTTGGTATAATTTCACACATCGTCGCATACTATGCCGGGAAAAAAGAAC
CATTTGGCTATATAGGAATAGTCTGAGCCATGATGGCCATTGGACTCCTC
GGGTTTATTGTCTGAGCACACCACATGTTTACAGTGGGTATAGACGTAGA
CACACGAGCCTA--------------------------------------

-------------------------TGACGAGTATATTGTGGTCTTCAGC
CGCTCTCTTAACCGGCTTATCCTGAACGAGGCAGAGCTGATCCTGGCACT

AGCGCAGGAGTTCCAAATGAAGGTTGTTACCGTGTCCCTGGAGGAGCAGT
CCTTTGCAGACATCGTCCGAGTCCTCAGCAGGGCGTCGATGCTGGTCAGT
ATGCATGGGGCCCAGCTGGTCACCTCTCTCTTCCTTCCTCGTGGGGCAGC
TGTGGTGGAGCTGTACCCATATGCAGTCAACCCAGAGCATTATGCTCCGT
ATAGGACGCTGACCTCGCTGCCGGGCATGGACCTGCAGTATGTGGCCTGG
AGGAACACCAAAGAGGAGAACTCTGTGACCTTCCCTGAGCGTGCCTGGGA
CCAGGGTGGCATTGCACACTTGGAGAAGGAGGAGCAGGAACGTATCATGA
AGAGCAAGGAGGTGCCGCGACACCTGTGCTGTCGGAACCCGGAGTGGCTC
TTCCGCATCTACCAGGACACCAATGTGGATGTTGCGTCTCTTCTGGAAGC
CCTGCG---CCAGGGACTGACC---TCCAGGCCAGGGCCCAAGAG---GG
CTAGGCCCGCCAGCACAGTCCACCCAGGCAGGGTGAGGGAGCCCAAGTGC
CAGACCTCCGTCCAGGCAACCAACGAG------------G----------

-----------AAGAAAGATACCAGCAAGGGAACACTGGAGGATCAAATT
ATCCAGGCAAACCCAGCGCTGGAGGCTTTTGGCAATGCCAAGACTTCGAG
GAATGACAACTCCTCACGTTTTGGGAAATTTATTCGTATTCATTTCGGAA
TAAGTGGCAAGCTGTCCTCTGCTGACGTAGAAACCTACCTACTCGAGAAA
TCCCGTGTCACCTATCAGCTAAAAGCTGAGAGGAACTATCACATTTTCTA
CCAGATCACATCCAACAAAAAGCCAGAATTACTGGACATGCTGTTGATCA
CCAACAACCCGTATGATTATGCTTATGTCTCCCAAGGAGAGGTGACGGTT
GCATCCATCGATGACTCAGAGGAACTGATCGCCACAGACAGTGCCTTCGA
TGTGCTGGGCTTCACGCCCGAGGAGAAGATGGGTGTCTACAAGCTGACGG
GTGCCATCATGCATTACGGAAACATGAAATTCAAGCAGAAGCAACGTGAG
GAGCAGGCCGAGCCTGATGGCTCTGAGTCTGCTGACAAGTCAGCCTTCCT
GATGGGGCTGAACTCGGCCGACCTTCTCAAGGGACTGTGCCATCCACGGG
TCAAAGTTGGAAATGAGTATGTCACCAAAGGTCAAAATGTGGATCAAGTC
TACTATCCCAACAAAGAGGCCTTCAAGTGCGAAGAGTGCGGCAAGAACTA
CAACACCAAGCTGGGCTACAAGCGCCACGTGGCCATGCACGCAGCCACCA
GCGGCGACCTCACCTGCAAGGTGTGTCTGCAGAGCTACGAGAGCACGCCT
GTGCTGTTGGAGCACCTCAAGAGCCACTCGGGGAAGTCCTCCGGTGGGGC
CAAGGAGAAAAAGCACCCCTGCGACCACTGCGACCGCCGCTTCTACACGC
GCAAGGACGTGCGACGCCACATGGTGGTGCACACCGGCCGCAAGGACTTC
CTGTGCCAGTACTGCGCCCAGCGCTTCGGCCGAAAGGACCACCTGACCCG
GCATGTGAAGAAGAGCCACTCGCAAGAGCTGCTGAAGATCAAGACAGAGC
CGCCGGACATGTTGAGCCTGCTGGGTTCCGGCTCTCCACCCTGCTCTGTA
AAAGAGGAGCTCAGCCCCATGATGTG---TATGGGCCCCTCCAAGGACTC
CCTGATGGGCAAGCCCTTCCCTGGTG------CTTTCCCCATGGGCATGT
ACAACCCCCAC---------CTGCAGGCTATGTCCAACTCAGGGGTGTCC
------------CACCACTCTCTGGTGTCTGGGTCACTGTCTGCTGCCAT
GGGAATGGGCTGCCACATGGAGTACCTGATCTACGCTTCGTTTTCGTTCA
TGGGATGTTTACAAATCAGCGACGGGTCGAACGTGATCAACCTCCTGGCC
AGCAACTCCCCGAGCGTCACCTACGCTCTGACCCAGCAGAAGTACTTCAG
CAACTACAGTCCGGTCATCGGGTTCTACATCTACGAACCCATCGAGTACT
GGAACGCCACGGTTCAAGAGCACCTGAGGACGCTGGGCCACGGTTTCAAT
AAGATTTCGTGGATCGACAACTTCTTTCAGCATCTGAAGGTGGTGAACGT
CAGCGTGTCGACCAAAAGCGAATTCATAACCATCCTCCAGACGTCCTTCC
TGAGGAGCCCAGAGTACCAGCACTTCAAGGACGACATCATATTTTACAAA
A---TTAGGG------------ACGAGATGGAGATCATGGCATCCCGGAT
GTATCTGGTGGCCCGAACCATGGAGAAGACTCGAGAGGAGGTGGTGGAGC
TACTGGAGAGGCTGAGGCCGCTCTCTCTCGTCAACAGCATCAAATTCATC
GTCTTCAACCCCACCTTCGTCTTCATGGATCGGTACAGCTCGTCGGTCAT
CTCCCCCATCCTCACTTCGTGCTTCAGCGTCCTGATCGTCCTGATCCTGA
CCTTCTTCCTGGTCATCAACCCTCTGGGAAACTTCTGGCTGATACTGACA

GTCACCTCGGTGGAGCTGGGAGTCCTGGGCCTAATG--------------

---TCCTACA
CTATTGAAATGGGATCCTGGGGGCCCCAGTGGAAGGAGAGCCCCCAGCCA
TTCTCCTGCTCCATAGAGGACCCCACCAAGCAGACCAAGTTCAAGGGCAT
AAAGACTTACATCTCATACCGTGTGACACCCAGCCACACAGGCCGGCCTG
TGTACCGCCGCTACAAGCATTTTGACTGGCTGTACAACCGGTTGCTGCAC
AAGTTCACCGTCATCTCTGTCCCTCACCTGCCTGAGAAGCAGGCCACAGG
TCGCTTCGAGGAGGACTTCATTGAGAAGCGCAAGCGGCGGCTCATCCTAT
GGATGAACCACATGACCAGTCACCCGGTGCTCTCGCAGTATGAGGGTTTC
GAGCACTTCCTGATGTGCGCTGATGACAAGCAGTGGAAGCTGGGAAAGCG
GCGGGCAGAGAAGGACGAGATGGTGGGCGCGCACTTCATGCTCACCTTCC
AGATTCCCAATGAGCACCAGGACCTGCAGGACGTGGAGGAGAGGGTGGAC
TCTTTCAAGGCCTTTGCCAAAAAGATGGATGACAGCGTACTGCAGCTCAC
GCACACGGCCTCCGAGCTGGTGCGCAAGCATCTGGGAGGCTTCCGGAAGG
AATTCCAGAAGCTGGGGAATGYCTTCCAGTCCATCAGCCAGGCGTTCACT
CTGGATCCTCCCCACTGCTCTGATGCCCTCAACACCGCTTAT-----C--

-----------------------------------GCCAAATCCCGCTTC
CACCCTGGCGCCGGGACCGGTCCTGGCACGGACC---GCAGCGTCCCACT
TAGCAACAGCTTGCTATCCCCGCAACAAGCCGAAGAGACCGCAGCAG---
CCTCCCCGCAGCGATGGTTTGTCACCC---CTGCCAACAATCGACTGGAC
TTTGCCGCCTCGGCATACGATGCCGCTGCCGCGGCTGATTTTGCCGGTAA
CGCTGCCACCCTGCTGTCTTACGCAGCTGCTGGAGTCAAGGCGC------
TTCCACTGCCCACTGCGGGGTGTTCAGGTAGACCTCTGGGTTATTACACC
GACCCTTCGG---GCTGG---GGCACGCGCACTCCACCCCAGTATTGT--
-------AATAAATCCAGCTCAGTTCTGTCCTGTTGGCCAACAAATTCCG
CGGGGAGTAGGTCTGGAA---CCTCAAATTACCTAG---------TGGAT
GA---GGGT---GACAC---GATGACAACGGAAAGGTCTCCA---CT---
CAGTGCGTCCGAGGAT---GCCAAACCTAAAGACCT------GTCGGA--
-GTCCAGCTGGATAGAG---ACACCTTCCTCCATAAAATCGATCGATTCA
AGTGATTCTGGAATTTTTG---AGCAGGCTAAACGGAGGCGAATATCCCC
TTCTGCCACACCA---------GTTTCTGAAACTGCGTCTCCCTTGAAAA
GCGAA---

>Banjos banjos

------------NNNNNNNNNNGAAACCTTCACCCATCTAACTGCCTTGGCATGCTGTTG
CTGTCTGATGCCCACCAGTGCACCAAGCTGTCAGAGCTCTCCTGGGGCAT
GTGCCTCAGCAACTTTCCAGCTATTTGCAAGACAGAGGACTTCCTCCAAC
TGCCCAAAGATATGGTGGTGCAGCTTTTGTCACACGAGGAGCTAGAGACA
GAAGATGAGAGACTGGTTTATGAAGCTGCCCTCAACTGGATCAACTATGA
CCTGGAAAAGAGGCACTGCCACCTTCCAGAGCTCCTGAGAACGGTCCGCC
TGGCCCTGCTGCCTGCCATCTTTCTCATGGAGAATGTTTCTACAGAAGAG
CTGATCAATGCCCAGGCCAAGAGCAAGGAGCTGGTGGATGAAGCTATCCG
CTGTAAGCTGAAGATCCTGCAGAATGATGGCGTCGTTAACAGTCCATGTG
CTCGACCAAGAAAAACCAGCCATGCTCTCTTTCTTCTGGGAGGGCAGACT
TTCATGTGTGACAAGTTGTACCTGGTGGACCAGAAAGCCAAAGAGATCAT
CCCCAAAGCTGACATTCCCAGCCCCAGGAAGGAGTTCAGCGCCTGCGCCA
TCGGCTGTAAGGTGTACATCACTGGTGGGA--GAGGCTC-AGAGAATGGT
GTGTCCAAAGATGTGTGGGTCTACGACACCGTCCACGAGGAATGGTCGAA
GGCGGCACCCATGCTCATTGCCAGGTTTGGCCATGGCTCTGCGGAGCTGA
AACACTGCCTCTACGNNNNNNNNNNNNNNNNNNNNNNNNNNNNNNNNNNNNNNNNNNNNNNNNNGGATG
AATACATTGTTGTGTTTAGTCGTTCAACAA
CGAGGTTGATACTGAATGAAGTGGAGCTAATCATGGCGCTGGCCCAAGAG
TTCCAGATGAGAGTGGTCACGGTATCCCTGGAGGAACAGTCTTTCCCCAG
TATCGTCCAGGTGATCAGTGGTGCTTCCATGTTAGTCAGTATGCATGGAG
CTCAGCTTATCACCTCACTCTTCCTCCCCAGAGGAGCTGCTGTGGTGGAG
CTGTTCCCCTTTGCTGTGAACCCAGAGCAGTACACCCCGTATAAAACCCT
TGCCTCCCTTCCAGGCATGGACCTTCACTATGTCTCTTGGAGGAACACTA
AGGAGGAGAACACCATCACCCACCCAGACAGACCCTGGGAACAAGGGGGC
ATTGCTCACTTGGAGAAGGAGGAGCAAGAGCGAATACTGGCGAGCAAAGA
TGTCCCCAGGCACCTGTGCTGCCGCAACCCAGAGTGGCTCTTCCGGATCT
ACCAGGACACTTTGGTGGACATCCCTTCCTTCCTGGAAGTCCTCAA---A
GAGGGCATGAAG---ACAAAGCCCAGCTTGAAGAA---GTCAAAGCCGGC
CAGCACAGTCCATCCGGGCCGGGTCAGAGAACCCCAATGTCAGACCTCAG
TACAAACCACTAATGAGGCTAAACTCACAGTCTCCTGGCAGATCCCGTGG
AATCTGAAATACCTGAAAGTGAGAGAGGTCAAATACGAGGTG--------
-AAAAAAGACACCAGTAAGGGGACCCTGGAGGATCAAATCATTCAGGCGA
ACCCTGCGCTGGAGGCCTTCGGCAACGCCAAAACGTTGAGAAACGACAAC
TCGTCTCGTTTTGGAAAATTCATCCGAATTCACTTCGGTACGAGTGGCAA
GCTGTCGTCTGCTGACATCGAGACGTACCTGCTGGAGAAGTCACGTGTCA
CCTTTCAGCTCAAGGCTGAGAGGAACTACCACATCTTCTACCAGATCCTG
TCCAATCAGAAGCCAGAGCTCCTGGACATGCTGCTGATCACCAACAACCC
GTACGACTACTCCTACATCTCCCAAGGAGAAGTAACGGTCGCCTCCATCA
ATGACTCAGAGGAGCTGATGGCCACCGACAGCGCCTTCGATGTGCTCGGC
TTCACTCCAGACGAGAAGATGGGTGTCTATAAACTGACTGGCGCCATCAT
GCACTACGGCAACATGAAGTTCAAACAGAAGCAGCGTGAGGAGCAAGCGG
AGCCGGATGGGACGGAAGCTGCTGATAAATCAGCTTACCTAATGGGGCTG
AACTCTGCTGACCTCATCAAAGGGCTGTGCCATCCCAGAGTCAAGGTAGG
AAATGAATACGTCACCAAAGGCCAAAGTGTGGACCAAGTCTACTACNNNNNNNNNNNNNNNNNNNNNNN
NNNNNNNNNNGGAA
GCACTACAACACCAAGCTGGGATATAAGCGCCATGTGGCCATGCACTCTG

CCACGGCAGGGGATCTTACCTGTAAAGTGTGCATGCAGACCTACGAGAGC
ACACCCGTGCTCTTGGAGCACCTCAAGAGCCACTCCGGGAAGTCTTCGGG
TGGTACCAAGGAGAAAAAACACCCGTGCGACCACTGTGACCGTCGTTTCT
ACACACGGAAGGATGTGAGACGCCACATGGTGGTCCACACGGGCCGAAAG
GACTTCCTGTGCCAGTACTGTGCCCAGCGCTTCGGCAGGAAGGACCATCT
GACACGGCACGTGAAGAAGAGCCACTCGCAGGAGCTGCTGAAGATCAAGA
CGGAGCCTCCTGATATGTTAGGTCTTTTAGCGTCTGGGTCACCACCCTGC
TCTGTGAAGGAGGAGCTCAGCCCCATGATGTGCGGCATGGGTCCCAACAA
AGACCCCATGATGGGCAAACCGTTCCCCAGTGGGGCCCCTTTCCCGATGG
GCATGTACAACCCCCACCAT------CTCCAGGCCATGTCTAATTCTGGG
GTGGGTCACCCA------CACCCGTCCCTGATGCCCAGTTCCTTGTCTGC
AGCTATGGGCATGGGCTGCCACATGNNNNNNNNNNNNNNNNNNNNNNNNNNNNNNNNNNNNNNNNNNNNNNNN
NNNNNNNNNNNNNNNNNNNTCGTGAACCTGCTGGCTAGTAACTC
TCCGAGTGTTTCTTACGCTCTGACCCAGCAGAAATACTTCAGTAACTACA
GTCCCGTGATTGGGTTTTACATTTACGAGCCCATCGAGTACTGGAACTCA
ACGGTGCAGGAGCACCTGAAGACTCTGAGTCATGGCTTCAACAAGATCTC
CTGGATGGACAACTTTTTCCACTACCTGCGGGTGGTGAATGTGAGTGCGT
CAACCAAGAGCGACTTCATCACCATCCTCAAGGGGTCCTTCCTGCGCAGC
CCGGAGTACCAGCACTTCACCGAGGACATCATATTCTCCAAGA---ACCG
CGAGACTG------ATGAGTACGACATTATCGCCTCACGGATGTACTTGG
TGGCGCGGACGACAGAGAAGAAGCGCGAAGAGGTGGTGGAGCTTCTGGAG
AAGCTTCGTCCGTTGATGCTGATCAACAGCATCAAGTTCATTGCCTTCAA
TCCTACGTTTGTGTTCATGGACCGCTACAGCTCCTCTGTCATCTCGCCCA
TTCTGACCTCAGGCTTCAGCGTACTCACAATCCTCATCCTCACTTTCTTC
CTGGTCATCAACCCCTTGGGAAACTTCTGNNNNNNNNNNNNNNNNNNNNNNNNNNNNNNNNNNNNNNNNNNN
NNNNNNNNNNNNNNNNNNNNNNNNNNNNNNNNNNNNNNNNNNNNNNNNNNNNNTCTTGCAATGTTGG
CATTATT
AATGGGCTCTCTGGATGGGTTTCCTCGGTGGATGACTCCCCAGCTGACAC
CATCACTCGTCGGTTTCGCTATGATGTGGCACTGGTGTCAGCAATAAAGG
ATCTGGAGGAGGACATCATGGAGGGGCTGAGAGAGAGTGGGATGGAAGAC
AGTGCGTGCACCTCAGGCTTCAGTGTCATGATCAAGGAATCTTGTGACGG
CATGGGCGATGTCAGCGAGAAGCACGGTGGAGGACCAGTTGTTCCTGAGA
AGGCTGTACGTTTCTCTTTCACTGTTATGTCTGTCTCTGTCCTGGCAGAC
GATGAGGAC--------------------------------GAAGAGGTTAC
CATCTTCACCGAGCCAAAGCCAAACTCAGAACTGTCCTGTAAGCCTCTTT
GCCTGACATTTGTGGATGAGTCAGACCATGAGACGCTCACAGCCGTCCTG
GGGCCTATAGTTGCAGAGCGTAATGCAATGAAAGAGAGCAGGCTCATCCT
ATCCATGGGTGGACTACCTCGCTCCTTCCGCTTTCACTTCAGAGGCACGG
GATACGATGAGAAGATGGTGCGTGAGATGGAGGGCCTCGAGGCCTCGGGG
TCCTCCTATATCTGCACTCTTTGTGACTCCAGTCGGGCAGAAGCCTCTCA
GAACATGGTGCTACACTCCGTCACCCGCAGTCATGAAGAGAACCTAGAAC
GTTACGAAATATGGAGAACCAACCCCTTTTCTGAGTCTGTAGATGAGCTG
CGAGACAGAGTCAAAGGGGTCTCTGCCAAGCCCTTCATGGAGACCCATCC
CACGCTGGATGCATTACACTGTGACATTGGCAATGCCACTGAGTTCTACA
AAATCTTCCAGGACGAGATCGGGGAGGTGTACCAAAAGGT---CAAC---
CCCAGCCGGGAGGAACGGCGTAGCTGGAGGGCAGCCCTAGATAAACAGCT
GAGGAAGAAGATAAAGCTTAAACCGGTAATGAGGATGAATGGGAACTATG
CCCGCAGGCTAATGACCCAGGAGACTGTGGAGGTGGTGTGTGAGCTGGTG
CCCTCAGAGGGGGAGGAGGGAGGCCCTGAGGGCGCTTATGAGGCTCTACCT
CCAGATGAAGCCTGTGTGGCGCGCCACCTGCCCAGCCAAGGAGTGCCCCG
ACCAGCTGTGCCGCTACAGCTTTAACTCCCAGCGCTTTGCCGACCTCCTC
TCCTCTACCTTCAAATATAGGTACAATGGAAAGATAACCAATTACCTGCA
CAAGACCCTGGCCCATGTGCCNNNNNNNNNNNNNNNNNNNNNNNNNNNNNNNNNNNNNNNNNNNNNNNNN

NNNNNNNNNNNNNNNNNN-----------------------------

-------------NNNNNNNNNNNNNNNNNNNNNNNNNNNNNNNNNNNNNNNNNNNCGGAGC---
GCAGCGTCCCACTCGGCAACAGCTTGCT
ATCCCCGCAGCAAACCGAGGAGCCCACTGTTGCCACCCCCCCGCAGCGAT
GGTTTGTCACCC---CTGCCAACAACCGACTGGACTTTGCTGCCTCGGCA
TACGACGCCGCT---------GATTTCGCCGGTAACGCGGCCACCTTGCT
GTCCTACGCAGCGGCCGGAGTGAAGGCTC------TTCCCCTGCCGACTG
CAGGCTGCTCCAACCGGCCTCTTGGCTATTACGCAGACCCGTCAG---GC
TGG---GGAGGACGCACGCCGCCGCAGTACTGTGGTGTAAATAGCAAATC
CAGCTCGGTCTTTTCCTGCTGGCCCGCTAACTCTATCGGTGGCAGAGCAG
GCA---CC---AACTACCTGG---------CGGAGGA---GGGA---GAC
TC---CATCCCGACAGAGAGGTCACCG---AT---CGGCGGCTCGGAGGA
G---ACCAAACCCAAAGACCTGAC---ATCAGA---GTCGAGCTGGATAG
AG---ACGCCGTCCTCCATTAAGTCCATTGATTCAAGCGATTCTGGTATC
TTTG---AACAGGCCAAAAGGAGAAGAATCTCACCTTCTGCCACGCCG--

GTTTCAGAGACTGTGTCCCCATTAAAATCNNNNNNNNNNNNNNNNNNNNNNNNNNNNNNNNNNNNNNNNN
NNNNNNNNNNNNNNNNGTTCGCGGATGGGA
TGGGCGCCTTCAAAATAAACCACAGCTCCCACGATATTGGCTCCGG---A
CAAACGGCGTTTTCCTCCCAAGCG---CCCGGCTAC---GCAGCAGCCGC

CCTGGGA---CACCATCA------CCACCCGACCCACGTTGGCTCT---T
ACTCCACGGCGGCTTTCAACTCCACCAGGGACTTTCTCTTCAGAAATCGG
GGTTTCGGGGACGCCACCGG------------GGCGCAGCACAGTTTGTT
CGCCTC---------CGGAAGTTT---C------GCAGGGCCACATGGAC
ACTCAGATGCAGCGGGGCACCTGCTCTTCCCAGGGCTCCACGAG---CAA
GCGGCGAGCCATGCGTCTTCCAACGTGGTCAACAGCCAGATGCGGCTGGG
CTTCTCGGGGGACATGTACGGACGGGCCGACCAGTACGGCCACGTTACAA
GCCCGCGGT---CCGACCACTATGCTTCGACCCAGCTGCACGGCTATGGC
CCCATGAACATGAATATGGCCGCA---CACCACGGAGCAGGGGCCTTCTT
TCGATACATGAGGCAGCCGATCAAACAAGAGCTCATCTGCAAGTGGATCG
AGCCGGAGCAGCTGACGAATCCCAAAAAGTCGTGCAACAAAACTTTTAGC
ACGATGCACGAGCTTGTGACCCATCTGACGGTGGAGCATGTGGGGGGACC
AGAGCAGACCAACCACATCTGCTTCTGGGAGGACTGGGCCCNNNNNNNNNNNNNNNNNNNNNNNNNNNNN
NNNNNNNNNNNNNNNNNNNNNNNNNNNNNNNNNNNNNNNNNNNNNNNNNNNNNNNNNNNNNNNNNNNNNN
NN
>Barathronus maculatus

------------TTTCTAGAGAGAAACCTTCACCCCTCTAACTGCCTCGG
CATGCTGTTGCTATCTGATGCCCATCAGTGCACCAAGCTATCAGAGCTGT
CCTGGGGCATGTGCCTCAGTAACTTTCCTGCTATTTGCAAGACAGAGGAC
TTCCTCCAACTACCCAAAGACATGGTGTTGCAGCTTTTGTCACATGAGGA
GCTCGAGACAGAAGATGAGAGACTGGTTTATGAAGCTGCCCTTAACTGGA
TCAACTATGACCTGGAAAGGAGGCACTGCCACCTCCCAGACCTTCTGAGA
ACGGTCCGCCTGGCCCTGCTTCCTGCCATCTTTCTCATGGAGAATGTCTC
GACAGAAGAGCTGATCAATGCCCAGGCCAAGAGCAAGGAGCTAGTTGATG
AGGCCATCCGCTGTAAGCTGAAGATCCTACAGAATGATGGCGTAGTTAAC
AGCCCTTGTGCCAGACCAAGAAAAACCAGCCATGCACTCTTTCTGCTGGG
AGGGCAGACTTTCATGTGTGACAAACTGTACCTGGTAGATCAGAAAGCCA
AAGAGATCATTCCCAAAGCTGACATCCCCAGCCCCAGGAAGGAGTTCAGT
GCCTGTGCCATTGGCTGTAAGGTGTACATCACAGGTGGGA--GAGGCTC-
AGAAAATGGTGTGTCCAAAGATGTATGGGTCTACGACACCATCCNNNNNNNNNNNNNNNNNNNNNNNNNN
NNNNNNNNNNNNNNNNNNNNNNNNNNNNNNNNNNNNNNNNNNNNNNNNNNNNNNNNNNNNNNNNNNNNNN
NNNNNNNNNNNNNNNNNNNNNNNNNNNNNNNNNNNGGATGA
ATACATTGTTCTGTTTAGCCGTTCAACAACAAGGCTGATACTGAATGAAG
CTGAGCTAATCATGGCGCTGGCCCAGGAATTTCAAATGAGAGTGGTTACG
GTCTCCCTGGAGGAACAATCTTTCCCAAGTATTGTCCAGGTGATCAGTGG
TGCTTCTATATTGATTAGTATGCATGGAGCTCAGCTTATCACCTCGCTTT
TCCTCCCCAGAGGAGCTGCTGTGGTGGAGCTGTTCCCCTATGCTGTAAAC

CCAGAGCAGTACACCCCATATAAAACCCTGGCCTCCCTTCCAGGCATGGA
CCTCCACTATGTCTCCTGGAGGAACAGCATGGAGGAGAACACCGTCATCC
ACCCACACAGACCCTGGGAACAAGGAGGAATCGCTCACTTGGAGAAGGAG
GAGCAACAGCGAATACTTACCAGCAAAGATGTCCCCAGACATCTGTGTTG
CCGCAACCCTGAATGGCTCTTCCGAATCTACCAGGACACTTTGGTGGACA
TCCCATCATTCATGGAAGTCCTCAA---AGAGGGCCTGAAG---ACCAAG
CCTAGCTTAAAGAA---GGCCAAGCCGGCCAGCACAGTTCACCCAGGCCG
GGTCAGAGAAGCCCAGTGTCAGACCTCAGTGCAATCCAGCNNNNNNNNNNNNNNNNNNNNNNNNNNNNNN
NNNNNNNNNNNNNNNNNNNNNNNNNNNNNNNNNNNNNNNNNNNNNNNNNNNNNNNNNNNNAAGAAAGACA
CCAGCAAGGGGACACTGGAAGATCAAATCATCCAGGCGAACCCAGCACTG
GAGGCCTTCGGTAATGCCAAAACATTGAGAAATGACAACTCCTCTCGTTT
TGGAAAATTCATCCGAATTCACTTTGGAACCAGTGGTAAGCTGTCATCCG
CTGACATTGAGACGTATCTGCTGGAGAAGTCACGTGTCACCTTTCAGCTC
AAGGCTGAGAGGGACTACCACATCTTCTACCAGATCCTGTCCAATCAGAA
GCCAGAGCTTTTGGATATGCTGCTGATCACAAACAACCCGTATGACTACT
CCTACATCTCCCAAGGAGAGGTGACAGTTGCATCCATCAATGACGCAGAG
GAGCTGATGGCCACCGATAGTGCCTTCGACGTGCTCGGCTTCACTCCAGA
GGAGAAGATGGGCGTCTATAAGCTGACTGGTGCCATCATGCACTACGGCA
ACATGAAGTTTAAACAGAAGCAGCGTGAGGAGCAGGCTGAATCTGATGGG
ACAGAGGCTGCCGATAAATCAGCTTACCTGATGGGTCTGAATTCTGCAGA
CCTCATCAAAGGGTTGTGTCATCCCAGAGTCAAGGTAGGGAATGAATACG
TCACAAAAGGCCAAAGTGTCGACCAAGTCTACTATCCCAACAAGGAGGCC
TTCAAGTGTGAGGAGTGTGGCAAGCACTACAACACCAAGCTGGGATATAA
GCGACATGTGGCCATGCACTCTGCCACAGCAGGGGATCTCACCTGTAAAG
TGTGCATGCAGAGCTATGAGAGCACACCAGTGCTCCTGGAGCACCTCAAG
AGCCACTCGGGGAAGTCTTCGGGTGGCACCAAGGAGAAAAAACACCCATG
TGACCACTGTGATCGTCGTTTCTACACACGGAAGGATGTGAGACGGCACA
TGGTGGTCCACACGGGCCGAAAGGACTTTCTGTGCCAGTACTGTGCCCAG
CGCTTTGGCAGGAAGGACCACCTGACACGGCACGTGAAGAAGAGCCACTC
TCAGGAGCTGCTGAAGATCAAGACGGAGCCCCCGGATATGTTAGGTCTTT
TAGCATCGGGGTCACCACCTTGCCCAGTGAAGGAGGAGCTTAGCCCCATG
ATGTGCGGCATGGGTCCAAACAAAGATCCCATGATGGGAAAACCTTTCCC
AAGTGGAGCCCCTTTTCCGATAGGCATGTACAACCCCCACCAT------C
TCCAGGCCATGTCCAGTTCTGGGGTGGGCCACCCC------
CACCCCNNNNNNNNNNNNNNNNNNNNNNNNNNNNNNNNNNNNNNNNNNNNNNNNNNNNTAT
CTCATCTATGCATCTTTCTCATTCATGGGATGTTTACAAATCAGTGATGG
ATCAAACATCGTGAACCTGCTGGCTAGTAACTCTCCCAGTGTTTCATATG
CTCTGACTCAGGAGAAATACTTCAGCAACTACAGTCCCGTGATTGGTTTT
TACATTTATGAGCCCATTGAGTACTGGAACTCCACGGTGCAGGAGCATCT
GAAGACTCTAGGTCACGGCTTCAACAAGATCTCCTGGATGGACAACTTTT
TCCACTACCTGCGGGTGGTGAATGTGAGCGCATCGACCAAGAGTGATTTT
ATCACCATCCTCAAGGGCTCTTTCTTACGCAGCCCGGAGTACCAGCACTT
CACTGAGGACATCATCTTCTCTAAGA---ACCGTGAGACTG------ATG
AATATGACATTATTGCCTCACGAATGTACTTGGTGGCGAGGACCACAGAG
AAGAAACGTGAAGAGGTGGTGCAGCTGCTGGAAAAGCTACGTCCATTGAT
GCTGATTAACAGCATCAAGTTCATTGCTTTCAATCCCACTTTTGTGTTCA
TGGACCGCTACAGTTCCTCTGTCATTTCACCCATCCTGACCTCAGGCTTT
AGTGTGCTCACTATCCTCATTCTCACTTTCTTCCTGGTCATCAATCCCTT
AGGGAACTTCTGGCTCATTCTGACAGTTACTTCTGTGGAGCTGGGCGTCT
TGGGTTTGANNNNNNNNNNNNNNNNNNNNNNNNNNNNNNNNNNNNNNNNNNNNNNNNNNNNNNNNNNNNN
NNNNNNNNNNNNNNNNNNNNNNNNNNNNNNNNNNNNNGTGGATGACTCCCCAGCTGACACCTTCACTCGA
CGGTTTCG
CTATGATGTGGCGCTGGTGTCAGCATTAAAGGATCTGGAGGAGGACATCA

```
TGGAGGGATTGAGAGATTGTGGGTTGGAAGACAGCACTTGCACCTCAGGC
TTCAGTGTTATGATCAAGGAATCTTGCGATGGCATGGGCGATGTCAGCGA
GAAGCATGGCGGAGGACCACCGGTTCCCGAGAAGGCTGTACGTTTCTCTT
TCACTATTATGTCTGTCTCTGTCCTGGCAGATGACAAGGAG---------
---------------------GAGGCGGTTACCATCTTTACAGAGCCAAA
GCCAAACTCAGAGCTGTCCTGCAAACCCCTTTGCATCACATTTGTGGATG
AGTCAGATCACGAGACACTCACAGCTGTCCTGGGCCCCGTAGTTGCAGAG
CGTGATGCAATGAAAGAGAGTAGACTCATTCTTTCCATGGGAGGCCTGCC
TCGCTCCTTCCGCTTCCACTTCAGAGGCACAGGTTATGATGAGAAGATGG
TGCGTGAGATGGAGGGCCTAGAGGCCTCAGGCTCTACCTATATCTGCACT
CTGTGCGACTCCAGTCGGGCAGAGGCTTCGCACAACATGGTGCTGCACTC
CGTCACCCGTAGTCACGAAGAGAACCTAGAGCGTTACGAAATATGGAGAA
CCAATCCCTTTTCTGAGTCTGTAGAGGAGCTGCGGGACCGAGTCAAAGGG
GTCTCTGCCAAGCCCTTTATGGAGACCCAGCCAACAATGGATGCTTTGCA
CTGTGACATTGGTAATGCCACTGAGTTCTACAAAATCTTCCAGGATGAGA
TTGGGGAGGTGTACCAAAAGGT---CAAC---CCCCCACGGGAGGAACGG
CGCAGCTGGAGGGCAGCCCTAGATAAACAGCTGAGGAAGAAAATTAAGCT
TAAACCGATAATGAGGATGAATGGGAACTATGCCCGGAAGCTAATGACCA
TGGAGGCTGTGGAGGTGGTGTGTGAGCTGGTGCCATCAGAAGAGAGGAGG
GAGGCCCTGAGGGAGCTCATGAGGCTCTACCTTCAGATGAAACCTGTTTG
GCGTGCCTCCTGCCCAGCCAAGGAATGCCCTGACCAGCTGTGCCGCTACA
GCTTCAACTCCCAGAGCTTTGCTGATCTCCTCTCCTCCACTTTCAAATAT
CGCTACAATGGAAAAATAACCAATTACTTGCACAAGACCCTGGCTCATGT
CCCCGAGATTATTGAGAGAGATGGATCCATAGGAGCTTGGGCCAGTGAGG
GGAATGAGTCAGCAAACAAA------------------------------
--------------------------------------------------
--------------------------------------------------
--------------------------------------------------
--------------------------------------------------
--------------------------------------------------
--------------------------------------------------
--------------------------------------------------
--------------------------------------------------
--------------------------------------------------
--------------------------------------------------
--------------------------------------------------
--------------------------------------------------
--------------------------------------------------
-------------------------CCTCTCGCCACGTTCCTCAAACTGA
CCTCTCTGGGCTTCATCATCGGAATCGGTGTGGTCGGAAACCTCCTGATC
TCCATCCTGCTGGTTAAAGACAAGAGCCTGCACCGCGCGCCCTACTACTT
CCTGCTGGACCTGTGCGCCTCTGACATCCTGCGTTCTGCTATCTGCTTCC
CCTTTGTCTTCACCTCTGTCAAGAATGGATCTGTCTGGACCTACGGCACG
CTCACCTGCAAAGTGATCGCCTTCCTGGGCGTGCTCTCCTGTTTCCACAC
AGCGTTCATGCTCTTTTGTGTCAGTGTGACCCGCTATCTGGCCATCGCAC
ATCACCGTTTCTACACTAAGAGGCTGACCTTCTGGACCTGTCTGGCTGTC
ATCTGCATGGTGTGGACGTTGTCAGTGGCCATGGCTTTCCCGCCGGTGCT
AGACGTAGGGACGTACTCTTTCATCCAGGAGGAGGACCAGTGCACATTCC
AGCACCATTCCTTCAGGGCGAACGACTCCCTGGGCTTCATGCTCCTGCTG
GCACTCATCCTCCTGGCCACGCAGCTGGTTTACCTCAAGCTCATCTTCTT
CGTCCATGACCGTCGGAAGATGAAGCCTGTCCAGTTCGTGCCTGCTGTCA
GCCAGAACTGGACCTTCCACGGGCCAGGCGCCAGCGGGCAGGCGGCGGCC
AACTGGCTGGCTGGATTTGGTAGAGGTCCCACACCTCCTACTTTACTGGG
```

CATCCGGCAGAACAGCAATGCAGCAGGCCGCAGGCGTCTACTGGTGCTGG
ATGAATTCAAAACAGAGAAGAGGATTAGCAGGATGTTCTACATCATGACG
TTTTTCTTCCTGGCACTGTGGGGGCCATATCTGGTAGCCTGCTACTGGAG
GGTGTTTGCAAGGGGTCCTGTAGTCCCCGTGGGCTACCTGACAGCAGCCG
TGTGGATGAGCTTTGCCCAGGCAGGGGTCAATCCTTTCATCTGCATCTTC
TCCAACAGGGAGGCCAAGTCTCGCTTTCACCCTGGCGTGGGGGCTGCGCC
TGGCACGGAGC---GCAGCGTCCCACTCGGCAACAGCTTGCTATCCCCGC
AGCAAACTGAGGAGCCCACTGTCGCCACCCCCCCGCAGCGATGGTTTGTC
ACCC---CTGCCAACAACCGACTGGACTTTGCTGCCTCGGCATACGACGC
GGCT---------GATTTTGCCGGTAACGCGGCCACCTTGCTGTCCTACG
CAGCGGCCGGAGTGAAGGCTC------TTCCCCTGCCGGCCGCAGGATGC
TCCAACCGGCCTCTTGGCTATTACGCTGACCCGTCGG---GCTGG---GG
AGGGCGCACGCCGCCGCAGTACTGTGGTGTAAATAGTAAATCCAGCTCGG
TCTTTTCCTGCTGGCCCGCTAACTCTATCGGGGGCAGAGCAGGCG---CC
---CACTACCTGG---------CCGAGGA---GGGA---GACTC---CGT
CCCCACGGAGAGGTCACCG---AT---CAGCGGCTCCGAGGAG---ACCA
AACCCAAGGACAT------ATCAGA---GTCGAGCTGGATAGAG---ACA
CCTTCCTCCATTAAGTCCATCGATTCAAGCGATTCTGGGATCTTTG---A
ACAGGCCAAAAGGAGAAGAATCTCACCTTCTGCCACGCCG---------G
TTTCAGAGGCAGTGTCCCCGTNNNNNNNNNNNCATCACTCAACAGGCGAAGTCACAGAGAG
AGAAGTGGCGTTGGGGATAAATCCTTTCGCGGACGGGATGGGCGCCTTCA
AAATAAACCACAGCTCCCATGATATTGGCTCCGG---ACAAACGGCGTTT
TCCTCTCAGGCG---CCCGGCTAC---GCAGCGGCCGCCCTGGGA---CA
CCATCA------CCACCCGACCCACGTTGGCTCT---TACTCCACGGCGG
CTTTCAACTCCACCAGGGACTTTCTCTTCAGAAATCGGGGTTTTGGAGAC
GCCACCGG------------GGCGCAGCACAGTTTGTTCGCCTC------
---CGGAAGTTT---C------GCAGGGCCACATGGACACTCAGATGCAG
CGGGGCACCTGCTCTTCCCAGGGCTCCACGAG---CAAGCAGCGAGCCAT
GCGTCTTCCAACGTGGTCAACAGCCAGATGCGACTGGGCTTTTCGGGGGA
CATGTACGGTCGGGCCGATCAGTACGGCCACGTTACAAGCCCGAGGT---
CCGACCACTACGCTTCGACCCAGTTGCACGGTTATGGCCCCATGAACATG
AATATGGCCGCA---CACCACGGAGCAGGGGCCTTCTTTCGATACATGAG
GCAGCCGATCAAACAAGAGCTCATCTGCAAGTGGATCGAGCCGGAGCAGY
TGACAAATCCCAAAAAGTCTTGCAACAAAACTTTTAGCACCATGCATGAG
CTTGTGACCCACCTGACGGTGGAGCATGTGGGGGGACCAGAGCAGACCAA
CCACATCTGTTTCTGGGAGGAGTGCGCCAGAGAAGGAAAACCATTCAAAG
CCAAATACAAACTTGTAAATCATATCAGAGTACACACCGGAGAAAAACCC
TTTCCGTGTCCGTTCCCAGGCTGTGGCAAA
>Barbourisia rufa
AGCCTTCTCATCCGAGCAGAGCTAAGCCAGCCAGGTGCCCTTTTAGGGGA
CGATCAAATCTACAACGTCATTGTTACAGCGCACGCCTTTGTAATAATTT
TCTTTATAGTAATACCAATTATGATTGGGGGGTTTGGAAATTGATTAATT
CCCCTAATGATTGGAGCCCCAGACATAGCATTCCCTCGAATAAATAATAT
GAGTTTTTGGCTCCTACCTCCATCCTTCTTACTGCTCCTGGCCTCTTCTG
GGGTAGAAGCAGGCGCTGGGACAGGATGAACAGTTTATCCGCCACTTGCG
GGCAATCTTGCCCACGCAGGAGCCTCAGTAGACCTAACTATTTTCTCCCT
CCACTTAGCAGGAGTCTCTTCAATTCTAGGGGCTATTAACTTTATTACAA
CTATTATTAACATAAAACCCCCAGCAATTTCCCAATATCAAACCCCCCTG
TTTGTGTGATCTGTACTAATTACAGCCGTTCTTCTCCTCCTCTCCCTACC
CGTTCTTGCAGCCGGCATCACCATACTATTAACCGACCGTAACCTAAACA
CGACCTTCTTTGACCCCTCAGGAGGAGGTGATCCCATCCTATATCAACAC
CTATTTTGATTCTTCGGCCACCCA--------------------------

------------TTTCTAGAGAGGAACCTTCACCCATCTAACTGCCTTGG
AATGCTGTTGCTGTCTGACGCCCACCAGTGTACCAAGCTATCAGAGCTCT
CCTGGGGCATGTGCCTCAGCAACTTTCCTGCTATTTGTAAGACAGAGGAC
TTTCTCCAACTGCCCAAAGACATGGTGGTGCAGCTTCTGTCCCATGAGGA
GCTGGAGACAGAAGATGAGAGACTGGTTTATGAAGCTGCCCTTAACTGGG
TCAACTATGACCTGGAAAGCAGGCACTGCCAYCTGCCAGAGCTGCTGAGA
ACGGTCCGCCTGGCCCTGCTACCCGCTATATTCCTCATGGAGAATGTCTC
CACAGAAGAGCTGATCAATGCCCAGGCCAAGAGCAAGGAGCTGGTGGATG
AGGCCATCCGCTGTAAGCTGAAGATCTTGCAGAATGATGGTGTGGTTAAC
AGCCCCTGTGCTCGACCGAGAAAAACCAGCCATGCCCTCTTTCTGCTGGG
AGGCCAGACCTTCATGTGTGACAAGCTGTATCTGGTGGATCAGAAGGCCA
AAGAGATCATCCCCAAGGCTGACATCCCCAGCCCCAGGAAGGAGTTCAGC
GCCTGCGCCATCGGCTGTAAGGTCTACATCACAGGTGGGA--GAGGCTC-
AGAGAATGGTGTGTCCAAAGATGTGTGGGTCTATGACACCGTCCATGAGG
AATGGTCCAAGGCAGCACCCATGCTCATTGCCAGGTTTGGTCATGGCTCT
GCTGAGCTGAAACACTGCCTCTATGTGGTAGGAGGTCACACAGCAGCAAC
TGGCTGTCTCCCAGCCTCTCCCTCCGGATGAATACATTGTCGTGTTCAGT
CGTTCAACAACAAGGCTAATTCTGAACGAAGCGGAACTAATCATGGCGCT
GGCCCAGGAATTTCAGATGAGAGTGGTTACAGTATCCCTGGAGGAACAAA
CTTTCGCCAGCATCGTCCAGGTGATCAGCAGGGCCTCCATGTTAGTCAGT
ATGCATGGAGCTCAGCTTGTCACCTCACTCTTCCTCCCCAGAGGAGCTGC
TGTGGTGGAGCTGTTCCCCTATGCTGTGAACCCAGAACAGTACACCCCAT
ATAAAACCCTCGCCTCCCTACCAGGCATGGACCTTCAATATGTTTCCTGG
AGGAACACTATGGAGGAGAACACCATCACCCACCCAGACCGACCCTGGGA
ACAAGGAGGTATCTCCCATTTGGAAAAGGATGAGCAAGAGCGAATACTGG
CGAGCAAGGATGTCCCCAGGCACCTGTGCTGCCGCGACCCAGAGTGGCTC
TTCCGAATCTATCAGGACACTTTGGTGGACATCCCTTCATTCTTGGAAGT
CCTCAA---AGAGGGCCTGAGG---GCTAGGCCCAGCTTGAGGAA---GG
CCAAGCCAGCCAGCACGGTTCATCCAGGCCGGGTAAGAGAACCCCAGTGC
CATACCTCAGTCCAAGCCACCAACGAGGCTAAACTCACGGTTTCCTGGCA
GATCCCGTGGAATCTGAAATACCTGAAGGTGAGAGAAGTGAAGTATGAGG
TGTGGATCCAGAAGAGGGATACCAGCAAGGGAACACTGGAGGATCAAATC
ATCCAGGCAAACCCTGCACTGGAAGCCTTTGGTAACGCCAAAACAGTGAG
GAATGACAACTCATCCCGTTTTGGAAAATTCATCCGAATTCACTTCGGAA
CCAGTGGCAAGCTGGCGTCTGCTGACATTGAGACTTACCTGCTAGAGAAG
TCGCGTGTCACCTTCCAGCTCAAGTCTGAGAGGAACTACCACATCTTCTT
CCAGATCTTGTCCAATCAGAAGCCAGAGCTGCTGGACATGCTGTTGATTA
CCAACAACCCATATGACTACTCCTACATCTCCCAAGGAGAGGTAACAGTA
CAATCCATCAACGACTCAGAGGAACTGATGGCCACTGACAATGCCTTCAA
TGTGCTTGGCTTCACTCAAGAGGAGAAGATGGGAGTCTATAAGCTGACCG
GTGCCATTATGCACTACGGCAACATGAAATTTAAGCAGAAGCAGCGTGAG
GAGCAGGCTGAGCCTGATGGAACGGAGGCTGCTGATAAGACAGCTTACCT
AATGGGGCTGAACTCTGCAGACCTTATCAAAGGGCTGTGCCATCCCAGAG
TCAAGGTAGGAAATGAATATGTCACCAAAGGCCAAGGTGTAGACCAAGTC
TACAACCCCAACAAGGAGGCCTTCAAGTGCGAGGAGTGTGGCAAGCACTA
CAACACCAAGCTGGGATACAAGCGCCATGTGGCCATGCACTCTGCCACGG
CAGGGGACCTTACCTGTAAAGTGTGCATGCAGAGCTATGAGAGCACGCCG
GTGCTCCTGGAGCACCTCAAGAGCCACTCAGGGAAGTCCTCAGGTGGCAC
CAAGGAGAAAAAACACCCATGCGACCACTGCGACCGTCGCTTCTACACTC
GGAAGGATGTAAGACGGCACATGGTGGTCCACACGGGCCGAAAGGACTTC
CTGTGCCAGTACTGTGCCCAGCGCTTTGGCAGGAAGGACCACCTGACACG

GCACGTAAAGAAGAGCCACTCGCAGGAGCTGCTGAAGATCAAGACAGAGC
CTCCGGATATGTTAGGTCTTTTAGGTTCTGGCTCGCCGCCTTGCTCCGTC
AAGGAGGAGCTTAGCCCCATGATGTGCAGCATGGGTCCCAACAAAGACCC
CATGATGGGCAAACCGTTCCCCAGCGGAACCCCCTTCCCGATGGGCATGT
ACAACCCCCACCAC------CTCCAGGCCATGTCCAATTCTGGGGTGGGT
CACCCC------CACCCCTCCCTGATGCCTAGTTCCCTGTCTGCAGCTAT
GGGCATGGGCTGTCACATGGAATATCTCATCTACGCCTCTTTCTCATTCA
TGGGATGTTTACAAATCAGTGATGGGTCAAACATTGTGAACTTGCTGGCT
AGTAATTCTCCGAGCGTTTCATACGCTCTGACCCAGCAGAAATACTTCAG
TAACTACAGTCCCGTGATTGGGTTTTACATTTACGAGCCCATTGAGTACT
GGAACTCCACGGTGCAGGAGCATCTGAAGACACTGAGTCATGGCTTCAAC
AAGATCTCCTGGATGGACAACTTCTTCCACTACCTGAGGGTGGTGAACGT
GAGCGCGTCGAACAAGAGCGACTTCATCACCATCCTCAAGGGCTCCTTCC
TGCGCAGCCCAGAGTACCAGCACTTCACTGAAGACATCATCTTCTCCAAG
A---ACCGTGAAAGTG------ATGAGTATGACATTATTGCCTCACGCAT
GTACCTGGTGGCGCGGACCACGGAGAAAAAGCGCGAGGAGGTGGTGGAGC
TTCTGGAGAAGCTGCGTCCGCTGATGCTGATCAACAGCATCAAATTCATC
GCCTTCAACCCCACCTTTGTTTTCATGGACCGCTACAGCGCCTCTGTCAT
CTCACCCATCCTGACCTCAGGCTTCAGCGTGCTCACCATCCTCATYCTCA
CTTTTTTCCTGGTCATCAAYCCCTTGGGGAACTTCTGGCTCATCCTGATG
GTTACTTCTGTGGAGCTGGGCGTCTTGGGTTTAATGGGCTATCACCTATT
CGAGTGGCAGCCGGCCCTCAAGAATGTGTCTGCATCCTGTCATGTGGGCA
TTATTAATGGGCTCTCTGGATGGACTGCCTCAGTGGATGATTCCCCAGCT
GACACCATCACTCGGCGATTTCGCTATGATGTGGCCCTGGTGTCAGCCTT
AAAGGATCTGGAGGAGGACATCATGGAGGGACTGAGAGAGTGTGGGCTGG
AAGACAGTGCTTGCACCTCAGGCTTCAGTGTTTCAATCAAGGAATCTTGT
GACGGCATGGGAGATGTCAGCGAGAAGCATGGCGGAGGGCCAATGGTCCC
TGAGAAGGCTGTGCGTTTCTCTTTTACTATTATGTCTGTCTCTGTCTTGG
CAGACGGAGAGGAG------------------------------AAGGCG
GTTACCATCTTCAGGGAGCCAAAGCCCAACTCTGAACTGTCCTGTAAGCC
CCTATGCCTGATGTTTGTGGATGAGTCAGACCACGAGACACTCACTGCTG
TCCTGGGGCCTGTAGTTGCAGAGCGTAATGCAATGAAGCAGAGTCGACTC
ATCCTATCTATGGGTGGCCTGCCTCGCTCCTTCCGCTTCGAATTCAGAGG
CACAGGATATGATGAGAAGATGGTGCGTGAGATGGAGGGCCTGGAGGCCT
CAGGTTCCACCTACATCTGCACTCTGTGTGACTCCAGTCGGGCAGAGGCC
TCTCACAACATGGTGCTGCACTCCATCACCCGCAGTCACGATGAGAACCT
GGAGCGTTATGAAATATGGAGGACCAATCCTTTTTCTGAGTCTACCGAGG
AGCTGCGAGACCGGGTCAAAGGGGTCTCCGCCAAGCCCTTTATGGAGACC
CATCCCACTCTGGATGCATTACACTGTGACATTGGTAATGCCACTGAGTT
CTACAAAATCTTCCAGGATGAGATTGGGGAGGTGTTCCAAAAGGT---CA
AC---CCCACCCGGGAGGAACGGCGGAGATGGAGGGCAGCCCTAGACAAG
CAGCTGAGGAAGAAGATGAAGCTTAAACCGGTGATGAGGATGAATGGGAA
CTTTGCCCGGAGGCTAATGTCCCAGGAGACTGTGGAGGTGGTGTGTGAGC
TTGTGCCCTCAGAAGAGAGGAGGGAGGCCCTGAGGGAGCTTATGGGGCTC
TACCTCCAGATGAAGCCTGTGTGGCGCACCACCTGCCCAGCCAAGGAGTG
CCCTGACCAGCTGTGCCGCTACAGCTTTAACTCCCAGCGCTTTGCCGATC
TCCTCTCCACTACCTTCAAATATAGGTACAATGGAAAAATCACCAATTAC
CTGCACAAGACCCTGGCCCATGTCCCTGAAATCATAGAGAGAGATGGCTC
CATAGGAGCCTGGGCCAGTGAGGGGAATGAGTCAGCAAACAAATCGTACA
CCATTGAGATGGGCCTCAAGGGGCCCCAATGGAAGGAGAGCCCGCAGCCT
TTCTCCTGCTCCATTGAAGACCCCACGAAACAGACCAAGTTCAAGGGCAT
CAAGACCTACATTTCGTACCGGGTCACGCCGAGCCACTCAGGGCGTCCCG
TCTACAGGCGCTACAAACACTTTGACTGGCTGTACAACCGCTTGCTGCAC

AAGTTCACTGTGATCTCGGTGCCCCACCTGCCTGAGAAGCAGGCCACGGG
GCGCTTCGAGGAGGACTTCATCGAGAAGCGTAAAAGACGACTGATCCTGT
GGATGAACCACATGACCAGTCACCCAGTCCTCTCCCAGTATGAGGGATTT
GAGCACTTCCTGATGTGTGCTGATGACAAGCAATGGAAACTGGGTAAGAG
GCGGGCGGAGAAGGACGAGATGGTGGGCGCCCACTTCATGCTGACCTTCC
AGATTCCTAACGAGCACCAGGACCTCCAGGACGTCGAGGAGCGAGTCGAC
TCCTTCAAGTCCTTCGCTAAGAAAATGGACGACAGCGTCATGCAGCTCAC
ACACGTTGCCTCTGAGCTGGTGCGTAAGCACCTGGGTGGGTTCAGGAAGG
AGTTCCAGCGGCTGGGAAATGCCTTCCAGTCTATCAGCCAGGCATTCATG
CTGGACCCTCCCCACAGCTCAGATGCTCTCAACAACGCCATCTCCCACTC
TCTCGCCACGTTCCTCAAACTGA-CTCTCTGGGTTTCATCATTGGAGTCG
GCGTGGTCGGAAACCTCCTGATCTCCATCCTGCTGGTCAAAGACAAGAGC
CTGCACCGCGCGCCCTACTACTTCCTGCTGGACCTGTGCGCCTCCGACAT
CCTGCGCTCCGCCATCTGCTTCCCCTTTGTCTTCACCTCTGTCAAGAATG
GATCCGCCTGGACCTATGGCACGCTCACCTGCAAAGTGATCGCCTTCCTG
GGCGTGCTCTCCTGTTTCCACACGGCGTTCATGCTATTCTGCGTCAGCGT
CACCCGCTACCTGGCCATCGCGCATCACCGTTTCTACACCAAGAGGCTGA
CCTTCTGGACCTGTCTGGCTGTCATCTGCATGGTGTGGACGTTGTCGGTG
GCCATGGCGTTCCCCCCGGTGCTGGACGTAGGGACGTACTCCTTCATCCG
GGAGGAGGACCAGTGCACATTCCAGCACCATTCCTTCAGGGCCAATGACT
CGCTGGGCTTCATGCTCCTGCTGGCGCTCATCCTCCTGGCCACACAACTG
GTTTACCTCAAGCTCATCTTCTTCGTCCACGACCGTCGGAAGATGAAGCC
CGTCCAGTTTGTGCCCGCCGTCAGCCAGAACTGGACCTTCCACGGGCCAG
GCGCCAGCGGGCAGGCGGCGGCAAACTGGCTCACCGGATTTGGGAGAGGT
CCCACCCCGCCTACCTTGCTGGGCATCCGGCAGAACAGCAACGCTGCGGG
CCGCAGGCGTCTACTGGTGTTGGATGAGTTCAAAACAGAGAAGAGGATTA
GTAGGATGTTCTACATCATGACGTTTTTCTTCCTGGCGCTGTGGGGGCCC
TATCTGGTAGCCTGCTACTGGCGGGTGTTTGCGAGGGGCCCTGTAGTCCC
CGGGGGCTACCTGACGGCAGCCGTGTGGATGAGCTTCGCCCAGGCTGGGG
TCAATCCCTTCATCTGCATCTNNNNNNNNNNNNNNGCCAAATCTCGCTTTCACCCTGGCGTGGG
GACTGGTCCTGGCACGGAGC---GCAGCGTCCCACTCGGCAACAGCTTGC
TATCCCCGCAGCAAGCCGAGGATCCCACCGTTGCCACCCCCCCACAGCGA
TGGTTTGTCACCC---CTGCCAACAACCGACTGGACTTTGCTGCCTCGGC
ATACGACGCCGCC---------GATTTCGCCGGTAACGCGGCCACCTTGC
TGTCCTACGCAGCGGCCGGTGTGAAGGCTC------TTCCCCTGCCGGCT
GCAGGCTGCTCCAACCGGCCTCTTGGCTATTACGCAGACCCGTCGG---G
CTGG---GGAGGACGCACACCGCCGCAGTACTGTAGTGTCAATGGTAAAT
CAAGCTCGGTGTTTCCCTGCTGGCCCGCTAACTCTGTCGGCGGCAGAGCA
GGCA---CC---AACTACATGG---------CCGAGGA---TGGA---GA
CGC---CATCCCCACGGAGAGGTCCCCG---AT---CGGCGGCTCTGAGG
AG---ACCAAACCCAAAGACCT------GTCAGA---GTCGAGCTGGATA
GAN---ACGCCGTCCTCCATTAAGTCAATTGATTCAAGCGATTCTGGTATC
TTTG---AACAAGCCAAAAGGAGGAGNATCTCGCCGTCTGCCACGCCG---

GTCTCAGAGACTGTGTCCCCGTTAAAATCTNNNNNNNNNNNNNNNNNNNNNNNNNNNNNNNNNNNNNNNN
NNNNNNNNNNNNNNNNNNNNNNNNNNNNNNNNNNNNNNNNNNNNNNNNNNNNNNNNNNNNNNNNNNNNNN
NNNNNN---NGAGACGGCGTT
TTCCTCCCAGGCG---CAAGGCTAC---GCAGCGGCCGCCCTGGGA---C
ACCATCA------CCACCCGACCCACGTTGGCTCT---TACTCCACGGCG
GCGTTCAACTCCACCCGGGACTTTCTCTTCAGAAATCGGGGTTTCGGAGA
CGCCACCGG------------GGCGCAGCACAGTTTGTTCGCCTC-----
----CGGAAGTTT---C------GCAGGGCCACATGGACACTCAGATGCG
GCGGGGCACCTGCTCTTCCCGGGGCTCCACGAG---CAGGCGGCGAGCCA

CGCGTCTTCCAACGTGGTCAACGGCCAGATGCGACTGGGCTTCTCGGGGG
ACATGTACGGTCGGGCCGATCAGTACGGCCACGTTACGAGCCCGAGGT--
-CCGACCACTACGCATCGACCCAGTTGCACGGCTATGGCCCCATGAACAT
GAATATGGCCGCG---CACCACGGAGCAGGGGCCTTCTTTCGATACATGA
GGCAGCCGATCAAGCAAGAGCTCATCTGCAAGTGGATCGAGCCGGAGCAG
CTGACGAATCCCAAAAAGTCGTGCAACAAAACTTTTAGTACGATGCACGA
GCTGGTGACCCATCTGACGGTGGAGCATGTGGGGGGGCCGGAGCAGTCCA
ACCACATCTGCTTCTGGGAAGACTGCTCCAGAGAAGGGAAACCATTCAAA
GCCAAATACAAACTTGTGAATCATATCAGAGTACACACCGGAGAAAAGCC
CTTCCCGTGTCNNNNNNNNNNNNNNNNNNNN
>Bathyclupea gracilis

-------------------------GGATGAATACATTGTTGTGTTTAGT
CGTTCAACAACGAGGCTGATACTAAATGAAGCGGAGCTAATCATGGCGCT
GGCCCAGGAGTTCCAGATGAGAGTGGTCACGGTATCCCTGGAGGAACAGC
CTTTCCCCAGTATCGTCCAGGTGATCAGCGGTGCTTCCATGTTAGTCAGT
ATGCATGGAGCTCAGCTTATCACCTCACTCTTCCTCCCCAGAGGAGCTGC
TGTGGTGGAGCTGTTCCCCTTTGCTGTGAACCCAGAGCAGTACACCCCGT
ATAAAACCCTTGCCTCCCTTCCAGGCATGGACCTTCACTATGTCTCCTGG
AGGAACACTAAGGAGGAGAACACCATCACCCACCCAGACAGACCCTGGGA
ACAAGGGGGCATTGCTCACTTGGAGAAGGAGGAGCAAGAGCGAATACTGG
AGAGCAAAGATGTCCCCAGGCACCTGTGCTGCCGCAACCCAGAGTGGCTC

TTCCGGATCTACCAGGACACTTTGGTGGACATCCCTTCCTTCCTGGAAGT
CCTCAA---AGAGGGCATGAAG---ACAAAGCCCAGCTTGAAGAA---GT
CAAAGCCGGCCAGCACAGTCCATCCGGGCCGGGTCAGAGAACCCCAATGT
CAGACCTCAGTACAAACCACTAATGAGGCTAAACTCACAGTGTCCTGGCA
GATCCCGTGGAATCTGAAATACCTGAAGGTGAGAGAGG------------
-----------AAAAGAGACACCAGCAAGGGGACACTGGAGGATCAAATC
ATCCAGGCGAACCCTGCGCTGGAGGCCTTCGGCAACGCCAAAACATTGAG
AAACGACAACTCGTCTCGTTTTGGAAAATTCATCCGAATTCACTTTGGTA
CGAGCGGCAAGCTGTCGTCTGCTGACGTTGAGACGTACCTGCTGGAGAAG
TCACGTGTCACCTTTCAGCTCAAGGCTGAGCGGAACTACCACATCTTCTA
CCAGATCCTGTCCAATCAGAAGCCAGAGCTCCTGGACATGCTGCTGATCA
CCAACAACCCGTACGACTACTCCTACATCTCCCAAGGAGAGGTAACGGTC
GCCTCCATCAATGACTCGGAGGAGCTGATGGCCACCGACAGCGCCTTTGA
TGTGCTCGGCTTCACTCCAGACGAGAAGACGGGCGTCTATAAGCTGACCG
GCGCCATCATGCACTACGGCAACATGAAGTTCAAACAGAAGCAGCGTGAG
GAGCAGGCGGAGCCGGACGGGACGGAGGCTGCTGATAAAACAGCTTACCT
AATGGGGCTGAACTCTGCTGACCTCATCAAAGGCCTGTGCCATCCCAGAG
TCAAGGTAGGAAATGAATACGTCACCAAAGGCCAAAGTGTGGACCAAGTC
TACTAC--

----------------------TATCTCATCTACGCCTCTTTCTCATTCA
TGGGATGTTTACAAATCAGTGATGGGTCAAATATCGTGAACCTGCTGGCT
AGTAACTCTCCGAGTGTTTCGTATGCTCTGACCCAGCAGAAATACTTCAG
TAACTACAGTCCCGTAATTGGGTTTTACATTTATGAGCCCATAGAGTACT
GGAACTCAACGGTGCAGGAGCACCTGAAGACTCTGAGTCATGGCTTCAAC
AAGATCTCCTGGATGGACAACTTTTTCCACTACCTGCGGGTGGTGAATGT
GAGTGCRTCAACCAAGAGCGACTTCATCACCATCCTCAAGGGCTCCTTCC
TGCGCAGCCCGGAGTACCAGCACTTCACTGAGGACATCATATTCTCCAAG
A---ACCGCGAGACTG------ATGAGTACGACATTATCGCCTCACGGAT
GTACTTGGTGGCGCGGACGACAGAGAAGAAGCGTGAAGAGGTGGTGGAGC
TTCTGGAGAAGCTTCGTCCGTTGATGCTGATCAACAGCATCAAGTTCATT
GCCTTCAATCCTACGTTTGTGTTCATGGACCGCTACAGCTCCTCTGTCAT
CTCGCCCATCCTGACCTCAGGCTTCAGCGTACTCACAATCCTCATCCTCA
CTTTCTTCCTGGTCATTAACCCCTTGGGGAACTTCTGGCTCATCCTCACT
GTAACGTCCGTGGAGCTGGGCGTCTTGGGTTTGATG--------------

---TCGTACA
CCATCGACATGGGCTCCTTGAGGCCCCGGTGGAAGGAGAGCCCACAGCCT
TTCTCCTGCTCCATTGAAGACCCCACAATACAGACAAAGTTCAAGGGCAT
CAAGACGTACATTTCGTACCGGGTCACACCGAGCCACACAGGGCGTCCCG
TCTACAGGCGCTACAAACACTTTGACTGGCTGTACAACCGCTTACTGCAC
AAGTTCACTGTGATCTCCGTGCCTCACCTGCCTGAGAAGCAGGCCACGGG
GCGATTTGAGGAAGACTTCATCGAGAAGCGTAAGAGGCGACTGATACTGT
GGATGAACCACATGACCAGTCACCCAGTCCTCTCCCAGTATGAAGGCTTT
GAGCACTTTCTGATGTGCGCTGATGACAAGCAGTGGAAACTGGGCAAGAG
ACGGGCGGAGAAGGACGAGATGGTGGGTGCCCATTTCATGCTGACCCTCC
AGATCCCTAATGAGCACCAGGACCTTCAGGATGTAGAGGAGCGGGTCGAC
TCCTTCAAGGCCTTTGCTAAGAAAATGGATGACAGCGTGATGCAGCTCAC
ACATGTTGCCTCGGAGCTGGTGCGTAAGCACCTGGGTGGGTTCAGGAAGG
AGTTCCAGCGGCTGGGAAATGCCTTCCAGTCTATCAGCCAGGCCTTCATG
CTGGACCCTCCACACAGCTCAGAAACCTTCAACAACGCCATCTCCCAC--
-------------------CTGACCTCTCTGGGTTTCATCATTGGCGTCG
GTGTGGTTGGAAACCTCCTGATCTCCATCCTGCTGGTCAAAGACAAGAGC
CTGCACCGAGCGCCCTACTATTTCCTGCTGGACCTGTGCGCCTCTGATAT
CCTGCGCTCCGCCATCTGCTTCCCCTTTGTCTTCACCTCGGTCAAGAATG
GATCTGCCTGGACCTATGGCACGCTGACCTGCAAAGTGATCGCCTTCCTG
GGTGTGCTCTCCTGTTTCCACACGGCGTTTATGCTGTTCTGTGTCAGTGT
CACGCGCTACCTGGCCATCGCACACCACCGTTTCTACACCAAGAGGCTGA
CCTTCTGGACCTGTCTAGCTGTCATCTGCATGGTGTGGACGTTGTCAGTG
GCCATGGCGTTCCCGCCAGTGCTAGACGTAGGGACGTACTCTTTTATCCG
AGAGGAGGACCAGTGCACATTCCAGCACCGCTCCTTCAGGGCGAATGATT
CGCTGGGCTTCATGCTCCTGCTGGCGCTCATCCTCCTGGCCACACAGCTG
GTTTACCTCAAGCTCATTTTCTTCGTCCACGACCGTCGAAAGATGAAGCC
TGTCCAGTTCGTGCCTGCTGTCAGCCAGAACTGGACCTTCCACGGGCCAG
GCGCCAGCGGGCAGGCGGCAGCCAACTGGCTGGCCGGATTTGGTCGAGGC
CCCACCCCGCCTACTTTGCTGGGCATCCGGCAGAACAGCAACGCAGCGGG
CCGCAGGCGTCTACTGGTATTGGATGAGTTCAAAACAGAGAAGAGGATTA
GTAGGATGTTCTACATCATGACGTTTTTCTTCCTGGCTCTGTGGGGGCCC
TATCTGGTCGCCTGTTACTGGCGGGTGTTTGCAAGGGGCCCTGTGGTCCC

TGGGGGCTACCTGACGGCAGCTGTGTGGATGAGCTTTGCCCAGGCTGGGG
TCAATCCGT-CATCTGC------------------GCCAAATCTCGCTTT
CACCCTGGCATGGGGACTGGTCCTGGCACGGAGC---GCAGCGTCCCACT
CGGCAACAGCTTGCTATCCCCGCAGCAAACCGAGGAGCCCACTGTTGCCA
CCCCCCCGCAGCGATGGTTTGTCACCC---CTGCCAACAACCGACTGGAC
TTTGCTGCCTCGGCATACGACGCCGCT---------GATTTCGCCGGTAA
CGCGGCCACCTTGCTGTCCTACGCAGCGGCCGGAGTGAAGGCTC------
TTCCCCTGCCGACTGCAGGCTGCTCCAACCGGCCTCTTGGCTATTACGCA
GACCCGTCAG---GCTGG---GGAGGACGCACGCCGCCGCAGTACTGTGG
TGTAAATAGCAAATCCAGCTCGGTCTTTTCCTGCTGGCCCGCTAACTCTA
TCGGTGGCAGAGCAGGCA---CC---AACTACCTGG---------CGGAG
GA---GGGA---GACTC---CATCCCGACAGAGAGGTCACCG---AT---
CGGCGGCTCGGAGGAG---ACCAAACCCAAAGACCTGAC---ATCAGA--
-GTCGAGCTGGATAGAG---ACGCCGTCCTCCATTAAGTCCATTGATTCA
AGCGATTCTGGTATCTTTG---AACAGGCCAAAAGGAGAAGAATCTCACC
TTCTGCCACGCCG---------GTTTCAGAGACAGTGTCCCCGTTGAAAT
CGGAGCATCACTCAACAGGCGAAGTCACAGAGAGAGAAGTGGCGTTGGGG
ATAAATCCGTTCGCGGATGGGATGGGCGCCTTCAAAATAAACCACAGCTC
CCACGATATCGGCTCCGG---ACAAACGGCGTTTTCCTCCCAGGCG---C
CCGGCTAC---GCAGCAGCCGCCCTGGGG---CACCATCA------CCAC
CCGACCCACGTTGGCTCT---TACTCCACGGCGGCTTTCAACTCCACCAG
GGACTTTCTCTTCAGAAATCGGGGTTTCGGGACGCCACCGG--------
----GGCGCAGCACAGTTTGTTCGCCTC---------CGGAAGTTT---C
------GCAGGGCCACATGGACACTCAGATGCAGCGGGGCACCTGCTCTT
CCCGGGGCTCCACGAG---CAAGCGGCGAGCCATGCGTCTTCCAACGTGG
TCAACAGCCAGATGCGGCTGGGCTTCTCGGGGGACATGTACGGACGGGCC
GACCAGTACGGCCACGTTACAAGCCCGCGGT---CTGACCACTACGCTTC
GACCCAGCTGCACGGCTATGGCCCCATGAACATGAATATGGCCGCA---C
ACCACGGAGCTGGGGCCTTCTTTCGATACATGAGGCAGCCGATCAAACAA
GAGCTCATCTGCAAGTGGATCGAGCCGGAGCAGCTGACGAATCCCAAAAA
GTCGTGCAACAAAACTTTTAGCACGATGCACGAGCTGGTGACCCATCTGA
CGGTGGAGCATGTAGGGGGACCGGAGCAGACCAACCACATCTGCTTCTGG
GAGGACTGCGCCAGAGAAGGAAAGCCATTCAAAGCCAAATACAAACTTGT
AAATCATATCAGAGTACACACCGGAGAGAAGCCCTTTCCATGTCCGTTCC
CCGGCTGTGGCAAG
>Bathylaco nigricans
AGCCTCCTAATTCGAGCCGAACTAAGCCAGCCCGGGGCCCTTCTAGGCGA
CGACCAAATTTATAACGTCATCGTCACAGCACACGCATTCGTAATAATCT
TCTTTATAGTAATACCCATCATGATCGGGGGCTTTGGAAACTGGCTGCTC
CCCCTAATACTGGGGGCCCCCGATATAGCATTCCCCCGAATAAATAACAT
AAGCTTCTGGCTTCTTCCCCCCTCACTTCTCCTCCTCCTGTCGTCCTCAG
GAGTTGAAGCCGGGGCAGGAACAGGCTGAACTGTCTACCCCCCCATTAGCC
GGTAACCTGGCCCACGCCGGCGCCTCCGTAGATCTCGCTATCTTCTCCCT
CCACCTAGCAGGCGTATCCTCAATCCTGGGCTCAATTAATTTTATTACTA
CAATCACCAACATGAAACCCCCAGCCATCTCCCAGTACCAAACACCTCTG
TTTGTGTGATCTCTGCTCGTCACAACTGTCCTGCTTCTCCTGTCACTTCC
AGTCCTGGCCGCAGCTATTACCATACTCCTCACAGATCGTAACCTCAACA
CAACATTCTTTGACCCAGCCGGAGGAGGGGACCCCATCCTGTACCAACAC
CTATTCTGATTCTTTGGCCACCCAGAAGTCTACATCTTAATCTTACCCGG
ATTTGGCATGATCTCACATGTCGTCGCCTACTACGCCGGTAAAAAAGAGC
CGTTCGGCTACATAGGCATGGTCTGAGCCATGATGGCTATCGGGCTCCTG
GGGTTTATTGTCTGAGCCCACCACATGTTTACAGTCGGATAGACGTAGA
CACTCGTGCCTA--------------------------------------

-------------------------GGACGAGTACATCGTGGTCTTCAGC
CGCTCCGTCAACCGGCTCATCCTCAACGAGGCTGAGTTGATCCTGGCTCT
GGCGCAAGAGTTCCAGATGAGGGCCGTCACGGTGTCCCTGGAGGAGCAGT
CGTTTGCCAGCATCGTTCAGGTGATCAGCGGGGCGTCCATGCTGGTCAGC
ATCCACGGAGCCCAACTGATCAGTTCCCTGTTCCTGCCACGCGGCGCTGC
CGTGGTGGAGCTCTTCCCCTACGCCGTCAACCCAGAGCAGTACACTCCCT
ACAAAACTCTGGCTTTGTTGCCAGGCATGGACCTCCAGTATGTGGCTTGG
AGGAACACCATGGAGCAGAACTCGGTGGCTTACCCTGAGCGCGCCTGGGA
CCAGGGTGGCATCGCCCACCTGGAGAAGGAGGAACAGGAGCGCATCCTGG
CGAGCGAGGAGGTGCCGCGGCACCTGTGCTGCCGCAACCCAGAGTGGCTC
TTCCGGATTTACCAGGACACACAGGTGGATGTCCCCTCCTTGCTGGAGGT
CCTCCG---AGAAAATCTGAAG---GCCAAGCCTAACCTGAGAAA---GG
CCAAGGCAGCCAGTACAGTACACCCCGGTAGAGTCAGGGAGCCCAAGTGC
CAGACCTCGGTCCAGGCCACCAACGAGGCCAAGCTGACAGTGTCGTGGCA
GATCCCCTGGAACCTCAAATACCTGAAGGTCAGGGAGGTCAAATATGAAG
TATGGATACAGAAGAAGGATGCCAGCAAAGGGACCTTGGAGGATCAAATC
ATTCAAGCTAACCCAGCACTGGAGGCTTTTGGCAATGCCAAAACAGTGAG
AAACGACAACTCCTCACGCTTTGGGAAGTTCATTCGTATTCATTTTGGAA
CGAGCGGCAAACTCTCCTCTGCTGACATAGAAACTTACTTGCTTGAGAAA
TCCCGTGTGACCTTTCAGCTCAAATCGGAGAGGAACTACCACGTCTTCTA
CCAGATATTGTCCAACGAAAAGCCAGAGCTGCTGGACATGCTGTTGATTA
CCAACAACCCTTATGACTATTGCTACATCTCCCAAGGAGAAGTAACAGTT
AAGTCCATCAATGACAATGAAGAGTTGCTTGCCACTGACAGTGCCTTTGA
TGTGCTTGGCTTCACTCCAGAGGAGAAGATGGGGATCTATAAGTTGACGG
GTGCCATTATGCACTATGGCAACATGAAGTTTAAGCAGAGGCAGCGTGAG
GAGCAGGCAGAGCCTGATGGCACTGAGGCAGCTGACAAGTCAGCCTACCT
GATGGGGCTCAACTCTGCTGATCTTGTGAAGGGACTCTGCCATCCCAGGG
TCAAAGTCGGCAATGAGTATGTAACAAAGGGACAAGGTGTAGATCAAGTC
TATTAC--

----------------------TACCTGATTTACGCATCCTTCTCGTTCA
TGGGATGTTTACAAATCACYGACGGATCCAACATAGTCAATCTTTTGGCC
AGCGACTCGCCGAGCGTGTTGTACGCTCTGACCCAGCAGAAGTATTTCAG
CAACTACAGCCCAGTGATAGGGTTCTATATCTACGAGCCCATTGACTACT
GGAACTCCACTGTGCAGGAGCACCTCAAGATACTGGGCCAGGGATTCAAT
ACGATATCGTGGATCAATAATTACTTTCAGTATCTGAAGGTGACGAACGT
CAGCGCGTCGACCAAGAGCGACTTCATCACCGTCCTCAAGACTTCSTTCC
TGAGGAGTCCCGAATATCAGCACTTCACGGACGACATCATCTTTTCCAAA
A---CGGGGG------------ACGACTTAAACATCATCGCGTCCAGGAT
GTACCTGGTGGCGCGGACCACGGAGAAGACCCGGGAGGAGGTGGTGGAGC
TGCTGGAGARGCTCCGCCCGCTCTCGCTCATCAACAGCATCAAGTTCATC
GTGTTCAACCCCACCTTCGTGTTCATGGACCGCTACAGCTCCTCGGTCGT
CTCCCCCATCATGACGTCCGGATTCAGCGTCCTGACCATTCTCGTGCTCA
CGTTCTTCCTCGTCGTCAACCCACTGGGAAACTTCTGGCTGATACTGACC
GTCACCTCCGTGGAGCTGGGCGTCCTGGGCCTGATG--------------

NNNNNNNNNNNNNNNNNNNNNNNNNNNNNNNNNNNNNNNNNNNNNNNCCCCCAG
CCTTTCGCTTGTTCCATTGAGGACCCCACTAARCAAACCAAGTTCAAGGG
GATTAAGACTTATATATCCTACCGCGTGACCCCCAGCCACGTTGGCCGGC
CTGTGTACCGTCGCTACAAGCACTTTGACTGGCTGTAYAACAGGTTGCTG
CACAAATTCACCGTCATCTCTGTGCCCCATCTGCCCGAGAAGCAGGCAAC
TGGGCGTTTTGAGGAGGACTTTATTGAAAAGCGCASGAGGCGGCTCATCC
TCTGGATGGACCACATGACCAGTCACCCTGTCCTCTCGCAGTACGAGGGC

TTTGAGCACTTCTTCATGTGCGGTGATGATAAACAGTGGAAGCTGGGCAA
GCGGAGGGCGGAGAAGGATGAGATGGTGGGGGCCCACTTCATGCTCACCT
TCCAGATCCCCAACGAGCACCAGGACCTGCAGGATGTGGAGGAGCGGGTG
GACTCCTTTAAGGCCTTTGCCAAGAARATGGACGACAGCGTCATGCAGTT
AACACATGTGGCCTCCGAGCTGGTGCGCAAACACCTGGGAGGGTTCCGCA
AAGAGTTCCAAAGGCTGGGGAACTCCTTCCAGTCCATCAGCCAGGCTTTC
ATGCTGGACCCCCCCTACAGCTCTGATGCCCTGAATAATGCCATCTCCCA
C---

--------------------------------------GCCAAATCCCGC
TTTCACCATGGCGTCGGCACGGGGCCTGGCACGGACC---GCAGCGTCCC
ACTC---AACAGCTTGCTATCCCCGCAACAAACCGATGAGACCGCAGTGG
---CCTCCCCGCAGCGATGGTTTGTCACCC---CTGCCAACAATCGACTG
GACTTTGCCGCCTCGGCATACGATGCCGCCGCTGCAGCTGATTTTGCCGG
TAACGCGGCCACCCTTCTGTCGTACGCAGCTGCCGGAGTGAAAGCGC---
---TCCCACTGCCCACTGCAGCCTGCTCCAACAGAGCCCTGGGCTATTAC
GCGGAGCCGCCAG---GGTGG---GGCACACGCACTCCACCGCAGTACTG
T---------AGTAAATCAAGCGCGGTTCTTTCATGCTGGCCCGCCAATT
CCATTGGGAGCAGAACATCCA---CCTCCAATTACCTGG---TTGGCTTG
GACGA---CGGG---GACGC---TATCGCACCTGAGAGGTCACCT---CT
---CGGGGTGGCAGACGAA---GCCAAGCCAAAAGACCT------GTCGG
A---ATCAAGCTGGATAGAG---ACCCCGTCTTCAATTAAATCAATCGAC
TCAAGTGATTCTGGGATCTTTG---AACAGGCCAAACGGAGGAGAATTTC
GCCATCTGCTACACCG---------GTTTCAGAGACGTCGTCCCCATTAA
AATCAGAA--

>Bathylagus euryops

-------------------------GGAGGAGTACATCGTGGTGTTCAGC
CGCTCCATTAACAGGTTGATTCTTAACGAGGCAGAGCTCATTCTGGCCCT
GGCTCAGGAGTTCCAGATGAAGGTTGTGACGGTGTCTCTGGAGGAGCAGT
CCTTTGCCGCTATAGTGAAGGTGGTCAGCGAGGCGACCATGTTAGTCAGT
ATGCACGGAGCCCAACTGGTGTCTTCTCTCTTCCTGCCCAGAGGGGCCGC
GGTGGTTGAGCTTTTCCCCTATGCAGTAAACCCGGAGCAGTACGCTCCCT
ACAAAACCTTAGCCTCCCTCCCTGGCATGGACCTGCAGTATGTGTCCTGG
AGAAACACGATAGAGGAGAACTCCCTGGCCTACCCCGAGAGGCCCTGGGA
GCAGGGGGGCATCGCCCACTTGGACAAGGACGAGCAAGAGCGCATCCTGG
CCAGCAAAGAAGTCCCGAGGCACCTGTGCTGCCGCAACCCGGAGTGGCTC
TACCGCATCTACCAGGACACCATYGTGGACRTCCCGTCTCTACTGGACGC
CCTCAG---AGAGAGCCTGAAA---ACCAGGCCGAACCTGAARAA---GG
CCAAGCCGGCCAGCACGGTCCACCCGGGCCGGGTGAGGGAGCCCCAGTGC

```
CAGACGTCGGTCCAGGCGACCAACGAGGCCAAGCTCACCGTGTCCTGGCA
GATCCCCTGGAACCTGAAGTACCTGAAGGTCCGGGAGGTGAAATACGAAG
TGTGGATCCAGAAGAGGGATACGAGCAAGGGTACCCTAGAGGATCAAATC
ATCCAGGCTAACCCTGCACTCGAGGCCTTCGGTAATGCCAAAACATTGAG
AAATGATAATTCATCACGTTTTGGCAAATTCATCCGTATTCATTTTGGAC
AAAGTGGCAAATTATCCTCTGCTGACATAGAGACTTATCTGCTTGAGAAG
TCACGTGTTACCTTTCAGCTGAAATCAGAGAGGAACTACCATATCTTCTT
CCAGATCTTGTCCAATCAAAAGCCAGAGCTGTTGGACATGCTGTTGATCA
CCAACAATCCATATGACTACGCCTATATCTCCCAAGGAGAGGTGACAGTA
GCATCCATCAATGATTCTGAGGAGCTGATGGCCACTGACAGTGCTTTTGA
TGTGCTGGGCTTTACTGCAGAGGAGAAAATGTCCGTCTACAAACTGACAG
GTGCCATAATGCACTATGGCAACATGAGGTTCAAGAACAAGCAGCGGGAG
GAGCAGGCAGAGCCTGACGGTACAGAGGCTGCTGACAAGTCCGCTTATCT
AATGGGGCTGAACTCTGCAGATCTTATTAAAGGACTATGCCACCCAAGAG
TCAAGGTTGGCAATGAGTATGTCACCAAAGGTCAGAGTGTCGATCAAGTC
TACTAC--------------------------------------------
--------------------------------------------------
--------------------------------------------------
--------------------------------------------------
--------------------------------------------------
--------------------------------------------------
--------------------------------------------------
--------------------------------------------------
--------------------------------------------------
--------------------------------------------------
--------------------------------------------------
--------------------------------------------------
--------------------------------------------------
--------------------------------------------------
--------------------------------------------------
--------------------------------------------------
--------------------------------------------------
--------------------------------------------------
--------------------------------------------------
--------------------------------------------------
--------------------------------------------------
--------------------------------------------------
--------------------------------------------------
--------------------------------------------------
--------------------------------------------------
--------------------------------------------------
--------------------------------------------------
--------------------------------------------------
--------------------------------------------------
--------------------------------------------------
--------------------------------------------------
--------------------------------------------------
--------------------------------------------------
--------------------------------------------------
--------------------------------------------------
--------------------------------------------------
--------------------------------------------------
--------------------------------------------------
```


---TCCTACA
CCATCGAGATGGGCACCAAAGGGCCTCAGTGGAAAGAGAGTCCCCAGCCT
TTCTCTTGCTCCATCGAAGACCCCACCAAGCAGACCAAGTTTAAAGGCAT
CAAGACCTACATATCGTACCGGGTGACCCCCAGCCACCTGGGGCGGCCCG
TGTACCGGCGCTACAAGCACTTTGACTGGCTGTACAGTCGCCTGCTGCAC
AAGTTCACGGTCATCTCCGTGCCCCACCTCCCGGAGAAGCAGGCCACAGG
GCGCTTCGAGGAGGACTTCATCGAGAAGCGCAAGAGGCGGCTGGTCCTCT
GGATCAACCACATGACCAGCCACCCKGTCCTGTCCCAGTACGAGGGGTTC
GAGCACTTCCTGATGTGCGCCGACGACAAGCAGTGGAAGCTGGGCAAGCG
GCGGGCGGAGAAAGACGAGATGGTGGGCGCCCACTTCATGCTCACCTTCC
AGATCCCCACGGAGCACCAGGACCTGCAGGACGTGGAGGAGCGCGTGGAC
AACTTCAAGTCCTTCGCCAAGAAGATGGATGACAGTGTTCTGCAGCTGAC
GCACGTGGCGTCGGAGCTGGTGCGGAAGCACGTGGGAGGGTTCCGGAAGG
AGTTCCAGCGGCTGGGGAACGCCTTCCAGTCGGTCAGCCAGGCCTTCATG
CTGGACCCCCCCCACAGCTCGGACGCCCTCAACAATGCCATCTCTCAC--

-----------------------------------GCCAAATCTCGCTTT
CACCCTGGCGTAGGGACTGGTCCTGGCACAGACC---GCAGCGTCCCACT

TAGTAACAGCTTGCTGTCCCCGCAACAAACCGAAGAGCACACAGTTG---
CTTCCCCGCAGCGATGGTTTGTCACCC---CTGCCAACAACCGACTGGAC
TTCGCCGCCTCGGCATACGACGCCGCCGCGGCTGCTGATTTCGCCGGTAA
CGCGGCCACCTTGCTGTCTTACGCAGCTGCTGGAGTAAAAGCCC------
TTCCCCTGCCCACTGCTGGCTGCTCCAACAGACCTTTGGGTTATTATGCC
GACCCATCCG---GCTGG---GGCGCCCGCACACCGCCTCAGTACTGC--
-------AGTAAATCCAGCTCGGTGCTTTCGTGCTGGCCCACAAATGCTG
TTGGAAGCCGAACAGGCA---CGTCCAATTACCTGC---------CGGAA
GA---CGCG---GACAC---CATCCCCACGGAGAGGTCTCCG---AT---
CGGAGTGCCAGAGGAG---ACCAAACCAAAAGACTT------GTCCGA--
-GTCCAGCTGGATAGAG---ACGTCGTCTTCAATAAAGTCGATAGATTCA
AGTGATTCTGGGATCTTTG---AGCAGGCCAAACGGAGAAGAATTTCACC
GTCTGCCACACCG---------GTTTCGGAGACAGTTTCCCCGCTGAAAT
CAGAG---

--------------------------CGGCGTTTTCCTCCCAAGCT---C
CGGGCTAC---GCGGCCGCCGCGCTGGGA---CACCACCA------CCAC
CCGACCCATGTCAGCTCC---TACTCCACCGCCGCCTTCAACTCCACCCG
GGACTTTCTTTTTCGAAATCGGGGCTTCGGAGACGCGACCAG--------
----CGCCCAACACAGCCTGTTCGCCTCCGC---AGCGGGAACTTT---C
------GCAGGGCCCCACGGACACTCCGATGCGGCGGGACACCTGCTCTT
CCCGGGACTTCACGAG---CAAGCCGCGGGCCACGCGTCCTCCAACGTGG
TAAACAGTCAGATGCGCTTGGGCTTTTCGGGGGACATGTACGGACGGGCC
GACCAGTACGGCCATGTGACGAGCCCGCGAT---CCGACCACTACGCGTC
GACCCAGTTGCACGGTTATGGCCCCATGAACATGAATATGGCCGCA---C
ACCACGGAGCAGGGGCCTTCTTCCGTTACATGAGGCAGCCGATCAAACAA
GAGCTCATCTGCAAGTGGGTCGAGCCGGAGCAGTTGTCGAACCCGAAAAA
GTCGTGCAACAAAACTTTCAGCACGATGCACGAGCTGGTGACCCACCTGA
CCGTGGAGCATGTGGGGGGACCGGAGCAGTCCAACCATATTTGCTTTTGG
GAAGAGTGCTGCCGAGAAGGAAAACCCTTCAAAGCTAAATACAAACTTGT
AAATCATATCAGAGTACACACCGGAGAGAAACCCTTCCCGTGTCCGTTCC
CCGG----------
>Bathymaster signatus
AGCCTCCTCATTCGAGCAGAGCTAAGCCAGCCCGGCGCCCTCTTAGGAGA
CGACCAAATTTATAATGTAATTGTTACAGCACATGCATTCGTAATAATTT
TCTTTATAGTAATACCAATCATGATCGGAGGTTTCGGAAACTGACTTATT
CCTCTAATGATTGGAGCCCCAGACATGGCATTCCCCCGGATAAATAATAT
GAGTTTTTGACTTCTTCCTCCCTCTTTCCTCCTTCTCCTTGCCTCTTCAG
GTGTAGAGGCGGGAGCCGGAACAGGGTGAACAGTTTATCCCCCTCTTGCT
GGCAATTTAGCCCATGCAGGAGCTTCTGTTGATTTAACAATCTTTTCCCT
TCATTTAGCAGGAATTTCTTCAATCCTAGGGGCAATTAATTTCATTACAA
CTATTATTAACATGAAACCCCCTGCCATTTCCCAATACCAAACACCCCTT
TTCGTTTGATCAGTACTCATTACCGCAGTTCTTCTACTTCTTTCTCTTCC
AGTTCTTGCAGCTGGTATTACCATGCTACTAACAGACCGTAATCTTAACA
CCACTTTCTTTGATCCTGCCGGGGGAGGAGATCCAATTCTTTACCAACAT
TTA---

------------TTCCTAGAGAGAAACCTTCACCCGTCTAATTGCCTTGG
CATGCTCTTGCTGTCTGATGCCCACCAGTGCACCAAGCTGTCAGAGCTCT
CCTGGGGCATGTGCCTCAGCAATTTCCCCGCTATTTGCAAGACAGAAGAC
TTCCTCCAACTGCCCAAAGATATGGTGGTGCAGCTTTTGTCACACGAGGA

GCTAGAGACAGAAGATGAGAAACTGGTTTATGAAGCTGCCCTTAACTGGA
TCAACTATGACCTGGAAAAGAGGCACTGCCACCTTCCAGAGCTCCTGAGG
ACTGTCCGCCTGGCCCTGTTGCCCGCCATCTTTCTCATGGAGAATGTTTC
GACAGAAGAGCTGATCAATGCCCAGCCCAAGAGCAAGGAGCTGGTGGATG
AAGCTATCCGATGTAAGCTGAAGATCCTGCAGAATGATGGTGTCGTTAAC
AGTCCGTGTGCCCGACCAAGAAAGACCAGCCATGCCCTATTTCTTCTGGG
AGGGCAGACGTTCATGTGTGACAAGTTGTACCTTGTGGACCAGAAAGCCA
AAGAGATCATCCCCAAGGCTGACATTCCCAGCCCCAGGAAGGAGTTCAGT
GCCTGCGCCATCGGCTGTAAGGTGTACATCACTGGTGGGA--GAGGCTC-
AGAGAATGGTGTGTCCAAAGATGTGTGGGTCTACGACACCGTCCAAGAGG
AATGGTCGAAGGCGGCGCCCATGCTCATTGCCAGGTTCGGCCATGGCTCT
GCAGAGCTGAAACACTGCCTCTACGTGGTAGGAGGTCACACGGCAGCAAC
TGGCTGCCTCCCAGCTTCTCCGTCTGGAGGATTACGTCGTGGTGTTCAGT
CGCTCAGCGACAAGGCTGATACTGAATGAGGTGGAGCTAATCATGGCGCT
GGCCCAGGAGTTCCAGATGAAAGTGGTCACAGTGTCCCTAGAGGAACAGT
CTTTCCCCAGTATCGTCCAGGTGATCAGCGGCGCGTCCGTGTTGGTCAGT
ATGCACGGCGCTCAGCTCATTACCTCACTCTTCCTCCCCAGAGGAGCTGC
AGTGGTGGAGCTGTTCCCCTTTGCTGTGAACCCAGAGCAGTACACCCCGT
ATAAAACCCTCGCCACCCTTCCAGGCATGGACCTTCACTATGTCTCCTGG
AGGAACACTAAGGAGGAGAACACCATCACCCACCCAGACAGACCCTGGGA
GCAAGGGGGCATCGTTCACTTGGAGAAGGAGGAGCAGGAGCGTATACTGG
CGAGCAGAGATGTCCCCAGGCACCTGTGCTGCCGCAACCCAGAGTGGCTC
TTCCGGATCTACCAGGACACTTTGGTGGACATCCCTTCCTTCCTGGAAAC
CCTTAA---AGAGGGCTTGAAG---ACAAAGCCCAGCGTGAAGAA---GT
CAAAGCCGGCCAGCACAGTCCACCCGGGCCGGGTCAGACAACCCCAGTGT
CAGACCTCGGTGCAAACCACCAACGAGGCTAAACTCACAGTCTCCTGGCA
GATCCCGTGGAATCTAAAATACTTGAAGGTGCGAGAGGTGAAGNNNNNNNNN-------
--AAAAGAGATGCTAGCAAGGGGACGCTGGAGGACCAGATCATCCAGGCC
AACCCGGCGCTGGAGGCTTTCGGCAACGCCAAAACGNTGAGAAACGACAAC
TCGTCTCGCTTTGGAAAATTCATCCGAATTCACTTCGGCACGAGCGGGAA
GCTGTCGTCTGCCGACGTCGAGACTTACCTGCTGGAGAAGTCCCGGTGCA
CCTTTCAGCTCAAGGCTGAGAGGAACTACCACATCTTCTACCAGATCCTG
TCCAATCAGAAGCCAGAGCTGCTGGACATGCTGCTGATCACCAACAACCC
CTATGACTACTCCTACATCTCCCAAGGGGAGATAACGGTCGCCTCCATCA
ACGACTCCGAGGAGCTGCTGGCCACCGACAGTGCCTTCGATGTGCTCGGC
TTCACTCCGGAAGAGAAGATGGGCGTGTACAAGCTAACCGGTGCCATCAT
GCACTACGGCAACATGAAGTTCAAACAAAAGCAGCGCGAGGAGCAGGCCG
AGCCGGACGGGACGGAGGCTGCCGATAAAACCGCATACCTAATGGGGCTA
AACTCTGCCGACGTCATCAAGGGGCTGTGTCATCCCAGAGTCAAGGTAGG
AAATGAATTTGTCACCAAAGGACAAAGTGTGGACCAAGTCTACTACCCCA
ACAAGGAGGCCTTCAAGTGTGAGGAGTGCGGGAAGCACTACAACACCAAG
CTGGGATATAAGCGCCATGTGGCCATGCACTCTGCCACGTCAGGAGATCT
CACCTGCAAAGTGTGCATGCAGAGCTATGAGAGCACACCCGTGCTCTTGG
AGCACCTCAAGACCCACTCGGGGAAGTCTTCGGGCGGCACCAAGGAGAAA
AAGCACCCGTGCGACCACTGTGACCGTCGTTTCTACACGCGCAAGGATGT
GAGACGGCACATGGTGGTCCACACGGGTCGAAAGGACTTCCTCTGCCAGT
ACTGTGCCCAGCGCTTCGGCAGGAAGGACCATCTGACACGTCACGTGAAG
AAGAGCCACTCGCAGGAGCTGCTGAGGATCAAGACGGAGCCTCCGGATAT
GTTGGGTCTTTTAGCGTCCGGGTCACCACCTTGCTCTGTGAAGGAGGAGC
TCAGCCCCATGATGTGCGGCATGGGTCCCAACAAAGACCCCATGATGGGC
AAACCGTTCCCCAGCGGGGCCCCTTTTCCGATGGGCATGTACAACCCCCA
CCAT------CTCCAGGCCATGTCTAATTCTGGGGTGGGTCACCCG----
--CACCCGTCCCTGATGCCCAGTCCCTTGTCTGCWGCTATGGGCATGGGC

TGCCACATGGAATATCTCATCTACGCCTCTTTTTCATTCATGGGATGTTT
ACAGATCAGTGATGGATCAAATATAGTGAACCTGCTGGCTAGTAACTCTC
CGAGTGTTTCGTACGCTCTGACCCAGCAGAAATACTTCAGTAACTACAGT
CCCGTGATTGGGTTCTACATTTACGAGCCCATCGAGTACTGGAACTCCAC
GGTGCAGGAGCACCTGAAGACCCTGAGTCATGGCTTCAACAAGATCTCTT
GGATGGACAACTTTTTCCACTACCTGCGCGTGGTGAATGTGAGCGCGTCA
ACCAAGAGCGACTTCATCACCATTCTCAAGGGCTCCTTCCTTCGCAGCCC
GGAGTACCAGCACTTCACTGAGGACATCATATTCTCCAAGA---ACCGCG
AGACTG------ATGAGTACGACATTATCGCCTCGCGGATGTACCTGGTG
GCACGGACGACAGAGAAGAAGCGAGAGGAGGTCGTGGAGCTTCTGGAAAA
GCTTCGTCCGTTGATGTTGATCAACAGCATCAAGTTCATTGCCTTCAATC
CCACGTTTGTGTTCATGGACCGCTACAGCTCCTCTGTCATCTCGCCCATC
CTGACCTCAGGCTTCAGCGTGCTCACAATCCTCATCCTCACTTTCTTCCT
GGTCATCAACCCGTTGGGGAACTTCTGGCTCATCCTCACGGTAACATCCG
TGGAGCTGGGCGTCTTGGNNNNNNNN--------------------------------

--------------------------TCGTACACCATCGAGATGGCCCCG
TGGGGCCCCTGTGGAAGGAGAGCCCGCAGCCKTTCTCCGTCTCCATCGAG
GACCCCACAAAACAGACAAAGTTCAAAGGCATCAAGACGTACATTTCATA
CCGGGTCACGGCGAGCCACACGGGGCGTCCCGTCTACAGGCGCTACAAAC
ACTTCGACTGGCTGTACAACCGCTTGCTGCACAAGTTCACTGTGATCTCC
GTGCCTCACCTGCCCGAGAAGCAGGCCACGGGGCGRTTCGAGGAGGACTT
CATCGAGAAGCGCAAGAGACGGCTGATACTGTGGATGAACCACATGACCA
GCCACCCGGTCCTCTCCCAGTACGAGGGCTTCGAGCACTTTCTGATGTGC
GCCGACGACAAGCAGTGGAAGCTGGGCAAGAGGCGGGCGGAGAAGGACGA
GATGGTGGGCGCCCATTTCATGCTGACCCTGCAGATCCCCAACGAGCACC
AGGACCTTCAGGACGTCGAGGAGCGGATCGACACCTTCAAGGCCTTCGCC

AAGAAGATGGACGACAGCGTGATGCAGCTCACGCACGTCGCCTCGGAGCT
GGTGCGCAAGCACCTGGGCGGGTTCAGGAAGGAGTTCCAGCGGCTGGGAA
ATTCTTTCCAGTCCATCAGCCAGGCGTTCATGCTGGACCCTCCCCACAGC
TCGGAGGCCCTCAACAANNNNNNNNNNNNN---------------------------------
----

----GCCAAATCTCGCTTTCACCCTGGCGTGGGGACTGGTCCTGGCACGG
AGC---GCAGCGTCCCACTCGGCAACAGCTTGCTGTCCCCGCAGCAAACC
GACGAGCCCACTGTTGCCACCCCCCCGCAGCGATGGTTTGTCACCC---C
TGCCAACAACCGACTGGACTTTGCTGCCTCGGCATACGACGCCGCT----
-----GATTTCGCCGGTAACGCGGCCACCTTGCTGTCCTACGCAGCGGCC
GGAGTGAAGGCTC------TTCCCCTGCCGACTGCAGGCTGCTCCAACCG
GCCTCTTGGCTATTACGCAGACCCGTCAG---GCTGG---GGAGGACGCA
CACCGCCGCAGTACTGTGGTGTAAACAGCAAATCCAGCTCGGTCTTTTCC
TGCTGGCCCACTAACTCTATCGGTGGCAGAGCGGGCA---CC---AACTA
CCTGG---------CCGAGGA---GGGA---GACTC---CATCACGACGG
AGAGGTCGCCG---AT---CGGCGGCTCGGATGAG---ACCAAACCCAAA
GACATGAC---ATCCGA---GTCGAGCTGGATAGAG---ACGCCGTCCTC
CATTAAGTCAATTGATTCGAGCGATTCTGGTATCTTTG---AACAGGCCA
AAAGGAGAAGAATCTCACCTTCTGCCACGCCG---------GTTTCAGAG
ACAGTGTCCCCGTTAAAATNNNNNNNNNNNNNNNCAGGCGAAGTCACAGAGAGAGAAGTAGCGTT
GGGGATCAATCCGTTCGCGGATGGGATGGGCGCCTTCAAAATAAACCACA
GCTCCCACGATATCGGCTCCGG---ACAAACGGCGTTTTCCTCCCAGGCG
---CCCGGCTAC---GCGGCAGCCGCCCTGGA---CACCATCA------
CCACCCGACCCACGTTGGCTCT---TACTCCACGGCGGCTTTCAACTCCA
CCAGGGACTTTCTCTTCAGAAATCGGGGTTTCGGGGACGCCACCGG----
--------GGCTCAGCACAGTTTGTTCGCCTC---------CGGAAGTTT
---C------GCAGGGCCACATGGACACTCGGATGCAGCGGGGCACCTGC
TCTTCCCGGGGCTCCACGAG---CAAGCGGCGAGCCATGCGTCTTCCAAC
GTGGTCAACAGCCAGATGCGGCTGGGCTTCTCGGGGGACATGTACGGACG
GGCCGACCAGTACGGCCACGTTACGAGCCCACGGT---CCGACCACTACG
CGTCGACCCAGCTGCACGGCTATGGCCCCATGAACATGAATATGGCCGCG
---CACCACGGAGCAGGGGCCTTCTTTCGATACATGAGGCAGCCGATCAA
GCAAGAGCTCATCTGCAAGTGGATCGAGCCGGAGCAGCTGACGAATCCCA
AAAAGTCGTGCAACAAAACTTTTAGCACGATGCACGAGCTCGTGACCCAT
CTGACGGTGGAGCATGTGGGGGGACCAGAGCAGACCAACCACATCTGCTT

CTGGGAGGAATGCGCCAGAGAAGGAAAGCCATTCAAAGCCAAATACAAAC
TTGTAAATCATATCAGAGTACACACCGGAGAAAAGCCCTTTCCGTGTCCG
TTCCCCGGCTGTGGCAAA
>Bathypterois atricolor

------------TTCCTTGAGAGGAACCTGCACCCATCCAACTGCCTTGG
CATGCTGCTACTGTCAGATGCCCACCAGTGCACCAAACTGTCAGAGCTGT
CCTGGGGTATGTGTCTCAGCAACTTCCCTGCCATCTGCAAGACAGAGGAC
TTCCTCCAGCTGCCCAAAGACATGGTGGTACAGCTTCTGTCCCACGAGGA
GCTGGAGACAGAAGATGAGAGACTGGTTTACGAGGCTGCCCTTAACTGGG
TCAACTATGACCTGGAGAGGAGACACTGCATCCTGCCAGAACTGCTGAGA
ACCGTGCGCCTGGCCTTGCTTCCTGCCATCTTCCTCATGGAGAACGTCTC
CACAGAAGAGCTGATCAATGCCCAAGCAAAGAGCAAGGAGTTGGTGGACG
AGGCCATCCGCTGCAAGCTGAAGATCCTGCAGAATGACGGTGTAGTCAAC
AGTCCCTGTGCCCGGCCCAGAAAGACCAGCCACGCCCTTTTCCTGCTGGG
AGGGCAGACCTTCATGTGTGACAAGCTGTACTTGGTGGACCAGAAGGCCA
AAGAGATCATTCCCAAGGCGGACATCCCCAGCCCCAGGAAGGAGTTCAGC
GCCTGTGCCATCGGCTGTAAGGTCTACGTCACAGGCGGGA--GAGGCTC-
AGAGAACGGCGTGTCGAAAGATGTGTGGGTCTATGACACCGTCCATGAGG
AGTGGTCTAAGGCAGCACCCATGCTCATTGCCAGGTTTGGCCATGGCTCT
GCTGAGCTCAAACACTGCCTCTATGNNNNNNNNNNNNNNNNNNNNNNNNNNNNNNNNNNNNNNNNNNNN
NNNNNGGATGAGTACATTGTAGTGTTCAGT
CGTTCTACGACGAGGCTGATTCTAAACGAAGCGGAGCTGATTCTGGCACT
GGCCCAAGATTTTCAGATGAGGGTGGTTACCGTGTCCCTGGAGGAGCAGT
CTTTCCCCAGCATCGTCCAGGTGATCAGCGGGGCTTCCATGTTGGTTAGT
ATGCATGGAGCTCAGCTCGTCACCTCGCTCTTCCTCCCTAGAGGAGCTGC
TGTAGTGGAGCTCTTCCCCTATGCTGTGAACCCAGAACAGTACACCCCAT
ACAAAACCCTAGCCTCCATACCAGGCATGGACCTTCAATATGTTTCCTGG
AGGAACACTATTGAGGAGAACACTGTCGCCCACCCAGACAGACCCTGGGA
CCAAGGAGGCATTGCCCATTTGGAGAAAGAAGAGCAGGAGAGAATCGTAG
CCAGCAAGGATGTCCCCAGGCACCTATGCTGCCGCAACCCAGAGTGGCTC
TTCAGGATCTACCAGGACACTCTGGTGGACATCCCCTCCTTCCTAGAAGT
CCTTAA---GCAGGGCCTGAAG---ATGAGGCCGAGCTTGAAGAA---GT
CCAAGCCGGCCAGCACGGTTCACCCGGGTCGGGTCCGGGAACCCCAGTGC
CAGACATCAGTCCAAGCCACCAACGAGGCTAAGCTCACCGTATCCTGGCA
GATCCCGTGGAACCTTAAGTACCTAAAGGTGCGAGAGGTGAAGTACGNNNNNNNNNNNNNNNAAA
AAGGATTCAAGCAAGGGCACCCTGGAGGATCAAATCATTCAGGCGAACCC
TGCGCTGGAGGCTTTTGGTAACGCCAAAACAGTGAGGAACGATAACTCCT

CCCGTTTTGGAAAGTTCATCCGAATTCACTTTGGAACCAGTGGTAAACTG
TCCTCTGCGGACATTGAGACCTACCTGCTGGAGAAGTCAAGGGTCACCTT
TCAGCTTAAGTCAGAGAGGAACTATCACATCTTCTTCCAAATCTTGTCCA
ATCAAAAGCCAGATCTGTTGGACATGCTGTTGATCACCAACAACCCTTAT
GACTACTCCTACATCTCCCAAGGAGAAGTAACCGTACCATCCATCAATGA
TTCAGAGGAGCTGATAGCCACTGACAGTGCCTTCGATGTGCTTGGCTTCA
CTCAAGAGGAGAAGATGGGAGTCTACAAGTTGACAGGGGCCATTATGCAC
TATGGCAACATGAGGTTCAAGCAAAAGCAGCGCGAGGAACAGGCTGAGTC
TGACGGTACAGAGGCTGCTGATAAGTCAGCTTATCTAATGGGGCTGAATT
CAGCAGACCTAATCAAAGGTCTCTGCCATCCCAGAGTCAAGGTAGGAAAT
GAGTATGTCACTAAAGGCCAGGGTGTAGATCAAGTCTACTACCCCAACAA
GGAGGCCTTCAAGTGTGAAGAGTGTGGCAAGCACTACAACACCAAGCTGG
GATACAAGCGCCATGTGGCCATGCACTCTGCCACGGCAGGGGACCTCACC
TGCAAGGTGTGCATGCAGAGTTATGAAAGCACACCGGTGCTCCTGGAACA
CCTCAAGAGCCACTCGGGGAAGTCCTCGGGTGGCGCCAAGGAGAAGAAAC
ACCCGTGCGACCACTGCGACCGTCGCTTCTACACGCGGAAGGATGTCAGG
CGGCACATGGTTGTCCACACGGGCCGAAAGGACTTCCTGTGCCAGTACTG
TGCCCAGCGCTTCGGCAGGAAGGATCATCTGACACGGCACGTGAAAAAGA
GCCACTCGCAGGAGCTGCTGAAGATTAAGACGGAGCCTCCGGATATGTTG
GGTCTCCTGGGCTCTGGCTCACCGCCTTGCTCTGTCAAGGAGGAGCTTAG
CCCCATGATGTGCAGCATGGGTCCCAATAAAGACCCCATGATGGGCAAAC
CCTTCCCCAGCGGGACCCCCTTCCCCATGGGCATGTACAACCCCCACCAC
------CTCCAGGCCATGTCAAATCCCGGCGTGGGTCACCCC------CA
CCCCTCTCTGATGCCGGGTTCCCTGTCTGCAGCTATGGGCATGGGCTGTC
ATATGGAATATCTCATCTACGCCTCCTTCTCCTTCATGGGATGTTTACAA
ATCAGCGACGGGTCGAACATTGTGAATTTGTTGGCCAGTAACTCTCCGAG
CGTCTCGTACGCCCTGACGCAGCAGAAGTACTTCAGTAACTATAGCCCTG
TGATCGGGTTTTATATCTACGAGCCCATAGAGTACTGGAACTCCACGGTG
CAGGAGCACCTGAGGACACTGAGCCACGGCTTCAACAAGATCTCCTGGAT
GGACAATTTCTACCACTACCTGCGGGTGGTGAACGTGAGCGCGTCGACCA
AGGCCGACTTCATCGCCGTCCTCAAGGGCTCCTTCCTGCGGAGCCCGGCG
TACCAGCACTTCACCGAGGACATCATCTTCTCCAAGA---GCCGGGACAA
CG------ACGAGTACGACATCATCGCCTCGCGCATGTACCTGGTGGCGC
CCACCACCGAGACGCGGCGCGACGATGTGGTGGAGCTGCTGGAGAAGTTG
CGGCCGCTCATGCTGATCAACAGCATCAAGTTCATCGCCTTCAACCCCAC
CTTCGTCTTCATGGACCGCTACAGCTCCTCGGTCATCTCGCCTATCCTCA
CCTCTGGCTTCAGCGTGCTCACCATCCTCATCCTCACCTTCTTCCTGGTC
ATAAACCCGCTGGGTAACCTGTGGCTCATCCTGACGGTCACGTCCGTGGA
GCTGGGCGTGCTGGGTCTGANN------------------------------

-----GAGAAGCACGGTGGAGGGCCAGCGATGCCTGAGAAGGCTGTGCGT
TTCTCTATCACCGTCATGTCTGTCTCTGTCCAGGCCTACGGAGAGGAG--
----------------------------GAGGCGGTTGTCATCTTCAGGG
AGCCAAAACCAAACTCTGAGCTGTCCTGTAAGCCCCTGTGCCTGATGTTT
GTGGATGAGTCTGACCACGAGACACTCACTGCTGTCCTGGGGCCTTTGGT
AGCAGAGAGGAATGCGATGAAGCAGAGCCGACTCATCCTTTCAGTTGGTG
GCCTCCCCCGCTCTTTCCGCTTCCACTTCAGAGGCACAGGCTATGATGAG
AAGATGGTGCGAGAGATGGAAGGGCTGGAGGCCTCAGGCTCCACCTATAT
CTGCACTCTTTGTGACTCCACTCGGGCAGAAGCCTCTCACAACATGGTGC

TCCACTCTGTCACCCGCAGCCATGATGAGAACCTGGAGCGCTACGAGATA
TGGAGGACCAACCCCTTCTCAGAGTCTGCCATGGAGTTGCGAGACCGGGT
CAAGGGGGTCTCTGCCAAACCATTTATGGAGACCCACCCGACTCTGGATG
CATTACACTGTGACATAGGTAACGCCACTGAATTCTACAAAATCTTCCAG
GATGAGATTGGGGAGGTGTTCCGGAAGGC---CAAC---CCTAGCCGGGA
GGAACGGCGCAGCTGGAGGGCAGCTCTCGATAAGCAGTTGAGGAAGAAGA
TGAAGCTGAGACCAGTGATGCGGATGAATGGGAACTTTGCCCGAAGGCTG
ATGACCCAGGAGGCTGTGGAAGTGGTGTGTGAACTGATTCCCACAGAAGA
GAGGCGGGAGGCCCTGAGGGAGCTGATGGGGCTCTATGTCCAGATGAAGC
CTGTGTGGCGTGCTACCTGCCCAGCCAAGGAGTGCCCTGACCAGCTGTGC
CGCTACAGCTTTAACTCTCAGCGCTTTGCTGATCTCCTCTCCTCCAACTT
TAAATACAGGTATGATGGAAAGATCACCAACTACCTGCACAAGACCCTGG
CCCATGTCCCTGAAATTATAGAGAGAGATGGCTCCATTGGAGCCTGGGCC
AGCGAGG--------------------TCATACACCATCGAGATGGGCCC
CAAAGGGCCCCAATGGAAAGAGAATCCTCAGCCTTTCTCTTGCTCCATTG
AAGACCCCACAAAACAGACCAAGTTCAAGGGTATCAAGACGTACATATCC
TACCGGGTCACCCCTAGCCACATTGGGCGACCCGTGTACAGACGCTACAA
ACACTTTGACTGGCTGTATAATCGCCTGCTGCACAAGTTCACTGTGATTT
CTGTGCCTCACCTGCCCGAGAAGCAGGCCACGGGGCGCTTTGAGGAGGAC
TTCATTGAGAAGCGCAAAAGACGACTGGTCCTCTGGATGAACCACATGAC
GAGTCACCCGGTCCTCTCCCAGTATGAGGGGTTCGAGCACTTCCTCATGT
GCGTCGACGACAAGCAGTGGAAGCTGGGTAAGAGGCGGGCTGAGAAGGAC
GAAATGGTGGGAGCCCACTTCATGCTGACGTTCCAGATCCCCAACGAGCA
CCAGGACCTGCAGGACGTCGAGGAGCGCGTCGACACCTTCAAGTCCTTCG
CCAAGAAAATGGACGACAGTGTCATGCAGCTCACGCACGTGGCCTCGGAA
CTGGTGCGCAARCACCTGGGAGGATTCAGGAAGGAGTTCCAGCGGCTTGG
GAAYGCCTTCCAGTCCATCAGTCAGGCGTTCATGCTGGACCCTCCCCACA
GCTCAGAAGCCCTCAACAACGCCNNNNNNNNN---------------------------

----------GCCAAGTCTCGCTTTCACCCTGGCGTAGGGACTGGTCCTG
GCACGGACC---GCAGCGTCCCACTCAGCAACAGCTTGCTATCCCCGCAA
CAAACCGAAGAGCCCACAGTAG---CTTCCCCACAGCGATGGTTTGTCAC
CC---CTGCCAACAACCGACTGGACTTTGCAGCCTCGGCATACGATGCCG
CT---------GATTTCGCCGGTAACGCAGCCACCTTGCTGTCCTACGCA
GCGGCTGGAGTGAAGGCAC------TGCCCCTGCCGACTGCAGGTTGCTC

CAACAGGCCTCTTGGGTATTACGCAGACCCATCAG---GCTGG---GGCG
CACGCACATCACCACAGTACTGTAGT------AGTAAATCCAGCTCAGTC
CTTTCTTGCTGGCCCACTAATTCTGTTGGTGGCAGANNNNNNN------NNNNNNNNNNNN--------
-NNNNNNN---NNNN---NNNNN---NNNNNNNNNNNNNNNNNNNNNN---NN---
NNNNNNNNNNNNNNNN---NNNNNNNNNNNNNNNNN------NNNNNN---NNNNNNNNNNNNNNNN---
NNNNNNNNNNNNNNNNNNNNNNNNNNNNNNNNNNNNNNNNNNNNNNNNN---
NNNNNNNNNNNNNNNNNNNNNNNNNNNNNNNNNNNNNNNNN---------
NNNNNNNNNNNNNNNNNNNNNNNNNNNNNNNNN-------
--ACAGGGGAAGTCACAGAGAGAGAAGTGGCTTTGGGGATMAATCCGTTC
GCAGACGGGATGGGCGCCTTCAAAATCAACCACGGCTCTCATGACATCGG
CTCCGG---GCAGACGGCGTTTTCCTCCCAGGCG---CCCGGTTAC---G
CAGCGGCCGCCCTTGGA---CACCACCA------CCACCCGACCCACGTC
AGCTCT---TATTCCACGGCGGCTTTCAACTCCACCCGGGACTTTCTCTT
CAGAAATCGGGGCTTCGGAGACGCCACCGG------------CGCGCAGC
ACAGTTTGTTCGCCTCTGC---CGCGGGGAGTTT---C------GCAGGG
CCGCACGGACACTCGGATGCCGCCGGACACCTGCTCTTCCCGGGGCTCCA
TGAG---CAAGCTGCGAGCCACGCGTCGTCTAACGTGGTAAACAGCCAGA
TGCGACTGGGCTTTTCTGGGGACATGTACGGGCGGGCCGACCAGTACGGC
CACGTCACAAGCCCGAGGT---CCGAACACTACGCGTCGACCCAACTGCA
CGGCTATGGCCCCATGAACATGAACATGGCCGCT---CACCACGGCGCAG
GGGCCTTCTTTCGCTACATGAGGCAGCCGATCAAACARGAGCTCATCTGC
AAGTGGATCGAACCGGAGCAGCTGACGAATCCTAAAAAGTCGTGCAACAA
AACTTTTAGCACCATGCACGAGCTCGTGACCCATCTGACGGTGGAGCACG
TGGGGGGTCCGGAGCAGTCGAACCACATCTGCTTCTGGGAGGACTGGCCC
C---

--
>Bathysaurus ferox
AGTCTTCTCATTCGAGCAGAACTTAGTCAGCCGGGGGCCCTCTTAGGCGA
CGACCAAATTTATAATGTTATCGTTACCGCACATGCTTTCGTTATAATTT
TCTTTATAGTAATACCAATTATGATCGGCGGGTTCGGAAACTGGCTGGTC
CCCCTAATGATCGGGGCCCCTGATATGGCCTTCCCCCGAATGAATAATAT
GAGTTTCTGACTTTTACCCCCCTCCTTCCTTCTCCTGCTGTCTTCCTCCG
GGGTAGAGGCTGGAGCTGGGACAGGTTGAACAGTTTATCCGCCCCTTGCC
AGCAACTTAGCACATGCCGGGGCCTCTGTAGACTTGACTATTTTTTCACT
TCACTTAGCAGGGGTGTCCTCAATTCTTGGGGCCATTAACTTCATTACTA
CTATTATTAACATAAAACCCCCAGCCATCTCTCAGTATCAAACCCCCTTA
TTTGTCTGATCCGTCCTTATTACAGCTGTTCTGCTACTACTATCCCTCCC
TGTCCTCGCAGCAGGTATTACTATACTTCTGACAGATCGAAACCTAAACA
CAACCTTTTTTGACCCGGCAGGAGGTGGGGATCCAATTCTGTACCAGCAC
CTATTCTGATTCTTCGGGCACCCAGAGGTATATATTCTTATCCTCCCCGG
GTTTGGAATAATTTCACACATTGTTGCCTACTATTCAGGTAAAAAGGAAC
CCTTTGGCTATATGGGTATGGTTTGGGCTATGATGGCAATTGGCCTTCTT
GGCTTTATTGTCTGAGCCCACCACATGTTTACCGTCGGAATAGATGTGGA
CACCCGAGCCTATTTCTTGAGAGGAACCTGCACCCATCCAACTGCCTTGG
CATGCTGCTACTGTCAGATGCTCACCAGTGCACCAAGCTGTCAGAGCTGT
CCTGGGGTATGTGTCTCAGCAACTTCCCTGCCATTTGCAAGACGGAGGAC
TTCCTCCAGCTGCCCAAAGACATGGTGGTGCAGCTTCTGTCCCATGAGGA
GCTGGAGACAGAAGATGAGAGACTGGTTTACGACGCTGCCCTTAACTGGG
TCAACTACGACCTGGAGAGGAGACACTGCCACCTGTCAGAGCTGCTGAGA
ACCGTGCGCCTGGCCTTGCTTCCCGCCATCTTCCTCATGGAGAACGTCTC
CACAGAGGAGCTGATCAATGCCCAAGCAAAGAGCAAGGAGTTGGTGGACG
AGGCGATCCGCTGCAAGCTGAAGATCCTGCAGAATGACGGTGTGGTCAAC

AGTCCCTGTGCCCGGCCCAGAAAGACCAGCCATGCCCTCTTCCTGCTGGG
AGGGCAGACCTTCATGTGTGACAAGCTCTACTTGGTGGATCAGAAGGCCA
AGGAGATCATCCCCAAGGCGGACATCCCCAGCCCCAGGAAGGAGTTCAGT
GCCTGTGCCATCGGCTGTAAGGTCTACGTCACGGGCGGGA--GAGGCTC-
AGAGAATGGCGTGTCTAAAGATGTGTGGGTCTATGACACCATCCATGAGG
AGTGGTCTAAGGCAGCACCCATGCTCATTGCCAGGTTTGGCCATGGCTCT
GCTGAGCTCAAACACTGCCTCTATGTGGTTGGAGGACACACAGCAGCCAC
TGGCTGCCTCCCAGCCTCTCCGTCTGGATGAGTACATTGTGGTGTTCAGT
CGTTCTACAACAAGGCTGATTCTAAACGAAGCAGAGCTGATCCTGGCACT
GGCCCAAGAGTTTCAGATGAGGGTGGTTACAGTGTCCCTGGAGGAGCAGT
CTTTCCCCAGCATCGTCCAGGTGATCAGCGGGGCCTCCATGTTGGTTAGT
ATGCACGGAGCTCAGCTCGTCACCTCACTCTTCCTCCCTAGAGGAGCTGC
TGTAGTGGAGCTCTTCCCCTATGCTGTGAATCCAGAACAGTACACCCCAT
ACAAAACTCTAGCCTCCTTACCAGGCATGGACCTTCAATATGTTTCCTGG
AGGAACACTATTGAGGAGAACACTATTGCCCACCCAGATAGACCCTGGGA
CCAAGGAGGCATTGCCCATTTGGAGAAAGAAGAGCAGGACAGAATCCTAG
CCAGCAAGGATGTCCCCAGGCACCTATGCTGCCGCAACCCAGAGTGGCTC
TTCAGGATCTACCAGGACACTCTGGTGGACATCCCCTCGTTCCTGGAAGT
CCTCAA---GGAGGGTCTGAAG---ACAAGGCCGAGCTTGAAGAA---GT
CCAAGCCGGCCAGCACGGTTCATCCGGGCCGGGTCCGAGAACCCCAGTGC
CAGACATCAGTCCAAGCCACCAACGAGGCTAAGCTCACGGTATCCTGGCA
GATCCCGTGGAACCTTAAGTACCT-AATGTGCGGGA-GTGAAGTACGAGG
TGTGGATCACAAAAAGAGATACAAGCAAGGGAACCCTGGAGGATCAAATC
ATTCAGGCAAACCCTGCGCTGGAGGCTTTTGGTAATGCCAAAACATCGAG
GAACGATAATTCCTCCCGTTTTGGAAAATTCATCCGAATTCACTTTGGAA
CCAGCGGTAAACTGTCCTCTGCGGACATTGAGACCTACCTGCTGGAGAAG
TCCCGGGTCACCTTTCAGCTCAAGTCAGAGAGGAACTACCACATCTTCTT
CCAGATCTTGTCCAATCAAAAGCCAGAGCTGTTGGACATGCTGTTGATCA
CCAACAACCCTTATGACTACTCCTTCATCTCCCAAGGAGAAGTAACCGTA
CCATCCATCAATGATTCAGAGGAGCTGATGGCCACTGACAGTGCCTTTGA
CGTGCTTGGCTTCACTCAAGAGGAGAAGATGGGAATCTACAAGTTGATTG
GGGCCATTATGCACTATGGCAACATGAGGTTCAAGCTAAAGCAGCGAGAG
GAACAGGCTGAGCCTGATGGTACAGAGGCTGCTGATAAGTCAGCTTATCT
CATGGGGCTGAATTCAGCAGACCTCATCAAAGGACTCTGCCATCCCAGAG
TCAAGGTAGGAAATGAGTATGTCACCAAAGGCCAGAGTGTAGATCAAGTC
TACTACCCCAACAAGGAGGCCTTCAAGTGTGAAGAGTGTGGGAAGCACTA
CAATACCAAGCTGGGATACAAGCGCCATGTGGCCATGCACTCTGCCACCG
CCGGGGATTTCACCTGTAAAGTGTGCCTGCAGAGTTACGAAAGCACCCCG
GTGCTCCTGGAACACCTCAAGAGCCACTCTGGGAAGTCCTCGGGTGGCAC
CAAAGAGAAAAAGCACCCTTGCGACCACTGCGACCGTCGTTTCTACACTC
GGAAAGATGTCAGGCGGCACATGGTTGTCCACACAGGCCGAAAGGACTTC
CTGTGCCAGTACTGTGCCCAGCGCTTTGGCAGAAAGGATCATCTGACACG
GCATGTGAAAAAAAACCCCTCCCAAGAACTGCTGAAGAATAAGAACGAGC
CTCCGGATATGTTGGGCTTCCTGGGATCTGGGTCGCCGCCTTGCTCTGTC
AAGGAAGAGCTTAGCCCCATGATGTGTGGTATGGGTCCCAATAAAGACCC
CATGATGGGCAAATCCTTCCCCAGTGGGACCCCCTTTCCCATGAGCATGT
ACAACCCCCACCAT------CTCCAGGCCATGTCTAATTCTGGGGTGGCT
CACCCC------CACCCCTCCCTGATGCCCAGTTCCCTGTCTGCAGCTAT
GGGCATGGGCTGCCACATGGAATATCTCATCTATGCTTCCTTCTCCTTCA
TGGGATGTTTACAAATCAGCGACGGGTCGAACATCGTGAATTTGCTGGCC
AGTAACTCTCCGAGCGTCTCGTACGCCCTGACACAGCAGAAGTACTTCAG
TAACTACAGCCCCGTGATCGGGTTTTACATCTACGAGCCCATTGAGTACT
GGAACTCCACGGTGCAGGAGCACCTGAAGACGCTGAGCCACGGCTTCAAC

AAGATCTCCTGGATGGACAATTTCTTCCACTACCTGCGGGTGGTGAACGT
GACTGCGTCAACCAAGGCCGACTTCATCACCGTCCTCAAGGGCTCCTTCC
TGCGGAGCCCGGCGTACGAGCACTTCACAGAGGACATCATCTTTTCCAAA
A---GCCGGGAGAACA------ACGAGTACGATATCATCGCCTCGCGCAT
GTACCTGGTGGCGCGCACCACGGAGAAGAAGCGCGACGATGTGGTGGAGC
TGCTGGAGAAGCTGAGGCCGTTGATGCTGATCAACAGCATCAAGTTCATC
GCCTTCAACCCCACGTTCGTCTTCATGGACCGCTACAGCTCCTCTGTCAT
CTCGCCCATTCTCACCTCAGGCTTCAGCGTGCTCACCATCCTCATCCTCA
CCTTCTTCCTGGTCATCAACCCGCTGGGGAACTTGTGGCTCATCCTGACG
GTCACGTCCGTGGAGCTGGGTGTGCTGGGTCTGATGGGCTACCACACATT
TGAGTGGCAGCCAGCCCTCAAGGATGTGTCCCCATCCTGCCATGTGGGTA
TCATCAATGGGCTCTCCGGGTGGGCTGCCTCGGTGGATGATGCCCCTGCT
GATACCATCACTCGTCGGTTTCGCTATGACGTGGCCCTGGTGTCAGCCCT
GAAGGACCTAGAAGAGGACATCATGGAAGGACTGAAGGAGCGTGGGCTGG
AAGACAGTGCTTGCACCACAGGCTTCAGTGTTATGATCAAGGAATCCTGT
GATGGCATGGGAGATGTCAACGAGAAGCACGGTGGAGGACCAGCGATACC
TGAGAAGGCTGTGCGTTTCTCTATCACCGTCATGTCTGTCTCTGTCCAGG
CCGACGGAGAGGAG------------------------------GAGGCG
GTTGTCATCTTCAGGGAGTCAAAGCCCAACTCTGAACTGTCCTGTAAGCC
CCTGTGCCTAATGTTTGTGGATGAGTCTGACCACGAGACACTCACCGCTG
TCCTGGGGCCTTTGGTAGCAGAGAGGAATGCAATGAAGCAGAGCCGACTC
ATCCTTTCAGTGGGTGGCCTCCCCCGCTCTTTCCGCTTCCATTTCAGAGG
CACAGGCTATGATGAGAAGATGGTGCGAGAGATGGAGGGCCTGGAGGCCT
CAGGCTCCACCTATATCTGCACTCTTTGTGACTCCACTCGGGCAGAAGCC
GCTCACAACATGGTGCTCCACTCTGTCACCCGCAGCCACGATGAGAACCT
GGAGCGCTACGAGATATGGAGGACCAACCCCTTCTCCGAGTCTGTCGTGG
AGCTGCGAGACCGGGTCAAAGGAGTCTCTGCCAAACCATTTATGGAGACA
CACCCAACTCTGGATGCATTACACTGTGACATAGGTAACGCCACTGAATT
CTACAAAATCTTCCAGGATGAGATTGGGGAGGTGTTCCGGAAGGC---CA
AC---CCTAGCCGGGAGGAACGGCGCAGCTGGAGGGCAGCCCTCGATAAG
CAGCTGAGGAAGAAATTGAAGCTGAAACCAGTGATGCGGATGAATGGGAA
CTATGCCCGAAGGTTGATGACCCAGGAGTCTGTGGAGGTGGTGTGTGAAC
TGGTGCCCTCAGAAGAGAGGCAGGAGGCCCTGAGGGAGCTGATGGGGCTC
TATATCCAGATGAAGCCTGTGTGGCGTGCTACCTGCCCAGCCAAGGAGTG
CCCTGACCAGCTGTGCCGCTACAGCTTTAACTCTCAGCGCTTTGCTGATC
TCCTCTCCTCCGCCTTTAAATACAGGTATGATGGAAAGATCACCAACTAC
CTGCACAAGACCTTGGCCCATGTCCCTGAAATTATAGAGAGAGATGGCTC
CATTGGAGCCTGGGCCAGCGAGGGGAATGAGTCTGCGAACAAG-------

--------------ACAGGCGAAGTCACAGAAAGAGAAGTGGCTTTGGGG
ATAAATCCGTTCGCAGACGGGATGGGCGCCTTCAAAATAAACCACAGCTC
TCATGACATTGGCTCCGG---GCAAACGGCGTTTTCCTCCCAGGCG---C
CCGGCTAC---GCAGCGGCTGCCCTGGGA---CACCATCA------CCAC
CCGACACACGTTAGCTCT---TATTCCACTGCAGCTTTCAATTCCACCCG
AGACTTTCTCTTCAGAAATCGGGGCTTCGGAGACGCGACCAG--------
----CGCGCAGCACAGTTTGTTCGCTTCCGC---CGCGGGAAGTTT---T
------GCAGGGCCACATGGACACTCAGATGCCGCGGGCCACCTGCTCTT
CCCTGGGCTTCATGAG---CAAGCCGCGAGCCATGCGTCGTCTAACGTTG
TAAACAGCCAGATGCGACTGGGCTTTTCTGGGGACATGTACGGGCGGGCC
GACCAGTATGGCCACGTTACAAGCCCAAGGT---CCGACCACTATGCTTC
GACCCAATTGCATGGCTATGGTCCTATGAACATGAATATGGCCGCT---C
ACCACGGAGCAGGGGCCTTCTTTCGCTACATGAGGCAGCCGATTAAACAA
GAGCTAATCTGCAAGTGGATCGAACCGGAGCAACTGACGAATCCCAAAAA
GTCGTGCAACAAAACTTTTAGCACGATGCACGAGCTCGTGACCCATCTGA
CGGTGGAGCATGTGGGGGGACCAGAGCAGTCGAACCACATTTGCTTCTGG
GAAGACTGTTCCCGAGAAGGGAAACCATTCAAAGCCAAATACAAACTTGT
AAATCATATCAGAGTTCACACCGGAGAAAAACCGTTCCCGTGTCCATTCC
CCGGCT--------
>Benthalbella dentata

NNNNNNNNNNNNNNNNNNNNNNNNNNNNNNNNNNNNNNNNNNNNNNNNNNNNNNNNNNNNNNNNNNNNNN
NNNNNNNNNNNNATGCTTTCGTGATAATCTTTTTTATAGTAATGCCGGTTATGATCGGCGGG
TTCGGAAACTGACTTATCCCCCTAATGATCGGGGCCCCTGACATAGCCTT
CCCCCGAATAAATAATATAAGCTTCTGACTCCTCCCTCCTTCCTTCCTCC
TCCTCCTGGCCTCCTCTGCAGTAGAAGCGGGTGCCGGCACAGGATGAACA
GTATATCCACCTCTCGCTAGTAACCTTGCACATGCCGGAGCCTCCGTTGA
CTTAACTATTTTTTCACTCCACCTGGCAGGAATCTCCTCTATCTTAGGTG
CTATTAATTTCATTACAACTATTATTAACATAAAACCCCCTGCCATTACC
CAGTATCAAACCCCCCTATTTGTATGAGCTGTTCTGATTACTGCCGTACT
TCTCCTACTATCCCTACCTGTCTTAGCGGCCGGCATTACAATGTTACTGA
CAGACCGAAATTTAAATACAACCTTCTTCGACCCTGCAGGAGGAGGTGAC
CCAATCCTCTACCAACATCTATTCTGATTCTTTGGTCACCCAGAAGTTTA
TATTCTCATCCTTCCCGGCTTTGGGATAATCTCCCACATTGTTGCCTATT
ATTCAGGGAAAAAAGAACCCTTCGGCTACATGGGCATGGTATGAGCCATG
ATAGCAATCGGCCTTCTCGGGTTTATTGTCTGAGCCCACCATATGTTTAC
AGTCGGATAGACGTAGACACACGTGNNNNTTCCTCGAGAGGAACCTGCACCCA
TCCAACTGCCTTGGCATGCTGGTACTGTCAGATGCCCACCAGTGCACCAA
GCTGTTAGAGCTGTCCTGGGGTATGTGTCTTAGCAACTTCCCTGCCATCT
GCAAGACAGAGGACTTCCTCCAGCTTCCCAAAGACATGGTGGTGCAGCTC
CTGTCCCATGAAGAGCTGGAGACGGAAGATGAGAGACTGGTTTATGAGGC
TGCCCTTAACTGGGTCAACTATGACCTGGAGAGGAGACACTGCCACCTGC
CAGAGCTGCTGAGAACCGTACGCCTGGCCTTGCTTCCCGCCATCTTCCTC
ATGGAGAACGTCTCCACAGAGGAGCTGATCAATGCCCAGGCAAAGAGCAA
GGAGTTGGTGGACGAGGCCATCCGCTGCAAGCTGAAGATCCTGCAGAATG
ACGGTGTGGTCAACAGTCCCTGTGCCCGGCCCAGAAAGACCAGCCACGCC
CTTTTCCTGCTGGGAGGGCAGACCTTCATGTGTGACAAGCTGTACTTGGT
GGACCAGAAGGCCAAAGAGATCATCCCCAAGACGGACATCCCCAGCCCCA
GGAAGGAGTTCAGCGCCTGTGCCATTGGCTGTAAGGTCTACGTCACAGGC
GGGA--GAGGCTC-AGAGAACGGCGTATCTAAAGACGTGTGGGTCTATGA
CACCGTCCATGAGGAGTGGTCCAAGGCAGCACCCATGCTCATTGCCAGGT
TTGGCCATGGCTCTGCTGAGCTCAAACACTGCCTCTATGTGGTTGGAGGA
CACACAGCAGCCACTGGCTGCCTCCCAGCCTCTCCATCTGGATGAGTACA
TTGTAGTGTTCAGTCGTTCCACGACGAGGCTCATTCTGAACGAAGCAGAG
CTGATACTGGCACTGGCCCAAGAGTTTCAGATGAGGGTGGTCACAGTCTC
CCTGGAGGAACAGTCTTTCCCCAGCATCGTACAGGTCATCAGTGGAGCCT
CCATGTTGGTCAGTATGCACGGAGCTCAGCTCGTCACCTCACTCTTCCTC
CCTAGAGGAGCTGCTGTGGTAGAGCTCTTCCCCTACGCTGTGAACCCGGA
ACAGTACAGCCCATACAAAACCCTAGCCTCCCTACCAGGCATGGACCTTC
AATATGTTTCCTGGAGGAACACTGTTGAGGAGAACACTGTCACCCACCCA
GACAGACCCTGGGACCAAGGAGGCATTGCCCATTTGGAGAAAGAAGAGCA
GGAGAGAATCCTAGCCAGCAAGGATGTCCCCAGGCACCTATGCTGCCGCA
ACCCAGAGTGGCTCTTCAGGATCTACCAGGACACTCTGGTAGACATCCCC
TCCTTCCTGGAAACCCTCAA---GGAGGGCCTAAAG---ACGAGGCCGAG
CTTGAAGAA---GTCCAAGCCGGCCAGCACGGTTCACCCAGGCCGGGTCA
GAGAACCCCAGTGCCAGACTTCGGTCCAAGCCACCAACGAGGCTAAACTC
ACAGTATCCTGGCAGATCTCGTGCAACCTTAAGTACCTGACG--------
--------------------------AAAAAGGATACAAGCAAGGAACCC
TGGAGGATCAAATCATTCAGGCAAACCCTGCACTGGAGGCTTTTGGCAAT
GCCAAAACATTGAGGAACGATAATTCCTCCCGTTTTGGAAAATTCATCCG
AATCCACTTTGGAACCAGTGGTAAACTGTCCTCTGCGGACGTTGAGACCT
ACCTGCTGGAGAAGTCACGGGTCACTTTTCAGCTTAAGGCAGAGAGGAAC
TACCACATCTTTTTCCAGATCTTGTCCAATCAAAAGCCAGAGCTGCTGGA
CATGATGTTGATCACCAACAACCCCTATGACTACTGCTACATCTCCCAAG

GAGAAGTAACCGTAGCATCCATCAATGATTCAGAAGAGCTGATGGCCACT
GACAGTGCCTTTGATGTGCTTGGCTTCACGCAAGAGGAGAAGATGGGAGT
CTACAAGTTGATAGGGGCCATTATGCACTATGGCAACATGAGGTTCAAGC
AAAAGCAGCGTGAGGAACAGGCTGAGTCTGATGGTACAGAGGCTGCTGAT
AAGTCAGCTTATCTAATGGGGCTGAATTCAGCAGACCTAATCAAAGGACT
CTGCCATCCCAGAGTCAAGGTAGGAAATGAGTATGTCACCAAAGGCCAGG
GTGTAGATCAAGTCTACTACCCCAACAAGGAGGCCTTCAAGTGTGAAGAG
TGTGGCAAGCACTACAACACCAAGCTAGGATACAAGCGCCATGTGGCCAT
GCACTCTGCCACAGCGGGGGATCTCACCTGCAAAGTGTGCATGCAGAGTT
ACGAGAGCACCCCGGTGCTCCTGGAACACCTCAAGAGCCACTCGGGGAAG
TCCTCGGGTGGCGCCAAGGAGAAGAAGCACCCGTGCGACCACTGCGACCG
TCGCTTCTACACGCGGAAGGATGTCAGGCGGCACATGGTTGTCCACACGG
GCCGAAAGGACTTCCTGTGCCAGTACTGTGCCCAGCGCTTCGGCAGGAAG
GATCATCTGACACGGCACGTGAAAAAGAGCCACTCGCAGGAGCTGCTGAA
GATTAAGACGGAGCCTCCGGATATGTTGGGTCTCCTTGGCTCTGGCTCGC
CGCCTTGCTCTGTCAAGGAGGAGCTGAGCCCCATGATGTGCAGCATGGGT
GCCAATAAAGACCCCATGATGGGCAAACCCTTCCCCAGCGGGACCCCCTT
CCCCATGGGCATGTACAACCCCCACCAC------CTCCAGGCCATGTCTA
ATCCTGGGGTGGGCCACCCC------CACCCCTCCCTTATGCCTGGGTCC
CTGTCTGCAGCTATGGGCATGGGCTGTCATATGGAATATCTTATCTACGC
TTCCTTCTCCTTCATGGGATGTTTACAAATCAACGACGGGTCGAACATCG
TGAATTTGCTGGCCAGTAACTCTCCGAGCGCCTCGTACGCCCTGACACAG
CAGAAGTACTTCAGTAACTATAGCCCCGTGATCGGGTTTTACATCTACGA
GCCCATCGAGTACTGGAACTCCACGGTGCAGGAGCACCTGAAGACGCTGA
GCCACGGCTTCAACAAGATCTCCTGGGTGGACAATTTCGTCCACTACCTA
CGGGCGGTGAACGTGAGCGCGTCTACCAAGGCCGACTTCGTCACCGTTCT
CAAGGGCTCCTTCCTGCGGAGCCCGGCGTACCAGCACTTCACCGAGGACA
TCATCTTCTCCAAGA---GCCACGAGAACA------GCGAGTACGACATC
ATCGCCTCGCGCATGTACCTGGTGGCGCGCACCACGGAGAAGAGGCGCGA
GGACGTGGTGGAGCTGCTGGAGAAGCTCCGGCCGCTGATGCTGATCAACA
GCATCAAGTTCATCGCCTTCAACCCCACGTTCGTCTTCATGGACCGCTAC
AGCTCCTCGGTCATCTCGCCCATCCTCACCTCGGGCTTCAGCGTGCTCAC
CATCCTCATCCTCACCTTCTTCCTGGTCATCAACCCGCTGGGGAACTTGT
GGCTCATCCTGATGGTCACGTCCGTGGAGCTGGGCGTGCTGGGTCTGATG
GGCTACCACACATTTGAGTGGCAGCCAGCCCTCAAGAATGTGTCCCCATC
CTGCCACGTGGGTATCATCAATGGGCTTTCCGGGTGGGCCGCCTCGGTGG
ATGACGTCCCTGCCGACATCATCACCCGTCGGTTTCGCTATGATGTGGCC
CTTGTATCAGCCCTGAAGGACCTAGAGGAGGACATCATGGAAGGACTGAG
GGAGCGCGGGCTGGAAGACAGCGCTTGCACCTCAGGCTTCAGTGTTATGA
TCAAGGAATCCTGTGATGGCATGGGAGATATCAGCGAGAAGCACGGTGGA
GGGCCAGCGATGCCTGAGAAGGCTGTGCGTTTCTCTATCACCGTCATGTC
TGTCTCTGTCCAGGCCGACGGAGAGGAG----------------------
--------GAGGCGGTTGTCATCTTCAGGGAGCCAAAGCCCAACTCTGAA
CTGTCCTGTAAGCCCCTGTGCCTGATGTTTGTGGATGAGTCTGACCACGA
GACACTCACTGCTGTCCTGGGGCCTTTGGTAGCAGAGAGGAATGCGATGA
AGCAGAGCCGACTCATCCTCTCAGTGGGTGGCCTCCTCCGCTCTTTCCAC
TTCCACTTCAGAGGCACAGGCTATGACGAGAAGATGGTGCGAGAGATGGA
AGGCCTGGAGGCCTCAGGCTCCACCTATATCTGCACTCTTTGTGACTCCA
CTCGGGCAGACGCCTCTCACAACATGGTGCTCCACTCTGTCACGCGCAGC
CACGATGAGAACCTGGAGCGCTACGAGATATGGAGGACCAACCCCTTCTC
AGAGTCTGTCATGGAGCTGCGAGATCGGGTCAAAGGGGTCTCTGCCAAAC
CATTTATCGAGACCCATCCGACTATGGATGCATTACATTGTGACATAGGT
AATGCCACTGAATTCTACAAAATCTTCCAGGATGAGATTGGGGAGGTGTT

```
CCGGAAGGC---CAGC---CCTAGCCTGGAGGAACGGCGCAGCTGGAGGG
CAGCCCTCGATAAACAGCTGAGGAAGAAGTTGAAGCTGAAACCAGTGATG
CGGATGAATGGGAACTATGCCCGAAGGTTGATGACCCAGGAGGCTGTAGA
GGTGGTGTGTGAACTGGTGCCCTCAGAAGAGAGACGGGAGGTCCTGAGGG
AGCTGATGGGGCTCTATATACAGATGAAGCCGGTGTGGCGTGCTACCTGC
CCAGCCAAGGAGTGCCCTGACCAGCTGTGCCGCTACAGCTTTAACTCTCA
GCGCTTTGCTGATCTCCTCTCCTCCACCTTTAAATACAGGTATGATGGAA
AGATCACCAACTACCTGCACAAGACCCTGGCCCATGTCCCTGAAATTATA
GAGCGAGATGGCTCCATTGGAGCCTGGGCCAGCGAGGGGAATGAGTCTGC
GAACAAG-------------------------------------------
--------------------------------------------------
--------------------------------------------------
--------------------------------------------------
--------------------------------------------------
--------------------------------------------------
--------------------------------------------------
--------------------------------------------------
--------------------------------------------------
--------------------------------------------------
--------------------------------------------------
--------------------------------------------------
--------------------------------------------------
--------------------------------------------------
--------------------------------------------------
--------------------------------------------------
--------------------------------------------------
--------------------------------------------------
--------------------------------------------------
--------------------------------------------------
--------------------------------------------------
--------------------------------------------------
--------------------------------------------------
--------------------------------------------------
--------------------------------------------------
--------------------------------------------------
--------------------------------------------------
--------------------------------------------------
--------------------------------------------------
--------------------------------------------------
--------------------------------------------------
--------------------------------------------------
--------------------------------------------------
--------------------------------------------------
--------------------------------------------------
--------------------------------------------------
--------------------------------------------------
--------------------------------------------------
--------------------------------------------------
--------------------------------------------------
--------------------------------------------------
--------------------------------------------------
--------------------------------------------------
--------------------------------------------------
```


-------------------CATCACTCAACAGGCGAAGTCACAGAAAGAG
AAGTGGCTTTGGGGATAAATCCGTTCGCAGACGGGATGGGCGCCTTCAAA
ATAAACCACAGCTCTCATGACATTGGCTCCGG---GCAAACGGCGTTTTC
CTCACAGGCG---CCCGGCTAC---GCAGCGGCTGCCCTGGGA---CATC
ATCA------CCACCCGACCCACGTTAGCTCC---TATTCCACGGCAGCT
TTCAATTCCACCCGGGACTTTCTCTTCAGAAATCGAGGCTTCGGAGACGC
CACCAG------------CGCGCAGCACAGTTTGTTCGCCTCCGC---CG
CGGGAAGTTT---T------GCAGGGCCACATGGACACTCAGATGCCGCG
GGCCACCTGCTCTTCCCTGGGCTTCACGAG---CAAGCCGCGAGCCATGC
CTCGTCTAACGTTGTGAACAGCCAGATGCGACTGGGCTTTTCCGGGGACA
TGTACGGGCGGGCCGACCAGTATGGCCACGTTACAAGCCCGAGGT---CC
GACCACTATGCTTCGACCCAATTGCATGGCTATGGCCCTATGAACATGAA
TATGGCCGCT---CACCACGGAGCAGGGGCCTTCTTTCGCTACATGAGGC
AGCCGATCAAACAAGAGCTCATCTGCAAGTGGATCGAACCGGAGCAACTG
ACGAATCCGAAAAAGGCGTGCAACAAAACTTTTAGCACGATGCACGAGCT
CGTGACCCACCTGACGGTGGAGCATGTGGGTGGACCGGAGCAGTCGAACC
ACATTTGCTTCTGGGAAGATTGTGCCCGAGAGGGGAAACCGTTCAAAGCC
AAATACAAACTTGTAAATCATATCAGAGTTCACACCGGAGAAAAACCGTT
CCCGTGTCCATTCCCCGGCT--------
>Benthosema glaciale
AGTCTCCTAATTCGAGCCGAGCTCGCCCAACCTGGGGCCCTCTTAGGGGA
CGACCAGATTTATAATGTAATCGTGACAGCTCACGCCTTTGTAATAATCT
TCTTCATAGTAATGCCAATCATAATCGGCGGGTTCGGAAACTGACTAATC
CCCCTAATGATTGGGGCCCCTGACATGGCATTCCCCCGAATAAATAACAT
AAGCTTCTGACTCCTCCCCCCATCTTTCCTTCTTCTCCTGGCCTCCTCTG
GCGTAGAAGCTGGGGCCGGAACTGGCTGAACAGTCTACCCTCCGCTTGCC
GGAAACCTCGCCCACGCCGGGGCCTCTGTTGATTTAACAATTTTCTCACT
CCATCTGGCCGGTGTCTCCTCAATTTTAGGGGCAATTAACTTCATTACGA
CTATTATCAATATAAAACCCCCTGGAACCTCCCAATACCAAACCCCTCTA
TTTGTATGGGCCGTCCTCATCACCGCTGTTCTCCTTCTCCTCTCCCTCCC
CGTTCTGGCCGCTGGTATCACAATACTGTTAACAGACCGAAACCTAAACA
CTACCTTCTTCGACCCCGCCGGCGGGGGCGACCCTATCCTCTATCAACAC
TTATTCTGATTCTTTGGGCACCCCGAAGTTTATATTCTTATCCTCCCCGG
CTTTGGCATGATCTCCCACATCGTTGCCTACTACTCGGGCAAAAAAGAGC
CCTTTGGATACATAGGCATGACCTGGGCAATAATGGCCATCGGGCTCCTT
GGCTTCATTGTATGAGCACACCACATGTTTACTGTCGGAATAGACGTGGA
CACACGAGCATA--------------------------------------


```
--------------------------------------------------
--------------------------------------------------
--------------------------------------------------
-------------------------GGATGAGTACATCGTAGTCTTCAGT
CGCTCAACGACGAGACTCATTCTCAACGAAGCAGAACTGATTATGGCACT
GTCCCAGGAGTTTCAGATGAGGGTGGTCACAGTGTCCCTTGAAGAGCAGT
CTTTCCCCAGTATAGTCCAGTTTATCAGTGGTGCCTCAATGTTGGTCAGT
ATGCATGGGGCTCAGCTCGTCACCTCACTCTTCCTCCCCAGAGGAGCTGT
GGTGGTGGAGCTCTTTCCCTATGCTGTGAACCCAGAACAGTACACCCCTT
ATAAGACCCTCGCCTCCTTACCGGGGATGGACCTGCAGTATGTTTCCTGG
AGGAATATGATGGAGGAAAACACCATCACCCACCCAGATAGATCCTGGGA
CCAAGGAGGCATCGCCCACCTGGAAAAGGACGAGCAAGAGCGAATCCTTG
CCAGCAAGGATGTCCCGCGGCACCTGTGTTGCCACAACCCAGAATGGTTC
TTCCGAATCTACCAGGACACTCTGGTGGACATCCCCTCATTTCTGGAGGT
TCTCAA---AGATGGCCTGAAGATCACCAGACCAAGCTTAAAAAA---GG
CCAAGCCGGCCAGCACAGTGCATCCAGGCCGGGTCAGGGAAGCACAGTGT
CAAACCTCAGTCCAAGCCACCAACGAAGCTAAACTTACGGTCTCCTGGCA
GATCCCATGGAACTTGAAATACCTGAAGGTGAGAGAGGTGAAGTACGAGG
TGTGGATCCAG---------------------------------------
--------------------------------------------------
--------------------------------------------------
--------------------------------------------------
--------------------------------------------------
--------------------------------------------------
--------------------------------------------------
--------------------------------------------------
--------------------------------------------------
--------------------------------------------------
--------------------------------------------------
--------------------------------------------------
--------------------------------------------------
--------------------------------------------------
--------------------------------------------------
--------------------------------------------------
--------------------------------------------------
--------------------------------------------------
--------------------------------------------------
--------------------------------------------------
--------------------------------------------------
--------------------------------------------------
--------------------------------------------------
--------------------------------------------------
--------------------------------------------------
--------------------------------------------------
----------------------TATCTGATCTACGCTTCATTCTCCTTCA
TGGGATGTTTACAAATCAGCGATGGGTCAAACATTGTGAACCTGCTGGCG
AGTAACTCTCCAAGTGTGTCTTTTGCRCACACCCAGCAGAAGTACTTCAG
TAACTACAGCCCTGTTATTGGGTTCTACATTTATGAACCCATTGAGTACT
GGAATGCCACGGTGCAGGAGCACCTGAAGACACTGAGTCACGGCTTCAAC
AAGATCTCCTGGATCGACAACTTCTTCCACTACCTGCGGGTGGTAAACGT
GAGCGCGTCGACCAAGAACGACTTCATCACCATCCTGAAGGGCTCCTTCC
TACGCAGCCCGGAGTACCAGCACTTCACAGAGGACATCATCTTCTCCAAG
A---ACCGCGAGAGCG------ACGAGTACGACATCATCGCCTCGCGCAT
```

GTACCTGGTGGCGCGCACCACCGAGAAGAAACGCGAGGAGGTGGTGGAGC
TGCTGGAGAAGCTGCGTCCGCTGATGCTGATCAACAGCATCAAGTTCATC
GCCTTCAACCCCACCTTCGTCTTCATGGACCGCTACAGCTCGTCGGTCAT
ATCGCCCATCCTTACCTCAGGCTTCAGCGTGCTCACCATCCTCATCCTCA
CGTTCTTTCTCGTCATCAACCCACTGGGGAACTTCTGGCTCATTCTGACG
GTGACGTCTGTGGAGCTGGGCGTGTTGGGTCTGATGGGCTACCACCCTTT
TGAATGGCAGCCAGCCCTCAAGAGCGTGTCCCCCTCTTGCCATGTGGGCA
TCATCGACGGGCTCTCTGGCTGGGCCGACTCGGTGGATGACTCACCAGCG
GACACAATCAGCCGGAGGTTTCGCTATGATGTGGCCCTTGTGTCTGCCTT
GAAGGACCTGGAAGAGGAAGTGATGGAGGGACTGAGAGAATGCGGGCTAG
ATGACAGCGCGTGCACCTCAGGCTTCAGAGTATTGATCAAGNAGTCCTGCG
ATGGCATGGGAGANGTCAGTGAGAAACATGGCGGGGGGCCCGCGGTTCCTG
AGAAAGCGGTCCGTTTCTCTTTCACCGTTATGTCCATCTCTGTGATGCCG
GATGGGAAAACG------------------------------GAGGCGGT
GACCATCTTCACAGAGCGAGAGCCCAACTCCGAACTGTCCTGTAAGCCCA
TGTGCNTGATGTTTGTGGATGAGTCGGACCACGAGATGCTCACTGCCGTTT
TGGGGCCCGTGGTCGCAGAGCGCAGGGCAATCACAGAAAGCCGGCTCATC
CTGTCCATGGGTGGCCTCCCCCGCTCGTTCCGCTTCTACTTCAGAGGCAC
AGGCTATGATGAGAAGATGGTGCGTGAGATGGAGGGCCTGGAGGCATCAG
GCTCTGCCTATGTCTGCACTCTGTGCGACTCCACCCGGGCGGAGGCCTCT
CGCAACATGGTGCTCCATTCCGTCACCCGCAGCCACGGCGAGAACCTGGA
GCGCTACGAGATATGGCGGAGCAACCCCTTTGCGGAGTCTGTCGAGGAGC
TGCGAGATCGAGTGAAGGGGGTCTCCGCCAAGCCCTTCATGGAAACCCAG
CCCACTCTGGATGCATTGCACTGTGACATTGGTAACGCCACTGAGTTCTA
CAAAATATTCCAAGACGAGATCGGGGAGGTGTTCCAGAAAGC---CAAG-
--CCCAGCCGCGAGGAAAGGCGAGGCTGGAGGGCTGCTCTCGACAAGCAG
CTGAGGAAAAAACTCAAGCTCAAGCCGGTGATGAGGATGAATGGGAACTA
CGCTCGGAGACTGATGTCCGAGGAGACCATGGATGTGGTGTGCGAACTGG
TGCCTTCAGAGGAAAGGCGGGAGGCTCTGAGGGAGCTAATGGGGCTTTAC
CTCCAGATGAAACCTGTGTGGCGTGCCAGCAACCCAGCCAAAGAATGCCC
TGACCAACTGTGTCGCTACAGTTTCAACTCCCAGCGCTTTGCAGATCTCC
TCTCCACTACCTTCAAATACAGGTATGACGGAAAGATTACCAATTACCTG
CACAAGACCTTGGCCCACGTACCTGAAATAGTAGAAAGAGACGGCTCTAT
TGGAGCATGGGCCAGCGAGGGGAACGAGTCGGCAAACAAATCGTACACCA
TCGAGATGGGCCCCAAAGGGCCATTGTGGAAGGAGAGCCCGCAGCCCTTC
ACCTGTTCCATCGAGGACCCCACTAAACAGACCAAGTTCAAGGGCATCAA
GACGTACATCTCCTACCGGGTCACGCCGAGCCACACMGGTCATCCTGTGT
ACAGGCGCTACAAGCACTTTGACTGGCTGTACAACCGCTTGCTGCACAAG
TTCACCGTGATCTCCATCCCACACTTGCCAGAAAAACAGGCAACTGGGCG
TTTCGAGGAGGACTTCATAGAAAAGCGCAAGAGGCGGTTGATCCTCTGGA
TAAACCACATGACCAGTCACCCTGTCCTCTCCCAGTACGAGGGGTTCGAA
CACTTCCTGATGTGTGGCGACGACAAGCAATGGAAGCTGGGCAAGCGGCG
AGCAGAGAAGGATGAAATGATCGGTGCCCACTTCATGCTGACCTTCCAGA
TCCCCAACGAGCACCAGGACCTGCAGGATGTGGAGGAGCGCGTAGACTCC
TTCAAGGCGTTTGCCAAGAAGATGGATGACAGTGTCATGCAGCTGACTCA
TGTGGCCTCGGAACTCGTGCGCAAGCACCTTGGTGGATTCCGTAAGGAGT
TCCAGCGGCTGGGTAATGCCTTCCAGAACATCAGCCAGGCCTTCATGCTT
GACCCGCCACATAGCTCGGACGGCCTCAACAACGCCATCTCACAC-----

--------------------------------GCCAAGTCCCGCTTTCAC
CCTGGCGTGGGGAGCGCTGCTGGCACGGAGC---GCAGCGTCCCACTCAG
CAACAGCTTGCTCTCTCCGCAACAAACCGAGGATCCCGCCGTCG---CCT
CCCCGCAGCGATGGTTTGTCACCC---CTGCCAACAACCGACTGGACTTC
GCAGCCTCGGCATACGACGCCGCT---------GATTTCGCCGGTAACGC
GGCCACCTTGCTGTCGTACGCCGCGGCCGGAGTGAAGGCTC------TCC
CCCTGCCGACCGCGGCCTGCTCCAACAGACCTCTGGGTTATTACGCGGAC
CCGTCGG---GCTGG---GGCGCGCGCACGCCGCCCCAGTACTGC-----
----AGCAAGTCGACCTCGGTCCTTTCTTGCTGGCCCGCGAACTCTGTCG
GGAGCAGAACGGGCA------CGAACTACCTAG---------TGGAGGA-
--CGGGTCGGACGC---CATCCCGACGGAGAGGTCCCCGATTAT---TAA
TGGCTCGGAGGAG---GTGAAAGCCAAAGACTT------GTCCGA---GT
CTAGCTGGATAGAG---ACGCCGTCTTCGATCAAATCGATTGACTCAAGT
GATTCTGGCATCTTTG---AGCAAGCAAAGCGGAGACGGATTTCACCGTC
TGCCACACCA---------GTTACAGAGACGGTGTCTCCGTTGAAATCTG
AA---------GCAGGCGAGGTCTCCGAGAGGGAGGTGGCGTTGGGGATC
AACCCGATCGCGGACGGCATGGGCGCCTTCAAAATAAACCACGGCTCCCA
CGACATCGGCTCCGG---ACAGACGGCGTTCTCCTCGCAGCCGGGCGCCG
GCTAC---GCGGCGGCCGCCCTGGGA---CACCACCA------CCACCCG
ACGCACGTCAGCTCG---TACAGCACGGCAGCCTTCAACTCCACGCGGGA
CTTTCTGTTCAGAAATCGGGGCTTCGGAGACGCCACCAG-----------
-CGCGCAGCACAGCTTGTTCGCCTCCGC---CGCGGGAAGTTT---C---
---GCGGGGCCACATGGACACTCGGATGCCGCGGGGCACCTGCTCTTCCC
CGGCTTGCACGAA---CAAGCCGCGAGCCACGCGTCCTCCAACGTGGTCA
ACAGCCAGATGCGACTGGGCTTTTCGGGGGACATGTACGGCCGGGCCGAC
CAGTATGGCCACGTCACGAGCCCGAGGT---CCGACCACTACGCGTCCAC
GCAGCTGCACGGCTACGGACCCATGAATATGAACATGGCCGCG---CACC
ACGGAGCCGGGGCCTTCTTTCGGTACATGCGGCAGCCCATCAAGCAGGAG
CTCATCTGCAAGTGGATCGAGCCGGAGCAGCTGTCGAACCCCAAGAAGTC
GTGCAACAAAACTTTCAGCACCATGCACGAGCTGGTGACCCACCTGACGG
TGGAACACGTGGGGGGCCCGGAGCAGTCCAACCACATATGCTTCTGGGAG
GAGTGCGCCCGGGAAGGGAAGCCGTTCAAAGCCAAATACAAACTGGTGAA
TCATATCAGAGTTCACACTGGAGAAAAGCCCTTTCCGTGTCCCTTCCCGG
CNN--------
>Beryx decadactylus
AGCCTCTTGATCCGAGCAGAACTAAGCCAGCCGGGGGCCCTTCTGGGAGA
TGACCAGATCTACAACGTCATTGTTACTGCACACGCCTTTGTAATAATTT
TCTTTATAGTAATGCCAATTATGATTGGAGGATTTGGAAACTGACTAATT
CCCCTAATGATTGGAGCACCCGACATGGCATTCCCCCGAATAAATAACAT

GAGCTTCTGACTTCTTCCTCCCTCCTTCCTCCTTCTCCTGGCCTCTTCTG
GAGTAGAAGCAGGGGCAGGAACAGGGTGAACTGTATACCCACCCCTTGCC
GGGAATCTTGCACACGCAGGAGCCTCGGTTGACCTTACAATCTTCTCTCT
TCACCTGGCAGGTGTCTCCTCAATTCTAGGGGCCATTAACTTTATTACAA
CCATCATCAACATGAAACCCCCAGCCATTTCACAATACCAAACACCCCTG
TTTGTTTGAGCAGTGCTGATTACGGCTGTCCTCCTTCTTCTTTCCCTGCC
CGTACTTGCGGCAGGTATCACGATACTACTAACAGATCGCAACCTAAACA
CCACCTTCTTTGACCCCGCAGGAGGAGGTGATCCAATTCTCTACCAACAC
CTG---

------------TTTCTAGAGAGGAACCTCCACCCATCTAACTGCCTTGG
CATGCTGTTGCTGTCTGATGCCCACCAGTGTACCAAGCTGTCAGAGCTCT
CCTGGAGCATGTGCCTCAGCAACTTTCCTGCTATTTGCAAGACAGAGGAC
TTCCTCCAACTGCCCAAAGACATGGTGGTGCAGCTTCTGTCCCATGAGGA
GCTGGAGACAGAAGATGAGAGACTGGTTTATGAAGCTGCCCTCAACTGGG
TCAACTACGACCTGGAAAGGAGGCACTGCCACCTGCCAGAGCTGCTGAGA
ACAGTCCGACTGGCCCTGCTTCCTGCCATATTCCTCATGGAGAATGTCTC
CACAGAAGAGCTGATCAATGCCCAGGCCAAGAGCAAGGAGCTGGTCGATG
AGGCCATCCGCTGCAAGCTGAAGATCTTGCAGAATGACGGTGTCGTTAAT
AGCCCCTGTGCTCGACCGAGAAAAACCAGCCATGCCCTCTTTCTGCTGGG
AGGGCAGACCTTCATGTGTGACAAGCTGTACCTGGTGGACCAAAAGGCCA
AAGAGATCATCCCCAAGGCGGACATCCCAAGCCCCAGGAAGGAGTTCAGC
GCCTGCGCCGTCGGCTGTAAGGTCTACATCTCAGGAGGGA--GAGGCTC-
AGAGAATGGTGTGTCCAAAGATGTATGGGTCTATGACACCGTCCATGAGG
AATGGTCCAAGGCAGCACCCATGCTCATTGCCAGGTTTGGTCATGGCTCT
CCTGAGCTGAAACACTGCCTGTACGTGGTAGGAGGTCACACAGCAGCAAC
TGGCTGCCTCCCAGCCTCTCCCTCGGGAAGAATACATTGTCGTGTTCAGT
CGTTCAACGACAAGGCTGATTCTGAACGAAGCGGAGCTAATCATGGCGCT
GGCCCAGGAATTTCAGATGAGAGTGGTTACGGTATCCCTGGAGGAACAAA
CTTTTGCCAGCATTGTCCAAGTGATCAGTGGGGCCTCCATGTTAGTCAGC
ATGCATGGAGCTCAGCTTGTCACCTCACTCTTCCTCCCCAGAGGAGCTGC
TGTGGTGGAGCTGTTCCCCTATGCTGTGAACCCAGAACAGTACACCCCAT
ACAAAACCCTTGCCTCCCTACCAGGCATGGACCTGCAATACGTTTCCTGG
AGGAACACTATGGAGGAGAACACCATCACCCACCCAGACCGACCCTGGGA
ACAAGGAGGAATCGCCCATTTGGAAAAGGACGAGCAAGAACGAATACTGG
CCAGCAAGGATGTCCCCAGGCACCTGTGCTGCCGCAACCCGGAGTGGCTC
TTCCGAATCTACCAGGACACTTTGGTGGACATCCCGTCATTGGTGGAAGT
CCTCAA---AGAGGGTCTGAGG---GCCAGGCCAAGCTTGAAGAA---GG
CCAAGCCGGCCAGCACGGTTCACCCGGGCCGGGTCAGAGAACCCCAGTGC
CACACCTCAGTCCAAGCCACCAACGAGGCAAAACTCACGGTTTCCTGGCA
GATCCCGTGGAATCTGAAGTACCTGAAGGTGCGAGAAGTGAAGTAYGAGG
TGTGGATCCAGAAGAGAGAGATACCAGCAAGGGAACACTGGAGGATCAAATC
ATCCAGGCAAACCCTGCCCTGGAGGCCTTTGGTAATGCCAAAACAGTGAG
GAATGACAACTCATCCCGTTTTGGAAAATTCATCCGAATTCACTTCGGAA
CCAGTGGTAAGCTGTCGTCTGCTGACATTGAGACTTACCTGCTGGAGAAG
TCACGTGTCACCTTTCAACTCAAGTCTGAGAGGAACTACCACATCTTCTT
CCAGATCTTGTCCAATCAGAAGCCAGAGCTGCTGGACATGCTATTGATTA
CCAACAACCCATATGACTACTCTTACATCTCCCAGGGAGAGGTAACAGTA
CAATCCATCAACGACTCAGAGGAACTGATAGCCACTGACAGCGCCTTCGA
CGTGCTTGGCTTCACTCAAGACGAGAAGATGGGAATCTATAAGCTGACCG
GTGCCATTATGCACTATGGCAACATGAAGTTTAAGCAGAAGCAGCGTGAA

GAGCAAGCTGAGCCTGACGGAACGGAGGCTGCTGATAAGTCAGCTTACCT
GATGGGGCTGAACTCTGCAGACCTCATCAAAGGGCTGTGCCATCCCAGAG
TCAAGGTAGGAAACGAATATGTCACCAAAGGCCAGGGTGTAGACCAAGTC
TACTACCCCAACAAGGAGGCCTTCAAGTGCGAGGAGTGCGGCAAGCACTA
CAACACCAAGCTGGGATATAAGCGCCATGTGGCCATGCACTCTGCCACGG
CAGGGGACCTCACCTGCAAAGTGTGCATGCAGAGCTACGAGAGCACGCCG
GTGCTGCTGGAGCACCTCAAGAGCCACTCGGGGAAGTCCTCGGGTGGCGC
CAAGGAGAAGAAACACCCGTGCGACCACTGCGACCGTCGCTTCTACACTC
GAAAGGATGTGAGACGGCACATGGTGGTCCACACGGGCCGAAAGGACTTC
CTGTGCCAGTACTGTGCCCAGCGCTTCGGCAGGAAGGACCACCTGACGCG
GCACGTCAAGAAGAGCCACTCGCAGGAGCTGCTGAAGATCAAGACGGAGC
CTCCGGATATGTTAGGTCTTTTAGGTTCTGGCTCGCCGCCTTGCTCCGTC
AAGGAGGAGCTCAGCCCCATGATGTGCAGCATGGGTCCCAACAAAGACCC
CATGATGGGCAAACCCTTCCCCAGCGGGGCCCCCTTCCCGATGGGCATGT
ACAACCCCCACCAC------CTCCAGGCCATGTCCAATACTGGGGTGGGT
CACCCC------CACCCATCCCTGATGCCCAGTCCCCTGTCTGCAGCTAT
GGGCATGGGCTGCCACATGGAATATCTCATCTACGCCTCTTTCTCGTTCA
TGGGATGTTTACAAATCAGTGATGGATCAAACATTGTGAATTTGCTGGCT
AGTAACTCTCCGAGCGTTTCATACGCTCTGACCCAGCAGAAATACTTCAG
TAACTATAGTCCCGTGATTGGGTTTTACATTTACGAGCCCATTGAGTACT
GGAACTCCACGGTGCAGGAGCATCTGAAGACACTGAGTCACGGTTTCAAC
AAGATCTCCTGGATGGACAACTTCTTCCACTACCTGCGGGTGGTGAACGT
GAGCGCGTCGACCAAGAGTGACTTTATTACCATCCTCAAGGGCTCCTTCC
TGCGCAGCCCGGAGTACCAGCACTTCACTGAGGACATCATCTTCTCCAAG
A---GCCACGAGAGCG------ATGAGTATGACATTATTGCCTCACGCAT
GTACCTGGTGGCACGGACCACGGAGAAAAGGCGYGAGGAGGTGGTGGAGC
TTCTGGAGAAGCTGCGTCCGCTGATGCTGATCAACAGCATCAAGTTCATC
GCCTTCAACCCCACCTTTGTTTTTATGGACCGCTACAGCTCCTCGGTCAT
CTCGCCCATCCTGACCTCAGGCTTCAGCGTGCTCACCATCCTCATCCTCA
CTTTCTTCCTGGTCATCAACCCCTTGGGGAACTTCTGGCTCATCCTGACG
GTTACTTCTGTGGAGCTGGGCGTCTTGGGTTTAATGNNNNNNNNNNNNNNNNNNNGCAGGCCGGCCCTC
AAGAATGTGTCTGCATCGTGCCATGTGGGCATCATTAATGGGCTCTCTGG
ATGGGCTGCCTCAGTGGACGATTCCCCAGCTGACACCATCACTCGGCGAT
TTCGCTATGATGTGGCCCTGGTGTCAGCCTTAAAGGATCTGGAGGAGGAC
ATCATGGAGGGACTGAAAGAGTGTGGGCTGGAGGACAGCGCTTGCACCTC
AGGCTTCAGTGTTATGATCAAGGAATCTTGTGACGGCATGGGAGATGTCA
GCGAGAAGCACGGCGGAGGGCCGATGGTCCCTGAGAAGGCTGTACGTTTC
TCTTTTACTGTTATGTCTGTCTCTGTCTTGGCAGAAGGAGAGGAG-----
-------------------------AAGGAGGTTACCATCTTTAGGGAGC
CAAAGCCTAACTCGGAACTGTCCTGTAAGCCCCTATGTCTGATGTTTGTG
GATGAGTCAGACCATGAGACACTCACTGCTGTCCTGGGGCCTGTAGTTGC
AGAGCGTAATGCAATGAAGCAGAGTCGACTCATCCTATCTGTGGGTGGCC
TGCCTCGCTCCTTCCGCTTCCATTTCAGAGGCACAGGATATGATGAGAAG
ATGGTGCGTGATTTGGAGGGCCTGGAGGCCTCAGGCTCCACCTACATCTG
CACTCTGTGTGACTCCAGCCGGGCAGAGGCCTCTCACAACATGGTGTTGC
ACTCCATCACCCGCGGTCATGATGAAAACCTGGAGCGTTACGAAATATGG
AGGACCAATCCTTTTTCTGAATCCGCCGAGGAGCTGCGAGATCGGGTCAA
AGGAGTCTCCGCCAAGCCCTTCATGGAGACCCACCCCACTCTGGATGCAT
TGCACTGTGACATTGGTAATGCCACTGAGTTCTACAAAATCTTCCAGGAT
GAGATAGGGGAGGTGTACCGGAAGCC---CAAT---CCCACTCGGGAACA
ACGGCGGAGCTGGAGAGCAGCTCTAGACAAGCAGCTGAGGAAGAAGATGA
AGCTTAAACCGGTGATGAGGATGAATGGGAACTATGCCCGGAAGCTAATG
ACCGAGGAGGCTGTGGAGGTGGTATGTGAGCTGGTGCCCTCAGAAGAGAG

GAGGGAGGCCCTGAGGGAGCTTATGGGCATCTACCTGCAGATGAAGCCTG
TGTGGCGCACCACTTGCCCAGCCAAGGAGTGCCCCGACCAGCTGTGCCGC
TACAGCTTTAACTCCCAGCGCTTTGCCGATCTCCTCTCCACTACCTTCAA
ATACCGGTACAATGGAAAGATTACCAACTACCTGCACAAGACGCTGGCCC
ATGTCCCTGAAATCATAGAGAGAGATGGCTCCATAGGAGCTTGGGCCAGT
GANNNNNNNNNNNNNNNNNNNNNNNNNNNNNNNNNNNNNNNNNNNNNNNNNNNNNNNNNGTGGAAGGAGA
GTCCGCAGCCGTTCTCCTGCTCCATTGAAGACCCCAC
AAAACAGACCAAGTTCAAGGGCATCAAGACCTACATCTCGTACCGGGTCA
CGCCNAGCCACACAGGGCGTCCCGTCTACAGGCGCTACAAACACTTTGACT
GGCTGTACAACCGCTTGCTGCACAAGTTCACTGTGATCTCGGTGGCCCAC
CTGCCGGAGAAGCAGGCCACGGGGCGCTTCGAGGAGGACTTCATCGAGAA
ACGCAAAAGACGACTGATCCTGTGGATGAACCACATGACCAGCCACCCGG
TCCTCTCCCAGTATGAGGGCTTTGAGCACTTCCTGATGTGCGCTGACGAC
AAGCAGTGGAAGCTGGGTAAGAGGCGGGCGGAGAAGGACGAGATGGTGGG
CGCCCACTTCATGCTGACCTTCCAGATTCCTAACGAGCACCAGGACCTGC
AGGATGTCGAGGAGCGAGTCGACTCCTTCAAGTCCTTCGCTAANAAAATGG
ACGACAGCGTCATGCAGCTCACGCACGTTGCCTCGGAGCTGGTGCGTAAA
CACCTGGGTGGGTTCAGGAAGGAGTTCCAGCGGCTGGGAAATGCCTTCCNNNC
CATCAGCCAGGCGTTCATGCTGGACCCTCCCCACTGCTCTGAATCTCTCA
ACAGCGCCATCTCCCACNNNNNNCGCACGTTCCTC-AACTGACCTCTCTGGGTTTC
ATCATCGGAGTCGGCGTGGTCGGAAACCTCCTGATCTCCATCCTGCTGGT
CAAAGACAAGAGCCTGCACCGCGCGCCCTACTACTTCCTGCTGGACCTGT
GCGCCTCCGACATCCTGCGCTCTGCCATCTGCTTCCCCTTCGTCTTCACC
TCCGTCAAGAATGGATCCGCCTGGACCTACGGCACGCTCACCTGCAAAGT
GATTGCCTTCCTGGGCGTGCTCTCCTGTTTCCACACGGCGTTCATGCTAT
TCTGCGTCAGCGTCACCCGCTACCTGGCCATCGCGCATCACCGTTTCTAC
ACCAAGAGGCTGACCTTCTGGACCTGTCTGGCCGTCATCTGCATGGTGTG
GACGTTGTCGGTGGCCATGGCTTTCCCCCCGGTGCTGGACGTAGGGACGT
ACTCCTTCATCCGGGAGGAGGACCAGTGCACATTCCAGCACCGTTCCTTC
AGGGCCAATGACTCGCTGGGCTTCATGCTCCTGCTGGCGCTCATCCTCCT
GGCCACACAGCTGGTTTACCTCAAGCTCATCTTCTTTGTCCACGACCGTC
GGAAGATGAAACCCGTCCAGTTCGTGCCCGCTGTCAGCCAGAACTGGACC
TTCCATGGGCCGGGCGCCAGCGGGCAGGCGGCGGCCAACTGGCTCGCCGG
ATTTGGGAGAGGCCCCACCCCGCCTACCTTGCTGGGCATCCGGCAGAACA
GCAACGCAGCGGGCCGCAGGCGTCTACTGGTGTTGGATGAGTTCAAAACA
GAGAAGAGGATTAGTAGGATGTTCTACATCATGACGTTTTTCTTCCTGGC
GCTGTGGGGGCCCTATCTGGTAGCCTGCTACTGGCGGGTGTTTGCGAGGG
GCCCGGTGGTCCCCGGGGGCTACCTGACGGCAGCCGTGTGGATGAGCTTC
GCCCAGGCTGGGGTCAATCCCTTCATCTGCATCTTCTCTAACANNNNN-------

>Betta splendens
AGTTTGCTTATTCGGGCAGAACTAAGCCAGCCAGGATCTCTTCTAGGGAA
TGATCAGATTTATAATGTAATTGTTACGGCACACGCTTTTGTAATAATTT
TTTTTATGGTAATGCCTATAATAATTGGAGGTTTTGGGAATTGACTTATT
CCCCTGATGATTGGAGCCCCGGACATAGCCTTTCCCCGTATAAATAATAT
AAGTTTTTGACTTCTCCCTCCTTCCTTCTTGCTTTTACTTACATCTGCCG
GTGTAGAGGCTGGGGCAGGTACCGGGTGAACTGTGTATCCCCCCCTATCT
GGTAACTTGGCACACACAGGTGCATCGGTGGATTTAACAATTTTTTCACT
ACACTTAGCAGGTGTCTCATCTATTTTAGGTGCTATTAACTTTATCACTA
CAATTTTTAATATAAAACCCCCTGCGATAACCCAATATCAAATACCTTTG
TTTGTATGATCCGTTTTAATTACTGCTTTCCTCCTCCTTTTATCTCTCCC
TGTCTTAGCCGCAGGAATTACAATACTCCTAACAGATCGAAACCTAAACA
CAACTTTCTTTGATCCAGCAGGCGGAGGAGATCCAATTCTATATCAACAC
TTA---

------------TTCCTGGAGAGAAACCTTCACCCATCTAACTGCCTTGG
CATGTTGTTGCTGTCTGACGCCCACCAGTGTACCAAGCTGTCAGAGCTCT
CCTGGGGCATGTGCTTGAGCAACTTCCCTGCTATTTGCAAGACGGAGGAC
TTCCTCCAACTGCCCAAAGACATGGTGGTGCAGCTTTTGTCGCACGAGGA
GCTGGAGACGGAAGACGAGAGGCTGGTGTACGAAACCGCACTCAACTGGA
TCAACTACGACCTGGAGAGGAGGCACTGCCAGCTCCCGGAGCTCCTGAGG
ACGGTTCGCCTGGCGTTGCTGCCYGCCATCTTTCTGATGGAGAACGTCTC
CACGGAGGAGCTGATCAACGCCCAGCCCAAGAGCAAGGAGCTGGTGGACG
AGGCCATCCGCTGCAAGCTGAAGATCCTGCAGAACGACGGCGTCGTTAAC
AGTCCGTGTGCTCGACCTCGAAAAACAAGCCACGCCCTCTTTCTTCTGGG
GGGGCAGACTTTCATGTGTGACAAGCTATACCTGGTGGACCAGAAGGCCA
AAGAGATCATYCCCAAAGCTGACATTCCCAGCCCCAGGAAGGAGTTCAGT
GCCTGTGCCATCGGTTGTAAGGTGTACATCACCGGAGGGA--GGGGCTC-
CGAGAACGGTGTGTCCAAAGACGTGTGGGTCTACGACACGGTTCACGAGG
AATGGTCCAAAGCAGCTCCCATGCTGATCACCAGACTTGGCCACGGCTCC
GCAGAGTTGAAACACTGCCTCTATGTAGTAGGAGGCCACACCGCAGCCAC
GGGCTGCCTGCCAGCGTCTCCGTCAAGATGAATACATTGTAGTGTTTAGT

CGATCAACAACAAGGCTTATTTTAAATGAAGCTGAGCTTATCTTGGCACT
GGCTCAGGAGTTCCAAATGAAAGTCGTCACAGTATCTATGGAGGAACAAT
CTTTTCCTAGCATTGTTGAGGTGATCAGTGGTGCTTCTATGTTAGTCAGC
ATGCACGGAGCTCAACTCATCGCTTCGCTTTTCCTTCCCAGAGGAGCTGC
TGTTGTCGAGCTGTTTCCATTTGCTGTGAATCCAGAGCAGTACACCCCAT
ATAAAACTCTTGCATCCCTTCCAGGCATGGATCTTCATTATATCTCTTGG
CAAAACAGTAAGGAGGACAACACTATTATTCACCCAGACAGGCCATGGGA
GCAAGGTGGCATTGCTCACTTAGAGAAGGAGGAACAAGAAAAAATCCTGG
CCAGTAAAAATGTTCCCAGGCACCTATGCTGCCGCAACCCAGAGTGGCTT
TTCAGGATCTACCAGGACACTTTAGTAGACATCCCTTCCTTTTTGGAAGT
TCTCAA---AGTGGGCATGAAA---ACTAAGCCTAGCTTAAAGAA---AG
CAAGACCTTCTACAACTGTCCACCCAGGCCGGGTCAGAGAACCTCAGTGT
CAGACATCAGTACTAACTCCTAATGAGGCTAAACTCACAGTCTCCTGGCA
GATCCCCTGGAATCTAAAATACCTAAAGGTAAGAGAAGTGAAATATGAGG
TTTGGATCCAGAAGAAAGACACCAGCAAGGGGACCTTGGAGGATCAGATC
ATACAGGCAAATCCTGCTCTCGAAGCCTTCGGCAATGCCAAAACACTGAG
GAATGACAACTCGTCCCGTTTTGGAAAATTCATCCGAATCCATTTCGGTA
CAAGTGGCAAGTTGTCGTCTGCTGACATTGAAACATACCTACTGGAGAAG
TCACGCGTGACTTTTCAGCTAAAGGCTGAAAGGAACTATCACATCTTCTA
CCAGATTCTGTCCAATCAGAAGCCGGAACTGCTGGATATGCTGCTGATCA
CCAACAACCCATATGACTACTCCTACATCTCCCAGGGAGAGGTAACAGTT
GCCTCCATCAACGACTCAGAGGAGCTGATGGCCACAGACAGTGCCTTCGA
TGTGCTGGGTTTCACCCCAGAGGAAAAGATGGGTGTCTATAAACTAATTG
GTGCCATCATGCACTATGGCAACATGAAGTTTAAGCAGAAGCAGCGCGAG
GAGCAGGCGGAACCAGATGGCACCGAGGCTGCTGATAAATCAGCATACCT
GATGGGGCTGAACTCTGCTGACCTCATCAAAGGCCTGTGCCATCCGAGAG
TCAAGGTAGGAAACGAATATGTGACCAAAGGCCAGAGTGTGGACCAGGTC
TACTAT--

----------------------TATCTCATCTATGCCTCTTTCTCATTCA
TGGGATGTTTACAAATCAGCGATGGATCAAATATTGTGAACCTGTTGGCT
AGTAACTCACCAAGTGTTTCATATGCTCTGACCCAGCAGAAGTATTTTAG
TAACTACAGTCCAGTGATCGGGTTTTACATTTATGAGCCCATTGAGTACT
GGAACTCCACGGTGCAGGAGCACCTAAAGACTCTAAGTCATGGCTTCAAC
AAAATTTCATGGATGGACAACTTTTTCCATTACCTTCGGGTAGTGAATGT
GAGTGCATCTACTAAGAGTGACTTCATCAACATCCTTAAGGGATCTTTCC
TAAACAGTCCAGAGTACCAGCACTTCACCGAGGACATCATTTTCTCCAAG
A---ACCGTGAGACTG------ATGAGTACGACATCATAGCCTCGCGGAT
GTACTTGGTGGCACGAACTACAGAGAAGAAGCGCGARGAAGTGGTGGAGC
TCCTAGAAAAACTTCGGCCCTTGATGCTAATCAACAGCATCAAATTCATT
GCCTTCAATCCCACATTTGTGTTTATGGACCGCTACAGCTCCTCCGTCAT
CTCGCCTATTCTGACCTCAGGCTTCAGTGTGCTCACTATCCTCATCCTCA

CTTTCTTCCTAGTCATTAACCCCTTGGGAAACTTCTGGCTCATCCTTACA
GTTACATCTGTGGAGCTAGGTGTTTTGGGGTTGATGGGCTTCCACCAGTT
TGAATGGCAGCCAGCTCTGAAGAATGTGTCTGCATCTTGCAATGTTGGCA
TCATTAATGGGCTCTCCGGATTTGCTTCCTCCGTGGACGACTCCCCGGCT
GACACCATCACTCGGCGGTTTCGCTATGATGTGGCACTGGTGTCGGCATT
AAAGGATCTGGAGGAGGACATCATGGAGGGGCTGAGAGACAGCGGCATGG
AAGACAGCGCTTGCACCTCGGGCTTTAACGTCATGATCAAGGAGTGCTGC
GATGGCATGGGTGATGTCAGCGAGAAGCACGGCGGCGGGCCGGTTGTTCC
CGAGAAGGCCGTGCGCTTCTCTTTCACCGTTATGTCTGTGTCGATCCTGG
CAGATGACGGTAAG------------------------------GAGGAG
GTTGCCATCTTCACTGAGCCAAAGCCGAACTCGGAGCTTTCCTGTAAGCC
CCTTTGTCTGACGTTTGTGGATGAGTCGGACCACGAGACGCTCACCGCCG
TGCTCGGGCCCATTATCGCAGAGCGAAAAGCCATGAAGGAGAGCAGGCTC
ATCCTGTCCATAGGGGGCCTGCTTCGCTCCTTCCGCTTCCACTTCAGGGG
CACGGGATACGACGAGAAGATGGTTCGCGAGATGGAGGGACTCGAGGCCT
CGGGGTCCAGTTATATCTGCACCTTGTGCGACTCGAGTCGAGCGGAGGCC
TCGCAAAACATGGTGCTGCACTCCATCACCCGCAGCCACGACGAGAATCT
GGAACGATATGAGATATGGAGGACCAACCCGTTCTCCGAGTCGGCGGAGG
AGCTGAGAGACAGAGTCAAAGGGGTCTCTGCCAAGCCCTTCATGGAGACC
CAGCCCACTCTGGACGCGTTGCACTGTGACATTGGCAACGCCACAGAGTT
CTACAAAATCTTCCAGGACGAGATCGGGGAGGTGTTTCAAAAGGC---CA
AC---CCCAGCCGGGAGGAGCGGCGCAGCTGGAGGGCAGCGCTGGATAAA
CAGCTGAGGAAGAAGATGAAGCTCAAGCCCGTAATGAGGATGAATGGGAA
CTACGCCCGCCGGCTAATGACCCTGGAGGCGGTGGAGGCGGTGTGCGAGC
TGGTGCCCTCAGAGGAAAGGAGGGAGGCCCTACGGGAGCTGATGAGGCTC
TACCTCCAAATGAAGCCCGTGTGGCGCTCCACCTGCCCGGCCAAGGAGTG
CCCCGACCAGCTGTGCCGCTACAGCTTTAACTCGCAGCACTTTGCTGACC
TGCTGTCCTCCACCTTCAAATACCGCTACAATGGCAAGATAACCAATTAC
CTGCACAAGACCCTGGCCCATGTGCCTGAAATCATAGAGAGAGATGGATC
CATAGGCGCCTGGGCCAGCGAGGGCAACGAGTCGGCCAACAAG-------

-----------------------------------GCCAAATCTCGCTTT
CACCCTGGCGTGGGGACTGGTCCTGGCACGGAGC---GCAGCGTCCCACT
CGGCAACAGCTTGCTATCCCCGCAGCAAACCGAGGAGCCCACTGTTGCCA
CCCCCCCGCAGCGATGGTTTGTCACCC---CTGCCAACAACCGACTGGAC
TTTGCTGCCTCGGCATACGACGCCGCT---------GATTTCGCCGGTAA
CGCGGCCACCTTGCTGTCCTACGCAGCGGCCGGAGTGAAAGCTC------
TTCCCCTGCCGGCGGCAGGCTGCTCCAACCGGCCTCTTGGCTATTACGCA
GACCCATCGG---GCTGG---GGAGGACGCACGCCGCCGCAGTACTGCGG
CGTCAATAGCAAATCCAGCTCGGTCTTTTCATGCTGGCCCACCAATTCTC
TCGGCGGCAGGGCGAGCA---CC---AACTACCTGG---------CCGAG
GA---GGGA---GACTC---CATCCCCACCGAGAGGTCACCG---AT---
TGGCGGCTCGGAGGAA---ACCAAACCTAAGGACATGAC---GTCGGA--
-GTCGAGCTGGATAGAG---ACGCCGTCCTCCATAAAGTCCATCGATTCG
AGTGATTCTGGTATCTTTG---AACAGGCCAAAAGGAGAAGGATCTCACC
TTCTGCCACGCCG---------GTTTCAGAGACAGTGTCTCCGTTAAAAT
CTGAGNNNNNNNCAACAGGCGAAGTCACAGAGAGAGAAGTAGCGTTGGGGATAAATC
CGTTCGCCGATGGCATGGGCGCCTTCAAAATCAACCACAGCTCCCACGAT
CTGGGCTCCGG---ACAAACGGCGTTTTCCTCCCAGGCA---CCGGGCTA
C---GCGGCGGCCGCCCTGGGA---CACCACCA------CCACCCGACCC
ACGTTGGCTCC---TACTCCACGGCGGCTTTCAACTCCACCAGGGACTTT
CTGTTCAGAAACCGGGGCTTCGGGGATGCCACCGG------------GGC
GCAGCACAGCTTGTTCGCCTC---------CGGGAGTTT---C------G
CGGGGCCACATGGACACTCAGATGCGGCGGGGCACCTGCTCTTCCCGGGG
CTCCACGAG---CAAGCGGCGAGCCACGCGTCTTCCAACGTGGTCAACAG
CCAGATGCGGCTGGGCTTCTCGGGGGACATGTACGGACGGGCCGACCAGT
ACGGCCACGTTACCAGCCCGCGGT---CCGACCACTACGCTTCGACCCAG
CTGCACGGCTACGGCCCTATGAACATGAATATGGCCGCA---CACCACGG
AGCAGGGGCCTTCTTTCGATACATGAGGCAGCCGATCAAGCAAGAGCTCA
TCTGCAAGTGGATCGAGCCGGAGCAGCTGACGAATCCCAAAAAGTCGTGC
AACAAAACTTTTAGCACGATGCACGAGCTTGTGACCCATCTGACGGTGGA
GCATGTGGGGGGACCGGAGCAGACGAACCACATCTGCTTCTGGGAGGACT
GCTCCAGAGAAGGAAAGCCATTCAAAGCCAAATACAAACTTGTGAATCAT
ATCAGAGTACACACCGGAGAAAAGCCCTTTCCGTGTCCGTTCCCCGGCTG
TGGCAAA
>Bothus lunatus
AGCCTTCTTATCCGGGCCGAGCTCAGCCAGCCCGGAGCCCTGCTGGGCGA
CGACCAGATTTATAATGTCATCGTCACAGCCCATGCGTTCGTGATGATCT
TCTTTATAGTGATGCCTATTATGATTGGGGGCTTCGGCAACTGACTGATC
CCCCTGATGGTGGGAGCCCCCGATATGGCATTCCCCCGCATAAATAACAT
GAGCTTCTGGCTACTTCCACCCTCCTTCCTACTCCTGCTAGCCTCCTCCG
GGGTAGAAGCAGGGGCAGGGACCGGGTGGACCGTCTACCCCCCATTGGCG
GGCAATCTTGCACACGCCGGGGCATCAGTGGACCTAACCATCTTCTCTCT
TCACCTTGCAGGTATCTCGTCCATCTTGGGGGCCATCAACTTCATTACCA

CGATTTTGAACATGAAACCCCCAGCCATGACGATGTACCAAGTTCCACTA
TTCGTCTGGGCAGTCCTAATCACTGCAGTCCTCCTGCTGCTCTCCTTGCC
AGTGCTCGCAGCAGGGATCACCATGCTTCTGACAGACCGAAACCTGAACA
CCACCTTCTTCGACCCAGCCGGAGGGGGGACCCAATCCTTTACCAACAT
CTATTC--

-------------------------AGATGATTACATTGTTGTGTTCAGT
CGATCAACAACGAGGCTGATCCTCAACGAGGCAGAGCTCATAATGGCTCT
GGCCCAAGAGTTCCAGATGCGAGTGGTCACAGTATCCCTGGAGGAACAGT
CCTTCCCAAGTATCGTCCAGGTGATCAGCGGCGCTTCAATGCTGGTCAGC
ATGCATGGAGCTCAGCTCATTACCTCAATGTTCCTCCCCAGAGGAGCATC
TGTGGTAGAACTCTTCCCCTTTGCTGTGAACCCCGAGCAGTACACGCCAT
ACAAAACCCTCGCCTCCCTCCCAGGCATAGACCTTCATTATGTTTCTTGG
AGGAACAGCAAGGAGGAGAACACTGTCACCCATCCAGACAGGCCTTGGGA
GCAAGGGGGCATTGCTCACCTGGAGAAGGACGAGCAGGAGCGGATATTGG
CCAGCAAGGAGGTCCCCAGGCATCTGTGTTGTCGCAATCCCGAGTGGCTC
TATCGGATCTACCAGGACACCCTGGTGGACATCCCTTCCTTTCTGGAGGC
CCTCAA---AGAGGGCAGGAAG---TCCAAGCCTAGCCTGAAGAA---GT
CTAAGCCGGCCAGCACAGTCCACCCGGGCCGTGTCAGAGAACCTCAGTGT
CAGACCACGGTGCAAACCAGCAATGAAGCCAAACTCACAGTCTCTNNNNNNNNNNNNNNNNNNNNNNNNN
NNNNNNNNNNNNNNNNNNNNNNNNNNNNNNNNNNNNNNNNNAAAAA
GGACTCCAGCAAGGGGACCCTGGAGGATCAGATCATCCAGGCAAACCCTG
CACTGGAGGCCTTTGGTAATGCCAAAACGGCGAGAAATGACAACTCGTCA
CGTTTTGGAAAATTTATTCGGATTCACTTTGGTACAAGTGGCAAACTTTC
CTCTGCAGACATTGAGACCTACCTGCTGGAAAAGTCCCGCGTCACTTTTC
AGCTCAAGGCGGAGAGAAACTACCACATTTTCTACCAAATTCTTTCCAAT
CAGAAGCCAGAGCTGCTGGATATGCTCCTCATCACCAACAACCCATATGA
CTACAGTTACATCTCCCAAGGAGAAGTGACGGTTGCGTCTATCAACGACT
CAGAGGAGCTGATGGCCACTGACAGCGCCTTTGATGTTCTCGGCTTCACT
CCTGAGGAGAAACTTGGCGTCTATAAACTAACCGGTGCCATCATGCACTA
TGGCAACATGAGATTCAAACAGAAGCAGCGTGAGGAGCAGGCTGAACCTG
ATGGGACAGAGGCTGCTGATAAATCGGCGTACCTGATGGGGCTGAACTCT
GCTGACCTCATCAAANNNNNNNNNNNNNNNNNNNNNNNNNNNNNNNNNNNNNNNNNNNNNNNNNNNNNNN
NNNNNNNNNNNNNNNNNNNN-----------------------------------

-------------------------------NNNNNNNNNNNGCCCTCTTTCTCATTCATG
GGATGTTTACAAATAAGTGATGGGTCGAATATTGTCAACTTGCTGGCTAG
TAACTCTCCAAGTGTCTCATATGCTGTGACTCAGCAGAAATACTTCAGTA
ACTATAGTCCTGTGATTGGGTTTTATATTTATGAGCCCATTGAATACTGG
AACTCCACGGTGCAGGAACACCTTAAGACACTGAGTCATGGCTTTAACAA
GATATCCTGGATGGACAACTTTTTCCACCACCTGCGGGTGGTAAATGTGA
GCGCATCAACCAAGAACGACTTCATCAGCATCCTCAAGGACTCTTTCCTG
CGGAGCCCGGAATATCAGCACTTCAATGAGGACATCATTTTTTCAAGGA-
--ACCGTGAGACAA------ATGAGTATGACATCATCGCCTCACGGATGT
ACCTTGTGGCGCGGACGACCGAGAAGAAGCGCGAGGAGGTAGTGGAACTT
CTGGAAAAGCTCCGCCCATTGATGCTAATCAACAGCATTAAGTTCATTGC
CTTCAATCCCACTTTTGTGTTCATGGACCGCTACAGCTCCTCAGTCATCT
CCCCCATCCTGACCTCAGGCTTCAGTGTACTCACCATCCTCATCCTCACT
TTCTTCCTGGTCATCAACCCGTTAGGGAACTTCTGGCTCATCCTTACAGT
CACATCTGTGGAACTGGGCGTCTTGGGTTTGATGGGTTTTCACCAGTTTG
AGTGGCAGCCAGCTCTCAAGAATGTGTCAACATCTTGCGACGTGGGCATC
ATTAACGGCCTCTCCGGATGGACCTCCTCAGTGGATGACTCCCCCGCCGA
CACCATCACCAGGCGCTTTCGTTATGATGTGGCGCTGGTGTCGGCAATGA
AAGACCTGGAGGAGGACATCATGGAGGGGTTGAGGGAGAGTGGGATGGAG
GACAGCGCCTGCACCTCAGGTTTCAAAGTCATGATCAAGGAATGCTGTGA
CGGCATGGGTGACGTCAGCGAGAAGCACGGGGGAGGGCCGGCACTTCCGG
AAAAGGCCGTACGTTTCTCCTTCACCGTCATGTCCGTCTCTGTCCTAGCA
GATGATGGAGAG------------------------------GAGGAGGT
CACCATCTTCACCGAGCCAAAGCCCAACTCAGAACTGTCGTGTAAGCCCC
TCTGCCTCATGTTTGTGGACGAATCAGACCATGAGACCCTCACAGCCGTC
CTGGGCCCCATAGTTGCTGAGCGCAATGCAATGAAGGAGAGCCGCCTCAT
CCTGTCCATAGGTGGCCTGCCTCGCTTGTTCCGCTTTCACTTCAGAGGCA
CGGGATATGACGAGAAGATGGTGCGCGAGCTGGAGGGTCTGGAGGCTTCG
GGCTCCACTTACGTCTGCACGCTGTGTGATTCCAGTCGTGCCGAAGCCTC
AGAGAACATGGTGCTGCACTCCATCACCCGCTCACATGAGGAAAACCTGG
AGCGCTATGAGATATGGCGCACCAATCCTTTCTCCGAGTCGGCGGAGGAG
CTGCGAGACAGGGTCAAAGGCGTCTCAGCCAAGCCCTTCATGGAGACCCA
GCCCACCATGGACGCTTTGCATTGTGACATTGGCAATGCCACTGAGTTCT
ACAAGATATTCCAGGACGAGATCGGCGAGATGCACAAGAGGGT---CAAC
---CCCAGCCGGGAGGAAAGGCGCAGCTGGCGGGCGGCGCTGGATAAGCA
GCTGAGGAGGAAGATGAAACTCAAACCAGTGATGAGGATGAACGGGAACT
ACGCCCGCAAGCTCATGACCCAGGAAACGGTGGAGGCGGTGTGTGAGCTG
GTGCCCTCGGAGGAGCGGAGGGAGGCCCTTAGGGAACTGGTCAGGCTCTA
CCTTCAGATGAAGCCTGTGTGGCGCGCCACCTGCCCTGCCAAGGAGTGCC
CCGACCAGCTGTGCCGCTACAGCTTCAACTCACAGCGCTTTGCCGAGCTC
CTCTCGTCCACCTTCAAGTACAGGTACAATGGGAAGATCACCAACTACCT
CCACAAGACCCTGGCTCATGTACCTGAGATCATCGAGAGGGATGGGTCAA

TAGGAGCCTGGGCCAGCGAAG--------------------
NNNNNNNNNNNNNNNNNNNNNCCTGTCGGG
CCCCGGTGGAAGGAGAGCCCACAACCTTTCTCCTGCTCAATTGAAGACCC
AACAAAACAGACGAAGTTTAAGGGCATCAAGACCTACATTTCCTATCGGG
TTACGCCAAGCCACACCGGGCATCCTGTGTATAGACGCTACAAACACTTT
GACTGGCTGTACAACCGCTTATTGCACAAGTTCACTGTGATCTCTGTGCC
TCATCTGCCGGAGAAGCAGGCCACAGGGCGCTTTGAGGAAGACTTCATTG
AGAAGCGCAAGAGACGACTGATACTGTGGATGAATCACATGACCAGTCAC
CCGGTCCTCTCGCAGTACGAAGGCTTTGAGCACTTTCTTATGTGTGTTGA
CGACAAACAGTGGAAACTCGGAAAACGACGGGCGGAAAAGGACGAGATGG
TCGGCGCCCATTTCATGCTGACCCTTCAAATCCCCAACGAGCACCAGGAC
CTGCAGGACGTGGAGGAACGGGTGGACACCTTCAAGTCGTTTGCCAAAAA
GATGGACGACAGCGTCATGCAGCTCACGCACGTGGCCTCGGAGTTGGTGC
GCAAACACCTGGGTGGCTTCAGGAAGGAGTTCCAGCGACTGGGGAACGCC
TTCCAGTCCATCAGCCAGGCCTTCATGCTGGATCCTCCTCACAGCTCGGA
CTCCCTGAATAACGCCATCTCCCAC-------------------------

>Brosmophycis marginata
AGCCTTCTTATTCGGGCAGAATTGAGCCAACCCGGCGCCCTCCTCGGAGA
TGACCAAATTTACAATGTTATTGTTACAGCCCATGCCTTTGTAATAATTT
TCTTTATAGTAATACCAATCATGATCGGAGGCTTTGGAAACTGACTTATT
CCACTAATAATTGGAGCCCCTGACATAGCATTTCCCCGAATAAATAACAT
AAGTTTTTGACTACTCCCCCCCTCTTTTCTTCTCCTATTAGCCTCTTCAG
GAGTCGAAGCCGGAGCAGGCACAGGATGAACAGTCTACCCGCCCCTAGCG
GGAAACCTGGCCCACGCGGGCGCCTCCGTCGACCTGACCATCTTCTCCCT
TCACTTGGCAGGAGTCTCCTCAATCCTAGGGGCTATTAACTTTATTACAA
CTATCATTAATATGAAGCCCCCTGCCATCTCCCAATACCAAACACCTCTC
TTCGTGTGAGCCGTCCTAATCACTGCCGTCCTGCTCCTCCTCTCTCTCCC
CGTCCTCGCTGCCGGCATCACCATACTTTTAACGGACCGGAACCTGAATA
CAACATTCTTCGACCCCGCTGGCGGAGGGGACCCTATTCTTTACCAACAT
CTGTTTTGATTCTTCGG---------------------------------

------------TTTCTCGAGAGAAACCTTCACCCCTCTAACTGCCTCGG
CATGCTGTTGCTGTCTGATGCCCATCAGTGCACCAAGCTATCCGAGCTGT
CCTGGGGCATGTGCCTCAGCAACTTTCCTGCTATTTGCAAGACCGAGGAC
TTCCTCCAACTGCCCAAAGACATGGTGGTGCAGCTTTTGTCACATGAGGA
GCTCGAGACAGAAGATGAGAGACTGGTTTATGAAGCTGCCCTTAACTGGA
TCAACTATGACCTGGAAAGGAGGCACTCCCACCTTCCAGAGCTTCTGAGA
ACGGTCCGCCTGGCCCTGCTTCCTGCCATCTTTCTCATGGAGAATGTCTC
GACAGAAGAGCTGATCAATGCCCAGGCCAAGAGCAAGGAGCTAGTGGATG
AGGCCATCCGCTGTAAGCTGAAGATCCTCCAGAATGATGGCGTAGTTAAC
AGCCCTTGTGCCAGACCAAGAAAAACCAGTCATGCACTCTTTCTGCTGGG
AGGGCAGACTTTCATGTGTGACAAACTGTACCTGGTGGATCAGAAAGCCA
AAGAGATCATTCCCAAAGCTGACATCCCCAGCCCCAGAAAGGAGTTCAGT
GCCTGCGCCATTGGCTGTAAGGTGTACATCACAGGTGGGA--GAGGCTC-
AGAAAACGGTGTGTCCAAAGATGTATGGGTCTACGACACCGTCCATGAGG
AATGGTCCAAGGCAGCACCCATGCTTATTGCCAGNNNNNNNNNNNNNNNNNNNNNNNNNNNNNNNNNNNN
NNNNNNNNNNNNNNNNNNNNNNNNNNNNNNNNNNNNNNNNNNNNNNNNNNNNNNNGGATGAATACATTGT
T
CTGTTTAGCCGTTCAACAACAAGGCTGATACTGAATGAAGCTGAGCTAAT
CATGGCACTGGCCCAGGAATTCCAGATGAGAGTGGTTACAGTCTCCCTGG
AGGAACAATCTTTCCCAAGTATCGTCCAGGTGATCAACGGTGCTTCCATG
TTGGTTAGTATGCATGGAGCTCAGCTTATCGCCTCACTCTTCCTGCCCAG

AGGAGCTGCTGTGGTGGAGCTGTTCCCCTTCGCTGTAAACCCAGAGCAAT
ACACCCCATATAAAACCCTGGCCTCCCTTCCAGGCATAGACCTCCACTAT
GTCTCCTGGAGGAACACCATGGAGGAGAACACCATCACCCACCCAGACAG
ACCCTGGGAACAAGGAGGAATCGCTCACTTGGAGAAGGAGGAGCAACAGC
GAATACTTTCCAGCAGAGATGTCCCCAGGCATCTGTGCTGCCGCAACCCA
GAATGGCTCTTCCGAATCTACCAGGACACTTTGGTGGACATCCCGTCATT
CATGGAAGTCCTCAA---AGAGGGCCTGAAG---ACCAGGCCTAGCTTAA
AGAA---GGCCAAGCCGGCCAGCACCGTCCACCCAGGCCGGGTCAGAGAA
GCCCAGTGTCAGACCTCAGTGCAAACCAGCAGTGAGGCTAAACTCACGGT
TTCCTGGCAGATACCGTGGAATCTGAAGTACCTGAAGGTGAGAGAAGTGA
AGTACGAGGTG---------AAGAAAGACACCAGCAAGGGGACACTGGAA
GATCAAATCATCCAGGCAAACCCAGCACTGGAGGCCTTCGGTAATGCCAA
AACATTGAGAAACGACAACTCCTCTCGTTTTGGAAAATTCATTCGAATTC
ACTTTGGAACCAGTGGTAAGCTGTCATCCGCTGACGTTGAGACATATCTG
CTGGAGAAGTCACGTGTTACCTTTCAGCTCAAGGCTGAGAGGGACTATCA
CATCTTCTACCAGATCCTGTCCAAACAGAAGCCAGAGCTTTTGGATATGC
TGCTGATCACAAACAACCCGTATGACTACTCCTACATCTCCCAAGGAGAA
GTGACAGTTGCATCCATCAATGACGCAGAGGAGCTGATGGCCACCGATAG
TGCCTTCGACGTGCTCGGCTTCACTTCAGAGGAGAAGATGACTGTCTATA
AGCTGACTGGCGCCATCATGCACTATGGCAACATGAAGTTTAAGCAGAAG
CAGCGTGAGGAGCAGGCTGAACCTGATGGGACAGAGGCTGCTGATAAATC
AGCTTACCTGATGGGTCTGAATTCTGCAGACCTCATCAAAGGGTTGTGCC
ATCCCAGAGTCAAGGTAGGGAATGAATATGTCACAAAAGGCCAAAGTGTG
GACCAAGTCTACTATCCCAACAAGGAGGCCTTCAAGTGTGAGGAGTGTGG
CAAGCACTACAACACCAAGCTGGGATATAAGCGACATGTTGCCATGCACT
CTGCCACAGCAGGGGATCTCACCTGTAAAGTGTGCATGCAGATCTACGAG
AGCACACCGGTGCTCCTGGAGCACCTCAAGAGCCACTCGGGGAAGTCTTC
GGGTGGCACCAAGGAGAAAAAACACCCGTGTGACCACTGTGATCGTCGTT
TCTACACACGGAAGGATGTGAGACGGCACATGGTGGTCCACACGGGCCGG
AAGGACTTTCTGTGCCAGTACTGTGCCCAGCGCTTTGGCAGGAAGGACCA
CCTGACACGTCACGTGAAGAAGAGCCACTCTCAGGAGCTGCTGAAGATCA
AGACGGAGCCCCCGGATATGTTA---CTTTTAGGATCGGGGTCACCACCT
TGCCCTGTGAAGGAGGAGCTTAGCCCCATGATGTGCGGCATGGGTCCAAA
CAAAGATCCCATGATGGGAAAACCTTTCCCAAGTGGGGCCCCTTTTCCGA
TGGGCATGTACAACCCCCACCAT------CTCCAGGCCATGTCCAATTCT
GGGGTGGGCCACCCC------CACCCCTCCTTGATGCCTAGTACCCTGTC
TGCAGCTATGGGTATGGGCTGTCACATGGAATATCTCATCTATGCCTCTT
TCTCATTCATGGGATGTTTACAAATCAGTGATGGATCAAACATCGTGAAC
CTGCTGGCTAGTAACTCTCCCAGTGTTTCATACGCTCTGACTCAGCAGAA
ATACTTCAGCAACTACAGTCCCGTTATTGGTTTTTACATTTATGAGCCCA
TTGAGTACTGGAACTCCACGGTGCAGGAGCATCTGAAGACTCTGAGTCAC
GGCTTCAACAAGATCTCCTGGATGGACAACTTTTTCCACTACCTGCGGGT
GGTGAATGTGAGCGCATCGACCAAGAGTGATTTTATCACCATCCTCAAGG
GCTCTTTCTTACGCAGCCCGGAGTACCAGCACTTCACTGAGGACATCATC
TTCTCTAAGA---ACCGTGAGACTG------ATGAGTATGACATAATTGC
CTCACGCATGTACTTGGTGGCGAGGACCACAGAGAAGAAACGTGAGGAGG
TGGTGGAGCTGCTGGAAAAGCTACGTCCATTGATGCTGATTAACAGCATC
AAGTTCATTGCCTTCAATCCCACTTTTGTGTTCATGGACCGCTACAGTTC
GTCTGTCATTTCACCCATCCTGACCTCAGGCTTTAGTGTGCTCACTATCC
TCATTCTCACTTTCTTCCTGGTCATCAATCCCTTAGGGAACTTCTGGCTC
ATTCTGACAGTTACATTCTGGGAGCTGGGCGTCTTGGGTTTGANNGGTTTTC
ACTTATTTGAATGGCAGCCAGCTCTCAAGAATGTGTCTGTATCCTGCAAT
GTTGGTATTATTAATGGGCTCCCTGGATGGGCTGCCACTGTGGATGACTC

CCCAGCTGACACCTTCACACGACGGTTTCGCTATGATGTGGCGCTGGTGT
CAGCATTAAAGGATCTGGAGGAGGACATCATGGAAGGATTGAGAGAGTGT
GGGATGGAAGACAGCACTTGCACCTCAGGCTTCAGTGTTATGATCAAGGA
ATCTTGTGATGGCATGGGCGATGTCAGCGAGAAGCATGGCGGAGGACCAC
CGGTTCCAGAGAAGGCTGTACGTTTCTCTATCACTGTTATGTCTGTCTCT
GTCCTGGCAGATGGCGAGGAG-----------------------------
-GAGGCGGTTACCATCTTTACGGAGCCAAAGCCAAACTCAGAGCTGTCCT
GTAAGCCCCTTTGCCTCACATTTGTGGATGAGTCAGACCACGAGACACTC
ACAGCCGCCCTGGGCCCCATAGTTGCAGAGCGTAATGCAATGAAAGAGAG
TAGACTCATTCTTTCCATAGGTGGCCTGCCTCGCTCCTTCCGCTTCCACT
TCAGAGGCACAGGTTATGATGAGAAGATGGTGCGTGAGCTGGAGGGCCTG
GAGGCCTCAGGTTCCACCTATATCTGCACTCTTTGTGACTCCAGTCGGGC
AGAGGCTTCACACAACATGGTGCTGCACTCCATCACCCGTAGTCACGATG
AGAACCTAGAGCGTTACGAAACATGGAGAACCAATCCCTTTTCTGAGTCT
GTAGAGGAGCTGCGGGACCGAGTCAAAGGAGTCTCTGCCAAGCCCTTTAT
GGAGACCCAGCCAACACTGGATGCTTTGCACTGTGACATTGGTAATGCCA
CTGAGTTCTACAAAATCTTCCAGGATGAGATTGGGGAGGTGTTCCAAAAG
GT---CAAC---CCCACACGGGAGGAACGGCGCAGCTGGAGGGCAGCCCT
AGATAAACAGCTGAGGAAGAAAATGAAGCTTAAACCGGTAATGAGGATGA
ATGGGAACTATGCCCGGAAGCTAATGACCCTGGAGACTATGGAGGTGGTG
TGTGAGCTGGTGCCATCAGAAGAAAGGAGGGAGGCCCTGAGGGAGCTCAT
GAGGCTCTACCTTCAGATGAAGCCTGTTTGGCGTGCCACCTGCCCAGCCA
AGGAATGCCCTGACCAGCTGTGTCGCTACAGCTTCAACTCCCAGCGCTTT
GCAGATCTCCTCGCCTCCACTTTCAAATATCGCTACAATGGAAAAATAAC
CAATTACTTGCACAAGACCCTGGCTCATGTCCCTGAGATCATTGAGAGAG
ATGGATCCATAGGAGCTTGGGCCAGTGAGGGGAATGAGTCAGCAAACAAA
TCATACACTATCGAGATGGGTCCTATGGGACCCGGGTGGAAGGAGAGCCC
ACAGCCTTTCTGCTGCTCCATTGAAGACCCCACAAAACAGACAAAGTTCA
AGGGTATCAAGACCTACATTTCCTATCGGGTCACACCAAGCCACACAGGG
CGTCCCGTCTATAGGCGCTACAAACACTTTGACTGGCTGTACAACCGCTT
ACTGCACAAGTTCACTGTGATCTCAGTACCCCACCTGCCTGAGAAGCAGG
CTACAGGTAGATTTGAGGAAGATTTCATTGAGAAGCGTAAAAGACGACTG
ATTCTGTGGATGAACCACATGACCAGTCACCCAGTCCTCTCCCAGTATGA
AGGCTTTGAGCATTTTCTGATGTGTGCTGATGACAAACAATGGAAACTGG
GTAAGAGACGAGCAGAGAAGGATGAAATGGTGGGTGCCCATTTCATGCTG
ACCCTCCAGATTCCTAATGAGCACCAGGACCTTCAGGATGTAGAGGAGCG
AGTTGACACCTTCAAGGCCTTTGCTAAGAAAATGGATGATAGTGTCTTGC
AGCTCACACATGTTGCCTCAGAGTTGGTGCGTAAACACCTGGGTGGATTC
AGGAAGGAGTTCCAGCGGCTTGGAAATTCCTTCCAGTCTATCAGCCAGGC
CTTCATGCTGGACCCTCCCCACAGCTCAGAAACTCTCAACANNNNNNNNNNNNNNNNNNNNNNNNNCGTTT
CTCA
AACTGACCTCTCTGGGTTTCATCATCGGAATCGGCGTGGTCGGAAACCTC
CTGATCTCCATCCTGCTGGTTAAAGACAAGAGCCTGCACCGCGCGCCCTA
CTATTTCCTGCTGGACCTGTGCGCCTCGGACATCCTGCGTTCCGCTATCT
GCTTCCCCTTTGTCTTCACCTCCGTCAAGAATGGATCCATCTGGACCTAC
GGCACGCTCACCTGCAAAGTGATCGCCTTCCTGGGCGTGCTCTCCTGTTT
CCACACGGCGTTCATGCTGTTTTGCGTCAGCGTGACCCGCTACCTGGCCA
TCGCGCACCACCGCTTCTACACGAAGAGGCTGACCTTCTGGACCTGCCTG
GCGGTCATCTGCATGGTGTGGACGCTGTCGGTGGCCATGGCTTTCCCACC
GGTGCTAGACGTGGGGACGTACTCTTTCATCCAGGAGGAGGACCAGTGCA
CATTCCAGCACCATTCCTTCAGGGCGAACGACTCCCTGGGCTTCATGCTC
CTGCTGGCGCTCATCCTCCTGGCCACACAGCTGGTTTACCTCAAGCTCAT
CTTCTTCGTCCACGACCGTCGGAAGATGAAGCCCGTCCAGTTCGTGCCTG

CCGTCAGCCAGAACTGGACCTTCCACGGGCCTGGCGCCAGCGGGCAGGCG
GCGGCCAACTGGCTGGCTGGATTTGGTAGAGGTCCCACGCCTCCTACTTT
GCTGGGCATCCGGCAGAACAGCAACGCAGCGGGCCGCAGGCGTCTGCTGG
TGCTGGACGAGTTCAAAACAGAGAAGAGGATTAGCAGGATGTTCTACATC
ATGACGTTTTTCTTCCTGGCACTGTGGGGGCCATATCTGGTAGCCTGCTA
CTGGCGGGTGTTTGCAAGGGGCCCTGTAGTCCCCGTGGGCTACCTGACAG
CAGCCGTGTGGATGAGCTTTGCCCAGGCAGGGGTCAACCCTTTCATCTGC
ATCTTCTCCAACAGGGAG--------------------------------

--------------------------------------CATCACTCAACA
GGCGAAGTCACAGAGAGAGAAGTGGCGTTGGGGATAAATCCTTTCGCGGA
CGGCATGGGCGCCTTCAAAATAAACCACAGCTCCCATGATATTGGTTCCG
G---ACAAACGGCGTTTTCCTCTCAGGCG---CCCGGCTAC---GCAGCG
GCCGCCCTGGGA---CACCATCA------CCACCCGACCCACGTTGGCTC
T---TACTCCACGGCGGCTTTCAACTCCACCAGGGACTTTCTCTTCAGAA
ATCGGGGTTTTGGAGACGCCACCGG------------GGCTCAGCACAGT
TTGTTCGCCTC---------CGGAAGTTT---C------GCAGGGCCACA
TGGACACTCAGATGCAGCGGGGCACCTGCTCTTCCCAGGGCTCCACGAG-
--CAAGCAGCGAGCCATGCGTCTTCCAACGTGGTCAACAGCCAGATGCGA
CTGGGCTTCTCGGGGGACATGTACGGTCGGGCCGATCAGTACGGCCACGT
TACAAGCCCGAGGT---CCGACCACTATGCTTCGACCCAGTTGCACGGTT
ATGGCCCCATGAACATGAATATGGCCGCA---CACCACGGAGCAGGGGCC
TTCTTTCGATACATGAGGCAGCCGATCAAACAAGAGCTCATCTGCAAGTG
GATCGAGCCGGAGCAGCTGACAAATCCCAAAAAGGCGTGCAACAAAACTT
TTAGCACGATGCATGAGCTTGTGACCCACTTGACAGTGGAGCATGTGGGG
GGACCAGAGCAGACCAACCACATCTGCTTCTGGGAGGAGTGCGCCAGAGA
AGGAAAACCATTCAAAGCCAAATACAAACTTGTAAATCATATCAGAGTAC
ACACCGGAGAAAAACCCTTTCCGTGTCCGTTCCCAGGCTGTGGCAAA
>Brotula multibarbata
AGCCTACTAATCCGAGCAGAACTCAGTCAACCAGGAGCCCTTCTTGGAGA
TGACCAAATTTATAATGTCATTGTTACAGCCCATGCTTTCGTAATGATTT
TCTTTATAGTAATACCAATTATGATTGGGGGCTTTGGAAACTGGTTAATT
CCACTTATGATTGGAGCCCCAGACATGGCATTCCCCCGAATGAACAACAT
AAGCTTCTGGCTTCTTCCTCCCTCATTCCTTCTTCTGCTGGCCTCATCAG
GAGTTGAAGCCGGGGCTGGTACTGGGTGAACTGTATATCCGCCCCTTGCT
GGAAACTTAGCCCATGCAGGAGCATCTGTAGATCTTACTATCTTCTCCCT
TCATCTGGCAGGGGTATCATCTATTCTAGGGGCCATTAACTTCATTACAA
CAATTATTAACATGAAACCCCCCGCAATTTCGCAATACCAAACACCTTTA
TTCGTATGAGCCGTTCTAATTACTGCAGTACTTCTTCTTCTTTCTTTACC
AGTCCTTGCTGCAGGAATTACAATGCTTCTAACAGATCGAAACCTTAACA
CCACTTTCTTTGACCCCGCTGGAGGAGGTGATCCTATCTTATACCAGCAC

CTA--

NNNNNNNNNNNNNNNNNNNNNNNNNNNNNNNNNNNGGCATGCTATTGCTGTCTGACGCCCATCAGTGTA
CCAA
GCTGTCAGAGCTCTCCTGGGGCATGTGCCTCAGCAACTTTCCCGCTATCT
GCAAGACGGAGGACTTCCTCCAATTGCCCAAAGACATGGTGGTGCAGCTG
CTGTCACACGAAGAGCTGGAGACAGAAGACGAGAGACTGGTCTACGAAGC
GGCCCTGAACTGGATCAACTATGACCTGGAGAGGAGGCACTGCCATCTTC
CAGAGCTCCTGAGAACAGTCCGCCTGGCCTTGCTGCCTGCCATCTTCCTC
ATGGAGAACGTCTCGACGGAAGAGCTGATCAACGCCCAGGCCAAGAGCAA
GGAGCTGGTGGACGAGGCCATCCGCTGCAAGCTGAAGATCCTGCAGAACG
ACGGCGTGGTCAACAGCCCGTGTGCCCGGCCGAGAAAAACCAGCCACGCC
CTCTTTCTGCTGGGAGGGCAGACTTTCATGTGCGACAAGCTGTACCTGGT
GGACCAGAAAGCCAAAGAGATCATCCCCAAAGCCGACATCCCCAGCCCCA
GGAAGGAGTTCAGCGCCTGTGCCATCGGCTGCAAGGTGTACATCACAGGT
GGGA--GGGGTTC-GGAGAACGGGGTGTCCAAAGACGTGTGGGTCTACGA
TACCGTCCACGAGGAATGGTCCAAGGCGGCGCCCATGCTCATTGCCAGGT
TCGGCCACGGTTCCGCCGAGCTGAAACACTGCCTGTACGTGGTGGGAGGT
CACACGGCAGCCACCGGTTGCCTCCCGGCCTCCCCCTCGGGATGAATACA
TAGTTGTGTTCAGTCGCTCAACAACAAGGCTGATCCTGAATGAAGCAGAG
CTGATCATGGCACTGGCCCAAGAGTTTCAGATGAGAGTGGTTACTGTATC
CCTGGAGGAACAGTCTTTTCCCAGTATTGTCCAGGTGATCAGCGGTGCTT
CCATGTTGGTTAGTATGCATGGGGCACAGCTCATTACGTCGCTCTTCCTC
CCCAGAGGAGCTGCTGTGGTGGAGCTTTTCCCCTTTGCTGTGAACCCGGA
GCAGTACACCCCATACAAAACCCTGGCCTCCCTTCCAGGCATGGACCTGC
ACTATGTCTCCTGGAGGAATGCCATGGAGGAGAACACGGTCACCCACCCA
GACAGACCCTGGGAGCAGGGGGGCATTGTTCACCTGGAGAAGGAGGAGCA
GGAGCGAATACTTACCAGCAAAGATATCCCAAGGCACCTGTGCTGCCGCA
ACCCGGAGTGGCTCTTCCGAATCTACCAGGACACTTTGGTGGACATCCCT
TCGTTCCTGGACGTCGTCAG---AGAGGGCTTGAAG---ACCAAGCCTAA
CTTGAGGAA---GAACAAGCCGGCCAGCACAGTTCACCCCGGTCGGGTCA
GAGAAGCACACTGTCAGACCTCAGTGCAAACCAGCAGCGAGGCTAAACTC
ACAGTTTCCTGGCAGATCCCGTGGAATCTGAAGTACCTGAAGGTGAGAGA
GGTGAAGTATGAGGTGTGGATCNNNAAGAAAGACACCAGCAAGGGGACGCTGG
AAGATCAAATCATCCAGGCCAACCCGGCATTGGAGGCCTTCGGTAACGCT
AAAACCTTAAGAAATGACAACTCATCACGTTTTGGGAAATTCATCCGAAT
TCACTTTGGAAACAGTGGCAAGTTGTCGTCCGCTGACATTGAGACATATT
TGCTGGAGAAGTCACGCGTCACCTTTCAGCTCAAGGCTGAGAGGAATTAC
CACATCTTCTACCAGATCCTGTCCAATCAGAAACCAGAGCTGTTGGACAT
GCTGTTGGTCACAAACAACCCATACGACTACTCCTACATCTCTCAAGGAG
AAGTAACGGTCGCATCAATCAATGACTCTGAGGAGCTGATGGCCACYGAC
AGTGCCTTCGATGTGCTCGGCTTTACMGCGGAAGAAAAAATGGGCGTTTA
TAAGCTGACTGGCGCCATCATGCATCACGGCAACATGAAGTTTAAGCAGA
AGCAACGTGAGGAGCAAGCCGAACCTGATGGGACGGAGGCTGCGGATAAA
TCAGCCTATCTGATGGGTCTGAACTCTGCAGACCTCATAAAGGGGCTGTG
CCATCCCAGAGTGAAGGTAGGGAATGAGTTTGTTACTAAAGGCCAAAGTG
TGGACCAGGTCTACTACCCCAACAAAGAGGCCTTCAAGTGTGAGGAGTGT
GGCAAGCACTACAACACCAAGTTGGGATACAAGCGGCATGTGGCCATGCA
CTCCGCCACAGCAGGGGATCTCACCTGTAAAGTTTGCATGCAGAGCTACG
AGAGCACACCAGTGCTCCTGGAACACCTCAAGAGCCACTCTGGGAAGTCC

TCGGGCGGCACCAAGGAGAAAAAACACCCGTGTGATCACTGCGATCGACG
TTTCTACACGCGGAAGGATGTGAGACGGCACATGGTGGTCCACACGGGCC
GGAAGGACTTTCTGTGCCAGTACTGTGCCCAGCGCTTTGGCAGGAAGGAC
CACCTGACACGTCACGTGAAGAAGAGCCACTCTCAGGAGCTGCTGAAGAT
CAAAACGGAGCCCCCGGATATGTTAGGTCTTTTAGCTTCCGGGTCGCCAC
CTTGCTCTGTGAAGGAGGAGCTTAGCCCCATGATGTGTGGCATGGGTCCA
AACAAAGACCCCATGATGGGGAAACCGTTCCCAAGTGGGGCCCCCTTTTCC
GATGGGCATGTACAACCCCCCACCAC------CTCCAGGCCATGTCCAATT
CTGGAGTGGGCCACCCC------CACCCCTCCTTGATGCAGAGCACCCTG
TCTGCTGCTATGGGTATGGGCTGTCACATGGAGTATCTCATCTATGCCTC
TTTCTCATTCATGGGATGTTTACAAATCAGCGACGGATCCAACATTGTGA
ATCTGTTGGCGAGTAACTCTCCGAGCGTGTCGTACGCCGCCACCCAGCAG
AAATACTTTAGCAACTACAGTCCAGTGATTGGGTTTTACATTTACGAGCC
CATTGAGTACTGGAATTCTACGGTGCAGGAGCATCTGAAGACTCTGAGTT
ACGGCTTCAATAAGATCTCCTGGATGGACAACTTTTTCCATTACCTGCGG
GTGGTGAACGTGAGCGCTTCAACCAAGAGCGATTTTATCACCATCCTCAA
GGGTTCTTTCTTGCACAGCCCGGAGTACCAGCACTTCACCGAGGACATCA
TTTTCTCTAAGA---ATCACGAGACTA------ACGAGTATGACATTATT
GCCTCGCGATTGTACTTGGTGGCGAGGACCACCGAGAAGAAGCGTGAGGA
GGTGGTGGAGCTTCTGGAAAAGCTTCGTCCGTTGATGCTGATTAACAGCA
TCAAGTTCATTGCCTTCAACCCCACCTTTGTGTTCATGGACCGCTACAGC
TCCTCTGTAATCTCACCCATACTGACCTCAGGCTTTAGCGTGCTCACTAT
CCTCATCCTCACTTTCTTCCTCGTCATCAACCCCTTAGGGAACTTCTGGC
TCATCCTGACAGTGACATCCGTGGAGCTGGGTGTCTTGGGTCTGATGGGT
TTTCATTCATTTGAATGGCAGCCAGCTCTCAAAAATGTGTCTGCGTCCTG
CAACACAGGCATTATTAACGGGCTCTCAGGGTGGGCCTCTACGGTGGATG
ACGCCCCAGCTGAGACAATCACTCGCCGCTTCCGCTACGATGTGGCGCTG
GTGTCAGCATTAAAGGATCTGGAGGAGGACATTATGGACGGACTGAGAGA
GTGTGGGCTGGAAGACAGCGCTTGTACCTCAGGCTTCAACGTTATGATCA
AGGAGTCTTGTGACGGCATGGGGGATGTCAGCGAGAAACACGGCGGAGGA
CCGGCGGTTCCTGAGAAGGCTGTACGTTTCTCTTTCACTGTTATGTCTGT
CTCCGTCCAGGCGAGCGGAGACGAG-------------------------
-----GAGGCGGTTACCATCTATACAGAGCCCAAGCCAAACTCAGAACTG
TCCTGCAAGCCCCTCTGCCTCATGTTTGTGGATGAGTCAGACCACGAAAC
TCTCACAGCTGTCCTGGGCCCCATCGTTGCAGAGCGAAGAGCAATGAAAG
AGAGCAGACTCATCCTCTCCATCGGGGGCCTGCAGCGATCCTTCCGCTTC
CACTTCAGGGGCACCGGGTACGACGAGAAGATGATGCGTGAGATGGAGGG
CCTGGAGGCGTCCGGTTCGACCTACGTCTGCACTCTCTGCGACGCGAGTC
GGGCAGAAGCCGCGCAGAACAAGGTGYTGCACTCCATCACCCGCGGACAC
GAAGAGAACTTAGAACGCTACGAGATTTGGAGAACCAACCCCTTTTCGGA
GTCCACAGAGGAGCTGCGCGATCGAGTCAAAGGGGTCTCTGCCAAACCCT
TTCTGGAGACGCACCCCACAATGGACGCTTTGCACTGCGACATCGGAAAT
GCCACTGAGTTCTACAAAATCTTCCAGGACGAGATAGGGGAGGTGTTCAA
AAAGGT---CAAT---CCCACGCGGGAGGAGCGGCGGAGCTGGAGGGCAG
CACTAGACAAACAGCTGAGGACGAAGATGAAGCTTAAGCCGGTAATGAGG
ATGAACGGGAACTACGCCCGGAAGTTAATGACCCTGGAGGCCGCGGAGGT
TGTGTGTGAGCTGGTGCCATCGGAGGAGCGCAGGGAGGCGTTGAGGGAGC
TCATGAGGCTGTACCTTCAGATGAAGCCCGTTTGGCGCGCCACCTGCCCC
GCCAAAGAATGCCCCGACCAGCTGTGCCGCTACAGCTTCAACTCCCAGCA
CTTCGCCGACCTCCTCTCCTCTACGTTCAGTTACCGCTACAACGGAAAAA
TAACCAATTATTTGCACAAAACCCTGGCCCACGTCCCCGAAATCATTGAG
AGAGATGGATCCATCGGAGCCTGGGCCAGCGAGGGGAACGAATCGGCCAA
CAAATCATACTGTATTGAGATGGGAGCCCTGGGGCCCCAGTGGAAGGAGA

GCCCGCAGCCTTTCAACTGCTCAGTGGAAGATCCGACGAAACAGACAAAG
TTCAAGGGCATCAAGACCTACATTTCCTACCGGGTCACGCCGAGCCACAT
AGGACGCCCCGTCTACAGACGCTACAAACACTTTGACTGGCTGTATAACC
GCTTACTGCACAAGTTCACTGTGATCTCCGTACCCCACCTGCCTGAGAAG
CAGGCCACTGGCCGATTTGAGGAAGACTTCATCGAGAAGCGCAAAAGACG
GCTGATACTGTGGATGAACCACATGACCAGTCACCCAGTCCTCTCCCAGT
ACGAAGGCTTTGAGCACTTTTTGATGTGTGCCGATGACAAGCAGTGGAAA
CTGGGCAAGAGGCGAGCCGAGAAGGACGAAATGGTGGGTGCCCATTTCAT
GCTGACCCTCCAGATTCCTAACGAACACCAGGACCTTCAGGATGTTGAGG
AGCGAGTTGACGCCTTCAAGTCCTTTGCAAGAAAAATGGATGATAGCGTC
CTGCAGCTCACTCACGTTTCCTCAGAGCTGGTGCGCAAACACATCGGTGG
ATTCCGGAAGGAGTTCCAGCGGCTTGGAAATGCATTCCAGTCCATCAGCC
AAGCCTTCATGCTGGACCCTCCCCACTCCTCAGAACCCCTCAACAACGCC
ATCTCCCATNNNNNNNNNNCGTTTCTCAAACTGACCTCTCTGGGCTTCATCATCGGCGTC
GGCGTGGTCGGCAACCTCCTGATCTCCATCCTGCTGGTCAAAGACAAGAG
CCTGCACCGCGCGCCCTACTACTTCCTGCTGGACCTGTGCGCCTCGGACA
TCCTGCGTTCCGCTATCTGCTTCCCTTTTGTCTTCACCTCGGTCAAGAAT
GGCTCTGCCTGGACCTACGGCACGCTCACCTGCAAAGTGATCGCCTTCCT
GGGCGTGCTCTCCTGTTTCCACACGGCGTTCATGCTGTTTTGCGTCAGCG
TGACACGCTACTTAGCCATCGCGCATCACCGTTTCTACACGAAGAGGCTG
ACCTTTTGGACCTGCCTGGCCGTCATCTGCATGGTGTGGACGTTGTCGGT
GGCCATGGCTTTCCCGCCGGTGCTAGACGTAGGGACGTACTCGTTCATCC
GGGAGGAGGACCAGTGCACATTCCAGCACCGCTCCTTCAGGGCCAATGAC
TCCCTGGGCTTCATGCTCCTGCTGGCGCTCATCCTCCTGGCCACACAGCT
GGTTTACCTCAAGCTCATCTTCTTCGTCCACGACCGCCGGAAGATGAAAC
CTGTCCAGTTCGTGCCTGCCGTCAGCCAGAACTGGACCTTCCACGGGCCG
GGCGCCAGCGGGCAGGCGGCGGCCAACTGGCTGGCCGGATTTGGTCGCGG
TCCCACCCCTCCTACTTTACTGGGCATCCGGCAGAACAGCAACGCAGCAG
GCCGCCGGCGTCTACTGGTGCTGGATGAATTCAAAACAGAGAAGAGGATT
AGTAGGATGTTCTACATCATGACGTTTTTCTTCCTGGCACTGTGGGGCCC
GTATCTGGTCGCCTGCTACTGGCGGGTGTTTGCTAGGGGCCCCGTGGTCC
CTGGGGGTTACCTGACGGCAGCCGTGTGGATGAGCTTTGCCCAGGCTGGG
GTCAATCCTTTCATCTGCATCNNNNNNNNNNNNNNN-----------------------------
----

---CATCACTCA
ACAGGCGAAGTCACAGAGAGAGAAGTGGCGTTGGGGATAAATCCTTTCGC
GGACGGCATGGGCGCCTTCAAAATCAACCACAGCTCCCACGATATTGGCT
CCGG---ACAGACGGCGTTTTCCTCCCAGGCG---CCCGGCTAC---GCA
GCGGCCGCCCTGGGA---CACCATCA------CCACCCGACCCACGTTGG
CTCT---TACTCCACGGCGGCTTTCAACTCCACCAGGGACTTTCTGTTCA
GAAATCGGGGTTTCGGGGACGCGGCCGG------------CGCGCAGCAC

AGTTTGTTCGCCTC---------CGGAAGTTT---C------GCAGGGCC
ACATGGACACTCAGATGCAGCGGGACACCTGCTCTTCCCGGGGCTCCACG
AG---CAAGCGGCGAGCCACGCGTCGTCCAACGTGGTCAACAGCCAGATG
CGCCTGGGCTTCTCGGGGGACATGTACGGCCGGGCCGAGCAGTACGGCCA
CGTTACGAGCCCCAGGT---CCGACCACTACGCGTCGACCCAGCTGCACG
GCTACGGCCCCATGAACATGAATATGGCCGCT---CACCACGGAGCGGGG
GCCTTCTTTCGATACATGAGGCAGCCGATCAAGCAAGAGCTCATCTGCAA
GTGGATCGAGCCGGAGCAGTTGACCAACCCCAAAAAGTCGTGCAACAAAA
CTTTTAGCACCATGCACGAGTTGGTGACCCATCTGACGGTGGAGCATGTG
GGGGGGCCCGAGCAGACCAACCATATCTGCTTCTGGGAGGACTGCTCCAG
AGAAGGGAAGCCGTTCAAAGCCAAATACAAACTTGTAAATCACATCCGAG
TACACACCGGAGAAAAACCCTTTCCGTGTCCCTTCCCGGGCTGTGGCAAA

>Callionymus bairdi
AGCCTTCTTATCCGTGCTGAACTAAACCAACCAGGAGCTCTTCTTGGCGA
CGACCAGATCTACAATGTCATTGTCACTGCACATGCATTTGTAATGATTT
TTTTTATGGTCATGCCTATCATGATTGGAGGCTTTGGAAACTGACTAGTT
CCAATGATGATTGGAGCCCCAGACATGGCCTTCCCTCGAATAAACAATAT
GAGCTTTTGACTTCTGCCCCCCTCATTCCTACTCCTTCTAGCCTCGTCTG
GGGTAGAGGCCGGGGCAGGGACTGGATGAACTGTCTACCCGCCCCTGTCC
AGCAATCTTGCCCACGCTGGGGCCTCAGTAGACTTAACCATCTTTTCCCT
TCACTTAGCAGGTATCTCATCTATTTTAGGAGCTATTAACTTCATCACTA
CAATTACGAACATGAAGCCCCCAGCCCTAACCCAATATCAAACCCCTCTC
TTTGTGTGGGCCGTCTTAATTACAGCCGTCCTTCTACTTCTATCCCTGCC
CGTCCTGGCCGCCGGAATCACAATACTTTTAACCGACCGTAACCTCAATA
CCACTTTTTTCGACCCTGCTGGCGGAGGAGACCCCATTCTTTATCAGCAC
CTCTTC--

------------TTCCTGGAAAGAAACCTTCACCCATCCAACTGCCTGGG
AATGCTGCTGCTGTCCGACGCACACCAGTGTACCAAGTTGTCCGAGCTTT
CCTGGGGAATGTGCCTCAGTAACTTCCCTGCCATTTGCAAGACAGAGGAT
TTTCTGCAACTGCCCAAAGATATGGTCGTCCAGCTTCTGTCGCACGAGGA
GTTGGAGACCGAAGACGAGCGGCTTGTCTACGAAGCCGCCTTGAACTGGA
TCAACTATGATCTGGAGAGACGGCACTGCCACCTACCAGACTTGCTCAGA
ACGGTCCGGCTGGCTCTTCTTCCCGCTATCTTTTTAATGGAGAACGTCTC
AACCGAAGACCTGATCAATGCTCAGGCCAAGAGCAAGGAGCTGGTGGACG
AGGCCATCAGATGCAAGCTGAAGATCCTCCAGAACGACGGTGTGGTGAAT
AGCCAGTGTGCCCGGCCGAGAAAGACCAGTCATGCCCTCTTTCTTCTGGG
AGGACAGACCTTCATGTGCGACAAGTTGTACCTGGTGGATCAGAAGGCCA
AAGAGATCATCCCAAAGGCCGACATTCCAAGTCCAAGAAAGGAATTCAGC
GCCTGTGCCATCGGCTGTAAGGTCTACATCACCGGCGGAA--GAGGGTC-
TGAGAACGGTGTGTCCAAAGACGTCTGGGTCTACGACACAGTGCAAGAAG
AATGGTCCAAAGCGGCTCCCATGCTCATCGCACGATTCGGCCATGGCTCT
GCTGAGCTCAAGCACNNNNNNNNNNNNNNNNNNNNNNNNNNNNNNNNNNNNNNNNNNNNNNNNNNNN
NNNNNAGACGAATACATTGTTGTGTTCAGTCGCTCCACAA
CACGACTCATACTGAACGAAGCTGAGCTCATCGTGGCGCTGGCTCAAGAA
TTCAAAATGAGAGTGTTTTCGGTGTCTCTAGAGGAGCAGTCCTTCCCGAG
CATTGTCCAGGTCGTCAGTGGAGCTTCGATGTTGGTCAGCATGCATGGAG
CTCAGCTTATCACCTCACTCTTTCTCCCCAAAGGAGCGACTGTTGTTGAA
CTTTTCCCCTTTGCTGTCAATCCAGAGCAGTACACCCCATATAAAACCTT
AGCCACCCTGCCAGGTATGGACCTGCACTATGTTTCCTGGAGGAACCCTA

AGGAGGAAAACACTGTCACTTACCCAGRCAGGCCATGGGAACAAGGAGGC
ATCGCTCACTTGGAGAAGGCAGAGCAGGAGCGAATACTCAACAGCAGAGA
CGTTCCCAGGCACTTGTGCTGCCGCAACCCAGAGTGGCTGTTCCGAATTT
ACCAGGACACTTTAATAGACGTTCCTTCATTCCTAGCTGTCCTCAA---G
GAGGGCATAAGG---ACCAAGCCCAGTGCGAAGAA---GTCCAAAATAGC
TAGCATGGTTCATCCAGGCCGCGTTAGGGAAGCTCGGTGTCAAACCTCAG
TACAGACCAGCAGTGAGGCCAAACTGTTAGTTTCCTGGCAGATCCCATGG
AACCTTAAATTCTTAAAAGTCAGAGAGGTGAAGTACGAAGTTTGGATTCA
AAAAAAAGACACCAGCAAAGGCACCCTGGAGGATCAAATCATACAGGCCA
ATCCCGCACTGGAGGCCTTTGGCAACGCAAAAACACTTCGGAACGACAAC
TCTTCCCGTTTCGGAAAATTTATCCGGATACACTTTGGCACCAGCGGAAA
GTTGTCGTCTGCCGACATTGAGACATACTTGCTGGAAAAGTCACGTGTTA
CTTTTCAGCTGAAGGCTGAGAGAAACTACCACATATTCTACCAGATCCTG
TCCAATCAGAAGCCGGAGCTTCTCGACCTGCTGCTCATCACCAACAATCC
ATACGATTACCCGTACATCTCCCAGGGTGAGGTGACAGTCGCCTCCATCA
ATGATGCCGATGAGTTGATGGCCACTGACAGTGCCTTTGATGTGCTGGGC
TTCACCCCTGAGGAGAAAATGGGCGTTTACAAGCTCACCGGCGCCATCAT
GCACCATGGCAACATGAAGTTCAAGCAGAAGCAGCGTGAGGAGCAGGCTG
AGCCGGATGGAACCGAAGCTGCCGACAAGGCTGCGTATCTGATGGGGCTG
AACTCTGCCGACCTCATCAAAGGGCTGTGCCACCCTCGGGTGAAGGTCGG
AAATGAGTTTGTCACCAAAGGTCAGAGTGTGGACCAAGTCTACTATCCCA
ACAAGGAGGCTTTCAAGTGCGAGGAGTGCGGGAAGCACTACAACACCAAG
CTTGGATATAAGCGCCATGTGGCTATGCACTCCGCTACCGCTGGTGACCT
CACCTGTAAAGTGTGCATGCAGAGCTATGAGAGCACGCCTGTTCTCCTGG
AGCACCTCAAGAGCCACTCTGGCAAGTCCTCAGGTGGAGCCAAGGAGAAG
AAACACCCATGTGACCACTGTGACCGGCGTTTCTACACACGAAAGGATGT
GAGAAGACACATGGTGGTGCACACAGGCCGGAAGGATTTCCTGTGCCAGT
ATTGTGCCCAGCGCTTCGGCAGGAAGGATCACCTGACACGCCATGTGAAA
AAGAGCCACTCGCAGGAGCTGCTGAAAATWAAGACTGAGCCTCCGGACAT
GTTAGGTCTTTTAGCTTCAGGGTCACCGCCTTGTTCTGTGAAAGAGGAGY
TAAGCCCCATGATGTGCGCCATGGGTACCAATAAAGACCCCATGATGGGT
AAAGCTTTCCCTAGTGGTGCCCCATTTTCAATGGGCATGTACAACCCCCA
CCAT------CTTCAGGCCATGTCAAATTCTGGGGTGGCTCATCCA----
--CACCCGACCCTGATGCCTGGCTCCCTGTCTGCAGCAATGGGCATGGGC
TGTCACGTGGACTATCTCATCTACGCCTCTTTTTCATTCATGGGATGTTT
ACAAATCAGCGATGGCTCAAATATTGTAAATCTCCTCGCCAGTAACTCAC
CAAGTGTTTCATACGCGCTGACACAGCAGAAATACTTCAGCAACTACAGT
CCTGTGATCGGATTTTACATTTATGAGCCCCTCGAGTACTGGAACTCCAC
AGTGCAGGAGCACATAAAGACTCTGAGTCACGGTTTCAACAAGATCTCCT
GGATGGACAATTTTTTCCACTACCTGCGGGTGGTGAATGTGAGTGCGTCG
ACAAAGAGTGATTTTATCTCGATTCTGAAGGGCTCCTTCCTGCGGAGCCC
CGAGTACCAGCACTTCACCGAGGACATCATATTTTCRAAGA---ACCATG
AAACTG------ATGAATACGACATCATCGCCTCGCGCATGTACCTGGTG
GCGCGGACCACCGAAAAGAAGCGGGAAGAAGTGGTGGAGCTTCTGGAGAA
GCTGCGCCCGTTGATGCTCATCAACAGCATCAAGTTCATTGCCTTCAACC
CCACGTTTGTTTTTATGGACCGCTACAGTTCCTCCGTCATTTCGCCCATA
TTAACCTCAGGCTTTAGTGTTCTAACTATTCTCGTCCTCACGTTATTCCT
CGTCATCAACCCCCTTGGGGAACTTTTGGCTCATCCTCACAGTTACATCCG
TGGAACTCGGTGTCTTGGGTCTGATGNNNNNNNNNNCAGTTTGAATGGCAGCCGTCGCTT
AAGAATGTCTCCGCGGATTGGGGCGTCGGCATTATCAATGGACTCTCCGG
ATGGACTTCCTCCGTGGATGAGTCTCCAGCTGCCACCATCACTCGACGCT
TCCGCTATGATGTTGCCTTGGTGTCAGCGCTAAAGGATATGGAGGACGAG
ATCTTAGAGGGACTGAGGGAGAATGGAATCGAAGACAGTGCTTGCACAGC

CGGCTTCAGTGTTCTGATAAAAGAGTCTTGTGATGGCATGGGAGACGTCA
GTGAAAAGCATGGGGGGGGACCGGCCATCCCAGAGAAGGCCGTGCGCTTC
TCCTTCACTCTGATGTCCATTTCTCTGTCAAAAGACAACGGCGAG-----
-------------------------AAAGACGTCACCATTTTCACAGAAC
CCGGGCCCAACTCAGAACTGTCCTGCAAGCCCCTTTGTTTGATGTTCGTC
GATGAATCGGATCACGAGACCCTCACTGCAGTGCTGGGGCCAATCGTTGC
TGAGCGTAACGCGATGAAGGAAAGCCGGCTCATCCTCTCCATCGGAGGGC
TTCAACGCTCGTTCCGCTTCCACTTCAGAGGGACGGGGTACGATGAAAAG
ATGGTGAGAGAACTGGAAGGCCTCGAATCCTCAGGGTCCGCCTATATTTG
CACTCTGTGTGACTCCAGTCGAGCGGAAGCCTCTAGAAACATGGTGCTGC
ACGCCGTCACCCGCAGCCACGGTGAGAACCTGGAGCGTTATGAAATATGG
AGAACCAATCCGTTTTCCGAGTCGGTAGAGGAACTACGAGATAGAGTCAA
GGGGGTCTCCGCTAAGCCCTTCATGGAAACTCAGTCCACACTAGACGCGC
TGCACTGTGACATCGGCAATGCCACAGAGTTATACAAAATATTCCAGGAT
GAAATCGGAGAGGTTTTTAAAAAGGT---CAAT---CCCAGCCGGGAGGA
GCGGCGCAGCTGGAGGGCAGCGCTGGACAAAGAGCTGAGGAGGAAGATGA
AGCTGAAACCTGTGATGAGGATGAATGGGAACTATGCCCGAAAGCTAATG
ACTCTAGAGACAGTGGAGGTTGTGTGTAAGCTGGTGCCCTCAGCGGAGAG
GAGGGAGGCCCTGAGGGAGCTCATGCGGCTGTATCTCCAGATGAAGCCTG
TGTGGCGGGCAACCTGCCCAGCCAAAGAATGCCCCGACCAGCTGTGCCGC
TACAGCTTTAACTCGCAGCACTTTGCCGACCTCCTCGCCACCACATTTAA
ATATAGATACAACGGAAAGATAACCAATTACCTGCACAAAACCCTGGCTC
ACGTGCCCGAAATCATAGAGAGAGACGGTTCCATCGGAGCGTGGGCGAGC
GAGNNNNNNNNNNNNNNNNNNNNNTCATATACTATTGAGATGTGCCCAATAGGACCTAAGTGGAAGGAAA
G
CCCACAGCCCTTCTCCTGCTCAATTGAAGATCCTACAAAACAGACCAAGT
TTAAAGGAATAAAGACATACATATCCTACCGGGTCACGCCAAGTCATACG
GGTCGACCTGTTTACAGACGTTATAAACACTTTGACTGGCTATACAACCG
CCTGTTGCACAAGTTCACCGTGATCTCAGTGCCTCACCTGCCAGAGAAGC
AGGCCACAGGGCGCTTTGAGGAAGACTTTATTGAGAAACGCAAGAGACGT
TTGATTTTGTGGATGAACCACATGACAAGTCACCCAGTCCTTTCTCAGTA
TGAAGGCTTTGAACATTTCCTTATGTGTGCTGATGATAAACAATGGAAGC
TGGGTAAAAGGAGAGCGGAGAAGGATGAGATGGTTGGGGCCCACTTTATG
CTGACTTTGCAAATTCCCAACGAACACCAGGACCTTCAGGATGTTGAGGA
AAGAGTGGACACTTTTAAGGCCTTTGCAAAGAAAATGGATGACAGTGTCT
TGCAGCTCACTCATGTGGCCTCCGAGCTGGTGCGAAAGCATCTGGGTGGA
TTCAGGAAAGAGTTCCAACGCCTTGGAAATTCCTTCCAGTCCGTCAGTCA
GTCGTTTATGCTTGACCCTCCTAACAGCTCTGAGGCCCTCAACAGCGCCNNNNNNNNNNNNNNNNNNA
CGTTCCTCAAATTGACCTCACTTGGTTTCATCATCGGCATCGGCGTGGTC
GGGAACCTCCTGATCTCCATCCTGCTGGTCAAAGACAAGAGTCTGCACCG
GGCGCCCTACTACTTCCTGCTGGACCTCTGCGCCTCGGACATCCTTCGCT
CAGCCATCTGCTTTCCCTTCGTCTTCACATCGGTCAAGAATGGTTCGGCG
TGGAGCTACGGCACGCTGACCTGCAAGGTGATCGCCTTCCTGGGCGTGCT
GTCTTGCTTCCACACGGCGTTTATGCTCTTCTGTGTCAGCGTTACACGGT
ACCTGGCCATCGCCCACCACCGCTTCTACACCAAGAGGTTGACCTTCTGG
ACCTGCCTGGCCATCATCTGCATGGTGTGGACGCTGTCGGTGGCGATGGC
ATTCCCGCCGGTACTGGACGTGGGGACGTACTCATTCATCCACGAGGAGG
ACCAGTGCACTTTCCAGCACCGGTCGTTCCGGGCCAACGACTCGCTAGGC
TTCATGCTCCTGCTGGCGCTCATCCTTCTCGCCACACAGCTGGTTTACCT
CAAGCTCATATTCTTTGTACACGACCGTCGGAAGATGAAGCCGGTCCAGT
TCGTACCCGCTGTGAGCCAGAATTGGACTTTCCATGGACCAGGGGCGAGC
GGGCAAGCCGCAGCCAACTGGTTAGCTGGATTTGGCAGAGGCCCAACACC
ACCGACTTTGCTGGGAATCCGTCAGAACAGCAATGCCGCCGGTCGACGCC

GTCTTCTGGTTCTGGATGAGTTTAAGACGGAGAAGAGGATTAGTAGGATG
TTCTACATCATGACGTTTTTCTTCTTGGCTCTGTGGGGCCCGTACCTGGT
CGCCTGCTACTGGCGGGTGTTTGCCAGGGGTCCGGTGGTCCCRGCGGGCT
ACCTGACGGCTGCCGTGTGGATGAGCTTTGCGCAGGCCGGGGTCAATCCT
TTCATCTGCATCTTCTCCAACCGAGAG-----------------------

---NNNNNCTC
AACAGGCGAAGTCACAGAGCGAGAAGTGGCGTTGGGGATCAATCCCTTCG
CAGACGGGATGGGCGCCTTCAAGATCAACCACAGTTCCCACGACATCGGC
TCCGG---GCAGACGGCGTTTTCCTCGCAGGCG---CCCGGTTAC---GC
AGCAGCCGCCCTCGGG---CACCACCA------CCATCCGACCCACGTTG
GCTCC---TACTCCA------CGTTCAACTCCACCCGGGAGTTTCTGTTC
AGGAATCGAGGCTTCGGGGACGCAGCCGG------------AGCGCAGCA
CAGTTTGTTCGCCTC---------CGGGAGTTT---C------GCGGGGC
CACACGGACACTCAGATGCGGCGGGGCACCTGCTCTTCCCCGGGCTCCAC
GAG---CAAGCGGCCAGCCACGCTACCTCCAATGTGGTCAACAGCCAGAT
GCGACTGGGCTTCTCCGGGGACATGTACGGACGGGCGGACCAGTACGGTC
ACGTTACGAGTCCACGCT---CCGACCACTACGCGTCCACCCAGTTGCAC
GGCTACGGCCCCATGAACATGAACATGGCCGCG---CACCACGGAGCCGG
GGCCTTCTTCAGGTACATGAGACAGCCCATCAAGCAGGAGCTCATCTGCA
AGTGGGTCGAACCGGAGCAGCTGACGAACCCCAAAAAGTCGTGCAACAAA
ACTTTCAGCACGATGCATGAACTGGTGACCCATCTGACCGTGGAGCATGT
GGGGGGGCCGGAGCAGACCAACCATATCTGCTTCTGGGAGGAATGTGCCA
GAGAGGGGAAGCCCTTCAAAGCGAAATACAAACTTGTGAATCATATCCGG
GTACACACGGGGGAGAAGCCCTTCCCGTGCCCTTTCCCAGGCTGTGGCAA
A
>Cantherhines pullus
AGCCTTTTAATTCGGGCGGAGCTAAGCCAGCCCGGCGCCCTTCTGGGGGA
CGACCAAATTTATAATGTGATCGTCACAGCTCACGCTTTCGTAATAATTT
TCTTTATAGTAATGCCAGTCATGATTGGAGGCTTCGGAAACTGGCTTGTT
CCACTGATAATTGGCGCCCCCGACATGGCCTTTCCTCGAATGAACAATAT
GAGCTTCTGACTGCTCCCCCCTTCTCTTCTTCTTCTCCTCGCTTCTTCAG
GAGTTGAGGCTGGAGCCGGAACTGGTTGGACCGTATACCCCCCTCTAGCA
GGCAACCTTGCCCATGCAGGAGCATCCGTAGATTTAACAATTTTCTCTCT
GCATCTAGCGGGTATCTCTTCAATCCTTGGTGCAATTAATTTCATTACAA
CCATCATTAACATGAAACCTCCGACTATTTCCCAATATCAAACCCCTTTA
TTCGTATGAGCTGTCCTAATTACAGCCGTACTTCTTCTTCTCTCATTACC
TGTACTTGCTGCTGGTATTACAATACTTTTAACTGACCGAAATTTAAATA
CCACCTTCTTCGACCCAGCCGGAGGGGGAGACCCAATTTTATATCAACAT
TTATTC--

------------TTTCTGGAACGGAACCTCCACCCAACTAACTGCCTTGG
CATGTTGCTGCTGTCCGACGCCCACCAGTGCACCAAGTTGTCTGAGCTTT
CTTGGGGCATGTGCCTCAGCAACTTTCCGGCCATTTGCAAAACTGAGGAT
TTCCTCCAACTGCCCAAAGACATGGTGGTGCAGCTCCTGTCACACGAGGA
GCTGGAGACCGAAGACGAGAGGCTGGTTTATGAAGCCGCCCTTAACTGGA
TCAACTACGACCTAGAAAAGAGGCACTGCCACCTGCCCGAGCTCCTGAAA
ACCGTCCGTCTGGCTCTCCTGCCTGCCATTTTTCTCATGGAAAATGTTTC
CATGGAGGAGCTAATCAATGCCCAGCCAAAAAGCAAGGAGCTGGTGGACG
AAGCTATCCGATGCAAATTGAAGATCCTGCAGAACGACGGCGTGGTCAAC
AGTCCGTGCGCTCGGCCGAGAAAAACCAGCCATGCGCTCTTTCTTCTGGG
CGGGCAAACATTCATGTGCGACAAGTTGTACCTGGTGGATCAAAAGGCCA
AAGAGATCATCCCCAAGGCGGACATACCCAGCCCCAGGAAGGAGTTTAGT
GCCTGTGCCATCGGCTGCAAAGTGTACATCACTGGAGGGA--GAGGCTC-
AGAGAATGGCGTATCCAAAGACGTCTGGGTCTACGACACAGTCCATGAGG
AGTGGTCAAAAGCGGCGCCCATGCTCATCGCCAGGTTTGGCCATGGCTCC
GCAGAGCTCAAACACTGCCTTTACGTCGTTGGAGGTCACACTGCAGCCAC
TGGCTGCCTCCCGGCCTCGCCGTCTGGACGAATACATTGTTGTATTCAGT
CGTTCGACGACAAGGCTGATMCTGAATGAAGCTGAGCTGATAATGGCTCT
AGCGCAAGAGTTTCAGATGAGGGTGGTTACAGTATCTCTGGAGGAGCAGT
CGCTCCCCAGCATCGTTCAGGTGATCAGCGGCGCCACCATGTTAGTCAGT
ATGCACGGAGCTCAGCTCATCACCTCGCTCTTCCTGCCCAGGGGAGCCGT
TGTGGTCGAGCTCTTTCCCTTTGCGGTGAACCCAGAGCAATACACCCCAT
ACAAAACCCTTACGTCCCTCCCAGGCATGGACCTTCACTACATTTCCTGG
AGAAACACACTGGAGGAAAATACAATCACCCATCCAGACAGACCTTGGGA
GCAAGGGGGCATCGGCCACTTGGAGAAGGACGAGCAGGAGAGAATCCTGG
CAAGCAGAGACGTCCCTCGGCACCTGTGCTGCCGCAACCCTGAATGGCTC
TTCAGGATCTACCAGGACACTCTGGTGGACATTCCATCCTTCTTGGACGT
CCTCAA---AGAGGGCTTAAAG---ACAAAGCCTGCTTTGAAAAA---GT
CAAAGCCAGCCAGTTCTCTACACCCGGGCAGAGTCAGAGAGCCTCAGTGC
CAGACGTCGGTGCAGACCAGCAACGAGGCTAAACTCACAGTCTCCTGGCA
GATCCCGTGGAATCTGAAATACCTAAAGGTCAGAGAGGTGAAGTACGAGG
TGTGGATCCGGAAGAGGGACACCAGCAAGGGGACTCTGGAGGACCAGATC
ATCCAGGCCAACCCGGCCCTGGAGGCCTTCGGAAATGCCAAGACGCTGAG
GAACGACAACTCGTCCCGGTTTGGAAAATTCATCCGGATCCACTTTGGCA
CCAGCGGCAAGTTGTCGTCAGCTGACATCGAGACGTACCTGCTGGAGAAG
TCCCGCGTCACCTTTCAGCTCAAGGCCGAGAGGAACTACCACATCTTCTA
CCAGATCCTATCCAACCAGAAGCCGGAGCTGCTGGACCTGCTGCTGATCA
CTAACAACCCCTACGACTACTCCTACATCTCCCAAGGCGAGGTGACGGTG
GCCTCCATCAACGACGCCGAGGAGCTGCTGGCCACCGACAGCGCCTTCGA
CGTGCTGGGCTTCACGGCTGATGAGAAGGCCGGCGTCTACAAGCTGACCG
GCGCCATCATGCACTACGGCAACATGAAGTTCAAGCAGAAGCAGCGTGAG
GAGCAAGCCGAGCCTGACGGACCGAGGCCGCCGACAAATCGGCGTACCT
GATGGGCCTGAACTCGGCCGACCTCATCAAGGGCTTGTGCCACCCGCGGG
TTAAGGTGGGGAACGAGTACGTCACCAAAGGCCAGAGCGTGGACCAGGTC
TACTAC--

------------------------NNNNNNNTCTACGCATCTTTCTCATTCATGGGATG
TTTACAAATCAGTGACGGTTCAAATATTGTGAACCTGTTGTCTAGTAACT
CTCCAAGTGTTTCATATGCTTTGACCCAGCAAAAATATTTCAGTAACTAC
AGTCCTGTTATTGGGTTTTACATATACGAGCCTATTGAGTACTGGAATTC
CACGGTGCAGGAGCACCTTAAGACTCTGAGTCATGGCTTCAACAAGATAT
CCTGGATGGACAACTTCTTCCACTACCTGAGGGTGGTAAATGTGAGCGCG
TCAACCAAGAGTGACTTCATTACCATCCTCAAAGGGTCTTTCTTGCGCAG
CCCAGAGTACCAACACTTCACTGAGGACATTATATTCTCCAAAA---ACC
GAGACACTG------ATGAATATGATATTATTGCCTCTCGGATGTACTTG
GTGGCTCGGACAACGGAGAAGAAGCGAGAAGAGGTAGTTGAGCTGTTGGA
GAAGCTCCGACCACTAATGCTGATAAACAGCATCAAGTTCATTGCTTTCA
ACCCTACTTTTGTCTTCATGGACCGCTACAGCTCCTCTGTCATTTCGCCT
ATCCTGACCTCAGGATTCAGTGTACTAACAATCCTTATTCTCACTTTCTT
CCTGGTCATCAACCCCCTGGGAAACTTTTGGCTCATTCTAACTGTAACAT
CTGTGGAGTTGGGCGTATTGGGTTTGATGGGTTTTCACCAGTTTGAATGG
CAGCCAGCTCTCAAGAATGTGTCCCCGTCCTGCGGCGTGGGCATCATTAA
TGGACTGGCTGGATGCTCTTCCACCGTGGACGACTCGCCCGCTGACACCA
TCACTCGCCGCTTTCGCTACGACGTGGCGCTGGTGTCGGCTTTAAAGGAC
CTGGAGGAAGACATCGTGGACGGGCTGAGAGAGAACGAGATGGATGACAG
CGCCTCCACGTCAGGCTTCAGCGTCATGATCAAAGAGTCCTGTGACGGCA
TGGGGGACGTCAGCGAGAAGCACGGCGGAGGGCCGCTCGTTCCGGAGAAG
GTTGTGCGCTTCTCTTTCACTATCATGTCTGTCTCCATCCTGGCGGAAGG
GAAAGAG------------------------------GGCAAAGTCACCA
TCTACACAGAGCCAAAGCCAAACTCGGAGCTGTCCTGCAAGCCCCTGTGC
CTGATGTCCGTCGATGAGTCTGACCACGAGACGCTCTCCGCCATCCTGAG
GCCCATCATTGCAGAGCGCAAAGCAATGAAAGAGAGCCGCCTCATCCTGT
CCATCGGTGGGCTGCCGCGCTCCTTCCGCTTTCACTTCAGAGGCACAGGG
TACGACGAGAAGATGGTGCGGGAGATGGAGGGTCTCGAGGGCTCTGGCTC
CACCTACATTTGCACGCTCTGCGATTCCAGTCGCCTGGAGGCCTCTCAGA
ACATGGTGCTGCATTCCGTCACCCGCAGCCACGAGGAGAACCTGGAACGT
TACGAAACATGGCGAAGCAACCCCTTCTCCGAGTCTGCGGATGAGCTGCG
GGAACGGGTCAAAGGGGTCTCCGCCAAGCCCTTCATGGAAACGCATCCCA
CGCTTGATGCCCTGCACTGCGACATAGGCAACGCCACTGAGTTTTACAAA
ATCTTCCAGGATGAGATCGGTGAGATGTACAAAAAGGT---CAGC---GC
CAGCCGGGAGGAGCGGCGCAGCTGGAGGGCAGCCCTGGATAAACAGCTGA
GGAAGAAGATGAAGCTCAAACCGGTGATGAGGATGAATGGGAACTACGCC
CGCCGGCTAATGACCCTGGAGGCTGCGGAGGTGGTGTGTGAGCTGGTGCC
CTCCGAGGAGAGGAGGGAGGTCCTGAGGGAGCTGATGAGGCTCTATCTCC
AGATGAAGCCTGTGTGGCGGGCAACCTGCCCGGCCAAGGAGTGCCCTGAC
CAGCTGTGCCGCTACAGCTTCAACTCTCAGCGCTTCGCCGACCTCCTCTC
CTCCAACTTGAAATACAGGTACAACGGGAAGATAACCAACTACCTGCACA
AGACCCTGGCGCACGTGCCCGAAATCATCGAGAGGGACGGATCGATTGGG
GCCTGGGCCAGCGAGGGAAACGAGTCAGNNNNNNNNNNNNNNNNNNNNNNNNNNNNNNNNNNGGGCCTC
GCTGGAAGGAGAGTC
CTCAGCCTTTGTCCTGCTCCGTGGAGGACCCCACCAAGCAAACCAAGTTC
AAGGGCATCAAAACCTACATCTCGTACCGGGTCACCCCCAGCCACACTGG
CCGGCCCGTCTACCGCCGCTACAAACACTTTGACTGGCTGTACAACCGTC

TGCTGCACAAGTTCACCGTCATCTCGGTGCCCCACCTGCCTGAGAAGCAG
GCTACGGGCCGCTTTGAGGAGGACTTCATCGAGAAGCGCAAGAGGCGGCT
GATACTCTGGATGAACCACATGACCAGCCACCCGGTGCTGGCCCAGTACG
AGGGCTTCGAGCACTTCCTCATCTGTGCCGACGACAAGCAGTGGAAGCTG
GGCAAGAGGCGGGCGGAGAAGGACGAGATGGTGGGTGCCCACTTCATGCT
GACCCTGCACATCCCCACCGAGCACCAGGACCTGCAGGACGTGGAGGAGC
GCGTCGATGCCTTCAAGGCCTTTGCCAAAAAGATGGACGACAGCGTGATG
CAGCTGACCCACGTGGCCTCGGAGCTGGTGAGGAAGCACCTGGGCGGCTT
CAGGAAGGAGTTCCAGCGGCTTGGCAACGCTTTCCAGTCGGTCAGCCAGG
CCTTTGCGCTGGACCCTCCGCACTGCTCGGAGCCCCTCAACCAGGCCATC
TCCCACNNNNNNNNNNNNNNNNNNNNNNNNNNNNNNCTGGGTTTCATCATCGGAGTCGGCGTGGTTGGGA
ACCTGCTGAT
CTCCATCTTGCTGGTCAAAGACAAGAGTCTGCACCGAGCGCCCTACTATT
TCCTGCTGGACCTGTGCGCCTCTGACATCCTTCGATCCGCCATCTGCTTC
CCCTTCGTCTTCACCTCGGTCAAGAATGGATCTGCCTGGACGTACGGCAC
GCTGACCTGCAAAGTGATTGCCTTCCTGGGTGTGCTCTCCTGTTTCCACA
CAGCGTTTATGCTGTTCTGTGTCAGTGTCACGCGCTATCTGGCCATCGCT
CATCACCGTTTCTACACTAAGAGGCTGACCTTCTGGACCTGCCTGGCTGT
CATCTGCATGGTGTGGACGTTATCAGTGGCGATGGCGTTCCCGCCTGTGC
TAGACGTAGGGACGTACTCTTTTATCCGGGAGGAGGATCAGTGCACGTTC
CAGCACCGCTCCTTCAGGGCGAATGATTCGCTGGGCTTCATGCTCCTGTT
GGCGCTCATCCTCCTGGCCACACAGCTGGTTTACCTCAAGCTCATCTTTT
TTGTCCATGACCGTCGAAAGATGAAGCCCGTCCAGTTCGTGCCTGCTGTT
AGCCAGAACTGGACCTTCCACGGGCCAGGTGCCAGCGGGCAGGCGGCCGC
CAACTGGCTGGCCGGATTCGGTCGAGGCCCCACTCCGCCTACTTTGCTGG
GCATCAGACAGAACAGCAATGCAGCGGGCCGCAGGCGTCTACTGGTATTG
GATGAATTCAAAACGGAGAAGAGGATTAGTAGGATGTTCTACATCATGAC
ATTTTTCTTCCTGGCACTGTGGGGGCCCTATCTGGTCGCCTGCTACTGGC
GAGTGTTTGCTAGGGGCCCTGTGGTCCCTGGGGGCTACCTGACAGCAGCT
GTGTGGATGAGCTTTGCCCAGGCTGGGGTCAATCCTTTCATCTGCNNNNNNNNNNNNNNNNNN-----
----

NNNNNNNNNNNNNNNNNNNNNNNNNNNNNNNNNNNNNNNNNNNNNNNNNNNNNNNNNNNNNNNNNNNNNN
NNNNNNNNNNNNNNNNNNNNNNNTACCACGATATCTGCTCACG---GCAGACGTCGTT
TTCTTCGCAGGCG---GCCTGTTAC---GCAGCAGCAGCCCTGGGA---C
ACCATCA------CCACCCGACCCACGTTGGCTCT---TACTCCACGGCG
GCTTTCAACTCCACCAGGGACTTTCTCTTCAGAAATCGGGGATTCGGGGA
TGCCACCGG------------GGCGCAGCACAGTTTGTTCGCGTC-----
----CGGAAGTTT---C------GCAGGGCCACATGGACACTCAGATGCA
GCGGGGCACCTGCTCTTCCCGGGGCTCCACGAG---CAAGCGGCGAGCCA

TGCGTCTTCCAACGTGGTCAACAGCCAGATGCGGCTGGGCTTCTCGGGGG
ACATGTACGGACGAGCCGACCAGTACGGCCACGTTACCAGCCCGCGGT--
-CCGACCACTATGCATCGACCCAGCTGCACGGCTACGGCCCCATGAACAT
GAATATGGCCGCG---CACCACGGAGCAGGGGCCTTCTTTCGATACATGA
GGCAGCCGATCAAGCAAGAGCTCATCTGCAAGTGGATCGAGCCGGAGCAG
CTGTCAAATCCCAAAAAGTCGTGCAACAAAACTTTTAGCACGATGCACGA
GCTGGTGACCCATCTGACGGTGGAGCATGTGGGGGGACCGGAGCAGACCA
ACCACATCTGCTTCTGGGAGGAGTGCTCCCGAGAAGGAAAGCCATTCAAA
GCCAAATACAAACTNNNNNNNNNNNNNNNNNNNNNNNNNNNNNNNNNNNNNNNNNNNNNNNNNNNNNNNN
NNNNNNNNNNN
>Capros aper
AGTTTACTTATTCGGGCCGAACTAAGCCAACCTGGCGCCCTCTTAGGCGA
CGATCAAATTTACAACGTGATCGTTACGGCCCATGCCTTTGTAATAATTT
TTTTTATAGTAATACCCATTATAATTGGGGGCTTTGGAAATTGACTTATT
CCGTTGATAATCGGGGCCCCCGATATGGCCTTCCCTCGAATAAATAACAT
AAGCTTTTGGCTCCTCCCACCCTCCTTTCTCCTCCTCCTTGCCTCTTCAG
GTGTAGAGGCAGGTGCGGGAACAGGTTGAACAGTTTATCCCCCTCTGGCC
GGCAACTTAGCCCACGCCGGGGCATCCGTCGATTTAACTATTTTTTCTCT
ACATTTAGCAGGAATCTCCTCTATCCTTGGGGCCATCAACTTTATTACAA
CAATTATTAATATAAAACCCCCTGCAATTTCCCAATACCAGACCCCGCTT
TTTGTATGAGCCGTTTTAATTACAGCTGTTCTGCTTCTCCTATCCCTCCC
TGTCCTCGCCGCCGGGATTACAATGCTTCTAACAGACCGAAACCTAAATA
CTACTTTCTTTGACCCGGCAGGAGGAGGGGATCCCATTCTTTATCAACAC
CTC---

NNNNNNNNNNNNNNNNNNNNNNNNNNNNNNNNNNNNNNNNNGCTGTTGCTGTCTGATGCCCACCAGTGCA
CCAAGCTGT
CAGAGCTCTCCTGGGGCATGTGCCTCAGCAACTTCCCCGCTATCTGCAAG
ACCGAGGACTTCCTCCAACTGCCCAAAGATATGGTGGTGCAGCTTTTGTC
ACACGAGGAGCTAGAGACCGAGGATGAGAGACTGGTTTATGAGGCTGCCC
TCAATTGGATCAACTATGACCTGGATAAGAGGCACTCTCACCTGCCGGAG
CTGCTGAGGACGGTTCGCCTGGCCCTACTGCCCGCCATCTTTCTCATGGA
GAACGTTTCTACGGAAGAGCTGATCAACGCCCAGGCCAAGAGCAAGGAGC
TGGTGGACGAGGCTATCCGCTGCAAGCTGAAGATCCTGCAGAATGATGGC
GTTGTTAACAGCCCGTGTGCTCGGCCAAGAAAAACCAGCCATGCCCTCTT
TCTCCTCGGCGGGCAGACCTTCATGTGCGACAAGTTGTACCTGGTGGACC
AGAAAGCCAAAGAGATCATCCCCAAAGCTGACATTCCCAGCCCAAGGAAG
GAGTTTAGCGCCTGCGCCATCGGCTGCAAGGTGTATATCACTGGCGGGA-
-GGGGCTC-GGAGAACGGCGTCTCCAAAGATGTGTGGGTCTACGACACCG
TCCACGAGGAATGGTCAAAGGCGGCGCCCATGCTCATCGCCAGGTTTGGC
CACGGTTCTGCTGAGCTTAAACACTGCCTGTACGTGGTGGGAGGTCACAC
CGCCGCAACTGGCTGCCTCCCGNNNNNNNNNNNNTGACAATTACATTGTTGTGTTTAGTCGT
TCAACAACGAGGCTAATACTGAATGAACCAGAGCTTATCATGGCCCTGGT
CCAGGAGTTCCAGATGAGAGTAGTCACTGTATCCCTGGAGGAGCAGTCTC
TCCCCAGCATCGTCCAGGTGATCAGCGGAGCTGCCATGTTGGTCAGCATG
CATGGAGCTCAGCTCATCACCTCACTCTTCCTCCCCAGAGGAGCTGCTGT
GGTGGAGCTGTTCCCCTTTGCTGTGAACCCAGAGCAGTATACCCCATATA
AAACCCTGGCCTCCCTTCCAGGCATGGACCTTCACTACATATCCTGGAGG
AACACGAAGGAGGAGAACACCGTCGCCCACCCAGACAGACCCTGGGAACA
AGGAGGCATCACTCACCTGGAGAAGGAGGAGCAAGAGCGAATATTGGCGA

GCAAAGATGTCCCAAGGCACCTGTGCTGCCGCAACCCAGAGTGGCTTTTC
CGGATCTACCAGGACACTTTGGTGGACATCCCTTCCTTCCTGGAAGTCCT
CAA---AGAGGGCATGAAG---ACAAAGCCCAGCTTGAAGAA---GTCAA
AGCCGGCCAGCACACTTCACCCGGGCCGGGTCAGAGAACCTCAGTGTCAG
ACCTCAGTACAAACCACTAATGAGGCTAAACTCACAGTCTCCTGGCAGAT
CCCCTGGAATTTAAAATACCTGAAGGTGAGAGAGGTCAAATACGAAGTG-
---------AAAAAAGACACGAGCAAGGGGACGCTGGAGGATCAAATCATC
CAGGCCAACCCGGCGCTGGAGGCCTTCGGAAACGCCAAAACCCTGAGGAA
CGACAACTCGTCTCGCTTTGGGAAATTCATCCGAATCCACTTTGGTACCA
GCGGGAAGTTGTCGTCTGCTGACATCGAGACGTACCTGCTGGAGAAGTCA
CGTGTCACCTTTCAGCTCAAGGCTGAGAGGAACTACCACATCTTCTACCA
GATCCTGTCCAATCAGAAGCCAGAGCTGCTGGACATGCTGCTGATCACCA
ACAACCCCTATGACTACTCCTACATCTCCCAAGGAGAGGTGACCGTCGCC
TCCATCAACGACTCAGAGGAGCTGATGGCCACAGACAGCGCCTTCGACGT
GCTGGGCTTCACTCCGGACGAGAAGATGGGCGTCTACAAACTGACGGGCG
CCATCATGCACTACGGAAACATGAAGTTCAAACAGAAGCAGCGGGAGGAG
CAGGCTGAGTCGGATGGGACCGAGTCTGCGGATAAATCGGCCTACCTCAT
GGGGCTGAACTCTGCAGACCTCATCAAGGGGCTCTGCCACCCCAGAGTCA
AGGTGGGAAACGAATATGTGACCAAAGGCCAAAGTGTGGATCAAGTCTAC
TANNNNNNNNNNNNNNNNNNNNNNNNNNNNNNNNNNNGGAAGCACTACAACACCAAGCTGGGATATAAGC
GCCATGTGGCCATGC
ACTCAGCCACGGCAGGGGATCTCACCTGTAAAGTGTGCATGCAGACCTAT
GAGAGCACGCCCGTGCTGTTGGAACACCTCAAGAGTCACTCTGGGAAGTC
CTCAGGTGGCACCAAGGAGAAAAAACACCCATGCGACCACTGTGATCGGC
GTTTCTACACACGAAAGGATGTGAGGCGGCATATGGTGGTCCACACAGGC
AGAAAGGACTTCCTGTGCCAGTACTGTGCCCAACGCTTTGGCAGGAAGGA
CCATCTGACGCGCCATGTGAAGAAGAGCCACTCGCAGGAGCTGCTGAAAA
TCAAGACGGAGCCACCCGATATGTTAGGTCTTTTAGCTTCAGGGTCACCA
CCGTGCTCTGTGAAGGAGGAGCTCAGCCCCATGATGTGCGGCATGGGTCC
CAACAAAGACCCCATGATGGGTAAACCATTCCCCAGTGGGGCTCCTTTTC
CCATGGGCATGTATAACCCCCACCAT------CTCCAGGCCATGTCTAAT
TCTGGGATGGGTCACCCA------CACCCGTCCCTGATGCCCAGTTCCTT
GTCAGCNNNNNNNNNNNNNNNNNNNNNNNNN---
NNNNNNNNNNNNNNNNNNNNNNNNNNNNNNNNNNNNNNNNNNNNNNNNNNNNNNNNNNNNNNGGTGAATC
TGCTGGCTAGTAACTCTCCAAGTGTATCATATG
CTCTCACCCAACAAAAGTACTTCAGTAACTACAGCCCTGTGATTGGGTTT
TACATTTATGAGCCCATCGAGTACTGGAACTCAACAGTGCAGGAGCACCT
GAAGACTCTGAGTCATGGCTTCAACAAGATTTCCTGGATGGACAACTTTT
TCCACTACCTGCGGGTGGTGAATGTGAGCGCGTCTACCAAGAGTGACTTC
ATCACCATCCTCAAGGGCTCCTTCCTACGCAGCCCGGAGTACCAGCACTT
CAATGAAGACATCATATTCTCCAAGA---ACCACGAGACTG------ACG
AGTACGACATTATCGTCTCACGGATGTACTTAGTGGCACGGACGACAGAG
AAGAAGCGCGAAGAAGTGGTGGAGCTTTTGGAAAAGCTTCGTCCATTGAT
GCTGATTAACAGCATCAAGTTCATTGCCTTCAATCCTACGTTTGTTTTCA
TGGACCGCTACAGCTCCTCTGTCATCTCGCCCATCCTGACCTCAGGATTC
AGCGTGCTCACAATCCTCATCCTCACTTTCTTCCTGGTCATCAACCCCTT
GGGAAACTTCTGNNNNNNNNNNNNNNNNNNNNNNNNNNNNNNNNNNNNNNNNNNNNNNNNNNNNNNNNNN
NNNNNNNNNNNNNNNNNNNNNNNNNNNNNNNNNNNNNNNNNNNNNNNNNNNNNNNNNNNNNNNCGGCTCT
CGGGATGGACGTCCTCATTGGATGACGCCC
CCGCTGACACCATCACTCGACGTTTCCGCTATGATGTGGCACTGGTGTCG
GCATTAAAGGATCTGGAGGAGGACATCATGGAAGGGCTGAGAGAAAGTGG
GATGGAAGACAGCGCTTGCACCTCCGGCTTTAGTGTCATGATCAAGGAGT
CTTGTGACGGCATGGGTGATGTCAGTGAGAAACACGGTGGAGGACCTGCT

ATTCCTGAGAAGGCTGTGCGCTTCTCCTTCACCGTTATGTCTATCTCTGT
CCTGGCTGAAGATGCAGAA------------------------------A
AACCAGTTACCATCTTCACTGAGCCGAAACCAAACTCAGAGCTGTCCTGT
AAGCCCCTTTGCCTGATGTTTGTGGATGAGTCAGACCACGAGACACTCAC
AGCCATCCTGTGGCCTGTAATTGCAGAGCGCAACGCAATGACAGAGAGCC
GGCTCATCGTATCCATCGGTGGACTGCCTCGCTCCTTCCGCTTTCACTTC
AGAGGTACGGGATACGATGAGAAGATGGTGCGTGAGGTGGAGGGCCTCGA
GGCTTCTGGATCCAGCTATGTCTGCACTCTGTGCGACTCGAGTCGGGCGG
AGGCCTCTCAAAACATGGTGCTACACTCCATCACCCGCAGTCACGAAGAG
AACCTAGATCGCTACGAGATATGGAGAACCAACCCCTTTTCTGAGTCTGC
AGATGAACTGCGAGACAGAGTCAAAGGGGTCTCTGCGAAGCCCTTCATGG
AGACCCATCCCACGCTGGACGCTTTACACTGCGACATAGGCAATGCCACA
GAGTTCTACAAAATCTTCCAGGATGAGATCGGGGAGGTGTTCCAAAAGGT
---CAAC---CCGACTCGGGAGGAACGGCGCAGCTGGAGGGCCGCCCTAG
ATAAACAGCTGAGGAAGAAGATGAAGCTCAAACCGATAATGAGGATGAAT
GGGAACTATGCCCGCCGGCTAATGACCCTGGAGGCTGTGGAGGTGGTGTG
CGAGCTGGTGCCCTCAGAGGATAGGAGGGTGGCCCTGAGGGAGCTCATGA
GGCTCTACCTCCAGATGAAGCCCGTGTGGCGCGCCACCTGCCCTGCCAAA
GAATGCCCCGACCAGCTGTGCCGCTACAGCTTTAACTCTCAACACTTCGC
TGACCTCCTCTCCTCGACCTTCAAATATAGGTACAATGGAAAGATAACCA
ATTACCTGCACAAGACCCTGGCCCATGTGCCTGAAATCATAGAGAGAGAT
GGATCCATCGGAGCGTGGGCCAGCGAGGGGAACGAGTCGGCAAACAAA--

NNNNNNNNNNNNNNNNNNNNNNNNNNNNNNNNNNNNNNNNNNNNNNNNNNNNNNNNNNNNNNNNNNNNNNNNNNNNN
NNNNNNNNNNNNNNNNNNNNNNNNNNNNNNNNNNNNNNNNNNNNNNNNNNNNNNNNNNNNNNNNNNNNNNNNNNNNN
NNNNNNNNNNNNNNNNNNNNNNNNNNNNNNNNNNNNNNNNNNNNNNNNNNNNNNNNNNNNNNNNNNNNNNNNNNNNN
NNNNNNNNNNNNNNNNNNNNNNNNNNNNNNNNNNNNNNNNNNNNNNNNNNNNNNNNNNNNNNNNNNNNNNNNNNNNN
NNNNNNCTTCTGTGTCAGTGTCACGCGCTATCTGGCCATCGCACATCACCGT
TTCTACACCAAGAGGCTGACCTTCTGGACCTGTCTAGCTGTCATCTGCAT
GGTGTGGACGTTGTCAGTGGCTATGGCGTTCCCGCCAGTGCTAGACGTAG
GGACGTACTCTTTTATCCGGGAGGAGGACCAGTGCACATTCCAGCACCGT
TCCTTCAGGGCGAATGATTCGCTGGGCTTCATGCTCCTGCTGGCGCTCAT
CCTCCTGGCCACACAGCTGGTTTACCTCAAGCTCATCTTCTTCGTCCACG
ACCGTCGAAAAATGAAGCCTGTCCAGTTCGTGCCTGCCGTCAGCCAGAAC
TGGACCTTCCATGGACCAGGCGCCAGCGGGCAGGCGGCAGCCAACTGACT
GGCAGGATTCGGTCGACGCCCCACCCCGCCTACTCTGCTGGGCATCCGGC
ATAACAGCAACGCACCAGGTCGCAAGCATCTACTGGTATTGGATGAGTTC
AAAACAAAGAACAGGATTAGTACGATGTTCTACATCATGACTTTTTTCTT
CCTGGCACTGTGGGGGCCCTACCTGGTCGCCTGCTACTGGCGCGTGTTCG

CAGGGGCCCCCGTGACCGCTGGGGGCTACCTGACGTCAGCCGTGTGGATG
AGCTTTGCCCAGGCTGGGGTCAATCCTTTCATCTGCATCTTCTCCAGCNNNNNNNNNNNNNNNNNNNNNN
NNNNNNNNNNNNNNNNNNNNNNNNNNNCG
GAGC---GCAGCGTCCCACTCGGCAACAGCTTGCTATCCCCGCAGCAAAG
CGAGGAGCCCACTGTTGCCACCCCCCCGCAGCGATGGTTTGTCACCC---
CTGCCAACAACCGACTGGACTTTGCTGCCTCGGCATACGACGCCGCT---
------GATTTCGCCGGTAACGCGGCCACCTTGCTGTCCTACGCAGCGGC
CGGAGTGAAGGCTC------TTCCCCTGCCGACTGCAGGCTGCTCCAACC
GGCCTCTTGGCTATTACGCAGACCCGTCAG---GCTGG---GGAGGACGC
ACGCCGCCGCAGTACTGTGGCGTGAACAGCAAATCCGGCTCGGTCTTTTC
CTGCTGGCCCTCTAACTCACTCGGAGGCAGGACGGGCA---CC---AACT
ACCTGG---------CTGATGA---GGTC---GACTC---CATCCCGACC
GAGAGGTCACCG---AT---CGGCGGCTCTGAGGAG---ACCAAACCCAA
AGACATAAC---ATCAGA---GTCGAACTGGATAGAG---ACGCCGTCCT
CCATTAAATCCATCGATTCGAGCGATTCTGGCATCTTTG---AACAGGCC
AAGAGGAGACGGATCTCACCTTCTGCCACGCCG---------GTTCCGGA
GACTGTGTCCNNNNNNNNNNNNNNNNNNNNNNNNNNNNNNNNNNNNNNNNNNNNNNNNNNNNNNNNNNNN
NNNAATCCGTTCGCGGATGGGATGGGCGCCTTCAAAATAAAC
CACAGCTCCCACGATATCGGCTCCGG---ACAAACGGCGTTTTCCTCCCA
GGCG---CCCGGCTAC---GCGGCAGCCGCCCTGGGA---CACCACCA--
----CCACCCGACCCACGTTGGCTCT---TACTCCACGGCGGCTTTCAAC
TCCACCAGGGACTTTCTCTTCAGAAATCGGGGATTCGGGGACGCCACCGG
------------CGCGCAGCACAGTTTGTTCGCCTC---------CGGAA
GTTT---C------GCAGGGCCACATGGACACTCAGATGCGGCGGGGCAC
CTGCTCTTCCCGGGGCTCCACGAG---CAAGCGGCGAGCCATGCGTCTTC
CAACGTGGTCAACAGCCAGATGCGGCTGGGCTTCTCGGGGGACATGTACG
GACGGGCAGACCAGTACGGCCATGTCACGAGCCCGCGGT---CAGACCAC
TACGCCTCGACCCAGCTGCACGGCTACGGCCCCATGAACATGAATATGGC
CGCA---CACCACGGAGCAGGGGCCTTCTTCCGATACATGAGGCAGCCGA
TCAAACAAGAGCTCATCTGCAAGTGGATCGAGCCGGAGCAGCTGACGAAC
CCCAAAAAGTCGTGCAACAAAACTTTTAGCACGATGCACGAGCTGGTGAC
CCATCTGACGGTGGAGCATGTTGGGGGACCGGAGCAGACCAACCACATCT
GCTTCTGGGAGGACTGCCCCAGAGAANNNNNNNNNNNNNNNNNNNNNNNNNNNNNNNNNNNNNNNNNNNN
NNNNNNNNNNNNNNNNNNNNNNNNNNNNNNNNNNNNNNNNNNNNNNNNNNNN
>Caranx crysos
AGCCTACTCATCCGAGCAGAACTAAGCCAACCTGGCGCCCTTCTAGGAGA
CGACCAAATTTACAATGTAATTGTTACGGCCCATGCGTTTGTAATGATTT
TCTTTATAGTAATGCCAATCATGATCGGAGGCTTTGGAAACTGACTCATT
CCCCTAATGATTGGTGCCCCCGATATAGCATTCCCCCGAATAAATAATAT
GAGCTTCTGACTTCTCCCTCCCTCTTTCCTCCTACTCCTAGCTTCCTCAG
GGGTAGAAGCCGGAGCTGGGACTGGCTGAACTGTTTATCCCCCACTAGCT
GGTAATCTTGCCCACGCGGGAGCATCAGTAGACTTAACCATCTTTTCCCT
TCACCTAGCAGGGGTTTCATCAATTCTAGGGGCTATTAACTTCATTACCA
CTATTATCAACATAAAACCGCCTGCAGTTTCAATATACCAAATCCCACTA
TTTGTCTGAGCCGTACTAATTACAGCTGTCCTTCTCCTTCTCTCTCTTCC
AGTATTAGCTGCTGGAATTACAATGCTTCTCACAGATCGGAACCTAAATA
CTGCCTTCTTCGACCCAGCAGGAGGTGGGGATCCCATTCTTTATCAACAC
TTA---

------------TTCCTAGAGAGAAACCTTCACCCCTCTAACTGTCTTGG
CATGTTGTTGCTGTCTGATGCCCACCAGTGTACCAAGCTGTCAGAGCTGT

CCTGGGGCATGTGCCTCAGCAACTTCCCTGCTATTTGCAAGACAGAGGAC
TTCCTCCAACTGCCCAAAGATATGGTGATCCAGCTTTTATCTCATGAGGA
GCTAGAGACAGAGGATGAGAGACTGGTGTATGAAGCTGCCCCTTAACTGGA
TCAACTATGACCTGGAGAGAAGGCACTGCCACCTTCCAGAGCTTCTGAGA
ACGGTCCGCCTTGCCCTGCTGCCTGCCATCTTTCTGATGGAGAACGTCTC
AACAGAGGAGCTGATCAATGCCCAGGCCAAGAGCAAGGAACTGGTAGATG
AAGCCATCCGCTGTAAGCTGAAAATCCTGCAGAATGATGGCGTTGTTAAC
AGCCCATGTGCTCGACCTAGAAAAACCAGCCATGCCCTGTTTCTTTTGGG
AGGGCAGACTTTCATGTGTGACAAGTTGTATCTGGTGGACCAGAAGGCCA
AAGAAATCATCCCCAAGGCAGACATCCCCAGCCCCAGGAAGGAGTTCAGC
GCCTGCGCCATCGGCTGTAAGGTCTACATCACTGGAGGGA--GAGGCTC-
AGAGAATGGTGTGTCCAAAGATGTATGGGTTTACGACACTGTCCACGAGG
AATGGTCCAAAGCAGCTCCCATGCTCATTGCMAGGTTTGGCCATGGCTCC
GCAGAGCTGAAACACTGCCTCTACGTGGTAGGAGGTCACACTGCAGCAAC
TGGCTGCCTCCCGGCTTCTCCATCAGGATGATTACATTGTTGTGTTCAGT
CGCTCATCAACAAGGCTGATACTTAATGAGGCTGAGCTAATCATGATGCT
GGCCCAGGAGTTCCAGATGAGAGTGGTCACAGTGTCCCTGGAGGAACAGT
CTTTTCCCAGTATTGTCCAGGTCATCAGTGGTGCATCTATGTTAGTCAGC
ATGCATGGAGCTCAGCTCATAACCTCACTCTTCCTCCCCACAGGAGCTAC
TGTGGTGGAACTGTTCCCCTTTGCTGTGAACCCAGACCATTACACCCCAT
ATAAAACTCTGGCCTCACTACCAGGCATCGACCTTCACTATATCTCTTGG
AGGAACACTAATGAGGAAAACACCATCACCCACCCAGACAGACTCTGGGA
ACAAGGAGGTATTGTTCACTTGGAAAAGGAGGAACAGGAGCGGATACTGG
CAAGTAAGGACGTCCCCAGGCACCTCTGCTGCCGCAACCCTGAGTGGCTC
TTCCGGATCTACCAGGACACTTTAGTGGACATCCCTTCCTTCCTGGAAGC
CCTCAA---AGAGGGCATGAAG---ATGAAACCCAGCTTGAAGAA---GT
CAAAGCCAGCCAACATGGTCCACCCAGGCCGAGTCAGAGAACCCCAGTGT
CAAACCTCAGTACAAACTACTCACGAAGCCAAACTTACAGTGTCCTGGCA
GATCCCATGGAATCTTAAGTACCTGAAGGTGAGAGAAGTAAAGTATGAGG
TGTGGATCCAGAAAAAAGACACCAGCAAGGGGACGCTGGAGGATCAGATC
ATCCAGGCGAACCCTGCACTTGAGGCCTTCGGCAAYGCCAAAACACTGAG
AAATGACAACTCGTCTCGCTTTGGAAAATTCATCCGAATTCACTTTGGTA
CAAGTGGCAAACTGTCATCTGCCGACATCGAGACCTACCTTCTGGAGAAG
TCACGTGTCACCTTCCAGCTAAAGGCTGAGAGAAACTACCACATCTTCTA
CCAGATCCTGTCCAATCAGAAGCCAGAGCTGCTGGACATGCTGCTGATCA
CCAACAACCCATATGACTACTCCTACATCTCGCAAGGAGAGGTAACAGTT
GCCTCCATCAACGACTCAGAGGAGCTGATGGCCACTGACAGTGCCTTTGA
TGTGCTTGGCTTCACTGCAGAGGAGAAGATGGCCGTCTACAAACTGACTG
GCGCCATCATGCACCATGGCAACATGAAATTCAAACAGAAGCAACGTGAG
GAGCAGGCTGAACCTGATGGGACTGAAGCTGCTGATAAGTCTGCTTATCT
CATGGGACTGAACTCTGCTGACCTCATCAAAGGCCTGTGTCACCCTAGAG
TCAAGGTAGGAAATGAGTACGTCACTAAAGGCCAAAGTGTGGACCAAGTC
TACTATCCCAACAAGGAAGCCTTTAAGTGTGAAGAGTGTGGGAAGCACTA
CAACACCCAGCTGGGATATAAGCGTCATGTGGCCATGCACTCCGCCACGT
CTGGGGATCTCACCTGTAAAGTGTGCATGCAGAGCTACGAGAGCACACCT
ATTCTCCTGGAGCACCTCAAGAGCCACTCTGGGAAGTCTTCGGGTGGCAC
CAAGGAGAAGAAGCACCCGTGCGACCACTGTGACCGACGTTTCTACACAC
GGAAGGATGTGAGACGACACATGGTGGTCCACACGGGCCGAAAGGACTTC
CTGTGCCAGTACTGTGCCCAGCGTTTCGGCAGGAAGGACCACCTGACACG
CCACGTAAAGAAGAGCCACTCACAGGAGCTGCTGAAGATCAAGACGGAGC
CTCCAGAYATGTTAGGTCTTTTAGCCTCAGGATCACCACCTTGCTCTGTG
AAGGAGGAGCTCAGCCCCATGATGTGCGGCATGGGTCCCAACAAAGACCC
TATGATGGGCAAACCGTTCCCCAGTGGGGCCCCTTTTCCAATGGGCATGT

ACAACCCCCACCAT------CTACAGGCGATGTCTAATACTGGGGTGGGT
CACCCG------CACCCGCCCCTAATGCCCAGTTCTTTGTCTGCAGCTAT
GGGCATGGGCTGTCACATGGAATATCTCATCTACGCCTCTTTTTCATTCA
TGGGATGTTTACAAATCAGTGATGGGTCAAATATTGTCAACCTGCTGGCT
AGTAACTCTCCAAGTGTTTCCTATGCTCTGACTCAGCAGAAATATTTCAG
TAACTACAGCCCTGTGATCGGGTTTTACATTTATGAGCCCATCGAGTACT
GGAACTCCACGGTGCAGGAGCACCTGAAGACCCTTAGTCATGGCTTCAAC
AAGATCTCCTGGATGGACAACTTTTTCCACTACCTACGTGTGGTCAACGT
GAGCGCGTCAACTAAAAGCGACTTCATCACCATCCTCAAAGGTTCGTTCC
TGCGCAGCCCAGAGTACCAGCACTTCACCGAGGACATCATATTCTCCAAG
A---ACCGCGAGACTG------ATGAATATGACATCATTGCCTCACGGAT
GTACTTGGTGGCGCGGACAACTGAGAAGAAGCGCGAGGAGGTGGTGGAGC
TTCTGGAGAAGCTTCGTCCATTGATGCTAATCAACAGCATCAAGTTCATT
GCCTTCAATCCTACGTTTGTGTTCATGGACCGCTACAGCTCCTCAGTCAT
CTCGCCCATCCTGACCTCAGGCTTCAGCGTACTCACTATCCTCATCCTCA
CTTTCTTCCTGGTCATCAACCCCTTGGGAAACTTCTGGCTCATCCTCACG
GTCACGTCCGTGGAGCTGGGCGTCTTGGGTTTGATGGGCTTTCACCAGTT
TGAATGGCAGCCAGCTCTCAAGAATGTGTCACCATCTTGCAATGTTGGCA
TTATTAATGGTCTCTCTGGATGGGCTTCCTCAGTGGATGACTCCCCGGCT
GACACCATCACTCGGCGCTTTCGCTATGATGTGGCACTGGTGTCAGCATT
AAAGGATCTGGAGGAGGACATCATGGAGGGACTGAGAGAGAAAGAAATGG
AAGACAGTGCTTGCACCTCAGGCTTTACTGTCATGATCAAGGAATGTTGT
GATGGCATGGGCGATGTCAGCGAGAAGCACGGCGGAGGACCAGCTGTTCC
TGAGAAAGCTGTACGTTTCTCCTTCACTGTTATGTCTGTCTCCGTCCTGA
CAGAGG---AGGGG------------------------------GAGGAG
GTTACTATCTTCACCGAAACAAGGCCAAACTCAGAGCTGTCCTGTAAGCC
CCTGTGCTTGATGTTTGTGGATGAGTCAGACCACGAGACACTCACAGCTC
TCCTGGGGCCTGTGGTTGCAGAGCGCAATGCAATGAAAGAGAGCAGGCTC
ATCCTATCCATAGGTGGCCTCTCTCGCTCCTTCCGCTTCCACTTCAGAGG
CACCGGATAYGATGAGAAGATGGTGCGAGACATGGAAGGTCTGGAGTCCT
CGGGGTCCACGTACGTCTGCACCCTGTGTGACTCCAGTCGGGCCGAGGCC
TCTCAAAACATGGTGCTACACTCCGTCACTCGCAGTCATACGGAGAACCT
AGAACGGTATGAAATATGGAGAACAAACCCTTTCTCCGAGTCAGCAGACG
AACTGCGAGACAGAGTCAAAGGGGTCTCTGCAAAGCCCTTCATGGAGACC
CAGCCCACAATTGATGCATTACACTGTGACATCGGCAATGCCACTGAGTT
CTACAAAATCTTCCAGGACGAGATTGGGGAGGTGTATAAGAAGGT---CA
AC---CCCAGCCGGGAGGAGAGGCGCAGCTGGAGGGCAGCCCTAGATAAA
CAGCTGAGGAAGAAGATGAAGCTTAAACCGGTAATGAGGATGAATGGTAA
CTACGCCCGCAGGCTAATGACCCTAGAGACTGTGGAGGTTGTGTGTGAAC
TGGTGCCCTCAGAGGAGAGGAAAGAGGCCCTGAGGGAGCTCATGAGGCTC
TATCTCCAGATGAGGCCTGTGTGGCGCGCCACCTGCCCAGCCAAGGAATG
CCCCGACCAGCTGTGCCGCTACAGCTTCAACTCCCAGCACTTTGCTGACC
TCCTGTCCTCTACCTTCAAATATAGGTACAATGGAAAGATAACCAATTAC
CTTCACAAGACTTTGGCCCATGTGCCTGAAATCATAGAGAGAGATGGATC
CATAGGCGCCTGGGCCAGTGAGGGGAACGAGTCGGCAAACAAATCCTACA
CCATTGAGATGGGTCCCTTGGGGCCCAAGTGGAAGGAGAACCCGCAACCT
TTCTCCTGCTCCATCGAAGACCCCACAAAACAGACAAAGTTCAAGGGCAT
CAAGACCTACATTTCATACCGGGTGACGCCGAGCCACATAGGACGCCCAG
TCTACAGGCGCTACAAACACTTTGACTGGCTGTACAACCGCTTACTGCAC
AAGTTCACTGTGATCTCTGTGCCCCATCTGCCCGAGAAGCAGGCCACGGG
GCGGTTTGAGGAAGACTTCATTGAGAAGCGTAAGAGACGACTGATACTGT
GGATGAACCACATGACCAGTCACCCAGTCCTCTCCCAGTATGAAGGCTTT
GAGCACTTTCTGATGTGTGCTGATGACAAGCAGTGGAAACTGGGAAAGAG

GAGGGCAGAGAAGGATGAGATGGTGGGAGCACATTTCATGCTGACGCTCC
AGATCCCTAATGAGCACCAGGACCTTCAGGACGTAGAGGAGCGGGTCGAC
TCCTTCAAGGCCTTCGCCAAGAAAATGGATGACAGTGTGATGCAGCTCAC
ACATGTTGCCTCAGAGCTGGTGCGTAAACACCTGGGTGGGTTCAGGAAGG
AGTTCCAGAGGTTGGGAAACGCCTTCCAGTCCATCAGCCAGGCATTTATG
CTCGACCCTCCCCACAGCTCAGACGCCCTCAACAACGCCATCTCCCAT--

------------------------------------GCCAAATCTCGCTTT
CACCCTGGCGTGGGGACTGGTCCTGGCACGGAGC---GCAGCGTCCCACT
CGGCAACAGCTTGCTATCCCCGCAGCAAACCGAGGAGCCCACTGTTGCCA
CCCCCCCGCAGCGATGGTTTGTCACCC---CTGCCAACAACCGACTGGAC
TTTGCTGCCTCGGCATACGACGCCGCT---------GATTTCGCCGGTAA
CGCGGCCACCTTGCTGTCCTACGCAGCGGCCGGAGTGAAGGCTC------
TTCCCCTGCCGACTGCAGGCTGCTCCAACCGGCCTCTTGGCTATTACGCA
GACCCATCAG---GCTGG---GGAGGACGCACGCCGCCGCAGTACTGCGG
CGTAAATAGCAAGTCCAGCTCGGTCTTTTCCTGCTGGCCCGCTAACTCTA
TCGGTGCCAGAGCGGGCA---CC---AACTACCTGG---------CCGAG
GA---GGGC---GACTC---CATACCGACGGAGAGGTCACCG---AT---
CGGCGGCTCGGAGGAG---CCCAAACCCAAAGACATGAC---ATCCGA--
-GTCGAGCTGGATAGAG---ACGCCGTCTTCGATCAAGTCAATTGATTCG
AGCGATTCTGGTATCTTTG---AACAGGCCAAGAGGAGGAGAATCTCTCC
GTCTGCCACGCCG---------GTTCCAGAGACAGTGTCCCCGTTAAAAT
CTGAGNNNNNNNNNNNNNNNNNNNNNNNNNNNNNNNNNNNNNNNNNNNNNNNNNNNNNNNNNNNN
NNNNNNNNNNNNNNNAATAAACCACAGCTCCCACGACATTGGCTCCGG---ACAGACGGC
GTTTTCCTCCCAGGCG---CCCGGCTACGCAGCAGCAGCCGCCCTGGGA-
--CACCATCA------CCACCCGACCCACGTTGGCTCT---TACTCCACG
GCGGCTTTCAACTCCACCAGGGACTTTCTCTTCAGAAATCGGGGTTTCGG
GGACGCCACCGG------------GGCGCAGCACAGTTTGTTCGCCTC--
-------TGGAAGTTT---C------GCAGGGCCACATGGACACTCAGAT
GCAGCGGGCCACCTGCTCTTCCCAGGGCTGCACGAG---CAAGCGGCGAG
CCATGCCTCTTCCAACGTGGTCAACAGCCAGATGCGGCTGGGCTTCTCGG
GGGACATGTACGGACGGGCCGATCAGTACGGCCACGTTACGAGCCCAMGG
T---CCGATCACTATGCTTCGACCCAGCTGCACGGCTATGGCCCCATGAA
CATGAATATGGCCGCG---CACCACGGAGCAGGGGCCTTCTTTCGGTACA
TGAGGCAGCCGATCAAACAAGAGCTCATCTGCAAGTGGATCGAACCGGAG

CAGCTGACGAATCCCAAAAAGTCGTGCAACAAAACTTTTAGCACGATGCA
CGAGCTCGTCACCCATCTGACGGTGGAGCATGTGGGGGGACCGGAGCAGA
CCAACCACGTCTGCTTCTGGGAGGACTGCTCCAGAGAAGGAAAGCCATTC
AAAGCCAAATACAAACTTGTAAATCATATCAGAGTACACACCGGAGAAAA
GCCCTTTCCGTGTCCGTTCCCCGGCTGTGGGAAA
>Carapus bermudensis
AGCCTTCTAATCCGAGCTGAGCTCGGCCAACCGGGCGCACTCCTTGGGGA
CGACCAAATCTATAATGTAATTGTCACAGCACACGCATTCGTGATAATCT
TTTTCATGGTTATACCCATTATAATCGGGGGGTTCGGGAATTGACTAATC
CCATTAATGATCGGGGCCCCTGACATAGCGTTCCCTCGCATAAATAACAT
AAGCTTCTGACTCCTTCCTCCGTCCTTCCTACTGCTCCTTGCATCTTCAG
GCGTAGAAGCAGGGGCGGGCACAGGATGAACGGTCTATCCGCCCCTTGCG
GGCAATCTAGCTCATGCTGGGGCCTCCGTGGACCTAACAATCTTCTCCTT
GCATCTTGCCGGGGTTTCATCAATTTTAGGTGCTATTAACTTTATTACCA
CTATTATTAACATAAAACCTCCCGCCGCCTCTCAATACCAGACCCCACTA
TTTGTCTGAGCAGTTCTAATCACGGCAGTCCTCCTGCTACTCTCTCTCCC
CGTGCTTGCTGCCGGCATCACAATGCTGCTCACCGACCGCAACCTAAACA
CAACCTTCTTTGACCCGGCGGGCGGAGGAGACCCAATCCTCTACCAGCAC
CTGTTC--

------------NNNNNNNNNNNGAACCTTCACCCATCTAACTGCCTTGGCATGCTGTTGC
TGTCTGATGCCCATCAGTGCACCAAGCTGTCAGAGCTTTCTTGGGGCATG
TGCCTCAGCAACTTTCCTGCTATTTGCAAGACAGAGGACTTCCTCCAACT
GCCCAAAGACATGGTGGTGCAGCTTTTGTCACATGAGGAGCTGGAGACAG
AAGATGAGAGACTGGTTTATGAAGCTGCCCTGAACTGGATCAACTATGAC
CTGGAAAGGAGGCACTGCCACCTTCCAGATCTTCTGAGAACGGTCCGTCT
GGCCCTGCTTCCTGCCATCTTTCTCATGGAGAATGTCTCGACAGAAGAGC
TGATCAATGCCCAGGCCAAGAGCAAGGAGCTAGTGGATGAGGCTATCCGC
TGTAAGCTGAAGATCCTGCAGAATGACGGCGTAGTTAACAGCCCGTGTGC
TAGACCAAGAAAAACCAGCCACGCACTCTTTCTTCTGGGAGGGCAGACTT
TCATGTGTGACAAGTTGTACCTGGTGGATCAGAAGGCCAAAGAGATCATT
CCCAAGGCTGACATTCCCAGCCCCAGGAAGGAGTTCAGTGCCTGTGCCAT
TGGCTGTAAGGTGTACATCACAGGTGGGA--GAGGCTC-AGAGAATGGCG
TGTCCAAAGATGTATGGGTCTACGACACCGTCCAAGAGGAATGGTCCAAA
GCGGCGCCCATGCTGATTGCCAGGTTTGGTCATGGCTCTGCTGAGCTGAA
ACACTGCCTGTACGTGGTNNNNNNNNNNNNNNNNNNNNNNNNNNNNNNNNNNNNNNNNNNNNNN------
----

----AAGAAAGACACCAGCAAAGGGACACTGGAGGATCAAATCATCCAGG
CAAACCCGGCTCTGGAGGCATTTGGGAATGCCAAAACAGCAAGAAATGAC
AACTCCTCCCGTTTTGGCAAATTCATACGAATTCACTTTGGGAACAGCGG
CAAGTTGTCATCCGCTGATATCGAGACGTATCTGTTGGAGAAATCACGTG
TCACTTTTCAGCTAAAGGCTGAAAGGAACTATCACATCTTCTACCAGATC
CTGTCCAATCAGAAACCAGAGCTTCTGGACATGCTTCTTGTTACCAACAA
CCCATATGACTACTCCTACATCTCTCAAGGGGAGGTAACGGTGGCGTCAA
TCAATGATTCAGATGAGCTCATGGCCACTGACAGTGCCTTCGATGTGCTT
GGTTTCACTCCAGAGGAGAAGATGGGCGTTTACAAACTGACTGGTGCCAT
CATGCACTACGGCAATTTGAAGTTCAAGCAGAAGCAACGTGAGGAGCAGG
CTGAACCTGATGGGACGGAGGCTGCTGATAAATCAGCATACCTCATGGGT
CTGAACTCTGCCGACCTCATTAAAGGGTTGTGCCACCCTAGAGTTAAAGT
GGGGAATGAGTANNNNNNNNNNNNNNNNNNNNNNNNNNNNNNNNNNNNNCCCAACAAGGAGGCCTTCAAG
TGCGAGGAGTGTGGCAA
GCACTACAACACCAAGCTGGGATACAAGCGACACGTGGCCATGCACTCTG
CCACGGCGGGAGATCTGACCTGCAAAGTGTGCATGCAGAGCTACGAGAGC
ACGCCGGTGCTCCTCGAACACCTCAAGAGCCACTCGGGGAAGTCCTCGGG
TGGCACCAAGGAGAAAAAGCACCCCTGCGACCACTGCGACCGTCGTTTCT
ACACGCGGAAGGACGTGAGACGGCACATGGTGGTCCACACGGGACGGAAG
GACTTTCTGTGCCAGTACTGTGCCCAGCGCTTTGGCAGAAAGGACCACCT
GACGCGTCACGTGAAAAAGAGCCACTCGCAGGAGCTGCTGAAGATCAAGA
CGGAGCCGCCGGACATGTTGGGGTTGTTAGCGTCGGGGTCGCCGCCCTGC
CCCGTGAAGGAGGAGCTCAGCCCCATGATGTGCGGCATGGGTCCGAATAA
AGACCCCATGATGGGGAAACCCTTCCCGAGTGGGGCCCCGTTTCCGATGG
GCATGTACAACCCCCACCAT------CTCCAGGCCATGTCCAATTCGGGG
GTGGGCCANNNN------
NNNNNNNNNNNNNNNNNNNNNNNNNNNNNNNNNNNNNNNNNNNNNNNNNNNNNNNNNNNNNNNNNNNNNN
NNNNNNNNNNNNNNNTTCATGGATGTTTACAGATCAGCGATGGCTCGAAC
ATTGTCAATCTGCTGGCCAGTAACTCGCCCAGCGTCTCGTACGCCCTGAC
GCAGCAGAAGTACTTCAGCAACTACAGCCCGGTGATCGGGTTCTACATTT
ATGAGCCAATCGACTATTGGAACTCAACAGTTCAGGAGCATCTGCGGACG
CTTAGTCACGGATTCAACAAAATCTCCTGGATGGACAACTTTTTCCACTA
CCTGCGGGTGGTGAATGTCAGCGCGTCCACCAAAAGCGACTTCATCTCAA
TTCTCAAGGGTTCCTTCCTGCGCAGCCCAGAATACCAGCACTTCACCGAG
GACATCATCTTCACCAAGA---ACCACGAGACAG------ACGAGTACGA
CATCATCGCCTCGCGCCTCTACCTGGTGGCCAGGACGACGGAGAAGAAGC
GCGAGGAGGTGGTGGAGCTGCTGGAGAAGCTGCGACCCCTGATGCTGATC
AACAGCATCAAGTTCATCGCCTTCAACCCCACCTTCGTCTTCATGGACCG
CTACAGCTCGTCCGTCATCTCGCCCATCCTGACCTCAGGCTTCAGCGTGC
TCACCATCCTCATCCTCNNNNNNNNNNNNNNNNNNNNNNNNNNNNNNNNNNNNNNNNNNNNNNNNNNNNN
NNNNNNNNNNNNNNNNNNNNNNNNNNNNNNN---------------------------------
----

NNNNNNNNNNNNNNNNNNNNNNNNNNNNNNNNNNNNNNNNNNNNNGGCGTTGGCGTGGTCGGA
AACCTCCTGATCTCCATCCTGCTGGTCAAAGACAAGAGCCTGCACCGCGC
GCCCTACTACTTCTTGCTGGACCTGTGCGCCTCTGACATCCTGCGCTCCG
CTATCTGCTTCCCCTTTGTCTTCACCTCTGTCAAGAATGGATCTGCCTGG
ACCTACGGCACGCTCACCTGCAAAGTGATAGCCTTTCTGGGCGTGCTCTC
CTGTTTCCACACGGCGTTCATGCTGTTTTGTGTCAGCGTGACCCGCTATC
TAGCCATCGCACATCACCGTTTCTACACCAAGAGGCTAACCTTCTGGACC
TGCCTGGCTGTCATCTGCATGGTGTGGACGTTGTCGGTGGCGATGGCGTT
CCCACCAGTGCTGGATGTGGGGACATACTCTTTCATCCGGGAAGAGGACC
AGTGCACATTCCAGCACCGTTCCTTCAGGGCAAACGACTCCCTGGGCTTC
ATGCTCCTGCTGGCACTCATCCTCCTGGCCACACAGCTGGTTTACCTCAA
GCTCATCTTCTTCGTCCATGACCGCCGGAAGATGAAGCCTGTCCAGTTCG
TGCCTGCTGTCAGCCAGAACTGGACCTTCCACGGGCCGGGAGCCAGCGGG
CAAGCGGCGGCCAACTGGCTGGCCGGATTTGGTAGAGGTCCCACCCCTCC
TACTTTATTGGGCATCCGACAAAACAGCAACGCAGCGGGCCGCAGGCGTC
TACTGGTGCTGGATGAGTTCAAAACAGAGAAGAGGATTAGTAGGATGTTC
TACATCATGACGTTTTTCTTCCTGTCTCTCTGGGGGCCGTATCTGGTAGC
CTGCTACTGGCGGGTGTTTGCAAGGGGTCCCGTGGTCCCTGGGGGCTACC
TGNNNNNNNNNNNNNNNNNNNNNNNNNNNNNNNNNNNNNNNNNNNNNNNNNNNNNNNNNNNNNNNNNNNN
NNNN--

>Cataetyx lepidogenys

------------TTTCTAGAGAGAAACCTTCACCCCTCTAACTGCCTCGG
CATGCTGTTGCTGTCTGATGCCCATCAGTGCACCAAGCTATCAGAGCTGT
CCTGGGGCATGTGCCTCAGCAACTTTCCTGCTATTTGCAAGACAGAGGAC
TTCCTCCAACTGCCCAAAGACATGGTGGTGCAGCTTTTGTCACATGAGGA
GCTCGAGACAGAAGATGAGAGACTGGTTTATGAAGCTGCCCTTAACTGGA
TCAACTATGACCTGGAAAGGAGGCACTGCAACCTTCCAGATCTTCTGAGA
ACGGTCCGCCTGGCCCTGCTTCCTGCCATCTTTCTCATGGAGAATGTCTC

GACAGAAGAGCTGATCAATGCCCAGGCCAAGAGCAAGGAGCTAGTGGATG
AGGCCATCCGCTGTAAGCTGAAGATCCTCCAGAATGATGGCGTAGTTAAT
AGCCCTTGTGCCAGACCAAGAAAAACCAGCCACGCACTCTTTCTGCTGGG
AGGGCAGACTTTCATGTGTGACAAACTGTACCTGGTGGATCAGAAAGCCA
AAGAGATCATTCCCAAAGCTGACATCCCCAGCCCCAGAAAGGAGTTCAGT
GCCTGTGCCATTGGCTGTAAGGTGTACATCACAGGTGGGA--GAGGCTC-
AGAAAATGGTGTGTCCAAAGATGTATGGGTCTACGACACCATCCAAGAAG
AATGGTCCAAGGCAGCACCCATGCTTATTGCCAGGTTTGGNNNNNNNNNNNNNNNNNNNNNNNNNNNNNN
NNNNNNNNNNNNNNNNNNNNNNNNNNNNNNNNNNNNNNNNNNNNNNNNNNNNNNNGGATGAATAC
ATTGTTCTGTTTAGCCGTTCAACAACAAGGCTGATACTGAATGAAGCTGA
GCTAATCAAAACGCTGGCCCAGGAATTTCAAATGAGAGTGGTTACCGTCT
CCCTGGAGGAACAATCTTTCCCAAATATCATCCAGGTGATCAGTGGTGCT
TCCATATTGATTAGTATGCATGGAGCTCAGCTTATCACCTCGCTCTTCCT
CCCCAGAGGAGCTGCTGTGGTGGAGCTGTTCCCCTATGCTGTAAACCCAG
AGCAGTACTCCCCATATAAAACCCTGGCCTCCCTTCCAGCCATGGACCTC
CACTATGTCTCCTGGAGGAACACCATGGAGGAGAACACCGTCACCCACCC
ACACAGACCCTGGGAACAAGGAGGAATCGCTCACTTGGAGAAGGAGGAGC
AACAGCGAATACTTGCCAGCAAAGATGTCCCCAGGTATCTGTGCTGCCGC
AACCCAGAATGGCTCTTCCGAATCTACCAGGACACTTTGGTGGACATCCC
ATCATTCATGGAAGTCCTCAA---AGAGGGCCTGAAG---ACCAAGCCTA
GCTTAAAGAA---GGCCAAGCCGGCCAGCACAGTTCACCCAGGCCGGGTC
AGAGAAGCCCAGTGTCAGACCTCAGTGCAAACCAGCAGTGAGGCTAAACT
CACGGTGTCCTGGCAGATACCGTGGAATCTGAAGTACCTGAAGGTGAGAG
AAGTGAAGTACGAGGNNNNNNNNNNNAAGAAAGACACCAGCAAGGGCACACTGGAAGATCA
AATCATCCAGGCGAACCCAGCACTGGAGGCCTTCGGTAATGCCAAAACAT
TGAGAAATGACAACTCCTCTCGTTTTGGAAAATTCATCCGAATTCACTTT
GGAACCAGTGGTAAGCTGTCATCCGCTGACATTGAGACATATCTGCTGGA
GAAGTCACGTGTCACCTTTCAGCTCAAGGCTGAGAGGGACTACCACATCT
TCTACCAGATCCTTTCCAATCAGAAGCCAGAGCTTTTGGATATGCTGCTA
ATCACAAACAACCCGTATGACTACTCCTACATCTCCCAAGGAGAGGTGAC
AGTTACATCCATCAATGACGCAGAGGAGCTGATGGCCACCGATAGTGCCT
TCGACGTGCTCGGCTTCACTCCAGAGGAGAAGATGACCGTCTATAAGCTG
ACTGGTGCCATCATGCACTACGGCAACATGAAGTTTAAACAGAAGCAGCG
TGAGGAGCAGGCTGAACCTGATGGGACAGAGGCTGCTGATAAATCAGCTT
ACCTGATGGGTCTGAATTCTGCAGACCTCATCAAAGGGTTGTGTCATCCC
AGAGTCAAGGTAGGGAACGAATACGTCACAAAAGGCCAAAGTGTCGACCA
AGTCTACTATCCCAACAAGGAGGCCTTCAAGTGTGAGGAGTGTGGCAAGC
ATTACAACACCAAGCTGGGATATAAGCGACATGTGGCCATGCACTCTGCC
ACAGCAGGGGATCTCACCTGTAAAGTGTGCATGCAGAGCTATGAGAGCAC
ACCAGTGCTCCTGGAGCACCTCAAGAGCCACTCGGGGAAGTCTTCGGGTG
GCACCAAGGAGAAAAAACACCCATGTGACCACTGTGATCGTCGTTTCTAC
ACACGGAAGGATGTGAGACGGCACATGGTGGTCCACACGGGCCGAAAGGA
CTTTCTGTGCCAGTACTGTGCCCAGCGCTTTGGCAGGAAGGACCACCTGA
CACGTCACGTGAAGAAGAGCCACTCTCAGGAGCTGCTGAAGATCAAGACG
GAGCCCCCGGATATGTTAGGTCTTTTAGCATCGGGGTCACCACCTTGCCC
TGTGAAGGAGGAGCTTAGCCCCATGATGTGCGGCATGGGTCCAAACAAAG
ATCCCATGATGGGAAAACCTTTCCCAAGTGGGGCCCCTTTTCCGATGGGC
ATGTACAACCCCCACCAT------CTCCAGGCCATGTCCAGTTCTGGGGT
GGGCCACCCC------CACCCCTCCTTGATGCCTAGTACCCTGTCTGCAG
CTATGGGTATGGGCTGTCACGTGGAATATCTCATCTATGCCTCTTTCTCA
TTCATGGGATGTTTACAAATCAGTGATGGATCAAACATCGTGAACCTGCT
GGCTAGTAACTCTCCCAGTGTTTCATACGCTCTGACTCAGCAGAAATACT
TCAGCAACTACAGTCCCGTGATTGGTTTTTACATTTATGAGCCCATTGAG

TACTGGAACTCCACGGTGCAGGAGCATCTGAAGACTCTAGGTCACGGCTT
CAACAAGATCTCCTGGATGGACAACTTTTTCCACTACCTGCGGGTGGTGA
ATGCGAACGCATCGACCAAGAGTGATTTTATCACCATCCTCAAGGGCTCT
TTCTTACGCAGCCCGGAGTACCAGCACTTCACTGAGGACATCATCTTCTC
TAAGA---ACCGTGAGACTG------ATGAGTATGACATTATTGCCTCAC
GCATGTACTTGGTGGCGAGGACCACAGAGAAGAAACGTGGGGAGGTGGTG
GAGCTGCTGGAAAAGCTACGTCCATTAATGCTGATTAACAGCATCAAGTT
CATTGCCTTCAATCCCACTTTTGTGTTCATGGACCGCTACAGTTCCTCTG
TCATTTCACCCATCCTGACCTCAGGCTTTAGTGTGCTCACTATCCTCATT
CTCACTTTCTTCCTGGTCATCAATCCCTTAGGGAACTTCTGGCTCATTCT
GACAGTTATTTCTGTGGAGCTGGGCGTCTTGGGTTTGANNNNNNNNNNNNNNNNNNNNNNNNNNNNNNNN
NNNNNNNNNNNNNNNNNNNNNNNGAATGTTGGCAT
TATTAATGGACTCTCTGGATGGGCTTCCACTGTGGATGACTCCCCAGCTG
ACACCTTCACTCGACGGTTTCGCTATGATGTGGCGCTGGTGTCAGCATTA
AAGGATCTGGAGGAGGACATCATGCAGGGATTGAGAGAGTGTGGAATGGA
AGACAGTACTTGCACCTCAGGCTTCAGTGTTTTAATCAAGGAATCTTGTG
ATGGCATGGGCGATGTCAGCGAGAAGCATGGCGGAGGACCACCGGTTCCA
GAGAAGGCTGTACGTTTCTCTTTCACCATCATGTCTGTCTCTGTCCTGGC
AGACGGCGAGGAG-------------------------------GAAGCGG
TTACCATCTTTACAGAGCCAAAGCCAAACTCAGAGCTGTCCTGTAAACCC
CTTTGCCTCATGTTTGTGGATGAGTCAGACCACGAGACACTCACAGCCGT
CCTGGGCCCCGTAGTTGCAGAGCGTAATGCGATGAAAGAGAGTAGACTCA
TTCTTTCCATGGGTGGCCTGCCTCGCTCCTTCCGCTTCCACTTCAGAGGC
ACAGGTTATGATGAGAAGATGGTGCGTGAGATGGAGGGCCTAGAGGCCTC
AGGCTCCACCTATATCTGCACTCTGTGTGACTCCACTCGGGCAGAGGCTT
CGCACAACATGGTGCTACACTCCGTCACCCGTAGTCACGAAGAGAACCTA
GAGCGTTACGAAATATGGAGAACCAATCCCTTTTCTGAGTCTGTAGAGGA
GCTGCGGGACCGAGTCAAAGGAGTCTCTGCCAAGCCCTTTATGGAGATCC
AGCCAACAATGGATGCTTTGCACTGTGACATTGGTAATGCCACTGAGTTC
TACAAAATCTTCCAAGATGAGATTGGGGAGGTGTACCAAAAGGC---CAA
C---CCCACACGGGAGGAACGGCGCAGCTGGAGGGCAGCCCTAGATAAAC
AGCTGAGGAAGAAAATGAAGCTTAAACCCGTAATGAGGATGAATGGGAAC
TATGCCCGGAAGCTAATGACCATGGAGACTATGGAGGTGGTGTGTGAGCT
GGTGCCATCAGAAGAGAGGAGGGAGGCCCTGAGAGAGCTCATGAGGCTCT
ACCTTCAGATGAAGCCTGTTTGGCGTGCCACCTGCCCAGCCAAGGAATGC
CCTGACCAGCTGTGCCGCTACAGCTTCAACTCCCAAAGCTTTGCAGATCT
CCTCTCCTCCACTTTCAAATATCGCTACAATGGAAAAATAACCAATTACT
TGCACAAGACCCTGGCTCAAGTCCCTGAAATCATTGAAAGNNNNNNNNNNNNNNNNNNNNNNNNNNNNNN
NNNNNNNNNNNNNNNNNNNNNNTCATACACTA
TTGATATGGGCCCCACTGGACCCCAGTGGAAGGAGAGCCCACAGCCTTTC
TGCTGCTCCATTGAAGACCCCACAAAACAGACAAAGTTCAAGGGTATCAA
GACCTACATTTCCTATCGGGTCACACCAAGCCACACAGGGCGTCCCGTCT
ATAGGCGCTACAAACACTTTGACTGGCTGTACAACCGCTTGCTGCACAAG
TTCACTGTGATCTCGGTACCCCACCTGCCTGAGAAGCAGGCTACAGGGCG
ATTTGAGGAAGATTTCATTGAGAAGCGTAAAAGACGACTGATTCTGTGGA
TGAACCACATGACCAGTCACCCAGTCCTCTCCCAGTATGAAGGCTTTGAG
CATTTTCTGATGTGTGCTGATGACAAACAATGGAAACTGGGTAAGAGACG
AGCAGAGAAGGATGAAATGGTGGGTGCCCATTTCATGCTGACCCTCCAGA
TTCCTAATGAGCACCNNNNNNNNNNNNNNNNNNNNNNNNNNNNNNNNNNNNNNNNNNNNNNNNNNNNNNN
NNNNNNNNNNNNNNNNNNNNNNNNNNNNNNNNNNNNNNNNNNNNNNNNNNNNNNNNNNNNNNNNNNNNNN
NNNNNNNNNNNNNNNNNNNNNNNNNNNNNNNNNNNNNNNNNNNNNNNNNNNNNNNNNNNNNNNNNNNNNN
NNNNNNNNNNNNNNNNNNNNNNNNNNNNNNNNNNNCCTCTCGCCACGTTCCTCAAACTGACCTCTCTGGG
CTTCATCATCGGAATCGGTGTGGTCGGAAACCTCCTGATCTCCATCCTGC

TGGTTAAAGACAAGAGCCTGCACCGCGCGCCCTACTACTTCCTGCTGGAC
CTGTGCGCCTCTGACATCCTGCGTTCTGCTATCTGCTTCCCCTTTGTCTT
CACCTCTGTCAAGAATGGATCTGTCTGGACCTACGGCACGCTCACCTGCA
AAGTGATCGCCTTCCTGGGCGTGCTCTCCTGTTTCCACACAGCGTTCATG
CTATTTTGTGTCAGCGTGACCCGCTATCTGGCCATCGCACATCACCGTTT
CTACACTAAGAGGCTGACCTTCTGGACCTGTCTGGCTGTCATCTGCATGG
TGTGGACGTTATCAGTGGCMATGGCTTTCCCACCGGTGCTAGACGTAGGG
ACGTACTCTTTCATCCAGGAGGAGGACCAGTGCACATTCCAGCACCATTC
CTTCAGGGCGAACGACTCCCTGGGCTTCATGCTCCTGCTGGCACTCATCC
TCCTGGCCACACAGCTGGTTTACCTCAAGCTCATCTTCTTCGTCCACGAC
CGTCGGAAGATGAAGCCTGTCCAGTTCGTGCCTGCTGTCAGCCAGAACTG
GACCTTCCACGGGCCAGGGGCCAGCGGGCAGGCGGCGGCCAACTGGCTGG
CTGGATTTGGTAGAGGTCCCACACCTCCTACTTTACTGGGCATCCGGCAG
AACAGCAATGCAGCAGGCCGCAGGCGTCTACTGGTGCTGGATGAATTCAA
AACAGAGAAGAGGATTAGCAGGATGTTCTACATCATGACGTTTTTCTTCC
TGGCACTGTGGGGGCCGTATCTGGTAGCCTGCTACTGGAGGGTGTTTGCA
AGGGGCCCTGTAGTCCCTGTGGGCTACCTGACAGCAGCCGTGTGGATGAG
CTTTGCCCAGGCAGGGGTCAATCCTTTCATCTGCATCTTCTCCAACAGGG
AGGCCAAATCTCGCTTTCACCCTGGCGTGGGGACTGCGCCTGGCACGGAG
C---GCAGCGTCCCACTCGGCAACAGCTTGCTATCCCCACAGCAAACTGA
GGAGCCCACTGTTGCCACCCCCCCGCAGCGATGGTTTGTCACCC---CTG
CCAACAACCGACTGGACTTTGCTGCCTCGGCATACGACGCGGCT------
---GATTTCGCCGGTAACGCGGCCACCTTGCTGTCCTACGCAGCGGCCGG
AGTGAAGGCTC------TTCCCCTGCCGGCCGCAGGCTGCTCCAACCGAC
CTCTTGGCTATTACGCTGACCCGTCGG---GCTGG---GGAGGGCGCACG
CCGCCGCAGTACTGTGGTGTAAATAGTAAATCCAGCTCGGTCTTTTCCTG
CTGGCCCGCTAACTCTATCGGGGGCAGAGCAGGCG---CC---CACTACC
TGG---------CCGAGGA---GGGA---GACTC---CGTCCCCACGGAG
AGGTCACCG---AT---CAGCGGCTCCGAGGAG---ACCAAACCCAAGGA
CAT------ATCAGA---GTCGAGCTGGATAGAG---ACACCTTCCTCCA
TTAAGTCCATCGATTCAAGCGATTCTGGGATCTTTG---AACAGGCCAAA
AGGAGAAGAATCTCACCTTCTGCCACGCCG---------GTTTCAGAGGC
AGTGTCCCCGTTANNNNNNNNNNCATCACTCAACAGGCGAAGTCACAGAGAGAGAAGTGG
CTTTGGGGATAAATCCTTTCGCGGACGGGATGGGCGCCTTCAAAATAAAC
CACAGCTCCCATGATATTGGCTCCGG---ACAAACGGCGTTTTCCTCTCA
GGCG---CCCGGCTAC---GCAGCAGCAGCCCTGGGA---CACCATCA--
----CCACCCGACCCACGTTGGCTCT---TACTCCACGGCGGCTTTCAAC
TCCACCAGGGACTTTCTCTTCAGAAATCGGGGTTTTGGAGACGCCACCGG
------------GGCGCAGCACAGTTTGTTCGCCTC---------CGGAA
GTTT---C------GCAGGGCCACATGGACACTCAGATGCAACGGGGCAC
CTGCTCTTCCCAGGGCTCCACGAG---CAAGCAGCGAGCCATGCGTCTTC
CAACGTGGTCAACAGCCAGATGCGACTGGGCTTCTCGGGGGACATGTACG
GTCGGGCCGATCAGTACGGCCACGTTACAAGCCCGAGGT---CCGACCAC
TATGCTTCGACCCAGTTGCACGGTTATGGCCCCATGAACATGAATATGGC
CGCA---CACCACGGAGCAGGGGCCTTCTTTCGATACATGAGGCAGCCGA
TCAAACAAGAGCTTATCTGCAAGTGGATCGAGCCGGAGCAGCTGACAAAT
CCCAAAAAGTCTTGCAACAAAACTTTTAGCACGATGCATGAGCTTGTGAC
CCACCTGACGGTGGAGCATGTGGGGGGACCAGAGCAGTCCAACCACATCT
GCTTCTGGGAAGAGTGCCCCAGAGAAGGAAAACCATTCAAAGCCAAATAC
AAACTTGTAAATCATATCAGAGTACACACCGGAGAAAAACCCTTTCCGTG
TCCGTTCCCAGGCTGTGGCAAA
>Centropomus undecimalis
AGCCTACTCATTCGAGCGGAACTTAGTCAACCTGGCGCCCTACTAGGAGA

CGACCAAATTTACAATGTTATTGTCACGGCACACGCCTTCGTAATAATTT
TCTTCATAGTGATGCCAATCATAATTGGAGGCTTCGGAAACTGACTCATC
CCTCTAATAATTGGAGCTCCAGACATGGCATTTCCCCGAATAAATAATAT
AAGCTTCTGACTTCTCCCTCCTTCCTTCCTGCTACTTCTAGCCTCCTCTG
GAGTAGAAGCCGGTGCCGGAACAGGATGAACAGTTTACCCCCCTCTAGCT
GGTAATCTCGCCCACGCCGGAGCATCGGTAGACCTCACCATCTTTTCACT
ACACTTAGCCGGAATCTCTTCAATTCTTGGAGCTATCAACTTTATCACAA
CCATTATCAATATAAAACCAGCCTCAACCTCTATATACCAAATTCCCCTA
TTCGTTTGATCTGTCCTAATTACGGCCGTCCTTCTCCTCCTTTCCCTCCC
CGTCCTAGCCGCCGGGATCACAATGCTCCTAACCGACCGAAACTTAAACA
CCTCATTCTTCGACCCAGCCGGAGGTGGAGACCCCATTCTTTACCAACAC
CTA---

------------TTCCTCGAGAGAAACCTCCACCCGTCTAACTGCCTTGG
CATGTTGTTGCTGTCTGACGCCCACCAGTGTACCAAGCTGTCGGAGCTCT
CCTGGGGCATGTGCCTCAGCAACTTCCCTGCTATTTGCAAGACAGAGGAC
TTCCTCCAACTGCCCAAAGATATGGTGGTGCAGCTTTTGTCACACGAGGA
GCTAGAGACAGAAGACGAGAGACTGGTTTATGAAGCTGCCCTGAACTGGA
TCAACTATGACCTGGAAAGGAGGCACTGCCACCTTCCAGAGCTTCTGAGA
ACGGTCCGCCTCGCCCTGCTGCCTGCCATCTTTCTGATGGAGAATGTCTC
GACGGAAGAGCTGATCAACGCCCAGGCCAAGAGCAAGGAGCTGGTGGATG
AAGCCATCCGCTGTAAGCTGAAAATCCTGCAGAATGATGGTGTCGTTAAC
AGCCTGTGTGCTCGACCAAGGAAAACCAGCCACGCCCTCTTTCTTCTGGG
AGGGCAGACTTTCATGTGTGACAAGTTGTACCTGGTGGACCAGAAGGCCA
AAGAAATCATCCCCAAGGCTGACATCCCCAGCCCCAGGAAGGAGTTCAGC
GCCTGCGCCATCGGCTGTAAGGTGTACATCACTGGTGGGA--GAGGCTC-
AGAGAACGGCGTGTCCAAAGACGTATGGGTCTACGACACCGTCCACGAGG
AATGGTCCAAAGCGGCTCCAATGCTCATTGCCAGGTTTGGCCACGGCTCT
GCGGAGCTGAAACATTGCCTCTATGTGGTCGGAGGTCACACTGCAGCAAC
CGGCTGCCTCCCGGCTTCTCCGTCAGGATGATTACATTGTTGTGTTCAGT
CGTTCCACAACGAGGCTGATACTTAATGAAGCAGAGCTAATCATGGCACT
GGCCCAGGAGTTCCAGATGAGAGTGGTAACTGTATCTCTGGAGGAACAGT
CTTTCCCCAGTATCATCCAGGTGATCAGCCGTGCTTCCATGTTAGTCAGC
ATGCATGGAGCTCAGCTTATCACCTCACTCTTCCTCCCCAGAGGAGCTGC
AGTGGTGGAACTGTTCCCCTTTGCTGTGAACCCTGAGCAGTACACCCCAT
ATAAAACCCTTGCCTCCCTTCCAGGCATGGACCTTCATTACATCTCCTGG
AGGAACACTAAGGAGGAAAACACCATCACCCACCCAGACAGACCCTGGGA
ACAAGGGGGCATCACTCACTTGGAGAAGGAGGAACAGGAGCGAATACTGG
CCAGTAAGGATGTCCCCAGGCACCTCTGCTGCCGCAACCCTGAGTGGCTC
TTCCGGATTTACCAGGATACTTTGGTGGACATCCCTTCCTTCCTGGAAAT
CCTCAA---AGAGGGCATGAAG---TCCAAGCCCAGTGTGAAAAA---GT
CAAAGCTGGCCGGCATGGTCCACCCGGGCCGCGTCAGAGAACCCCAGTGT
CAGACCTCAGTACAAACCACTAATGAGGCTAAACTCACAGTCTCCTGGCA
GATCCCATGGAATTTGAAATACTTGAAGGTAAGAGAGGTGAAGTATGAGG
TGTGGATCCAGAAAAAAGACACCAGCAAGGGGACACTGGAGGATCAAATC
ATCCAGGCAAACCCTGCACTGGAGGCCTTTGGCAACGCCAAAACATTAAG
AAACGACAACTCGTCACGTTTTGGAAAATTCATTCGAATTCACTTCGGGA
CAAGTGGCAAACTGTCATCTGCTGACGTCGAGACGTACCTGCTAGAGAAG
TCACGCGTCACCTTTCAACTCAAGGCTGAGAGGAACTACCACATCTTCTA
CCAGATTCTGTCCAATCAGAAGCCAGAGTTGCTGGACATGCTGCTGATCA
CCAACAACCCGTATGACTACTCCTACATCTCCCAAGGAGAGGTAACAGTC

GCCTCCATCAACGACTCAGAAGAGCTGATGGCCACCGACAGCGCCTTCGA
TGTGCTCGGCTTCACTCCAGAGGAGAAGATGGGCGTCTATAAGCTGACCG
GTGCCATCATGCACTACGGCAACATGAAGTTCAAACAGAAACAGCGCGAG
GAGCAGGCTGAACCTGACGGGACGGAGGCTGCTGATAAATCGGCATACCT
AATGGGGCTGAACTCCGCTGACCTCATCAAAGGCCTGTGCCACCCCAGAG
TCAAGGTAGGAAATGAATACGTCACCAAAGGCCAAAGTGTGGACCAAGTC
TACTATCCCAATAAGGAGGCCTTCAAGTGTGAGGAGTGTGGGAAGCACTA
CAACACCAAGCTGGGATATAAGCGCCATGTGGCCATGCACTCTGCCACGG
CAGGGGATCTCACCTGTAAAGTGTGCATGCAGAGCTACGAGAGTACACCA
GTTCTCCTGGAGCACCTCAAAAGCCACTCTGGGAAGTCTTCGGGTGGCGC
CAAGGAGAAAAAACACCCGTGTGACCACTGTGACCGTCGTTTCTACACGC
GGAAGGATGTCAGACGGCACATGGTGGTCCACACGGGCCGAAAGGACTTC
CTGTGCCAGTACTGTGCCCAGCGCTTCGGCAGGAAGGACCATCTGACACG
CCACGTGAAGAAGAGCCACTCGCAGGAGCTGCTGAAGATCAAAACGGAGC
CTCCGGATATGTTAGGTCTTTTAGCTTCTGGATCACCACCTTGCTCTGTG
AAGGAGGAGCTCAGCCCCATGATGTGTGGCATGGGTCCCAACAAAGACCC
CATGATGGGCAAACCATTCCCCAGTGGGGCTCCTTTTCCAATGGGCATGT
ACAACCCCCACCAT------CTCCAGGCGATGTCTAATACTGGGGTGGGT
CACCCA------CACCCGTCCCTAATGCCCAGTTCCATGTCTGCAGCTTT
GGGCATGGGCTGTCACATGGAANNNNNNNNNNNNNNNNNNNNNNNNNNNNNNNNNGTTTACAGATCAGT
GATGGATCGAATAT
CGTCAACCTGTTGGCTAGTAACTCTCCAAGTGTTTCGTATGCCCTGACCC
AGCAGAAATACTTCAGTAACTACAGTCCTGTGATTGGGTTTTACATTTAC
GAACCCATCGAGTACTGGAACTCCACGGTGCAAGAGCACCTGAAGACTCT
GAGTCATGGCTTCAACAAGATCTCCTGGATGGACAACTTTTTCCACTACC
TGCGGGTGGTGAACGTGAGTGCGTCAACCAAGAGCGACTTCATCAGCATC
CTAAAAGGCTCCTTCCTGCGCAGCCCTGAGTACCAGCACTTCACTGAGGA
CATCATATTCTCCAAGA---ACCGCGAGACTG------ATGAGTACGACA
TCATTGCCTCACGGATGTACTTGGTGGCACGGACGACTGAGAAGAAGCGC
GAGGAGGTTGTGGAGCTACTGGAAAAGCTTCGTCCATTGATGCTAATAAA
CAGCATCAAGTTCATTGCCTTCAACCCCACATTTGTGTTCATGGACCGCT
ACAGCTCCTCCGTCATCTCGCCCATCCTGACCTCAGGCTTCAGCGTACTC
ACTATCCTCATCCTCACTTTCTTCCTGGTCATCAACCCCTTGGNNNNNNNNNNNNNNNNNNNNNNNNNN
NNNNNNNNNNNNNNNNNNNNNNNNNNNNNNNNGGCTTTC
ACCAGTTTGAATGGCAGCCGGCTCTCAAGAATGTGTCCACATCTTGCAAT
GTTGGCATTATTAATGGGCTCTCTGGATGGACTTCCTCGCTGGATGACTC
CCCGGCTGACACCATCACTCGGCGGTTTCGCTATGATGTGGCACTGGTGT
CAGCCTTAAAGGATCTGGAGGAGGACATCATGGAGGGGCTGAGAGAGAGT
GGGATGGAAGACAGCGCTTGCACCTCAGGCTTTAGTGTCATGATCAAGGA
GTGTTGTGATGGCATGGGCGATGTCAGCGAGAAGCACGGCGGAGGACCAG
CTGTTCCTGAGAAGGCTGTGCGTTTCTCCTTCACTATTATGTCTGTCTCG
GTCCTGGCAGAAGAGGAGGAG-----------------------------
-GAGGAGGTTACCGTCTTCACCGAGCCAAAGCCAAACTCAGAACTGTCCT
GTAAGCCCCTTTGCCTGATGTTTGTGGATGAGTCAGACCATGAGACGCTC
ACAGCTGTCCTGGGGCCTATAATTGCGGAGCGTAACGCAATGAAAGAAAG
CAGGCTCATCCTGTCCATTGGCGGCCTGCCTCGCTCCTTCCGCTTCCACT
TCAGAGGCACGGGATACGATGAGAAGATGGTGCGAGACATGGAGGGCCTG
GAGGCCTCGGGGTCCACCTATATCTGCACTCTGTGCGACTCCAGCCGGGC
AGAGGCCTCTCAAAATATGGTGCTTCACTCCATCACTCGCTGTCATGAAG
AGAACCTAGAACGTTACGAAATATGGAGAACCAACCCCTTCTCTGAGTCT
GTAGACGAGCTGCGAGACAGAGTGAAAGGGGTCTCTGCCAAGCCCTTCAT
GGAGACCCAGCCCACACTGGATGCATTACACTGTGACATTGGCAATGCCA
CTGAGTTCTACAAAATATTCCAGGATGAGATCGGGGAGGTGTACCAAAAG

GT---CAAC---CCGACCCGGGAGGAGCGGCGCAGCTGGAGGGCAGCCCT
AGATAAACAGCTGAGGAAGAAGATGAAGCTTAAGCCCGTAATGAGGATGA
ATGGGAACTACGCCCGCAGGCTAATGACCCTGGAGGCTGTGGAGGTGGTG
TGTGAGCTGGTGCCCTCAGAGGAGAGGAGGGAGGCCCTGAGGGAGCTTAT
GAGGTTCTACCTCCAGATGCGGCCTGTGTGGCGCGCCACCTGCCCGGCCA
AGGAATGCCCCGACCAGCTGTGCCGCTACAGCTTTAACTCCCAGCACTTT
GCCGACCTCCTCTCCTCTACCTTCAAATATAGGTACAATGGAAAGATAAC
CAATTACCTTCACAAGACCCTGGCCCATGTGCCTGAAATCATAGAGAGGG
ATGGATCCATAGGAGCCTGGGCCAGCGAGGGGAACGAGTCGGCAAACAAA
TCGTACACCATCGAGATGGGTCCCCTGGGGCCCCGGTGGAAGGAGAACCC
ACAGCCTTTCTGCTGCTCCATTGAAGACCCCACAAAACAGACGAAGTTCA
AGGGCATCAAGACCTACATTTCATACCGGGTCACGCCGAGTCACACAGGG
CGTCCCGTCTACAGGCGTTACAAACACTTTGACTGGCTTTACAACCGCTT
GCTGCACAAGTTCACTGTGATCTCCGTGCCTCACCTGCCTGAGAAGCAGG
CCACGGGACGATTTGAAGAAGACTTCATCGAGAAGCGCAAGAGACGACTG
ATACTGTGGATGAACCACATGACCAGTCACCCAGTCCTCTCCCAGTATGA
AGGCTTTGAGCACTTTCTGATGTGTGCTGACGACAAGCAGTGGAAACTGG
GGAAGAGGCGGGCAGAGAAGGACGAGATGGTGGGCGCCCATTTCATGCTG
ACCCTCCAGATCCCTAACGAGCACCAGGACCTTCAGGATGTGGAGGAGCG
GGTCGACTCCTTCAAGGCCTTTGCTAAAAAAATGGACGACAGCGTGATGC
AGCTCACACACGTTGCCTCGGAGCTGGTGCGCAAACACCTGGGTGGGTTC
AGGAAGGAGTTCCAGCGGCTGGGGAATGCCTTCCAGTCCATCAGCCAGGC
GTTCATGCTGGACCCTCCCCACAGCTCAGACGCCCTCAACAACGCCATCT
CCCATNNNNNNNNNNNNNTCCTCAAACTGACCTCTCTGGGTTTCATCATCGGAGTCGGTGTGG
TCGGAAACCTCCTGATCTCCATCCTGCTGGTCAAAGACAAGAGCCTGCAC
CGAGCGCCCTACTATTTCCTGCTGGACCTGTGCGCCTCCGACATCCTGCG
CTCCGCCATCTGCTTCCCATTTGTCTTCACCTCGGTCAAGAATGGATCTG
CCTGGACCTACGGCACGCTGACCTGCAAAGTGATCGCCTTCCTGGGTGTG
CTCTCCTGTTTCCACACGGCGTTCATGCTCTTCTGCGTCAGCGTCACCCG
CTACCTGGCCATCGCGCATCACCGTTTCTACACCAAGAGGCTGACCTTCT
GGACCTGCCTGGCCGTCATCTGCATGGTGTGGACGTTGTCGGTGGCTATG
GCGTTCCCGCCGGTGCTAGACGTGGGGACGTACTCTTTTATCCGGGAGGA
GGACCAGTGCACGTTCCAGCACCGCTCCTTCAGGGCGAACGACTCGCTGG
GCTTCATGCTCCTGCTGGCCCTCATCCTCCTGGCCACACAGCTGGTTTAC
CTCAAGCTCATCTTTTTTGTCCACGACCGTCGAAAGATGAAGCCCGTCCA
GTTCGTGCCTGCTGTCAGCCAGAACTGGACCTTCCACGGGCCTGGCGCCA
GTGGGCAGGCGGCGGCCAACTGGCTGGCCGGATTTGGTCGAGGCCCCACC
CCGCCTACTTTGCTGGGCATCCGGCAGAACAGCAACGCAGCGGGCCGCAG
GCGTCTACTGGTATTGGATGAGTTCAAAACAGAGAAGAGGATTAGTAGGA
TGTTCTACATCATGACGTTTTTCTTYCTGGCACTGTGGGGGCCCTATCTG
GTCGCCTGCTACTGGCGGGTGTTTGCAAGGGGCCCAGTAGTCCCTGGGGG
GTACCTGACGGCGGCCGTGTGGATGAGCTTTGCCCAGGCCGGGGTCAATC
CTTTCATCTGCATCTTCTNNNNNNNNNNNGCCAAATCTCGCTTTCACCCTGGCGTGGGGAC
TGGTCCTGGCACGGAGC---GCAGCGTCCCACTCGGCAACAGCTTGCTAT
CCCCGCAGCAAACCGAGGAGCCCACTGTTGCCACCCCCCCGCAGCGATGG
TTTGTCACCC---CTGCCAACAACCGACTGGACTTTGCTGCCTCGGCATA
CGACGCCGCT---------GATTTCGCCGGTAACGCGGCCACCTTGCTGT
CCTACGCAGCGGCCGGAGTGAAGGCTC------TTCCCCTGCCGACTGCA
GGCTGCTCCAACCGGCCTCTTGGCTATTACGCAGACCCGTCCG---GCTG
G---GGAGGACGCACGCCGCCGCAGTACTGTGGCGTAAATAGCAAATCCA
GCTCGGTCTTTTCCTGCTGGCCCGCCAACTCTCTCGGCGGCAGAGCGGGC
A---CC---AACTACCTGG---------CCGAGGA---GGGA---GACTC
---CATCCCGACGGAGAGATCACCG---AT---CGGGGGCTCGGAGGAGA

CCACCAAACCCAAAGACATGAC---ATCTGA---GTCGAGCTGGATAGAG
---ACGCCGTCCTCCATTAAGTCCATTGATTCGAGCGATTCTGGTATCTT
TG---AACAGGCCAAAAGGAGGAGAATCTCACCTTCTGCCACGCCG----
-----GTTTCAGAGACAGTGTCCCCGTTAAAATCTGAGCATCACTCAACA
GGCGAAGTCACAGAGAGAGAAGTGGCGTTGGGGATAAATCCATTCGCGGA
TGGGATGGGAGCCTTCAAAATAAACCACAGCTCCCACGATATTGGCTCCG
G---ACAGACGGCGTTTTCCTCCCAGGCA---CCCGGCTAC---GCAGCA
GCCGCCCTGGGA---CACCATCA------CCACCCGACCCACGTTGGCTC
T---TACTCCACGGCGGCTTTCAACTCCACCAGGGACTTTCTCTTCAGAA
ATCGGGGTTTCGGGGACGCCACCGG------------CGCGCAGCACAGT
TTGTTCGCCTC---------TGGAAGTTT---C------GCAGGGCCACA
TGGACACTCAGATGCAGCGGGGCACCTGCTCTTCCCAGGGCTCCACGAG-
--CAAGCAGCGAGCCATGCCTCTTCCAACGTGGTCAACAGCCAGATGCGA
CTGGGCTTCTCGGGGGACATGTACGGACGTGCCGATCAGTATGGCCACGT
TACGAGCCCACGGT---CCGACCACTACGCTTCGACCCAGCTGCACGGCT
ATGGCCCCATGAACATGAATATGGCCGCA---CACCACGGAGCAGGGGCC
TTCTTTCGATACATGAGGCAACCGATCAAACAAGAGCTCATCTGCAAGTG
GATCGAGCCGGAACAGCTGACGAATCCCAAAAAGTCGTGCAATAAAACTT
TTAGCACGATGCACGAGCTCGTGACCCATCTGACGGTGGAGCATGTGGGG
GGACCGGAGCAGACCAACCACATCTGCTTCTGGGAGGACTGCTCCAGAGA
AGGGAAGCCATTCAAAGCCAAATACAAACTTGTAAATCATATCAGAGTAC
ACACCGGAGAAAAGCCCTTTCCGTGTCCGTTCCCCGGCTGTGGCAAA
>Cephalopholis argus
AGCCTATTAATTCGAGCTGAATTAAGCCAGCCAGGTGCTCTTCTGGGCGA
TGATCAGATTTATAATGTTATTGTCACGGCACACGCTTTCGTAATAATCT
TCTTCATAGTAATGCCAATTATAATTGGCGGTTTCGGAAACTGACTTATC
CCCCTAATAATTGGTGCTCCTGACATAGCATTCCCCCGAATAAATAACAT
GAGCTTCTGACTTCTTCCCCCATCTTTCCTACTTCTGCTGGCCTCCTCTG
GAGTAGAAGCAGGTGCTGGAACTGGCTGAACAGTTTACCCCCCTCTAGCT
GGCAACTTAGCCCATGCAGGCGCATCTGTTGACCTAACCATTTTCTCCCT
GCATTTAGCAGGTATTTCATCAATCCTAGGGGCGATTAATTTTATCACAA
CCATTATTAACATGAAACCTCCAGCTATTTCCCAATATCAAACGCCCCTG
TTTGTATGAGCTGTTCTAATTACAGCTGTTCTTCTTCTCCTCTCTCTTCC
TGTCCTTGCTGCCGGCATTACAATACTTCTAACAGATCGAAATCTAAATA
CCACCTTCTTTGACCCAGCTGGCGGAGGAGACCCAATTCTTTATCAGCAC

------------TTCCTGGAGAGAAACCTTCACCCATCTAACTGCCTTGG
CATGCTGTTGCTGTCTGACGCTCACCAGTGCACCAAGCTGTCAGAGCTCT
CCTGGGGTATGTGCCTCAGCAACTTTCCAGCTATTTGCAAGACAGAGGAC
TTCCTCCAACTGCCCAAAGATATGGTGGTGCAGCTTTTGTCACACGAGGA
GCTAGAGACAGAAGATGAGAGACTGGTTTATGAAGCTGCCCTTAACTGGA
TCAACTATGATCTGGAAAAGAGGCACTGCCACCTTCCAGAGCTCCTGAGA
ACGGTCCGTCTGGCCCTGCTGCCTGCCATCTTTCTCATGGAGAACGTTTC
TACCGAAGAGCTGATCAACGCCCAGCCCAAGAGCAAGGAGCTGGTGGATG
AAGCTATCCGTTGTAAGCTGAAGATCCTGCAGAACGATGGCGTCGTTAAC
AGCCCATGTGCTAGACCAAGAAAGACCAGCCATGCCCTCTTTCTTCTGGG
AGGGCAGACGTTCATGTGTGACAAGTTGTACCTGGTGGACCAGAAAGCCA
AAGAGATCATCCCCAAGGCTGACATTCCCAGCCCCAGGAAGGAGTTCAGC
GCCTGTGCCATCGGCTGCAAGGTGTACATCACTGGTGGGA--GAGGCTC-
AGAGAATGGTGTGTCCAAAGATGTGTGGGTCTATGACACCGTCCAYGAGG

AATGGTCGAAGGCGGCACCCATGCTCATTGCCAGGTTTGGCCATGGCTCT
GCGGAGCTGAAACACTGCCTCTACGTGGTAGGAGGTCACACAGCAGCAAC
TGGCTGTCTGCCAGCCTCTCCGTCCGGATGAATACATTGTTGTGTTCAGT
CGTTCAACAACAAGGCTGATACTGAATGAAGCAGAGCTAATCATGGTGCT
GGCCCAGGAGTTCCAGATGAGAGTGGTGACAGTATCCCTGGAGGAACAGT
CTTTCCCCAGTATTGTCCAGGTGATCAGTCGTGCTTCTGTGTTAGTCAGT
ATGCACGGAGCTCAGCTGATCACCTCACTCTTCCTCCCCAGAGGAGCTGT
TGTGGTGGAGCTATTCCCCTTTGCTGTGAACCCAGAGCAGTACACCCCAT
ATAAAACCCTTGCCACCCTTCCAGGCATGGACCTTCACTATATCCCCTGG
AGGAACTCTAAGGAGGAGAACACCATCACCCACCCAGACAGACCCTGGGA
ACAAGGGGGCATTGCTCACCTGGAGAAGGAGGAGCAAGAGCGAATACTAG
CAAGCAAAGATGTCCCCAGGCACCTGTGCTGCCGCAACCCAGAGTGGCTC
TTCCGGATCTACCAGGACACTTTGGTGGACATCCCTTCCTTCCTGGAAGT
CCTTAA---AGAGGGCATGAAG---ATGAAGCCCAGCATAAAGAA---AT
CAAAGCTGGCTAGCACAGTCCACCCGGGCCGGGTCAGAGAACCCCAGTGT
CAGACCTCAGTACAAACCACTAACGAGGCCAAGCTCACTGTCTCCTGGCA
GATCCCATGGAATCTGAAATACCTGAAGGTGAGAGAGGTGAAGTACGAGG
TGTGGATCCAGAAAAAAGACACCAGCAAGGGCACGCTGGAGGATCAAATC
ATCCAGGCGAACCCCGCGCTGGAGGCCTTCGGCAACGCCAAAACGTTGAG
AAACGACAACTCGTCTCGTTTTGGAAAATTCATCCGAATTCACTTTGGGA
CGAGCGGCAAGCTGTCGTCTGCTGACATCGAGACGTACCTGCTGGAGAAG
TCACGTGTCACCTTTCAGCTCAAGGCTGAGAGGAACTACCACATCTTCTA
CCAGATCCTGTCCAATCAGAAGCCAGAGCTGCTGGACATGCTGCTGATCA
CCAACAACCCGTATGACTACTCCTACATCTCCCAAGGAGAGGTGACAGTT
GCCTCCATCAATGACTCGGAGGAGCTGATGGCCACCGACAGCGCCTTCGA
TGTGCTCGGCTTCACTGCAGACGAGAAGATGGGTGTCTACAAACTGACCG
GCGCCATCATGCACTACGGCAACATGAAGTTCAAACAGAAGCAGCGTGAG
GAGCAGGCTGAGCCGGACGGGACGGAGGCTGCTGATAAATCGGCGTACCT
AATGGGGCTGAACTCTGCTGACCTCATCAAAGGGCTGTGCCATCCCAGAG
TCAAGGTAGGAAATGAATACGTCACCAAAGGTCAAAGTGTGGACCAGGTC
TACTACCCCAACAAGGAGGCCTTCAAGTGTGAGGAGTGTGGGAAGCACTA
CAACACAAAGCTTGGATATAAGCGCCATGTGGCCATGCACTCCGCCACGG
CAGGAGATCTCACCTGTAAAGTGTGCATGCAGAGCTACGAGAGCACGCCT
GTGCTCTTGGAGCACCTCAAGAGCCACTCCGGGAAGTCTTCGGGTGGCGC
CAAGGAGAAAAAACATCCGTGCGACCACTGTGACCGTCGTTTCTACACGC
GGAAGGATGTGAGACGGCACATGGTGGTCCACACGGGCCGAAAGGACTTC
CTGTGCCAGTATTGTGCCCAGCGCTTTGGCAGGAAGGATCATCTGACACG
CCACGTGAAGAAGAGCCACTCGCAGGAGCTACTGAAGATTAAGACGGAGC
CTCCTGATATGTTAGGTCTTTTAGCATCTGGGTCACCCCCTTGCTCTGTG
AAGGAGGAGCTCAGCCCCATGATGTGCGGCATGGGTCCCAATAAAGACCC
CATGATGGGCAAACCGTTTCCCAGTGGGGCCCCTTTTCCGATGGGCATGT
ACAACCCCCACCAT------CTCCAGGCCATGTCTAATTCTGGGGTGGGT
CACCCA------CACCCGTCCCTGATGCCCAGTCCCTTGTCTGCAGCTAT
GGGCATGGGCTGTCACATGGAATATCTGATCTATGCCTCTTTCTCATTCA
TGGGATGTTTACAAATCAGTGATGGATCAAATATTGTGAACCTGCTGGCT
AGTAACTCTCCAAGTGTTTCATATGCTCTGACCCAGCAGAAATACTTCAG
TAACTACAGTCCTGTAATCGGATTTTACATTTATGAGCCCATCGAGTACT
GGAACTCCACAGTGCAAGAGCACCTGAAGACTCTGAGTCATGGCTTCAAT
AAGATCTCTTGGATGGACAACTTTTTCCACTACCTGCGGGTAGTGAATGT
GAGTGCATCAACCAAGAGCGACTTCATCACCATCCTCAAGGGCTCCTTTC
TGCGCAGCCCAGAGTACCAGCATTTCACTGAGGACATCATATTCTCCAAG
A---ACCGTGAGACTG------ATGAGTACGACATCATCGCCTCACGGAT
GTACTTGGTGGCACGGACAACAGAGAAGAAGCGCGAAGAGGTGGTGGAGC

TTCTGGAAAAGCTTCGTCCATTGATGCTGATTAACAGCATCAAGTTCATT
GCATTCAATCCTACGTTTGTGTTCATGGACCGCTACAGCTCCTCTGTCAT
CTCACCCATCCTGACCTCAGGCTTCAGCGTACTCACAATCCTCATCCTCA
CTTTCTTCCTAGTCATCAACCCCTTGGGGAACTTCTGGCTCATCCTTACG
GTAACATCTGTGGAGCTGGGCGTCTTGGGTTTGATGGGCTTTCACCAGTT
TGAATGGCAGCCGGCTCTCAAAAATGTGTCCACATCGTGCAGTGTTGGCA
TTATTAATGGGCTCTCTGGATGGGCTTCCTCGGTGGATGACATCCCAGCT
GACACCATCACTCGGCGGTTTCGCTATGATGTGGCACTGGTGTCAGCATT
AAAGGATCTGGAGGAGGACATCATGGAGGGGCTGAAAGAGTGTGGGATGG
AAGACAGTACTTGCACCTCAGGCTTCAGTGTCATGATCAAGGAATCCTGT
GATGGCATGGGCGATGTCAGCGAGAAGCACGGGGGAGGACCAGTTGTTCC
TGAGAAGGCTGTGCGTTTCTCTTTCACTATTATGTCTGTCTCTGTCCTGG
CAGACGAGCAGGAG------------------------------AAAGAG
GTTACCATCTTCACTGAGCCAAAGCCAAACTCAGAAATGTCCTGTAAGCC
CCTTTGCCTGATGTTTGTGGATGAGTCAGACCACGAGACACTCACAGGTG
TCCTGGGGCCTATAGTTGCGGAGCGTAATGCAATGAAAGAGAGCAGACTC
ATCCTATCCCTGGGTGGACTCCCTCGCGCCTTCCGCTTTCACTTCAGAGG
CACGGGATACGATGAGAAGATGGTGCGTGAGATGGAGGGCCTCGAGGCCT
CAGGATCCACCTATGTCTGCACTCTTTGTGACTCCACTCGGGCAGAGGCC
TCCAAAAACATGGTGCTCCACTCCATCACCCGCAGTCATGAAGAGAACCT
AGATCGTTATGAAATATGGAGAACCAACCCTTTTTCTGAGTCTGCAGAAG
AGCTGCGAGACAGAGTCAAAGGAGTCTCTGCCAAGCCTTTCATGGAGACC
CAGCCCACGCTGGATGCATTACACTGCGACATTGGCAATGCAGCAGAGTT
CTACAAAATCTTCCAGGATGAGATCGGGGAGGTGTACCAAAAGGT---CA
AC---CCCAGCCGGGAGGAGCGGCGCAGCTGGAGGGCAGCCTTAGACAAA
CAGCTGAGGAGGAAGCTGAAGCTTAAACCAGTGATGAGGATGAATGGTAA
CTACGCTCGCAGGCTAATGACCCAGGAGGCTGCGGAGGTGGTGTGTGAGC
TGGTGCCCTCAGAGGAGAGAAGGGAGGCCCTGAGGGAGCTCATGAGGATC
TACCTCCAGATGAAGCCTGTGTGGCGCGCCACCTGCCCTGCCAAGGAGTG
CCCCGACCAGCTGTGCCGCTACAGCTTTAACTCCCAGCGCTTTGCCGACC
TTCTCTCCTCTACATTCAAATACAGGTACAATGGTAAAATAACCAATTAC
CTGCACAAGACCCTGGCCCATGTGCCTGAAATCATAGAGAGAGAAGGATC
CATAGGAGCCTGGGCCAGCGAGGGGAACGAGTCAGCGAACAAG-------

---NNNNNNNNNNNNNNNNNNNNAC
TGACCTCTCTGGGTTTTATCATCGGAGTCGGTGTGGTTGGAAACCTCCTG
ATCTCCATCCTGCTGGTCAAAGACAAGAGCCTGCATCGGGCGCCCTACTA
TTTCCTGCTGGACCTGTGCGCCTCTGACATCCTGCGCTCCGCCATCTGCT
TCCCCTTTGTCTTCACCTCGGTCAAAAATGGATCTGCGTGGACCTATGGC
ACGCTGACCTGTAAGGTGATCGCCTTCCTGGGTGTGCTGTCCTGTTTCCA
CACGGCGTTTATGCTGTTCTGTGTCAGCGTCACTCGCTACCTGGCCATCG

CGCACCACCGTTTCTACACCAAGAGGCTGACCTTCTGGACCTGCTTAGCT
GTCATCTGCATGGTGTGGACGTTGTCAGTGGCTATGGCGTTCCCACCGGT
GCTAGACGTAGGGACGTACTCTTTTATCCGGGAGGAGGACCAGTGCACGT
TCCAGCACCGTTCCTTCAGGGCGAATGATTCGCTGGGCTTCATGCTCCTG
CTGGCGCTCATCCTCCTGGCCACACAGCTGGTTTACCTCAAGCTCATCTT
CTTCGTCCAYGATCGTCGGAAGATGAAGCCTGTCCAGTTCGTGCCTGCTG
TCAGCCAGAACTGGACCTTCCACGGGCCAGGCGCCAGCGGGCAGGCGGCG
GCCAACTGGCTGGCTGGATTCGGTCGAGGCCCCACCCCGCCTACCTTGCT
GGGCATCCGGCAGAACAGCAACGCAGCGGGCCGTAGGCGTCTACTGGTAT
TGGATGAATTCAAAACAGAGAAGAGGATTAGTAGGATGTTCTACATCATG
ACGTTTTTCTTCCTGGCACTGTGGGGGCCATATCTCGTTGCCTGCTACTG
GCGGGTGTTTGCAAGGGGCCCTGTAGTCCCTGGAGGCTACCTGACGGCAG
CCGTGTGGATGAGCTTTGCCCAGGCTGGGGTCAATCCTTTCATNNNNNNNNNNNNNNNNNNNNNNNGCCAA
AT
CTCGCTTTCACCCTGGCGTGGGGACTGGTCCTGGCACGGAGC---GCAGC
GTCCCACTCGGCAACAGCTTGCTGTCCCCGCAGCAAACCGACGAGCCCAC
TGTTGCCACCCCCCCGCAGCGATGGTTTGTCACCC---CTGCCAACAACC
GACTGGACTTTGCTGCCTCGGCATACGACGCCGCT---------GATTTC
GCCGGTAACGCGGCCACCTTGCTGTCCTACGCAGCGGCCGGAGTGAAGGC
TC------TTCCCCTGCCGACTGCAGGCTGCTCCAACCGGCCTCTTGGCT
ATTACGCAGACCCGTCAG---GCTGG---GGAGGACGCACGCCGCCGCAG
TACTGTGGCGTAAACAGCAAATCCAGCTCGGTCTTTTCCTGCTGGCCCGC
TAACTCTATCGGTGGCAGGGCGGGCA---CC---AACTACCTGG------
---CTGAGGA---GGGG---GACTC---CATCCCGACAGAGAGGTCACCG
---AT---CGGCGGCTCGGAGGAG---GCCAAACCCAAAGACATGAC---
ATCAGA---GTCGAGCTGGATAGAG---ACCCCGTCCTCCATTAAGTCCA
TTGATTCGAGTGATTCTGGTATCTTTG---AACAGGCCAAGAGGAGAAGA
ATCTCACCCTCTGCCACGCCG---------GTTTCAGAGACAGTGTCCCC
GTTAAAATCTGAGCATCACTCAACAGGCGAAGTCACAGAGAGAGAAGTGG
CGTTGGGGATAAATCCGTTCGCGGATGGGATGGGCGCCTTCAAAATAAAC
CACAGCTCCCACGACATTGGCTCCGG---ACAAACGGCGTTTTCCTCCCA
GGCG---CCCGGCTAC---GCAGCAGCCGCCCTGGGA---CATCACCA--
----CCACCCGACCCACGTTGGCTCT---TACTCCACGGCGGCTTTCAAC
TCCACCAGGGACTTTCTCTTCAGAAATCGGGGTTTCGGGGACGCCACCGG
------------GGCGCAGCACAGTTTGTTCGCCTC---------CGGAA
GTTT---C------GCAGGGCCACATGGACACTCAGATGCAGCGGGGCAC
CTGCTCTTCCCAGGGCTCCACGAG---CAAGCGGCGAGCCATGCGTCTTC
CAACGTGGTCAACAGCCAGATGCGGCTGGGCTTCTCGGGGGACATGTACG
GACGGGCCGACCAGTACGGCCACGTTACAAGCCCACGGT---CCGACCAC
TATGCTTCGACCCAGCTGCACGGCTATGGCCCCATGAACATGAATATGGC
CGCA---CACCACGGAGCAGGGGCCTTCTTTCGATACATGAGGCAGCCGA
TCAAACAAGAGCTCATCTGCAAGTGGATCGAGCCGGAGCAGCTGACGAAT
CCCAAAAAGTCGTGCAACAAAACTTTTAGCACGATGCACGAGCTCGTGAC
CCATCTGACGGTGGAGCATGTGGGGGGACCAGAACAGACCAACCACATCT
GCTTCTGGGAGGACTGCGCCAGAGAAGGAAAGCCATTCAAAGCCAAATAC
AAACTTGTAAATCATATCAGAGTACACACCGGAGAAAAGCCCTTTCACGT
GTCCGTCCCCGGCNNNNNNNNN
>Ceratias holboelli
AGCTTGCTTATCCGGGCCGAACTAAGCCAACCAGGCGCACTCTTAGGGGA
CGACCAAATCTACAACGTTATTGTAACAGCACATGCCTTTGTAATAATCT
TTTTTATGGTTATGCCCATCATGATCGGGGGCTTTGGGAACTGACTAATC
CCACTAATAATCGGAGCCCCTGACATAGCATTTCCCCGGATGAATAATAT
GAGCTTCTGACTTCTCCCCCCATCTTTTCTTCTTCTACTCGCCTCCTCAG

GGGTCGAGGGGGGGGCGGGGACTGGCTGAACGGTTTACCCTCCCCTAGCA
GGCAACCTGGCACATGCGGGAGCTTCTGTAGACCTCACAATTTTCTCACT
CCACCTGGCAGGAGTGTCGTCAATTTTAGGGGCCATTAACTTTATCACAA
CAATCATTAATATGAAGCCCCCAGCTATCTCCCAATACCAAACACCCTTA
TTCGTGTGATCCGTTTTAATTACCGCGGTCTTACTTTTACTATCCCTGCC
CGTTCTGGCTGCCGGCATTACCATGCTCCTCACGGACCGAAATCTAAACA
CAACATTCTTTGACCCTGCAGGAGGGGGAGACCCGATTCTCTACCAACAC
CTA---

-------------------------GGATGAATACATTGTTGTGTTTAGT
CGTTCAACAACAAGACTAATACTGAATGAAGCGGAGCTAATAATGGCACT
GGCTCAGGAGTTCCAGATGAGAGTGGTCACAGTATCCCTGGAGGAACAGT
CATTCCCTAGTATCATCCAGGTGATCAGCAGTGCTACCATGTTAGTCAGT
ATGCATGGAGCTCAACTTATCACCTCACTCTTCCTCCCCAGAGGAGCTGT
TGTGGTGGAGCTGTTCCCCTTTGCTGTGAACCCAGAGCAGTACACCCCAT
ATAAAACCCTTGCCTCCCTTCCGGGCATGGACCTGCATTATATCTCCTGG
AGGAACACTAAGGAGGATAACACCATCACCCACCCAGACAGGCCCTGGGA
ACAAGGGGGTATTGCTCATTTGGATAAGGAGGAGCAAGAACGAATACTGA
TGAGTAAAGATGTGCCCAGGCATCTGTGCTGCCGAAACCCAGAGTGGCTC
TTCCGGATCTATCAGGATACATTGGTGGACATTCCTTCCTTCCTGGAAGT
CCTCAA---AGAGGACATGAAG---ACAAAGCCCAACTTGAAGAA---GT
CAAAGGGGGCCAGCACACTCCACCCGGGCCGGGTCAGAGAACCTCAGTGT
CAGACTTCAGTTCAAACCAGTAATGAGGCTAAACTCACAGTGTCCTGGCA
GATACCGTGGAATCTGAAATACCTGAAGGTCAGAGAGGTGAAATATCAGG
TG---------AAAAAAGACACCAGCAAGGGGACCCTGGAGGATCAAATC
ATCCAAGCGAACCCTGCATTGGAGGCGTTTGGCAACGCCAAAACAGTAAG
AAATGACAACTCCTCCCGTTTTGGAAAGTTCATCCGAATTCACTTTGGTA
CAAGCGGCAAGCTTTCGTCTGCTGACATCGAAACGTACCTGCTGGAGAAG
TCCCGTGTTACCTTCCAGCTCAAGGCTGAGAGAAATTATCACATCTTCTA
CCAGATCCTGTCAAATCAGAAACCAGAGCTGCTGGACATGCTGCTGATCA
CCAACAATCCCTATGACTACTCCTACATCTCCCAAGGAGAGGTAACCGTC
GCCTCCATCAACGACTCAGAGGAGCTGCTGGCCACGGACAGCGCCTTCGA
TGTGCTCGGCTTCACTCCCGATGAGAAGATGGGCGTCTATAAACTCACAG
GTGCCATCATGCACTACGGCAACATGAAATTCAAACAAAAGCAGCGCGAG
GAGCAGGCTGAGCCGGACGGACCGAGGCTGCCGATAAATCGGCTTACCT

CATGGGGCTGAACTCTGCTGACCTCATCAAAGGGCTGTGTCACCCCAGGG
TCAAGGTAGGAAATGAATACGTCACCAAAGGCCAAAGTGTGGATCAAGTC
TACTATCCCAACAAGGAGGCCTTCAAATGTGAGGAGTGTGGGAAGCACTA
CAACACCAAGCTGGGATACAAGCGCCATGTTGCCATGCATTCAGCCACGG
CGGGGGATCTCACCTGTAAAGTGTGCATGCAGACCTACGACAGCACGCCC
ATGCTTCTGGAGCACCTGAAGAGCCACTCTGGGAAGTCCTCAAGTGGCAC
CAAGGAGAAAAAACACCAGTGTGACCACTGTGACCGGCGTTTCTACACAA
GGAAGGATGTGAGACGCCACATGGTGGTCCACACAGGCCGAAAGGACTTC
CTCTGCCAATACTGTGCCCAGCGCTTTGGCAGGAAGGACCACCTGACACG
CCATGTGAAGAAGAGCCACTCACAGGAGCTGCTGAAAATCAAGACAGAGC
CTCCTGATATGTTAAGTCTTTTAGCTTCTGGGTCACCACCATGCTCCGTG
AAGGAGGAGCTCAGCCCCATGATGTGCGGTATGGGTCCCAACAAAGACCC
CATGATGAGCAAACCCTTTCCGAGTGGGGCCCCTTTTCCAATGGGCATGT
ACAACCCTCATCAT------CTCCAGGCCATGTCTAATTCTGGGGTAGGT
CACTCA------CACCCGTCCCTGATGCCCAGTTCTTTGTCTGCAGCTAT
GGGCATGGGCTGTCACATGGAATACCTCATCTACGCCTCCTTCTCATTCA
TGGGATGTTTACAAATCAGTGACGGATCAAATATCGTGAACCTGCTGGCA
AGTAACTCTCCAAGTGTCTCATATGCTCTGACCCAGCAAAAATACTTCAG
TAACTATAGTCCAGTCATTGGGTTTTACATTTATGAGCCCATCGAGTACT
GGAACTCCACAGTGCAGGAGCACCTGAGGTCTCTGAGTCACGGCTTCAAC
AAGATCTCCTGGATGGACAACTTCTTCCACTACCTGCGGGTGGTGAACGT
GAGTGCATCGACCAAGAGCGACTTCATCACCATCCTCAAGGGCTCCTTCC
TGCGCAGCCCGCAGTACCAGCACTTCACGGAGGACATCATTTTCTCAAGG
A---ACCGTGAGACCG------ACGAGTACGACATCATCGCCTCGCGGAT
GTACTTGGTGGCGGAGACCACAGAGAATAAGCGTGAGGAGGTGGTGGAGC
TCCTGGAAAAACTCCGTCCACTGATGCTGATCAACAGCATCAAGTTCATC
GCCTTCAATCCCACGTTTGTGTTCATGGACCGCTACAGCTCCTCTGTCAT
CTCACCCATCCTGACGTCCGGATTCAGCGTACTCACAATCCTCATCCTCA
CTTTCTTCCTGGTCATCAACCCCTTGGGTAACTTATGGCTCATCCTCACT
CTAACGTCCNNNNNNNNNNNNNNNNNNNNNNNNNNN----------------------------------
----

----------------NNNNNNNCCATTGAGATGGGTCCCGTGGGGCCCCGATGGAT
GGAGAGCCCGCAGCCGTTCTCCTGCTCCATCGAAGACCCCACAAAGCAGA
CCAAGTTCAAGGGCATCAAGACGTACATTTCGTACCGGGTGACACCGAGC
CACACGGGGCGCCCCGTCTACAGGCGCTACAAACACTTTGACTGGCTGTA
CAACCGCCTACTGCACAAGTTCACTGTGATCTCCGTGCCTCACCTGCCCG
AGAAGCAGGCCACGGGGCGATTCGAGGAGGACTTCATCGAGAAGCGCAAG
AGGCGACTGATACTGTGGATGAACCACATGACCAGTCACCCAGTCCTCTC
CCAGTATGAAGGGTTCGAGCACTTTCTGATGTGCGCCGACGACAAGCAGT
GGAAGCTGGGTAAGAGGCGGGCGGAAAAAGACGAGATGGTGGGCGCCCAT
TTCATGCTGACCCTCCAGATCCCCCAGGAGCACCAGGACCTTCAGGATGT
GGAGGAGCGCATCGACTCCTTCAAGTCCTTCGCCAAGAAGATGGACGAGA
GCGTGATGCAGCTCACGCACGTCGCCTCGGAGCTGGTGCGCAAGCACCTG
GGCGGGTTCAGGAAGGAGTTCCAGCGGCTGGGGAACGCCTTCCAGTCCAT
CAGCCAGGCATTCATGCTGGACCCTCCCCACAGCTCGGACACCCTSAACA
ACGCCANNNNNNNN--

>Cetostoma regani
AGCCTTCTTATTCGAGCAGAATTGAGCCAGCCTGGAGCCCTCCTGGGGGA
TGACCAAATTTACAATGTAATTGTTACAGCACATGCCTTCGTGATAATTT
TCTTTATAGTAATACCAATTATAATTGGAGGATTTGGAAATTGGTTGGTT
CCTTTAATAATTGGGGCCCCTGATATAGCATTTCCCCGAATAAATAACAT
AAGTTTTTGGCTCCTTCCCCCATCCTTTTTACTACTTTTAGCTTCCTCTG
GAGTTGAAGCAGGCGCTGGGACAGGCTGAACAGTTTACCCCCCTCTTGCA
GGAAACCTTGCCCACGCAGGGGCCTCAGTGGATTTAACCATCTTTTCCTT
ACACTTAGCAGGCGTATCCTCAATTCTAGGGGCTATTAACTTTATTACAA
CTATTATTAATATAAAACCCCCAGCCATCTCACAATACCAGACCCCCCTT
TTTGTATGATCTGTTTTAGTAACAGCCGTCCTCCTCCTTCTTTCCTTACC
AGTTCTTGCAGCCGGTATCACCATACTACTTACTGACCGTAATTTAAACA
CAACCTTTTTTGACCCTGCTGGAGGAGGGGACCCTATTCTTTACCAACAC
TTATTCTGATTTTTTGGGCACCCGGAGGTTTATATTTTAATTTTACCAGG
ATTCGGAATAATCTCACACATCGTTGCCTATTATTCTGGTAAAAAAGAGC
CCTTTGGTTATATAGGAATAGTATGAGCTATAATAGCCATCGGTTTACTA
GGATTTATCGTGTGAGCCCACCATATATTTACAGTAGGAATAGACGTTGA
CACACGAGCATATTTCTAGAGAGGAACCTTCATCCATCTAACTGCCTTGG
CATGCTGTTGCTGTCTGATGCCCACCAGTGTACCAAGCTATCAGAGCTGT
CCTGGGGCATGTGCCTCAGCAACTTTCCTGCTATTTGCAAGACAGAGGAC
TTTCTCCAACTGCCCAAAGACATGGTGGTGCAGCTTCTGTCCCATGAGGA
GCTAGAGACAGAAGATGAGAGACTGGTTTATGAAGCTGCCCTTAACTGGG
TCAACTATGACCTGGAAAGCAGGCACTGCCACCTGCCAGAGCTGCTGAGT
ACGGTCCGCCTGGCCCTGCTACCCGCTATATTCCTCATGGAGAATGTCTC
CACAGAAGAGCTGATCAATGTCCAGGCCAAGAGCAAGGAGCTGGTGGATG
AGGCCATCCACTGCAAGCTGAAGATCTTGCAAAATGATG---TGGTTAAC
AGCCCCTGTGCTCGACCAAGAAAAACCAGCCATGCCCTCTTTCTTCTGGG
AGGGCAGACCTTCATGTGTGACAAGCTGTATCTGGTGGATCAGAAGGCCA
AAGAGATCATCCCCAAGGCTGACATCCCCAGCCCCAGGAAGGAGTTCAGT
GCCTGTGCCATCGGCTGTAAGGTCTACATCACAGGTGGGA--GAGGCTC-
AGAGAATGGTGTGTCCAAAGATGTATGGGTCTATGACACCATCCACGAGG
AATGGTCCAAGGCAGCACCCATGCTCATAGCCAGGTTTGGTCACGGCTCT
GCTGAGCTGAAACACTGCCTCTATGTGGTAGGAGGTCACACAGCTGCAAC
TGGCTGTCTCCCAGCCTCTCCCTCTGGATGAATACATTGTAGTGTTCAGT

CGTTCAACAACAAGGCTAATTCTGAACGAAGCCGAGCTAATCATGGCACT
GGCCCAGGAATTTCAGATGAGAGTGGTTACAGTATCCCTGGAGGAACAAA
CTTTCGCCAGCATCATCCAGGTGATCAGCAGGGCCTCCATGTTAGTCAGT
GTGCATGGAGCTCAGCTTGTCACCTCACTCTTCCTCCCCCGAGGAGCTGC
TGTGGTGGAGCTGTTCCCCTATGCTGTGAACCCAGAACAGTACACCCCAT
ATAAAACACTTGCCTCCTTGCCAGGCATGGACCTTCAATACGTTTCCTGG
AGGAACACTATGGAGGGGAACACCATCACCCACCCAGACCGACCCTGGGA
ACAAGGAGGTATCGCYCATTTGGAAAAAGATGAACAAGAGCGAATACTGG
CCAGCAAGGACGTCCCCAGGCACCTGTGCTGCCGCGACCCGGAGTGGCTC
TTCCGAATCTACCAGGACACTTTGGTAGACATCCCTTCATTCCTGGAAGT
CATCAA---AGAGGGCATCAAG---GCTAGGCCCAGCCTGAGGAA---GG
CCAAGCCGGCCAGCACGGTTCATCCGGGCCGGGTCAGAGAACCGCAGTGC
CACACCTCAGTCCAAGCCACCAACGAGGCTAAACTCACGGTGTCCTGGCA
GATCCCGTGGAATCTGAAATACCTGAAGGTGAGAGAAGTGAAGTATGAGG
TG---------AAGAAGGACACCAGCAAGGGAACACTGGAGGATCAAATC
ATCCAGGCAAACCCTGCACTGGAAGCCTTTGGAAATGCCAAAACAGTGAG
GAATGACAACTCATCCCGTTTTGGAAAATTCATYCGAATTCACTTTGGAA
CCAGTGGCAAGCTGGCGTCTGCTGACATTGAGACTTACCTGCTGGAGAAG
TCGCGTGTCACCTTCCAGCTCAAGTCTGAGAGGAACTACCACATCTTCTT
CCAGATCTTGTCCAATCAGAAGCCAGAGCTGCTGGACATGCTGTTGATTA
CCAACAACCCATATGACTACTCCTACATCTCCCAAGGAGAGGTAACAGTA
CAATCCATCAACGACTCAGAGGAACTGTTGGCCACTGACAGTGCCTTCAC
TGTACTTGGCTTCAATCAAGAGGAGAAGTTGGGAATCTATAAGCTGACCG
GTGCCATTATGCACTAYGGCAACATGAAGTTTAAGCAGAAGCAGCGTGAG
GAGCAGGCTGAACCTGATGGAACGGAGGCTGCTGATAAGGCAGCTTACCT
AATGGGGCTGAACTCTGCAGACCTTATCAAAGCCCTGTGCCATCCCAGAG
TCAAGGTAGGAAATGAATATGTTACCAAAGGCCAGGGTGTAGACCAAGTC
TACTACCCCAACAAAGAGGCCTTCAAGTGTGAGGAGTGTGGCAAGCACTA
CAACACCAAGCTGGGATACAAGCGCCATGTGGCCATGCACTCTGCCACGG
CAGGGGACCTTACCTGTAAAGTGTGCATGCAGAGCTTCGAGAGCACGCCC
GTGCTCCTGGAGCACCTCAAGAGCCACTCAGGGAAGTCCTCGGGTGGCGC
CAAGGAGAAGAAACACCCATGTGACCACTGCGACCGTCGCTTCTACACTC
GGAAGGATGTAAGACGGCACATGGTGGTCCATACAGGTCGAAAGGACTTC
CTGTGCCAGTACTGTGCCCAGCGCTTTGGCAGGAAGGACCACCTGACACG
GCATGTGAAGAAGAGCCACTCGCAGGAGCTGCTGAAGATCAAGACAGAGC
CTCCGGATATGTTAAGTCTTTTAGGTTCTGGCTCTCCACCTTGTTCCGTC
AAGGAGGAGCTTAGCCCCATGATGTGCAGCATGGGTCCCAACAAAGACCC
CATGATGGGCAAACCTTTCCCCAGCGGGACCCCCTTCCCGATGGGCATGT
ATAACCCCCATCAT------CTCCAGGCCATGTCCAATTCTGGNGNGGGTCA
CCCC------CACCCCTCCCTGATGCCCAGTCCCCTGTCTGCAGCTATGG
GCATGGGCTGTCACATGGAATATCTCATCTACGCCTCGTTCTCATTCATG
GGATGTTTACAAATCAGTGATGGGTCAAACATTGTGAACTTGCTGGCCAG
TAACTCTCCGAGCGTTTCGTACGCGCTGACCCAGCAGAAATACTTCAGTA
ACTACAGYCCCGTGATCGGGTTTTACATTTACGAGCCCATTGAGTACTGG
AACTCCACGGTGCAGGAGCATCTAAAGACACTGAGTCACGGCTTCAACAA
GATCTCCTGGATGGACAACTTCTTCCACTACCTGCGGGTGGTGAACGTGA
GYGCGTCGAACAAGAGCGACTTCATCACCATCCTCAAGGGCTCCTTCCTG
CGCAGCCCAGAGTACCAGCACTTCACCGAAGACATCATCTTCTCTAAGA-
--ACCGTGAGAGTG------ATGAGTATGACATTATTGCCTCACGCATGT
ACCTGGTGGCGCGGACCACAGAGAAAAAGCGCGAGGAGGTGGTGGAGCTT
CTGGAGAAACTGCGTCCGCTGATGCTAATCAACAGCATYAAGTTCATCGC
CTTCAACCCCACCTTTGTTTTCATGGACCGTTACAGCGCCTCTGTCATCT
CACCCATCCTGACCTCAGGCTTCAGCGTGCTCACCATCCTCATCCTCACT

TTTTTCCTGGTYATCAACCCCTTGGGGAACTTCTGGCTCATCCTGATGGT
TACTTCTGTGGAGCTGGGCGTCTTGGGTTTAATGGGCTATCACCTATTTG
AGTGGCAGCCGGCCCTCAAGAACGTGTCTGTATCCTGTCATATGGGCATA
ATCAATGGGCTCTCAGGATGGCCTGCCTCAGTGGATGATTCCCCAGCTGA
CACCATCACTCGTCGATTTCGCTATGATGTGGCCCTGGTGTCAGCCTTGA
AGGATCTGGAGGAGGACATCATGGACGGACTGAGAGAGTGTGGGCTGGAA
GACAGCGCTTGCTCCTCAGGCTTCAGTGTTTCAATCAAGGAATCTTGTGA
TGGCATGGGAGATGTCAATGAGAAGCATGGCGGAGGGCCAATGGTCCCTG
AGAAGGCTGTGCGTTTCTCTTTTACTATTATGTCTGTCTCTGTCTTTGCA
GATGGAGAGGAT------------------------------GAGCCGGT
TACCATCTTCAGGGAGCCAAAGCCCAACTCAGAGCTTTCCTGTAAGCCCC
TATGCCTGATGTTTGTAGATGAGTCAGACCACGAGACACTCACTGCTATA
CTGGGGCCTGTAGTTGCAGAGCGTAATGCAATGAAGCAGAGTCGACTCAT
CCTATCTATGGGGGGCCTGCTTCGCTCCTTCTGCTTCCAGTTCAGAGGCA
CAGGATATGATGAGAAGATGGTGCGTGAGATGGAGGGCCTGGAGGCCTCA
GGTTCCACCTACATCTGCACTCTGTGTGACTCCAGTCGGGCAGAGGCCTC
TCGCAACATGGTGCTGCACTCCATCACTCGCAGTCACGATGAGACCCTGG
AGCGTTATGAAATATGGAGGACCAATCCATTTTCTGAATCTACTGAGGAA
CTGCGAGACCGGGTCAAAGGGGTCTCCGCCAAGCCCTTTATGGAGACCCA
TCCGACTCTGGATGCATTGCACTGTGACATTGGTAATGCCACTGAGTTCT
ACAAATTGTTCCAGGATGAGATTGGGGAGGTGTTCAAAAAGGT---CAAC
---CCCACCCGGGAGGAACGACGGAGCTGGAAGGCAGCCTTAGACAAGCA
GCTGAGGAAGAAGATGAAGCTTAAACCCGTGATGAGGATGAATGGGAACT
TTGCCCGTAGGCTAATGTCCCAGGAGACTATAGAGGTGGTGTGTGAGCTG
GTGCCCTCAGAAGAGAGGAGGGAGGCCCTGAGGGAGCTTATGGGGCTCTA
CGTCCAGATGAAGCCTGTGTGGCGCTCCACCTGCCCAACCAAGGAGTGCC
CCGACCAACTGTGCCGCTACAGCTTTAACTCCCAGCGATTTGCCGATCTC
CTCTCCACTACCTTCAAATATAGGTACAATGGAAAAATTACCAATTACCT
GCACAAGACCCTAGCCCATGTCCCTGAAATCATAGAGAGAGATGGCTCCA
TAGGAGCCTGGGCCAGTGAAGGGAATGAGTCAGCAAACAAATCGTACACC
ATTGAGATGGGCCTCAAGGGGCCCCAGTGGAAGGAGAGCCCRCAGCCGTT
CTCCTGCTCCATTGAAGACCCCACAAAACAGACCAAGTTCAAGGGCATCA
AGACCTACATTTCGTACCGGGTCACGCCGAGYCACACGGGGCGTCCCGTC
TACAGGCGCTACAAACACTTTGACTGGCTGTACAACCGCTTACTGCACAA
GTTCACTGTGATCTCGGTGCCCCACCTGCCCGAGAAGCAGGCCACGGGGC
GCTTCGAGGAGGACTTCATCGAGAAGCGTAAAAGACGACTGATCCTGTGG
ATAAACCACATGACCAGTCACCCAGTCCTCTCCCAGTATGAGGGCTTTGA
ACACTTCCTGATGTGTGCTGATGACAAGCAATGGAAGCTGGGYAAGAGGC
GGGCGGAGAAGGACGAGATGGTGGGCGCCCACTTCATGCTGACCTTCCAG
ATTCCTAACGAGCACCAGGACCTCCAGGACGTCGAGGAGCGAGTYGACTC
CTTCAARTCRTTCGCTAAGAAAATGGACGACAGCGTCATGCAGCTGACAC
ACGTTGCCTCGGAGCTGGTGCGTAAACACCTGGGTGGGTTCAGGAAGGAG
TTCCAGCGGCTGGGAAACGCCTTCCAGTCTATCAGCCAGGCCTTCATGCT
GGACCCTCCCCACAGCTCAGACGCTCTCAACAACGCCATCTCCCAC----

>Chaetodon striatus
AGTCTGCTCATCCGAGCAGAACTCAGCCAGCCAGGCACCCTCCTAGGCGA
CGATCAGATTTATAATGTAATCGTTACGGCACATGCGTTCGTAATGATTT
TCTTTATAGTAATACCGATCATGATTGGGGGATTTGGGAATTGACTAATT
CCCTTAATGATTGGGGCTCCGGACATGGCCTTCCCTCGAATAAATAACAT
AAGCTTTTGACTTCTCCCTCCATCCTTTTTCCTACTTCTTGCCTCCTCTG
GCGTAGAGTCCGGGGCCGGCACTGGGTGAACAGTCTATCCCCCGCTGGCG
GGTAATCTAGCCCACGCCGGGGCATCCGTTGATTTAACTATTTTCTCCCT
TCACCTTGCAGGGATCTCCTCTATTCTAGGGGCTATTAATTTTATTACGA

CAATCCTTAACATGAAACCCCCTGCTATGTCTCAGTATCAAACTCCCCTT
TTCGTCTGATCTGTCTTAATTACAGCCGTTCTACTTCTCTTGTCCCTCCC
CGTTCTTGCAGCCGGAATTACAATACTCCTTACAGATCGAAACCTTAATA
CAACCTTCTTTGACCCTGCGGGGGGCGGCGATCCCATTCTTTACCAACAT
CTATTC--

------------TTCCTAGAGAGAAACCTTCACCCATCTAACTGCCTCGG
CATGCTGTTGCTGTCCGATGCCCACCAGTGCACCAAGCTGTCAGAGCTCT
CCTGGGGCATGTGCCTCAGCAACTTTCCCGCTATTTGCAAGACAGAGGAC
TTCCTCCAACTGCCCAAAGATATGGTGGTGCAGCTTTTGTCACATGAGGA
GCTCGAGACAGAAGATGAGAGACTGGTTTATGAAGCTGCCCTCAATTGGA
TCAACTATGACCTGGAAAAGAGGCACTGCCATCTTCCAGAACTCCTGAGA
ACGGTCCGCCTGGCCCTGCTGCCTGCCATCTTTCTCATGGAGAACGTATC
TACAGAAGAGCTGATCAACGCCCAGGCCAAGAGCAAGGAGCTGGTGGATG
AAGCTATCCGCTGTAAGCTGAAGATCCTCCAGAATGATGGTGTCGTCAAC
AGCCCGTGTGCCCGACCAAGAAAAACCAGCCATGCCCTCTTTCTTCTGGG
AGGGCAGACTTTCATGTGTGACAAGTTGTACCTGGTGGACCAGAAAGCCA
AAGAGATCATCCCCAAGGCTGACATCCCCAGCCCCAGGAAGGAGTTCAGC
GCCTGCGCCATCGGATGTAAGGTGTACATCACTGGTGGGA--GAGGCTC-
AGAGAATGGTGTGTCCAAAGATGTGTGGGTCTACGACACCGTCCACGAGG
AATGGTCGAAGGCGGCGCCCATGCTCATTGCCAGGTTTGGCCATGGTTCT
GCGGAGCTGAAACACTGCCTCTACGTGGTTGGAGGTCACACAGCTGCAAC
TGGCTGCCTCCCGGCTTCTCCATCTGGATGAATACATTGTTGTGTTTAGT
CGCTCAACAACGAGGCTGATACTGAATGAAGCSGAGCTAATCATGGTGCT
AGCCCAGGAGTTCCAGATGAGAGTGGTCACAGTATCTCTGGAGGAACAGT
CTTTCCCCAGTATAGTCCAGGTGATCAGCAGTGCTACCATGTTGGTTAGT
ATGCATGGAGCTCAGCTCATCACCTCACTGTTCCTTCCAAGAGGAGCTGT
TGTGGTGGAACTCTTCCCTTTTGCTGTAAACCCAGAGCAGTACACCCCAT
ATAAAACCCTTGCCTCCCTGCCAGGCATGGACCTTCACTATGTCTCTTGG
AGGAACACTAAGGAGGAGAATACCATCACCCATCCAGACAGACCCTGGGA
ACAAGGGGGCATTGCTCACTTGGAGAAAGAGGAGCAAGAGCGAATACTGT
TGAGCAAAGATGTCCCTAGGCACTTGTGCTGCCGCAACCCAGAGTGGCTC
TTCCGGATCTACCAGGACACTTTGGTGGACATTCCTTCCTTCCTGGAAGT
CCTCAA---AGAGGGCATGAAG---ACAAAGCCCAATTTGAAGAA---GT
CAAAGCCGGCCAACGCAGTCCACCCGGGCCGGGTCAGAGAACCCCAGTGT
CAAACCTCAGTTCAAACCACTAATGAGGCTAAACTCACAGTCTCCTGGCA
NATCCCGTGGAATCTGAAATACCTTAAGGTCAGAGAGGTGAAATATGAGGT
GTGGATCCGGAAAAAAGATACCAGCAAGGGAACCCTGGAGGATCAAATCA
TCCAGGCGAACCCTGCGTTGGAGGCTTTCGGCAACGCCAAAACGCTGAGA
AACGACAACTCGTCTCGTTTTGGAAAATTCATCCGAATTCACTTTGGTAC
AAGCGGCAAACTGTCRTCTGCTGACATCGAGACATACCTTCTGGAGAAAT
CACGTGTCACCTTTCAGCTCAAGGCTGAGAGGAATTATCACATCTTCTAC
CAGATCCTGTCCAATCAGAAGCCAGAGCTGCTGGATATGCTGCTGATCAC
CAACAACCCGTATGACTACTCCTACATCTCCCAAGGAGAAGTGACAGTGG
CGTCCATCAACGACTCTGAGGAGCTGATGGCCACAGACAGCGCCTTTGAT
GTGCTTGGCTTCACTCCAGATGAGAAGATGGGTGTCTATAAACTGACGGG
AGCCATCATGCACTATGGCAACATGAAGTTCAAACAGAAACAGCGTGAGG
AGCAGGCTGAGCCTGATGGGACGGAGGCTGCTGATAAATCAGCTTACCTA
ATGGGGCTGAACTCTGCTGACCTCATCAAAGGGCTGTGCCACCCCAGAGT
CAAGGTAGGAAATGAATATGTCACCAAAGGCCAAAGTGTAGACCAAGTCT
ACTATCCTAACAAGGAGGCCTTTAAGTGTGAGGAGTGTGGGAAGCACTAC

AACACCAAGCTGGGATATAAGCGCCATGTGGCCATGCACTCTGCCACAGC
AGGGGATCTTACCTGTAAAGTGTGCATGCAGACCTACGAGAGCACGCCTG
TGCTTTTGGAGCACCTCAAGAGCCACTCCGGGAAGTCTTCAGGTGGCACC
AAGGAGAAAAAGCACCCATGTGACCACTGTGACCGTCGTTTCTACACACG
GAAGGATGTGAGACGGCACATGGTGGTCCACACGGGCCGAAAGGACTTCC
TGTGCCAGTACTGTGCCCAGCGCTTTGGTAGAAAAGACCATCTGACACGC
CATGTGAAGAAGAGCCACTCACAGGAGCTGCTGAAAATCAAGACAGAGCC
TCCTGATATGCTAGGTCTTTTAGCTTCCGGGTCACCACCTTGCGCTGTGA
AGGAGGAGCTCAGTCCCATGATGTGTGGCATGGGTCCCAACAAAGACCCC
ATGATGGGCAAGCCGTTCCCCAGTGGGCCCCCTTTTCAGATGGGAATGTA
CAACCCCCACCAT------CTCCAGGCCATGTCTAATTCTGGGGTGGGAC
ACCCA------CACCCGTCCCTGATGCCCAGTTCYTTGTCTGCAGCTATG
GGCATGGGCTGTCACATGGAANNNNNNNNNNNNNNCCTCCTTCTCATTCATGGGATGTTTACAA
ATCAGTGATGGATCAAATATTGTGAACCTGCTGGCGAGTAACTCTCCGAG
TGTTTCGTATGCTCTGACCCAGCAAAAATACTTCAGTAACTACAGTCCTG
TGATTGGGTTTTACATTTACGAGCCCATAGAGTACTGGAACTCCACAGTG
CAGGAGCACCTGAAGACTCTGAGTCATGGTTTCAACAAGATCTCCTGGAT
GGACAACTTTTTCCACTACCTGCGGGTGGTGAATGTGAGTGCGTCAACCA
AGAGCGACTTCATCACCATCCTCAAGGGCTCCTTCCTGCGCAGCCCGGAG
TACCAGCACTTCACTGAGGACATCATATTCTCTAAGA---ACCGCGAGAC
TG------ATGAGTACGACATTATCGCCTCACGGATGTACTTGGTGGCAC
GGACGACAGAGAAGAAGCGTGAGGAGGTGGTGGAGCTTCTGGAAAAGCTT
CGTCCGTTGATGCTGATTAACAGCATCAAATTCATTGCCTTCAATCCTAC
GTTTGTGTTCATGGACCGCTACAGCTCCTCTGTCATCTCGCCCATCCTGA
CCTCAGGATTCAGCGTACTCACAATCCTCATCCTCACTTTCTTCCTGGTC
ATCAACCCCTTGGGGAATTTCTGGCTCATCCTCACTGTAACGTCCGTGGA
GCTGGGTGTCTTGGGTTTGATGNNNNNNNNNNNNNNNNNNNNNNNNNNNNNNNNNNNNNNNNNNNNCTACA
TCTTGCAATGTTGGCATTATTAA
TGGGCTCTCTGGATGGGCTTCCTCAGTGGATGACTCCCTGGCTGACACTA
TAACTCGGCGGTTTCGCTATGATGTGGCACTGGTGTCAGCGTTAAAAGAT
CTGGAGGAGGACATCATGGAGGGGCTGAGAGAGAGTGGGATGGAAGACAG
CGCTTGCACCACAGGCTTCAGTGTCATGATCAAGGAATCTTGTGATGGCA
TGGGTGATGTCAGTGAGAAGCACGGTGGAGGACCAGCTGTTCCTGAGAAG
GCTGTACGTTTCTCTTTCACGATTATGTCTGTTTCCATCCTGGCAGCCGA
CAAGGAG------------------------------GAAGAGGTTACCA
TCTTCACGGAGACAAAGCCAAACTCAGAATTGTCCTGTAAGCCCCTTTGC
CTGACATTTGTGGATGAGTCAGACCATGAGACACTCACAGCCATCCTGTC
GCCTGTAGTTGCAGAGCGTAGTGCAATGAAAGAGAGCAGGCTCATCCTAT
CCATCGGTGGACTGCTTCGCTCCTTCCGCTTTCACTTCAGAGGCACGGGA
TATGATGAGAAGATGGTGCGTGAGATGGAGGGCCTCGAGGCCTCAGGGTC
CACCTATATCTGCACCCTCTGTGACTCCGGTCGGGCAGAAGCTTCTGAAA
ACATGGTACTACACTCCATTACTCGCAGTCATGAAGAGAACCTAGAACGT
TATGAAATTTGGAGAACCAACCCCTTTTCTGAGTCCGCAGATGAGCTACG
AGACAGAGTCAAAGGGGTCTCTTCCAAGCCCTTTATGGAGACCCATCCCA
CGATGGATGCATTACACTGTGACATAGGCAATGCTACTGAGTTCTACAAA
ATCTTCCAGGATGAGATCGGGGAGATGTACCAAAAGGT---CAAC---CC
CAGCCGGGAGGAGCGGCGCAGCTGGAGGGCAGCCCTAGATAAACAGCTGA
GGAAGAAGGTGAAGCTGAAACCAGTAATGAGAATGAATGGGAACTTTGCC
CGCCGGCTACTGACCCTGGAGACTGTGGAGGCAGTGTGTGAGCTGGTGCC
CTCTGAGGCAAGGAGGGAGGCCCTGAGGGAGCTTATAAGACTCTACCTCC
AGATGAAGCCTGTGTGGCGTGCCAGCTGCCCAGCAACGGAGTGCCCTGAC
CAGCTGTGCCGCTATAGCTTTAACTCCCAGCGCTTTGCTGACCTCCTCTC
CTCTACTTTCAAATATAGGTACAAGGGGAAGATACCCAATTACATGCACA

AGACCCTGGCCCATGTGCCTGAAATCATAGAGAGAGATGGATCCATAGGA
GCCTGGGCCAGCGAAGGAAACGAGTNNNNNNNNNNNNNNNNNNNNNNNNNNNNNNNNNNCCTTAGGGCCCC
GGTGGAAGGAGAG
CCCACAGCCTTTCTCCTGTTCCATTGAAGACCCCACTAAACAAACAAAGT
TCAAGGGCATCAAGACGTACATATCGTACCGGGTCACGCCGAGCCACACT
GCGCATCCTGTCTACAGGCGCTACAAACACTTTGACTGGCTGTACAACCG
CTTACTGCACAAGTTCACTGTGATCTCTGTGCCTCACCTGCCTGAGAAGC
AGGCCACGGGGCGATTCGAGGAAGACTTCATCGAGAAGCGCAAGAGGCGA
CTGATACTGTGGATGAACCACATGACCAGTCACCCAGTCCTCTCCCAGTA
TGAAGGCTTCGAGCACTTTCTGATGTGTGCCGATGACAAGCAGTGGAAAC
TGGGCAAGAGACGGGCGGAGAAGGACGAGATGGTGGGTGCCCATTTCATG
CTGACCCTCCAGATCCCTAACGAACACCAGGACCTTCAGGATGTAGAGGA
GCGGGTCGACTCCTTCAAGGCCTTCGCTAAGAAAATGGATGACAGCGTGA
TGCAGCTCACTCATGTTGCCTCGGAGCTGGTGCGKAAGCACCTGGGTGGG
TTCAGGAAGGAGTTCCAGCGACTGGGAAATGCCTTCCAGTCTATCAGCCA
GGCGTTCATGCTGGACCCTCCCCACTGCTCAGAGACCTTCAACAACGCCA
TCTCCCATNNNNNNNNNNNNNNNNNNNNNNNNNNNNNNNNNNNNNNNNNNNNNNNNNNNNNNNNNNNNNNNN
NNNNNNNNNNNNNNNNNNNNNNNNNNNNNNNNNNNNNNNNNNNNNNNNNNNNNNNNNNNNNNNNNNNNNNNN
NNNNNNNNNNNNNNNNNNNNNNNNNNNCCGCCATCTGCTTCCCCTTTGTCTTCACCTCGGTCAAGAATG
GATCTGCCTGGACCTACGGCACGCTGACCTGCAAGGTGATCGCCTTTCTG
GGTGTCCTCTCCTGTTTCCACACAGCGTTTATGCTGTTCTGTGTCAGTGT
CACACGCTACCTGGCCATCGCACATCACCGTTTCTACACCAAGAGGCTGA
CCTTCTGGACCTGTCTAGCTGTCATCTGCATGGTGTGGACGTTGTCTGTG
GCTATGGCGTTCCCACCGGTGCTAGACGTGGGGACATACTCTTTCATCCG
GGAGGAGGACCAGTGCACATTCCAGCACCGTTCCTTCAGGGCGAATGATT
CGCTGGGCTTCATGCTCCTGCTGGCGCTCATCCTCCTGGCCACACAGCTG
GTTTACCTCAAGCTCATCTTCTTCGTCCACGACCGCCGAAAGATGAAGCC
TGTCCAGTTCGTGCCTGCCGTCAGCCAGAACTGGACCTTCCACGGGCCAG
GYGCCAGCGGGCAGGCGGCGGCCAACTGGCTGGCCGGGTTTGGTCGAGGC
CCCACCCCGCCTACTTTGCTGGGCATCCGGCAGAACAGCAACGCAGCGGG
CCGCAGGCGTCTGCTGGTTTTGGATGAATTCAAAACAGAGAAGAGGATTA
GTAGGATGTTCTACATCATGACGTTTTTCTTCCTGGCATTGTGGGGGCCC
TATCTGGTCGCCTGCTACTGGCGGGTGTTTGCAAGGGGCCCCGTGGTCCC
TGGGGGCTACCTGACAGCAGCCGTGTGGATGAGCTTTGCCCAGGCTGGGG
TCAATCCTTTCATCTGCATCTTTCTCCAACAGGGANNNNNNNNNNNNNNNNNNNNGGCGTGGGGACTGG
T
CCTGGCACGGAGC---GCAGCGTCCCACTCGGCAACAGCTTGCTATCCCC
GCAGCAAACCGAGGAGCCCACTGTTGCCACCCCCCCGCAGCGATGGTTTG
TCACCC---CTGCCAACAACCGACTGGACTTTGCTGCCTCGGCATACGAC
GCCGCT---------GATTTCGCCGGTAACGCGGCCACCTTGCTGTCCTA
CGCAGCGGCCGGAGTGAAGGCTC------TTCCCCTGCCGACTGCAGGCT
GCTCCAACCGGCCTCTTGGCTATTACGCAGACCCGTCAG---GCTGG---
GGAGGACGCACGCCGCCGCAGTACTGTGGCGTGAATAGCAAATCCAGCTC
GGTCTTTTCCTGCTGGCCCACTAACTCTATCGGTGGCAGAGCAGGCA---
CC---AACTACCTGG---------CTGAGGA---GGGA---GACTC---C
ATCCCGACGGAGCGGTCACCG---AT---CGGCGGCACGGAGGAG---AC
CAAACCCAAAGACATGAC---ATCAGA---GTCAAACTGGATAGAG---A
CGCCGTCCTCCATTAAGTCCATCGACTCAAGCGATTCTGGTATCTTTG--
-AACAGGCCAAAAGGAGAAGGATCTCACCTTCTGCAACGCCA--------
-GTTTCAGAGACAGTGTCCCCGTTAAAATCAGAGCATCACTCAACAGGCG
AAGTCACAGAGAGAGAAGTGGCGTTGGGGATAAATCCGTTCGCGGATGGG
ATGGGCGCCTTCAAAATAAACCACAGCTCCCACGATATTGGCTCCGG---
ACAGACGGCGTTTTCCTCCCAGGCG---CCCGGCTAC---GCAGCAGCCG

```
CCCTGGA---CACCACCA------CCACCCGACCCACGTTGGCTCT---
TACTCCACGGCGGCTTTCAACTCCACCAGGGACTTTCTCTTCAGAAATCG
AGGATTCGGGGACGCCACCGG------------CGCGCAACACAGTTTGT
TCGCCTC---------CGGAAGTTT---C------GCAGGCCCACATGGA
CACTCAGATGCAGCGGGGCACCTGCTCTTCCCGGGGCTCCACGAG---CA
AGCGGCGAGCCATGCGTCTTCCAACGTGGTCAACAGCCAGATGCGGCTGG
GCTTCTCGGGGGACATGTACGGACGGGCCGACCAGTACGGCCACGTTACA
AGCCCGCGGT---CCGACCACTATGCTTCGACCCAGCTGCACGGCTACGG
CCCCATGAACATGAATATGGCCGCG---CACCACGGAGCAGGGGCCTTCT
TTCGATACATGAGGCAACCGATCAAGCAAGAGCTCATCTGCAAGTGGATC
GAGCCGGAGCAGCTGACAAATCCCAAAAAGTCGTGCAACAAAACTTTTAG
CACGATGCACGAGCTGGTGACCCATCTGACGGTGGAGCATGTGGGGGGAC
CGGAGCAGACCAACCACATCTGCTTCTGGGAGGAGTGCTCCCGAGAAGGA
AAGCCATTCAAAGCCAAATACAAACTTGTAAATCATATCAGAGTACACAC
CGGAGAAAAGCCCTTTCCGTGTCCGTTTCCCGGCTGTGGCAAA
>Chanos chanos
AGCCTCTTAATTCGAGCAGAGCTTAGCCAACCAGGATCTCTTCTGGGCGA
TGATCAAATCTATAACGTCATCGTCACAGCGCACGCTTTTGTAATAATCT
TCTTTATAGTAATGCCTATCCTCATTGGAGGGTTCGGGAACTGACTTGTC
CCACTAATGATCGGGGCCCCAGACATGGCATTCCCTCGAATGAACAACAT
AAGCTTCTGGCTTCTTCCACCTTCGTTCCTTCTCCTCCTAGCATCGTCTG
GAGTTGAAGCCGGAGCCGGAACAGGATGAACAGTCTACCCCCCACTAGCC
GGAAATCTTGCTCACGCAGGAGCCTCCGTGGACTTAACAATTTTCTCTCT
TCACCTAGCAGGGGTCTCTTCAATTCTTGGAGCAATTAATTTCATTACTA
CTATTATTAACATGAAACCCCCAGCCATCTCCCAATATCAAACACCTCTA
TTTGTTTGAGCCGTTCTCGTTACAGCCGTGCTTCTCCTTCTATCTCTTCC
AGTGCTAGCCGCTGGAATTACGATGCTCCTGACAGATCGAAACCTTAATA
CAACATTCTTCGACCCGGCTGGAGGAGGAGACCCAATTCTGTACCAACAC
CTGTTCNNNNNNNNNNNNNNNNNNNNNNNNNNNNNNNNNNNNNNNNNNNNNNNNNNNNNNNNNNNNNNNNNN
NNNNNNNNNNNNNNNNNNNNNNNNNNNNNNNNNNNNNNNNNNNNNNNNNNNNNNNNNNNNNNNNNNNNNN
NNNNNNNNNNNNNNNNNNNNNNNNNNNNNNNNNNNNNNNNNNNNNNNNNNNNNNNNNNNNNNNNNNNNNN
NNTTCCTGGAGAAGAACTTGCATCCATCCAACTGCCTGGGCATGCT
CCTGCTGTCTGATGCCCACCAGTGCACGCAGCTTTTCCAGCTCTCCTGGA
GTATGTGCCTGAGCAACTTTCCTGCCATCTGTAAGACTGAAGAATTCCTG
CAACTTCCTAAAGACATGCTAGTCCAGCTACTGTCTCATGAGGAACTGGA
GACTGAGGATGAACGGCTAGTCTATGAATCTGCGCTCAACTGGGTGAACT
ATGACCTGGAACGTCGGCATTGCCACCTTCCGGAGCTGCTCCGGACAGTG
CGGCTGGCGCTGCTTCCTGCCATATTCCTGATGGAGAACGTCTCTACAGA
GGAGCTGATCAATACTCAAGCCAAAAGCAAGGAGCTGGTGGATGAGGCCA
TCCGATGTAAGCTGCGCATCCTACAGAACGATGGGGTGGTCAACAGCCCC
TGTGCCCGTCCCCGTAAAACTAGCCATGCCCTTTTCCTACTGGGAGGTCC
TACCTTCATGTGTGATAAGCTGTACCTGGTGGATCAGAAGGCCAAGGAGA
TCATTCCTAAAGCTGACATTCCTAGTCCACGAAAGGAGTTCAGCGCTTGT
GCCATTGGTTGCAAAGTATATGTGACGGGCGGGC--GTGGCTC-CGAAAA
CGGCGTATCCAAAGACGTTTGGGTGTATGACACACTGCATGAGGAGTGGT
CCAAAGCAGCACCAATGCTAATTGCACGATTTGGCCACGGCTCAGCCGAG
TTACGGCACTGCCTCTATGTGGTTGGAGGTCACACGGCTGCAACAGGTTG
CCTTCCTGCATCGCCGTCTGGACGATTACATAGTGGTTTTCAGTCGCTCT
ACCAATCGGCTAATCCTCAACGAAGCGGAGCTGATCCTGGCTCTCGCACA
AGAGTTTCAGATGAGGACCATCACTGTGTCTCTGGATGAGCAGTCGTTTG
CCAGCATAATCCAGATTATCAGCGGGGCATCGATGTTGGTCAGCATGCAT
GGGGCCCAACTGATAACCTCCATATTCCTGCCCAGAGGGGCTGCTGTGAT
TGAGCTCTTCCCCTATGCAGTTAACCCAGAACACTACACACCATACAAGA
```

CCCTCGCCTCTTTGCCAGGCATGGACCTCCAGTACGTTGCATGGAGAAAC
ACAATAGGAGAGAACTCGGTGGCTTACCCTAACCGTCCTTGGGACCAGGG
TGGCATCGCACATCTGGAGAAGGAAGAGCAGGACCGAATAATGGCCAGCA
AGGAGGTCCCTCGGCACCTGTGTTGCCGTAACCCAGAATGGCTGTTCCGG
ATCTACCAGGACACTGTTGTGGACGTCCCCTCCTTCTTGGATGTCCTCAG
---AGAAAGTCTGAAG---GGCAAGCCTAGCCTTAAAAA---GACCAAGG
CTACCAGCACAGTTCATCCCGGTCGAGTTCGAGAGCCCAAGTGCCAGACA
TCTGTGCAAGCCACCAACGAGGCCAAGCTGACAGTGTCGTGGCAGATCCC
TTGGAATCTCAAGTACCTGAAAGTTCGAGAGGTCAAATACGAAGTGTGGA
TTCAGAAGAAGGATTCTAATAAGGGGACCCTGGAAGATCAGATTATCCAG
GCTAATCCTGCACTGGAGGCGTTCGGCAATGCCAAAACGGTGAGAAATGA
CAATTCCTCTCGCTTTGGGAAGTTCATTCGCATTCATTTCGGGACAAGTG
GCAAACTGTCTTCTGCAGACATAGAAACATACTTGCTTGAAAAATCTCGG
GTAACATTTCAGCTTAAAGCGGAGAGGAACTATCACATCTTCTATCAGAT
ATTATCCAATGAAAAGCCAGAGTTACTGGACATGCTGCTGATTACCAATA
ACCCTTATGATTACGCATACATCTCCCAAGGAGAAGTAACTGTTGCTTCC
ATCAATGATAACGAAGAGCTTGTTGCAACAGACAGTGCCTTTGATGTGCT
TGGCTTTACTTCAGATGAAAAGGTAGGGGTCTATAAGTTGACTGGTGCAA
TAATGCACTATGGCAACATGAAGTTTAAGCAGAAGCAGCGTGAGGAGCAG
GCAGAGCCTGATGGAACAGAGTCTGCTGACAAGTCAGCATACCTGATGGG
GCTGAATTCTGCAGATCTTCTAAAGGGACTCTGCCACCCCAGGGTAAAAG
TGGGAAATGAATACGTCACAAAAGGTCAGAGCGTCGATCAAGTCTACTAC
CCCAACAAAGAAGCCTTCAAGTGTGAGGAGTGTGGCAAGCACTATAACAC
CAAGCTTGGTTATAAGCGTCACGTGGCCATGCACTCCGCCACGGCGGGTG
ACCTTACTTGCAAGGTGTGCCTGCAAAGTTATGAAAGCACGCCGGCCCTC
CTGGAGCACCTAAAGAGTCACTCGGGTAAGTCGTCCGGCGGTACCAAGGA
GAAGAAGCATCCATGTGACCACTGTGATCGTCGTTTCTACACGCGCAAGG
ACGTACGACGCCATATGGTGGTACACACGGGACGCAAGGACTTCCTATGC
CAGTACTGCGCCCAGCGCTTTGGCCGCAAGGACCACCTGACACGGCATGT
CAAGAAGAGCCACTCGCAGGAGCTGCTGAAGATTAAATCGGAACCTCCGG
ATATGCTGGGTCTTTTGGGGTCAGGCTCACCACCGTGTTCTGTCAAAGAA
GAACTCAACCCTATGATGTGTAGCATGGGACCTGCCAAGGATCCCATGAT
GACCAAGCCCTTTCCCCCAGGAAACCCCTTCCCCATGGGTATGTACAACC
CGCATCAT------ATCCAGGCCATGTCCAATCCTGGAGTGGGTCAT---
------CACCACTCTTTGGTGTCTGGCTCTCTGTCCGCTGCTATGGGAAT
GAGCTGCCATATGGAGTATCTGATTTATGCCTCCTTTTCCTTTATGGGAT
GTTTACAAATCAGTGATGGCTCCAACATTGTGAACCTGTTAGCTAGCAAT
TCCCCTAGCGTCTCGTTCGCCCTGACCCAACAGAAGTACTTCAGCAACTA
CAGCCCAGTGATTGGGTTTTACATATATGAGCCCATAGAGTACTGGAACT
CCACTGTGCAGGATCATCTTAAGACTCTGGGCCAGGGTTTCAATAAGATT
TCATGGATCGACAATTATTTTCACTACCTGAAGGTAGTGAACGTTAGCGC
GTCAACCAAAAGTGATTTTATAAACATCCTCAAGAACTCTTTCCTGAGGA
GTCCTGAATATCAGCATTTCACGGACGACATCATCTTTTCCAAAA---TC
GGAG------------ACGAGTACGACATTATTGTGTCCCGGATGTACTT
GGTCGCCAGAACCACGGAAAAGACCAGAGAAGAGGTGGTGGAGCTGCTCG
AGAGACTCAGACCCCTCTCCCTCATAAACAGCATCAAATTTATTGTCTTC
AACCCCACCTTCATCTTCATGGATCGTTACAGCTCCTCTGTCATCTCCCC
TATCCTGACATCAGGCTTCAGTGTCCTGACCATCCTCATCCTAACATTTT
TCCTGGTCATTAATCCACTGGGGAACTTCTGGTTGATTCTGACCGTTACC
TCCGTGGAGCTGGGCGTTCTGGGTCTTATG--------------------
----------------------TCCAGTTCTTGGGATGTGGGCATCATTG
ATGGACTGTCTGGTTCTTCAACCTCAGTTGACGATTTCCCCGCTGACACC
ATCTCTCGACGTTTCCGCTATGATGTGGCTCTGGTTTCCGCTTTAAAAGA

CTTGGAAGAGGACATCATGGAAGGCCTGAGAGAGAAGGAACTAGATGACA
GTGCCTGCACCTCTGGCTTCACTGTTGTGATCAAGGAGTCCTGTGACGGC
ATGGGAGACGTCAGTGAGAAACATGGAGGTGGGCCGGCAGTTCCAGAGAA
AGCAGTGAGGTTCTCCTTTACAGTGATGTCTATCTCTGTCCAAGCTGAGG
GTGAAGAT------------------------------GAAGCAGTCACC
ATCTACCAAGAGCTGAAGCCCAACTCTGAACTTTCCTGCAGGCCTCTATG
CCTCATGTTTGTTGATGAGTCAGACCATGAGACCCTCACTGCAATTCTGG
GTCCTGTGGTGGCTGAACGCAAAGCCATGCAAGAGAGTCGCCTCATTCTG
TCAATTGGAGGGATGCTTCGCTCCTTCCGCTTTCACTTCCGAGGCACAGG
ATATGATGAGAAGATGGTACGGGAGCTGGAGGGCCTGGAAGCCTCAGGCT
CCACTTATATCTGTACCCTCTGCGACTCCACTCGGGCTGAGGCCTCACAC
AACATGGTGCTGCACTCCATCACCCGGAGCCATGAGGAGAACCTTGAACG
CTATGAGATCTGGAGGACCAATCCATTTTCAGAGTCTGTTGAAGAGCTCA
GAGATCGGGTCAAAGGTGTTTCTGCCAAGCCCTTTATGGAGACCCAGCCG
ACCTTAGACGCCCTTCACTGTGACATTGGCAATGCCACTGAGTTCTACAA
GATATTCCAGGATGAGATCGGCGAGATGTACCTGAAGAA---CTCC---C
CAGCACGGGAAGAGAGACGTCGTTGGCGCTCTGCCCTAGACAAACAGCTG
AGGAAGAAGATGAAGCTCAAGCCAGTCATGCGGATGAATGGAAACTACGC
TCGTCGCCTGATGACCCGGGAGGCGGTGGAGGCCGTTTGCGAGCTTGTGC
CATCACAGGAACGTCGTGAAGCCTTGAGGGAGCTCATGGAGCTATACCTT
CAGATGAAGCCAGTGTGGCGCTCCACGTGCCCCGCCCAGGAGTGTCCAGA
TCAGCTCTGCCGGTACAGTTACAACTCCCAGCAATTCGCTGAGTTGCTCT
CCACCACTTTCAAGTACCGATACGACGGTAAAATCACCAACTACCTGCAC
AAGACCTTAGCTCACGTGCCTGAGATTGTGGAAAGGGATGGATCCATTGG
TGCATGGGCTAGT------------------------
NNNNNNNNNNNNNNNNNNNNNNNNNNNNNCCGCAGTGGAAGG
AGAGCCCCCAGCCTTTCACTTGCTCTATTGAGGACCCCACCAAACAGACT
AAGTTCAAGGGCATTAAGACCTACATATCGTATCGTGTGACCCCTAGCCA
TACCACTCGACCCGTGTACCGCCGCTACAAACATTTCGACTGGTTGTATA
ATCGGCTCCTGCACAAGTTCACTGTCATCTCAGTGCCCCATCTACCCGAA
AAGCAGGCAACTGGGCGCTTTGAGGAGGACTTCATTGAGAAACGCAAAAG
GCGTCTCATCTTATGGATGAACCACATGACCAGTCACCCAGTTCTGTCAC
AGTATGAAGGTTTTGAGCACTTCCTCATGTGTGGTGATGACAAGCAGTGG
AAGCTGGGCAAGCGGAGAGCTGAGAAGGATGAGATGGTGGGTGCCCACTT
CATGCTTACCTTCCAGATCCCCAGCGAGCACCAGGATCTACAGGATGTGG
AGGAGCGTATAGACTCTTTTAAGGCCTTCGCCAAGAAAATGGATGACAGT
GTCATGCAGCTAACACATGTGGCCTCTGAGTTGGTGCGCAAGCACCTTGG
AGGCTTCCGGAAGGAGTTCCAGAGGCTTGGCAATGCTTTCCAATCCATCA
GCCAGKCCTTCCTGCTGGAANNNNNNNNNNNNNNNNNNNNNNNNNNNNNNNNNNNNNNNNNNNNNNNNNN
NNNNNNNNNNNNNTTAACCTCTCTGGGTTTCATCATCGGTGTC
GGTGTGGTTGGCAACCTCTTAATTTCCATCCTGCTCGTCAAAGACAAGAG
CCTCCACCGTGCGCCCTACTACTTCCTTTTGGACCTGTGTGCCTCTGATA
TTCTACGCTCAGCCATCTGCTTCCCCTTTGTGTTCACCTCAGTCAAAAAT
GGCTCGGCCTGGTCGTACGGCACTCTCACCTGCAAAGTGATTGCCTTCCT
GGGTGTTCTCTCGTGTTTTCATACCGCCTTCATGCTCTTCTGCATCAGTG
TCACGCGCTACCTGGCCATTGCCCATCACCGCTTCTATACCAAGAGGTTG
ACCTTCTGGACCTGCCTGGCTGTCATCTGCATGGTGTGGACACTGTCGGT
CGCTATGGCATTCCCACCGGTACTTGATGTTGGTACCTACTCGTTCATCC
GAGAGGAAGACCAGTGCACGTTTCAACACCGCTCCTTTCGTGCCAACGAC
TCGCTGGGCTTCATGCTGCTCCTTGCACTCATTCTCCTGGCCACTCAGCT
TGTCTACCTCAAGCTCATCTTCTTTGTGCACGACCGCCGGAAGATGAAGC
CTGTCCAGTTTGTGCCAGCGGTGAGCCAGAACTGGACCTTCCATGGACCG
GGAGCCAGCGGCCAGGCAGCAGCTAATTGGTTGGCTGGATTTGGCCGTGG

CCCTACCCCGCCCACCCTTTTGGGGATCCGGCAGAACAGCAACGCAGCAG
GCCGCAGGAGATTGCTGGTGTTGGATGAGTTTAAGACTGAAAAGAGGATT
AGCAGGATGTTCTACATTATGACCTTCTTCTTTCTCAGCTTGTGGGGGCC
CTACCTGGTGGCTTGCTACTGGAGGGTGTTTGCCAGGGGCCCCGTGGTCC
CTGGGGGTTACCTGACAGCCGCTGTGTGGATGAGCTTTGCCCAGGCAGGA
GTCAACCCCTTCANNNNNNNNNNNNNNNNNNNNNNNNGCCAAATCTCGCTTTCACCCTGGCGTCGGGACCG
GTC
CTGGCACGGACC---GCAGCGTCCCACTCAGTAACAGCTTGCTATCACCG
CAACAAACCGAGGAGACCGCAGTGG---CCTCCCCGCAGCGATGGTTTGT
CACCC---CTGCCAACAACCGACTGGACTTTGCCGCCTCGGCATACGATG
CCGCCGCGGCTGCTGATTTTGCCGGTAACGCGGCCACCCTTCTGTCGTAC
GCAGCTGCTGGAGTTAAGGCGC------TTCCCTTGCCCACTGCAGGCTG
CTCCAACAGACCCTTGGGTTATTACGGAGAGCCACCGG---GATGG---G
GCACGCGCACCTCACCGCAATACTGC---------AGCAAATCCAGCACA
GTTCTCGCGTGCTGGCCATCCAACTCTGTCGGAAGCAGAACGACCA---C
TTCTAATTACCTGG---CCGGAATGGAAGA---CGGG---GACCC---TA
TCGCACCTGAGAGGTCACCT---CT---CGGTGGCGCCGAAGAA---GGC
AAGCCAAAAGACCT------CTCGGA---ATCCAGCTGGATAGAA---AC
CCCATCGTCTATTAAGTCTATTGATTCAAGCGATTCTGGGATTTTTG---
AGCAAGCCAAACGGAGGAGGATTTCACCATCTGCCACACCG---------
GTTTCAGAGACGTCCTCTCCATTAAAATCTGAA----------CAGGCGA
CGTTACAGACAGAGAAGTGGCTTTGGGGATAAATCCGTTCGCCGACGGCA
TGGGCGCCTTCAAAATTAACCACAGCTCTCACGATCTTGGCTCTGG---G
CAAACGGCGTTTTCCTCGCAAGCA---CCCGGCTAT---GCAGCTGCTGC
CCTGGGA---CATCATCA------TCACCCCACTCATGTCAGCTCC---T
ACTCCACCGCGGCTTTCAATTCTACTCGGGACTTTCTTTTTCGGAATCGG
GGCTTCGGAGACGCTACCAC------------AGCACAGCACAGTCTTTT
CGCCTCAGC---CGCGGGAAGTTT---T------GCAGGGCCACATGGAC
ATTCTGATGCCGCAGGACACCTGCTCTTCCCAGGACTGCACGAG---CAA
GCAGCGACGCACGCGTCCTCGAACGTAGTGAACAGCCAAATGCGCCTGGG
GTTTTCAGGGGACATGTATGGCAGAGCTGAGCAATACGGTCACGTTACGA
GCCCCAGGT---CCGAGCATTATGCCTCCACTCAGTTGCATGGCTATGGC
CCCATGAACATGAATATGGCTGCC---CATCACGGTGCTGGGGCCTTCTT
TCGTTATATGAGACAACCGATCAAACAAGAGCTCATCTGCAAGTGGGTCG
AACCGGAGCAACTGACAAATCCGAAAAAGTCGTGCAACAAAACTTTCAGC
ACCATGCATGAACTGGTGACCCACATCACAGTGGAACATGTTGGGGGACC
AGAACAATCGAATCACATTTGTTTTTGGGAAGAGTGTTCGCGAGAAGGAA
AGCCATTTAAAGCCAAGTACAAACTTGTAAATCATATCAGAGTGCACACC
GGAGAAAAACCGTTCCCTTGCCCATTTCCGGG----------
>Chauliodus danae
AGCCTGCTAATTCGGGCAGAACTCAGCCAGCCCGGTGCATTTATGGGCGA
CGACCAGATCTACAACGTTATCGTTACAGCACATGCCTTCGTTATAATTT
TCTTCATAGTAATACCCATTATGATCGGGGGTTTCGGAAACTGGTTAATC
CCTCTAATAATTGGGGCCCCTGACATAGCCTTCCCCCGAATAAACAACAT
GAGCTTCTGACTTCTCCCTCCCTCATTTCTTCTTCTACTCGCCTCTTCCG
GGGTAGAAGCTGGGGCCGGGACCGGATGAACTGTATACCCACCCCTTTCA
GGCAACCTCGCTCACGCTGGTGCCTCTGTTGACCTTACAATTTTCTCCCT
CCACCTTGCAGGAATCTCTTCTATTCTAGGAGCAATTAATTTCATTACTA
CTATTTATAATATGAAACCCGCCGGTATATCCCAATACCAGGCTCCCCTG
TTCGTGTGATCTGTTCTCATTACTGCTGTTCTTCTCCTTCTATCCCTACC
CGTCCTAGCCGCCGGAATTACCATGCTTCTTACAGACCGAAACCTTAACA
CAACCTTCTTCGATCCTGCAGGGGGTGGAGACCCCATCCTTTACCAGCAC
CTCTTCTGATTCTTCGGCCACCCTGAGGTCTATATTCTCATTCTCCCGGG

```
CTTCGGAATAATCTCCCACATTATTGCTTACTACGCTGGTAAAAAGGAAC
CCTTCGGCTACATGGGGATGGCCTGAGCCATAATGGCAATTGGGCTTCTA
GGCTTTATCGTATGGGCCCACCACATGTTCACTGTAGGCATGGACGTAGA
TACTCGCGCCTNTTCCTGGAGAGGAACCTCCACCCGTCCAACTGCCTGGGC
ATGCTGCTTCTCTCCGACGCCCATCAGTGCGC-AAGCTGTCCGAGCTGTC
CTGGGGCATGTGCCTCAGCAACTTTCCCGCCATTTGCAAGACTGAGGACT
TCCTGCAGTTGCCCAAAAACATGGCGGTGCAGCTGCTGTCTCACGAGGAG
CTGGAGACAGAGGACGAGAGGCTGGTGTACGAGGCCGCTCTCAGCTGGGT
CAACTACGACCTGGAGAGGCGGCACTGCCACCTGCCAGAGCTGCTGAGAA
CCGTCCGTCTGGCCTTGCTGCCCGCCATCTTCCTCATGGAGAACGTCTCC
ACAGAGGAGCTGATCAACGCCCAGGCCAAGAGCAAGGAGGTGGTGGACGA
GGCCATCCGCTGCAAGCTGAGGATCCTGCAGAACGAGGGTGTGGTCACCA
GCCCGCTGGCCAGGCCCAGGAAGACCAGCCACGCCCTCTTCCTGCTGGGC
GGCCAGACCTTCATGTGCGACAAACTCTACCTGGTGGACCAGAAGGCCAA
GGAGATCATCCCCAAGGCGGACATCCCCAGCCCCAGGAAGGAGTTCAGCG
CCTGCGCCATCGGCTGCAAGGTCTACATCACCGGAGGCA--GGGGCTC-T
GAGAACGGAGTCTCCAAGGACGTCTGGGTGTACGACACGTCCCACGAGGA
GTGGTCGAAGGCGGCTCCCATGCTCATCGCCCGGTTCGGCCACGGCTCCG
CGGAGCTGAAACACTGCCTCTACGTGGTAGGCGGACACACGGCCGGAACC
GGCTGCCTCCCCGCCTCCCCCTCG--------------------------
--------------------------------------------------
--------------------------------------------------
------------------------------------CCATGCTGGTGAGCA
TGCACGGAGCGCAGCTCGTCACCTCCCTCTTCCTCCCCAGAGGAGCAGCT
GTGGTGGAACTCTTCCCCTACGCAGTCAACCCTGAACAGTACACGCCCTA
CAAGACTTTGGCCACCTTGCCCGGCATGGACCTCCAGTACGCGGCCTGGA
GGAACACTGTGGAGGAGAACTCCGTGGCCTACCCGGACAGACCCTGGGAC
CAAGGTGGCATCTCCCACCTGGACAAAGATGAGCAGGAGCGCATTCTGGC
CAGCAGCGAGGTTCCCAGGCACCTGTGCTGCCGAAACCCGGAGTGGCTCT
TCCGCATTTACCAGGACACCAGAGTGGACATCCCGTCTCTGCTAGAGGTT
CTGAG---GCAGAGCCTGAAG---ACCAAGCCCAGTTTGAAGAA---GAC
CCGGCCTGCCAGCACCGTCCATCCGGGCAGGGTCAGGGAGCCGCATTGCC
AGACCTCGGTCCAGGCGACCAACGAAGCCCAGCTCACAGTGTCCTGGCAG
ATCCCTTGGAACCTCAAGTACTTGAAGGT---------------------
----------AAGAGGGATCCGAGCAAGGGAACCTTGGAGGATCAAATCA
TCCAGGCTAACCCTGCACTGGAGGCGTTTGGTAATGCCAAAACATTGAGA
AATGACAACTCATCACGCTTTGGTAAATTCATCCGGATTCACTTCGGAAC
CACTGGCAAGTTGTCCTCTGCAGACATAGAGACTTACCTTCTGGAAAAGT
CCCGATGCACCTTCCAGCTCAAGTCAGAGAGGAACTATCATATCTTCTTC
CAGATTTTGTCCAATCAAAAGCCAGAGCTGTTGGACATGCTTTTGATCAC
CAACAATCCATATGACTACGCCTACATCTCCCAAGGAGAGGTAACGGTAG
CATCCATCAATGATTCAGAGGAGTTGATAGCCACGGACAGTGCATTCGAT
GTGCTTGGCTTTACTCAAGAGGAGAAAAATGGAGTGTACAAGCTGACAGG
TGCAATCATGCATTACGGCAACATGAAGTTCAAGCAAAAGCAGAGAGAGG
AGCAGGCAGAGCCTGACGGCACAGAGGCTGCTGACAAGTCAGCTTACCTG
ATGGGCCTGAACTCTGCCGATCTAGTGAAGGGACTCTGCCATCCCAGAGT
TAAGGTTGGCAATGAGTATGTCACAAAAGGGCAGGGTGTAGACCAAGTCT
ACTAC---------------------------------------------
--------------------------------------------------
--------------------------------------------------
--------------------------------------------------
--------------------------------------------------
--------------------------------------------------
```


---------------------TACATGATCTACGCCTCCTTCTCCTTCAT
GGGATGTTTACAAATCAGCGACGGGTCCAACGTGGTCAACCTGTTAGCCA
GCAACTCGCCCAGCGTGTCGTACGCGCTGACCCAGCAGAAGTACTTCAGC
AACTACAGCCCCGTGATCGGCTTCTACATCTACGAGCCCATCGAGTACTG
GAACTCCACGGTGCAGGAGCACCTGAGGACGCTGGGCCAAGGCTTCAACA
AAATCTCCTGGATGGACAATTACTTCCACTACCTGAAGGTGGTGAACGTG
ACCGCCGCCACCAAGAGCGACTTCATCGCCGTCCTCAAGGGCTCCTTCCT
GAAGAGCCCGGAGTACCAGCACTTCACCGAGGACATCATCTTCTCCAAGA
---AGGGCG------------AGGAGTACGACATCATCGCGTCGCGGATG
TACCTGGTGGCGCGCACCACGGAGAAGACCCGCGAGGAGGTGGTGGAGCT
GCTGGAGAGGCTCCGGCCCCTGGCGCTCATCAACAGCATCAAGTTCATCG
TCTTCAACCCCACCTTCGTCTTCATGGACCGCTACAGCTCCTCCGTGGTG
TCGCCCATCCTCACCTCGGCCTTCAGCGTGCTCACCATCCTCATCCTCAC
CTTTTTCCTGGTCATCAACCCGCTGGGGAACTTCTGGTTGATCCTGACCG
TCACCTCGGTGGAGCTGGGCGTTCTGGGTCTGATG---------------

--TCCTACAC
CATCGAGATGGGCCCCCGGGGGCCTCAGTGGAAGGAGAGCCCGCAGCCCT
TCTCCTGCTCCGTGGAGGACCCCACCAAGCCGACCAAGTTCAAGGGCATS
AAGACCTACATCTCCTACCGGGTCGCCCCCAGCCACACCGGGCGGCCCGT

CTACCGGCGGTACAAGCACTTTGACTGGCTGTACAACCGCCTGCTGGCCA
AGTTCACCGTCATCTCCGTGCCCCACCTGCCGGAGAAGCAGGCCACGGGC
CGCTTCGAGGAGGACTTCATCGAGAAGCGCAAGAGGCGGCTGGTGCTCTG
GATGAACCACATGACCAGCCACCCGGTGCTGTCCCAGTACGAGGGCTTCG
AGCACTTCCTGATGTGCGCCGACGACAAGCAGTGGAAGCTGGGCAAGCGG
CGGGCGGAGAAGGACGAGATGGTGGGCGCCCACTTCATGCTGACGTTCCA
GATCCCCAACGAGCACCAGGACCTGCAGGACGTGGAGGAGCGCGTCGACT
CCTTCAAGTCCTTCGCCAAGAAGATGGACGACAGCGTCATGCAGCTGACG
CACGTCACCTCGGAGCTGGTGAGGAAGCACCTGGGGGGGTTCCGCAAGGA
GTTCCAGCGCCTGGGCAACGGCTTCCAGTCCATCAGCCAGGCGTTCACGC
TGGACCCGCCCCACTGCTCCGAGGGCCTCAACAACGCCATCTCGCAC---

----------------------------------GCCAAATCTCGCTTTC
ACCCTGGCGTGGGGACTGGTCCTGGCACGGACC---GCAGCGTCCCACTT
AGTAACAGCTTGCTATCCCCGCAACAAACCGAGGAGCCCGCGGTTG---C
CTCCCCGCAGCGATGGTTTGTCACCC---CTGCCAACAACCGACTGGACT
TTGCCGCCTCGGCATACGATGCTGCCGCCGCTGCAGATTTCGCCGGTAAC
GCGGCCACCTTGCTGTCGTACGCGGCGGCTGGAGTGAAGGCCC------T
TCCCCTGTCCACGGCGGGCTGCTCAAACAGACCGCTCGGGTATTACGCAG
ACCCGTCAG---GCTGG---GGCACCCGCACACCACCGCAGTACTGT---
------ACCAAGTCCAGCTCGGTTCTCTCTTGCTGGCCT---AATACTGT
TGGGGGCAGAACGGGCC---CCTCCAGCTACCTGG---------CCGAGG
A---TGGA---GACGC---GCTGCCCACCGAGAGGTCTCCG---AT---A
GGCGCGTCAGACGAA---ACCAAACCAAAAGACTT------GACCGA---
ATCCAGCTGGATAGAG---ACGCCGTCTTCAATAAAGTCAATTGATTCAA
GTGATTCTGGAATCTTTG---AACAAGCAAAAAGGAGAAGAATATCTCCG
TCTGCCACACCA---------GTTTCGGAGGCTGTGTCCCCGTTGAAATC
CGAGNNNNNNN---------NNGTCACCGAAAGAGAAGAGGCTTTGGGGATAAATCCTT
TCGCAGACGGGATGGGTGCTTTCAAAATCAACCACAGCTCGCACGACCTC
GGCTCCGG---CCAAACGGGGTTTTCCTCCCAGGCG---CCCGGCTAC--
-GCGGCCGCAGCCCTCGGA---CACCACCA------CCACCCGACGCACG
TCAGCTCT---TACTCCACCGCGGCGTTCAACTCCACCCGGGACTTTCTC
TTCAGAAACCGGGGCTTCGGGGACGCCGCCAG------------CGCGCA
GCACAGTCTGTTCGCGTCCGC---AGCGGGAAGTTT---T------GCAG
GGCCACATGGACACTCCGATGCCGCGGACACCTGCTCTTCCCGGACTT

```
CACGAG---CAAGCCGCGAGCCACGCGTCCTCGAATGTGGTTAACAGCCA
GATGCGGCTGGGGTTTTCGGGGGACATGTACGGGAGAGCCGACCAGTATG
GCCACGTTACGAGCCCGCGGT---CCGACCACTACGCTTCGACCCAGTTG
CACGGCTATGGCCCTATGAACATGAATATGGCCGCG---CACCACGGAGC
CGGGGCCTTCTTCCGTTACATGAGGCAGCCGATCAAGCAAGAGCTCATCT
GCAAGTGGATCGAGCCGGAGCAACTATCGAATCCTAAAAAGTCGTGCAAC
AAAACTTTTAGCACGATGCACGAGCTGGTGACCCACTTGACGGTGGAGCA
TGTGGGGGGACCCGAGCAGTCCAACCACATTTGCTTCTGGGAAGAGTGCG
CCCGAGAAGGAAAGCCCTTCAAAGCCAAATTCAAACTTGTGAACCACATC
CGAGTGCACACCGGAGAGAAGCCGTTTCCTTGTCCGTTTCCTGGCTGTGG
CAAA
>Chaunax suttkusi
--------------------------------------------------
--------------------------------------------------
--------------------------------------------------
--------------------------------------------------
--------------------------------------------------
--------------------------------------------------
--------------------------------------------------
--------------------------------------------------
--------------------------------------------------
--------------------------------------------------
--------------------------------------------------
--------------------------------------------------
--------------------------------------------------
--------------------------------------------------
--------------------------------------------------
--------------------------------------------------
------------TTCCTAGAGCGAAACCTTCACCCATCTAACTGCCTTGG
CATGCTGTTGCTGTCTGACGCACACCAGTGCACCAAGCTGTCGGAGCTCT
CCTGGGGCATGTGCCTCAGCAACTTCCCCGCTATTTGCAAGACAGAGGAC
TTTCTCCAACTCCCAAAAGATATGGTGGTGCAGCTTTTGTCACATGAGGA
GTTAGAGACTGAAGATGAGAGACTTGTTTATGAAGCTGCCCTCAATTGGA
TCAACTATGACCTGGAAAAGAGGCACTGCCACCTTCCAGAGCTTTTGAGA
ACGGTCCGTCTGGCCCTGCTGCCCGCCATCTTTCTCATGGAGAATGTCTC
TACAGAAGAGCTGATCAATGCCCAGGCCAAGAGCAAGGAATTGGTGGATG
AAGCTATCCGCTGTAAGCTGAAGATCTTGCAGAATGATGGCGTTGTAAAC
AGCCCGTGTGCTCGACCAAGAAAAACAAGCCATGCCCTTTTTCTTCTGGG
CGGTCAGACATTCATGTGTGACAAGTTGTATCTGGTGGACCAGAAAGCCA
AAGAGATCATCCCCAAAGCTGACATTCCCAGCCCCAGGAAGGAGTTCAGC
GCCTGTGCCATCGGCTGTAAGGTGTACATCACTGGAGGGA--GAGGCTC-
AGAGAATGGTGTGTCCAAAGATGTATGGGTCTACGACACAGTTCAAGAGG
AATGGTCGAAGGCCGCACCCATGCTCATTGCAAGATTTGGCCATGGCTCT
GCAGAGCTTAAACACTGCCTCTACGTGGTAGGAGGTCACACTGCAGCAAC
TGGCTGCCTCCCAGCCTCTCCATCTGGATGAATACATTGTTGTGTTTAGT
CGTTCAACAACGAGACTGATACTGAATGAAGCGGAGCTCATCATGGCACT
GGCTCAGGAGTTCCAGATGAGAGTGGTCACAGTGTCCCTGGAGGAACAGT
CATTCACTAGTATCATCCAGGTGATCAGCAGTGCTACCATGTTAGTCAGT
ATGCATGGAGCTCAACTTATTACCTCGCTCTTCCTCCCCAGAGGAGCTGT
TGTGGTGGAGCTGTTCCCCTTTGCTGTGAACCCAGAGCAGTACACCCCAT
ATAAAACACTTGCCTCCCTTCCGGGCATGGACCTTCATTATATCTCCTGG
AGGAACACTAAGGAGGATAACACTATCACCCACCCAGACAGGCCCTGGGA
ACAAGGGGGTATCGCTCATTTGGATAATGAGGAGCAAGAACGAATACTGA
```

CGAGTAAAGATGTGCCCAGGCATCTGTGTTGCCGAAACCCAGAGTGGCTC
TTCCGGATCTATCAGGACACATTGGTGGACATTCCTTCCTTCCTGGAAGT
CCTCAA---AGAGGACATGAAG---ACAAAGCCCAACTTGAGGAA---GT
CAAAGCCGGCCAGCACACTCCACCCTGGCCGGGTCAGAGAACCTCAGTGT
CAGACTTCAGTTCAAACCAGTAATGAGGCTAAACTCACAGTCTCCTGGCA
GATACCGTGGAATCTGAANNNNNNNNNNNNNNNNNNNNNNNNNNNNNNNNNNNNNNNNNNNAAAAAAGAC
ACCAACAAGGGGACCCTGGAGGA
TCAAATCATCCAAGCAAACCCTGCATTGGAGGCTTTTGGCAACGCAAAAA
CAGCAAGAAATGATAACTCATCACGTTTTGGAAAGTTCATCCGAATTCAC
TTTGGTACAAGCGGCAAGCTGTCATCTGCTGACATCGAAACGTACCTGCT
GGAGAAGTCCCGCGTTACCTTTCAGCTGAAGGCTGAGAGAAATTATCACA
TTTTCTACCAGATCCTGTCAAATCAGAAACCAGAGCTGCTGGACATGCTG
CTGATCACCAACAATCCCTATGACTATTCCTACATCTCCCAAGGAGAGGT
AACTGTCGCCTCCATCAACGACTCAGAAGAGCTGATGGCCACGGACAGCG
CCTTCGATGTGCTCGGCTTCACTCCAGAGGAGAAGATGGGCGTCTATAAA
CTCACGGGTGCCATCATGCACTATGGCAACATGAAATTCAAACAAAAGCA
GCGTGAGGAGCAGGCTGAGCCAGATGGGACGGAGGCTGCTGATAAATCAG
CTTACCTAATGGGGCTGAACTCTGCTGACCTCATCAAAGGGCTGTGTCAT
CCCAGAGTCAAGGTAGGAAATGAATANNNNNNNNNNNNNNNNNNNNNNNNNNNNNNNNNNNNNCCCAACA
AGGAGGCCTTCAAATGT
GATGAGTGTGGGAAGCACTACAACACCAAGCTGGGATATAAGCGCCATGT
TGCCATGCATTCAGCCACGGCGGGGGATCTCACCTGTAAAGTGTGCATGC
AGACCTACGACAGCACACCCATGCTCTTGGAGCACCTGAAGAGCCACTCT
GGGAAGTCCTCAGGTGGCACCAAGGAGAAAAAACACCAGTGCGACCACTG
TGACCGGCGTTTCTACACAAGGAAGGACGTGAGACGGCACATGGTTGTCC
ACACGGGCCGAAAGGACTTCCTCTGCCAGTACTGTGCGCAACGCTTTGGC
AGGAAGGACCATCTGACACGCCATGTGAAGAAGAGCCACTCACAGGAGCT
GCTGAAAATCAAGACAGAGCCTCCTGATATGTTAGGTCTTTTAGCTTCTG
GGTCACCACCGTGCTCTGTGAAGGAGGAGCTCAGCCCCATGATGTGCGGT
ATGGGCCCCAACAAAGACCCCATGATGAGCAAACCCTTTCCGAGTGGGGC
CCCTTTTCCAATGGGCATGTACAACCCTCATCAT------CTCCAGGCCA
TGTCTAATTCTGGGGTAGGTCACTCA------
CACCCGTCCCNNNNNNNNNNNNNNNNNNNNNNNNNNNNNNNNNNNNNNNNNNNNNNNNNNNNNNNNNNNN
NNNNNNNNNNNTCATTCAT
GGGATGTTTACAGATCAGTGATGGATCAAATATCGTGAACCTGCTGGCGA
GTAACTCTCCAAGTGTTTCATATGCTCTGACCCAGCAAAAATACTTCAGT
AACTATAGTCCAGTCATTGGGTTTTACATTTATGAGCCCATCGAGTACTG
GAACTCCACAGTGCAGGAGCACCTGAAGACTCTGAGTCATGGCTTCAATA
AGATCTCCTGGATAGACAACTTTTTCCACTACCTGCGGGTGGTGAATGTG
AGTGCATCAACCAAGAGCGACTTCATCACCATCCTCAAGGGCTCCTTCCT
GCGCAGCCCGGAGTACCAGCACTTCACCGAGGACATCATTTTCTCAAAGA
---ACCGTGAGACCG------ATGAGTACGACATCATCGCCTCGCGGATG
TACTTGGTGGCACGGACCACGGAGAAGAAGCGTGAAGAAGTGGTGGAGCT
CCTGGAAAAACTTCGTCCACTGATGCTGATCAACAGCATCAAGTTCATTG
CCTTCAATCCTACGTTTGTGTTCATGGACCGCTACAGCTCCTCCGTCATC
TCACCCATCCTGACGTCAGGATTCAGCGTACTCACAATCCTCATCCTCAC
TTTCNNNNNNNNNNNNNNNNNNNNNNNNNNNNNNNNNNNNNNNNNNNNNNNNNNNNNNNNNNNNNNNNNN
NNNNNNNNNNNNNNNNGGTTTCACCAGTTTGAGTGGCAGCCAGTTCTCAAGAATGTGTCTA
CATCTTGCAATGTTGGCATTATTAATGGTCTCTCTGGATGGGATTCCTCG
GTGGATGACTTCCCAGCTGACACCAYCACTCGACGGTTTCGCTATGATGT
GGCACTAGTATCAGCATTAAAGGATCTGGAGGAGGACATCATGGAGGGAC
TGAGAGAGAATGGAATGGAAGACAATGCTCACA------ACTTCACTGTT
ATGATCAAAGAATCCTGTGATGGCATGGGCGATGTCAATGAGAAGCATGG

TGGAGGACCAGCTGTCCCTGAGAAGGCTGTGCGTTTCTCTTTCACTGTTA
TGTCTGTCTCTTTCGTAGCAGACAGTCAGGAG------------------
-------------GAAGAGGTCGCCATCTTCACAGAGCCAAAGCCAAACTC
AGAACTGTCCTGTAAGCCCCTTTGCCTGATGTTTGTGGATGAATCMAACC
ATGAGACACTCACAGCCATCCTGTGTCCTTTAATTGCAGAGCGTAACGCA
ATGAAAGAGAGCAAGCTCATCCTGTCCATCGGTGGACTTCTTCGCTCCTT
CTGCTTTCACTTTAGAGGGACAGGATATGATGAGAAGATGGTGCGTGAGA
TGGAAGGCTTTGAGGCCTCTGGGTCCACCTACATCTGCACTCTCTGTGAC
TCCAGTCGTGCAGAGGCCTCTCAAAACATGGTGCTGCACTCCATCACCCG
CAGTCATGAAGAGAACCTAGAACGTTATGAAATGTGGAGAACCAACCCAT
TTTCTGAGTCTGTAGATGAGTTGCGAGATAGAGTCAAAGGAGTCTCTGCC
AAGCCCTTCATGGAGACCCATCCCACACTGGATGCCTTACACTGTGACAT
CGGCAATGCCACTGAGATGTACAAAATATTCCAAGATGAGATTGGGGAGG
TGTACAAAAAGGT---CAAT---CCCAGCCGGGAGGAGCGGCGGGGCTGG
AGGACAGCCTTAGATAAACAGCTGAGGAAGAGGATGAAGCTCAAACCAGT
AATGAGGATGAATGGGAACTATGCCCGCAGGCTAATGACCCTGGAGACTG
TGGAGGTGGTGTGTGAGCTGGTGCCCTCGGAGGAGAGGAGAGAGGCCCTG
AGGGAGCTTATGAAACTCTACATCCAGATGAAGCCTGTGTGGCGCGCCAC
CTGTCCAGCCAAGGAGTGCCCTGACCAGCTGTGCCGCTACAGCTTTAACT
CCCAGTGCTTTGCTCAGGTCCTCTCCTCTAAGTTCAAATATAGATACAAT
GGAAAGATAACCAATTACCTGCATAAGACTCTGGCTCATGTGCCTGAAAT
CATAGACAGAGATGGATCCATAGGAGCCTGGGCCAGCGANNNNNNNNNNNNNNNNNNNNNTCTTACACC
AT
CGAGATGGGTCCCTTGGGGCCCCGATGGAAGGAGAGCCCACAGCCGTTCT
CCTGCTCCATTGAAGACCCCACAAAACAGACAAAGTACAAGGGCATCAAG
ACGTACATTTCGTACCGGGTGACACCGAGCCACACTGGGCATCCTGTCTA
CAGACGCTACAAACACTTTGACTGGCTGTACAACCGCTTACTGCACAAGT
TCACTGTGATCTCCGTGCCTCACCTGCCTGAGAAGCAGGCCACGGGGCGA
TTTGAGGAAGACTTCATCGAGAAGCGCAAGAGGCGACTGATACTGTGGAT
GAACCACATGACCAGTCACCCAGTCCTCTCCCAGTATGAAGGGTTCGAGC
ACTTTCTGATGTGTGCTGATGACAAGCAGTGGAAACTGGGTAAGAGACGG
GCAGAAAAAGACGAGATGGTGGGTGCCCATTTCATGCTGACCCTTCAGAT
CCCTAACGAGCACCAGGACCTTCAGGATGTTGAGGAGCGGATCGACTCCT
TCAAGTCCTTTGCTAAGAAGATGGATGACAGTGTGATGCAGCTCACACAT
GTGGCCTCAGAGCTGGTGCGTAAGCACCTGGGTGGGTTCAGGAAGGAGTT
CCAGAGGCTGGGAAATGCCTTCCAGTCAATCAGCCAGGCATTCATGCTGG
ACCCTCCCCACAGCTCACAGAACTTAAACAACGCCATCTCCCATNNNNNNNNNNNNNNNNNNNNNNNNN
NNNNNTGGGTT
TCATCATCGGAGTCGGTGTGGTTGGAAACCTCCTGATCTCCATCCTGCTG
GTCAAAGACAAGAGCCTGCACCGAGCGCCCTACTATTTCCTGCTGGACCT
GTGCGCCTCTGACATCCTGCGCTCCGCCATCTGCTTCCCCTTTGTCTTCA
CCTCGGTCAAGAATGGATCTGCCTGGACCTATGGCACGCTCACCTGCAAA
GTGATCGCCTTCCTGGGTGTGCTCTCCTGTTTCCACACGGCGTTTATGCT
ATTCTGCGTGAGCGTCACGCGCTACCTGGCCATAGCGCATCACCGTTTCT
ACACCAAGAGGCTGACCTTCTGGACCTGTCTAGCCGTCATCTGCATGGTG
TGGACGTTGTCGGTGGCTATGGCGTTCCCGCCAGTGCTAGACGTAGGGAC
GTACTCGTTTATCCGGGAGGAGGACCAGTGCACGTTCCAGCACCGCTCGT
TCAGGGCGAATGATTCGCTGGGCTTCATGCTCCTGCTGGCGCTCATCCTC
CTGGCCACACAGCTGGTTTACCTCAAGCTCATCTTCTTCGTCCACGACCG
TCGAAAGATGAAGCCTGTCCAGTTCGTGCCTGCTGTCAGCCAGAATTGGA
CCTTCCACGGGCCAGGCGCCAGCGGGCAGGCAGCGGCCAACTGGCTGGCA
GGATTCGGTCGAGGCCCCACCCCACCTACTTTGCTGGGCATTCGGCAGAA
CAGCAACGCAGCGGGCCGCAGGCGTCTACTGGTATTGGATGAGTTCAAAA

CAGAGAAGAGGATTAGTAGGATGTTCTACATCATGACGTTTTTCTTCCTG
GCGCTGTGGGGGCCCTATCTGGTTGCTTGCTACTGGCGGGTGTTTGCAAG
GGGCCCCGTGGTCCCTGGGGGCTACNNNNNNNNNNNNNNNNNNNNNNNNNNNNNNNNNNNNNNNNNNNNN
NNNNNNNNNNNNNNNNNNNNNNNNNNNNNNNNNNNATCTCGCTTTCACCCTGGCGTGGGG
ACTGGTCCTGGCACGGAGC---GCAGCGTCCCACTCGGCAACAGCTTGCT
ATCCCCGCAGCAAACCGAGGAGCCCACTGTTGCCACCCCCCCGCAGCGAT
GGTTTGTCACCC---CTGCCAACAACCGACTGGACTTTGCTGCCTCGGCA
TACGACGCCGCC---------GATTTCGCCGGTAACGCGGCCACCTTGCT
GTCCTACGCAGCGGCCGGAGTGAAGGCTC------TTCCCCTGCCGGCTG
CAGGCTGCTCCAATCGGCCTCTTGGCTATTACGCAGACCCGTCAG---GC
TGG---GGAGGACGCACGCCGCCTCAGTACTGTGGCGTGAATAGCAAATC
CAGCTCGGTCTTTTCCTGCTGGCCCTCTAACACTATCGGAGGCAGAGTGG
GGA---CC---AACTACCTGG---------CCGAGGA---GGGA---GAC
TC---AATCCCCACAGAGAGGTCACCG---AT---CGGCGGCTCGGAGGA
G---GCCAAACCCAAAGATATCAC---ATCAGA---GTCGAACTGGATAG
AG---ACGCCGTCATCTATTAAGTCCATCGATTCTAGCGACTCTGGGATC
TTTG---AACAGGCCAAAAGGAGACGGATCTCACCTTGTCCTNNNNNN--------
-
NNNNNNNNNNNNNNNNNNNNNNNNNNNNNNNNNNNNNNNNNNNNNNNNNAAGTCACAGAGAGAGAAGTGG
CGCTGGGGATAAATCCGTTCGCGGATGG
GATGGGCGCCTTCAAAATAAACCACAGCTCCCACGATATTGGCTCCGG--
-ACAAACGGCGTTTTCCTCCCAGACG---CCCGGCTAT---GCAGCAGCC
GCCCTGGGA---CACCATCA------CCACCCGACCCACGTTGGCTCT--
-TACTCCACGGCTGCTTTCAACTCCACCAGGGACTTTCTCTTCAGAAATC
GAGGATTCGGGGACGCCACCGG------------AGCGCAGCACAGTTTG
TTCGCCTC---------CGGGAGTTT---C------GCAGGGCCACACGG
ACACTCAGATGCAGCAGGGCACCTGCTCTTCCCCGGGCTCCACGAG---C
AAGCGGCGAGCCATGCGTCTTCCAACGTGGTCAACAGCCAGATGCGGCTG
GGCTTCTCGGGGGACATGTACGGACGGGCCGACCAGTATGGCCACGTTAC
AAGCCCGCGGT---CCGACCACTATGCTTCGACCCAGTTGCACGGCTACG
GCCCTATGAACATGAATATGGCCGCA---CACCACGGAGCAGGGGCCTTC
TTTCGATACATGAGGCAGCCGATCAAACAAGAGCTCATCTGCAAGTGGAT
CGAGCCGGAGCAGCTGACGAATCCCAAAAAGTCGTGCAACAAAACTTTTA
GCACGATGCACGAGCTCGTGACCCATCTGACGGTGGAGCATGTTGGNNNNNNNNNNNNNNNNNNNNNNNN
NNNNNNNNNNNNNNNNNNNNNNNNNNNNNNNNNNNNNNNNNNNNNNNNNNNNNNNNNNNNNNNNNNNNNN
NNNNNNNNNNNNNNNNNNNNNNNNNNNNNNNNNNNNNNNNNNNNNNNNNNNNNN
>Chelmon rostratus
AGCCTACTTATTCGAGCAGAACTGAGTCAACCAGGAGCTCTTTTAGGGGA
TGACCAGATTTATAATGTTGTTGTAACAGCACATGCGTTTGTAATGATTT
TCTTTATAGTAATACCAATTATGATTGGCGGCTTCGGAAACTGACTTATT
CCACTAATAATTGGAGCCCCCGATATGGCCTTCCCACGAATAAATAACAT
AAGCTTCTGGCTTCTCCCACCCTCCTTTTTCCTACTACTTGCCTCTTCTG
GCGTAGAGGCTGGGGCCGGCACTGGCTGAACTGTTTACCCCCCCATTAGCA
GGCAACCTCGCACACGCGGGAGCTTCTGTTGACCTGACCATTTTCTCCCT
TCATTTAGCAGGAATTTCTTCCATTCTCGGTGCCATTAACTTTATTACTA
CAATTATTAATATAAAACCCCCTGCTATAACTCAATATCAAACCCCTTTA
TTTGTATGATCCGTTCTTATCACAGCCGTTTTACTCCTGCTGTCCCTTCC
TGTACTTGCCGCTGGGATTACCATGTTACTAACAGATCGTAACCTGAATA
CAACCTTCTTCGATCCTGCGGGGGGAGGAGATCCTATTCTGTACCAACAT
CTT---

------------TTCCTAGAGAGAAACCTTCACCCATCTAACTGCCTTGG
CATGCTGTTGCTGTCTGATGCCCACCAGTGCACCAAGCTGTCAGAGCTCT
CCTGGGGCATGTGCCTCAGCAACTTTCCCGCTATTTGCAAGACAGAGGAC
TTCCTCCAACTGCCCAAAGATATGGTGGTGCAGCTTTTGTCACATGAGGA
GCTAGAGACAGAAGATGAGAGACTGGTTTATGAAGCTGCCCTGAACTGGA
TCAACTATGACCTGGAAAAGAGGCACTGCCACCTTCCAGAGCTCCTGAGA
ACGGTCCGTCTGGCCCTGCTGCCTGCCATCTTTCTCATGGAGAACGTATC
TACAGAAGAGCTGATCAACGCCCAGGCCAAGAGCAAGGAGCTGGTGGATG
AAGCTATCCGCTGTAAGCTAAAGATCCTGCAGAATGATGGCGTTGTTAAC
AGCCCGTGTGCCCGACCAAGAAAAACCAGCCATGCCCTCTTTCTCCTGGG
AGGACAGACTTTCATGTGTGACAAGTTGTACCTGGTGGACCAGAAAGCCA
AAGAGATCATCCCCAAGGCTGACATCCCCAGCCCCAGGAAGGAGTTCAGC
GCYTGCGCCATCGGATGTAAGGTGTACATCACTGGTGGAA--GAGGCTC-
AGAGAATGGTGTGTCCAAAGATGTATGGGTCTACGACACAGTCCACGAGG
AATGGTCGAAGGCGGCGCCCATGCTCATCGCCAGGTTTGGCCACGGCTCT
GCGGAACTGAAACACTGCCTCTACGTGGTGGGAGGTCACACGGCTGCAAC
TGGCTGCCTCCCGGCTTCCCCGTCTGGATGACTACATTGTGGTGTTTAGT
CGCTCAACAACGAGGCTGATACTGAATGAAGCAGAGTTAATCATGGCGCT
AGCCCAAGAGTTCCAGATGAGAGTGGTCACGGTGTCTCTGGAGGAACAGT
CTTTCCCCAGTATAGTCCAGGTGATCAGCGCTGCCACCATGTTGGTCAGT
ATGCATGGAGCTCAGCTCATCACCTCACTCTTCCTTCCAAGAGGAGCTGT
TGTGGTGGAGCTGTTCCCTTTTGCTGTAAACCCAGAGCAGTACACTCCGT
ATAAAACCCTTGCCTCCCTCCCAGGCATGGACCTTCACTATGTCTCTTGG
AGGAACACCAAGGAGGAGAATACCATCACGCATCCAGACAGACCCTGGGA
ACAAGGGGGCATCGCTCACTTGGAGAAGGAGGAGCAAGAGCGAATACTGT
TGAGCAAAGATGTCCCCAGACACCTGTGCTGCCGCAACCCAGAGTGGCTC
TTCCGGATCTACCAGGACACTTTGGTAGATATCCCTTCCTTCCTGGAAGT
CGTCAA---AGAGGGCATGAAG---ACAAAGCCCAACTTGAAGAA---GT
CAAAGCCGGCCAACACAGTCCACCCGGGCCGGGTCAGAGAACCCCAGTGT
CAAACCTCAGTACAAACCACTAATGAGGCTAAACTCACAGTCTCCTGGCA
GATCCCGTGGAATCTGAAATACCTTAAGGTCAGAGAGGTGAAATACGAGG
TGTGGATCCAGAAAAAAGATACCAGCAAGGGGACTCTGGAGGATCAAATC
ATCCAGGCAAACCCTGCGTTGGAGGCTTTCGGCAATGCCAAAACGTTGAG
AAATGACAACTCATCTCGGTTTGGAAAATTCATCCGAATTCACTTTGGTA
CGAGCGGCAAGCTGTCGTCTGCCGACATTGAGACATACCTGCTGGAGAAG
TCACGGGTCACCTTTCAGCTCAAGGCTGAGAGGAATTACCACATCTTCTA
CCAGATCCTGTCCAATCAGAAGCCAGAGCTGCTCGACATGCTGCTGATCA
CCAACAACCCGTACGACTACTCCTACATCTCCCAAGGAGAAGTGACGGTC
GCCTCCATCAACGACTCAGAGGAGCTGATGGCCACGGACAGCGCCTTTGA
TGTGCTCGGCTTCACTCCGGATGAGAAGATGGGTGTCTATAAACTGACAG
GCGCCATCATGCACTATGGCAACATGAAGTTCAAACAGAAGCAGCGTGAG
GAGCAGGCTGAGCCAGATGGGACGGAGGCTGCTGATAAATCAGCTTACCT
AATGGGTCTGAACTCTGCCGACCTCATCAAAGGGCTGTGCCACCCCAGAG
TCAAGGTAGGAAATGAATATGTCACCAAAGGCCAAAGTGTAGACCAAGTC
TACTACCCCAACAAGGAGGCCTTCAAGTGTGAGGAGTGTGGGAAGCACTA
CAACACGAAGCTGGGATATAAGCGGCATGTGGCCATGCACTCTGCCACGG
CAGGGGATCTCACCTGTAAAGTGTGCATGCAGACCTATGAGAGCACGCCT
GTGCTCCTGGAGCACCTCAAGAGCCACTCCGGGAAGTCTTCAGGTGGCAC
CAAGGAGAAAAAGCACCCGTGTGACCACTGTGACCGTCGTTTCTACACAC
GGAAGGATGTGAGACGGCACATGGTGGTCCACACGGGCCGAAAGGACTTC
CTGTGCCAGTACTGTGCCCAACGCTTTGGCAGAAAGGACCATCTGACTCG
CCACGTGAAGAAGAGCCACTCACAGGAGCTGCTGAAAATCAAGACGGAGC
CTCCGGATATGTTAGGGCTTTTAGCTTCTGGGTCACCACCTTGCTCCGTG

AAGGAGGAGCTCAGCCCCATGATGTGTGGCATGGGTCCCAACAAAGACCC
CATGATGGGCAAGCCGTTCCCCAGTGGAGCCCCTTTTCAGATGGGCATGT
ACAACCCCCACCAT------CTCCAGGCCATGTCTAATTCTGGGGTGGGT
CACCCA------CACCCGTCCCTGATGCCCGGTCCCTTGTCTGCAGCTAT
GGGCATGGGTTGTCACATGGAATATCTCATCTACGCCTCCTTCTCGTTCA
TGGGATGTTTACAAATCAGTGACGGGTCAAATATCGTGAACCTGCTGGCG
AGTAACTCTCCGAGCGTTTCGTATGCTCTGACCCAGCAAAAATACTTCAG
TAACTACAGTCCTGTGATTGGGTTTTACATTTATGAGCCCATAGAGTACT
GGAACTCCACAGTGCAGGAGCACCTGAAGACTCTGAGTCATGGCTTCAAC
AAGATCTCCTGGATGGACAACTTTTTCCACTACCTGCGGGTGGTGAATGT
GAGTGCGTCAACGAAGAGCGACTTCATCACCATCCTCAAGGGCTCCTTCC
TGCGCAGCCCGGAGTACCAGCACTTCACCGAGGACATCATATTCTCCAAG
A---ACCGGGAGACCG------ATGAGTACGACATTATCGCCTCGCGGAT
GTACTTGGTTGCACGGACGACAGAGAAGAAGCGCGAGGAGGTGGTGGAGC
TGCTGGAGAAGCTTCGTCCGCTGATGCTGATCAACAGCATCAAATTCATC
GCCTTCAATCCTACGTTTGTGTTCATGGACCGGTACAGCTCCTCTGTCAT
CTCGCCCATCCTGACCTCAGGATTCAGCGTACTCACAATCCTCATCCTCA
CTTTCTTCCTGGTCATCAACCCCTTGGGGAACTTCTGGCTCATCCTCACT
GTAACGTCCGTGGAGCTGGGTGTCTTGGGTTTGATGGGCTTTCACCAGTT
TGAATGGCAGCCTGCTCTCAAAAATGTGTCTGCATCTTGCAATGTTGGCA
TTATTAATGGGCTCTCTGGATGGGCTTCCTCAGTGGATGACTCCCTGGCT
GACACTATCACTCGGCGGTTTCGCTATGATGTAGCACTGGTGTCAGCATT
AAAAGATCTGGAGGAGGACATCATGGAGGGGCTGAGAGAGAGTGGGATGG
AAGACAGCGCTTGCACCTCAGGCTTCAGTGTCATGATCAAGGAATCTTGT
GATGGCATGGGTGATGTCAGTGAGAAGCACGGTGGAGGACCAGTTGTTCC
TGAGAAGGCTGTACGTTTCTCTTTCACTATTATGTCTGTCTCTATCCTGG
CAGCTGACAAGGAG------------------------------GAAGAG
GTTACCATCTTCATGGAGCCAAAGCCAAACTCAGAATTGTCCTGTAAGCC
CCTTTGCCTGACATTTGTGGATGAGTCAGACCATGAGACGCTCACAGCCA
TCCTGCGGCCTATAGTCGCAGAGCGTAATGCAATGAAAGAGAGCAGACTC
ATCCTATCCATCGGTGGACTGCTTCGCTCCTTCCGCTTTCACTTCAGAGG
TACAGGGTACGATGAGAAGATGGTGCGTGAGATGGAGGGCCTTGAGGCCT
CACGGTCCACCTATGTCTGCACTCTGTGTGACTCTGGTCGGGCAGAAGCT
TCTGAAAACATGGTGCTACACTCCATCACTCGCAGCCATGAAGAGAACCT
AGAACGTTACGAAATATGGAGAACCAACCCCTTTTCTGAGTCTGCAGATG
AGCTGCGAGACAGAGTCAAAGGGGTCTCCGCCAAGCCCTTCATGGAGACC
CATCCCACAATGGATGCATTACATTGCGACATAGGCAATGCCACTGAGTT
CTACAAAATCTTCCAGGATGAGATCGGGGAGGTGTACCAAAAGGC---CA
AC---CCCAGCCGGGAGGCGCGGCGCAGCTGGAGGGCAGCCCTAGATAAA
CAGCTGAGGAACAAGGTGAAGCTGAAACCAGTAATGAGGATGAATGGGAA
CTTTGCCCGCCGGCTAATGACCCTGGAGGCTGTGGAGGCAGTGTGTGAGC
TGGTGCCCTCTGAGGCGAGGAGGGAGGCCCTGAGGGAGCTTATGAGGCTT
TACCTCCAGATGAAGCCTGTGTGGCGCGCCACCTGCCCAGCCAAGGAGTG
CCCCGACCAGCTGTGCCGCTACAGCTTTAATTCCCAGCGCTTTGCTGACC
TCCTCTCCTCTACCTTCAAATATAGGTACAATGGAAAGATACCCAATTAC
CTGCACAAGACCTTGGCCCATGTGCCTGAAATAATAGAGAGAGAGATGGATC
CATAGGAGCCTGGGCCAGCGAGGGCAACGAGTCGGCAAACAAATCGTACA
CCATCGAGATGGGTCCCTTAGGGCCCCGGTGGAAGGAGAGCCCACAGCCT
TTCTCCTGTTCCATTGAAGACCCCACTAAGCAAACAAAGTTCAAGGGCAT
CAAGACGTACATTTCGTACCGTGTCACGCCGAGCCACACTGGGCATCCTG
TATACAGGCGCTACAAACACTTTGACTGGCTGTACAACCGCTTACTGCAC
AAGTTCACTGTGATCTCTGTGCCTCACCTGCCCGAGAAGCAGGCCACGGG
GCGATTTGAGGAAGACTTCATCGAGAAGCGCAAGAGGCGACTGATACTGT

GGATGAACCACATGACCAGTCACCCAGTCCTCTCCCAGTATGAAGGCTTC
GAGCACTTTCTGATGTGTGCTGATGACAAGCAGTGGAAACTGGGCAAGAG
ACGGGCGGAGAAGGACGAGATGGTGGGTGCCCATTTCATGCTGACCCTCC
AGATCCCTAACGAGCACCAGGACCTTCAGGATGTAGAGGAGCGGGTGGAC
TCCTTCAAGGCCTTCGCTAAAAAAATGGATGACAGCGTGATGCAGCTCAC
GCATGTTGCCTCGGAGCTGGTGCGTAAGCACCTCGGTGGGTTTAGGAAAG
AGTTCCAGCGACTGGGGAATGCCTTCCAGTCTATCAGCCAGGCGTTCATG
CTGGACCCTCCCCACTGCTCAGAGACCTTCAACAACGCCATCTCCCATNNNNNNNNNNNNNNNNNNNNAC
TGACCTCTCTGGGTTTCATCATTGGAGTCGGTGTGGTTGGAAACCTCCTG
ATCTCCATCCTGCTGGTCAAAGACAAGAGCCTGCACAGAGCGCCCTACTA
CTTCCTGTTGGACCTGTGCGCCTCTGATATCCTCCGCTCCGCCATCTGCT
TCCCCTTCGTCTTCACCTCGGTCAAGAATGGATCTGCCTGGACCTATGGC
ACGCTGACCTGTAAGGTGATCGCCTTTCTGGGGGTCCTCTCCTGTTTCCA
CACGGCGTTTATGCTGTTCTGTGTCAGTGTCACGCGCTACCTGGCCATCG
CACATCACCGTTTCTACACCAAGAGGCTGACCTTCTGGACCTGTCTCGCC
GTCATCTGCATGGTGTGGACGTTGTCAGTGGCTATGGCATTTCCGCCGGT
GCTAGACGTGGGGACGTACTCTTTCATCCGCGAGGAGGACCAGTGCACGT
TCCAGCACCGTTCCTTCAGGGCGAACGATTCGCTGGGCTTCATGCTCCTG
CTGGCGCTCATCCTCCTGGCCACACAGCTGGTTTACCTCAAGCTCATCTT
CTTCGTCCACGACCGTCGAAAGATGAAGCCTGTCCAGTTCGTGCCTGCCG
TCAGCCAGAACTGGACCTTCCACGGGCCAGGCGCCAGCGGGCAGGCGGCG
GCCAACTGGCTGGCGGGATTCGGTCGAGGCCCCACCCCGCCTACTTTGCT
GGGCATCCGGCAGAACAGCAACGCTGCGGGCCGCAGGCGTCTGCTGGTAT
TGGATGAATTCAAAACAGAGAAGAGGATTAGTAGGATGTTCTACATCATG
ACGTTTTTCTTCCTGGCACTGTGGGGGCCCTATCTGGTCGCCTGCTACTG
GCGGGTGTTTGCAAGGGGCCCCGTGGTCCCCGGGGGCTACCTGACGGCAG
CCGTGTGGATGAGCTTTGCCCAGGCTGGGGTCAATCCNNNNNNNNNNNNNNNNNNNNNNNNNNNNNNNN
NNCTCGCTTTCACCC
TGGCATGGGGACTGGTCCTGGCACGGAGC---GCAGCGTCCCACTCGGCA
ACAGCTTGCTATCCCCGCAGCAAACCGAGGAGCCCACTGTTGCCACCCCC
CCGCAGCGATGGTTTGTCACCC---CTGCCAACAACCGACTGGACTTTGC
TGCCTCGGCATACGACGCCGCT---------GATTTCGCCGGTAACGCGG
CCACCTTGCTGTCCTACGCAGCGGCCGGAGTGAAGGCTC------TTCCC
CTGCCGACTGCAGGCTGCTCCAACCGGCCTCTTGGCTATTACGCAGACCC
GTCAG---GCTGG---GGAGGACGCACGCCACCGCAGTACTGCGGCGTGA
ACAGCAAATCCAGCTCGGTCTTTTCCTGCTGGCCCACTAACTCTATCGGT
GGCAGAGCGGGCA---CC---AACTACCTGG---------CCGAGGA---
GGGA---GACTC---CATCCCGACGGAGCGGTCACCG---AT---CGGCG
GCTCGGAGGAG---ACCAAACCCAAAGACATGAC---ATCAGA---GTCG
AACTGGATAGAG---ACGCCGTCCTCCATTAAGTCCATCGACTCGAGCGA
TTCTGGCATCTTTG---AACAGGCCAAAAGGAGACGGATCTCACCTTCTG
CCACGCCG---------GTTTCAGAGACAGTGTCCCCGTTAAAATCAGAG
NNNNNNNNNNNNNNNNNNNNNNNNNNNNNNNNNNNNNNNNNNNNNNNNNNNNNNNNNNNNNNNNNNNNNN
NNNNNNNNNNNNNNNNNCCACAGCACCCACGATATTGGCTCCGG---ACAGACGGCGTTTTCCTCCC
AGGCG---CCCGGCTAC---GCAGCAGCCGCCCTGGGA---CACCATCA-
-----CCACCCGACCCACGTTGGCTCT---TACTCCACGGCGGCTTTCAA
CTCCACCAGGGACTTTCTCTTCAGAAATCGGGGATTCGGGGACGCCACCG
G------------CGCGCAGCACAGTTTGTTCGCCTC---------CGGA
AGTTT---C------GCAGGCCCACATGGACACTCAGATGCAGCGGGGCA
CCTGCTCTTCCCGGGGCTCCACGAG---CAAGCGGCGAGCCATGCGTCTT
CCAACGTGGTCAACAGCCAGATGCGGCTGGGCTTCTCGGGGGACATGTAC
GGACGGGCCGACCAGTACGGCCACGTTACAAGCCCGCGGT---CCGACCA
CTACGCTTCGACCCAGCTGCACGGCTACGGCCCCATGAACATGAATATGG

CCGCA---CACCACGGAGCAGGGGCCTTCTTTCGATACATGAGGCAGCCG
ATCAAGCAAGAGCTCATCTGCAAGTGGATCGAGCCGGAGCAGCTGACAAA
CCCCAAAAAGTCGTGCAACAAAACTTTCAGCACGATGCACGAGCTGGTGA
CCCATCTGACGGTGGAGCATGTGGGGGACCGGAGCAGACCAACCACGTC
TGCTTCTGGGAGGATTGCGCCCGAGAAGGAAAGCCATTCAAAGCCAAATA
CAAACTTGTGAATCATATCAAAGTACACACCGGAGAAAAGCCCTTCCCGT
GTCCGTTTCCCGGNNNNNNNNNN
>Chiasmodon sp
AGCCTTCTTATCCGAGCTGAACTCAGCCAACCAGGCGCCCTACTTGGGGA
TGACCAAATCTATAATGTTATCGTCACAGCCCACGCCTTCGTAATAATTT
TCTTTATAGTAATACCAATCATAATTGGAGGATTCGGGAACTGGTTAATC
CCACTTATGATCGGGGCCCCCGATATAGCCTTCCCTCGAATGAACAACAT
AAGCTTCTGACTTTTACCCCCTTCGTTCCTTCTCCTTCTCGCTTCTTCCG
GAGTTGAAGCCGGTGCCGGAACCGGGTGAACAGTCTACCCCCCTTTAGCC
GGTAATTTAGCCCATGCCGGAGCATCCGTTGATTTAACTATTTTTTCTCT
TCATCTGGCAGGTATCTCATCAATCCTGGGGGCCATCAATTTTATCACGA
CAATTATTAACATAAAACCTGCAGCTATCTCGCAGTACCAAACTCCCTTA
TTTGTTTGGGCCGTCTTAATTACAGCTGTTCTTCTTCTACTCTCTCTCCC
AGTTCTTGCCGCAGGCATTACAATACTTCTAACAGACCGAAACCTAAATA
CAACCTTTTTTGATCCGGCAGGAGGGGGAGACCCAATTCTTTATCAACAC
TTATTTTGATTCTTC-----------------------------------

------------NNNNNNNNNAGAAACCTTCACCCGACTAACTGCCTCGGCATGCTGTT
GCTGTCTGACGCCCACCAGTGCACCAAGCTGTCAGAGCTCTCTTGGGGAA
TGTGCCTCAGCAACTTCCCCGCTATTTGTAAGACAGAGGACTTCCTCCAA
CTGCCCAAAGATATGGTGGTGCAGCTTTTGTCACACGAGGAGCTGGAGAC
AGAAGATGAGAGACTGGTTTATGAAGCTGCCCTGAATTGGATCAACTATG
ACCTGGAAAGGAGGCACTGTCACCTTCCAGAGCTCCTGAGAACGGTCCGC
CTTGCCCTGCTGCCTGCCATCTTTCTCATGGAAAACGTCTCGACAGAAGA
GCTGATCAACGCCCAGGCCAAGAGCAAGGAGCTGGTGGACGAGGCTATCC
GCTGTAAGCTGAAGATCTTGCAGAATGACGGCGTCGTTAACAGCCCGTGT
GCTCGACCACGGAAAACCAGCCATGCTCTCTTCCTGCTGGGAGGGCAGAC
GTTCATGTGTGACAAGTTGTACCTGGTGGACCAGAAGGCCAAAGAGATCA
TCCCGAAGGCGGACATCCCCAGCCCCAGGAAGGAGTTCAGCGCCTGTGCC
ATCGGCTGTAAGGTGTACATCACCGGCGGGA--GAGGCTC-AGAGAAYGG
TGTGTCCAAAGATGTGTGGGTCTATGACACCATCCACGAGGAATGGTCCA
AAGCGGCGCCCATGCTCATCGCCAGGTTTGGCCACGGCTCTGCTGGCCTG
ANNNNNNNNNNNNNNNNNNNNNNNNNNNNNNNNNNNNNNNNNNNNNNNNNNNNNNNNNNNNNNNNNGGAT
GAATACATCGCTGTCTTCAGTCGTTCAACAACAAGACTGATACTG
AACGAACCCGAGCTAATCATGGCTCTGGCCCAGGAGTTCCAGATGAGAGT
TGTCACAGTTTCCCTAGAGGAACAGTCTTTCCCCAGCATTGTCCAGGTGA
TCAGCGGTGCTTCAATGTTAGTCAGCATGCATGGAGCTCAGCTTATCACC
TCACTCTTCCTCCCCAAAGGAGCTACTGTAGTGGAGCTGTTCCCCTTTGC
TGTGAACTCAGAGCAGTACACCCCATATAAAACCCTTGCCTCCCTTCCAG
GAATGGACCTTCATTATATTTCCTGGAGGAATACTCAGGAGGAAAACACT
GTCACCCATCCAGACAGACCCTGGGAACAAGGAGGCATCATTCACTTGGA
CAAGGAGGAGCAGGAGCGAATTCTGAGCAGCAAAGATGTCCCCAGGCACC
TGTGCTGCCGCAACCCGGAGTGGCTCTTCCGAATCTATCAGGACACATTG
GTAGACATTCCTTCCTTCCTGGARGTCCTCAA---CGAGGGAATGAAG--
-ACCAAGCCCAGCTYGAAGAA---GTCCAAGTCAGCTAGTACAGTCCATC
CAGGCCGGGTCAGAGAAGCCCAGTGTCAGACCTCAGTACAGACCACCAAT

GAGGCTAAACTCACAGTCTCCTGGCAGATCCCATGGAATCTGAAATACTT
GAAAGTAAGAGAGGTGAAGTACGAGGTG---------AAAAGAGACACCA
GCAAGGGAACTCTGGAGGATCAGATCATCCAGGCGAACCCGGCGCTGGAG
GCCTTCGGGAACGCCAAAACGCTGAGGAATGACAACTCGTCCCGTTTTGG
AAAGTTCATCCGAATTCACTTTGGAACCAGCGGAAAACTGTCGTCCGCTG
ACATCGAGACGTACCTGCTGGAGAAGTCTCGCGTCACCTATCAGCTCAAG
GCTGAGAGGGACTACCACATCTTCTACCAGATCCTGTCCAATCAGAAGCC
AGAGCTGCTGGACCTGCTGCTAATCACCAACAACCCCTACGACTACTCCT
ACATCTCCCAAGGAGAGGTAACGGTCGCCTCCATCAACGACTCAGAGGAG
CTGATGGCCACCGACAGCGCCTTCGACGTGCTGGGCTTCACGCCGGAGGA
GAAGATGGGCATCTATAAACTGACCGGCGCCATCATGCACCACGGCAACA
TGAAGTTCAAGCAGAAGCAGCGTGAGGAGCAGGCGGAGCCTGACGGGACG
GAGGCTGCTGATAAATCAGCTTACCTGATGGGACTGAACTCTGCTGACCT
CATCAAAGGATTGTGTCATCCCAGAGTCAAGGTAGGAAACGAGTACGTCA
CCAAAGGCCAGAGCGTGGACCAGGTCCACTACCCCAACAAGGAGGCCTTC
AAGTGTGAGGAGTGTGGGAAGCACTACAACACCAAGCTGGGATATAAGCG
CCATGTGGCCATGCACTCCGCCACGGCGGGGGATCTCACCTGTAAAGTGT
GCATGCAGAGTTATGAGAGCACCCCTGTTCTCTTAGAGCACCTCAAGAGC
CACTCGGGGAAGTCCTCGGGCGGAGCCAAGGAGAAAAAGCATCCGTGTGA
CCACTGCGACCGCCGTTTCTATACGCGGAAAGATGTGAGACGGCACATGG
TGGTTCACACAGGCCGAAAGGACTTCTTGTGCCAGTACTGTGCCCAGCGC
TTTGGAAGGAAGGACCACCTGACCCGCCACGTGAAGAAGAGCCACTCACA
GGAGCTGCTGAAGATCAAGACGGAGCCTCCTGATATGTTAGGTCTTTTAG
CCTCGGGGTCACCACCGTGCTCTGTGAAGGAGGAGCTTAGCCCCATGATG
TGCGGCATGGGTCCCAATAAAGACCCCATGATGGGCAAACCGTTCCCTAG
TGGCGCCCCTTTTCCGATGGGCATGTACAACCCCCACCAC------CTTC
AGGCCATGTCTAATTCTGGGGTGGGTCATGCA------CACCCGTCCCTG
ATGCCCAGCTCCCTGTCTGCAGCTATGGGCATGGGCTGTCACATGGAATA
TCTCATCTACGCCTCATTCTCATTCATGGGATGTTTACAAATCAGTGACG
GATCGAACATAGTGAACCTGCTGGCTAGTAACTCTCCGAGTGTTTCATAC
GCTCTGACCCAGCAGAAATACTTCAGCAACTACAGTCCTGTGATTGGCTT
TTACATTTACGAGCCCATCGAGTACTGGAACTCCACAGTGCAGGAGCACC
TGAAGACTCTGAGTCACGGCTTCAACAAGATCTCCTGGATGGACAACTTC
TTCCACTACCTGCGGGTGGTGAATGTGAGCGCATCGACCAAGAACGACTT
CATCACCATCCTCAAGGGCTCCTTCCTGCGCAGCCCAGAGTACCAGCATT
TCACTGAGGACATCATATTTTCCAAAA---ACCGCGAGACTG------AC
GAATATAACATTATCGCCTCACGCTTGTATTTGGTGGCACGGACCACAGA
GAAGAAGCGCGAGGAGGTGGTGGAGCTTCTGGAGAAGCTTCGCCCCTTGA
TGCTGATCAACAGCATCAAGTTCATTGCCTTCAATCCCACGTTTGTGTTC
ATGGACCGCTACAGCTCCTCCGTCATCTCACCCATCCTGACCTCAGGCTT
CAGCGTGCTCACCATCCTCATCCTCACTTTCTTCCTGGTCATCAACCCTT
TGGGGAACTTCTGGCTCATCCTCACGGTTACGTCCGTGGAGCTGGGCGTNNNNNNNNNNNNNNNNNNNNN
NNNNNNNNNNNNNNNNNNNNNNNNNNNNNNNNNNNNNNNNNNNNNNNNNNNNNNNNNNNNNNNNNNNNNN
NNNNNNNNNNNNNNNNNNNNNNNNNNNNNNNNNNNNNNNNNNNNNNNNNNNNNNNNNNNNNNNNNNNNNN
NNNNNNNNNNNNNNNNNNNNNNNNNNNNNNNNNNNNNNNNNNNNNNNNNNNNNNNNNNNNNNNNNNNNNN
NNNNNNNNNNNNNNNNNNNNNNNNNNNNNNNNNNNNNNNNNNNNNNNNNNNNNNNNNNNNNNNNNNNNG
AGAAACACGGTGGAGGACCACCTGTTCCTGAAAAGGCTGTACGTTTCTCT
TTCACTACTATATGTCTGTCTGTCCTGGCAGATGATGAGGAG--------
----------------------GAGGCAGTTACAATTTTCACGGAGCCGA
AGCCCAACTCAGAACTGTCCTGTAAGCCTCTTTGCTTGATGTTAGTGGAT
GAATCAGACCATGAGACCCTCACAGCTCTCCTGGGGCCCATAGTTGCAGA
GCGTAATGCAATGAAAGAGAGCAGGCTTATCCTTTCAATCGGCGGCCTGC
TTCGCTCCTTCCGCTTCCACTTCAGAGGCACGGGATATGATGAGAAGATG

GTGCGTGAGATGGAGGGCCTGGAGGCCTCAGGGTCCAGCTATGTCTGCAC
TCTGTGTGATTCCAGTCGGGCAGAGGCATCTCAAAACATGGTGCTACACT
CCATCACACGCAGCCATGACGAGAACCTTGAATGTTATGAAATATGGAGA
ACCAACCCTTTTTCTGAGTCAGGAGACGAGCTGCGGGACAGAGTCAAAGG
GGTCTCTGCCAAGCCCTTCATGGAGACCCATCCCACACTGGATGCATTAC
ACTGTGACATTGGCAATGCCACTGAGTTCTACAAAATTTTCCAGGATGAA
ATTGGGGAGGTGTTCCACAAGGT---CAAT---CCCAGCAGAGAGGAACG
GCGGAGCTGGAGGGCAGCCCTAGATAAACAGCTGAGGAAGAAGTTGAAGC
TTAAACCAGTAATGAGGATGAATGGGAACTATGCCCGCAGGCTAATGACT
CAGGAGGCTGTGGAGGCGGTGTGTGAGCTGGTGCCCTCAGAGGAGAGGAG
GGAGGTCCTGAGGGAGCTTATGAGACTATACATCCAGATGAAGCCTGTGT
GGCGCGCCACCTGCCCATCCAAGGAGTGCCCTGACCAGCTGTGCCGCTAT
AGCTTTAACTCCCAGAGCTTTGCTGACCTCCTCTCTTCTGCCTTCAAATA
TAGGTACAATGGAAAGATAAGCAATTACCTGCACAAGACTCTGGCCCATG
TCCCTGAAATCATAGAGAGAGAGATGGATCCATCGGAGCCTGGGCCAGCGAA
GGAAACGAGTCANNNNNNNNNTCATACACAATTGAGATGGGCCCGATGGGGCCCCAGTG
GAAGGAGAGCCCACAGCCTTTTTCCTGCTCAGTTGAAGACCCAACAAAAC
AGACAAAGTTCAAAGGCATCAAGACGTACATTTCGTATCGGGTCATGCCG
AGTCACACAGGGCACCCCGTCTACAGGCGCTACAAACACTTTGACTGGCT
GTACAACCGCCTACTGCACAAGTTCACTGTGATCTCTGTGCCTCACCTGC
CCGAGAAGCAGGCCACGGGGCGATTTGAGGAAGACTTTATCGAGAAGCGG
AAGAGACGACTGATATTGTGGATGAATCACATGACCAGTCACCCTGTCCT
CTCCCAGTACGAAGGCTTTGAGCACTTTCTGATGTGTGCCGATGACAAGC
AGTGGAAGCTGGGCAAAAGACGAGCWGAGAAGGATGAGATGGTGGGCGCC
CATTTCATGCTGACTCTCCAGATCCCTAATGAGCACCAGGACCTTCAGGA
TGTAGAGGAGAGGGTAGACACCTTCAAGGCCTTTGCTAAGAAAATGGACG
ACAGCGTCATGCAGCTCACACATGTTGCCTCGGAGCTGGTGCGTAAGCAC
CTGGGTGGATTCAGGAAGGAGTTCCAGCGGCTGGGAAATGCCTTCCAGTC
CATTAGCCAGGCATTCATGCTGGACCCTCCCCATAGCTCAGACACCCTCA
ACAACGCCNNNNNNNNNNNNNNNNNNCACGTTCCTCAAACTGACCTCTCTGGGTTTCATCATTGGCGT
CGGTGTGGTTGGAAACCTCCTGATCTCCATCCTGCTGGTCAAAGACAAGA
GCCTTCACCGAGCACCCTACTATTTCCTGCTGGACCTGTGCGCCTCCGAT
ATCCTTCGCTCCGCCATCTGCTTCCCCTTTGTCTTCACCTCGGTCAAGAA
TGGATCCGCCTGGACGTATGGCACGCTGACCTGCAAAGTGATCGCCTTCC
TGGGTGTGCTCTCCTGTTTCCACACGGCGTTCATGCTATTCTGCGTGAGC
GTCACCCGCTACCTGGCCATCGCCCACCACCGTTTCTACACCAAGAGGCT
GACCTTCTGGACCTGTCTGGCTGTCATCTGCATGGTGTGGACGTTGTCGG
TGGCTATGGCGTTCCCGCCGGTGCTAGACGTAGGGACATACTCTTTTATC
CGGGAGGAGGACCAGTGCACGTTCCAGCACCGCTCCTTCAGGGCGAATGA
TTCGCTGGGCTTCATGCTCCTGCTGGCGCTCATTCTCCTGGCCACACAGC
TGGTTTACCTCAAGCTCATCTTCTTCGTCCACGACCGTCGAAAGATGAAG
CCTGTCCAGTTTGTGCCTGCTGTCAGCCAGAACTGGACCTTCCACGGGCC
AGGCGCCAGCGGGCAGGCGGCGGCCAACTGGCTGGCCGGATTTGGTAGAG
GCCCCACCCCGCCTACTTTGCTGGGCATCCGGCAGAACAGCAACGCAGCA
GGCCGCAGGCGTCTACTGGTATTGGATGAATTCAAAACAGAGAAGAGGAT
TAGTAGGATGTTCTACATCATGACTTTTTTCTTCCTGGCACTGTGGGGGC
CCTATCTGGTAGCCTGCTACTGGCGGGTGTTTGCAAGGGGCCCTGTAGTC
CCTGGAGGCTACCTGACGGCAGCCGTGTGGATGAGCTTTGCGCAGGCTGG
GGTCAATCCATTCATCTGNNNNNNNNNNNNNNNNNNNNGCCAAATCTCGCTTTCACCCTGGCGTGGGGAC
TGGTCCTGGCACGGAGC---GCAGCGTCCCACTCGGCAACAGCTTGCTAT
CCCCGCAGCAAACCGAGGAGCCCACTGTTGCCACCCCCCCGCAGCGATGG
TTTGTCACCC---CTGCCAACAACCGACTGGACTTTGCTGCCTCGGCATA
CGACGCCGCT---------GATTTCGCCGGTAACGCAGCCACCTTGCTGT

CCTATGCAGCGGCCGGAGTGAAGGCTC------TTCCCCTGCCGACCGCA
GGCTGCTCCAACCGACCTCTTGGCTATTACGCAGACCCGTCCG---GCTG
G---GGAGGACGCACGCCGCCGCAGTACTGCGGCGTAAACAGCAAAACCA
GCTCGGTCTTTTCCTGCTGGCCCGCCAACTCTATCGGCGGCAGAGCAGGC
A---CC---AACTACCTGG---------CCGAGGA---GGGA---GACTC
---CATCCCGACGGAGAGGTCACCC---AT---C---GGCTCCGAGGAG-
--ACCAAACCCAAAGACATAAC---ATCAGA---GTCGAGCTGGATAGAG
---ACACCGCCCTCCATTAAGTCCATCGATTCAAGCGATTCTGGTATCTT
TG---AACAGGCCAAGAGGAGAAGAATCTCACCCTCTGCCACGCCG----
-----GTTTCAGAGACAGTGTCCCAGTTAAAATCCGAGNNNNNNNNNNCAGGCGAAGTCA
CAGAGAGAGAAGTGGCGTTGGGGATAAATCCGTTCGCAGATGGGATGGGC
GCCTTCAAAATAAACCACAGCTCCCACGATATTGGCTCCGG---ACAAAC
GGCGTTTTCCTCCCAGGCA---CCCGGCTAC---GCAGCAGCCGCCCTGG
GA---CACCACCA------CCACCCGACCCACGTTGGCTCT---TACTCC
ACGGCGGCTTTCAACTCCACCAGGGACTTTCTCTTCAGAAATCGGGGCTT
CGGGGACGCCACCGG------------GGCGCAGCACAGTTTGTTCGCCT
C---------CGGAAGTTT---C------GCAGGGCCACATGGACACTCA
GATGCGGCAGGGCACCTGCTCTTCCCGGGGCTCCATGAG---CAAGCGGC
GAGCCATGCGTCCTCCAACGTGGTCAACAGCCAGATGCGGCTGGGCTTCT
CGGGGGACATGTACGGACGGGCTGACCAGTACGGCCACGTTACGAGCCCG
AGGT---CCGACCACTACGCTTCGACCCAGCTGCACGGCTATGGCCCCAT
GAACATGAATATGGCCGCA---CACCACGGAGCAGGGGCCTTCTTTCGAT
ACATGAGGCAGCCGATCAAACAAGAGCTCATCTGCAAGTGGGTCGAGCCG
GAGCAGCTGACGAATCCCAAAAAGTCGTGCAACAAAACTTTTAGCACGAT
GCATGAGCTAGTGACCCATCTGACGGTGGAGCATGTGGGGGGACCGGAGC
AGACCAACCACATCTGCTTCTGGGAAGAGTGCTCCAGAGAAGGGAAGCCA
TTCAAAGCCAAATACAAACTTGTGAATCATATCAGAGTACACACCGGAGA
AAAGCCCTTTCCNNNNNNNNNNNNNNNNNNNNNNNNN
>Chirocentrus dorab
AGCCTGCTCATCCGAGCCGAATTGAGCCAACCGGGGGCCCTCCTTGGAGA
CGACCAGATCTACAACGTCATTGTTACCGCACATGCATTCGTAATAATCT
TCTTTATGGTCATGCCCATTCTCATCGGAGGGTTCGGCAACTGACTAGTC
CCCCTCATGATCGGGGCGCCCGATATAGCATTCCCCCGTATAAACAACAT
GAGCTTTTGGCTACTGCCCCCCTCATTTCTTTTACTGCTTGCCTCATCCG
CAGTTGAGGCGGGAGCGGGCACTGGGTGAACGGTCTACCCCCCCCTGGCC
GGTAATCTTGCCCACGCAGGTGCCTCAGTCGACCTGACCATTTTCTCACT
CCACCTGGCAGGTGTCTCATCTATTCTCGGGGCTATTAATTTCATTACCA
CAATTATCAACATGAAACCTCCCGCCATTTCACAATACCAGACACCCCTA
TTTGTCTGAGCGGTACTAGTCACTGCAGTACTCCTCCTCCTTTCTCTTCC
CGTGCTAGCGGCAGGAATTACCATGCTGCTTACGGACCGAAACTTAAACA
CGACGTTCTTCGACCCCGCTGGTGGGGGGACCCTATCCTCTACCAACANNNNNNNNNNNNNNNNNNNNNN
NNNNNNNNNNNNNNNNNNNNNNNNNNNNNNNNNNNNNNNNNNNNNNNNNNNNNNNNNNNNNNNNNNNNNN
NNNNNNNNNNNNNNNNNNNNNNNNNNNNNNNNNNNNNNNNNNNNNNNNNNNNNNNNNNNNNNNNNNNNNN
NNNNNNNNNNNNNNNNNNNNNNNNNNNNNNNNNNNNNNNNNNNNNNNNNNNNT
TCCTGGAGAAGAACCTGCACCCGTCCAACTGTCTGGGCATGCTCCTGCTG
TCCGACGCCCACCAGTGCACCCAGCTCTCCCAGCTGTCCTGGAGCATGTG
CCTGAGCAACTTCCCCGCCATCAGCAAGAGCGAGGAGTTCCTGCAGCTGC
CCAAGGACATGCTGGTCCAGCTGCTGTCCCACGAGGAGCTGGAGACGGAG
GACGAGCGGCTGGTCTACGAGTCCGCCCTCAACTGGGTCAACTACGACCT
GGAGCGCCGCCACTGCCACCTGCCCGAGCTGCTGCGCACCGTGCGCCTGG
CGCTGCTTCCCGCCATCTTCCTGATGGAGAACGTCTCCACCGAGGAGCTG
ATCAACGCCCAGGCCAAGAGCAAGGAGCTGGTGGACGAGGCCATCCGGTG
CAAGCTGCGCATCCTGCAGAACGACGGCCTGGTCAACAGCCCCTGCGCCC

GGCCCCGCAAGACCAGCCACGCCCTCTTCCTGCTGGGCGGCCCCACCTTC
ATGTGCGACAAGCTCTACCTGGTGGACCAGAAGGCCAAGGAGATCATCCC
CAAGGCCGACATCCCCAGCCCCCGCAAGGAGTTCAGCGCCTGCGCTATCG
GCTGCAAGGTGTACGTGACGGGCGGGC--GGGGCTC-CGAGAACGGCGTG
TCCAAGGACGTGTGGGTGTACGACACGCTGCACGAAGAGTGGTCCAAGGC
GCCGCCCATGCTGATCGCCCGCTTCGGGCACGGCTCGGCCGAGCTGCGCC
ACTGCCTGTACGTGGTGGGCGGGCACACGGCCGCCACGGGCTGCCTGCCG
GCCTCCCCGTCCTGATGACTACATTGTAGTGTTCAGCAGATCTATTAATA
GGCTGATACTCAACGAGGCTGAGCTGATTCTGGCACTAGCCCAAGAGTTC
CAGATGAGGACCGTCACAGTGTCCCTTGAGGACCAGTCGTTCTCAAGTAT
CATTCAAGTGATCAGTGGGGCATCCATGCTAGTGAGCATGCATGGAGCCC
AGCTCATCACTTCCTTGTTTCTGCCTCGAGGAGCAGTTATGGTGGAACTT
TTCCCCTATGCTGTGAACCCCGACCATTATACTCCTTACAAGACTCTTGC
TTCCTTGCCAGGCATGGACCTTCAATATGCAGCTTGGAAGAACACGATAA
TGGAGAACTCTGTAGCTTATCCAGAGCGACCCTGGGATCAAGGTGGCATC
TCTCATTTGGATAAGGATGAACAAGACCGCATCCTTGCAAGTAAGGAAGT
GCCCAGGCATCTATGTTGCAGTAACCCGGAATGGCTTTTCCGCATTTATC
AGGATACATTGGTGGACATACCATCGTTCATGGCTACCCTCAG---AAGA
ACTGTACAA---ATCAAGCCGAATTTGAAAAA---GGCCAGACCTGCAAG
CACAGTTCACCCAGGCCGAGTAAGGGAGCCTAAATGTCAGACTTCAGTCC
AAGCTGCCAATGAGGCCAAGTTGACGGTGTCTTGGCAAATTCCCTGGAAC
CTCAAGTACCTAAAGGTTCGTGAAGTCAAATATGAGGTGTGGATACAGAA
AAAGGATGCTAGTAAGGGGACTCTGGAGGATCAAATCATCCAGGCCAACC
CCGCTCTGGAGGCGTTTGGGAATGCCAAAACACTGAGAAACGACAACTCC
TCTCGCTTTGGGAAATTCATTCGTATTCACTTTGGAACCAGTGGAAAGCT
TTCCTCTGCTGACATTGAGACTTACCTCCTGGAAAAGTCCAGGGTGACCT
TTCAGCTTAAGGCTGAGAGGAACTACCACATCTTCTATCAGATCATGTCC
AATGAAAAGCCTGAGTTGTTAGACATGTTGTTGATCACCAATAACCCTTA
TGATTACTCTTATGTCTCTCAAGGAGAGATCGCTGTGGCCTCCATCAACG
ACTCAGAGGAGCTGATTGCCACCGACAGTGCCTTTGATGTGCTTGGCTTC
ACTGGTGAAGAGAAAATGGCAGTCTACAAGCTGACTGGTGCCATCATGCA
CCATGGCAACATGAAATTCAAGCAGAAGCAGAGAGAAGAGCAGGCCGAGC
CAGACGGCACAGAGTCTGCTGACAAGTCTGCTTACCTGATGGGATTAAAC
TCCGCAGACCTCCTCAAAGGACTCTGCCACCCAAGGGTCAAGGTAGGCAA
TGAGTACGTCACCAAAGGTCAGAATGTAGACCAAGTTTACTATCCCAACA
AGGAGGCCTTCAAGTGCGAGGAGTGCGGCAAGCACTACAACACCAAGCTG
GGCTACAAGCGGCACGTGGCCATGCACTCAGCCACGGCAGGCGACCTGAC
TTGCAAGGTCTGCCTGCAGAGCTACGAGAGCACGCCGGTGCTGCTGGAGC
ACCTCAAGAGCCACTCGGGCAAGAGCTCGGGTGGCGCCAAGGAGAAGAAG
CACCCGTGTGACCACTGCGAACGGCGCTTCTACACGCGCAAGGACGTGAG
GCGCCACATGGTGGTGCACACGGGACGCAAGGACTTCCTGTGCCAGTACT
GTGCCCAGCGCTTCGGCCGCAAGGATCACCTCACGCGTCACGTCAAGAAG
AGCCACTCACAGGAGCTGTTGAAAATCAAGACAGAGCCGCCGGACATGCT
GGGTCTGTTGGGCTCAGGATCGCCTCCCTGCTCTGTCAAGGAGGAACTCA
GCCCCATGATGTGCAGCATGGGCTCGGCCGGAGACCCCATGATGGCCAAG
CCCTTCCCCACTGGAGCGCCATTCCCCATGAGTATGTACAACCCGCACCA
C------CTCCAGGCCATGTCCAACCCGGGGGTGGGCCACCATCACCACC
ACCACTCCCTGGTGCCCAGCTCGCTGTCAGCTGCTATGGGCATGGGCTGC
CACATGGAG---

-----------------------GGTTTCCACCCCTTTGAGTGGCAGCCG
GCCCTGAAGACCGTGTCCACCAGCTGGGACGTAGGCATGCTGGACGGTCT
CTCGGGCTGGACCAGCTCGGTGGAGGACATCCCCGTGGACACCATCGCCC
GGCGTTTCCGTTACGACGTGGCCCTGGCGGCAGCGGTGAAGGACCTGGAG
GAGGACCTGATGGAGGGCATGAAGGAGCACAAGCTGGACGACAGC---TC
CGTGACGGGCTTCAGCGTCACCATCAAGGAGTCTTGCGACGGCATGGGGG
ACGTGAGCGAGAAGCACGGCAACGGCCCGGCGGTGCCCGAGAAGGCCGTG
CGCTTCTCCTTCACCATCATGTCGGTGTCGGCGCAGGTGGAGGGGGCGGA
G------------------------------GCTGCCGTGGTCATCTTCC
AGGAGATGAAGCCCAACTCGGAGCTCTCCTGCAAGCCGCTGTGCCTGATG
TTCGTGGACGAGTCGGACCACGAGATGCTGACTGCCGTCTTGGGGCCGCT
GGTCGCCGAGCGCGACGCCATGAAGGAGAGCCGTCTCATCCTCTCGCTGG
GCGGCCTGCAGCGCTCCTTCCGCTTCCACTTCCGGGGCACGGGCTACGAC
GAGAAGATGGTGCGCGAGATGGAGGGGCTGGAGGCGTCAGGCTCCACCTA
CATCTGTACGCTGTGTGACTCCACGCGCGCCGAAGCCTCGCACAACATGG
TGCTGCACTCCATTACGCGCAGCCACCAGGAGAACCTGGAGCGCTATGAG
ATGTGGCGCACCAACCCCTTCTCCGAGTCGGCGGACGAGCTGCGTGACCG
CGTTAAAGGCGTGTCCTCCAAACCCTTCATGGAGACGCAGCCCACACTAG
ACGCGCTGCACTGCGACATCGGCAACGCCACCGAGTTCTACAAGATCTTC
CAGGACGAGATCGGCGAGATGCACGCTCGCGGCGACAACCCGCCTACACG
TGAGGAACGACGCAGGTGGCGCACAGCGCTGGACAAGCAGCTGCGCAACG
TGATGAAGCTGAAACCAGTGATGCGCATGAACGGCAACTACGCCCGGAAG
CTGATGACGACAGAGGCGGCGGAGGCAGTGTGCGAGCTGGTGCCCACGGA
GCAGCGTCGGGAAGCGCTGAGGGAGCTCATGGCACTCTACCTGCAGATGA
AGCCGGTGTGGCGCTCCACCTGGCCTGCCCGCGAGTGCCCCGACCAGCTG
TGCCGCTACAGCTTCAACTCGCAGCGATTTGCTGAGCTGCTCTCCACCAC
CTTCAAGTACCGCTACGACGGCAAGATCACCAACTACCTACACAAGACGC
TGGCGCACGTGCCCGAGATTGTGGAGAGGGACGGCTCCATCGGTGCCTGG
GCCAGTGAGGGGAACGAGTCCGGCAACAAG--------------------

-
>Chitala chitala

```
AGCCTGTTAATCCGAGCAGAACTAAGCCAACCCGGCTCACTACTTGGTGA
CGACCAAATCTATAAWGTTATCGTTACAGCACACGCATTCGTAATAATCT
TCTTCATGGTAATGCCTATTATAATTGGAGGCTTTGGAAACTGGCTAATC
CCCTTAATAATTGGAGCCCCAGATATAGCATTCCCCCGAATAAACAATAT
AAGCTTTTGACTCCTGCCCCCTTCATTCCTACTACTCCTGGCCTCGTCCG
GAGTAGAAGCCGGTGCCGGGACCGGATGAACAGTATACCCACCTCTAGCA
GGAAACTTAGCACACGCCGGTGCCTCTGTAGACCTTACAATTTTTTCACT
CCATCTTGCTGGCGTCTCCTCAATTCTAGGAGCCATTAACTTTATTACAA
CAGTATTTAATATAAAACCCCCTGCCGTCTCACAATACCAAACACCACTA
TTCATCTGAGCTGTAATGATCACTGCAGTTTTACTCTTACTATCTCTTCC
AGTTCTAGCTGCCGGTATTACAATACTACTCACAGACCGCAACCTTAACA
CAACATTCTTTGACCCGGCAGGCGGAGGAGACCCAATTCTTTACCAACAT
CTATTTTGATTCTTTGGCCACCCAGAAGTATACATCTTAATTCTCCCAGG
TTTCGGTATAATTTCTCACATTGTCGCCTACTATTCTGGTAAGAAAGAAC
CATTCGGCTACATAGGCATGGTCTGAGCTATAATGGCCATCGGGCTGCTA
GGCTTTATTGTATGAGCCCACCATATATTTACAGTAGGAATAGATNNNNNNNNNNNNNNNNN-----
--------------------------------------------------
--------------------------------------------------
--------------------------------------------------
--------------------------------------------------
--------------------------------------------------
--------------------------------------------------
--------------------------------------------------
--------------------------------------------------
--------------------------------------------------
--------------------------------------------------
--------------------------------------------------
--------------------------------------------------
--------------------------------------------------
--------------------------------------------------
--------------------------------------------------
--------------------------------------------------
--------CAATGAATACATTGTCGTCTTCAGCCGCTCCATTAACAGACT
CATTCTGAATGAGGCAGAGCTGATTCTGGCACTGGCTCAGGAGTTTCAGA
TGAAAACCATCACAGTGTCCCTCGAGGAACATTCCTTCGCGGAGATTGTC
CGGATCATCAGCGGAGCCTCCATGCTGGTTAGCATGCACGGGGCACAGCT
GGTCACCTCCCTCTTCCTCCCTCGCGGTGCTGCTGTGGTCGAGCTCTTCC
CGTACGCAGTCAACCCTGAGCACTACACACCCTACAAAACACTGGCCTCT
TTGCCTGGGATGGACCTTCACTATGTGGCCTGGAGGAACACCATTGTGGA
GAACTCTGTGACCTTCCCAGATCGTCCTTGGGAACAGGGTGGCATTGCCC
ACCTGGAGAAGGAAGAGCAAGAACGAATCCTTAAAAGCAAGGAGGTGCCT
CGCCACCTGTGCTGTCGCAACCCCGAGTGGCTCTTCCGCATCTACCAGGA
CACCAAGGTGAACATCCCTTCTCTGCTGGAGGTCCTGAG---GCTGACCC
TCAAG---TCCAAGCCCAACCCAAAGAG---GTCAAAGACGGTCAGCACT
GTGCACCCGGGTCGAGTGAGGCAGCCCAAGTGTGAGACCTCGGTTCAGGC
CACCAATGAGGCCCAGATGACAGTGTCCTGGCAGATCCCCTGGAACCTGA
AGTACCTCAAGGTCAGGGACGTTAAGTATGAAGTGTGGATACAGAAGAAG
GACACAAGCAAGGGCACACTGGAGGACCAGATCATTCAAGCTAACCCAGC
CCTTGAAGCTTTYGGCAACGCTAAAACTCTACGAAATGACAACTCGTCAC
GTTTCGGAAAATTCATCCGCATTCACTTTGGAGTGAGCGGAAAACTGTCC
TCCGCTGACATTGAGACGTACCTGCTGGAGAAATCTCGTGTCACCTTCCA
GCTCAAAGCGGAGAGGAACTACCACATATTCTATCAAATTTTGTCCAACA
AAAAGCCAGAGCTGCTGGACATGTTGTTGATTACCACCAACCCTTATGAC
```

TACKCCTACATCTCCCAAGGAGAAGTAGCGGTTGCGTCCATCGATGACTC
AGAAGAGCTGGTAGCTACTGACAGTGCGTTTGACGTGCTGGGCTTCACTG
CAGAGGAGAAGATGAGCGTGTACAAGCTCACAGGTGCAATCATGCACTAT
GGAAACATGAAGTTCAAACAGAAGCAGCGCGAGGAACAAGCTGAGCCTGA
CGGCACAGAATCAGCTGACAAGTCGGCCTACCTTATGGGATTAAATTCAG
CTGACCTGCTGAAGGGCCTGTGCCATCCTAGAGTCAAGGTTGGTAATGAA
TTTGTCACAAAAGGTCAAAATGTGCCACAAGTNNNNNNNCCACACAAGGAGGCCTTC
AAGTGCGAAGAGTGCGGCAAGAACTACAACACCAAGCTGGGTTACAAGCG
CCACGTGGCGATGCATGCGGCCACCAGCGGCGACCTCACCTGCAAGGTGT
GCCAGCAGCGGTACGAGAGCACTCCTGTGCTGCTGGAGCACCTGAAGAGC
CACTCGGGGAAGTCGTCCGGCGGCGCCAAGGAGAAGAAGCACCCGTGTGA
GCACTGCGACCGCCGCTTCTACACACGCAAGGACGTGCGGCGTCACATGG
TGGTGCACACGGGCCGCAAGGACTTCCTGTGCCAGTACTGCGCCCAGCGC
TTCGGCCGCAAGGACCACCTGACGCGGCACGTCAAGAAGAGCCACTCCCA
GGAGCTGCTCAAGATCAAGACAGAACCGCCGGACCTGCTGGGCCTCCTGG
GCTCCGGGTCGCCCCCCTGTGCTGTGAAGGAGGAGCTGAGTCCCATGATG
TG---TATGGGACCCTCCAAGGACTCCATGCTGTCCAAGACCTTCCCGGG
CA------CTTTCCCCATGGGCATGTACACCCCCCAC---------CTGC
AGGCCATGACCAGCTCCGGGCCGNNN------------
NNNNNNNNNNNNNNNNNNNNNNNNNNNNNNNNNNNNNNNNNNNNNNNNNNNNNNNNNNNN----------
-----

NNNNNNNNNNNNNNNNNNNNNNNNNNNNNNNNNNNNNNNNNNNNNNNNNNNNNNNNNNNNNNNNNNNNNN
NNNNNNNNNNNNNNNNNNNNNNNNNNNNNNNNNNNNNNNNNNNNNNNNNNNNNNNNNNNNNNNNNNNNNN
NNNNNNNNNNNNNNNNNNNNNNNNNNNNNNNNNNNNNNNNNNNNNNNNNNNNNNNNNNNNNNNNNNNNNN
NNNNNNNNNNNNNNNNNNNNNNNNNNNNNNNNNNNNNNNNNNNNNNNNNNNNNNNNNNNNNNNNNNNNNN
NNNNNNNNNNNNNNNNNNNNNNNNNNNNNNNNNNNNNNA
AGGCAGTTCGGTTCTCCTTCACGGTCATGACAATTTCTGTTCGGCCTGAG
GGTCAAGAG------------------------------GAAGCAATTAT
CATCTTCCAGGAGACCAAGCCAAATTCAGAGCTCTCCTGCAAACCACTTT
GCCTGATGTTTGTGGATGAGTCAGACCATGAGACCTTGACTGCAGTTCTG
GGTCCTGTGATTGCAGAGAGGAACTCCATGAAGGAGAGCCGGCTCATCCT
GTCCATCGGAGGCATGCCTCGCTCCTTCCGCTTCTTCTTCCGTGGCACTG
GCTATGATGAGAAGATGGTGAGAGAAATGGAGGGCCTGGAGGCCTCGGGA
TCCACCTACATTTGCACCCTGTGTGATTCCACCAGGGCGGAGGCCTCAAA
GAATATGGTGCTGCACTCCATCACCCGAAGCCACGAGGAGAACTTGGAGC
GCTACGAGGTTTGGAGGACCAACCCGTTTTCCGAGTCTGTGGACGAGCTC
CGTGAGCGAGTCAAAGGAGTGTCGGCGAAGCCCTTCATGGAGACGCAGCC
AACGCTGGACGCGTTGCATTGCGACATTGGAAATGCCACAGAGTTCTACA
AGATCTTCCAGGACGAAATCGGGGAAGTCTACTTGAAACC---TAAT---

CCTAGCCGAGACGAGAGAAGACGCTGGCGCTCAGCGCTGGACAAGCAGCT
GAGGAAGGAGCTGAAGCTAAAGCCTGTCATGCGCATGAATGGCAACTATG
CTCGCCGGCTCATGACCCGTGAAGCTGTGGAAGCCGTGTGTGAACTGGTG
CCTACTCCAGAAAGGAGGGAAGCCCTGAAGGAGCTCATGGAGATCTACCT
CCAGATGAAGNNNNNNNNNNNNNNNNNNNNNNNNNNNNNNNNNNNNNNNNNNNNNNNNNNNNNNNNNNNN
NNNNNNNNNNNNNNNNNNNNNNNNNNNNNNNNNNNNNNNNNNNNNNNNNNNNNNNNNNNNNNNNNNNNNN
NNNNNNNNNNNNNNNNNNNNNNNNNNNNNNNNNNNNNNNNNNNNNNNNNNNNNNNNNNNNNNNNNNNNNN
NNNNNNNNNNNNNNNNNNNNNNNNNNNN--

---------------NNNNNNNNNNNNNNNNNCAAACTGACTTCCCTGGGCTTCATCATTGGCATCG
GCGTGGTTGGAAACCTCCTGATCTCCATCCTGCTCATCAAAGACAAAAGC
CTTCATAGAGCCCCGTACTACTTTCTTCTGGATCTTTGTGCGTCGGATGT
GCTGCGGTCGGCGATTTGTTTCCCGTTCGTGTTCACCTCAGTCAAGAATG
GGTCAGCGTGGACGTACGGCTCGCTCACCTGCAAAGTGATTGCGTTCCTG
GGCGTGCTCTCCTGCTTCCACACGGCCTTCATGCTGTTCTGCGTCAGCGT
GACCCGCTACCTGGCCATCGCCCACCACCGCTTCTACACCAAGAGGCTGA
CATTTTGGACGTGCCTGGCTGTTATATGCATGGTGTGGACGCTGTCTGTG
GCCATGGCCTTCCCGCCGGTGCTGGACGTGGGCACCTACTCCTTCATCCG
AGAGGAGGACCAGTGCACCTTCCAACACCGCTCCTTCCGGGCCAACGACT
CCCTGGGCTTCATGCTGCTCCTGGCCCTCATCCTCCTCGCCACCCAGCTG
GTCTACCTCAAGCTCATCTTCTTCGTCCACGATCGACGAAAGATGAAGCC
TGTCCAGTTTGTGCCGGCCGTCAGCCAGAACTGGACTTTCCACGGTCCCG
GGGCGAGCGGGCAGGCTGCAGCTAACTGGCTGGCTGGCTTTGGGCGAGGC
CCGACTCCGCCCACCCTACTGGGGATCCGGCAGAACGCCAACCCGGCAGG
CCGTCGGCGCTTGTTGGTCCTGGACGAGTTCAAGACCGAGAAGAGGATCA
GCAGGATGTTCTATGTCATGACCTTCTTCTTTCTGACACTGTGGGGGCCC
TATCTGGTGGCATGCTACTGGAGAGTGTTTGCCAGGGGGCTGGCAGTGCC
AGGGGGCTACCTGACGGCCGCAGTCTGGATGAGCTTTGCTCAGGCCGGGG
TCAACCCCTTCATCTGCATTTTCTCNNNNNNNNNN-------------------------

----ACAAGCGAAGTCACAGACAGAGAAGTGGCTTTGGGGATAAATCCGT
TTGCCGACGGGATGGGTGCTTTTAAAATCAACCACAGTACCCACGATTTG
GGGACTGG---TCAAACAGCGTTCACCTCGCAGGCT---CCGGGCTAT--
-GCGGCTGCAGCCCTGGGA---CATCATCA------CCATCCTACCCATG
TCAGCTCC---TATTCCACGGCGGCTTTCAACTCTACCCGGGACTTTCTG
TTTCGAAACAGGGGGTTTGGAGATGCGGCGAC------------TGCTCA
GCACAGTCTTTTTGCCACCGC---AGCAGGAGGCTT---C------GCAG
GGCCACACGGACACTCAGATGCTGCGGGACATCTTCTTTTCCCGGGACTT
CACGAA---CAAGCGGCCACCCACGCCACTTCCAATGTTGTAAACAGTCA
GATGCGACTGGGATTTTCGGGGGAAATGTATGGCAGAGCAGAGCAGTACG
GCCACGTAACGAGCCCCAGGT---CCGATCACTATGCGTCTACCCAGTTA
CACGGCTATGGCCCCATGAACATGAATATGGCAGCA---CACCATGGAGC
CGGGGCCTTCTTTCGCTATATGAGGCAGCCGATAAAACAAGAGCTTATTT
GCAAATGGATTGAACCGGAACAGCTAACGAACCCGAAAAAGTCTTGCAAC
AAAACTTTCAGCACAATGCACGAGCTTGTGACTCACCTTACAGTGGAACA
TGTTGGGGGACCAGAACAATCGAATCACATATGCTTTTGGGAAGAGTGTC
CCCGGGAAGGAAAACCGTTTAAAGCGAAGTACAAGCTTGTGAATCACATC
AGAGTACACACTGGCGAGAAACCCTTCCCGTGCCCTTTCCCGGGGTGTGG
GAAA
>Chlorophthalmus agassizi
AGCCTCCTTATTCGGGCAGAACTAAGCCAGCCCGGAGCCCTTCTAGGCGA
CGATCAGATTTACAATGTTATCGTTACAGCCCACGCCTTTGTAATGATTT
TCTTCATAGTAATGCCAATTATGATTGGAGGGTTTGGAAACTGGCTCATC
CCGCTTATGATTGGAGCCCCCGACATGGCATTCCCCCGGATAAATAACAT
GAGCTTCTGGCTTCTTCCTCCATCTTTCCTCCTCCTCCTCGCTTCCTCTG
CAGTAGAGGCAGGAGCCGGCACAGGGTGAACAGTCTACCCTCCTCTTGCC
AGTAATCTAGCGCACGCCGGAGCCTCTGTTGACCTAACCATCTTTTCGCT
TCACCTAGCAGGAATTTCCTCCATCTTGGGTGCTATCAACTTTATTACAA
CCATCATTAATATGAAGCCCCCAGCAATTACTCAATATCAAACCCCCCTG
TTTGTATGAGCAGTTCTGATTACCGCCGTCCTTCTTCTCCTATCCCTCCC
TGTTTTAGCAGCAGGCATTACGATGCTTCTTACTGATCGTAATCTAAACA
CAACGTTCTTCGACCCGGCGGGGGGAGGAGACCCAATCCTCTATCAACAC
CTATTCTGATTCTTTGGCCACCCAGAGGTTTATATTTTAATTCTTCCAGG
CTTTGGTATAATCTCCCATATTGTTGCTTACTACTCGGGTAAAAAAGAAC
CTTTCGGCTACATGGGTATAGTTTGAGCCATGATAGCAATTGGCCTTCTT
GGGTTCATTGTTTGGGCTCCCCTCATGTTTACAGTCGGGATGGACGTAGA
CACACGTGCCTATTCCTGGAGAGGAACCTGCACCCATCCAACTGCCTTGG
CATGCTGCTACTGTCAGATGCCCACCAGTGCACCAAGCTGTCAGAGCTGT
CCTGGGGTATGTGCCTCAGCAACTTCCCTGCCATCTGCAAGACGGAGGAC
TTCCTCCAGCTGCCCAAAGACATGGTGGTGCAGCTCCTGTCCCATGAAGA
GCTGGAGACAGAAGATGAGAGACTAGTTTATGAGGCTGCCCTTAACTGGG
TCAACTATGACCTGGAGAGGAGACACTGCCACCTGCCAGAGCTGTTGAGA
ACCGTGCGCCTGGCCTTGCTTCCCGCCATCTTCCTCATGGAGAACGTCTC
CACAGAGGAGCTGATCAATGCCCAGGCAAAGAGCAAGGAGTTGGTGGATG
AGGCCATCCGCTGCAAGCTGAAGATCCTGCAGAATGACGGTGTGGTCAAC
AGTCCCTGTGCCCGGCCCAGAAAGACCAGCCACGCCCTTTTCCTGCTGGG
AGGGCAGACCTTCATGTGTGACAAGCTGTACTTGGTGGACCAGAAGGCCA
AAGAGATCATCCCCAAGGCGGACATCCCCAGCCCCAGGAAGGAGTTCAGT
GCCTGTGCCATTGGCTGTAAGGTCTACGTCACAGGCGGGA--GAGGCTC-
AGAGAACGGTGTATCTAAAGACGTGTGGGTCTATGACACCGTCCACGAGG
AGTGGTCCAAGGCAGCACCCATGCTCATTGCCAGGTTTGGCCATGGCTCT

GCTGAGCTCAAACACTGCCTCTATGTGGTTGGAGGACACACAGCAGCCAC
TGGCTGCCTCCCAGCCTCTCCGTCTGGATGAGTACATTGTGGTGTTCAGT
CGTTCCACAACAAGGCTAATTCTGAACGAAGCAGAGCTGATACTGGCACT
GGCCCAAGAGTTTCAGATGAGGGTTGTTACAGTGTCCCTGGAGGAACAGT
CTTTCCCCAGCATCGTACAGGTCATCAACGGGGCCTCCATGTTGGTCAGT
ATGCACGGAGCTCAGCTCGTCACCTCACTCTTCCTCCCTAGAGGAGCTGC
TGTAGTGGAGCTCTTCCCCTATGCTGTGAACCCGGAGCAGTACACCCCAT
ACAAAACCCTAGCCTCCCTACCAGGCATGGACCTTCAGTACGTTTCCTGG
AGGAACACTATTGAGGAGAACACGGTCACCCACCCAGACAGACCCTGGGA
CCAAGGAGGCATTGCCCATTTGGAGAAAGAGGAGCAGGAGAGAATCCTAG
CCAGCAAGGATGTCCCCAGGCACCTYTGCTGCCGCAACCCAGAGTGGCTC
TTCAGGATCTACCAGGACACTCTGGTAGACATCCCCTCCTTCCTGGAAGT
CCTCAA---GGAGGGCCTGAAG---ACAAGGCCGAGCTTGAAGAA---GT
CCAAGCCAGCCAGCACGGTTCACCCGGGCCGGGTCCGAGAACCCCA-TGC
CAGACTTCAGTGCAAGCCGCCGACGAGGCAAAACTCACAGTATCCTGGCA
GATCCCATGGAACCTTAAGTATCT-AAGGTGCGAGAGGTGAAGTACGA-G
TGTGGATCCAGAAAAAGGACACAAGCAAGGGAACCCTGGAGGATCAAATC
ATTCAGGCGAACCCTGCACTGGAGGCTTTTGGTAATGCCAAAACACTGAG
GAACGATAATTCCTCCCGTTTTGGAAAATTCATCCKCATCCACTTTGGAA
ACAGTGGTAAACTGTCCTCTGCGGACATTGAGACCTACCTGCTGGAGAAG
TCACGGGTCACCTTTCAGCTTAWGGCAGAGAGGAACTACCACATCTTCTT
CCAGATCTTGTCCAATCAAAAGCCAGAGCTGTTGGACATGCTACTGATCA
CCAACAACCCCTATGACTACTCGTACATCTCCCAAGGAGAAGTAACCGTA
GCATCCATCAATGATTCAGAGGAGCTGATGGCCACTGACAGTGCCTTTGA
TGTGCTTGGCTTCACSCAMGAGGAGAAGATGGGAGTCTACAAGTTGATAG
GGGCCATTATGCACTATGGCAACATGAGGTTCAAGCAAAAGCAACGTGAG
GAGCAGGCTGAGCCTGATGGTACAGAGGCTGCTGATAAGTCAGCTTATCT
AATGGGGCTGAATTCGGCAGACCTAATCAAAGGACTCTGCCATCCCAGAG
TCAAGGTAGGAAATGAGTATGTCACCAAAGGCCAGGGTGTAGATCAAGTC
TACTACCCCAACAAGGAGGCCTTCAAGTGTGAAGAGTGTGGCAAGCACTA
CAACACCAAGCTGGGCTACAAGCGCCATGTGGCCATGCACTCTGCCACGG
CAGGGGACCTCACCTGCAAAGTGTGCATGCAGAGCTACGAGAGCACCCCG
GTGCTCCTGGAACACCTCAAGAGCCACTCGGGGAAGTCCTCGGGCGGCGC
CAAGGAGAAGAAGCACCCGTGCGACCACTGCGACCGTCGCTTCTACACGC
GGAAGGACGTCAGGCGGCACATGGTTGTCCACACGGGCCGAAAGGACTTC
CTGTGCCAGTACTGTGCCCAGCGCTTCGGCAGGAAGGATCATCTGACACG
GCACGTGAAAAAGAGCCACTCGCAGGAGCTGCTGAAGATTAAGACGGAGC
CTCCGGATATGTTGGGTCTCCTGGGCTCTGGCTCGCCACCTTGCTCTGTC
AAGGAGGAGCTGAGCCCCATGATGTGCAGCATGGGTCCCAATAAAGACCC
CATGATGGGCAAACCCTTCCCCAGCGGGACCCCCTTCCCCATGGGCATGT
ACAACCCCCACCAC------CTCCAGGCCATGTCTAATCCTGGGGTGGGC
CACCCC------CACCCCTCCCTTATGCCTGGTTCTCTATCTGCAGCTAT
GGGCATGGGCTGTCATATGGAGTATCTCATCTACGCYTCCTTCTCCTTCA
TGGGATGTTTACAAATCAGCGACGGGTCGAACGTCGTGAATTTGCTGGCC
AGTAACTCTCCGAGCGTCTCGTACGCCCTGACGCAGCAGAAGTACTTCAG
TAACTATAGCCCCGTGATCGGGTTTTACATCTATGAGCCCATCGAGTACT
GGAACTCCACGGTGCAGGAGCACCTGAAGACGTTGAGCCACGGCTTCAAC
AAGATCTCCTGGGTGGACAACTTCGTCCACTACCTGCGGACGGCGAACGT
GAGCGCGTCGACCAAGGCCGACTTCGTCGCCGTCCTCAAGGGCTCCTTCC
TGAGGAGCCCGGCGTACCAGCACTTCACGGAGGACATCATCTTCTCCAAG
A---GCCACGAGAGCG------ACGGCTACGACATCATCGCCTCGCGCAT
GTACCTGGTGGCGCGCACCACCGAGAAGAGGCGCGAGGACGTGGTGGAGC
TCCTGGAGAAGCTCCGGCCGCTGATGCTGATCAACAGCATCAAGTTCATC

GCCTTCAACCCCACGTTSGTCTTCATGGACCGCTACAGCTCCTCGGTCGT
CTCGCCCATCCTCACCTCGGGCTTCAGCGTGCTCACCATCCTCATCCTCA
CCTTTTTCCTGGTCATCAACCCGCTGGGGAACCTGTGGCTCATCCTGACG
GTCACGTCCGTGGAGCTGGGCGTGCTTGGCCTCATGGGCTTCCACACATT
CGAGTGGCAGCCAGCCCTCAAGAATGTGTCCCCATCCTGCCATGTGGGCA
TCATCAATGGGCTCTCTGGGTGGGCCGCCTCGGTGGATGACGTCCCCGCT
GACACCATCACCCGTCGGTTTCGCTATGACGTGGCCCTCGTATCGGCCCT
GAAGGACCTCGAGGAGGACATCATGGAGGGACTGAGGGAGCGTGGGCTGG
AAGACAGTGCTTGCACCACAGGCTTCAGCGTTATGATCAAGGAATCCTGC
GATGGCATGGGAGATGTCAGCGAGAAGCATGGCGGTGGGCCAATGATGCC
CGAGAAGGCTGTGCGCTTCTCTCTCACTGTCATGTCTGTCTCTTTCCAGG
CCGAGGGAGGGGAG------------------------------GAGGCG
GTTGTCATCTTCAGGGAGCCAAAGCCCAACTCTGAACTTTCCTGTAAGCC
CCTTTGCCTGATGTTTGTGGACGAGTCTGACCACGAGACGCTCACCGCTG
TCCTGGGGCCTCTGGTAGCGGAGAGGAATGCGATGAAGCAGAGCCGACTC
ATCCTCGAGGTGGGCGGCCTCCCCCGCTCTTTCCGCTTCCACTTCAGAGG
CACAGGCTACGATGAGAAGATGGTGCGAGCGATGGAGGGCATGGAGGCCT
CAGGCTCCACCTATGTCTGCACTCTCTGTGACTCCACTCGGGCAGAGGCC
TCTCACAATATGGTGCTCCACTCTGTCACGCGTGGCCATGATGAGAACCT
GGAGCGCTACGAGATATGGAGGACCAACCCCTTCTCGGAGTCTGCCATGG
AGCTGCGAGATCGGGTCAAAGGGGTCTCTGCCAAACCATTTATCGAGACC
CATCCGACTATGGATGCGTTACACTGTGACATAGGCAATGCCACTGAATT
CTACAAAATCTTCCAGGATGAGATTGGGGAGGTGTTCCAGAACGC---CA
AC---CCCAGCCGGGAGGAACGGCGCAGCTGGAGGGCAGCCCTCGATAAA
CAGCTGAGGAAGAAGTTGAAACTGAAACCAGTGATGCGGATGAATGGGAA
CTACGCCCGCAGGTTGATGACCCAGGAGGCTGTGGAGGTGGTGTGTGAGC
TGGTGCCCTCAGAAGAGAGACGGGAGGCCCTGAGGGAGCTGATGGGGCTC
TATATACAGATGAAGCCGGTGTGGCGTGCTACCTGCCCAGCCAAGGAGTG
CCCTGACCAGCTGTGCCGCTATAGCTTTAACTCTCAGCGCTTTGCTGATC
TCCTTTCCTCCACCTTTAAATACAGGTACGACGGGAAGATCACCAACTAC
CTGCACAAGACCCTGGCTCATGTCCCTGAAATTATAGAGCGAGATGGCTC
CATTGGAGCTTGGGCCAGCGAGGGGAATGAGTCCGCGAACAAG-------

--------------ACAGGCGAAGTCACAGAAAGAGAGGTGGCTTTGGGG
ATAAATCCTTTCGCAGACGGGATGGGCGCCTTCAAAATAAACCACAGCTC
TCATGACATTGGCTCCGG---GCAAACGGCGTTTTCCTCCCAGGCG---C
CCGGCTAC---GCAGCGGCTGCACTGGGA---CACCATCA------CCAC
CCGACCCACGTTAGCTCC---TATTCCACGGCAGCTTTCAATTCCACCCG
GGACTTTCTCTTCAGAAATCGGGGCTTCGGAGATGCCACCAG--------
----CGCGCAGCACAGTTTGTTCGCCTCCGC---CGCGGGAAGTTT---T
------GCAGGGCCACATGGACACTCAGATGCCGCGGGCCACCTGCTCTT
CCCTGGGCTTCATGAG---CAAGCCGCGAGCCATGCGTCGTCTAACGTTG
TAAACAGCCAGATGCGACTGGGCTTTTCTGGGGACATGTACGGGCGGGCC
GACCAGTATGGCCACGTTACAAGCCCAAGGT---CCGACCACTATGCTTC
GACCCAATTGCATGGCTATGGCCCTATGAACATGAATATGGCCGCT---C
ACCACGGAGCAGGGGCCTTCTTTCGGTACATGAGGCAGCCGATTAAACAA
GAGCTTATCTGCAAGTGGATCGAACCGGAGCAACTGACGAATCCCAAAAA
GTCGTGCAACAAAACTTTTAGCACGATGCATGAGCTCGTGACCCATCTGA
CGGTGGAGCATGTGGGGGGACCCGAGCAGTCGAACCACATTTGCTTCTGG
GAAGACTGTGCCCGAGAAGGGAAACCATTCAAAGCCAAATACAAACTTGT
AAATCATATCAGAGTTCACACCGGAGAAAAACCGTTCCCGTGTCCATTCC
CCGGCT--------
>Chologaster cornuta
AGCCTCCTCATCCGAGCTGAATTAAGCCAACCCGGCGCCCTACTAGGCGA
CGACCAAATCTACAACGTGCTTGTTACAGCGCACGCCTTCGTTATGATTT
TCTTTATAGTTATGCCTATCATAATTGGCGGCTTCGGCAACTGACTCATC
CCCCTAATAATCGGCGCCCCTGACATAGCCTTCCCCCGAATGAACAACAT
AAGCTTTTGATTACTTCCCCCCTCTTTCCTTCTCCTACTAGCCTCCTCCG
GCGTAGAAGCTGGCGCCGGAACTGGTTGAACTGTTTACCCTCCCTTGGCA

GGAAACCTGGCCCACGCAGGAGCTTCCGTAGACCTAACAATCTTCTCCCT
ACACCTTGCAGGTATCTCCTCAATTCTTGGAGCCATTAACTTTATTACAA
CTATTATCAACATGAAGCCCCCGGCAATCTCACAATACCAGACCCCTCTA
TTCGTGTGGGCTCTACTAATCACCGCCGTTCTTCTACTCCTCTCCCTCCC
CGTTCTAGCAGCCGGCATCACAATGCTCCTAACAGATCGAAATCTTAACA
CAACTTTCTTTGACCCAGCAGGAGGGGGTGACCCCATTCTATACCAACAC
CTC---

------------TTCCTGGAGAGGAACCTGCACCCGTCCAACTGCCTAGG
CATGCTGCTGCTCTCGGACGCCCACCAGTGCGCCAAGCTGTCGGAGCTCT
CCTGGGGCATGTGCCTCAGCAACTTCCCCGCCATCTGTAAGACGGAGGAC
TTCCTGCAGCTGCCCAAGGACATGGTAGTGCAGCTGCTCTCCCACGAGGA
GCTGGAGACGGAGGACGAGCGGCTGGTGTACGAGGCGGCCCTCGGCTGGC
TGAACTACGACCTGGACGGGAGGCACTGCCACCTGCCGGAGCTGCTCCAG
ACAGTCCGCCTGGCTCTGCTGCCGGCCATCTTCCTGATGGAGAACGTGTC
GACGGAGGAGCTGATCAACTCCCAGGCCAGGAGCAAGGAGCTGGTGGACG
AGGCCATCCGCTGCAAGCTGAAGATCCTGCAGAACGATGGCGTGGTCAAC
AGCCCGTGCGCACGGCCCCGCAAGACCAGCCACGCCCTCTTCCTGCTGGG
CGGCCAGACGTTCATGTGCGACAAGCTGTACCTGGTGGACCAGAAGGCCA
AGGAGATCATCCCCAAGGCTGACATCCCGAGCCCCCGCAAGGAGTTCAGC
GCGTGTGCCATTGGCTGCCGGGTGTACATCACCGGCGGGC--GGGGCTC-
CGAGAACGGCGTCTCCAAAGACGTGTGGGTGTACGACACCGTGCACGAAG
AGTGGTCCAAGGCAGCACCCATGCTCATCGCCCGCTTCGGCCACGGCTCG
GCCGAGCTGAAACACTGCCTCTATGTGGTGGGAGGTCATACTGCGGCTAC
AGGCTGCCTACCGGCCTCCCCGTCGGGATGAGTACGTCGTGGTGTTCAGT
CGTTCGACAACAAGGCTGATTCTGAATGAAGCGGAGCTAATTATGGCTCT
TGCACAAGAGTTCCAGATGAGGGTGGTCACCGTCTCCCTGGAGGAACAGC
CGTTTGCCAGTATCGTGCAGGTGATCAGCGGAGCCAGCGTGCTGGTAAGC
ATGCATGGAGCTCAGCTCGTCACATCACTGTTCCTGCCCAGAGGAGCAGC
CGTGGTGGAGCTGTTCCCGTACGCTGTGAACCCACAGCAGTACACACCAT
ATAAGACGCTGGCATCCCTGCCAGGCATGGACCTTCACTACGTTTCATGG
AGGAACACTATGGAGGAGAACACAGTCACCCACCCAGACAGACCTTGGGA
CCAAGGAGGCATCACGCACTTGGAAAAGGAGGAGCAAGAGCGAATACTGG
CCAGCAAGGACGTGCCCAGGCATTTGTGTTGCCGCAACCCAGAATGGCTT
TTCCGGATCTACCAGGACACTTTGGTAGACATTCCTTCAATGCTGGAGGT
CCTGAA---AGAGGGCCTGAAG---ACGAGGCCTAGCTTAAAGAA---AA
CCAGGCCGGCCAGCACAGTCCATCCTGGACGGGTCAGGGAAGCCCAATGT
CAGACCTCCGTCCAAGCTGCCAATGAGGCTAAGCTCACAGTCTCCTGGCA
GATCCCATGGAATCTCAAATACCTGAAGGTGAGAGAAGTTNNNNNNNNNNNNNNNNNNNNNAAGAAGGAT
G
CCAGCAAGGGAACACTTGAGGATCAGATCATCCAGGCCAACCCGGCCCTG
GAGGCCTTTGGCAATGCCAAGACCGCCCGCAATGACAACTCCTCCCGTTT
TGGAAAATTCATTCGGATCCACTTCGGAACAAGCGGGAAGCTGTCATCCG
CTGACATCCAAACATACCTTCTGGAAAAGTCCCGGGTCACCTTTCAGCTC
AAAGCTGAGAGGAATTACCACATCTTCTTCCAGATCCTATCTAATCAGAA
ACCAGAGCTCTTGGATATGCTGCTGATCACAAACAATCCCTACGACTATT
GCTACATCTCCCAGGGAGAAGTGACCGTCTCCTCTATCAACGACTCCGAG
GAGCTAATGGCCACGGACAGTGCCTTTGACNTGCTTGGATTCACTCCTGAG
GAGAAGATGGGCGTGTACAAGCTAATAGGAGCCATCATGCATTACGGCAA
CATGAAGTTTAAACAGAAACAGCGTGAGGAGCAGGCAGAGCCAGACGGAA
CCGAANCAGCTGATAAGTCAGCATACCTGATGGGTCTAAACTCTGCTGACC

TCATCAAAGGACTGTGCCATCCCAGGGTCAAGGTAGGAAATGAGTATGTC
ACCCAAGGACAAAGTGTAACCCACGTCTACTACNCCAACAAGGAGGCTTTC
AAGTGCGACGAGTGTGGCAAGCACTACAACACCAAGCTGGGCTACAAGCG
CCATGTGGCCATGCACTCGGCCACGGCAGGGGACCTCACGTGCAAGGTGT
GCATGCAGAGCTACGAGAGCACCCCCGTCCTCCTGGAGCACCTCAAGAGC
CACTCGGGAAAGTCGTCGGGCGGCGCCAAGGAGAAAAAGCACCCGTGCGA
CCACTGCGACCGCCGCTTCTACACCCGCAAGGACGTGCGGCGGCACATGG
TGGTCCACACGGGCCGCAAGGACTTCCTGTGCCAGTACTGCGCCCAGCGC
TTCGGCAGGAAGGACCACCTGACGCGGCACGTGAAGAAGAGCCACTCGCA
GGAGCTGCTGAAGATCAAGACGGAGCCGCCTGACATGCTGGGCCTCTTG-
-----GGCCCCTCCCCCTGCTCCGTCAAAGAGGAGCTGAGCCCCATGATG
TGCAGCATGGCCCCCAGTAAGGACCCCCTGATGGGCAAGCCCTTTCCCAG
CGGGACCCCCTTCCCCATGGGCATGTACAACCCCCAC---------CTCC
AGGCCATGTCCAACTCAGGGGCGGCCCACCCC------CACCCCTCCCTG
ATGCCTNNNNNNNNNNNNNNNNNNNNNNNNNNNNNNNNNNNNNNNNNNNTATCTCATATATGCGTCCTTCT
CGTTCATGGGATGTTTACAAAT
CAGCGATGGATCGAACATTGTGAATTTGCTGGCCAGTAACTCTCCAAGCG
TGTCCTACGCCCTGACCCAGCAGAAGTACTTCAGCAACTACAGTCCGGTG
ATCGGGTTCTACATTTACGAACCCATTGAGTACTGGAACTCTACGGTGCA
GGAGCACCTGAAGACGCTGAGTCATGGCTTCAACAAGATCTCCTGGATGG
ACAACTTCTTCCACTACCTGCGGGTGGTGAACGTGAGTGCGTCAACAAAG
AACGACTTCATCMACATACTCAAGGGCTCCTTCTTGCGCAGTCCGGAGTA
CCAGCACTTCACAGAGGACATCATCTTCTCCAAGA---ACCGTGACAGTG
------ACGAGTACGACATCATCGCCTCGCGGATGTATCTGGTGGCGCGC
ACCACAGAGAAGAAGCGCGAGGAGGTTGTGGAGCTCCTGGAAAAGCTGCG
TCCGCTAATGCTGATCAACAGCATTAAATTCATCGCCTTCAACCCCACTT
TTGTGTTCATGGACCGTTACAGCTCTTCGGTAATCTCGCCCATACTGACT
TCAGGCTTTAGCGTGTTAACCATCCTCATCCTCACCTTCTTCCTGGTCAT
CAACCCCTTGGGGAACTTCTGGCTCATATTGACAGTCACTTCTGTGGAGT
TAGGGGTCCTGGGTTTGATGGGCTACCACCCATTTGAGTGGCAGCCTGCG
CTTAAGAACGTGTCCGCCTCCTGCCACGTGGGCATTATCAACGGGCTCTC
TGGTTGGGCCGCTTCCGTGGATGACGTCCCCATCGAGACCATCACTCGGC
GGTTCCGCTACGACGTAGCCCTGGTGTCGGCCATGAAGGACCTGGAGGAG
GACATCATGGAGGGCCTGACAGAACATGGCCTGGACGACAGCTCATGCAA
CACGGGCTTTGACGTCATGATAAAGGAGTCCTGCGACGGCATGGGAGATG
TCAGCGAGAAGCACGGCGGGGGCCCGGCTATCCCAGAGAAGGCTGTGCGC
TTCTCGTTCACCATCATGTCGGTATCCGTGCTGCCTGAAGGGAAGGAC--
----------------------------GAGACGGTGACCGTTTTCAGGG
AGCCAAAGCCAAACTCTGAGTTGTCTTGTAAGCCCCTGTGCCTGATGTTT
GTGGATGAGTCGGACCACGAGATGCTCACCGCCGTCCTGGACCCCCTGCT
GGCCGAGAGGGCCGCCATGAAACACAGCCGGCTCATCTTATCCATAGGTG
GCCTGCCCCGGTCCTTCCGCTTCCATTTCCGAGGCACCGGCTACGATGAG
AAGATGGTGCGCGAGATGGAGGGCCTGGAGGCCTCTGGCTCCACGTATAT
CTGCACCCTGTGTGACTCCACCAGAGCGGAGGCGTCCCACAACATGGTGC
TCCACTCTGTAAGCCGCAACCACGATGAGAACCTGGAGCGCTATGAAATC
TGGAGGACCAATCCCTTCTCGGAGTCCGCCGAAGAGCTGCGGGACCGGGT
GAAAGGGGTTTCCGCCAAGCCGTTCTTGGAGACCCATCCCACTCTGGATG
CGCTGCACTGCGACATCGGCAACGCCACTGAGTTTTACAAGATCTTCCAG
GATGAGATTGGGGAGGTGTTCCGGAAGCC---CAAC---CCCACACGGGA
GGAACGACGAAGCTGGAGGGCAACGCTGGACAAGCAGCTGCGGAAGAAGA
TGAAGCTCAAACCGGTGATGAGGATGAACGGGAACTACGCCCGCAAGCTG
ATGACCGAGGACGCTGTTGATGTAGTCTGTGAGCTGGTGCCCTCCGACGA
ACGCCGGCAGGCTCTGCGAGAGCTCATGGGCCTCTACCTACAGATGAAAC

CTGTCTGGCGTGCCACCTGCCCTGCTAAAGAGTGCCCAGACCAGCTGTGC
CGCTACAGTTTCAACTCGCAACGCTTTTCCGAACTCCTCTCCTCCACCTT
CAAGTACAGGTACAATGGAAAGATTACCAATTACCTGCACAAAACGCTAG
CTCATGTTCCTGAAATCATAGAGAGAGACGGCTCTATTGGAGCCTGGGCT
AGCGAGGGGAATGAGTCGGCCAACAAATCATACACCATTGAAATGGGTCT
CAAAGGGCCACAGTGGAAGGAAAGTCCTCAGCCTTTCTCGTGCTCCATTG
AAGACCCCACTAAACAAACTAAGTTCAAGGGCATCAAAACGTACATTTCG
TACAGGGTCACGCCGAGCCACACGGGGCGACCCGTTTACAGACGCTACAA
GCATTTTGATTGGCTGTACAACCGCCTGCTGCACAAGTTCACTGTGATAT
CGGTACCCCACTTGCCCGAGAAGCAGGCCACAGGGCGCTTCGAGGAGGAC
TTTATTGAAAAGCGCAAACGGCGACTTGTCCTGTGGATGAACCACATGAC
AAGTCACCCGATCCTCTCCCAGTACGAGGGCTTTGAGCACTTTCTCATGT
GTGCAGATGACAAGCAGTGGAAGCTGGGCAAGAGGCGCGCAGAGAAGGAT
GAGATGGTGGGCGCTCATTTTATGCTGACCTTCCAGATTCCTAGCGAGCA
TCAGGACCTACAGGATGTCGAGGAGCGCATCGACTCCTTCAAGTCTTTCG
CTAAGAAGATGGACGACAGCGTCATGCAGCTCACGCACGTCGCATCGGAG
CTGGTGCGTAAGCACCTCGGAGGGTTCAGGAAGGAGTTCCAGCGGTTGGG
AAATTCGTTCCAATCGATCAGCCAGGCATTCACGCTGGACCCTCCTCACG
GCTCTGATGCCCTAAACAGCGCCATCTCCCAC------------------

-------------------GCCAAATCCCGATTTCACCCTGGCGTAGGGA
CTGGTCCTGGCACTGAGC---GTAGCGTCCCACTCAGCAACAGCTTGCTG
TCTCCGCAACAAACCGAGGAGCCCACTGTTG---CCTCCCCACAGCGATG
GTTTGTCACCC---CTGCCAACAACCGACTGGACTTCGCTGCTTCGGCAT
ACGATGCCGCT---------GATTTCGCCGGCAACGCCGCCACGTTGCTA
TCCTACGCAGCGGCTGGAGTGAAGGCGC------TTCCCTTGCCGGCTGC
AGGTTGCTCTAACAGGCCTCTTGGGTATTACGCAGACCCATCTG---GCT
GG---GGCGCTCGTACTCCGCCGCAGTACTGTACC------AGTAAACCA
AGCACGGTGCTCTCCTGCTGGCCA---AATTCTGTTAGCGGCAGAGCGGG
CT------CAAACTATCTGG---------CTGAGGA---CGGA---GATG
C---CATTCCCACCGAGAGGTCCCCG---AT---TGGTGGGACCGAGGAG
---ACAAAACCCAAAGACCT------GTCAGA---ATCCAGCTGGATAGA
G---ACCCCGTCTTCGATCAAATCAATCGATTCTAGCGATTCTGGGATCT
TTG---AACAAGCCAAACGGAGAAGAATTTCTCCTTCAGCCACACCG---
------GTTGCAGAAACTGTGTCCCCGTTAAAATCAGAGCATCACTCAAC

AGGCGAAGTCACAGAACGAGAAGTAGCTTTGGGGATAAATCCGTTCGCGG
ACGGGATGGGAGCCTTCAAGATCAACCACAGTTCCCATGATATCGGCTCC
GG---ACAGACAGCGTTTTCCACACAGGCG---CCCGGGTAC---GCAGC
GGCGGCGCTGGGA---CATCATCA------TCACCCCTCACACGTCAGCT
CT---TACTCCACGGCGGCGTTCAACTCTACCCGGGACTTTCTCTTCAGA
AATCGGGGCTTTGGAGACGCCGCGAG------------TGCTCAGCACAG
TTTATTTGCATCCGC---CGCGGGAAGTTT---C------GCAGGGCCAC
ATGGACACTCAGATGCCGCAGGGCACCTTCTCTTCCCGGGGCTCCACGAG
---CAAGCCACTACGCATTCATCTTCTAACG---TCAACAGCCAAATGAG
GCTTGGCTTTTCCGGGGACATGTACGGCCGGGCTGACCAGTACGGGCACG
TAACCAGCCCGAGGT---CCGACCACTATGCTTCTACTCAATTGCACGGT
TATGGGCCTATGAATATGAACATGGCCGCG---CACCATGGAGCGGGGGC
CTTCTTTCGGTACATGAGGCAGCCGATCAAACAGGAGCTCATCTGCAAGT
GGATCGAACCGGAGCAATTGACGAACCCAAAAAAGTCGTGTAACAAAACT
TTTAGCACAATGCATGAGCTGGTGACCCACCTGACTGTAGAGCATGTGGG
GGGCCCAGAACAGTCGAACCACGTATGTTTCTGGGAAGATTGCTCTCGAG
AAGGAAAACCGTTCAAAGCTAAATACAAACTTGTCAATCACATTCGAGTG
CACACCGGAGAGAAACCATTTCCATGCCCATTCCCCGGCTGTGGCAAA
>Chromis cyanea
AGCCTCCTCATTCGGGCAGAACTAAGCCAACCAGGCGCTCTCCTTGGAGA
CGACCAGATTTATAATGTAATTGTTACAGCACACGCCTTTGTAATAATTT
TCTTTATAGTAATGCCAATTATAATCGGGGGGTTCGGAAACTGACTTATC
CCTCTTATGATTGGAGCCCCAGATATAGCATTCCCCCGAATAAATAACAT
AAGCTTTTGACTTCTACCACCCTCATTCCTTCTCCTACTCGCCTCCTCTG
GTGTTGAAGCAGGTGCCGGAACCGGGTGGACCGTCTATCCCCCCTTATCA
GGAAACCTAGCTCACGCAGGAGCCTCCGTAGACTTAACTATTTTCTCTCT
CCACTTAGCAGGGATTTCCTCAATTCTGGGGGCAATCAATTTCATTACCA
CCATTATTAACATGAAACCCCCAGCCATCTCTCAATATCAAACCCCTCTC
TTTGTATGNGCTGTTCTGATTACCGCCGTTCTTCTCCTCCTATCCCTTCCA
GTCCTAGCTGCTGGCATCACCATGCTCTTAACCGACCGAAACTTAAACAC
CACATTTTTCGACCCTGCAGGAGGGGGAGATCCTATCCTCTACCAGCACT
TATTC---

-----------TTCCTAGAGAGAAACCTCCACCCGTCCAACTGTCTTGGC
ATGCTGTTGCTGTCTGAYGCCCACCAGTGCACCAAGCTGTCAGAGCTCTC
CTGGGGCATGTGTCTCAGCAACTTCCCCGCAATTTGCAAGACAGAGGACT
TCCTCCAACTGCCCAAAGACATGGTGGTGCAGCTTTTGTCACATGAAGAG
CTAGAGACTGAAGATGAGAGACTGGTTTATGAAGCTGCCCTCAACTGGAT
CAACTACGACCTGGAAAGGAGGCACTGCCATCTTCCAGAGCTCCTGAGAA
CAGTCCGCCTTGCCCTGCTGCCKGCCATCTTTTTAATGGAGAAYGTCTCA
ACAGAGGAGCTGATCAACGCCCAGGCTAAGAGCAAGGAGCTGGTGGATGA
AGCCATCCGCTGTAAGCTGAAGATCCTGCARAATGATGGTGTCGTCAACA
GCCCATGTGCTCGACCAAGAAAAACCAGCCATGCCCTCTTTCTTCTGGGT
GGGCAGACTTTCATGTGCGACAAGTTGTACCTGGTTGACCAGAAGGCCAA
AGAGATCATCCCGAAGGCTGACATCCCCAGTCCCAGGAAGGAGTTCAGCG
CCTGTGCCATTGGCTGTAAGGTGTACATCACCGGTGGGA--GGGGCTC-A
GAGAATGGTGTGTCCAAAGATGTGTGGGTCTACGACACTGTCCATGAAGA
ATGGTCAAAGGCGGCACCCATGCTCATYGCCAGGTTCGGCCATGGCTCAG
CAGAGCTGAAACACTGCCTCTACGTCGTAGGAGGCCACACTGCAGCAACC
GGCTGCCTCCCAGCTTCTCCGTCAGGATGAATACATTGTTGTGTTTAGTC
GTTCAACAACAAGGCTGATATTGAATGAAGCAGAGCTTATCATGGCACTG

GCCCAGGAGTTCCAAATGAGAGTGGTCACAGTGTCCTTAGAGGAACAATC
TTTCCCCAGTATCGTCCAGGTGATTAGCGGTGCTTCCATGTTAGTCAGCA
TGCACGGAGCTCAGCTTATCACCTCACTCTTTCTTCCCAGAGGAGCTGCT
GTGGTGGAGTTGTTCCCTTTTGCTGTGAACCCAGAGCAGTACACCCCATA
TAAAACCCTTGCCTCTCTTCCGGGCATGGACCTTCACTATGTCTCCTGGA
GGAACACGAAGGAGGAGAACACTATGACCCACCCAGACAGACCCTGGGAG
CAAGGAGGCATTGCTCACTTGGAAAAAGACGAGCAGGAACGAATCCTGGC
CAGTACGGACGTCCCCAGGCACCTGTGCTGCCGCAATCCAGAGTGGCTCT
TCCGGATCTATCAGGACACTCTGGTGGACATCCCATCCTTACTGGAGGTC
CTCAA---AGAAGCCATGAAG---ACCAAGCCCAACTTGAAGAA---GGC
CAAGGCAGCAAGCACAGTCCACCCAGGCCAGGTCAGAGAGCCCCAGTGTC
AAACTTCAGTACAAACCTCTAATGAGGCAAAACTTACAGTCTCTTGGCAG
ATCCCGTGGAATCTGAAATACCTTAAGGTGAGAGAGGTAAAGTACGAGGT
GTGGATCCAG--

-----CCCAACAAGGAGGCCTTCAAGTGCGAGGAGTGTGGGAAGCACTAC
AACACCAAGCTGGGATACAAGCGCCATGTGGCCATGCACTCGGCCACCGC
GGGCGATCTGACCTGCAAAGTGTGCATGCAGAGCTACGAGAGCACGCCGG
TTCTCCTGGAGCACCTCAAGAGCCACTCAGGGAAGTCCTCGGGCGGCGCC
AAGGAGAAGAAACACCCGTGCGACCACTGCGACCGTCGTTTCTACACCCG
GAAGGATGTGAGGCGGCACATGGTGGTGCACACGGGCCGAAAGGACTTCC
TGTGCCAGTACTGTGCCCAGCGCTTCGGCAGGAAAGACCATCTGACGCGT
CACGTGAAGAAGAGCCACTCGCAGGAGCTGCTGAAGATCAAGACGGAGCC
TCCGGATATGTTGGGTCTGTTGGCCTCGGGGTCTCCACCCTGCTCCGTGA
AGGAGGAGCTCAGCCCCATGATGTGTGGTATGGGGCCCAACAAAGACCCC
ATGATGGGCAAACCCTTCCCCAGCGGGGCCCCATTCCCGATGGGCATGTA
CAACCCCCACCAT------CTCCAGGCCATGTCTAATTCTGGGGTGGGTC
ACTCA------CACCCGTCCCTGATGCCCGGTTCCCTGTCTGCAGCTATG
GGCATGGGCTGTCACATGGAGTATCTCATCTATGCCTCTTTCTCATTCAT
GGGATGTTTACAAATCAGTGACGGATCGAATATCGTGAACCTGCTGGCTA
GTAACTCTCCGAGTGTTTCATATGCTTTGACCCAGCAGAAATACTTCAGT
AACTACAGTCCCGTGATTGGGTTTTATATTTACGAGCCCATCGAGTACTG
GAACTCCACGGTGCAGGAGCATCTGAAGACTCTGAGTCACGGCTTCAACA
AGATCTCCTGGATGGACAACTTTTTCCACTACCTGCGGGTAGTGAATGTG
AGCGCGTCAACTAAAAGCGACTTTATCAGCATCCTTAAGGGTTCCTTCCT
GCGCAGTCCGGAGTACCAGCACTTCACCGAGGACATCATATTCTCCAAGA
---ACCGCGAGACTG------ATGAGTACGACATTATCGCCTCACGGATG
TACTTGGTAGCACGGACCACAGAGAAGAAGCGCGAGGAGGTGGTGGAGCT
TTTAGAAAAGCTTCGTCCCTTGATGCTGATAAACAGCATCAAATTCATTG
CCTTTAATCCCACGTTTGTGTTCATGGACCGCTACAGCTCCTCCGTCATC
TCACCTATCCTGACCTCAGGCTTCAGTGTACTCACCATCCTCATCCTCAC
TTTCTTCCTGGTCATCAACCCCTTGGGGAACTTCTGGCTCATCCTCACGG

TTACGTCCGTGGAGCTGGGCGTCTTGGGTTTGATGGGCTTTCACCAGTTT
GAATGGCAGCCAGCTCTCAAGAATGTGTCTACATCTTGCAACGTTGGCAT
TATTAACGGGCTCTCTGGATGGGTTTCCTCGGTGGATGACTCCCCGGCTG
ACACAATCACTCGGCGCTTTCGTTATGATGTGGCCCTGGTGTCTGCATTA
AAGGATCTGGAGGAGGACATCATGGACGGGCTGAGAGAGAGTGGGATGGA
AGACAGCGCTTGCACCTCAGGCTTTAGTGTCATGATCAAGGAATGTTGCG
ACGGTATGGGTGATGTCAGCGAGAAGCACGGCGGAGGACCTGCTGTTCCT
GAGAAGGCTGTACGCTTCTCTTTCACCATTATGTCTGTCTCTGTCCTCGC
AGATGATAAGAAG------------------------------GAGGAGG
TTACCATTTTCACTGAGCCAAAACCAAACTCAGAGCTGTCCTGTAAGCCC
CTCTCCCTGATGTTCGTGGATGAGTCAGACCATGAGACACTCACAGCTGT
CCTGGGGCCCATAGTTGCAGAGCGGAATGCAATGAAAGAGAGCCGACTCA
TCCTATCCATCGGCGGCCTGCCTCGATCCTTTCGCTTCCACTTCAGGGGC
ACGGGGTATGATGAGAAGATGGTGCGTGAGATGGAGGGCCTGGAGGCCTC
GGGGTCCACGTATGTCTGCACTCTATGTGACTCCACTCGTGCAGAAGCCT
CGAAAAACATGGTCCTTCACTCAGTCACTCGCAGTCACGAGGAGAATCTA
GAACGATATGAGATATGGAGAAGCAACCCCTTCTCTGAGTCTGCAGACGA
GCTGCGGGACAGAGTCAAAGGGGTCTCCTCCAAGCCCTTCCTGGAGACCC
AGCCCACGCTAGATGCCTTACACTGCGACATTGGCAACGCCAGTGAATTC
TACAAAATCTTCCAGGATGAGATCGGGGAAGTATACAAAAAGGT---CAA
C---CCCACCCGGGAGCAACGGCGCAGCTGGAGGGCAGCGCTGGATAAAC
AGCTGCGGAAGAAGATGAAGCTTAAACCGGTAATGAGGATGAATGGGAAC
TACGCCCGCAGGCTAATGACCCAGGAGGCTGTGGAGGTGGTGTGTGAGCT
GGTGCCCTCTGAGGAGAGGAAGGAGGCCCTGAGGGAGCTCATAAGGCTCT
ACCTTCAGATGAAGCCTGTGTGGCGTGCCACCAATCCGGCCAAGGAATGC
CCTGACCAGCTGTGCCGCTACAGCTTTAACTCCCAGCGCTTTGCCGACCT
CCTCTCAACCTCCTTCAAATATAGGTACAACGGGAAGATAACCAATTACC
TGCACAAGACCCTGGCCCATGTGCCTGAAATCATAGAGAGAGACGGATCC
ATAGGAGCCTGGGCGAGCGAGGGGAACGAGTCAGCAAACAAATCGTACAC
CATTGAGATGGGTCCCCAGGGGCCCTGGTGGAAGGAGAACCCACAGCCTT
TCTCCTGCTCCATTGAAGAYCCCACCAAACAGACAAAGTTCAAAGGCATC
AAGACATACATTTCATACCGGGTCACGCCGAGCCACACGGGGCGTCCKGT
CTACAGGCGCTACAAACACTTTGACTGGCTGTACAACCGTTTACTGCACA
AGTTCACTGTGATCTCTGTTCCCCACCTGCCTGAGAAGCAGGCCACGGGG
CGCTTTGAGGAAGACTTCATCGAGAAGCGCAAGAGGCGACTGATACTGTG
GATGAACCACATGACCAGTCACCCAGTCCTCTCCCAGTACGAAGGCTTTG
AGCACTTTCTGATGTGCGCTGACGACAAGCAGTGGAAACTGGGCAAGAGA
CGGGCAGAGAAGGACGAGATGGTGGGCGCACATTTCATGCTGACACTCCA
AATCCCCAACGAGCAYCAGGACCTTCAGGATGTTGAGGAGCGGGTCGACA
ACTTCAAGGCCTTTGCCAAGAAAATGGACGACAGCGTGATGCAGCTGACA
CACGTGGCCTCGGAGCTGGTGCGTAAACACCTGGGTGGATTCAGGAAGGA
GTTCCAGCGGCTGGGAAACGCCTTCCAGTCTATCAGCCAGGCTTTCATGC
TGGACCCTCCCCATAGGTCAGACACCCTCAACAACGCCATCTCCCATNNNNNNNNNNNNNNNNNNNNN
NNNNNNNNTGG
GTTTTATCATCGGAGTCGGCGTGGTTGGAAACCTCCTGATCTCCATCCTG
CTGGTCAAAGACAAGAGCCTGCACCGAGCGCCCTACTATTTCCTGCTGGA
CCTGTGCGCCTCYGACATCCTGCGCTCTGCCATCTGCTTCCCCTTCGTCT
TCACCTCGGTCAAGAATGGATCTGCCTGGACGTACGGCACRCTGACCTGC
AAAGTGATCGCCTTCCTGGGTGTGCTCTCCTGTTTCCACACGGCGTTCAT
GCTMTTCTGCGTCAGCGTCACACGCTACCTGGCCATTGCACATCACCGTT
TCTACACCAAGAGGCTGACTTTCTGGACGTGCTTGGCTGTCATCTGCATG
GTGTGGACGTTGTCTGTRGCRATGGCATTYCCGCCGGTGCTAGACGTAGG
GACGTACTCTTTCATCCGAGAGGAGGACCAGTGCACCTTCCAGCACCGCT

CCTTCAGGGCGAACGATTCATTGGGATTCATGCTCCTGCTGGCGCTCATC
CTCCTGGCCACACAGCTGGTTTACCTCAAGCTCATCTTCTTCGTCCACGA
CCGTCGAAAGATGAAGCCCGTCCAGTTCGTGCCTGCTGTCAGTCAGAACT
GGACCTTCCACGGGCCAGGCGCCAGCGGGCAGGCGGCGGCCAACTGGCTG
GCTGGATTTGGTCGAGGCCCCACTCCGCCTACTCTGCTGGGCATCCGGCA
GAACAGCAACGCAGCGGGCCGCAGGCGYCTACTGGTTTTGGATGAGTTCA
AAACGGAGAAGAGGATTAGTAGGATGTTCTACATCATGACGTTTTTCTTC
CTGGCGCTGTGGGGGCCCTATCTGGTCGCCTGCTACTGGCGGGTGTTTGC
AAGGGGCCCTGTGGTCCCTGGAGGCTACCTGACAGCAGCCGTGTGGATGA
GCTTCNNNNNNNNNNNNNNNNNNNNNNNNNNNNNNNNNNNNNNNNNNNNNNNNGCCAAATCTCGCTTTCA
CCCTGGCGTGGGGACTGGTCCTGGCACG
GAGC---GCAGCGTCCCACTCGGCAACAGCTTGCTATCCCCGCAGCAAAG
CGAGGAGCCCACTGTTGCCACCCCCCCGCAGCGATGGTTTGTCACCC---
CTGCCAACAACCGACTGGACTTTGCTGCCTCGGCATACGACGCCGCT---
------GATTTCGCCGGTAACGCGGCCACCTTGCTGTCCTACGCAGCGGC
CGGAGTTAAGGCTC------TTCCCCTGCCGACTGCAGGCTGCTCCAACC
GGCCTCTTGGCTATTACGCAGACCCGTCTG---GCTGG---GGAGGACGC
ACGCCGCCGCAGTACTGTGGCGTAAATAGTAAATCCAGCTCGGTCTTTTC
CTGCTGGCCCGCCAACTCCATCGGTGGCAGGGCCGGCG---CC---AACT
ACCTGT---------CCGAGGA---GGGA---GACTC---CATCACGACG
GAGAGGTCACCG---AT---CGGCGCGTCGGAGGAG---ACCAAACCCAA
AGACATGAC---GTCCGA---GTCGAGCTGGATAGAG---ACGCCGTCCT
CCATAAAGTCCATCGATTCCAGCGACTCTGGGATCTTTG---AACAGGCC
AAACGGAGGAGAATCTCACCTTCTGCCACGCCG---------GTTTCAGA
GACAGTGTCCCCGTTAAAATCCGAGCATCACTCAACAGGCGAAGTCACAG
AGCGAGAAGTGGCGTTGGGGATAAATCCGTTCGCAGATGGGATGGGCGCC
TTCAAAATCAACCACAGCTCCCACGATATTGGCTCCGG---ACAGACGGC
GTTTTCCTCCCAGGCG---CCCGGTTAC---GCAGCAGCCGCCTTGGGA-
--CACCATCA------CCACCCGACCCACGTTGGCTCT---TACTCCACG
GCGGCGTTCAACTCCACCAGGGACTTTCTGTTCAGAAATCGGGGTTTCGG
GGATGCCACCGG------------CGCGCAGCACAGTTTGTTCGCCTC--
-------GGGAAGTTT---C------GCAGGGCCACACGGACACTCAGAT
GCGGCGGGGCACCTGCTCTTCCCGGGGCTCCACGAG---CAGGCGGCGAG
CCACGCGTCTTCCAACGTGGTCAACAGCCAGATGCGGCTGGGCTTCTCGG
GGGACATGTACGGACGCGCCGAGCAGTACGGCCACGTTACGAGCCCCAGG
T---CCGACCACTACGCCTCGACCCAGCTGCACGGCTACGGCCCCATGAA
CATGAATATGGCCGCG---CACCACGGCGCAGGGGCCTTCTTTCGGTACA
TGAGGCAGCCGATCAAGCAAGAGCTCATCTGCAAGTGGATCGAGCCGGAG
CAGCTGACGAATCCCAAAAAGTCGTGCAACAAAACTTTCAGCACGATGCA
CGAGCTGGTGACCCATCTGACGGTGGAGCATGTGGGGGGACCGGAGCAGA
CCAACCACATTTGCTTCTGGGAGGACTGTGCCCGAGAAGGGAAGCCGTTC
AAAGCCAAATACAAACTTGTGAATCATATCAGAGTACACACCGGAGAAAA
GCCCTTTCCGTGTCCGTTCCCCGGCTGTGGCAAA
>Coccorella atlantica
AGCCTTCTCATCCGAGCAGAGCTGAACCAACCCGGTGCCCTCTTAGGGGA
CGACCAGATCTACAATGTAATCGTCACCGCTCACGCCTTCGTAATAATCT
TCTTTATAGTAATGCCAATTCTAATCGGCGGATTCGGAAACTGACTAGTC
CCCCTGATACTCGGCGCACCCGACATGGCATTCCCACGCATGAACAACAT
AAGCTTCTGGCTTCTTCCCCCCTCCTTCCTGCTCCTCCTAGCCTCTTCCG
CCGTTGAAGCGGGGGCCGGCACAGGCTGAACTGTTTATCCGCCCCTTGCC
AGCAACCTGGCTCACGCCGGAGCTTCTGTTGACCTGACAATCTTCTCACT
CCATCTGGCCGGTATTTCCTCCATTCTAGGCGCCATTAATTTCATTACAA
CCATCATTAACATGAAACCCCCAGCTATTACCCAATACCAAACCCCCCTA

TTTGTGTGAGCAGTACTTATTACTGCCGTCCTTCTCCTCCTCTCTCTCCC
TGTTCTAGCAGCAGGGATTACAATACTCCTCACAGACCGAAACTTAAACA
CAACCTTCTTTGACCCCGCAGGCGGGGGTGACCCAATTCTTTACCAACAT
CTATTTTGATTCTTTGGCCACCCAGAAGTCTATATCCTCATCCTCCCCGG
TTTTGGTATTATTTCTCACGTTGTTGCCTACTATGCAGGAAAAAAAGAGC
CCTTTGGCTACATGGGAATGGTCTGAGCTATAATAGCAATCGGCCTCCTG
GGGTTTATTGTCTGAGCCCCCCACATATTTACCGTCGGCATAGACGTTGA
TACACGCGCCTATTCCTCGAGAGGAACCTGCACCCGTCCAACTGCCTTGG
CATGCTGCTGCTGTCAGACGCCCACCAGTGCACCAAGCTGTCGGAGCTGT
CCTGGGGTATGTGCCTCAGCAACTTCCCTGCCATCTGCAAGACGGAGGAC
TTCCTCCAGTTGCCCAAAGACATGGTGGTGCAGCTCCTGTCCCACGAAGA
GCTGGAGACGGAAGACGAGAGACTGGTTTATGAGGCCGCCCTTAACTGGG
TCAACTATGACCTGGAGAGGAGACACTGCCACCTGCCGGAGCTGCTGAGA
ACCGTACGCCTGGCCTTGCTTCCTGCCATCTTTCTCATGGAGAACGTCTC
CACAGAGGAGCTGATAAATGCCCAGGCAAAGAGCAAGGAGTTGGTGGATG
AGGCCATCCGTTGCAAGCTGAAGATCCTGCAGAACGACGGTGTGGTCAAC
AGTCCCTGTGCCCGGCCCAGAAAGACCAGCCACGCCCTTTTCCTGCTGGG
AGGGCAGACCTTCATGTGTGACAAGCTGTACTTGGTGGACCAGAAGGCCA
AAGAGATCATCCCCAAGGCGGACATCCCCAGCCCCAGGAAGGAGTTCAGC
GCCTGTGCCATCGGCTGCAAGGTGTACGTCACGGGCGGGA--GAGGCTC-
AGAGAACGGCGTCTCTAAGGACGTGTGGGTCTACGACACCGTCCACGAGG
AGTGGTCCAAGGCGGCGCCGATGCTCATCGCCCGGTTCGGCCACGGCTCT
GCCGAGCTCAAACACTGCCTCTATGTGGTCGGAGGACACACTGCGGCCAC
CGGCTGCCTCCCCGCCTCTCCGTCCGGACGAGTACATCGTAGTGTTCAGC
CGTTCCACAACGCGGCTGATTCTGAACGAAGCGGAGCTGATACTGGCACT
GGCCCAGGAGTTCCAGATGAGGGTGGTCACGGTGTCCCTGGAGGAGCAGT
CCTTCCCCAGCATCGTGCAGGTCATCAGCGGGGCCTCCATGTTGGTCAGC
ATGCACGGAGCTCAACTCGTCACCTCGCTCTTCCTCCCCAGAGGAGGCGT
CGTGGTGGAGCTCTTCCCCTACGCCGTGAACCCGGAGCAGTACACCCCAT
ACAAGACCCTAGCCTCCCTGCCGGGCATGGACCTCCAATACGTTTCCTGG
AGGAACACTATTGAGGAGAACACTGTCGCCCACCCAGACAGAGCCTGGGA
CCAAGGAGGCATTGCCCATTTGGAGAAAGAAGAGCAGGAGAGAATCCTAG
CCAGCAAGGATGTCCCCAGGCACCTATGCTGCCGCAACCCAGAGTGGCTC
TTCAGGATCTACCAGGACACTCTGGTGGACATCCCCTCCTTGCTGGAAGT
CCTCAA---GGAGGGCCTGAAG---ACGAGGCCGAGCTTGAAGAA---GT
CCAAGCCGGCCAGCACGGTTCACCCGGGCCGGGTCCGAGAACCCCAGTGC
CAGACTTCGGTCCAAGCCACCAACGAGGCTAAACTCACAGTGTCCTGGCA
GATCCCGTGGAACCTTAAGTACCTGAAGGTGCGAGAGGTGAAGTACGAAG
-----------AAAAAGGATACAAGCAAGGGAACCCTGGAGGATCAAATC
ATTCAGGCAAACCCTGCGCTGGAGGCTTTTGGTAACGCCAAAACACTGAG
GAACGATAATTCCTCCCGTTTTGGAAAATTCATCCGAATCCACTTCGGAA
CCAGCGGTAAATTGTCCTCAGCGGACATTGAGACCTACCTGCTGGAGAAG
TCACGGGTCACCTTTCAGCTCAAGGCGGAGAGGAACTACCACGTCTTCTT
CCAGATCTTGTCCAATCAAAAGCCAGAGCTGCTGGACATGCTGTTGATCA
CCAACAACCCTTACGACTACTGCTACATCTCCCAAGGAGAAGTAACCGTA
GCGTCCATCAACGACTCAGAAGAGCTGATGGCCACCGACAGTGCCTTCGA
TGTGCTCGGCTTCACGCAAGAGGAGAAGATGGGAGTCTACAAGTTGGTAG
GGGCCATTATGCACTATGGCAACATGAGGTTCAAGCAGAAGCAGCGCGAG
GAACAGGCAGAGCCTGATGGCACAGAAGCTGCCGATAAGTCGGCTTATCT
AATGGGGCTGAATTCAGCAGACCTAATCAAATGTCTCTGCCATCCCAGAG
TCAAGGTAGGAAATGAGTATGTCACCAAAGGCCAGGGTGTAGATCAAGTC
TACTAC--------GGAGGCGTTCAAGTGCGAGGAGTGCGGCAAGCATTA
CAACACCAAGCTGGGCTACAAGCGCCATGTGGCCATGCACTCCGCCACCG

CGGGGGACCTCACCTGCAAGGTGTGCATGCAGAGCTACGAGAGCACCCCC
GTGCTGCTGGAGCACCTCAAGAGCCACTCGGGGAAGTCGTCGGGCGGCGC
CAAGGAGAAGAAGCACCCGTGCGACCACTGCGACCGCCGCTTCTACACGC
GGAAGGACGTCCGGCGGCACATGGTGGTCCACACGGGCCGCAAGGACTTC
CTGTGCCAGTACTGCGCCCAGCGCTTCGGCAGGAAGGACCACCTGACGCG
CCACGTGAAGAAGAGCCACTCGCAGGAGCTGCTGAAGATCAAGACGGAGC
CTCCGGACATGCTGGGCCTCCTGGGCTCCGGCTCCCCGCCCTGCTCCGTC
AAGGAGGAGCTGAGCCCCATGATGTGCAGCATGGGTCCCAACAAAGACCC
CATGATGGGCAAGCCCTTCCCCAGCGGGACGCCCTTCCCCATGGGCATGT
ACAACCCCCACCAC------CTCCAGGCCATGTCTAACCCCGGGGTGGGC
CACCCC------CACCCCTCCCTCATGCCCGGTTCCCTGTCCGCASCCAT
GGGCATGGGCTGTCACATGGAGTATCTCATCTACGCTTCTTTCTCCTTCA
TGGGATGTTTACAGATCAGCGACGGCTCGAACGTGGTGAACCTGCTGGCC
AGTAACTCTCCGAGCGTCTCGTACGCCCTGACGCAGCAGAAGTACTTCAG
CAACTACAGCCCCGTGATTGGGTTCTACATCTACGAGCCCATCGAGTACT
GGAACTCCACGGTGCAGGAGCACCTGAAGACGCTCAGCAATGGCTTCAAC
AAGATCTCCTGGGTGGACAACTTCGTCCACTACCTACGGGCGGCAAACGT
GAGCGCGTCCACCAAGGCCGACTTCGTCGCCGTCCTCAAGGGCTCCTTCC
TGCGGAGCCCTGTGTACCAGCACTTCACCGACGACATCATCTTCTCCAAG
A---GCCACGAGAACA---GCGACGACTACGACATCATTGCCTCGCGCAT
GTACCTGGTGGCGCGCACCACGGAGAAGCGGCGCGAGGACGTGGTGGAGC
TGCTGGAGAAGCTCCGGCCGCTGATGCTGATCAACAGCATCAAGTTCATT
GCCTTCAACCCCACGTTCGTCTTCATGGACCGCTACAGCTCCTCGGTCAT
CTCGCCCATCCTCACCTCGGGCTTCAGCGTGCTCACCATCCTCATCCTCA
CCTTCTTCCTGGTCATCAACCCGCTGGGGAACCTCTGGCTCATCCTGACG
GTCACGTCCGTGGAGCTGGGCGTGCTGGGCCTGATGGGCTACCACGCGTT
CGAGTGGCGGCCGGCCCTCCAGAACGTGTCCCCGTCCTGCCACGTGGGTA
TCATCAACGGGCTTTCCGGGTGGGCCGCGTCGGTGGACGAGGTCCCCGCC
GACACCATCGCCCGTCGGTTCCGCTACGACGTGGCCCTCGTGTCGGCCCT
GAAAGACCTGGAGGAGGACATCGTGGACGGGCTGAGGGAGCACGGGCTGG
CTGAC------TGCGCCTCACGCTTCAGCGTCATGGTCAAGGAGTCCTGC
GACGGCTTGGGAGACGTCAACGAGAAGAACGGCGGCGGGCCGGCGATGCC
CGAGAAGGCCGTGCGCTTCTCCTTCACCGTCATGTCCGTCTCCGTCCACG
CCGACGGAGAGGAGGAGGAGGAGGAGGAG---------------GAGGCG
GTCGTCGTCTTCAGGGAGCCGAAGCCCAACTCCGAACTGTCCTGTAAGCC
CCTGTGCCTGATGTTCGTGGACGAGTCTGACCACGAGACGCTCACCGCCG
TCCTGGGACCTCTGGTGGCGGAGAGGAACGCGATGAAGCAGAGCCGGCTC
GTCCTCCCGGTGGGGGGCGTCCCCCGCTCCTTCCGCTTCCGCTTCCGGGG
CTCGGGCTACGACGAGAAGATGGTGCGCGAGATGGAAGGCCTGGAGGCCT
CGGGCTCCACCTACGTCTGCACCCTCTGCGACGCCACCCGGGCGGAGGCC
TCTCGCAACATGGTGCTCCACTCCGTCACGCGTGGCCACGACGAGAACCT
GGAGCGCTACGAGACGTGGAGGACCAACCCCTTCTCCGAGTCCGCCGCGG
AGCTGCGGGACCGGGTCAAGGGGGTCTCCGCCAAGCCGTTCATCGACACC
CATCCGACGATGGACGCGTTGCACTGCGACATAGGCAACGCCACGGAGTT
CTACAAGATGTTCCAGGACGAGATCGGGGAGGTGTTCCGGAAGGCCGCCA
AC---CCCAGCCGGGAGGAGCGGCGCGGCTGGCGGGCGGCCCTCGACAAG
CAGCTGAGGAAGAGGCTGAAGCTGAAGCCGGTGATGCGGATGAACGGGAA
CTACGCCCGGAGGCTGATGACCGAGGAGGCGGCGGAGGCGGTGTGTGAAC
TGGTGCCCTCGGAGGAGAGGCGGGCGGCGCTGAGGGAGCTGATGGCGCTC
TACGTCCAGATGAAGCCGGTGTGGCGCGCCACCCGCCCGGCCGAGGAGTG
CCCCGACCAGCTGTGCCGCTACAGCTTCAACTCCCAGCGCTTCGCCGAGC
TCCTCTCCTCCACCTTCAAATACAGGTACGACGGAAAGATCACCAACTAC
CTGCACAAGACCCTGGCCCACGTCCCCGAGATCATAGAGAGGGACGGCTC

```
CATCGGGGCCTGGGCCAGCGAGGGGAACGAGTCGGCGAACAAG-------
--------------------------------------------------
--------------------------------------------------
--------------------------------------------------
--------------------------------------------------
--------------------------------------------------
--------------------------------------------------
--------------------------------------------------
--------------------------------------------------
--------------------------------------------------
--------------------------------------------------
--------------------------------------------------
--------------------------------------------------
--------------------------------------------------
--------------------------------------------------
--------------------------------------------------
--------------------------------------------------
--------------------------------------------------
--------------------------------------------------
--------------------------------------------------
--------------------------------------------------
--------------------------------------------------
--------------------------------------------------
--------------------------------------------------
--------------------------------------------------
--------------------------------------------------
--------------------------------------------------
--------------------------------------------------
--------------------------------------------------
--------------------------------------------------
--------------------------------------------------
--------------------------------------------------
--------------------------------------------------
--------------------------------------------------
--------------------------------------------------
--------------------------------------------------
--------------------------------------------------
--------------------------------------------------
--------------------------------------------------
--------------------------------------------------
--------------------------------------------------
--------------------------------------------------
--------------------------------------------------
--------------------------------------------------
--------------------------------------------------
--------------------------------------------------
--------------------------------------------------
--------------------------------------------------
--------------------------------------------------
--------------ACAGGCGAAGTCACAGAAAGAGAAGTGGCTTTGGGG
ATAAATCCGTTCGCGGACGGGATGGGCGCCTTCAAAATAAACCACAGCTC
TCATGACATTGGCTCCGG---GCAAACGGCGTTTTCCTCCCAGGCG---C
CCGGCTAC---GCAGCGGCTGCCCTGGGA---CACCATCA------CCAC
```

```
CCGACCCACGTCAGCTCC---TATTCCACGGCAGCTTTTAATTCCACCCG
GGACTTTCTCTTCAGAAATCGGGGCTTCGGAGACGCCACCAG--------
----CGCGCAGCACAGTCTGTTCGCCTCCGC---CGCGGGAAGTTT---T
------GCAGGGCCACATGGACACTCAGATGCCGCGGGCCACCTGCTCTT
CCCTGGGCTTCATGAG---CAAGCCGCCAGCCATGCGTCGTCTAACGTTG
TAAACAGCCAGATGCGACTGGGCTTTTCTGGGGACATGTACGGGCGGGCC
GACCAGTATGGCCACGTTACAAGCCCACGGT---CCGACCACTACGCTTC
GACCCAACTGCATGGCTATGGCCCTATGAACATGAATATGGCCGCT---C
ACCACGGAGCAGGGGCCTTCTTTCGGTACATGAGGCAGCCGATTAAGCAA
GAGCTCATCTGCAAGTGGATCGAACCGGAGCAACTGACGAATCCCAAAAA
GTCGTGCAACAAAACTTTTAGCACGATGCACGAGCTCGTGACCCATCTGA
CGGTGGAGCATGTGGGTGGACCCGAGCAGTCGAACCACATTTGCTTCTGG
GAAGACTGTGCCCGAGAAGGGAAACCGTTCAAAGCCAAATACAAACTTGT
AAATCATATCAGAGTTCACACCGGAGAAAAGCCGTTCCCGTGTCCATTCC
CCGGCT--------
>Coilia nasus
AGCCTCTTAATTCGAGCAGAACTCAGCCAACCGGGGGCCCTCTTAGGAGA
CGACCAGATCTACAACGTCATTGTTACTGCCCACGCATTCGTAATAATTT
TCTTTATAGTTATACCGGTCATAATCGGAGGTTTCGGAAATTGACTAGTT
CCTCTAATACTTGGAGCACCCGACATGGCATTCCCCCGAATAAACAATAT
AAGCTTTTGACTCCTGCCCCCCTCATTTCTTCTTCTTTTAGCCTCATCTG
GGGTAGAAGCAGGAGCAGGAACAGGATGAACAGTCTACCCGCCCTTGGCA
GGAAACCTAGCTCACGCGGGGGCTTCGGTAGACCTAACAATCTTTTCACT
TCACCTGGCCGGAATCTCATCCATCCTAGGAGCTATCAACTTTATCACAA
CAATCATTAACATAAAACCGCCTGCAATTTCACAATACCAAACACCCTTA
TTTGTCTGAGCCGTATTAATTACAGCAGTACTTTTACTTCTATCCCTCCC
AGTTTTAGCTGCCGGAATCACAATGCTCCTTACAGACCGAAACCTAAACA
CTACTTTTTTCGACCCTGCAGGAGGAGGTGACCCCATTCTCTATCAACAC
TTATTCTGATTCTTCGGACACCCAGAAGTATATATTTTAATTCTCCCAGG
ATTCGGAATTATCTCCCACGTCGTAGCCTATTATGCAGGGAAAAAAGAAC
CTTTCGGCTACATAGGAATAGTGTGAGCTATAATGGCCATCGGACTCCTA
GGCTTTATCGTTTGAGCCCACCACATATTTACAGTAGGAATGGACGTTGA
CACCCGAGCTTA--------------------------------------
--------------------------------------------------
--------------------------------------------------
--------------------------------------------------
--------------------------------------------------
--------------------------------------------------
--------------------------------------------------
--------------------------------------------------
--------------------------------------------------
--------------------------------------------------
--------------------------------------------------
--------------------------------------------------
--------------------------------------------------
--------------------------------------------------
--------------------------------------------------
--------------------------------------------------
--------------------------------------------------
--------------------------------------------------
--------------------------------------------------
--------------------------------------------------
--------------------------------------------------
```


------------------------------------GGCTTCCACGCCTT
CGAGTGGCAGCCGGCCCTGAAGGCCGTGTCCAGCAGCTGGGACGTGGGCA
TCCTGGACGGGCTGTCGGGCTGGACCAGCTCGGTGGAGGACGTGCCGGCG

```
GATACCATCGCCCGGCGCTTCCGCTATGACGTGGCCCTGGTGTCGGCGCT
CAAGGACCTGGAGGAGGACATCTTGGAGGGCCTGAGGGAGCACAGCCTGG
ACGACAGC---TCCGTCACCGGCTTCAGCGTCACCATCAAGGAGTCGTGC
GACGGCATGGGCGATGTGAGCGAGAAGCACGGCAGCGGCCCCGCGGTGCC
CGAGAAGGCCGTGCGCTTCTCCTTCACCGTCATGTCGGTGTCGGCCCACG
TGGAGGGCGAGGAG------------------------------GCCTCG
GTGAGCATCTTCCAGGAGACGAAGCCCAACTCGGAGCTCTCCTGCAAGCC
GCTGTGCCTCATGTTCGTGGACGAGTCGGACCACGAGACGCTGACGGCCA
TCCTGGGCCCTGTGGTGGCCGAACGCAACGCCATGAAGGAGAGCCGGCTC
ATCCTGTCGCTGGCCGGCTTGCCGCGCTCCTTCCGCTTCCACTTCCGGGG
CACCGGCTACGATGAGAAGATGGTGCGCGAGATGGAGGGGCTGGAGGCGT
CCGGCTCCACCTACGTCTGCACGCTGTGCGACTCCACCCGCGCCGAAGCC
TCCAGCAACATGGTGCTGCACAGCATCACACGCAGCCACCAGGAGAACCT
GGAGCGCTACGAGCTGTGGCGCACCAACCCCTTCTCCGAGTCGGCGGACG
AGCTGCGCGACCGGGTCAAGGGCGTCTCGGCCAAGCCCTTCATGGAGACC
CAGCCGACCCTCGATGCGCTGCACTGCGACATCGGCAACGGCACCGAGTT
CTACAAGATCTTCCAGGACGAGATCGGCGAGATGCACGCGCGTGG---CG
ACGTCCCGAGCCGGGAGGAGCGCCGTCGTTGGCGGGCGGCGCTGGACAAG
CAGCTGCGCAAGAAGATGAAGCTGAAGCCCGTGATGCGCATGAACGGCAA
CTACGCCCGCCGCCTCATGACGACCGAGGCGGTGGAGGCGGTGTGCGAGC
TGGTGCCCTCGGAGGAGCGTCGGCAGGCCCTGCGGGAGCTGGTGGCGCTC
TACCTGCAGATGAAGCCCGTGTGGCGCTCCACCTGGCCAGCCCGCGAGTG
CCCCGACCAGCTGTGCCGCTACAGCTTCAACTCGCAGCGCTTCGCCGAGC
TGCTCTCCAGCACGTTCAAGTACCGCTACGACGGCAAGATCACCAACTAC
CTGCACAAGACGCTGGCCCACGTGCCCGAGATCGTGGAGAGGGACGGCTC
CATCGGGGCCTGGGCCAGCGAGGGCAACGAGTCCGGCAACAAG-------
--------------------------------------------------
--------------------------------------------------
--------------------------------------------------
--------------------------------------------------
--------------------------------------------------
--------------------------------------------------
--------------------------------------------------
--------------------------------------------------
--------------------------------------------------
--------------------------------------------------
--------------------------------------------------
--------------------------------------------------
--------------------------------------------------
--------------------------------------------------
--------------------------------------------------
--------------------------------------------------
--------------------------------------------------
--------------------------------------------------
--------------------------------------------------
--------------------------------------------------
--------------------------------------------------
--------------------------------------------------
--------------------------------------------------
--------------------------------------------------
--------------------------------------------------
--------------------------------------------------
--------------------------------------------------
```


>Collichthys lucidus
AGTCTCCTTATTCGAGCAGAACTGAGCCAACCCGGCTCACTTCTCGGAGA
CGACCAGATTTTTAATGTAATTGTTACGGCACATGCCTTCGTTATAATTT
TCTTTATAGTAATACCCGTTATGATTGGAGGTTTCGGGAACTGGCTCGTG
CCCTTAATAATCGGCGCCCCCGACATAGCATTCCCCCGGATAAATAACAT
GAGCTTCTGACTCATCCCTCCTTCTTTCCTCCTGCTCCTAACCTCATCAG
GGGTCGAAGCGGGAGCCGGAACAGGGTGAACAGT---CCCCCCACTTGCT
GGAAACCTTGCACACGCAGGGGCTTCAGTTGACTTAGCCATCTTTTCTCT
ACACCTCGCAGGTGTATCCTCAATCCTGGGGGCTATCAACTTCATTACAA
CAATTATTAACATAAAACCCCCCGCCATCTCCCAATACCAGACACCCCTG
TTTGTCTGAGCCGTCCTCATTACAGCAGTACTCCTGCTACTTTCACTACC
TGTCTTAGCTGCCGGCATCACAATGCTCTTAACAGACCGCAATCTTAACA
CAACCTTTTTCGACCCTGCAGGAGGAGGCGACCCCATCCT---CCAACAC

CTCTTCTGATTCTTCGGTCACCCTGAAGTA--------------------


```
--------------------------------------------------
--------------------------------------------------
--------------------------------------------------
--------------------------------------------------
--------------------------------------------------
--------------------------------------------------
--------------------------------------------------
--------------------------------------------------
--------------------------------------------------
--------------------------------------------------
--------------------------------------------------
--------------------------------------------------
--------------------------------------------------
--------------------------------------------------
--------------------------------------------------
--------------------------------------------------
--------------------------------------------------
--------------------------------------------------
--------------------------------------------------
--------------------------------------------------
--------------------------------------------------
--------------------------------------------------
--------------------------------------------------
--------------------------------------------------
--------------------------------------------------
--------------------------------------------------
--------------------------------------------------
--------------------------------------------------
--------------------------------------------------
--------------------------------------------------
--------------------------------------------------
--------------------------------------------------
--------------------------------------------------
--------------------------------------------------
--------------------------------------------------
--------------------------------------------------
--------------------------------------------------
--------------------------------------------------
--------------------------------------------------
--------------------------------------------------
--------------------------------------------------
--------------------------------------------------
--------------------------------------------------
--------------------------------------------------
--------------------------------------------------
--------------------------------------------------
--------------------------------------------------
--------------------------------------------------
--------------------------------------------------
--------------------------------------------------
--------------------------------------------------
--------------------------------------------------
--------------------------------------------------
--------------------------------------------------
--------------------------------------------------
--------------------------------------------------
```


>Conger oceanicus

-------------------------GGACGAGTATATTGTGGTCTTCAGC
CGCTCACTTAACAGGCTGATCCTGAACGAGGCAGAGCTGATCCTGGCGCT
AGCGCAGGAGTTCCAGATGAAGGTTGTCACCATCTCCCTGGAGGAACAGT
CCTTTGCAGACATCATCCGAGCCCTCAGCAGGGCGTCTATGCTGGTCAGT
ATGCATGGGGCCCAGTTGGTCTGCTCTCTCTTCCTCCCTCGTGGCGCTGC
TGTGGTGGAACTCTTCCCATATGGCGTCAACCCAGAGCATTATGCTCCAT
ACAGGACACTGACCTCGCTACCAGGCATGGACCTGCAGTACGTGGCCTGG
AGGAACACCAAGGAGGAGAACTCTGTGACCTTCCCTGAGCGTGCCTGGGA

CCAGGGTGGCATTGCTCACTTGGAGAAGGAGGAGCAGGAACGCATCCTTA
AGAGCAAGGAGGTGCCACGTCACCTGTGCTGTCGGAACCCGGAGTGGCTC
TTCCGTATCTACCAGGACACCCAGGTGGACATTGCATCCCTACTCGACGC
CCTGCG---CCAGGGACTGACC---TCCAAGCCAGGGCCCAAGAA---GG
CTAGGCCCAGCAGCACAGTCCATCCAGGCAGGGTGAGGGAGCCCAAGTGC
CAGACCTCCGTCCAGGCCACCAACGAGGCCAAGCTGACCGTGTCCTGGCA
GATCCCCTGGAACCTCAAGTACCTGAAGGTGAGGGAGGTGAAGTATGAGG
TATGGATTCAGAAGAAGGATACCAGCAAGGGGACACTGGAGGATCAAATC
ATCCAGGCAAACCCAGCGCTGGAGGCTTTCGGCAATGCCAAGACGGCAAG
GAATGACAACTCCTCACGTTTTGGTAAATTTATTCGCATTCATTTCGGAG
TAAGTGGCAAGCTGTCCTCTGCTGACATAGAAACCTACCTACTTGAGAAA
TCTCGTGTGACCTTTCAGCTCAAGGCTGAGAGGGACTATCACATATTCTA
CCAGATCATGTCCAACAAAAAGCCAGAATTGCTGGACATGCTGCTGATCA
CCAACAACCCATACGATTATGCTTACGTCTCCCAAGGAGAGGTGACGGTC
ACATCCATTGATGACTCAGATGAACTGATTGCCACAGACAGTGCCTTTGA
TGTGCTAGGCTTCACGGCAGAGGAGAAGACAGGTGTCTATAAGCTAACAG
GCGCCATCATGCATTATGGAAATATGAAATTCAAGCAGAAGCAGCGCGAG
GAGCAGGCAGAGCCTGATGGCAGTGAGTCTGCTGACAAGTCAGCCTTCCT
AATGGGGCTGAACTCTGCTGACCTTCTCAAGGGACTCTGCCATCCAAGGG
TTAAAGTTGGAAATGAGTTTGTCACCAAAGGCCAAAATGTGGATCAAGTT
TACTATCCCAACAAAGAGGCCTTCAAGTGCGAGGAGTGCGGCAAGAACTA
CAACACCAAGCTGGGCTACAAGCGGCACATGGCCATGCACGCGGCCACCA
GCGGCGACCTCACCTGCAAGGTGTGCCTGCAGAGCTATGAGAGCACGCCC
GTGCTGCTGGAGCACCTGAAGAGCCACTCGGGGAAGTCCTCCGGCGGGGC
CAAGGAGAAGAAGCACCCCTGTGACCACTGCGACCGCCGCTTCTACACAC
GCAAGGATGTACGGCGCCACATGGTGGTGCACACCGGCCGCAAGGACTTC
CTGTGCCAGTACTGCGCCCAGCGCTTCGGCCGCAAGGACCACCTGACCCG
GCACGTGAAGAAGAGCCACTCGCAGGAGCTGCTGAAGATCAAGACCGAGC
CGCCGGACATGCTGGGTCTGCTGGGGTCCGGCTCCCCGCCCTGCCCCATA
AAGGAAGAGCTCAGCCCCATGATGTG---TATGGGCCCCTCCAAGGACTC
CCTGATGGGCAAGCCCTTCCCCGGTG------CCTTCCCCATGGGCATGT
ACAACCCTCAC---------CTGCAGGCTATGTCCAACTCAGGGGTGTCC
------------CATCACTCCCTGGTGTCCGGGTCGCTGTCTGCAGCCAT
GGGAATGGGCTGCCACATGGAGTACCTGATCTACGCTTCCTTCTCTTTCA
TGGGATGTTTACAGATCAGCGACGGGTCGAACGTCGTCAACCTGCTGGCC
AGCAACTCCCCGAGCGTCTCCTACGCTCTGACCCAGCAGAAGTACTTCAG
CAACTACAGCCCGGTGATCGGGTTCTACATCTACGAGCCCATCGAGTACT
GGAACTCCACCGTCCAGGAGCACCTGAAGACGCTGGGCCACGGGTTCAAT
AAGATTTCGTGGATTGACAACTTCTTTCAGTATCTGAAGGTGGTGAACGT
TAGCGCGTCGACCAAAAGCGAATTCATAACCATCCTCCAGTCCTCCTTCC
TGAGGAGCCCAGAGTACCAGCACTTCAAGGACGACATAATATTTTACAAA
A---TAAGGG------------ACGAGACAGAGATCATCGCCTCCCGGAT
GTACCTGGTGGCCCGGACCACGGAGAAGACCCGGGAGGAGGTGGTGGAGC
TCCTGGAGAGGCTSAGGCCCCTCTCCCTCATCAACAGCATCAAATTCATC
GTCTTCAACCCCACCTTCGTGTTCATGGATCGATACAGCTCCTCGGTCAT
TTCGCCCATCCTCACCTCAGGCTTCAGCGTCCTGATCATCCTGATCCTGA
CCTTCTTTCTGGTCATCAACCCCCTGGGGAACTTCTGGCTGATTCTGACG
GTCACCTCGGTGGAGCTGGGGGTCCTGGGCCTAATG--------------

--------------ACCGGAGAAGTCACAGACAGAGAAGTGGCTTTGGGG
ATAAATCCCTTTGCTGATGGGATGGGAGCTTTTAAAATCAACCACAGCAC
CCACGACCTGGGCTCTGG---ACAAACGGCGTTTTCCTCGCAAGCG---C
CAGGCTAT------GCCGCTGCCCTGGGA---CACCATCA------CCAC
CCCACCCATGTCAGCTCC---TATTCCACCGCGGCTTTCAATTCCACTCG
GGACTTTCTGTTTCGAAATCGGGGTTTCGGAGACGCAACGAG--------
----CGCACAGCACAGCCTCTTCGCCTCCGC---AGCGGGAAGTTT---T
------GCAGGGCCACATGGACACTCGGATGCAACTGGGCACCTGCTTTT
TCCGGGACTTCACGAG---CAAGCGGCCACTCACGCGACTTCCAATGTTG
TAAACAGTCAGATGCGTCTGGGCTTTTCTGGGGACATGTACGGTAGAGCC
GAGCAGTACGGCCATGTAACCAGTCCCAGGT---CCGATCATTACGCTTC
GACCCAGTTGCACGGATATGGCCCCATGAACATGAACATGGCTGCA---C
ATCACGGAGCAGGGGCCTTCTTTCGGTACATGAGGCAGCCGATAAAGCAG
GAACTCATCTGCAAATGGATCGAACCAGAACAACTGACGAACCCGAAAAA
GTCCTGCAACAAAACTTTCAGCACAATGCACGAGCTTGTTACACATCTCA
CAGTGGAACATGTCGGGGGGCCAGAACAATCGAATCATATCTGCTTTTGG
GAAGAGTGTCCGAGGGAAGGAAAACCCTTTAAGGCGAAGTACAAACTTGT
AAATCACATCAGGGTGCACACCGGTGAAAANNNNNNNNNNNNNNNNNNNNNNNNNNNNNNNNNN
>Coregonus clupeaformis
AGCCTTTTAATCCGAGCGGAACTAAGCCAACCCGGGGCTCTTCTAGGGGA
TGATCAGATTTATAATGTGATCGTCACGGCCCACGCCTTCGTTATGATTT
TCTTTATAGTTATGCCAATTATGATTGGAGGCTTTGGAAACTGATTAATC
CCACTTATAATCGGGGCCCCCGACATGGCATTTCCCCGAATGAATAATAT
GAGCTTTTGGCTCCTTCCCCCCTCCTTTCTCCTTCTCCTGGCCTCGTCCG
GAGTTGAAGCCGGTGCCGGCACAGGATGAACAGTCTACCCCCCTCTGGCA
GGCAACCTCGCCCACGCAGGAGCCTCCGTCGATTTAACTATTTTCTCCCT
CCACTTAGCTGGTATTTCCTCTATCTTGGGGGCCGTTAATTTTATTACAA
CCATTATTAATATGAAACCCCCAGCTATTTCCCAGTATCAAACCCCTCTT
TTTGTCTGGGCCGTCTTAATTACCGCAGTCCTTCTACTGCTTTCCCTTCC
TGTCCTAGCAGCAGGTATTACCATGCTACTCACAGACCGGAATCTGAACA
CCACTTTCTTTGACCCAGCGGGCGGGGGAGATCCAATCCTGTATCAACAC
CTCNNNNNNNNNNNNNNNNNNNNNNNNNNNNNNNNNNNNNNNNNNNNNNNNNNNNNNNNNNNNNNNNNNN
NNNNNNNNNNNNNNNNNNNNNNNNNNNNNNNNNNNNNNNNNNNNNNNNNNNNNNNNNNNNNNNNNNNNNN
NNNNNNNNNNNNNNNNNNNNNNNNNNNNNNNNNNNNNNNNNNNNNNNNNNNNNNNNNNNNNNNNNNNNNN
NN---

```
--------------------------------------------------
--------------------------------------------------
--------------------------------------------------
--------------------------------------------------
--------------------------------------------------
--------------------------------------------------
--------------------------------------------------
--------------------------------------------------
--------------------------------------------------
--------------------------------------------------
--------------------------------------------------
--------------------------------------------------
--------------------------------------------------
--------------------------------------------------
--------------------------------------------------
----------------GGACGAGTACATCGTAGTGTTCAGTCGCTCCATC
AACAGACTCATCCTGAACGAAGCNGAGCTGATCCTRGCGTTGGCGCAGGAG
TTCCAGATGAGAGCCGTGACGGTGTCTCTGGAGGAGCAGACCTTCCCAAA
CATCGTCAAGGTCATCAGCGGGGCCTCCATGTTGGTCAGCATGCACGGGG
CCCAGCTGGTCTCCTCTCTCTTCCTCCCCCGGGGGGCCGTCGTAGTGGAG
CTCTTCCCCTATGCGGTCAACCCTGAGCAGTACACCCCTTACAAAACCTT
GGCCTCTCTACCAGGCATGGACCTGCAGTATGTGGCCTGGAGKAACATGG
TGGARGAGAACTCTGTGGCCTACCCAGAGAGGCCCTGGGAGCAGGGAGGT
ATAGCCCACCTGGAGAAGGAAGAGCAGGAACACATCCTGGCCAGTAAGGA
AGTACCCAGACACCTKTGCTGCCGCAACCCTGAGTGGCTCTATCGCATCT
ACCAGGACACCATWGTGGACATCCCTTCTTTACTAGAGGCTCTCAG---A
GAGACAGTGAAA---ACCAGGCCCAACCTGAAGAA---GGCCAAGCCTGC
CAGCACGGTCCACCCGGGCCGGGTCAGAGAGCCCCAGTGCCAGACGTCGG
TCCCGGCCACCAACGAGGCCAWGCTGACTGTGTCGTGGCAAATCCCCTGG
AACCTCAAGTACCTGAAGGTCAGGGAGGTGAAGTACGAAGTGTGGATCCA
GAAGAGGGATCCAAGCAAGGGCACCCTGGAAGATCAAATCATCCAGGCTA
ACCCTGCACTGGAGGCTTTCGGTAATGCCAAAACACTGAGAAATGACAAC
TCATCACGTTTCGGCAAATTCATCCGTATTCACTTCGGGACCAGTGGGAA
ACTGTCCTCTGCCGACGTAGAGACTTACCTTCTTGAGAAGTCCCGTATCA
CCTTTCAGCTCAAAGCTGAGAGGAACTACCATATTTTCTTCCAGATATTG
TCCAATCAAAAGCCAGAGCTGTTGGACATGCTGTTAATCACCAACAATCC
ATATGACTACTCCTACATCTCTCAAGGAGAGGTAACAGTAGCATCCATCA
ATGATTCTGAGGAACTGATGGCCACTGACAGTGCCTTCGATGTACTCGGC
TTTACTGCAGAGGAGAAACAGGGGGTCTACAAGTTGACAGGTGCCATCAT
GCACTACGGCAACATGAGGTTCAAACAAAAGCAGCGGGAAGAGCAGGCAG
AGCCTGACGGTACAGAGGCTGCTGACAAGTCAGCTTACCTAATGGGGATC
AACTCTGCAGATCTGATTAAAGGACTTTGCCATCCCAGAGTCAAGGTTGG
CAATGAGTATGTCACCAAAGGTCAAGGTGTAGATCAGGTCTACTACCCCA
ACAAAGAGGCCTTCAAGTGTGAGGAGTGTGGCAAGCACTACAACACCAAG
CTGGGCTACAAGCGTCACGTGGCCATGCATTCAGCCACGGCAGGGGACCT
CACCTGTAAGGTGTGCCTGCAGAGCTACGAGAGTACCCCTGCCCTGCTGG
AGCATCTCAAGAGCCACTCGGGCAAGTCCTCCGGAGGTGCCAAGGAGAAG
AAGCACCCGTGTGACCACTGTGACCGCTGCTTCTACACACGGAAGGACGT
GCGCCGCCACATGGTGGTCCACACCGGCAGAAAGGACTTCCTGTGTCAGT
ACTGCGCCCAGCGCTTCGGCAGGAAGGACCACCTGACGCGGCATGTGAAG
AAGAGCCACTCTCAGGAGCTGCTAAAGCTAAA------GCCTCCGGACAT
GCTGGGC---------TCTGGCTCCTCCCCCTGCATTGTCAAAGAGGAGC
TCAGCCCCATGATGTGCAGCATGGGTCCCAACAAGGACCCCCTGATGGCC
```

AAGCCGTTCCCCACTGGCACCCCCTTCCCCATGGGCATGTACAACCCCCA
CCAC------CTCCAGGCCATGTCCAACCCTGGGGTGGGTCAC-------
--CACCATTCTCTGATGCCCGGCTCCCTGTCTGCTGCCATGGGGATGGGC
TGCCACATGGAGTACCTGATCTACGCCTCCTTCTCCTTCATGGGATGTTT
ACAAATCAGTGATGGATCCAACATCGTCAACCTCCTAGCCAGCAACACGC
CCAGTGTGTCCTAYGCYACGACCCAGCAGAAGTACTTCAGTAACTACAGC
CCCGTGATCGGCTTCTACATATACGAGCCCATCGAGTACTGGAACTCCAC
GGTGCAGGAGCACCTCACAACGCTKAGCCACGGCTTCAACAAGATCTCCT
GGATGGATAACTATTTTCAGTATCTGAAGGTGGTGAATGTGAGCGCCTCG
ACCAAAAGTGATTTYATCACCATCCTRCAGGGCTCCTTCCTGCGCAGCCC
AGAGTATCAGCACTTCATGGARGACATCATTCTGTCCAAGACAGATGGRG
------------ATGAAATGGAGATCATCGCRTCCAGGATGTACCTGGTG
GCGCGGACCACAGAGAAGACAAGGGAGGAGGTGGTGGARCTGYTGGAGAG
GCTCCGCCCMCTCTCGCTCATCAACAACATCAAGTTCATTGTCTTCAACC
CCACCTTCGTCTWCATGGACCGTTACAGCTCCTCCGTGGTGTCACCCATC
CTCACMTCGGCYTTCAGYGTGCTCACCATCCTCATCCTCACCTTCTTCCT
GGTCATCAACCCTCTGGGGAACTTCTGGTTGATCCTGACYGTCACCTCCG
TAGAGCTGGGGGTCCTGGGCCTCATGGGCTACCACCCCTTCGAGTGGCAG
CCGGCCCTCAAGAGTGTGTCCACATCCTGCCATGTGGGGATCATTGACGG
GCTATCAGGGTGGGTCGCTTCGGTGGATGACTCCCCAGCAGACACAGTCA
CGCGTCGGTTCCGCTACGACGTAGCCCTGGTGTCGGCCTTGAAGGACCTG
GAGGAAGACATCATGGAGGGGCTGAGAGAGCGAGGCCTGGAGGACAGCGC
TTGCACCTCGGGCTTCAGCGTCATGATCAAGGAGTCCTGCGATGGCATGG
GGGACGTCAGTGAGAAGCATGGTGGAGGGCCGGCCATCCCGGAAAAGGCT
GTGCGTTTCTCCTTCACCATCATGTCCATCTCTATTCAAGCTGAGGGAGA
AGAT------------------------------GAGGCGATCACCATTT
TCCGGGAGCCCAAGCCCAACTCAGAGATGTCCTGCAAGCCGCTCTGCCTG
ATGTTTGTGGATGAGTCGGACCACGAGACGCTCACAGGCGTCCTGGGGCC
TGTGGTGGCCGAAAGGAACGCTATGAAGCACAGCCGTCTCATCCTGTTTG
TGGGCGGCCTTTCTCGCTCCTTCCGCTTCCACTTCCGGGGCACGGGCTAC
GACGAGAAGATGGTGCGAGAGATGGAGGGTTTGGAGGCCTCTGGCTCCAC
TTACATCTGCACGCTGTGCGACTCTACTCGGGCAGAGGCCTCCCACAACA
TGACTCTCCACTCTGTCACCCGCAGCCACGATGAGAACCTGGAGCGTTAC
GAACTTTGGAGGACCAACCCTTATTCTGAATCAGCTGAGGAGCTGCGAGA
CCGAGTCAAAGGCGTCTCTGCCAAGCCCTTCATGGAGACCCAGCCCACAC
TGGACGCCCTGCACTGTGATATCGGCAACGCCACCGAGTTCTACAAGATT
TTCCAGGATGAGATAGGGGAGGTCTATCACAAGGC---CAAC---CCCAG
CCGGGAGCAGCGTCGGAGCTGGCGAGCAGCCCTGGACAAACAGCTGAGGA
AGAAGATGAAGCTGAAGCCTGTGATGAGAATGAATGGGAACTATGCACGG
AAGCTGATGACCCGGGAGGCAGTGGAGGCAGTGTGTGAGCTGGTGCACTC
AGAGGAGCGTCAGGAAGCC-------------------------------

---------------------------------TCGTACACCATTGAGAT
GGGCCCCAAAGGGCCTCAATGGAAAGAAAGCCCCACACCTTTCTCTTGCT
CCATCGAGGACCCTACCAAGCAGACCAAGTTCAAAGGCATCAAGACCTAC
ATATCGTACCGAGTGACCCCAAGCCACAATGCGAGGCCTGTGTACCGGCG
TTACAAGCACTTTGACTGGTTGTACAACCGTCTACTGCACAAGTTCACAG
TCATCTCTGTGCCCCACCTGCCCGAGAAACAGGCAACGGGGCGCTTCGAA
GAGGACTTCATCGAGAAGCGCAAGAGGCGGCTGATCCTCTGGATGGACCA
CATGACCAGCCACCCGGTCCTGTCGCAGTACGAGGGCTTTGAGCACTTCC

TCATGTGCGCTGACGACAAGCAGTGGAAGCTGGGCAAGCGGCGGGCAGAG
AAGGACGAAATGATGGGCGCCAACTTCATGCTCACCTTCCAGATCCCAAA
CGAGCACCAGGACCTGCAGGATGTGGAGGAACGTGTGGACTCCTTCAAGT
CCTTCGGCAAGAAAATGGATGACAGCGTCATGCAGCTTACGCATGTGGCC
TCGGAACTGGTGCGGAAGCACCTCGGAGGCTTCCGCAAGGAATTTCAGCG
GCTGGGGAATGCTTTCCAGAACGTCAGTCAGGCGTTCATGCTGGACCCTC
CCCACTNNNNNNNNNNNNNNNNNNNNNNNNNNNNNNNNCCTCTCGCCACTTTTCTCAAACTGACATCCCT
GGGTTTTATCAT
CGGAGTCGGCGTCGTTGGCAACCTCCTGATCTCCATCCTACTAGTCAAAG
ACAAAAGCCTGCACCGCGCGCCATACTACTTCCTGCTGGACCTGTGCGCC
TCGGACATCCTGCGCTCCGCCATCTGCTTCCCCTTCGTCTTCACCTCCGT
CAAGAATGGTTCCACCTGGACGTATGGCACACTTACCTGCAAAGTGATAG
CCTTCCTAGGGGTGCTGTCCTGCTTTCATACAGCGTTCATGCTCTTCTGT
GTGAGTGTGACACGCTACCTGGCCATAGCCCACCACCGCTTCTACACCAA
GAGGCTCACCTTCTGGACGTGTCTGGCAGTCATCTGCATGGTGTGGACGT
TGTCGGTGGCCATGGCCTTTCCCCCGGTGCTGGACGTAGGGACGTATTCC
TTCATAAGGGAGGAGGACCAGTGTACGTTCCAGCACCGCTCGTTCAGAGC
CAACGACTCCCTGGGCTTCATGTTGCTCCTCGCCCTGATCCTCCTGGCCA
CCCAGCTTGTCTACCTCAAGCTTATCTTCTTCGTCCACGACCGCCGGAAG
ATGAAACCGGTCCAGTTTGTGCCTGCCGTCAGCCAGAACTGGACCTTCCA
TGGCCCCGGGGCCAGCGGCCAGGCAGCGGCTAACTGGTTGGCGGGGT---

---NNGCAATCT
CGCTTTCACCCTGGCGTAGTGACTGGACCTGGCACGGACC---GCAGCGT
CCCACTTAGTAACAGCTTGCTATCCCCGCAACAAACCGAAGAGCCCACAG
TTG---CATCCCCGCAGCGATGGTTTGTCACCC---CTGCCAATAACCGA
CTGGACTTTGCCGCCTCGGCGTACGAYGCTGCCGCGGCTGCTGATTTTGC
CGGTAACGCGGCCACCCTGCTGTCCTACGCAGCAGCTGGAGTAAAGGCAC
------TTCCACTGCCCACTGCAGGGTGCTCAAACAGACCTCTAGGTTAC
TATGCCGACCCATCCG---GCTGG---GGCACACACACACCACCCCAGTA
CTGC---------AATAAGTCTAGCTCTGTGCTCTCTTGCTGGCCAACAA
ATTCTGTTG---GYAGAACAGGCA---CCTCCWACTACCTGG--------
-TKGATGA---CGAG---GACAC---CATCCCAACAGAAAGGTCRCCC--
-AT---CGGAGGGTCTAATGAG---GCAAAACCAAAAGACTT------AT
CAGA---ATCCTACTGGATAGAG---ACGCCGTCTTCAATGAAGTCGATT
GATTCATGTGATTCTGGTATTTTTG---AGCAAGCTAAACGGAGAAGAAT
CTCACCGTCTGCTACACCG---------GTTTCGGAAACAGTGTCCCGGT
TGAAATCTGAG---------ACAGGCGAAGTCACAGAAAGAGAAGTGGCT
TTGGGGATAAATCCGTTCGCAGACGGGATGGGAGCTTTTAAAATCAACCA
CAGCTCCCACGACCTGAGCTCCGG---TCAGACGGCGTTCTCCTCCCAGG
CT---CCCGGCTAY---GCTGCTGCCGCCCTKGGT---CACCACCA----
--CCACCCGACACACGTCAGCTCC---TACTCCACCGCAGCATTCAACTC
CACCCGGGACTTTCTCTTCAGAAACCGGGGCTTCGGAGACGCAACCAG--
----------CGCGCAGCACAGCCTGTTTGCCTCCGC---AGCGGGAAGT
TT---T------GCAGGGCCACATGGACACTCCGATGCCACGGGGCACCT
GCTCTTCCCYGGACTCCACGAG---CAAGCCGCCAGCCATGCGTCGTCTA
ATGTCGTCAACAGCCAGATGCGCCTTGGCTTTACCGGGGACATGTACGGC
CGGGCTGACCAGTAYGGCCACGTTACGAGCCCCCGMT---CCGACCACTA
CGCCTCCTCCCAGCTGCATGGCTATGGCCCTATGAACATGAACATGGCGG

CT---CACCACGGGGCAGGGGCCTTCTTCCGATACATGAGGCAGCCCATC
AAACAAGAGCTCATCTGTAAGTGGGTCGAGCCCGAACAGTTGTCGAACCC
CAAAAAGGCTTGCAACAAAACTTTCAGCACGATGCACGAGCTCGTGACCC
ACCTGACCGTGGAGCATGTGGGGGGACCGGAGCAGTCGAACCATATTTGC
TTTTGGGAAGAGTGCGTCCGAGAAGGAAAGCCGTTCAAAGCCAAATATAA
ATTTGTTAATCATATCAGAGTRCACACCGGAGAGAAACCATTCCCATGTC
CCTTTCCCCGGCTGTGGAAA
>Coryphaena hippurus
AGTCTTCTCATTCGAGCTGAGTTAAGCCAGCCTGGGTCACTTCTAGGAGA
TGACCAAACCTATAATGTCATCGTTACAGCACATGCCTTCGTAATAATTT
TCTTTATAGTTATGCCAATTATGATCGGAGGCTTCGGGAACTGATTAATC
CCACTAATGCTTGGCGCTCCTGATATAGCATTCCCTCGAATAAATAACAT
AAGCTTTTGACTTCTTCCACCATCATTTCTTCTCCTTCTAGCCTCTTCAG
GGGTAGAAGCAGGAGCAGGAACTGGTTGAACGGTCTACCCACCTCTGGCG
GGTAACTTAGCCCATGCTGGGGCCTCTGTAGATTTAACAATTTTCTCCCT
GCATTTAGCCGGGGTATCATCAATTCTTGGGGCAATCAATTTTATTACAA
CTATTATTAATATAAAACCCCCCACAGTAACGATATACCAAATTCCACTA
TTCGTGTGAGCTGTACTAATTACAGCTGTACTACTACTCCTATCACTTCC
TGTCCTAGCTGCGGGAATTACAATACTGCTAACAGACCGAAATTTAAATA
CAGCTTTCTTTGACCCAGCGGGAGGAGGGGATCCTATCCTATACCAACAC
CTGTTT--

------------TTCCTGGAGAGAAACCTTCACCCCTCTAACTGCCTTGG
CATGCTGTTGCTGTCCGACGCCCACCAGTGCACCAAGCTGTCAGAGCTCT
CTTGGGGCATGTGCCTCAGCAACTTTCCTGCTATTTGCAAGACAGAGGAC
TTCCTTCAACTGCCCAAAGATATGGTAGTCCAGCTTTTATCACACGAGGA
GCTAGAGACAGAAGATGAGAGACTGGTTTATGAAGCTGCCCTTAACTGGA
TCAACTACGACCTGGAGAGGAGGCACTGCCACCTTCCAGAGCTTCTGAGA
ACGGTCCGCCTCGCCCTGCTGCCTGCCATTTTTCTGATGGAAAACGTCTC
AACGGAGGAGCTGATCAATGCCCAAGCCAAGAGCAAGGAGCTGGTGGATG
AAGCTATTCGCTGCAAGCTGAAAATCCTGCAGAACGAYGGCGTTGTCAAC
AGCCCGTGCGCTCGGCCGAGAAAAACCAGCCAYGCCCTGTTCCTTCTGGG
CGGGCAGACTTTCATGTGTGACAAGTTGTAYCTGGTAGAYCAGAAGGCCA
AAGAGATCATACCCAAGGCTGACATCCCCAGCCCCAGGAAGGAGTTCAGT
GCCTGCGCCATCGGCTGTAAGGTGTACATCACCGGTGGGA--GAGGCTC-
CGAGAATGGTGTGTCCAAAGACGTGTGGGTCTACGATACCGTCCACGAGG
AATGGTCCAAAGCAGCTCCCATGCTCATCGCTCGGTTCGGCCATGGCTCC
GCAGAGCTGAAACATTGCCTCTACGTGGTAGGAGGTCACACGGCGGCCAC
CGGTTGCCTCCCAGCTTCTCCGTCAGGACAATTACATTGTTGTGTTTAGT
CGCTCGACGACGAGGCTGATACTGAACGAAGCGGAGCTCATCATGGCGCT
GGCCCAGGAGTTCCAGATGAGAGTGGTGACAGTTTCCCTGGAGGATCAGT
CCTTCCCCAGTATTGTGCAGGTGATCAGCRGCGCTTCCATTCTAGTTAGC
ATGCACGGAGCCCAGCTCATCACCTCRCTCTTCCTCCCCAGAGGAGCCAC
TGTGGTGGAACTGTTTCCCTTCGCCGTGAACCCGGAGCAGTACACCCCTT
ATAAAACCCTGGCCTCCCTTCCTGGCATGGACCTTCACTACATCTCCTGG
AGGAACAGCAAGGAGGAAAACACTATCACCCACCCAGACAGACCCTGGGA
ACAAGGAGGCATTATTCACTTGGACAAAGAGGAGCAGGAGCGAATACTGG
CCAGCAAGGACGTTCCCAGGCACCTGTGCTGCCGAAACCCCGAGTGGCTC
TTCCGGATCTACCAGGACACATTGGTGGACATCCCTTCCTTCCTGGAAGT
CCTCAA---AGAGGGCATGAAG---GCCAAGCCCAGCTTTAAGAA---GT
CTAAGCCAGCCAGCATGGTCCACCCAGGCCGAGTCCGGGAGCCTCAATGT

CAGACCTCAGTCCAAACCAGTAATGAGGCCAAGCTCACAGTCTCCTGGCA
GATCCCATGGAATTTAAAATACCTGAAGGTAAGAGAGGTGAAGTACGAGG
TCTGGATCCAGAAAAAAGATGCCAGCAAGGGAACACTGGAGGATCAAATC
ATCCAGGCTAACCCTGCGCTGGAAGCCTTTGGCAACGCCAAGACGTTGAG
AAATGACAACTCATCCCGTTTTGGAAAATTCATTCGAATTCACTTCGGCA
CGAGTGGCAAACTGTCATCCGCTGACATCGAGACATACCTGCTGGAGAAA
TCACGTGTCACCTTTCAGCTCAAGGCTGAGAGGAACTACCACATCTTCTA
TCAGATCCTGTCCAATCAGAAGCCGGAACTGCTGGACATGCTGCTGATCA
CCAACAACCCGTATGACTACTCCTACATCTCCCAAGGAGAGGTGACGGTC
GCTTCTATCAATGACTCGGAGGAGCTAATGGCCACTGACAGCGCCTTTGA
CGTGCTTGGCTTCACTGCAGAGGAGAAGATGGGCGTCTATAAACTGACTG
GTGCCATCATGCATTATGGCAACATGAAGTTCAAACAGAAACAGCGTGAA
GAGCAGGGTGAACCGGATGGGACCGAGGCCGCTGACAAATCAGCTTACCT
AATGGGACTGAACTCCGCTGACCTCATCAAAGGCCTTTGCCACCCAAGAG
TCAAGGTAGGAAATGAATATGTCACCAAAGGCCAAAGTGTGGATCAAGTC
TACTATCCCAACAAGGAGGCCTTCAAGTGTGAGGAGTGTGGGAAACACTA
CAACACCAAGCTGGGGTATAAGCGTCATGTGGCCATGCACTCTGCCACAG
CAGGCGATCTCACCTGTAAAGTGTGCATGCAGAGCTACGAGAGTACACCC
ATCCTCCTGGAGCACCTCAAGAGCCACTCCGGGAAATCCTCCGGTGGCAC
CAAGGAGAAGAAACACCCGTGCGACCACTGTGACCGTCGTTTCTACACAC
GCAAGGATGTGCGACGACACATGGTGGTCCACACGGGCCGGAAGGACTTC
CTGTGCCAGTACTGTGCCCAGCGCTTCGGCAGGAAAGACCACCTGACGCG
CCACGTGAAGAAGAGCCACTCACAGGAGCTGTTGAAGATCAAGGCGGAGC
CTCCAGACATGTTAGGTCTCTTAGCCTCCGGATCCCCACCTTGCTCTGTG
AAAGAGGAACTCAGCCCCATGATGTGCGGCATGGGTCCTAACAAAGACCC
CATGATGGGCAAAGCGTTCCCCAGTGGGGCCCCTTTTCCAATGGGCATGT
ACAACCCCCACCAT------CTCCAGGCAATGTCGAATACTGGGGTGGGT
CACCCA------CACCCGTCCCTAATGCCCGGCTCCCTGTCTGCAGCTAT
GGGCATGGGCTGTCACATGGAATATCTCATCTACGCCTCTTTCTCATTCA
TGGGATGTTTACAAATTAGYGATGGGTCAAATATCGTCAACCTGCTGGCA
AGTAACTCTCCGAGTGTTTCATATGCTCTGACCCAGCAGAAATACTTCAG
TAACTACAGCCCCGTGATTGGGTTTTACATTTATGAGCCCATCGAGTACT
GGAACTCCACGGTGCAGGAGCACCTGAAAACCCTGAGTCATGGTTTCAAC
AAGATCTCCTGGATGGACAACTTTTTCCACTACCTGCGGGTGGTCAACGT
GAGCGCGTCAACTAAAAGCGACTTCATCACCATCCTCAAAGGCTCCTTCC
TGCGCAGCCCAGAGTACCAGCATTTCACAGAGGACATCATATTCTCCAAG
A---ACCGTGAGACCG------ATGAGTATGAYATCATTGCCTCACGGAT
GTACCTGGTGGCGCGGACGACCGAGAAGAAGCGCGAGGAAGTGGTGGAGC
TCCTGGAAAAGCTTCGTCCGTTGATGCTAATCAACAGCATCAAGTTCATT
GCCTTCAATCCCACATTTGTGTTCATGGACCGCTACAGCTCCTCTGTCAT
CTCACCCATCCTGACCTCAGGCTTCAGCGTACTCACTATCCTCATCCTCA
CTTTCTTCCTGGTCATCAACCCATTGGGAAACTTCTGGCTCATCCTCACG
GTCACGTCTGTGGAGCTGGGCGTCTTGGGTTTGATGGGCTTTCACCAGTT
TGAATGGCAGCCAGCTCTCAAGAATGTGTCAACATCTTGCAATGTTGGCA
TTATTAATGGCCTCTCTGGATGGGCTTCCTCGGTGGATGACTCCCCAGCT
GACACCATCACTCGACGGTTCCGCTATGATGTGGCACTGGTGTCAGCATT
AAAAGATCTGGAGGAGGACATCATGGAGGGGCTGAGAGATAGTCGGATGG
AAGACAGTGCTTGCACCTCAGGCTTTACTGTCATGATCAAGGAATGTTGT
GATGGCATGGGCGATGTCAGCGAGAAGCATGGTGGAGGACCAGTTATTCC
TGAGAAGGCTGTACGTTTCTCTTTCACTGTTATGTCTATCTCTGTCATGG
TAGACAATGAGGGG------------------------------AAGGAG
GTTACCATCTTCACCGAACCAAGGCCAAACTCAGAACTGTCCTGTAAGCC
CCTTTGCTTGATGTTTGTGGATGAGTCAGACCACGAGACGCTCACAGCGC

TCCTGGGGCCTGTAGTTGCAGAGCGTAATGCCATGAAAGAGAGCAGGCTC
ATCCTATCCATAGGTGGCCTACCTCGCTCCTTCCGCTTCCACTTCAGAGG
TACAGGATACGATGAGAAGATGGTGCGAGAGATGGAAGGCCTGGAGTCTT
CTGGGTCCACATATATCTGCACTCTATGCGACTCCAGTCGAGCAGAGGCC
TCTCAAAACATGGTCCTACACTCCATCACCCGCTGCCACGAAGAGAACCT
AGATCGTTATGAAATATGGAGAACCAACCCCTTCTCTGAGTCTGCAGATG
AACTGCGAGACAGAGTCAAAGGAGTCTCTGCAAAACCCTTTATGGAGACC
CATCCCACACTGGACGCATTACACTGTGACATTGGCAATGCCACTGAGTT
CTACAAAATCTTCCAGGATGAGATTGGAGAAGTGTATCAAAAGGT---CA
AC---CCCAGCCGAGAGGAAAGGCGCAGCTGGAGGGCAGCTCTAGATAAA
CAGCTGAGGAAGAAGATGAAGCTGAAACCGGTGATGAGGATGAATGGGAA
CTACGCCCGCAGGCTAATGACCCTGGAGGCGGTTGAGGTGGTGTGTGAAC
TGGTGCCCTCAGAGGAGAGGAGGGAGGCCCTGAGGGAGCTCATGAGGCTT
TACCTCCAGATGAGGCCTGTGTGGCGCGCCACCTGCCCAGCAAAGGAATG
CCCAGACCAGCTGTGCCGCTATAGCTTTAACTCTCAGCACTTTGCTGACC
TCCTCTCCTCCACCTTCAAATATAGATACAATGGAAAGATAACCAATTAC
CTTCACAAGACTCTGGCCCATGTCCCTGAAATCATAGAGAGAGATGGATC
CATAGGAGCATGGGCCAGTGAGGGGAATGAATCGGCAAACAAATCGTACA
CCATCGAGATGGGCCCCCTGGGTCCTCGGTGGAAGGAGAACCCGCAGCCT
TTCTGCTGCTCCATCGAAGACCCCACAAAACAGACAAAGTTCAAGGGCAT
CAAGACCTATATTTCATACCGGGTCACCCCGAGCCACACGGGGCGTCCTG
TCTACAGGCGCTACAAACACTTTGACTGGCTCTACAATCGCCTGCTGCAC
AAGTTCACTGTGATCTCTGTGCCTCACCTGCCCGAGAAACAGGCCACGGG
GCGATTTGAGGAAGACTTCATTGAGAAGCGTAAAAGACGACTGATACTGT
GGATGAACCACATGACCAGTCACCCGGTCCTCTCCCAGTACGAAGGCTTT
GAGCACTTTCTGATGTGTGCTGACGACAAACAGTGGAAACTGGGAAAGAG
ACGAGCGGAGAAGGACGAGATGGTCGGCGCCCATTTCATGCTTACACTTC
AAATCCCCAACGAGCACCAGGACCTTCAGGATGTAGAGGAGCGGGTGGAC
TCCTTCAAATCCTTTGCTAAAAAAATGGACGACAGCGTGATGCAGCTCAC
GCATGTTGCCTCGGAGCTGGTGCGAAAACATCTGGGTGGGTTCAGGAAGG
AGTTCCAGCGGCTGGGAAATGCCTTCCAGTCTATCAGCCAGGCCTTTATG
CTGGACCCCCCCCACAGCTCGGATGCCCTCAACAACGCCATCTCACATCC
TCTCGCCACGTTCCTCAAACTGACCTCTCTGGGTTTCATCATTGGAGTCG
GTGTGGTTGGGAACCTCCTGATCTCCATCCTGCTGGTCAAAGACAAGAGC
CTGCACCGAGCGCCCTACTATTTCCTGCTGGACCTGTGCGCCTCTGATAT
CTTGCGCTCCGCCATCTGCTTCCCCTTTGTCTTCACCTCAGTCAAGAATG
GCTCTGCCTGGACCTACGGCACGCTGACCTGCAAAGTGATTGCCTTCCTG
GGTGTGCTCTCCTGTTTCCACACAGCGTTCATGCTGTTCTGTGTCAGTGT
CACTCGCTACCTGGCCATCGCACATCACCGTTTCTACACCAAGAGGCTGA
CCTTCTGGACCTGTCTGGCTGTCATCTGCATGGTGTGGACGCTGTCAGTA
GCTATGGCATTCCCGCCGGTGCTAGATGTAGGGACGTACTCTTTTATCCG
GGAAGAGGACCAGTGCACATTCCAGCACCGTTCCTTCCGGGCGAATGATT
CGCTGGGCTTCATGCTCCTCCTGGCGCTCATCCTCCTGGCCACACAGCTG
GTTTACCTCAAGCTCATCTTTTTTGTCCATGACCGTCGAAAGATGAAGCC
TGTCCAGTTCGTGCCTGCTGTCAGCCAGAACTGGACCTTCCATGGGCCAG
GTGCCAGTGGGCAGGCGGCGGCCAACTGGCTGGCTGGATTTGGTCGTGGC
CCCACCCCACCTACTTTGCTGGGTATCCGGCAGAACAGCAACGCAGCGGG
CCGCAGGCGTCTACTGGTATTGGATGAATTCAAAACAGAGAAGCGGATTA
GTAGGATGTTCTACATCATGACGTTTTTCTTCCTGGCACTGTGGGGGCCC
TACCTGGTCGCCTGCTACTGGCGGGTGTTTGCAAGGGGCCCCGTAGTCCC
TGGGGGCTATCTGACGGCAGCCGTGTGGATGAGCTTTGCCCAGGCTGGCG
TCAATCCTTTCATCTGCNNNNNNNNNNNNNNNNNN----------------------------------
----

-------------------------------------NNNNNNNNNNNNNNNNAAGTCACAGAGAG
AGAAGGGGCGCTGGGGATCAATCCGTTCGCGGATGGGATGGGCGCCTTCA
AAATAAACCACAGCTCCCACGATATAGGCTCCGG---ACAGACGGCGTTT
TCCTCCCAGGCA---CCCGGCTAC---GCGGCGGCCGCCCTGGGA---CA
CCATCA------CCACCCGACCCACGTTGGCTCT---TACTCCACGGCGG
CTTTCAACTCCACCAGGGACTTTCTCTTCAGAAATCGGGGTTTCGGGGAC
GCCGCCGG------------GGCGCAGCACAGTTTGTTCGCCTC------
---TGGAAGTTT---C------GCAGGGCCACATGGACACTCGGATGCGG
CGGGCCACCTGCTCTTCCCCGGGCTGCACGAG---CAAGCGGCGAGCCAT
GCCTCCTCCAACGTGGTCAACAGCCAGATGCGCCTGGGCTTCTCGGGGGA
CATGTACGGACGGGCCGACCAGTACGGCCACGTTACCAGCCCGCGCT---
CCGACCACTATGCCTCGACCCAGCTGCACGGCTACGGCCCCATGAACATG
AATATGGCCGCG---CACCACGGAGCAGGGGCCTTCTTTCGCTACATGAG
GCAGCCGATCAAACAAGAGCTCATCTGCAAGTGGATCGAGCCGGAGCAGC
TGACGAATCCCAAAAAGTCGTGCAACAAAACTTTTAGCACGATGCACGAG
CTGGTGACCCATCTGACGGTGGAGCATGTGGGGGGACCGGAGCAGACCAA
CCACGTCTGCTTCTGGGAGGACTGCTCCAGAGAAGGAAAGCCATTCAAAG
CCAAATACAAACTTGTAAATCATATCAGAGTACACACCGGAGAAAAGCCC
TTTCCCTGCCCGTTCCCCGGCTGTGGCAAG
>Coryphaenoides rupestris

------------TTCCTGGAGAGGAACCTGCACCCCCCCAACTGCCTGGG
CATGCTGCTGCTCTCCGACGCCCACCAGTGCACCAAGCTCTCCGAGCTCT
CCTGGAGCATGTGCCTGAGCAACTTCCCCACCATTTGCAAGACAGAGGAC
TTCCTCCAACTGCCCAAAGACATGGTCGTGCAGCTTCTGTCCCACGAGGA
GCTGGAGACGGAAGACGAGAAACTGGTCTACGAGGCCGCGCTGAACTGGG

TTACCTACGACCTTCCGGGGAGGCACCCCCACTTGCCGGAACTGCTGAAA
ACGGTGCGTCTGGCTCTCCTGCCCGCCATCTTCCTCATGGAGAACGTCTC
CATGGAGGAGCTGATCAACGCGCAGACCAAGAGCAAGGAGCTGGTGGATG
AGGCCATCTGCTGCAAGCTGAAGATCCTGCAGAACGACGGCGTGGTCGAC
AGCCCGTGCGCCCGTCCCCGGAAGACCAGCCACGCGCTCTTCCTGCTGGG
CGGGCAGACCTTCATGTGCGACAAGCTCTACCTGGTGGACCAGAAGGCCA
AAGAGATCATTCCCAAGGCGGACATCCCCAGCCCCCGCAAGGAGTTCAGC
GCCTGCGCCATCGGCTGCAAGGTCTACATCACGGGCGGCC--GCGGCTC-
GGAGAACGGCGTGTCCAAGGATGTGTGGGTCTACGACACCGTGCAGGAGG
AGTGGTCCAAGGCGGCGCCCATGCTCATCGCCCGCTTCGGCCACGGCTCC
ACGGAGCTGAAGCACTGCCTGTACGTGGTGGGCGGGCACACGGCCGCCAC
GGGCTGCCTCCCCGCCTCGCCGTCCGGAGGATTACATTGTGGTCTTCTCT
CGCTCCACAACGAGGTTGATCCTAAACGAAGCGGAGCTGATCATGGCGCT
GGCCCAGGAGTTTCAGATGAGAGTCTTGACGGTGTCCCTGGAGGAGCAGT
CTTTTACCAGCATCGTGCAGGTGATCAGCGGAGCCTCCATGCTGTTCAGT
ATGCACGGCGCACAGCTCATCACGGCGCTGTTCCTGCCCCGAGGTGCAGC
CGTGGTGGAGCTGTTCCCCTACGCCGTGAACCCAGAGCAGTACACCCCAT
ATAAAACCCTGGCCTCCCTGCCGGGCATGGACCTCCAGTATGTCTCCTGG
AGGAACACGATGGAGGAGAACACCGTCACTCACCCGGACCGGCCCTGGGA
CCAAGGGGGCGTCGTACACTTGGAGAAGGAGGAACAAGAGCGCATCCTAG
CCAGCAAGGACGTCCCCAGGCACCTGTGCTGCCGTAACCCAGAGTGGCTT
TTCAGAATCTACCAGGACACTTTGGTGGACATCCCTTCACTCCTCGAGGT
GCTCAA---GGACGGCCTCAGA---ACCAGGCCCAACTTGAAAAA---GA
GCAAGGCGGCCAGCACAGTTCACCCTGGGCGAGTCCGAGAACCCCAGTGC
CAGACCTCGGTCCAAGCCACCAACGAGGCCAAACTCACAGTGTCCTGGCA
GATCCCGTGGAACCTGAAGTTCCTGAAGGTGAGAGAGGTGAAGTACGAGG
TGTGGATCCAGAAGAAGGACGCCAGCAAAGGAACCCTGGAGGATCAAATC
ATCCAGGCCAACCCTGCCTTGGAGGCCTTTGGTAATGCCAAAACCCTGAG
AAATGACAACTCCTCACGTTTTGGAAAGTTTATCCGGATTCACTTTGGGA
ACAGTGGCAAGCTGAGCTCTGCGGACATTGAGACCTACCTCCTAGAAAAG
TCCAGAGTCACCTTTCAGCTTAAGTCAGAGAGGAACTATCACATATTCTT
CCAGATCTTGTCCAATCAGAAGCCAGATCTGCTGGACATGCTGCTGATAA
CCAATAACCCGTATGATTACTCCTACATCTCCCAAGGAGAGGTAACTGTG
GCGTCCATCAACGACTCTGAGGAGCTCATGGCCACTGACAGTGCATTTGA
CGTCCTTGGCTTCACCCTGGAAGAGAAGATGGGGGTGTACAAGCTGACAG
GTGCCATCATGCACTATGGCAACATGAGGTTCAAGCAGAAGCAGCGTGAG
GAGCAGGCAGAGTCCGATGGGACTGAAGCAGCTGATAAATCAGCGTACCT
CATGGGTTTGAACTCTGCAGACCTCATCAAATGCCTCTGCCACCCCAGGG
TGAAGGTAGGAAATGAATATGTCACCAAAGGTCAAGGGGTGGACCAAGTC
TACTAC--

----------------------TACCTCATCTACGCTTCCTTCTCCTTCA

TGGGATGTTTACAGATCAGCGACGGTTCCAACGTGGTCAACCTGCTGGCC
AGTAACTCTCCCAGCGTGTCCTACGCTCTGACGCAGCAGAAGTACTTCAG
TAACTACAGTCCTGTCATTGGCTTCTACATCTACGAGCCCATCGAGTACT
GGAACTCCACGGTGCAGGAGCACCTGAAGACCCTGAGCCACGGCTTCAAC
AAGATCTCCTGGATGGACAACTTTTACCAGTACCTGCGCGTGGCCAACGT
CAGCGCGGCCACCAAGAACGAGTTCATCAGCATCCTGAAGGGCTCCTTCT
TGCGCAGCCCGGAGTACCAGCACTTCACCGAGGACATAATTTTCTCCAAG
A---ACCGCGAGAGCG------ACGAGTACGACATCATCGCCTCGCGCAT
GTACCTGGTGGCCCGCACCACGGAGAAGAAGCGCGAAGAGGTGGTGGAGC
TGCTGGAGAAGCTGCGGCCGCTGATGCTCATCAACAGCATCAAGTTCATC
GCCTTCAACCCCACCTTCGTGTTCATGGACCGCTACAGTTCCTCTGTCAT
CTCGCCCATCCTCACCTCCGGCTTCAGCGTGCTCACCATCCTCATCCTCA
CCTTCTTCCTGGTCATCAACCCCCTGGGGAACCTCTGGTTGATCCTGACC
GTGACCTCGGTGGAGCTGGGCGTCCTGGGCCTGATGNNNNNNNNNNNNNNNNNNNNNNNNNNNNNNNNNN
NNNNNNNNNNNNNNNNNNNNNNNNNNNNNNNNNNNNNNNNNNNNNNNNNNNNNNNNNNNNNNNNNNNNNN
NNNNNNNNNNNNNNNNNNNNNNNNNNNNNNNNNNNNNNNNNNNNNNNNNNNNNNNNNNNNNNNNNNNNNN
NNNNNNNNNNNNNNNNNNNNNCTGCAGGAGCGCGG
CCTGGACGACAGCTCCTGCACCGCGGGCTTCTCCGTCATGATCAAAGAGT
CCTGCGACGGCATGGGAGACGTGAGCGAGAAGCACGGCGGTGGTCCGGCG
GTCCCGGAGAAAGCCGTGCGATTCTCCTTCACCGTTATGTCCATCTCTTT
ACTCATGGAAGGTGGAAAG------------------------------G
AGTCAGTGATCATCTTCCAGGAGTCAAAGCCCAACTCTGAGATGTCCTGT
AAACCCCTGTGCCTCATGTTTGTAGACGAGTCCGACCACGAGACGCTCAC
CGCTGTCCTGGGCCCCGTGGCGGCCGAGCGCAGCGCCATGAAGCGCAGCA
GGCTCATCCTCTCCATCGGCGGGCTTCCGCGCTTCTTCAGCTTCCAGTTC
CGGGGCTCGGGCTACGATGAGAAGATGGTGCGCGACGTGGAGGGCCTGGA
GGCCTCCGGCTCTATGTACGTCTGCACGCTGTGCGATTCCACCCGCGCCG
AGGCCTCCCACAACATGGTGCTTCACTCCGTCACCCGCAGCCACGAGGAG
AACCTGGAGCGTTACGAGACCTGGAGGACCAACCCCTTCTCGGAGTCAGC
GGACGAGCTGAGAGACAGGGTGAAGGGGGTCTCCGCCAAACCCTTCCTGG
AGACCCAGCCGACACTGGACGCGCTGCACTGCGACATCGGCAACGCCACA
GAGTTCTACAAAATCTTCCAGGACGAGATCGGCGAGGTGTTCCTGAAGAC
---CAAC---CCCAGCCGGGAGGAGCGGCGGGGCTGGAGGGCGGCCCTGG
ACAAGCAGNNNNNNNNNNNNNNNNNNNNNNNNNNNNNNNNNNNNNNNNNNNNNNNNNNNNNNNNNNNNNN
NNNNNNNNNNNNNNNNNNNNNNNNNNNNNNNNNNNNNNNNNNNNNNNNNNNNNNNNNNNNNNNNNNNNNN
NNNNNNNNNNNNNNNNNNNNNNNNNNNNNNNNNNNNNNNNNNNNNNNNNNNNNNNNNNNNNNNNNNNNNN
NNNNNNNNNNNNNNNNNNNNNNNNNNNNNNNNNNNNNNNNNNNNNNNNNNNNNNNNNNNNNNNNNNNNNN
NNNNNNNNNNNNNNNNNNNNNNNNNNNNNNNNNNNNNNNNNNNNNNNNNNNNNNNNNNNNNNNNNNNNNN
NNNNNNNNNNNNNNNNNNNNNNNNNNNNNNNNNNNNNNNNNNNNN---
TCTTACACCATCGATATGAGTCCCGTGGGGCCCCAGTGG
AAGGAGAGCCCACAGCCTTTCTCCTGCTCCATCGAAGACCCTACAAAACA
GACCAAGTTCAAGGGCATCAAGACGTATATTTCTTACCGGGTCACGCCGA
GCCACACAGGGCACCCGGTCTACAGGCGCTACAAACACTTTGACTGGCTG
TACAACCGCTTACTGCACAAGTTCACTGTGATCTCTGTGCCTCACCTGCC
CGAGAAGCAGGCCACGGGGCGATTCGAGGAAGACTTCATCGAGAAGCGCA
AGAGGCGACTGATACTGTGGATGAACCACATGACCAGTCACCCAGTCCTC
TCCCAGTATGAAGGCTTTGAGCACTTTCTGATGTGTGCCGATGACAAGCA
GTGGAAGCTGGGCAAGAGGCGGGCGGAGAAGGACGAGATGGTGGGCGCCC
ATTTCATGCTGACCCTCCAGATCCCCAACGAGCACCAGGATCTTCAGGAT
GTAGAGGAGCGGATCGACTCCTTCAAGTCCTTTGCGAAGAAAATGGACGA
CAGCGTGATGCAGCTCACACATGTCGCTTCCGAGCTGGTGCGGAAACACC
TGGGTGGCTTCAGGAAGGAGTTCCAGCGGCTGGGAAACTCCTTCCAGTCC
GTCAGCCAGGCGTTCATGCTGGACCCTCCCCACAGCTCAGAGACCCTCAA

CAACGCCATCTCTCAT----------------------------------

>Cottus carolinae
AGCCTCCTAATTCGAGCAGAATTAAGTCAACCCGGCGCCCTTTTAGGGGA
CGACCAGATTTATAATGTAATTGTTACAGCCCATGCTTTCGTAATAATTT
TCTTTATAGTAATACCGATTATAATCGGAGGTTTCGGGAACTGGCTCGTT
CCCCTAATGATTGGCGCTCCTGATATGGCCTTTCCTCGAATGAATAATAT
GAGCTTTTGACTTCTTCCCCCATCTTTCTTACTCCTCCTTGCCTCTTCGG
GAGTCGAAGCAGGAGCCGGAACCGGATGAACAGTTTACCCGCCCCTCGCC
GGAAACCTCGCCCACGCAGGAGCCTCTGTTGACCTAACAATCTTCTCCCT
TCACCTAGCAGGTATCTCCTCTATTCTTGGAGCAATCAACTTTATCACAA
CTATCATTAACATAAAGCCCCCTGCTATTTCACAATACCAGACCCCGCTC
TTTGTATGATCTGTTCTTATTACTGCTGTCCTACTGCTTCTTTCCCTCCC
CGTTCTTGCCGCCGGCATCACAATACTCCTGACAGACCGAAATCTTAACA
CCACCTTCTTTGACCCTGCCGGAGGAGGGGACCCAATCCTTTACCAACAT
CTC---

-------------------------GGATGATTACGTTGTTGTCTTCAGT
CGCTCCTCAACCAGGCTGATTCTGAATGAAGCAGAGCTAATCATGGTACT
GGCCCAGGAGTTCCAGATGAGAGTGGTCACGGTGTCCCTTGAGGATCAGT
CTTTCCCCAGTATCGTCCAGGTGATCAGCGGCGCTTCTGTGTTGGTCAGT
ATGCATGGCGCTCAGCTCATCACCTCACTCTTCCTCCCCAGAGGAGCTGC
TGTGGTGGAGCTGTTCCCCTTTGCTGTGAACCCAGAGCAGTACACCCCGT
ATAAAACCCTCGCCACCCTTCCAGGCATGGACCTTCACTATATCTCCTGG
AGGAACATTCAGGAGGAGAACACCATCACCCACCCTGACAGACCCTGGGA
GCAAGGGGGCATCGCTCACTTGGAGAAGGAGGAGCAAGAGCGTATACTGG
CGAGCAGAGATGTCCCCAGGCACCTGTGCTGTCGCAACCCAGAGTGGCTC
TTCCGGATCTACCAGGACACTTTGGTGGACATCCCTTCCTTYCTGGAAGC
CCTTAA---AGAGGGCATGAAG---ACAAAGCCCAGCATGAAGAA---GT
CAAAGCCGGCCAGCACAGTCCACCCGGGCCGGGTCAGAGAACCCCAGTGT
CAGACCTCGGTACAAACCACCAACGAGGCTAAACTCACAGTATCCTGGCA
GATCCCTTGGAACCTGAAATACTTGAAGGTGAGAGAGNNNNNNNNNNNNNNNNNNNNNNNNAAAAGAGAC
ACCA
GCAAGGGGACGCTGGAGGACCAGATCATCCAGGCTAACCCAGCGCTGGAA
GCTTTCGGTAATGCCAAAACGCTGAGGAACGACAACTCGTCTCGTTTTGG
AAAATTCATCCGAATTCACTTTGGTACGAGCGGGAAGCTGTCGTCTGCCG
ACATCGAGACGTACTTGCTGGAGAAGTCTCGGTGCACCTTTCAACTCAAG

GCAGAGAGGAACTACCACATCTTCTACCAGATCCTGTCCAATCAGAAGCC
AGAGCTGCTGGACATGCTGCTGATCACCAACAACCCGTACGACTACTCCT
ACATCTCCCAAGGGGAGGTGACGGTGCCATCCATCAACGACTCGGAGGAG
CTGCTGGCCACCGACAGCGCCTTCGATGTGCTCGGCTTCACTGCGGAGGA
GAAGATGGGCGTCTACAAACTCATCGGCGCCATCATGCACTACGGCAACA
TGAAGTTCAAACAGAAGCAGCGTGAGGAGCAGGCTGAGCCAGACGGCACA
GAGGCGGCTGATAAAACGGCTTACCTCATGGGCCTGAACTCTGCTGACAT
CATCAAGGGGCTGTGTCATCCCAGAGTCAAGGTGGGAAATGAATATGTAA
CCAAAGGCCAAAGTGTAGACCAGGTGTACTACNNNNACAAGGAAGCCTTCAAGT
GTGAGGAGTGCGGCAAGCACTACAACACCAAGCTGGGATACAAGCGTCAT
GTGGCCATGCACTTTGCCACGTCCGGAGATCTCACCTGCAAAGTGTGCAT
GCAGAGCTACGAGAGCACACCGGTGCTCCTGGAGCACCTCAAGACCCATT
CCGGGAAGTCTTCGGGCGGTGCCAAGGAGAAAAAGCACCCGTGCGACCAC
TGTGACCGTCGTTTCTACACGCGCAAGGATGTGAGACGGCACATGGTGGT
CCACACGGGTCGCAAGGACTTCCTGTGCCAGTACTGTGCCCAGCGCTTCG
GCAGGAAGGACCATCTGACACGTCACGTGAAGAAGAGCCACTCGCAGGAG
CTGCTGAGGATCAAGACGGAGCCTCCAGATATGTTAGGTCTTTTAGCGTC
CGGGTCACCACCTTGCTCTGTGAAGGAGGAGCTCAGCCCCATGATGTGCG
GCATGGGTCCCAACAAAGACCCCATGATGGGCAAACCGTTCCCCAGCGGG
GCCCCTTTCCCGATGGGCATGTACAACCCCCACCAT------CTCCAGGC
CATGTCTAATTCTGGGGTGGGTCACCCG------CACCCGTCCCTGATGC
CCAGTCCCTTGTCTGCAGCTATGGGCNNNNNNNNNNNNNNNNNNTATCTCATCTACGCCTCTTTCTCA
TTCATGGGATGTTTACAGATCAGTGATGGATCAAATATAGTGAACCTGCT
GGCCAGTAACTCCCCGAGTGTTTCGTACGCTACGACGCAGCAGAAATACT
TCAGTAACTACAGTCCCGTGATCGGCTTCTACATTTACGAGCCCATTGAG
TACTGGAACTCCACGGTGCAGGAGCATCTGAAGACCCTGAGTCATGGCTT
CAACAAGATCTCTTGGATGGACAACTTTTTCCACTACCTGCGTGTGGTGA
ACGTGAGCGCGTCGACCAAGGGCGACTTCATCTCCATCCTCAAGGGCTCC
TTCCTCCGCAGCCCGGAGTACCAGCACTTCACTGAGGACATCATATTCTC
CAAGA---ACCGCGAGACTG------ATGAGTACGACATTATCGCCTCGC
GGATGTACCTGGTGGCACGGACGACGGAGAAGAAGCGCGAGGAGGTGGTG
GAGCTTCTGGAAAAGCTTCGTCCCTTGATGCTGATCAACAGCATCAAGTT
CATTGCCTTCAATCCCACGTTTGTGTTCATGGACCGCTACAGCTCGTCCG
TCATCTCGCCCATCCTGACCTCAGGGTTCAGCGTACTCACAATCCTCATC
CTCACTTTCTTCCTGGTCATCAACCCGTTGGGGAACTTCTGGCTCATCCT
CACGGTAACGTCCGTGGAGCTGGGCGTCTTGGGTTTGATGGGCTTTCACC
AGTTTGAGTGGCAGCCAGCTCTCAAGAATGTGTCTACATCTTGCAGTGTT
GGCATTATTAATGGGCTCTCTGGATGGGCTTCCTCGGTGGATGATTTTCC
AGCTGACACCATCACTAGACGGTTTCGCTACGATGTGGCTCTGGTGTCAG
CATTAAAGGATCTGGAGGAGGACATCATGGAGGGGCTGAGAGAGAGTGGG
ATGGAAGACTGTGCTTGCACCTCAGGCTTCAGTGTCATGATCAAGGAATC
CTGTGATGGCATGGGCGATGTCAGCGAGAAGCACGGTGGAGGACCAGCTG
TTCCTGAGAAGGCTGTGCGTTATTCTTTCACTGTTATGTCTGTCTCTGTC
CTGGCAGACGAGCAGGAG--------------------------------GA
AGAGGTTACCATCTTCACTGAGCCAAAGCCAAACTCCGAACTGTCCTGTA
AGCCCCTTTGCCTGATGTTTGTGGACGAGTCAGACCATGAGACGCTCACA
GCTGTCCTGGGGCCTGTAGTTGCCGAGCGTAATGCAATGAAAGAGAGCAG
GCTCATTCTGTCCGTGGGTGGACTACCTCGTTCCTTCCGCTTTCACTTCA
GAGGCACGGGATATGATGAGAAGATGGTGCGCGAGGTTGAGGGCATGGAG
GCCTCAGGGTCCACCTACGTCTGCACTCTTTGCGACTCCACTCGGGCAGA
GGCCTCTCAAAACATGGTGCTACACTCCATCACTCGCAGTCATGAAGAGA
ACCTAGATCGTTACGAAATATGGAGAACCAACCCCTTTTCTGAGTCTGTA
GATGAGCTGCGGGACAGGGTCAAAGGGATCTCTGCTAAGCCCTTCATGGA

GACCCAGCCAACGATGGATGCATTACATTGTGACATCGGCAATGCCACTG
AGTTCTACAAAATCTTCCAGGACGAGATCGGAGAGGTGTACCAAAAGGT-
--CAAA---CCCAGCCGGGAGGAACGGCGCAGCTGGAGGGCAGCCCTGGA
TAAACAGCTAAGGAAGAAAATGAAGCTTAAACCGGTGATGAGGATGAATG
GGAACTATGCCCGCAGGCTGATGACCCAAGAGGCTGTGGAGGTGATCTGT
GAGCTGGTGCCCTCTGAGGAGAGGAGGGAGGCCCTGAGGGAGCTGATGAG
GATCTACATTCAGATGAAGCCTGTGTGGCGCGCCACCTGCCCTGCCAAGG
AGTGCCCCGACCAGCTGTGCCGCTACAGCTTCAACTCCCAGCGCTTTGCT
GACCTTCTCTCCTCCACCTTCAAATACAGGTACAATGGAAAGATAACCAA
TTACCTGCACAAGACCCTGGCCCATGTGCCCGAAATCATAGAGAGAGATG
GATCCATAGGAGCCTGGGCCAGCGAAGGGAACGAGTCTGCAAACAAGTCG
TACACCATCGAGATGGGCCCCGCGGGGCCCCTGTGGAAGGAGAGCCCGCA
ACCGTTTTCCTGCTCCATCGAGGACCCCACGAAACAGACAAAGTTCAAGG
GCATCAAGACGTACATTTCGTACCGGGTCACGCCAAGCCACACGGGGCGC
CCCGTCTACCGGCGCTACAAGCACTTCGACTGGCTGTACAACCGCTTGCT
GCACAAGTTCACTGTGATCTCCGTGCCTCACCTGCCCGAGAAGCAGGCCA
CGGGGCGCTTCGAGGAGGACTTCATCGAGAAGCGCAAGCGGCGACTGATT
CTGTGGATGAACCACATGACCAGCCACCCGGTGCTCTCCCAGTACGAGGG
CTTCGAGCACTTCCTGATGTGCGCCGACGACAAGCAGTGGAAGCTGGGCA
AGAGGCGGGCGGAGAAGGACGAGATGGTGGGCGCCCACTTCATGCTGACC
CTGCAGATCCCCAACGAGCACCAGGACCTTCAGGACGTCGAGGAGCGCAT
CGACACCTTCAAGGCCTTTGCCAAGAAAATGGACGACAGCGTGATGCAGC
TCACGCACGTCGCCTCGGAGCTGGTGCGTAAGCACCTGGGCGGTTTCAGG
AAGGAGTTCCAGCGGCTGGGGAATTCTTTCCAGTCCGTCAGCCAGGCGTT
CATGCTGGACCCCCCCCAAAGATCAGAGGCCCTCAACAAAGCCATCTCCC
ACCCTCTCGCCACGTTCCTCAAACTGACCTCTCTGGGTTTCATCATTGGA
GTCGGTGTGGTTGGAAACCTCCTGATCTCCATCCTGCTGGTCAAAGACAA
GAGTCTGCACCGAGCGCCCTACTATTTCCTGCTGGACCTGTGCGCCTCTG
ACATCCTGCGCTCTGCCATCTGCTTCCCCTTTGTCTTCACCTCGGTCAAG
AATGGATCGGCCTGGACCTACGGCACGCTGACCTGCAAGGTGATCGCCTT
CCTGGGTGTGCTCTCCTGTTTCCACACGGCGTTTATGCTCTTCTGCGTCA
GCGTCACACGCTACCTGGCCATCGCACACCACCGTTTCTACACCAAGAGG
CTGACCTTCTGGACCTGTCTAGCCGTCATCTGCATGGTGTGGACGTTGTC
CGTGGCTATGGCGTTCCCGCCGGTGCTAGATGTAGGGACGTACTCTTTTA
TCCGGGAGGAGGACCAGTGCACGTTCCAGCACCGCTCCTTCAGGGCGAAT
GATTCGCTGGGCTTCATGCTCCTGCTGGCGCTCATCCTCCTGGCCACACA
GCTGGTTTACCTCAAGCTCATCTTCTTCGTGCACGACCGTCGGAAGATGA
AGCCTGTCCAGTTTGTGCCTGCTGTCAGCCAGAACTGGACCTTCCACGGG
CCAGGCGCGAGCGGGCAGGCGGCGGCCAACTGGCTGGCGGGATTCGGCCG
AGGCCCCACCCCGCCTACCTTGCTGGGCATCCGGCAAAACAGCAACGCAG
CGGGCCGCAGGCGTCTGCTGGTATTGGATGAGTTCAAAACAGAGAAGAGG
ATTAGTAGGATGTTCTACATCATGACGTTTTTCTTCCTGGCGCTGTGGGG
GCCCTATCTGGTCGCCTGCTACTGGCGGGTGTTTGCAAGGGGCCCTGTGG
TCCCTGGGGGCTACTTGACGGCCGCCGTGTGGATGAGCTTTGCCCAGGCC
GGGGTCA-TCCTTTCATCTGCATCTTCTCANNNNNNNNNGCCAAATCTCGCTTTCACCC
TGGCGTGGGGACTGGTCCTGGCACGGAGC---GCAGCGTCCCACTCGGCA
ACAGCTTGCTGTCCCCGCAGCAAACCGACGATCCCACTGTTGCCACCCCC
CCGCAGCGATGGTTTGTCACCC---CTGCCAACAACCGACTGGACTTTGC
TGCCTCGGCATACGACGCCGCC---------GATTTCGCCGGTAACGCGG
CCACCTTGCTGTCCTACGCAGCGGCCGGAGTGAAGGCTC------TTCCC
CTGCCGACTGCAGGCTGCTCCAACCGGCCTCTTGGCTATTACGCAGACCC
GTCGG---GCTGG---GGAGGACGCACGCCGCCGCAGTACTGCGGCGTGA
ACAGCAAATCCAGCTCGGTCTTTTCCTGCTGGCCCGCTAACTCTCTCGGC

GGCCGAGCAGGTA---CC---AACTACCTGG---------CCGAGGA---
AGGA---GACTC---CATCACGACGGAGAGGTCGCCA---AT---CGGCG
GCTCGGACGAG---ACCAAACCCAAAGACATGAC---ATCCGA---GTCG
AGCTGGATAGAG---ACGCCGTCCTCCATCAAGTCGATTGATTCGAGCGA
TTCTGGTATCTTTG---AACAGGCCAAACGGAGAAGAATCTCACCTTCTG
CCACGCCG---------GTTTCAGAGACAGTGTCCCCGCTAAAATCTGAG
CATCACTCAACAGGCGAAGTCACAGAGAGAGAAGTAGCGTTGGGGATAAA
TCCGTTCGCGGATGGATGGGCGCCTTCAAAATAAACCACAGCTCCCACG
ACATCGGCTCCGG---ACAAACGGCGTTTTCCTCCCAGGCG---CCCGGC
TAC---GCGGCAGCCGCCCTGGGA---CACCATCA------CCACCCGAC
CCACGTTGGCTCT---TACTCCACGGCGGCTTTCAACTCCACCAGGGACT
TTCTCTTCAGAAATCGGGGCTTCGGGGACGCCACCGG------------G
GCGCAGCACAGTTTGTTCGCCTC---------CGGAAGTTT---C-----
-GCAGGGCCACATGGACACTCGGATGCAGCGGGGCACCTGCTCTTCCCGG
GGCTCCACGAG---CAGGCGGCGAGCCATGCGTCTTCCAACGTGGTCAAC
AGCCAGATGCGGCTGGGCTTCTCGGGGGACATGTACGGACGGGCCGACCA
GTACGGCCACGTTACGAGCCCGCGGT---CCGACCACTACGCTTCGACCC
AGCTGCACGGCTATGGCCCCATGAACATGAATATGGCCGCA---CACCAC
GGCGCAGGGGCCTTCTTTCGATACATGAGGCAGCCGATCAAGCAAGAGCT
CATCTGCAAGTGGATCGAGCCGGAGCAACTGACGAATCCCAAGAAGTCGT
GCAACAAAACTTTTAGCACGATGCACGAGCTTGTGACCCATCTGACGGTG
GAGCATGTGGGGGGACCGGAGCACACCAACCACATCTGCTTCTGGGAGGA
ATGCGCCAGAGAGGGAAAGCCATTCAAAGCCAAATACAAACTTGTAAATC
ATATCAGGGTACACACCGGAGAAAAGCCCTTTCCGTGTCCGTTCCCCGGC
TGTGGCAAA
>Cromeria nilotica
AGCCTTTTAATTCGAGCAGAACTCAGCCAACCCGGCTCACTTATAGGCGA
CGACCAAATTTATAATGTAATCGTCACAGCACATGCTTTCGTAATAATTT
TCTTTATAGTAATGCCAATTTTAATTGGGGGCTTTGGGAACTGGCTAATT
CCACTTATAATTGGGGCCCCAGATATGGCCTTTCCCCGAATAAATAATAT
AAGTTTCTGGTTACTCCCCCCGTCATTCCTTCTGCTCTTGGCTTCCTCCG
GCGTAGAGGCAGGTGCCGGGACAGGCTGAACTGTTTATCCTCCCTTAGCA
GGTAATCTAGCACATGCCGGGGCCTCTGTAGACCTTACAATCTTTTCGCT
GCACCTAGCAGGTGTGTCATCTATCCTTGGTGCAATTAACTTCATTACCA
CAATTATTAACATGAAACCTCCAGCAATCTCACAATACCAAACCCCCTTA
TTTGTGTGAGCCACTTTGATCACAGCAGTACTTCTTCTGCTTTCCCTTCC
AGTGTTAGCTGCCGGGATTACAATGCTACTTACAGACCGAAATATTAACA
CCACATTCTTCGACCCCGCTGGAGGTGGTGATCCCATCCTATACCAGCAC
TTGTTCTGATTCTTTGGCCACCCAGAAGTATATATTTTAATCCTGCCAGG
CTTCGGTATAATTTCTCATATCGTTGCATACTATGCGGGCAAAAAAGAGC
CCTTCGGGTACATAGGGATAGTATGGGCCATGATAGCAATCGGACTTCTG
GGGTTCATTGTCTGAGCCCATCATATGTTCACCGTAGGCATGGACGTAGA
CACACGGGNNNNTTCCTGGAGAAGAACTTTCACCCATCAAATTGCTTAGGAATG
TGCTTTCTCTGTGATGCCCATCAATGCACACAGCTCTTCCAGCTTTCCTG
GAACATGTGCCTGAGTAACTTTCCAGCTATCTGCAAGACCGAGGAGTTCC
TACAGCTTCCCAAAGACATGCTTGTCCAGCTCCTGTCCCATGAGGAACTG
GAGACAGAGGACGAGCGGCTTGTATACGAGTCAGCACTCAACTGGGTCAA
CTATGATCTGGAACGTAGACACTGCCACTTGCCAGAGCTGCTGCGTACGG
TTCGGCTAGCTCTGCTTCCCGCCATCTTCTTGATGGAAAACGTTTCAACA
GAGGAACTGATAAATGCACAAGCCAAAAGTAAAGAGCTGGTGGACGAAGC
GATTCGCTGTAAGCTGCGCATTTTGCAAAACGATGGAGTGGTAAACAGTC
CCTGTGCCCGTCCTCGCAAAACTAGCCATGCACTCTTTTTATTGGGTGGT
CCCACCTTCATGTGCGATAAGCTGTACCTGGTGGACCAGAAGGCCAAAGA

AATCATTCCTAAGGCTGACATCCCTAGCCCACGCAAAGAATTCAGTGCCT
GTGCTATTGGCTGTAAAGTGTATGTGACGGGTGGGC--GTGGCTC-TGAA
AATGGTGTGTCTAAGGACGTGTGGGTGTATGACACACTGCATGAGGAATG
GTCTAAAGCAGCACCAATGTTAATAGCACGATTCGGCCATGGATCGGCAG
AGTTGCGGCATTGCCTGTATGTGGTTGGTGGTCACACAGCNNNNNNNNNNNNNNNNNNNNNNNNNNNN
NGGATGATTAC
ATTGTCGTTTTAGTCGTTCTAACAACAGGTTGATCTTTAATGAACCAGA
ATTGATTCTAGCTCTTGCTCAAGAATTCCAAATGAGAACCGTCACTGTAT
CCCTGGATGAACAGTCATTTCCAAACATTGTCCAGATCATTAGTGGAGCG
TCAATGTTGGTCAGCATGCATGGAGCTCAATTGGTTACCTCCATGTTTCT
CCCTCGAGGTGCAGTTGTGATTGAACTCTTCCCCTTTGGAGTTAAACCAG
ATCAGTACACACCATACAAAACTCTTGCCTCCTTGCCTGGAATGGACCTC
CAATACGTTGCATGGAGAAACACAATAGAGGAGAATTCGATAGCCTACCC
TGACCGTCCCTGGGATCAGGGTGGCATCGCTCACTTGGACAAGGAGGAAC
AGGACCGCATAATTGCCAGCAAGGAGGTACCACGTCACTTGTGTTGTCGC
AACCCAGAGTGGCTATACCGCATCTATCAAGACACGATTGTGGACATTCC
CACCTTTATGGAGGTAGTTCG---GGAAGGTCTTAAG---GCCAAATTAA
ATTTGAAAAA---GACCAAGGCCGCCAGCACAGTGCACCCTGGCCGCGTT
CGAGATCCCAAATGCCAGACTTCTGTGCAGGCGACCAGTGAAGCCAAGTT
GACTGTGTCTTGGCAGATTCCTTGGAACCTCAAATACCTGAAAGTTCGGG
AAGTCAAATACGAAGTGTGGATACAAAAAAAGGATGCTAGCAAAGGGACA
CTGGAAGATCAAATTATTCAGGCTAACCCTGCACTGGAGGCTTTTGGCAA
TGCCAAAACAGTAAGAAATGACAATTCCTCCCGCTTTGGAAAGTTCATTC
GCATTCATTTTGGAACAAGTGGTAAACTAGCTTCTGCTGACATAGAGACT
TACCTACTTGAAAAGTCTCGTGTAACCTTTCAATTAAAATCTGAAAGGAA
CTACCACATCTTTTTTCAGATATTGTCTGCTGAAAAGCCAGAGTTGTTGG
ATATGTTATTGATTACCAACAATCCTTATGATTACTCTTACATTTCCCAA
GGAGAAGTAACAGTCGCATCCATCAATGACAACGAAGAACTTCTGGCTAC
TGACAATGCTTTTGATGTTCTTGGTTTTACAGCAGATGAAAAGATGGGTG
TCTACAAGTTGACGGGTGCAATAATGCACTACGGCAACATGAAATTCAAG
CAGAAGCAGCGAGAGGAGCAGGCAGAGCCTGATGGAACTGAGTCTGCAGA
CAAGTCAGCCTACCTAATGGGGCTAAACTCAGCAGATCTCCTAAAGGGAC
TCTGCCATCCGAGAGTTAAAGTAGGCAATGAGTATGTTACAAAAGGACAG
AATGTGGATCAAGTCTACTACCCCAACAAAGAGGCCTTTAAGTGTGAAGA
GTGTGGTAAGCACTACAACACCAAGCTAGGTTACAAGCGCCACGTAGCCA
TGCACTCTGCTACAGCCGGTGATTTGACTTGCAAGGTGTGCCTGCAGAGC
TATGAGAGTACTCCAGCTCTTTTAGAGCACTTGAAGAGTCATTCAGGCAA
ATCATCAGGGAGCACCAAGGAGAAGAAGCATCCATGTGACCACTGTGATC
GGCGCTTCTACACGCGTAAAGACGTACGCCGTCACATGGTCGTGCACACA
GGTCGCAAGGACTTCCTGTGCCAGTACTGTGCACAACGTTTTGGCCGCAA
AGATCACCTGACACGGCATGTCAAGAAGAGCCACTCACAGGAGTTACTGA
AGATCAAGACAGAACCTCCGGACATGTTAAGCCTGTTGGGGTCTGGATCT
CCACCCTGTGCTGTGAAGGAAGAACTCAGCCCAATGATGTCAGGCATGGG
GCCTGCCAAGGACCCCCTGATGGCTAAGCCTTTTCCCACAGGAACCCCTT
TTCCCATGGGTATGTATAACCCCCACCAC------ATCCAGGCCATGTCT
AATCCCGGTGTGGGTCAC---------CACCACACCTTGGTACCTGGCTC
TCTGTCTGCTGCTATGGGGATGGGCTGTCATATGGAATATTTAATTTATG
CTTCTTTTTCCTTCATGGGATGTTTACAAATTAGTGATGGTTCCAACATT
GTGAACCTACTTGCTAGCAACTCTCCTAGTGTATCATTTGCTCTGACGCA
GCAGAAGTACTTCAGTAACTACAGTCCAGTTATTGGGTTCTACATTTATG
AGCCCATTGAGTATTGGAACACTACTGTGCAGGAACACCTTAAGACGTTA
GGACATGGTTTCAACAAGATTTCGTGGATTGATAATTACTTTCATTACCT
TAAAGTGGTGAATGTCAGTGCATCAACCAAAAGTGATTTATAAACATCC

TCAAGAATTCGTTCTTGAGGAGTCCTGAGTATCAACACTTCATGGAGGAC
ATTATTTTCTCCAAAA---ATGGAG------------ATGAGTATGACAT
TATTGTGTCCCGGATGTACTTGGTCGCCAGAACCACGGAGAAGACCCGAG
AGGAGGTTGTGGAGCTGCTGGAGAGGTTAAGACCTCTGTCCCTAATAAAC
AGCATCAAATTTATTGTCTTTAACCCTACCTTCGTCTTCATGGACCGCTA
CAGTTCTTCRGTCATTTCACCTATACTGACGTCAGGCTTTAGYGTTTTGA
CCGTCATTATTCTTACGTTCTTCCTAGTCATTAATCCACTGGGGAACTTC
TGGTTGATACTAACCGTCACTTCTGTGGAGTTAGGGGTTCTGGGTCTCAT
GNNNNNNNNNNNNNNNNNNNNNNNNNNNNNNNNNNNNNNNNNNNNNNNNNNNNNNNNNNNNNNNNNNNNNN
NNNNNNNNNNNNNNNNNNNNNNNNNNNTGGATGATTTCCCTGCAGACACCATCGCTCGGCGTTTTCGCT
ATGATGT
TGCCCTGGTTTCAGCTTTGAAAGACTTGGAAGAGGACATTATAGAGGGGC
TAAGGGAGAGAAGGCTGGATGACAGCCTCTGCACCTCTGGTTTTAGTGTG
GTTATCAAAGAATCATGTGATGGTATGGGAGATGTTAGTGAAAAACATGG
AGGTGGTCCATCAGTTCCTGAAAAAGCAGTAAGATATTCCTTCACTGTGA
TGTCTATTTCTGTTCAAGCTGAAGGGGAAGAT------------------
------------GAGGCAGTCTCTATTTTCCAGGAAATGAAGCCCAACTC
TGAGCTATCTTGCAGACCTCTGTGTCTCATGTTTGTTGACGAATCAGACC
ACGAAACGCTCACGGCTATCCTGGGGCCTGTAGTGGCTGAACGTAAGGCC
ATTAAAGAGAGTCGCCTCATTCTGTCAATTGGAGGGATGCCCCGATCTTT
TCGATTTCACTTCCGGGGCACTGGATATGATGAAAAAATGGTCAGAGATA
TGGAGGGCCTAGAGGCATCAGGTTCCACCTATGTCTGCACACTTTGTGAC
TCTACCCGAGCAGAGGCCTCTCAAAACATGGTGCTGCATTCCATCACTCG
AAGCCATGAAGAGAACCTTGAACGATACGAAATCTGGAGAACAAATCCAT
TTTCTGAGTCTGCAGATGAACTCAGAGATCGGGTAAAAGGTGTATCAGCC
AAGCCTTTTATGGAGACTCAGCCAAGTTTGGATGCTTTGCACTGTGACAT
TGGTAATGCCACTGAGTTCTACAAGATCTTTCAGGATGAAATTGGTGAAG
TACACCTGAAAAA---TAAC---CCAGGACGTGAAGAACGTCGTCGCTGG
CGCTCAGCTCTAGACAAACAGCTGAGGAAAAAATTGAAACTGAAACCAGT
AATGAGAATGAATGGTAATTACGCAAGACGTCTAATGACCCTAGAGGCAA
TAGATGCTATTTGTGAGCTTGTGCCATCTGAGGAACGTCGCAAGGCTTTG
AGGGAACTTATGGAACTTTACATCCAGATGAAACCAGTGTGGCGCTCAAC
ATGCCCAGCACGTGAATGCCCGGATCAACTGTGCCGCTATAGTTATAACT
CTCAACGCTTTGCTGAGCTTCTGTCCTCCACATTCAAATACCGATATGAT
GGCAAAATTACCAACTATCTTCATAAGACCCTGGCTCATGTTCCTGAGAT
TGTTGAGAGGGATGGATCTATCGGTGCATGGGCAAGTGAAGGGAATGAAT
CTGGAAACAAGNNNNNNNNNNNNNNNNNNNNNNNNNCCAGGACCTCAGTGGAAGGAGAACCCCAAGCCTTT
TAAT
TGTTCCATTGAAGATCCTACCAAACAAACCAAGTTCAAGGGTATAAAGAC
GTACATATCATACCGCGTGACCCCCAGCCACACTGGTCGCCCTGTGTATC
GTCGTTACAAGCACTTTGATTGGCTGTATAATAGGCTACTGCACAAGTTC
ACTGTTATCTCAGTGCCTCATTTACCTGAGAAGCAAGCAACTGGCCGCTT
TGAGGAGGACTTTATTGAGAAGCGCAAGAGACGCCTCATTTTATGGATGG
ACCACATGACCAGTCACCCTGTTTTATCACAATATGAGGGCTTTGAACAT
TTTCTGATGTGTGCTGATGATAAGCAGTGGAAACTTGGGAAACGAAGAGC
GGAKAAGGATGAGATGGTAGGAGCGCACTTTATGCTCACTTTACAGATTC
CCAATGAGCACCAGGACTTGCAGGATGTCGAAGAACGAGTGGACTCTTTT
AAGGCCTTTGCAAAAAAGATGGATGACAGTGTCATGCAGTTGACCCATGT
GGCCTCTGAGCTGGTACGTAAGCACCTGGGGGGCTTCCGAAAGGAGTTTC
AGAGACTGGGTAATGCATTCCAATCTATCAGTCAGGCCTTCATGCTGGAT
CCACCTTATTGCTCAGATGCTTWAAACAATGCCATCACTCACNNNNNNNNNNNNNTTCCTCAA
ACTGACGTCTTTGGGTTTTATCATTGGTGTCGGAGTGGTCGGTAATCTCC
TGATCTCCATCCTTCTCGTTAAAGACAAAAGCTTGCACCGTGCCCCCTAC

TATTTCCTTTTGGACCTGTGCGCCTCTGACATCCTTCGTTCAGCAATCTG
TTTCCCCTTTGTGTTTACTTCAGTCAAAAATGGCTCAGCATGGTCATATG
GCACACTTACCTGCAAAGTGATTGCCTTCCTGGGTGTTCTATCGTGTTTC
CACACCGCCTTTATGCTTTTCTGTGTCAGTGTTACGCGGTACCTGGCCAT
CGCCCATCACCGCTTCTATACCAAGAGGCTCACCTTCTGGACTTGCCTGG
CTGTTATCTGTATGGTGTGGACGTTRTCAGTCGCCATGGCATTCCCACCA
GTGTTAGATGTGGGCACATATTCGTTCATCCGTGAAGAAGACCAGTGTAC
ATTTCAGCACCGCTCTTTCCGTGCCAACGATTCTCTGGGCTTCATGTTAC
TCCTTGCCCTCATTCTCCTGGCTACCCAGCTTGTCTACCTCAAGCTCATC
TTCTTTGTCCATGATCGTCGAAAAATGAAGCCTGTCCAGTTCGTTCCAGC
CGTTAGCCAGAACTGGACCTTCCATGGCCCWGGAGCCAGTGGCCAGGCAG
CAGCTAATTGGCTGGCTGGATTTGGCCGTGGTCCCACCCCACCCACACTA
CTGGGGATAAGGCAGAACAGCAACGCGGCAGGCCGCAGAAGACTGCTGGT
GTTGGATGAGTTTAAGACTGAAAAGAGGATTAGCAGGATGTTCTACATTA
TGACCTTCTTTTTCCTTAGCTTGTGGGGACCCTACCTGGTGGCTTGCTAC
TGGAGAGTGTTTGCCAGGGGTCCAGTTGTCCCTGGGGGTTACCTGACAGC
CGCTGTGTGGATGAGCTTTGCCCAGGCTGGAGTCAACCCCTTCATTTGCA
TCNNNNNNNNNNNNNNNNGCCAAATCCCGCTTTCACCCTGGCGTCGGGAGCGGTCCTGGCACGGAC
C---GCAGCGTCCCACTCAGTAACAGCTTGCTATCACCGCAACAAACCGA
GGAGACCGCTGTGG---CCTCCCCGCAGCGATGGTTTGTCACCC---CTG
CCAACAACCGACTGGACTTTGCCGCGTCAGCTTACGACGCCGCTGCGGCT
GCTGATTTCGCCGGTAACGCTGCGACCCTTCTGTCGTATGCGGCTGCTGG
AGTAAAAGCGC------TTCCATTGCCCACCGCAGGCTGCTCCAACAGGC
CATTAGGTTACTATGGAGACCCGCCGG---GTTGG---GGCACGCGTACT
CCTCCTCAGTATTGC---------AGTAAATCTAGCGCAGTTCTCGCGTG
TTGGCCGTCCAACTCTGTGGGTAGTAGAACGACCA---CTACCGGTTATC
TGA---CCGGCTTGGAGGATGGTGGT---GACCC---CATAGCGCCTGAA
AGATCCCCT---CT---CGGCGGAGCAGAGGAA---ACCAAGCCGAAAGA
CCT------TACGGA---ATCCAGCTGGATAGAG---ACACCGTCCTCTA
TCAAGTCCATTGATTCAAGCGATTCTGGAATTTTTG---AACAAGCCAAA
CGGAGGAGGATTTCGCCATCTGCCACACCA---------GTTTCAGAGAC
CTCCTCTCCTTTAAAATCTGAA---------ACAGGCGACGTTACAGACA
GAGAAGTGGCTTTGGGGATAAATCCGTTCGCCGACGGCATGGGTGCTTTC
AAAATTAACCACAGCTCCCACGATCTTGGCTCTGG---GCAAACGGCGTT
TTCCTCACAGGCT---CCCGGCTATGCAGCCGCTGCTGCCCTGGGACATC
ACCATCA------TCACCCTACTCATGTCAGTTCC---TACTCCACGGCG
GCTTTCAACTCAACACGGGACTTTCTTTTTCGGAATCGGGGGTTTGGAGA
CGCCACCAG------------CGCGCAACACAGTCTTTTTGCTTCTGC--
-CGCGGGAAGTTTTGCT------GCAGGGCCACATGGACATTCAGATGCC
GCGGGGCACCTGCTCTTTCCGGGATTGCACGAGGCTCAGGCGGCGACACA
TGCTTCCTCCAACGTGGTGAATGGTCAAATGCGCCTGGGCTTTTCCGGGG
ACATGTATGGCAGGGCTGACCAATACGGCCACGTTACGAGCCCTAGGT--
-CCGAGCATTACGCGTCGACTCAGTTGCATGGCTATGGCCCTATGAACAT
GAACATGGCTGCC---CATCACGGCGCCGGGGCCTTCTTTCGATACATGA
GACAGCCCATCAAACAAGAGCTCATCTGCAAGTGGGTCGAGCCCGAGCAG
CTTACGAATCCCAAAAAGTCGTGTAACAAAACTTTCAGCACCATGCATGA
GCTGGTTACCCATCTCACGGTGGAACACGTTGGGGGACCCGAACAATCCA
ATCACATTTGCTTTTGGGAAGAGTGTGCCCGGGAAGGAAAACCTTTTAAA
GCCAAGTACAAACTTGTAAATCATATCAGAGTCCACACCGGAGAAAAGCC
TTTTCCTTGCCCATTTCCGGNNNNNNNNNNN
>Cryptopsaras couesi
AGCCTATTGATCCGTGCTGAGCTCAGCCAGCCAGGCACACTTTTAGATAA
CGACCAGATCTACAACGTTGTTGTAACAGCGCATGCCTTTGTAATAATTT

TTTTTATGGTTATACCAATTATGATTGGAGGCTTTGGGAATTGGCTAATT
CCTTTAATGATCGGAGCGCCTGATATAGCGTTCCCTCGAATAAATAACAT
AAGCTTCTGGTTACTTCCCCCTTCTTTCTTTCTCCTGCTGGCATCCTCAG
GCATTGAAGGGGGCGCAGGAACAGGGTGAACAGTATACCCGCCCCTCGCA
GGTAACTTGGCGCACGCTGGGGCATCTGTAGACCTAACAATTTTCTCTCT
ACACCTGGCAGGTGTGTCCTCAATTTTAGGGGCTATCAACTTTATCACAA
CAATTATTAATATAAAACCTCCCGCCATGTCACAGTACCAGACACCTTTG
TTCGTATGGTCCGTGTTAATTACGGCCGTATTACTTTTACTCTCACTACC
CGTTCTTGCGGCTGGTATCACCATGCTACTAACAGACCGTAACCTAAATA
CAACATTTT---

------------NNNNNNNNGAGAAACCTTCACCCGWCTAACTGCCTTGGCATGCTGT
TGCTGTCTGATGCCCACCAGTGCACCAAGCTGTCAGAGCTCTCTTGGGGC
ATGTGCCTCAGCAACTTCCCCACTATTTGCAAGACAGAGGACTTCCTCCA
ACTACCCAAAGATATGGTGGTGCAGCTTTTGTCACATGAAGAGCTGGAGA
CAGAAGATGAGAGACTGGTTTATGAAGCTGCCCTGAACTGGATCAACTAT
GACCTAGAAAGGAGGCACTGTCACCTTCCAGAGCTCCTGAGAACGGTCCG
TCTTGCCCTGCTGCCTGCCATCTTTCTAATGGAGAATGTCTCGACAGAAG
AGCTGATCAATGCCCAGGCCAAGAGCAAGGAGCTGGTGGATGAGGCTATC
CGCTGTAAGCTGAAGATCCTGCAGAATGACGGCGTCGTTAACAGCCCGTG
TGCTCGACCAAGAAAAACCAGCCATGCTCTTTTTCTCCTGGGAGGGCAGA
CTTTCATGTGTGACAAGTTATACCTGGTGGATCAGAAGGCCAAAGAGATC
ATCCCAAAGGCAGACATTCCCAGCCCCAGGAAAGAGTTCAGCGCCTGTGC
CATCGGCTGTAAGGTGTACATCACAGGTGGGA--GAGGCTC-CGAGAATG
GCGTGTCCAAAGATGTATGGGTCTATGACACCGTCCATGAGGAATGGTCC
AAAGCGGCGCCCCTGCTCATTGCCAGGTTTGGTCATGGCTCTGCTGAGCT
GAAACACTGCCTCTATGTCGTAGGAGNNNNNNNNNNNNNNNNNNNNNNNNNNNNNNNNNNNNNNNNNGGA
TGAATACATTGTTGTGTTTAG
TCGTTCAACAACAAGACTAATACTGAATGAAGCGGAGCTAATAATGGCAC
TGGCTCAGGAGTTCCAGATGAGAGTGGTCACAGTATCCCTGGAGGAACAG
TCGTTCCCTAGTATCATCCAGGTGATCAGCAGTGCTACCATGTTAGTCAG
TATGCATGGAGCTCAACTTATCACCTCACTCTTCCTCCCCAGAGGAGCTG
TTGTGGTGGAGCTTTTCCCCTTTGCTGTGAACCCAGAGCAGTACACCCCA
TATAAAACCCTTGCCTCCCTTCCGGGCATGGACCTGCATTATATCTCCTG
GAGGAACTCTAAGGAGGATAACACCATCACCCACCCAGACAGGCCCTGGG
AACAAGGGGGTATTGCTCATTTGGATAAGGAGGAGCAAGAACGAATACTA
ATGAGTAAAGATGTGCCCAGGCATCTGTGCTGCCGAAACCCAGAGTGGCT
CTTCCGGATCTATCAGGATACATTGGTGGACATTCCCTCCTTCCTGGAAG
TCCTCAA---AGAGGACATGAAG---ACTAAGCCCAACTTGAAGAA---G
TCAAAGCCGGCCAGCAACCTCCACCCGGGCCGGGTCAGAGRACCTCAGTG
TCAGACTTCAGTTCAAACCAGTAATGAGGCTAAACTCACAGTGTCCTGGC
AGATACCGTGGAATCTGAAATACCTGAAGGTCAGGGAGGTCAAATATGAG
GTG---

-------CCCAACAAGGAGGCCTTCAAATGTGATGAGTGTGGGAAGCACT
ACAACACCAAGCTGGGATACAAGCGCCATGTTGCCATGCATTCAGCCACA
GCGGGGGATCTCACCTGTAAAGTGTGCATGCAGACCTACGACAGCACGCC
CATGCTCTTGGAGCACCTGAAGAGCCACTCTGGGAAGTCCTCAAGTGGCA
CCAAGGAGAAAAAACACCAGTGCGACCACTGTGACCGGCGTTTCTACACA
AGGAAGGATGTGAGACGCCACATGGTGGTCCACACAGGCCGAAAGGACTT
CCTCTGCCAATACTGTGCCCAGCGCTTCGGCAGGAAGGACCACCTGACAC
GCCATGTGAAGAAGAGCCACTCACAGGAGCTGCTGAAAATCAAGACAGAG
CCTCCTGATATGTTAAGTCTTTTAGCTTCTGGGTCACCACCATGCTCTGT
GAAGGAGGAGCTCAGCCCCATGATGTGCGGTATGGGTCCCAACAAAGACC
CCATGATGAGCAAACCCTTTCCTAGTGGGGCCCCTTTTCCAATGGGCATG
TACAACCCTCATCAT------CTCCAGGCCATGTCTAATTCTGGGGTAGG
TCACTCG------CACCCATCCCTGATGCCCAGTTCTTTGTCTGCAGCTA
TGGGCATGGGCTGTCACATGGAATACCTCATCTACGCCTCCTTCTCATTC
ATGGGATGTTTACAAATCAGTGACGGATCAAATATCGTGAACCTGCTGGC
AAGTAACTCTCCAAGTGTCTCATATGCTCTGACACAGCAAAAATACTTCA
GTAACTATAGTCCAGTCATTGGGTTTTACATTTATGAGCCCATCGAGTAC
TGGAACTCCACAGTGCAGGAGCACCTGAGGTCTCTGAGTCACGGCTTCAA
TAAGATCTCCTGGATGGACAACTTTTTCCACTACCTGCGGGTGGTGAACG
TGAGCGCTTCAACCAAGGGCGACTTCATCACCATCCTCAAGGGCTCCTTC
CTGCGCAGCCCGCAGTACCAGCACTTCACGGAGGACATCATTTTCTCGAG
GA---ACCGTGAGACCG------ATGAGTACGACATCATCGCCTCGCGGA
TGTACTTGGTGGCGGAGACCACAGAGAATAAGCGTGAGGAGGTGGTGGAG
CTCCTGGAAAAACTCCGTCCACTGATGCTGATCAACAGCATCAAGTTCAT
CGCCTTCAATCCCACGTTTGTGTTCATGGACCGCTACAGCTCCTCCGTCA
TCTCACCCATCCTGACGTCCGGATTCAGCGTACTCACAATCCTCATCCTC
ACTTTCTTTCTGGTCATCAACCCCTTGGGTAACTTCTGGCTCATCCTCAC
TCTAACGTCCATGGAGCTGGGCGTCTTGGGTTTGATGGGCTTTCACCAGT
TTGAATGGCAACCAGTTCTCAAGAATATGTCTACATCTTGCGATGTTGGC
ATTATTAATGGTCTCTCTGGATGGGATTCCTCTGTGGATGCCTCCCCAGC
TGACACCATCGCACGACGGTTTCGCTACGATGTGGCACTGGTATCAGCAT
TGAAGGATCTGGAGGAGGACATCATGGAGGGACTGAGAGAGACTGGAATG
GAAGACAATGCTTGCA------ACTTCACTGTCATGATCAAAGAATCCTG
TGATGGCATGGGCGATGTCAGTGAGAAGCATGGCGGAGGACCAGCTGTCC
CCATGAAGGTTGTGCGTTTCTCTTTCACGGTTATGTCTGTCTCTTTCTTA
GCAAACAGTCAGGAG------------------------------GAAGA
GGTCACCATCTTCAGGGAGCCAAAGCCAAATTCAGAACTCTCCTGTAAGC
CTCTTTGCCTGATGTCTGTGGATGAATCAGACCATGAGACACTCACAGCC
ATCCTGTCTCCTTTAATTGCAGAGCGTAAGGCAATGAAAGAGAGCAGGCT
CATCCTGTCCATTGGTGGACTCCCACGCTCCTTCCGCTTTCACTTTAGAG
CATCAGGATATGATGAGAAGATGGTGCGTGAGATGGAGGGCCTCGAGGCC
TCTGGGTCCACCTACATTTGCACTCTCTGTGACTCCAGTCGTGCACAGGC
CTCTCAAAACATGGTGCTGCACTGCATCACCCGGAGTCATGAAGAAAACC
TAGAACGTTATGAAACATGGAGAAGCAACCCATTTTCCGAGTCTGCGGAT
GAGTTGCGAGATAGAGTCAAAGGAGTCTCTGCCAAGCCCTTCATGGAGAC
CCATCCCACACTGGATGCCTTACACTGTGACATTGGCAATGCCACCGAGT
TTTACAAAATATTCCAAGATGAGATTGGGGAGGTGTACCAAAAGGT---C
AAT---CCCAGCCGGGAGGAGCGGCGCAAATGGAGGGCAGCCCTAGATAA

ACAGCTGAGGGAGAGGATGAAGCTCAAACCAGTAATGAGGTTGAACGGGA
ACTATGCCCGCAGGCTAATGACCATGGAGGCTGTGGAGGTGGTGTGTGAC
CTGGTGCCTACAGAGGAGAGGAGGGAGGCCCTGAGGGAGCTTATGAAACT
CTACCTCCAGATGAAGCCTGTGTGGCGCGCCACCTGTCCAGCCAAGGAGT
GCCCTGACCAGCTGTGCCGCTATAGCTTTAACTCCCAGCGCTTTGCTGAG
GTCCTCTCCTCGAAGTTCAAATATAGATACAATGGAAAGATAACCAATTA
CCTGCACAAGACTCTGGCTCATGTGCCTGAAATCATAGACAGAGATGGAT
CCATAGGAGCCTGGGCCAGCGAGGGGAATGAGTCGGCAAACAAA------

------NNNNNNNNNNNAGGCGAAGTCACAGAGAGAGAAGTGGCGTTGGGGATAAATCCGT
TCGCGGATGGGATGGGCGCCTTCAAAATAAACCACAGCTCCCACGATATT
GGCTCCGG---ACAGACGGCGTTTTCCTCCCAGGCG---CCCGGCTAC--
-GCAGCAGCCGCCGTGGA---CACCATCA------CCACCCGACYCACG
TTGGCTCT---TACTCCACGGCGGCTTTCAACTCCACCAGGACTTTCTC
TTCAGAAATCGGGGTTTCGGAGACGCCACCGG------------GGCGCA
GCACAGTCTGTTCGCCTC---------CGGAAGTTT---C------GCAG
GACCACATGGACACTCAGATGCAGCNGGGCACCTGCTCTTCCCAGGGCTGC
ACGAG---CAAGCAGCGAGCCATGCGTCTTCCAACGTGGTCAACAGCCAG
ATGCGGCTGGGCTTCTCGGGGGACATGTACGGACGGGCCGAYCAGTACGG
CCACGTTACGAGCCCGAGGT---CCGACCACTATGCGTCGACCCAGCTGC
ACGGCTATGGCCCCATGAACATGAATATGGCCGCA---CATCACGGAGCA
GGGGCCTTCTTTCGATACATGAGGCAGCCGATCAAACAAGAGCTCATCTG
CAAGTGGATCGAGCCGGAGCAGCTGACGAATCCCAAAAAGTCGTGCAACA
AAACTTTTAGCACGATGCACGAGTTAGTGACCCATCTGACGGTGGAGCAT
GTGGGAGGACCAGAGCAGACCAACCACATCTGCTTCTGGGAGGACTGCTC
CAGAGAAGGAAAGCCATTCAAAGCCAAATACAAACTTGTAAATCATATCA
GAGTACACACCGGAGAANNNNNNNNNNNNNNNNNNNNNNNNNNNNNNNNNNNN
>Cubiceps baxteri

------------TTCCTAGAGAGAAACCTTCACCCAACTAACTGCCTTGG
CATGCTGTTGCTGTCTGACGCCCACCAGTGCACCAAGCTGTCAGAGCTTT
CTTGGGGCATGTGCCTCAGCAACTTCCCCGCTATTTGCAAGACAGAGGAC
TTCCTCCAACTGCCCAAAGATATGGTGGTGCAGCTTTTGTCACACGAGGA
GCTGGAGACAGAAGATGAGAGACTGGTGTATGAAGCTGCCCTGAACTGGA
TCAACTATGACCTGGAAAGGAGGCACTGCCACCTTCCAGAGCTCCTGAGA
ACGGTCCGTCTTGCCCTGCTGCCCGCCATCTTTCTAATGGAGAACGTCTC
GACAGAAGAGCTGATCAACGCCCAGGCCAAGAGCAAGGAGCTGGTGGATG
AGGCTATCCGCTGTAAGCTGAAAATCCTGCAGAATGACGGCGTCGTAAAC
AGCCCGTGTGCACGACCAAGAAAAACCAGCCATGCTCTCTTTCTCCTGGG
AGGGCAGACTTTCATGTGTGACAAGTTGTACCTGGTGGACCAGAAGGCCA
AAGAGATCATCCCGAAGGCGGACATTCCCAGCCCAAGGAAGGAATTCAGC
GCCTGCGCCATCGGCTGTAAGGTGTACATCACAGGTGGGA--GAGGCTC-
AGAGAATGGCGTGTCCAAAGATGTATGGGTCTATGACACCGTCCACGAGG
AATGGTCCAAAGCGGCGCCGATGCTGATCGCCAGGTTTGGTCATGGCTCT

GCAGAGCTGAAACACTGCCTGTACGTGGTAGGAGGTCACACCGCAGCAAC
TGGCTGCCTCCCTGCTTCTCCATCCGGATGAATACATTGTTGTGTTCAGT
CGTTCAACAACAAGGCTGATACTGAACGAAGCCGAGCTAATCATGGCACT
GGCCCAGGAGTTCCAGATGAGAGTGGTCACAGTTACCCTAGAGGAACAAT
CTTTCCCCAGTATTGTCCAGGTGATCAGTGGTGCATCCATGTTAGTCAGC
ATGCATGGAGCTCAGCTTATCACCTCACTCTTCCTCCCCAGAGGAGCTAC
TGTGGTGGAGCTGTTCCCCTTTGCTGTGAACTCAGAGCAGTACACCCCAT
ATAAAACCCTTGCCTCGCTTCCAGGCATGGACCTTCATTATATTTCCTGG
AGGAACACTCAGGAGGAGAACACTGTCACCCATCCAGACAGACCCTGGGA
ACAAGGAGGCATCACTCACTTGGAGAAGGAGGAGCAGGAGCGAATACTGA
CCAGCAAAGACGTCCCCAGGCACCTGTGCTGCCGCAACCCGGAGTGGCTC
TTCCGAATTTACCAGGACACTTTGGTAGACATCCCTTCCTTCCTGGAAGC
CCTCAA---GGAAGGAATGAAG---ACCAAGCCCAGCTTGAAGAA---GT
CCAAACCAGCCAGTGCAGTCCATCCGGGCCGGGTCAGAGAAGCCCAGTGT
CAGACCTCAGTACAAACCACCAATGAGGCTAAACTCACAGTCTCCTGGCA
GATCCCATGGAATTTGAAATACCTGAAAGTAAGAGAGGTGAAGTACGAGG
TGTGGATCCAGAAAAGAGATACCAGCAAGGGAACTCTGGAGGATCAAATC
ATCCAGGCGAACCCGGCGCTGGAGGCCTTTGGAAATGCCAAAACGCTGAG
AAATGACAACTCATCCCGTTTTGGAAAATTCATTCGAATTCACTTCGGAA
ACAGTGGCAAGCTGTCATCTGCTGACATCGAGACATACCTGCTGGAGAAG
TCTCGTGTCACCTTTCAGCTCAAGGCTGAGAGGGACTACCACATCTTCTA
TCAGATCCTGTCCAATCAGAAGCCAGAGCTGCTGGACATGCTTTTAATCA
CCAACAACCCGTACGACTACTCCTACATCTCCCAAGGAGAGGTAACGGTC
GCCTCCATCAATGACTCGGAGGAGCTGATGGCCACTGACAGCGCCTTTGA
TGTGCTCGGCTTCACTCCGGAGGAGAAGATGGGCGTCTATAAACTGACTG
GCGCCATCATGCACTACGGCAACATGAAGTTCAAGCAGAAGCAGCGTGAG
GAGCAGGCCGAGCCTGACGGGACGGAGGCTGCTGATAAATCAGCTTACTT
GATGGGGCTGAACTCTGCTGACCTCATCAAAGGGCTGTGCCATCCCAGAG
TCAAGGTAGGAAATGAGTATGTTACCAAAGGCCAGAGTGTGGACCAAGTC
AACTACCCCAACAAGGAGGCCTTCAAGTGTGAGGAGTGTGGGAAGCACTA
CAACACCAAGCTGGGATATAAGCGCCATGTGGCCATGCACTCTGCCACGG
CAGGGGATCTCACCTGTAAAGTGTGCATGCAGAGCTACGAGAGCACACCT
GTTCTCCTGGAGCACCTCAAGAGCCACTCGGGGAAGTCCTCGGGCGGAGC
AAAGGAGAAAAAGCACCCATGTGACCACTGCGACCGCCGTTTCTACACAC
GGAAGGATGTGAGACGGCACATGGTGGTCCACACGGGCCGAAAGGACTTC
CTGTGCCAGTACTGTGCCCAGCGCTTTGGCAGGAAGGATCACCTGACCCG
CCACGTGAAGAAGAGCCACTCACAGGAGCTGCTGAAGATCAAGACGGAGC
CTCCTGATATGTTAGGTCTTTTAGCTTCGGGGTCACCNCCTTGCTCTGTGA
AGGAGGAGCTTAGCCCCATGATGTGCGGCATGGGTCCCAACAAAGACCCC
ATGATGGGCAAACCGTTCCCCAGTGGCGCCCCTTTTCCGATGGGCATGTA
CAACCCCCACCAC------CTTCAGGCMATGTCTAATTCTGGGGTGGGTC
ATCCA------CACCCGTCCCTGATGCCCAGCTCGCTGTCTGCAGCTATG
GGCATGGGCTGTCACATGGAATATCTCATCTACGCCTCTTTTTCATTCAT
GGGATGTTTACAAATCAGTGATGGATCAAACATTGTGAACCTGCTGGCTA
GTAACTCTCCAAGTGTTTCGTATGCTGTGACCCAGCAGAAATACTTCAGC
AACTACAGTCCCGTGATTGGGTTCTACATTTACGAGCCCATTGAGTAYTG
GAACTCCACGGTGCAGGAGCACTTGAAGACTCTGAGTCACGGTTTCAACA
AGATCTCCTGGATGGACAACTTTTTCCACTACCTGCGGGTGGTGAATGTG
AGTGCGTCGACCAAAAGCGACTTCATCACCATCCTCAAGGGCTCCTTCCT
GCGCAGCCCGGAGTACCAGCACTTCACTGAGGACATCATATTCTCCAAGA
---ACCGTGACACTG------ACGAATACGACATTATCGCCTCACGCATG
TATTTGGTGGCACGGACCACAGAGAAGAGGCGGGAGGAAGTGGTGGAGCT
TCTAGAGAAGCTTCGCCCGTTGATGCTGATCAACAGCATCAAGTTCATTG

CCTTCAATCCCACGTTTGTGTTCATGGACCGCTACAGCTCCTCCGTCATC
TCGCCCATCCTTACCTCAGGCTTCAGCGTGCTCACCATCCTCATCCTCAC
TTTCTTCCTGGTCATCAACCCCTTGGGGAACTTCTGGCTCATCCTCACGG
TTACGTCCGTGGAGCTGGGCGTCTTGGGTTTGATGGGTTTTCAACAGTTT
GAGTGGCAGCCAGCTCTCAAGAATGTGTCTACATCTTGTAATGTTGGCAT
TATTAATGGGCTCTCTGGATGGAGTTCTTCAGTGGATGACTCCCCAGCTG
ACACCATCACTCGGCGGTTTCGTTATGATGTGGCACTAGTGTCAGCATTA
AAGGATCTGGAGGAGGACATGATGGAGGGGCTGAGAGAGAGTGGGATGGA
AGACAGTGCTTGCACATCAGGCTTCAGTGTTATGATCAAGGAATCGTGTG
ACGGCATGGGCGATGTCAGCGAAAAGCATGGTGGAGGACCAGTTGTTCCT
GAGAAGGCTGTACGTTTCTCTATCACTATTATGTCTGTCTCTGTCCTGGC
AGATGATGAGGAG------------------------------GAGGCGG
TTACTATCTTCACGGAGCCAAAGCCAAACTCAGAACTGTCCTGTAAGCCC
CTTGGCCTGATGTTTGTGGATGAATCAGACCATGAAACACTCACAGCTCT
CCTGGGGCCTATAGTTGCAGAGCGTAATGCAATGAAAGAGAGCAGGCTCA
TCCTTTCCATCGGTGGCCTGCCTCGCTCCTTCCGCTTCCACTTCAGAGGC
ACGGGATACGATGAGAAGATGGTGCGAGAGATGGAGGGCATGGAGGCCTC
AGGGTCCAGCTATGTCTGCACGCTGTGTGATTCCAGTCGGGCAGAGGCAT
CTCAAAACATGGTGCTACACTCCATCACCCGTAACCATGAAGAGAACCTT
GAACGTTACGAAATATGGAGAACCAACCCTTTTTCTGAATCTGGAGAAGA
GCTGCGGGAGAGAGTCAAAGGGGTGTCTGCCAAGCCCTTCATGGAGACTC
AGCCCACGATGGATGCATTACATTGCGACATTGGCAATGCCACTGAGTTC
TACAAAATCTTCCAGGATGAAATTGGGGAGGTGTACCAAAAGGT---CAA
T---CCCAGCCGGGAGGAACGGCGTAGCTGGAGGGCAGCCCTAGATAAAC
AGCTGAGGAAGAAGCTGAAGCTTAAACCGGTAATGAGGATGAATGGGAAC
TATGCCCGCAGGCTAATGACCCAGGAGGCTGTGGAGGTGGTGTGTGAGCT
GGTGCGCTCAGAAGAGAGGAGGGAGGCCCTGAGGGAGCTTATGAGGCTAT
ACCTCCAGATGAAGCCTGTGTGGCGTGCCACCTGCCCAGCCAAGGAATGC
CCTGACCAGTTGTGCCGCTATAGCTTTAACTCCCAGAGCTTTGCCGACCT
TCTCTCGTCTACCTTCAAATATAGGTACAATGGAAAGATAACCAATTACC
TGCACAAGACCCTGGCCCATGTCCCTGAAATCATAGAGAGAGATGGATCG
ATCGGAGCCTGGCCAGCGAGGNNNNNNNNNNNNNNNNNNNNNTCATACACCATCGACATGGGTCCCCTTG
GGCCTCGGTGGAAGGAGAGCCCGCAGCCTTTCTCCTGCTCTGTTGAAGAC
CCCACGAAACAGACAAAGTTCAAAGGCATTAAGACGTACATTTCGTACCG
GGTCACTCCGAGCCACACGGGGCATCCCGTCTACAGGCGCTACAAACACT
TTGACTGGCTGTACAACCGCCTACTGCACAAGTTCACTGTGATCTCCGTG
CCTCACCTACCTGAGAAGCAGGCCACGGGGCGATTTGAGGAAGACTTTAT
TGAGAAGCGKAAGAGACGACTGATACTGTGGATGAACCACATGACCAGTC
ACCCTGTGCTCTCCCAGTACGAAGGCTTTGAACACTTTCTGATGTGTGCC
GACGACAAGCAATGGAAGCTGGGCAAGAGACGAGCTGAGAAGGACGAGAT
GGTGGGCGCCCATTTCATGCTGACCCTCCAGATCCCTAACGAGCACCAGG
ACCTTCAGGATGTAGAGGAAAGAGTCGACACCTTCAAGGCCTTTGCTAAG
AAAATGGACGACAGCGTCATGCAGCTCACACATGTTGCCTCGGAGCTGGT
GCGTAAACACCTGGGTGGATTCAGGAAGGAGTTCCAGCGGCTGGGAAATT
CCTTCCAGTCTATTAGCCAGGCATTCATGCTGGACCCTCCCCATAGCTCA
GACACCCTCAACAACGCCATCTCCCATNNNNNNNNNNNNNNNNNNNNNNNNNNNNNNNTGGGTTTCATCA
TTGGAGTCGGT
GTGGTCGGAAACCTCCTGATCTCCATCCTGCTGGTCAAAGACAAGAGCCT
GCACCGAGCGCCCTACTATTTCCTGCTGGACCTGTGCGCCTCCGATATCC
TTCGCTCTGCCATCTGCTTCCCCTTCGTCTTCACCTCGGTCAAGAATGGA
TCTGCCTGGACCTATGGCACGCTGACCTGCAAAGTGATCGCCTTCTTGGG
TGTGCTCTCCTGTTTCCACACGGCATTCATGCTATTCTGCGTGAGCGTCA
CCCGCTACCTGGCCATCGCACATCACCGTTTCTACACCAAGAGGCTGACC

TTCTGGACCTGTCTGGCCGTCATCTGCATGGTGTGGACGTTGTCAGTGGC
TATGGCGTTCCCGCCCGTGCTAGACGTAGGGACGTACTCTTTTATCCGYG
AGGAGGACCAGTGCACATTCCAGCACCGCTCCTTCAGGGCGAATGATTCG
CTGGGCTTCATGCTCCTGCTGGCGCTCATTCTCCTGGCCACACAGCTGGT
TTACCTCAAGCTCATCTTCTTTGTCCACGACCGTCGAAAGATGAAGCCCG
TCCAGTTCGTGCCTGCTGTCAGCCAGAACTGGACCTTCCACGGGCCAGGC
GCCAGCGGGCAAGCGGCGGCCAACTGGCTGGCCGGATTTGGTAGAGGCCC
CACCCCGCCTACTTTGCTAGGCATMCGGCAGAACAGCAACGCAGCGGGCC
GCAGGCGTCTACTGGTATTGGATGAATTCAAAACAGAGAAGAGGATTAGT
AGGATGTTCTACATCATGACGTTTTTCTTCCTGGCACTGTGGGGGCCCTA
TCTGGTAGCCTGCTACTGGCGGGTGTTTGCGAGGGGCCCTGTAGTCCCCG
GGGGCTATCTGACGGCAGCCGTGTGGATGAGCTTTGCNNNNNNNNNNNNNNNNNNNNNNNNNNNNNNNN
NNNNNNNNNNNNNNGCCAAATCTCGCT
TTCACCCTGGCGTGGGGACTGGTCCTGGCACGGAGC---GCAGCGTCCCA
CTCGGCAACAGCTTGCTATCCCCGCAGCAAACCGAGGAGCCCACTGTTGC
CACCCCCCCGCAGCGATGGTTTGTCACCC---CTGCCAACAACCGACTGG
ACTTTGCTGCCTCGGCATACGACGCCGCT---------GATTTCGCCGGT
AACGCGGCCACCTTGCTGTCCTACGCAGCGGCCGGAGTGAAGGCTC----
--TTCCCCTGCCGACCGCAGGCTGCTCCAACCGGCCTCTTGGCTATTACG
CAGACCCGTCAG---GCTGG---GGAGGACGCACGCCGCCGCAGTACTGT
GGCGTAAATAGCAAACCCAGCTCGGTGTTTTCCTGCTGGCCCGCCAACTC
TATCGGCGGCAGAGCAGGCA---CC---AACTACCTGG---------CCG
AGGA---GGGA---GACTC---CATAACCACGGAGAGGTCACCC---AT-
--C---GGCTCTGAGGAG---ACCAAACCCAAAGACATGAC---ATCAGA
---GTCGAGCTGGATAGAG---ACGCCGTCCTCCATTAAGTCCATTGATT
CAAGCGATTCTGGTATCTTTG---AGCAGGCCAAGAGGAGGAGAATCTCT
CCTTCTGCCACGCCG---------GTTTCAGAGACAGTGTCCCCGTTAAA
ATCTGAGNNNNNNNNNNNNNNNNNNNNNNNNNNNNNNNNNNNNNNNNNNNNNNNNNNNNNNNNNNNNNNNNN
NNNNNNNNNNNNNNNNNNNNNCCACAGCTCCCACGATATTGGCTCCGG---ACAAACGGCGTTT
TCCTCCCAGGCA---CCCGGCTAC---GCAGCAGCCGCCCTGGGA---CA
CCACCA------CCACCCGACCCACGTTGGCTCT---TACTCCACGGCGG
CTTTCAACTCCACCAGGGACTTTCTCTTTAGAAATCGGGGTTTCGGAGAC
GCCGCCGG------------GGCGCAGCACAGTTTGTTCGCCTC------
---CGGAAGTTT---C------GCAGGGCCACATGGACACTCGGATGCAG
CGGGACACCTGCTCTTCCCGGGGCTCCACGAG---CAAGCGGCGAGCCAT
GCGTCTTCCAACGTGGTCAACAGCCAGATGCGTCTGGGCTTCTCGGGGGA
CATGTACGGACGGGCCGACCAGTACGGCCACGTTACAAGCCCGCGGT---
CCGACCACTATGCTTCGACCCAGTTGCATGGCTATGGCCCCATGAACATG
AATATGGCCGCG---CACCACGGTGCAGGGGCCTTCTTTCGATACATGAG
GCAGCCGATCAAACAAGAGCTCATCTGCAAGTGGGTCGAGCCGGAGCAGC
TGACGAATCCCAAAAAGTCGTGCAACAAAACTTTTAGCACGATGCACGAG
TTGGTGACCCATCTGACGGTGGAGCATGTGGGGGGACCAGAGCAGACCAA
CCACATCTGCTTCTGGGAAGAGTGCTCCAGAGAAGGAAAGCCATTCAAAG
CCAAATACAAACTTGTAAATCATATCAGAGTACACACCGGAGAAAAGCCC
TTNNNNNNNNNNNNNNNNNNNNNNNNNNN
>Cyclothone microdon
AGCCTGCTCATCCGAGCCGAGCTTAACCAACCCGGCGCCCTTCTGGGCGA
CGACCAAGTGTACAACGTCATTGTTACTGCTCACGCTTTTGTTATGATCT
TTTTTATGGTCATGCCAATCATGATCGGCGGCTTTGGCAACTGGCTAATT
CCTCTGATGCTCGGCGCCCCCGACATGGCTTTCCCACGGATAAATAACAT
AAGCTTCTGGTTACTCCCCCCTTCCTTCTTTCTCCTGCTAGCCTCGGCTG
GGGTAGAAGCAGGCACAGGCACAGGTTGAACCGTCTACCCCCCTCTCGCT
AGCAACCTAGCTCACGCTGGGGCCTCAGTAGACCTGACTATCTTCTCCCT

CCATTTGGCTGGCGTCTCTTCAATTCTCGGTGCTATCAACTTCATCACCA
CAATCATCAACATGAAGCCCCCTGCCTCAACTCAGTATCAAACCCCCCTC
TTCGTGTGGGCCGTCCTTATTACTGCTGTCCTCCTCCTCCTCTCACTACC
TGTTCTAGCCGCAGGCATTACCATGCTTCTTACTGACCGGAACTTGAACA
CCTCTTTTTTGACCCGGCCGGAGGGGGAGATCCGATCCTATACCAGCAC
CTANNNNNNNNNNNNNNNNNNNNNNNNNNNNNNNNNNNNNNNNNNNNNNNNNNNNNNNNNNNNNNNNNNN
NNNNNNNNNNNNNNNNNNNNNNNNNNNNNNNNNNNNNNNNNNNNNNNNNNNNNNNNNNNNNNNNNNNNNN
NNNNNNNNNNNNNNNNNNNNNNNNNNNNNNNNNNNNNNNNNNNNNNNNNNNNNNNNNNNNNNNNNNNNNN
NNNNNCTGGAGAGGAACCTGCACCCGTCCAACTGCCTGGGCATGCTGCTGCT
CTCCGACGCACACCATTGTACCAAGCTGTTTGAGCTCTCCTGGGGCATGT
GCCTGAGCAACTTCCCTGCCATCTGCAAGACAGAGGACTTTCTCCAGCTG
CCCAAGGACATGGTGGTACAGCTGCTGTCCCACGAGGAGCTGGAGACTGA
GGACGAGCGGCTGGTGTACGAGGCCGCCCTCAACTGGGTCAACTACGACC
TGGAGAGGCGCCACTGCCACCTTGCGGAGCTGCTGCGGACGGTGCGCCTG
GCGCTGCTACCCGCCATCTTCCTGATGGAGAACGTGTCCACAGAGGAGCT
GATCAACGCCCAGATCAAGAGCAAGGAGCTGGTGGATGAGGCCATTCGCT
GCAAGCTGAAGATACTGCAGAACGACGGCGTGGTCAACAGCCCCTGCGCC
CGTCCGCGCAAGACCAGCCATGCTCTCTTTCTGTTGGGCGGCCAGACCTT
CATGTGCGACAAGCTCTACCTGGTGGACCAGAAAGCGAAGGAGATCATCC
CCAAGGCCGACATCCCCAGCCCCAGGAAGGAGTTCAGCGCCTGCGCCATC
GGCTGCAAGGTCTACGTCACCGGTGGAC--GGGGCTC-GGAGAACGGCGT
GTCCAAGGACGTGTGGGTGTACGACACGGTCCAGGAGGAGTGGTCCAAGG
CGGCGCCCATGCTCATCGCCCGTTTTGGCCACGGCTCGGCCGAGCTGAAG
CACTGCCTCTACGTGGTGGGTGGCCACACGGCGGCCACCGGCTGCCTCNNNNNNNNNNNNNNNGG
ACGAGTACATTGTGGTGTTCAGCCGCTCCATCACCAGGCTGATCCTCAAC
GAGGCGGAGTTGATCTTGGCGCTGGCACAGGAGTTCCAGATGCGCGTGGT
GACAGTGTCCCTGGAGGAGCAGAGCTTCTCCAGCATCGTCCAGCTGCTCA
GCGGAGCCGCCATGCTGGTCTCGATGCACGGAGCCCAGCTGGTCACATCC
CTCTTCCTCCCCAGGGGAGCCGCCGTGGTGGAACTCTTCCCTTATGCCGT
CAACCCCGAACAGTACACCCCCTACAAGACCCTGGTCACCCTGCCGGGCA
TGGACCTCCAGTACGCGTCCTGGAGGAACACTGCGAAGGAGAACACCGTG
GCCCACCCGGACAGACCGTGGGACCAGGGAGGCATCGATCACCTCGACCA
GGACGAACAGCAGCGCATCTTAGCCAGCAAGGAGGTCCCCAGGCACCTGT
GCTGCCGAAACCCAGAGTGGCTCTTCCGCATTTACCAGGACACCATA-TG
GACGTCCCCTCCCTGCTGGAGGTGCTGCG---GCAGAGTCTGAAG---AC
GCGGCCCGGCCTGAAGAA---GAGTCGGCCCGCCAGCACCGTCCACCCTG
GAAGGGTCCGGGAGCCACACTGCCAGACCTCCGTCCAGGCTACCAATGAA
GCCAAGCTCACTGTGTCCTGGCAAATCCCCTGGAACCTCAANNNNNNNNNNNNNNNNNNNNNNNNNNNNN
NNNNNNNNNNNNNNAAGAGAGAT
CCCAGCAAGGGGACCTTGGAGGATCAAATCATCCAGGCTAACCCTGCCTT
GGAGGCGTTCGGTAATGCCAAAACAATAAGAAATGACAACTCATCACGCT
TTGGCAAGTTCATTCGGATTCACTTTGGAACCAGTGGCAAGCTGTCCTCT
GCAGACGTAGAAACGTACCTTCTGGAAAAGTCACGTGTCACCTTTCAGCT
CAAGGCAGAGAGGAACTACCACATCTTCTTYCAGATCTTGTCCAATCACA
AGCCAGAACTGTTGGACATGCTTTTAATCACCAACAATCCATACGACTAC
TCCTACATCTCCCAAGGAGAGGTAACAGTAGCGTCCATCAACGATGCTGA
GGAGTTGGTCGCCACTGACAGTGCGTTCGATGTGCTCGGCTTTACTCAGG
AGGAGAAGATGGGGATCTACAAGTTGACGGGTGCGATCATGCATTTCGGC
AACATGAAGTTCAAGCAAAAGCAGCGTGAAGAGCAGGCTGAGCCTGATGG
CACCGAGGCTGCCGACAAGTCAGCTTACCTAATGGGGCTGAACTCTGCCG
ATCTGGTGAAAGGACTCTGCCATCCCAGGGTTAAGGTAGGCAATGAGTAT
GTTACTAAAGGGCAGGGTGTAGACCAAGTCA-----CCCAACAAGGAAGC
CTTCAAGTGTGAGGAGTGTGGGAAGCACTACAACACCAAGCTGGGCTACA

AGCGCCACGTTGCCATGCACTCTGCCACGGCGGGCGACCTCACCTGTAAG
GTGTGCCTGCAGAGCTACGAGAGCACGCCGGCGCTCCTGGAGCACCTGAA
GAGCCACTCGGGGAAGTCGTCGGGCGGCGCCAAGGAGAAGAAGCACCCGT
GCGACCACTGCGACCGCCGCTTCTACACGCGCAAGGACGTCAGACGCCAC
ATGGTGGTGCACACCGGCCGCAAGGACTTCCTGTGCCAGTACTGCGCCCA
GCGCTTCGGCAGGAAGGACCATCTGACACGGCACGTGAAGAAGAGCCACT
CGCAGGAGCTGCTGAAGATCAAGGCGGAGCCTCCGGACATGCTGGGGCTG
CTGGGCTCCGGCTCGCCGCCCTGCCCCGTCAAAGAGGAGCTCAGCCCCAT
GATGTGCAGCATGGGTCCCTCCAAGGACCCCCTGATGGCCAAGCCTTTCC
CCAGCGGAACCCCCTTCCCCATGGGCATGTACAACCCCCACCAC------
CTGCAGGCCATGTCTGGCCCTGGGGGAGGGCAC---------CACCCCTC
TCTAATGCCCGGTTCTCTGTCTGCGGCTATGGGCATGGGCTGCCACATGG
ACTACCTGATCTACGCCTCGTTCTCCTTCATGGGTTGTTTGCAGATCAGC
GACGGCTCCAACGTGGTCAACCTGCTGGCCAGCAACTCCCCCAGCGTCTC
CTACGCGCTGACMCAGCAGAAATACTTYAGCAACTACAGCCCGGTCATCG
GCTTCTACATCTACGAGCCCATCGAGTACTGGAACTCTACGGTGCAGGAG
CACCTGAAGACGCTGGGCCACGGCTTCAACAAGATCTCCTGGATGGACAA
CTATTTCCACTACYTGAAGGTGGTGAACGTGAGCGCCTCCACCAAGAGTA
GCTTCATCGCCATCCTGCGGGGCTCCTTCCTGAGGAGCCCGGAGTACCAG
CACTTCACCGAGGACATCATCTTCTCCAAGA---ACGGCG----------
--AGGAGTACGACATCATCGCCTCGCGGATGTACCTGGTGGCGCGCACCA
CGGAGAAGACCCGCGAGGAGGTGGTGGAACTGCTGGAGCGTCTCCGGCCG
CTCTCGCTCATCAACAGCATCAAGTTCATYGTCTTCAACCVCACCTTCGT
CTTCATGGACCGATACAGCTCCTCGGTCATCTCGCCCATCCTCACCTCAG
GTTTCAGYGTCCTCACCATCCTCATCCTCACCTTCTTCCTAGTCATCAAC
CCCCTGGGGAACTTCTGGCTCATCCTGACTGTCACCTCGGTGGAGCTGGG
CGTGCTGGGCCTGATG----------------------------------

-------------------GTGTCTGGACTCAGAGATCTGGAAGAGGACA
TCATGGAGGGGCTGAGAGAGCGCAGCCTGGAAGACAGCACCTGCACCTTG
GGCTTCAGTGTGATGATCAAAGAGTCCTGCGACGGCATGGGGGACGTCAG
TGAGAAACACGGCGGCGGGCCAGCCATGCCAGAAAAGGCTGTGCGCTTCT
CCTTCACCATCATGTCCATCTCCATCCAGGCTGAGGGGGAGAAT------
------------------------GAGGCCGTCACTGTCTTCAGGGAGTC
CAAGCCCAACTCAGAGCTGTCCTGCAAGCCTTTGGTTCTGATGTTTGTGG
ATGAGTCTGACCATGAGACGCTGACGGGCGTCCTTGGGCCCGTGGTGGCA
GAGAGAAATGCCATGAAGAACAGCCGCCTCATCCTGTCCCTGGGAGGCCT
CGCCAGATCCTTCCGCTTCCTCTTCAGAGGCAGCGGCTATGATGAGAAGA
TGGTACGCCAGTTGGAGGGTCTGGAGGCCTCAGGCTCCACGTACATCTGC
ACCCTCTGCGACTCGACCCGTGCGGAGGCCTCTCACAACATGGTGCTCCA
CTCCATCACCCGCAGCCACGAGGAGAACATGGAGCGCTACGAGATCTGGA
GGACCAACCCCTACTCCGAGTCTGCAGATCAGCTTCGGGATCGGGTGAAA
GGTGTCTCAGCCAAACCCTTCATGGAGACCCAGGCCACTCTGGACGCTCT
GCACTGTGACATTGGTAATGCCACTGAGTTCTACAAGATCTTTCAGGATG
AGATTGGAGAGGTGTACCAGAGGCC---AAAC---CCAAGCACGGAGGAG
CGCAGGAGCTGGCGGGCAGCTCTGGATAAGCAGCTCAGGCACAGGATGAA
GCTGAGGCCGGTGATGAGGATGAACGGGAACTACGCCCGGCGGCTGATGA
CCAGCGAGGCAATAGAGGTGGTGTGTGAGCTGGTCCCCTCGGAGCAGCGG
CGGGAGGCCCTCAGGGAGCTGATGGCACTCTACATTCAAATGAAGCCTGT
GTGGCGCTCCACCTGCCCATCCAAAGAGTGCCCTGACCAGCTCTGTCGCT
ACAGCTTCAACTCCCAGAGGTTTGC-------------------------

-----------------------TCCTACACTATAGAAATGGGCCCCAAA
GGCCCTCAGTGGAAAGAGAGCCCTCAGCCTTTCTCTTGTTCTATTGAGGA
CCCCACCAAACAGACCAAGTTCAAAGGCATCAAGACCTACATATCTTACC
GGGTCACTCCCAGCCACACCGGGCGGCCCGTGTACCGTCGGTACAAGCAC
TTTGACTGGCTCTACAACCGCCTGCTTCACAAGTTCACTGTCATCTCTGT
TCCCCACCTGCCAGAGAAGCAAGCCACAGGACGCTTCGAGGAGGACTTCA
TCGAAAAGCGCAAAAGGCGTCTGGTCCTGTGGATGGACCACATGACCAGT
CACCCAGTCCTCTCCCAGTATGAGGGCTTTGAGCACTTCCTCATGTGCGC
TGATGACAAACAGTGGAAGCTGGGGAAGAGGCGGGCGGAGAAAGATGAGA
TGGTGGGGGCCCACTTCATGCTGACCTTCCAGATCCCCAACGAGCACCAG
GACCTGCAGGATGTGGAGGAGMGAGTCGACTCCTTCAAGGCTTTTGCCAA
GAAAATGGACGACAGTGTCATGCAGCTGACACACGTGACCTCTGAACTGG
TGCGGAAACATCTAGGGGGGTTCAGGAAGGAGTTTCAGCGTCTGGGGAAC
GCTTTCCAGTCCATTAGTCAGGCCTTCACGCTGGACCCTCCTCACTGCTC
TGACGCCCTCAACAACGGCATCTCACAC----------------------

---------------GCCAAATCTCGCTTTCACCATGGCGTAGGGACTGG
TCCTGGCACGGAGC---GCGGCGTCCCACTTAGCAACAGCTTGCTATCCC
CGCAACAACCCGAGGAGCCCCCGGTTG---CCTCCCCGCAGAGATGGTTT
GTCACCCCTGCTGCCAACAACCGACTGGACTTTGCCGCCTCGGCATACGA
TGCTGCCGCTGCTGCAGATCTGGCCGGAAACGCAGCCACTTTGCTGTCCT
ACGCAGCAGCTGGAGTSAAGGCGCTACCGGTTCCCCTGTCCACWGCTGGA
TGCTCCAACAGACCTCTKGGCTATTACGCAGACCCATCGG---GCTGG--
-GGCGCACGCACTCCACCGCAATACTGC---------AGCAAGTCCAGCT
CAGTYCTCTCTTGTTGGCCG---------GTAGGGGGCAGAACAGGCA--
-CC---GGTTACCTGG------------AGGA---CGGG---GACAC---
---------CGATAGATCTCCA---AT---AGGCGCACCGCCGGACGACC
CCAAATCAAAAGACTT------GTCCGA---ATCCAGTTGGATAGAG---
ACGCCGTCTTCAATCAAGTCAATTGATTCGAGTGATTCTGGAATCTTTG-
--AGCAGGCAAAGCGGAGAATAATTTCTCCTTCTGCCACACCA-------
--GTTTCAGAGGCCATGTCACAGTTGAAATCCGAA---------ACGGGC
G------AAGAACGAGAGGTGGCTTTGGGGATCAACCCGTTCGCAGACGG
GATGGGCGCTTTCAAAATCAACCACAGCTCGCACGATCTGGGAACCGG--
-GCAAACAGCGTTTTCCTCCCAGGCG---CCCGGCTACGCAGCCGCTGCA

GCTCTTGGA---CACCACCA------CCACCCGACACATGTCAGCTCC--
-TACTCCACCGCGGCGTTCAACTCCACCCGGGACTTTCTCTTCAGAAATC
GGGGTTTCGGAGACGCCACCAG------------TGCGCAGCACAGTCTG
TTCGCCTCTGC---AGCGGGAAGTTT---T------GCTGGGCCACATGG
ACACTCAGAT------GGACACCTGCTCTTCCCTGGACTCCACGAG---C
AAGCCGCCAGCCACGCGTCCTCTAATGTCGTTAACAGTCAGATGCGGTTG
GGCTTTTCGGGGACATGTACGGTCGGCCGACCAGTATGGCCATGTTAC
CAGCCCGCGAT---CCGATCACTATGCGTCAACCCAGTTGCATGGCTATG
GCCCTATGAACATGAATATGGCCGCA---CACCACGGGGCAGGGGCCTTT
TTCCGTTACATGAGGCAGCCGATCAAACAAGAGCTGATCTGCAAGTGGAT
CGAACCAGAGCAACTCTCCAATCCGAAAAAGTCGTGCAACAAAACTTTCA
GCACGATGCACGAGCTGGTGACCCATCTCACAGTGGAGCATGTGGGGGGA
CCGGAGCAGTCGAACCACATTTGCTTCTGGGAAGAGTGTGCCCGCGAAGG
AAAACCATTCAAAGCCA---------------------------------

>Cyttomimus affinis

------------TTTCTAGAGCGGAACCTTCACCCATCCAATTGTCTAGG
CATGCTGTTGTTGTCTGATGCCCACCAGTGTACCAAGCTGTCAGAGCTCT
CATGGAGTATGTGCCTCAGCAACTTCCCTGCTATTTGCAAGACAGAGGAT
TTTCTCCAACTGCCCAAAGACATGGTTGTGCAGCTTTTGGCCCATGAGGA
GCTGGAGACAGAAGATGAAAGACTGGTTTACGAGGCTTCACTCAATTGGG
TGAACTATGATCTTGAAAGACGGCACTGCCACTTGCCAGAGCTGCTGAAA
ACTGTCCGTTTGGCACTGCTTCCTGCCATCTTCCTAATGGAGAATGTCTC
CACAGAAGAGCTGATAAATACCCAGGCCAAGAGCAAGGAGCTAGTGGATG
AGGCCATTCGCTGCAAACTCAAGATCTTGCAGAACGATGGTGTGGTTAAC
AGTCCATGCGCCCGTCCAAGAAAAACTAGCCATGCACTATTTCTGCTAGG
TGGGCAGACCTTTATGTGTGACAAGCTATACCTGGTGGACCAGAAAGCCA
AAGAGATCATCCCCAAGGCAGACATCCCGAGTCCCAGGAAGGAGTTCAGT
GCCTGTGCCATTGGCTGTAAAGTTTACATAACAGGTGGGA--GAGGCTC-
CGAAAATGGCGTTTCCAAAGATGTATGGGTGTACGATACAGTACATGAGG
AATGGTCCAAGGCAGCCCCCATGCTCATAGCCAGGTTTGGCCATGGCTGT
GCAGAGCTGAAACACTGCCTGTACNNNNNNNNNNNNNNNNNNNNNNNNNNNNNNNNNNNNNNNNNNNNNN
NNNNNGGATGAATACATTGTAGTGTTCAGTC
GCTCCGTGACCAGACTGATCCTGAACGAAGCCGAGCTGATCATGGTGCTG
GCTCAGGAGTTTCAGATGAGAGTTGTTACGGTATCCCTGGAGGACCAATC
TTTCCCCAGCATAGTTCAGGTGATCAGTGGAGCCTCCATGTTGGTCAGCA
TGCACGGGGCTCAGCTTGTTGCCTCTCTTTTCCTGCCCAGAGGAGCTGCC

ATTGTGGAACTGTTCCCCTATGGTGTAAACCCAGAACAGTACACTCCATA
TAAAACCCTGGCTTCCCTGCCAGGTATGGACCTCCACTACTTGTCCTGGA
GGAACACTTTGGAGGAGAACACAGTCACTCACCCAGACAGACCCTGGGAC
CAAGGTGGCATCGTCCACTTGGAGAAGGAAGAGCGAGAGCGAATACTGGC
CAGCAAGGACGTCCCCAGGCACCTGTGCTGCCGGAACCCTGAGTGGCTCT
TCAGGATCTACCAGGACACTTTGGTGGACATCCCTTCACTGCTCGAAGTC
CTCAG---AGAGGGTCTGAAG---ACCAGGCCCAGCTTGAGGAA---GGC
CAAGCCAGCCAGCACAGTTCATCCGGGACGGGTCAGAGAGGCCCAGTGCC
AAACCTCAGTCCAAGCTGCCAATGAGGCCAAGCTCACAGTCTCCTGGCAA
ATCCCCTGGAATCTGAAATATCTCAAGGTGAGGGAAGTGAAGTATGAGGT
G---

---------------------TACCTCATCTACGCTTCCTTCTCCTTCAT
GGGATGTTTACAAATTAGCGACGGATCCAACATCGTGAATTTGCTGGCCA
GCAACTCGCCGAGCGTTTCCTACGCCCTGACCCAGCAGAAGTACTTCAGC
AACTACAGTCCCGTGATCGGGTTCTACATCTACGAGCCCATCGAGTACTG
GAATGCCACGGTGCAGGAGCACCTGAAGACGCTGAGCCACGGCTTCAACA
AGATCTCCTGGATGGACAACTTTTTCCACTACCTGCGGGTGGTGAACGTG
AGCGCCTCCACCAAGAACGACTTCATCAGCATCCTCAAGGGCTCGTTCCT
GCGCAGCCCGGAGTACCAGCACTTCACGGAGGACATCATCTTCTCCAAGA
---ACCGCGACAACG------ACGAGTACGACATCATCGCCTCGCGCATG
TACCTGGTGGCGCGCACCACAGAGAAGAAGCGGGAGGAGGTGGTGGAGCT
CCTGGAGAAGCTGCGGCCGCTGATGCTGATCAACAGCATCAAGTTCATCG
CTTTCAACCCGACGTTCGTCTACATGGACCGTTACAGTTCCTCTGTGATC
TCGCCCATCCTTACCTCAGGCTTCAGCGTCCTCACCATCCTCATCCTCAC
CTTCTTCCTGGTCATCAACCCCTTGGGCAACTTCTGGCTCATCCTGACCG
TCACCTCGGTGGAGTTGGGCGTGCTCGGCTTGATGNNNNNNNNNNNNNNNNNNNNNNNNNNNNNNNNNNN
NNNNNNNNNNNNNNNNNNNNNNNTTGGCATTATTAAT
GGTCTCTCTGGATGGGCTTCCTCAGTGGATGATGCCCCGGCTGACACCAT

CACTCGGCGCTTTCGCTATGATGTGGCATTGGTGTCAGCATTAAAGGATC
TGGAGGAGGACATCCTGGAGGGACTGAGAGAGAAAGGAATGGAAGACAGT
GCTTGCACCTCAGGCTTTACTGTTATGATCAAGGAATGTTGTGATGGCAT
GGGCGATGTCAGTGAGAAGCACGGCGGAGGACCAGCTGTTCCTGAGAAAG
CTGTTCGTTTCTCCTTCACTGTTATGTCTGTCTCCGTCCTGACAGAC---
GAGGTG------------------------------GAGGAGGTTACTAT
CTTCACCGAAACAAGGCCAAACTCAGAACTGTCCTGTAAGCCCCTGTGCT
TGATGTTTGTGGATGAGTCAGACCATGAGACGCTCACAGCTCTGCTGGGG
CCTGTGGTTGCAGAGCGCAATGCAATGAAAGAGAGCAGGCTCATCCTATC
CATAGGTGGCCTCTCTCGCTCCTTCCGCTTCCACTTCAGAGGTACTGGAT
ATGATGAGAAGATGGTGCGAGATATTGAAGGTCTGGAGTCCTCGGGGTCC
ACGTACGTCTGCACCCTTTGCGATTCCAGCCGTGCAGAAGCTTCTGAAAA
CATGGTGCTGCATTCCGTCACCCGCAGTCATGACGAGAACTTAGAGCGTT
ATGAAATATGGCGAACCAACCCCTTTTCTGAGTCTGTGGATGAGCTGCGK
GACAGAGTCAAAGGGGTCTCTGCCAAGGCATTTATGGAGACCCATCCCAC
TCTGGATGCGCTGCACTGTGAGATAGGAATTGCCACTGAATTTTACAAAA
TCTTCCAGGATGAGATTGGGGAGGTGTACCAAAGGCT---AAAT---CCT
AGTCGGGAGGAGCGGCGCGGGTGGAGGGCAGCCCTGGACAAACAGCTGAG
GGAGTCCATGAARCTTAAACCAGTAATGAGGATGAATGGGAACTACGCAC
GCCGGCTCATGAGCCTGGAGGCTGTGGAGTTGGTGTGCCAGTTAGTGCCC
ACAGAAGGTAGGAGGGAAGCCCTGAGGGAGCTCATGAGGCTCTACCTCCA
GATGAAGCCTGTATGGCGTGCCACCTGCCCCGCCAAGGAGTGCCCCGACC
AGCTGTGTCGCTATAGCTTCAACTCTCAGCGCTTTGCAGAGCTCCTCTCC
TCCTCATTCAAATATAGGTACAATAAGAAAATCACCAATTACCTCCACAA
AACCCTCGCCCACGTACCCGAAATCATAGAGAGAGATGGATCAATAGGAG
CATGGGCCAGCGAGGNNNNNNNNNNNNNNNNNNNN-----------------------------------
----

>Cyttopsis rosea
AGCCTTCTTATTCGTGCTGAACTCTGCCAACCAGGAGCATTCCTTGGAGA
TGACCAAATCTACAATGTGCTAGTAACGGCCCATGCTTTTGTAATAATCT
TTTTTATAGTTATACCAGTTATAATTGGGGGCTTTGGGAACTGACTTATT
CCTTTAATGATTGGGGCCCCCGACATAGCCTTCCCCCGAATAAATAATAT
AAGCTTCTGACTTCTCCCCCCCTCATTCCTCCTCCTATTAGCCTCCTCCG
GGGTTGAAGCTGGGGCTGGGACAGGGTGAACCGTTTACCCACCCCTCGCT
GGGAACTTAGCCCATGCAGGGGCCTCCGTAGACTTGACTATCTTTTCCCT
ACACTTAGCAGGGGTATCCTCAATTCTGGGGGCTATCAACTTCATCACAA
CAATTATTAACATGAAACCCCCCGCCATCTCACAATACCAAACCCCTTTA
TTTGTTTGATCCGTTCTAATTACAGCAGTCCTTCTCCTCCTCTCACTTCC
TGTTCTAGCAGCTGGGATCACAATACTCCTAACAGACCGTAACCTAAACA
CCTCTTTCTTTGACCCCGCTGGGGGAGGAGACCCAATTTTATACCAACAC
CTA---

```
--------------------------------------------------
--------------------------------------------------
--------------------------------------------------
------------TTTCTAGAGCGGAACCTTCACCCGTCCAATTGTCTAGG
CATGCTGTTGTTGTCTGATGCCCACCAGTGTACCAAGCTGTCAGAGCTCT
CATGGAGTATGTGCCTCAGCAACTTCCCTGCTATTTGCAAGACAGAGGAT
TTCCTCCAACTGCCCAAAGACATGGTTGTGCAGCTTTTGGCCCATGAGGA
GCTAGAGACAGAAGATGAAAGACTGGTTTATGAGGCTTCACTCAATTGGG
TGAACTATGATCTTGAAAGAAGGCACTGCCACTTGCCAGAGCTGCTGAAA
ACTGTCCGTTTGGCACTGCTTCCTGCCATCTTTCTAATGGAGAATGTCTC
CACAGAAGAGCTGATAAATACCCAGGCCAAGAGCAAGGAGCTAGTGGATG
AGGCCATTCGCTGCAAACTCAAGATCCTGCAGAACGATGGTGTGGTTAAC
AGTCCATGCGCCCGTCCAAGAAAAACTAGCCATGCACTATTTCTGCTAGG
TGGGCAGACCTTTATGTGTGACAAGCTATACCTGGTGGACCAGAAAGCCA
AAGAGATCATCCCCAAGGCAGACATCCCCAGCCCCAGGAAGGAGTTCAGT
GCCTGTGCCATTGGCTGTAAAGTTTACATAACAGGTGGGA--GAGGCTC-
CGAAAATGGCGTTTCCAAAGATGTATGGGTGTACGATACAGTACACGAGG
AATGGTCCAAGGCAGCCCCCATGCTGATAGCCAGGTTTGGCCATGGCTCT
GCAGAGCTGAAACACTGCCTGTACGTGGTCGGAGGTCACACGGCAGCAAC
AGGCTGTCTTCCAGCCTCCCCGTCCGGATGAATACATTGTAGTGTTCAGT
CGCTCCATGACCAGACTGATCCTGAACGAAGCCGAGCTGATCATGGTGCT
GGCTCAGGAGTTTCAGATGAGAGTTGTTACGGTATCCCTGGAGGACCAAT
CTTTCCCCAGCATTGTTCAGGTGATCAGTGGAGCCTCAATGTTGGTCAGC
ATGCACGGGGCTCAGCTTGTTGCCTCGCTTTTCCTGCCCAGAGGAGCTGC
CGTGGTGGAACTGTTCCCCTATGCTGTAAACCCAGAACAGTACACTCCAT
ATAAAACCCTGGCTACCCTGCCAGGTATGGACCTCCACTACGTGTCCTGG
AGGAACACTTTGGAGGACAATACAGTCACTCACCCAGACAGACCCTGGGA
CCAAGGTGGCATCGTCCACTTGGAGAAGGAAGAGCGAAAGCGAATACTGG
CCAGCAAGGACGTCCCCAGGCACCTGTGCTGCCGGAACCCTGAGTGGCTC
TTCAGGATCTACCAGGACACTTTGGTGGACATCCCTTCACTGCTCGAAGT
CCTCAG---AGAGGGTCTGAAG---ACCAGGCCCAGCTTAAGTAA---GG
CCAAGCCAGCCAGCACAGTTCATCCGGGCCGGGTCAGAGAGGCCCAGTGC
CAAACCTCAGTCCAAGCTGCCAATGAGGCCAAGCTCACAGTCTCCTGGCA
AATCCCCTGGAATCTGAAATATCTCAAGGTGAGAGAAGTGAAGTATGAGG
TGTGGATCCAGAAAAAGGATACGAGCAAAGGAACCCTGGAGGATCAAATC
ATCCAAGCCAACCCTGCGCTAGAGGCGTTCGGTAATGCCAAAACGCTGAG
GAATGACAACTCTTCCCGTTTTGGAAAATTCATCCGAATTCACTTTGGAA
CAAGTGGCAAGCTGAGCTCTGCTGACATAGAGACTTACCTGCTGGAAAAG
TCCAGAGTTACCTTCCAGTTGAAGGCTGAGAGGAACTACCACATCTTCTT
CCAGATCTTGTCCAATCAGAAGCCAGAGCTGCTGGACATGCTGTTGATCA
CCAACAACCCGTATGACTATTGCTACATCTCCCAAGGAGAGGTAACAGTA
GCATCCATCAACGACTCCGAGGAGCTGATGGCCACAGACAGTGCCTTCGA
TGTGCTTGGCTTCACTCCTGAGGAGAAGATGGGAGTGTACAAGCTAATCG
GGGCCATAATGCACTATGGCAACATGAGGTTCAAGCAGAAGCAACGTGAG
GAGCAGGCAGAGCCCGATGGAACTGAGGCTGCTGATAAGTCAGCTTATCT
AATGGGGCTGAACTCTGCAGACCTCATCAAAGGCCTTTGCCACCCCAGGG
TCAAGGTGGGAAATGAATATGTCACCAAAGGTCAAGGGGTAGACCAAGTC
TACTACCCCAACAAAGAGGCCTTCAAGTGCGAGGAGTGCGGCAAGCATTA
CAACACCAAGCTGGGCTACAAGCGCCATGTGGCCATGCACTCCGCCACGT
CGGGGGACCTCACCTGCAAGGTGTGCATGCAGAGCTACGAGAGCACCCCG
GTGCTCCTGGAACACCTCAAGAGCCACTCGGGGAAGTCCTCGGGCGGCGC
CAAGGAGAAGAAGCACCCGTGCGACCACTGCGACCGCCGCTTCTACACCC
GCAAAGATGTGAGACGGCACATGGTGGTCCACACGGGCCGCAAGGACTTC
```

CTGTGCCAGTACTGCGCCCAGCGCTTCGGCAGGAAGGACCACCTGACGCG
CCACGTCAAAAAGAGCCACTCGCAGGAGCTAATGAAGATCAAGACGGAGC
CTCCCGATATGCTGGGGCTCCTGGGCTCCGGCTCGCCGCCTTGCTCCGTC
AAGGAGGAGCTAAGCCCCATGATGTGCGGCATGGGTCCCAACAAAGACCC
CATGATGGGCAAGCCCTTCCCCAGCGGCACTCCCTTCCCCATGGGCATGT
ACAACCCTCACCAC------CTCCAGGCCATGTCCAATTCTGGGGTGGGC
CACCCG------CACCCCTCTCTGATGTCCAGCTCGCTGTCTGCGGCCAT
GGGCATGGGCTGTCACATGGAATACCTCATCTACGCTTCCTTCTCCTTCA
TGGGATGTTTACAAATTAGCGATGGATCAAACATCGTGAATTTGCTGGCC
AGCAACTCTCCGAGTGTTTCCTATGCTCTGACCCAGCAGAAGTACTTCAG
CAACTACAGTCCCGTGATCGGGTTCTACATCTATGAGCCCATCGAGTACT
GGAATGCCACGGTGCAGGAGCACCTGAAGACGCTGAGCCACGGCTTCAAC
AAGATCTCCTGGATGGACAACTTTTTCCATTACCTGCGGGTGGTGAATGT
GAGCGCCTCCACCAAGAACGACTTCATCAACATCCTCAAGGGCTCCTTCC
TGCGCAGCCCAGAGTACCAGCACTTCACAGAGGACATCATCTTCTCCAAG
A---ACCGCGAGAGCG------ACGAGTACGACATCATCGCCTCTCGCAT
GTACCTCGTGGCGCGGACCACAGAGAAGAAGCGGGAGGAGGTGGTAGAGC
TTCTGGAGAAGCTGCGACCGCTGATGCTGATCAACAGCATCAAGTTCATC
GCTTTCAACCCGACCTTTGTCTATATGGACCGTTACAGTTCCTCTGTGAT
CTCACCCATCCTTACCTCAGGCTTCAGCGTCCTCACCATCCTCATCCTCA
CTTTCTTCCTGGTCATCAACCCCTTGGGTAACTTCTGGCTCATCCTGACC
GTCACCTCAGTGGAGTTGGGTGTGCTAGGTTTGATGNNNNNNNNNNNNNNNNNNNNNNNNNNNNNNNNNN
NNNNNNNNNNNNNNNNNNNNNNNNNNNNNNNNNNNNNNNNNNNNNNNNNNNNNNNNNNNNNNNNNNNNNN
NNNNNNNNNNNNNNNNNNNNNTCGCCGTTTTCGCT
ATGATGTTGCCCTAGTGTCCGCCTTAAAGGATCTGGAAGAGGACATCGTG
GAGGGGCTGAGGGAAAGGGGGCTCGAAGACAGCGCCTGTACCTCAGGCTT
CACTGTTATGATAAAAGAATCCTGTGACGGCATGGGAGATGTCAGCGAGA
AGCACGGTGGAGGCCCAGCGGTGCCGGAGAAGGCCGTGCGCTTCTCCTTT
ACGATCATGTCTGTGTCTGTGCTGGCAGATGGAGAGGCG-----------
-------------------AAGGCAGTGACCGTCTTCAGAGAGCCGAAAC
CAAACTCCGAGCTGTCCTGTAAGCCCCTGTGTATCATGTTTGTAGACGAG
TCGGACCGCGAGACTCTCACCGCCGTCCTGGGGCCTTTGGTAGCTGAGCG
GGACGCGATGAAGCGGAGCCAACTCATCCTTTCAATCTCTGGTCTTCCTC
GCTCGTTCTGCTTCCAATTCAGAGGCACCGGCTACGATGAGAAGATGGTG
CGCGACCTTGAGGGCCTGGAGGCCTCCGGGTCGACCTATATCTGCACCCT
CTGCGATTCTACTCGAGCAGAGGCCTCTCAAAACATGTCGCTTCACTCCA
TCACCCGCAGCCATGATGAGAACCTAGAGCGCTATGAGATGTGGAGGACC
AATCCATTCTCAGAGTCTGCGGAACAGCTGCGAGACAGAGTGAAAGGGGT
GTCCGCCAAGCCTTTCCTGGAAACCCATCCCACTCTGGATGCGTTACACT
GTGACATTGGCAATGCCACGGAGTTCTACAAAATCTTCCAAGATGAGATT
GGGGAGGTGTTTCAGAAAGA---CAAC---CCGACTCGGGAAGAACGGCG
CCACTGGCGGGCGGTCCTCGATAAGCAGCTGAGGAAGAAGTTAAAGCTTA
AACCGGTAATGAGGATAAATGGGAACTACGCTCGGAAGTTGATGACCCCG
GAGGCTGTGGACGTGGTGTGCGAGCTGGTGCCCTCAGAAGAGCGACGGGA
GGCCCTGAGGGCGCTCATGCAGCTATACCTGGAGATGAAGCCCGTGTGGC
GCACCACCTGTCCGGCCAGAGAATGCCGCGATCAGCTGTGTCGCTTCAGC
TTCAACTCCCAGAGCTTTGCTGAGCTCCTCTCCACTAGCTTCAAATACAG
ATACAACGGAAAGATCACCAATTACTTTCACAAGACCCTCTCCCACGTCC
CGGAAATCATAGAAAGAGATGGCTCTATTGGAGCCTGGGCCAGCGAAGGA
AACGAGTCAGCAAAAAAATCATACACCATTGAGATCGGCCCCAAAGGGCC
AGAATGGAAGGAGAGTCCGCAGCCCTTCTCTTGCTCCATCGAAGACCCCA
CAAAGCAAACCAAGTTCAAGGGCATCAAGACTTACATTTCATACAGGGTG
ACCCCGAGCCACACGGGGCAACCCGTCTACAGACGTTACAAACACTTTGA

CTGGCTGTACAACCGCTTGCTGCACAAGTTCACTGTGATCTCGGTGCCCC
ACCTGCCCGAGAAGCAGGCCACGGGCCGCTTCGAGGAGGACTTCATCGAG
AAGCGTAAGAGACGCCTAATTCTGTGGATGAACCACATGACCAGTCACCC
GGTCCTCTCCCAGTACGAGGGATTTGAACACTTCCTCATGTGTGCCGATG
ACAAGCAGTGGAAGCTGGGAAAGAGGCGGGCGGAGAAGGACGAGATGGTG
GGCGCCCACTTCATGCTGACCTTCCAGATTCCCAGTGAGCACCAGGACCT
CCAGGATGTCGAGGAGCGAGTTGACTCCTTCAAGTCCTTCGCCAAGAAGA
TGGACGACAGCGTCATGCAGCTCACCCACGGTGCTTCGGAGATGGTGCGG
AAGCACCTCGGAGAATTCCGGAAGGAGTTCCAGCGCATGGGAAACGCCTT
CGCATCTCTGAGCCAGGCGTTCATGCTGGACCCTCCCCACAGCTCAAAGG
CCCTCAACAANNNNNNNNNNNNNNNNNN--

>Denticeps clupeoides
AGCCTTCTTATCCGAGCAGAACTCAGTCAACCAGGTGCCCTTCTTGGGGA
TGACCAAATTTACAACGTAATTGTAACAGCCCACGCCTTTGTTATAATTT
TCTTTATAGTAATACCCATTATGATTGGGGGCTTCGGAAATTGACTAGTG
CCCTTAATAATTGGTGCCCCAGACATGGCCTTCCCACGAATAAACAACAT
AAGCTTCTGACTTCTCCCACCCTCTTTTCTACTACTATTAGCTTCTTCCG
GAGTTGAAGCTGGAGCTGGTACAGGGTGAACAGTCTATCCCCCCCTTGCA
GGAAATCTTGCTCACGCAGGTGCTTCTGTAGATCTGACTATTTTTTCACT
ACACTTAGCAGGAATCTCATCAATTCTAGGAGCAATTAACTTTATTACTA
CCATCATTAACATGAAACCGCCAGCTATCTCCCAGTACCAAACACCACTA
TTCGTTTGAGCAGTCCTAATTACGGCAGTTTTACTCCTTCTTTCTCTACC
CGTTTTGGCCGCCGGAATTACAATACTTCTTACAGACCGCAACTTAAATA
CAACTTTCTTCGACCCAGCTGGAGGAGGGGACCCAATTTTATACCAACAC
CTATTCTGATTTTTCGGACACCCAGAAGTTTATATTTTAATTTTACCAGG
TTTCGGCATAATCTCCCACATTGTTGCTTACTACGCAGGAAAAAAAGAAC
CATTTGGTTATATAGGAATAGTATGAGCTATAATGGCAATTGGCCTTCTA
GGATTTATTGTTTGAGCGCATCACATGTTTACTGTGGGTATAGATGTAGA
CACACGCGNNNN--

-------AAGAAAGATTCAAGCAAGGGCACACTGGAGGATCAAATCATCC
AGGCTAACCCAGCTCTGGAGGCATTTGGAAATGCAAAAACGCTCCGAAAT
GACAATTCCTCGCGTTTTGGGAAGTTCATCCGCATTCATTTTGGAACAAG
TGGGAAACTTTCCTCAGCAGACATAGAGACTTATTTGCTTGAGAAATCCA
GAGTGACCTTTCAGCTGAAATCTGAGAGAAACTACCACATCTTTTTCCAA
ATCCTGTCCAATGAAAAGCCAGAGCTTTTAGACATGTTGCTGATCACAAA
CAATCCCTACGACTACTCTTACATCTCTCAAGGAGAAGTCTCCGTTGCAT
CTATCAACGATTCTGAAGAACTTCTTGCCACTGACAGCGCTTTTGACGTG
CTTGGCTTTACGGCAGAAGAAAAAAATGGGAGTGTACAAGCTCACAGGGGC
CATTATGCACTATGGCAACATGAAGTTCAAGCAAAAGCAACGCGAAGAGC
AGGCAGAGCCAGATGGCACAGAGTCAGCGGATAAGTCTGCCTATCTAATG
GGACTGAACTCTGCTGACCTCTTGAAAGGGCTTTGTCACCCCAGGGTGAA
GGTGGGCAATGAGTACGTCACCAAGGGCCAAAGTGTGGATCAGGTTTACT
AC--------AGAGGCCTTCAAATGCGAGGAATGTGGCAAGCACTACAAC
ACCAAGCTGGGGTACAAGCGCCATGTTGCCATGCACTCAGCCACAGCAGG
TGATCTCACCTGCAAGGTCTGCCTCCAGAGCTACGAGAGCACACCTGCTC
TCCTCGAACACCTCAAGAGCCACTCAGGCAAAAGCTCGGGCGGTACCAAG
GAGAAGAAGCACCCATGCGATCACTGCGAGCGTCGCTTCTACACCCGCAA
GGACGTTCGGCGCCACATGGTGGTGCACACAGGACGCAAGGATTTCCTGT
GCCAGTACTGTGCCCAGCGATTTGGTCGCAAGGACCACCTGACTCGGCAC
GTAAAGAAGAGCCACTCACAAGAGCTGCTGAAGATCAAGAACGAACCGCC
CGACATGCTAGGCTTGCTGGGATCTGGGTCGCCAACTTGTGTTGTCAAAG
AGGAGCTAAGCCCAATGATGTGTACCATGGGACCCACCAAGGACCTCATG
ATGGGCAAGGCCTTCCCAACCAGCACCCCGTTCCCTATGGGCATGTACAA
CCCTCACCAC------CTCCAGGCTATGTCCAATCCTGGAGTGGGCCAC-
--------CACCACTCTCTAATGCCTGGTCCACTGTCAGCTGCTATGGGT
ATGGGCTGCCATATGGAGTATCTGATTTACGCCTCGTTCTCCTTCATGGG
ATGCTTACAAATCAGCGACGGGTCCAACGTGGTCAACCTGCTGGCCAGCA
ACTCGCCCAGCGTGTCCTACGCCCTCACCCAGCAGAGGTACTTCAGCAAC
TACAGCCCGGTGATTGGCTTCTACATATACGAGCCCATCGAGTACTGGAA
CGCCACTGTGCAGGAGCACCTCAAGACCCTTGGCCATGGATTTAATAAGA
TCTCCTGGATAGACAATTACTTCCACTACCTGAAGGTGGTSAACATGAGT
GCGTCGAACAAAAGCGATTTCATAAACGTTCTCAAGACGTCCTTTTTGAA
GAGCCCCGAGTATCAGCACTTCAATGATGACATCATCTTCTCCAAGA---
AGGGGG------------AGGAGTATGAGATCATTGCCTCCAGGATGTAT
CTGGTGGCCAGAACGACAGAAAAGACCCGCGAGGAGGTGGTGGAGCTGCT
GGAGCGGCTGCGGCCCCTGTCGCTCATCAACAGCATCAAGTTCATCGTCT
TCAACCCCACCTTCGTCTTCATGGACCGCTACAGCTCCTCCGTCATCTCA
CCCATCCTGACGTCAGGCTTCAGCGTCCTCACCGTCCTCGTCCTCACGTT
CTTCCTGGTCATCAACCCCCTGGGGAACTTCTGGCTGATCCTGACCGTCA
CGTCTGTGGAGCTGGGGGTGCTCGGCCTGATGGGCTACCATCAGTTTGAG
TGGCAGCCTGCTCTGAAGAATGTGTCGAGTGCATGCGATGTAGGCATCAT
CAATGGACTCTCGGGCTGGACCAGCTCAGTAGATGACGTTCCTGCTGACA
CGATTTCACGGCGCTTCCGCTATGATGTGGCCTTGGTCTCAGCGCTGAAA
GACTTGGAAGAGGACATCATGGAGGGCTTGAGAGAGCGAAACCTGGATGA
CAGCACCTGCACCTCAGGCTTCAGTGTTGTCATCAAAGAGTCTTGTGATG
GCATGGGAGACGTTAGCGAGAAACATGGAGGTGGTCCAGCAGTTCCAGAA
AAGGCGGTGCGCTTCTCTTTCACGGTGATGTCCATATCTGTTCAGGCTGA

GGGTGAAGAC------------------------------GAGACAGTTA
CTATCTTCCAGGAACCAAAGCCCAACTCTGAACTCTCCTGCAAGCCAATG
TGCCTGATGTTCGTTGATGAATCTGACCATGAGACCCTTACCGCCATCCT
AGGTCCCGTGGTGGCAGAGCGAAATGCCATGAAGGAGAGCAGACTCATCC
TATCAGTCGGCAACCTTCCACGTTCCTTCCGCTTTCACTTCCGGAGCACG
GGATACGACGAGAAGATGGTACGAGAGCTAGAAGGTCTGGAGGCATCTGG
CTCCACTTACATATGCACCCTCTGCGACTCCACCCGTGCGGAGGCCTCTG
AGAACATGGTGCTTCACTCAATCACCCGAAGTCATGAAGAAAATCTGGAA
CGATATGAAATCTGGCGGACGAATCCATTCTCAGAGTCTGCAGACGAATT
ACGCGAACGGGTCAAGGGCGTCTCAGCCAAACCCTTTATGGAGACCCAAC
CCACTTTGGACGCCCTGCACTGCGACATCGGGAACGCCACAGAGTTCTAC
AAGATCTTTCAGGATGAGATTGGAGAGGTCTACCAAAAGGC---GAAT--
-CCGAGCCGAGAAGAACGTCGTCGCTGGCGAGCTGCCCTGGACAAGCAGC
TGAGGAAGACGATGAAGCTGAAGCCGGTGGCGCGGATGAACGGTAACTAC
GCTCGCCGGCTGATGACCACCGAGGCGGTGGAAGTTGTTTGCGAGCTTGT
GCCTTCAGAGGAGCGCCGTGAAGCCTTGCGTGAGCTCATGGGTCTGTACC
TACAAATGAAACCAGTGTGGCGCTCAACCTGCCCCGCCACTGACTGCCCA
GACCAGCTCCTCCGCTACAGCTTCAACTCCCAGCGCTTTGCTGAGATCCT
GTCCTCCACCTTCAAGTACCGGTATGATGGCAAGATCACGAACTACCTGC
ACAAGACCCTGGCCCACGTACCTGAGATAGTTGAGAGAGACGGCTCTATC
GGAGCCTGGGCCAGCGAAGGGAACGAATCCGGGAACAAGTCCTACACTAT
AGAGATGGGGCCAAAAGGGCCGCAGTGGAAGGAAAACCCACAGCCTTTCA
CCTGCTCCATCGAGGACCCCACCAAGCAGACCAAGTTTAAAGGGATCAAG
ACCTACATCTCCTACCGCGTCACCCCCAGCCACATCGGCCGGCCCGTGTA
TCGCCGCTACAAGCACTTTGATTGGCTGTACAACCGGCTGTTGCACAAAT
TCACGGTCATCTCTGTGCCCCACCTGCCTGAGAAGCAGGCGACGGGCCGT
TTTGAGGAGGACTTCATTGAAAAGCGCAAACGGAGGCTCATCCTCTGGAT
GGAGCACATGACCAGCCACCCGGTGCTGTCCCAGTACGAGGGCTTTGAGC
ACTTCCTCATGTGCGGCGACGACAAGCAGTGGAAGCTGGGAAAGCGGCGG
GCAGAGAAGGACGAGATGGTGGGTGCTCACTTCATGCTCACCTTCCAGAT
CCCCAAAGAGCACCAAGACCTGCAGGACGTTGAGGAGCGGGTGGACACCT
TCAAGGCTTTTGCCAAAAAGATGGACGACAGTGTCCTACAGCTCACTCAT
GTGACTTCAGAACTGGTGCGCAAACATCTCGGAGGCTTCCGGAAGGAGTT
CCAGAGGCTGGGCAACGCTTTCCAGTCCATCAGCCAGGCTTTCATGTTGG
ATCCTCCATACAGCTCTGATGCTCTGAGCAATGCCATCTCCCACCCTCTC
GTCACGTTCCTCAAACTGACCTCCCTGGGATTCATCATTGGCGTCGGCGT
GGTCGGCAACCTCCTGATTTCCATCCTGTTGGTCAAAGACAAAAGCCTGC
ACCGGGCTCCCTACTACTTCCTGCTGGACCTGTGTGCCTCGGATATCTTG
CGCTCTGCAATCTGCTTCCCCTTCGTCTTCACCTCCGTCAAGAACGGCTC
CGCCTGGACGTACGGCACCCTCACCTGCAAGGTGATCGCTTTCCTGGGGG
TCCTTTCCTGCTTCCACACGGCCTTCATGCTCTTCTGCGTCAGCGTCACC
CGCTACCTAGCCATAGCCCACCACCGATTCTACACCAAGAGGCTAACGTT
CTGGACCTGCTTGGCTGTCATTTGCATGGTGTGGACGCTTTCGGTGGCCA
TGGCCTTCCCTCCGGTGCTGGACGTGGGCACGTACTCCTTCATCAGGGAG
GAGGACCAGTGCACCTTCCAGCACCGCTCTTTCCGGGTCAACGACTCGCT
GGGCTTCATGCTCCTGCTGGCCCTCATCCTGCTGGCCACCCAGCTGGTCT
ACCTCAAACTCATCTTCTTCGTTCACGACCGCCGGAAGATGAAGCCCGTC
CAGTTCGTGCCCGCAGTCAGCCAGAACTGGACCTTCCACGGGCCGGGAGC
GAGCGGCCAGGCGGCGGCCAACTGGCTAGCGGGCTTCGGACGAGGCCCGA
CTCCGCCCACCCTGCTGGGCATCAGGCAAAACAGCAACGCGGCGGGGCGG
CGGCGGCTCCTGGTGCTGGACGAGTTTAAGACAGAGAAGCGGATCAGCCG
GATGTTCTACATCATGACCTTCTTCTTTCTGGCTCTGTGGGGGCCGTACC
TGGTGGTGTGCTACTGGAGGGTGTTCGGCCCGTGGCCGGTGGTGCCAGGC

GGGTACCTGACGGCTGCAGTGTGGATGAGCTTCGCCCAGGCTGGGGTCAA
CCCCTTCATCTCATTCTNNNNNNNNNNNNNN---------------------------------
----

>Dibranchus tremendus
AGCCTGCTCATTCGAGCTGAACTAAGCCAACCGGGCGCCCTCTTAGGGGA
TGACCAGATTTACAATGTTATCGTCACGGCTCATGCTTTTGTAATAATTT
TTTTTATAGTAATGCCAATCATGATTGGCGGCTTTGGGAACTGGCTAGTG
CCCCTAATAATCGGCGCCCCCGATATAGCATTCCCGCGAATGAACAACAT
AAGTTTTTGACTGCTGCCCCCATCCTTCCTTCTACTCCTAGCTTCTTCTG
GCATTGAAGCCGGGGCTGGAACCGGATGAACAGTATACCCCCCGCTAGCC
GGGAATCTCGCCCATGCAGGAGCCTCGGTTGATTTAACTATCTTCTCACT
TCACCTTGCAGGGGTGTCATCAATTCTTGGTGCGATCAACTTTATCACCA
CAATTATCAACATAAAACCCCCCGCCACATCTCAGTACCAGACGCCCCTG
TTCGTGTGGTCAGTATTAATTACTGCAGTACTCCTTCTTCTAGCATTACC
AGTCCTTGCTGCTGGCATTACAATGCTACTTACTGACCGAAACCTGAACA
CTACTTTCTTTGATCCTGCTGGGGGAGGCGACCCTATTCTTTACCAACAC
TTA---

-----------AAAAAAGACGCCAACAAGGGGACTCTGGAGGATCAAATC
ATCCAAGCGAACCCTGCGCTAGAGGCCTTCGGGAACGCCAAAACGCTCCG
AAACGACAACTCGTCCCGTTTTGGAAAGTTCATCCGAATTCACTTTGTAA
CCAGCGGAAAGCTGTCGTCCGCTGACATCGAGACGTACCTGCTGGAGAAG
TCCCGAGTCACCTTTCAGCTCAAGGCTGAGAGAAACTACCACATCTTCTA
CCAGATCCTGTCCAACCATAAGCCAGAGCTGCTGGACATGCTGCTGATCA
CCAACAATCCCTACGACTACTGCTACATCTCCCAGGGAGAGGTGACCGTC
GCCTCCATCAACGACTCTGAGGAGCTGATGGCCACAGATAGCGCCTTCGA
CGTGCTCGGCTTCACTCCGGACGAGAAGATGGGCGTCTATAAACTGACCG
GCGCCATCATGCACTACGGCAACATGAAATTCAAGCAGAAGCAGCGCGAG
GAGCAGGCCGAGCCGGACGGGACGGAGTCTGCCGATAAATCCGCTTACCT
AATGGGGCTGAACTCTGCTGACCTCATCAAAGGGCTGTGCCATCCCAGAG
TCAAGGTGGGGAATGAATATGTCACCAAAAGACAAAGTGTGGATCAAGTG
TACTAC---------------TTCAAATGCGATGAGTGTGGGAAGCACTA
CAACACCAAGCTGGGATATAAGCGCCATGTCGCCATGCATTCCGCCACGG
CAGGGGATCTCACCTGTAAGGTGTGCATGCAGACCTACGACAGCACGCCG
GTGCTCCTGGAGCACCTGAAGAGCCACTCAGGGAAGTCCTCGGGTGGCAC
CAAGGAGAAGAAACACCAGTGCGACCACTGTGATCGGCGTTTCTACACAA
GGAAGGACGTGAGACGCCACATGGTGGTCCACACGGGCCGGAAGGACTTC
CTGTGCCAGTACTGTGCCCAGCGCTTCGGCAGGAAGGACCACCTGACGCG
CCACGTGAAGAAGAGCCACTCGCAGGAGCTGCTGAAGATCAAGACGGAGC
CTCCAGACATGTTGGGCCTCCTGGCTTCCGGGTCGCCACCGTGCTCCGTG
AAGGAGGAGCTCAGCCCCATGATGTGTGGTATGGGACCCAACAAAGACCC
CATGATGGGCAAACCCTTTCCCAGCGGGGCCCCTTTTCCAATGGGCATGT

ACAACCCTCACCAT------CTGCAGGCCATGTCTCATTCTGGGGTGGGT
CACTCG------CACCCGTCCATGATGCCCGGCTCCCTGTCTGCAGCTAT
GGGCATGGGCTGTCCCATGGAC----------------------------

-----------------------------------GCCAAATCTCGCTTT
CACCCTGGCATGGGGACCGGTGCTGGCACGGAGC---GCAGCGTCCCACT
CGGCAACAGCTTGTTGTCCCCGCAGCAAAGCGAGGAGCCCACTGTGGCCA
CCCCCCCGCAGCGATGGTTTGTCACCC---CTGCCAACAACCGGCTGGAC
TTCGCCGCCTCGGCATACGACGCCGCC---------GATTTCGCCGGTAA
CGCGRCCACCTTGCTGTCCTACGCGGCGGCCGGAGTGAAGGCTC------
TTCCGCTGCCGGCCGCGGGCTGCTCCAGTCGGCCTCTTGGCTACTACGCA
GACCCGTCGG---GCTGG---GGGGGACGCACGCCGCCGCAGTACTGCGG
CATGAATAGCAARCCCAGCTCGGTCTTCTCCTGCTGGCCCTCCAACTCTA
TCGGGGGCAGAGTGGCCA---CC---GGCTACCTGA---------CTGAG
GA---CGGA---GACTC---GATCCCCACAGAGAGGTCGCCG---GT---
CGGCGCGTCGGAGGAC---GCCAAACCCAAAGAYATCAC---ACCGGA--
-GTCGAACTGGATAGAG---ACCCCCTCCTCCATCAAGTCCATCGACTCG
AGCGATTCTGGGATCTTTG---AACAGGCCAAGAGGAGAAGGATCTCACC
CTCTGCCACACCA---------GTGTCAGAGACAGTGTCCCCCTTAAAGT
CTGAG---


```
-----------------------------------------------------
-----------------------------------------------------
-----------------------------------------------------
-----------------------------------------------------
-----------------------------------------------------
--------------
>Diodon holocanthus
AGCCTCCTAATCCGAGCCGAACTTAGTCAACCTGGGAGCCTCCTTGGAGA
CGACCAAATTTACAATGTCATTGTTACAGCACACGCCTTTGTAATAATTT
TCTTTATAGTAATGCCAATTATGATCGGAGGCTTTGGAAACTGACTGGTG
CCACTAATAATCGGCGCCCCAGACATGGCCTTCCCCCGAATAAATAATAT
GAGCTTTTGACTTCTCCCTCCCTCCTTCCTTCTCCTCCTTGCTTCCTCAG
GCGTAGAAGCCGGTGCCGGCACAGGATGAACAGTATACCCACCACTCGCG
GGCAACCTGGCCCACGCAGGAGCCTCCGTAGACCTGACTATTTTCTCTCT
TCACCTTGCAGGAGTTTCTTCTATTCTAGGAGCAATTAATTTTATTACAA
CAATTATCAACATAAAACCCCCTGCAATCTCCCAATACCAAACCCCCCTT
TTCGTCTGAGCCGTTCTAATCACCGCCGTCCTCCTGCTTCTCTCCCTTCC
AGTCCTTGCTGCAGGAATTACAATGCTCCTCACCGACCGAAACCTCAACA
CCACTTTCTTTGACCCAGCAGGGGGCGGTGATCCTATCCTTTATCAACAC
CTC-----------------------------------------------
--------------------------------------------------
--------------------------------------------------
--------------------------------------------------
------------TTTCTGGAGAGAAACCTTCACCCATCTAACTGCCTAGG
CATGCTGTTGCTGTCCGATGCTCACCAGTGCAGTAAGCTCTCAGAGCTCT
CCTGGGGTATGTGCCTCAGCAACTTTCCTGCTATTTGCAAGACAGAGGAC
TTCCTCCAATTGCCCAAAGATATGGTGGTGCAGCTGTTGTCCCACGAGGA
GCTTGAGACCGAAGATGAGAGGCTGGTTTATGAAGCTGCACTTAACTGGA
TCAACTACGACCTGGAAAAGAGGCACTGCTACCTTCCAGAGCTTCTAAGA
ACAGTTCGTCTTGCCCTTTTGCCCGCCATCTTCCTTATGGAGAAYGTTTC
CACAGAAGAGCTGATCAACACCCAGGCTAAGAGCAAGGAGCTAGTGGATG
AGGCCATCCGCTGTAAGCTGAAGATCCTGCAGAACGATGGTGTTGTTAAC
AGCCCATGTGCCCGACCAAGAAAAACCAGCCACGCCCTCTTTCTTCTTGG
TGGGCAGACTTTCATGTGTGACAAGTTGTACCTGGTGGACCAGAAGGCCA
AAGAAATCATCCCTAAGGCTGACATTCCCAGCCCTAGGAAGGAGTTCAGT
GCCTGTGCCATTGGCTGCAAGGTGTACATCACTGGTGGGA--GAGGTTC-
AGAAAATGGTGTCTCCAAAGATGTGTGGGTGTATGACACTGTCCWCGAAG
AATGGTCGAAGGCAGCACCCATGCTTATTGCCCGGTTTGGCCATGGCTCT
GCAGAGCTGAAGCACTGCCTCTATGTGGTGGGAGGTCATACTGCAGCAAC
CGGTTGCCTTCCAGCCTCTCCGTCTGGATGAGTACATTGTTGTGTTTAGC
CGTTCCACAACAAGACTGATACTGAACGAAGCAGAGCTTATTATGGCTCT
CGTCCAGGAGTTCCAGATGAGAGTGGTGACAGTTTCCTTGGAGGAACAGT
CTTTTTCCAGTATCATCCAGGTAATTAGCAGCGCCGCCATGTTAGTCAGT
ATGCATGGAGCTCAACTTATCACCTCACTCTTCCTCCCAAGAGGAGCCAT
TGTGGTTGAGCTTTTCCCCTTTGCTGTGAACCCAGAGCAGTACACCCCAT
ATAAAACTCTTGCCTCCCTTCCAGGCATGGACCTCCACTATATCTCTTGG
AGGAACACTAAGGAGGAGAACACTGTCACCCACCCAGACAGATCCTGGGA
ACAAGGGGGTATTGCTCATTTGGAAAAGGAGGAGCAAAAGCGAATACTAG
TGAGCCAAGATGTCCCCAGACACCTGTGCTGCCGCAACGCAGAGTGGCTT
TTCCGGATCTACCAGGATACCTTTGTGGATATCCCTTCTTTCCTATATGT
TCTCAA---GGAAGGATTAAAG---ACAAAGCCCACTTTGAAGAA---GT
CAAAGCCAGCCAGCACACTTCATCCGGGCCGGGTCAGAGACCCCCAGTGT
CAGACCTCAGTACAAACCAGTAATGAGGCTAAGCTGACAGTCTCCTGGCA
```

GATCCCATGGAATCTCAAATACCTGAAGGTCAGAGAGGTGAAATACGAGNNNNNNNNNNNNNA
GGAAAGATTCCAGCAAGGGGACCCTGGAGGATCAGATCATCCAGGCGAAC
CCGGCACTAGAAGCCTTCGGCAATGCCAAAACATTGAGAAATGACAACTC
GTCTCGGTTTGGAAAATTCATCCGGATTCACTTTGGTACAAGTGGTAAGC
TGTCATCTGCAGACATAGAGACATATTTGTTGGAGAAGTCCAGGGTGACT
TTTCAGCTGAAAGCAGAGAGAAACTACCACATCTTTTACCAGATCCTGTC
CAATCACAAGCCGGAGCTTTTGGATATGTTACTAATCACCAACAACCCAT
ATGACTACTCCTACATCTCCCAAGGGGAGGTAACGGTCGCTTCCATCAAT
GACTCAGAGGAGCTGATGGCCACAGACAGCGCCTTTGATGTTCTTGGATT
CACCACAGAGGAGAAGATGGGTGTCTACAAATTGACAGGTGCCATCATGC
ACTACGGCAACATGAAGTTCAAGCAGAAGCAACGTGAGGAACAGGCTGAA
CCTGATGGCACAGAGTCTGCTGATAAGTCAGCTTACCTAATGGGGCTGAA
CTCTGCAGACCTCATCAAAGGGCTGTGCCACCCTCGAGTCAAGGTGGGAA
ATGAGTATGTGACCAAAGGCCAAAGTGTAGACCAAGTCTACTACCCCAAC
AAGGAGGCCTTTAAGTGTGAGGAGTGTGGGAAGCACTACAACACCAAACT
GGGATATAAACGCCATGTGGCCATGCATTCTGCCACAGCAGGTGATCTCA
CCTGTAAAGTGTGCATGCAAACCTATGAGAGCACGCCGGTGCTCCTGGAG
CACCTGAAGAGCCACTCTGGGAAGTCTTCAGGTGGCACCAAGGAGAAGAA
ACATCCCTGCGACCACTGTGACCGTCGTTTCTACACACGGAAGGATGTGA
GGCGACACATGGTGGTCCACACGGGCCGGAAAGACTTCCTGTGCCAGTAC
TGTGCCCAGCGCTTTGGCAGGAAGGATCACCTGACTCGCCATGTGAAGAA
AAGCCACTCACAGGAGCTGCTGAAGATTAAGACGGAGCCTCCAGATATGT
TAGGTCTTTTAGCATCGGGGTCACCACCCTGTTCTGTGAAGGAGGAGCTC
AGCCCCATGATGTGTGGTGTGGGCCCTAACAAAGACCCTATGATGGTTAA
ACCTTTCCCCAGTGGAGCCCCATTTCCAATGGGTATGTACAATCCCCACC
AT------CTCCAGGCCATGTCCAACTCTGGGGTGGGTCACCCA------
CACCCGTCCCTCATGCCCAGTCCCTTGTCTGCAGCTATGGGCATGNNNNNNNNNNNNNNNNTATCT
CATTTATGCATCTTTCTCATTCATGGGATGTTTACAAATCAGTGATGGGT
CAAATATTGTAAACCTGTTGGCCAGTAACTCCCCAAGTGTTTCATTTGCT
TTGACCCAGCAAAAATACTTCAGTAACTACAGCCCTGTGATTGGATTTTA
TATTTACGAGCCTATAGAGTACTGGAACTCAACAGTGCAAGAGCACTTGA
AAACTCTGAGTCATGGCTTCAATAAGATCTCCTGGATGGACAACTTTTTC
CACTACCTTAAAGTGGTGAACGTGAGTGCTTCAACCAAGAATGACTTCAT
CACCAGCCTCAAGGGCTCTTTCCTGCGCAGCCCGGAGTACCAGCACTTCA
CTGAGGACATCATATTCTCAAAGA---ACCGTGAGACTG------ATGAA
TACGACATTATTGCTTCACGGATGTACTTAGTGGCACGAACAACAGAGAA
GAAGCGCGAGGAGGTGGTGGAACTGTTGGAAAAGCTTCGTCCATTAATGC
TAATCAACAGCATTAAGTTCATTGCTTTCAACCCGACATTTGTGTTCATG
GACCGCTACAGCTCCTCGGTCATCTCGCCCATCCTGACCTCAGGATTCAG
TGTACTCACAATCCTCATCCTCACCTTCTTCCTGGTCATCAATCCTTTGG
GTAACTTCTGGCTCATCCTCACTGTAACATCAGTGGAACTGGGTGTCTTG
GGTCTGATGGGCTTTCATCAGTTTGAGTGGCAGCCAGCTCTCAAGAATGT
GTCTACATCTTGCAATGTTGGAATTATTAATGGCCTCTCTGGATGGACTT
CCTCAGTAGATGACTTCCCAGCTGACACCATAACACGACGGTTTCGCTAT
GATGTGGCACTAGTGTCAGCATTAAAGGATCTGGAGGAGGATATCATGGA
GGGACTGAAAGAGAATGGGATGGAAGACAGCATTTGTACATCAGGCTTCA
GTGTCATGATCAAAGAATCTTGTGATGGTATGGGTGATGTCAGTGAGAAG
CAYGGTGGAGGACCACCTGTTCCTGAGAAGGCTGTCCGTTTCTCTTTCAC
TGTTATGTCTGTCTCTGTCATGGCAGATGGTGAGACC-------------
-----------------GAAAAGTT---ATCTTCAGTGAGCCAAAGCCA
AATTCAGAGTTGTCCTGTAAGCCTCTTAGTCTGATGTTTGTGGATGAGTC
AGATCATGAAACACTCACAGCTATCATGTCGCCCCTTATTGCAGAACGTA
AYGCAATGAAAGATAGCAGACTCATAGTTTCCATCGGTGGACTGCCTCGC

TCCTTCCGCTTTCACTTCAGAGGCACAGGATACGATGAGAAGATGGTGCG
TGAGATGGAGGGCCTCGAGGCCTCTGGGTCCACCTATATCTGCACGCTTT
GTGATTCCAGTCGCGCAGAGGCTTCTCAAAACATGGTACTACACTCAATC
ACTCGCAATCATGAAGAGAACCTAGAGCGCTATGAAATATGGAGAACCAA
TCCTTTTTCTGAGTCTGCAGATGAGCTGCGAGACAGAGTCAAAGGGGTCT
CTGCAAAGCCCTTTATGGAAACCCATCCCACTCTGGATGCACTACACTGT
GACATCGGCATTGCCACTGAATTTTACAAAATCTTCCAGGATGAGATAGG
GGAAGTGTACCAAAAGGT---CAAC---CCTAGCCGGGAGGAACGGCGCA
GCTGGAGGGCAGCCCTAGATAAACAGCTGAGGAAGAAACTGAAGCTAAAA
CCAATAATGAGGATGAATGGGAACTTTGCACGCCGGCTCATGACCCTAGA
GGCTGTGGAGGCGGTGTGTGAGTTGGTGCCCACAGAGGAGAGGAGGGAGG
CCCTGAGGGAACTTATGAGGCTCTTCCTACAGATGAAGCCTGTGTGGCGA
GCTACCTGCCCAGTCAAAGAGTGCCCTGACCAGCTGTGCCGCTATAGCTT
TAACTCTCAACGCTTCGCAGACCTCCTCTCCTCTTCCTTCAAATATAGGT
ACAATAAAAAGATAAGTAATTACATGCACAAAACTTTGGCCCATGTTCCT
GAGATCATAGAGAGAGATGGATCCATAGGAGCGTGGGCTAGTGAGGGGAA
TGAGTCTGCAAACAAATGTTACACCATTGAGATGGGKCTGTTGGGACCCC
AGTGGAAGGAGAGCCCACAGCCTTTCTCATGCTCCATTGAAGACCCCACA
AAACAGACAAAGTTCAAGGGTATCAAGACKTATATTTCATACCGGGTCAC
TCCAAGCCACACRGCACATCCCGTGTACAGACGCTATAAACACTTTGATT
GGCTATACAACCGCTTACTGCATAAATTTACTGTGATCTCAGTGCCTCAC
TTGCCTGAGAAACAGGCCACGGGGCGATTTGAGGAAGACTTCATTGAGAA
GCGCAAGAGRAGACTGATATTGTGGATGAACCAYATGACCAGTCATCCMG
TCCTCTCCCAGTATGAGGGCTTTGAGCACTTCCTGATGTGTGCTGATGAC
AAGCAGTGGAAACTGGGCAAGAGACGGGCAGAGAAGGATGAGATGGTGGG
TGCCCATTTCATGCTGACCCTCCAGATCCCAAATGAACACCAGGACCTTC
AGGATGTAGAGGAGCGGGTTGATTCCTTCAAGTCCTTTGCCAAGAAAATG
GATGACAGTGTGATGCAACTCACACACGTCACCTCAGAGTTGGTGAGTAA
GCACCTCGGGGGATTCAGGAAGGAGTTCCAGCGGCTGGGAAATGCCTTCC
AGTCTATCAGCCAGGCATTCATGCTGGACCCTCCCYACAGCTCAGAGACC
CTAAACAACNNNNNNNNNNNNNNNNNNNNNNNNNNTCCTCAAACTGACCTCTCTGGGTTTCATCATTGGAG
TTGGT
GTGGTTGGAAACCTCCTGATCTCCATCCTGCTGGTCAAAGATAAGAGCCT
GCACCGAGCACCCTACTATTTCCTGCTGGACCTGTGTGCCTCCGATATAC
TTCGATCCGCCATCTGCTTCCCGTTTGTCTTCACCTCTGTCAAGAATGGA
TCTGCCTGGACGTACGGCACGCTGACCTGCAAGGTGATCGCCTTCCTGGG
TGTGCTCTCCTGTTTTCACACGGCTTTTATGCTGTTCTGTGTCAGTGTCA
CACGCTACCTAGCCATAGCACACCACCGTTTCTACACCAAGAGGCTGACC
TTCTGGACCTGTCTGGCTGTCATCTGCATGGTGTGGACGTTGTCCGTGGC
GATGGCGTTCCCTCCTGTGCTAGATGTAGGGACGTACTCTTTTATCCGAG
AGGAGGACCAGTGCACGTTCCAGCACCGCTCTTTCAGGGCGAACGATTCC
CTGGGCTTCATGCTCCTACTGGCACTCATCCTCCTGGCCACACAGCTGGT
TTACCTCAAGCTCATTTTTTTTGTCCATGACCGTCGGAAGATGAAGCCTG
TCCAGTTTGTGCCTGCCGTTAGCCAGAACTGGACCTTCCACGGACCAGGT
GCCAGCGGGCAAGCGGCTGCCAACTGGCTGGCTGGATTCGGTCGAGGACC
CACCCCGCCTACTCTGCTGGGCATTCGACAGAACAGCAACGCGGCAGGCC
GCAGACGTCTACTGGTTTTGGATGAATTCAAAACAGAGAAGAGGATTAGT
AGGATGTTCTACATCATGACGTTTTTCTTCCTGGCACTGTGGGGGCCCTA
TCTGGTTGCCTGTTACTGGCGGGTATTTGCAAGGGGCCCCGTGGTCCCTG
GGGGCTACCTGACAGCAGCTGTGTGGATGAGCTTTGCCCAGGCTGGGGTC
AATCCTTTCATCTGCNNNNNNNNNNNNNNNNNNNGCCAAATCTCGCTTTCACCCTGGCATGGGGACTGG
TCCTGGCACGGAGC---GCAGCGTCCCACTCGGCAACAGCTTGCTATCCC
CGCAGCAAACCGAGGAGCCCACTCTTGACACCCCCCCGCAGCGATGGTTT

GTCACCC---CTGCCAACAACCGACTGGACTTTGCAGCCTCGGCATACGA
CGCCGCT---------GATTTCGCCGGTAACGCGGCCACCTTGCTGTCCT
ACGCAGCGGCCGGAGTGAAGGCGC------TTCCTCTGCCGACTGCAGGC
TGCTCCAATCGGCCTCTTGGCTATTACGCAGACCCGTCAG---GCTGG--
-GGAGGACGCACGCCGCCGCAATACTGTGGCGTGAACAGCAAATCCAGTT
CGGTCTTTTCTTGCTGGCCCTCCAACTCTATCGGAGCCAGAGCAGGTA--
-CC---AATTACCTGG---------CTGAGGA---GGGG---GACTC---
ACTGCCGACAGAGAGGTCACCG---AT---CGGCGGTTCTGAGGAC---A
CCAAACCTAAAGACATAAC---GTTAGA---GTCGAACTGGATAAAG---
ACGCCGTCCTCCATTAAATCCATTGATTCAAGCGACTCTGGAATCTTTG-
--AACAGGCTAAACGGAGAAGGATGTCACCTTCTGCCACACCA-------
--GTTTCAGAGACAATGTCCCCGTTAAAGTCTGAGCATCACTCAACAGGC
GAAGTCACAGAGAGAGAGAAGTGGCGTTGGGGATAAATCCGTTCGCAGATGG
GATGGGCGCCTTCAAAATAAACCACAGCTCCCATGATATTGGCTCCGG--
-ACAAACAGCGTTTTCCTCTCAGGCG---CCCAGCTACGCAGCGGCAGCA
GCCCTGGGA---CACCACCA------CCACCCGACCCACGTAGGCTCT--
-TACTCCACGGCAGCTTTCAACTCCACCAGGGACTTTCTCTTCAGAAATC
GGGGATTTGGTGACGCCACCGG------------TGCACAGCACAGTTTG
TTCGCGTC---------CGGAAGTTT---C------GCAGGGCCACATGG
ACACTCAGATGCAGCGGGGCACCTGCTGTTCCCGGGGCTTCACGAG---C
AAGCGGCGAGCCACGCGTCTTCCAATGTGGTTAACAGCCAGATGCGACTT
GGCTTCTCGGGGGACATGTATGGAAGGGCCGACCAGTATGGCCACGTTAC
GAGCCCACGGT---CCGACCATTACGCGTCGACCCAGCTCCACGGCTACG
GCCCCATGAACATGAATATGGCCGCG---CACCACGGAGCAGGGGCCTTC
TTTCGATACATGAGGCAGCCGATCAAACAAGAGCTCATCTGCAAATGGAT
CGAGCCGGAGCAGCTGACAAATCCCAAAAAGTCGTGCAACAAAACTTTTA
GCACGATGCACGAGCTCGTCACCCATATCACCGTGGAGCATGTTGGGGGA
CCTGAGCAGACCAACCACATCTGCTTTTGGGAGGACTGCGCCAGAGAAGG
AAAGCCATTCAAAGCCAAATACAAACTTGTGAATCATATCAGAGTACACA
CCGGCGAAAAGCCCTTTCCGTGTCCGTTCCCGGGCTGTGGCAAA
>Diplophos taenia
AGCCTACTTATCCGGGCGGAGCTTAGCCAGCCTGGCGCGCTTCTAGGCGA
TGATCAAATTTATAATGTAATCGTTACGGCGCACGCCTTCGTAATAATCT
TCTTTATAGTAATACCAATTATGATCGGGGGATTTGGAAACTGATTAATT
CCTCTTATGATTGGGGCCCCGGACATGGCCTTTCCTCGAATGAACAACAT
AAGCTTCTGGCTCCTCCCGCCCTCGTTCCTTCTTCTCCTGGCTTCCTCCG
GGGTGGAAGCTGGGGCCGGGACGGGGTGAACCGTGTACCCCCCTCTCGCC
GGCAATCTTGCCCATGCAGGAGCCTCCGTTGACCTGACCATCTTCTCCCT
CCACCTCGCAGGGATCTCCTCTATTCTTGGGGCAATCAACTTCATCACTA
CAATCATTAACATAAAACCCCCTGCCATCTCCCAGTATCAAACCCCTCTA
TTCGTGTGAGCAGTCCTAGTCACTGCCGTTCTCCTTCTATTATCCCTCCC
TGTTCTGGCTGCGGGGATTACAATACTTCTCACGGACCGGAACCTGAACA
CCACCTTCTTTGACCCGGCAGGAGGGGGGGACCCAATCCTTTACCAGCAC
CTCTTTTGATTCTTCGGGCACCCCGAAGTGTACATTCTAATCCTCCCGGG
CTTTGGTATGATCTCCCACATTGTTGCCTACTACGCAGGGAAAAAAGAGC
CCTTCGGGTATATAGGGATGGTGTGAGCCATGATGGCCATCGGACTTCTA
GGCTTCATCGTCTGGGCCCACCACATGTTCACGGTCGGAATGGACGTTGA
CACCCGAGCATATTCCTGGAGAGGAACCTGCACCCGTCCAACTGCCTGGG
CATGCTGCTGCTGTCCGACGCCCACCAGTGCACCAAGCTGTCCGAGCTGT
CCTGGGGCATGTGCCTCGGCAACTTCCCCGCCATCTGCAAGACGGAGGAC
TTCCTCCAGCTCCCCAAGGACATGGTGGTGCAGCTGCTGTCCCACGAGGA
GCTGGAGACGGAGGACGAGAGGCTGGTGTACGAGGCCGCCCTCAACTGGG
TCAACTACGACCTGGAGAGGCGCCACTGCCACCTGGCCGAGCTGCTGCGG

ACGGTGCGCCTGGCGCTGCTGCCCGCCATCTTCCTCATGGAGAACGTGTC
CACGGAGGAGCTGATCAACGCCCAGGCCAAGAGCAAGGAGCTGGTGGACG
AGGCCATCCGCTGCAAGCTGAAGATCCTGCAGAACGACGGCGTGGTCAAC
AGCCCCTGCGCCCGCCCCCGCAAGACCAGCCACGCCCTGTTCCTGCTGGG
CGGACAGACCTTCATGTGCGACAAACTGTACCTGGTGGACCAGAAGGCCA
AAGAGATCATCCCCAAGGCGGACATCCCCAGCCCCAGGAAGGAGTTCAGC
GCCTGCGCCATCGGCTGCAAGGTCTACGTCACCGGCGGCA--GGGGCTC-
GGAGAACGGCGTGTCCAAAGACGTGTGGGTGTACGACACCGTCCAGGAGG
AGTGGTCCAAGGCGGCGCCCATGCTCATCGCCCGGTTCGGCCACGGCTCG
GCCGAGCTGAAACACTGCCTCTACGTGGTGGGAGGGCACACGGCGGCCAC
GGGCTGCCTCCCGGCCTCGCCCTCGGGACGAGTACATAGTGGTGTTCAGT
CGCTCCTCTACGAGGCTGATTCTGAACGAGCCAGAGCTAATCATGGTGTT
GGCGCAGGAGTTTCAGATGAGGGTGGTCACGGTGTCTCTGGAAGAGCAAT
CCTTCCCCAGCATCATCCAGGCGATCAGCGGCGCCTCCATGCTGGTCAGT
ATGCACGGAGCTCAGCTTGTTACCTCCCTCTTCCTCCCCAGAGGGGCTGC
CATTGTGGAGCTCTTCCCCTATGCTGTGAACCCAGAGCAGTATACTCCAT
ACAAAACCCTGGCGTCCCTCCCAGGCATGGATCTTCAATATGTTTCCTGG
AGAAACACAATCGAAGAGAACTCAGTTAGCCACCTCGATAGGTCCTGGGA
CCAAGGAGGCATTGCTCACCTGGACAAGGAAGAGCAGGAGAGAATTCTCG
CCAGCAAGGAGGTCCCCAGGCACCTGTGCTGCCGCAACCCCGAGTGGCTC
TTCCGAATCTACCAGGACACTCTGGTGGACATCCCCTCATTCCTCAAGAC
CCTCAA---GGAGGGCCTAAAG---ACCAGGCCCAGCTTGAAGAA---GT
CCAAGTCGGCCAGCACTGTTCACCCGGGCCGGGTCAGGGAAGCCCAGTGC
CAGACCTCGGTCCAGGACACCAACGAGGCTAAACTCACTGTCTCCTGGCA
GATCCCGTGGAATCTCAAGTACCTGAATGTACGAGAGGTTAAGTACGAGG
TCTGGA-----AAAAGGGATTCCAGCAAGGGAACCTTGGAGGATCAAATC
ATCCAGGCTAATCCTGCCCTGGAGGCTTTCGGTAATGCTAAAACATTGAG
AAATGACAACTCATCACGCTTTGGCAAATTCATCCGGATTCACTTTGGAA
CTASTGGCAAGTTGTCCTCTGCAGACATAGAGACATACCTTCTGGAAAAG
TCACGCGTCACCTTTCAGCTCAAGTCAGAGAGGAACTACCATATCTTCTT
CCAGATCTTGTCCAATCAAAAGCCAGAGCTGTTGGACATGCTTTTGATCA
CCAACAATCCATATGACTACTCCTACATCTCCCAAGGAGAAGTAACAGTA
GCATCCATCAATGATTCTGAGGAGCTGATGGCCACTGACAGTGCATTTGA
TGTGCTTGGCTTTACTCAAGAGGAGAAAATGGGGGTSTACAAGTTGACAG
GTGCAATCATGCATTACGGCAACATGAGGTTCAAGCAAAAGCAGCGTGAG
GAGCAGGCAGAACCTGACGGCACTGAGGCTGCTGACAAGTCAGCTTACCT
AATGGGGCTGAACTCTGCAGATCTAGTGAAAGGACTCTGCCATCCCAGGG
TTAAGGTTGGCAATGAGTATGTCACWAAAGGGCAGGGTGTTGACCAAGTC
TATTACCCCAACAAGGAGGCCTTCAAGTGCGAGGAGTGCGGCAAGCACTA
CAACACCAAGCTGGGCTACAAGCGCCATGTGGCCATGCACTCGGCCACGG
CCGGCGACCTCACCTGCAAGGTGTGCCTGCAGAGCTACGAGAGCACGCCG
GCCCTGCTGGAGCACCTGAAGAGCCACTCCGGCAAGTCGTCGGGCGGCGC
CAAGGAGAAGAAGCACCCGTGCGACCACTGCGACCGGCGCTTCTACACGC
GCAAGGACGTCCGGCGCCACATGGTGGTGCACACGGGCCGCAAGGACTTC
CTGTGCCAGTACTGCGCCCAGCGCTTCGGCAGGAAGGACCACCTGACGCG
CCACGTGAAGAAGAGCCACTCGCAGGAGCTGCTGAAGATCAAGGCGGAGC
CTCCGGACATGCTCGGCCTGCTGGGTACCGGCTCCCCGCCCTGCTCCGTC
AAAGAGGAGCTCAGCCCCATGATGTGCAGCATGGGTCCCTCCAAGGACCC
TCTGATGGCTAAGCCCTTCCCCGGCGGGACCCCCTTCCCCTTAGGCATGT
ACAACCCTCAC---------CTGCAGGCCATGTCCGGCCCCGGGGGGGCC
CAC---------CACCCCTCCCTGATGCCCGGCTCCCTGTCCGCGGCCAT
GGGCATGGGCTGCCACATGGACTACCTGATCTACGCCTCCTTCTCCTTCA
TGGGATGTTTACAAATCAGCGACGGGTCCAACGTGGTCAACCTGTTGGCC

AGCAACTCGCCCAGCGTGTCGTACGCGCTGACCCAGCAGAAGTACTTCAG
CAACTACAGCCCGGTGATCGGCTTCTACATCTACGAGCCCATCGAGTACT
GGAACTCCACGGTGCAGGAGCACCTGAGGACGCTGGGCCAAGGCTTCAAC
AAGATCTCCTGGATGGACAACTACTTCCACTACCTGAAGGTGGTGAACGT
GACCGCCGCCACCAAGAGCGACTTCATCGCCGTGCTCAAGGGCTCCTTCC
TGAAGAGCCCGGAGTACCAGCACTTCACCGAGGACATCATCTTCTCCAAG
A---AGGGCG------------AGGAGTACGACATCATCGCGTCGCGGAT
GTACCTGGTGGCGCGCACCACCGAGAAGACCCGCGAGGAGGTGGTGGAGC
TGCTGGAGAGGCTGCGGCCCCTGGCGCTCATCAACAGCATCAAGTTCATC
GTATTCAACCCCACCTTCGTCTTCATGGACCGCTACAGCTCCTCGGTGGT
GTCGCCCATCCTCACCTCCGCCTTCAGCGTGCTCACCATCCTCATCCTCA
CCTTCTTCCTGGTCATCAACCCGCTGGGGAACTTCTGGTTGATCCTGACC
GTCACCTCGGTGGAGCTGGGCGTCCTGGGTCTGATGGGCTACCACCCCTT
TGAGTGGCAGCCGCCCCTCAAGAACATGTCCACATCCTGCCAGGTGGGCA
TTATCGATGGGCTGTCAGGGTGGGCTGCCTCCGTAGATGACTGCCCAGTG
GACACTGTCACCCGGCGGTTCCGCTATGACGTGGCGCTGGTATCAGCTCT
GAAGGACCTAGAGGAGGACATCATGGAGGGGCTGATGGAGTGCGGCCTGG
AGGACAGCATCTGCACCTTGGGCTTCAGCGTGATGATCAAGGAGTCCTGC
GATGGCATGGGGGACGTCAGCGAGAAGCATGGTGGGGGGCCGATGGTGCC
TGAAAAGGCTGTGCGATTCTCCTTCACTGTCATGGCTGTCTCTGTCCAGG
CAGAGGGAGAGGAT------------------------------GAGGCC
GTCACCGTCTTCAGAGAGTCCAAGCCCAACTCTGAGCTGTCCTGCAAGCC
TCTGTGTCTGATGTTTGTGGATGAGTCTGATCACGAGACACTGACAGGCA
TCCTTGGGCCTGTGGTGGCTGAGAGGAACGCTATGAAGAACAGCCGCCTC
ATACTGTCTCTCGGGGGCCTCCCTCGATCCTTTTGCTTCCACTTCAGGGG
CACAGGCTATGATGAGAAGATGGTGCGTGAGATGGAGGGCCTGGAGGCCT
CAGGCTCCACATATGTCTGCACCCTCTGTGACTCCACCCGTGCAGAGGCC
TCCCACAACATGGTGCTTCACTCCATTACCCGCAGCCACGATGAGAACAT
GGAGCGCTACGAGATCTGGAGGACTAACCCGTACTCCGAGTCCGCAGATG
AGCTTCGGGATCGGGTGAAAGGTGTCTCTGCCAAACCCTTCATGGAGACT
CAGCCCACCCTAGACGCTCTGCACTGTGATATCGGTAACGCCACCGAGTT
CTACAAGATCTTTCAGGATGAGATCGGGGAGGTGTACCGGAAGCC---CA
AC---CCAAGCAGGGAGGAGCGCCGGAGCTGGCGGGCTGCTCTGGACAAG
CAGCTGAGGAGGAAGATGAAGCTGAGGCCAGTGATGAGGATGAATGGGAA
CTATGCCCGGAGGCTGATGACCAGCGAGGCAGTGGAGAGTGTGTGTGAGC
TAGTCCCCTCAGAACAGCGGCGTGAGGCTCTCAGGGAGCTGATGGGACTC
TATCTCCAAATGAAGCCTGTGTGGCGCTCCACCTGCCCGGCCAAAGAGTG
CCCTGACCAGCTGTGCCGCTACAGCTTCAACTCCCAGAGGTTTGGTGAGC
TCCTCTCCACCACCTTCAAGTACAGATATGACGGTAAGATCACCAACTAC
CTTCACAAGACACTGGCCCACGTGCCGGAGATCATAGAGAGGGACGGCTC
TATCTGGGCCTGGGCCAGCGAGGGAAATGAGTCAGGCAATAAGTCTTACA
CAATCGAAATGGGCCCCAAAGGGCCTCAGTGGAAGGAGAGCCCTCAGCCT
TTCTCTTGTTCTGTTGAGGACCCTACCAAACAGACCAAATTTAAAGGCAT
CAAGACCTACATATCTTACCGAGTCACGCCCAGCCACACAGGGCGACCCG
TATACCGTCGGTACAAGCACTTTGATTGGCTATACAACCGCCTGCTGCAT
AAGTTCACTGTCATCTCTGTGCCTCACCTGCCAGAGAAGCAGGCCACAGG
GCGTTTCGAGGAGGACTTCATCGAAAAGCGCAAAAGGCGACTGATCCTCT
GGATGGACCACATGACCAGTCACCCTGTCCTCTCCCAATATGAGGGCTTC
GAGCACTTCCTCATGTGCGCTGATGACAAACAGTGGAAGCTGGGCAAGCG
GCGGGCGGAGAAGGATGAGATGGTGGGTGCCCACTTCATGCTGACCTTTC
AGATTCCCAATGAGCACCAGGACCTGCAGGACGTGGAGGAGCGAGTGGAC
TCCTTCAAGTCTTTTGCCAAGAAAATGGATGATAGTGTCATGCAGCTGAC
GCATGTGGCCTCAGAACTGGTGCGGAAACATCTAGGGGGTTTCAGGAAGG

```
AGTTCCAACGTCTGGGGAATGCTTTCCAGTCTATAAGTCAGGCATTTATG
CTGGACCCTCCTCACAGCTCTGATGCCCTCAACAATGCCTTCTCGCAC--
-----------------AACTGACCTCTCTGGGTTTCATAATTGGAGTCG
GCGTGGTAGGAAACCTGCTGATCTCTATCCTGCTGGTCAAAGACAAAAGC
CTGCACCGCRCNCCCTACTACTTCCTGCTGGACCTGTGCGCCTCCGACATC
CTGCGCTCCGCCATCTGCTACCCCTTCGTCTTCACCTCTGTCAAGAATGG
ATCCGCCTGGACCTACGGCACGCTCACCTGCAAAGTGATTGCCTTCCTTG
GCGTACTCTCCTGTTTCCACACGGCATTTATGCTGTTCTGCGTCAGCGTC
ACCCGCTACCTGGCCATCGCCCACCACCGTTTCTACACCAAGAGGCTGAC
TTTCTGGACCTGCCTGGCCGTCATCTGCATGGTGTGGACGTTATCCGTGG
CCATGGCCTTCCCCCCAGTTCTCGATGTAGGGACTTATTCCTTTATCCGG
GAGGAGGACCAGTGTACATTCCAGCACCGCTCCTTCAGGGCTAACGACTC
CCTGGGCTTTATGCTCTTGCTGGCACTTATCCTGCTGGCTACGCAGCTGG
TTTACCTCAAGCTCATCTTCTTCGTCCATGACCGCAGGAAGATGAAGCCT
GTCCAGTTTGTGCCAGCTGTCAGCCAGAACTGGACCTTCCATGGGCCTGG
TGCCAGCGGGCAGGCAGCGGCCAACTGGCTGGCCGGATTCGGGAGAGGCC
CCACCCCGCCTACCTTGCTGGGCATCCGTCAGAACAGCAACGCGGCGGGC
CGCAGGCGTCTGCTGGTGCTGGATGAGTTTAAAACAGAGAAGAGGATAAG
TAGGATGTTCTACATCATGACGTTTTTTTTCCTGGTGCTGTGGGGGCCCT
ATCTAGTGGCCTGCTACTGGCGGGTGTTCGCCA-----------------
--------------------------------------------------
--------------------------------------------------
--------------------------------------------------
--------------------------------------------------
--------------------------------------------------
--------------------------------------------------
--------------------------------------------------
--------------------------------------------------
--------------------------------------------------
--------------------------------------------------
--------------------------------------------------
--------------------------------------------------
--------------------------------------------------
--------------------------------------------------
--------------------------------------------------
--------------------------------------------------
-------------CAGGCGNAAGTCACAGAANGAGAGGTGGCTTTGGGGATA
AATCCGTTCGCAGACGGGATGGGCGCTTTCAAAATCAACCACAGCTCGCA
TGATCTTGGCTCCGG---GCAAACAGCGTTTTCCTCTCAAGCA---CCCG
GCTAC---GCAGCCGCTGCCCTCGGA---CACCACCA------CCATCCA
ACACATGTCAGCTCT---TACTCCACCGCGGCTTTCAACTCCACTCGGGA
CTTTCTCTTCAGAAATCGGGGCTTCGGAGACGCCACCAG-----------
-TGCGCAACACAGTCTCTTCGCATCTGC---AGCGGGAAGTTT---T---
---GCAGGGCCACATGGACACTCAGATGCCGCTGGACACCTGCTCTTCCC
AGGACTTCACGAG---CAAGCTGCGAGCCATGCGTCCTCAAATGTTGTTA
ACAGTCAGATGCGGTTGGGCTTTTCAGGGGACATGTACGGGCGGGCCGAC
CAGTATGGCCACGTTACTAGCCCGCGGT---CCGACCACTATGCTTCGAC
CCAGTTGCATGGCTACGGCCCTATGAACATGAATATGGCCGCA---CATC
ATGGAGCAGGGGCCTTCTTCCGTTACATGAGGCAGCCGATAAAACAAGAG
CTGATCTGCAAGTGGATCGAACCAGAGCAACTTACGAATCCGAAAAAGTC
GTGCAACAAAACTTTTAGCACGATGCACGAGCTGGTGACCCATTTGACGG
TGGAGCATGTGGGGGGACCAGAGCAGTCGAACCATATTTGCTTCTGGGAA
GAGTGTGCCCGAGAAGGAAAACCATTCAAAGCCAAATACAAACTTGTGAA
```

CCACATCAGAGTGCACACCGGGGAGAAACCGTTTCCATGTCCTTTCCCNGG
CTTATGCTCG
>Diretmichthys parini

NNNNNNNNNNNNNNNNNNNNNNNNNNTAATTGCCTGGGCATGCNGTTGCTGTCTGATGCACACCA
GTGTACCAAGCTATCAGAGCTCTCCTGGGGCATGTGCNTCAGCAACTTTCC
TGCTATTTGCAAGACAGAGGACTTCCTCCAACTGCCCAAAGACATGGTGG
TGCAGCTTCTATCCCACGAGGAGCTGGAGACAGAAGATGAGAGANTGGTTT
ATGAAGCTGCCCTTAACTGGGTCAACTATGACCTGGAAAGGAGGCACTGC
CATCTGCCAGAGCTGCTGAGAACGGTTCGCCTGGCCCTGCTACCTGCCAT
CTTCCTCATGGAGAATGTCTCGACAGAAGAGCTGATCAATGCCCAAGCCA
AGAGCAAGGAGCTGGTGGATGAGGCCATACGCTGCAAGCTGAAGATCTTG
CAGAATGACGGTGTGGTTAACAGCCCCTGTGCTCGGCCGAGAAAAACCAG
CCATGCCCTTTTTCTGCTGGGAGGGCAGACCTTCATGTGTGACAAGCTGT
ACCTGGTGGACCAGAAGGCGAAAGAGATCATCCCCAAGGCTGACATCCCC
AGCCCTAGGAAGGAGTTCAGTGCCTGTGCCATTGGCTGTAAGGTCTACAT
CACAGGTGGCA--GAGGCTC-AGAAAATGGTGTGTCTAAAGANGTATGGGT
CTATGACACCGTCCAAGAAGAATGGTCCAAGGCAGCACCCATGCTCATCG
CCAGATTTGGTCATGGCTCTGCTGANCTGAAACACTGCCTTTACGTAGTANG
ANGTCACACAGCAGCAACTGGCTGCCTCCCAGCCTCTCCCTCCGGATCACT
ACATTGTAGTGTTCAGCCGTTCAACAACGAGGCTGATTCTGAATGAAGCG
GAGCTAATCATGACGCTGGCCCAGGAATTTCAGATGAGAGTGGTCACGGT
GTCCTTGGAGGAACAAAAGTTCCCCAGCATCGCCCAGGTGATCAGCGGGG
CCTCCATGCTAGTCAGTATGCATGGAGCTCAGCTCGTCACCTCACTCTTC
CTCCCCAGAGGAGCTGCTGTGGTGGARCTGTTCCCCTACGCTGTTAACCC
AGAACAGTACACCCCATATAAAACCCTCGCCACCCTACCAGGCATGGACC
TTCAGTATGTTTCCTGGAGGAACACTATAGAGGAGAACACTGTCACCCAC
CCAGACAGACACTGGGAACAAGGAGGCATCGCCCATTTGGAAAAGGACGA
GCAAGAACGAATATTAGCCAGCAAGGATGTCCCCAGACACCTGTGCTGCC
GCAACCCGGAGTGGCTATTCCGAATCTACCAGGACACTTTGGTGGACATC
CCTTCATTCCTGGAGGCCCTCAA---AGAGGGCCTGAAG---ACCAGGCC
CAGCTTGAAGAA---GGCCAAGCTGGCCAGCACGGTTCATCCGGGCCGGG
TCAGAGAACCCCAGTGCCACACCTCAGTCCAAGCCACCAACGAGGCTAAA
CTCACAGTTTCCTGGCAGATCCCGTGGAATCTGAAATACCTGAAGGTGAG
AGAAGTGAAGTACGAGGTG---------AAGAAGGATACTAGCAAGGGAA
CACTGGAGGATCAAATCATCCAGGCAAACCCTGCGCTGGAGGCTTTCGGT
AATGCCAAAACAATGAGGAATGACAACTCATCCCGCTTTGGAAAATTCAT

CCGAATTCATTTTGGAACCAGTGGCAAGTTATCCTCTGCTGACGTTGAAA
CGTACCTATTGGAGAAGTCACGTGTCACCTTTCAGCTCAAGGGTGAGAGA
AACTATCACATTTTCTTCCAGATATTGTCCAATCAGAARCCAGAGCTATT
AGACATGATGTTGATCACTAACAACCCATATGACTACTCCTACATCTCCC
AAGGAGAGGTAACAGTAGCATCCATCAATGACTCAGAGGAGCTGCTGGCC
ACTGACAGCGCCTTTGATGTGCTTGGCTTCACTCCAGAGGAGAAGATGGG
ACTATATAAGCTAATTGGTGCCATTATGCACTTTGGCAACATGAAGTTTA
AGCAGAAGCAGCGTGAGGAGCAGGCTGAGCCTGACGGAACAGAGGCTGCT
GACAAGTCAGCTTATCTTATGGGGCTGAACTCTGCAGACCTCATCAAAGG
GCTATGCCATCCCAGAGTCAAGGTAGGAAATGAATATGTCACCAAAGGCC
AAGGTGTAGACCAAGTCTACTACCCAAACAAGGAAGCCTTCAAGTGTGAG
GAGTGTGGCAAGCACTACAACACCAAGCTGGGATACAAACGTCATGTGGC
CATGCACTCTGCCACAGCAGGGGACCTCACCTGTAAAGTGTGCATGCAGA
GCTATGAGAGCACGCCGGTGCTCCTGGAGCACCTTAAGAGCCACTCGGGG
AAGTCCTCGGGTGGCGCCAAGGAGAAAAAACACCCATGCGACCACTGCGA
CCGCCGTTTCTACACACGGAAGGATGTGAGACGGCACATGGTGGTCCACA
CGGGCCGAAAAGACTTCCTGTGCCAGTACTGTGCCCAGCGCTTTGGCAGG
AAGGACCACCTGACACGCCACGTAAAGAAGAGCCACTCGCAGGAGCTGCT
GAAGATCAAGACAGAGCCTCCAGATATGTTAGGTCTTCTAGGTTCTGGCT
CGCCACCTTGCTCAGTGAAGGAGGAGCTTAGCCCTATGATGTGCAGCATG
GGTCCCAACAAAGACCCCATGATGGGCAAACCCTTCCCCAGTGGGACCCC
CTTCCCCATAGGCATGTACAACCCCCACCAC------CTCCAGGCCATGT
CCAATTCTGGGGTGGGTCACCCC------CACCCCTCCCTGATGCCTAGC
CCCCTGTCTGCAGCTATGGGCATGGGCTGTCACATGGAATATCTCATCTA
TGCCTCTTTCTCATTCATGGGATGTTTACAAATCAGTGATGGTTCAAACA
TCGTGAACTTGCTGGCTAGTAACTCTCCGAGCGTTTCATATGCTCTGACC
CAGCAGAAATACTTCAGTAACTACAGTCCCGTGATTGGGTTTTACATTTA
CGAGCCCATTGAGTACTGGAACTCCACAGTGCAGGAGCACCTGAAGACAC
TGAGTCACGGCTTCAACAAGATCTCCTGGATGGACAACTTCTTCCACTAC
CTGCGGGTGGTGAACGTGAGTGCTTCGACCAAGAGTGACTTTATCACCAT
CCTCAAGGGCTCTTTCCTGCGCAGCCCGGAGTACCAGCACTTCACTGAGG
ACATCATCTTCTCCAAGA---ACCCTGAGAGTG------ATGAGTATGAC
ATTATCGCCTCACGCATGTACCTAGTGGCACGGACCACAGAGAAGAAGCG
CGAGGAGGTGGTGGAGCTTCTGGAGAAACTGCGTCCACTGATGCTGATCA
ATAGCATCAAGTTCATCGCCTTCAACCCCACCTTTGTTTTCATGGACCGC
TACAGCTCCTCAGTCATCTCGCCCATCCTGACCTCAGGCTTCAGYGTGCT
CACCATCCTCATCCTCACTTTCTTCCTGGTCATCAACCCCTTGGGGAACT
TCTGGCTCATCCTTACAGTTACCTCTGTGGAGCTGGGCGTCTTGGGTTTA
ATGGGCTATCATCTATTCGAATGGCAGCCAGCTCTCAAGAATGTGTCTAC
ATCCTGCCATGTGAGCATTATTAATGGGCTCTCTGGATGGGCTGCCTTGG
TGGATGACTCCCCAGCTGACACCATTACTCGACGGTTTCGCTATGACGTG
GCCCTGGTATCAGCCTTAAAGGATCTGGAGGAGCACATCATGGAGGGACT
GAGAGAGTGTGGGCTGGAAGACAGTGCTTGCACCTCAGGGTTCAGTGTAA
TGATCAAGGAATCTTGTGACGGCATGGGAGATGTCAGCGAGAAGCACGGG
GGAGGGCCAATGGTCCCCGAGAAGGCTGTACGCTTCTCTTTTACTGTTAT
GTCTGTCTCTGTCCTGGCAGATGGAGAGGAA------------------
-----------GAGGCGGTTACCATCTTTAGCGAGCCAAAGCCCAACTCA
GAACTGTCCTGTAAGCCCCTATGCCTGATGTTTGTGGATGAGTCAGACCA
TGAGACAGTCACTGCTGTCCTGGGACCTGTAGTTGCAGAGCGTAATGCAA
TGAAGCAGAGTCAACTCATCCTATCTATGGGTGGCCTCCCTCGCTCCTTC
CGCTTCCACTTCAGAGCCACAGGATATGATGAGAAGATGGTGCGTGAGAT
GGAGGGCCTGGAAGCCTCGGGTTCCACCTATATCTGCACTCTGTGTGATT
CCAGTCGAGCAGACGCCTCTCACAATATGGTGCTACACTCCATCACCCGC

AGCCAYGATGAGAACCTGGAGCGTTATGAATTATGGAGGACCAATCCCTA
TTCTGAGTCTGCTGACGAGCTGCGAGACCGGGTCAAAGGGGTCTCTGCCA
AGCCCTTTATGGAGACCCAGCCCACTATGGATGCATTACACTGTGACATT
GGTAATGCCACTGAGTTCTACAAAATCTTCCAGGATGAGATTGGGGAGGT
GTACCGGAAGGC---CAAT---CCCAGCCGTGAGGAACGGCGGAGCTGGA
GAGCAGCCCTAGACAAGCACCTGAGGAAGAAGATGAAGCTTAAACCAGTG
ATGAGGATGAATGGGAACTTTGCCCGGAAGCTAATGACCCAGGAAGCTGT
GGAGGTGGTGTGTGAGCTGGTGCCTTCAGAAGAGAGGCGAGAGGCCTTGA
GGGAGCTTATGGGGCTCTACATCCAGATGAAGCCTGTGTGGCGTGCCACC
TGCCCAGCCAAGGAATGCCCTGACGAGCTGTGCCGCTACAGCTTTAACTC
CCAGCACTTTGCCGATCTCCTCTCCAGTACCTTCAAATATAGGTACAATG
GAAAGATCACCAATTACCTGCATAAGACCCTCGCCCATGTCCCTGAAATC
ATTGAAAGAGATGGCTCTATAGGAGCCTGGGCCAGTGAGGGGAATGAGTC
AGCAAACAAATCGTACACCATCGAGATGGGTCCCAAGGGGCCCCAGTGGA
AGGAGAGTCCTCAGCCTTTCTCCTGCTCCATTGAAGACCCCACGAAACAG
ACCAAGTTCAAAGGCATCAAGACCTACATTTCGTACCGGGTCACGCCGAG
CCACATAGATCGTCCCGTCTACAGGCGCTACAAACACTTTGACTGGTTGT
ATAACCGCTTGCTGCACAAGTTCACTGTGATCTCGGTGCCCCACCTGCCT
GAAAAGCAGGCCACGGGGCGCTTCGAGGAGGACTTCATCGAGAAGCGTAA
AAGACGACTGATTCTGTGGATGAACCACATGACCAGTCACCCGGTTCTCT
CCCAGTACGAGGGATTTGAGCACTTCCTTATGTGTGCCGATGACAAGCAA
TGGAAGCTGGGTAAGAGGCGGGCGGAGAAGGACGAGATGGTGGGCGCCCA
CTTCATGTTGACCTTCCAGATCCCTAACGAGCATCAGGACCTCCAGGATG
TGGAGGAGCGGGTCGACTCCTTCAAGTCCTTCGCTAAGAAAATGGATGAC
AGCGTCATGCAGCTCACGCACGTTGCCTCGGAGCTGGTGCGTAAACACCT
GGGTGGATTCAGGAAGGAGTTCCAGCGGCTAGGAAACGCCTTCCAGTCCA
TCAGCCAGGCCTTCATGCTGGAACCCCCCCACAGCTCTGAGGCGCTCAAC
AACGCCATCTCCCACNNNNNNNNNNNCGTTCCTCAAACTGACCTCTCTGGGTTTCATCATT
GGAGTCGGTGTGGTCGGTAACCTGCTGATCTCCATCCTGCTGGTCAAAGA
CAAGAGCCTGCACCGTGCACCCTATTACTTCCTGCTAGACCTCTGCGCCT
CCGACATCCTGCGCTCTGCCATCTGCTTCCCCTTCGTCTTCACCTCTGTC
AAGAATGGATCCGCTTGGACCTACGGCACGCTCACCTGCAAAGTGATCGC
CTTCCTGGGCGTGCTCTCCTGTTTCCACACGGCGTTCATGCTATTCTGCG
TCAGCGTCACTCGCTACCTGGCTATCGCGCATCACCGCTTCTACACCAAA
AGGCTGACCTTCTGGACCTGTCTAGCCGTCATCTGCATGGTGTGGACGTT
GTCAGTAGCCATGGCGTTCCCCCCGGTGCTGGACGTAGGGACGTACTCCT
TCATCCGGGAGGAGGACCAGTGCACATTCCAGCACCGTTCTTTCAGGGCC
AATGACTCACTGGGCTTCATGCTCCTGCTGGCGCTCATCCTCCTCGCCAC
ACAGCTGGTTTACCTCAAGCTTATCTTCTTTGTCCACGACCGTCGGAAGA
TGAAGCCCGTCCAGTTCGTGCCTGCCGTCAGCCAGAACTGGACCTTCCAC
GGGCCGGGCGCCAGCGGGCAGGCGGCGGCTAACTGGCTCGCTGGATTTGG
GAGAGGCCCCACCCCGCCTACCTTGCTGGGCATCCGGCAGAACAGCAACG
CGGCGGGCCGCAGGCGTCTGCTGGTGCTGGATGAGTTCAAAACGGAGAAG
AGGATTAGTAGGATGTTCTACATCATGACGTTTTTCTTCCTGGCGTTGTG
GGGGCCCTACCTGGTCGCCTGCTACTGGCGGGTGTTTGCAAGGGGCCCCG
TAGTTCCCGGAGGATACCTGACGGCGGCCGTGTGGATGAGCTTTGCCCAG
GCTGGGGTCAATCCCTTCATCTGCATCTTCTCTAACNNNNNN--------------

------CATCACTCAACAGGCGAAGTCACGGAGAGAGAAGTGGCGTTGGG
GATCAATCCGTTCGCGGACGGGATGGGCGCCTTCAAAATCAACCACAGCT
CCCACGACATCGGCTCCGG---GCAGACGGCGTTCTCCTCCCAGGCG---
CCCGGGTAC---GCGGCGGCCGCGCTGGGA---CACCACCA------CCA
CCCCACCCACGTTGGCTCT---TACTCCACGGCGGCTTTCAACTCCACCC
GGGACTTTCTCTTCAGAAATCGGGGGTTCGGAGACGCCGCCGG-------
-----GGCGCAGCACAGTTTGTTCGCCTC---------CGGAAGTTT---
C------GCAGGGCCACACGGACACTCGGATGCCGCGGGGCACCTGCTCT
TCCCGGGACTGCACGAG---CAGGCGGCGAGCCACGCGTCTTCTAACGTG
GTCAACAGCCAGATGCGACTGGGCTTCTCGGGGGACATGTACGGTCGGGC
CGACCAGTACGGCCACGTTACGAGCCCGAGGT---CCGACCACTACGCTT
CGACCCAGTTGCACGGCTATGGCCCCATGAACATGAACATGGCCGCG---
CACCACGGAGCGGGGGCCTTCTTTCGATACATGAGGCAGCCGATCAAACA
AGAGCTCATCTGCAAGTGGATCGAACCGGAGCAGCTGACGAATCCCAAAA
AGTCGTGCAACAAAACTTTTAGCACCATGCACGAGCTGGTGACCCACCTG
ACCGTGGAGCACGTGGGGGGACCGGAGCAGTCCAACCACATCTGCTTCTG
GGAAGACTGCGCCAGAGAAGGGAAACCGTTCAAAGCCAAATACAAACTTG
TGAATCATATCAGAGTACACACCGGAGAGAAACCTTTTCCATGTCCGTTC
CCGGGCTGTGGANNN
>Diretmus argenteus
AGCCTTCTCATCCGAGCTGAACTCAGCCAGCCGGGGGCCCTCCTTGGTGA
TGACCAAATTTATAATGTCATTGTCACAGCCCACGCCTTCGTAATAATTT
TCTTTATGGTCATACCTATAATGATCGGCGGGTTCGGGAATTGACTCATT
CCCCTAATAATTGAAGCCCCTGACATAGCCTTCCCTCGAATAAATAACAT
AAGCTTTTGACTTCTACCCCCATCCTTTCTCCTCCTCCTTGCCTCTTCTG
GCGTCGAAGCAGGGGCAGGCACTGGGTGAACAGTCTATCCACCTCTCGCG
GGGAACCTGGCACATGCAGGAGCCTCCGTCGACCTAACCATCTTCTCTCT
GCACTTAGCAGGGATCTCCTCTATCCTCGGGGCAATTAACTTTATTACAA
CTATCTTTAACATAAAACCCCCGGGCGTCTCTATCTACCAAATCCCCCTA
TTTGTGTGGGCTGTCTTAATCACAGCAGTTCTACTCCTTCTCTCTCTGCC
AGTCCTTGCAGCTGGCATCACAATGCTTCTCACAGACCGCAACCTGAATA
CAACATTCTTCGACCCCGCAGGAGGGGGTGACCCCATCCTTTACCAACAC
CTG---

------------TTTCTAGAGCGAAACCTTCATCCATCTAATTGCCTGGG
CATGCTGCTGCTGTCTGATGCACACCAGTGTACCAAGCTNTCAGAGCTCTC
CTGGGGCATGTGCCTCAGCAACTTTCCTGCTATTTGCAAGAAAGAGGACT
TCCTCCARCTGCCCAAAGACATGGTGGTGCAGCTTCTATCCCAYGAGGAG
CTGGAGACAGAAGATGAGAGACTGGTTTATGAAGCTGCCCTTAACTGGGT
CAACTATGACCTGGAAAGGAGGCACTGCCATCTGCCAGAGCTGCTGAGAA
CGGTTCGCCTGGCCCTGCTACCTGCCATCTTCCTCATGGAGAATGTCTCG
ACAGAAGAGCTGATCAATGCCCAGGCCAAGAGCAAGGAGCTGGTGGATGA
GGCCATACGCTGCAAGCTGAAGATCTTGCAGAATGACGGTGTGGTTAACA

GCCCCTGTGCTCGGCYGAGAAAAACCAGCCATGCCCTTTTTCTGCTKGGA
GGGCAGACCTTCATGTGTGACAAGCTGTACCTGGTGGACCAGAAGGCGAA
AGAGATCATCCCCAAAGCTGACATCCCCAGCCCTAGGAAGGAGTTCAGTG
CCTGCGCCATTGGCTGTAAGGTCTACATCACAGGTGGCA--GAGGCTC-A
GAAAATGGTGTGTCTAAAGACGTATGGGTCTATGACACCGTCCACAAGGA
ATGGTCCAAGGCAGCACCCATGCTCATCGCCAGATTTGGTCATGGCTCTG
CTGANCTGAAACACTGCCTTTANNNNNNNNNNNNNNNNNNNNNNNNNNNNNNNNNNNNNNNNNNNNNNNN
NNNNGGATCACTACATTGTAGTGTTCAGCCGTT
CAACAACGAGGCTGATYCTGAACGAAGCGGAGCTAATCATGACGCTGGCC
CAGGAATTTCAGATGAGAGTRGTCACGGTGTCCTTGGAGGAACAAACGTT
CCCCAGCATTGCCCAGGTGATCAGCGGGGCCTCCATGCTAGTCAGCATGC
ATGGAGCTCAGCTTGTCACCTCACTCTTCCTCCCCAGGGGAGCTGCTGTG
GTGGAGCTGTTCCCCTACGCTGTTAACCCAGAGCAGTACACCCCATATAA
AACCCTTGCCACCCTACCAGGCATGGACCTTCAGTATGTTTCCTGGAGGA
ACAGTATAGAGGAGAACACTGTCACCCACCCAGACAGACCCTGGGAAGAA
GGAGGCATCGCCCATTTGGAAAAGGACGAGCAAGAACGAATACTAGCCAG
CAAGGATGTCCCCAGACACCTGTGCTGCCGCAACCCGGAGTGGCTATTCC
GAATCTACCAGGACACTTTGGTGGACATCCCTTCATTCCTGGAGGCCCTC
AA---AGAGGGCCTGAAG---ACCAGGCCCAGCTTGAAGAA---GGCCAA
GCTGGCCAGCACGGTTCATCCGGGCCGGGTCAGAGAACCCCAGTGCCACA
CCTCAGTCCAAGCCACCAACGAGGCTAAACTCACGGTTTCCTGGCAGATC
CCGTGGAATCTGAAATACCTGAAGGTGAGAGAAGTGAAGTACGAGGTGTG
GATCCAGAAGAAGGATTCTAGCAAGGGAACACTGGAGGATCAAATCATCC
AGGCAAACCCTGCGCTGGAGGCTTTCGGTAATGCCAAAACAATGAGGAAT
GACAACTCGTCCCGCTTTGGAAAATTCATCCGAATTCATTTCGGAACCAG
TGGCAAGCTGTCCTCTGCTGACATTGAAACGTACCTATTGGAGAAGTCAC
GTGTCACCTTTCAGCTCAAGGGTGAGAGAAACTATCACATTTTCTTCCAG
ATATTGTCTAATCAGAAGCCAGAGCTATTAGACATGATGTTGATCACTAA
CAACCCATATGACTACTCCTACATCTCCCAAGGAGAGGTAACAGTAGCAT
CCATCAACGACTCAGAGGAGCTGTTGGCCACTGACAGCGCCTTTGACGTG
CTTGGCTTCACTCCAGAGGAGAAGATGGGACTMTATAAGCTGATTGGTGC
CATTATGCACTTTGGCAACATGAAGTTTAAGCAGAAGCAGCGTGAGGAGC
AGGCTGAGCCTGACGGAACAGAGGCTGCTGACAAGTCAGCTTATCTTATG
GGGCTGAACTCTGCAGACCTCATCAAAGGGCTATGCCATCCCAGAGTCAA
GGTAGGAAATGAATATGTCACCAAAGGCCAAGGTGTAGACCAAGTCTACT
ACCCAAACAAGGAAGCCTTCAAGTGCGAGGAGTGTGGCAAGCACTACAAC
ACCAAGCTGGGATACAAACGCCATGTGGCCATGCACTCTGCCACAGCAGG
GGACCTCACCTGTAAAGTGTGCATGCAGAGCTATGAGAGCACGCCGGTGC
TCCTGGAGCACCTTAAGAGCCACTCGGGGAAGTCCTCGGGTGGCGCCAAG
GAGAAAAAACACCCATGCGACCACTGCGACCGCCGTTTCTACACACGGAA
GGATGTGAGACGGCACATGGTGGTCCACACGGGCCGAAAGGACTTCCTGT
GCCAGTACTGTGCCCAGCGCTTTGGCAGGAAGGACCACCTGACACGCCAC
GTAAAGAAGAGCCACTCGCAGGAGCTGCTGAAGATCAAGACAGAGCCTCC
AGATATGTTAGGTCTTCTAGGTTCTGGCTCGCCACCTTGCTCAGTGAAGG
AGGAGCTTAGCCCTATGATGTGCAGCATGGGTCCCAACAAAGACCCCATG
ATGGGCAAACCCTTCCCCAGTGGACCCCCTTCCCCATGGGCATGTACAA
CCCCCATCAC------CTCCAGGCCATGTCCAATTCTGGGGTGGGTCACC
CC------CACCCCTCCCTGATGCCTAGCCCCCTGTCTGCAGCTATGGGC
ATGGGCTGTCACATGGAATATCTCATCTATGCCTCTTTCTCATTCATGGG
ATGTTTACAAATCAGTGATGGTTCAAACATCGTGAACTTGCTGGCTAGTA
ACTCTCCGAGCGTTTCATATGCTCTGACCCAGCAGAAATANTTCAGTAACT
ACAGTCCTGTGATTGGGTTTTACATTTATGANCCCATTGAGTACTGGAACT
CCACAGTGCAGGAGCACCTGAAGACACTGAGTCATGGCTTCAACAAGATC

TCCTGGATGGACAACTTCTTCCACTACCTGCGGGTGGTGAACGTGAGTGC
TTCGACCAAGAGTGACTTTATCACCATCCTCAAGGGCTCTTTCCTGCGCA
GCCCGGAGTACCAGCACTTCACTGAGGACATCATCTTCTCCAAGA---AC
CCTGAGAGTG------ATGAGTATGACATTATCGCCTCACGCATGTACCT
AGTGGCACGGACCACAGAGAAGAAGCGCGAGGAGGTGGTGGAGCTTCTGG
AGAAATTGCGTCCACTGATGCTGATCAATAGCATCAAGTTCATCGCTTTC
AACCCCACCTTTGTTTTCATGGACCGCTACAGCTCCTCAGTCATCTCGCC
CATCCTGACCTCAGGCTTCAGCGTGCTCACCATCCTCATCCTCACTTTCT
TCCTGGTCATCAACCCCTTGGGGAACTTCTGGCTCATCCTTACAGTTACC
TCTGTGGAGCTGGGCNNNNNNNNNNNNNNNNNNNNNNNNNNNNNNNNNNNNNNNNNNNNNNNNNNNNNNNN
NNNNNNNNNNNTGCCATGTGGGCATTATTAATGGGCTCTCTGGATG
GGCTGCCTTGGTGGATGACTCCCCAGCTGACACCATTACTCGACGGTTCC
GCTATGACGTGGCCCTGGTGTCAGCCTTAAAGGATCTGGAGGAGCACATC
ATGGACGGACTGAGAGAGTGTGGGCTGGAAGACAGTGCTTGCACCTCAGG
GTTCAGTGTAATGATCAAGGAATCTTGTGATGGCATGGGAGATGTCAGCG
AGAAGCACGGGGGAGGGCCAGTGGTCCCCGAGAAGGCTGTACGCTTCTCT
TTTACTGTTATGTCTGTCTCTGTCCTGGCAGATGGAGAGGAA--------
----------------------GAGGCGGTTACCATCTTTAGCGAGCCAA
AGCCCAACTCAGAACTGTCCTGTAAGCCCCTATGCCTGATGTTTGTGGAT
GAGTCAGACCATGAGACAGTCACTGCTGTCCTGGGACCTGTAGTTGCAGA
GCGTAGTGCAATGAAGCAGAGTCAACTCATCCTATCTATGGGTGGCCTCC
CTCGCTCCTTTCGCTTCCACTTCAGAGCCACAGGATATGATGAGAAGATG
GTGCGTGAGATGGAGGGCCTGGAGGCCTCGGGTTCCACCTATATCTGCAC
TCTGTGTGATTCCAGTCGAGCAGACGCCTCTCACAATATGGTGCTACACT
CCATCACCCGCAGCCACGATGAGAACCTGGAGCGTTATGAATTATGGAGG
ACCAATCCCTTTTCTGAGTCTGCCGACGAGCTGCGAGACCGGGTCAAAGG
GGTCTCTGCCAAGCCCTTTATGGAGACCCAGCCCACTATGGATGCATTAC
ACTGTGACATTGGTAATGCCACTGAGTTCTACAAAATCTTCCAGGATGAG
ATTGGGGAGGTGTACCGGAAGGC---CAAC---CCCAGCCGCGAGGAACG
GCGGAGCTGGAGAGCAGCCYTAGACAAGCACCTGAGGAAGAAGATGAAGC
TTAAACCAGTGATGAGGATGAATGGGAACTACGCCCGGAAGCTAATGACC
CAGGAAGCCGTGGAGGTGGTGTGTGAGCTGGTGCCTTCAGAAGAGAGGCG
AGAGGCCTTGAGGGAGCTTATGGGGCTCTACCTCCNNNNNNNNNNNNNNNNNNNNNNNNNNNNNNNNN
NNNNNNNNNNNNNNNNNNNNNNNNNNNNNNNNNNNNNNNNNNNNNNNNNNNNNNNNNNNNNNNNNNNNNNN
NNNNNNNNNNNNNNNNNNNNNNNNNNNNNNNNNNNNNNNNNNNNNNNNNNNNNNNNNNNNNNNNNNNNNNN
NNNNNNNNNNNNNNNNNNNNNNNNNNNNNNNNNNNNNNNNNNNNNNNNNNNNNNNNNNNNNNNNTCGTACACC
ATCGAG
ATGGGTCCCAAGGGGCCCCAGTGGAAGGAGAGTCCTCAGCCTTTCTCCTG
CTCCATTGAAGACCCCACGAAACAGACCAAGTTCAAAGGCATCAAGACCT
ACATCTCRTACCGGGTYACGTCGAGCCACATAGATCGTCCCGTCTACAGG
CGCTACAAACACTTTGACTGGTTGTATAACCGCTTGCTGCACAAGTTCAC
TGTGATCTCGGTGCCCCACCTGCCYGAAAAGCAGGCCACGGGGCGCTTCG
AGGAGGACTTCATCGAGAAGCGTAAAAGACGACTGATTCTGTGGATGAAC
CACATGACCAGTCACCCGGTTCTCTCCCAGTAYGAGGGATTTGAGCACTT
CCTTATGTGTGCCGACGACAARCAATGGAAGCTGGGTAAGAGGCGGGCGG
AGAAGGAYGAGATGGTGGGYGCCCACTTCATGTTGACCTTCCAGATCCCT
AATGAGCATCAGGACCTCCAGGATGTGGAGGAGCGGGTCGACTCCTTCAA
GTCYTTCGCTAAGAAAATGGACGACAGCGTCATGCAGCTCACGCACGTTG
CCTCGGAGCTGGTGCGTAAACACCTGGGTGGATTCAGGAAGGAGTTCCAG
CGGCTAGGGAATGCCTTCCAGTCCATCAGCCAGGCCTTCATGCTGGAACC
CCCCCACAGCTCTGAGGCGCTCAACAACGCCATCTCCCACNNNNNNNNNNCATTCCTCAA
ACTGACCTCTCTGGGTTTCATCATCGGAGTCGGTGTGGTCGGGAACCTGC
TGATCTCCATACTGCTGGTCAAAGACAAGAGCCTGCACCGCGCGCCCTAT

TACTTCCTGCTGGACCTCTGCGCCTCCGACATCCTGCGCTCCGCCATCTG
CTTCCCCTTCGTCTTCACCTCTGTCAAGAATGGGTCGGCCTGGACCTACG
GCACGCTCACCTGCAAAGTGATCGCCTTCCTGGGCGTGCTCTCCTGTTTC
CACACGGCGTTCATGCTCTTCTGCGTCAGCGTCACGCGCTACCTGGCTAT
CGCGCACCACCGCTTCTACACCAAAAGGCTGACCTTCTGGACCTGTCTAG
CCGTCATCTGCATGGTGTGGACGTTGTCAGTAGCCATGGCGTTCCCCCCG
GTGCTGGACGTAGGGACGTACTCCTTCATCCGGGAGGAGGACCAGTGCAC
ATTCCAGCACCGTTCTTTCAGGGCCAATGACTCACTGGGCTTCATGCTCC
TGCTCGCACTCATCCTCCTGGCCACACAGCTGGTTTACCTCAAGCTCATC
TTCTTTGTCCATGACCGTCGGAAGATGAAGCCCGTCCAGTTCGTGCCTGC
CGTCAGCCAGAACTGGACCTTCCACGGGCCGGGCGCCAGCGGGCAGGCGG
CGGCTAACTGGCTGGCTGGATTTGGGAGAGGCCCCACCCCGCCTACCTTG
CTGGGCATCCGGCAGAACAGCAACGCGGCGGGCCGCCGGCGTCTGCTGGT
GCTGGACGAGTTCAAAACGGAGAAGAGGATTAGTAGAATGTTCTACATCA
TGACCTTTTTCTTCCTGGCGCTGTGGGGGCCCTACCTGGTCGCCTGCTAC
TGGCGGGTGTTTGCAAGGGGCCCCGTAGTTCCCGGAGGATACCTGACGGC
GGCCGTGTGGATGAGCTTTGCCNNNNNNNNNNNNNNNNNNNNNNNNNNNNNNNNNNNNNNNNNNNNN---
----

NNNNNNNNNNNNNNNNNNNNNNNNNNNNNNNNNNNNNNNNNNNNNNNNNNNNNNNNNNNNNNNNNNNNNN
NNNNNNNNNNNNNNNNNNNNNNNNNNNNNNNNNNNNNNNNNNN---NNNGACGG
CGTTTTCCCTCCAGGCG---CCCGGCTAC---GCGGCGGCCGCCCTGGGA
---CACCACCA------CCACCCGACCCACGTTGGCTCT---TACTCCAC
GGCGGCGTTCAACTCCACCCGGGACTTTCTCTTCAGAAATCGGGGGTTCG
GGGACGCCGCCGG------------GGCGCAGCACAGTTTGTTCGCCTC-
--------CGGAAGTTT---C------GCAGGGCCACACGGACACTCGGA
TGCCGCGGGACACCTGCTCTTCCCGGGACTCCACGAG---CAGGCAGCCA
GCCACGCGTCCTCTAACGTGGTCAACAGCCAGATGCGGCTGGGCTTCTCG
GGGGACATGTACGGCCGGGCCGACCAGTACGGCCACGTCACGAGCCCCAG
GT---CCGACCACTACGCCTCGACCCAGTTGCACGGCTACGGCCCCATGA
ACATGAACATGGCCGCG---CACCACGGAGCCGGGGCCTTCTTTCGGTAC
ATGCGCCAGCCGATCAAACAGGAGCTCATCTGCAAGTGGATCGAGCCCGA
GCAGCTGACGAACCCCAAAAAGTCGTGCAACAAAACTTTCAGCACCATGC
ACGAGCTGGTGACCCACCTGACCGTGGAGCATGTGGGGGGCCCGGAGCAG
TCCAACCACATCTGCTTCTGGGAGGACTGCGCCAGAGAGGGGAAACCGTT
CAAAGCCAAATACAAACTTGTGAATCATATCCGAGTGCACACCGGAAAGA
AACCCTTCCCGTGTCCGTTTCCCNNNNNNNNNNNN
>Dissostichus eleginoides
AGCCTGCTCATCCGGGCAGAACTCAGCCAACCNGGCGCCCTATTGGGAGAC
GACCAAATTTATAACGTGATTGTTACCGCTCACGCCTTCGTAATAATCTT

TTTTATGGTCATACCGATTATAATTGGAGGCTTCGGAAACTGACTCATCC
CCCTCATGATCGGAGCCCCCGACATGGCTTTCCCCCGAATAAATAATATG
AGTTTCTGGCTCCTTCCTCCCTCCTTCCTACTACTACTAGCTTCTTCAGG
TGTAGAAGCCGGGGCGGGAACCGGCTGGACCGTCTATCCCCCCCTCTCTG
GAAACTTAGCCCACGCAGGTGCATCCGTTGACCTAACTATTTTTTCTCTG
CATTTAGCAGGCATTTCTTCCATCTTAGGTGCAATTAATTTTATCACAAC
AATTATTAACATGAAACCCCCTGCCATCTCTCAATACCAAACCCCTCTGT
TCGTATGGGCCGTCCTAATTACTGCCGTTCTTCTACTTCTTTCACTTCCT
GTGCTAGCTGCCGGAATTACCATGCTTCTCACGGACCGAAACCTCAACAC
CACCTTCTTTGACCCTGCAGGAGGGGGAGACCCTATTCTTTACCAACATC
TTTTC---

-----------TTCCTGGAGAGAAACCTGCACCCGTCTAACTGCCTTGGC
ATGCTGTTGCTGTCTGACGCCCACCAGTGCATCAAGCTGTCAGAGCTCTC
CTGGGGAATGTGCCTCAGCAACTTTCCCACTATTTGCAAGACAGAGGACT
TCCTCCAACTGCCCAAAGATATGGTGGTGCAGCTTTTGTCCCATGAGGAG
CTAGAGACAGAAGATGAGAGACTGGTCTATGAGGCGGCCCTTAACTGGAT
CAACTACGACCTGGAAAAGAGGCACTGCCACCTTCCAGATCTCCTGAGAA
CGGTCCGCCTGGCCCTGCTGCCCGCCATCTTTCTCATGGAGAACGTTTCT
ACAGAAGAGCTGATCAACGCCCAGCCTAAGAGCAAGGAGCTGGTGGATGA
AGCTATCCGCTGTAAGCTGAAGATCCTGCAGAACGATGGCGTGGTTAACA
GCCCTTGTGCCCGACCAAGGAAGACCAGCCATGCCCTCTTTCTTCTGGGA
GGGCAGACTTTCATGTGTGACAAGTTGTACCTGGTGGACCAGAAAGCCAA
AGAGATCATCCCCAAGTCTGACATCCCCAGCCCCAGGAAGGAGTTCAGCG
CCTGCGCCATTGGCTGTAATGTGTACATCACCGGTGGCA--GAGGTTC-A
GAGAATGGTGTATCCAAAGATGTGTGGGTCTATGACACCGTCCAAGAGGA
ATGGTCGAAGGCGGCACCCATGCTCATTGCCAGGTTTGGCCATGGCTCTG
CGGAGCTGAAACACTNNNNNNNNNNNNNNNNNNNNNNNNNNNNNNNNNNNNNNNNNNNNNNNNNNNNNNN
NNNNGGATGAATACATTGTACTGTTTAGTCGTTCAACAA
CACGGCTGATACTGAACGAGGCGGAGCTGATCATGGCGCTGGTCCAGGAG
TTCCAGATGAGAGTGGTCACAGTATCCCTAGAGGAACAGTCCTTCCCCAG
TACCGTCCAGGTGATCAGTGGGGCTTCCATGTTAATCAGTATGCATGGAG
CTCAGCTAATCACCTCACTCTTCCTCCCTAGAGGAGCTGTTGTGGTGGAG
TTGTTCCCTTTTGCTGTTAACCCTGAACAGTACACCCCGTATAAAACCCT
TGCCACCCTTCCAGGCATGGATCTTCACTATATCTCTTGGAGGAACACCA
AGGAGGAGAACACCATCACCCACCCAGATAAACCTTGGGATCAAGGGGGC
ATTACTCACTTGGAGAAGGAGGAGCAAGAGCGAATCCTGGCGAGCAAAGA
TGTGCCCAGGCACCTGTGCTGTCGCAACCCGGAGTGGCTCTTCCGGATCT
ACCAGGACACTTTGGTGGACATCCCTTCCTTCTTGGAAGTCCTTAA---A
GAGGGAATGAAG---ACAAAGCCCAGTGTGAGAAG---ATCCAAACCGGC
CAGCACAGTTCATCCGGGCCGGGTCAGAGAACCCCAGTGTCAGACCTCGG
TACAAACCACTAATGAGGCTAAACTCACAGTCTCCTGGCAGATCCCGTGG
AATCTGAAATACCTGAAAGTGAGAGAGTTGAAGTACGAGGTGTGGATCCA
GAAAAGAGACCCCAGCAAGGGCACGCTGGAGGACCAAATCATCCAGGCCA
ACCCCGCACTGGAGGCTTTCGGCAACGCCAAGACGCTGAGAAACGACAAC
TCCTCTCGTTTTGGAAAATTCATCCGAATTCACTTTGGGACGAGCGGCAA
GTTGTCGTCTGCTGACGTCGAGACGTACCTGCTGGAGAAGTCCCGCTGCA
CCTTTCAGCTCAAGGCTGAGAGGAACTACCACATCTTCTACCAGATCCTG
TCCAACCAGAAGCCAGAGCTCCTGGACTTGCTGCTGATCACCAACAACCC
GTACGATTACGCCTACATCTCCCAAGGAGAGGTAACCGTCGCCTCCATCA
ACGACGCAGAGGAGCTGATGGCCACCGACAGCGCCTTCGACGTGCTCGGC

TTTACTGCGGAAGAGAAGATGGGCGTCTACAAGCTAACAGGCGCCATCAT
GCACTACGGCAACATGAGGTTCAAACAGAAGCAGCGCGAGGAGCAGGCTG
AGTCGGACGGGACGGAGGCGGCAGATAAATCTGCGTACCTGATGGGGCTG
AACTCTGCCGACCTCATCAAAGGGCTGTGCCATCCCAGAGTCAAGGTAGG
AAACGAATACGTCACCAAAGGCCAAAGTGTGGACCAAGTCTACTACCCCA
ACAAGGAGGCCTTCAAGTGTGAGGAGTGTGGGAAGCACTACAACACCAAG
CTGGGGTACAAGCGCCATGTGGCCATGCACTCTGCCACGGCGGGGGATCT
CACCTGTAAAGTGTGCATGCAGAGCTACGAGAGCACCCCCGTTCTGTTGG
AGCACCTCAAAAGCCACTCCGGGAAGTCTTCAGGCGGCACCAAGGAGAAG
AAGCACCCGTGTGATCACTGTGACCGTCGCTTCTACACCCGGAAGGACGT
GAGACGGCACATGGTGGTGCACACGGGCCGAAAGGACTTCCTCTGCCAGT
ACTGTGCCCAGCGCTTCGGCAGGAAGGACCACCTGACCCGACACGTGAAG
AAGAGCCACTCTCAGGAGCTGCTGAGGATCAAGACGGAGCCCCCTGACAT
GTTGGGTCTTTTAGCCTCTGGGTCTCCACCCTGCTCTGTGAAGGAGGAGC
TCAGCCCCATGATGTGCGGCATGGGGCCCAACAAAGACCCCATGATGGGC
AAACCGTTCTCCGGTGGGGCCCCTTTTTCAATGGGCATGTACAACCCCCA
CCAT------CTCCAGACCATGTCTAATACTGGGGGGGGTCACCCT----
--CACCCGTCCCTGATGCCCAGTCCGTTGTCTGCAGCTATGGGCATGGGG
TGTCACATGGAATATCTCATCTACGCATCTTTCTCATTCATGGGATGTTT
ACAAATCAGTGATCGATCAAATATTGTGAACCTGCTGGCGAGTAACTCTC
CGAGTGTTTCGTATGCCCTGACCCAGCAGAAATACTTCAGTAACTACAGT
CCCGTGATTGGGTTTTACATTTACGAACCCATCGAGTACTGGAATTCCAC
GGTGCAGGAGCACCTGAAGACTTTGAGTCATGGCTTCAACAAGATCTCTT
GGATGGACAACTTTTTCCACTACCTGCGGGTGGTGAATGTGAGTGCGTCA
ACTAAGGGTGACTTCATCACAATCCTCAAGGGCTCCTTTCTGCGCAGCCC
TGAGTACCAGCACTTCTCTGAGGACATCATATTCTCCAAGA---ACCGTG
AGACCG------ATGAGTACGACATCATCGCCTCTCGGATGTATTTGGTT
GCCCGGACGACGGAGAAGAAGCGAGGGGAGGTGGTGGAGCTTCTGGAGAA
GCTTCGTCCGTTGATGCTGATCAACAGCATCAAGTTCATTGCCTTCAATC
CTACGTTTGTGTTCATGGACCGCTACAGCTCCTCTGTCATCTCGCCTATC
CTGACCTCAGGCTTCAGCGTACTCACAATCCTCATCCTCACTTTCTTCCT
GGTCATCAACCCCTTGGGTAACTTCTGGCTCATCCTCACAGTAACGTCCG
TCGAGCTGGGCGTCTTGGGTTTGATGGGCTTCCTCGAGTTTGAATGGCAG
CCAGCTCTCAAGAATGTGTCTACATCTTGCAATGTTGGCATTATTAACGG
GCTCTCTGGATGGACTTCCTCGGTGGATGACTTCCCAGCTGACACCATCA
CTCGCCGGTTTCGCTACGATGTGGCTCTGGTGTCAGCATTAAAGGATCTG
GAGGAGGACATCATGGAGGGTCTCAGAGAGGCTGGTATGGAAGACAGTAC
TTGTATCTCAGGCTTCAGTGTCAAGATCAAAGAATCCTGTGATGGCATGG
GCGATGTCAGTGAGAAACATGGCGGGGGACCAGCTGTCCCTGAGAAGGCT
GTTCGTTTTTCTTTTACTGTCATGTCTGTCTCTGTCAAGCCAGACAACGA
GGAG------------------------------AAGGAGGTTACCATTT
TCACTGAGCCCAAGCCGAACTCAGAACTGTCATGTAAGCCCCTTTGCCTG
ATGTTTGTGGATGAGTCAGACCATGAGACACTCACAGCTGTCCTGGGGCC
TGTAGTTGCCGAGCGTAAGGCAATGAAGGAGAGCAAGCTCATCCTATCCT
TGGGTGGACTAGCTCGCTCCTTCCGATTCCACTTCAGAGGTACAGGATAC
GATGAGAAGATGGTGCGTGAGATGGAGGGCCTTGAAGCCTCTGGGTCCAC
CTATGTCTGCACTCTCTGTGACTCCACCCGGGCAGAGGCCTCTGAAAACA
TGGTGCTACACTCGGTCACTCGCAGTCATGTAGAGAACTTGGATCGCTAC
GAGATATGGAGAACCAACCCCTTTTCCGAGTCTGTAGACGAGCTGCGGGA
CAGAGTCAAAGGTGTCTCCGCCAAGCCGTTCATGGAGATGCACCCCACGC
TGGATGCGCTACACTGTGACATTGGCAATGCGACAGAGTTCTACAAAATC
TTCCAGGACGAGATCGGGGAGGTGTACAAAAAGGT---CAAC---CCCAG
TCGGGAGGAGCGGCGCAGCTGGAGGGCAGCCCTGGATAAACAGCTGAGGA

AAAAGCTGAAGGTCAAACCGGTCATGAGGATGAATGGGAACTATGCCCGC
AGGCTAATGACCCAGGAGGCTGTGGATGTGGTGTGTGAGCTGGTGCCCTC
AGAGGAGCGGAGGGAAGCCCTCAGGGAGCTTATGAGTATCTACGTCCAGA
TGAAGCCCGTGTGGCGTGCCACCTGCCCTGCCAAGGAGTGCCCCGACCAG
CTGTGCCGCTTCAGCTTTAACTCACAGCGCTTTGCAGACCTTCTCTCTTC
TACCTTCAAATATAGATACAATGGAAAGATAACCAATTATTTGCACAAGA
CCCTGGCTCATGTGCCTGAAATCATAGAGAGAGATGGATCCATAGGAGCC
TGGGCCAGCGAAGGGAACGAGTCGGCAAACAAATCATACACCATTGAGAT
GGGTCAATTGGGGCCTGTTTGGCAGGCGAGCCCAAAGCCTTTCTCCTGCT
CCGTCGAAGACCCCACAAAACAGACAAAGTTCAAGGGTATCAAGACGTAC
ATTTCCTACCGTGTCACGCCGAGCCACATAGGGCGTGCCGTGTACAGACG
CTACAAACATTTTGACTGGCTGTACAACCGCTTACTGCACAAGTTCACTG
TGATCTCCGTGCCTCACCTGCCTGAGAAGCAGGCCACGGGGCGATTTGAG
GAAGACTTCATCGAGAAGCGCAAGAGGCGACTGATACTGTGGATGAACCA
CATGACCAGTCACCCAGTACTCTCCCAGTATGAAGGCTTAGAGCACTTTC
TAATGTGTACTGATGACAAGCAGTGGAAACTGGGCAAGAGGCGGCAAGAA
AAGGACGAGATGGTTGGTGCCCATTTCATGCTGACCCTTCAGATCCCCAA
CGAGCACCAGGACCTTCAGGATGTAGAGGAGCGCATCGACACCTTCAAGG
CCTTTGCTAAGAAAATGGATGACAGCGTTTTGCAGCTCACACATGTTGCT
TCGGAGCTGGTTCGTAAACACCTGGGTGGGTTCAGGAAGGAGTTCCAGCG
GCTGGGAAATTCTTTCCAATCTATCAGCCATGCGTTCACGCTGGACCCTC
CCAACAGCTCAGAGAATCTCAACAAAGCCATCTCCCATCCTCTCGCCACG
TTCCTCAAACTGACCTCTCTGGGTTTCATCATTGGAGTCGGTGTGGTTGG
AAACCTCCTGATCTCCATCCTGCTGGTCAAAGACAAGAGCCTGCATCGGG
CGCCCTACTATTTCCTGCTGGACCTGTGCGCCTCTGACATCCTGCGCTCC
GCCATCTGCTTCCCCTTTGTCTTCACCTCGGTCAAGAATGGATCGGCCTG
GACCTACGGCACGCTGACCTGCAAAGTGATCGCCTTCCTGGGTGTCCTCT
CCTGTTTCCACACAGCGTTTATGCTCTTCTGTGTCAGCGTCACACGCTAC
CTTGCCATCGCGCACCACCGTTTCTACACCAAGAGGCTGACCTTCTGGAC
CTGTCTGGCTGTCATCTGCATGGTGTGGACGTTGTCAGTGGCTATGGCGT
TCCCGCCGGTGCTAGACGTAGGGACATACTCTTTTATCCGGGAGGAGGAC
CAGTGCACGTTCCAGCACCGTTCCTTCAGGGCGAACGACTCGCTGGGCTT
CATGCTCCTGCTGGCGCTCATCCTCCTGGCCACACAGCTGGTTTACCTCA
AGCTCATCTTCTTCGTGCATGACCGTCGGAAGATGAAGCCTGTCCAGTTC
GTGCCTGCTGTCAGCCAGAACTGGACCTTCCACGGGCCTGGCGCCAGCGG
GCAGGCGGCGGCCAACTGGCTGGCCGGATTCGGTCGAGGCCCCACCCCCC
CTACCCTGCTGGGCATCCGGCAGAACAGCAACGCAGCAGGCCGCAGGCGT
CTACTGGTATTGGATGAATTCAAAACAGAGAAGAGGATTAGTAGGATGTT
CTACATCATGACGTTTTTCTTCCTGGCACTGTGGGGGCCCTATCTGGTCG
CCTGCTACTGGCGGGTGTTTGCGAGGGGCCCAGTGGTCCCTGGGGGCTAC
CTGACGGCGGCCGTGTGGATGAGCTTCGCCCAGGCCGGGGTCAATCCCTT
CATCTGCATCTTCTCCAACAGGGAGGCCAAATCTCGCTTTCACCCTGGCG
TGGGGACTTGTCCTGGCACGGAGC---GCAGCGTCCCACTCGGCAACAGC
TTGCTGTCCCCGCAGCAAACCGACGAGCCCACTGTTGCCACCCCCCCGCA
GCGATGGTTTGTCACCC---CTGCCAACAACCGACTGGACTTTGCTGCCT
CGGCATACGACGCCGCT---------GATTTCGCCGGTAACGCGGCCACC
TTGCTGTCCTACGCAGCGGCCGGAGTGAAGGCTC------TTCCCCTGCC
GAGTGCGGGCTGCTCCAACCGGCCTCTTGGCTATTACGCAGACCCGTCAG
---GCTGG---GGAGGACGCACGCCGCCGCAGTACTGTGGCGTAAACAGC
AAACCCAGCGCGGTCTTTTCCTGCTGGCCCGCTAACTCTCTCGGTGGCAG
GTCGGGCA---CC---AACTACCTGA---------GCGAGGA---GGGA-
--GACCC---CATCCCAACAGAGAGGTCACCT---AT---CGGCGTCTCC
GAGGAG---CCCAAAGCCAAAGACATGAC---ATCAGA---GTCGAACTG

GATAGAG---ACGCCGTCCTCCATTAAGTCCATCGACTCGAGCGATTCTG
GTATCTTTG---AACAGGCCAAAAGGAGGAGAATCTCACCTTCTGCCACG
CCG---------GTTTCAGAGACAGTGTCCCCGTTAAAATCGGAGCATCA
CTCAACAGGCGAAGTCACGGAGAGAGAAGTGGCGTTGGGGATCAATCCCT
TCGCGGATGGGATGGGCGCCTTCAAAATCAACCACAGCGCCCACGATATT
GGCTCCGG---ACAGACGGCGTTCTCCTCCCAGGCT---CCCGGGTAC--
-GCAGCCACCGCGCTGGGA---CACCACCA------CCACCCGAGCCACT
CTTACTCC---------------ACCTTCAACTCCACCAGGGACTTTCTG
TTCAGAAATCGGGGTTTCGGGGATGCCACCGG------------CGCGCA
GCACAGTTTGTTCGCCTC---------CGGAAGTTT---C------GCAG
GGCCACATGGACACTCGGATGCGGCGGGGCACCTGCTCTTCCAGGGGCTC
CACGAG---CA-G-----AACCACGCGCCCTCCAACGTGGTTAACAGCCA
GATGCGGCTGGGCTTCACTGGGGACATGTATGGACGTGCAGACCAGTATG
GCCACGTTACGAGCCCCAGGT---CCGACCATTACGCATCATCCCAGCTG
CACGGCTACGGCCCCATGAACATGAATATGGCCGCG---CACCACGGGGC
AGGGGCCTTCTTCCGATACATGAGGCAGCCCATCAAACAGGAGCTCATCT
GCAAGTGGATCGAGCCGGAGCAGCTGACGAACCCCAAAAAGTCATGCAAC
AAAACTTTTAGCACCATGCACGAGCTGGTGACCCATCTGACGGTGGAGCA
TGTGGGGGGGCCCGAGCAGACCAACCACATCTGCTTCTGGGAGGAATGCG
TCCGAGAAGGGAAGCCATTCAAAGCCAAATACAAACTTGTGAATCATATC
AGAGTACACACCGGAGAGAAGCCGTTTCCCTGTCCGTTCCCCGGCTGTGG
GAAA
>Echeneis naucrates
AGCTTACTCATTCGGGCAGAACTTAGTCAACCAGGCTCATTATTAGGTGA
TGATCAGATTTATAATGTTATCGTCACAGCACATGCCTTTGTAATAATTT
TCTTTATAGTTATACCAGTAATGATTGGAGGTTTTGGTAATTGATTAGTA
CCTCTTATAATTGGTGCACCAGACATAGCCTTCCCTCGAATAAATAATAT
AAGCTTCTGACTACTGCCTCCTTCCTTCCTCCTACTGCTAACATCTTCAG
GAGTAGAAGCAGGAGCAGGAACTGGTTGAACTGTTTATCCTCCTTTAGCC
GGAAACCTTGCCCATGCAGGAGCATCTGTTGACCTAACTATCTTTTCACT
TCATCTGGCAGGAATTTCCTCAATTCTTGGAGCAATTAATTTTATTACAA
CAATCATTAATATGAAACCTGCAGCTGCTTCTATATATCAACTCCCATTA
TTTGTATGAGCCGTATTAATTACAGCAGTTCTTCTTCTCCTATCCCTCCC
TGTTTTAGCTGCTGGAATTACAATACTACTAACAGACCGTAATCTTAATA
CCGCCTTCTTTGATCCTGCAGGAGGGGGAGATCCCATCCTTTATCAACAC
TTATTC--

------------TTCCTGGAGAGAAACCTTCACCCCTCTAACTGCCTTGG
CATGCTGTTGCTGTCCGACGCCCACCAGTGCACCAAGCTGTCGGAGCTCT
CCTGGGGCATGTGCCTCAGCAACTTCCCTGCCATTTGCAAGACAGAGGAC
TTCCTTCAGCTGCCCAAAGATATGGTGGTCCAGCTTTTATCACACGAGGA
GCTCGAGACAGAAGACGAGAGACTGGTTTATGAAGCTGCCCTTAACTGGA
TCAACTATGACCTGGAGAGGAGGCACTGCCACCTTCCAGAGCTTCTGAGA
ACGGTCCGCCTCGCCCTGCTGCCCGCCATCTTTCTGATGGAGAACGTCTC
AACAGAGGAGCTGATCAACGCCCAGCCCAAGAGCAAGGAGCTGGTGGACG
AAGCCATCCGTTGTAAGCTGAAAATCCTGCAGAACGATGGCGTCGTTAAC
AGCCCATGTGCTCGACCGAGAAAAACCAGCCATGCCTTGTTTCTTCTGGG
AGGGCAGACATTCATGTGTGACAAGTTGTACCTAGTTGACCAGAAGGCCA
AAGAGATCATACCCAAAGCTGATATCCCGAGCCCCAGAAAGGAGTTCAGT
GCCTGCGCCATCGGCTGTAAGGTGTACATCACTGGGGGGA--GAGGCTC-
AGAGAAYGGTGTGTCCAAAGATGTATGGGTCTACGACACTGTCCACGAGG

AATGGTCCAAAGCTGCTCCCATGCTCATCGCTAGATTTGGCCATGGCTCC
GCAGAGCTCAAACATTGCCTCTACGTGGTGGGAGGTCACACGGCAGCGAC
CGGTTGCCTCCCAGCTTCTCCGTCAGGACAATTACATTGTTGTGTTCAGT
CGTTCAACAACAAGGCTGATAGTGAACGAAGCAGAGCTCATCATGGCTCT
GGCCCAGGAGTTCCAGATGAGAGTGATCACAGTATCCCTGGAAGAGCAGT
CGTTCCCCAGTATTATTCAGGTGATCAGTGCTGCCTCCATATTGGTCAGC
ATGCACGGAGCTCAACTAATCACTTCACTGTTCCTCCCCAGAGGAGCTGT
TGTGGTGGAACTTTTTCCCTTTGCTGTGAACCCAGAGCAATACACCCCTT
ACAAAACCTTGGCCTCTCTTCCTGGCATGGACCTTCACTACATTTCCTGG
AGGAACACCAAAGAGGAAAACACCATCACCCACCCGGACAGACCCTGGGA
ACAAGGAGGCATTGTTCACTTGGAGAAAGAGGAGCAGGAGAGAATAGTGG
CCAGTAAGGACGTCCCCAGGCACTTGTGCTGTCGTAACCCGGAGTGGCTC
TTCCGAATCTATCAGGATACATTGGTGGACATCCCTTCCTTTGTGGAAGT
CTTACA---AGAGGGTATGAAG---ACCAAGCCCAGCTTCAAAAA---GT
CTAAACTGGCCAGTACAGTCCACCCAGGCCGAGTCAGAGAGCCTCAATGT
CAAACCTCAGTCCAAACCAGTAACAAGGCAAAACTCACAGTCTCCTGGCA
TATCCCGTGGAATCTGAAATACCTGAAGGTAAGAGAGGTAAAATATGAAG
TGTGGATCCAG---------------------------------------

------CCCAACAAGGAGGCCTTCAAGTGTGAGGAATGTGGTAAGCACTA
CAACACCAAGCTGGGATATAAGCGCCATGTGGCCATGCACTCTGCCACGG
CAGGGGATCTCACCTGTAAAGTGTGCATGCAGAGCTATGAGAGCACACCT
GTCCTCTTGGAGCACCTCAAGAGTCACTCTGGGAAGTCTTCAGGTGGCAC
CAAGGAGAAAAAGCACCCGTGTGACCATTGTGACCGTCGTTTCTACACAC
GGAAGGATGTGCGACGACATATGGTTGTTCACACGGGCCGAAAGGACTTC
TTGTGCCAGTACTGCGCACAGCGCTTTGGCAGAAAGGACCATCTAACACG
CCACGTAAAGAAGAGCCACTCACAGGAGCTGCTGAAGATCAAGGCGGAGC
CTCCAGATATGTTAGGTCTCTTAGCCTCCAGATCGCCACCTTGTTCTGTG
AAGGAGGAACTCAGCCCCATGATGTGCGGCATGGCTCCCAACAAAGACCC
CATGATGGGCAAACCATTCCCCAGTGGTGCCCCTTTTCCAATGGGCATGT
ACAACCCCCACCAT------CTCCAGGCAATGTCTAATACTGGGGTGGGT
CACCCA------CACCCGTCCCTGATGCCCAGTTCTCTGTCTGCCGCTAT
GGGCATGGGATGTCACATGGAATATCTCATTTATGCCTCATTCTCATTTA
TGGGATGTTTACAAATCAGTGATGGGTCAAACATTGTCAATCTGCTGGCG
AGTAACTCTCCGAGTGTTTCATATGCTCTGACCCAGCAGAAATACTTCAG
TAACTACAGTCCTGTGATCGGGTTTTACATTTACGAGCCCATCGAGTACT
GGAACACCACGGTGCAGGAGCACCTGAAGACCCTGAGTCATGGCTTCAAT
AAGATCTCCTGGATGGACAATTTTTTCCACTACCTGCGGGTGGTCAATGT
GAGTGCGTCAACCAAGAACGACTTCATTACCATCCTCAAAGGTTCCTTCC
TGCGCAGCCCAGAGTACCAGCATTTCACAGAGGACATAATATTTTCCAAG
A---ACCGTGAGACTG------ACGAATACGACATCATTGCCTCACGGAT
GTACTTGGTTGCACGAACGACCGAGAAGAAGCGTGAGGAGGTAGTTGAGC

TTCTGGAAAAGCTTCGGCCGTTGATGCTAATTAACAGCATCAAGTTCATT
GCCTTCAATCCCACGTTTGTGTTCATGGACCGCTACAGCTCCTCTGTCAT
CTCGCCCATCCTGACCTCAGGCTTCAGCGTACTCACTATCCTCATCCTCA
CTTTCTTCCTGGTCATCAACCCCTTGGGGAACTTCTGGCTCATCCTCACG
GTCACTTCTGTGGAGCTGGGCGTCCTGGGTTTGATGGGCTTCCACCAGTT
TGAATGGCAGCCAGCTCTCAAGAATGTGTCAACATCTTGCAATGTTGGCA
TTATTAATGGGCTTTCAGGCTGGGCTTCCTCGGTGGATGACTCCCCAGCT
GACACCATCAGTCGGCGCTTTCGCTATGATGTGGCTCTGGTGTCAGCATT
AAAGGATCTGGAGGAGGACATTATGGAGGGGCTGAGAGAGAGTGGGAGGG
AGGACAGTGCTTGCACCTCAGGCTTTTCTGTCATGATCAAGGAATGTTGT
GATGGCATGGGCGACGTCAGCGAGAAGCACGGTGGAGGACCAGCTGTTCC
TGAGAAGGCTGTACGCTTCTCTTTCACCGTTATGTCTATATCCGTTCGGG
CAGACGATGATGAG------------------------------GAGGAG
GTTACCATCTTCACCGAACCAAGGCCAAACTCAGAGCTGTCCTGTAAGCC
CCTCTGCTTGATGTTTGTTGATGAGTCAGACCATGAGACGCTCACAGCTC
TCCTGGGCCCCATAGTTGCAGAGCGTAATGCCATGAAAGAGAGCAGGCTC
ATTCTGTCCGTCGGCGGCCTGCCTCGCTTCTTCCGCTTCCAATTCAGAGG
CACAGGATATGATGAGAAGATGGTGCGAGAATTGGAAGGCCTGGAGTCCT
CGGGGTCAACGTACATCTGTACCCTGTGTGACTCGAGTCGGGCAGAGGCC
TCTAAAAACATGGTGCTGCACGCCATCACCCGCAGCCATGAAGAGAACTT
AGATCGTTATGAAATATGGAGAACCAATCCCTTCTCCGAGTCTGCGGATG
AACTGCGGGACAGAGTCAAAGGGGTCTCTGCGAAGCCCTTCATGGAGACG
CAGCCCACAATGGATGCATTGCACTGTGACATCGGCAACGCCACTGAGTT
CTACAAAATTTTCCAGGATGAGATTGGTGAAGTGTACCAAAAGGT---CA
GC---CCCAGCCGGGAGGAGAGGCGCAGCTGGAGGGCGGCCCTGGATAAA
CAGCTGAGGAAGAAGATGAAACTTAAGCCAGTAATGAGGATGAATGGGAA
CTACGCCCGCAGGCTAATGACCCAGGAGGCTGTGGAGGTGGTGTGTGAAC
TGGTGCCCTCAGAGGAGAGGAGGGAGGCCCTGAGGGAGCTCATGAGGCTC
TACCTCCAGATGAGGCCGGTGTGGCGCGCCACCTGCCCAACAAAGGAATG
TCCAGACCAGCTGTGCCGCTACAGCTTCAACTCCCAGCGCTTTGCCGACC
TCCTCTCTTCTACCTTCAAATATAGGTACAATGGAAAGATAACCAATTAC
CTCCACAAGACTCTGGCCCATGTGCCTGAAATCATAGAGAGAGACGGATC
CATAGGAGCCTGGGCCAGTGAGGGGAATGAGTCAGCAAACAAATCATACA
CTATCGAGATGGGTCCTTTGGGACCTCGGTGGAAGGATAACCCGCAACCT
TTCTCCTGCTCCATTGAAGACCCCACAAAACAGACAAAGTTCAAGGGCAT
CAAGACCTACATTTCCTACCGGGTCACGCCGAGCCACACTGGGCGTCCCG
TCTACAGGCGTTACAAACATTTTGATTGGCTCTACAACCGCCTACTGCAC
AAGTTCACTGTGATCTCTGTGCCTCATCTGCCTGAGAAACAGGCTACGGG
GCGATTTGAGGAAGACTTTATTGAGAAGCGTAAAAGGAGACTTATACTAT
GGATGAACCACATGACCAGTCACCCAGTCCTCTCCCAGTATGAAGGCTTT
GAACACTTTCTGATGTGTGCTGACGACAAGCAGTGGAAACTGGGAAAGAG
ACGCGCGGAGAAGGATGAGATGGTGGGCGCCCATTTCATGCTGACGCTTC
AGATCCCAAATGAGCACCAGGACCTTCAGGATGTCGAGGAGCGGGTTGAC
TCCTTCAAGTCCTTTGCTAAGAAAATGGACGACAGTGTGATGCAGCTCAC
CCACGTTGCCTCCGAGCTGGTGCGTAAACACCTGGGTGGGTTCAGGAAGG
AGTTCCAGCGGCTGGGAAATGCCTTCCAGTCTATCAGCCAGGCGTTCATG
CTGGACCCTCCTCACAGCTCAGATGCCCTCAACAACGCCATCTCACAT--

-----------------------------------GCCAAATCTCGCTTT
CACCCTGGCGTGGGGACTGGTCCTGGCACGGAGC---GCAGCGTCCCACT
CGGCAACAGCTTGCTCTCCCCGCAGCAAACCGAGGAGCCCACTGTTGCCA
CCCCCCCGCAGCGATGGTTTGTCACCC---CTGCCAACAACCGACTGGAC
TTTGCTGCCTCGGCATACGACGCCGCT---------GATTTCGCCGGTAA
CGCGGCCACCTTGCTGTCCTACGCTGCGGCCGGAGTGAAGGCGC------
TTCCCCTGCCGACGGCGGGCTGCTCCAACCGGCCTCTTGGCTATTACGCA
GACCCGTCGG---GCTGG---GGAGGACGCACGCCGCCGCAGTACTGTGG
YGTCAACAGCAAATCCAGCTCGGTGTTTTCCTGCTGGCCCGCTAACTCGA
TCGGCGGCAGGCCTGGAG---CC---AACTACCTGG---------CCGAG
GA---GGGG---GACTC---CATCCCCACCGAGAGATCCCCG---AT---
CGGTGGCTCGGAGGAG---GCCAAACCCAAAGACATGAC---GTCCGA--
-GTCGAGCTGGATAGAG---ACGCCGTCCTCCATTAAGTCCATCGATTCG
AGCGATTCTGGTATCTTTG---AACAGGCCAAGAGGAGACGGATCTCACC
GTCTGCCACGCCG---------GTTTCAGAGACAGCRTCCCCGTTAAAGT
CTGAGCATCACTCAACAGGCGAAGTCACAGAGAGAGAAGTGGCGTTGGGG
ATAAATCCGTTCGCGGATGGGATGGGCGCCTTCAAAATAAACCACAGCTC
CCACGACATCGGCTCCGG---ACAGACGGCATTTTCCTCCCAGGCG---C
CCGGCTAC---GCAGCAGCCGCCCTGGGA---CACCATCA------CCAC
CCGACCCACGTTGGCTCT---TACTCCACGGCGGCTTTCAACTCCACCAG
GGACTTTCTCTTCAGAAATCGGGGTTTCGGGGACGCCACCGG--------
----GGCGCAGCACAGTTTGTTCGCCTC---------TGGAAGTTT---C
------GCAGGGCCACATGGACACTCAGATGCGGCGGGCCACCTGCTCTT
CCCGGGGCTCCACGAG---CAAGCGGCCGGCCATGCCTCTTCCAACGTGG
TCAACAGCCAGATGCGGCTGGGCTTCTCGGGGGACATGTACGGACGGGCC
GACCAGTACGGCCACGTTACGAGCCCGCGGT---CCGACCACTATGCTTC
GACCCAGCTGCACGGCTATGGCCCCATGAACATGAATATGGCCGCG---C
ACCACGGAGCAGGGGCCTTCTTTCGCTACATGAGGCAGCCGATCAAACAA
GAGCTCATCTGCAAGTGGATCGAGCCGGAGCAGCTGACAAACCCCAAAAA
GTCGTGCAACAAAACTTTTAGCACGATGCATGAGCTGGTGACCCATCTGA
CGGTGGAGCATGTGGGTGGACCGGAGCAGACCAACCACGTCTGTTTCTGG
GAGGACTGCTCCAGAGAAGGAAAGCCATTCAAAGCCAAATACAAACTTGT
AAATCATATCAGAGTACACACCGGAGAAAAGCCCTTTCCCTGTCCGTTCC
CCGGCTGTGGCAAA
>Elassoma zonatum
AGCTTGCTTATCCGAGCCGAGCTAAGCCAACCTGGGGCCCTTCTTGGGGA
CGACCAGATTTATAATGTAATTGTTACTGCACACGCCTTTGTTATAATCT
TCTTTATAGTAATACCAATCATAATCGGAGGTTTTGGTAATTGACTTGTT
CCTTTAATAATTGGGGCCCCTGACATGGCCTTCCCTCGTATGAATAATAT
GAGCTTTTGACTCCTGCCCCCATCTTTCTTACTTTTACTTGCTTCCTCCG

GAGTAGAAGCTGGAGCAGGTACAGGGTGAACCGTATATCCCCCTTTAGCT
GGGAATCTAGCCCATGCGGGGGCATCTGTTGACCTAACAATTTTTTCACT
CCATCTTGCAGGGGTTTCCTCAATTCTCGGGGCAATCAATTTTATCACAA
CTATTGTTAACATAAAACCCCCTGCTTTATCACAATATCAGACCCCTTTA
TTTGTATGATCTGTTTTAATTACAGCTGTACTACTTCTCTTATCCCTTCC
TGTGCTAGCTGCCGGCATTACAATACTACTAACAGACCGTAACCTCAATA
CTACCTTCTTCGATCCGGCCGGAGGGGGAGATCCGATTCTCTACCAACAC
CTG---

------------NNNNNNNNNNGAACCTTCACCCATCTAACTGCCTTGGTATGCTGCTGC
TGTCTGATGCCCACCAGTGCACCAAGCTGTCGGAGCTCTCCTGGGGCATG
TGCCTCAGCAACTTTCCTGCTATTTGCAAGACAGAGGACTTCCTCCAACT
GCCCAAAGACATGGTGGTGCAGCTTTTGTCACATGAGGAGCTTGAGACGG
AAGATGAAAGACTGGTTTATGAAGCTGCCCTTAACTGGATCAACTATGAC
TTGGAAAGGAGGCATTGTAGCCTTCCAGAGCTCCTGAGAACGGTCCGTCT
GGCCCTGCTGCCTGCCATCTTTCTCATGGAGAATGTATCTACAGAAGAGC
TGATCAACGCCCAGGCCAAGAGCAAGGAGCTGGTGGACGAAGCCATACGG
TGTAARCTGAAGATCCTGCAGAATGACGGCGTTGTTAACAGCCCGTGTGC
TCGACCAAGAAAAACCAGCCATGCCCTCTTCCTTCTGGGTGGGCAGACTT
TTATGTGTGACAAGTTGTACCTGGTAGACCAGAAAGCCAAAGAGATCATC
CCCAAAGCGGACATTCCCAGCCCCAGGAAAGAGTTCAGTGCCTGTGCCAT
TGGCTGTAAGGTGTACATCACAGGAGGGA--GGGGCTC-AGAGAATGGCG
TTTCCAAAGATGTATGGGTCTATGACACAGTCCACGAGGAATGGTCGAAA
GCGGCACCGATGCTCATTGCCAGGTTTGGCCATGGCTCCGCTGAGCTGAA
ACACTGCCTTTATGTGGTGGGAGGTCACACGGCAGCAACTGGCTGCCTCC
CGGCCTCTCCGTCTAGATCAATACATTGTTGTGTTTAGTCGTTCAACAAC
AAGACTGATGCTGAACCAAGCAGAGCTTATCATGATGCTATCCCAGGAAT
TCCAAATGAGAGTGGTCACGGTATCACTAGAGGAACAATCCTTCCCCAGC
ATCGTCCAGGTGATTAGCGGTGCATACATGTTAGTCAGTATGCATGGAGC
TCAGCTAATCACCTCACTCTTTCTGCCCAGAGGAGCTTCTGTGGTGGAGC
TTTACCCTTTTGCTGTTAACCCAGAGCAATACACTCCATATAAAACCCTT
GCCTCCCTTCCAGGTATGGACCTTCATTATATCTCCTGGAGGAACACTAG
AGAAGAGAACACCATCACCCACCCAGACAGACCCTGGGAACAAGGGGGCA
TAGCTCATTTGGAGAAGGAAGAGCAAGAGCGTATACTGGCTAGCAAAGAT
GTACCCAGACACTTGTGCTGCCGCAACCCAGAGTGGCTCTTCCGGATCTA
CCAGGACACTTTGGTTGATATCCCTTCCTTTTTAGAAGTCCTCAA---AG
AGGGAATGAAG---ACCAAACCCAGCTTTTTAAA---GTCAAAGCCAGCA
AGCACAGTCCATCCAGGTCGGGTCAGAGAACCTCAGTGTCAGACCTCAGT
ACAAACCACTAATGAGGCTAAGCTCACAGTCTCCTGGCAGATCCCATGGA
ATCTGAAATACCTAAAGGTTAGAGAGGTGAAGTATGAGGTGTGGATTCAG
AAGAAAGACAGCAGCAAAGGGACGCTGGAGGATCAGATCATTCAGGCGAA
TCCGGCGCTGGAGGCGTTTGGCAATGCAAAGACGTCCAGAAACGACAACT
CGTCCCGCTTTGGAAAATTCATAAGAATTCACTTCGGCACGAGCGGCAAG
CTGTCGTCCGCCGACATCGAGACATACCTGCTGGAGAAGTCACGAGTCAC
CTTCCAGCTGAAGGCGGAGAGGAACTACCACATATTCTACCAGATCCTGT
CAAACCAGAAGCCCGAGCTGCTGGACATGCTCCTGATCACAAACAACCCA
TACGACTACTCCTACATCTCACAGGGAGAGCTGACGGTCGCCTCCATCGA
CGACTCCGAGGAGCTGGTGGCCACAGACAGCGCGTTTGACGTGCTCGGCT
TCACTGCTGATGAAAAGATGGGCGTTTACAAGCTGACGGGCGCCATCATG
CACTACGGCAACATGAAGTTTAAGCAGAAGCAGCGCGAGGAGCAGGCGGA
GCCAGACGGGACGGAGGCGGGCGATAAATCAGCTTACCTAATGGGGCTGA

ACTCCGCCGACCTCATCAAAGGGCTGTGTCACCCGAGAGTCAAGGTTGGA
AACGAGTACGTGACCAAAGGGCAAAGTGTGGACCAAGTCTACTACCCTAA
CAAGGAGGCCTTCAAGTGTGAGGAGTGTGGGAAGCACTACAACACCAAGC
TGGGATACAAGCGGCACGTGGCCATGCACTCTGCAACGGCAGGGGATCTC
ACCTGTAAAGTTTGCATGCAGACCTATGAGAGCACACCCATACTCTTGGA
GCACCTCAAGAGCCACTCCGGGAAGTCTTCTGGTGGTGTCAAGGAGAAAA
AGCATCCGTGCGACCACTGTGACCGGCGTTTCTATACACGGAAGGATGTG
AGACGGCACATGGTGGTCCACACGGGCCGAAAGGACTTCCTGTGCCAGTA
CTGTGCCCAACGCTTTGGCAGGAAGGACCATCTGACACGCCACGTGAAGA
AAAGCCACTCGCAGGAGCTGCTGAAGATCAAGACGGAGCCTCCGGATATG
TTAGGTCTTTTAGCCTCTGGGTCTTCCCCGTGCTCTGTGAAGGAGGAGCT
CAGCCCTATGATGTGCGGAATGGGTCCCAACAAAGACCCAATGATGGGAA
AACCATTTCCCAGTGGAGCCCCTTTTCCGATGGGCATGTACAACCCCCAC
CAT------CTTCAGGCCATGTCTAATTCTGGGGTGGGTCACCCA-----
-CACCCGTCCCTGATGCCTAGTTCCTTATCTGCAGCTATTGGCATGGGCT
GTCACATGGACTATCTCATCTATGCATCATTCTCATTTATGGGATGTTTA
CAAATTAGTGATGGATCAAATATTGTTAACCTGCTGGCTAGTAATTCTCC
TAGTGTTTCTTACGCTCTGACCCAGCAGAAATACTTCAGTAATTACAGTC
CGGTAATTGGGTTTTACATTTACGAGCCCATTGAATACTGGAACTCCACA
GTGCAGGAGCATCTGAAGACTCTAAGTCATGGCTTCAACAAGATTTCATG
GATGGACAACTTTTTCCATTACCTGCGGGTGGTAAATGTGAGTGCATCTA
CCAAAAGCGACTTCATTGCCATTCTCAAAGGCTCCTTCCTGCAAAGCCCA
GAGTATCAGCACTTCACTGAGGACATCATATTCTCCAAAA---ACCGTGA
GACTG------ATGAGTACGACATTATTGCTTCACGAATGTACTTGGTGG
CAAGAACAACAGAGAAGAAGCGCGAAGAGGTGGTAGAGCTTCTGGAGAAG
CTTCGTCCATTGATGCTGATCAACAGCATCAAGTTCATTGCCTTCAATCC
TACGTTTGTGTTCATGGACCGGTACAGCTCCTCTGTCATCTCGCCCATCC
TGACCTCAGGCTTCAGCATACTCACAATCCTCATACTCACTTTCTTCTTA
GTTATCAACCCCTTGGGGAATTTCTGGCTCATCCTCACGGTAACGTCCGT
GGAGCTGGGGGTCTTGGGCTTGATGGGCTTTCACCAATTTGAATGGCAGC
CAGCTCTCAAGAATGTGTCTACATCATGCCATGTTGGCATTATTAATGGG
CTCTCCGGATGGGCCTCATCGGTGGATGACGCGCCTGCTGACACTATCAC
TCGGAGGTTTCGCTATGATGTGGCACTAGTGTCAGCATTAAAAGATCTGG
AGGAGGACATCGTGGAGGGGCTGAGAGAGAGTGGGATGGAAGACAGTGCT
TGCACCTCAGGCTTCAATGTCATGATCAAGGAATCTTGTGATGGCATGGG
TGATGTCAGCGAGAAGCACGGTGGAGGACCGATGGTTCCTGAGAAGGCTG
TGCGCTACTCATTCACCGTGATGCGTATCTCTATCCTGGCAGAYGATGAG
GAG------------------------------GAAGAAGTAACCATCTT
TACTGAGCCAAAGCCAAACTCAGAACTGTCCTGTAAGCCCCTTTGCCTAA
TGTTTGTGGATGAGTCAGACCATGAAACACTCACAGCTGTCCTGAGCCCC
ATCATTGCAGAGCGTAGTGCAATGAAAGAGAGCAGGCTCATTCTATCCAT
CGGTGGACTACCTCGGTCCTTCCGCTTCCACTTCAGAGGCACGGGATACG
ATGAGAAAATGGTGCGTGAGATGGAGGGCCTCGAGGCCTCGGGGTCCACC
TATATCTGCACTCTTTGCGACTCTACTCGGACCGAGGCCTCTCAAAACAT
GGTGCTACATTCCATCACGCGTAGTCACGACGAGAACCTAGAGCGTTACG
AAATATGGAGGACCAACCCCTTTTCTGAGTCTGTAGATGAGTTGCGAGAC
AGAGTCAAAGGTGTCTCTGCAAAGCCCTTTTTAGAGACCCAGCCCACACT
GGATGCATTACACTGTGACATTGGAAATGCCACTGAGTTCTACAAACTCT
TCCAGGATGAGATTGGAGAGGTTTACCAAAAGGT---CAAC---CCCAGC
CGGGAGGAACGGCGCAGCTGGAGGGCAGCCCTAGATAAACAGCTGAGGAA
GAAGTTGAAGCTTAAACCGGTGATGAGAATGAACGGGAACTACGCCCGCA
AGCTAATGACACAGGAGGCCGTGGAGGTGGTCTGTGAACTGGTGCCCTCA
GAGGAGAGGAGGGAAGCCCTGAGGGAGCTCATGAGGCTCTATATCCAGAT

GAAGCCTGTGTGGCGTGCCACCTGTCCGGCCAAGGAATGCCCCGACCAGC
TGTGYCGCTACAGCTTTAACTCCCAGAGCTTCGCAGACCTCCTCTCTTCT
ACATTCAAATACAGGTACAATGGAAAGATAACCAATTACCTCCACAAGAC
TTTGGCCCATGTGCCTGAAATCATAGAGAGAGATGGATCCATAGGAGCCT
GGGCTAGCGAGGGAAATGAGTCAGCAAACAAATCGTACACCATCGATATG
GGTCCCATGGGGCCCCGGTGGAAGGACAACCCGCAGCCTTTCTTCTGCTC
TATTGAAGATCCAACWAAACAGACAAAGTTCAAGGGCATCAAGACGTACA
TTTCGTACAGAGTGACGCCGAGTCACACGGGGCATCCYGTCTACAGGCGT
TACAAACACTTTGACTGGCTTTACAACCGTTTACTSCACAAGTTCACTGT
GATCTCTGTGCCTCACCTGCCTGAGAAGCAGGCCACGGGGCGATTTGAGG
AAGACTTCATCGAGAAGCGTAAGCGACGACTGATACTGTGGATGAACCAC
ATGACCAGTCACCCCGTTCTCTCCCAGTATGAAGGCTTTGAGCACTTTCT
GATGTGCGTCGATGACAAACAGTGGAAACTGGGCAAGAGGCGGGCAGAGA
AGGACGAGATGGTGGGTGCCCACTTCATGCTGACCCTTCAGATCCCTAAT
GAGCACCAAGACCTTCAGGATGTAGAGGAGCGGATCGACACCTTCAAGGC
GTTTGCCAAGAAAATGGATGACAGTGTAATGCAGCTCACACACGTTGCGT
CGGAGCTGGTGCGTAAACATCTGGGGGGGTTCAGGAAGGAGTTCCAGCGG
CTGGGAAACGCCTTCCAGTCCATCAGCCAGGCTTTCATGCTGGACCCTCC
CCACAGTTCAGACACCTTCAACAATGCCATCTCACATNNNNNNNNNNNGTTCCTCAAACTG
ACCTCTCTGGGCTTCATCATTGGAGTAGGTGTGGTTGGAAACCTCCTGAT
CTCCATCCTGCTGGTCAAAGACAAGAGCTTGCACCGAGCGCCCTACTACT
TCCTGCTGGACCTGTGCGCCTCCGATATCTTGCGCTCCGCCATCTGCTTC
CCCTTTGTCTTCACGTCCGTCAAGAATGGATCTGCCTGGACCTACGGCAC
ACTGACCTGCAAAGTGATCGCCTTCCTGGGTGTGCTCTCCTGCTTCCACA
CAGCGTTTATGCTGTTCTGCGTCAGTGTCACGCGCTACCTGGCCATCGCC
CATCACCGTTTCTACACCAAGAGGCTGACCTTCTGGACCTGTCTAGCTGT
CATCTGCATGGTGTGGACGTTGTCAGTGGCTATGGCGTTCCCGCCGGTGC
TCGACGTGGGGACATACTCTTTTATCCGGGAGGAGGACCAGTGCACATTC
CAGCACCGTTCCTTCAGGGCGAATGATTCACTGGGCTTCATGCTGCTGTT
GGCGCTCATCCTCCTGGCCACACAGCTGGTTTACCTCAAGCTCATCTTCT
TCGTCCACGACCGTCGAAAGATGAAGCCCGTCCAGTTTGTGCCTGCTGTC
AGCCAGAATTGGACCTTCCACGGGCCCGGCGCCAGCGGTCAGGCTGCGGC
CAACTGGCTCGCCGGATTTGGTCGAGGCCCCACCCCGCCTACTTTGCTGG
GCATTCGGCAAAACAGCAACGCAGCTGGCCGCAGGCGTCTGCTAGTATTG
GATGAATTTAAAACAGAGAAGAGGATTAGTAGGATGTTCTACATCATGAC
GTTTTTCTTCCTGGCACTGTGGGGACCCTATCTGGTTGCGTGCTACTGGC
GGGTGTTTGCAAGGGGTCCTGTGGTCCCTGGGGGCTACCTGACGGCAGCC
GTGTGGATGATCTTTGCCCAGGNNNNNNNNNNNNNNNNNNNNNNNNNNNNNNNNNNNNNNNNNNGCCAAAT
CTCGCTTTCACCCTGGCATGG
GGACTGGTCCTGGCACGGAGC---GCAGCGTCCCACTCGGCAACAGCTTG
CTATCCCCGCAGCAAAGCGAGGAGCCTACTGTTGCCACCCCCCCGCAGCG
ATGGTTTGTCACCC---CTGCCAACAACCGACTGGACTTTGCTGCCTCGG
CATACGACGCAGCT---------GATTTCGCCGGTAACGCGGCCACCTTG
CTGTCCTACGCAGCCGCCGGAGTGAAGGCTC------TTCCCCTGCCGAC
GGCAGGCTGCTCCAACCGGCCTCTTGGCTATTACGCAGACCCGTCAG---
GCTGG---GGAGGACGCACGCCGCCGCAGTACTGTGGCGTGAACAGCAAA
TCCAGCTCGGTCTTTTCCTGTTGGCCCGCTAACTCTATCGGCGGCAGAGC
GGGCA---CT---AACTACCTTG---------CTGAGGA---GGGA---G
ATTC---CATCCCGACAGAGAGGTCACCG---AT---CGGCGGCTCCGAC
GAG---ACCAAACCCAAAGACATAAC---GTCAGA---ATCGAGCTGGAT
AGAG---ACGCCGTCCTCGATAAAGTCAATTGATTCGAGCGATTCTGGAA
TCTTTG---AACAGGCCAAAAGGAGGAGAATCTCACCTTCTGCCACACCG
---------GTCTCAGAGACAGTGTCACCATTAAAATCTGAGCATCACTC

AACAGGCGAAGTCACAGAGAGAGAAGTGGCGTTGGGGATAAATCCGTTCG
CAGATGGGATGGGCGCCTTCAAAATAAACCACAGCTCCCACGATATTGGC
TCGGG---ACAAACTGCATTTTCCTCCCAGGCG---CCCGGCTAC---GC
AGCAGCCGCCCTGGGG---CACCATCA------CCATCCGACCCACGTTG
GCTCT---TACTCCACGGCGGCTTTCAACTCTACCAGGGACTTTCTCTTC
AGAAATCGGGGCTTCGGTGACGCCACCGG------------GGCGCAGCA
CAGTTTATTCGCCTC---------CGGAAGTTT---C------GCAGGGC
CACACGGACACTCCGATGCAGCGGGGCACCTGCTCTTYCCAGGGCTCCAC
GAG---CAAGCGGCGAGCCATGCCACTTCCAACGTGGTCAACAGCCAGAT
GCGTCTGGGCTTCTCGGGGGACATGTACGGACGGGCCGACCAGTACGGCC
ACGTTACAAGCCCGCGGT---CCGACCACTATGCTTCGACCCAGCTGCAT
GGCTATGGCCCCATGAACATGAATATGGCTGCG---CACCACGGAGCAGG
GGCCTTCTTTAGATACATGAGGCAGCCCATCAAACAAGAGCTTATCTGCA
AGTGGATCGAGCCGGAGCAGCTGACGAATCCCAAAAAAGCGTGCAACAAA
ACTTTTAGCACGATGCACGAGCTTGTGACCCATCTGACGGTGGAGCATGT
GGGGGGACCTGAGCAGACCAACCACATCTGCTTCTGGGAGGAATGCGCCA
GAGAAGGAAAACCATTCAAAGCCAAATACAAACTTGTAAATCACATCAGA
GTACACACCGGAGAAAAGCCCTTTCCGTGTCCGTTCCCCGGCTGTGGCAA
A
>Electrona antarctica
AGCCTCCTAATTCGAGCTGAACTCAGCCAGCCTGGGGCCCTCCTGGGGGA
CGACCAGATTTATAATGTAATCGTCACAGCTCATGCCTTTGTTATAATCT
TCTTTATAGTTATGCCTCTCATAATTGGGGGCTTCGGAAACTGACTAATC
CCCCTAATAATCGGGGCCCCCGACATGGCATTTCCCCGAATAAATAATAT
AAGCTTCTGACTCCTCCCTCCCTCCTTCCTCCTCCTTCTGGCTTCCTCCG
GCGTAGAAGCCGGGGCCGGAACCGGCTGAACAGTCTATCCCCCCCTTGCT
GGAAACCTCGCCCACGCCGGGGCCTCTGTTGACCTAACAATCTTCTCCCT
TCACCTAGCAGGCGTCTCCTCAATTTTAGGCGCAATTAACTTCATTACAA
CCATCATCAATATGAAGCCCCCCGGGATTACCCAGTACCAAACCCCCCTA
TTTGTCTGAGCCGTCCTCATTACAGCTGTACTTCTGCTCCTCTCACTCCC
TGTACTAGCTGCCGGTATCACTATACTTTTAACGGACCGGAACCTAAACA
CCACCTTCTTCGACCCCGCAGGCGGGGGAGACCCCATCCTCTACCAACAC
TTANNNNNNNNNNNNNNNNNNNNNNNNNNNNNNNNNNNNNNNNNNNNNNNNNNNNNNNNNNNNNNNNNNN
NNNNNNNNNNNNNNNNNNNNNNNNNNNNNNNNNNNNNNNNNNNNNNNNNNNNNNNNNNNNNNNNNNNNNN
NNNNNNNNNNNNNNNNNNNNNNNNNNNNNNNNNNNNNNNNNNNNNNNNNNNNNNNNNNNNNNNNNNNNNN
NN---

----------------GGATGAGTACATCGTAGTGTTCAGTCGTTCAACG
ACGAGACTCATTCTCAACGAAGCAGAACTGATTATGGCACTGTCCCAGGA

GTTTCAGATGAGGGTGGTCACTGTGTCCCTTGAAGAGCAATCTTTCCCCA
GTATAGTCCAGCTTATCAGTGGTGCCTCAATGTTGGTCAGTATGCATGGG
GCTCAGCTTGTCACCTCACTCTTCCTCCCCAGAGGAGCTGTGGTGGTGGA
ACTCTTTCCTTATGCTGTGAACCCAGAACAGTACACCCCTTATAAAACCC
TCGCCTCCTTACCGGGGATGGACCTGCAGTATGTGTCCTGGAGGAATATG
ATGGAGGAAAACACCATCACCCACCCAGATAGATCCTGGGACCAAGGAGG
CATCGCCCACCTGGAAAAGGACGAGCAAGAGCGAATCCTTGCCAGCAAGG
ATGTTCCGCGGCACCTATGCTGCCACAACCCAGAATGGTTCTTCCGAATC
TACCAGGACACTCTGGTGGACATCCCCTCATTTCTGGAGGTTCTCAA---
AGATGGCCTGAAGATCACCAGACCAAGCTTAAAAAA---GGCCAAGCCAG
CTAGCACAGTACATCCAGGCCGGGTCAGGGAAGCRCAGTGTCAAACCTCA
GTCCAAGCCACCAACGAAGCTAAACTCACKGTCTCCTGGCAGATCCCATG
GAACCTGAAATACCTGAAGGTGAGAGAGGTGAAGTACGAGGTGTGGATCC
AGAAGAAGGACACAAGCAAGGGAACCCTGGAGGATCAAATCATCCAGGCC
AACCCCGCGCTGGAGGCCTTCGGGAACGCCAAGACCATCCGGAACGACAA
CTCGTCCCGCTTCGGGAAGTTCATCCGCATCCACTTCGGGACAAGCGGAA
AGCTGTCGTCCGCCGACGTCGAGACCTACCTGCTGGAGAAGTCGCGCATC
ACCTTCCAGCTCAAGGCCGAGCGGAACTACCACATCTTCTTCCAGATCCT
GTCCCAAGAGAAGCCGGAGCTGCTGGAAATGCTGCTGATCACCAACAACC
CGTACGACTACTGCTACATCTCCCAGGGGGAGGTGACGGTGGCGTCCATC
AACGACAACGAGGAGCTGATCGCCACGGACAGCGCCTTCGACGTGCTCGG
CTTCACCCAGGAGGAGAAGATGTCCATCTACAAGCTGACCGGCGCCATCA
TGCACTACGGCAACATGAAGTTCAAGCAGAAGCAGCGCGAGGAGCAGGCC
GAGCCCGACGGCACMGAGGCCGCCGATAAGTCGGCGTACCTGATGGGGCT
GAACTCGGCCGACCTCATCAAAGGACTCTGCCATCCCCGAGTCAAGGTGG
GAAACGAGTTTGTCACGAAAGGCCAGGGCGTAGATCAAGTCTACTAC---

-------------TATCTCATCTACGCGTCATTCTCCTTCATGGGATGTT
TACAAATCAGCGACGGGTCAAACATTGTGAACCTGCTGGCAAGTAACTCT
CCGAGTGTGTCTTTTGCGCTCACCCAGCAGAAGTACTTCAGTAACTACAG
CCCTGTTATTGGGTTCTACATTTATGAACCCATCGAGTACTGGAATTCCA
CGGTGCAGGAGCACCTGAAGACACTGAGTCACGGCTTCAACAAGATCTCC
TGGATCGACAACTTCTTCCACTACCTGCGGGTGGTAAACGTGAGCGCGTC
GACCAAGAACGACTTCATCACCATCCTAAAGGACTCCTTCCTACACAGCC
CAGAGTACCAGCACTTCACCGAGGACATCATCTTCTCCAAGA---ACCGC
GAGAGCG------ACGAGTACGACATCATCGCCTCGCGCATGTACCTGGT
GGCGCGCACCACCGAGAAGAAACGCGAGGATGTGGTGTTGCTGCTGGAGA
CGCTGCGTCCGCTGATGCTGATCAACAGCATCAAGTTCATCGCCTTCAAC
CCCACCTTCGTCTTCATGGACCGCTACAGCTCCTCGGTCATATCGCCCAT
CCTTACCTCAGGCTTCAGCGTGCTCACCATCCTCATCCTCACCTTCTTTC
TCGTCATCAACCCACTGGGGAACTTCTGGCTCATCCTGACGGTCACGTCG

GTGGAGCTGGGCGTGTTGGGTCTGATG-----------------------
----------------------CCCCTCTTGGCATGTGGGCATCATCAACG
GGCTCTCTGGCTGGGCCGACTCGGTGGATGACTCACCAGCGGACACAATC
AGCCGGAGGTTTCGCTATGATGTGGCCCTGGTGTCTGCCTTGAAGGACCT
GGAAGAGGAAGTGATGGAGGGGCTGAGAGAACACGGGCTAGATGACAGTG
CTTGCACCTCAGGCTTCAGAGTAATGATCAAAGAGTCCTGCGATGGCATG
GGAGATGTCAATGAGAAACATGGCGGGGGCCCGCGATCCCTGAGAAAGC
GGTCCGTTTCTCTTTCACCGTTATGTCCATCTCTGTGATGCCGGATGGGA
CAACG-------------------------------GAGGCGTTGACCATC
TTCACGGAGCCGAAGCCCAACTCCGAACTGTCCTGTAAGCCCATGTGCCT
GATGTTTGTGGATGAGTCGGACCACGAGATGCTCACTGCCGTCTTGGGGC
CCGTGGTCGCAGAGCGCACGGCAATGACAGAAAGCCGACTCATCCTGTCC
ATGGGTGGCCTCCCCCGCTCGTTCCGCTTCTACTTCAGAGGCACAGGCTA
TGATGAGAAGATGGTGCGCGAGATGGAGGGCCTGGAGGCATCAGGCTCTA
CCTATGTCTGCACTCTGTGTGACTCCACCCGGAAGGAGGCCTCTCGCAAC
ATGGTGCTCCATTCCATCACCCGCAGCCACGGCGAGAACCTGGAGCGCTA
CGAGATATGGCGGCGCAACCCCTTTGCGGAGTCTGTCGACGAGTTGCGAG
ATCGGGTGAAGGGGGTCTCCGCCAAGCCCTTCATGGAAACCCAGCCCACT
CTGGATGCATTGCACTGTGACATTGGTAACGCCACTGAGTTCTACAAAAT
CTTCCAAGATGAGATCGGGGAGGTGTTCCAGAAAGC---CAAC---CCCA
GCCGTGAGGAAAGGCGCCTCTGGAGGGCTGCTCTCGACAAGCAGCTGAGG
AAAAAACTCAAGCTCAAGCCTGTGATGCGGATGAATGGGAACTACGCCCG
GAGACTGATGTCTGAGGAGACCATCGATGTGGTGTGTGAACTGGTACCCT
CAGAGGAAAGGCGGGAGGCTCTGAGGGAGCTAATGGGGCTTTACATCCAG
ATGAAACCTGTGTGGCGTGCCAGCAACCCAGCCAAAGAATGTCCTGACCA
ACTGTGCCGCTACAGTTTCAACTCCCAGCGCTTTGCAGACCTCCTCTCCA
CTACCTTCAAATACAGGTATGACGGAAAGATTACCAATTACCTGCACAAG
ACCTTGGCCCACGTACCTGAAATCATAGAAAGAGATGGCTCTATTGGAGC
ATGGGCCAGTGAGGGGAACGAGTCAGCAAACAAATCGTACACCATCGAGA
TGGGCCCCAAAGGGCCCCTGTGGAAGGAGAGCCCGCAGCCCTTCACCTGT
TCCATCGAGGACCCCACTAAACAGACCAAGTTCAAGGGCATCAAGACGTA
CATCTCCTACCGGGTCACGCCGACCCACACGGGTCATCCCGTGTACAGAC
GCTACAAGCACTTTGACTGGCTGTACAACCGCCTGCTGCACAAGTTCACC
GTGATCTCCGTCCCCCACCTGCCGGAGAAGCAGGCGACTGGGCGCTTCGA
GGAGGACTTCATAGAAAAGCGCAAGAGGCGGTTGATCCTCTGGATAAACC
ACATGACCAGTCACCCTGTCCTCTCCCAGTACGAGGGGTTCGAACACTTC
CTGATGTGTGGCGACGACAAGCAATGGAAGCTGGGCAAGCGGCGAGCAGA
GAAGGACGAAATGATCGGTGCCCACTTCATGCTGACCTTCCAGATCCCCA
ACGAGCACCAGGACCTGCAGGATGTGGAGGAGCGCGTAGACTCCTTCAAG
GCATTTGCCAAGAAGATGGACGACAGTGTCATGCAGCTGACACACGTGGC
CTCGGAACTCGTGCGCAAGCACCTTGGTGGGTTTCGCAAGGAGTTCCACC
GGCTGGGTAATGCCTTCCAGAACATCAGCCAGGGCTTCATGCTTGACCCG
CCACATAGCTCGGACGGCCTCAACAACGCCATCTCACACNNNNNNNNNNNNNNNNNNNNNACTGACCTCTC
TGGGTTTCATCATCGGCGTCGGCGTGGTTGGAAACCTCCTGATCTCCATC
CTGCTGGTCAAAGACAAAAGCCTCCACCGGGCGCCCTACTACTTCCTGCT
GGACCTGTGCGCCTCCGACATCCTCCGCTCCGCCATCTGCTTCCCCTTCG
TCTTCACCTCGGTCAAGAATGGATCCGCCTGGACCTACGGCACGCTGACC
TGCAAAGTGATCGCCTTCCTGGGCGTGCTCTCCTGTTTCCACACGGCGTT
CATGCTGTTCTGCGTGAGCGTCACCCGCTACCTGGCCATCGCGCACCACC
GTTTCTACACCAAGAGGCTGACCTTCTGGACCTGTCTGGCCGTCATCTGC
ATGGTGTGGACGTTGTCGGTGGCTATGGCGTTCCCGCCGGTGCTAGACGT
AGGGACGTACTCTTTTATCCGCGAGGAGGACCAGTGCACATTCCAGCACC
GCTCCTTCCGGGCGAACGATTCGCTGGGCTTCATGCTCCTGCTGGCGCTC

ATTCTCCTGGCCACACAGCTGGTTTACCTCAAGCTCATCTTCTTCGTCCA
CGACCGTCGAAAGATGAAGCCCGTCCAGTTCGTGCCTGCCGTCAGCCAGA
ACTGGACCTTCCACGGGCCGGGCGCCAGCGGGCAGGCGGCGGCCAACTGG
CTGGCCGGATTTGGTAGAGGCCCAACCCCGCCTACTTTATTGGGCATCCG
GCAGAACAGCAACGCGGCGGGTCGTAGGCGTCTACTGGTGTTGGATGAGT
TCAAAACAGAGAAGAGGATTAGTAGGATGTTCTACATCATGACATTTTTT
TTCCTGGCACTGTGGGGGCCCTACCTGGTAGCCTGCTACTGGCGGGTGTT
TGCAAGGGGCCCCGTAGTACCTGGGGGCTACCTGACGGCAGCCGTGTGGA
TGAGCTTTGCCCAGGCGGGGGTCAACTCCNNNNNNNNNNNNNNNNNNNNNNNNNNNGCCAAGTCCCGCTT
TCACCCT
GGCGTGGGGAGCGCTGCTGGCACGGAGCGCTGCAGCGTCCCACTCAGCAA
CAGCTTGCTCTCTCAGCAACAAACCGAGGATCCCGCCGTCG---CCTCCC
CGCAGCGATGGTTTGTCACCC---CTGCCAACAACCGACTGGACTTCGCA
GCCTCGGCATACGACGCCGCT---------GATTTCGCCGGTAACGCGGC
CACCTTGCTGTCGTACGCCGCGGCCGGAGTGAAGGCTC------TCCCCC
TGCCGACCGCAGCTTGCTCCAACAGACCTCTGGGTTATTACGCGGACCCG
TCGG---GCTGG---GGCGCGCGCACGCCTCCCCAGTACTGC--------
-AGCAAGTCGGCCTCGGTCCTTTCTTGCTGGCCCACGAATTCTGTCGGGA
GCAGAACGGGCA------CAAACTACCTAT---------TGGAGGA---C
GGATCGGACGC---CATTCCAACGGAGAGGTCCCCG---AT---TAATGG
CTCGGAGGAG---GCGAAAGCCAAAGACTT------GTCCGA---GTCTA
GCTGGATAGAG---ACGCCATCTTCGATCAAATCAATTGACTCAAGTGAT
TCTGGCATCTTTG---AGCAAGCAAAGCGGAGACGAATTTCACCGTCTGC
CACACCA---------GTTACAGAAACGGTGTCTCCGTTGAAATCTGAA-

>Eleotris pisonisA
AGCCTACTGATCCGCGCTGAACTAAGTCAACCTGGTGCACTACTGGGGGA
CGACCAAATCTACAATGTTATCGTTACGGCTCATGCCTTCGTAATGATTT
TCTTTATAGTAATGCCAATTATGATTGGGGGATTTGGAAACTGATTAATC
CCACTAATAATCGGTGCCCCCGACATGGCCTTCCCTCGAATAAATAATAT
GAGCTTCTGACTTCTCCCCCCCTTCCTTCCTCCTCCTCCTGGCATCCTCTG
GGGTTGAAGCGGGGGCAGGCACAGGGTGAACCGTCTACCCCCCTTTAGCA
GGGAACCTTGCCCACGCAGGGGCCTCTGTAGACCTAACCATCTTCTCACT
ACACCTAACAGGGGTATCCTCAATCCTGGGGGCAATCAACTTTATCACAA
CAATTATCAACATGAAACCCCCAGCAATCTCGCAATATCAGACCCCCTTG

TTTGTTTGAGCCGTTCTAATTACCGCTGTCCTACTACTACTCTCACTGCC
CGTGCTTGCCGCTGGCATTACAATGCTCCTAACAGATCGAAATCTAAATA
CGACTTTCTTTGATCCCGCGGGAGGAGGGGACCCAATCCTGTACCAACAT
CTTTTCTGATTCTTTGGGCACCCTGAAGTCTACATTCTTATTCTCCCGGG
CTTTGGAATAATCTCTCACATTGTTGCATACTATGCAGGCAAAAAAGAAC
CTTTCGGCTACATGGGAATGGTGTGAGCCATGATGGCTATCGGCCTCCTG
GGCTTTATTGTTTGAGCCCACCACATGTTCACAGTAGGCATGGATGTTGA
CACCCGAGCCTANNNNNNNNNGAGAAACCTCCATCCATCAAACTGTCTGGGCATGCTGC
TGCTGTCGGACGCTCACCAGTGCACCAAGCTCTCGGAGCTCTCCTGGGGC
ATGTGTCTCAGCAACTTTCCTGCAATCTGCAAGACTGAGGACTTCCTCCA
ACTGCCCAAAGACATGGTTGTGCAACTTTTATCTCATGAGGAGCTAGAAA
CGGAAGATGAGAGACTGGTTTATGAAGCTGCTCTCAACTGGGTCAACTAT
GACTTGGAACGGAGACACTGCCACCTTCCTGAACTCCTCAGAACAGTGCG
ACTTGCTTTGCTGCCAGCCATTTTTCTGATGGAGAATGTGTCAACAGAGG
AGCTGATCAATGCCCAGGTCAAGAGCAAAGAGCTGGTGGATGAGGCCATA
CGCTGTAAGCTGAAGATCCTGCAGAATGACGGGGTCGTCAATAGTCCCTG
TGCTCGACCCAGAAAAACCAGCCACGCCTTGTTTCTTTTAGGAGGGCAGA
CTTTCATGTGTGACAAGCTGTACCTGGTGGACCAAAAGGCCAAAGAGATT
ATCCCAAAGGCTGACATACCAAGTCCCAGAAAAGAGTTCAGTGCTTGCGC
CATTGGTTGTAAGGTGTATATAACGGGAGGAA--GGGGTTC-CGAGAATG
GTGTATCCAAGGACGTATGGGTTTATGATACTGTTCATGAGGAATGGTCC
AAAGCAGCACCTATGCTCATTGCCAGGTTTGGCCATGGTTCTGCGGAGCT
CAAACACTGCCTTTATGTTGTTGGAGGTCACACAGCAGCAACTGGCTGCNNNNNNNNNNNNNNNNNNG
GAGGAATACATTGTCGTTTTTAGCCGTTCCACTACTAGGCTTATATTGAA
CGAAGCAGAGTTGATAATGGCATTAGCGCAAGAGTTTCAGATGCGAGTGG
TTACGGTTTCCCTTGAAGACCAGTCCTTTGCTAGCATTGTCCAAGTTATA
AGTGGAGCCTTTATGCTAGTTAGCATGCATGGCGCACAGCTAATCACCTC
ACTATTTTTGCCTAGGGGTGCTGTAGTTGTTGAATTGTTTCCATTTGCAG
TCAACCCAGAACAGTATACCCCTTACAAAACATTGGCGTCCCTTCCTGGT
ATGGACTTGCACTATATACCATGGAGAAACTCAAATGAAGAGAATACAAT
CACCCATCCTGATAGGCCATGGGAGCAAGGCGGCATCAGTCACTTGGAAA
AATCAGAACAAGAACGGATATTGGCCAGCAAAGATGTCCCGCGACACTTG
TGTTGTCGTAACCCGGAGTGGCTTTTCCGAATTTACCAGGATACGTTAGT
GGATATACCATCATTTTTGGACGCGGTCAA---GGAAGGCATGAAA---A
CTAAACCAATTTTGAAAAA---GTCCAAAACGGCAACTTTGGTCCACCCA
GGACGCGTTCGCGAGGCGCAGTGTCAAACTTCAGTACAGACATCAAATGA
GGCGAAACTTACAGTGTCTTGGCAAATACCGTGGAATTTAAAGTTTTTGA
AAGTAAGAGAGGTCAAGTACGAAGTGNNNNNNNNNNAAGAAGGACCAGAACAAGGGTACT
CTGGAAGACCAGATTATCCAGGCCAACCCGGCCTTAGAGGCTTTCGGAAA
CGCCAAGACCGTTCGCAACGACAACTCGTCCCGGTTTGGGAAGTTTATCC
GGATCCACTTCGGGACGAGCGGGAAGCTGTCGTCTGCCGACATCGAGACC
TACCTCCTGGAGAAGTCCCGAGTCACGTTCCAGCTCAAAGCGGAACGGAA
CTACCACATCTTCTACCAGATCCTGTCCAATCAGAAGCCGGAGCTGCTGG
ACCTGCTGCTCATCACCAATAACCCGTATGATTACTCGTACATCTCTCAG
GGCGAGGTCACCGTCGCCTCCATCAACGACTCCGAGGAGCTCATCGCCAC
CGACAGCGCCTTCGACGTGCTCGGGTTCACTCCGGAGGAGAAGGTGGGCG
TCTACAAACTCACCGGGGCCATCATGCACCACGGAAACATGAAGTTCAAA
CAGAAACAACGTGAGGAACAGGCCGAACCCGACGGCACCGAGGCCGCCGA
TAAATCCGCCTTCCTCATGGGTCTGAACTCAGCCGATCTCATCAAAGGTC
TGTGCCACCCCCGGGTTAAAGTCGGGAATGAGTACGTGACCAAAGGCCAG
GGTGTGGACCAGGTGTACTACNNNNNNNNNNNNNNNNNCAAGTGTGAGGAGTGCGGGAAGCACTACA
ACACCAAGCTGGGATACAAGCGGCACGTGGCCATGCACTCGGCCACTGCG
GGCGACCTGACGTGTAAGGTGTGCCTACAGAGCTACGAGAGCACCCCGGT

GCTCCTCGAGCACCTCAAGAGCCACTCGGGGAAGTCCTCGGGTGGCACCA
AGGAGAAGAAGCACCCGTGCGACCACTGCGACCGCCGCTTCTACACCAGG
AAGGACGTGCGGCGCCACATGGTGGTGCACACGGGCAGGAAAGACTTCCT
GTGCCAGTACTGCGCCCAGCGCTTCGGCCGAAAGGACCACCTGACTCGCC
ACGTGAAGAAGAGCCACTCTCAGGAGCTGCTGAAGATCAANAGCGAGCCCC
CGGACATGTTGGGTCTGTTGGGGTCGGGGTCGCCGCCCTGCGCCATCAAA
GAGGAGCTGAGCCCCATGATGTGCGCCATGGGTCCCAACAAAGACCCTCT
GATGGGTAAGCCCTTCCCCGANGGGGCGCCCTTTCACCAGGGCATGTACAA
CCACCNNNNNNNNNNNNNNNNNNNNNNNNNNNNNNNNNNNNNNNNNNNNNNNNNNNNNNNNNNNNNNNNN
NNNNNNNNNNNNNNNNNNNNNNNNNNNNNN------------------
TATCTCATCTATGCCTCTTTTTCGTTC
ATGGGATGTTTACAAATCAGCGACGGATCCAACATTGTAAACTTGCTCGC
CAGTAACTCTCCGAGTGTTTCGTATGCACTGACCCAGCAGAAGTACTTCA
GCAACTACAGCCCAGTGATTGGATTTTACATTTACGAGCCCATCGAATAC
TGGAACTCTACCGTGCAGGAGCATCTCAAGACTCTGAGTCACGGATTTAA
TAAGATCTCTTGGATGGACAACTTTTTCCATTACCTCCGGGTGGTGAACG
TGAGCGCGTCCACCAAAAGCGACTTCATCAACATCCTGAAGGGCTCGTTC
CTGCGCAGCCCGGAGTACCAGCACTTCACCGAGGACATCATCTTCTCCAA
GA---ACCGCGAGACGG------ACGAGTACGACATCATCGCCTCACGCA
TGTACCTGGTGGCCCGGACCACGGAGAAGAAGCGCGAGGAGGTGGTGGAG
CTATTGGAAAAGCTTCGCCCGTTGATGTTGATTAACAGCATCAAGTTCAT
TGCCTTCAACCCCACTTTTGTGTTCATGGACCGCTACAGTTCCTCGGTCA
TCTCGCCCATTCTGACCTCAGGCTTCAGCGTGCTCACCATCCTCATCCTC
ACCTTCTTCCTGGTCATCAACCCCTTGGGAAACTTCTGGCTCATCCTGAC
GGTGACCTCCGTGGAGCTGGGCGTGCTGGGTTTGATGGGTTTCCATGAGT
TTGAATGGCAGCCAGCTCTCAAGAATGTGTCTCCATGCTGCAATGTAGGC
ATTATTAATGGCCTCTCCGGATGGACATCTTCACTGGATGATGCCCCGGT
TGACACAATCACCCGGCGATTTCGCTATGATGTGGCGCTGGTGTCAGCAC
TCAAAGATCTTGAGGAGGATATTATGGAAGGTCTGAGAGACAGTGGACTG
GATGACAGCTCGTGCACCTCTGGCTTCAATGTTATGATCAAGGAATCGTG
CGACGGGATGGGTGATGTCAGTGAAAAGCATGGCGGTGGGCCCATGGTTC
CAGAGAAAGCCGTTCGTTTCTCTTTTACGATAATGTCAGTGTCTTTGGTG
ACAGATGACAAGGAG------------------------------GGGGA
GGTCTCCATCTTCACTGAACCCAAACCAAACTCAGAGCTGTCCTGTAAGC
CCCTCTGCCTCATGTTTGTTGATGAATCTGACCACGAGACCCTTACTGCT
GTCTTGTGGCCTATTGTAGCAGAGCGTAATGCCATGAAAGAAAGCAGACT
GATTCTATCCATCGGCGGTCTATCACGGTCTTTCCGCTTCCACTTCAGGG
GGACAGGTTATGATGAGAAGATGGTGCGTGAGATGGAGGGACTTGAAGCC
TCAGGATCCACCTATGTCTGCACACTGTGTGACACCACTAGAGCGGAGGC
CTCTCAAAATATGGTTCTGCACGCCATCACCCGAAGTCATGATGAAAACC
TAGAACGGTATGAAATCTGGAGGACAAACCCTTTTTCTGAGTCTGCAAAT
GAGCTTCGAGACAGAGTAAAAGGTGTGTCAGCTAAACCCTTCATGGAGAC
CCAACCGACACTTGATGCATTGCATTGTGACATTGGCAATGCCACAGAGT
TCTACAAAATCTTTCAGGATGAGATTGGAGAGGTGTATGCAAGGGC---T
AAC---CCTAGCCGGGAAGAGCGGCGCAGCTGGAGGACGGCCTTGGATAA
GCAGCTGAGGAAGAAGCTGCGTCTCAAGCCGGTAATGAGGATGAATGGGA
ACTATGCCCGCCGGCTGATGACCCTGGAGGCTGTGGAGGCCGTGTGCGAG
CTGGTACCCACTGAGCAGCGGAGAGAGGCCCTGAGGGAGCTCATGAGGCT
TTACCTCCAGATGAAGCCTGTCTGGAGAGCCACCTGCCCGGCCAAGGAGT
GCCCCGACCAGCTGTGCCGCTACAGCTTCAATTCCCAAAGGTTTGCGGAC
CTCCTCTCCTCTACATTCAAATATAGGTACAATGGAAAGATAACCAATTA
CCTACACAAGACCCTGGCCCATGTCCCTGAGATTATCGAGAGGGATGGGT
CCATTGGGGCTTGGGCCAGCGAGGGGAATGAGTCTGCCAATAAATCCTAC

WCTATAGATATGGGACCCGTAGGCCCACGCTGGAAGGACAACCCGAATCC
CTTCATATGCTCTATCGAAGACCCAACAAAGCAGACCAAATTCAAGGGTA
TAAAGACTTACATATCATACCGGGTCACCCCAACCCACACGGGACGCCCG
GTTTATAGGCGCTACAAGCATTTCGACTGGCTCTACAACCGTCTTCTGAA
CAAGTTCACTGTAATCTCCGTCCCTCATTTGCCCGAAAAGCAAGCCACGG
GTCGGTTTGAGGAGGACTTTATCGAAAAACGCAAAAGACGGCTTATATTG
TGGATGAATCACATGACCAGTCACCCAGTTCTGGCCCAGTACGAGGGGTT
TGAACACTTTCTGATGTGCGCCGACGACAAACAGTGGAAACTAGGCAAGA
GGCGAGCGGAGAAGGACGAAATGGTGGGYGCYCACTTCATGCTCACCCTT
CAGATCCCAAACGAGCACCAGGATTTACAAGACGTGGAGGAGAGGGTGGA
CACCTTCAAGAAYTTCGCCCGGAAAATGGACGATAGCGTCTTGCAGCTCA
CGCATGTMGCTTCGGAGCTGGTTCGTAAACATCTGGGWGGATTTAGGAAA
GAGTTYCARCGTCTGGGTAATGCGTTTCAGTCCATAAGCCATTCGTTTTC
ATTGGACCCCCCTCACTGCTCGGACACCCTCAATAACGCCATCTCGCACNNNNNNNNNNNNNNNNNNNNN
C
TGACCTCCCTGGGTTTCATCATTGGCGTCGGCGTGGTCGGAAACCTCCTG
ATCTCCATCCTGCTGGTCAAAGACAAGAGCCTGCACCGAGCGCCCTACTA
TTTCCTATTGGACCTGTGTGCGTCTGACATCCTGCGCTCGGCTATTTGCT
TCCCTTTCGTCTTCACCTCTGTAAAGAATGGATCCGCCTGGACATACGGC
ACGCTAACCTGCAAAGTCATYGCCTTCCTGGGGGTGCTGTCCTGTTTCCA
CACAGCATTCATGTTGTTTTGTGTGAGCGTCACTCGCTACCTGGCCATAG
CACACCACCGCTTCTACACCAAGAGGCTGACCTTCTGGACCTGCCTGGCA
GTCATCTGTATGGTGTGGACGTTGTCGGTGGCTATGGCGTTCCCACCGGT
GCTAGACGTAGGGACGTACTCGTTTATCCGCGAGGAGGACCAGTGCACGT
TCCAGCATCGCTCATTCAGGGCCAACGACTCGCTGGGCTTTATGCTGCTG
CTGGCGCTCATCCTCCTAGCCACACAGCTGGTTTACCTCAAGCTCATTTT
CTTTGTACACGACCGCAGAAAAATGAAGCCGGTCCAGTTTGTGCCTGCCG
TCAGCCAGAACTGGACCTTTCATGGGCCAGGAGCCAGCGGGCAGGCAGCG
GCTAATTGGCTAGCCGGGTTTGGTCGCGGCCCCACCCCGCCCACTCTGCT
GGGCATTCGGCAGAACAGCAACGCGGCGGGCCGCAGACGTCTGCTGGTGC
TGGACGAGTTCAAAACGGAGAAGAGGATTAGTAGGATGTTCTACATCATG
ACGTTTTTCTTCCTGGCTCTGTGGGGGCCCTATCTGGTGGCCTGCTACTG
GAGGGTGTTCGCCAGGGGCCCAGTGGTCCCTTGGGGCTACCTGACCGCAG
CCGTGTGGATGAGCTTTGCCCAGGCTGGGGTCAATCCGTTCATCTGCNNNNNNNNNNNNNNNNNN---
----

-----------------CATCACTCAACAGGCGAAGTCACGGAGCGAGAA
GTAGCGCTGGGGATCAACCCGTTCGCTGATGGGATGGGCGCCTTCAAAAT
CAACCACAGCTCACACGACCTGGGGTCCGG---ACAGACCGCCTTCTCAT
CCCAGGCG---CCGGGTTAC---------GCCGCTTTAGGA---CACCAC
CA------CCACGCGACCCATGTTGGCTCT---TACTCCACA------TT

CAACTCCACACGGGACTTTCTCTTTAGAAATCGGGGGTTTGGAGACGCTG
CCGG------------GGCACAGCACAGTTTATTCGCGTC---------A
GGAAGTTT---C------CCCGGGCCACATGGACACTCTGAAGCGGCGGG
GCACCTGCTTTTCCCCGGGCTGCACGAA---CAAGCGT---------CGT
CGTCCAACGTTGTCAACAGTCAGATGCGCTTGGGCTTCTCCGGGGATGTG
TACGGACGGGCCGAGCAGTACGGCCATGTCACCAGCCCGAGGT---CCGA
CCACTACGCGTCCACACAACTGCACGGATACGGCCCCATGAACATGAATA
TGGCCGCGGCACACCATGGAGCGGGGGCCTTCTTTAGATACATGCGGCAG
CCCATCAAACAGGAGCTCATCTGTAAGTGGATTGAACCCGAGCAGCTAAG
CAACCCCAAGAAGTCGTGCAACAAAACTTTCAGCACCATGCACGAGCTGG
TCACCCATCTAACTGTGGAACACGTGGGGGGACCGGAGCAGACAAACCAC
ATCTGTTTCTGGGAGGAGTGCTCCAGAGAGGGGAAACCTTTTAAAGCCAA
ATACAAACTGGTCAATCATATCCGAGTGCACACAGGGGAGAAACCCTTCC
CGTGCCCGTTCCCCGGCTGTGGCAAA
>Elops saurus
AGCCTCCTGATCCGAGCCGAATTAAGCCAACCCGGGGCGCTTCTGGGAGA
CGACCAGATTTATAATGTCATCGTCACAGCACACGCCTTTGTAATAATCT
TCTTTATAGTAATGCCAATCATAATTGGTGGCTTTGGAAACTGACTGATC
CCTCTCATGATCGGAGCCCCTGACATGGCGTTTCCCCGAATAAATAATAT
AAGCTTCTGACTTCTACCACCCTCTTTCCTGCTGTTGTTGGCCTCTTCTG
GAGTGGAAGCAGGAGCGGGGACCGGATGAACCGTCTATCCGCCCCTGGCG
GGAAACCTCGCCCACGCGGGAGCATCCGTCGACCTAACCATCTTCTCCCT
CCACCTTGCAGGTGTGTCTTCTATCCTGGGTGCTATCAACTTTATTACTA
CAATTATTAACATGAAACCGCCAGCAATAACACAATACCAAACGCCACTA
TTCGTTTGAGCAGTACTGATCACCGCCGTTCTTCTCCTCCTATCGCTGCC
AGTGCTAGCTGCTGGCATCACAATGCTGCTAACAGACCGAAACCTGAACA
CAACCTTCTTTGACCCGGCAGGCGGAGGAGACCCAATCCTTTACCAACAC
CTATTCTGGTTCTTCGGGCACCCAGAAGTATACATTCTAATTCTCCCAGG
CTTCGGAATAATCTCACACATCGTAGCCTACTATGCAGGCAAAAAAGAAC
CATTCGGCTACATGGGCATAGTATGGGCAATGATGGCTATTGGTCTTCTT
GGCTTTATCGTATGGGCACACCACATGTTCACGGTAGGAATGGATGTAGA
CACACGTNNNNNTTCCTGGAGAGGAACCTGCACCCCACCAACTGCCTGGGCATGC
TGCTGCTGTCGGACGCGCACCAGTGCACCAAGCTGTCGGAGCTCTCCTGG
GGCATGTGCCTGAGCAACTTCCCCACCATCTGCAAGACGGAGGACTTTCT
GCAGCTGCCCAAGGACATGCTGGTGCAGCTCCTGTCCCACGAGGAGCTGG
AGACGGAGGACGAGAGGCTGGTGTACGAGTCGGCCCTCAACTGGGTCAAC
TACGACCTTGAGAGGAGGCACTGCTACCTCCCGGAGCTGCTGCGCACCGT
CCGCCTCGCCCTCCTGCCCGCCATCTTCCTCATGGAGAACGTCTCCACCG
AGGAGCTCATCAACAAGCAGGCCAAGAGCAAGGAGCTGGTGGACGAGGCC
ATCCGGTGCAAGCTGAAGATCCTGCAGAACGACGGCGTGGTCACAAGCCT
GTGCGCCCGGCCCAGGAAGACAAGCCACGCCCTCTTCCTCCTGGGCGGCC
AGACCTTCATGTGCGACAAGCTGTATCTGGTGGACCAGAAGGCCAAAGAG
ATCATCCCCAAAGCTGACATCCCCAGCCCCCGCAAGGAGTTCAGCGCCTG
CGCCATCGGCTGCAAGGTCTACGTGACGGGTGGCC--GAGGCTC-GGAGA
ACGGCGTGTCCAAAGACGTCTGGGTCTACGACACCTTGCACGAGGAGTGG
TGCAAGGCCGCGCCCATGCTCATCGCCCGGTTCGGTCACGGCTCGGCCGA
GCTGAAGCACTGCCTGTACGTGGTGGGCGGGCACACGGCCGCCACCGGCT
GCCTGCCGGCCTCGCCATCATGATGCGTACATTGTGGTTTTCAGCCGCTC
CATCAACAGGCTCATCCTGAACGAGGCGGAGCTAATTCTGGCACTGGCGC
AGGAGTTCCAGATGAGGGTGGTCACCGTGTCCCTGGAAGAGCACTCGCTG
GCGGACATCGTTCGGGTTGTTGGTGGGGCGTCCATGCTGGTCAGCATGCA
TGGGGCCCAGCTCATAACCTCCCTGTTTCTACCACGTGGCGCCGCTGTGG
TGGAGCTTTTCCCGTACGCTGTAAACCCAGAGCACTATGCTCCCTACAGG

ACACTGGCCTCGCTGCCGGGCATGGACCTGCAGTACGTGGTTTGGAGGAA
TACCATGGAGGAGAACTCTGTGGCCTTTCCCGAGCGCTCCTGGGACCAGG
GTGGCATTGCACACCTGGAGAAGGAGGAGCAGGAGCGCATCCTGAAGAGC
AAGGAGGTGCCGCGACACCTGTGCTGCCGGAACCCAGAGTGGCTTTTCCG
CATCTACCAGGACACCAAGGTGGACATCGCCTCCCTGCTGGACACTCTAC
G---TCAGGGGTTGACC---TCCAAGCCAAACCTCAAGAG---AGCCAGG
GCTATCAGCGCAGTCCATCCAGGCAGAGTGAGGGAGCCCAGGTGCCAGAC
CTCTGTCCAAGCCGCCAATGAGGCCAAGCTGACTGTGTCCTGGCAGATCC
CCTGGAACCTCAAATACCTGAAGGTAAGGGAGGTGAAATATGAGGTGTGG
ATTCAGAAGAAGGACACCAGCAAGGGAACACTGGAGGATCAAATCATCCA
AGCCAACCCAGCGCTGGAGGCTTTTGGCAATGCAAAGACCGTAAGGAATG
ACAACTCCTCACGTTTCGGGAAATTCATCCGCATTCATTTTGGAGTGAGT
GGTAAACTGTCCTCCGCTGACATCGAGACCTACCTGCTGGAGAAATCCCG
AGTCACCTTTCAGCTCAAAGCTGAGAGGAACTACCATATCTTTTATCAGA
TTTTATCCAACAAGAAGCCAGAGTTGCTGGACATGTTGCTTATTACCAAC
AACCCATATGATTATGCTTACGTCTCCCAAGGAGAGGTAACAGTTGCATC
AATTGATGATACTGAGGAGCTCATTGCCACAGACAGTGCCTTTGATGTGC
TGGGATTCACGGCAGAGGAGAAGATGGGTGTCTACAAGCTGACAGGTGCC
ATCATGCACTATGGTAACATGAAGTTCAAGCAGAAACAGCGTGAGGAGCA
GGCAGAGCCTGATGGTACTGAGTCTGCTGATAAGTCAGCCTACCTAATGG
GGCTAAACTCAGCTGACCTTGTCAAAGGACTGTGTCACCCAAGAGTCAAA
GTTGGCAATGAGTATGTCACCAAGGGCCAAAGTGTGGACCAAGTTTACTA
CCCGAACAAGGAGGCCTTCAAGTGCGAGGAGTGCGGCAAGAACTACAACA
CCAAGCTGGGCTACAAGCGTCACGTGGCCATGCATGCGGCCACCAGTGGC
GACCTCACCTGCAAGGTGTGTCTACAGAGCTACGAGAGCACGCCAGTGCT
GCTGGAGCACCTCAAGAGCCACTCGGGGAAGTCCTCGGGCGGGGCTAAGG
AGAAGAAGCACCCCTGCGACCACTGCGACCGCCGCTTCTACACGCGAAAG
GACGTGCGCCGCCACATGGTGGTGCACACTGGCCGCAAGGACTTCTTGTG
CCAGTACTGCGCCCAGCGCTTTGGCCGCAAGGACCACCTGACCCGGCACG
TGAAGAAGAGCCACTCCCAGGAGTTGCTGAAGATCAAGACGGAGCCGCCT
GACATGCTGGGCCTGCTGGGGTCTGGCTCTCCACCCTGCCCTGTGAAGGA
GGAGCTTAGCCCTATGATGTG---CATGGGTCCCTCCAAGGACTCCCTGA
TGGGCAAGCCCTTCCCCGGAG------CCTTCCCCATGGGTATGTACAAC
CCCCAC---------CTGCAGGCCATGTCCAACTCTGGGGTGTCC-----
-------CACCACTCCCTGGTGTCCGGTTCACTGTCTGCAGCCATGGGCA
TGGGGTGCCACATGGATTACCTAATTTATGCGTCATTTTCGTTCATGGGA
TGTTTACAAATTAGTGATGGATCAAACATAGTCAACCTTTTAGCCAGCAA
CTCTCCGAGCGTCTCCTATGCTCTGACCCAGCAAAAATATTTCAGCAACT
ACAGTCCTGTAATTGGGTTCTATATTTATGAGCCCATAGAGTACTGGAAT
TCCACTGTTCAGGAGCACCTGAAGACGCTGGGCCACGGATTCAATAAGAT
TTCATGGATCGACAACTACTTTCAGTATCTGAGGGTGGTGAATGTGAGCG
CATCAACCAAAAGCGAATTCATAACTATCCTCCAGACTTCTTTTTTAAGG
AGCCCAGAATACCAGCACTTCAAGGATGACATCATTTTTTACAAAA---T
GAGGG------------ACGAGACAGAGATCATCGCATCTAGGATGTACC
TGGTGGCCAGAACCACAGAGAAGACGAGGGAGGAGGTCGTGGAGTTACTG
GAGAGACTGAGACCTCTGTCTCTTATCAACAGCATCAAGTTCATCGTCTT
CAACCCTACCTTCGTCTTCATGGATCGATACAGCTCCTCCGTCATTTCYC
CYATTCTGACCTCTGGGTTCAGCGTCCTGATTGTTCTGATCCTGACATTC
TTCCTGGTCATCAATCCCCTTGGAAACTTCTGGTTGATTCTGACGGTCAC
CTCAGTGGAGCTGGGAGTTCTTGGCCTCATG-------------------

---A
AGGCAGTGCGGTTCTCCTTCACCATCATGTCAATCTCGGTCCAGCTGGCG
GACGGGGGC------------------------------AAGCAGGTGAC
CGTGTTCCAGGAGCCGAAACCCAACTCCGAGCTGAGCTGCAAGCCGCTGT
GCCTGATGTTTGTTGACGAGTCAGACCACGAGACGCTAACCGCTGTGCTG
TGGCCCGTGGTGGCCGAGCGGAACGCCATGAAGGAGAGCCGGCTCATCCT
CTCCATCGGGGGGCTGCAGCGGTCCTTCCGCTTCCACTTCCGGGGCACGG
GCTACGACGAGAAGATGGTGAGAGAGATGGAGGGCCTGGAGGCCTCAGGC
TCCACCTACATCTGCACGCTGTGTGACTCCACCAGGGCTGAGGCTTCCCA
GAACATGGTCCTTCACTCCATCACCAGGAGCCACGATGAAAACCTGGAGC
GGTACGAGATCTGGAGGACCAACCCCTACTCGGAGTCTGCCGAGGAGCTA
CGTGACCGCGTCAAGGGCGTGTCGGCCAAGCCCTTCATGGAGACCCAGCC
CACCCTGGACGCGCTGCACTGCGACATCGGCAACGCCACTGAGTTCTACA
AGATCTTCCAGGACGAGATTGGTGAGGTTCACCTGAATGG---CAAC---
CCCAGCCGCGAGGAGCGCCGCCGCTGGAGGTCGGCCCTCGACAAGCAGCT
GAGGAAGAGGATGAAGCTGAAGCCGGTGATGCGGATGAACGGGAACTACG
CCCGCCGGCTCATGACCCGGGAGGCGGTGGAGGCAGTGTGTGAGCTGGTG
CCATCGGAGGAGCGCAGGCAGGCCCTGAAGGAGCTGATGGAGCTCTACCT
GCAGATGAAG--

NNNNNNNNNNNNNNNNNNNNNNNNNNNNNNNNCCCAGTGGAAGG
AGAGCCCTCAGCCCTTCTCCTGCTCCATCGAAGATCCCACGAAACAGACC
AAGTTCAAGGGCATCAAGACGTACATTTCGTACCGTGTGACGCCCAGCCA
CACCGGCCGGCCCGTGTACCGCCGCTACAAGCACTTCGACTGGCTCTACA
ACCAGCTGCTGCACAAGTTCACCGTTATCTCTGTGCCCCACCTGCCTGAG
AAGCAGGCCACCGGGCGCTTCGAGGAGGACTTCATCGAGAAGCGGAAGAG
GCGGCTGATACTCTGGATGGACCACATGACCAGCCACCCGGTGCTGTCGC
AGTACGAGGGCTTCGAGCACTTCCTCATGTGTGCGGACGACAAGCAGTGG
AAGCTGGGTAAGCGGCGGGCAGAGAAGGATGAGATGGTGGGCGCCCACTT
CTTGCTCACCTTCCAGATCCCCAACGAGCACCAGGACCTGCAGGACGTGG
AGGAGCGTGTAGACTCCTTCAAGGCCTTCACCCGGAAGATGGATGAGAGC
GTCATGCAGCTGACGCACGTCGCCTCCGAGCTGGTGCGCAAGCACCTGGG
CGGCTTCCGCAAGGAGTTCCAGAGGCTGGGCAACGCCTTCCAGTCCATCA
GCCAGGCCTTCACCATGGACCCGCCCCACTGCTCGGACGCCCTGAACAAT
GCCATATCACACCCACTGGCCACGTTCCTCAAACTGACATCCCTGGGTTT
CATCATCGGAGTCGGAGTGGTCGGCAATCTCCTGATCTCCATCCTGCTTG
TCAAAGACAAAAGCTTGCACAGAGCCCCCTACTACTTCCTGCTGGACCTT
TGTGCCTCTGACATACTCCGATCTGCCATTTGTTTCCCTTTTGTTTTCAC
CTCGGTCAAAAATGGGTCAACGTGGACGTACGGCACGCTGACCTGCAAGG
TAATCGCTTTCCTGGGGGTGCTGTCGTGCTTTCACACCGCGTTTATGCTC
TTCTGCATCAGCGTCACCCGCTACCTGGCCATCGCCCACCACCGCTTCTA
CACCAAGAGGCTGACATTCTGGACCTGTCTGGCTGTCATTTGCATGGTGT
GGACGTTGTCAGTGGCCATGGCCTTTCCCCCAGTTCTGGATGTAGGGACC
TACTCCTTCATTAGGGAAGAGGACCAGTGCACCTTCCAGCACCGCTCCTT
CCGAGCCAACGACTCGCTGGGGTTCATGCTGCTCCTGGCCCTCATCCTCC
TCGCCACCCAGCTTGTCTACCTCAAGCTAATCTTCTTCGTCCACGACCGC
AGGAAGATGAAGCCGGTTCAGTTCGTGCCAGCCGTCAGCCAGAACTGGAC
CTTCCACGGCCCCGGGGCCAGTGGCCAGGCGGCCGCCAACTGGCTGGCGG

GTTTTGGCCGGGGCCCGACGCCGCCCACCCTGCTGGGGATCCGTCAGAAC
ACTAACACGGCGGGCCGGAGACGACTGCTAGTGCTGGACGAGTTTAAAAC
CGAGAAAAGGATAAGCAGGATGTTCTACATCATGACCTTCTTCTTCCTGA
CCTTGTGGGGGCCCTATCTGGTGGCCTGCTACTGGAGGGTGTTCGCCAGG
GGCCCAGTGGTGCCGGGAGGATACCTAACCGCCGCCGTCTGGATGAGTTT
TGCTCAGGCCGGAGTCAATCCATTCATCTGCANNNNNNNNNNNNNNNNNNGCCAAATCACGCTTCCAC
CCTGGCGCAGGGAACGGGTCTGGCACGGACC---GCAGCGTCCCACTTAG
CAACAGCTTGCTGTCCCCACAACAAGCCGAGGAGGTCGCGGCGG---CTT
CCCCGCAACGATGGTTTGTCACCT---CAGCCAACAATCGACTGGACTTT
GCAGCCTCGGCTTACGATGCAGCAGCAGCCGCTGATTTTGCCGGTAACGC
GGCCACATTGCTGTCTTATGCGGCCGCTGGGGTCAAGGCAC------TTC
CACTGYCAACAGCAGGGTGCTCAGGTAGACCACTAGGTTATTACCCGGAC
CCCTCGG---GGTGG---GGCACGCGCACTCCTCCGCAGTATTGC-----
----AGTAAGTCCAGCTCTGTCCTGTCCTGCTGGCCCTCCAAT------G
GGAGCAGGACTGGCG---CCTCGAACTACCTGC---------TAGATGA-
--ATCG---GACAC---CCTCTCAACTGAAAGATCTTCG---CT---TGG
TGGGTCTGAAGAC---GCCAAACCCAAAGAACT------GTCGGA---AT
CAAACTGGATAGAG---ACACCTTCTTCCATAAAGTCAATTGATTCCAGC
GATTCTGGAATTTTTG---AGCAGGCCAAACGGAGAAGAATATCACCTCC
CCCTACGCCG---------GTTTCGGAAACGGCTTCTCCGCTGAAAAGTG
AA---------ACCGGCGAAGTCACAGACAGAGAAGTGGCTTTGGGGATA
AATCCCTTTGCTGATGGCATGGGCGCTTTTAAAATCAACCACAGCACCCA
CGACCTGGGCTCTGG---ACAAACAGCCTTTTCCTCGCAAGCA---CCAG
GCTAC------GCCGCTGCCCTGGGA---CACCACCA------CCACCCC
ACCCATGTCAGCTCC---TATTCCACAGCGGCGTTCAATTCCACCCGGGA
CTTTCTGTTTAGAAATCGGGGCTTCGGAGACGCCACCAG-----------
-TGCACAGCACAGTCTTTTCGCCTCCGC---AGCAGGAAGTTT---T---
---GCAGGGCCACATGGACACTCGGATGCCACGGGGCACCTGCTTTTCCC
GGGACTTCACGAG---CAGGCAGCCACTCACGCGTCTTCCAATGTTGTAA
ATAGTCAGATGCGTTTGGGCTTTTCGGGGGACATGTATGGTAGAGCCGAG
CAGTACGGCCATGTAACTAGCCCCAGGT---CCGATCATTATGCTTCGAC
CCAGTTGCACGGCTATGGCCCCATGAACATGAAYATGGCTGCA---CACC
ATGGGGCAGGGGCCTTCTTTCGTTACATGAGGCAACCGATAAAGCAGGAG
CTCATCTGCAAATGGATCGAACCGGAACAATTGACGAACCCGAAAAAGTC
TTGCAACAAAACTTTCAGTACCATGCACGAGCTGGTTACGCACCTCACAG
TGGAGCATGTTGGGGGACCAGAACAATCGAATCACATCTGCTTCTGGGAA
GAGTGTCCCCGGGAAGGAAAACCTTTTAAAGCCAAGTATAAACTTGTAAA
TCACATCAGAGTACATACCGGCGAGAAACCCTTCCCGTGCCCTTTTCCTG
GCTGTGGCAAA
>Esox lucius
AGCCTTTTAATCCGGGCCGAACTAAGCCAGCCAGGGGCTCTCTTAGGTGA
CGACCAGATTTATAATGTTATCGTTACAGCCCATGCCTTTGTTATAATCT
TTTTTATAGTTATACCCGTTATAATTGGGGGTTTTGGAAACTGATTAATT
CCCCTAATGATTGGTGCCCCCGACATGGCCTTCCCCCGCATAAATAATAT
AAGCTTCTGACTTCTCCCCCCCTCCTTTTTACTTCTCTTAGCCTCCTCAG
GTGTTGAAGCTGGTGCTGGTACTGGCTGAACAGTTTATCCGCCTTTGGCC
GGAAACTTAGCACACGCAGGTGCTTCTGTAGACTTAACTATTTTCTCTCT
CCACCTGGCCGGAATTTCTTCTATTCTAGGAGCTATTAATTTTATTACCA
CAATTATCAACATAAAACCCCCCGCCATCTCACAATATCAGACACCATTA
TTTGTTTGAGCAGTCCTGATTACAGCTGTACTTCTACTTCTGTCTCTCCC
AGTCCTAGCCGCTGGAATTACCATATTGCTCACAGACCGAAATTTAAACA
CCACATTCTTTGACCCCGCTGGTGGTGGAGACCCTATTCTATACCAACAC
CTCNNNNNNNNNNNNNNNNNNNNNNNNNNNNNNNNNNNNNNNNNNNNNNNNNNNNNNNNNNNNN

NNNNNNNNNNNNNNNNNNNNNNNNNNNNNNNNNNNNNNNNNNNNNNNNNNNNNNNNNNNNNNNNNNNNNNNNN
NNNNNNNNNNNNNNNNNNNNNNNNNNNNNNNNNNNNNNNNNNNNNNNNNNNNNNNNNNNNNNNNNNNNNNNNN
NNTTCCTGGAGAGGAACCTGCACCCCTCCAACTGCCTGGGCATGCTGCT
CCTGTCCGACGCCCACCAGTGCACCAAACTGTCTGAGCTCTCCTGGGGTA
TGTGCCTTGGCAACTTCCCTGCTATCTGCAAGACAGAGGAATTCCTGCAG
CTTCCCAAAGACATGGCGGTGCAGCTGTTGTCCCATGAGGAGCTGGAGAC
GGAGGACGAGAGGCTGGTTTACGAGGCCGCCCTCAACTGGGTCAACTACG
ACCTGGAGAGGAGGCACTGCCACCTGCCGGAGCTGCTGCAGACCGTCCGC
CTGGCCCTCCTTCCCGCCATCTTCCTCATGGAGAACGTGTCCACAGAGGA
GCTCATCAACGCCCAGCTGAAGAGCAAGGAGCTGGTGGACGAGGCTATCC
GCTGCAAGCTGAAGATACTTCAGAACGACGGTGTGGTGAACAGCCCCTGC
GCCCGGCCCAGGAAGACCAGCCACGCCCTGTTCCTCCTGGGAGGTCAGAC
CTTCATGTGTGACAAGCTGTACCTGGTGGACCAGAAGGCCAAGGAGATAA
TCCCCAAAGCAGACATCCCTAGCCCCAGGAAGGAGTTCAGCGCTTGCGCC
ATTGGCTGTAAGGTCTATGTGACCGGTGGGA--GGGGCTC-CGAGAACGG
CGTGTCCAAAGACGTGTGGGTCTACGACACCGTGCACGAGGAGTGGTCCA
AGGCGGCACCCATGCTTATCGCCCGCTTCGGCCACGGCTCGGCTGAGCTG
AAACACTGCCTCTATGTGGTCGGAGGGCACACCGCGGGTACAGGCTGCCT
CCCTGCCTCTCCCTCGGGACGAGTACATTGTAGTGTTCAGTCGCTCAATC
AACAGACTCATCTTGAACGAAGCGGAGCTGATCATGGCGTTGTCGCAGGA
GTTTCAGATGAGAACCGTAACAGTGTCTCTGGAAGAGCAAACCTTCCCCA
GCATTGTCAAAGTCATCAGCGGGGCGTCCATGTTGGTCAGCATGCACGGG
GCACAGCTGGTCTCTTCTCTCTTCCTCCCCCGGGGGGCTGTTGTAGTGGA
ACTATTCCCTTACGCAGTCAACCCGGAGCAGTACACCCCTTACAAAACCC
TGGCCTCTCTACCAGGCATGGAACTGCAGTATGTGGCCTGGAGGAACATG
GTGGAGGAGAACTCTGTGGCCTACCCTGAGAGGCCCTGGGAGCAGGGAGG
TATTGCTCATCTGGAGAAAGATGAGCAGGAGCGCATCTTGGCCAGTAAGG
AGGTACCCAGACACCTTTGCTGCCGCAATCCCGAGTGGCTCTACCGTATC
TACCAGGACACTATAGTGGACATCCCTTCTTTACTGGAGGCTCTCAG---
AGAGGCTGTGAAA---ATCAGGCCAAATTTGAAGAA---GGCCAAGCCGG
CCAGCATGGTTCACCCAGGCCGGGTCAGAGAGCCTCTGTGCCAGACGTCA
GTCCAGGCCACCAACGAGGCCAAGCTGACCGTGTCGTGGCAAATCCCCTG
GAACCTCAAGTACCTGAAGGTCAGGGAAGTGAAGTATGAAGTGTGGATCC
AGAAGAGGGATCCCAGCAAGGGGACCCTAGAAGATCAAATCATCCAGGCC
AACCCTGCACTGGAGGCTTTTGGTAATGCCAAAACAGTTAGAAATGACAA
CTCATCACGTTTTGGCAAATTCATCCGTATTCACTTTGGAACAAGTGGAA
AACTGTCCTCTGCTGATATTGAGACTTACCTTCTTGAGAAGTCCCGTATC
ACCTTTCAGCTCAAAGCCGAGAGGAACTACCATATTTTCTTCCAGATATT
GTCCAACCAAAAGCCAGAGCTGCTGGACATGCTGTTAATCACCAATAACC
CATATGACTACTCCTACATCTCTCAAGGAGAGGTAACAGTAGCATCCATC
AATGATTCTGATGAACTGTTGGCTACTGACAGCGCCTTTGATGTACTCGG
CTTTACTGCAGAGGAGAAACAGGGGGTCTACAAGTTGACAGGTGCCATCA
TGCACTATGGCAACATGAAGTTCAAACAAAAGCAGCGGGAGGAGCAGGCA
GAGCCTGACGGTACAGAAGCTGCTGACAAGTCAGCATACCTAATGGGCAT
TAACTCTGCAGATCTCATTAAAGGACTTTGTCATCCCAGAGTCAAGGTTG
GCAATGAGTATGTTACCAAAGGTCAAGGAGTAGATCAGGTCTACTACCCC
AACAAGGAGGCCTTCAAGTGCGAGGAGTGTGGCAAGCACTACAACACCAA
GCTGGGCTACAAGCGCCACGTGGCCATGCACTCGGCCACGGCCGGCGATC
TCACCTGCAAGGTGTGTCTGCAGAGCTACGAGAGCACGCCGGCCCTGCTG
GAGCACCTGAAGAGCCACTCGGGCAAGTCATCCGGTGGCGCCAAGGAGAA
GAAGCACCCGTGCGACCACTGTGACCGGCGCTTCTACACCCGGAAGGACG
TGCGCCGCCACATGGTCGTGCACACCGGCCGCAAGGACTTCCTGTGCCAG
TACTGCGCCCAGCGCTTCGGCCGGAAGGATCACCTCACACGTCACGTGAA

GAAGAGCCACTCGCAGGAGCTGCTGAAGATCAAGACGGAGCCCCCGGACC
TGCTGGGCCTGCTGGGCTCCGGCTCGCCTGCCTGCACCGTCAAGGAGGAG
CTCAACCCCGTGATGTGCAGCATGGGTCCCAGCAAGGACACCGTGATGAA
CAAACCCTTCCCCGGCGGCGGCCCCTTCCCCATGGGCATGTACAACGCCC
ACCCG------CTTCAGGCCATGCCCGGCCCAGGCGGGGGGCAC------
---CACCACCTCCTGATGCCCGGCCCCCTCTCCGCCGCCATGGGGATGGG
CTGTCACGTGGAGTACCTGATATATGCCTCCTTCTCCTTCATGGGATGTT
TACAGATCAGCGACGGTTCCAACATTGTCAATCTCCTCGCCAGCAACACG
CCCAGCGTCTCCTACGCCATGACACAGCAGAAGTACTTCAGTAACTACAG
CCCGGTGATCGGCTTCTACATCTACGAGCCCATAGAGTACTGGAACTCCA
CGGTGCAGGAGCACCTGACCACTCTGAGCCACGGCTTCAACAAGATCTCC
TGGATGGATAACTACTTTCAGTATCTGAAGGTGGTGAATGTGAGTGCCTC
TACCAAAAGTGATTTCATCGCTATCCTGCAGGGATCCTTCCTGCGCAGCC
CAGAGTATCAGCACTTCATGGAGGACATCATTCTGTCCAAGACGGACGGC
G------------ACGAGATGGAGATCATCGCGTCGCGGATGTATCTGGT
GGCACGGACCACAGAGAAGACAAGGGAGGAGGTGGTTGAGCTGCTGGAGA
GGCTCCGCCCCCTCTCGCTCATCAACAACATCAAGTTCATCGTCTTCAAC
CCCACCTTCGTCTTCATGGACCGCTACAGTTCCTCCGTGGTCTCGCCCAT
CATCACGTCGGCCTTCAGCGTGCTCACCATCCTCGTCCTCACCTTCTTTC
TGGTCATCAACCCCCTGGGGAACTTCTGGTTGATCCTGACCGTCACCTCC
GTGGAGCTGGGGGTTCTGGGTCTCATG-----------------------

---CCATC
CCTCGTCGGTTCCGATATGATGTGGCCCTGGTGTCGGCCCTAAAGGACCT
TGAAGAGGATATTATGGAGGGGCCGAGGGAGCGAGGCCTGGAGGACAGTG
TCTGTACCGCAGGCTTCAGTGTGGTGATTAAGGAGTCCTGCGACGGCATG
GGGGACGTGAGCGAGAAGCATGGTGGAGGCCGGGCCATCCCGGAAAAAGC
AGTGCGTTTCTCTTTCACCATCATGTCCGTCTCCCTCCAGGCGGAGGGAG
AGGAC------------------------------GAGGCGGTCAACATC
TTCCAGGAGTCCAAGCCGAACTCAGAGATGTCCTGCAAGCCTCTCTGCCT
GATGTTTGTTGATGAGTCAGATCACGAAACGCTGACAGCTGTCTTGGGGC
CCGTAGTGGCTGAAAGGAACGCCATGAAGAACAGTCGTCTTATCTTGTCC
CTGGGTGGACTTTCTCGCTCCTTCCGCTTCCACTTCCGAGGCACGGGCTA
TGATGAGAAGATGGTGCGAGAGATGGAGGGTTTGGAGGCCTCTGGCTCCA
CGTACATCTGCACCCTGTGCGACTCCACTCGGGCTGAAGCCTCTCACAAC
ATGACTCTCCACTCTGTCACCCGCAACCACGACGAGAACCTGGAGCGCTA
CGAACTCTGGAGAACCAACCCTTATTCTGAGTCTGTTGAGGAGCTGCGAG
ACCGGGTGAAGGGCGTCTCAGCCAAACCTTTCATGGAGATCCAGCCCACC
CTGGACGCGCTGCACTGCGACATAGGCAATGCCACTGAGTTCTACAAGAT
CTTCCAGGATGAGATAGGGGAGATCTACGACAATCC---CAAC---CCCA
GCAGGGAGCAGCGTCGGAGTTGGCGGGCTGCACTCGACAAGCAGCTAAGA
CAGAAGATGAAGCTGAAGCCAGTGATGAGGATGAATGGAAATTATGCAAG
GAGGCTGATGACCCGAGAGGCTGTGGAAGCGGTGTGTGAGCTGGTGCGTT
CAGAGGAGCGTCAGGAGGCCCTGAGGGAACTGATGGGGCTCTACCTCAAG
ATGAAGCCTGTGTGGCGCTCCACATGCCCGGCCAAGGAGTGCCCAGACCA
ACTCTGCCAGTATAGCTTCAACTCCCAGCGCTTCGCAGAGCTGCTTTCCA
CAGTCTTTAAGTACAGGTATGATGGGAAGATCACCAACTACCTGCACAAG
ACCTTGGCCCATGTGCCAGAGATTGTGGAGAGGGATGGCTCCATCGGGGC
CTGGGCTAGTGAGGGAAATGAGTCTGGGAATAAGTCGTACACAATTGAGA
TGGGCCCCAAAGGGCCTCAATGGAAAGACAGCCCTACCCCTTTCTCCTGC
TCCATCGAGGACCCCACCAAGCAGACTAAGTTCAAAGGCATTAAGACTTA
CATATCCTACCGGGTGACCCCAAGCCACACAGGCAGGCCTGTTTACCGGC
GTTACAAGCACTTTGACTGGTTGTACAACCGTTTACTGCACAAGTTCACT

GTCATCTCAGTGCCCCACCTGCCCGAAAAACAGGCGACAGGGCGCTTCGA
AGAAGACTTCATCGAGAAGCGCAAGAGGCGGCTGATCCTTTGGATGGACC
ACATGACCAGCCACCCAGTCCTATCACAGTACGAGGGCTTTGAGCACTTC
CTTATGTGTGCCGACGACAAGCAGTGGAAGCTAGGAAAACGGCGGGCGGA
GAAGGATGAGATGATGGGCGCCAACTTCATGCTCACCTTCCAGATCCCCA
ACGAGCACCAGGACCTGCAGGATGTGGAGGAACGCGTAGACTCTTTCAAA
TCCTTCGCCAAGAAAATGGACGACAGCGTCATGCAGCTGACACATGTGGC
CTCGGAACTGGTGCGGAAGCACCTTGGAGGCTTCCGGAAGGAGTTCCAGC
GGCTGGGGAACGCGTTCCAGAATGTTAGTCAGGCGTTCATGCTAGACCCT
CCCCACTGCTCAGATGCCCTCAATACTGCCATCTCACACNNNNNNNNNNNNNTCTGCAGAATG
TCTCTCCTCGGTTTTATCATCGGCGTCGGTGTTGTCGGCAATCTCCTGAT
CTCCATTCTACTGGTCAAAGACAAAAGCCTGCACCGTGCACCCTACTACT
TCCTGCTCGACCTGTGCGCCTCGGATATCCTGCGCTCAGCCATCTGCTTC
CCCTTTGTCTTCACCTCCGTCAAGAATGGTTCCACCTGGACGTACGGCAC
GCTGACCTGCAAGGTGATCGCCTTCCTCGGAGTGCTGTCCTGCTTTCACA
CGGCGTTCATGCTCTTCTGTGTGAGCGTGACACGCTACCTGGCCATAGCC
CACCACCGCTTCTACACCAAGAGGCTAACCTTCTGGACGTGTCTGGCAGT
CATCTGCATGGTGTGGACATTGTCTGTGGCCATGGCCTTCCCGCCAGTGC
TGGACGTGGGGACGTACTCCTTTATAAAGGAAGAGGACCAGTGTACATTC
CAGCATCGCTCGTTCCGAGCCAACGACTCCCTGGGCTTTATGTTGCTCCT
AGCCCTCATCCTCCTGGCTACGCAGCTTGTCTACCTCAAGCTAATCTTCT
TCGTCCACGACCGCCGGAAGATGAAACCGGTCCAGTTTGTACCTGCCGTC
AGCCAGAATTGGACCTTCCATGGTCCCGGGGCTAGCGGCCAGGCGGCGGC
CAACTGGCTAGCGGGGTTCGGCAGGGGCCCCACCCCTCCCACCCTGCTAG
GCATCAGGCAGAACAGCAACGCGGCCGGCAGGCGACGCCTGCTTGTGCTG
GATGAGTTTAAAACTGAAAAGAGGATAAGTAGAATGTTCTACATCATGAC
CTTCTTCTTCCTCACTCTGTGGGGACCCTATCTGGTGGCCTGCTATTGGA
GAGTGTTTGCCAGGGGCCCGGCGGTCCCTGGGGGTTACCTGACTGCTGCG
GTGTGGATGAGCTTTGCCCAGGCGGGTGTAAATCCCTTCATCTGCANNNNNNNNNNNNNNNNNNNNNACT
C
TCGCTTTCACCCTGGCGTAGGGACCGGTCCTGGCACGGACC---GCAGCG
TCCCACTTAGTAACAGCTTGCTATCCCAGCAACAAACCGAAGAGCCGACA
GTTG---CATCCCCGCAGCGATGGTTTGTCACCC---CTGCCAACAACCG
ACTGGACTTTGCAGCCTCGGCATACGACGCGGCCGCAGCTGCTGATTTTG
CCGGTAACGCGGCCACCCTCCTGTCCTACGCAGCGGCTGGAGTAAAGGCA
C------TTCCTCTTCCTACCGCAGGTTGCTCAAACAGGCCTCTAGGTTA
CTATGCCGACCCATCCG---GCTGG---GGCACACGCACACCACCCCAGT
ACTGC---------AGTAAGTCTAGCTCTGTTCTCTCTTGCTGGCCTACA
AATTCGGTTGGTGGCAGAACAGGCA---CCTCCAACTACCTGG-------
--TTGATGA---CGCG---GAAGC---GATTCCAACGGAGAGGTCACCC-
--AT---CGGTGGGTCGGATGAG---GCAAAACCAAAAGATTT------A
TCCGA---ACCCAACTGGATAGAG---ACGCCGTCTTCGATAAAGTCTAT
TGACTCAAGTGATTCTGGGATCTTTG---AGCAAGCCAAACGGAGAAGAA
TTTCACCGACTGCCACACCG---------GTAACGGAAACAGTGTCCCAG
TTGAAATCTGAG---------ACAGGCGAAGTCACTGAAAGAGAAGTTGC
TTTGGGGATAAATCCGTTTGCAGACGGGATGGGCGCTTTTAAAATCAACC
ACAGCTCTCACGACCTAGGCTCCGG---CCAAACGGCGTTCTCCTCACAA
GCA---CCTGGCTAC---GCCGCCGCAGCACTGGGT---CACCACCA---
---CCACCCGACACACGTTAGCTCG---TACTCCACCGCAGCGTTCAACT
CAACCCGGGACTTTCTCTTCAGAAACCGGGGCTTCGGGGACGCGGCCAG-
-----------CGCGCAGCACAGCCTCTTCGCCTCAGC---AGCGGGAAG
TTT---T------GCTGGACCACACGGACACTCAGATAACGCGGGGCACC
TGCTCTTCCCGGGACTTCATGAG---CAAGCCGCGAGCCATGCGTCCTCG

AATGTAGTCAACAGCCAGATGCGACTGGGCTTTACCGGGGACATGTACGG
CCGGGCTGACCAGTATGGCCACGTTACCAGCCCCCGCT---CCGACCACT
ATGCATCCACCCAGCTCCACGGCTACGGCCCCATGAACATGAATATGGCG
GCT---CATCACGGAGCTGGGGCCTTCTTCAGATACATGAGGCAGCCGAT
CAAACAAGAGCTCATCTGCAAATGGGTCGAACCGGAGCAGTTGTCGAACC
CCAAGAAGTCCTGCAACAAAACGTTCAGCACGATGCACGAGCTGGTGACC
CATCTTACGGTGGAGCACGTCGGTGGACCGGAGCAGTCGAACCATATCTG
TTTTTGGGAAGAGTGTGTCCGAGAAGGAAAACCATTTAAAGCCAAGTACA
AACTTGTAAATCACATCAGAGTGCACACTGGAGAAAAACCATTCCCATGT
CCATTCCCCGGTTGTGGCAAA
>Etheostoma atripinne
AGCTTACTTATCCGAGCCGAACTCAGCCAACCGGGCGCACTCCTTGGAGA
CGACCAGATTTATAACGTAATTGTTACAGCGCATGCCTTTGTAATAATTT
TCTTTATAGTGATACCAATTATGATTGGGGGTTTTGGTAACTGACTCATT
CCACTCATGATTGGCGCTCCCGACATGGCATTTCCTCGGATGAACAATAT
GAGCTTTTGACTTCTCCCCCCTTCCTTCCTTTTACTTCTTGCCTCCTCAG
GAGTAGAAGCAGGGGCTGGAACCGGGTGAACCGTTTACCCCCCACTGGCT
GGGAACTTGGCACACGCCGGGGCATCAGTAGATTTAACTATTTTTTCCCT
ACACCTGGCGGGGATCTCTTCGATTCTAGGTGCCATTAATTTTATTACGA
CAATTATTAACATAAAACCCCCAGCCATCTCGCAGTACCAGACGCCCCTG
TTCGTATGGGCTGTTCTAATTACCGCCGTACTTCTTCTACTTTCCCTCCC
GGTGCTTGCCGCGGGTATTACCATGCTCCTTACGGATCGCAACTTAAATA
CCACCTTCTTTGACCCCGCAGGAGGGGGGGATCCCATCCTCTATCAACAC
CTC---

------------TTCCTAGAAAGAAACCTTCACCCAACTAACTGCCTTGG
CATGCTGTTACTGTCTGACGCTCACCAGTGCACCAAGCTGTCAGAGCTCT
CCTGGGGTATGTGCCTCAGCAACTTTCCCGCTATTTGCAAGACAGAGGAC
TTCCTCCAACTGCCCAAAGATATGGTTGTGCAGCTTTTGTCACATGAGGA
GCTAGAGACAGAAGATGAGAGACTGGTTTATGAAGCTGCTCTTAACTGGA
TCAACTATGACCTGGAAAAGAGACACTGCCACCTTCCAGAGCTCCTAAAA
ACGGTCCGCCTGGCCTTGCTGCCTGCCATCTTTCTCATGGAGAACGTGTC
TACAGAAGAGCTGATCAACGCAAAGCCCAAGAGCAAAGAGCTTGTGGATG
AAGCTATCCGCTGTAAGCTGAAGATCCTGCAAAACGATGGCGTGGTTAAC
AGCCCGTGTGCTCGACCAAGAAAGACCAGCCATGCCTTATTTCTTCTGGG
AGGGCAGACTTTCATGTGCGACAAGTTGTACCTGGTGGACCAGAAAGCCA
AAGAGATCATCCCCAAGGCCGACATTCCCAGCCCCAGGAAGGAGTTCAGC
GCCTGCGCCATCGGATGTAAGGTGTACATCACTGGTGGGA--GAGGCTC-
AGAGAATGGTGTGTCCAAAGATGTGTGGGTCTACGACACCGTCCAAGAGG
AATGGTCAAAGGCAGCACCCATGCTCATTGCCAGGTTTGGCCATGGCTCT
GCAGAGCTGAAACACTGCCTCTACGTGGTAGGAGGTCACACGGCAGCAAC
TGGCTGCCTCCCAGCTTCTCCTTCTGGCTGAATACATAGTTGTGTTTAGT
CGTTCAGCAACGAGGCTGATTGTGAATGAAGCAGAGCTAATCATGGCACT
AGCCCAGGAGTTCCAGATGAGAGTGGTCACAGTGTCCCTGGAGGAACAGT
CTTTCCCTGGTGTCGTCCAGGTGATCAGCGGGGCGTCCATGTTGGTCAGT
ATGCATGGGGCTCAGCTGATCACCTCACTCTTCCTCCCCAAAGGAGCTGC
TGTAGTGGAGCTGTTCCCTTTTGCAGTAAACCCAGAGCAGTACACCCCGT
ATAAAACCCTAGCCACCCTCCCAGGCATGGACCTTCACTACATCTCCTGG
AGAAACACCAAGGAGGAGAACACCATCACCCACCCAGACAGACCCTGGGA
ACAAGGAGGCATCACTCACTTGGAGAAAGAGGAGCGAGAGCGAATACTGG
CGAGCAAAGATGTCCCCCGACACCTGTGCTGCCGCAACCCAGAGTGGCTC

TACCGGATCTACCAGGACACTTTGGTCGACATCCCTTCCTTTGTGGAAGT
CCTCAA---AGAGGGCATGAAG---ACAAAGCCCAGCATGAAGAA---GT
TAAAGTCGACCAGCACAGTCCACCCAGGCCGGGTCAGAGAAGCCCAGTGT
CAGACCTTAGTCCAAACCCCTAATGAGGCTAAACTCACCGTCTCCTGGCA
GATCCCGTGGAATCTGNNNNNNNNNNNNNNNNNNNNNNNNNNNNNNNNNNNNNNNNNNNNN---------
----

-----------CCCAACAAGGAGGCCTTCAAGTGCGAAGAGTGCGGGAAG
CACTACAACACCAAGCTGGGATATAAGCGCCATGTGGCCATGCACTCTGC
CACGGCCGGGGATCTCACCTGTAAAGTGTGCATGCAGAGCTACGAGAGCA
CGCCYGTGCTCTTGGAGCACCTCAAGAGCCACTCCGGGAAATCTTCGGGC
GGCACCAAGGAGAAAAAACACCCGTGTGACCACTGTGACCGCCGTTTCTA
CACACGGAAGGATGTGCGACGGCACATGGTGGTCCACACTGGCCGAAAGG
ACTTCTTATGCCAGTACTGTGCCCAGCGCTTTGGCAGGAAGGACCATCTG
ACACGCCATGTGAAGAAGAGCCACTCGCAGGAGCTGCTAAAGATCAAGCC
GGAACCTCCCGACATGCTAGGGCTTTTGGCTTCTGGGTCGCCCCCTTGCT
CTGTGAAGGAGGAGCTCAGCCCCATGATGTGCGGCATGGGTCCTAACAAA
GACCCCATGATGGGCAAACCGTTCCCCAGTGGGGCCCCTTTTCCGATGGG
CATGTACAACCCCCACCAT------CTCCAGGCCATGTCTAATTCTGGGG
TGGGTCACCCG------CACCCGTCCCTGATGCCAAGTCCCTTGTCTGCA
GCTATGGGCATGGGCTGTCACATGGAATATCTCATCTACGCCTCTTTCTC
ATTCATGGGATGTTTACAAATCAGTGACGGCTCAAACATCGTGAACCTGC
TCGCTAGTAACTCTCAGAGTGTTTCGTATGCACTGACCCAGCAGAAGTAC
TTCAGTAACTACAGTCCCGTGATTGGGTTTTACATTTATGAGCCTATCGA
GTACTGGAACTCCACGGTGCAGGAGCACCTGAAGACTCTAAGTCATGGCT
TCAACAAGATCTCTTGGATGGACAACTTTTTCCACTACCTGCGGGTGGTG
AATGTGAGTGCTTCAACCAAGAGCGACTTCATCACCATCCTGAAGGGCTC
CTTCCTGCGCAGCCCGGAATACCAGCACTTCACTGAGGACATTATATTCT
CCAAGA---ACCGTGAGACCG------ATGAGTACGACATTATCGCCTCC
CGGATGTACTTGGTGGCACGGACGACAGAGAAGAAGCGGGAAGAGGTGGT
GGAGCTTCTGGAAAAGCTTCGTCCGTTGATGCTGATCAACAGCATCAAGT
TCATTGCCTTCAATCCTACGTTTGTGTTCATGGACCGCTACAGCTCCTCC
GTTATCTCACCAATCCTGACCTCAGGCTTCAGCGTACTCACAATCCTCAT
CCTCACTTTCTTTCTGGTCATCAACCCCTTGGGGAACTTCTGGCTCATCC
TCACGGTAACGTCCGTAGAGCTGGGTGTCTTGGGTTTGATGNNNNNNNNNNNNNNNNNNNNNCAGCCCGC
T
CTCAAGAAYGTGTCTACATCTTGCAGTGTTGGCATTATTAATGGAATCTC
TGGATGGGCTTCCTCGGTGGATGACTTCCCAGCTGACACCATCACTCGGC
GGTTTCGTTATGATGTGGCACTCGTGTCCGCATTAAAGGATCTGGAGGAG
GACATCATGGAAGGGCTGAGAGAGAGTGGGATGGAAGACAGTGCTTGCAC
CTCAGGCTTCAGTGTCATGATCAAGGAATCCTGTGATGGCATGGGCGATG
TCAGCGAGAAGCACGGTGGAGGACCAGCTGTTCCTGAGAAGGCTGTACGC

TTTTCTATCACTATTATGTCTGTCTCTGTCATAGCAGACGAGGAGGAG--
------------------------------AAAGAGGTTACCATCTTCACTG
AGCCAAAACCAAACTCAGAACTGTCCTGTAAGCCCCTTTGCCTGATGTTT
GTGGATGAGTCAGACCATGAGACGCTGACAGCCGTCCTGGGGCCTATAGT
TGCAGAGCGTAATGCAATGAAAGAGAGCAGGCTCATCATATCCATGGGTG
GACTACYTCGCTCCTTTCGCTTTCACTTCCGAGGCACGGGATATGATGAG
AAGATGGTGCGTGAGATGGAGGGCCTCGAGGCCTCAGGATCCTCCTATGT
GTGCACTCTTTGTGACTCCACTCGGGCAGAGGCCTCTGAAAACATGGTGT
TACATTCCGTCACCCGCAGTCATGSAGAGAACCTAGATCGGTACGAAATA
TGGAGAACCAATCCTTTTTCTGAGTCTGTTGATGAGCTGCGAGACAGAGT
TAAGGGGGTCTCTGCCAAGCCCTTCATGGAGACCCATTCCACACTGGATG
CATTGCACTGTGACATTGGCAATGCCACTGAGTTCTACAAAATCTTCCAG
GATGAGATCGGAGAGGTGTACAAAGGGGT---CAAC---CCCAGTCGGGA
GGAACGGCGCAGCTGGAGGGCAGCCCTAGATAAACAGCTGAGGAAGAAGT
TGAAGCTGAGACCGGTGATGAGGATGAATGGCAACTATGCCCGGCGGCTA
ATGACCCAAGAGGCTGTGGAGGTGGTGTGTGAGCTGGTGCCCTCAGAGGA
GAGGAGGGAGGCCCTGAGGGAGCTCATAAGGCTCTACCTCCAGATGAAGC
CTGTTTGGCGCTCCTCCTGCCCTGCCAAGGAGTGCCCCGACCAGCTGTGT
CGCTACAGCTTTAACTCCCAGCGCTTTGCTGACATTCTCTCCTCTACATT
TAAATATAGGTATGATGGAAAGATAACCAATTACCTGCACAAGACCCTGG
CCCATGTGCCTGAAATCATAGAGAGAGAYGGATCCATAGGANNNNNNNNNNNNNNNNNNNNNNNNNN
NNNNNNNTCGTACACC
ATCGAGATGGGTCCCATGGGGCCCCTGTGGAAGGAGAGCCCACAGCCTTT
CTCCTGCTCCATTGAAGACCCCACAAAACAGACAAAGTTCAAAGGCATCA
AGACGTACATTTCATACAGGGTCACACCAAGCCACACGGGGCGTCCCGTC
TACAGGCGCTACAAACACTTTGACTGGCTGTACAACCGTTTACTGCACAA
GTTCACTGTGATCTCCGTGCCTCACCTGCCTGAGAAGCAGGCCACGGGGC
GATTTGAGGAAGACTTCATCGAGAAGCGCAAGAGACGACTGATACTGTGG
ATGAACCACATGACCAGTCACCCAGTCCTCTCCCAATATGAAGGCTTCGA
GCACTTTCTGATGTGTGCTGATGACAAGCAGTGGAAACTGGGCAAGAGAC
GGGCGGAGAAGGATGAGATGGTGGGTGCCCATTTCATGCTGACCCTCCAG
ATCCCTAATGAGCACCAGGACCTACAGGATGTAGAGGAGCGGATCGACAC
CTTCAAGGCCTTCGCTAAGAAAATGGATGACAGTGTGATGCAGCTCACAC
ATGTTGCCTCGGAGCTGGTGCGTAAGCACCTGGGTGGATTTAGGAAGGAG
TTCCAGCGGCTAGGTAATTCATTCCAGTCCATCAGCCAGGCTTTCATGCT
GGACCCTCCCCACAGCTCAGAGGCCCTCAACAAAGCAATCTCCCATNCTCT
CGCTACGTTCCTCAAACTGACCTCTCTGGGTTTCATCATTGGAGTCGGTG
TGGTTGGTAACCTCCTAATCTCCATCCTGCTGGTCAAAGACAAGAGCCTA
CACCGGGCGCCCTACTATTTCCTGTTGGACCTCTGCGCCTCTGACATCCT
GCGCTCCGCCATCTGCTTCCCCTTTGTCTTCACCTCGGTCAAGAATGGAT
CAGCCTGGACCTATGGCACGCTGACCTGCAAAGTGATCGCCTTCCTGGGT
GTGCTGTCCTGTTTCCACACGGCGTTTATGCTKTTCTGTGTCAGTGTCAC
GCGTTACCTGGCAATCGCACACCACCGTTTCTACACCAAGAGGTTGACCT
TCTGGACTTGTCTAGCTGTCATCTGCATGGTGTGGACGTTATCAGTGGCA
ATGGCGTTCCCGCCGGTGTTAGACGTAGGGACGTACTCTTTTATCCGGGA
GGAGGACCAGTGCACGTTCCAGCATCGTTCCTTCAGGGCGAATGATTCGC
TGGGCTTCATGCTCCTGCTGGCGCTCATTCTCCTGGCCACACAGCTGGTT
TACCTCAAGCTCATCTTCTTTGTCCACGACCGTCGAAAGATGAAGCCAGT
CCAGTTTGTGCCTGCTGTCAGCCAGAACTGGACCTTCCACGGGCCAGGCG
CCAGCGGGCAGGCGGCAGCCAACTGGCTGGCTGGATTCGGTCGAGGCCCC
ACCCCGCCTACCTTGCTGGGCATTCGGCAGAACAGCAACGCAGCGGGCCG
CAGGCGTCTACTGGTGTTGGATGAATTCAAAACAGAGAAGAGGATAAGTA
GGATGTTCTACATCATGACGTTTTTCTTCCTGGCACTGTGGGGGCCCTAT

CTGGTCGCCTGCTACTGGCGGGTGTTTGCAAGGGGCCCTGTAGTCCCTGG
GGGCTACCTGACGGCAGCCGTGTGGATGAGCTTTGCCCAGGCTGGGGTAA
ATCCTTTCATCTGCATTCTCTCCNNNNNNNNNGCCAAATCTCGCTTTCACCCTGGCGTG
GGGACTGGTCATGGCACGGAGC---GCAGCGTCCCACTCGGCAACAGCTT
GCTGTCCCCGCAGCAAACCGACGAGCCCACTGTTGCCACCCCCCCGCAGC
GATGGTTTGTCACCC---CTGCCAACAACCGACTGGACTTTGCTGCCTCG
GCATACGACGCCGCT---------GATTTCGCCGGTAACGCGGCCACCTT
GCTGTCCTACGCAGCGGCCGGAGTGAAGGCTC------TTCCCCTGCCGA
CTGCAGGCTGCTCCAACCGGGCTCTTGGCTATTACGCCGACCCGTCAG--
-GTTGG---GGAGGACGCACTCCGCCACAATACTGTGGCGTAAACAGCAA
ATCCAGCTCGGTATTTTCCTGCTGGCCTGCTAACTCCATCGGGGGCAGAG
CGGGCA---CC---AACTACCTGG---------CTGAGGA---GGGA---
GACTC---CATCCCGACAGAGAGGTCACCG---AT---CGGCGGCTCGGA
GGAC---ACCAAACCCAAAGACATGGC---ATCTGA---GTCCAGCTGGA
TAGAG---ACGCCGTCCTCCATTAAATCCATTGATTCGAGCGACTCTGGT
ATCTTTG---AACAGGCCAAAAGGAGAAGAATCTCACCTTCTGCCACGCC
G---------GTTTCAGAGACAGTGTCCCCGTTAAAATCTNNNCATCACTCAA
CAGGCGAAGTCACAGAGAGAGAAGTGGCTTTGGGGATAAATCCGTTCGCC
GATGGGATGGGCGCTTTCAAAATAAACCACAGCTCCCACGATATTGGCTC
CGG---ACAAACGGCGTTTTCCTCCCAGGCG---CCCGGCTAC---GCYG
CAGCCGCCCTGGGA---CACCATCA------CCACCCGACCCACGTTGGC
TCT---TACTCCACGGCGGCTTTCAACTCCACCAGGGACTTTCTCTTCAG
AAATCGGGGATTCGGGGACGCCACCGG------------GGCACAACACA
GTTTGTTCGCCTC---------CGGAAGTTT---C------GCAGGGCCA
CATGGACACTCAGATGCAGCGGGGCACCTGCTCTTCCCAGGGCTCCACGA
G---CAAGCGGCAAGCCATGCGTCTTCTAAYGTGGTCAACAGCCAGATGC
GGCTGGGCTTCTCGGGGGACATGTACGGACGGGCCGACCAGTACGGCCAC
GTTTCAAGCCCACGGT---CCGACCACTATGCTTCGACCCAGCTGCACGG
CTATGGCCCCATGAACATGAATATGGCCGCA---CATCACGGAGCAGGGG
CCTTCTTTCGCTACATGAGGCAGCCGATCAAACAAGAGCTCATCTGCAAG
TGGATCGAGCCGGAGCAGYTGACAAATCCCAAAAAGTCGTGCAACAAAAC
TTTTAGCACGATGCACGAGCTTGTGACCCATCTGACTGTGGAGCATGTGG
GGGGACCAGAACAGACCAACCACATCTGCTTCTGGGAGGAATGCGCCAGA
GAAGGAAAGCCATTCAAAGCCAAATACAAACTTGTAAATCATATCAGAGT
ACACACCGGTGAAAAGCCCTTTCCGTGTCCGTTCCCCGGCTGTGGCAAA
>Euclichthys polynemus

--------------------GAGGAACCTCCACCCGACCAACTGCCTTGG

```
CATGCTGCTGCTGTCCGACGCCCATCAGTGCACCAAGCTCTCCGAGCTCT
CTTGGAGCATGTGCCTGAGCAACTTCCCTGCCATTTGCAAGACAGAGGAT
TTTCTCCAACTTCCCAAAGACATGGTTGTGCAACTTTTATCCCATGAGGA
GCTGGAGACRGAAGATGAGAAACTTGTTTACGAGGCCGCACTTAACTGGG
TCACCTATGACCTGCAAGGGAGGCACCCCCACCTGCCAGAACTGCTGAAA
ACAGTGCGCCTGGCCCTTCTGCCCGCCATCTTCCTCATGGAAAATGTTTC
CACGGAAGAACTGATAAACGCCCAGACCAAGAGCAAGGAGCTGGTGGACG
AGGCCATCTGCTGCAAGCTGAAGATCTTGCAAAATGACGGCGTGGTCAAC
AGCCCGTGTGCCCGTCCAAGGAAGACCAGCCACGCACTTTTTCTGCTGGG
AGGGCAGACCTTCATGTGTGACAAGCTATACCTGGTGGACCAGAAAGCCA
AAGAGATCATCCCCAAGGCCGACATACCCAGCCCCCGGAAGGAGTTCAGT
GCCTGTGCCATCGGCTGCAAGGTGTACATCACGGGTGGCC--GAGGCTC-
GGAGAACGGCGTGTCCAAAGATGTGTGGGTGTACGACACGGTACAGGAAG
AGTGGTCCAAGGCGGCACCCATGCTCATCGCCCGCTTCGGCCACGGCTCC
ACGGAGCTGAAACACTGCTTGTACGTGGTGGGCGGTCACACGGCCGCCAC
CGGCTGCCTCCCG-------------------------------------
--------------------------------------------------
--------------------------------------------------
--------------------------------------------------
--------------------------------------------------
--------------------------------------------------
--------------------------------------------------
--------------------------------------------------
--------------------------------------------------
--------------------------------------------------
--------------------------------------------------
--------------------------------------------------
--------------------------------------------------
--------------------------------------------------
--------------------------------------------------
--------------------------------------------------
--------------------------------------------------
--------------------------------------------------
--------------------------------------------------
--------------------------------------------------
--------------------------------------------------
--------------------------------------------------
--------------------------------------------------
--------------------------------------------------
--------------------------------------------------
--------------------------------------------------
--------------------------------------------------
--------------------------------------------------
--------------------------------------------------
--------------------------------------------------
--------------------------------------------------
--------------------------------------------------
--------------------------------------------------
--------------------------------------------------
--------------------------------------------------
--------------------------------------------------
--------------------------------------------------
--------------------------------------------------
```



```
---------------------------------------------------
---------------------------------------------------
---------------------------------------------------
---------------------------------------------------
---------------------------------------------------
---------------------------------------------------
---------------------------------------------------
---------------------------------------------------
---------------------------------------------------
---------------------------------------------------
---------------------------------------------------
---------------------------------------------------
---------------------------------------------------
---------------------------------------------------
---------------------------------------------------
---------------------------------------------------
---------------------------------------------------
---------------------------------------------------
---------------------------------------------------
---------------------------------------------------
---------------------------------------------------
---------------------------------------------------
---------------------------------------------------
---------------------------------------------------
---------------------------------------------------
---------------------------------------------------
---------------------------------------------------
---------------------------------------------------
---------------------------------------------------
---------------------------------------------------
---------------------------------------------------
---------------------------------------------------
---------------------------------------------------
---------------------------------------------------
---------------------------------------------------
---------------------------------------------------
---------------------------------------------------
---------------------------------------------------
---------------------------------------------------
---------------------------------------------------
---------------------------------------------------
---------------------------------------------------
---------------------------------------------------
---------------------------------------------------
---------------------------------------------------
---------------------------------------------------
---------------------------------------------------
---------------------------------------------------
---------------------------------------------------
---------------------------------------------------
---------------------------------------------------
---------------------------------------------------
---------------------------------------------------
```


>Eurypharynx pelecanoides
AGCCTATTAATTCGCGCTGAACTTACTCAACCAGGAGCCCTTCTTGGAGA
TGATCAGATTTATAATGTTATCGTAACAGCCCATGCTTTTGTAATAATTT
TCTTTATGGTGATACCAATTATAATCGGAGGATTCGGCAATTGACTAATC
CCCCTAATAATTGGAGCCCCAGACATAGCATTCCCCCGTATGAACAATAT
AAGCTTCTGACTTCTCCCCCCCTCATTTCTTCTCCTTTTAGCCTCTTCCG
GGGTAGAAGCCGGAGCCGGCACAGGGTGAACCGTTTATCCTCCCCTAGCT
GGAAATTTAGCTCATGCCGGAGCATCAGTAGATTTAACAATCTTCTCACT
TCATCTTGCAGGAATCTCCTCAATTCTTGGGGCCATTAATTTTATTACGA
CCATTATTAATATAAAACCCCCAGCCATTTCACAATACCAAACACCCCTT
TTTGTTTGATCTGTGTTAGTCACCGCTGTCCTCTTACTCTTATCCCTCCC
CGTCCTAGCTGCGGGCATCACAATACTCCTAACTGACCGAAACTTAAACA
CAACATTCTTTGATCCTGCAGGAGGGGGGACCCCATCCTTTACCAACAC
CTATTCTGATTTTTTGGGCACCCAGAAGTTTATATCTTAATTTTACCCGG
ATTCGGAATAATTTCACACATTATTACCTATTACTCGGGCAAAAAAGAGC
CCTTCGGATATATGGGCATAGTTTGAGCCATAATAGCCATTGGCCTATTA
GGCTTCATCGTATGAGCCCACCATATATTCACGGTTGGCATAGATGTAGA
CACCCGTGNNNN--

---------------------AGGCGAGTATATTGTGGTATTCAGTCGCT
CTCTTAATAGGCTGATCCTGAATGAGGCAGAGTTGATCCTGGCACTAGCG
CAAGAGTTCCAGATGAAGGCCGTTACTGTTTCCCTGGAAGAGCAGTCCTT
TGCAGACATCGTCCGAGTCCTCAGCAGGGCGTCCATGCTGGTCAGTATGC
ACGGGGCCCAGCTTGTCACCTCTCTTTTCCTTCCTCGTGGGGCCGCTGTG
GTGGAGCTATACCCATATGCAGTCAACCCAGAGCATTATGCTCCCTACAG
GACACTGACCTCGCTGCCAGGCATGGACCTGCAGTATGTGGCCTGGAGGA
ACACCAGGGAGGAGAACTCTGTTACCTTCCCTGAGCGTGCTTGGGACCAG
GGTGGCATTGCACACTTGGAGAAGGAGGAGCAGGCACGTATCATGAAGAG
CAAGGAGGTGCCGCGACACCTGTGCTGTCGGAACCCGGAGTGGCTCTTCC
GCATCTACCAGGACACCAAGGTGGACATTGCGTCTCTTCTGGATGCCCTG
CG---CCAGGGACTGACC---TCCAGGCCAGGGCCCAAGAG---GGCTAG
GCCTGCCAGCACAGTCCACCCAGGCAGGGTGAGGGAGCCCAAGTGCCAGA

CCTCCGTCCAGGCCACTAACGAGGCCACGCTGACCGTTTCTTGGCAGATC
CCCTGGAACCTCAAATACCTGAAGGTGAAGGAGGTGAAGTATGAGGTATG
GATTCAAAAGAAGGATACCAGCAAGGGGACCCTGGAGGATCAAATTATCC
AGGCAAACCCAGCGCTGGAGGCTTTTGGCAATGCCAAGACTGCGAGGAAT
GACAACTCCTCACGTTTTGGGAAATTTATTCGCATTCATTTTGGAGTAAG
TGGCAAGCTGTCCTCTGCTGATATAGAAACCTACCTACTCGAGAAATCTC
GTGTCACCTTTCAGCTTAAATCTGAGAGGAACTATCACATTTTCTACCAG
ATCACATCCAACAAAAAGCCAGAATTGCTGGACATGCTGTTGATCACCAA
CAACCCCTACGATTATGCTTATGTCTCCCAAGGAGAGGTGACAGTTGCAT
CCATTGATGACTCAGAGGAACTGATCGCCACAGACAATGCCTTTGATGTG
CTGGGCTTCACGGCGGAGGAGAAGATGGGTGTGTATAAGCTAACGGGTGC
CATCATGCATTATGGAAACATGAAATTCAAGCAGAAGCAGCGTGAGGAGC
AGGCCGAGCCTGATGGCACTGAGTCTGCTGACAAGTCAGCCTACCTGATG
GGGCTGAACTCTGCTGACCTTCTCAAGGGAATCTGCCATCCACGGGTTAA
AGTTGGAAATGAGTTTGTCACCAAAGGTCAAAATGTGGATCAAGTCTACT
AT--

------------------NNNNNGATCTATGCCTCATTTTCTTTCATGGATGTT
TACAAATCAGTGATGGGTCGAACATAGTCAACCTCCTGGCCACCAACTCC
CCAAGCGTATCCTACGCTCTGACCCAGCAGAAATACTTCAGCAACTACAG
TCCGGTCATTGGGTTCTACATCTACGAACCCATTGAGTACTGGAACGCCA
CGGTCCAAGAGCACCTGATGACACTGGGCCACGGATTCAATAAGATTTCG
TGGATTGACAACTTCTTTCAGTATCTGAAGGTGGTGAATGTCACCGTGTC
GACGAAAAGCGAATTCATAACCGTCCTCCAATCCTCCTTCCTGAGAAGCC
CCGAGTACCAGCACTTTAAGGATGACATCATATTTTACAAAA---TGAGG
G------------ACGAGACGGAGATAATTGCGTCCCGGATGTATCTGGT
GGCCCGAACCACGGAGAAGACCCGAGAGGAGGTGGTGGAGCTCCTGGAGA
GACTGAGACCGCTCTCTCTCATCAACAGCATCAAATTCATCGTCTTCAAC
CCAACCTTCGTCTTCATGGATCGGTACAGCTCGTCGGTCATTTCCCCCAT
CCTTACCGCGGGCTTCAGTGTCCTGATCGTCCTGATCCTGACCTTCTTCC
TGGTTATCAACCCACTGGGAAACTTCTGGCTGATACTGACGGTCACCTCA
GTGGAGCTGGGAGTCCTGGGCCTAATG-----------------------

TTTGCTGACGGGATGGGAGCTTTCAAAATCAACCATGGTACTCACGAACT
TAGTTCTGG---ACAAACTGCTTTCTCTTCGCAGACT---CCGAGCTAC-
--GCAGCAGCTGCTTTGGGG---CATCACCA------CCACCCGGCTCAT
GTCAGCTCC---TACTCAACCGCCGCGTTCAACTCCACCAGGGACTTTCT
GTTCCGAAATCGGGGATTCGGCGACGCAGCAAG------------CGCAC
AACACAGTCTTTTTGCCTCAGC---GGCAGGAACTTT---C------GCA
GGGGTTCATGGACACTCAGATGCCACCGGACACCTTCTATTCCCGGGACT
GCACGAG---CAGACAGCCACACATGGGTCTCCGAACGTTGTAAATGGCC
AGATGCGCCTGGGGTTTACCGGGGATATGTACGGCAGAGCAGAACAGTAT
GGGCAGGTGACGAGTCCAAGGT---CTGAACACTATGCATCGACACAGTT
GCACGGTTATGGTGCTATGAAC------ATGGCTGCA---CACCACGGGG
CTGGGGCCTTTTTTCGATTCATGAGGCAACCGAATAAACAAGAGCTCATC
TGCAAATGGATAGAACCGGACCAGCTGTCGAATCCCAAAAAGTTTTGTAA
CAAAACGTTCAGCACCATGCACGAGCTCGTGACTCACCTCACTGTTGAAC
ATGTTGGGGGGGCAGAACAGTCAAATCACATCTTCTTTTGGGAAGAATGT
CCCCGGGAAGGCAAGCCATTTAAACCAAAATACAAGTTT-----------

>Evermannella indica
NNNNNNNNNNNNNNNNNNNNNNNNNNNNNNNNNNNNNNNNNNNNNNNNNNNNACCAGATTTACAATGTAA
TCGTCACCGCCCACGCCTTCGTAATAATCTTC
TTTATAGTAATGCCAATTCTGATCGGTGGCTTTGGAAACTGACTAGTTCC
CCTGATACTCGGTGCGCCTGATATGGCATTCCCACGAATGAACAACATGA
GCTTTTGACTCCTTCCACCCTCCTTCCTTCTTCTCCTTGCCTCTTCCGCC
GTTGAAGCAGGGGCGGGTACGGGCTGAACCGTTTACCCCCCTCTAGCCAG
CAACCTGGCTCACGCTGGAGCCTCCGTTGACCTAACCATTTTCTCTCTCC
ATCTTGCCGGCATCTCCTCCATCCTGGGAGCCATTAACTTCATCACAACC
ATTATTAACATGAAACCCCCAGCCATCACACAATACCAAACCCCCCTGTT
TGTGTGAGCCGTGCTCATTACTGCCGTTCTTCTTCTTCTTTCCCTCCCTG
TTCTAGCAGCAGGGATTACCATGCTCCTCACGGACCGCAATTTAAATACA
ACTTTCTTTGATCCTGCGGGCGGAGGGGACCCAATTCTTTACCAACACCT
ATTCTGATTTTTCGGCCATCCAGAGGTATACATCCTTATTCTCCCCGGCT
TCGGTATTATCTCTCACGTTGTCGCCTACTACGCGGGGAAAAAAGAACCC
TTTGGCTACATGGGGATGGTCTGGGCCATGATGGCAATCGGCCTCCTAGG
ATTT--
----------TTCCTGGAGAGGAACCTGCACCCCTCCAACTGCCTGGGCA
TGCTGCTGCTGTCGGACGCCCACCAGTGCACCAAGCTGTCGGAGCTGTCC
TGGGGGATGTGCCTCGGCAACTTCCCCGCCATCTGCAAGACGGAGGACTT
CCTCCAGTTGCCCAAAGACATGGTGGTGCAGCTCCTGTCCCACGAAGAGC

TGGAGACGGAAGACGAGAGGCTGGTTTACGAGGCGGCCCTGAACTGGGTC
AACTATGACCTGGAGAGGAGACACTGCCACCTGCCGGAGCTGCTGAGAAC
CGTGCGTCTGGCCCTGCTTCCTGCCATCTTCCTCATGGAGAACGTCTCCA
CGGAGGAACTKATCAACGCCCAGGCCAAGAGCAAGGAGCTGGTGGACGAG
GCCATCCGCTGCAAGCTGAAGATCCTGCAGAACGACGGCGTGGTCAACAG
TCCCTGCGCCCGGCCCAGAAAGACCAGCCACGCCCTCTTCCTGCTGGGGG
GGCAGACCTTCATGTGTGACAAGCTGTACCTGGTGGACCAGAAGGCCAAA
GAGATCATCCCCAAGGCGGACATCCCCAGCCCCAGGAAGGAGTTCAGCGC
CTGCGCCATCGGCTGTAAGGTGTACGTCATGGGCGGCA--GAGGCTC-CG
AGAACGGCGTCTCTAAAGACGTGTGGGTATACGACACCGTCCACGAGGAG
TGGTCCAAGGCGGCGCCGATGATCATCGCCAGGTTCGGCCACGGCTCCGC
CGAGCTCAAACACTGCCTCTACGTGGTCGGAGGACACACCGCGGCCACCG
GCTGCCTCCCCGCCTCTCC----GGACGAGTACATCGTGGTGTTCAGCCG
CTCCTCCACGCGGCTGATCCTGAACGAGGCGGAGCTGATCCTGGCGCTGG
CCCAGGAGTTCCAGATGCGGGTGGTGACGGTGTCCCTGGAGGAGCAGTCC
TTCCCCGGCATCGTGCAGGTCATCAGCGGGGCCTCCATGTTGGTCAGCAT
GCACGGCGCGCAGCTCGTCACCTCGCTCTTCCTCCCCAGGGGGGCCGCCG
TGGTGGAGCTCTTCCCCTACGCCGTGAACCCGGAGCAGTACACCCCGTAC
AAGACCCTGGCCTCCCTGCCGGGCATGGACCTCCAGTACGTCTCCTGGAG
GAACACTATGGAGGAGAACACCGTCGCCCACCCGGACAGAGCCTGGGACC
AGGGAGGCATCACCCATCTGGAGAAAGAGGAGCAGGAGAGAATCCTGGCC
AGCAAGGACGTCCCCAGGCACCTGTGCTGCCGCAACCCGGAGTGGCTCTT
CAGGATCTACCAGGACACTCTGGTGGACATCCCCTCCTTGCTGGAGGTCC
TCAA---GGAGGGCCTGAAG---ACGAGGCCGAGCTTGAAGAA---GTCC
AAGCCGGCCAGCACGGTTCACCCGGGCCGCGTCCGAGAGCCCCAGTGCCA
GACTTCGGTCCAGGCCACCAACGAGGCCAAGCTCACGGTGTCCTGGCAGA
TCCCGTGGAACCTCAAGTACCTGAAGGTGCGAGAGG--------------
---------AAAAAGGACACAAGCAAGGGAACCCTGGAGGATCAGATCAT
TCAGGCGAACCCTGCGCTGGAGGCTTTCGGTAACGCCAAAACACTGAGGA
ACGATAATTCCTCCCGTTTTGGAAAATTCATCCGAATCCACTTCGGAACC
AGCGGTAAATTGTCCTCGGCGGACGTTGAGACGTACCTGCTGGAAAAGTC
GAGGGTCACCTTTCAGCTCAAGGCCGAGAGGAACTACCACATCTTCTTCC
AGATCTTGTCCAATCAAAAGCCAGAGCTGCTGGACATGCTGTTGATCACC
AACAACCCTTACGACTACTGCTACATCTCCCAAGGAGAAGTGACCGTAGC
GTCCATCAACGACTCCGAAGAGCTGATGGCCACCGACAGTGCCTTCGACG
TGCTCGGCTTCACGCAAGAGGAGAAGATGGGAGTCTACAAGTTGACAGGG
GCCATTATGCACTATGGCAACATGAGGTTCAAGCAGAAGCAGCGCGAGGA
ACAGGCGGAGCCTGATGGCACAGAAGCTGCCGATAAGTCGGCTTATCTAA
TGGGGCTGAATACAGCAGACCTAATCAAAGGTCTCTGCCATCCCAGAGTC
AAGGTAGGAAACGAGTATGTCACCAAAGGCCAGGGTGTGGATCAAGTCTA
CTAC----ACAAGGAGGCGTTCAAGTGCGAGGAGTGCGGCAAGCATTACA
ACACCAAGCTGGGCTACAAGCGCCATGTGGCCATGCACTCGGCCACCGCG
GGCGACCTCACCTGCAAGGTGTGCATGCAGAGCTACGAGAGCACCCCCGT
GCTGCTGGAGCACCTCAAGAGCCACTCGGGGAAGTCGTCCGGCGGCGCCA
AGGAGAAGAAGCACCCGTGCGACCACTGCGACCGCCGCTTCTACACGCGG
AAGGACGTCCGGCGGCACATGGTCGTCCACACGGGCCGCAAGGACTTCCT
GTGCCAGTACTGCGCCCAGCGCTTCGGCCGCAAGGACCACCTGACGCGGC
ACGTGAAAAAGAGCCACTCGCAGGAGCTGCTGAAGATCAAGACGGAGCCG
CCGGACATGCTGGGTCTCCTGGGCTCCGGCTCGCCGCCGTGCTCCGTCAA
GGAGGAGCTGAGCCCCATGATGTGCAGCATGGGTCCCAACAAAGACCCCA
TGATGGGCAAGCCCTTCCCCAGCGGGACGCCCTTCCCCATGGGCATGTAC
AACCCGCACCAC------CTCCAGGCCATGTCTAACCCCGGGGTCGGCCA
CCCG------CACCCCTCCCTCATGCCCGGCTCCCTGTCCGCAGCCATGG

GCATGGGCTGTCACATGGAG------------------------------

----------------------------------GGCTACCACGCGTTCG
AGTGGCAGCCGGCCCTCCGGAACGTGTCCCCCTCCTGCCACGTGGGTATC
ATCAACGGGCTTTCCGGGTGGGCCGCTTCGGTGGACGAGGTCCCCGCCGA
CACCATTACCCGTCGGTTCCGCTACGACGTGGCCCTCGTGTCGGCCCTGA
AGGACCTGGAGGACGACATCGTGGACGGGCTGAGGGAGCGCGGGCTGGAA
GACAACCCTTGCACCTCGGGCTTCAGCGTCATGATCAAGGAGTCCTGCGA
CGGCATGGGAGACGTCAACGAGAAGAACGGCGGGGGGCCGGCGATGCCCG
AGAAGGCCGTGCGTTTCTCCTTCACCGTCATGTCCGTCTCCGTCCGCGCC
GACGGAGAGGAG------------------------------GAGGCGGT
CGTCGTCTTCAGGGAGCCGAAGCCCAACTCCGAACTGTCCTGTAAGCCCC
TGTGCCTGATGTTTGTGGACGAGTCTGACCACGAGACGCTCACGGCTGTC
CTGGGACCTTTGGTGGCGGAGAGGAACGCGATGAAGCAAAGCCGACTCAT
CCTCCCCGTGGGGGGGCTCCCCCGCTCCTTCCGCTTCCACTTCAGAGGCT
CGGGCTACGACGAGAAGATGGTGCGAGAGATGGAAGGCCTGGAGGCCTCG
GGCTCCACCTATATCTGCACCCTCTGCGACTCCACCCGGGCGGAGGCCTC
TCACAACATGGTGCTCCACTCCATCACGCGCAGCCACGACGAGAACCTGG
AGCGCTACGAGACGTGGAGGACCAACCCCTTCTCCGAGTCCGCCGCGGAG
CTGCGAGATCGGGTCAAAGGGGTCTCCGCCAAACCGTTCATCGACACCCA
TCCGACGATGGACGCGTTGCATTGTGACATCGGTAACGCCACCGAATTCT
ACAAGATCTTCCAGGATGAGATCGGGGAGGTGTTCCGGAAGGCCAACAAC
---CCCAGCCGGGAGGAGCGGCGCGGCTGGCGGGCGGCGCTCGACAAACA
GCTGAGGAAGAAGCTGAAGCTGAAGCCGGTGATGCGGATGAACGGGAACT
ACGCCCGAAGGCTGATGACCGAGGAGGCGGCGGAGGCGGTGTGCGAACTG
GTGCCCTCGGAAGAGAGACGGGAGACCCTGAGGGAGCTGATGGCGCTCTA
C---

---CATCACTCAACAGGCGAAGTCACAGAAAGAGAAGTGGCTTTGGGGAT
AAATCCGTTCGCGGACGGGATGGGCGCCTTCAAAATAAACCACAGCTCTC
ATGACATTGGCTCCGG---GCAAACGGCGTTTTCCTCCCAGGCG---CCC
GGCTAC---GCAGCGGCTGCCCTGGGA---CACCATCA------CCACCC
GACCCACGTCAGCTCC---TATTCCACGGCAGCTTTTAATTCCACCCGGG
ACTTTCTCTTCAGAAATCGGGGCTTCGGAGACGCCACCAG----------
--CGCGCAGCACAGTCTGTTCGCCTCCGC---CGCGGGAAGTTT---T--
----GCAGGGCCACATGGACACTCAGATGCTGCGGGCCACCTGCTCTTCC
CTGGGCTTCATGAG---CAAGCCGCCAGCCATGCGTCGTCTAACGTTGTA
AACAGCCAGATGCGACTGGGCTTTTCTGGGGACATGTACGGGCGGGCCGA
CCAGTATGGCCACGTTACAAGCCCACGGT---CCGACCACTACGCTTCGA
CCCAACTGCATGGCTATGGCCCTATGAACATGAATATGGCCGCT---CAC
CACGGAGCAGGGGCCTTCTTTCGGTACATGAGGCAGCCGATTAAGCAAGA
GCTCATCTGCAAGTGGATCGAACCGGAGCAACTGACGAATCCCAAAAAGT
CGTGCAACAAAACTTTTAGCACGATGCACGAGCTCGTGACCCATCTGACG

GTGGAGCATGTGGGTGGACCCGAGCAGTCGAACCACATTTGCTTCTGGGA
AGACTGCGCCCGAGAAGGGAAACCGTTCAAA-------------------

>Facciolella gilbertii
AGTCTTCTAATTCGAGCCGAACTAAGCCAACCCGGAGCATTACTAGGCGA
CGACCAAATTTATAATGTAATCGTCACAGCACATGCTTTCGTAATAATTT
TCTTTATAGTAATACCAGTAATAATCGGGGGTTTCGGCAACTGACTCGTA
CCCTTAATGATTGGGGCCCCAGACATGGCATTCCCACGAATAAATAACAT
AAGCTTCTGATTACTACCCCCATCATTTCTTCTATTATTAGCTTCTTCAG
GAGTTGAAGCTGGGGCGGGCACAGGTTGAACCGTGTACCCACCCCTTGCA
GGAAACCTCGCTCACGCAGGAGCCTCAGTTGACCTAACAATCTTTTCTTT
ACATCTCGCAGGAGTTTCATCTATTCTAGGGGCCATTAATTTTATTACCA
CTATTATTAATATGAAGCCCCCTGCTATCACACAATACCAAACCCCCTTA
TTTGTTTGATCTGTACTAATTACAGCTGTACTACTCCTCCTTTCATTACC
AGTTCTAGCCGCAGGCATTACCATACTCCTCACGGACCGAAACCTAAACA
CAACATTCTTTGACCCGGCAGGGGGAGGAGACCCAATTCTTTACCAACAC
CTATTCTGATTCTTCGGACACCCAGAAGTCTATATTTTAATTTTACCCGG
ATTTGGCATGATCTCACACATTGTTGCCTACTACGCCGGAAAAAAAGAAC
CTTTCGGGTATATAGGAATAGTGTGAGCCATGATGGCCATCGGCCTTCTA
GGATTTATCGTCTGAGCCCATCATATATTTACAGTAGGCATGGATGTAGA
CACCCGAGCATNTTCCTGGAGCGTANCCTGCACCCCACCAACTGCCTGGGCA
TGCTGCTGCTGTCGGACGCCCACCAGTGCGCCAAGCTGTCCCAGCTCTCC
TGGGGTATGTGTCTCAGCAACTTCCCCACCATCTGCAAGACCGAAGACTT
TCTGCAGCTGCCCAAAGACCTGCTGGTGCAGCTGCTGTCCCACGAGGAGC
TGGAGACAGAGGACGAGAGGCTGGTGTACGAGTCGGCCTTCAACTGGGTG
AACTACGATCTGGAGCGGAGGCACTGCCACCTGCCCGAGCTGCTACGCAC
TGTGCGCCTGGCCCTGCTGCCTGCCATCTTCCTCATGGAGAACGTCTCCA
CAGAGGACCTCATCACCCAGCAGACTCAGAGCAGGGAGCTGGTGGACGAG
GCGATCCGCTGCAAGCTGAAGATCCTGCAGAACGACGGTGTGGTGACCAG
CCTGTGTGCCCGGCCCAGGAAGACCAGCCATGCCCTCTTCCTGCTGGGCG
GCCAGACCTTCATGTGTGACAAGCTGTACCTGGTGGACCAGAAGGCCAAA
GAGATCATCCCCAAGGCAGACATCCCGAGCCCCCGCAAGGAGTTTAGCGC
CTGTGCTATCGGCTGCAGAGTGTTCGTGACGGGGGGAC--GTGGCTC-TG
AGAATGGCGTGTCGAAGGACGTGTGGGTGTACGACACGCTTCAGGAGGAG
TGGAGCAAGGCTGCGTCCATGCTGATTGCGCGGTTCGGGCACGGCTCAGC
CGAGCTGCAGCACTGCCTCTACGTGGTGGGGGGACACACAGCCGCCACCG
GCTGCCTCCCGGCCTCCCCCTCC---------------------------

---------AAGAGGGATACCAGCAAGGGGACACTGGAGGATCAAATCAT

CCAGGCAAACCCAGCACTGGAGGCTTTTGGCAATGCCAAGACTGCAAGGA
ATGACAACTCCTCACGTTTTGGTAAATTTATTCGCATTCATTTCGGAGTA
AGTGGCAAGCTATCCTCCGCTGACATAGAAACCTACCTACTTGAGAAATC
TCGTGTCACCTTTCAGCTCAAATCTGAGAGGAACTATCACATATTCTATC
AGATCATGTCCAACAAAAAGCCAGAATTGCTGGACATGCTGCTCATCACA
AACAACCCATACGATTATGCTTATGTCTCCCAAGGAGAGGTGACGGTCGC
ATCCATCGATGACTCAGAGGAACTGATTGCCACAGACAGTGCCTTTGATG
TGCTGGGCTTCACGGCAGAGGAGAAAACAGGTGTCTATAAGCTAACAGGT
GCCATCATGCATTATGGAAATATGAAATTCAAGCAGAAGCAGCGTGAGGA
GCAGGCAGAGCCTGATGGCAGTGAGTCTGCTGACAAGTCGGCCTTTCTAA
TGGGGCTGAACTCTGCTGACCTTCTCAAGGGACTTTGCCATCCAAGGGTT
AAAGTTGGAAATGAGTATGTCACCAAAGGCCAAAATGTGGATCAAGTTTA
CTATCCCAACAAGGAGGCCTTCAAGTGTGAGGAGTGTGGGAAGCACTACA
ACACCAAGCTGGGATACAAGCGTCATATGGCGATGCACTCTGCCACGGCA
GGGGATCTCACCTGCAAAGTGTGCATGCAGAGCTACGAGAGTACCCCTGT
TCTCCTGGAGCACCTCAAGAGCCACTCCGGGAAGTCGTCAGGTGGCACCA
AGGAGAAAAAACACCCGTGTGACCACTGTGACCGTCGCTTCTACACAAGG
AAGGATGTGAGGCGGCACATGGTGGTCCACACAGGCCGCAAGGACTTCCT
GTGCCAGTACTGTGCCCAGCGCTTTGGCAGGAAGGACCACCTGACACGCC
ATGTGAAGAAGAGCCACTCGCAGGAGCTGCTGAAGATCAAGACAGAGCCA
CCAGACATGTTAGGTCTTTTAGCTTCCGGATCCCCACCCTGCTCTGTGAA
GGAGGAGCTCAGTCCAATGATGTGCGGCATGGGTCCCAACAAAGATCCGA
TGATGGGCAAACCTTTCCCCAGTGGGGCCCCTTTTCCAATGGGCATGTAC
AACCCCCACCAT------CTCCAGGCAATGTCTAATACGGGGGTGGGTCA
CCCA------CACCCGTCCATGATGCCCAGTTCCTTGTCTGCAACTATGG
GCATGGGCTGCCACATGGAATACCTGATTTACGCTTCCTTCTCTTTCATG
GGATGTTTACAAATCAGCGACGGGTCGAACATCGTCAACCTCCTGGCCAG
CAACTCCCCGAGCGTCTCCTACGCCCTCACCCAGCAGAAGTACTTCAGCA
ACTACAGTCCGGTGATCGGGTTCTACATCTACGAGCCCATCGAGTACTGG
AACTCCACCGTCCAGGAGCACCTGAAGACGCTGGGCCACGGGTTCAATAA
GATTTCCTGGATTGACAACTTCTTTCAGTATCTGAAGGTGGTGAATGTCA
GCGCGTCAACCAAAAGTGAATTCATAACCATCCTCCAGACCTCCTTCTTG
AGGAGCCCAGAGTACCAGCACTTCAAGGACGACATCATATTTTACAAAA-
--TGAGGG------------ACGAGACGGAGATCATCGCCTCCCGGATGT
ACCTGGTGGCCCGGACCACGGAGAAGACCAGGGAGGAGGTGGTGGAGCTC
CTGGAGAGGCTGAGGCCGCTCTCCCTCATCAACAGCATCAAGTTCATCGT
CTTCAACCCCACCTTCGTGTTCATGGACCGGTACAGCTCGTCGGTCGTTT
CGCCCATCCTCACCTCGGGTTTCAGCGTCCTGATCATCCTCATCCTGACC
TTCTTCCTGGTCATCAACCCCCTGGGGAACTTCTGGCTGATACTGACGGT
CACCTCCGTGGAGCTGGGAGTTCTGGGCCTAATG----------------

---TCATACACC
ATCGACATGGGTCC-ATGGGGCCCCAGTGGAAGGCAAACCCCCAGCCATT
CGTCTGCTCAATNGAAGACCCCACCAAACAGACCAAGTTCAAGGGCATCAA
GACCTACATTTCTTACCGGGTCACRCCSAGCCACACAGGGCGTCCTGTGT
ACMGACGGTACAARCACTTYGACTGGCTSTACAACCGGTTACTGCACAAG
TTCACCGTSATCTCTGTGCCCCACCTGCCCGAGAAGCAGGCCACGGGGCG
ATTTGAGGAAGACTTCATCGAGAAGCGCAAGAGACGACTGGTCCTGTGGA
TGAACCACATGACCAGTCACCCGGTSCTCTCTCAGTATGARGGCTTGGAG
CACTTTCTGATGTGCGCCGACGACAAACAGTGGAAACTGGGAAAGAGRCG
AGCGGAGAAGGACGAGATGGTGGGCGCCCATTTCATGCTGACCTTCCAGA
ATCCCAACGAGCACCAGGACCTTCAGGATGTAGAGGAACGGGTGGACACC
TTCAAGTCCTTCGCCAAAAAAATGGACGACAGTGTGATGCAGCTCACGCA
CGTGGCCTCGGAGCTGGTGCGCAAACATCTGGGAGGGTTCAGGAAGGAGT
TCCAGAGGCTKGGRAATGCCTTCCAGTCCATCAGCCAGGCTTTCACGCTG
GACCCTCCCCACAGCTCAGACACCTCCACCAACGCCCTTCTCCCA-----

---------ACGTGATGATCTCCATCCTGCTGGTCAAAGACAAGAGCCTG
CACCGGGCACCCTACTATTTCCTGCTGGACCTGTGCGCCTCCGACATCCT
GCGCTCCGCCATCTGCTTTCCCTTCGTCTTCACTTCGGTCAAGAATGGGT
CGGCCTGGACGTACGGCACGCTGACTTGCAAGGTGATCGCCTTCCTGGGT
GTCCTCTCCTGTTTCCACACGGCGTTCATGCTGTTTTGCGTCAGCGTCAC
CCGCTACCTGGCCATCGCTCATCACCGTTTCTACACCAAGAGGCTGACCT
TCTGGACCTGCCTCGCCGTCATCTGCATGGTGTGGACGTTGTCGGTGGCT
ATGGCGTTCCCGCCAGTGCTAGATGTAGGGACGTACTCTTTTATCCGGGA
GGAGGACCAGTGCACATTCCAGCACCGCTCCTTCAGGGCAAACGATTCGC
TGGGCTTCATGCTCCTGCTGGCGCTCATCCTCCTGGCCACACAGCTGGTT
TACCTCAAGCTCATCTTTTTCGTCCACGACCGCCGAAAGATGAAGCCTGT
CCAGTTCGTGCCTGCCGTCAGCCAGAACTGGACCTTCCACGGGCCGGGGG
CCAGCGGGCAGGCGGCGGCCAACTGGCTGGCTGGATTTGGCCGAGGCCCC
ACCCCGCCTACTTTGCTGGGCATCCGGCAGAACAGCAACGCAGCAGGCCG
CAGGCGTCTACTGGTGTTAGATGAGTTCAAAACGGAGAAGAGGATTAGTA
GGATGTTCTACATCATGACGTTTTTCTTCCTGGCTCTGTGGGGGCCCTAT
CTGGTTGCCTGCTACTGGCGGGTGTTTGCAAGGG-CCCCGTGGTCCCTGG
GGGCTACCTGACGGCAGCCGTGTGGATGAGCTTTGCC-------------
--------------------------------GCCAAATCCCGCTTCCAC
CCTGGCACCGGGACCGGTCCTGGCACGGACC---GCAGCGTCCCACTTAG
CAACAGCTTGCTATCCCCGCAACAAGCCGAAGAGACCGCAGCAG---CCT
CCCCGCAGCGATGGTTTGTCACCC---CTGCCAACAATCGACTGGACTTT
GCCGCCTCGGCATACGATGCCGCTGCCGCGGCTGATTTGCCGGTAACGC

GGCCACCCTGCTGTCTTACGCAGCTGCTGGAGTCAAGGCGC------TTC
CACTGCCCACTGCGGGGTGTTCAGGTAGACCTCTGGGTTATTACGCCGAC
CCCTCGG---GCTGG---GGCACGCGCACTCCACCCCAGTATTGT-----
----AGTAAATCCAGCTCAGTTTTGTCCTGTTGGCCAACAAATTCCACTG
GGAGCAGGACTGGAA---CTTCAAATTACCTGG---------TGGATGA-
--GGGG---GACAC---GATGACAACGGAAAGGTCTCCA---CT---AAG
TGCGTCCGAGGAT---GCCAAACCTAAAGACCT------GTCGGA---AT
CCAGCTGGATAGAG---ACACCTTCCTCCATTAAATCGATCGATTCAAGT
GATTCTGGAATTTTTG---AGCAGGCGAAACGGAGGCGAATATCACCTTC
TGCCACGCCG---------GTTTCTGAAACTGCATCTCCATTGAAAAGCG
AA---------ACAGGCGAAGTCACCGATAGAGAAGTTGGGTTGGGGTTA
AATCCCTTTGCTGACGGGATGGGCGCTTTCAAAATCAACCAMGGTACTCA
CGAGCTTGCTTCTGG---ACAGACCGCTTTCTCATCACAGGCT---CCCA
GSTAC---GCAGCAGCCGCTTTGGGG---CATCACCA------CCACCCT
GCTCATGTCAGCTCC---TACTCAACCGCAGCGTTCAACTCCACCCGGGA
CTTTCTGTTCCGAAATCGGGGATTCGGAGACGCCGCAAG-----------
-TGCGCAACACAGTCTTTTTGCCTCAGC---GGCAGGAACTTT---C---
---GCAGGGCCACATGGACACTCAGATGCCACCGGACACCTTCTTTTCCC
GGGACTGCACGAG---CAGGCAGCCACACATGGGTCTCCAAACGTTGTAA
ATAGTCAGATGCGCCTGGGGTTTACCGGGGACATGTACGGCAGAGCAGAA
CAGTATGGACAGGTGACGAGTCCCAGGT---CTGAGCACTATGCTTCGAC
ACAGTTGCACGGGTATGGTGCCATGAACATGAACATGGCTGCA---CACC
ACGGGGCTGGGGCCTTTTTTCGATACATGCGGCAACCCATTAAACAAGAG
CTTATCTGCAAGTGGATTGAACCGGACCAGCTGTCGAATCCCAAAAAGTC
TTGTAATAAAACTTTCAGCACCATGCACGAGCTCGTGACTCACCTCACGG
TAGAACATGTTGGGGGACCTGAACAATCAAATCACATTTGCTTTTGGGAA
GAATGTCCCCGGGAAGGCAAGCCATTTAAAGCAAAATACAAACTCGTTAA
CCATATCAGAGTGCACACGGGCGAAAAGCCGTTCCCATGCCCATT-----

>Fistularia petimba
AGCCTTCTCATCCGCGCAGAGCTCAGCCAGCCCGGTGCACTACTGGGCGA
CGACCAGATCTATAATGTAATCGTTACGGCCCACGCCTTTGTAATAATTT
TCTTTATAGTAATACCAATCATGATCGGAGGCTTCGGAAACTGATTAATT
CCTCTTATGATCGGTGCCCCAGACATAGCCTTTCCCCGGATAAATAACAT
AAGCTTCTGACTTCTTCCCCCATCTTTCTTACTTCTATTAGCATCCTCTG
GAGTTGAAGCTGGGGCCGGAACAGGGTGAACAGTCTACCCTCCTCTTGCA
GGAAATCTGGCCCACGCTGGAGCCTCTGTAGACCTAACGATCTTCTCCCT
GCACCTAGCAGGCATCTCATCAATCCTAGGAGCAATTAACTTCATCACAA
CCATTATCAACATAAAACCTCCAGCCATCTCACAGTACCAGACACCTCTT
TTCGTCTGAGCTGTCCTCATTACCGCTGTGCTTCTCCTACTTTCACTGCC
TGTTCTTGCTGCCGGCATTACCATGCTCTTAACGGACCGAAATCTAAACA
CCACATTTTTCGACCCAGCGGGAGGAGGCGACCCCATCCTGTATCAACAC
CTA---

------------NNNNNNNNGAGAAACCTTCACCCATCCAACTGTCTTGGCATGCTGT
TGCTTTCTGATGCCCACCAGTGCACCAAGCTGTCAGAGCTTTCCTGGGGC
ATGTGCCTCAGCAACTTCCCTGCTATTTGCAAGACGGAGGACTTCCTTCA
ACTGCCAAAAGATATGGTGGTTCAGCTTTTGTCACACGAGGAGCTAGAAA
CAGAGGATGAGCGTCTAGTTTATGAAGCTGCCCTGAACTGGATCAACTAT
GACCTGGAAAGACGGCACTGCCACCTTCCGGAGCTCCTCAGAACCGTCCG
TTTAGCTCTTTTGCCAGCGATCTTTCTCATGGAGAATGTCTCAACAGAAG

AGCTGATCAATGCCCAAGCCAAGAGCAAGGAGCTGGTGGATGAGGCTATC
CGCTGTAAGCTGAAGATCCTGCAGAACGACGGTGTTGTGAACAGCCAGTG
TGCTAGGCCAAGAAAAACAAGTCATGCTCTGTTTCTGCTGGGGGGACAGA
CTTTCATGTGTGACAAGTTGTATCTAGTGGATCAGAAAGCCAAAGAGATC
ATCCCGAAGGCTGACATTCCTAGTCCCAGGAAAGAATTCAGTGCTTGTGC
AATCGGTTGTAAGGTGTATATCACAGGTGGAA--GAGGCTC-AGAGAATG
GCGTCTCCAAAGATGTATGGGTTTATGACACTGTCCATGAAGAATGGTCC
AAAGCGGCACCCATGCTCATTGCCAGATTTGGTCATGGTTCTGCAGAGCT
GAAACACTGCCTATATGTGGNNNNNNNNNNNNNNNNNNNNNNNNNNNNNNNNNNNNNNNNNNNNNNN---
----

------AAAAAAGACACCAGTAAAGGAACGCTGGAGGATCAAATCATCCA
GGCCAACCCAGCACTGGAAGCCTTTGGTAACGCCAAAACTTTGAGGAACG
ACAACTCATCCCGATTCGGCAAGTTCATCCGGATCCACTTTGGAACCAGC
GGAAAGTTGTCGTCGGCCGACATCGAGACCTACCTGCTGGAGAAGTCGCG
TGTGACCTTTCAGCTTAAGGCCGAGAGGAACTACCACATCTTCTACCAGA
TCCTGTCCAACGAGAAGCCTGAGCTTCTGGACATGCTGTTAATCACCAAC
AACCCATATGACTACTCTTACATCTCCCAAGGAGAGGTAACGGTCGCCTC
CATCAACGACTCGGAGGAGCTCATGGCCACTGACAGCGCCTTCGATGTGC
TGGGCTTCACTCCAGAGGAGAAGATGGCCGTCTACAAACTGACCGGCGCC
ATCATGCACTACGGTAACATGAAGTTCAAGCAGAAGCAGCGTGAGGAGCA
GGCCGAGCCTGACGGGACGGAGGCTGCAGATAAATCGGCCTATCTGATGG
GGCTGAACTCGGCGGACCTCATCAAGGGACTCTGTCATCCTAGAGTCAAG
GTGGGAAATGAGTACGTGACCAAGGGCCAAAGTGTGGACCAAGTCTACTA
CNNNNNNNNNNNNNNNNNNNNNNNNNNNNNNNNNNNNNAGCACTACAACACCAAGCTGGGATACAAGCGT
CACATGGCCATGCACTC
CGCCACTGCAGGGGACCTCACCTGCAAGGTGTGCATGCAGAGCTACGAGA
GCACACCCATCCTCCTGGAGCACCTCAAGAGCCACTCGGGGAAGTCATCC
GGTGGCACCAAGGAGAAGAAACACCCTTGTGACCACTGTGACCGCCGCTT
CTACACACGCAAGGACGTGCGGAGGCACATGGTGGTCCACACGGGCCGCA
AAGACTTCCTGTGCCAGTACTGTGCCCAGCGCTTCGGCAGGAAGGACCAC
CTGACACGCCACGTGAAGAAGAGCCACTCACAGGAGCTTCTGAAGATCAA
GACGGAGCCCCCCGACATGTTGGGTCTGCTGGCATCTGGGTCTCCTCCCT
GTGCCGTGAAGGAGGAGCTCAGTCCCATGATGTGCGGCATGGGTCCCAAC
AAAGACCCCATGATGGGCAAACCCTTCCCCGGAGGGGCCCCCTTCCCGAT
GGGCATGTACAACCCCCACCAC------CTTCAGGCCATGTCCAATTCTG
GGGTGGGTCACCCT------CACCCCCCCTCATGCCTGGCTCGCTCTCT
GCAGCTATGGGCATGGNNNNNNNNNNNNNNNNNNNNNNNNNNNNNNNNNNNNNNNNNNNNNNNNNNNNNN
NNNNNNNNNNNNNNNNNNNNNTCGTCAACCTTCTGGCCAGTAACTCCCCGAGCGT
CTCGTACGCTCTGACCCAGCAGAAGTACTTCAGCAACTACAGCCCCGTCA

TCGGCTTCTACATTTACGAGCCCATCGAGTACTGGAACGCCACGGTGCAG
GAGCACCTGAAGACTCTGAGTCACGGCTTCAACAAGATCTCCTGGATGGA
CAACTTTTTCCACTACCTGAAGGTGGTCAACGTGAGCGCGTCCACCAAGA
GCGACTTCATCTCCATCCTCAAGGGCTCCTTCCTGCGCAGCCCCGAGTAC
CAGCACTTCACCGAGGACATCATCTTCACCAAGA---ACCGCGACACCG-
-----ACGAGTACGACATCATCGCCTCGCGCATGTACCTGGTGGCGCGGA
CCACGGAGAAGAAGCGCGAGGAGGTGGTGGAGCTTCTGGAGAAGCTGCGC
CCGCTGATGCTGATCAACAGCATCAAGTTCATCGCCTTCAATCCGACGTT
CGTGTTCATGGACCGCTACAGCTCCTCGGTCATCTCGCCCATCCTGACCT
CGGGCTTCAGCGTGCTCACCATCCTCATCCTCACTTTCTTCCTGGTCATC
AACCCCTTGGAAAACTTCTGNNNNNNNNNNNNNNNNNNNNNNNNNNNNNNNNNNNNNNNNNNNNNNNNNN
NNNNNNNNNNNNNNNNNNNNNNNNNNNNNNNNNNNNNNNNNNNNNNNNNNNNNNNNNNNNNNNNNNNNNN
NNNNNNNNNNNNNNNNNNNNNNNNNNNNNNNNNNNNNNNNNNNNNNNNNNNNNNNNNNNNNNNNNNNNNN
NNNNNNNNNNNNNNNNNNNNNNNNNNNNNNNNNNNNNNNNNNNNNNNNNNNNNNNNNNNNNNGAAAAGGC
TGGAGGACAGTGCCTGCACCTC
AGGCTTTAGGGTTATGATCAAAGAATGTTGTGATGGCATGGGAGACGTGA
GTGAGAAGCACGGAGGAGGTCCAGCTGTTCCTGAGAAGGCAGTACGCTTC
TCTTTCACTGTTATGTCTATTTCTGTTATGGTTGATG------AG-----
-------------------------GAGTACGTCACAATCTTCACTGAGC
CCAAGCCTAACTCAGAACTGTCTTGTAGGCCCCTTTGCCTGATGTTTGTG
GATGAATCAGACCATGAGACACTGACAGCTATCCTGGGGCCTATAGCTGC
AGAGCGTAACGCAATGAAAGACAGCAGACTCATCCTATCCATTGGTGGGT
TGCCTCGCTTCTTCCGCTTCATCTTCAGAGGTACAGGGTATGACGAGAAG
ATGGTTCGGGAAATGGAGGGCCTGGAAGCATCTGGGTCCACCTATGTCTG
TACATTGTGTGACTCCAGTCGAGCAGAGGCCTCTCAAAATATGGTACTGC
ACTCCATCACACGGAGCCATGATGAGAACTTAGAGCGTTATGAAATATGG
CGAACCAACCCATTCTCTGAGACTGTGGACAAGCTGCGAGACAGAGTCAA
AGGAGTATGTGCCAAGCCCTTCATGGAGACCCAGACAACACTTGATGCAT
TACACTGTGACATTGGCAATGCCACAGAGTTCTACAAAATTTTCCAGGAT
GAAATTGGCGAGGTATACAGAAAGGT---CAGT---CCTAGCCGGGAGGA
GAGGCGCAGTTGGAGGGCGGCTCTGGATAAGGAGCTGAGGAGAAAGATGA
AGCTGATACCAGTAATGAGGATGAATGGTAATTATGCACGAAAGCTAATG
TCCATGGAGACCATAGAGGTGGTGTGTGAACTGGTACCCTCTGAAGAGAG
AAGGGAGGCCCTCAGGGAGCTCATGAGGCTATACCTTCAGATGAAGCCCG
TGTGGCGAGCCACCTGTCCAGCACAGGAATGCCCTGACCAGCTGTGCCGC
TACAGCTTTAATTCCCAGCGCTTTGCTGACCTCCTCTCCTCTACTTTCAA
ATATAGGTACAATGGAAAGATAACCAATTATCTGCACAAGACCCTGGCCC
ATGTCCCAGAANNNNNNNNNNNNNNNNNNNNNNNNNNNNNNNNNNNNNNNNNNNNNNNNNNNNNNNNNNN
NNNNTCGTACTCCATCGAGATGGCGCCGCCGGGGCCCCGGTGG
AAGGAGAGCCCGCAGCCCTTCTCTTGTTCCATCGAAGACCCCACCAAGCA
GACCAAGTTCAAGGGGATCAAGACGTACATCTCGTACCGGGTCACGCCGA
GCCACACGGGTCGCCCCGTGTACAGGCGCTACAAACACTTCGACTGGCTG
TACAACCGCCTGCTGCACAAGTTCACTGTGATCTCCGTGCCTCACCTGCC
CGAGAAGCAGGCCACGGGGCGCTTCGAGGAGGACTTCATCGAGAAGCGCA
AGCGGCGACTGATCCTGTGGATGAACCACATGACCAGCCACCCGGTGCTG
TCCCAGTACGAAGGCTTCGAGCACTTTCTGATGTGCGGCGACGACAAGCA
GTGGAAGCTGGGCAAGAGGCGAGCCGAGAAGGACGAGATGGTCGGCGCCC
ACTTCATGCTGACCCTCCAGATACCCAACGAGCACCAGGACCTCCAGGAC
GTGGAGGAGCGGGTGGACTCCTTCAAGGCGTTTGCCAAGAAAATGGACGA
CAGCGTCATGCAGCTCACCCACGTGGCGTCCGAGCTGGTCCGGAAGCACC
TCGGCGGCTTCAGGAAGGAGTTCCAGCGGCTRGGGAACGCGTTCCAGTCC
ATCAGCCAGTCCTTCATGCTGGACCCCCCGCACAGCTCGGAGGCCCTCAA
CAACGCCATCTCCCACNNNNNNNNNNNNNNNNNNNNNNNNNNNNNNNNNNNNNNNNTCATTGGCGTCGGT

GTGGTTGGGAACCTCCTGAT
CTCCATCCTGCTGGTCAAAGACAAGAGCCTGCACCGAGCGCCCTACTATT
TCCTGCTGGACCTGTGCGCCTCTGACATCCTGCGCTCGGCCATTTGCTTC
CCCTTCGTCTTCACCTCGGTCAAGAACGGTTCCGCCTGGACGTACGGCAC
GCTAACCTGCAAAGTAATTGCCTTCCTGGGTGTGCTCTCCTGTTTCCACA
CGGCGTTCATGCTCTTCTGTGTCAGCGTCACCCGCTACCTGGCCATCGCG
CATCACCGGTTTTACACCAAGAGGTTGACCTTCTGGACCTGTCTGGCCGT
CATCTGCATGGTGTGGACATTGTCGGTGGCGATGGCTTTCCCTCCGGTGC
TAGATGTAGGAACGTATTCTTTCATCCAGGAGGAGGACCAGTGCACATTC
CAGCACCGTTCCTTCAGGGCAAACGATTCACTGGGCTTCATGCTCCTCCT
GGCGCTTATCCTCCTCGCCACACAGCTGGTTTACCTCAAGCTCATTTTCT
TCGTCCACGACCGTCGAAAGATGAAGCCCGTCCAGTTTGTGCCTGCAGTC
AGCCAGAACTGGACCTTCCACGGGCCGGGTGCCAGCGGGCAGGCGGCGGC
CAACTGGCTAGCTGGATTTGGTCGAGGCCCCACTCCACCAACTTTGTTGG
GCATCCGGCAGAACAGCAACGCCGCGGGCCGCAGGCGTCTACTGGTATTG
GATGAATTCAAAACGGAGAAGAGGATTAGTAGGATGTTCTACATCATGAC
TTTTTTCTTCCTGGCACTGTGGGGGCCCTATCTGGTAGCCTGCTACTGGC
GGGTGTTTGCAAGGGGCCCTGTAGTCCCCGCGGGTTATCTCACGGCGGCC
GTTTGGATGAGCTTNNNNNNNNNNNNNNNNNNNNNNNNNNNNNNNNNNNNNNNNNNNNNNNNN-------
----

>Forcipiger flavissimus

AGCCTACTTATCCGAGCAGAACTTAACCAACCAGGCTCTCTTCTAGGAGA
CGACCAGATTTACAATGTTATCGTGACAGCTCACGCGTTTGTAATAATTT
TCTTTATAGTAATACCTATCATAATTGGAGGATTCGGCAACTGACTGATC
CCTCTAATAATTGGGGCCCCAGATATGGCCTTCCCCCGAATAAATAATAT
AAGCTTCTGACTACTCCCCCCTTCCTTCTTCCTCCTCCTTGCCTCATCTG
GCGTAGAAGCCGGGGCTGGTACTGGATGAACTGTCTACCCACCGCTCGCT
GGCAACCTTGCCCACGCAGGGGCCTCTGTTGACTTAACAATCTTCTCTCT
ACACCTAGCAGGAATTTCTTCAATTCTTGGAGCCATCAATTTCATTACTA
CCATTATTAACATAAAACCCCCAGCTATAACCCAATATCAGACTCCACTT
TTCGTGTGATCTGTCCTAATCACCGCCGTCTTGCTCCTCCTATCCCTCCC
TGTTCTTGCCGCCGGAATTACAATGCTACTTACAGACCGAAACTTAAATA
CAACTTTCTTTGACCCGGCAGGAGGAGGAGACCCTATTCTTTACCAACAC
CTG---

------------TTCCTAGAGAGAAACCTTCACCCATCTAACTGCCTTGG
CATGCTGTTGCTGTCTGACGCCCACCAGTGCACCAAGCTGTCCGAGCTCT
CCTGGGGCATGTGCCTCGGCAACTTTCCCGCTATTTGCAAGACAGAGGAC
TTCCTCCAACTGCCCAAAGATATGGTGGTGCAGCTTTTGTCACATGAGGA
GCTAGAGACAGAAGATGAGAGACTGGTTTATGAAGCTGCCCTCAATTGGA
TCAACTATGACTTGGAAAAGAGGCACTGCCACCTTCCAGAGCTCCTGAGA
ACGGTCCGTCTCGCCCTGCTGCCTGCCATCTTTCTCATGGAGAACGTATC
TACAGAAGAGCTGATCAATGCCCAGGCCAAGAGCAAGGAGCTGGTGGATG
AAGCTATCCGCTGTAAGCTGAAGATCCTGCAGAATGATGGCGTCGTTAAC
AGCCCATGTGCCCGTCCGAGAAAAACCAGCCATGCCCTCTTTCTCCTGGG
AGGGCAGACTTTCATGTGTGACAAGTTGTACCTGGTGGACCAGAAAGCCA
AAGAGATCATCCCCAAGGCTGACATTCCCAGCCCCAGGAAGGAGTTCAGC
GCCTGCGCCATCGGATGTAAGGTGTACATCACTGGTGGGA--GAGGCTC-
AGAGAATGGTGTGTCCAAAGATGTTTGGGTCTATGATACGGTCCACGAGG
AATGGTCAAAGGCGGCACCCATGCTCATTGCCAGGTTCGGCCATGGCTCT
GCGGAGCTGAAACACTGCCTCTACGTGGTGGGAGGTCACACAGCTGCAAC
TGGCTGCCTCCCGGCTTCCCCGTCTGGATGAATACATTGTTGTGTTTAGT
CGCTCAACAACGAGGCTGATATTGAATGAAGCAGAGTTAATCATGGCACT
WGCCCAGGAGTTCCAGATGAGAGTGGTCACGGTGTCTCTGGAGGAACAGT
CTTTCMCCAGTATAGTCCAGGTGATCAGCAGTGCTACCATGCTGGTCAGT
ATGCATGGAGCTCAGCTCATCACCTCACTCTTCCTTCCAAGAGGAGCTCT
CGTGGTGGAGCTGTTCCCTTTCGCTGTAAACCCAGAGCAGTACACCCCGT
ATAAAACCCTTGCCTCCCTGCCAGGCATGGACCTTCACTACGTTTCTTGG
AGGAACACTAAGGAGGAGAATACCATCACCCATCCAGACAGACCCTGGGA
ACAAGGGGGCATCGCTCACTTGGACAAAGAGGAGCAAGAGCGAATACTGT
TGAGCAAAGATGTCCCCAGACACCTGTGCTGCCGCAACCCGGAGTGGCTC
TTCCGGATCTACCAGGACACTTTGGTGGATATCCCCTCCTTCCTGGAAGT
CCTCAA---AGAGGGCATGAAG---ACCAAGCCCAGCTTGARGAA---GT
CAAAGCCGGCCAACACAGTCCACCCGGGCCGGGTCAGAGAACCCCAGTGT
CAAACCTCAGTACAAACCACTAATGAGGCTAAACTCACAGTCTCCTGGCA
GATCCCGTGGAATCTGAAATACCTTAAGGTCAGAGAAGTGAAATACGAGG
TGTGGATCCAGAAAAAAGATACCAGCAAGGGGACCCTGGAGGATCAAATC
ATCCAGGCGAACCCGGCCTTGGAGGCTTTCGGCAATGCCAAAACATTGAG
AAACGACAACTCGTCTCGTTTTGGAAAATTCATCCGAATTCACTTTGGTA
CGAGCGGCAAGCTGTCGTCTGCTGACATCGAGACGTACCTGCTGGAGAAG
TCACGTGTCACCTTTCAGCTCAAGGCTGAGAGGAATTATCACATCTTCTA
TCAGATCCTGTCGAATCAGAAGCCAGAGCTGCTGGACATGCTGCTGATCA

CCAACAACCCGTATGACTACTCCTACATCTCCCAAGGAGAAGTGACGGTC
GCCTCCATCAACGACTCAGAGGAGCTGATGGCCACGGACAGCGCATTCGA
TGTGCTTGGCTTCACTCCAGACGAGAAGATGGGCGTCTACAAACTGACGG
GCGCCATCATGCACTATGGCAACATGAAGTTCAAACAGAAGCAGCGTGAG
GAGCAGGCTGAGCCGGATGGGACGGAGGCTGCTGATAAATCTGCTTACCT
AATGGGGCTGAACTCTGCTGACCTCATCAAAGGGCTGTGCCACCCCAGAG
TCAAGGTAGGAAATGAATACGTAACCAAAGGCCAAAGTGTGGACCAGGTC
TACTACCCCAACAAGGAGGCCTTCAAGTGTGAGGAGTGTGGGAAGCACTA
CAACACCAAGCTGGGATATAAGCGCCATATGGCCATGCACTCTGCCACGG
CAGGGGATCTCACCTGTAAAGTGTGCATGCAGACCTATGAGAGCACGCCT
GTGCTCTTGGAGCACCTCAAGAGCCACTCCGGGAAGTCTTCAGGTGGCAC
CAAGGAGAAAAAGCACCCGTGTGACCACTGTGACCGTCGTTTCTACACGC
GGAAGGATGTGAGACGGCACATGGTGGTCCACACGGGCCGAAAGGACTTC
CTGTGCCAGTACTGCGCCCAACGCTTTGGCAGAAAGGACCACCTGACACG
CCACGTGAAGAAGAGCCACTCACAGGAGCTGCTGAAAATCAAGACGGAGC
CTCCTGATATGTTAGGTCTTTTAGCTTCCGGGTCACCACCTTGCTCTGTG
AAGGAGGAGCTCAGCCCCATGATGTGCGGCATGGGTCCCAACAAAGACCC
CATGATGGGCAAGCCATTCCCCAGTGGCGCCCCTTTTCAGATGGGCATGT
ACAACCCCCACCAT------CTCCAGGCAATGTCCAATTCTGGGGTGGGT
CACCCA------CACCCGTCCCTGATGCCCAGTTCCTTGTCTGCAGCTAT
GGGCATGGGCTGTCACATGGAATATCTCATCTACGCCTCCTTCTCATTCA
TGGGATGTTTACAAATCAGTGATGGATCAAATATCGTGAACCTGCTGGCT
AGTAACTCTCCAAGTGTTTCGTATGCTCTGACCCAGCAAAAATACTTCAG
TAACTACAGTCCTGTGATTGGGTTTTATATTTATGAGCCCATAGAGTACT
GGAACTCCACAGTGCAGGAGCACCTGAAGACTCTGAGTCATGGCTTCAAC
AAGATCTCCTGGATGGACAACTTTTTCCACTACCTGCGGGTGGTGAATGT
CAGTGCGTCAACCAAGAGCGACTTCATCACCATCCTCAAGGGCTCCTTCC
TGCGCAGCCCGGAGTACCAGCACTTCACTGAGGACATCATATTCTCCAAG
A---ACCGTGAGACTG------ATGAGTATGACATTATCGCCTCACGGAT
GTACTTGGTGGCACGGACGACAGAGAAGAAGCGCGAGGAGGTGGTGGAGC
TTCTGGAAAAGCTTCGTCCGTTGATGCTGATCAACAGCATCAAATTCATT
GCCTTCAATCCTACGTTCGTGTTCATGGACCGATACAGCTCCTCCGTCAT
CTCGCCCATCCTGACCTCAGGATTCAGCGTACTCACAATCCTCATCCTCA
CTTTCTTCCTGGTCATCAACCCCTTGGGGAACTTCTGGCTCATCCTCACT
GTAACGTCCGTGGAGCTGGGTGTCTTAGGTTTGATGGGCTTTCACCAGTT
TGAATGGCAGCCCGCTCTCAAGAATGTGTCTACATCTTGCAATGTTGGCA
TTATTAATGGGCTTTCTGGATGGGCTTCCTCAGTGGATGACTCCCCAGCT
GACACTATCACTCGGCGGTTTCGCTATGATGTGGCACTGGTGTCAGCATT
AAAAGATCTGGAGGAGGACATCATGGAGGGGCTGAGAGAGAGTGGGATGG
AAGACAGCGCTTGCACCTCAGGCTTCAGTGTCATGATCAAGGAATCTTGT
GATGGCATGGGCGATGTCAGCGAGAAGCACGGTGGCGGACCAGTTGTTCC
TGAGAAGGCTGTACGTTTCTCTTTCACTRTTATGTCTGTCTCTATCCTGG
CAGCTGACAGGGAG------------------------------GAAGAG
GTTACCATCTTCACAGAGCCAAAGCCGAACTCAGAATTGTCCTGTAAGCC
CATTTGCCTGACATTTGTGGATGAGTCAGACCACGAGACGCTCACAGCCA
TCCTGTGGCCTCTTGTTGCAGAGCGTAATGCAATGAAAGAGAGCAGGCTC
ATCCTATCCATTGGTGGACTGCTTCGCTCCTTCCGCTTTCACTTCAGAGG
CACGGGATACGATGAGAAGATGGTGCGTGAGATGGAGGGCCTCGAGGCCT
CAGGGTCCACCTATATCTGCACCCTTTGTGACTCCGGTCGGGCAGAAGCT
TCTGAAAACATGGTGCTACACTCCATTACTCGCAGTCATGAAGAGAACCT
AGAACGTTACGAAATATGGAGAACCAACCCCTTCTCTGAGTCTGCAGATG
AGCTGCGAGACAGAGTCAAAGGGGTCTCTGCCAAGCCCTTCATGGAGACT
CACCCAACAATGGACGCATTACATTGCGACATAGGCAATGCCACTGAGTT

CTACAAAATCTTCCAGGATGAGATCGGGGAGGTGTACCAAAAGGT---CA
AC---CCCAGCCGGGAGGAGCGGCGCAGCTGGAGAGCAGCCCTAGATAAA
CAGCTGAGGAAGAAGGTGAAGCTGAAACCAGTAATGAGGATGAACGGGAA
CTTTGCCCGCCGGCTAATGACCATGGAGGCTGTGGAGGCAGTGTGTGAGC
TGGTGCCCTCTGAGTTGAGGAGGGAGGCCCTGAGGGAGCTTATGAGGCTC
TACCTACAGATGAARCCTGTGTGGCGCGCCACCTGCCCAGCCAAGGAGTG
CCCCGACCAGCTGTGCCGCTATAGCTTTAACTCCCAGCGCTTTGCTGACC
TCCTCTCCTCTACCTTCAAATATAGGTACAATGGAAAGATACCCAATTAC
CTGCACAAGACCCTGGCCCATGTGCCTGAAATCATAGAGAGAGATGGATC
CATAGGAGCCTGGGCCAGCGAGGGGAACGAGTCGGCAAACAAATCGTACA
CCATCGAGATGGGTCCCTTAGGGCCCCGGTGGAAGGAGAGCCCACAGCCT
TTCTGCTGCTCCATTGAAGACCCCACTAAACAAACAAAGTTCAAAGGCAT
CAAGACGTACATTTCGTACCGTGTCACGCCRAGCCACACTGGGCATCCTG
TCTACAGGCGCTACAAACACTTTGACTGGCTGTACAACCGCTTACTGCAC
AAGTTCACTGTGATCTCTGTGCCTCACCTGCCTGAGAAGCAGGCCACGGG
GCGATTCGAGGAAGACTTTATCGAGAAGCGCAAGAGGCGACTGATACTGT
GGATGAACCACATGACCAGTCACCCAGTCCTCTCCCAGTATGAAGGCTTC
GAGCACTTTCTGATGTGTGCTGATGACAAGCAGTGGAAACTGGGCAAGAG
ACGGGCGGAGAAGGACGAGATGGTGGGTGCCCATTTCATGCTGACCCTCC
AGATCCCCAACGAGCACCAGGACCTTCAGGATGTAGAGGAGCGGGTCGAC
TCCTTCAAGGCCTTCGCTAAGAAAATGGATGACAGCGTGATGCAGCTCAC
GCATGTCGCCTCGGAGCTGGTGCGTAAGCACCTGGGTGGGTTCAGGAAGG
AGTTCCAGCGACTGGGAAATGCCTTTCAGTCTATCAGCCAGGCGTTCATG
CTGGACCCTCCCCACTGCTCAGAGACCTTCAACAACGCCATCTCCCACNNNNNNNNNNNNNNNNTC
AAACTGACCTCTCTGGGTTTCATCATTGGAGTCGGTGTGGTTGGAAACCT
CCTGATCTCCATCCTGCTGGTCAAAGACAAGAGCCTGCACAGAGCGCCCT
ACTACTTCCTGCTGGACCTGTGCGCCTCTGATATCCTACGCTCCGCCATC
TGCTTCCCCTTTGTCTTCACCTCGGTCAAGAATGGATCTGCCTGGACCTA
CGGCACGCTGACCTGCAAGGTGATCGCCTTTCTGGGTGTCCTCTCCTGTT
TCCACACGGCGTTTATGCTGTTCTGTGTCAGTGTCACACGCTACCTGGCC
ATCGCACATCACCGTTTCTACACCAAGAGGCTGACCTTCTGGACCTGTCT
AGCTGTCATTTGCATGGTGTGGACGTTATCAGTGGCTATGGCGTTCCCGC
CGGTGCTAGACGTAGGGACGTACTCTTTCATCCGGGAGGAGGACCAGTGC
ACCTTCCAGCACCGTTCCTTCAGGGCSAATGATTCGYTGGGCTTCATGCT
CCTGCTGGCGCTCATCCTCCTGGCCACACAGCTGGTTTACCTCAAGCTCA
TCTTCTTCGTCCACGACCGTCGAAAGATGAAGCCCGTCCAGTTCGTGCCT
GCTGTCAGTCAGAACTGGACCTTCCACGGGCCAGGCGCCAGCGGGCAGGC
GGCCGCCAACTGGCTGGCTGGATTTGGTCGAGGCCCCACCCCGCCTACTC
TGCTGGGCATCCGGCAGAACAGCAACGCAGCGGGCCGCCGGCGTCTGCTG
GTATTGGATGAGTTCAAAACAGAGAAGAGGATTAGTAGGATGTTCTACAT
CATGACGTTTTTCTTCCTGGCGCTGTGGGGGCCCTACCTGGTCGCCTGCT
ACTGGCGGGTGTTTGCGAGGGGCCCTGTGGTCCCTGGAGGCTACCTGACA
GCAGCCGTGTGGATGAGCTTTGCCCAGGCTGGGGTCAACCCTTTCATCTG
TATCTTCTCCAACAGGNNNGCCAAATCTCGCTTTCACCCTGGCATGGGGACTG
GTCCTGGCACGGAGC---GCAGCGTCCCACTCGGCAACAGCTTGCTATCC
CCGCAGCAAACCGAGGAGCCCACTGTTGCCACCCCCCCGCAGCGATGGTT
TGTCACCC---CTGCCAACAACCGACTGGACTTTGCTGCCTCGGCATACG
ACGCCGCT---------GATTTCGCCGGTAACGCGGCCACCTTGCTGTCC
TACGCAGCGGCCGGAGTGAAGGCTC------TTCCCCTGCCGACTGCAGG
CTGCTCCAACCGGCCTCTTGGCTATTACGCAGACCCGTCAG---GCTGG-
--GGAGGACGCACGCCGCCGCAGTACTGCGGCGTGAACAGCAAATCCAGC
TCGGTCTTTTCCTGCTGGCCCACTAACTCTATCGGAGGCAGAGCGGGTA-
--CC---AACTACCTGG---------CTGAGGA---GGGA---GACTC--

-CATCCCGACGGAGCGGTCACCG---AT---CGGCGGCTCGGAGGAG---
ACCAAACCCAAAGACATGAC---ATCAGA---GTCGAACTGGATAGAG--
-ACGCCGTCCTCGATTAAGTCCATCGACTCAAGCGATTCTGGTATCTTTG
---AACAGGCCAAAAGGAGAAGGATCTCACCTTCTGCCACGCCA------
---GTTTCAGAGACAGTGTCCCCGCTAAAATCAGAGCATCACTCAACAGG
GGAAGTCACAGAGAGAGAAGTGGCGTTGGGGATAAATCCCTTCGCAGATG
GGATGGGCGCTTTCAAAATAAACCACAGCTCCCACGATATTGGCTCCGG-
--ACAGACGGCGTTTTCCTCCCAGGCG---CCCGGCTAC---GCAGCAGC
CGCCCTGGGA---CACCACCA------CCACCCGACCCACGTTGGCTCT-
--TACTCCACGGCGGCTTTCAACTCCACCAGGGACTTTCTCTTCAGAAAT
CGGGGATTCGGGGACGCCACCGG------------CGCGCAGCACAGTTT
GTTCGCCTC---------CGGAAGTTT---C------GCAGGCCCACATG
GACACTCAGATGCAGCGGGGCACCTGCTCTTTCCGGGGCTCCACGAG---
CAAGCGGCGAGCCATGCGTCTTCCAACGTGGTCAACAGCCAGATGCGGCT
GGGCTTCTCGGGGGACATGTACGGACGGGCCGACCAGTACGGCCACGTTA
CAAGCCCGCGGT---CCGACCACTACGCCTCGACCCAGCTGCACGGCTAC
GGCCCCATGAACATGAATATGGCCGCA---CACCACGGAGCAGGGGCCTT
CTTTCGATACATGAGGCAGCCGATCAAGCAAGAGCTCATCTGCAAGTGGA
TCGAGCCGGAGCAGCTGACAAATCCCAAAAAGTCGTGCAACAAAACTTTT
AGCACGATGCACGAGCTGGTGACCCATCTGACGGTGGAGCATGTGGGGGG
ACCGGAGCAGACCAACCACGTCTGCTTCTGGGAGGATTGCGCCCGAGAAG
GAAAGCCATTCAAAGCCAAATACAAACTTGTAAATCATATCAGAGTACAC
ACCGGAGAAAAGCCCTTTCCGTGTCCGTTCCCCGGCTGTGGCAAA
>Gadella jordani
AGTCTTCTTATCCGAGCCGAATTAAGCCAGCCCGGTGCCCTTTTCGGTGA
CGACCAAATTTATAACGTAATCGTCACGGCCCATGCTTTCGTAATAATCT
TTTTTATAGTTATACCCATTATGATCGGTGGATTTGGAAACTGATTAATT
CCACTAATGATTGGAGCCCCCGACATAGCCTTCCCCCGTATAAATAATAT
AAGCTTCTGACTCCTCCCCCCCTCTTTCCTATTACTCTTAGCATCCTCAG
GAGTAGAAGCAGGAGCAGGGACAGGATGAACTGTCTATCCCCCATTAGCG
GGTAACCTGGCCCACGCAGGTGCTTCCGTAGACTTGACCATCTTTTCCCT
ACATTTAGCAGGAGTTTCCTCAATTCTAGGGGCAATCAACTTTATTACAA
CTATTATTAATATGAAACCACCCGCAGTGTTGCAATATCAAACACCTTTA
TTCGTCTGATCCCTCCTAATTACAACTGTTCTTCTTCTTTTAGCACTTCC
TGTTTTAGCCGCCGGAATTACAATACTTCTTACAGACCGTAACCTTAACA
CCTCTTTCTTCGACCCTGCTGGAGGAGGAGACCCCATCTTATACCAACAC
TTATTCTGATTCTTTGGCCACCCCGAAGTCTACATTTTAATTCTTCCAGG
CTTTGGAATGATCTCCCATATCGTAGCATATTATTCAGGTAAAAAAGAGC
CCTTCGGTCACATGGGAATAGTATGAGCTATAATAGCAATTGGTTTCCTT
GGCTTCATCGTTTGAGCCCACCATATGTTTACCGTTGGGATAGACGTTGA
CACACGGGC---TTCCTCGAGAGGAACCTTCACCCGACCAACTGCCTTGG
CATGCTGCTGCTGTCCGACGCCCACCAGTGCACCAAGCTTTCCGAGCTCT
CTTGGAGCATGTGCCTGAGCAACTTCCCTGCCATATGCAAGACAGAGGAT
TTTCTCCAACTGCCCAAAGATATGGTTGTGCAACTTTTATCCCACGAGGA
GCTGGAGACAGAAGAYGAGAAACTTGTTTACGAGGCCGCACTCAACTGGG
TCACCTATGACCTGCAAGGGAGGCACCCCCACTTGCCCGAACTGCTGAAA
ACAGTGCGTCTGGCTCTTCTCCCCGCCATCTTCCTCATGGAAAATGTCTC
CACGGAAGAGCTGATAAACACCCAGAGCAAGAGCAAGGAGCTGGTGGACG
AGGCCATATGCTGCAAGCTGAGGATCCTGCAGAAYGACGGCGTGGTCAAC
AGCCCGTGCGCCCGTCCGAGGAAGACCAGCCATGCGCTCTTTCTGCTGGG
AGGGCAGACCTTCATGTGCGACAAGCTGTACCTGGTGGACCAGAAAGCCA
AAGAGATCATCCCCAAGGCCGACATACCCAGCCCGCGGAAGGAGTTCAGC
GCCTGTGCCATCGGCTGCAAGGTGTACATCACGGGCGGGC--GGGGCTC-

TGAGAACGGCGTGTCCAAAGACGTGTGGGTGTACGACACGGTGCAGGAGG
AGTGGTCMAAGGCGGCGCCCATGCTCATTGCCCGCTTCGGCCACGGCTCC
ACCGAGCTGAAGCACTGCCTGTACGTGGTGGGCGGTCACACGGCCGCCAC
CGGCTGCCTCCCCGCCTCCCCGTCCGGAGGATTACATTGTGGTCTTCTCC
CGATCCACAACGAGGTTGATCCTGAATGAAGCAGAGCTGATCATGGCACT
GGCCCAGGAGTTTCAGATGAGAGTCTTYACGGTGTCCCTGGAGGAGCAGT
CTTTTGCCAGCATAGTGCAGGTGATCAGCGGAGCCTCCATGCTGTTCAGT
ATGCATGGCGCTCAGCTCGTCACGTCATTGTTTCTGCCCAGAGGTGCAGC
TGTGGTGGAGCTGTTCCCTTATGCCGTTAACCCAGAGCAGTACACCCCAT
ATAAAACCCTAGCCTCCCTGCCAGGCATGGACCTCCAATATGTTTCCTGG
AGCAACACGATGGAGGAGAACACTGTCACTCATCCGGACCGGCCCTGGGA
CCAAGGGGGCATTGTGCACTTGGAGAAGGAGGAACAAGAGCGCATACAAG
CCAGCAAGGATGTCCCCAGGCACCTGTGCTGTCGTAACCCAGAGTGGCTT
TTCAGAATCTATCAGGACACTTTGGTGGACATCCCTTCACTCCTAGAGGT
AATCAA---GGACGGCCTCAAA---ACCAGACCCAACTTGAAAAA---GG
GCAAGGCGGCCAGCACAGTTCACCCTGGGCGAGTCAGAGAGCCCCAGTGC
CAAACCTCGGTCCAAGCCACCAACGAGGCCAAA-----------------

-----------AAGAGGGATGCCAGCAAAGGAACCCTGGAGGATCAAATC
ATCCAGGCCAACCCTGCCCTGGAGGCCTTCGGTAATGCCAAAACCTTGAG
AAATGATAACTCCTCYCGTTTTGGAAAGTTCATTCGGATTCACTTTGGGA
ACAGTGGTAAGCTGAGCTCTGCAGACATTGAGACCTACCTGCTGGAAAAG
TCCAGAGTCACTTTCCAGCTGAAGTCAGAGAGGAATTATCACATATTCTT
CCAGATCTTGTCCAATCAGAAGCCGGAGCTGCTGGACATGCTGCTGATAA
CCAATAACCCATACGATTACTCCTACATCTCCCAAGGAGAGGTAACCGTA
GCCTCCATCAACGACTCTGAGGAGCTCATGGCCACTGACAATGCATTTGA
CGTCCTTGGCTTCACCCTGGAAGAAAAGATGGGGGTGTACAAACTGACTG
GTGCCATCATGCACTACGGCAACATGAGGTTCAAGCAGAAGCAGCGTGAG
GAGCAAGCAGAGTCAGACGGGACTGAAGCAGCGGACAAATCAGCGTACCT
CATGGGTTTGAACTCTGCAGACCTCATCAAAGGCCTCTGCCACCCCAGGG
TGAAGGTAGGAAATGAATATGTCACCAAAGGTCAAGGGGTGGACCAAGTC
TACTAC--
----------------------------TGTGGCCATGCACTCGGCCACCG
CGGGGGACCTGACGTGCAAGGTGTGCTTGCAGAGCTACGAGAGTACGCCC
GTGCTGCTGGAGCACCTCAAGAGCCACTCGGGGAAGTCCTCGGGCGGCGC
CAAGGAGAAGAAGCACCCCTGCGACCACTGCGACCGCCGCTTCTACACCC
GCAAGGACGTGCGCCGGCACATGGTGGTGCACACGGGCCGCAAGGACTTC
CTGTGCCAGTACTGCGCCCAGCGGTTCGGCCGCAAGGATCACCTGACGCG
GCACGTGAAGAAGAGCCACTCGCATGAGCTGCTCAAGATCAAGACGGAGC
CTCCGGACATGCTGGGACTGCTGAGCTCCGGGTCGCCCCCTTGCTCCATC
AAGGAAGAGCTCAGCCCCATGATGTGCAGTATGGGTCCCAATAAAGACCC
CATGATGGGGAAGCCCTTCCCCAGCGGCACTCCCTTCCCCATGGGCATGT
ACAACCCCCATCAC------CTCCAGGCAATGTCTAATTCCGGGGTGGGT
CACCCT------CACCCTTCCCTGATGCCTGGTTCACTGTCTGCGGCAAT
GGGCATGGGCTGTCACATGGAG----------------------------

---TCGTACA
CCATCGAGATCGGCCCCAAGGGCCCCCAGTGGAAGGCGAACCCCCAGCCC
TTCTCCTGCTCCATCGAGGACCCCACCAAACAGACAAAGTTCAAGGGCAT
CAAGACCTACATCTCGTACCGGGTGACGCCCAGCCACACCGACAGGGCCG
TGTACAGGCGCTACAAACACTTTGACTGGCTGTACAACCGCCTGCTGCAC
AAGTTCACCGTGATCTCCGTGCCCCACCTACCCGAGAAGCAGGCCACKGG
GCGCTTCGAGGAGGACTTCATCGACAAGCGCAAGCGACGGCTCATCCTGT
GGATGAACCACATGACCAGCCACCCGGTGCTGTCGCAGTACGAGGGCTTC
GAGCACTTCCTCATGTGCGGCGACGAGAAGCAGTGGAAGCTGGGCAAGAG
GCGGGCGGAGAAGGACGAGATGGTGGGCGCCCACTTCATGCTCACCTTCC
AGATCCCCAACGAGCACCAGGACCTGCAGGACGTGGAGGAGCGTGTGGAC
TCCTTCAAGTGCTTCACCAAGAAGATGGACGACAGCGTCATGCAGCTGAC
GCACGGCGCCTCCGAGATGGTGCGGAGGCACCTGGGAGAGTTCCGGAAGG
AGTTCCACCGGCTCGGGACCGCCTTCCAGTCCCTGTCGCAGGCCTTCATG
TTGGAACCCCCGCACAGCTCGGAGCCACTCAACAACGCCTTCTCGCAC--

>Gadus morhua
AGCCTGCTCATTCGAGCAGAGCTAAGTCAACCTGGTGCACTTCTTGGTGA
TGATCAAATTTATAATGTGATCGTTACAGCGCACGCTTTCGTAATAATTT
TCTTTATAGTAATACCACTAATAATTGGAGGCTTTGGGAACTGACTCATT
CCTCTAATGATCGGTGCACCAGATATAGCTTTCCCTCGAATAAATAACAT

AAGCTTCTGACTTCTTCCTCCATCTTTCCTGCTCCTTTTAGCATCCTCTG
GTGTAGAAGCTGGGGCTGGAACAGGCTGAACTGTCTATCCACCTTTAGCC
GGAAACCTCGCTCATGCTGGGGCATCTGTTGATCTCACTATTTTTTCTCT
TCATCTAGCAGGGATTTCATCAATTCTTGGGGCAATTAATTTTATTACCA
CAATTATTAATATGAAACCTCCGGCAATTTCACAGTACCAAACACCCCTA
TTTGTTTGAGCAGTACTAATTACAGCTGTGCTTCTACTATTATCTCTCCC
CGTCTTAGCAGCTGGTATCACAATACTTCTAACTGACCGTAATCTTAATA
CTTCTTTCTTTGACCCTGCTGGAGGAGGTGATCCCATTTTATACCAACAC
TTA---

------------TTCCTGGAGAGGAACCTCCACCCGACCAACTGCCTGGG
CATGCTGCTGCTTTCCGACGCCCACCAGTGCATCAAGCTCTCGGAGCTGT
CCTGGAGCATGTGCCTGAGCAACTTCCCCGCCATATGCAAGACGGAGGAG
TTTCTCCAACTGCCCAAAGACATGTTTGTGCAGCTTTTGTCCCACGAGGA
GCTGGAGACGGAGGACGAGAAACTTGTTTACGAGGCCGCGCTTAACTGGG
TCACCTACGACCTGCAAGGAAGGCACCCCCACTTGCCGGAGCTTCTGAAA
ACGGTGCGTCTGGCCCTTCTCCCCGCCGTCTTCCTCATGGAGAACGTCTC
CACCGAGCCACTGATAAACAGCCAGACCAAGAGCAAGGAGCTGGTGGACG
AGGCCATCTGCTGCAAGCTGAAGATCCTGCAGAACGACGGCGTGGTCAAC
AGCCCGTGCGCCCGTCCCAGGAAGACCAGCCACGCGCTCTTCCTGCTGGG
AGGCCAGACCTTCATGTGCGACAAGCTGTACCTGGTGGACCAGAAAGCCA
AAGAGATCATCCCCAAGGCCGACATCCCGAGCCCCAGGAAGGAGTTCAGC
GCCTGCGCCATCGGCTGCAAGGTGTACATCACGGGCGGGC--GGGGCTC-
GGAGAACGGCGTGTCCAAAGACGTGTGGGTGTACGACACGGTGCAGGAGG
AGTGGTCCAAGGCGGCGCCCATGCTGATCGCCCGCTTCGGCCACGGCTCG
GCGGAGCTGAAGCACTGCCTGTACGTGGTGGGCGGGCACACGGCCGCCAC
GGGCTGCTTCCCGGCCTCTCCGTCCGGAGGATTACATCGTGGTTTTCTCC
CGCTCCACCACGAGGTTGATCCTGAATGAGGCGGAGTTGATCATGGCACT
AGCCCAGGAGTTTCAGATGAGGGTCTTGACGGTGTCGCTGGAGGAGCAGT
CGTTTGCCAGCATAGTGCAGGTGATCAGCGGGGCGTCCATGCTGTTCAGC
ATGCACGGCGCTCAGCTCGTCACGGCACTGTTCCTACCCCGAGGGGCCGC
CGTGGTGGAGCTGTTCCCCTATGCGGTGAACCCTGAGCAGTACACCCCGT
ATAAGACCCTGGCCTCCCTGCCGGGCATGGACCTGCAGTACGTCTCCTGG
AGGAACTCCATGGAGGAGAACACGGTCACCCATCCGGAACGGCCCTGGGA
CCAAGGGGGGATCGTGCACCTGGAGAAGGAGGAGCAGGAGCGCATACAGG
TCAGCAAGGACGTCCCCAGGCACCTGTGCTGTCGGAACCCAGAGTGGCTT
TATAGGATCTACCAGGACACTTTGGTAGACATCCCTTCACTCCTAGAAGT
GCTGAA---GGAGGGCCTCAAA---ACCAGGCCCAGCGTGAAAAA---GA
ACAAGGCGGCCAGCACCGTTCACCCAGGGCGAGTCAGAGAACCCCAATGC
CAGACCTCTGTCCAGGCCACCAACGAGGCCAAACTCACAGTGTCCTGGCA
GATCCCGTGGAACCTGAAGTTCCTGAAGGTGCGCGAGGTGAAGTATGAGG
TGTGGATACAGAAGAAGGATGCCAACAAAGGGACTCTGGAGGATCAAATC
ATCCAGGCCAACCCTGCGCTAGAGGCCTTCGGTAATGCCAAAACCCTGAG
GAATGACAACTCCTCTCGTTTTGGAAAGTTCATCCGGATTCACTTCGGGA
ACAGCGGCAAGCTTAGCTCTGCAGACATCGAGACCTACCTGCTTGAAAAG
TCCAGAGTCACTTTCCAGCTGAAGTCTGAGAGGAACTATCACATATTCTT
CCAGATCTTGTCCAATCAGAAGCCGGAGCTGTTGGACATGCTGTTAATTA
CCAACAACCCCTACGATTACTCCTACATCTCCCAAGGAGAGGTAACCGTC
GCCTCCATCAACGACTCTGAGGAGCTCATAGGCACTGATAGTGCGTTTGA
TGTCCTCGGCTTCACCCTGGAAGAAAAGATGGGGGTGTACAAGCTGACTG
GTGCCATCATGCACTATGGCAACATGAGGTTCAAGCAGAAGCAGCGCGAG

GAGCAGGCAGAGTCAGACGGGACTGAAGCGGCTGATAAATCAGCATACCT
CATGGGTCTGAACTCGGCGGATCTCATCAAAGGTCTCTGCCACCCTAGGG
TGAAGGTAGGGAATGAATATGTCACCAAAGGTCAAGGGGTGGATCAGGTC
TACTACCCCAACAAAGAGGCCTTCAAGTGCGAGGAGTGCGGCAAGCACTA
CAACACCAAGCTGGGCTACAAGCGCCACGTGGCCCTGCACTCGGCCACCG
CCGGGGACCTGACCTGCAAGGTGTGCCTGCAGGGCTACGAGAGCACGCCA
GTGCTGCTGGAGCACCTCAAGAGCCACTCGGGGAAGTCGTCTGGCGGCGC
CAAGGAGAAGAAGCACCCGTGCGACCATTGCGACCGCCGCTTCTACACCC
GCAAGGACGTGCGCCGGCACATGGTGGTGCACACGGGCCGCAAGGACTTC
CTGTGCCAGTACTGTGCCCAGCGCTTCGGCCGCAAGGACCACCTGACGCG
GCACGTGAAGAAGAGCCACTCGCAGGAGCTGCTCCGGATCAAGACGGAGC
CCCCAGACATGCTGGGCCTGCTGAGCTCCGGGTCACCGCCCTGCTCCATC
AAGGAGGAGCTCAGCCCGATGATGTGTGGGATGGGTCCCAATAAAGACCC
CATGATGGGGAAGCCCTTCCCCAGTGGCACTCCCTTCCCCATGGGCATGT
ACAACCCCCATCAC------CTGCAGGCGATGGCCAATCCCGGGGTGGGT
CACCCT------CACCCGTCCCTGATGCCCGGCTCCCTGTCTGCTGCGAT
GGGCATGGGCTGTCACATGGAG----------------------------

------------------------------------GGGTTCCACGCCTT
CGAGTGGCAGCCGGCGCTGGYGAGCGTCTCGCCGTCCTGCCACGTGGGCA
TCATCGAYGGGCTCTCGGGCTGGGCGGCCTCGGTGGACGACGCCCCCGCC
GACACCATCACCCGCCGCTTCCGCTACGACGTGGCGCTGGCGTCGGCGCT
CAAGGACCTGGAGGAGGACATCATGGACGGGCTGAGGCAGCAAGGGCTGG
AGGACAGCGCCTGCACCGAGGGCTTCTCCGTCATGATCAAGGAGTCCTGC
GACGGCATGGGGGACGTGAGCGAGAAGCACGGCGGGGGGCCGGCCGTCCC
GGAGAAGGCGGTGCGTTTCTCCTTCACCGTGATGTCCGTCTCCTTGCGCG
TGGCCGACGACGGAGAGGAAGCGGGGAA------------CGCTGAGGAG
GTGATCATCTTCCAGGAGCCGAAGCCCAACTCTGAGCTGTCCTGTAAGCC
ACTGTGCCTCATGTTCGTGGACGAGTCGGACCACGAGACGCTGACCGCCG
TCCTGGGGCCCGTGTCGGCCGAGCGCAGCGCCATAAAGCGCAGCCGCCTC
ATCCTCTCCATCGGCGGGCTTTCTCGGCTCTTCAGCTTCCGCTTCCGGGG
ATCGGGCTACGACGAGAAGATGGTGCGGGACGTGGAGGGCATGGAGGCCT
CCGGCTCCACCTACGTCTGCACGCTCTGCGACTCCACCCGGGCCGATGCC
GCCCGCAACATGGTGCTCCACTCCGTCACCCGCAGCCACCAGGAGAACCT
GGAGCGCTACGAGACCTGGAGGACCAACCCCTTCTCGGAGTCCGCCGACG
AGCTTAGGGACCGGGTGAAGGGGATCTCCGCCAAGCCCTTCCTGGAGACC
CACCCCACGCTGGACGCGCTGCACTGCGACATCGGCAACGCCACCGAGTT
CTACAAGATCTTCCAGGACGAGATCGGCGAGGTGTTCCAGAAGAC---CA
AC---CCCAGCCGGGAGGAGCGGCGGAGCTGGAGGGCGGCTCTGGACAAG
CAGCTGAGGAAGACCATGAAGCTCAAGCCGGTGATGAGGATGAACGGCAA
CTACGCCCGCAAGCTGATGAGCGTGGAGGCGGTGGAGGCGGTGTGCGAGC

TGGTGCCGTCGGAGAGCCGGCGCCAGGCCCTGAGGCAGCTGATGGAGATC
TACCTGGAGATGAAACCGGTGTGGCGCGCCACCAGCCCAGCCACGGAGTG
CCCCGACCAGCTGTGCCGCTACAGCTTCAACTCCCAGCGCTTCGCGGACC
TCCTGGCCTCCACGTTCAGCTACAGGTACAACGGGAAGATCACCAACTAC
CTGCACAAGACCCTGGCCCACGTGCCGGAGATCATAGAGAGGGACGGCTC
CATCGGGGCCTGGGCGAGTGAGGGGAACGAGTCGGCCAACAAATCGTACA
CCATCGAGATCGGCCCCAAGGGGCCCCAGTGGAAGGAGAGCCCCCAGCCC
TTCTCCTGCTCTGTCGAGGACCCCACCAAACAGACTAAGTTCAAGGGCAT
CAAGACCTACATCTCGTACCGGGTGACGCCCAGCCACACCGACGGCGCCG
TGTACCGGCGCTACAAACACTTTGACTGGCTGTACAACCGGCTGCTGCAC
AAGTTCACCGTGATCTCGGTGCCCCACCTGCCCGAGAAGCAAGCCACGGG
CCGCTTCGAGGAGGACTTCATCGACAAGCGCAAGCGGCGTCTCATCCTGT
GGATGAACCACATGACCAGCCACCCGGTGCTGTCGCAGTACGAGGGCTTC
GAGCACTTCCTCATGTGCGCCGACGACCAGCAGTGGAAGCTGGGCAAGCG
GCGGGCGGAGAAGGACGAGATGGTGGGCGCCCACTTCATGCTCACCTTCC
AGATCCCCAACGAGCACCAAGACCTGCAGGACGTGGAGGAGCGCGTGGAT
TCTTTCAAGACTTTCACCAAGAAGATGGAAGACAGCGTCATGCAGCTGAC
CCACGGCGCTTCCGAGATGGTGCGGAGGCACCTGGGAGAGTTCCGGAAGG
AGTTCCACCGGCTCGGCGGAGCCTTCCAGGCGCTGTCGCAGGCGTTCATG
CTGGAGCCGCCGCACAGCTGGGAGCCGCTCAACAACGCCATCTCGCATCC
GCTCGCCACGTTCCTCAAACTGACCTCGCTGGGCTTCATCATCGGCGTCG
GCGTGGTCGGCAACCTCCTGATCTCCATCCTGCTGGTGAAAGACAAGAGC
CTGCACCGGGCGCCCTACTACTTCCTGCTGGACCTGTGCGCCTCCGACAT
CCTGCGCTCGGCCATCTGCTTCCCGTTCGTCTTCACCTCGGTGAAGAATG
GCTCGGCGTGGACGTACGGCACGCTCACCTGCAAAGTGATCGCCTTCCTG
GGCGTGCTCTCGTGTTTCCACGCGGCGTTCATGCTCTTCTGCGTGAGCGT
CACGCGCTACCTGGCCATCGCCCACCACCGCTTCTACACCAAGCGGCTCA
CCTTCTGGACGTGCCTGGCCGTGGTGTGCATGGTGTGGACGCTGTCGGTG
GCCATGGCCTTCCCGCCCGTGCTGGACGTGGGCACGTACTCGTTCATCCG
CGAGGAGGACCAGTGCACCTTCCAGCACCGCTCGTTCCGCGCCAACGACT
CGCTGGGCTTCATGCTCCTGCTGGCCCTCATCCTGCTCGCCACGCACCTG
GTCTACCTCAAGCTCATCTTCTTCGTGCACGACCGGCGGAAGATGAAGCC
CGTGCAGTTCGTGCCGGCCGTCAGCCAGAACTGGACCTTCCACGGCCCCG
GGGCCAGCGGGCAGGCGGCGGCCAACTGGCTGGCGGGCTTCGGCCGGGGC
CCCACGCCGCCCACCCTGCTGGGCATCCGGCAGAACAGCAACGCGGCCGG
GCGGCGCCGGCTGCTGGTGCTGGACGAGTTCAAGACGGAGAAGCGGATTA
GCAGGATGTTCTACATCATGACCTTTTTCTTCCTGGCTCTGTGGGGCCCC
TACCTGGTGGCCTGCTACTGGCGGGTGTTTGCCAGGGGCCCCGTGGTCCC
CGGGGGGTACCTGACGGCGGCCGTGTGGATGAGCTTCGCCCAGGCAGGGG
TGAACCCCTTCATCTGCATCTTCTCCAACCGCGAG---------------


```
--------------------------------------------------
-----CATCACTCATCAGCCGAAGTCACAGAAAGAGAAGTCGCGTTGGGG
ATAAACCCGTTCGCAGACGGAATGGGCGCCTTCAAAATAAACCACAGTTC
TCACGACATTGGCTCCGG---ACAAACTGCGTTTTCCTCCCAGGCG---C
CCGGCTAC---GCAGCGGCTGCCCTCGGA---CACCACCA------TCAC
CCCACCCATGTTAGCTCT---TACTCCACCGCCGCCTTCAACTCTACACG
GGACTTTCTTTTCAGAAACCGGGGCTTCGGAGACGCCACCAG--------
----CGCGCAGCACAGTTTGTTCGCCTCCGC---CGCGGGAAGTTT---T
------GCAGGGCCACATGGACACTCCGATGCCACGGGACACCTGCTCTT
CCCCGGGCTGCACGAG---CAAGCGGCGAGCCACGCGTCTTCCAATGTGG
TCAACAGCCAGATGCGACTGGGCTTTTCCGGAGACATGTACGGCCGGGCC
GACCAGTACGGCCACGTTACGAGCCCGAGGT---CCGACCACTACGCGTC
GACCCAGTTGCACGGTTATGGCCCTATGAACATGAATATGGCCGCG---C
ACCACGGAGCCGGGGCCTTCTTTCGGTACATGAGGCAGCCGATCAAGCAA
GAGCTGATCTGCAAGTGGATCGAGCCGGAGCAACTGTCAAACCCCAAAAA
GTCGTGCAACAAAACTTTCAGCACGATGCACGAGCTGGTGACCCATCTCA
CGGTGGAGCACGTGGGGGGACCAGAACAGTCGAACCACATCTGTTTCTGG
GAAGAGTGTTCCCGAGAAGGGAAACCGTTCAAAGCAAAATACAAACTGGT
GAATCATATCAGAGTGCACACCGGAGAAAAGCCCTTCCCGTGTCCCTTCC
CCGGCTGTGGCAAA
>Galaxias maculatus
AGCCTGTTAATTCGGGCAGAGCTAAGCCAGCCAGGTGCTCTTTTAGGTGA
CGACCAGATTTATAATGTAATCGTCACAGCACACGCCTTCGTAATAATTT
TCTTTATAGTAATACCTATCATGATTGGAGGCTTTGGAAACTGGCTAATT
CCACTTATGATTGGGGCTCCGGATATAGCATTTCCCCGTATGAACAACAT
AAGCTTTTGGCTACTCCCCCCCTCTTTTCTTCTTCTTTTAGCTTCCTCTG
GCGTTGAAGCCGGGGCTGGTACGGGCTGAACTGTTTACCCCCCTTTAGCA
GGTAATCTTGCTCATGCTGGAGCTTCCGTAGACCTGACTATCTTTTCCCT
ACACTTAGCAGGGATCTCTTCAATCTTAGGTGCAATTAACTTCATTACCA
CAATTATTAACATGAAACCACCTGCTATCTCCCAATATCAGACCCCTTTA
TTTGTATGGGCAGTCTTAATTACTGCCGTCCTTCTTCTTCTTTCTTTACC
CGTTCTTGCTGCGGGAATTACGATACTATTGACGGACCGGAACCTAAATA
CGACTTTCTTCGACCCTGCCGGTGGGGGAGATCCTATTCTGTACCAGCAT
TTGTTTTGGTTCTTTGGCCACCCCGAAGTTTACATTCTTATTCTTCCGGG
ATTCGGAATAATCTCTCATATCGTTGCATACTACTCTGGAAAGAAAGAAC
CTTTTGGTTATATGGGAATAGTATGAGCTATGATAGCGATTGGTCTTCTA
GGGTTTATCGTCTGGGCCCACCATATGTTTACTGTGGGAATAGATGTAGA
CACCCGAGNNNN------------------------------------------
--------------------------------------------------
--------------------------------------------------
--------------------------------------------------
--------------------------------------------------
--------------------------------------------------
--------------------------------------------------
--------------------------------------------------
--------------------------------------------------
--------------------------------------------------
--------------------------------------------------
--------------------------------------------------
--------------------------------------------------
--------------------------------------------------
--------------------------------------------------
--------------------------------------------------
```

NNNNNNNNNNNNNNNNNNNNNNNNNNNNNNNNNNNNNNGGCTCATCCTCAACGAGGCAGAGGTCATC
CGGGCGCTGGCCCAGGAGTTCCAGATGAGGGTGGAGACGGTGTCCCTGGA
GGAGCAGTCGTTCCCCGCCATCGTCCAGGTCCTCAGCAGGGCCTCCATGT
TGGTGAGCATGCACGGCGCCCAGCTCATCACCTCGCTCTTCCTCCCGCGC
GGGGCGGTGGTGGTGGAGCTCTTCCCTTACGCCGTCAACCCCGAGCAGTA
CACGCCCTACAAGACGCTGGCCTCCCTGCCCGGCATGAACCTCCACTACG
CGGCCTGGAGGAACTCTCTGGAGGAGAACACGGTGGCCCACCCGGACAGG
CCCTGGGACCAGGGGGGCATCGCTCACCTGGACAAGGAGGAGCAGGACCG
CATCCTGGCCAGCCAGGACGTCCCACGGCACCTGTGCTGCCGCAACCCGG
AGTGGCTGTACCGGATCTACCAGGACACCAAGGTGGACGTTCCCTCTCTC
CTGGAGCTCCTCCG---GGAGAACCTAAAG---ACCCGGCCCAGTCTGAG
GAA---GGCCCGGCCGGTCAGCACCGTCCACCCGGGTCGGGTCCGCCAGG
CCCAGTGCCTGACGTCCGTCCAGGCCGCCAACGAGGCCAAGCTGACGGTG
TCGTGGCAGATCCCGTGGAACCTGAAGTACCTGAAGGTCCGGGAGGTGAA
GTNNNNNNNNNNNNNNNNNAAGAGGGATACAAGTAAGGGGACCCTGGAGGATCAAATAATTCAGGCA
AATCCTGCACTGGAGGCTTTTGGTAATGCCAAAACGTTAAGAAATGACAA
CTCATCCCGCTTTGGTAAATTCATCAGAATTCACTTTGGAACTACTGGAA
AATTATCCTCTGCTGACATAGAGACTTACCTTCTTGAGAAATCGCGGGTT
ACCTTTCAGCTAAAGTCAGAGAGAAACTACCATATTTTTTTCCAGATCTT
ATCCAATCAAAAGCCAGAGTTGTTGGACTTGCTGTTAATCACCAACAATC
CCTATGATTATTCCTACATCTCCCAGGGAGAGGTGACAGTACAGTCAATT
AATGATGCAGAAGAGCTAATGGCCACTGACAGTGCCTTTGATGTGCTGGG
CTTTACTCAGGAGGAAAAATTGGGTGTCTACAAGTTGACAGGTGCTATCA
TGCACTACGGCAACATGAGGTTCAAGCAAAAGCAGCGTGAAGAGCAGGCC
GAGCCAGATGGTACAGAAGCTGCTGACAAATCAGCTTACCTGATGGGTCT
CAATTCTGCAGACCTGATTAAAGGGCTTTGTCATCCCCGAGTCAAAGTAG
GTAATGAATACGTCACCAAAGGTCAAGGAGTAGATCAAGTCTACTAC---

---------------------------GGCTACCACGACTTCGAGTGGCA
GCCGGCCCTGAGGAACGTKTCCCCGGACTGCCACGTGGGCATCGTCGACG
GCCTCTCGGGCTGGGCGGCGTCGGTGGACGACTCCCCGGCGGACACCATC
ACCCGCCGCTTCCGCTACGACGTGGCCCTGGCGTCCGCCCTGAAGGACCT
GGAGGAGGACATCCTGGCGGGCCTGAGGGAGCGCGGCCTGGAGGACGGCG
CCGCCGCCACCCACTTCAGCGTCATGGTCAAGGAGTCCTGCGACGGCATG
GGCGACGTGAGCGAGAAGCACGGCGGCGGGCCGGCCGTCCCGGAGAAGGC
GGTGCGCTTCTCCTTCACCGTCATGTCCGTCTCCGTCCAGGCGGAGGGGC
GGGAG------------------------------GAGGCCGTGACGGTG
TTCAGGGAGGCCAAGCCCAACTCTGAGCTSTCCTGCAAGCCCCTGAGYCT
GATGTTCGTGGACGAGTCAGACCACGAGACGCTCACGGCCATCCTGGGCC
CGGTGGTGGGCGAGCGGGACGCCATGAAGCGGAGCCGGCTCCTSYTGACC
CTGGGGGGCCTCCAGCGGTCCTTCCGCTTTCACTTCCGGGGCAGCGGCTA
CGAYGAGAAGATGGTGCGCGAGATGGAGGGGCTGGAGGCCTCGGGCTCCA
CCTACATCTGCACCCTGTGCGACTCCACGCGGGCGGAGGCCTCGTCCAAC
ATGGTGCTCCACTCCATCACCCGCAGCCACGAGGAGAACCTGGAGCGCTA
CGAGGTGTGGAGGACCAACCCGTACGCCGAGTCGGCCGACGAGCTGCGAG
ACCGGGTGAAGGGGGTCTCCGCCAAACCCTTCCTGGAGACCCAGCCCACC
CTGGACGCGCTGCACTGCGACATCGGCAACGCCACAGAGTTCTACAAGAT
GTTCCAGGACGAGATCGGGGAAGTGTTCCGGAAGCC---CAAC---CCCA
GTCGGGAGGAGAGGCGCAGCTGGCGGGCGGCCCTGGACAAACARCTCAGG
AAGAAGCTGAAGCTGAAACCGGTYATGCGCATGAACGGGAACTACGCCCG
CCGCCTGATGACCCAGGAGGCGGTGGAGGTGGTGTGTGAGCTGGTCCCCT
CGGAGGAGAGGCGGCTGGCCCTCCAGGAGCTCATGGGCCTGTACATCCAG
ATGAAGCCGGTGTGGCGGGCCTCCTGCCCAGCCAAGGAGTGCCCGGACCA
GCTGTGTCGCTACAGCTTCAACTCGCAGCGCTTCGCCGAGCTCCTCTCCA
CCACSTTYAAGTACAGATACGACGGCAAGATCACCAACTACCTCCACAAA
ACGCTGGCCCATGTGCCTGAGATCATAGAGAGGGACGGGTCCATTGGAGC
GTGGGCCAGTGAGGGGAATGAGTCGGGTAACAAG----------------

---------------------------------------NNNNNNNNNNNNTTCCTTAAACT
GACCTCTTTGGGTTTTATCATTGGTGTTGGCGTGGTCGGCAATCTCCTAA
TCTCCATCCTGCTGGTCAAAGACAAGAGCCTGCACCGCGCTCCCTACTAT
TTCCTGTTGGACCTGTGCGCTTCGGACATCCTGCGCTCAGCCATTTGCTT
CCCCTTTGTCTTCACCTCAGTCAAGAATGGCTCGACGTGGACCTACGGCA
CGCTTACCTGCAAAGTGATTGCCTTCCTGGGCGTCCTCTCCTGCTTCCAC
ACGGCCTTCATGCTATTCTGTGTCAGTGTCACACGCTACCTGGCCATCGC
CCACCACCGCTTTTACACCAAGCGGCTGACTTTCTGGACGTGCCTCGCGG
TCATCTGCATGGTGTGGACCCTGTCTGTGGCAATGGCCTTTCCGCCAGTG

CTGGACGTGGGGACGTACTCGTTCATCCGCGAGGAGGACCAGTGCACGTT
CCAGCATCGCTCGTTCCGTGCCAACGACTCGCTGGGTTTCATGCTCCTGC
TGGCGCTCATCCTCCTGGCCACACAGCTGGTCTACCTCAAGCTCATCTTC
TTTGTCCACGACCGGCGAAAGATGAAGCCAGTCCAGTTTGTACCAGCTGT
CAGCCAGAACTGGACCTTTCACGGACCCGGCGCCAGCGGCCAGGCGGCAG
CCAACTGGCTGGCTGGATTTGGCCGCGGCCCGACGCCACCTACCTTGCTA
AACATCCGTCAAAACAGCAACGCGGCAGGCCGCAGGCGTCTGCTGGTGCT
GGATGAGTTCAAAACAGAGAAGCGGATTAGCAGAATGTTTTACATCATGA
CCTTTTTCTTCTTGGCGCTGTGGGGACCCTATCTGGTAGCGTGCTACTGG
CGGGTGTTTGCTCGTGGACCCGCCGTCCCTGGGGGCTACCTGACTGCTGC
CGTGTGGATGAGCTTTGCCCAGGCGGGCGTCAACCCCTTTATCTGCATCT
TCTCCCACAGGGAGGCCAAATCTCGATTTCACCCTGGCGTAGGGACCGGC
CCTGGCACAGACC---GCAGCGTCCCACTTAGCAACAGCTTGCTATCTCC
GCAACAAACCGAGGAGCCCACAGTTG---CTTCCCCACAGCGTTGGTTTG
TCACCC---CTGCCAATAACCGACTGGACTTTGCAGCCTCGGCATACGAT
GCCGCTGCTGCWGCTGATTTTGCCGGTAACGCAGCCACCTTGCTATCCTA
CGCAGCGGCTGGAGTAAAGGCGC------TACCGCTGCCCACAGCAGGTT
GTTCAAATAGGCCACTTGGTTATTATGCCGATCCGTCAG---GGTGG---
GGCGCCCGGACACCTCCTCAATACTGT---------AGTAAATCCAGTTC
AGTACTCTCTTGTTGGCCCAGTAATTCTGTTGGCGGGAGAACAGCTA---
------ACTACCTGG---------CGGATGA---TGTT---GACGC---C
CTTCCCACAGAGAGATCACCC---AT---TGGAGGTTCCGAGGAG---AC
AAAACCTAAAGACTT------GTCAGA---ATCAAGCTGGATAGAG---A
CGCCATCTTCAATAAAGTCAATTGATTCAAGTGATTCTGGAATCTTTG--
-AGCAAGCCAAACGAAGAAGAATTTCACCGTCTGCCACTCCA--------
-GTTTCAGAGACTGTGTCCCCGTTGAAATCTGAA----------------

------GCTTTCAAAATCAACCATAGTTCACATGATTTGGGCTCTGG---
GCAAACTGCATTTTCGTCCCAAACA---CCAGGCTAC---GCTGCCGCTG
CCCTTGGA---CACCATCA------CCACCCTACTCATGTCAGCTCT---
TATTCGACTGCAGCTTTCAATTCTACACGGGACTTTCTCTTCAGAAATCG
GGGTTTTGCAGATGCCACCAG------------TGCACAGCACAGCCTGT
TTGCCTCCGC---AGCGGGAAGTTT---T------GCAGGACCACATGGR
CACTCTGATGCCGCAGGACACCTGCTCTTCCCGGGACTTCATGAG---CA
AGCCGCAAGCCACGCATCCTCAAATGTTGTAAATAGTCAAATGCGATTGG
GTTTTTCGGGGGACATGTATGGACGTGCCGATCAATATGGCCACGTTTCT
AGCCCAAGGT---CCGATCATTACGCGTCAACCCAATTGCATGGTTATGG
TCCTATGAACATGAATATGGCCGCA---CATCATGGTGCGGGGGCCTTCT
TCCGATACATGAGGCAGCCCATCAAACAAGAGCTAATCTGCAAGTGGATT
GAACCGGAACAACTGGCAAACCCAAAAAAGTCATGCAACAAAACTTTCAG
CACAATGCATGAGCTCGTGACCCATCTAACAGTGGAACATGTCGGGGGGC
CGGAGCAGTCAAACCACATTTGCTTCTGGGAAGAGTGTCCC---------

>Galaxiella nigrostriata
AGCTTATTAATTCGAGCCGAGCTAAGCCAACCTGGGACCCTTTTAGGTGA
CGACCAGATTTATAACGTAATTGTAACCGCACACGCCTTTGTAATAATCT
TTTTTATGGTGATACCGATTATAATTGGTGGTTTTGGTAACTGATTGGTG
CCTCTGATAATTGGAGCTCCAGATATAGCATTTCCACGCATGAATAATAT
AAGCTTTTGACTTCTTCCACCCTCTTTTCTTCTCTTATTAGCGTCTTCCG
GAGTTGAAGCTGGGGCTGGCACAGGATGAACAGTTTACCCGCCCTTAGCG
GGGAACCTAGCTCATGCTGGAGCCTCTGTAGACCTGACAATTTTTTCCCT
TCACCTGGCAGGAATTTCTTCGATTTTAGGTGCAATTAACTTTATTACTA

CAATTATTAATATAAAACCCCCAGCAGTTTCACAATATCAAACCCCCTTA
TTTGTATGGTCCGTTTTAATTACAGCTGTACTCCTCCTTCTTTCTTTGCC
AGTTCTTGCTGCAGGAATCACGATACTATTAACCGACCGAAATTTAAACA
CAACTTTCTTCGACCCCGCGGGAGGAGGCGACCCTATCTTATACCAGCAC
TTGTTTTGATTTTTTGGACATCCCGAAGTCTATATTCTTATTTTACCCGG
CTTCGGAATAATTTCACACATTGTCGCTTATTACTCAGGCAAAAAAGAGC
CATCTGGTTACATAGGAATAGTTTGAGCTATGATGGCAATCGGCCTATTA
GGCTTCATTGTTTGAGCCCACCATATATTTACAGTCGGTATAGACGTAGA
TACTCGAGNNNN--

NNNNNNNNNNNNNNNNNNNNNNNNNNNNNNNNNNNNNNNGGCTAATCCTCAACGAGGCCGAGGTGATC
CTGGCCCTGGCCCAGGAGTTCCAGATGAGGGTGGTGACGGTGTCGCTGGA
GGAGCAGTCCTTCTCCGCCATCGTTCAGGTCCTCAGCAGGGCCTCCATGC
TGGTGAGCATGCACGGAGCCCAGCTCATCACCTCACTGTTCCTCCCCCGG
GGGGCCGTGGTGGTGGAGCTCTTCCCGTTCGCCGTCAACCCCGAGCAGTA
CACGCCTTACAAGACCCTGGCTTCCCTGCCGGGCATGGACCTCCACTACG
CCGCCTGGAGGAACCACCTGGAGGAGAACACGGTGACCCACCCGGACAGG
CCCTGGGACCAGGGGGGCATCGCTCACCTGGAGAAGGAGGAGCGGGAGCG
CATCGTGGCCAGCCGGGACGTTCCCCGGCACCTGTGCTGCCGGAACCCAG
AGTGGCTCTTCCGGATCTACCAGGACACCAGGGTGGATGTGCCCTCCCTC
CTGGAGCTCCTGCG---GGAGAACCTGAAG---ACCCGACCCAGCCTGAA
GAA---GGCCAGGCCCGCCAGCACCGTCCACCCCGGGCGGGTCCGCGAGG
CCCGGTGCCAGACGTCCGTCCAGGCCGCCAACGAGGCCAAGCTGACAGTT
TGGTGGCAGATCCCGTGGAACCTGAAGTACCTGAAGGTCAGGGAGGTGAA
GTNNNNNNNNNNNNNNNNNAAGAAAGATCCAAGCAAGGGGACCCTGGAGGATCAAATCATTCAGGCA
AATCCAGCACTGGAAGCTTTTGGTAATGCCAAAACTGTGAGAAACGACAA
CTCATCCCGCTTTGGTAAATTCATCCGAATTCACTTTGGAACTACTGGAA
AATTATCTTCTGCTGACATAGAGACTTACCTACTTGAAAAATCACGTGTT
ACTTTTCAGTTAAAGTCAGAGAGGAACTATCACATCTTCTTTCAGATCCT
GTCCAATCAAAAGCCAGAGCTGTTGGACATGCTTTTAATCACCAACAATC
CCTATGACTATGCCTATATCTCCCAGGGAGAGGTTACAGTACAATCAATT
AATGATGCAGAGGAGTTGATGGCCACAGACAGTGCCTTTGATGTGCTGGG
CTTTACTCAAGAAGAAAAAATGGGCGTCTACAAATTGACCGGTGCCATCA
TGCACTACGGAAACATGAGGTTCAAGCAAAAGCAGCGTGAAGAGCAGGCT
GAGCCGGATGGTACAGAAGCTGCTGACAAATCCTCTTACCTGATGGGTCT
AAATTCTGCAGACCTTATTAAAGGGCTTTGTCATCCCAGAGTCAAAGTTG
GCAATGAGTATGTTACCAAAGGTCAAGGTGTAGATCAAGTCTACTAT---

---------------------------GGCCACTACGAGTTCGAGTGGCA
GCCGGCGCTGAAGAACGTGTCGCCGCAGTGCCACGTGGGCATCATCGACG
GCCTCTCCGGCTGGGCGGCGTCAGTGGACGACTCGCCGGCGGACACCATC
ACGCGGCGCTTCCGCTACGACGTGGCGCTGGCGTCGGCCTTGAAGGACCT
GGAGGAGGACATCATGGAGGGCCTGCGGGAGAGCGGCTTGGAGGACGCCG
CC---------CACTTCAGCGTCACGGTGAAGGAGTCCTGCGACGGCATG
GGCGACGTGAGCGAGAAGCACGGCGGCGGGCCGGCCGTGCCCGAGAAGGC
CGTGCGCTTCTCCTTCACCGTCATGTCCGTCTCCGTCCAAGTCGGGGGCC
GGGCC------------------------------GAGGCCGCGACGGTG
TTCCGGGAGGCCAAACCCAACTCGGAGCTCTCCTGCAGGCCCCTGAGTCT
GATGTTTGTGGACGAGTCGGACCACGAGACACTCACAGCCATCCTGGGGC
CTGTGGTGGGCGAGCGGGACGCCATGAAGCAGAGCCGTCTCCTCCTGACC
CTGGGGGGCCTCCAGCGCTCCTTCCGCTTTGACTTCCGGGGCACCGGCTA
CGACGAGAAGATGGTGCGCGAGATGGAGGGCCTAGAGGCCTCGGGCTCCA
CATACATCTGCACCCTGTGTGACTCCACTCGCGCGGAGGCCTCGACCAAC
ATGGTGCTCCACTCCATCACCCGCAGCCACAATGAGAACCAGGAGCGCTA
CGAGCAATGGAGGACCAACCCGTTTTCCGAGTCGGTCGACGAGCTGCGCG
ACCGGGTGAAGGGAGTCTCCGCCAAACCCTTCCTGGAGACCCAGCCCACC
CTGGACGCGCTGCACTGCGACATTGGCAACGCCACCGAGTTCTACAAGAT
GTTCCAGGACGAGATCGGCGAGGTGTTCCGGAAGCC---CAAC---CCCG
GCCGGGAGGAGAGGCGCGGCTGGCGGGCGGCCTTGGACAAGCAGCTGAGG
AAGAAGCTGAAGCTGAAGCCCATCATGCGTATGAACGGGAACTACGCCCG
GCGCCTGATGACCCAGGAGGCCGTGGAGGTGGTGTGTGAGCTGGTGCCCT
CAGAGGAGAGGCGACAGGCCCTCAGGGAGCTCATGGGCCTCTACGTACAG
ATGAAGCCGGTGTGGCGAGCCACCTGCCCGGCCAAGGAGTGCCCCGACCA
GCTGTGCCGCTACAGCTTCAACTCGCAGCGCTTTGCTGAGCTCCTCTCCA
CCACCTTCAAGTACAGGTACGACGGAAAGATCACCAACTACCTCCACAAA

```
ACGCTGGCCCACGTGCCTGAGATCATAGAAAGAGACGGGTCCATCGGGGC
GTGGGCCAGCGAGGGGAACGAGTCCGGTAACAAG----------------
--------------------------------------------------
--------------------------------------------------
--------------------------------------------------
--------------------------------------------------
--------------------------------------------------
--------------------------------------------------
--------------------------------------------------
--------------------------------------------------
--------------------------------------------------
--------------------------------------------------
--------------------------------------------------
--------------------------------------------------
--------------------------------------------------
--------------------------------------------------
--------------------------------------------------
--------------------------------------------------
--------------------------------------------------
--------------------------------------------------
--------------------------------------------------
--------------------------------------------------
--------------------------------------------------
--------------------------------------------------
--------------------------------------------------
--------------------------------------------------
--------------------------------------------------
--------------------------------------------------
--------------------------------------------------
--------------------------------------------------
--------------------------------------------------
--------------------------------------------------
--------------------------------------------------
--------------------------------------------------
--------------------------------------------------
--------------------------------------------------
--------------------------------------------------
--------------------------------------------------
--------------------------------------------------
--------------------------------------------------
--------------------------------------------------
--------------------------------------------------
--------------------------------------------------
--------------------------------------------------
--------------------------------------------------
--------------------------------------------------
--------------------------------------------------
--------------------------------------------------
--------------------------------------------------
--------------------------------------------------
--------GGCGAAGTCACAGAGAGAGAAGTAGCATTGGGGATAAACCCG
TTTGCAGACGGAATGGGCGCTTTCAAAATCAACCACAGTTCACATGATTT
GGGCTCTGG---GCAAACTGCATTTTCGTCCCAAACA---CCAGGCTAC-
```

--GCTGCGGCTGCCTTGGGA---CACCATCA------CCACCCTACTCAT
GTCAGTTCA---TATTCGACCGCAGCTTTCAATTCCACCCGGGACTTTCT
CTTCAGAAATCGGGGTTTTGCAGATGCCACCAG------------TGCCC
AGCACAGTCTGTTTGCCTCCGC---AGCGGGAAGTTT---T------GCA
GGACCACATGGACACTCTGATGCCGCAGGACACCTGCTCTTCCCAGGACT
TCATGAG---CAAGCCGCGAGTCATGCGTCCTCAAATGTTGTGAACAGTC
AAATGCGATTGGGCTTTTCTGGGGACATGTATGGACGTGCCGATCAATAT
GGCCACGTTTCTAGCCCAAGGT---CCGACCATTATGCGTCGACCCAATT
GCATGGTTATGGTCCTATGAACATGAATATGGCCGCA---CATCACGGTG
CGGGGGCCTTCTTCCGATACATGAGGCAGCCCATCAAACAAGAGCTAATC
TGCAAGTGGATTGAACCGGAACAACTGGCAAACCCCAAAAAGTCTTGCAA
CAAAACTTTCAGCACAATGCATGAGCTCGTGACCCATCTAACAGTGGAAC
ATGTCGGGGGGCCTGAGCAGTCAAATCACATTTGCTTCTGGGAAGAATGT
CCCCGAGAAGGAAAACCATTCAAAGCAAAATACAAACTTGTAAATCATAT
TAGAGTACACACCGGAGAA-------------------------------

>Gambusia affinis
AGCCTACTGATCCGGGCCGAACTCAGTCAGCCAGGCACACTTCTTGGAGA
CGACCAGATCTACAATGTAATCGTTACAGCTCATGCTTTTGTAATAATCT
TTTTTATAGTTATACCCATCATAATTGGAGGGTTTGGTAACTGACTAGTC
CCCCTAATAATTGGTGCCCCAGACATAGCCTTTCCACGAATAAACAACAT
AAGCTTTTGATTACTTCCCCCCTCATTTCTCCTCCTCCTTGCATCTTCTG
GGGTTGAAGCAGGGGCAGGAACAGGCTGAACTGTCTATCCCCCTCTTGCA
GGTAACCTAGCACATGCCGGACCTTCTGTAGACCTAACCATCTTTTCCCT
TCACCTAGCGGGCATCTCCTCTATTCTGGGAGCTATCAATTTTATTACCA
CCATTATTAATATAAAACCTCCCGCAGCCTCCCAGTACCAAACACCATTG
TTTGTGTGGGCAGTCCTAATTACAGCTGTACTCCTCCTTCTTTCCCTTCC
AGTTCTTGCCGCAGGTATTACTATGCTTCTTACAGATCGAAACCTAAACA
CCACTTTCTTTGATCCAGCGGGGGGCGGAGACCCAATCCTCTATCAACAC
CTGTTC--

------------TTCCTGGAGAGAAACCTTCATCCGTCCAACTGTCTTGG
CATGCTGTTGCTGTCTGACGCCCACCAGTGTACAAAGCTGTCAGAGCTCT
CCTGGGGCATGTGTCTAAGCAACTTCCCTGCCATTTGCAAGACAGAGGAC
TTTCTCCAATTGCCCAAAGATATGGTAGTGCAGCTTTTGTCACACGAGGA
GCTGGAGACAGAAGATGAGAGACTGGTTTATGAAGCTGCCCTTAATTGGA
TTAACTATGACCTAGAAAAGAGGCACTGCTACCTCCCAGAGCTTCTGAGA
ACGGTTCGTCTCGCCTTACTGCCTGCCATCTTTCTGATGGAAAATGTTTC
AACAGAAGAGCTGATTAATGATCAGGCCAAAAGCAAAGAACTGGTTGATG
AAGCTATTCGCTGCAAGCTGAAGATCCTACAGAATGATGGCGTGGTCAAC
AGCCCATGTGCTCGACCTAGAAAAACCAGCCATGCCCTTTTCCTGCTTGG
CGGACAGACATTCATGTGTGACAAATTGTACCTGGTAGACCAGAAAGCCA
AAGAGATAATCCCAAAGGCTGACATCCCCAGTCCCAGGAAGGAGTTCAGT
GCCTGTGCCATTGGCTGTAAGGTGTACATCACAGGTGGGA--GAGGATC-
AGAGAACGGTGTTTCCAAAGACGTATGGGTCTACGACACTGTGCATGAGG
AATGGTCCAAAGCAGCACCTATGCTCATCGCCAGGTTCGGCCATGGCTCC
GCGGAGCTGAAACACTGCCTATATGTTGTTGGAGGACACACAGCGGCAAC
GGGCTGTCTCCCAGCCTCTCCGTCCAGATGAATACATCGCTTTGTTTAGT
CGCTCGACAACAAGATTAATTCTGAATGAAGCAGAACTAATAATGGCCTT
AGCCCAGGAGTTCCAGATGAGAGTGGTTACAGTATCCCTGGAGGAACAGT
CTTTTCCTAGTATCATCCAGGTGATTAGTGCTGCTTCCATGTTGGTCAGC

ATGCATGGAGCTCAGCTCATCACCTCAATGTTCCTCCCCAGAGGTGCAAC
TGTGGTAGAGTTGTTCCCCTTTGCTGTGAACCCAGAGCAGTATACTCCAT
ATAAAACCCTCGCCACTCTACCAGGCATGGACCTTCACTACATATTTTGG
AGGAATTCTAAGGAGGAAAACACTRTCACCCATCCAGACAGACCGTGGGA
GCAAGGCGGCATTGCCCACTTAGAAAAAGATGAGCAGCAGCGCATCCTGG
CAAGTACGGATGTCCCAAGGCACCTGTGCTGCCGGAACCCGGAATGGCTT
TTCCGGATCTACCAGGATACTTTGGTAAACATTCCGTCATTTCTCGAGGT
CCTCAA---AGATGGCATGAAG---AGTAAGCCTAGCCTGAAGAA---AA
CAAAAACTGCCAGCACAGTTCACCCTGGCCGGGTCAGAGAAGCTCACTGT
CAGACATCAGTACAAACCCCTAATGAGGCCAAACTCACAGTCTCTTGGCA
GATCCCATGGAATCTAAAGTACCTGAAGGTGAGAGAGGTTAAGTACGAGG
TATGGATCCAGAAAAAGGACAGCAGCAAGGGGACTCTGGAGGATCAAATC
ATCCAGGCAAACCCGGCACTCGAGGCCTTTGGCAATGCCAAAACGCTGAG
AAATGACAACTCGTCTCGTTTTGGAAAATTCATCAGGATTCACTTTGGCA
CAAGTGGGAAACTGTCATCGGCCGACATCGAGACGTATCTGCTGGAGAAG
TCCCGTGTTACATTCCAGCTTAAGGCTGAGAGGAACTACCACATCTTCTA
CCAGATCCTGTCCAACCAGAAGCCCGAGCTTCTGGATTTGCTGCTCATCA
CCAACAACCCGTACGACTACTGCTACATCTCCCAAGGAGAGGTAACGGTC
GCCTCCATTAACGACTCTGAGGAGCTTATGGCCACAGACAGCGCCTTCGA
TGTGCTAGGCTTCACTGCTGAGGAGAAAACAGCCGTCTACAAACTGATCG
GTGCCATCATGCACTACGGCAACATGAAGTACAAACAGAAGCAGCGTGAG
GAGCAGGCTGAACCGGACGGTACAGAGGCTGCAGATAAATCAGCTTACCT
AATGGGGCTTAACTCTGCAGATCTCATCAAGGGGGTGTGCCATCCCAGAG
TGAAGGTTGGAAATGAATATGTCACCAAGGGCCAAAGTGTGGATCAAGTC
TACTATCCCAACAAAGAGGCCTTCAAGTGTGAGGAGTGTGGGAAGCACTA
CAATACCAAGCTGGGATACAAGCGCCATGTGGCCATGCACTCGGCTACTG
CAGGGGATTTAACCTGCAAAGTGTGCATGCAGAATTACGAAAGCACACCT
GTTCTCCTGGAGCACCTGAAGAGCCACTCGGGGAAGTCTTCCGGTGGTGC
CAAGGAGAAAAAACACCCCTGCGACCACTGTGACCGACGTTTCTACACCA
GGAAAGATGTGAGACGACACATGGTGGTCCATACAGGGCGAAAAGACTTT
CTGTGCCAGTACTGTGCCCAGCGCTTTGGCAGGAAAGACCATCTGACACG
GCATGTGAAGAAGAGCCACTCACAGGAGCTGCTGAAGATTAAGACGGAGC
CTCCGGATATGCTAGGCCTGTTGACGACAGGATCTCCACCGTGCTCAGTA
AAGGAGGAACTCAGCCCTATGATGTGCGGCATGGGGCCCAACAAAGACCC
CATGATGGGAAAATCATTCCCTAGCGGGGCACCGTTTCCCATGAGCATGT
ATAACCCTCACCAT------CTCCAGGCCATGTCTAATTCTGGTGTGGGT
CCTCCA------CATCCTTCCCTAATGCCCAATTCCTTGTCTGCAGCTAT
GAGCATGGGCTGTCATATGGAATATCTTATCTATGCTTCTTTTTCATTCA
TGGGATGTTTACAAATTAGTGATGGATCGAACATAGTGAACCTGCTGGCT
AGTAACTCGCCAAGCGTTTCGTACGCCGTGACCCAGCAGAAGTACTTCAG
TAACTACAGTCCTGTGATTGGGTTTTACATCTACGAGCCCATCGAGTACT
GGAACTCCACTGTGCAGGAGCACCTGAAGACTCTCAGTCATGGCTTCAAC
AAGATCTCTTGGATGGACAACTTTTTCCACTACCTCCGGGTGGTGAACAT
CAGCGCGTCGACCAAGAGTGACTTCATCAATATTCTGAAGGGTTCTTTCC
TGCGCAGCCCGGAGTACCAGCACTTTACAGAGGACATCATCTTCTCGAAA
A---ACCGCAACACTG------ACGAGTACGACATCATCGCCTCGCGCAT
GTACTTGGTAGCGCGGACCACGGAGAAGAAGAGAGAGGAGGTGGTGGAGC
TCTTGGAGAAGCTTCGCCCCTTGATGCTGATCAACAGCATCAAGTTCATT
GCCTTCAATCCCACGTTTGTTTTCATGGACCGCTACAGTTCCTCCGTCAT
CTCGCCCATCCTGACCTCAGGCTTCAGCGTGCTCACCATCCTCATCCTCA
CGTTCTTCCTGGTCATCAACCCGTTGGGAAACTTCTGGCTCATTCTCACG
GTTACGTCCGTGGAGCTGGGCGTCTTGGGTTTGATGNNNNNNNNNNNNNNNNNNNNNNNNNNNNNNN
NNNNNNNNNNNNNNTCTTGTAGCGTTGG

CATCATAAATGGGCTGTCAGGATGGACATCTTCGTTAGATGACTCCCCAG
CTGACACCATCACCCGGCGCTTTCGCTATGATGTGGCGCTTGTTGGAGCA
CTGAAGGACCTTGAGGAGGACATCATGGAAGGTCTGAGAGACACTGGGAT
GGAAGATAGCGCTTGCACCTTGGGCTTTAGAGTCATGATCAAGGAGTGCT
GTGATGGCATGGGTGATGTTAGCGAAAAGCATGGTGGAGGACCAGCTGTT
CCTGAGAAGGCTGTTCGCTTCTCTTTCACTGTTATGTCTGTCTCTATCCA
GGCTGAAGATGAACAG------------------------------GAGG
AGGTCACAATTTTCACAGAGCCAAAGCCCAACTCTGAACTATCCTGTAAG
CCCCTTTGCCTTATGTTTGTGGATGAGTCTGACCACGAGACTCTCACTGC
TGTTTTGGGGCCTATAGTTGCAGAGCGAAATGCGATGAAAGAGAGCAGGC
TCATTCTATCTATGGGCGGCATGCCACGCTCCTTTCGCTTCCACTTCAGG
GGCACAGGGTATGATGAAAAGATGGTGCGTGAAATGGAGGGCCTGGAAGC
TTCGGGATCTACATATATCTGTACTTTGTGTGACTCCAGTCGAGCAGAGG
CTGCTCAAAACATGGTGTTACACTCCATCACCCGCAGCCATGATGAGAAT
CTGGAACGTTATGAAATATGGAGAACCAATCCCTTTTCTGAGTCTGTAGA
TGAGCTACGAGACAGAGTCAAAGGAGTCTCAGCCAAGCCCTTTCTGGAGA
CCCAGCCCACGCTAGATGCATTGCACTGCGACATAGGCAATGCCACTGAA
TTCTACAAAATCTTCCAGGATGAAATTGGAGAAGTGTTTAAGAACCC---
CAAC---CCCAGCAGAGAGCAACGGCGCAGCTGGAGGGCAGCCTTGGATA
AACAGCTGAGGAAGAAGATGAAGCTTAAACCAGTGATGAGGATGAATGGG
AACTATGCCCGAAGGTTAATGACCATGGAGGCTACCGAGGTGGTTTGTGA
ACTGGTGCCCTCAGAGGAGAGGAGAGAGGTCCTGAGGGAACTCATGAGGC
TCTACCTCCAAATGAAACCTGTGTGGCGTGCCACCTGTCCAGCCAAGGAG
TGCCCTGACCAGCTGTGCCGCTACAGTTTTAACTCCCAGCGCTTTGCCGA
CCTGCTCTCCGCTACCTTCAAGTACAGGTATAACGGAAAAATAACCAATT
ACCTGCACAAGACCCTGGCCCATGTGCCTGAAATCATAGAGAGAGATGGA
TCTTTTGGAGCCTGGGCCAGCGAGNNNNNNNNNNNNNNNNNNNNTCATATACCATTGAGATGGGTTCAC
T
GGGACCTCAATGGAAGGCAAATCCAAGGCCATTCATCTGCTCTATTGAAG
ACCCAACCAAACAGACAAAGTTCAAGGGTATCAAGACCTACATATCGTAC
CGAGTGACGCCGAGCCACATAGGCCGGCCAGTTTACAGACGATACAAACA
CTTTGACTGGTTGTACAACCGTTTGTTGCACAAGTTCACTGTGATCTCTG
TGCCTCACCTCCCTGAGAAGCAGGCCACTGGTCGGTTCGAGGAAGACTTC
ATCGAGAAGCGTAAGAGGCGACTGATACTGTGGATGAACCACATGACCAG
CCACCCAGTCCTCTCGCAGTATGAAGGCTTTGAGCACTTTCTGATGTGTG
GTGATGATAAGCAGTGGAAACTGGGTAAGAGGCGGGCAGAGAAGGACGAG
ATGGTGGGAGCCCATTTCATGCTGACCCTTCAGATCCCCAACGAACACCA
GGACCTTCAGGACGTCGAGGAGAGGGTTGACAACTTCAAGACTTTCGCCA
AGAAAATGGACGACAGTGTGATGCAGCTCACGCACGTTGCCTCAGAGCTC
GTCCGTAAGCACCTGGGCGGGTTCAGGAAGGAGTTCCAGCGACTTGGGAA
TGCCTTCCAGTCTATTAGCCAGGCTTTCACACTGGACCCACCCTACAAGT
CAGATGCCCTCAACAACGCCATCTCCCACNNNNNNNNNNNTTTCCTCAAATTAACCTCCCT
GGGTTTCATCATTGGAGTCGGTGTGGTAGGGAACCTCCTGATCTCAATCC
TGCTGGTTAAAGACAAGAGCCTGCACCGGGCACCCTACTATTTCCTACTG
GACCTGTGCGCCTCGGATATCCTACGCTCCGCTATCTGCTTCCCCTTTGT
CTTCACCTCTGTAAAGAATGGATCTGTCTTAACCTACAGCATACTGACCT
GCAAAGTGATCGCCTTCCTCGGTGTGCTCTCCTGTTTCCATACAGCATTC
ATGCTCTTCTGCGTCAGCGTCACCCGCTACCTGGCCATCGCGCATCACCG
CTTCTACACCAAGAGGCTGACGTTCTGGACGTGCTTGGCCGTCATTTGCA
TGGTGTGGACGTTGTCGGTGGCCATGGCGTTCCCTCCGGTGCTAGACGTC
GGGACGTACACGTTCATCCCGGAGGAGGACCAGTGTACGTTTCAGCACCG
CTCCTTCCGGGCGAACGACTCGCTGGGCTTCATGCTCCTGCTGGCGCTCA
TCCTTCTGGCCACGCAGCTGGTTTACCTCAAGCTCATCTTCTTCGTCCAC

GACCGCCGAAAGATGAAGCCCGTCCAGTTCGTGCCTGCCGTTAGCCAGAA
CTGGACCTTCCATGGGCCGGGCGCCAGCGGGCAGGCGGCGGCCAACTGGC
TGGCCGGGTTTGGCCGCGGCCCCACCCCGCCTACTTTGCTGGGGATCCGA
CAGAACAGCAACGCAGCGGGCCGCAGACGTCTTCTGGTGCTCGATGAGTT
CAAAACGGAGAAGAGGATTAGTAGGATGTTCTACATAATGACATTTTTCT
TCTTGGCTCTGTGGGGGCCCTACCTGGTTGCCTGCTACTGGCGCGTGTTT
GCCAGGGGCTCTGTGGTCCCTAGAGGGTACCTGACAGCAGCCGTGTGGAT
GAGCTTTGCCCAGGCCGGGGTCAATCCTTTCATCTGCATCTTCTCCAACA
GGGAGGCCAAATCTCGCTTTCACCCTGGCGTGGGGACTGGTCCTGGCACG
GAGC---GCAGCGTCCCACTCGGCAACAGCTTGCTGTCCCCGCAGCAAAG
CGAGGAGCCCACTGTTGCCACCCCCCCGCAGCGTTGGTTTGTCACCC---
CTGCCAACAACCGACTGGACTTTGCCGCCTCGGCATACGACGCCGCC---
------GATTTCGCCGGTAACGCGGCCACCTTGCTGTCCTACGCAGCGGC
CGGAGTAAAGGCTC------TCCCCCTCCCGACAGCGGGCTGCTCCAATC
GGCCTCTTGGCTATTACGCAGACCCGTCTG---GCTGG---GGAGGACGC
ACGCCACCACAGTACTGCGGCGTAAACAGTAAATCCAGCTCGGTCTTTTC
CTGCTGGCCGACCAACTCTCTTGGAGGCAGGGCGAGCA---CC---AACT
ACCTGT---------CGGAGGA---GGGA---GACTC---TATCGCGACC
GAGAGGTCACCG---AT---CGGTGTTTCGGAAGAG---ACCAAACCCAA
AGACATCAC---ATCAGA---GTCCAGCTGGATAGAG---ACGCCGTCGT
CSATAAAGTCGATTGATTCCAGCGATTCTGGGATCTTTG---AACAGGCC
AAAAGAAGACGGATCTCCCCCTCTGCCACCCCG---------GTCTCAGA
GACAGTGTCCCCATTAAAATCCGAGCATCACTCAACAGGAGAGGTGGCGG
AGCGGGAAGTGGCGCTGGGGATCAACCCTTTCGCGGACGGCATGGGCGCC
TTCAAGATCAACCACAGCTCCCACGACATCAGCTCCGG---GCAGACAGC
GTTCTCATCCCAGGCT---CCCGGCTATGCAGCGGCGGCCGCCCTGGGA-
--CACCACCA------TCACCCGACCCACGTCGGTTCC---TACTCCACG
GCGGCGTTCAACTCCACCAGAGACTTTCTCTTCAGAAATCGGGGTTTCGG
GGACGCGGCTGG------------GGCGCAGCACAGCCTGTTTGCCTC--
-------CGGCAGCTT---C------GGGGGGCCGCACGGACATTCGGAC
GCGGCGGGGCACCTACTCTTCCCAGGGCTCCACGAG---CAGGCGGCGAG
CCACGCGTCCTCCAACGTGGTGAACAGCCAGATGCGACTGGGCTTCTCAG
GGGACGTGTACGGCCGGGCTGATCAGTATGGGCATGTCACGAGCCCGCGT
T---CCGATCACTACGCATCCACACAGCTACACGGCTACGGCCCCATGAA
CATGAATATGGCTGCG---CACCACGGAGCGGGGGCCTTTTTCCGCTACA
TGAGGCAGCCCATCAAGCAGGAGCTCATCTGCAAGTGGATCGAGCCGGAG
CAGTTGACAAACCCCAAAAAGTCGTGTAACAAAACTTTCAGCACCATGCA
CGAGCTGGTGACTCACCTGACAGTGGAGCACGTGGGGGGGCCTGAACAGA
CGAACCATATCTGCTTTTGGGAGGACTGCTCCAGAGAAGGGAAGCCCTTT
AAAGCCAAATACAAACTTGTAAATCACATCAGAGTGCACACCGGAGAAAA
GCCCTTTCCGTGTCCGTTTCCCGGCTGCGGCAAA
>Gasterosteus aculeatus
AGCCTTCTAATTCGAGCTGAATTAAGTCAACCCGGAGCTCTCCTTGGGGA
CGACCAAATTTATAACGTAATTGTTACAGCCCATGCTTTCGTAATAATCT
TCTTTATAGTTATACCAATCATGATCGGAGGCTTTGGCAACTGACTTATC
CCCCTAATGATCGGAGCCCCCGATATAGCATTTCCACGAATAAACAACAT
GAGCTTCTGATTGCTCCCACCCTCTTTCTTACTTCTCCTTGCCTCTTCAG
GGGTTGAAGCTGGTGCAGGGACAGGGTGAACAGTTTATCCACCCCTCTCT
GGGAACCTCGCCCATGCAGGTGCTTCAGTCGACCTAACAATCTTTTCACT
TCATCTTGCAGGTATTTCATCAATTCTGGGGGCAATCAACTTCATTACCA
CAATTATTAACATGAAACCTCCCGCTATTTCTCAGTACCAAACGCCCCTT
TTCGTCTGATCTGTTCTCATCACTGCAGTCCTTCTTCTCCTATCCCTGCC
CGTCCTTGCAGCCGGAATCACTATGCTTTTAACAGACCGAAACTTAAACA

CCACTTTCTTCGACCCAGCAGGGGGTGGAGACCCAATTCTTTACCAACAC
TTATTTTGATTCTTCGG---------------------------------

NNNNNNNNNNNNNNNNNNNNNNNNNNNNNNNNNNNNNNNNNNNNNNNNNNNNNNNNNNNNNNNNNGTGCA
CCAAGCTGTCCGAGCTCTCCTGGGGCATGTGCC
TCAGCAATTTTCCCGCCATTTGCAAGACAGAAGACTTCCTCCAGCTGCCC
ACAGATATGGCGGTGCAGCTGTTGTCACACGAGGAGCTGGAGACGGAAGA
CGAGAGACTGGTTTATGAAGCTGCCCTTAACTGGATCAACTACGACCTGG
AGAAGAGGCACTGCCACCTTCCAGAGCTCCTGAGGACTGTCCGTCTGGCC
TTGCTGCCCGCCATCTTCCTCATGGAGAACGTCTCGACAGAAGAGCTGAT
CAATTCCCAGCCCAAGAGCAAGGAGCTGGTGGACGAGGCCATCCGATGCA
AGCTGAAGATCCTGCAGAACGACGGCGTCGTCAACAGCCCGTGTGCCCGG
CCCAGAAAGACCAGCCACGCCCTGTTTCTTCTGGGAGGTCAGACGTTCAT
GTGTGACAAGTTGTACCTCGTGGACCAGAAAGCCAAGGAGATCATCCCCA
AGGCTGACATCCCCAGCCCCAGGAAGGAGTTCAGCGCCTGCGCCATCGGC
TGCAAGGTGTACATCACTGGTGGGA--GAGGCTC-AGAGAACGGCGTGTC
CAAAGACGTGTGGGTTTACGACACCGTCCACGAGGAGTGGTCGAAGGCGG
CGCCGATGCTCATCGCCCGCTTCGGCCACGGCTCGGCAGAGCTGAAACAC
TGCCTCTACGTGGTCGGCGGTCACACGGCGNNNNNNNNNNNNNNNNNNNNNNNNNNNNNNGGATGATTAC
GTCGTCGTGT
TCAGTCGCTCGGCAACGAGGCTGATACTGAACGAAGCTGAGCTAATCCTG
GCGCTGGCCCAGGAGTTCCAGATGAGAGTGGTCACGGTGTCCCTGGAGGA
TCAGTCCTTCCCCGGTATCGTCCAGGTGATCAGCGGCGCCTCCGTGTTGG
TCAGTATGCACGGCGCTCAGCTCATCGCGTCGCTCTTCCTCCCCAGAGGG
GCGACCGTGGTGGAGCTGTTCCCCTTTGCTGTGAACCCAGAACAGTACAC
CCCGTATAAAACCCTGGCCACCCTTCCGGGAATGGACCTTCACTATATCT
CCTGGAGGAACACTAAGGAGGAGAACACCATCACCCACCCAGACAGACCC
TGGGAGCAGGGGGGCATCGCTCACTTGGAGAAGGAGGAGCAAGAGCGCAT
ACTGGCGAGCCGAGACGTCCCCAGGCACCTGTGCTGCCGCAACCCAGAGT
GGCTCTTCCGGATCTACCAGGACACGTTGGTGGACATCCCCTACTTCCTG
GAGACCCTTAG---AGAGGGCATGAAG---ACAAAGCCCAGCGGGAAGAA
---GTCAAAGCCGGCCAGCACAGTCCACCCGGGCCGGGTGAGGGAGCCGC
AGTGTCAGACCTCGGTACAAACCACCAACGAGGCTAAACTCACAGTTTCC
TGGCAGATCCCATGGAATCTGAAATACCTAAAGGTGAGAGAGGTGAAGTA
CGAGGTGTGGATCCAGAAACGGGACACTAGTAAGGGGACGCTGGAGGACC
AGATTATCCAGGCCAACCCGGCACTGGAGGCCTTTGGAAATGCCAAAACG
CTGAGGAATGACAACTCATCTCGTTTTGGGAAATTCATCCGAATTCACTT
TGGTACAAGTGGGAAGCTGTCATCTGCCGATATCGAGACTTACCTGCTGG
AAAAGTCCCGGTGCACCTTTCAGCTCAAGGCTGAGAGGAACTACCACATC
TTCTACCAGATCCTCTCCAATCAGAAGCCAGAGCTGCTGGACATGCTGCT
GATCACCAACAACCCATACGACTACTCCTACATCTCCCAGGGAGAGGTGA
CGGTGGCCTCCATCAATGACTCGGAGGAGCTGCTGGCCACCGACAGCGCC
TTTGACGTGCTGGGCTTCACTCCGGAGGAGAAGATGGGTGTCTATAAACT
CACCGGGGCCATTATGCACTACGGCAACATGAGGTTCAAACAGAAGCAGC
GCGAGGAGCAGGCTGAGCCGGACGGGACCGAGGCCGCTGATAAAACAGCC
TACTTGATGGGGCTGAACTCTGCCGACCTCATCAAGGGGTTGTGTCATCC
CAGGGTGAAGGTCGGGAATGAATACGTAACCAAAGGTCAAAGTGTGGACC
AAGTCTACTACCCAAACAAGGAAGCCTTCAAGTGTGAGGAGTGCGGGAAG
CACTACAACACCAAGCTGGGATATAAGCGCCATGTGGCCATGCACTCTGC
CACGTCAGGGGATCTCACCTGCAAAGTGTGCATGCAGAGCTACGAGAGCA

CACCCGTGCTCCTGGAGCACCTCAAGACCCACTCCGGAAAGTCTTCGGGT
GGCGCCAAGGAGAAGAAGCACCCGTGCGACCACTGTGACCGCCGTTTCTA
CACGCGCAAGGACGTGAGACGACACATGGTGGTCCACACGGGCCGAAAAG
ATTTCCTCTGCCAGTACTGTGCCCAGCGCTTTGGCAGRAAGGACCATCTG
ACGCGTCACGTTAAGAAGAGCCACTCACAGGAGCTGCTGAGGATCAAGAC
GGAGCCTTCGGATATGTTAGGTCTTTTAGCGTCCGGGTCACCGCCCTGCT
CTGTGAAGGAGGAGCTCAGCCCCATGATGTGCGGTATGGGTCCCAACAAA
GACCCAATGATGGGCAAACCGTTCCCTAGCGGGGCCCCTTTTCCGATGAG
CATGTACAACCCCCACCAT------CTCCAGGCCATGTCTAATTCTGGGG
TGGGTCACCCG------CACCCATCCCTGATGCCCGGTCCCTTGTCTGCA
GCTATGGGTATGGGCTGCCACATGGAATATCTGATATACGCCTCTTTTTC
GTTCATGGGATGTTTACAGATCAGCGATGGATCAAACGTAGTGAACCTCT
TGGCAAGTAACTCTCCGAGTGTTTCGTACGCTATGACGCAGCAGAAATAC
TTCAGCAACTACAGTCCCGTGATTGGGTTCTACATTTATGAGCCCGTCGA
GTACTGGAACTCCACGGTGCAGGAACACCTGAAGACCCTGAGTCATGGTT
TCAACAAGATCTCTTGGATGGACAACTTTTTCCACTACCTGCGTGTGGTA
AATGTGAGTGCGTCGACCAAGAGTGACTTCATCTCCATCCTCAAGGGCTC
CTTCCTTCGCAGCCCGGAGTACCAGCACTTCAATGAGGACATCATATTTT
CCATGA---ACCGCGAAACCG------AAGAGTACGACATTATCGCCTCG
CGGATCTACCTGGTGGCGCGGACAGCAGAGAAGAAGCGAGAGGAGGTGGT
GGAGCTTCTGGAAAAGCTTCGACCGTTGATGCTGATCAACAGCATCAAGT
TCATTGCATTCAATCCTACGTTTGTGTTCATGGACCGCTACAGCTCCTCT
GTTATCTCACCCATCCTGACCTCAGGCTTCAGCGTACTCACAATCCTCAT
CCTCACTTTCTTCCTGGTCATCAATCCGTTGGGAAACTTCTGGCTCATCC
TCACGGTAACGTCCGTGGAGCTCGGCGTCTTGGGTTTGATGNNNNNNNNNNNNNNNGAATGGCAA
CCAGCWCTCAAGAACGTGTCTACGTCCTGCACCGTTGGCATCATTAATGG
GCTCTCTGGATGGGCCTCCTCGGTGGACGACTTCCCTGCTGACACCACCA
CTCGACGGTTTCGGTACGACGTGGCTCTGGTGTCAGCTTTAAAGGATCTG
GAGGAGGACATCCTGGAGGGGTTGAGAGAGAGCGGCATGGAAGACAGCGC
TTGCACCTCAGGCTTCAGCGTCATGATCAAGGAGTCCTGTGACGGCATGG
GGGACGTCAGCGAGAAGCACGGCGGAGGACCACCAGTTCCTGAGAAGGCT
GTGCGTTTTTCTTTTACTGTCATGTCTGTCTCTGTGCTGGCAGACGAGGA
GGTG------------------------------GAAGAGGTTACCATCT
TCACTGAGCCAAAGCCAAACTCAGAACTGTCCTGTAAGCCTCTTTGCTTG
ATGTTCGTGGATGAGTCGAACCACGAGACGCTCACAGCTGTCCTGGGGCC
AATAGTTGCCGAGCGCAATGCGATGAAAGAAAGCAGGCTCATCCTCTCCG
TGGGCGGACTCCCTCGCTCCTTCCGCTTTCACTTCAGGGGCACGGGATAT
GATGAGAAGATGGTGCGCGAGGTAGAGGGCCTGGAGGCGTCAGGGTCCAC
CTACGTCTGCACTCTTTGTGACTCCACTCGCGCGGAGGCCTCTAAAAACA
TGGTGCTGCACTCCATCACCCGCAGTCACGAAGAGAACCTGGATCGTTAC
GAGATATGGAGAACCAACCCGTTTTCTGAATCTGTGGAGGAGCTGCGGGA
CAGAGTCAAAGGGATCTCTGCCAAACCCTTCATGGAGACCCAGCCCACGA
TGGATKCACTGCACTGTGACATCGGCAACGCCACAGAGTTCTACAAGATC
TTCCAGGACGAGATCGGGGAGGTGTACCGAAAGGT---CAAC---CCCAG
TCGGGAAGAACGGCGAAGCTGGAGGGCGGCGCTGGACAAACAGCTCAGGA
CGAAGATGAAGCTCAAACCGGTGATGAGGATGAATGGGAACTACGCCCGC
CGGCTAATGACCCAAGAGGTCGTGGAGGTGATTTGTGAGCTGGTGCCCTC
CGAGGAGAGGAGGGAGGCCCTGAGGGAGCTGATGAGGCTCTACATCCAAA
TGAAGCCTGTGTGGCGCGCCACCTGCCCGGCCAAGGAGTGCCCCGACCAG
CTCTGCAGCTACAGCTTTAACTCCCAGCGCTTCGCCGACCTTCTCTCCTC
CACCTTCAAGTACAGGTACAACGGAAAGATAACCAACTACTTGCACAAGA
CTTTGGCCCATGTGCCTGAAATCATAGAGAGANNNNNNNNNNNNNNNNNNNNNNNNNNNNNNNNNNNNNN
NNNNNNNNNNNNNTCGTACACCATCGAGATG

GGCCCCCTGGGGCCCCTGTGGAAGGAGAGCCCRCAGCCGTTCTCCTGCTC
CRTYGAGRACCCCACGAAACAGACCAAGTTCAARGGCATCAAGACGTACA
TCTCGTACCGGGTCACGCCGAGYCACACGRGGMATCCCGTCTACAGGCGC
TACAAACATTTCGACTGGCTCTACAACCGCCTGCTGCACAAGTTCACTGT
GATCTCCGTGCCTCACCTGCCCGAGAAGCAGGCCACGGGGCGATTTGAGG
AAGACTTCATCGAAAAGCGCAAGAGGCGACTGGTACTGTGGATGAAYCAC
ATGACCAGCCACCCGGTCCTCTCCCAGTATGAGGGCYTYGARCACTTTCT
GATGTGCGGCGAYGACAAGCAGTGGAAGCTCGGCAAGAGGCGGGCGGAGA
AGGACGAGATGGTGGAGCCCATTTCATGTTGACCCTGCAGATCCCCACC
GAGCACCAGGACCTYCAGGATGTGGAGGAGCGCATCGACACCTTCAAGTT
CTTCGCCAAGAAGATGGACGACAGCGTGATGCAGCTCACGCACGTCGCCT
CGGAGCTGGTGCGCAAGCACCTGGGCGGGTTCAGGAAGGAGTTCCAGCGG
CTGGGAAATTCTTTCCAGTCCGTGAGCCAGGCGTTCATGCTTGATCCTCC
CCAAAGTTCAGAGGCCCTCAACAAAGCCATCTCCCATCCTCTCGCCACGT
TCCTCAAACTGACCTCTCTGGGTTTCATCATTGGAGTCGGTGTGGTCGGA
AACCTCCTGATCTCCATCCTGCTCGTCAAAGACAAGAGTCTGCACCGAGC
GCCCTACTATTTCCTGCTGGACCTGTGCGCCTCTGACATCCTGCGCTCTG
CCATTTGCTTCCCCTTTGTCTTCACCTCGGTCAAGAATGGATCCGCCTGG
ACGTATGGCACGCTGACCTGCAAAGTGATCGCCTTCCTGGGTGTGCTCTC
CTGTTTCCACACGGCGTTTATGCTCTTCTGCGTCAGCGTCACGCGCTACC
TGGCCATCGCGCACCACCGCTTCTACACCAAGAGGCTGACCTTCTGGACC
TGCCTAGCCGTCATCTGCATGGTGTGGACGTTGTCGGTGGCCATGGCCTT
CCCGCCGGTGCTCGACGTGGGGACGTACTCTTTCATCCGGGAGGAGGACC
AGTGCACGTTCCAGCACCGCTCCTTCAGGGCGAATGATTCGCTGGGCTTC
ATGCTCCTGCTGGCGCTCATCCTCCTGGCCACGCAGCTGGTTTACCTCAA
GCTCATCTTCTTCGTCCACGACCGTCGGAAGATGAAGCCCGTCCAGTTTG
TGCCTGCCGTCARCCAGAACTGGACCTTCCACGGGCCTGGTGCCAGCGGG
CAGGCGGCGGCCAACTGGCTGGCCGGATTCGGTCGAGGCCCCACCCCGCC
TACCTTGCTGGGCATCCGGCAGAACAGCAACGCAGCGGGCCGCAGGCGTC
TGCTGGTATTGGATGAGTTCAAAACAGAGAAGAGGATTAGCAGGATGTTC
TACATCATGACGTTTTTCTTTCTGGCGCTATGGGGGCCCTACCTGGTCGC
CTGCTACTGGCGGGTGTTTGCAAGGGGCCCCGTGGTCCCCGGAGGCTACC
TGACGGCCGCCGTGTGGATGAGCTTTGCCCAGGCCGGGGTCAATCCTTTC
ATCTGCNNNNNNNNNNNNNNNNNNNGCCAAATCTCGCTTTCACCCTGGCGTGGGGACCGGCCCTGGCAC
GGAGC---GCAGCGTCCCACTCGGCAACAGCTTGCTCTCCCCGCAGCAAA
CCGACGAGCCCACTGTTGCCACCCCCCCGCAGCGATGGTTTGTCACCC--
-CTGCCAACAACCGACTGGACTTTGCTGCCTCGGCATACGACGCCGCC--
-------GATTTCGCCGGCAACGCGGCCACCTTGCTGTCCTACGCAGCGG
CCGGAGTGAAGGCTC------TTCCCCTGCCGACTGCAGGCTGCTCCAAC
CGGGCCCTTGGCTATTACGCGGACCCGTCGG---GCTGG---GGAGGACG
CACGCCGCCGCAGTACTGCGGYGTCAACAGCAAATCCAGCTCGGTCTTTT
CCTGCTGGCCCGCAAACTCTATCGGTGGCAGAGCAGGCA---CC---AAC
TACCTGG---------CCGAGGA---GGGG---GACTC---CATCACGAC
GGAGAGGTCGCCG---AT---CGTCGGCTCGGAMGAG---AGCAAAGCCA
AAGACATGAC---GTCCGA---GTCGAGCTGGATAGAG---ACGYCGTCC
TCCATTAAGTCAATTGATTCAAGCGATTCTGGTATCTTTG---AACAGGC
TAAGAGGAGAAGGATCTCACCTTCTGCCACGCCG---------GTTTCTG
AGACAGTGTCCCCGTTAAAGTCTGAGCATCACTCAACAGGCGAAGTCACA
GAGAGAGAAGTAGCGTTGGGGATCAATCCGTTCGCGGATGGGATGGGCGC
CTTCAAAATAAACCACAGCTCCCACGACATCGGCTCCGG---ACAAACGG
CGTTTTCCTCTCAGGCG---CCCGGCTAC---GCAGCGGCCGCCCTGGGA
---CACCATCA------CCACCCGACCCACGTTGGCTCT---TACTCCAC
GGCGGCTTTCAACTCCACCAGGGACTTTCTCTTCAGAAATCGGGGTTTCG

GGGACGCCACCGG------------GGCGCAGCACAGTTTGTTCGCCTC-
--------CGGGAGTTT---C------GCAGGGCCACATGGACACTCGGA
TGCAGCGGGGCACCTGCTCTTCCCGGGACTCCACGAG---CAAGCGGCGA
GCCATGCGTCTTCCAACGTGGTCAACAGCCAGATGCGGCTGGGCTTCTCG
GGGGACATGTACGGGCGGGCCGACCAGTACGGCCACGTTACAAGCCCGCG
GT---CCGACCACTACGCATCGACCCAGCTGCACGGCTACGGCCCCATGA
ACATGAATATGGCCGCG---CACCACGGAGCGGGGGCCTTCTTTCGATAC
ATGAGGCAGCCGATCAAGCAAGAGCTCATCTGCAAGTGGATCGAGCCGGA
GCAGCTGACGAATCCCAAAAAGTCGTGCAACAAAACTTTAGCACGATGC
ACGAGCTCGTGACCCATCTGACGGTGGAGCATGTGGGGGGACCAGAGCAG
ACCAACCACATCTGCTTCTGGGAGGAATGCGCCCGAGAAGGAAAGCCGTT
CAAAGCCAAATACAAACTTGTGAACCATATCAGAGTACACACCGGAGAAA
AGCCCTTCCCGTGTCCGTTCCCCGGCTGTGGCAAA
>Gazza minuta
AGCCTGCTTATCCGGGCAGAATTAAGCCAACCCGGCGCCCTCCTCGGAGA
CGACCACATCTACAATGTTATCGTCACCGCCCATGCATTCGTAATAATTT
TTTTTATAGTCATACCTATTATGATTGGAGGCTTTGGCAACTGACTAATC
CCCCTAATGATCGGGGCCCCTGATATGGCATTCCCCCGAATAAATAACAT
AAGCTTTTGGCTTCTGCCTCCCTCGTTTCTGCTGCTGTTAGCATCTTCCG
GCATTGAAGCTGGTGCTGGCACAGGGTGGACAGTCTACCCCCCTTTGGCG
GGGAATCTTGCCCATGCAGGAGCTTCTGTTGACCTGACAATCTTCTCACT
ACACTTGGCTGGGATTTCCTCAATTTTAGGGGCAATCAACTTTATTACAA
CAATCATTAATATAAAACCCCCTGCAATTTCACAATTCCAAACTCCTTTG
TTTGTTTGGGCTGTGCTTATCACAGCCGTGCTTCTTCTACTTTCCTTACC
AGTTCTCGCAGCCGGCATCACTATGCTCCTAACAGATCGAAATCTAAACA
CTACTTTCTTCGACCCTGCAGGAGGGGGAGACCCAATTCTGTACC-----

------------TTCCTTGAGAGAAACCTTCACCCATCCAACTGCCTTGG
CATGCTGCTGCTGTCCGATGCCCACCAGTGCACCAAGCTATCTGAGCTCT
CCTGGGGCATGTGCCTCAGCAACTTTCCTGCTATTTGTAAGACGGAGGAC
TTCCTCCAACTGCCCAAAGATATGGTGGTGCAACTTTTGTCACATGAGGA
GTTAGAGACAGAAGATGAGAGACTCGTTTACGAAGCTGCTCTCAACTGGA
TCAACTATGACCTGGAAAAGAGGCACTGTCACCTTCCAGAGCTTCTGAGA
ACGGTGCGTCTGGCCCTGCTGCCTGCCATCTTTCTCATGGAGAATGTGTC
CACAGAAGAGCTCATCAACACCCAGGCCAAGAGCAAGGACCTGGTAGATG
AAGCCATCCGTTGCAAGCTGAAGATCCTGCAGAATGACGGTGTGGTCAAC
AGCCCGTGTGCCAGACCGAGAAAAACCAGTCATGCCCTCTTTCTTCTGGG
AGGTCAGACTTTTATGTGCGACAAGTTGTACCTGGTGGACCAGAAAGCCA
AAGAGATCATACCCAAGGCTGACATTCCCAGCCCTCGGAAGGAGTTCAGC
GCTTGTGCCATTGGTTGTAAGGTATACATCACTGGTGGGA--GAGGGTC-
TGAGAATGGCGTGTCCAAAGATGTGTGGGTCTATGACACTGTCCATGAAG
AATGGTCAAAGGCAGCACCCATGCTCATTGCTCGGTTTGGCCATGGGTCC
GCAGAGCTGAAACACTGCCTGTACGTTGTGGGAGGTCACACAGCCGCAAC
CGGTTGCCTTCCTGCCTCTCCATCCGGAGGAGTACATTGTTGTGTTCAGC
CGCTCGACAACAAGGCTGATATTGAATGAAGCAGAGCTAATCATGGCACT
GGCCCAAGAATTCCAGATGCGAGTCGTCACAGTATCCCTGGAGGACCAGT
CGTTCCCCAGTATCGTCCAGGTGATCAGCAGCGCTACTATGCTGGTCAGT
ATGCATGGAGCTCAACTTATCACATCGCTTTTCCTTCCCAGAGGAGCTGC
TGTGGTGGAGCTGTTCCCCTTTGCTGTGAACCCAGAGCAGTACACCCCAT
ACAAGACCCTTGCCTCCCTACCAGGTATGGACCTTCATTATGTCTCCTGG

AGGAATACCAAGGAGGAGAACACCATCACCCATCCAGACAGGCCCTGGGA
GCAAGGGGGCATCGTGCACCTGGAGAAGGAGGAGCAGGAAAGAATCATGA
GGAGCAAAGATGTCCCCAGGCATCTGTGCTGCCGCAACCCAGAGTGGCTC
TTTCGGATCTATCAGGACACTCTGGTGGACATCCCGTCCTTCCTGGAGGT
CCTCAA---GGAGGGCATGAAA---ACAAAGCCCATCTTGAAGAA---AT
CAAAGCCAGTCACCACAGTCCACCCGGGCCGGGTCAGAGAAGCCCAGTGT
CAGACCTCTGTACAAGCTGCTAACGAGGCCAAACTCACAGTCTCCTGGCA
GATCCCGTGGAATCTGAAATACCTGAAGGTCAGGGAGGTGAAATATGAGG
TGTGGATCCAGAAAAAAGACAGCAGCAAAGGGACCCTGGAGGACCAGATC
ATCCAGGCCAATCCTGCTCTGGAGGCCTTCGGCAACGCCAAGACGCTGAG
GAATGACAACTCATCACGTTTTGGAAAATTCATCCGAATTCACTTTGGTA
CAAGCGGCAAACTTTCATCTGCCGACATTGAGACGTACCTGCTGGAGAAG
TCAAGAGTCACCTTTCAGCTCAAGGCTGAGAGGAACTACCACATCTTCTA
CCAGATCCTGTCCAATCAGAAGCCCGAGCTGCTGGACATGCTGCTGATCA
CCAACAACCCCTACGACTACTCCTACATCTCCCAAGGAGAAGTAACAGTC
GCCTCCATTAACGACTCGGAGGAGCTGATGGCCACTGACAGCGCCTTCGA
TGTGCTTGGCTTCACCGCAGAGGAGAAGATAGGTGTTTACAAGCTGACGG
GCGCCATCATGCACTATGGCAACATGAAGTTCAAGCAGAAACAGCGTGAG
GAGCAAGCTGAGCCAGATGGCACCGAAGCTGCTGATAAATCTGCATACCT
GATGGGGCTGAACTCCGCTGACCTCATCAAAGGGCTCTGCCACCCCCGAG
TCAAGGTAGGAAACGAATACGTCACCAAAGGTCAGAGTGTAGATCAGGTC
TACTACCCCAACAAGGAGGCCTTCAAGTGCGAGGAGTGTGGCAAGCACTA
CAACACCAAGCTGGGATACAAGCGTCATGTGGCCATGCACTCCGCCACGG
CAGGCGACCTCACCTGCAAGGTGTGCATGCAGACCTACGAGAGCACGCCC
GTGCTGCTGGAGCACCTCAAGAGCCACTCGGGGAAGTCCTCGGGCGGCAC
CAAGGAGAAGAAACACCCGTGCGACCACTGCGACCGCCGCTTCTACACGC
GGAAGGATGTCAGGCGGCACATGGTGGTCCACACGGGCCGGAAGGACTTC
CTGTGCCAGTACTGTGCCCAGCGCTTCGGCAGGAAGGACCACCTGACGCG
GCACGTGAAGAAGAGCCACTCGCAGGAGCTGCTGAAGATCAAGACGGAGC
CTCCCGACATGCTGGGCCTGCTCGCGTCCGGGTCGCCGCCGTGCTCCGTG
AAGGAGGAGCTCAGCCCCATGATGTGCGGCATGACTGCCAACAAAGACCC
CATGATGGGCAAGCCCTTCCCCGGGGGGGCCCCGTTTCAGATGGGCATGT
ACAACCCCCACCAC------CTCCAGGCCATGTCGAATTCCGGGGTGGGT
CACCCG------CACCCGTCCCTGATGCCCGGCCCGCTGTCTGCGGCCAT
GGGCATGGGCTGCCACATGGAANNNNNNNNNNNNNNNNNNNNNNNNNNNNNNNNTGTTTACAAATCAGT
GATGGATCGAACA
TTGTGAACCTGCTAGCTAGTAACTCTCCGAGTGTTTCGTATGCTCTGACC
CAGCAAAAATACTTTAGTAACTACAGCCCTGTGATTGGCTTTTATATTTA
TGAGCCCATAGAGTACTGGAACTCTACAGTGCAGGAGCACCTGAAGACTC
TGAGTCATGGCTTCAACAAGATCTCCTGGATGGACAACTTTTTCCATTAC
CTGCGGGTGGTGAACGTGAGTGCGTCAACCAAGAGCGACTTCATCACCAT
CCTCAAGGGCTCATTCCTGCGCAGCCCMGAGTACCAGCACTTCACTGAGG
ACATCATATTCTCCAAGA---ATCGCGAGACAG------ACGAGTATGGC
ATCATCGCCTCTCGGATGTACCTGGTGGCGAGGACGACAGAGAAGAAGCG
CGAAGAGGTGGTGGAGCTCCTGGAGAAGCTTCGCCCTTTGATGCTGATCA
ACAGCATCAAATTCATCGCCTTCAATCCCACGTTTGTGTTCATGGATCGC
TACAGCTCGTCCGTCATCTCGCCCATCCTGACCTCAGGATTCAGCGTGCT
CACAATCCTCATCCTCACTTTCTTTCTGGTCATCAACCCCCTGGGAAACT
TCTGGCTCATCCTCACTGTAACGTCCGTGGAGCTGGGTGTCTTGGGTCTG
ATGGGCTTTCACCAGTTTGAATGGCAGCCAGCACTCAAGAATGTATCTAC
ATCTTGCAATGTTGGCATTATTAATGGACTCTCTGGATGGACCTCCTCAG
TGGATGACGCCCCAGCTGACACCATAACACGTCGTTTCCGCTACGACGTG
GCTCTAGTGTCAGCACTAAAGGACCTGGAGGAGGACATCATGGATGGGCT

GAGGGAGCAAGAGATGGAGGACAGTGCTTGCACTTCAGGTTTCACTGTCA
TGATCAAGGAGTCTTGCGATGGCATGGGTGACGTCAGTGAGAAGCACGGA
GGAGGACCAGCTGTTCCGGAGAAGGCTGTGCGTTTCTCTTTCACTATCAT
GTCTATCTCAGTGAGGACAGATG---AGGAG-------------------
-----------AAAGAAGTCACTATCTTCACAGAGATGAAGCCAAACTCA
GAGCTGTCTTGTAAGCCCCTCAGCCTCACATTTGTGGATGAGTCAGACCA
TGAGACACTCACAGCCATCCTGTGGCCTATTGTTGCAGAGCGTAACGCGA
TGAAAGAGAGCAGGCTGATCCTGTCCATTGGCGGACTGCTTCGCTCCTTC
CGCTTTCATTTCAGAGGCACAGGATATGATGAGAAGATGGTACGTGAGAT
GGAGGGCCTCGAAGCCTCAGGGTCCACTTACATCTGCACACTTTGTGACT
CAAGTCGAGCGGGGGCCTCTGAAAACATGGTGCTGCATGCCATAACTCGG
TCCCATGAAGAAAACCTAGAGCGATACGAGATATGGAGAAGCAACCCCTT
TTCTGAGTCAGCAGATATGCTTCGAGACAGAGTCAAAGGCGTCTCTGCTA
AGCCCTTCATGGAGACACATCCCACGATGGACGCATTACACTGCGACATC
GGCAACGCCACTGAGTTCTACAAAATCTTCCAGGATGAGATCGGGGAGGT
GTTTGAAAAGCC---CAAT---CCCAGCCGGGATGAGCGCCGCAGCTGGA
GGGCAGCCCTGGATAAACAGCTGAGGAAGAAGCTGAAGCTCAAACCGGTG
ATGAGGATGAATGGGAACTATGCCCGCCGGCTAATGACCACAGAGGCTGT
GGAGGCGGTGTGTGAGCTGGTGCCCTCAGAGGCAAGGAGGGAGGCCCTGA
GGGAGCTGATGGAGCTCTACCTCCAGATGAAGCCTGTGTGGCGAGCCAGC
TGCCCAGCCAAGGAGTGCCCCGACCAGCTGTGCCGTTACAGCTTCAACTC
CCATCGCTTTGCTGACCTTCTGTCTTCCACCTTCAAATATAGGTACAAAG
GCAAGATACCCAATTACCTGCACAAGACCCTGGCCCATGTGCCTGAAATC
ATAGAGAGAGATGGATCCATAGGAGCCTGGGCCAGTGAGGGGAACGAGTC
AGCTAACAAATCATACACCATAGAGATGGGTCCCTTGGGTCCCAGGTGGA
AGGAGAGCCCTCAGCCTTTCTCCTGCTCCATTGAAGACCCTACAAAACAG
ACAAAGTTCAAAGGCATCAAGACGTACATTTCGTACCGGGTGACGCCGAG
CCATACGGGGCACCCAGTCTACCGGCGCTACAAACACTTTGACTGGCTGT
ACAACCGCCTGCTGCACAAGTTCACTGTGATCTCCGTGCCTCACCTGCCC
GAGAAGCAGGCCACGGGGCGATTCGAGGAAGACTTCATCGAGAAGCGCAA
GAGGCGACTGATACTGTGGATGAACCACATGACCAGCCACCCGGTCCTCT
CCCAGTACGAAGGCTTCGAGCACTTTCTGATGTGCGCCGACGACAAGCAG
TGGAAACTGGGCAAGAGACGGGCGGAGAAGGACGAAATGGTTGGCGCCCA
TTTCATGCTGACTCTGCAGATCCCCAACGAGCACCAAGACCTTCAGGACG
TGGAGGAGCGTATCGACTCCTTCAAGTCCTTCGCAAAGAAGATGGACGAC
AGCGTGATGCAGCTGACACACGTGGCCTCGGAGCTGGTTCGTAAGCACTT
GGGTGGCTTCAGGAAGGAGTTTCAGCGACTGGGAAACGCCTTTCAGTCCA
TCAGCCAGGCATTCATGCTGGACCCCCCGCACTGCTCAGAGGCCCTCAAC
ANNNNNNNNNNNNNN---

--------CATCACTCAACAGGCGAAGTCACGGAGAGGGAAGTGGCTCTC
GGGATAAACCCCTTTGCGGACGGGATGGGAGCCTTCAAGATCAACCACAG
CTCCCACGACCTGGGTTCCGG---ACAGACGGCGTTTTCCTCCCAGGCA-
--CCGGGCTACGCTGCCGCGGCCGCGCTGGGG---CACCACCA------C
CACCCGACGCACGTCGGCTCC---TACTCCACA------TTCAACTCCAC
CAGGGACTTTCTCTTCAGGAACCGGGGTTTCGGGGATGCAACCGG-----
-------GGCGCAGCACAGTTTGTTCGCCTC---------TGGAAGTTT-
--C------CCCGGCGCACATGGACACACGGAGGCTGCAGGACACCTGCT
CTTCCCGGGGCTCCATGAG---CAGGCAGCGGGACACGCATCCACCAACG
---------GGCAGATGCGACTCGGCTTCTCCGGTGATATGTACGGACGT
GCAGACCAGTACGGCCACGTTACCAGCCCCAGGT---CCGAGCACTACGC
CTCCACCCAGCTGCACGGCTACGGCCCCGTCAACATGAATATGGCTGCA-
--CACCACGGTGCAGGGGCCTTCTTTCGGTACATGCGGCAGCCGATCAAA
CAAGAGCTCATCTGCAAGTGGATCGAGCCGGAGCAGCTGACGAACCCCAA
AAAGTCGTGCAACAAAACTTTCAGCACCATGCACGAGCTGGTGACCCACT
TGACGGTGGAACACGTGGGGGGACCCGAGCAGACCAACCACGTCTGCTTC
TGGGAGGATTGCTCCAGAGAGGGGAAGCCCTTCAAAGCCAAATACAAACT
TGTAAATCACATCCGAGTGCACACCGGAGAAAAGCCCTTCCCGTGTCNNNNNNNNNNNNNNNNNN
>Gephyroberyx darwinii
AGCCTACTTATTCGAGCTGAGCTTAGCCAGCCCGGGGCACTTTTAGGAGA
CGACCAGATTTATAATGTTATTGTTACGGCGCATGCCTTTGTAATAATTT
TCTTTATAGTAATACCAATTATAATTGGAGGATTTGGAAACTGACTAGTC
CCCATAATGATTGGAGCCCCTGATATGGCTTTCCCTCGAATAAACAACAT
AAGCTTCTGACTCCTCCCTCCCTCATTCCTTCTGCTCCTTGCCTCTTCTG
GCGTTGAAGCAGGGGCAGGAACAGGATGAACGGTCTACCCACCCCTCGCG
GGGAACCTTGCACACGCAGGGGCTTCTGTAGACCTAACTATTTTCTCACT
GCATTTAGCAGGTGTTTCTTCCATTCTTGGGGCCATCAACTTTATCACAA
CCATTATTAACATAAAACCCCCAGCAATTTCCCAATACCAAACCCCATTA
TTTGTATGATCCGTTTTAATTACAGCAGTCCTTCTTCTTCTATCACTTCC
CGTCCTTGCAGCCGGCATTACCATGCTCCTAACAGACCGTAATCTAAATA
CAACCTTCTTTGACCCCGCAGGAGGGGGAGACCCCATTCTATATCAACAT
CTA---

------------TTTCTAGAGAGAAACCTTCATCCATCTAACTGCCTAGG
CATGCTGTTGCTGTCTGATGCACACCAGTGTACCAAGCTATCAGAGCTCT
CCTGGGGCATGTGTCTCAGCAACTTTCCGGCTATTTGCAAGACAGAAGAC
TTCCTCCAGCTGCCCAAAGACATGGTGGTGCAGCTTCTATCCCACGAGGA
GCTGGAGACAGAAGATGAGAGACTGGTTTATGAAGCTGCCCTTAACTGGG
TCAACTATGACTTGGAAAGGAGGCACTGCCATCTGCCAGAGCTGCTGAGA
ACGGTTCGCCTGGCCCTGCTACCTGCCATCTTCCTCATGGAGAATGTCTC
TACAGAAGAGCTGATCAATGCCCAGGCCAAGAGCAAGGAGCTCGTGGATG
AGGCCATCCGGTGCAAGCTAAAGATCTTGCAGAATGACGGTGTGGTTAAC
AGCCCCTGTGCTCGGCCGAGAAAAACCAGCCATGCCCTTTTTCTGTTGGG
AGGTCAGACCTTCATGTGTGACAAGCTGTATCTGGTGGACCAGAAGGCAA
AAGAGATCATCCCCAAGGCTGATATCCCCAGTCCTAGGAAGGAGTTCAGT
GCCTGCGCCATCGGCTGTAAGGTCTACATCACAGGTGGCA--GAGGCTC-
AGAAAATGGTGTGTCTAAAGATGTATGGGTCTATGACACCGTCCATGAGG
AATGGTCCAAGGCAGCACCCATGCTCATTGCCAGATTTGGTCATGGCTCT
GCTGAGCTGAAACACTGCCTTTATGTAGTAGGAGGTCACACGGCAGCAAC
TGGCTGCCTCCCAGCCTCTCCCTCCGGATGAATATATTGTAGTGTTCAGC
CGTTCAACAACAAGGCTGATTCTGAACGAAGCGGAGCTAATCATGACGCT
GGCCCAGGAATTTCAGATGAGAGTGGTTACGGTCTCCCTGGAGGAACAAA
CTTTCCCCAGCATCGCCCAGGTGATCAGTGGGGCCTCCATGTTAGTCAGC
ATGCATGGAGCTCAGCTTGTCACCTCACTCTTCCTCCCCAGAGGAGCTGC
TGTGGTAGAGCTGTTCCCCTATGCTGTCAACCCAGAACAGTACACCCCAT
ATAAAACCCTCGCCTCCTTACCAGGCATGGATCTTCAATATGTTTCCTGG
AGGAACACTATAGAGGAGAACACTGTCACCCACCCAGACAGACCTTGGGA
AGAAGGAGGCATCTCCCATTTGGAAAAGGACGAGCAAGAGCGAATACTGG
CCAGCAGGGATGTGCCCAGACACCTGTGCTGCCGCAACCCAGAGTGGCTC
TTTCGAATCTACCAGGACACTTTGGTGGACATCCCTTCATTCCTGGAGGT
CCTCAA---AGAGGGCCTGAAG---ACCAGGCCCAGCTTGAAGAA---GG
CCAAGCTGGCCAGCACGGTTCATCCAGGCCGGGTCAGAGAACCCCAGTGC
CACACCTCAGTCCAAGCCACCAACGAGGCTAAACTCACGGTTTCCTGGCA
GATCCCATGGAATCTGAAATACCTGAAGGTGAGAGAAGTGAAGTACGAGG
TGTGGATCCAGAAGAAGGATACCAGCAAGGGGACACTGGAGGATCAAATC
ATCCAGGCAAACCCCGCGCTGGAGGCTTTTGGTAACGCCAAAACAGTGAG
GAATGACAACTCATCCCGTTTTGGAAAATTCATCCGAATTCATTTCGGAA
CCAGTGGCAAGTTGTCCTCTGCTGATATTGAAACTTACTTATTGGAGAAG
TCACGTGTCACCTTTCAGCTCAAGGCTGAGAGGAACTACCATATCTTCTT
CCAGATATTGTCCAATCAGAAGCCAGAGCTGTTGGACATGCTGTTGATCA
CTAACAACCCATATGACTACTCCTACATCTCCCAAGGAGAGGTAACAGTA
GCATCCATCAACGACTCAGAGGAGCTGATGGCCACTGACAGCGCCTTTGA
TGTGCTTGGCTTTACTCAAGAGGAGAAGATGGGAGTATATAAGCTGATTG
GTGCCATTATGCACTATGGCAACATGAAGTTTAAGCAGAAGCAGCGTGAG
GAGCAGGSTGAGCCTGACGGAACAGAGGCTGCTGATAAGTCAGCTTACCT
TATGGGGCTGAACTCTGCAGACCTCATCAAAGGGCTATGCCATCCCAGAG
TCAAGGTAGGAAATGAATATGTCACCAAAGGCCANNNNNNNNNNNNNNNNNNNNNCCAAACAAGGAGGC
CT
TCAAGTGCGAGGAGTGTGGCAAGCACTACAACACCAAGCTGGGATACAAA
CGCCATGTGGCCATGCACTCTGCCACAGCAGGGGACCTCACCTGTAAAGT
GTGCATGCAGAGCTATGAGAGCACGCCGGTGCTCCTGGAGCACCTTAAGA
GCCACTCGGGGAAGTCCTCGGGTGGCGCCAAGGAGAAAAAACACCCATGC
GACCACTGCGACCGCCGTTTCTACACACGGAAGGATGTGAGACGGCACAT
GGTGGTCCACACGGGCCGAAAAGACTTCCTGTGCCAGTACTGTGCCCAGC
GCTTTGGCAGGAAGGACCACCTGACACGCCACGTAAAGAAGAGCCACTCG

CAGGAGCTGCTGAAGATCAAGACGGAGCCTCCAGATATGTTAGGTCTTTT
AGGTTCTGGCTCGCCGCCATGCTCTGTCAAGGAGGAGCTTAGCCCTATGA
TGTGCAGCATGGGTCCCAACAAAGACCCCATGATGGGCAAACCCTTCCCC
AGCGGGACCCCCTTCCCCATGGGCATGTACAACCCCCACCAC------CT
CCAGGCCATGTCCAATTCTGGGGTGGGTCACCCC------CACCCCTCCC
TGATGTCTAGCTCCCTGTCTGCAGCTATGGGCATGGGCTGTCACATGGAA
TATCTCATTTATGCCTCTTTCTCATTCATGGGATGTTTACAAATCAGTGA
TGGCTCAAACATTGTGAACTTGCTGGCTAGTAACTCTCCGAGCGTTTCGT
ATGCTCTGACCCAGCAGAAATACTTCAGTAACTACAGTCCCGTGATTGGA
TTTTACATTTACGAGCCCATTGAGTACTGGAACTCCACAGTGCAGGAGCA
CCTGAAGACACTGAGTCATGGCTTCAACAAGATATCCTGGATGGACAACT
TCTTCCACTACCTGCGGGTGGTGAACGTAAGCGCGTCAACCAAGAATGAC
TTTATCAGCATCCTCAAGGGCTCTTTCCTGCGCAGCCCGGAGTACCAGCA
CTTCACTGAGGACATCATCTTCTCCAAGA---ACCGTGAGAGTG------
ATGAGTATGACATTATTGCCTCACGCATGTACCTAGTGGCGCGGACCACA
GAGAAGAAGCGCGAGGAGGTGGTGGAGCTTCTGGAGAAACTGCGTCCACT
GATGCTGATCAATAGCATCAAGTTCATCGCCTTCAACCCCACCTTTGTTT
TCATGGACCGCTACAGCTCCTCAGTCATCTCGCCCATCCTGACCTCAGGC
TTCAGCGTGCTTACCATCCTCATCCTCACTTTCTTTCTGGTCATCAACCC
CTTGGGGAACTTCTGGCTCATCCTCACAGTTACTTCTGTGGAGCTGGGTG
TCTTGGGTTTAATGGGCTATCATCTATTCGAATGGCAGCCGGCCCTCAAG
AATGTGTCTGCATCCTGTCATGTGGGCATTATTAATGGGCTCTCAGGATT
GGCTGCCTTGGTGGATGACATCCCAGCTGACACCATTACTCGACGGTTTC
GCTACGACGTGGCCCTGGTATCAGCCTTAAAGGATCTGGAGGAGCACATC
ATGGAGGGACTGAAAGAGTGTGGGCTGGAAGACAGTGCTTGCACCTCAGG
CTTCAGTGTTCTGATCAAGGAATCTTGTGACGGCATGGGAGATGTCAGCG
AAAAGCACGGGGGAGGGCCAGTGGTCCCCGAGAAGGCTGTACGCTTCTCT
ATTACTGTTATGTCTGTCTCTGTCCTGGCAGATGGAGAGGAA--------
----------------------GAGGCAGTTACCATCTTTACCGAGCCAA
AGCCCAACTCAGAACTGTCCTGTAAGCCCCTAAGCCTGATGTTTGTGGAT
GAGTCAGACCATGAGACACTCACTGCTGTCCTGGGACCTTTAGTTGCAGA
GCGTAATGCAATGAAGCAGAGTCAACTCATCCTTTCTATGGGTGGCCTCC
CTCGCTCCTTCCGCTTCCACTTCAGAGCCACAGGATATGATGAGAAGATG
GTGCGTGACATGGAGGGCCTGGAGGCCTCAGGCTCCACCTATATCTGCAC
TCTGTGTGATTCCAGTCGAGCAGAAGCCTCTCACAACATGATCCTACACT
CCATCACCCGCAACCACGGTGAGAACCTGGAGCGTTATGAAATATGGAGG
ACCAACCCCTTTTCTGAGTCTGTTGAGGAGCTGCGAGACCGGGTCAAAGG
GGTCTCTGCCAAGCCCTTTATGGAGACCCAGCCCACTATTGATGCATTAC
ACTGCGACATTGGTAATGCAACAGAGTTCTACAAAATCTTCCAGGATGAG
ATTGGGGARGTGTACCAGAAGGC---CAAC---CCCACCCGGGAGGAGCG
GCGGAGATGGCGAGCAGCTCTAGACAAGCACCTGAGGAAGAAGATGAAGC
TTAAACCAGTGATGAGGATGAATGGGAACTATGCCCGGAGGCTAATGACC
CAAGAGGCTGTGAAGGTGGTGTGTGAGCTGGTACCCTCAGAAGAGAGGCG
AGAGGCCCTGAGGGAGCTCATGGGGCTCTACGTCCAGATGAAGCCTGTGT
GGCGCGCTACCTGCCCAGCCAAGGAATGCCCTGACGAGCTGTGCCGCTAC
AGCTTTAACTCCCAGAACTTTGCCAATCTCCTCTCCACTACCTTCAAATA
TAGGTACAATGGAAAGATCACCAATTATCTGCATAAGACCCTCGCCCATG
TCCCCGAAATCATTGAAAGAGATGGCTCTATAGGAGCCTGGGCCAGCGAG
GAAAACGAGTCAGCAAACAAATCGTACACCATTGAGATGGCTCCAAAGGG
GCCCCAATGGAAGGAGAGTCCTCAGCCTTTCTCCTGCTCCATTGAAGACC
CCACAAAACAGACCAAGTTCAAAGGCATCAAGACCTACATTTCGTACCGG
GTCACACCGAGCCACACAGGGCGTCCCGTCTACAGACGCTACAAACACTT
TGACTGGCTGTACAACCGCTTGCTGCACAAGTTCACTGTGATCTCGGTGC

CCCACCTGCCTGAGAAGCAGGCCACGGGGCGCTTCGAGGAGGACTTCATC
GAGAAGCGTAAAAGACGACTGATTCTGTGGATGAACCACATGACCAGTCA
CCCAGTCCTCTCCCAGTACGAGGGATTTGAGCACTTCCTTATGTGTGCTG
ACGACAAGCAGTGGAAGCTGGGTAAGAGGCGGGCGGAGAAGGACGAGATG
GTGGGCGCCCACTTCATGCTGACCTTCCAGATTCCTAACGAGCATCAGGA
TCTCCAGGATGTGGAGGAGCGGGTTGACTCCTTCAAGTCCTTTGCTAAGA
AAATGGACGACAGCGTCATGCAGCTCACACATGTTGCCTCGGAGCTGGTG
CGTAAGCACCTGGGTGGATTCAGGAAGGAGTTCCAGCGGCTAGGAAATGC
CTTCCAGTCCATCAGCCAGGCGTTCATGCTGGAACCTCCGCACAGCTCTG
ATGCCCTCAACAACGCCATCTCCCACCTCTCTCGCACGTTCCTCAAACTG
ACCTCCCTGGGTTTCATCATTGGAGTCGGTGTGGTCGGGAACCTCCTGAT
CTCCATCCTGCTGGTCAAAGACAAGAGCCTGCACCGCGCACCCTACTACT
TCCTGCTAGACCTGTGCGCCTCCGACATCCTGCGCTCTGCCATCTGCTTC
CCCTTCGTCTTCACCTCTGTCAAGAATGGATCCGCCTGGACCTATGGCAC
GCTCACCTGCAAAGTGATCGCCTTCCTCGGCGTGCTCTCCTGTTTCCACA
CAGCGTTCATGCTATTCTGTGTCAGTGTCACTCGCTACTTGGCTATTGCA
CATCACCGCTTCTACACCAAGAGGCTGACCTTCTGGACCTGTCTAGCTGT
CATCTGCATGGTGTGGACGTTGTCAGTAGCCATGGCTTTTCCCCCGGTGC
TGGACGTAGGGACGTACTCCTTCATCCGGGAGGAGGACCAGTGCACATTC
CAGCACCGTTCTTTCAGGGCCAATGACTCACTAGGCTTCATGCTCCTGCT
GGCACTCATCCTCCTGGCCACACAGCTGGTTTACCTCAAGCTCATCTTCT
TTGTCCACGACCGTCGGAAGATGAAGCCCGTCCAGTTCGTGCCTGCCGTC
AGCCAGAACTGGACCTTCCATGGGCCAGGCGCCAGTGGGCAGGCGGCGGC
TAACTGGCTCGCTGGATTTGGGAGAGGCCCCACCCCGCCTACCTTGCTGG
GCATCCGGCAGAACAGCAACGCGGCGGGCCGCAGGCGTCTGCTGGTGCTG
GATGAGTTCAAAACAGAGAAGAGGATTAGTAGGATGTTCTACATCATGAC
GTTTTTCTTCCTGGCGTTGTGGGGGCCCTATCTGGTAGCCTGCTACTGGC
GGGTGTTTGCAAGGGGCCCCGTAGTCCCCGGGGGCTACCTGACGGCAGCC
GTGTGGATGAGCTTTGCCCAGGCTGGGGTCAATCCATTCATCTGCATCTT
CTCTAACAGGGAGGCCAAATCTCGCTTTCACCCTGGCGTGGGGACTGGTC
CTGGCACGGAGC---GCAGCGTCCCACTCGGCAACAGCTTGCTATCCCCG
CAGCAAACCGAGGAGCCCACTGTTGCCACCCCCCCGCAGCGATGGTTTGT
CACCC---CTGCCAACAACCGACTGGACTTTGCTGCCTCGGCATACGACG
CGGCC---------GATTTCGCCGGTAACGCGGCCACCTTGCTGTCCTAC
GCAGCGGCCGGAGTGAAGGCTC------TTCCTCTGCCGGCTGCAGGCTG
CTCCAACCGGCCTCTTGGCTATTACGCAGACCCGTCGG---GCTGG---G
GCGGACGCACGCCGCCGCAGTACTGTAGCGTAAACAGCAAACCAAGCTCG
GTCCTTTCCTGCTGGCCCTCTAACTCTGTCGGCGGCAGAGCAGGCG---C
C---AACTATCTGG---------CCGAGGA---CGGA---GACGC---CA
TCCCGACGGAGAGATCCCCG---AT---CGGTGGCTCCGAGGAG---ACG
AAACCCAAGGACCT------GTCGGA---GTCGAGCTGGATAGAG---AC
GCCGTCCTCCATTAAGTCAATTGATTCAAGCGATTCTGGTATCTTCG---
AACAGGCCAAAAGGAGAAGAATCTCGCCCTCTGCCACGCCG---------
GTTTCAGAAACTGTGTCCCCGTTAAAATCTNNNCATCACTCAACAGGCGAAGT
CACAGAGAGAGAAGTGGCGTTGGGGATAAATCCGTTCGCGGACGGGATGG
GCGCCTTCAAAATCAACCACAGCTCCCACGATATTGGCTCCGG---ACAG
ACGGCGTTTTCCTCCCAGGCG---CCCGGCTAC---GCAGCGGCCGCCCT
GGGA---CATCATCA------CCACCCGACCCACGTTGGCTCT---TACT
CCACGGCGGCTTTCAACTCCACCCGGGACTTTCTCTTTAGAAATCGGGGT
TTCGGAGACGCCACCGG------------GGCGCAGCACAGTTTGTTCGC
CTC---------CGGAAGTTT---C------GCAGGGCCACATGGACACT
CAGATGCCGCGGGACACCTGCTCTTCCCAGGACTCCACGAG---CAAGCA
GCGAGCCATGCGTCTTCTAATGTGGTCAACAGCCAGATGCGATTGGGCTT

CTCGGGGGACATGTATGGTCGGGCCGACCAGTATGGCCACGTTACAAGCC
CAAGGT---CCGACCACTATGCTTCGACCCAGTTGCACGGCTATGGCCCC
ATGAACATGAATATGGCCGCA---CACCACGGAGCAGGGGCCTTCTTTCG
ATACATGAGGCAGCCGATCAAACAAGAGCTCATCTGCAAGTGGATCGAGC
CGGAGCAACTGACGAATCCCAAAAAGTCGTGCAACAAAACTTTTAGTACG
ATGCATGAGCTGGTGACCCATCTGACGGTGGAGCATGTGGGGGGACCAGA
GCAGTCCAACCACATCTGCTTCTGGGAAGACTGCGCCAGAGAAGGGAAAC
CATTCAAAGCCAAATACAAACTTGTAAATCATATCAGAGTACACACCGGA
GAGAAACCCTTTCCGTGTCCGTTTCCCGGCTGTGGCAAA
>Gigantactis vanhoeffeni
AGCCTATTAATCCGCGCCGAGCTCAGCCAACCAGGCGCCCTCTTGGGTGA
CGACCAGATTTATAATGTGATTGTCACAGCCCATGCCTTTGTAATAATCT
TCTTTATGGTAATACCCATTATGATTGGGGGATTTGGGAATTGATTAATC
CCCCTAATAATCGGTGCCCCTGATATGGCCTTTCCCCGCATGAATAATAT
AAGCTTCTGACTACTCCCCCCCTCTTTCCTCCTCCTCCTCGCCTCATCGG
GGGTAGAGGCCGGGGCGGGCACTGGGTGAACAGTTTACCCGCCTCTGTCG
GGCAATTTAGCTCATGCTGGGGCCTCTGTAGATCTAACAATCTTCTCCCT
TCACCTAGCAGGGGTGTCGTCAATTTTAGGGGCCATCAACTTCATTACAA
CTATTATTAATATAAAACCCCCTGCCACCTCTCAATACCAAACGCCACTA
TTCGTATGATCAGTCCTAATCACAGCAGTCCTCCTTCTACTCTCTCTGCC
TGTCCTTGCTGCCGGTATTACCATACTTTTAACTGACCGCAACCTAAACA
CAACCTTCTTTGACCCCGCAGGGGGGGGCGACCCCGTCCTCTACCAGCA-

------------TTCCTGGAGAGGAACCTTCACCCGTCTAACTGCCTCGG
CATGCTGTTGCTGTCTGACGCACACCAGTGCACCAAGCTGTCGGAGCTCT
CCTGGGGCATGTGCCTCAGCAACTTCCCCGCCATTTGCAAGACAGAGGAC
TTTCTCCAACTGCCAAAAGATATGGTGGTGCAGCTCTTGTCACACGAGGA
GTTAGAGACCGAAGACGAGAGACTTGTTTATGACGCCGCCCTCAATTGGA
TCAACTATGACCTGGAGAAGAGGCACTGCCACCTTCCAGAGCTTTTGAGA
ACGGTCCGCCTGGCCCTGCTGCCTGCCATCTTTCTCATGGAGAATGTCTC
CACAGAAGAGCTGATCAACGCCCAAGCCAAGAGCAAGGAGCTGGTGGACG
AGGCGATCCGCTGCAAGCTGAAGATCTTGCAGAACGACGGCGTCGTAAAC
AGCCCGTGTGCTCGACCAAGAAAAACCAGCCACGCCCTTTTTCTCCTGGG
CGGTCAGACGTTCATGTGTGACAAGCTGTATCTGGTGGACCAGAAAGCCA
AAGAGATCATCCCCAAAGCCGACATTCCCAGCCCCAGGAAGGAGTTCAGC
GCCTGTGCCATCGGCTGCAAGGTGTACATCACTGGAGGGA--GGGGCTC-
AGAGAACGGCGTGTCCAAAGACGTGTGGGTCTACGACACGGTCCACGAGG
AATGGTCCAAGGCGGCGCCCATGCTCATCGCCAGGTTCGGCCACGGCTCC
GCAGAGCTCAAACACTGCCTCTACGTGGTGGGAGGTCACACCGCGGCCAC
CGGCTGCCTCCCGGCCTCTCCGTCCGGGTGAATACATTGTTGTGTTCAGT
CGTTCAACAACCAGACTGGTRCTGAATGAAGCGGAGCTRATCATGGCACT
GGCTCAGGAGTTCCAGATGAGAGTGGTCACAGTATCCCTGGAGGAGCAGT
CRTTCCCTAGTATCATCCAGGTGATCAGCAGCGCTACCATGTTAGTCAGT
ATGCATGGAGCTCAACTCATCACCTCACTCTTCCTCCCCAGAGGAGCTGT
CGTGGTGGAGCTGTTCCCCTTTGCAGTGAACCCAGAGCAGTACACCCCGT
ATAAAACCCTYGCCTCCCTTCCGGGCATGGACCTTCATTATATCTCCTGG
AGGAACACTAAGGAGGATAACACCATCACCCACCCAGACAGGCCCTGGGA
ACAAGGGGGTATTGCTCACTTGGATAAGGAGGAGCAAGAACGAATACTAA
TGAGTAAAGACGTGCCCAGGCATCTGTGCTGCCGGAACCCAGAGTGGCTC
TTCAGGATCTATCAGGACACATTGGTGGACATTCCTTCCTTCCTGGAAGT

CATCAA---AGAGGACATGAAG---ACAAAGCCCAACTTGAAGAA---GT
CAAAGCCGGCCAGCACACTCCACCCGGGCCGGGTCAGAGAACCTCTGTGT
CAGACCTCAGTTCAAACCAGTAATGAGGCTAAACTCACAGTCTCCTGGCA
GATACCGTGGAATCTGAAGTATCTGAAGGTCAGAGAGGTGAAGTACGAGG
TG---------AAAAGAGACACCAGCAAGGGGACCCTGGAGGATCAAATC
ATCCAAGCAAACCCGGCGTTGGAGGCCTTTGGCAACGCCAAAACAGTGAG
GAATGACAACTCATCCCGTTTTGGAAAGTTCATCCGAATCCACTTTGGTA
CAAGCGGCAAGCTGTCGTCTGCCGACATCGAAACGTACCTGCTGGAGAAG
TCCCGTGTTACCTTCCAGCTCAAGGCTGAGAGAAACTATCACATCTTCTA
CCAGATCCTGTCCAACCAGAAACCAGAGCTGCTGGACATGCTGCTGATCA
CCAACAATCCCTACGACTACTCCTACATCTCCCAAGGAGAGGTAACCGTC
GCCTCCATCAACGACTCAGAGGAGCTGATGGCCACGGACAGCGCCTTCGA
TGTGCTCGGCTTCACTCCCGATGAGAAGGTGGGCGTCTATAAACTCACAG
GTGCCATCATGCACTATGGCAACATGAAGTTCAAACAGAGGCAGCGCGAG
GAGCAGGCGGAGCCCGATGGGACGGAGGCCGCTGATAAGTCAGCTTACCT
AATGGGGCTGAACTCTGCTGACCTCATCAAGGGGCTGTGTCATCCCCGAG
TCAAGGTGGGGAATGAGTATGTCACCAAAGGTCAAAGTGTGGATCAAGTC
TACTATCCCAACAAGGAGGCCTTCAAATGTGACGAGTGTGGGAAGCACTA
CAACACCAAGCTGGGATACAAGCGCCATGTTGCCATGCATTCAGCCACGG
CGGGGGATCTCACCTGTAAAGTGTGCATGCAGACCTACGACAGCACACCC
ATGCTCCTGGAGCACCTGAAGAGCCACTCTGGGAAGTCCTCAAGTGGCAC
CAAGGAGAAGAAACACCAGTGCGACCATTGTGACCGGCGTTTCTACACGA
GGAAGGATGTGAGACGCCACATGGTGGTCCACACGGGCCGAAAGGACTTC
CTCTGCCAGTACTGTGCCCAGCGCTTCGGCAGGAAGGACCATCTGACACG
CCATGTGAAGAAGAGCCACTCACAGGAGCTGCTGAAAATCAAGACAGAGC
CCCCCGATATGTTAGGTCTTCTAGCTTCTGGGTCACCACCGTGTTCTGTG
AAGGAGGAGCTCAGCCCCATGATGTGCGGTATGGGTCCCAACAAAGACCC
CATGATGAGCAAACCCTTTCCCAGTGGGGCCCCTTTTCCAATGGGCATGT
ACAACCCTCATCAC------CTCCAGGCCATGTCGAATTCTGGGGTAGGT
CACTCC------CACCCATCCCTGATGCCCAGTTCTTTGTCTGCAGCTAT
GGGCATGGGCTGTCACATGGAGTACCTCATCTACGCCTCTTTCTCATTCA
TGGGATGTTTACAAATCAGCGATGGATCAAATATCGTGAACCTGCTGGCA
AGTAACTCTCCAAGTGTTTCATATGCTCTGACACAGCAAAAATACTTTAG
TAACTATAGTCCAGTCATTGGGTTTTACATTTATGAGCCTATCGAGTACT
GGAACTCCACAGTGCAGGAGCACCTGAAGACTCTGAGTCATGGCTTCAAC
AAGATCTCCTGGATGGACAACTTTTTTCACTACCTGCGGGTGGTGAATGT
GAGCGCATCAACCAAGAGCGACTTCATCTCCATCCTCAAGGGCTCCTTCC
TGCGCAGCCCGGAGTACCAGCACTTCACTGAGGACATCATTTTCTCAAAG
A---ACCGTGAGACCG------ATGAGTACGACATCATCGCCTCGCGGAT
GTACTTGGTGGCACGGACTACAGAGAAGAAGCGTGAAGAAGTGGTGGAGC
TCCTGGAAAAACTCCGTCCACTGATGCTGATCAACAGCATCAAGTTCATC
GCCTTCAATCCTACGTTTGTGTTCATGGACCGCTACAGCTCCTCCGTCAT
CTCACCCATCCTGACGTCAGGATTCAGCGTACTCACAATCCTCATCCTCA
CTTTCTTCCTGGTCATCAACCCCTTGGGTAACTTCTGGCTCATCCTCACT
CTAACGTCTATGGAGCTGGGCGTTTTGGGTTTGATGNNNNNNNNNNNNNNNNNNNNNNNNNNNNNNNN
NNNNNNNNNNNNNNNCTTCCAATGTTGGC
ATTATTAATGGTCTCTCTGGATGGGATTCCTCAGTGGATGACTCCCCAGC
TGACACCATCACTCGACGGTTTCGCTATGATGTGGCACTGGTAGCAGCAT
TAAAGGATCTTGAGGAGGACATCATGGAAGGACTGAGAGAGAAGGGAATG
GAGGACAATGCTTGTA------ACTTCACTGCCATGATCAAAGAATCCTG
TGATGGCATGGGCGACGTCAGTGAGAAGCATGGTGGAGGACCAGCTATCC
CTGAGAAGGTTGTGCGTTTCTCTTTCACTGTTATGTCCGTGTCTTTCCTC
GCAGACAGTCAGCAG-------------------------------GAAGA

GGTCACCATCTTCACAGAGCCAAAGCCAAATTCAGAACTGTCCTGTAAGC
CCCTTTGCCTGATGTCTGTGGATGAATCGGACCATGAGACACTCACAGCC
ATCCTGTGTCCTTTAATTGCAGAGCGCAACGCAATGAAAGAGAGCCGGCT
CATCCTGCCCATTGGTGAACTTCCTCGCTCCTTCCGCTTTCATTTAGAG
GCACGGGATATGATGAGAAGATGGTGCGTGTGATGGAGGGCCTGGAGGCC
TCTGGGTCCACCTACATCTGCACCCTCTGTGACTCCAGTCGTGCAGAGGC
CTCTCAAAACATGGTGCTACACTGCATCACCCGCAGTTACGAGGAGAACC
AAGAACGTTATGAAATATGGAGAACCAACCCATTTTCTGAGTCTGTGGAT
GAGTTGCGAGATAGAGTCAAAGGAGTCTCTGCCAAGCCCTTCATGGAGAC
CCATCCCACACTGGACGCCTTACACTGTGACATCGGCAATGCCTCCGAGT
TTTACAAAATATTCCAAGATGAGATTGGGGAGGTGTACCAAAAGGT---C
AAT---CCCAGCCGGGAGGAGCGGCGCAGCTGGAGGACAGCCCTCGATAA
ACAGCTGAGGGATAGGATGAAGCTCAAACCAGTGATGAGGTTGAATGGGA
ACTATGCCCGCAGGCTAATGACCATGGAGGCTGTGGAGGTGGTATGTGAA
CTGGTGCCCTCAGAGGAGAGGAGGGAGGCCCTGAGGGAGCTTATGAAACT
CTACCTCCAGATGAGGCCGGTGTGGCGCGCCACCTGTCCGGCCAAGGAGT
GCCCCGACCAGCTGTGCCGCTACAGCTTTAACTCCCAGCGCTTTGCTGAG
GTCCTCTCCTCTAAGTTCAAATATAGATACAATGGAAAGATAACCAATTA
CCTGCACAAGACTCTAGCTCATGTGCCTGAAATCATAGAGAGAGATGGAT
CCATAGGAGCCTGGGCCAGCGAGNNNNNNNNNNNNNNNNNNNNNTCCTACACCATTGAGATGGTTCCGCT
G
GGGCCCCGGTGGAAGGAGAGCCCACAGCCGTTCTCCTGCTCCGTTGAAGA
CCCCACGAAACAGACCAAGTTCAAGGGCATCAAGACGTACATTTCGTACC
GGGTGACGCCGAGCCACACGGGGCGCCCCGTCTACCGACGCTACAAACAC
TTCGACTGGCTGTACAACCGCCTACTGCACAAGTTCACCGTGATCTCCGT
GCCTCACCTGCCCGAGAAGCAGGCGACGGGGCGATTTGAGGAAGACTTCA
TCGAGAAGCGCAAGAGGCGACTGATACTGTGGATGAACCACATGACCAGC
CACCCGGTCCTCTCCCAGTACGAAGGGTTCGAGCACTTCCTGATGTGTGC
CGACGACAAGCAGTGGAAGCTGGGCAAGCGACGGGCGGAAAAAGACGAGA
TGGTGGGTGCCCATTTCATGCTGACCCTCCAGATCCCTAAGGAGCACCAG
GACCTGCAGGACGTCGAGGAGCGGATTGACACCTTCAAGTCCTTCGCCAA
GAAGATGGACGAGAGCGTGATGCAGCTCACGCACGTCGCCTCGGAGCTGG
TCCGCAAGCACCTGGGCGGGTTCAGGAGGGAGTTCCAGAGGCTGGGAAAC
GCCTTCCAGTCCATCAGCCANNNNNNNNNNNNNNNNNNNNNNNNNNNNNNNNNNNNNNNNNNNNNNNNNN
NNNNNNNN------------------------------

>Gigantura indica
AGCCTGCTTATCCGAGCGGAACTCAGCCAACCCGGCGCCCTTTTAGGCGA
TGACCAGATCTACAACGTTATCGTTACCGCCCACGCCTTCGTAATGATTT
TCTTTATGGTAATGCCCATCATGATTGGCGGGTTCGGGAACTGACTTATC
CCCCTTATAATCGGAGCCCCCGACATGGCATTTCCCCGAATGAACAACAT
GAGCTTCTGGCTCTTACCTCCCTCTTTCCTGCTGCTCCTGGCCTCCTCTG
GAGTAGAAGCCGGGGCCGGAACGGGCTGAACCGTTTACCCACCCCTCTCC
GGCAACCTCGCGCATGCGGGTGCCTCCGTAGACCTAACCATTTTCTCCCT
ACATCTGGCGGGTGTTTCTTCCATCCTCGGTGCTATCAACTTTATCACGA
CAATTCTTAACATGAAACCCCCCGCTATTACCCAATACCAGACCCCCTTA
TTCGTCTGATCAGTTCTTATCACAGCCGTCCTACTACTGCTCTCTCTACC
AGTCCTTGCGGCGGGAATCACTATGCTCCTCACAGACCGAAACTTAAACA
CAACCTTCTTCGACCCCGCAGGCGGGGGGGACCCCATTCTTTACCAACAC
CTATTCTGGTTCTTCGGACACCCGGAGGTATACATCCTTATCCTCCCTGG
ATTCGGCATGATCTCCCACATTGTTGCCTACTACTCGGGGAAAAAAGAAC
CTTTCGGCTACATGGGCATGGTCTGGGCAATAATGGCCATCGGCCTTCTT
GGCTTCATTGTCTGAGCCCATCACATGTTCACAGTAGGCATGGACGTAGA
CACACGTGCTTATTCCTGGAGAGGAACCTGCACCCATCCAACTGCCTTGG
CATGCTGCTGCTGTCAGATGCCCACCAGTGCACCAAGCTGTCAGAGCTGT

CCTGGGGTATGTGTCTCAGCAACTTCCCTGCCATCTGCAAGACAGAGGAC
TTCCTCCAGCTTCCCAAAGACATGGTGGTGCAGCTTCTGTCCCACGAGGA
GCTGGAGACGGAGGATGAGAGACTGGTTTACGAGGCTGCCCTTAACTGGG
TCAACTACGACCTGGAGAGGAGACACTGCCACCTGCCGGAGCTGCTGAGG
ACCGTGCGCCTGGCCTTGCTTCCCGCCATCTTCCTCATGGAGAACGTCTC
CACAGAGGAGCTGATCAATGCCCAAGTAAAGAGCAAGGAGTTGGTGGACG
AGGCCATCCGCTGCAAGCTGAAGATCCTGCAGAATGATGGTGTGGTCAAC
AGTCCTTGTGCCCGGCCCAGGAAGACCAGCCATGCCCTCTTCTTGCTGGG
AGGACAGACCTTCATGTGTGACAAGCTGTACTTGGTGGACCAGAAGGCCA
AGGAGATCATCCCCAAGGCGGACATCCCCAGCCCCAGGAAGGAGTTCAGC
GCCTGTGCCATCGGCTGTAAGGTCTACGTCACGGGCGGGA--GAGGCTC-
CGAGAACGGCGTGTCTAAAGATGTGTGGGTCTACGACACCGTCCATGAGG
AGTGGTCTAAGGCAGCACCTATGCTCATTGCCAGGTTTGGCCACGGCTCT
GCTGAGCTCAAACACTGTCTCTATGTGGTCGGAGGACACACAGCAGCCAC
CGGCTGCCTCCCAGCCTCTCCGTCTAGATGAGTACATCGTAGTGTTCAGT
CGTTCTACAACAAGGCTGATTCTAAACGAAGCAGAACTGATCCTGGCACT
GGCCCAAGAGTTTCAGATGAGGGTGGTTACAGTGTCCCTAGAGGATCAGT
CTTTCCCCAGCATCGTCCAGGTGATCAGCGGGGCCTCCATGTTGGTTAGT
ATGCACGGAGCTCAGCTCGTCACCTCCCTCTTCCTCCCTAGAGGAGCTGC
TGTAGTGGAGCTCTTCCCCTATGCTGTGAATCCAGAACAGTACACCCCGT
ACAAAACCCTAGCCTCCCTACCAGGCATGGACCTTCAATACGTTTCCTGG
AGGAACACCGTTGAGGAGAACACTGTCGCTCACCCGGACAGACCCTGGGA
CCAAGGAGGCATCGCCCATTTGGAGAAAGAAGAGCAGGAGAGAATCCTAG
CCAGCAAGGATGTCCCCAGGCACCTATGCTGCCGCAACCCAGAGTGGCTC
TTCAGGATCTACCAGGACACTCTGGTGGACATCCCCTCCTTCCTTGAAGT
CCTCAA---GGAGGGCCTGAAG---ACGAGGCCAAACTTGAAAAA---GT
CCAAGCCTGCCAGCACAGTTCACCCGGGCCGGGTCAGAGAACCCCAGTGC
CAGACATCACTCCAAGCCACCAATGAG-CTAAGCTCACTGTATCCTGGCA
GATCCCGTGGAACCTGAAGTACCTAAAGGTGCGAGAGGTGAAGTACGAGG
TATGAATCCGGAAAAAGGATTCAAGCAAGGGAACCCTGGAGGATCAGATT
ATCCAGGCAAACCCTGCCCTGGAGGCTTTTGGAAACGCCAAAACAGCGAG
GAACGATAATTCTTCCCGTTTTGGTAAATTCATCAGAATTCACTTTGGAA
CCAGTGGKAAACTGTCCTCTGCGGACATTGAGACCTACCTGCTGGAGAAG
TCACGGGTCACTTTTCAGCTTAAGGCAGAGAGAAATTACCACATCTTCTA
CCAGATCTTGTCCAATCAAAAGCCAGAGCTGTTGGACATGCTGTTGATCA
CCAACAACCCTTATGACTACTCCTACATCTCCCAAGGAGAAGTAACCGTA
GCATCCATCAACGATTCAGAGGAGCTGATGGCCACTGACAGTGCCTTTGA
TGTGCTTGGCTTCACTCAAGAGGAGAAGATGGGAGTCTACAAGTTGACAG
GGGCCATTATGCACTATGGCAACATGAAGTTCAAGCAAAAGCAGCGTGAG
GAACAGGCTGAGCCTGATGGTACAGAAGCTGCTGATAAGTCAGCTTATCT
CATGGGGTTGAATTCAGCAGACCTCATCAAAGGACTCTGCCATCCCAGAG
TCAAAGTAGGAAATGAGTATGTCACCAAAGGCCAGGGTGTAGATCAGGTC
TACTAC----ACAAGGAGGCCTTCAAGTGTGAGGAGTGCGGCAAGCACTA
CAACACCAAGCTGGGGTACAAGCGCCACGTAGCCATGCACTCCGCCACGG
CGGGGGACCTCACCTGCAAAGTGTGCATGCAGAGTTACAAAAGCACGCCG
GCGCTCCTGGAGCACCTCAAGAGCCACTCGGGCAAGTCCTCGGGCGGCGC
CAAGGAGAAGAAGCACCCGTGCGACCACTGCGACCGTCGCTTCTACACGC
GGAAGGACGTCAGGCGACACATGGTCGTCCACACGGGCCGAAAGGACTTC
CTGTGCCAGTACTGTGCCCAGCGCTTCGGCAGGAAGGACCATCTGACGCG
GCACGTGAAAAAGAGCCACTCGCAGGAGCTGCTGAAGATCAAAACGGAGC
CTCCGGATATGTTGGGTCTCCTGGGCTCCAGCTCGCCGCCTTGCTCTGTC
AAGGAGGAGCTCAGCCCCATGATGTGCAGCATGGGTCCCAATAAAGACCC
CATGATGGGCAAACCCTTCCCCAGCGGACCCCCTTCCCCATGGGCATGT

ACAACCCCCACCAC------CTCCAGGCCATGTCTAATCCCGGGGTGGGC
CACCCC------CACCCCTCCCTGATGCCCGGTTCCCTGTCTGCAGCTAT
GGGCATGGGCTGTCACATGGAATACCTGATCTACGCCTCCTTCTCCTTCA
TGGGATGTTTACAAATCAGCGACGGATCCAACGTTGTCAACCTGTTAGCC
AGCAACTCCCCCAGTGTATCGTACGCTCTGACCCAGCAGAAGTACTTCAG
TAACTACAGCCCTGTAATCGGTTTCTACATCTACGAACCCATCGAGTACT
GGAACTCCACAGTGCAGGAGCACCTGAGAACTCTGGGCCAAGGCTTTAAC
AAGATCTCCTGGATGGACAACTACTTCCACTACCTGAAGGTAGTGAACGT
GACCGCCTCCACCAAGAGCGACTTCATCACCATCCTCAAAGGCTCCTTCC
TGAAGAGCCCGGAGTACCAGCACTTCACTGAGGACATCATCTTCTCAAAG
A---AAGGCG------------AGGAGTATGACATCATCGCATCCAGGAT
GTACCTGGTAGCGCGGACTACGGAGAAGACCCGGGAGGAGGTGGTGGAGC
TGCTGGAGAGGCTCCGCCCCCTCTCCCTCATCAACAGCATCAAGTTCATC
GTCTTCAACCCCACCTTCGTCTTCATGGACCGTTACAGCTCTTCAGTCGT
ATCACCCATTCTCACCTCGGCTTCAGTGTGCTCACCATCCTCATCCTCA
CCTTCTTCCTGGTCATCAACCCCCTGGGGAACTTTTGGTTGATCCTGACG
GTGACCTCTGTGGAGCTGGGTGTTCTGGGCCTGATGGGCTACCACACGTT
CGAGTGGCAGCCGGCCCTCAAGAACGTGTCCCCGTCCTGCCATGTGGGTA
TCATCAACGGGCTCTCCGGATGGGCTGCCTCGGTGGAGGACGCCCCCTCC
GACACCATCACTCGTCGGTTTCGCTACGACGTGGCCCTGGTGTCGGCCCT
GAAGGACCTAGAGGAAGACATCATGGAAGGACTGGCGGAGCGTGGGCTGG
AAGACAGCGCTTGCACCTCAGGCTTCAGAGTTATGATCAAGGAGTCCTGC
GATGGCATGGGAGATGTCAGCGAGAAGCACGGCGGAGGACCACCGACGCC
TGAGAAGGCTGTGCGTTTCTCTATCACCGTCATGTCCGTCTCTGTCCAGG
CCGACGGAGAGGAG------------------------------GAGCCG
GTCGTCATCTTCAGGGAGCCCAAGCCCAACTCTGAACTGTCCTGTAAGCC
CCTCTGCCTGATGTTTGTGGATGAGTCTGACCACGAGACACTCACCGCCG
TTCTGGGGCCTTTGGTAGCGGAGAGGTATGCGATGACGCAGAGTCGACTC
ATCCTTTCATTGGGTGGCCTCCCCCGCTCTTTCCGCTTCCACTTCAGAGG
CACGGGCTACGACGAGAAGATGGTGCGAGAGATGGAAGGTCTAGAGGCCT
CAGGCTCCACCTATATCTGCACTCTTTGTGACTCCACTCGGGCAGAAGCC
GCTCACAACATGGTGCTCCACTCTGTCACCCGCAGCCACGACGAGAACCT
GGAGCGCTACGAGATATGGAGGACCAACCCCTTCTCGGAGTCGGCCGTGG
AGCTGCGAGACCGGGTCAAAGGGGTCTCCGCCAAACCATTTATGGAGACC
CACCCGACTCTGGATGCATTACACTGTGACATAGGTAACGCCACTGAATT
CTACAAAATCTTCCAGGATGAGATCGGGGAGGTGTTCCAGGGGGC---CA
AC---CCGAGCCGGGAGGAACGGCGCAGCTGGAGGGCAGCCCTTGATAAG
CAGCTGAGGAAGAAAGTGAAGCTGAAACCTGTGATGCGGATGAATGGGAA
CTATGCCCGAAGGTTGATGACCCAGGAGGCTGTGGAGGCGGTGTGTGAAC
TGGTGCCCTCGGAAGAGAGGAGGGCAGCCCTGAGGGAGCTGATGGGGCTC
TATATCCAGATGAAGCCTGTGTGGCGTGCCACCTGCCCAGCCAAGGAGTG
CCCTGACCAGCTGTGCCGCTACAGCTTTAACTCACAGCGCTTCGCTGATC
TCCTCTCCTCCACGTTTAAATACAGGTATGATGGAAAGATCACCAACTAC
CTGCACAAGACCCTGGCCCACGTCCCTGAAATTATAGAAAGAGACGGTTC
CATCGGAGCCTGGGCCAGCGAGGGGAACGAGTCTGCGAACAAG-------


```
--------------------------------------------------
--------------------------------------------------
--------------------------------------------------
--------------------------------------------------
--------------------------------------------------
--------------------------------------------------
--------------------------------------------------
--------------------------------------------------
--------------------------------------------------
--------------------------------------------------
--------------------------------------------------
--------------------------------------------------
--------------------------------------------------
--------------------------------------------------
--------------------------------------------------
--------------------------------------------------
--------------------------------------------------
--------------------------------------------------
--------------------------------------------------
--------------------------------------------------
--------------------------------------------------
--------------------------------------------------
--------------------------------------------------
--------------------------------------------------
--------------------------------------------------
--------------------------------------------------
--------------------------------------------------
--------------------------------------------------
--------------------------------------------------
--------------------------------------------------
--------------------------------------------------
--------------------------------------------------
--------------------------------------------------
--------------------------------------------------
--------------------------------------------------
--------------------------------------------------
--------------------------------------------------
--------------------------------------------------
--------------------------------------------------
--------------------------------------------------
--------------ACAGGCGAAGTCACAGAGAGAGAAGTGGCTTTGGGG
ATCAATCCGTTCGCAGACGGGATGGGCGCCTTCAAAATAAACCACAGCTC
TCATGACATTGGCTCCGG---GCAAACGGCGTTCTCCTCCCAGGCG---C
CCGGCTAC---GCAGCGGCCGCCCTGGGA---CACCACCA------CCAC
CCGACCCACGTCAGCTCT---TATTCCACGGCAGCTTTCAACTCCACCCG
GGACTTTCTCTTCCGAAATCGGGGCTTCGGAGACGCCACCAG--------
----CGCGCAGCACAGTTTGTTCGCCTCCGC---CGCGGGAAGTTT---C
------GCAGGGCCACATGGACACTCTGATGCCGCGGGCCACCTGCTCTT
CCCCGGGCTCCACGAG---CAAGCCGCGAGCCACGCGTCGTCCAACGTTG
TAAACAGCCAGATGCGACTGGGCTTTTCTGGGGACATGTACGGGCGGGCC
GACCAGTATGGCCACGTTACAAGCCCAAGGT---CCGACCACTATGCTTC
GACCCAACTTCATGGCTATGGCCCCATGAACATGAATATGGCCGCT---C
ACCACGGAGCAGGGGCCTTCTTCCGCTACATGAGGCAGCCGATCAAACAA
```

GAGCTCATCTGCAAGTGGATTGAACCGGAGCAGCTGGCGAATCCCAAAAA
GGCGTGCAACAAAACTTTTAGCACGATGCACGAGCTCGTGACCCACCTGA
CGGTGGAGCATGTGGGGGGACCGGAGCAGTCGAACCACATTTGCTTCTGG
GAAGACTGTGCCCGAGAAGGGAAACCATTCAAAGCCAAATACAAACTTGT
AAATCATATCAGAGTTCACACCGGAGAAAAACCGTTCCCGTGTCCGTTCC
CCGGCT--------
>Glaucosoma hebraicum
AGCCTGCTAATTCGAGCAGAGCTCTGTCAGCCGGGCGCCCTCTTAGGGGA
CGACCAAATCTATAACGTCATCGTTACGGCACATGCCTTCGTAATAATTT
TCTTTATAGTAATACCAATCATGATTGGAGGCTTCGGAAATTGACTGGTG
CCTCTTATGATTGGTGCCCCCGACATGGCTTTCCCCCGAATAAACAACAT
AAGTTTCTGACTTCTTCCGCCATCCTTTCTTCTCCTTCTCGCCTCCTCGG
GCGTAGAGGCTGGTGCTGGAACCGGATGAACTGTTTATCCCCCTCTGGCA
GGAAACTTAGCTCATGCAGGGGCATCAGTCGACTTAACCATCTTTTCTCT
CCATCTGGCAGGTATCTCCTCAATTCTGGGTGCAATTAACTTTATCACAA
CAATTATTAATATGAAGCCCCCTGCCATCTCTCAGTATCAAACTCCTCTG
TTCGTATGGGCGGTACTGATTACTGCAGTACTTCTTCTTCTTTCTCTCCC
AGTCCTTGCCGCCGGCATTACAATGCTTCTCACGGACCGGAACCTAAACA
CTACCTTCTTCGACCCATCCGGAGGAGGGGACCCCATTCTTTACCAACAC
CTCTTT--

------------TTCCTGGAGAGAAACCTTCACCCATCTAACTGCCTTGG
CATGCTGTTGCTGTCTGACGCCCACCAGTGCACCAAGCTGTCGGAGCTCT
CCTGGGGCATGTGCCTCAGCAACTTTCCAGCCATTTGCAAGACAGAGGAC
TTCCTCCAACTGCCCAAAGATATGGTGGTGCAGCTTTTGTCACATGAGGA
GCTTGAGACAGAAGATGAGAGACTGGTTTATGAAGCCGCCCTTAACTGGA
TCAACTATGACCTGGAAAAAAGGCACTGCCACCTTCCAGAGCTCCTGAGA
ACGGTCCGCCTGGCCCTGCTGCCTGCCATCTTTCTCATGGAGAACGTTTC
TACAGAAGAGCTGATCAATGCCCAGGCCAAGAGCAAGGAGCTGGTGGATG
AAGCTATCCGCTGTAAGCTGAAGATCCTGCAGAATGATGGCGTTGTTAAC
AGTCCGTGTGCTCGACCAAGAAAAACCAGCCATGCCCTCTTTCTCCTGGG
AGGGCAGACTTTCATGTGTGACAAGTTGTACCTGGTGGACCAGAAAGCCA
AAGAGATCATCCCCAAAGCTGACATTCCCAGTCCCAGGAAGGAGTTCAGC
GCCTGCGCAATCGGCTGCAAGGTGTACATCACTGGCGGGA--GAGGCTC-
AGAGAACGGTGTGTCTAAAGATGTGTGGGTCTACGACACCGTCCACGAGG
AATGGTCGAAGGCGGCACCCATGCTCATTGCCAGGTTTGGCCACGGCTCC
GCAGAGCTGAAACACTGCCTCTACGTGGTAGGAGGTCACACTGCAGCAAC
TGGCTGCCTCCCGGCCTCTCCGTCT-------------------------

-----------GCAAAAGACAGCAGCAAGGGGACGCTGGAGGATCAAATC
ATCCAGGCGAACCCGGCGCTGGAGGCCTTCGGGAACGCCAAAACGCTGAG
AAACGACAACTCGTCTCGTTTTGGAAAATTCATCCGAATTCACTTCGGTA
CGAGCGGGAAGCTGTCGTCTGCTGACGTCGAGACGTACCTGCTGGAGAAG
TCACGTGTCACCTTTCAGCTGAAGGCTGAGAGGAACTACCACATCTTCTA
CCAGATCCTGTCCAATCAGAAGCCGGAGCTCCTGGACATGCTGCTGATCA
CCAACAACCCGTACGACTACTCCTACATCTCCCAGGGAGAGGTGACGGTC
GCCTCCATCAACGACTCGGAGGAGCTGATGGCCACCGACAGCGCCTTCGA
TGTGCTCGGCTTCACTCCAGACGAGAAGATGGGCGTCTACAAACTGACCG
GCGCCATCATGCACTACGGAAACATGAAGTTCAAACAGAAGCAGCGCGAG
GAGCAGGCGGAGCCTGACGGGACGGAGGCGGCTGATAAGTCTGCTTACCT
GATGGGTCTGAACTCTGCTGACCTGATTAAGGGGCTGTGCCATCCCAGAG
TGAAGGTCGGAAACGAATACGTCACCAAAGGCCAAAGCGTGGACCAAGTC
TACTACCCCAACAAGGAGGCCTTCAAGTGTGAGGAGTGTGGGAAGCACTA
CAACACCAAGCTGGGATATAAGCGCCACGTGGCCATGCACTCTGCCACAG
CAGGGGATCTCACCTGTAAAGTGTGCATGCAGACCTACGAGAGCACACCT
GTGCTCTTGGAGCACCTCAAGAGCCACTCTGGGAAGTCCTCGGGTGGCAC
CAAGGAGAAAAAACACCCGTGTGACCACTGTGACCGTCGTTTCTACACAC
GGAAGGATGTGAGACGACATATGGTGGTCCACACGGGCCGAAAGGACTTC
CTGTGCCAATACTGTGCCCAGCGCTTCGGCAGGAAGGACCATCTGACACG
TCATGTGAAGAAGAGCCACTCACAGGAGCTGCTGAAGATCAAGACAGAGC
CTCCCGATATGTTAGGTCTTTTAGCGTCTGGGTCACCACCCTGCTCTGTG
AAGGAGGAGCTCAGCCCCATGATGTGCGGCATGGGTCCCAACAAAGACCC
CATGATGGGCAAACCGTTCCCCGGTGGGGCTCCTTTCCCGATGAGCATGT
ATAACCCCCACCAT------CTCCAGGCCATGTCTAATTCTGGTGTGAGT
CACCCG------CACCCGTCCCTGATGCCCAGTTCCTTGTCTGCAGCTAT
GGGCATGGGTTGTCACATGGAATATCTCATCTACGCCTCTTTCTCATTCA
TGGGATGTTTACAAATCAGTGATGGATCAAATATTGTGAATCTGCTGGCT
AGTAACTCTCCGAGTGTTTCGTACGCTCTGACCCAGCAGAAATACTTCAG
TAACTACAGTCCGGTGATTGGGTTTTACATTTATGAGCCCATCGAGTACT
GGAACTCAACGGTGCAGGAGCACCTGAAGACTCTGAGTCATGGCTTCAAC
AAGATCTCTTGGATGGACAACTTTTTCCACTACCTGCGGGTGGTGAATGT
GAGTGCGTCGACCAAGAGCGACTTCATCACCATCCTCAAGGGCTCCTTCC
TGCGCAGCCCAGAGTACCAGCACTTCACAGAGGACATCATATTCTCCAAG
A---ACCGCGAGACTG------ATGAGTACGACATTATCGCCTCACGGAT
GTACTTAGTGGCACGAACCACAGAGAAGAAGCGTGAAGAGGTGGTGGAGC
TTCTGGAGAAGCTTCGTCCGTTGATGCTGATCAACAGCATCAAGTTCATT
GCCTTCAATCCTACATTTGTGTTCATGGACCGCTACAGCTCCTCTGTCAT
CTCGCCCATCCTGACCTCAGGCTTCAGCGTACTCACAATCCTCATCCTTA
CTTTTTTCTTGGTCATTAATCCCTTGGGGAACTTCTGGCTCATCCTCACT
GTAACATCCGTGGAGCTGGGCGTCTTGGGTTTGATGGGCTTTCACCAGTT
TGAATGGCAGCCAGCTCTCAGAAATGTGTCTACATCTTGCAATGTAGGCA
TTATTAATGGGATCTCTGGATGGGCTTCCTCTGTGGATGACTCCCCGGCT
GACACCATCACTCGGCGGTTTCGCTATGATGTGGCACTGGTGTCAGCAAT
AAAGGATCTGGAGGAGGACATCATAGAGGGGCTGAGAGAGAGTGGGATGG
AAGACAGTACGTGCACCTCAGGCTTCAGTGTCATGATCAAAGAATCTTGT
GATGGCATGGGCGATGTCAGCGAGAAGCACGGTGGAGGACCAGCTGTTCC
TGAGAAGGCCGTACGTTTCTCTTTCACTATTATGTCAGTCTCTGTCCTGG
CAGATGAGGAGGAG------------------------------GAAGAG
GTTACCATCTTCACCGAGCCAAAGCCAAACTCAGAACTGTCCTGTAAGCC
CCTTTGCCTAACGTTTGTGGATGAGTCAGACCACGAGACACTTACTGCCG
TCCTGGGGCCTATAGTTGCAGAGCGCAATGCAATGAAAGAGAGCAGACTC

ATCCTATCCGTGGGTGGACTACCTCGCTCCTTCCGCTTTCACTTCCGAGG
CACCGGATACGATGAGAAGATGGTGCGTGAGATGGAGGGCCTCGAGGCCT
CAGGGTCCTCCCATATCTGCACTCTTTGTGACTCCAGTCGGGCAGAGGCC
TCTGAAAACATGGTGCTACACTCCGTCACCCGCAGCCACGAAGAGAACCT
AGAACGTTACGAAATATGGAGAACCAACCCCTTTTCTGAGTCTGCAGATG
AGCTGCGAGACAGAGTCAAAGGGGTGTCTGCCAAGCCTTTCATGGAGACC
CATCCCACCCTAGATGCATTACACTGTGACATTGGCAATGCCACTGAGTT
CTACAAAATCTTCCAGGATGAGATTGGGGAGGTATACAAAAAGGT---CA
AC---CCCAGCCGGGAGGAACGACGCAGCTGGAGGGCAGCCCTAGATAAA
CAGCTGAGGAAGAAGATAAAGCTTAAACCGGTAATGAGGATGAATGGGAA
CTATGCCCGCAGGCTAATGACCCAGGAGACTGTGGAGGTGGTGTGTGAGC
TGGTGCCCTCAGAGGAGAGGAGGGAGGCCCTGAGGGAGCTTATGAGGCTC
TACCTCCAGATGAAGCCTGTATGGCGTGCCACCTGCCCAGCCAAGGAGTG
CCCCGACCAGCTGTGCCGCTACAGCTTTAACTCCCAGCACTTTGCCGACC
TCCTCTCCTCTGCCTTCAAATATAGGTACAACGGAAAGATAACCAATTAC
CTGCACAAGACCCTGGCCCATGTGCCTGAAATCATAGAGAGAGATGGATC
CATAGGAGCCTGGGCCAGCGAGGGGAATGAGTCGGCAAACAAATCGTACA
CCATCGACATGGGCCCCTTGGGTCCCCGGTGGAAGGAGAGCCCACAGCCG
TTCTCCTGCTCCATTGAAGACCCCACAAAACAGACAAAGTTCAAGGGCAT
CAAGACGTATATTTCGTACCGGGTCACGCCGAGCCACACAGGACGTCCTG
TCTACAGGCGCTACAAACACTTTGACTGGCTGTACAACCGCTTACTGCAC
AAGTTCACTGTGATCTCTGTGCCCCACCTGCCAGAGAAGCAGGCCACGGG
CCGATTTGAGGAAGACTTCATCGAGAAGCGCAAGAGGCGACTGATACTGT
GGATGAACCACATGACCAGTCACCCAGTCCTCTCCCAGTATGAAGGCTTT
GAGCACTTTCTGATGTGCGCTGATGACAAACAGTGGAAACTGGGCAAGAG
ACGGGCAGAGAAGGACGAGATGGTGGGGGCCCATTTCATGCTGACCCTCC
AGATCCCCAACGAGCACCAAGACCTTCAGGATGTGGAGGAACGAATCGAC
TCCTTCAAGGCCTTTGCTAAGAAAATGGATGACAGCGTGATGCAGCTCAC
ACATGTTGCCTCGGAGCTGGTGCGCAAGCACCTGGGAGGATTCAGGAAGG
AGTTCCAACGGCTGGGAAATGCCTTCCAGTCTATCAGCCAGGCGTTTATG
CTGGACCCTCCCCACTGCTCAGAAACCTTCAACAACGCCATCTCCCATNNNNNNNNNNNCG
TTCCTCAAACTGACCTCTCTGGGTTTCATCATCGGAGTCGGTGTGGTTGG
AAACCTCCTGATCTCCATCCTGCTGGTCAAAGACAAGAGCCTGCACCGAG
CGCCCTACTATTTCTTGCTGGACCTGTGCGCCTCTGATATCCTGCGCTCC
GCCATCTGCTTCCCCTTTGTCTTCACCTCTGTCAAGAATGGATCTGCCTG
GACCTATGGCACGCTGACCTGCAAAGTGATCGCCTTCCTGGGTGTGCTGT
CCTGTTTCCACACGGCGTTTATGCTGTTCTGTGTCAGTGTCACGCGCTAC
CTGGCCATCGCACATCACCGTTTCTACACCAAGAGGCTGACCTTCTGGAC
CTGTCTAGCTGTCATCTGCATGGTGTGGACGTTGTCAGTGGCTATGGCGT
TCCCACCGGTGCTAGACGTAGGGACGTACTCTTTTATCCGGGAGGAGGAC
CAGTGCACATTCCAGCACCGTTCCTTCAGGGCGAATGATTCTCTGGGCTT
CATGCTCCTGCTGGCGCTCATCCTCCTGGCCACACAGCTGGTTTACCTCA
AGCTCATCTTCTTCGTCCACGACCGTCGAAAGATGAAGCCTGTCCAGTTC
GTGCCTGCTGTCAGCCAGAACTGGACCTTCCACGGGCCAGGCGCCAGCGG
GCAGGCGGCGGCCAACTGGCTGGCTGGATTTGGTCGAGGCCCCACCCCGC
CTACTTTGCTGGGCATACGGCAGAACAGCAACGCAGCAGGCCGCCGGCGT
CTTCTGGTATTGGATGAATTCAAAACAGAGAAGAGGATTAGTAGGATGTT
CTACATCATGACGTTTTTCTTCCTGGCTCTGTGGGGGCCCTATCTGGTCG
CCTGCTACTGGCGGGTGTTTGCAAGGGGCCCTGTGGTCCCTGGGGGCTAC
CTGACGGCAGCCGTGTGGATGAGCTTTGCCCAGGCTGGGGTCAATCCGTT
CATCTGCATCTTCTCCAANNNNNNNNGCCAAATCTCGCTTTCACCCTGGCATGGGGAC
TGGTCCTGGCACGGAGC---GCAGCGTCCCACTCGGCAACAGCTTGCTAT
CCCCGCAGCAAACCGAGGAGCCCACTGTTGCCACCCCCCCGCAGCGATGG

TTTGTCACCC---CTGCCAACAACCGACTGGACTTTGCTGCCTCGGCATA
CGACGCCGCC---------GATTTCGCCGGTAACGCGGCCACCTTGCTGT
CCTACGCAGCGGCCGGAGTGAAGGCTC------TTCCCCTGCCGACTGCA
GGCTGCTCCAACCGGCCTCTTGGCTATTACGCAGACCCGTCAG---GCTG
G---GGAGGACGCACGCCGCCGCAGTACTGTGGCGTAAATAGCAAATCCA
GCTCGGTGTTTTCCTGCTGGCCCGCTAACTCTATCGGTGGCAGAGCGGGC
A---CC---AACTACCTGG---------CCGAGGA---GGGA---GACTC
---CATCCCGACGGAGAGGTCACCG---AT---CGGCGGCTCGGAGGAG-
--ACCAAACCCAAAGACCTGAC---ATCAGA---GTCGAGCTGGATAGAG
---ACGCCGTCCTCCATTAAGTCCATCGATTCGAGCGATTCTGGTATCTT
TG---AACAGGCCAAAAGGAGAAGAATCTCACCTTCTGCCACGCCG----

GTTTCAGAGACAGTGTCCCCGTTAAAATCGGAGNNNNNNNNNNNNNNNNNNNNNNNNNNNNNNNNNNNNN
NNNNNNNNNNNNNNNNNNNNNNNNNNNNNNNNNNNNNNNNNNNNNNNAACCACAGCTCC
CACGATATCGGCTCCGG---ACAAACGGCGTTTTCCTCTCAGGCG---CC
CGGCTAC---GCAGCGGCCGCCCTGGGA---CACCACCA------CCACC
CGACCCACGTTGGCTCT---TACTCCACGGCGGCTTTCAACTCCACCAGG
GACTTTCTCTTCAGAAATCGGGGTTTCGGGGACGCCACCGG---------
---GGCGCAGCACAGTTTGTTCGCCTC---------CGGAAGTTT---C-
-----GCAGGGCCACATGGACACTCAGATGCAGCGGGGCACCTGCTCTTC
CCGGGGCTCCACGAG---CAAGCGGCGAGCCATGCGTCTTCCAACGTGGT
CAACAGCCAGATGCGACTGGGCTTCTCGGGGGACATGTACGGACGGGCCG
ACCAGTACGGCCACGTTACGAGCCCGCGCT---CCGACCACTATGCTTCG
ACCCAGCTGCACGGCTACGGCCCCATGAACATGAATATGGCCGCA---CA
CCACGGAGCAGGGGCCTTCTTTCGATACATGAGGCAGCCGATCAAACAAG
AGCTCATCTGCAAGTGGATCGAGCCGGAGCAGCTGACGAACCCCAAAAAG
TCGTGCAACAAAACTTTTAGCACGATGCACGAGCTGGTGACCCATCTGAC
GGTGGAGCATGTGGGGGGACCGGAGCAGACCAACCACATCTGCTTCTGGG
AGGACTGCGCCAGAGAAGGGAAGCCATTCAAAGCCAAATACAAACTTGTA
AATCATATCAGAGTACACACCGGAGAAAAGCCCTTCCCGTGTCCGNNNNNNNNNNNNNNNNNN
>Gnathonemus petersii
AGCCTACTCATCCGAGCGGAACTAAACCAACCTGGAGCCCTACTTGGGGA
TGACCAGATTTATAATGTTATCGTCACAGCACACGCCTTCGTAATAATTT
TCTTCATGGTAATGCCCATTATGATCGGCGGCTTCGGCAACTGATTAATC
CCCCTCATGCTCGGCGCTCCTGACATAGCATTCCCTCGAATGAACAACAT
AAGCTTCTGACTCCTGCCCCCATCGTTCCTTCTTCTACTGGCCTCTTCTG
GCGTAGAAGCTGGGGTCGGTACGGGGTGAACCGTTTATCCACCGCTAGCC
GGTAACCTTGCCCATGCTGGAGCCTCCGTAGACCTAGCCATTTTCTCACT
TCACCTGGCCGGGGTCTCTTCCATCCTTGGTTCAATTAACTTTATTACCA
CAATTATCAACATAAAACCCCCAGCAATTTCCCAATACCAAACCCCATTG
TTTATTTGAGCCCTGCTAGTAACCACCGTGCTTCTACTGCTGTCATTACC
AGTCTTAGCTGCAGGAATTACGATATTACTGACAGATCGGAACCTAAACA
CAACATTCTTTGATCCGGCAGGCGGAGGAGACCCAATCCTCTACCAACAC
TTANNNNNNNNNNNNNNNNNNNNNNNNNNNNNNNNNNNNNNNNNNNNNNNNNNNNNNNNNNNNNNNNNNN
NNNNNNNNNNNNNNNNNNNNNNNNNNNNNNNNNNNNNNNNNNNNNNNNNNNNNNNNNNNNNNNNNNNNNN
NNNNNNNNNNNNNNNNNNNNNNNNNNNNNNNNNNNNNNNNNNNNNNNNNNNNNNNNNNNNNNNNNNNNNN
NN---

----------------CGAGGAGTACATTGTGGTCTTCAGCCGTACTGCT
AACAGGCTCATCCTGAATGAGGCGGAGCTGCTCCTGGCACTAGCTCAGGA
GTTTCAGATGAAGACCGTCACAGTGTCCCTCGAGGAGCATTCCCTCTCGG
AGGTGATCCGTGTCATTGCCGGTGCCTCCATGTTGGTCAGCATGCACGGA
GCACAGTTGGTCACCTCCCTTTTTCTCCCACGTGGTGCTGCTGTAGTAGA
GCTCTTCCCCTACGCTGTCAACCCAGAGCACTATACCCCTTACAAAACCT
TGGCCTCCTTACCAGGAATGGACCTCCAGTATGTAGCTTGGAGAAACACC
ATTGAGGAAAATTCCGTGACCCATCCAGAGCGACCTTGGGAACAGGGTGG
GATTGCCCACCTGGAACCGGAGGAGCAGGCGCGAATTCTCCAGAGCAAGG
AGGTACCACGCCATCTGTGTTGTCGCAACCCCGAGTGGCTCTTCCGGATC
TACCAGGACACCAAGGTGAATATTCCTTCTCTGCTGGAGGTCCTGAG---
GCAGATTCTGAAG---TCCAAGCCAAACTTGAAGAG---AACCAAGACAG
GCAGCGTTGTGCACCCAGGACGGGTTCGGGAGCCCAAGTGCCAGACTTCA
GTGCAGGCCACCAACGAGGCTAAGATGATGGTGTCGTGGCAAATTCCCTG
GAACCTGAAGTATCTTAAGGTCAAGGATGTCAAGTATGAAGTGTGGATTC
AG--

---CCA
AACAAGGAGGCCTTCAGGTGCGAGGAGTGCGGCAAGAACTACAACACCAA
GCTGGGCTACAAGCGTCACGTGGCTATGCACGCGGCGACCAGTGGAGATC
TCACCTGCAAGGTGTGCCTGCAGAGCTACGAGAGCACCCCCGTGCTGCTG
GAGCACCTCAAGAGCCACTCGGGCAAGTCGTCCGGTGGCACCAAGGAGAA
GAAGCACCCCTGTGACCACTGTGACCGGCGTTTCTACACACGCAAGGACG
TCCGCCGCCACATGGTGGTGCACACGGGCCGCAAGGACTTCCTGTGCCAA
TACTGCGCCCAGCGCTTCGGCCGGAAGGACCACTTGACGCGACATGTGAA
GAAGAGCCACTCGCAGGAGCTGCTGAAGATCAAGGCCGAGCCGCCTGACC
TGCTCAGCCTTCTGGGCTCTGGGTCGCCTCCATGCGCTGTCAAGGAAGAG
CTCAGTCCCATGGTGTG---CATGGGTTCCTCTAAGGACTCCATGCTGGG
CAAGCCCTTCCCCGGAG------CATTCCCCATGGGCATGTACAACCCCC
AC---------CTGCAGGCCATGTCCAACTCTGGGCCGCCT---------
---CACCACTCCCTGGTGTCGGGTCCTCTGTCCACCGCCATGGGGATGGG
CTGCCACATGGAGTATCTCATTTATGCATCATTTTCTTTCATGGGATGTT
TACAAATCAGCGATGGATCAAACATTGTTAACCTCCTAGTTAGCAACTCA
CCGAGTGTTTCCTATGCCCTGACCCAGCAAAAATACTTCAGCAACTACAG

```
CCCTGTGATTGGTTTTTACATATATGAGCCCATTGAATACTGGAATTCAA
CCGTTCAGGAACATTTGAAAACCCTCGGCCATGGATTCAATAAAATTTCC
TGGATTGACAATTTCTTTCACTATCTGAAGGTGGTGAACGTCAGTGCATC
CACAAAAAGCGAGTTCATAAATATTCTCCAGACCTCGTTCCTTAGGAGCC
CTGAGTACCAGCACTTCAAGGATGACATCATTTTCTCTAAAA---CAGAA
G------------ATGGCATCGAGATCATCGCCTCTAGGATGTACCTGGT
GGCACGAACCACAGAGAAGACACGAGAGGAGGTTGTGGAGCTTCTGGAGA
AGCTGAGGCCGCTCTCCCTTATCAACAGCATAAAATTCATCGTCTTTAAC
CCCACCTTTGTCTTCATGGATCGCTACAGTTCCTCCATCATTTCCCCAAT
ATTAACATCCGGGTTCAGCGTTTTGACTGTCTTGATATTGACGTTCTTCC
TTGTCATCAACCCCCTGGGAAACTTCTGGCTCATACTAACCATCACCTCC
GTAGAGCTGGGGGTTCTTGGCCTAATG-----------------------
--------------------------------------------------
--------------------------------------------------
--------------------------------------------------
--------------------------------------------------
--------------------------------------------------
------GTCAGCGAGAAGCATGGGGGTGGACCTTGCGTGCCTGAAAAGGC
TGTGCGATTCTCCTTCACAGTGATGTCAGTCTCCGTCCAGGTAGAGGGAC
GTCAG------------------------------GAAGCGATCACCATC
TTTCAGGAATTAAAGCCGAATTCGGAGCTGTCCTGCAAGCCGCTCTGTCT
CATGTTTGTAGACGAGTCGGACCACGAGACCCTCACTGCTGTACTGGGGC
CTGTGGTGGCCGAGCGAAATGCCATGAAGGAGAGTCGGCTCATCCTGTCC
ATCGGAGGGCTTCCTCGTTTCTTCCAGTTCCAGTTCCGGGGCACAGGTTA
CGACGAGAAGATGGTGAGAGAGATGGAGGGCCTTGAGGCTTCTGGGTCCA
CGTACATCTGCACCATGTGTGATTCCACTAGGATGGAGGCGTCAAAGAAC
ATGGTTCTGCACTCTATTACAAGGAGCCACGAAGAGAACCTAGAGCGCTA
TGAAATCTGGCGGAGCAACCCCTTCTCAGAGTCTGTGGAGGAGCTTCGGG
ACCGGGTCAAGGGCGTCTCAGCTAAGCCTTTCATGGAGACCCAACCGACC
CTGGATGCCCTGCACTGCGACATCGGCAATGCCACCGAATTCTATAAAAT
CTTCCAAGATGAAATCGGTGAGGTCTACCTGAAGGG---CAAC---CCTA
GTCGGGAGGAGAGGAGAAGCTGGAGGTCTGCTCTGGACAAACAGTTGAGA
AAGAAGCTGAAGGTGAAACCTGTCATGAGGAT------------------
--------------------------------------------------
--------------------------------------------------
--------------------------------------------------
--------------------------------------------------
--------------------------------------------------
--------------------------------------------------
--------------------------------------------------
--------------------------------------------------
--------------------------------------------------
--------------------------------------------------
--------------------------------------------------
--------------------------------------------------
--------------------------------------------------
--------------------------------------------------
--------------------------------------------------
--------------------------------------------------
--------------------------------------------------
--------------------------------------------------
--------------------------------------------------
--------------------------------------------------
```

```
--------------------------------------------------
--------------------------------------------------
--------------------------------------------------
--------------------------------------------------
--------------------------------------------------
--------------------------------------------------
--------------------------------------------------
--------------------------------------------------
--------------------------------------------------
--------------------------------------------------
--------------------------------------------------
--------------------------------------------------
--------------------------------------------------
--------------------------------------------------
--------------------------------------------------
--------------------------------------------------
--------------------------------------------------
--------------------------------------------------
--------------------------------------------------
--------------------------------------------------
--------------------------GCCAAATCCCGCTTCCACCCTGGC
ACCGGGACCGGTCCTGGCACGGACC---GCAGCGTCCCTTTAAGCAACAG
CTTGCTGTCCCCGCAACAAGCCGACGAGACCGCAGCGG---CCTCCCCGC
AGCGATGGTTTGTCACCC---CTGCCAACAACCGACTGGACTTTGCCACC
TCGGCATACGACGCTGCCGCCGCTGCTGATTTTGCCGGTAACGCGGCCAC
CCTGCTGTCTTACGCAGCTGCTGGAGTAAAGGCAC------TTCCACTCC
CCGCTGCAGGGTGCTCTGGTAGACCTCTGGGCTATTACGCCGACCCCTCA
G---GGTGG---GGCGCCCGTACGCCACCTCAGTACTGC---------AG
CAAATCAAGCTCGGTTCTTTCCTGCTGGCCACCAAATTCTGCCGGGAGCA
GGACTAGCG---CCTCAAACTACGTGG---------TGGATGA---AGGA
---GACAC---GCTGGCAACGGAAAGATCTCCG---CT---GGGTGCCTC
CGAAGAC---GCCAAGCCAAAAGACCT------GTCGGA---ATCCAGCT
GGACAGAG---ACGCCTTCATCAATAAAATCTATTGATTCGAGCGACTCT
GGGATATTCG---AACAGGCCAAGAGAAGGCGTATTTCACCGTCGGCCAC
GCCG---------GTGTCGGAAACGGCTNNNNNNNNNNNNNNNNNN----------------------
--------------------------AATCCGTTTGCCGAGGGGATGGGT
GCTTTCAAGATCAACCACAGTACCCACGATTTAGGGTCTGG---ACAGAC
GGCATTCACCTCGCAGGCT---TCGGGATAT---GCGGCTGCCGCTCTGG
GA---CATCACCA------CCACCCCGCCCATGTCAGCTCA---TACTCT
ACGGCGGCGTTCAACTCCACCCGGGACTTTCTGTTTCGAAATCGGGGCTT
CGGGGATGCGGCGAG------------CGCTCAGCACAGCCTCTTCGCCA
CCGC---TGCAGGCGGTTT---C------GCGGGGCCGCACGGACACTCG
GATGCCGCGGGACATCTCCTTTTCCCGGGACTTCACGAG---CAGGCGGC
CACTCACGCGACATCCAACGTCGTGAACAGCCAGATGCGCCTGGGCTTTT
CCGGGGAAATGTACGGCAGAGCAGAGCAGTATGGTCACGTAACGAGCCCC
AGGT---CCGACCACTATGCTTCGACCCAGCTGCACGGCTACGGCCCCAT
GAACATGAATATGGCTGCA---CATCACGGAGCTGGGGCCTTCTTTCGAT
ATATGAGACAACCGATAAAACAAGAGCTCATTTGCAAATGGATTGAGCCG
GAGCAGCTATCGAATCCAAAAAAGTCTTGCAACAAAACTTTTAGCACGAT
GCACGAGCTGGTCACCCACCTCACAGTGGAGCATGTTGGGGGGCCGGAGC
AGTCGAACCATATCTGCTTTTGGGAAGAATGCGCCCGTGAAGGAAAGCCC
TTTAAAGCCAAGTACAAACT------------------------------
-------------------------------------
```

>Gonorynchus greyi
AGCCTCTTAATCCGGGCCGAGCTAAGCCAACCAGGCTCCCTATTAGGCGA
TGACCAAATCTATAACGTCATTGTCACGGCACACGCCTTCGTTATGATCT
TCTTTATAGTGATACCCATTCTAATTGGGGGTTTCGGGAATTGACTAATT
CCCCTCATGATTGGGGCCCCAGACATGGCCTTCCCTCGAATGAATAACAT
GAGTTTCTGACTACTTCCCCCCTCTTTCCTTCTTCTTTTAGCCTCCTCGG
GGGTCGAAGCCGGGGCAGGGACCGGGTGAACTGTCTACCCACCCCTAGCC
GGCAACCTCGCTCACGCAGGGGCCTCGGTAGACCTAACTATTTTCTCCCT
CCACCTCGCGGGTGTATCCTCTATTCTAGGGGCTATTAACTTCATCACAA
CTATCATCAACATGAAACCTCCGGCTATTTCCCAATACCAAACTCCGCTA
TTCGTATGGGCGGTTCTAATTACGGCCGTGCTACTTCTTCTGTCCCTTCC
AGTACTAGCTGCCGGCATTACCATACTTCTTACGGATCGAAATCTGAACA
CTACCTTCTTCGACCCAGCGGGGGGAGGAGATCCCATCCTCTACCAACAC
TTGTTTTGATTCTTCGGGCACCCCGAAGTTTATATTCTAATTCTACCCGG
CTTTGGGATGATCTCCCACATCGTTGCTTACTACTCAGGTAAAAAAGAGC
CGTTCGGCTACATGGGCATGGTCTGGGCTATGATGGCAATTGGCCTTCTA
GGCTTCATTGTCTGAGCCCACCACATGTTTACAGTTGGAATGGACGTCGA
CACCCGGGNNNNNNNNNNNNNNNNAGAACTTTCATCCATCCAACTGCCTGGGAATGCTGCTGCTTT
CAGATGCCCACCAGTGCACGCAGCTCTCGCAGCTGTCCTGGAGCATGTGC
CTGAGCAACTTCCCTGCCATCTGCAAAACAGAGGAGTTCCTGCAGCTTCC
CAAGGATATGCTGGTCCAGCTCCTGTCGCACGAGGAGCTGGAGACGGAGG
ACGAGCGGCTGGTCTATGAGTCAGCTCTCAACTGGGTGAACTATGACCTG
GAGAGGCGGCACTGCCACCTTCCTGAACTGTTGCGCACAGTGCGCTTGGC
ACTTCTGCCCGCCATCTTCCTGATGGAGAACGTCTCCACCGAGGAGCTAA
TCAACGCACAGGCTAAGAGCAAAGAGCTAGTGGATGAAGCCATCCGCTGC
AAGCTGCGAATCTTGCAGAACGACGGCGTGGTCAACAGCCCCTGTGCCCG
TCCACGAAAGACCAGCCATGCTCTTTTCTTGTTGGGCGGGCCCACCTTCA
TGTGTGACAAGCTCTACCTGGTAGATCAGAAGGCCAAGGAGATCATCCCC
AAGGCAGACATCCCCAGCCCACGCAAGGAGTTCAGTGCCTGCGCCATTGG
CTGCAAGGTGTACGTGACAGGTGGTC--GTGGCTC-AGAGAACGGTGTGT
CTAAGGATGTGTGGGTCTATGACACCGTGCACGAGGAGTGGTCCAAAGCG
GCGCCCATGCTGATCGCTCGGTTTGGCCATGGCTCAGCTGAGCTGCGTCA
CTGTCTCTACGTGGTTGGGGGTCACACAGCCGCNNNNNNNNNNNNNNNNNNNNNNNNNNNCGATGACTA
CATAGTAG
TTTTCAGTCGTTCTGCTAATAGGCTCATCCTAAATGAGGCAGAGTTGCTT
CTAGCCCTGGCACAGGAATTCCAAATGAGGACAGTCACTGTGTCCCTTGA
GGAGCAGTCATTTGCTAGCATTGTCCAAGTCATCAGTGGAGCATCCATGC
TGGTCAGCATGCATGGAGCCCAACTGATCACCTCCATGTTCCTGCCACGA
GGTGCTGCTGTGGTAGAGCTCTACCCCTATGCAGTAGACCCGGAGCACTA
CGCTCCTTACAAGACACTAGCCTCCTTGCCAGGCATTGACATTCAGTATA
TTGCATGGAGAAACAACTTGGAGGAGAACTCTGTGGCCTACCCTGAGCGC
CCCTGGGACCAGGGTGGAATTTCACATCTGGAGAAGGAGGAGCAGGAGCG
CATAGTTAACAGCAGGGAGGTGCCCAGGCACCTGTGTTGCCGCAACCCAG
AATGGCTCTATAGGATCTACCAGGACACAATAGTGGACATCCCTTCCTTG
CTGGAGGTTCTCAG---GGAAGGTCTGAAG---ATCAAGCCTAATCTAAA
AAA---GACTAAGGCTGCCAGCACAGTTCATCCTGGACGAGTTCGAGAGC
CCAAGTGCCAGACTTCAGTGCAGGCCACCAATGAGGCTAAACTGTCAGTG
TCATGGCAGATCCCGTGGAACCTCAAGTACCTAAAAGTTAGAGAGGTCAA
ATATGAGGTATGGATACAGAAGAAGGATACCAGTAAGGGAACCCTGGAGG
ATCAAATTATCCAGGCTAACCCTGCCCTGGAGGCCTTCGGAAATGCTAAA
ACGGTGAGAAATGACAATTCCTCTCGCTTTGGAAAGTTCATACGTATTCA
TTTTGGCACAAGTGGTAAATTGGCYTCTGCTGACATAGAGACCTACCTGC
TTGAAAAATCTCGAGTAACTTTTCAGCTCAAAGCAGAGAGGAATTACCAC

ATCTTTTTTCAGATTCTGTCCAATGAAAAGCCAGAGTTGCTAGACATGAT
GTTGGTTACGAATAATCCTTATGATTACTCCTACATCTCCCAAGGAGAAG
TTACTGTTCAATCCATCAATGACAATGAAGAGCTATTAGCTACAGACAGT
GCTTTTGATGTACTTGGCTTTACCTTGGAAGAAAAAATGGCGGTCTACAA
GTTGACAGGTGCAATAATGCATTATGGCAACATGAAGTTTAAGCAGAAGC
AGCGGGAGGAGCAAGCAGAACCAGATGGTACAGAGTCTGCCGACAAGTCA
GCTTACCTAATGGGGCTAAACTCTGCAGATCTYGYAAAGGGACTCTGCCA
CCCGAGGGTCAAGGTCGGCAATGAATATGTCACAAAAGGGCAGAGTGTGG
ATCAAGTCTACTACNNTACAAAGAGGGCCTTTAAGTGTGAGGAGTGTGGCAA
GAACTACAACACCAAGCTGGGCTACAAGCGCCACGTGGCCATGCACTCGG
CCACGGCGGGTGACCTCACCTGCAAGGTGTGCCTGCAGAGCTACGAGAGC
ACGCCGGCGCTGCTGGAGCACCTGAAGAGCCACTCGGGCAAGTCCTCGGG
CGGCGCCAAGGAGAAGAAGCACCCGTGCGACCACTGCGACCGGCGCTTCT
ACACGCGCAAGGACGTGCGGCGCCACATGGTGGTGCACACCGGACGCAAG
GACTTCCTGTGCCAGTACTGCGCCCAGCGCTTCGGCCGCAAGGACCACCT
GACGCGGCACGTGAAGAAAAGCCACTCGCAGGAGCTGCTGAAGATCAAGA
CGGAGCCGCAGGACATGCTGGGCCTGCTGGGCTCGGGATCTCCACCCTGT
GCCATCAAGGAGGAGCTCAGCCCCATGATGTGCAGCATGGCAGCCGGCAA
GGACCCCATGATGGCCAAGCCCTTCCCTGCGGGCACCCCCTTCCCCATGG
GCATGTACAACACCCATCAC------ATCCAGGCCATGTCCAGCCCTGGA
GTGGGCCAC---------CACCACTCTCTGGTGCCGGGCTCCTTGTCTGC
TGCTATGGGAATGGGCTGNNNNNNNNNNTACCTGATCTATGCCTCCTTCTCATTCATGGG
ATGTTTACAAATCAATGATGGCCTGAACATTGTGAACCTGTTGGCCAGTA
ATTCACCCAGTGTGTCGTTCGCTCTGACGCAGCAGAAGTACTTCAGCAAC
TACAGTCCAGTGATTGGCTTTTATATCTATGAGCCCATAGAGTACTGGAA
CTCCACTGTGCAGGAGCACCTCAAGACTCTGGGCCAGGGCTTCAACAAGA
TCTCCTGGATCGATAACTACTTTCACTACCTTAAGGTGGTGAACGTCAGC
GCGTCAACCAAAAGCGATTTCATCAACATCCTCAAGACGTCTTTCCTSAA
AAGCCCAGAGTATCAGCACTTCACCGACGACATCATCTTCTCCAAAA---
CCGGGG------------ACGAGTATGACATCATCGCGTCGCGGATGTAC
CTGGTGGCCAGGACGACGGAGAGGACGCGGGAGGAGGTGGTGGAGCTGCT
GGAGAGGCTCCGGCCGCTGTCCCTCATCAACAGCATCAAGTTCATCGTCT
TCAACCCCACCTTCGTCTTCATGGACCGCTACAGCTCCTCCGTCATCTCC
CCCATCCTGACATCGGGCTTCAGCGTCCTGACCATCTTAATCCTCACCTT
CTTCCTGGTCATCAACCCGCTGGGGAACTTCTGGTTGATCCTGACGGTCA
CCTCCGTGGAGCTGGGCGTCCTGGGGCTGATGNNNNNNNNNNNNNNNNNNNNNNNNNNNNNNNNNNNN
NNNNNNNNNNNNNNNNNNNNNNNNNNNNNNNNNNNNNNNNNNNNNNNNNNNNNNNNNNNNNNTGATGATGTG
CCAGCGGA
CACCATTGCCCGTCGTTTCCGCTATGACGTAGCTCTGGTGTCTGCGCTGA
AAGACATGGAGGAGGACATCATGGAGGGTCTGAGGGAGAGAGGGCTGGAT
GACAGCACCTGCACCTCTGGTTTCATAGTGATGATCAAAGAGTCCTGCGA
CGGTATGGGGGATGTCAGCGAGAAACATGGAGGTGGACCAGCTGTTCCAG
AGAAAGCAGTGAGGTTCTCGTTCACGGTGATGTCCATCTCTGTCCATGCA
GAAGGTAAGGAG-------------------------------GAAGCCGT
CACCATCTTCCAGGAGCGAAAGCCCAACTCCGAGCTGTCCTGCAGGCCTC
TATGTCTGATGTTTGTTGATGAGTCAGACCATGAGACGCTCACGGCCATC
CTGGGCCCTGTGGTGGCTGAACGGAGAGCCATGAAAGAGAGCCGGCTGAT
CTTGTCTGTAGGGGGCTTGGAACGTTCCTTCAGGTTTCAGTTCCGGGGCT
CAGGCTATGACGAAAAGATGGTGCGTGAGATGGAGGGCCTGGAGGCCTCA
GGGTCCACCTACGTCTGCACCTTGTGCGACTCCACCCGAGCTGAGGCTTC
CAAGAACATGGTGCTGCACTCCATCACCCGAAACCATGACGAGAACCTTG
AGCGCTACGAAGTGTGGAGGACCAACCCTTACTCTGAGTCTATTGAGGAG
CTCAGAGACAGGGTCAAGGGTGTCTCAGCCAAGCCCTTCATGGAGACCCA

GGCCACAATGGACGCTCTGCACTGTGACATCGGCAACGCCACGGAGTTTT
ACAAGATCTTCCAAGATGAGATCGGTGAAGTCTATCTGAGGAG---CAAC
---CCGAGCAGGGAAGAGCGACGTCGCTGGCGCTCTGCCCTGGACAAGCA
GCTGAGGCAGAAGATGAAGCTCAAGCCAGTGATGCGGATGAATGGAAATT
ACGCCAGGCGACTGATGACCCGCGAGGCAGTAGATGTAGTCTGTGAATTG
GTGCCATCAGAGGAACGGCAGCAGGCCCTCCGGGAGCTGGTGGAGCTCTA
CCTCCAGATGAAGCCAGTGTGGCGCGCCTCCTGCCCTGCCCAGGAGTGCC
CAGACCAGCTGTGCCGTTACAGCTTCAACTCGCAGCGCTTCGCTGACCTG
CTCTCCACTACATTCAAGTACCGCTATGACGGCAAGATCACCAACTACCT
GCACAAGACCTTGGCCCACGTGCCTGAGATTGTGAGNNNNNNNNNNNNNNNNNNNNNNNNNNNNNNN---
----
----------TCCTACTCCATAGAGATGAGCCCCCGAGGTCCGCAGTGGA
AAGAGAGCCCTCAGCCTTTCACCTGCTCTATCGAGGATCCCACGAAACAG
ACTAAGTTCAAAGGCATCAAAACATACATCTCCTACCGCGTGACTGCTAG
CCACACTGGACGGCCCGTGTACCGCCGCTATAAGCACTTTGACTGGCTCT
ACAACCGGCTACTGCACAAGTTCACGGTCATCTCGGTGCCCCACCTGCCA
GAGAAGCAGGCCACGGGCCGCTTTGAGGAAGACTTCATCGAGAAGCGCAA
GAGGAGGCTCATTCTCTGGATGGACCACATGACCAGCCACCCGGTCCTGT
CCCAATACGAGGGCTTCGAACACTTCCTCATGTGCGGAGAYGACAAACAG
TGGAAGCTGGGCAAGCGGCGGGCGGAGAAGGACGAGATGGTGGGCGCCCA
CTTCATGCTGACCCTCCAGATCCCCAACGAGCACCAGGACCTGCAGGACG
TGGAGGAGCGCGTGGACACTTTCAAGGCCTTCACCAAGAAGATGGACGAC
AGCGTCATGCAGCTGACGCACGTCACCTCTGAACTGGTGCGCAAGCATCT
TGGAGGCTTCCGGAAGGAGTTCCAGAGGCTGGGCAATGCCTTCCAGTCCA
TCAGCCAGGCCTTCATGTTGGACCCCCCCTATAGCTCAGATGCCCTCAAC
AACGCCATCTCCCAC-----------------------------------

--GCCAAATCCCGCTTTCACCCTGGCGTCGGGAGCGCTCCTGGCACGGAC
C---GCAGCGTCCCACTCAGTAACAGCTTGCTATCCCCGCAACAAACCGA
GGAGACCGCAGTGG---CCTCCCCGCAGCGATGGTTTGTCACCC---CTG
CCAACAACCGACTGGACTTTGCTGCCTCGGCATACGATGCCGCCGCCGCT
GCTGATTTCGCCGGTAACGCAGCCACCCTTCTGTCATACGCAGCTGCTGG
AGTGAAGGCGC------TTCCATTGCCTACCGCAGGCTGCTCCAACAGAG
CTTTGGGCTACTATGCTGAACCACCCG---GGTGG---GGCACTCGCACT
CCACCTCAATACTGC---------AGCAAATCCGGCACGGTCCTCTCATG

CTGGCCCTCCAACTCAGTCAGTAGCAGAACGTCCA---CTTCTAATTACC
TAT---CAGGCCTGGAGGA---AGGG---GATCC---TATTGCACCTGAG
AGATCACCT---CT---CGGCGGCGCAGATGAT---GCCAAACCGAAGGA
CCT------GTCTGA---GTCCAGCTGGATAGAG---ACGCCTTCCTCTA
TTAAGTCGATTGATTCAAGTGATTCTGGGATTTTG---AGCAAGCCAAA
CGGAGGAGGATTTCACCATCTGCCACGCCG---------GTTTCAGAGAC
GTCGTCCCCATTAAAATCAGAA---------
NNNNNNNNNNNNNNNNNNNNNNNNNNNNNNNNNNNNNNNNNNNNNNNNNNNNNGGCATGGGCGCCTTCA
AAA
TAAACCACAGCTCTCACGACATAGGCTCTGG---ACAAACAGCATTTTCC
TCGCAAGCT---CCCGGCTAT---GCTGCCGCTGCGCTGGGA---CGCCA
TCA------CCACCCGACTCATGTCAGCTCA---TACTCCACAGCGGCTT
TCAATTCTACCCGGGACTTTCTCTTTCGGAATCGGGGCTTTGGTGACGCT
ACCAG------------TGCTCAGCACAGTCTCTTCGCCTCAGC---CGC
GGGAAGTTT---C------GCAGGGCCACATGGACACTCTGATGCCGCAG
GACACCTGCTCTTCCCGGGACTGCACGAG---CAAGCGGCAACGCACGCG
TCTTCGAACGTAGTTAACAGCCAAATGCGGCTTGGCTTTTCGGGTGACAT
GTACGGAAGAGCTGACCAATACGGCCATGTTACGAGCCCCAGGT---CCG
AGCATTATGCCTCCACACAGTTGCATGGCTATGGCCCCATGAACATGAAT
ATGGCTGCC---CATCACGGTGCAGGGGCCTTCTTTCGTTACATGAGGCA
GCCGATTAAACAAGAACTCATCTGCAAGTGGATCGAACCTGAGCAGCTGA
CAAATCCGAAAAAGTCTTGCAACAAAACATTCAGCACGATGCACGAGCTG
GTGACCCACCTCACAGTGGAACATGTCGGGGGACCAGAGCAGTCGAATCA
CGTTTGTTTTTGGGAAGAGTGTTCAAGAGAAGGAAAACCGTTCAAAGCAA
AGTNNNNNNNNNNNNNNNNNNNNNNNNNNNNNNNNNNNNNNNNNNNNNNNNNNNNNNNNNNNNNN---
----
>Gymnorhamphichthys petiti
AGTCTATTAATCCGGGCAGAGCTTAGCCAACCCGGAGCCTTAATGGGTGA
CGACCAAATCTACAATGTTATTGTTACAGCCCACGCCTTCGTTATAATCT
TCTTCATGGTTATACCCATTATGATCGGAGGGTTTGGAAACTGGCTTATC
CCCCTAATAATCGGCGCTCCTGATATAGCCTTCCCTCGAATAAATAACAT
AAGCTTTTGACTCCTGCCCCCTTCATTTCTCCTTCTCCTCGCCTCCTCTG
GGGTCGAAGCCGGTGCAGGCACAGGTTGAACTGTTTACCCGCCTCTTGCA
GGCAATCTTGCCCACGCAGGTGCCTCAGTTGACCTCACTATTTTTTCCCT
CCATTTAGCAGGAGTTTCTTCCATTCTTGGCTCCATCAACTTCATTACAA
CAATTATTAATATAAAACCCCCAGCAATAACTCAATATCAAACGCCTTTA
TTTATCTGAGCCACTCTCGTCACCACCGTTCTTCTTCTCTTGTCTCTTCC
GGTTCTAGCAGCAGGGATTACAATACTACTAACAGACCGCAACCTTAATA
CAACCTTCTTTGACCCTGCAGGAGGAGGA---------------------

-------------------------TGACGATTATATAGTAGTTTTCAGC
CGTGCTTCAAACAGGCTCATACTCAACGAGGCAGAGCTGATTCTTGCTTT
AGCCCAAGAGTTCAAGATGAGGACAGTCACAGTGTCCTTGGAAGATCAGC
CATTTGACAGCATAGTTCGGGTCATCAGTGGGGCATCCATGTTGGTCAGC
ATGCATGGAGCACAGCTGATCACCTCCCTGTTCTTACCACGCGGGGCTGC
TGTGATAGAGCTCTTTCCATATGCAGTAAACCCAGAGCATTATTCTCCAT
ACAAAACCCTGGCCTCCTTGCCAGGTATGGACCTCCAGTACATATCGTGG
AGAAATACTATTGAGGAGAATTCCGTGGCCTACCCTGACCGCCCTTGGGA
CCAGGGAGGCATCTCCCACTTAGAGAAAGAGGAGCAGGAACGCATCCTAG
CTAGCACAGAGGTGCCAAGACACCTGTGTTGCCGCAACCCCGAGTGGCTT
TATCGGATTTATCAAGACACCGTTGTAGACATCCCGTCCTTTCTCGAGGT
CCTTAG---AGAAAGTCTAAAA---GTTAAGCCTAATCTGAAAAA---GA
CCAGACCTCTCAGCACAGTACATCCAGGTCGGGTCCGAGAACCCAAGTGT
CAGACATCAGTGCAGGCCACAAATGAAGCAAAGCTCGCCGTGTCATGGCA
GATCCCTTGGAATCTCAAGTACCTGAAGGTGAGGGAGGTCAANNNNNNNNNNNNNNNNNNN--------
----

-------------------------------------CCCTACAAGGAGG
CCTTCAAGTGTGAGGAGTGCGGCAAGCACTATAACACGAAACTGGGTTAC
AAGCGTCATGTGGCCATGCACTCGGCCACGGCGGGCGACCTGACCTGCAA
GGTGTGTCTGCAGAGTTACGAGAGCACCCCAGCCCTGCTGGAACACCTCA
AGAGTCACTCGGGCAAGTCTTCGGGCGGCGCCAAGGAGAAGAAACACCCG
TGCGACCACTGCGACCGCCGTTTCTACACCCGCAAGGACGTGCGCCGTCA
CATGGTGGTGCACACGGGCCGTAAGGACTTCCTGTGCCAGTACTGCGCCC
AGCGCTTCGGGAGGAAGGACCATCTCACGCGACATGTCAAGAAGAGCCAC
TCACAGGAGCTGCTGAAGATCAAGACGGAGCCACCCGACATGCTGGGCCT
CCTGGGCTCGGGCTCGCCACCGTGTGCTGTGAAGGAAGAAATAAGCCCCA
TGATGTGCAACATGGGCTCCAACAAGGACTCCATGATGGCCAAGTCGTTC
CGCAGCGGCCCACCCTTCCCTATGGGGATGTACAACCCCCACCCC-----
-CTGCCAGCCATGTCCAGTCCCGGAATGGGCCAC---------
CATCNNNNNNNNNNNNNNNNNNNNNNNNNNNNNNNNNNNNNNNNNNNNNNNNNNNNNNNNNNNNNNNNNN
NNNNNNNNNNNNNNNNNNNNNNNNNNNNNNNNNNNNNNNNNNNNNNNNNNNNNNNNNATC
TGTTAGCCAGCAATTCACCCAGTGTGTCATTTGCTCTGACTCAGCAGAAA
TTGTTCAGCAACTACAGCCCGGTTATAGGCTTCTACATCTATGAGCCCAT
TGAATACTGGAACTCTACAGTGCAGGAGCACCTTAAGACTCTAGGTCAGG
GTTTCAACAAGATTTCATGGATTGATAATTACTTTAATTATCTGAAGGTA
ACAAACGTCAGTGCGTCTACCAAAAGTGATTTCATAAACATTCTCAAGAC

GTCTTTTCTGAAGAGCCCGGAGTATCAGCACTTCTCTGAGGACATCATCT
TCTCAAAAA---ATAGGG------------ATGAGTATGACATCATTGCA
TCCAGGATGTACTTGGTTGCCCGGACAACTGAGAAAACAAGGGAGGAGGT
GGTTGAACTTCTGGAGAGACTCAGACCGCTCTCACTCATAAACAGCATCA
AGTTTATCGTCTTCAATCCCACCTTCGTATTCATGGATCGCTACAGTTCC
TCGGTCGTCTCCCCAATCCTCACCTCTGGCTTCAGTGTCCTGACCATTCT
CATCCTCACCTTCTTCCTCGTCATCAACCCATTGGNNNNNNNNNNNNNNNNNNNNNNNNNNNNNNNNNNN
NNNNNNNNNNNNNNNNNNNNNNNNGGTTTTCCACAGTTT
GAGTGGCAGCCTGCTCTCAAAAATGTGTCCAGCTCTTGGGATGTGGGCAT
CCTTAATGGGCTTTCTGGCTGGACAGCCTATGTAGATGACGTACCAGCAG
ACACAATTTCTAGAAGGTTCCGCTATGATGTAGCACTTGTATCCGCCCTG
AAGGACTTGGAGGAGGACATCATGGAAGGACTGAAAGAAAGAGGTTTGGA
AGACAGCGCCTGCACATCAGGATTCACGGTGCTCATTAAGGAGTCATGTG
ACGGCATGGGGGACGTCAGTGAGAAGCATGGGGGGGGCCCAGCCGTGCCG
GAGAAGGCAGTGCGATTCTCTTTCACAGTGATGTCTATCTCCATTCAAGT
AGAAGGTGCTGAA------------------------------GAAGCAG
TCACCATCTTCCGCGAGCCAAAGCCCAATTCCGAGCTGTCCTGCAGACCC
CTGTGCCTCATGTTTGTGGATGAGTCAGATCACGAGACGCTGACCGGCAT
CTTGGGGCCTGTGATTGCAGAGCGAAAAGCTATGGCAGACAGTCGCCTCA
TTCTGTCTGTAGGTGGTCTCCTTCGTTCATTTCGGTTTTACTTCTGGGGC
ACAGGCTACGATGAGAAGATGGTGAGAGACATGGAAGGTTTGGAGGCCTC
GGGCTCCACCTACATCTGCACCCTGTGTGACTCTACACGAGCAGAAGCTT
CTCAGAACATGGTGTTGCATTCCATAACTCGCAGCCATGATGAAAACCTC
GACCGTTACGAGATGTGGAGGACCAACCCTTTCTCTGAGTCTGCAGAGCA
GCTGCGTGATCGGGTCAAAGGGGTCTCTGCCAAGCCCTTCATGGAGACCC
ATCCCACATTAGATGCCCTGCACTGTGACATTGGCAATGCTACTGAGTTC
TATAAGATCTTCCAAGATGAGATCGGGGAGGTGTACCTGAAACA---AAA
C---CCATCCCGAGAGGAGCGTCGTTGCTGGCGTTCAGCTCTTGACAAGC
AGCTGAGGAACAAGCTCAAACTGAAGCCGGTGATGAGGATGAACGGGAAC
TATGCACGGCGGCTCATGACCCGCGACTCCGTGGAGGTGGTGTGTGAGCT
GGTTCCTTCAGAGGAGCGTCGCGAGGCTCTGAAGGAGCTGATGGAGCTGT
ATCTCCAGATGAAGCCCGTGTGGCGGTCCAGCTGCCCTGCTCAGGAGTGC
CCCGACCAGCTATGCCGCTACAGCTACAACTCCCATCGCTTCGCCGACCT
GCTCTCCACCACATTCAAGTATCGCTACGACGGTAAGATCTCCAACTACC
TCCACAAGACACTGGCCCACGTGCCTGAGATTGTGGAGCGGGACGGCTCG
ATCGGCGCCTGGCCAGCGNNNNNNNNNNNNNNNNNNNNNNNNGCATACACCATAGAGATGGGCCCCTGGG
GGC
CTCAGTGGAAGGAGAGTCCCCAGCCGTTCTCCTGCTCCATTGAAGATCCC
ACCAAACAGACTAAGTTCAAAGGCATTAAGACTTACATTTCTTACCGAGT
CACTCCCAGTCACACCGGCAGACCCGTGTACCGCCGCTACAAGCACTTCG
ACTGGCTATACAATCGCCTGCTGCACAAGTTCACTGTCATCTCCGTCCCA
CACCTGCCCGAGAAGCAGGCCACCGGGCGCTTCGAGGAGGACTTCATCGA
GAAGAGGAAGAGAAGGCTCATCCTATGGATGGATCACATGACCAGCCACC
CTGTCCTGTCCCAGTATGAGGGTTTCGAGCACTTCCTCATGTGCGCCGAT
GACAAGCAGTGGAAGCTGGGGAAGCGGCGGGCAGAGAAGGACGAACTGGT
GGGCGCTCACTTCATGCTCACCCTGCACATCCCCAACGAGCACCAGGACC
TGCAGGATGTGGAGGAGCGCGTCGACACCTTCAAGGCCTTTGCCAAGAAA
ATGGACGACAGTGTGATGCAGCTGACGCATGTGGCCTCCGAACTCGTCCG
CAAACATCTCGGGGGCTTCCGCAAGGAGTTCCAACGACTGGGTCTCGCTT
TCCAGTCCATCAGCCAGGCATTCATGCTTGACCCTCCACATGCCACGGAC
ACCCTCAATGCCGCCATCTCGCAC--------------------------

--NNNNNNNNNGTCACAGAGA
GAGAAGTGGCGTTGGGGATAAATCCGTTTGCCGACGGTATGGGCGCTTTT
AAAATCAACCACAGTTCCCACGATCTTGGCTCCGG---GCAAACGGCGTT
TTCCTCGCAGGCA---CCCGGCTAC---GCGGCCGCAGCCCTGGGA---C
ACCATCA------TCATCCCACTCATGTCAGCTCC---TACTCCACCGCA
GCTTTCAACTCAACTCGGGACTTTCTTTTTCGGAATCGAGGCTTCGGAGA
CGCCACCAG------------CGCGCAACACAGTCTTTTCGCCTCTGC--
-AGCTGGAAGTTT---C------GCGGGGCCACATGGACACTCCGATGCT
GCTGGACACCTGCTTTTCCCGGGACTGCACGAG---CAAGCGGCGACGCA
CGCGTCCTCGAACGTGGTGAACAGTCAGATGCGTTTAGGCTTTTCCGGGG
ACATGTACGGTAGGGCCGATCAGTACGGCCATGTTACAAGCCCCAGGT--
-CTGAGCACTACGCCTCGACACAGTTACACGGCTATGGCCCCATGAACAT
GAATATGGCTGCC---CATCACGGGGCAGGGGCCTTTTTTCGATACATGA
GACAACCGATCAAACAAGAGCTCATCTGCAAGTGGATTGAACCGGAGCAA
CTAACGAACCCGAAAAAGTCATGCAACAAAACTTTCAGCACCATGCATGA
GCTTGTGACTCATCTCACCGTGGAACACGTTGGGGGACCAGAGCAGTCGA
ATCATATTTGCTTCTGGGAGGACTGGCCCNNNNNNNNNNNNNNNNNNNNNNNNNNNNNNNNNNNNNNNNN
NNNNNNNNNNNNNNNNNNNNNNNNNNNNNNNNNNNNNNNNNNNNNNNNNNNNNNNNNNNNN
>Halichoeres bivittatus
AGCCTGCTTATTCGGGCTGAACTAAGCCAACCCGGCGCTCTCCTTGGGGA
CGATCAAATCTATAATGTAATCGTTACAGCGCATGCTTTCGTAATAATTT
TCTTTATAGTAATACCAATTATGATTGGCGGATTCGGAAACTGACTGATT

CCCCTAATGATTGGAGCCCCCGACATGGCCTTCCCTCGAATGAACAACAT
AAGCTTCTGACTCCTTCCTCCCTCCTTCCTTCTCCTGCTCGCCTCTTCTG
GGGTAGAAGCCGGTGCTGGAACTGGTTGAACGGTTTATCCCCCTCTAGCA
GGAAATCTTGCTCACGCCGGTGCGTCTGTAGACCTAACAATCTTCTCCCT
TCACTTAGCCGGTATCTCATCAATTCTAGGGGCCATCAACTTTATTACAA
CTATCATTAACATGAAGCCTCCTGCTATCTCCCAATACCAAACCCCACTA
TTTGTATGAGCTGTGCTGATTNCAGCCGTCTTGCTCCTGCTTTCTCTCCCC
GTCCTCGCTGCCGGAATTACAATGNTTNTGACAGATCGAAATCTAAACACCA
CTTTCTTTGACCCCGCAGGAGGAGGTGATCCTATTCTCTATCAACATCTG
TTC---

---------NNNNTTGAGAAGAACCTTCATCCATCAAACTGCCTTGGCATGCTG
TTGCTGTCCGATGCTCATCAGTGCACCAAGCTCTCAGAGCTTTCCTGGGG
CATGTGCCTCAGCAACTTCCCYGCTATTTGCAAGACAGAGGACTTCCTCC
AACTGCCCAAAGATATGGTGGTACAGCTTTTGTCACATGAGGAGCTAGAG
ACAGAAGATGAGAGACTGGTRTATGAAGCTGCCCTGAACTGGATCAACTA
TGATTTGGAAAAGAGGCACAGCCACCTCCCAGAACTCCTGAGGACTGTTC
GTCTGGCTCTGCTTCCAGCCATCTTTCTGATGGAGAACGTATCCACWGAA
GAGCTTATCAACGCCCAGGCCAAGAGCAAGGAGCTGGTGGATGAAGCTAT
CAGGTGTAAGCTGAAGATCCTRCAAAATGATGGYGTTGTAAACAGCCCAT
GTGCCAGGCCAAGAAAAACCAGCCATGCCCTGTTTCTTCTGGGTGGGCAG
ACCTTCATGTGCGACAAACTGTACTTGGTGGATCAGAAAGCCAAAGAGAT
TATCCCTAAAGCTGACATCCCCAGCCCKAGGAAAGAGTTTAGCGCCTGTG
CCATTGGCTGTAAAGTGTACATTACTGGTGGCA--GAGGCTC-TGAGAAT
GGTGTGTCCAAAGATGTGTGGGTCTACGACACAGTCCATGAGGAATGGTC
CAAGGCRGCACCCATGCTGATTGCAAGGTTTGGTCATGGTTCTGCTGAGC
TGAAACACTGCCTGTATGTGGTAGGAGGTCATACTGCTGCAACTGGCTGC
CTTCCAGCCTCTCCATCAGGACGAGTACATTGTTGTGTTCAGTCGATCAA
CAACGAGACTGATCCTGAATGAAGCGGAGCTGATCATGGTGCTGGCCCAG
GAGTTCCAGATGAGGGTGGTCACCGTGTCTCTGGAGGAGCAGTCCCTCCC
CAGCATCGTGCAGATGATCAGCGGGGCGTCCATGTTAGTCAGCATGCATG
GAGCCCAGCTCATTACCTCACTCTTCCTCCCCAGGAGAGCTACCGTGGTG
GAGCTCTTCCCCTTTGCTGTGAACCCGGAGCAGTATACCCCCTACAAGAC
CCTGACCTCTCTGCCAGGCATGGACCTTCACTACATCGCCTGGAGGAACA
CGATGGAGGAAAACACCATCACCCACCCAGACAGAGGCTGGGAACAAGGG
GGCATCACTCACCTGGAGAGGGACGAGCAAGACCGGATCTTGGCCAGCAA
AGACGTCCCGAGACACTTGTGCTGCCGGAACCCCGAGTGGCTCTTCCGGA
TCTACCAGGACACCCTGGTGGACATCCCTTCTTTCTTGGAAGTCCTCAA-
--AGAGGGAATGAAG---ACAAAGCCCAGCATGAAGAA---GTCTAAGCC
AGCAAGCATGGTCCACCCGGGCCGGGTGCGGGAGCCGCAGTGTCAGACCT
CCGTACAAACCACCAATGAGGCTAAACTGACAGTCTCTTGGCAGATCCCC
TGGAATCTGAAGTACCTGAAGGTGAGGGAGGTGAAGTATGAAGTCTGGAT
CNNNAAAAAGGACACCAGCAAGGGGACCCTGGAGGATCAAATCATCCAGGCTA
ACCCTGCGCTGGAGGCCTTTGGAAACGCCAAAACCTTGAGAAATGACAAC
TCATCACGTTTTGGAAAATTCATCCGAATTCACTTCGGTACGAGCGGCAA
GCTGTCGTCTGCCGACATCGAGACGTACCTACTGGAGAAGTCTCGAGTCA
CCTTTCAGCTCAAGGCTGAGAGGAACTACCACATCTTTTACCAGATCCTG
TCCAACCAGAAGCCAGAGCTGCTGGACATGCTGCTGATCACCAACAACCC
TTACGACTACTCCTACATCTCCCAAGGAGAGGTAACAGTCGCCTCCATCA
AYGAYGCCGAGGAGCTGATGGCCACTGACAGCGCCTTTGACGTSCTGGGC
TTCACTCCGGAGGAGAAGATGGGCGTCTACAAACTGACYGGCGCCATCAT

GCACTACGGAAACATGAAGTTTAAGCAGAAGCAGCGCGAGGAGCAGGCTG
AGCCAGATGGGACGGAGGCTGCAGATAAATCCGCTTACCTCATGGGGCTG
AACTCTGCTGACCTCATCAAAGGACTGTGCCATCCCAGAGTCAAGGTCGG
GAATGAATACGTCACCAAAGGACAAAGTGTGGACCAGGTCTACTACCCCA
ACAAGGAGGCCTTTAAATGTGAGGAGTGTGGGAAGCACTACAACACCAAG
CTGGGCTACAAGCGCCATGTGGCCATGCACTCCGCCACTGCCGGGGACCT
CACCTGTAAGGTGTGCATGCAGACGTACGAGAGCACGCCGGTTCTCCTGG
AGCACCTGAAGAGCCACTCCGGGAAGTCCTCTGGAGGCGCCAAGGAGAAG
AAACACCCGTGTGACCACTGCGACCGCCGCTTCTACACCAGGAAGGACGT
AAGACGCCACATGGTCGTCCACACGGGGCGGAAGGACTTCCTGTGTCAGT
ACTGTGCCCAGCGCTTTGGCAGGAAGGACCACCTGACGCGCCACGTGAAG
AAGAGCCACTCCCAGGAGCTGCTGAAGATCAAGACGGAGCCTCCTGATAT
GCTGGTTCTTCTGGCATCAGCGTCGCCCCCCTGCTCCGTGAAGGAGGAGC
TCAGCCCTATGATGTGCGGCGTGGGGCCCAACAAAGACCCCATGATAGGC
AAACCCTTCCCTGGGGGCCCCCCCTTCCCCATGGGCATGTACAACCCCCA
CCAC------CTCCAGGCCATGTCAGGCCCC-------------------
--
CACCCGTCCCTGATGCCCCCCTCCCTGNNNNNNNNNNNNNNNNNNNNNNNNNNNNNNNNNTACCTCATTT
ATGCCTCTTTC
TCATTCATGGGATGTTTACAAATCAGTGATGGATCCAATATAGTGAACCT
GCTGGCTAGTAACTCCCCGAGTGTAACCTATGCTATGACCCAGCAGAAAT
ACTTCAGCAACTATAGTCCTGTGATCGGGTTTTACATTTATGAGCCAATT
GAGTACTGGAACTCCACAGTACAGGATCACCTGAAAACTCTGAGTCATGG
TTTTAACAAGATCTCCTGGATTGACAATTTCTTCCACTACTTGAGGGTGG
TGAATGTGAGTGCAACAACAAAAAGCGATTTCATCACYATACTCAAGGGC
TCTTTCCTGCGCAGCCCAGAGTATCAGCACTTCACCGAGGATATTATATT
CTCCAAGA---ACCGTGAGACCG------ATGAGTACGACATCATCGCTT
CTAGGATGTACTTGGTGGCGCGGACGACGGAGAAGAAGCGCGAAGAAGTG
GTGGAGCTTCTGGAGAAACTTCGTCCACTAATGCTTATCAACAGCATCAA
GTTCATTGCCTTCAACCCTACGTTTGTGTTCATGGACCGCTACAGCTCTT
CTGTGATCTCGCCCATCCTGACCTCAGGCTTCAGCGTACTCACAATCCTC
ATCCTCACTTTTTTTCTGGTTATCAATCCCTTGGGGAACTTCTGGCTCAT
TCTCACGGTAACCTCTGTAGAGCTCGGTGTTTTGGGTTTAATGGGATTTC
ACCAGTTTGAATGGCAACCAGCTCTCAAAAATGTGTCCACTTCTTGCAAT
GTAGGCATTATTAACGGGCTCTCTGGATGGGCCTCATCGGTGGATGACTC
CCCAGCTGACACCATCACTCGTCGGTTTCGCTATGATGTGGCACTGGTKT
CAGCGTTAAAGGATCTGGAGGAGGACATCATGGAGGGGCTGAGAGAGAGT
GGGCTGGAAGACAGTGCTTGTACTTCAGGCTTCAGTGTCCTGATCAAGGA
ATCCTGTGACGGCATGGGTGATGTCAGCGAAAAGCATGGCGGAGGACCAG
CTGTTCCTGAGAAGGCTGTGCGTTTTTCTTTCACCATCATGTCTGTCTCT
GTAAGGGCAGAGACTAAGGAG-----------------------------
-GAAGACATTACCCTTTTCACCGAGCCAAAGCCAAACTCGGAGCTGTCCT
GTAAACCCCTCTGCCTGACATTTGTGGATGAGTCAGACCATGAGACACTC
ACAGCAGTCCTGAGCCCCGTAGTTACAGAGCGTAAAGCAATGGCAGAAAG
CAGGCTCATCCTATCCATAGGTGGACTGCCTCGCTCATTCCGCTTTCACT
TCAGAGGCACAGGATATGATGAGAAGATGGTACGTGAGATGGAGGGCCTT
GAGGCTTCAGGGTCCACCTATATCTGCACTCTTTGTGACTCCACTCGAGC
AGAGGCCTCTCAAAACATGGTGCTGCACTCTGTCACCCGCAGTCACGACG
AAAACCTGGAGCGCTATGAAATCTGGCGAACCAATCCCTATTCTGAGTCT
GTGGAGGAGCTGAGAGAAAGAGTAAAGGGGGTATCAGCCAAACCCTTCAT
GGAGACTCAACCAACGCTTGACGCTCTGCATTGTGACATTGGCAATGCCA
CTGAGTTCTACAAAATCTTCCAAGACGAGATTGGAGAGGTGTACGAAAAG
GT---CACT---CCTAGCCGTGAGGAACGGCGCAGCTGGAGGGCAGCCTT

AGATAAACAGCTGAGGAAGAAGATGAAGCTCAAACCGGTGATGAGGATGA
ATGGAAACTATGCCCGCAAGCTGATGACCATGGAGGCTGTGGAGGTGGTG
TGCGAGTTGGTGCCCTCAGAGGAGAGGAAAGAGGCCCTGAGGGAGCTCAT
GAGACTGTACCTCCAGATGAAGCCTGTCTGGCGTGCCACCTGCCCAGCCA
AGGAGTGCCCTGACCAGCTGTGCCGCTACAGCTTTAACTCTCAGCGCTTT
GCAGACCTCCTCTCTTCCACCTTCAAGTACAGGTATAATGGAAAGATAAC
AAATTACCTGCACAAGACCCTGGCCCATGTGCCCGAAATCATAGAGAGAG
ATGGATCCATAGGGGCCTGGGCCAGTGAGGGGAACGAATCAGCNNNNNNNTCGTACA
ACATCGAGATGGGACCCTTGGGGCCTCGGTGGAAGGACAGCCCACAGCCT
TTCAACTGCTCTATCGAAGATCCAACTAAACAGACAAAGTTCAAGGGTAT
CAAGACGTATATTTCATACCGGGTCACACCGAGCCACACAGGACGACCTG
TGTACAGACGCTACAAACACTTCGACTGGCTGTACAACCGCTTACTGCAC
AAGTTCACTGTGATCTCTGTTCCTCACCTGCCTGAGAAGCAGGCCACRGG
GCGATTTGAAGAAGACTTCATTGAGAAGCGAAAGAGACGATTGATACTCT
GGATGAACCACATGACCAGCCACCCAGTCCTCTCCCAGTACGAAGGCTTT
GAGCACTTTCTGATGTGTGCCGACGACAAACAGTGGAAACTGGGCAAGAG
ACGGGCGGAGAAGGACGAGATGGTAGGTGGACACTTTATGCTGACCCTCC
AGATTCCCAACGAGCACCAGGATCTTCAGGACGTAGAGGAGCGCATTGAC
TCCTTCAAGTCCTTTGCTAAGAAAATGGACGACAGCGTGATGCAGCTCAC
GCACGTTGCCTCGGAGCTGGTTCGTAAACACCACGGCGGGTTCAGGAAGG
AGTTCCAGCGACTTGGAAACGCCTTCCAGTCTGTCAGCCAGGCGTTCATG
CTGGACCCTCCGCACAGCTCTGACACCTTCAACAACGCCATCTCACACCC
TCTCGCTACGTTCCTCAAACTGACCTCTTTGGGTTTTATCATCGGAGTCG
GTGTGGTCGGGAACCTCCTGATCTCCATCCTGCTGGTCAAAGACAAGAGC
TTGCACCGAGCGCCCTATTATTTCCTGCTGGATTTGTGTGCGTCCGATAT
CCTGCGCTCCGCCATCTGCTTCCCCTTCGTCTTCACCTCGGTCAAGAATG
GATCCGCGTGGACGTACGGGACGCTGACCTGCAAAGTCATCGCCTTCCTG
GGTGTGCTCTCCTGTTTCCACACGGCATTTATGCTATTCTGTGTTAGCGT
CACRCGCTACCTAGCCATCGCACATCACCGTTTCTACACCAAGAGGCTGA
CCTTCTGGACCTGCTTGGCTGTCATCTGCATGGTTTGGACGTTGTCAGTG
GCGATGGCGTTCCCGCCGGTGCTGGACGTAGGGACGTACTCGTTTATCCG
CGAGGAGGACCAGTGCACGTTCCAGCACCGCTCCTTCAGGGCGAACGATT
CGTTGGGATTCATGCTCCTGCTGGCGCTCATCCTCCTCGCCACACAGCTG
GTTTACCTCAAGCTCATCTTCTTTGTCCACGACCGCCGAAAAATGAAGCC
CGTCCAGTTTGTGCCCGCCGTCAGCCAGAACTGGACCTTCCACGGGCCAG
GCGCAAGCGGGCAGGCGGCGGCAAACTGGCTGGCCGGCTTCGGCCGAGGC
CCTACTCCGCCTACTTTGCTGGGAATCCGGCAGAACAGCAACGCAGCGGG
CCGCCGGCGTCTTTTGGTGTTGGATGAGTTCAAAACAGAGAAGAGGATTA
GTAGGATGTTCTACATCATGACGTTTTTCTTCCTGGCGCTCTGGGGGCCC
TATCTGGTCGCCTGCTACTGGCGGGTGTTTGCGAGGGGCCCCGTGGTCCC
CGGGGGCTACCTGACGGCAGCTGTGTGGATGAGCTTTGCCCAGGCTGGAG
TCAATCCGTTCATCAGCATCTTCTCCAACAGCGAGGCCAAATCTCGCTTT
CACCCTGGCGTGGGGACTGGGCCTGGCACAGAGC---GCAGCGTCCCACT
CGGCAACAGCTTGCTCTCCCCGCAGCAGAGCGAGGAGCCCACTGTTGCCA
CCCCCCCGCAGCGATGGTTTGTCACCC---CTGCCAACAACCGACTGGAC
TTTGCTGCCTCGGCATACGACGCCGCG---------GATTTCGCCGGTAA
CGCCGCCACCTTGCTGTCCTACGCGGCGGCCGGAGTGAAGGCTC------
TTCCCCTGCCGACTGCGGGCTGCTCCAACCGGCCTCTTGGCTATTACGCA
GACCCATCGG---GCTGG---GGAGGACGCACGCCGCCGCAGTACTGTGG
CGTCAACACCAAATCCAGCTCGGTCTTTTCCTGCTGGCCCACTAACTCTC
TCGGTGGCAGGGCAGGAG---CC---AATTACCTGT---------CCGAG
GA---GGGT---GACTC---CATCCCCACGGAGAGGTCACCA---AT---
CGGCGGCTCGGAGGAG---ACCAAACCCAAAGACATGAC---GTCAGA--

-GTCGAGCTGGATAGAG---ACGCCGTCCTCCATAAAGTCCATCGACTCC
AGCGATTCTGGGATCTTTG---AACAGGCCAAGAGGAGGAGAATCTCACC
TTCTGCCACGCCT----------GTTTCAGAGACTGTGTCCCCGCTAAAT
CTGAGCATCACTCAACAGGCGAAGTCACAGAGAGAGAAGTGGCGTTGGGG
ATAAATCCGTTCGCTGATGGGATGGGCGCCTTCAAAATAAACCACAGCTC
CCACGACATCGGCTCCGG---ACAGACGGCGTTTTCCTCCCAGGCG---C
CCGGCTAC---GCAGCAGCCGCCCTGGGA---CACCACCA------CCAC
CCGACCCACGTTGGCTCT---TACTCCACGGCGGCGTTCAACTCCACCAG
GGACTTTCTCTTCAGAAATCGGGGTTTCGGGGACGCCGCCGG--------
----GGCTCAGCACAGTTTGTTCGCTTC---------CGGAAGTTT---C
------GCAGGGCCACATGGACACTCAGATGCAGCGGGGCACCTGCTCTT
CCCGGGGCTCCACGAG---CAAGCTGCGAGCCATGCGTCCTCCAACGTGG
TCAACAGCCAGATGCGGCTGGGCTTCTCGGGGGACATGTACGGACGCGCA
GACCAGTACGGCCACGTTACGAGCCCGCGGT---CCGACCACTACGCGTC
CACCCAGCTGCACGGCTACGGCCCCATGAACATGAATATGGCAGCA---C
ACCACGGAGCCGGGGCCTTCTTCCGATACATGAGGCAACCCATCAAACAA
GAGCTCATCTGCAAGTGGATCGAGCCGGAGCAGCTGACGAACCCCAAAAA
GTCGTGCAACAAAACTTTTAGCACGATGCACGAGCTCGTCACGCATCTGA
CGGTGGAGCATGTTGGGGGACCAGAGCAGACCAACCACATCTGTTTCTGG
GAGGACTGCGCCCGAGAGGGGAAGCCCTTCAAAGCCAAATACAAACTTGT
GAATCATATCAGAGTACACACCGGAGAGAAGCCCTTTCCCTGCCCCTTCC
CGGGCTGTGGCAAA
>Halieutichthys aculeatus

--------------------------GGATAAAAACATTGTCGTGTTCAGT
CGTTCAACCACGAGACTAATCCTGAACGAAGCAGAGCTCATCATGGCGCT
GGCCCAGGAGTTTCAGATGAGGGTGGTGACGGTAAACCTGGAGGAGCAGG
CGTTCCCCAGCATCGTCCAGGTGATCAGCAGCGCCACCATCTTAGTCAGC
ATGCACGGAGCTCAACTCATCACCTCACTCTTCCTGCCCAGAGGAGCCGT
TGTGGTGGAGTTGTTCCCCTTCGCTGTGAACCCGGAGCAGTACACCCCAT
ATAAAACTCTCGCTTCCCTTCCGGGCATGGACCTTCATTACATCTCCTGG
AGGAACACTGTGGAGGAGAACACTGTCACCCACCCAGACAGGCCCTGGGA
ACAAGGGGGTATCGCTCATTTGGATAAAGAGGAGCAGGAACGAATCCTGG
CGAGTAAGGATGTGCCCAGGCATCTCTGCTGCCGAAACCCAGAGTGGCTC
TTCCGGATCTACCAGGACACGTTTGTGGACGTCGCCTCTCTCCTAGAAGT
CCTTAA---AGAGGGCATGAAT---GCAAAGCCCAACTTGAGGAA---GT
CAAAACCGGCCAGCACGCTGCACCCGGGCAGGGTCAGAGAACCCCAGTGT
CAGACTTCAGTTCAAACCAGCAGCGAGGCTAAACTCACGGTCTCCTGGCA
GATACCGTGGAATCTGAAATACCTGAAGGTCAGAGAGGTGAAGTACGAGG
TGTGGATCCAGAAGAAGGACGCCAACAAGGGGTCCCTGGAGGATCAGATC
ATCCAGGCGAACCCGGCGCTGGAGGCCTTCGGGAACGCCAAGACGCTCCG
GAACGACAACTCGTCCCGCTTCGGGAAGTTCATCCGAATCCACTTCGGCA
CCAGCGGGAAGCTGTCGTCTGCCGACATCGAGACGTACCTGCTGGAGAAG
TCCCGGGTCACCTTCCAGCTCAAGGCTGAGAGGAACTACCACATCTTCTA
CCAGATCCTGTCCAACCAGAAGCCGGAGCTGCTGGACATGCTGCTGATCA
CCAACAACCCCTACGACTACTCCTACATCTCGCAGGGGGAGGTGACCGTC
GCCTCCATCAACGACTCGGAGGAGCTGATGGCCACGGACAGCGCCTTCGA
CGTGCTCGGCTTCACCCCCGACGAGAAGATGGGCGTCTACAAGCTGACGG
GTGCCATCATGCACTACGGCAACATGAAGTTCAAGCAGAAGCAGCGCGAG
GAGCAGGCCGAGCCGGACGGGACGGAGTCCGCGGACAAATCGGCCTACCT
GATGGGGCTGAACTCCGCCGACCTCATCAAAGGGCTGTGCCACCCCAGGG
TCAAGGTGGGGAACGAGTACGTCACGAAAGGCCAGAGCGTGGACCAAGTC
TACTACCCCAACAAGGAGGCCTTCAAATGCGATGAGTGTGGGAAGCACTA
CAACACCAAGCTAGGATACAAGCGCCATGTCGCCATGCATTCCGCCACAG
CAGGGGATCTCACCTGTAAGGTGTGCATGCAGACCTACGACAGCACGCCG
GTGCTCCTGGAGCACCTGAAGAGCCACTCTGGGAAGTCCTCAGGTGGCAC
CAAGGAGAAGAAACACCAGTGTGACCACTGTGATCGGCGTTTCTACACAA
GGAAGGACGTGAGACGCCACATGGTGGTCCACACGGGCCGGAAGGACTTC
CTGTGCCAGTACTGTGCCCAGCGCTTCGGCAGGAAGGACCACCTGACGCG
CCACGTGAAGAAGAGCCACTCGCAGGAGCTGCTAAAAATCAAGACGGAGC
CCCCAGACATGTTGGGCCTCCTGGCTTCGGGGTCGCCACCATGCTCCGTG
AAGGAGGAGCTTAGCCCCCTGATGTGCGGTATGGGGCCCAACAAAGACCC
CATGATGGGCAAACCCTTTCCCAGCGGGGCCCCTTTTCCAATGGGCATGT
ACAACCCTCACCAC------CTGCAGGCCATGTCTCATTCTGGGGTGGGT
CATTCA------CACCCGTCCCTGATGTCCAGCCCCCTGTCTGCAGCTAT
GGGCATGGGCTGTCCCATGGACTACCTCATCTACGCTTCTTTCTCATTCA
TGGGATGTTTACAAATCAGTGACGGGTCGAATATCGTGAACCTGCTGGCG
AGTAACTCTCCAAGTGTCTCATATGCTCTGACCCAGCAAAAATACTTCAG
TAACTACAGTCCGGTCATTGGGTTTTACATTTATGAGCCCATCGAGTACT
GGAACTCGACAGTGCAGGAGCACCTGAAGACTTTGAGTCACGGCTTCAAC
AAGATCTCCTGGATGGACAACTTTTTCCACTACCTGCGGGTGGTGAATGT
GAGCGCATCAACCAAGAGCGACTTCATCAACATCCTCAAGGGCTCCTTCC
TGCGCAGCCCGGAGTACCAGCACTTCACCGAGGACATCATTTTTTCAAAG
A---ACCGCGAGACCG------ACGAGTACGACATCATCGCCTCACGGAT
GTACTTGGTGGCGCGGACTACAGAGAAGAAGCGCGAGGAAGTGGTAGAGC

TTCTGGAAAAACTTCGCCCGTTGATGCTGATCAACAGCATCAAGTTCATT
GCCTTCAACCCCACGTTTGTGTTCATGGACCGCTACAGCTCCTCGGTCAT
CTCGCCCATCCTGACCTCAGGATTCAGCGTGCTCACAATCTTCATCCTCA
CTTTGTTCCTGGTCATCAACCCCTTGGGTAATTTCTGGCTCATCCTCACT
GTAACGTCGGTGGAGCTGGGCGTCTTGGNNNNNNNN----------------------

-----------------------------------TCTTACACCATTGAG
ATGGGTTCCGTGGGGCCCCGATGGAAGGAGAGCCCACAGCCGTTCTCCTG
TTCCGTTGAAGACCCCACAAAACAGACAAAGTTCAAGGGTATCAAGACGT
ACATTTCATACAGGGTGACACCAAGCCACTCTGGGCGTCCCGTGTACAGA
CGCTACAAACACTTTGACTGGCTGTACAACCGCTTACTGCACAAGTTCAC
TGTGATCTCTGTGCCTCATCTGCCCGAGAAGCAAGCCACGGGGCGCTTTG
AGGATGACTTCATTGAGAAGCGCAAGAGGCGACTGATACTGTGGATGAAC
CACATGACCAGTCACCCAGTCCTCTCCCAGTATGAGGGCTTTGAGCACTT
TCTGATGTGCGCTGACGACAAGCAGTGGAAACTGGGAAAGAGACGGGCAG
AGAAAGACGAGATGGTGGGAGCCCATTTCATGCTGACTCTCCAGATCCCT
AATGAGCACCAGGACCTTCAGGATGTGGAGGAGCGGATCGACTCCTTTAA
GTTCTTTGCTAAAAAGATGGATGACAGCGTGATGCAGCTCACGCACGTTG
CCTCGGAGCTGGTGCGTAAGCACCTGGGCGGGTTCAGGAAGGAGTTCCAG
AGGCTGGGAAATGCCTTCCAGTCTATCAGCCAGGCGTTCATGTTGGACCC
TCCCCAGAGCTCAGAGACCTTCAACAACGCCNNNNNNNNN-------------------

>Halosauropsis macrochir
AGCCTTCTTATTCGAGCAGAGCTAAGTCAACCCGGAGCCCTTCTAGGAGA
TGACCAAATTTATAATGTTATCGTTACAGCACACGCCTTCGTAATGATTT
TCTTCATGGTAATACCCGTTATAATTGGAGGCTTTGGAAACTGACTAGTT
CCCTTAATAATTGGAGCCCCCGATATAGCATTCCCACGAATAAATAACAT
AAGCTTTTGACTTCTACCCCCATCACTCCTCCTTCTATTATCCTCTTCTG
GAGTTGAAGCAGGAGCAGGGACAGGATGAACAGTCTACCCCCCGCTTGCT

GCTAATCTAGCCCATGCCGGAGCATCCGTAGACCTAACTATCTTCTCACT
CCACCTAGCAGGCGTTTCATCAATTCTCGGAGCCATTAACTTTATTACCA
CAATTATTAACATAAAACCCCCAGCCATCTCACAATACCAAACCCCACTT
TTCGTGTGATCAGTGCTTGTTACTGCAGTACTGCTACTATTATCTCTGCC
AGTCTTAGCCGCAGGAATCACTATACTACTTACAGACCGAAATCTAAACA
CAACTTTCTTTGATCCAGCAGGAGGAGGAGACCCAATCCTTTACCAACAC
CTANNNNNNNNNNNNNNNNNNNNNNNNNNNNNNNNNNNNNNNNNNNNNNNNNNNNNNNNNNNNNNNNNNN
NNNNNNNNNNNNNNNNNNNNNNNNNNNNNNNNNNNNNNNNNNNNNNNNNNNNNNNNNNNNNNNNNNNNNN
NNNNNNNNNNNNNNNNNNNNNNNNNNNNNNNNNNNNNNNNNNNNNNNNNNNNNNNNNNNNNNNNNNNNNN
NN---

----------------GGACGAGTATATTGTGGTCTTCAGCCGCTCCGTT
AACAGGCTGATCCTGAACGAGGCAGAGCTGATCCTGGCACTAGCCCAGGA
GTTTCAGTTGAAGGTCGTTACAGTGTCCCTAGAGGAACAGTCTTTTGCAG
ACATCATCCGAGTCATTAGCAGGGCATCTATGCTGGTCAGTATGCACGGG
GCCCAGCTGGTCACCTCTCTCTTCCTTCCCCGTGGCGCTGCTGTGGTGGA
GCTCTTTCCATATGCCGTCAACCCAGAGCATTATGCTCCCTACAAGACAC
TCACCTCGCTGCCAGGCATGGACCTGCAGTATGTGGCCTGGAGGAACACC
AAGGAGGAGAACTCTGTGACCTTCCCCGAGCGTGCCTGGGACCAGGGCGG
CATTGCACACTTGGAGAAGGAGGAGCAGGAACGTATCCTGAAGAGCAGCG
AGGTGCCGCGACACCTGTGCTGTCGGAACCCGGAGTGGCTCTTCCGCATC
TACCAGGACACCAAGGCGGACATTGCATCCCTACTTGAAGCACTGCG---
CCAGGGACTGACC---TCCAGGCCAGGGCCCAAAAG---GGCTAGGCCCA
GCAGCACAGTCCACCCAGGCAGGGTGAGGGAGCCCAAGTGCCAGACCTCC
GTCCAGGCTACCAACGAGGCCAAGCTGACCGTGTCCTGGCAGATCCCGTG
GAACCTCAAGTACCTGAAGGTCAGGGAGGTGAAGTATAGAGTCNNNNNNNNNAAGAAGG
ATACCAGCAAGGGCACACTGGAGGATCAAATCATCCAGGCTAACCCAGCG
CTGGAGGCTTTCGGCAATGCTAAGACTGCGAGGAACGATAACTCCTCGCG
TTTTGGGAAATTCATCCGCATTCATTTTGGAGTAAGTGGTAAGCTTTCCT
CTGCTGACATCGAAACTTACCTGCTTGAGAAATCTCGTGTCACCTTTCAG
CTCAAAGCCGAGAGGAACTATCACATCTTTTACCAGATTATGTCCAACAA
AAAGCCTGAATTGCTGGACATGATGTTGATTACTAACAACCCATACGATT
ATGCTTACGTCTCCCAAGGAGAGGTGACAGTCGCGTCAATCGATGACTCA
GAGGAACTGATCGCCACAGACAGTGCCTTTGATGTGCTGGGATTCACAGC
TGAGGAGAAGATGGGTGTCTACAAGCTGACAGGTGCTATCATGCACTATG
GAAATATGAAATTCAAGCAGAGGCAGCGTGAGGAGCAGGCTGAGCCTGAT
GGCACTGAGTCTGCTGACAAGTCAGCCTACCTGATGGGGTTGAACTCAGC
CGACCTTCTCAAAGGACTTTGCCACCCAAGGGTTAAAGTGGGGAATGAGT
ATGTCACAAAGGGCCAAAGTGTGGATCAGGTTTACTTTCCCAAC-AAGAG

```
GCCTTCAAGTGCGAGGAGTGCGGCAAGAACTACAACACCAAGCTGGGCTA
CAAGCGTCACATGGCCATGCATGCGGCCACCAGCGGCGACCTCACCTGCA
AGGTGTGCCTGCAGAGCTAYGAGAGCACGCCCGTGCTGCTGGAGCACCTC
AAGAGTCACTCGGGGAAGTCCTCGGGTGGGGCCAAGGAGAAGAAGCACCC
CTGTGACCACTGCGACCGGCGCTTCTACACGCGCAAGGACGTGCGGCGCC
ACATGGTGGTGCACACCGGCCGCAAGGACTTCCTGTGCCAGTACTGCGCC
CAGCGCTTTGGCCGCAAGGACCACCTGACCCGGCATGTGAAGAAGAGCCA
CTCGCAGGAGCTGCTGAAGATCAAGACGGAGCCACCGGACATGTTGGGCC
TGCTGGGCTCCGGCTCCCCGCCCTGCTCTATCAAGGAGGAGCTCAGCCCC
ATGATGTG---TATGGGCCCCTCCAAGGACTCCCTGATGGGCAAGCCCTT
CCCTGGTG------CCTTCCCCATGGGCATGTACAACCCCCAC-------
--CTGCAGGCTATGTCTAACTCTGGGGTGTCC------------CACCAC
TCCCTGGTGTCCGGGTCTCTGTCTGCGGCCATGGGGATGGGCTGCCACAT
GGAGTACCTTATTTATGCATCGTTTTCCTTCATGGGATGTTTACAAATCA
GCGACGGATCGAACATAGTCAACCTTTTAGCCAGCAACTCCCCGAGTGTC
TCGTATGCTCTGACCCAGCAGAAATATTTCAGCAATTACAGTCCTGTGAT
TGGGTTCTACATCTATGAACCAATAGAGTACTGGAATTCCACAGTTCAAG
AACACCTGAAGACGCTGGGCCATGGATTCAATAAGATTTCATGGATCGAC
AACTTCTTTCAGTATCTGAAGGTAGTGAATGCCAGCGCGTCAACCAAAAG
TGAATTCATAACCATCCTCCAGACTTCCTTCTTAAGGAGCCCAGACTACG
AGCACTTCAAGGATGACATCATTTTTTACAAAA---TGAGGG--------
----ATGAAACTGAAATCGTCGCGTCCCGCATGTACCTGGTGGCCAGAAC
CACGGAGAAGACCAGAGAGGAGGTGGTGGAATTACTGGAGAGACTGAGAC
CTCTGTCTCTCATCAACAGCATCAAATTCATCGTCTTCAATCCAACCTTT
GTGTTCATGGATCGATATAGCTCCTCAGTCATTTCTCCGATCCTCACCTC
TGGATTCAGTGTCCTGATTGTCTTGATCCTGACGTTCTTTCTGGTCATAA
ACCCCCTCGGAAACTTCTGGTTGATACTGACGGTCACCTCAGTGGAGCTG
GGAGTTCTTGGCCTNNNN------------------------------------
--------------------------------------------------
--------------------------------------------------
--------------------------------------------------
--------------------------------------------------
--------------------------------------------------
--------------------------------------------------
--------------------------------------------------
--------------------------------------------------
--------------------------------------------------
--------------------------------------------------
--------------------------------------------------
--------------------------------------------------
--------------------------------------------------
--------------------------------------------------
--------------------------------------------------
--------------------------------------------------
--------------------------------------------------
--------------------------------------------------
--------------------------------------------------
--------------------------------------------------
--------------------------------------------------
--------------------------------------------------
--------------------------------------------------
--------------------------------------------------
--------------------------------------------------
```


---------------------TCATACACCATCGAGATGGGCCCCTGGGG
CCCCCAGTGGAAGGAGAGCCCCCAGCCCTTCTCCTGTTCCATCGAGGACC
CCACCAAACAGACTAAATTCAAGGGGATAAAGACCTACATCTCGTACCGC
GTGACGCCCAGCCACACAGCTCAGCCCGTATACCGCCGCTACAAGCACTT
CCACTGGCTCTACAACCGGTTGCTACACAAGTTCACGGTCATCTCCGTGC
CACACCTGCCCGAGAAGCAGGCCACGGGCCGCTTCGAGGAGGACTTCATC
GAGAAGCGGAAGAGGCGGCTCATCCTGTGGATGGACCACATGACCAGCCA
CCCGGTGCTGTCGCAGTACGAGGGCTTCGAGCACTTCCTCATGTGCGCCA
ACGACAAGCAGTGGAAGCTGGGCAAGCGGCGGGCGGAGAAGGAAGAGTTG
GTGGGCGCGCACTTCATGCTCACCTTCCAGATCCCCAGCGAGCACCAGGA
CCTGCAGGACGTGGAGGAGCGCGTGGACTCCTTCAAGGCCTTCGCCAAGA
AGATGGACGACAGCGTGCTGCAGCTCACACACGTGGCGTCCGAGCTGGTT
CGGAAGCACCTGGGCGGTTTCCGGAGGGAGTTCCAGCGGCTGGGGAACGC
CTTCCAGTCCATCAGCCAGGCCTTCACCCTGGACCCTCCCTACTGCTCCG
ACGCCCTCAACACCGCCATCNNNNNN----------------------------------

>Harpadon nehereus
AGCCTTTTGATCCGTGCTGAGCTGAGCCAGCCGGGGGCCCTGCTCGGTGA
CGATCAAATTTATAACGTAATCGTTACTGCCCACGCCTTCGTAATAATTT
TCTTTATAGTAATGCCAATTATGATCGGGGGCTTTGGAAATTGACTCATT
CCCCTGATGATCGGTGCCCCCGATATGGCGTTTCCCCAAATGAATAACAT
AAGCTTTTGACTCCTCCCACCCTCTTTCCTTCTTCTCTTGGCATCATCGG
GAGTCGAAGCAGGGGCTGGAACCGGCTGAACAGTCTATCCTCCGTTAGCG
GGAAACCTTGCTCACGCCGGGGCCTCTGTAGATCTAACCATCTTCTCGCT
ACACTTGGCTGGGATTTCCTCTATTTTGGGAGCCATTAATTTTATTACGA
CAATTATCAATATAAAACCTCCCGCCATTTCACAATACCAGACACCCCTC
TTTGTTTGGGCTGTACTGATTACGGCTGTCCTTCTCCTCCTCTCCTTACC
CGTTCTTGCAGCCGGAATCACAATGCTCTTAACTGATCGAAATCTTAATA
CCACCTTCTTTGACCCTGCAGGGGGCGGCGATCCCATCCTCTATCAGCAC
TTATTCTGATTCTTTGGCCACCCCGAAGTGTATATTTTAATCCTCCCTGG
ATTCGGCATGATCTCCCACATTGTAGCCTACTACGCAGGGAAGAAAGAGC
CGTTTGGCTATATGGGCATGGTCTGGGCAATGATGGCAATCGGCCTATTA
GGTTTCATCGTCTGGGCCCACCACATGTTTACNNNNNNNNNNNNNNNNNNNNNNNNNNNNNNNNNNNNNN
NNNNNNNNNNNNNNNNNNNNNNNNNNNNNNNNNNNNNNNNNNNNNNNNNNNNNNNNNNNNNNNNNNNNNN
NNNNNNNNNNNNNNNNNNNNNNNNNNNNNNNNNTTCCCCGCCATCTGCAAG
ACGGAGGACTTCCTGCAACTCCCCAAAGACATGGTCATACAGCTTCTGTC
CCACGAAGAGCGGGAGACAGAGGATGAGAGGATGGTGTATGAGGNAGCCAT
TCACTGGGTCAACTATGACGGGGAGAGGAGGCACTGCCACCTACCAGAAC
TTCTGAGGACAGTTAGTCTAGCCCTTCTCCCAGCCATTTTCCTCATGGAG
AATGTCTCCACGGAGGAGCTGATCAATAACCAGACCAAAAGCAAGGAGCT
GGTGGACGAGGCCATTCGCTGCAAGTTGAGAATCCTCCAGAACGACGGGG
TGGTCAACAGTCCCTGTGCCAAACCTAGAAAGACCAGCCATGCCTTGTTC
CTTCTGGGAGGGCAGACTTTCATGTGTGACAAACTTTACTTGGTGGATCA
AAAGGCCAAGGAGATCATTCCCAAGGCTGACATCCCCAGTCCCAGGAAGG
AGTTCAGTGCTTNTGCCATCGGGTGTAAGGTNTACGTGACAGGTGGAA--GT
GGCTC-TGAGAACGGAGTGTCCAAANACGTGTGGGTGTTAGACACCATCCA
TGAGGAGTGGTTACAAGGAGCACCCAGCGANNNNNNNNNNNNNNNNNNNNNNNNNNNNNNNNNNNNNNNN
NNNNNNNNNNNNNNNNNNNNNNNNNNNNNNNNNNNNNNNNNN------------------AG
ATGCGTACATTGTTGTGTTTAGTCGTTCGAGCACAAGGCTGATTCTAAAC
GAAGCAGAGTTGATCCTCGCGCTGGCCCAAGAGTTTCAGATGAGGGTGGT
TACCGTGTCCTTGGAGGAGCAAACCTTTCCCAGCATTATCCAGGTGATCA
GCAGTGCCTCCATGCTAGTTAGTATGCATGGAGCTCAGCTCGTCACGTCT
CTCTTTCTCCCCAGAGGAGCTGCCGTGGTGGAGCTTTTTCCCTAYGCTGT

GAACCCAGAACAGTACACCCCATACAAAACCCTTGCCTCCCTGCCAGGCA
TGGACCTTCAGTACGTTTCGTGGAGGAACACGATGGAAGAGAACACTGTG
GCCCACCCTGAAAGACCGTGGGACCAAGGAGGTATCGCCCACTTAGACAA
GGAAGAACAGGAGCGAATCTTAGCCAGCAAAGATGTCCCAAGGCACCTGT
GCTGCCGCAACCCAGAGTGGCTTTTCAGGATTTACCAGGATACTCTGGTG
GACATCCCGTCCTTCATGGAGGTCCTTAA---GGAGGGTCTGAAA---AC
GAGGCCGAGCTTGAAGAA---GTCCAAGCCGACCAGCACAGTTCACCCGG
GACGGGTCAGAGAACCTCAGTGCCASACATCAGTCCAAGCCACTAATGAG
GCTAAACTCACAGTATCCTGGCAGATCCCGTGGAACCTTAAGTACTTAAA
GGTGCGAGAGGTGAAGTACGAGGTTTGGATCCCGAAAAAGGATCCCAGCA
AGGGAACCCTGGAAGATCAAATCATTCAGGCAAACCCTGCACTGGAGGCT
TTTGGTAATGCCAAAACAGTGAGGAATGATAATTCCTCCCGCTTTGGGAA
ATTCATTCGAATTCACTTTGGAAYAAGTGGGAAGCTGTCCTCTGCGGACA
TTGAAACGTACCTGTTGGAAAAATCCCGGGTCACCTTTCAGCTTAAGGCA
GAGCGGAATTATCATATCTTCTTCCAGATCTTATCCAACCATAAGCCAGA
GCTGTTGGATATGCTGTTGATCACCAACAACCCCTATGACTACTCATTCA
TCTCCCAAGGAGAGGTGACAGTAGCGTCCATCAACGATGCAGAGGAGCTG
ATGGCCACTGACAGTGCCTTCGATGTACTCGGCTTCACTCTAGAAGAGAA
GTTGGGAGTCTACAAGCTGACAGGGGCTATCATGCACTACGGCAACATGA
AGTTCAAGCAGAAGCAACGCGAGGAACAGGCTGAGCCTGATGGCACAGAG
GCTGCTGATAAGTCAGCTTACCTAATGGGGCTGAACTCAGCTGACCTCAT
TAAAGGACTCTGTCATCCCAGAGTTAAGGTAGGAAATGAGTATGTAACTA
AAGGTCAGGGTGTAGATCAAGTCTACTACCCCAACAAAGAGGCCTTCAAG
TGCGAGGAGTGTGGCAAGCACTACAACACCAAGCTGGGATACAGGCGCCA
TGTGGCCATGCACTCCGCCACCGCAGGGGACCTCACCTGTAAAGTCTGCA
TGCAGAGCTACGAGAGCACGCCGGTGCTCCTGGAACACCTCAAGAGCCAC
TCGGGGAAGTCCTCGGGCGGCGCCAAGGAGAAGAAGCACCCATGTGATCA
CTGCGACCGTCGGTTTTACACGCGGAAGGACGTGAGGCGGCACATGGTCG
TCCACACGGGCCGAAAGGACTTCCTGTGCCAGTACTGTGCCCAACGCTTC
GGCAGGAAGGACCACCTGACGCGACACGTGAAAAAGAGCCACTCGCAAGA
GCTGCTGAAGATCAAGACGGAGCCTCCGGATATGTTGGGTCTCCTGGGCT
CTGGTTCGCCTCCCTGCTCCGTCAAGGAGGAGCTGAGTCCCATGATGTGT
AGCATGGCTCCCAATAAAGACCCGATGATGGGGAAGCCGTTTCCCAGTGG
GACGCCCTTCCCTATGGGGATGTACAACCCTCACCAC------CTCCAGG
CCATGTCTAACCCTGGGGTTGGCCATCCT------CACCCCTCCCTGATG
CC-----------C-------------------------------TATCT
CATCTACGCCTCCTTCTCATTCATGGGATGTTTACAGATCAGCGACGGGT
CGAACATTGTCAACCTGCTGGCCAGCAACTCCCCGAGCGTCTCGTATGCG
CTCACGCAGCAGAAATACTTCAGCAACTACAGCCCCGTCATCGGGTTTTA
CATCTATGAACCCATTGAGTACTGGAACTCGACGGTGCAGGAGCACCTGA
AGACACTGAGCCACGGCTTCAACAAGATCTCCTGGATGGACAACTTCTTC
CAGTACCTGCGGGTGGCGAACGTGAGCGCGTCCACCAAGGCTGACTTCGT
CACCATCCTCCGGGGCTCCTTCCTGCGCAGCCCTGAGTACCAGCACTTCA
CTGAGGACATCATCTTCTCCAAGA---ACCGCGAGAGCG------ACGAG
TACGACGTCATCGCCTCCCGCATGTACCTGGTGGCGCGCACCACGGAGAA
GAAACGCGAGGAGGTGGTGGAGCTCCTGGAGAAGCTGCGGCCATTGATGC
TCATCAACAGCATCAAGTTCATTGCCTTCAACCCCACCTTCGTCTTCATG
GACCGTTACAGCTCCTCCGTCGTCTCGCCCATCCTCACCTCTGGCTTCAG
CGTGCTCACCATCCTCATCCTCACCTTCTTCCTCGTCATTAACCCGCTGG
GGAACTTCTGGCTCATCTTGACGGTCACGTCCGTGGAGCTGGGCGTGCTG
GGCCTCATGGGGTACCATCCATTCTAATGGCAGCCAGCTCTTAGGAACGT
GTCACCATCCTGTCATGTGGGTATCATTAATGGGCTCTCTGGATGCACCA
CGTCTGTGGAGGACTCTCCGGTCGACACTATAACCCGTCGTTTTCGCTAT

GATGTGGCCCTGGTCTCAGCCCTGAAAGACTTAGAGGAAGACATCATGAC
AGGGCTGAAAGAGCGTGATCTGGAAGACAGTGTATGCACCTTGGGCTTTA
ATGTTATGATCAAGGAATCATGTGACGGCATGGGAGATGTCAGCGAGAAG
CACGGTGGAGGGCCAGTGATCCCCGAGAAGGCTGTACGTTTCTCCTTCAC
CATCATGTCAGTCTCTGTTCAGCCAGACGGAGAGAGT-------------
-----------------GAAGAAGTCGTCATCTTCAGGGAGACAAAGCCC
AACTCTGAGCTGTCCTGTAAGCCACTATGCCTGATATTTGTGGATGAGTC
TGATCACGAGACACTGACTGCTGTGCTGGGGCCTGTGGTGGCAGAGAGGG
ATGCGATGAAGGACAGCAGACTCATCCTCTCACTAGGAGGCCTCCCCCGC
TCTTTCCGGTTCAGCTTCCGAGGGACAGGCTACGACGAGAAGATGGTGCG
GGAGATGGAAGGCGTGGAGGCCTCAGGCTCCACCTACATCTGCACTCTTT
GCGATGCCACCAGGGCAGAAGCCTCTCGCAACATGCTGCTTCACTCCGTC
ACCCGCAGTCACGAGGAGAACCTGGAGCGCTATGAGCTATGGAGGACCAA
CCCCTTCCAGGAGTCCCCTGCTGAGCTGCGGGACAGGGTCAAAGGGGTCT
CGGCCAAGCCGTTCATGGAGACCCAGCCAACGCTGGACGCATTGCACTGC
GACATCGGCAACGCCACCGAATTCTACAAAATCTTCCAGGACGAGATCGG
GGAGGTGTACCGAAAGAC---CAAC---CCCAGTCGGGAGGAACGGCGCA
GCTGGAGAGCGGCCCTGGATAAACAGCTGAGGAAGAAGATGAAGCTAAAG
CCAGTGATGCGTATGAACGGGAACTACGCCCGGAAGCTGATGACCCTGGA
GGCCGTGGAGGTGGTGTGTGAACTGGTCCCCTCACAAGAGAGACGGGAGG
CCCTGAAGGAGCTGATGGGGCTCTATATTCAGATGAAGCCTGTGTGGCGC
GCCACCTGCCCAGCCAAGGAATGCCCTGACCAGCTGTGCCGCTACAGCTT
TAACTCCCAGCGCTTCGCTGAGCTCCTGTCGTCCACCTTCCAGTACAGGT
ATGACGGAAAGATCACCAACTACCTTCACAAAACTCTGGCCCATGTGCCC
GAAATTATAGAGAGAGACGGCTCCATCGGTGCCTGGGCCAGTGAGGGGAA
TGAGTCGGGGAACAAA----------------------------------

----------------------------NNNNNNN---------AAGTCACAGAACG
AGAAGTCGCTTTGGGGATCAATCCGTTCACAGACGGGATGGGAGCCTTCA
AAATCAACCACAGCTCCCACGACATTGGCTCTGGTGGGCAAGCGGTGTTT
TCCTCCGAGGCT---CGCTGCTAC------TCCGCCGACCCTCAG---CA
CCACCA------CCACCCGACACACGTCAGCTCC---TACTCCACGGCGG
CTTTCAACTCCACCCGGGACTTTCTCTTCAGAAATCGGGGCTTTGGAGAC
GCCACCAG------------CGCTCAGCACAGTCTGTTCGCCTCCGC---
CTGCGGGGGTTT---C------GCAGGGCCACATGGACACTCGGATGCCC
CGGGACACCTGCTCTTCCCGGGGCTGCATGAG---CAAGCCGCCACGCAC
GCCTCCTCCAACGTGGTCAACAGCCAGATGCGCCTGGGGTTTTCAGGGGA
CATGTACGGACGGGCCGAGCAGTACGGTCACGTTACGAGCCCCAGGT---
CCGACCACTATGCCTCGACCCAGCTGCACGGCTATGGCCCTATGAACATG
AACATGGCCGCG---CACCACGGAGCCGGGGCCTTCTTTCGGTACATGAG
GCAACCGATCAAACAAGAGCTGATCTGCAAGTGGATCGAACCGGAGCAAC
TGACGAATCCCAAAAAGTCGTGCAACAAAACTTTCAGCACGATGCACGAA
CTCGTGACCCATCAGACGGTGGAGCACGTTTGTGGACCGGAGCAGGCCAA
CCACATATGCTTGGGGGAGGAATTTGCCCGAGAAGGGAAACCGTTCAAAG
CCAAATACAAACTGGTCAATCACATCAGAGTTCACACCGGAGAGAAACCA
TTCCCNNNNNNNNNNNNNNNNN--------
>Helostoma temminckii

NNNNNNNNNNNNNNNNNNNNNNNNNNNNNNNGCTTGGCATGTTGTTGCTGTCTGATGCCCACCAATGTA
CCAAGCTGTCAGAGCTCTCCTGGGGCATGTGCCTGAGCAACTTCCCTGCT
ATTTGCAAGACAGAGGACTTCCTCCAACTGCCCAAAGATATGGTGGTGCA
GCTTTTGTCACATGAGGAGTTAGAGACAGAAGATGAGAGACTGGTTTATG
AAGCTGCCCTTAACTGGATCAACTATGACCTGGAGAGGAGGCACTGCCAC
CTTCCAGAGCTCCTAAGAACAGTTCGCCTCGCTTTGCTGCCTGCCATCTT
TCTGATGGAGAATGTCTCAACAGAAGAGCTGATTAATGCCCAGCCCAAGA
GCAAAGAGCTGGTGGATGAAGCTATCCGCTGTAAGCTGAAGATCCTGCAA
AATGATGGTGTTGTTAACAGCCCATGTGCTCGACCAAGAAAAACTAGCCA
TGCCCTCTTTCTTCTGGGAGGGCAGACTTTCATGTGTGACAAGTTGTATC
TGGTGGACCAGAAGGCCAAAGAGATCATACCCAAAGCTGACATTCCCAGC
CCCAGGAAGGAGTTTAGTGCCTGTGCCATTGGCTGTAAAGTGTACATCAC
CGGTGGGA--GAGGCTC-TGAGAATGGTGTGTCCAAAGATGTATGGGTGT
ACGACACTGTCCACGAGGAATGGTCCAAAGCAGCTCCCATGCTCATCGCC
AGGTTTGGTCATGGCTCTGCAGAGTTGAAACACTGTCTATACGTAGTAGG
AGGTCACACTGCCGCAACAGGCTGCCTCCCGNNNNNNNNNNNNGGATGAATACATTGTCGTG
TTCAGTCGTTCGACAACAAGGCTGATAATAAATGAAGCAGAGCTTATCAT
GGCGTTAGCACAGGAGTTCCAGATGAGAGTGGTGACAGTATCCCTGGAGG
AACAGTCTTTTCCCAGTATCATCCAGGTGATCAGTGGTGCTTCTATGTTA
GTCAGCATGCACGGAGCTCAGCTTATCACTTCACTCTTCCTCCCAAGAGG
AGCTGCTGTAGTGGAGCTATTCCCATTTGCTGTGAACCCAGAGCAGTACA
CCCCATATAAAACCCTTGCCACCCTTCCAGGCATCGACCTACATTATATT
TCCTGGAGGAACACTAAGGAGGAGAACACCATCACTCACCCAGACAGACC
GTGGGAGCAAGGAGGCATCGCTCACTTGGAGAAGGAGGAACGAGAGCGAA
TACAGGCCAGTAAAGACGTCCCCAGGCACCTGTGCTGCCGCAACCCAGAG
TGGCTCTTCAGGATCTACCAGGACACTTTAGTCGACATCCCTTCCCTTTT
GGAAGTTCTCAA---AGAGGGCATGAAG---GCTAAGCCAAGCTTGAAGA
A---GACAAAGCCATCTGCTGTCGTGCACCCGGGCCGGGTTAGAGAACCC
CAATGTCAGACCTCAGTACAAACCACTAATGAGGCTAAACTCACAGTCTC
CTGGCAGATTCCCTGGAATCTGAAATATCTAAAGGTAAGAGAGGTCAAAT
ACGAAGTGTGGATCCAGAAAAAAGACACCAGCAAGGGGACACTGGAGGAT
CAAATCATCCAGGCCAACCCTGCTCTGGAAGCCTTCGGCAATGCCAAAAC
GGCAAGGAATGACAACTCATCTCGTTTTGGAAAATTCATCCGAATACATT
TTGGTACCAGTGGCAAACTGTCATCTGCTGATATTGAGACGTATCTGCTG
GAGAAGTCACGTGTCACCTTTCAGCTGAAGGCTGAGAGGAACTACCACAT
CTTCTACCAGATTCTGTCCAATCAGAAGCCAGAACTCCTAGACATGCTGC
TGATCACTAACAATCCATATGACTACTCCTACATCTCCCAAGGAGAGGTC
ACAGTTGCCTCCATCAACGACTCTGAGGAGCTGATGGCCACAGACAGCGC
CTTCGATGTGCTCGGCTTCACTCCAGAGGAGAAGATGGGCGTCTATAAAC
TGACCGGTGCCATCATGCACTACGGAAACATGAAGTTCAAACAGAAGCAG
CGTGAGGAGCAGGCTGAACCTGATGGCACGGAGGCTGCTGACAAATCGGC
TTACCTAATGGGGCTGAACTCCGCTGACCTCATCAAAGGTCTTTGCCATC
CCAGAGTCAAAGTAGGAAATGAGTATGTTACCAAAGGCCAAAGTGTGGAT
CAAGTCTACTANNNNNNNNNNNNNNNNNNNNNNNNNNNNNNNNNNNGGAAGCACTACAACACCAAGCTGG
GATATAAGCGCCATG
TGGCCATGCACTCTGCCACAGCAGGTGATCTCACCTGTAAAGTGTGCATG
CAGAGCTATGAGAGTACACCTGTTCTCCTGGAGCACCTCAAGAGCCACTC
TGGGAAGTCTTCAGGTGGGGCCAAGGAGAAAAAACACCCGTGTGACCACT
GTGACCGGCGTTTTTACACGCGTAAGGATGTGAGACGGCACATGGTAGTA

CATACCGGCCGAAAGGACTTCCTGTGCCAGTACTGTGCCCAGCGCTTTGG
CAGGAAGGATCACCTGACACGCCACGTGAAGAAGAGCCACTCACAAGAGC
TGCTGAAGATCAAGACAGAGCCTCCAGATATGTTAGGTCTTTTAGCTTCC
GGGTCGCCACCTTGCTCTGTGAAGGAGGAGCTCAGCCCCATGATGTGCGG
CATGGGTCCCAACAAAGACCCCATGATGGGCAAACCCTTCCCCAGTGGGG
CCCCTTTTCCAATGGGCATGTACAACCCCCACCAT------CTCCAGGCC
ATGTCTACTACTGGGGTGGGTCACCCA------CACCCGTCCCTGATGCC
CAGTTCCTTGTCTGCAGCTATGGCATGGGTTGCNNNNNNNNNNNNNNNNNNNNNNNNNNNNNNNNNNNNN
NNNNNNNNNNNNNNNNNNNNNNNNNNNNNNNNNNNNNTGAACCTGCTGGCTAG
TAACTCACCAAGTGTTTCATATGCTCTGACCCAGCAGAAATACTTTAGTA
ACTACAGTCCGGTTATTGGGTTTTACATTTATGAGCCCATCGAGTACTGG
AACTCCACAGTGCAGGAGCACTTGAAGACTCTAAGTCATGGCTTCAACAA
GATCTCCTGGATGGACAACTTTTTCCATTACCTGCGGGTAGTGAATGTGA
GTGCATCAACCAAGAGCGACTTCATCAACATCCTAAAGGGATCCTTTCTG
CGCAGCCCAGAGTACCAGCACTTCACTGAGGACATCATTTTCTCCAAAA-
--ATCGTGAGACTG------ACGAGTATGATATAATTGCCTCACGGATGT
ACTTGGTGGCACGGACCACGGAAAAGAAGCGTGAGGAGGTAGTGGAGCTT
CTGGAAAAACTTCGTCCACTGATGCTAATTAACAGTATTAAGTTCATTGC
CTTCAATCCCACGTTTGTTTTCATGGACCGCTACAGCTCCTCTGTCATCT
CACCAATCCTGACCTCAGGCTTCAGTGTGCTTACTATCCTCATCCTCACT
TTCTTCCTGGTCATCAACCCCTTGGGAAACTTCTGNNNNNNNNNNNNNNNNNNNNNNNNNNNNNNNNNNN
NNNNNNNNNNNNNNNNNNNNNNNNNNNNNNNNNNNNNNNNNNNNNNNNNNNNNNNNNNNNNNNNNNNNNN
NNNNNNNNNNNNNNNNNNNNNNNNNNNNNNNNNNNNNNNNNNNNNNNNNNNNNNNNNNNNNNNNNNNNNN
NNNNNNNNNNNNNNNNNNNNNNNNNNNNNNNNNNNNNNNNNNNNNNNNNNNNNNNNNNNNNNNNNNNNNN
NNNNNNNNNNNNNNNNNNNNNNNNNNNNNNNNNNNNNNNNNNNNNNNNNNNNNNNNNNNNNNNNNNNNNN
NNNNNNNNNNNNNNNCAGCGAGAAGCACGG
TGGTGGACCAGTTGTTCCTGAGAAGGCTGTACGTTTCTCTTTCACTATTA
TGTCAGTCTCAGTCCTGGCAGATGATGTGGAG------------------
------------GAGCAGGTCACCATCTTCACTGAGCCAAAGCCAAACTC
AGAACTGTCCTGTAAACCCCTTTGCCTGATGTTTGTGGATGAATCAGACC
ATGAGACTCTCACAGCTGTTCTGGGGCCCATTATTGCAGAGCGAAATGCG
ATGAAAGAGAGCAGGCTCATCCTGTCCATTGGCGGCCTGCCTCGCTCATT
CCGCTTCCACTTCAGAGGCACGGGATATGATGAGAAGATGGTACGTGAGA
TGGAGGGCCTGGAGGCCTCGGGGTCCACTTATATCTGCACTTTGTGTGAC
TCGAGTCGGGCCGAGGCCTCTCAAAACATGGTGCTGCACTCCATCACCCG
CAATCATGATGAGAATCTAGAACGATATGAAATATGGAGAAAGAATCCCT
TCTCTGAGTCGGCAGACGAGCTGAGAGACAGAGTCAAAGGGGTCTCTGCC
AAGCCCTTCATGGAGACCCAGCCCACTCTAGATGCATTACACTGTGACAT
TGGCAATGCTACTGAGTTCTATAAAATCTTCCAGGACGAAATTGGGGAGG
TGTATAAAAGGT---CAAC---CCCAGCCGGGAGCAACGGCGCAGCTGG
AGGGCAGCACTAGATAAACAGCTGAGGAAGAAGATGAAGCTTAAGCCGGT
AATGAGGATGAATGGGAACTACGCCCGTAGGCTAATGACCCTGGAGGCTT
TGGAGGTGGTGTGTGAGCTGGTGCCCTCAGAGGAGAGACAGGAGGCTCTG
AGGGAGCTTATGAGGCTCTACCTCCAAATGAAACCTGTGTGGCGCGCCAC
CTGCCCAGCCAAGGAGTGTCCTGACCAGCTATGCCGCTACAGCTTTAACT
CCCAGCGCTTTGCTGACCTTCTCTCCTCTGCCTTTAAGTATAGGTACAAT
GGAAAGATAACCAATTACCTGCACAAGACCCTGGCACATGTGCCTGAAAT
CATAGAGAGAGATGGATCCATAGGGGCCTGGGCCAGNNNNNNNNNNNNNNNNNNNNNNNNNTCATACACC
ATTGA
AATGGGTCCTTTGGGGCCTCAGTGGCAGGAGAACCCAAAGCCTTTCTCCT
GCTCGATTGAAGACCCCACAAAGCAGACAAAGTTCAAGGGCATCAAGACC
TACATTTCATACCGGGTCACGCCAAGCCACACAGGGCGTCCTGTCTACCG
ACGCTACAAACACTTTGACTGGCTGTACAACCGTTTGCTGCACAAGTTCA

CTGTGATCTCTGTGCCTCACCTGCCTGAGAAACAGGCCACAGGGCGATTC
GAGGAAGACTTCATCGAGAAGCGCAAGAGACGACTGATACTGTGGATGAA
CCACATGACCAGTCACCCGGTCCTCTCCCAGTATGAAGGCTTTGAGCACT
TTCTGATGTGTGCTGACGACAAGCAGTGGAAACTGGGCAAGAGACGGGCG
GAGAAGGACGAGATGGTGGGGGCCCATTTTATGCTAACTTTCCAAATCCC
TAATGAACACCAGGATCTTCAGGATGTGGAGGAGCGGGTCGACACCTTCA
AGGCCTTTGCTAAGAAAATGGATGACAGCGTGATGCAGCTCACACATGTT
GCCTCGGAGCTGGTGCGTAAACACCTGGGTGGGTTCAGGAAGGAGTTCCA
GCGATTGGGAAATGCCTTCCAGTCCATTAGCCAGGCCTTCATGCTGGATC
CCCCCCACAGCTCAGAAGCCCTCAACAATNNNNNNNNNNNNNNNNNNNNNNNNNNNNNNNNNNNNNNNNN
NNTGGGTTTCATCATGGGCGTCG
GTGTGGTTGGAAACCTCCTGATCTCCATTCTGCTGGTCAAAGACAAGAGC
CTGCACAGAGCACCCTACTATTTCCTGCTGGACCTGTGCGCTTCAGATAT
CCTGCGCTCAGCCATCTGCTTCCCCTTTGTCTTCACCTCAGTTAAGAATG
GATCTGCCTGGACCTATGGCACGCTGACCTGCAAAGTTATAGCCTTCCTG
GGTGTGCTCTCCTGTTTCCACACAGCGTTCATGCTATTCTGTGTCAGTGT
CACCCGCTACCTGGCCATCGCACATCACCGTTTCTACACCAAGAGGCTGA
CCTTCTGGACCTGTTTGGCTGTCATCTGCATGGTTTGGACGTTGTCAGTG
GCTATGGCGTTCCCGCCAGTGCTAGATGTAGGGACGTATTCTTTTATCCG
GGAGGAGGACCAGTGCACATTCCAGCATCGTTCCTTCAGGGCCAATGATT
CACTGGGCTTCATGCTCCTGCTGGCACTCATCCTCCTGGCCACACAACTG
GTTTACCTCAAGCTCATCTTCTTCGTTCATGACCGTCGAAAAATGAAGCC
CGTCCAGTTTGTGCCTGCTGTAAGCCAGAACTGGACCTTCCATGGACCAG
GCGCCAGTGGGCAGGCAGCGGCCAACTGGCTGGCTGGGTTTGGTCGAGGC
CCCACCCCGCCTACTTTGCTAGGCATCCGGCAGAACAGCAATGCAGCGGG
CCGCAGGCGTCTACTGGTGTTGGATGAATTCAAAACAGAGAAGAGGATTA
GTAGGATGTTCTACATCATGACATTTTTCTTCCTGGCACTGTGGGGTCCC
TATCTGGTCGCCTGCTACTGGCGGGTGTTTGCAAGGGGCCCTGTAGTCCC
TGGGGGATACCTGACAGCAGCCGTGTGGATGAGCTTTGCNNNNNNNNNNNNNNNNNNNNNNNNNNNNNNN
NNNNNNNNNNNNNNNNNNNNNNNNNNNNNNNNNNNNNNNNNNNNNNNNNNNNNNNNNNCGGAGC---GC
AGCGTCCCACTCGGCAACAGCTTGCTATCCCCGCAGCAAACCGAGGAGCC
CACTGTTGCCACCCCCCCGCAGCGATGGTTTGTCACCC---CTGCCAACA
ACCGACTGGACTTTGCTGCCTCGGCATACGACGCCGCT---------GAT
TTCGCCGGTAACGCGGCCACCTTGCTGTCCTACGCAGCGGCCGGAGTGAA
AGCTC------TTCCCCTGCCGACTGCAGGCTGCTCCAACCGGCCTCTTG
GCTATTACGCAGACCCGTCGG---GCTGG---GGAGGACGCACGCCGCCG
CAGTACTGTGGTGTAAATAGCAAATCCACCTCGGTCTTTTCCTGCTGGCC
CACTAACTCTCTCGGTGGCAGAGCGGGCA---CC---AACTACCTGA---
------CCGAGGA---GGGA---GACTC---CATCCCGACAGAGAGGTCA
CCG---AT---CGGCGGTTCAGAGGAG---ACCAAACCTAAAGACATGAC
---ATCTGA---GTCGAGCTGGATAGAG---ACGCCGTCCTCCATAAAAT
CCATCGACTCAAGTGATTCGGGTATCTTTG---AACAGGCCAAAAGAAGA
AGAATCTCACCTTCTGCAACACCG---------GTTTCAGAGACAGTGTC
CCNNNNNNNNNNNNNNNCATCACTCAACAGGTGAAGTCACAGTGAGAGAAGTGGCGTTGGGATA
AATCCGTTTGCCGATGGGATGGGCGCCTTCAAAATAAACCACAGCTGCCA
CGATATTGGCTCCGG---ACAGACGGCGTTTTCCTCCCAGGCA---CCGG
GTTAT---GCAGCAGCCGCCCTGGGA---CACCATCA------CCACCCG
ACCCACGTTGGCTCT---TACTCCACGGCGGCTTTCAACTCCACCAGGGA
CTTTCTCTTCAGAAATCGGGGTTTCGGGGACGCCACCGG-----------
-GGCGCAGCACAGTTTGTTCGCCTC---------CGGAAGTTT---C---
---GCAGGGCCACATGGACACTCAGATGCAGCGGGGCACCTGCTCTTCCC
AGGGCTCCACGAG---CAAGCGGCGAGCCATGCGTCTTCCAACGTGGTCA
ACAGCCAGATGCGGCTGGGCTTCTCGGGGGACATGTACGGACGGGCCGAC

AAGTACGGCCACGTTACAAGCCCACGAT---CCGACCACTATGCTTCAAC
CCAGCTGCACGGCTATGGCCCCATGAACATGAATATGGCCGCA---CATC
ACGGAGCAGGGGCCTTCTTTCGATACATGAGGCAGCCGATAAAACAAGAG
CTCATCTGCAAGTGGATCGAGCCGGAGCAGCTGACAAATCCCAAAAAGTC
GTGCAACAAAACTTTTAGCACGATGCACGAGCTTGTGACCCATCTGACGG
TGGAGCATGTGGGGGGACCAGAGCAGACGAACCATATCTGCTTCTGGGAG
GACTGCTCCGGAGAAGGAAAGCCATTCCAAGCCAAGTACAAACTTGTAAA
TCATATCAAAGTACTCACCGGAGAAAAGCACTTTCCGTGTNNNNNNNNNNNNNNNNNNNNN
>Heteroconger hassi
AGCCTGCTAATTCGGGCAGAACTGAGTCAACCTGGCGCCTTGCTCGGCGA
CGACCAAATCTACAACGTAATCGTTACGGCGCATGCTTTCGTGATGATTT
TTTTTATAGTAATACCCATTATAATCGGAGGCTTTGGAAATTGACTTATT
CCTCTTATAATCGGTGCGCCCGATATGGCCTTTCCCCGGATAAACAATAT
AAGCTTCTGACTACTCCCCCCCTCATTCCTGCTACTGCTAGCATCCTCAG
GTGTAGAAGCCGGGGCTGGCACCGGATGAACCGTATATCCCCCCTTATCG
GGCAATCTCGCCCATGCAGGGGCATCCGTCCATCTTACCATTTTTTCCCT
TCATCTAGCCGGTATTTCATCAATCCTAGGGGCTATTAATTTCATCACCA
CAATTATCAATATAAAACCCCCTGCTATTTCGCAATACCAAACTCCTCTA
TTCGTGTGAGCAGTTTTAATTACCGCAGTTCTTCTATTATTATCACTGCC
TGTCCTCGCAGCAGGTATTACAATGCTTCTCACAGACCGAAACTTAAATA
CCACCTTCTTCGACCCTGCAGGAGGGGGAGATCCCATTCTTTATCAACCC
CTNNNNNNNNNNNNNNNNNNNNNNNNNNNNNNNNNNNNNNNNNNNNNNNNNNNNNNNNNNNNNNNNNNNN
NNNNNNNNNNNNNNNNNNNNNNNNNNNNNNNNNNNNNNNNNNNNNNNNNNNNNNNNNNNNNNNNNNNNNN
NNNNNNNNNNNNNNNNNNNNNNNNNNNNNNNNNNNNNNNNNNNNNNNNNNNNNNNNNNNNNNNNNNNNNN
NN--

---------------TGACGAGTATATTGTTGTCTTCAGCCGCTCACTTA
ACAGGCTAATCCTGAACGAGGCAGAGCTGATCCTGGAGCTAGCGCAGGAG
TTCCAGATGAAGGTGCTCACTGTGTCCCTGGAGGAACAGCCCTTTGCAGA
CATCGTCCGAGTCATCAGCAAGGCGTCTATGTTGGTCAGCATGCACGGGG
CCCAGCTGGTGACCTCTCTCTTCCTTCCTCGTGGTGCCGCTGTGGTGGAA
CTCTTCCCATATGGCGTCAACCCAGAACATTATGCTCCCTACAAGACACT
GACCTCACTGCCAGGCATGGACTTGCAGTATGTGGCTTGGAGGAACACCA
AGGAGGAGAACTCTGTCACCTTCCCTGAGCGTGCCTGGGACCAGGGAGGC
ATTGCACACTTAGAGAAGGAGGAACAGGAACGCATCCTTAAGAGCAAGGA
GGTGCCACGCCACCTATGCTGTCGAAACCCAGAGTGGCTCTTCCGTATCT
ACCAGGACACCAAGGTGGACATTGCGTCCCTACTCGACACACTGCG---T
CAGGGACTGACC---TCCCGGCCAGGGCCCAAGAG---GGCTCGGCCGAG
CAGCACAGTCCACCCAGGCAGGGTGAGGGAGCCCAAGTGCCAGACCTCCG

TCCAAGCCACCAACGAGGCCAAGCTAACCGTGTCTTGGCAGATCCCCTGG
AACCTCNNNNNNNNNNNNNNNNNNNNNNNNNNNNNNNNNNNNNNNNNNNNN-------------------
----

-----------------TACTTGATTTATGCTTCCTTTTCTTTTATGGGA
TGTTTACAAATCAGCGATGGATCGAACATAGTCAACCTCTTGGCCAGCAA
CTCCCCGAGCGTCTCCTACGCTCTGACCCAGCAGAAATACTTCAGCAACT
ACAGCCCYGTGATCGGGTTCTACATCTAYGAACCCATCGAGTACTGGAAC
TCCACCGTTCAAGAGCACCTAAAGACGCTGGGTCATGGRTTCAATAAGAT
TTCATGGATTGACAACTTCTTTCAGCACCTGAAGGTGGTGAATGTCAGTG
CCTCGACCAAAAGAGAGTTCATAACCATYCTCCAGACCTCCTTCTTAAGG
AGCCCAGAGTACCAGCACTTCAAGGACGACATCATATTTTACAAAA---T
AAGGG------------AYGAGACCGAGATCATCGCGTCCCGGATGTACC
TGGTTGCCCGGACGACGGAGAAGACCCGRGAGGAGGTGGTGGAGCTCCTG
GAGAGACTGAGGCCGCTCTCCCTMATCAACAGCATCAAATTCATCGTCTT
CAACCCCACCTTTGTGTTCATGGATCGATACAGCTCCTCGGTCATTTCGC
CCATCCTCACCTCGGGCTTCAGTGTCCTGATCATCCTGATCCTGACCTTC
TTTCTGGTCATCAACCCCCTGGGAAACTTCTGGCTGATACTGACGGTCAC
CTCGGTGGAGCTGGGCGTTCTGGGCCTCATG-------------------

NNNNNNNNNNNNNNNNNNNNNNNNNNNNNNNNNNGGATAAATCCCTTTGCTGAGGGGAGGGGCGCTTTTA
AAAT
CAACCACAGCACTCACGACTTTGGCTATGG---ACAAACGGCGTTTTCCT
CGCAAGCG---CCTGGCTAT------GCCGCTGCCCTGGGA---CACCAT
CA------CCACCCCACCCATGTCAGTTCC---TATTCCACCGCGGCTTT
CAATTCCACCCGGGACTTTCTGTTTCGAAATCGGGGTTTTGGAGACGCAA
CCAG------------CGCACAGCACAGTCTCTTTGCCTCTGC---TGCG
GGAAGTTT---T------GCAGGGCCACATGGACACTCGGATGCAACTGG
GCACCTGCTTTTTCCGGGACTTCACGAG---CAAGCGGCAACYCACGCAT
CTTCCAATGTTGTAAACAGTCAGATGCGCCTGGGCTTTTCTGGGGACATG
TACGGYAGAGCTGAGCAATACAGCCATGTWACCAGTCCCAGAT---CCGA
TCATTATGCTTCGACCCAGTTGCACGGATATGGCCCCATGAACATGAACA
TGGCTGCA---CATCACGGGGCAGGGGCCTTCTTTCGGTACATGAGGCAG
CCAATAAAACAGGAACTCATCTGCAAATGGATCGAACCAGAACAATTGAC
GAACCCGAAAAAGTCCTGCAACAAAACTTTCAGCACAATGCACGAGCTTG
TTACACCTCTCACTGTGGAACACGTCGGGGGGCCAGAACAGTCCAATCAT
GTCTGCTTTTGGGAAGAGNNNNNNNNNNNNNNNNNNNNNNNNNNNNNNNNNNNNNNNNNNNNNNNNNNNN
NNNNNNNNNNNNNNNNNNNNNNNNNNNNNNNNNNNNNNNNNNNNNNNNNNNNNNNN
>Heteromycteris japonica
AGCCTACTCATTCGAGCTGAACTAAGCCAACCGGGAGCCCTGCTAGGTGA
TGATCAAATTTATAATGTTATCGTTACCGCACATGCTTTCGTAATAATTT
TCTTCATAGTAATACCAATTATAATTGGGGGCTTTGGTAACTGACTAATC
CCTCTAATAATTTGTGCCCCGGACATGGCCTTCCCACGAATAAATAACAT
AAGCTTCTGGCTCCTACCACCTTCTTTCCTCCTACTATTAGCCTCCTCAG
GAGTTGAAGCCGGCGCAGGAACTGGCTGAACTGTCTACCCGCCTTTAGCA
GGCAACCTCGCACATGCAGGAGCATCAGTCGATTTAACAATTTTCTCCCT
TCATCTGGCTGGTATTTCCTCAATCCTGGGTGCAATTAATTTTATTACAA
CCATTATTAATATAAAACCAGCAACTATGTCAATATACCAAATCCCACTA
TTTGTATGATCCGTCCTAGTCACAGCCGTACTACTTCTCTTATCACTACC
AGTTCTAGCCGCCGGAATTACAATACTTCTTACGGATCGAAATCTAAACA
CGGCCTTCTTTGACCCCGCGGGAGGAGGAGACCCCATCCTTTACCAACAC
CTT---

------------TTCCTGGAGAGGAACCTTCACTCGTCTAACTGTCTTGG
CATGCTGCTGCTGTCTGATGCCCATCAGTGTACTAAGCTCTCGGAGCTTT
CCTGGGGCATGTGCCTCAGCAACTTCCCTGCTATTTGCAAGACAGAGGAC
TTCCTCCAATTGCCCAAAGATATGGTGGTGCAGCTTTTGTCACACGAGGA

GCTGGAGACAGAGGATGAGAGACTGGTGTATGAAGCTGCTCTAAACTGGA
TCAACTATGACCTAGAAAAGAGGCACTGCCACCTTGCAGAGCTTCTGAGA
ACAGTCCGCCTTGCTCTGCTGCCTGCCATCTTTCTGATGGAGAACGTTTC
AACGGAGGAGTTGATCAATGCCCAGGCCAAGAGCAAGGACCTGGTGGATG
AAGCAATCCACTGTAAGCTGAAGATTCTGCAGAATGATGGGGTTGTTAAC
AGCCCATGTGCTCGACCACGAAAGACCAGCCATGCTCTGTTCCTTCTAGG
AGGACAGACTTTCATGTGTGACAAATTGTACCTGGTCGACCAGAAAGCCA
AAGAGATTATCCCCAAAGCTGACATCCCGAGCCCGAGGAAGGAGTTCAGT
GCCTGTGCGATAGGCTGTAAGGTCTACATCACTGGAGGAA--GAGGCTC-
AGAGAACGGTGTGTCTAAAGACGTGTGGGTCTATGACACCGTCCATGAAG
AATGGTCCAAAGCAGCTCCAATGCTCATCGCCAGGTTTGGCCATGGCTCT
GCAGAGCTAAAGCACTGCCTGTATGTGGTAGGAGGTCACACTGCTGCAAC
CGGCTGCCTCCCGGCATCACCATCGGGAGGACTACATTGTTGTGTTCAGT
CGTTCGACGACGAGGCTCATTCTGAATGAAGCAGAGCTAATAATGGCTCT
GGCCCAGGAACTCCAGATGAGAGTGGTCACCGTGTCACTGGAGGAGCAGT
CTTTTCCCAGTATTGTCCAGGTGATCAGTGGTGCTTCCATTTTAGTCAGC
ATGCATGGAGCCCAGCTTGTCACGTCACTCTTCCTCCCCAGGGGAGCCAC
GGTGGTTGAGCTGTTCCCATTTGCTGTGAACCCAGAGCAGTACACACCAT
ACAAAACACTCGCCTCCCTTCCAGGGATGGACCTTCACTATATCTCGTGG
AGGAACACTAAGGAGGAAAACACCATCACCCATCCAGACAGAGCCTGGGA
GCAAGGAGGCATCGCTCATCTGGAGAAGGAGGAGCAGGAGAGAATCCTGG
CCAGTAAAGAGGTTCCGAGGCACCTGTGTTGCCGCAACCCAGAGTGGCTC
TTCAGGATCTACCAGGACACTTTGGTGGACATCCCTTCCTTGCTAGAAAT
GCTCAA---AGAGGCCAGAAAG---ACCAAGGCCAACTTCAAGAA---AT
CCAAGCCAGCCAGCACAGTTCACCCAGGACGAGTCAGAGAAGCCAAATGT
CAGACCTCGGTACAAACAAGGAACGAGGCTAAACTCACAGTGTCCTGGCA
GATACCNTGGAATCTAAAATNNNNNNNNNNNNNNNNNNNNNNNNNNNNNNNNNNNNNNNNAAAAAAGAC
ACGAGCAAGGGGACCCTGGAGG
ATCAGATCATCCAGGCCAACCCGGCTCTGGAGGCCTTCGGCAACGCCAAG
ACGTCCAGAAACGACAACTCGTCTCGGTTTGGAAAATTCATTCGCATCCA
CTTTGGCACAAGCGGCAAACTGTCCTCGGCAGACATCGAGACATACCTTC
TGGAAAAGTCCCGTGTCACGTATCAGCTGAAGGCTGAAAGGGACTATCAT
ATCTTCTATCAGATCCTGTCCAATCAGAAGCCGGAGCTGCTTGACATGCT
GCTGATCACCAACAACCCCTACGACTACTCCTACATCTCCCAAGGAGAAG
TAACGGTGGCCTCCATCAACGACTCCGAGGAGCTCATGGCTACAGACAGT
GCCTTTGACGTGCTTGGCTTCACCGCAGAGGAGAAGACGGCTGTTTATAA
ACTCACCGGCGCCATCATGCACTATGGAAACCTGAAGTTCAAACAGAAAC
AGCGTGAGGAGCAGGCGGAGCCTGACGGGACGGAGGCTGCTGATAAATCT
GCTTATCTGATGGGCCTGAACTCTGCCGACCTCATCAAAGGCCTGTGTCA
CCCGCGAGTCAAGGTAGGAAATGAATANNNNNNNNNNNNNNNNNNNNNNNNNNNNNNNNNNNNNNNNNAC
AAGAAGGCCTTCAAGTGTGAG
GAGTGTGGGAAGCACTACAACACCAAGCTGGGCTACAAGCGCCACATGGC
CATGCACTCTGCGACCGCCGGGGATCTGACCTGTAAAGTGTGCATGCAGA
GCTACGAGAGCACGCCCGTTCTCCTGGAACACCTCAAGAGCCACTCTGGG
AAATCTTCGGGAGGCGCCAAGGAGAAGAAACACCCGTGTGACCACTGTGA
CCGCCGTTTCTACACCAGGAAGGATGTGAGAAGGCACATGGTGGTCCACA
CGGGACGAAAGGACTTCCTGTGCCAGTACTGTGCCCAGCGCTTTGGCAGG
AAGGACCACCTGACCCGCCACGTGAAGAAGAGCCACTCGCAGGAGCTGCT
GAAGATCAAGACGGAGCCACCAGATATGTTAGGTCTCCTGGCGTCTGGAT
CACCTCCCTGCTCTGTGAAGGAGGAGCTCAGCCCCATGATGTGTGGCATG
GGTCCCAACAAAGATCCCATGATGGGCAAGCCGTTCCCCAGTGGAGCTCC
TTTCCCAATGGGCATGTACAACCCACACCAC------CTCCAGGCGATGT
CCAACACCGGGGTGGGTCACCCT------

CACCCATCCNNNNNNNNNNNNNNNNNNNNNNNNNNNNNNNNNNNNNNNNNNNNNNNNNNNNNNNNNNN
NNNNNNNNNNNNNNTTCATGGAGAGT
TTACAGATCAGTGATGGCTCGAATATAGTTAATCTGCTGGCTAGTAACTC
TCCAAGTGTTTCTTATGCGCTGACTCAGCAGAAATATTTAGTAACTACA
GTCCCGTGATTGGATTTACATTTATGAGCCCATCGAGTACTGGAACTCC
ACGGTGCAGGAGCACCTGAAGACTCTAAGTCATGGTTTCAACAAGATCTC
CTGGATGGATAATTTCTTTCACTATCTGCGGGTGGTGAATGTAAGTGCGT
CAACCAAGGGCGACTTCATTAATATCCTTAAAGGCTCCTTCCTGCGTAGC
CCGGAGTACCAGCACTTCACCGAGGACATCATCTTCTCGAGGA---ACCG
TGAGACCG------ATGAGTACGACATCATTGCCTCACGGATGTACTTGG
TGGCACGGACGACTGAGAAGAAGCGTGAGGAGGTGGTGGAGCTTCTGGAA
AAGCTCCGCCCGCTGATGCTAATCAACAGCATCAAGTTCATTGCCTTCAA
TCCCACGTTTGTGTTCATGGACCGCTACAGCTCCTCCGTCATCTCCCCCA
TCCTGACCTCAGGCTTCAGTGTACTCACCATCCTCATCCTCNNNNNNNNNNNNNNNNNNNNNNNNNNN
NNNNNNNNNNNNNNNNNNNNNNNNNNNNNNNNNNNNNNNNNNNNNNNNNNNNNNNNNNNNNNNNNNNG
TTTGAGTG
GCAGCCAGCTCTGAAAAATGTGTCTCCATCTTGCAATGTTGGCATTATTA
ATGGCTTGTCAGGATGGGCTTCCTCAGTAGACGACTCCCCAGCCGATACC
ATCGCTCGGAGGTTTCGCTATGATGTGGCACTGGTGTCAGCACTAAAGGA
TCTGGAGGAGGACATCATGGAGGGGCTGAGAGAGAGTGGGATGGAAGACA
GCGCTTGCACCTCAGGTTTCCGTGTCACAATCAAAGAATGTTGTGATGGC
ATGGGCGATGTCAGCGAGAAGCACGGCGGAGGCCCTGTTATTCCTGAGAA
GGCAGTTCGTTACTCTTTCACTGTGATGTCTGTCTCGGTCAAAGCTGATG
ATGAGGAG------------------------------AAGGAGGTAACA
ATCTTCACTGAGCCAAAGCCAAACTCAGAACTGTCCTGTAAGCCCCTATG
CTTGATTTTTGTAGATGAGTCAGACCATGAGACACTCACTGCTCTTCTGG
GGCCCGTAGTTGCAGAGCGTAACGCAATGAAAGAGAGTAGGCTCATCTTA
TCCATAGGTGGCATGCCTCGCTTTTTCCAGTTCCACTTCAGAGCCACAGG
ATATGATGAGAAGATGGTACGAGAGATGGAAGGGCTGGAGGCCTCGGGGT
CCGCGTACATCTGCACTCTGTGTGACTCAACTCGGGCAGACGCATCGCAA
AACATGGTGTTACACTCGGTCACACGTTCTCATGAAGAGAACCTAGAACG
TTACGAAATATGGAGAACCAACCCTTTCTCTGAGTCTATAGACGAACTGA
GAGACAGAGTCAAAGGCGTCACGGCCAAACCCTTCATGGAGACCCAGCCC
ACACTCGACGCGCTGCACTGTGATATTGGCAACGCCACTGAGTTCTACAA
AATCTTCCAGGATGAGATAGGGGAGGTGCACCAAAAGGT---CAAC---C
CCAGCCGGGAGGAACGCCGCAGCTGGAGAGCAGCCCTGGATAAACAACTG
AGGAAGAAGATGAAGCTTAAACCAGTAATGAGGATGAATGGGAACTATGC
CCGCAAGCTAATGACCATGGAGGCGGTGGAGGTGGTGTGTGAGCTGGTGC
CATCAGAGGAGAGGAGGGAGGCCCTCAGAGAGCTTATGAGGCTCTACATC
CAGATGAAGCCTGTGTGGCGCGCCACATGCCCAGCAAAGGAATGCCCCGA
CCAGCTGTGCCGCTACAGCTTCAACTCCCAGCGCTTTGCCGATCTCCTCT
CCTCTGCCTTCAAATATAGGTACACTGGGAAGATCACCAATTACCTCCAC
AAGACCCTGGCACATGTGCCGGAAATCGTAGAGAGAGACGGATCCATAGG
CGCCTGGGCCAGCGAGGNNNNNNNNNNNNNNNNNNNNTTGTACCCCATTGAAATGGTCCTGTGGGGCCC
AGGTGGAAGGAAAACCCACTTCCTTTTGCTGCTCCATTGAAGACCCCAC
AAAACAAACAAAGTTCAAGGGCATCAAGACCTACATTTTGTACCGGGTCA
CGGCCAGTCACACAGGACGTTCCGTTTACCGGCGTTACAAACACTTTGAC
TGGCTGTACAACCGCTTACTGCACAAGTTCACTGTGATTTTTGTGCCTCA
CCTGCCCGAGAAACAGGCCCCGGGGCGATTTGAGGAAGATTTCATAGAGA
AGCGTAAGAGACGACTGATTCTGTGGATGAACCACATGACCAGTCACCCG
GTCCTTTTCCAGTATGAAGGCTTTGAACACTTTTTAATGTGTGCTGATGA
CAAGCAGTGGAAAATGGGGAAGAGACGGGCGGAGAAGGACGAGATGGTGG
GTGCACATTTCATGGTGACCCTCCAGATTCCTAACGAGCACCCGGACCTT

CAGGATGTCGAGGAGCGGGTTGACACCTTCAAGGCCTTTGCTAAGAAAAT
GGACGACAGTGTGATGCAGCTTACACATGTTGCCTCAGAGCTTGTGCGTA
AACATCTGGGTGGGTTCAGGAAAGAGTTCCAGCGACTGGGAAATGCCTTC
CAGTCAATCAGCCAGGCATTTATGCTGGACCCTCCCCACAGCTCAGACGC
CCTCAACAAAGCAATCTCCCACNNNNNNNNNNNNNNNNNNNNNNNNNNNNNNNTGGGTTTCATCATTGGA
GTCGGTGTGGT
TGGAAACCTCCTGATCTCCATCTTGCTGGTCAAAGATAAGAGCATGCATC
GAGCGCCATACTATTTCCTGCTGGACCTGTGCGCCTCGGACATCCTGCGC
TCGGCCATCTGTTTCCCCTTCGTCTTCACTTCGGTCAAGAATGGGTCAGC
GTGGACCTACGGTACGTTGACCTGCAAAGTGATCGCCTTCCTGGGTGTGC
TCTCCTGTTTCCACACGGCCTTCATGCTCTTCTGCGTCAGTGTCACCCGC
TACCTTGCCATCGCTCATCACCGTTTTTACACCAAGAGGCTGACGTTCTG
GACGTGTCTGGCCGTCATCTGCATGGTGTGGACACTGTCTGTGGCTATGG
CATTCCCTCCGGTGCTGGATGTAGGGACATACTCTTTCATAAGGGAGGAG
GACCAGTGCACATTCCAGCACCGCTCCTTCAGGGCGAATGATTCGCTGGG
TTTTATGCTCCTGCTGGCGTTGATCCTCCTGGCCACACAGCTGGTTTACC
TCAAGCTCATCTTTTTTGTCCATGACCGCCGAAAAATGAAGCCAGTCCAG
TTTGTGCCTGCCGTCAGCCAGAACTGGACCTTTCATGGGCCAGGTGCCAG
TGGGCAGGCAGCGGCTAACTGGCTGGCTGGGTTCGGTCGTGGCCCCACCC
CGCCAACTTTACTGGGCATCCGGCAGAACAGCAATGCAGCAGGCCGCAGG
CGTCTACTGGTGTTGGATGAATTCAAAACAGAGAAGAGGATTAGTAGGAT
GTTCTACATTATGACGTTTTTCTTCCTGGCGCTGTGGGGGCCCTATCTGG
TCGCCTGCTACTGGCGGGTGTTTGCGAGGGGCCCCGTGGTCCCCGGGNNNNNNNNNNNNNNNNNNNNNNN
NNNNNNNNNNNNNNNNNNNNNNNNNNNNNNNNNNNNNNNNNNNNNNNNNNNNNNNNNN---
----

-----------------NNNNNNNNNACAGGGGAAGTCACAGAGAGAGAAGTGGCGTTG
GGGATAAATCCGTTCGCGGATGGGATGGGCGCCTTCAAAATCAACCACAG
CTCCCACGATATTGGCTCCGG---ACAGACTGCGTTTTCCTCCCAGGCT-
--CCCGGCTAC---GCGGCAGCCGCGTTGGGA---CACCCGCA------C
CATCCGACCCATGTGGGCTCT---TACTCCACGGCGGCGTTCAACTCCAC
CAGGGACTTTCTCTTCAGAAATCGGGGTTTCGGGGACGCCGCCGG-----
-------GGCGCAGCACAGTTTGTTCGCCTC---------CGGAAGTTT-
--C------GCAGGGCCACATGGACACTCAGATGCAGCGGGGCACCTGCT
CTTCCCGGGGCTCCACGAG---CA-G--GCGAGTCATGCCTCCTCCAACG
TGGTCAACAGCCAGATGCGGCTGGGCTTCTCGGGGGACATGTACGGACGC
GCCGACCAGTACGGCCACGTTACAAGCCCGCGCT---CCGACCACTACGC
TTCGACCCAGTTGCACGGCTACGGCCCCATGAACATGAATATGGCCGCT-
--CACCACGGAGCCGGGGCCTTCTTCCGATACATGAGGCAGCCGATCAAG
CAAGAGCTCATCTGCAAGTGGATCGAGCCGGAGCAGCTGACGAGTCCCAA
AAAGTCGTGCAACAAAACTTTTAGCACCATGCACGAGCTCGTGACCCACC

TGACAGTGGAGCATGTGGGGGGACCNNNNNNNNNNNNNNNNNNNNNNNNNNNNNNNNNNN
NNNNNNNNNNNNNNNNNNNNNNNNNNNNNNNNNNNNNNNNNNNNNNNNNNNNNNNNNNNN
NNNNNNNNNNNNNNNNNNNNNNN
>Himantolophus sagamius
AGCTTACTTATCCGTGCCGAACTAAGCCAACCCGGCGCACTTTTAGGAGA
CGACCAGATCTACAACGTAATTGTTACAGCGCATGCCTTTGTAATAATTT
TCTTTATAGTTATACCAATTATAATCGGGGGGTTCGGAAACTGGCTGATT
CCCCTAATGATTGGGGCCCCAGATATGGCATTCCCTCGAATAAATAACAT
AAGTTTCTGGCTACTCCCCCCCTCTTTTCTCCTTCTCCTTGCTTCCTCGG
GAGTAGAGTCCGGAGCAGGAACCGGGTGAACAGTCTACCCCCCACTAGCG
GGCAACCTAGCACATGCTGGGGCCTCTGTAGACCTAACAATTTTCTCCCT
TCATCTAGCAGGGGTGTCCTCAATTCTCGGGGCCATCAACTTTATTACAA
CAATTATTAATATGAAGCCCCCGGCCATCTCTCAATACCAAACCCCCCTG
TTCGTGTGGTCCGTCTTGATCACTGCAGTCCTCCTTTTACTCTCCCTGCC
TGTCCTTGCCGCGGGTATCACCATACTTCTTACCGACCGAAACCTAAACA
CCACTTTCTTCGACCCTGCAGGAGGAGGBGACCCTATCCTCTACCAACAC
CTA---

------------TTCCTAGAGAGAAACCTTCACCCGTCCAACTGCCTCGG
CATGCTGTTGCTGTCTGACGCCCACCAGTGCACCAAGCTGTCGGAGCTCT
CCTGGGGCATGTGCCTCAGCAACTTCCCCGCTATTTGCAAGACAGAGGAC
TTTCTCCAGCTGCCAAAAGATATGGTGGTGCAGCTTTTGTCACATGAGGA
GTTAGAGACTGAAGACGAGAGACTTGTTTATGAAGCTGCCCTCAACTGGA
TCAACTATGACCTGGAAAAGAGGCACTGCCACCTCCCAGAGCTGCTGAGA
ACGGTCCGTCTGGCCCTGCTGCCCGCCATCTTTCTCATGGAGAATGTCTC
GACGGAAGAGCTGATCAATGCCCAGGCCAAGAGCAAGGAATTGGTGGATG
AAGCTATCCGCTGTAAGCTGAAGATCCTGCAGAACGACGGCGTCGTAAAC
AGCCCGTGCGCTCGACCCAGAAAGACCAGCCATGCCCTTTTTCTCCTGGG
CGGTCAGACATTCATGTGTGACAAGTTGTATCTGGTGGACCAGAAAGCCA
AAGAGATCATCCCCAAAGCTGACATTCCCAGCCCCAGGAAGGAGTTCAGC
GCCTGTGCCATCGGCTGTAAGGTATACATCACCGGCGGGA--GGGGCTC-
CGAGAACGGCGTCTCCAAAGATGTCTGGGTCTACGACACAGTCCACGAGG
AATGGTCCAAGGCGGCACCCATGCTCATTGCCCGGTTTGGCCACGGCTCT
GCAGAGCTTAAACACTGCCTGTACGCGGTAGGAGGTCACACCGCCGCCAC
TGGCTGCCTCCCGGCCTCTCCGTCCGGATGAATACATTGTTGTGTTTAGT
CGTTCAACAACGAGACTGATACTGAATGAAGCGGAGCTAATCATGGCATT
GGCTCAGGAGTTCCAGATGAGAGTGGTCACAGTATCCCTGGAGGAACAGT
CTTTCCCTAGTATCATCCAGGTGATCAGCAGTGCTACCATGTTAGTCAGT
ATGCATGGAGCTCAACTTATCACCTCACTCTTCCTCCCCAGAGGAGCTGT
TGTGGTGGAGCTGTTCCCCTTTGCTGTGAACCCAGAGCATTACACCCCAT
ATAAAACCCTTGCCTCCCTTCCGGGCATGGATCTTCATTATATTTCCTGG
AGGAACACTAAGGAAGATAACACCATCACCCACCCAGACAGGCCCTGGGA
ACAAGGGGGTATTGCTCATTTGGATAAGGAGGAGCAAGAACGAATACTGA
TGAGTAAAGATGTGCCCAGGCATCTGTGCTGCCGAAACCCAGAGTGGCTC
TTCCGGATCTATCAGGACACATTGGTGGATATTCCTTCCTTCCTGGAAGT
CCTCAA---AGAGGAAATGAAG---ACAAAGCCCAACTTGAAGAA---GT
CAAAGCTGGCCAGCACACTTCACCCGGGCCGGGTCAGAGAACCTCAGTGT
CAGACTTCAGTTCAAACCAGTAATGAGGCTAAACTCACAGTCTCCTGGCA
GATACCATGGAATCTGAAATACCTGAAGGTCAGAGAGGTGAAATACGAGG
TGTGGATCCAGAAAAAAGACACAAACAAGGGGACCCTGGAGGATCAAATC
ATCCAGGCAAACCCTGCATTGGAGGCCTTTGGCAACGCCAAAACAGTAAG

AAATGACAACTCATCCCGTTTTGGAAAGTTCATCCGAATTCACTTTGGTA
CAAGCGGCAAGCTGTCATCTGCTGACATCGAAACGTACCTGCTGGAGAAG
TCACGCGTTACCTTCCAGCTCAAGGCTGAGAGAAATTATCACATCTTCTT
CCAGATCCTGTCAAATCAGAAACCAGAGCTGCTGGACATGCTGCTCATCA
CCAACAATCCCTACGACTACTCCTACATCTCCCAAGGAGAGGTAACTGTC
GCCTCCATCAACGACTCAGAAGAGCTGATGGCCACGGACAGCGCCTTCGA
TGTGCTCGGCTTCACCCCGGAAGAGAAGATGGGCGTCTATAAACTCATAG
GTGCCATCATGCACTATGGCAACATGAAATTCAAACAAAAGCAGCGTGAG
GAGCAGGCCGAGCCAGATGGGACGGAGGCCGCTGATAAATCCGCTTACCT
CATGGGGCTGAACTCTGCTGACCTCATCAAAGGGCTGTGTCACCCCAGAG
TCAAGGTAGGAAATGAATATGTCACCAAAGGCCAAAGTGTGGATCAAGTC
TACTACCCTAACAAGGAGGCCTTCAAATGTGATGAGTGTGGCAAGCACTA
CAACACCAAGCTGGGATACAAGCGCCATGTTGCCATGCATTCGGCCACGG
CGGGGGATCTCACCTGTAAAGTGTGCATGCAGACCTACGACAGCACACCC
ATGCTCTTGGAGCACCTGAAAAGCCACTCTGGGAAGTCCTCAAGTGGCAC
CAAGGAGAAAAAACACCAGTGCGACCACTGCGACCGGCGTTTCTACACAA
GGAAGGACGTGAGGCGCCACATGGTGGTCCACACAGGCCGAAAGGACTTC
CTCTGTCAGTACTGTGCCCAGCGCTTTGGCAGGAAGGACCATCTGACACG
CCATGTGAAGAAGAGCCACTCGCAGGAGCTGCTGAAAATCAAGACGGAGC
CTCCGGATATGTTAGGTCTTTTAGCTTCTGGGTCACCGCCGTGCTCCGTG
AAGGAGGAGCTCAGCCCCATGATGTGCGGTATGGGTCCCAACAAAGACCC
CATGATGAGCAAACCCTTTCCGAGTGGGGCCCCTTTTCCGATGGGCATGT
ACAACCCTCATCAT------CTCCAGGCCATGTCTAATTCTGGGGTAGGT
CACTCA------CACCCGTCCCTGATGCCCACCTCTTTGTCTGCAGCTAT
GNNNNNNNNNNNNNNNNNNNNTACCTCATCTACGCCTCGTTCTCATTCATGGGATGTTTACAAATCAGT
G
ATGGGTCAAATATCGTGAACCTGCTGGCAAGTAACTCTCCAAGTGTTTCA
TATGCTCTGACACAGCAAAAATACTTCAGTAACTACAGTCCAGTCATTGG
GTTTTACATTTATGAGCCCATCGAGTACTGGAACTGCACAGTGCAGGAGC
ATCTGAAGACTCTGAGTCATGGCTTCAACAAGATCTCCTGGATGGACAAC
TTTTTCCACTACCTGCGGGTGGTGAACGTGAGTGCATCAACCAAGAGCGA
CTTCATCACCGTCCTGAAGGGCTCCTTCCTGCACAGCCCAGAGTACCAGC
ACTTCACTGAGGACATCATTTTCTCAAAGA---ACCGCGAGACCG-----
-ACGAGTACGACATCATCGCCTCGCGGATGTACTTGGTGGCAGAGACTAC
GGAGAACAAGCGTGAAGAGGTGGTGGAGCTCCTGGAGAAACTCCGTCCAC
TGATGCTGATCAACAGCATCAAGTTCATTGCCTTCAATCCTACGTTTGTG
TTCATGGACCGCTACAGCTCCTCTGTCATCTCACCCATCCTGACGTCAGG
ATTCAGCGTACTCACAATCCTCATCCTCACTTTCTTCCTGGTCATCAACC
CCTTGGGTAACTTCTGGCTCATCCTCACTCTAACGTCTATGGAGCTGGGC
GTCTTGGGTTTGATGGGCTTCCACCAGTTTGAATGGCAGCCAGTCCTCAG
GAACGTGTCTACATCTTGCAATGTTGGCATTATTAACGGTCTCTCTGGAT
GGGATTCCTCAGTGGATGACTCCCCAGCTAACACCATCGCTCGACGGTTT
CGCTATGATGTGGCACTGGTATCAGCATTGAAGGATCTGGAGGAGGACAT
CATGGTGGGACTGAGAGAGAACGAAATGGAAGACAATGCTTGCA------
GCTTCACTGTCATGATCAAAGAATCCTGTGATGGCATGGGCGATGTCACT
GAGAAGCACGGTGGAGGGCCAGCTGTCCCCGAGAAGGTCGTGCGTTTCTC
TTTCACCGTTATGTCTGTCTCTTTCCTAGCAGACAGTCAGGAG-------
-----------------------GAAGAGGTCACCGTCTTCACAGAGCCA
AAGCCAAACTCAGAACTGTCCTGTAAGCCCCTTTGCCTGATGTCTGTGGA
TGAAGCAGACCACGAGACACTCACAGCCATCCTGTGTCCTGTAATTGCAG
AGCGCAACGCAATGAAGGAGAGCAGGCTCATCCTGTCCATTGGTGGACTT
CCTCGCTCCTTCCGCTTTCATTTCAGAGGCACAGGATACGATGAGAAGAT
GGTGCGTGAGATGGAGGGCCTCGACGCCTCTGGGTCCACCTACATTTGCA

CGCTCTGTGACTCCAGTCGCGCAACGGCCTCTCAAAACATGGTGCTGCAC
TGCATCACCCGCAGCCATGAGGAGAACCTGGAACGTTATGAAATATGGAG
AACCAACCCATTTTCTGAGTCTGTGGATGAGTTGCGAGATAGAGTCAAAG
GAGTCTCTGCCAAGCCCTTCATGGAGACCCATGCCACGCTGGATGCCTTA
CACTGCGACATCGGCAATGCCACTGAGTTCTACAAAATATTCCAAGATGA
GATTGGGGAGGTGTACCAAAAGGT---CAAT---CCCAGCCGAGAGGAGC
GGCGCAGCTGGAGGACAGCCCTAGATAAACAGCTGAGGGAGAGGATGAAG
CTCAAACCAGTAATGAGGTTGAATGGGAATTACGCCCGCAGGCTAATGAC
CATGGAGGCGGTGGAGGCGGTGTGTGAGCTGGTGCCCTCCGAGGAGAGGA
GGGAGGCCCTGAGGGAGCTCATGAGACTCTACGTCCAGATGAAGCCCGTG
TGGCGCGCCACCTGTCCAGCCAAGGAGTGCCCCGACCAGCTGTGCCGCTA
CAGCTTTAACTCCCAGCGCTTTGCTGAGGTCCTCTCCTCTAAGTTCAAAT
ATCGATACAACGGAAAGATAACCAATTACCTGCACAAGACTCTGGCTCAT
GTGCCTGAAATCATAGACAGAGATGGATCCATAGGAGCCTGGGCCAGCGA
GGGGAATGAGTCGGCAAACAAATTGTACACCATCGAGATGGGCCCCTTGG
GGCCCCAATGGAAGGAGAGCCCACAGCCGTTCTCCTGCTCCATTGAGGAC
CCCACAAAACAGACCAAGTTCAAGGGCATCAAGACGTACATATCGTACCG
GGTGACGCCGAGCCACACGGGGCGTCCCGTCTACAGACGCTACAAACACT
TTGACTGGCTGTACAACCGCTTACTGCACAAGTTCACTGTGATCTCCGTG
CCTCACCTGCCCGAGAAGCAGGCCACGGGGCGATTCGAGGAGGACTTCAT
CGAGAAGCGCAAGAGGCGACTGATACTGTGGATGAACCACATGACCAGTC
ACCCGGTCCTCTCCCAGTATGAAGGGTTCGAGCACTTTCTGATGTGTGCT
GATGACAAGCAGTGGAAACTGGGCAAGAGGCGGGCGGAGAAAGATGAGAT
GGTGGGTGCCCACTTCATGCTGACCCTCCAGATCCCCAAGGAGCACCAGG
ACCTTCAGGACGTTGAGGAGCGCATTGACTCCTTCAAGTCCTTTGCTAAG
AAGATGGACGAGAGTGTGATGCAGCTCACGCATGTGGCCTCGGAGCTGGT
GCGCAAGCACCTGGGTGGGTTCAGGAGGGAGTTCCAGAGGCTGGGAAACG
CCTTCCAGTCCATCAGCCAGGCATTCATGCTGGGCCCTCNNNNNNNNNNNNNNNNNNNNNNNNNNNNNNN
NNNNNNN-----------

--------------------------GCCAAATCTCGCTTTCACCCTGGC
ATGGGGACCGGTCCTGGCACAGAGC---GCAGCGTCCCACTCGGCAACAG
CTTGCTATCCCCGCAGCAAAACGAGGAGCCCACTGTTGCCTCCCCTCCGC
AGCGGTGGTTTGTCACCC---CTGCCAACAACCGACTGGACTTTGCCGCC
TCGGCATACGACGCCGCC---------GATTTCGCCGGTAACGCGGCCAC

CTTGCTGTCTTACGCAGCGGCCGGAGTGAAGGCTC------TTCCGCTGC
CGACTGCGGGCTGCTCCAATCGGGCTCTTGGCTATTACGCAGATGCGTCA
G---GCTGG---GGAGGACGCACGCCGCCGCAATACTGTGGCGTGAACAG
CAAGTCCANCTCGGTCTTTTCCTGCTGGCCCTCCAACACTATCGGAGGCAG
AGCCGGGA---CC---AACTACCTGG---------CCGAGGA---GGGG-
--GACTC---GATCCCCACGGAGAGGTCACCG---AT---CGGCGGCTCG
GAGGAG---GCCAAACCCAAAGACATCAC------------GTCGAACTG
GATGGAG---ACGCCATCATCCATTAAGTCCATTGATTCCAGCGACTCTG
GGATCTTTG---AACAGGCCAAAAGGAGACGGATCTCACCTTCTGCCACG
CCG---------
CTCTCAGACACGGTGTCCCCGTNNNNNNNNNNNNNNNNNNNNNNNNNNNNNNNNNNNNNNNNNNNNNNNN
NNNNNNNNNNNNNNNNNNNNNNNNNNNNNNNNNNNNNNNNNNNNNNAAACCACACCTCCCTC
GATCTTGGCTCAGG---ACAAACGGCGTTTTCCTCCCAGACG---CCCGG
CTAC---GCTGCAGCCGCCCTGGGA---CACCATCA------CCACCCGA
CGCACGTTGGCTCT---TACTCCACGGCCGCTTTCAACTCCACCCGGGAC
TTTCTCTTCAGAAATCGAGGCTTCGGGGACGCCACCGG------------
AGCGCAGCACAGTTTGTTCGCCTC---------CGGCAGTTT---C----
--GCGGGGCCACATGGACACTCGGATGCAGCAGGGCACCTGCTCTTCCCC
GGGCTCCACGAG---CAAGCGGCGAGCCACGCGTCCTCCAACGTGGTCAA
CGGCCAGATGCGGCTGGGCTTCTCGGGGGACATGTACGGACGGGCAGACC
AGTATGGCCACGTCACAAGCCCGCGGT---CCGACCACTACGCCTCGACC
CAGTTGCACGGCTACGGCCCCATGAACATGAATATGGCCGCG---CACCA
CGGGGCCGGGGCCTTCTTTCGATACATGAGGCAGCCGATCAAACAGGAGC
TCATCTGCAAGTGGATCGAGCCAGAGCAGCTGGCCAACCCCAAAAAGTCG
TGCAACAAAACTTTCAGCACGATGCACGAGCTCGTGACGCATCTGACGGT
GGAGCACGTTGGCGGACCGGAGCAGACGAACCACGTCTGCTTCTGGGAGG
ACTGCCCCAGGGAAGGGAATCCNNNNNNNNNNNNNNNNNNNNNNNNNNNNNNNNNNNNNNNNNNNNNNNN
NNNNNNNNNNNNNNNNNNNNNNNNNNNNNNNNNNNNNNNN
>Hime japonicus
AGCCTCTTAATTCGAGCAGAGCTTAGTCAACCAGGGGCCCTTCTTGGAGA
CGATCAAATTTATAACGTAATTGTTACCGCACACGCATTTGTTATAATTT
TCTTTATAGTAATACCAATTATAATTGGGGGTTTTGGCAACTGACTGATT
CCCCTAATGATCGGGGCCCCAGATATGGCGTTTCCTCGAATAAATAACAT
AAGCTTCTGACTTCTTCCCCCCTCTTTTCTGTTGCTACTAGCTTCCTCTG
GCGTTGAAGCTGGAGCCGGAACCGGCTGAACAGTGTACCCCCCCTTAGCT
GGGAACCTGGCACACGCCGGGGCTTCTGTAGATCTGACCATCTTCTCCCT
TCACCTAGCCGGGATCTCCTCAATCCTAGGTGCAATCAACTTTATTACTA
CTATCATTAACATAAAACCCCCCGCGATTTCTCAGTACCAGACGCCCCTT
CTAGTTTGAGCTGTTCTAATCACGGCCGTCCTCCTACTTCTTTCCCTCCC
CGTCCTGGCAGCAGGCATCACTATACTTCTAACAGATCGAAATCTTAATA
CAACATTTTTTGACCCGGCAGGCGGGGGAGACCCAATCCTTTATCAACAT
CTATTCTGATTTTTTGGACACCCAGAGGTTTACATCTTAATTTTACCCGG
GTTTGGGATAATCTCCCATATTGTCGCCTACTACGCCGGTAAGAAAGAGC
CCTTTGGCTACATGGGAATAGTCTGAGCCATAATAGCAATCGGGCTTCTT
GGGTTTATTGTTTGAGCCCACCACATATTTACAGTAGGCATGGATGTCGA
CACACGAGCCTATTCCTTGAGAGGAACCTGCACCCATCCAACTGCCTTGG
CATGCTGCTGTTGTCAGACGCCCACCAGTGCACAAAGTTGTCAGAGCTGT
CCTGGGGTATGTGCCTCAGCAATTTCCCTGCCATCTGCAAGACGGAGGAC
TTCCTCCAGTTGCCCAAAGACATGGTAGTGCAGCTTCTGTCCCATGAGGA
ACTGGAGACTGAAGATGAGAGACTGGTTTACGAGGCTGCCCTCAACTGGG
TCAACTATGACCTTGAGAGGAGACACTGCCACCTGCCAGAGCTGCTGAGA
ACCGTGCGCCTGGCCTTGCTTCCCGCTATCTTCCTGATGGAGAACGTCTC
CACAGAGGAGCTGATCAATGCCCAGGCCAAGAGCAAGGAGCTGGTGGACG

AGGCCATCCGCTGCAAACTGAAGATCCTGCAGAATGACGGAGTGGTCAAC
AGTCCCTGTGCCCGGCCCAGAAAGACCAGCCACGCCCTCTTCCTGCTGGG
AGGACAGACCTTCATGTGTGACAAGCTATACCTGGTGGACCAGAAGGCCA
AAGAGATCATCCCCAAGGCTGACATCCCCAGCCCCAGGAAGGAGTTCAGC
GCCTGTGCCATCGGCTGTAAGGTCTACGTCACAGGCGGGC--GGGGCTC-
AGAGAACGGTGTGTCTAAAGACGTGTGGGTCTACGACACCGTCCATGAGG
AGTGGTCCAAGGCAGCACCCATGCTCATTGCCAGGTTTGGCCACGGCTCT
GCTGAGCTCAAACACTGCCTCTATGTGGTGGGAGGACACACAGCAGCCAC
CGGCTGTCTCCCAGCCTCTCCCTCTAGATGCGTACATTGTCGTGTTCAGT
CGCTCTACAACGAGGCTGATTCTCAACGAAGCAGAGCTAATCCTGGCGCT
GGCCCAAGAGTTTCAGATGAGGGTGGTTACGGTGTCCCTGGAGGAACAAG
CTTTCCCCAGCCTCGTCCAGGTGATCAGCGGGGCCTCCATGTTGGTTAGT
ATGCATGGAGCTCAGCTCGTCACCTCACTCTTCCTCCCTAGAGGAGCTGC
TGTGGTGGAGCTCTTTCCCTATGCTGTGAATCCAGAGCAGTACACCCCGT
ACAAAACCCTAGCCTCCCTGCCAGGCATGGAACTTCAATATGTCTCCTGG
AGGAACACTGTTGAGGAGAACACCGTCGTCCACCCAGACAGGCCCTGGGA
CCAAGGTGGCATTGCCCACTTGGAGAAAGAAGAACAGGAGCGTATCCTAG
CCAGCAAGGATGTCCCCAGGCACTTGTGCTGCCGCAACCCAGAGTGGCTC
TTCAGGATCTACCAGGATACTCTGGTGGACATCCCCTCCTTCATAGAAGT
ACTTAA---AGAGGGCCTGAAG---ACAAGACCGAGCTTGAAGAA---GT
CCAAGCCGGCCAGCACGGTTCACCCGGGCCGAGTCAGAGAACCCCAGTGC
CAGACATCAGTCCAAGCCACAAWCGAGCCTAAGCTCACGGTGTCCTGGCA
GATCCCGTGGAACCTTAAGTACCTGAAGGTGCGAGAAGT-AAGTACGAGG
TGTGATCCAGGAAGAAGGATACAAGTAAGGGAACCCTGGAGGATCAAATC
ATTCAGGCAAACCCTGCACTGGAGGCTTTTGGTAATGCCAAAACACTGAG
GAACGACAACTCCTCCCGTTTTGGGAAATTCATCCGAATTCACTTTGGAA
CCAGTGGGAAGCTGTCCTCTGCGGACATTGAGACTTACCTGCTGGAGAAA
TCACGGGTCACCTTTCAGCTGAAGTCAGAGAGGAACTACCACATCTTCTT
CCAGATCTTGTCCAATCAAAAGCCAGAGCTGTTGGACATGCTGTTGATCA
CCAACAACCCCTATGACTATTCCTACATCTCGCAAGGAGAGGTAACTGTA
GCATCCATCAATGATTCAGAGGAGCTGATGGCCACTGACAGTGCCTTTGA
TGTGCTTGGTTTCACTCAAGAGGAGAAGCTGGGTGTCTACAAGTTGACGG
GGGCTATCATGCACTATGGCAACATGAGGTTCAAGCAGAAGCAACGCGAG
GAGCAGGCTGAACCTGATGGCACAGAGGCCGCTGATAAATCAGCCTACCT
AATGGGGCTGAATTCAGCTGATCTCATCAAAGGACTCTGCCATCCCAGAG
TCAAGGTAGGAAATGAGTATGTTACCAAAGGCCAGGGTGTAGATCAAGTC
TACTACCCCAACAAGGAGGCTTTCAAGTGTGAGGAGTGTGGGAAGCACTA
CAACACCAAGCTGGGATATAAGCGCCATGTGGCCATGCACTCCGCCACCG
CTGGTGACCTCACCTGTAAAGTGTGCATGCAGAGCTATGAGAGCACGCCT
GTTCTCCTGGAGCACCTCAAGAGCCACTCTGGCAAGTCCTCAGGTGGAGC
CAAGGAGAAGAAACACCCATGTGACCACTGTGACCGTCGTTTCTACACAC
GGAAGGATGTGAGAAGACACATGGTGGTCCACACAGGCCGGAAGGATTTC
CTGTGCCAGTATTGTGCCCAGCGCTTCGGCAGGAAGGATCACCTGACACG
CCATGTGAAAAAGAGCCACTCGCAGGAGCTGCTGAAAATTAAGACTGAGC
CTCCTGACATGTTAGGTCTTTTAGCTTCAGGGTCACCGCCTTGTTCTGTG
AAGGAGGAGCTCAGCCCCATGATGTGTGCCATGGGTACCAATAAAGACCC
CATGATGGGTAAAGCTTTCCCTAGTGGGGCCCCATTTTCAATGGGCATGT
ACAACCCCCACCAT------CTTCAGGCCATGTCAAATTCTGGGGTGGCT
CACCCA------CACCCAACCCTGATGCCCGGCTCCCTGTCTGCAGCAAT
GGGCATGGGCTGTCACATGGAGTATCTCATCTACGCCTCWTTCTCMTTCA
TGGGATGTTTACAAATCAGCGACGGGTCAAACATTGTGAACCTGCTGGCC
AGTAACTCTCCGAGCGTYTCGTACGCTCTGACCCAGCAGAAGTACTTCAG
CAACTACAGTCCCGTGATCGGGTTCTACATCTACGAGCCCATCGAGTACT

GGAACTCCACGGTGCAGGAGCACCTGAAGACTCTGAGCCACGGTTTCAAC
AAGATCTCCTGGATGGACAACTTYTTCCACTACCTGCGSGTGGTGAACGT
GAGCGCGTCGACCAAGRCCGACTTCGTCACCATCCTCCGGGGCTCCTTCC
TGCGGAGCCCCGAGTACCAGCACTTCACCGAGGACATCATCTTCTCCAAG
A---ACCGCGAGAGTG------ACGAGTACGACATCATCGCGTCGCGCAT
GTACCTGGTGGCGCGCACCACGGAGAAGAAGCGCGAGGAGGTGGTGGAAC
TCCTGGAGAAGCTGCGGCCGCTGATGCTGATCAACAGCATCAAGTTCATC
GCGTTCAACCCCACGTTCGTGTTCATGGACCGCTACAGCTCCTCGGTCGT
ATCGCCCATCCTCACCTCCGGTTTCAGCGTGCTCACCATCCTCATCCTCA
CCTTCTTCCTGGTCATCAACCCGCTGGGGAACTTCTGGCTCATCCTCACG
GTCACGTCCGTGGAGCTGGGCGTCTTGGGTCTGATGGGCTATCACCCATT
CGAGTGGCAGCCAGCCCTAAAGAATGTGTCCCCATCCTGCTATGTGGGCA
TCATCAATGGGCTCTCTGGATGGGCTGGCACAGTGGATGACTCCCCGGCA
GACACCATCACCCGTCGGTTTCGCTATGACGTGGCCCTGGTGTCAGCCCT
GAAGGACCTGGAGGAGGACATCATGAAGGGACTGAGAGAGCGCAAGCTGG
AAGACCATGCATGCACCTCAGGCTTCAATGTTATGATCAAGGAATCCTGT
GATGGCATGGGAGATGTCAGCGAGAAGCATGGTGGAGGGCCAGCGATCCC
TGAGAAGGCTGTGCGTTTCTCTATTACAATCATGTCTGTCTCTATCCAGG
CAGATGGCGAGGAC------------------------------AAAGAG
GTTGTTATCTTCAGGGAGTCAAAGCCCAACTCTGAACTGTCCTGTAAGCC
CCTATGCCTGATATTTGTGGATGAGTCTGACCACGAGACACTCACTGCTG
TCCTGGGGCCTTTGGTAGCAGAGAGAAATGCAATGAAGGAGAGCCGACTC
ATCCTCTCAGTGGGTGGTCTCCCCCGCTCTTTCCGCTTCCACTTCAGAGG
CTCAGGCTATGATGAGAAGATGGTGCGAGAGATGGAAGGCCTGGAGGCCT
CAGGCTCCACTTATATCTGCACTCTTTGTGACGCCACTAGGGCAGAAGCT
TCTCACAACATGGTGCTCCACTCCGTCACCCGCAGCCACGAGGAGAACCT
GGAGCGCTACGAGTTATGGAGGACCAACCCCTTCTTGGAGTCTGCGGCAG
AGCTGCGAGACCGGGTCAAAGGGGTCTCTGCGAAACCCTTTATGGAGACC
CAGCCGACGCTGGATGCATTGCACTGTGACATCGGTAACGCCACTGAATT
CTACAAAATCTTCCAGGATGAGATCGGGGAGGTGTTCCAAAAGGC---CA
AC---CCTAGCCGGGAGGAACGACGCAGCTGGAGGGCAGCCCTCGATAAG
CAGCTGAGGAAGAAGTTGAAGCTGAAACCAGTGATGCGGATGAATGGGAA
CTATGCCCGGAGGCTGATGACCCAGGAGGCTGTGGAGGTGGTGTGTGAAC
TGGTGCCCTCAGAAGAGAGGAGGAAGGCCCTGAGGGAGCTGATGGGACTC
TATATCCAGATGAAGCCTGTGTGGCGTGCCACCTGCCCAGCCAAGGAGTG
CCCTGACCAGCTGTGCCGGTACAGCTTTAATTCCCAGAGCTTTGCTGAAC
TCCTCTCCTCCTCCTTCAAATATAGGTATGATGGAAAGATCACCAACTAC
CTGCACAAGACCCTGGCCCATGTTCCTGAAATTATAGAGAGAGACGGCTC
CATTGGAGCCTGGGCCAGCGAGGGGAATGAGTCTGCAAACAAG-------

--------------ACAGGCGAAGTCACAGAAAGAGAAGTGGCTTTGGGG
ATAAATCCGTTCGCAGACGGGATGGGCGCCTTCAAAATAAACCACAGCTC
TCATGACATTGGCTCCGG---GCAAACGGCGTTTTCCTCCCAGGCG---C
CCGGCTAC---GCGGCGGCTGCCCTGGGA---CACCACCA------CCAC
CCGACCCACGTTAGCTCT---TACTCCACGGCAGCTTTCAATTCCACTCG
GGACTTTCTCTTCAGAAACCGGGGCTTCGGAGATGCCACCAG--------
----CGCACAGCACAGTTTGTTCGCTTCCGC---CGCGGGAAGTTT---T
------GCAGGGCCACATGGACACTCAGATGCCGCGGGACACCTGCTCTT
TCCTGGGCTTCATGAG---CAAGCCGCGAGCCATGCGTCGTCTAACGTTG
TAAACAGCCAGATGCGGTTGGGCTTTTCAGGGGACATGTACGGGCGGGCC
GAACAGTATGGCCACGTTACAAGCCCAAGGT---CCGACCACTATGCTTC
GGCCCAGTTGCACGGCTATGGCCCTATGAACATGAATATGGCCGCA---C
ACCACGGAGCGGGGGCCTTCTTTCGGTACATGAGGCAGCCGATCAAACAA
GAGCTCATCTGCAAGTGGATCGAACCGGAGCAACTGACGAATCCCAAAAA
GTCGTGCAACAAAACTTTCAGCACGATGCACGAGCTCGTGACCCATCTGA
CGGTGGAGCATGTGGGGGGACCAGAGCAATCGAACCACATTTGCTTCTGG
GAAGACTGTGCTCGAGAAGGAAAACCGTTCAAAGCCAAATACAAACTTGT
GAATCATATCAGAGTTCACACCGGTGAAAAACCGTTCCCCTGTCCATTCC
CAGGCTNNNNNNNN

>Hiodon tergisus
AGCCTCTTAATTCGAGCAGAATTAAGCCAACCTGGGGCCCTACTAGGGGA
TGACCAAATTTACAATGTAATCGTTACAGCACACGCCTTCGTAATAATTT
TCTTTATAGTAATACCAATTATAATTGGTGGCTTCGGAAACTGACTAGTG
CCCCTAATGATCGGGGCCCCAGACATGGCCTTCCCACGAATAAATAACAT
AAGCTTCTGACTTCTTCCTCCATCATTTCTTCTTCTACTAGCCTCCTCAG
GCGTAGAAGCCGGGGCTGGTACAGGTTGAACTGTTTACCCCCCGCTAGCA
GGTAACCTGGCACACGCCGGGGCATCCGTTGACCTAACTATCTTCTCACT
CCATTTAGCCGGGGTTTCTTCAATCCTTGGGGCAATTAACTTTATCACCA
CAATTATTAATATAAAACCCCCTGCCATTTCACAATATCAAACACCGCTA
TTTGTATGAGCTGTACTAATTACCGCAGTCCTCCTGCTCCTATCACTCCC
TGTACTAGCCGCTGGCATTACAATACTTCTTACAGACCGAAATCTAAATA
CAACCTTCTTTGACCCAGCAGGAGGAGGTGACCCAATCCTATACCAACAT
TTANNNNNNNNNNNNNNNNNNNNNNNNNNNNNNNNNNNNNNNNNNNNNNNNNNNNNNNNNNNNNNNNNNN
NNNNNNNNNNNNNNNNNNNNNNNNNNNNNNNNNNNNNNNNNNNNNNNNNNNNNNNNNNNNNNNNNNNNNN
NNNNNNNNNNNNNNNNNNNNNNNNNNNNNNNNNNNNNNNNNNNNNNNNNNNNNNNNNNNNNNNNNNNNNN
NN---

----------------CGAAGAGTACATTGTGGTTTTTAGCCGTTCTGTA
AACAGGCTCATTCTGAATGAGGCAGAGCTGATACTGACTCTGGCTCAAGA
ATTTCAGATGAAGGTCATTACTGTATCCCTTGAGGAGCACTCCTTTTCAG
ACATAATCCAGGCCATCAGTGGGGCATCCATACTGGTCAGCATGCACGGG
GCCCAGCTGGTAACCTCGCTCTTTCTCCCACGTGGCGCAGTTGTGGTCGA
ACTCTTTCCCTACGGGGTCAACCCAGACCACTACACTCCTTACAAAACCC
TGGCCTCCTTACCAGGCATGGACCTTCAGTACGTAGCCTGGAGGAACATC
TTTGAGGAAAATTCTGTGACCTTCCCAGAGCGATCCTGGGAGCAGGGAGG
TATAGCCCACCTGGAAAAAAAAGAACAGGAGCGAATTATGAAAAGCAAGG
AGGTTCCACGGCACCTGTGCTGCCGAAACCCAGAGTGGCTTTTCCGCATC
TACCAGGACACTAAGGTTGACTTATCTTCTCTGCTGGCAGCTCTGAG---
GCAGACTCTAAAA---CCCAAGTCAAATTTGAAGAG---GACCAAGCCAG
CCAGCACAGTCCACCCGGGCAAAGTGAGGGAACCAAAGTGCCAGACTTCA
GTCCAGACCACCAGCGAGGCCAAGATGACAGTGTCCTGGCTGATCCCCTG
GAACCTGAAGTATTTAAAGGTCAGGGATGTCACTTACGAAGTGTGGATAC
AGAAGAAGGACACAAGCAAGGGGACGCTTGAGGACCAGATCATCCAGGCT
AACCCAGCCCTGGAGGCTTTTGGCAATGCCAAAACCTTGAGGAACGACAA
CTCTTCTCGTTTCGGCAAATTCATCCGCATTCATTTTGGAGTGAGCGGCA
AACTGTCTTCTGCCGACATAGAGACATACTTGCTTGAGAAATCCAGAGTG
ACATTTCAGCTCAAGGCAGAGAGGAATTACCACATATTTTATCAGATTCA
GTCCAACAAAAAGCCTGAGCTACTGGACATGCTGCTGATCACCAACAACC

CATATGATTATGCCTACGTCTCCCAAGGAGAGGTGGCGGTTGCCTCCATT
GACGACTCTGAGGAACTGGTGGCCACAGACAGTGCCTTTGATGTACTGGG
CTTCACTGCAGAGGAGAAGATGACTGTCTATAAGCTGACTGGTGCAATCA
TGCACTACGGCAACATGAAGTTCAAGCAGAAACAGCGCGAGGAGCAGGCA
GAGCCTGACGGCACAGAGTCTGCCGACAAGTCAGCTTATCTAATGGGGCT
GAATTCGGCTGACCTGGTGAAAGGGCTGTGTCACCCCAGGGTCAAGGTTG
GCAATGAGTACGTCACCAAGGGCCAAAGTGTGTCACAGGTCTACTACCCT
AACAAGGAAGCTTTCAAGTGCGAGGACTGTGGCAAGAACTACAACACCAA
GCTAGGCTATAAGCGTCACATGGCTATGCATGCCGCCAGCAGCGGCGACC
TCACCTGCAAGGTGTGCCTGCAGAGCTACGAGAGCACACCCGTCCTGCTG
GAGCACYTGAAGAGCCACTCAGGGAAGTCCTCCAGCGGGGCCAAGGAGAA
GAAGCACCCCTGCGACCACTGCGACCGCCGCTTCTACACGCGGAAGGACG
TGCGACGCCACATGGTGGTGCACACCGGACGCAAGGACTTCCTGTGCCAG
TACTGCGCCCAGCGCTTTGGCCGCAAGGACCACCTGACCCGGCACGTGAA
GAAGAGCCACTCACAGGAGCTGCTCAAGATCAAGTCGGAGCCGCCGGATT
TGCTGGGCTTCCTGGGTTCCAGTTCGCCTCCCTGCACTGTCAAGGAGGAG
CTCAGTCCCATAATGTG---CATGGGCCCCTCCAAGGACTCCATGATGGC
CAAGCCCTTCCCCAGTG------CCTTCCCTATGGGCATGTACAACCCCC
AC---------CTGCAGGCTATGTCCAATTCTGGGGCA------------
---------TCCCTGGTGTCTGGTTCGCTCTCTGCAGCCATGGGGATGAG
CTGCCATATGGAGTACCTCATCTACGCCTCCTTTTCTTTCATGGGATGTT
TACAGATAAGCGATGGGTCAAACATTGTCAACCTCTTAGCCAGCAATTCG
CCGAGTGTCTCCTATGCTCTTACCCAGCAGAAATATTTTAGCAACTACAG
TCCTGTGATTGGGTTCTATATTTACGAGCCCATAGAATACTGGAATTCCA
CTGTTCAGGACCACCTAAAAACACTGGGGCATGGGTTCAATAAGATTTCA
TGGATCGACAATTACTTTCAGCATCTGAAGGTAGTGAACATCAGTGCGTC
AACCAAAAGTGATTTCATAACCGTCCTCCAGACTTCTTTCCTGAGGAGCC
CCGAGTATCAGCACTTCAAAGATGACATCATTTTTTCCAAAA---CGGAG
G-------------AAGGATTGAGATCATTGCCTCCAGGATGTACCTTGT
GGCCAGAACCACGGAGAAAACAAGGGAGGAGGTGGTGGAGCTGCTGGAGA
AGCTGAGGCCGCTGTCCCTCATCAACAGCATCAAATTCATAGTGTTCAAC
CCGACCTTCGTCTTCATGGATCGCTACAGCGCCTCTGTAATTTCCCCCAT
CCTCACCTCTGGTTTCAGTGCATTGACAGTCCTAATACTGACGTTCTTTC
TCGTTATCAACCCCCTCGGAAACTTCTGGTTGATTCTGACGGTCACCTCG
GTCGAGCTAGGGGTCCTGGGCCTAATG-----------------------

-------------------------------------TCTTACACTATTGAGA
TGGGGCCGCGGGGGCCCCAGTGGAAAGAGAGCCCCCAGCCGTTTTCTTGC
TCCATAGAGGACCCCACCAAGCAGACCAAATTCAAGGGCATAAAGACATA
CATATCCTACCGGGTGACGCCCAGCCACACGGGCCACCCTGTATACCGCC
GTTACAAGCACTTTGACTGGCTGTACAACCGGCTACTGCACAAGTTTACC
GTCATCTCCGTGCCCCACCTCCCAGAGAAGCAGGCCACCGGGCGCTTCGA
GGAGGACTTCATCGAGAAGCGAAAACGGCGGCTGATCCTCTGGATGGATC
ACATGACCAGCCACCCCGTCCTCTCGCAGTACGAGGGCTTCGAGCACTTC
CTCATGTGTGCCGACGACAAGCAGTGGAAGCTGGGCAAGCGGCGGGCAGA
GAAGGACGAGATGGTGGGCGCCCACTTCATGCTCACCTTCCAGATCCCCA
ACGAGCACCAGGACCTGCAGGACGTGGAGGAGCGGGTGGACACCTTCAAG
GCGTTCGCCAAGAAGATGGACGACAGTGTCCTGCAGCTCACTCACGTGGC
CTCGGAGCTGGTGCGCAAGCACCTGGGCGGGTTCCGCAAAGAGTTCCAGC
GGCTGGGGAATGCTTTCCAGTCCATCAGCCAGGCCTTTACCCTGGACCCA
CCATACAGCGCTGACGCCCTGAACAACGCCATCTCCCACCCCCTTGTCAC
ATTCCTCAAACTGACCTCCCTGGGCTTCATCATTGGTGTTGGCGTGGTTG
GCAACCTCCTGATATCCATCCTGCTCATCAAAGACAAAAGCTTGCACAGA
GCTCCCTACTACTTCCTGCTGGATCTCTGCGCTTCAGACATACTGCGCTC
CGCTATCTGTTTCCCCTTCGTGTTCACCTCCGTCAAAAACGGCTCGTTGT
GGACCTACGGCACACTCACCTGCAAAGTGATCGCTTTCCTGGGAGTCCTC
TCCTGCTTCCACACCGCCTTCATGCTGTTCTGCATCAGCGTGACCCGGTA
CCTGGCAATCGCCCACCACCGCTTCTACACAAAACGGCTGACGTTCTGGA
CCTGCCTGGCCGTCATCTGCATGGTGTGGACGCTGTCAGTGGCCATGGCC
TTCCCCCCGGTGCTGGACGTGGGGACGTACTCCTTCATCAAGGAGGAGGA
CCAGTGTACCTTCCAGCACCGTTCCTTCAGGGCCAACGACTCGCTGGGGT
TTATGTTGCTCCTGGCCCTCATCCTCCTCGCCACGCAGCTTGTCTACCTC
AAGCTAATATTCTTCGTCCATGACCGCAGGAAGATGAAACCAGTTCAGTT
TGTGCCTGCCGTCAGCCAGAACTGGACTTTCCATGGTCCCGGGGCAAGCG
GTCAGGCAGCTGCCAACTGGCTGGCAGGGTTCGGGCGAGGCCCGACGCCA
CCCACCCTGCTGGGGATACGGCAAAACACCAACGCAGCCGGCAGGAGGCG
GCTACTGGTGCTGGACGAGTTCAAAACCGAGAAGAGGATCAGCAGGATGT
TCTATGTCATGACGTTCTTCTTCCTGACCCTCTGGGGGCCCTACTTGATC
GCGTGCTACTGGAGGGTGTTCGCCCGGGGTCTGGCCGTGCCTGGGGGCTA
CCTGACGGCCGCCGTCTGGATGAGTTTTGCCCAGGCGGGGGTCAACCCGT
TCATCTGCATTTTCTCCAACCGAGAGGCCAAATCCCGCTTCCACCCTGGC
GCCGGGACCGGTCCTGGCACGGACC---GCAGCGTCCCACTTAGCAACAG
CTTGCTGTCCCCGCAACAAGCCGAGGAGACCGCAGCGG---CCTCCCCGC
AGCGATGGTTTGTCACCC---CTGCCAACAACCGACTGGACTTTGCCGCG
TCGGCATACGATGCCGCCGCCGCTGCTGATTTTGCTGGTAACGCGGCCAC
CCTGCTGTCTTACGCAGCTGCTGGAGTAAAAGCAC------TTCCACTGC
CCACTGCAGGGTGCTCAGGCAGACCTCTGGGTTATTATGCAGATCCCTCG
G---GATGG---GGCACGCGTACTCCGCCCCAGTACTGC---------AC
TAAATCAAGCTCCGTTCTCCCCTGCTGGCCCTCAAATTCCGTCGGGAGTA
GGACTGGCA---CATCTAACTACGTGG---------TGGACGA---AGGT

```
---GAAAC---CCTGGCAACGGAGCGATCTCCA---CT---GGGTGGAAC
CGAAGAC---GCGAAACCAAAAGACCT------GTCGGA---ATCTAGCT
GGATAGAG---ACACCTTCTTCCATAAAGTCAATTGATTCGAGTGATTCT
GGGATTTTTG---AACAGGCAAAACGGAGACGAATATCACCCTCCGCTAC
GCCT---------GTGTCAGAAACCTCATCCCCACTAAAAAGCGAA----
-----ACAGGCGAAGTCACCGACAGAGAAGTTGCTTTGGGGATAAATCCG
TTCGCTGATGGAATGGGCGCTTTTAAAATTAACCACAGTACCCACGATCT
TGGTTCTGG---GCAAACGGCATTTACTTCGCAGGCG---CCAGGCTAC-
--GCCGCCGCTGCCCTGGA---CATCATCA------TCACCCCACCCAT
GTCAGCTCC---TACTCAACAGCCGCTTTCAACTCAACCCGGGACTTCTT
GTTTCGAAATAGGGGTTTTGGAGACGCAACGAG------------CGCAC
AGCATAGTCTGTTCGCCTCCGC---AGCAGGCGGCTT---T------GCA
GGGCCACATGGACACTCAGATGCCACAGGACACCTGCTCTTCCCGGGACT
TCATGAG---CAAGCAGCTAGTCACGCCACATCCAATGTTGTAAATAGTC
AAATGCGACTGGGCTTTTCTGGAGAAATGTACGGCAGGCCAGAGCAGTAC
GGCCATGTAACAAGCCCCAGGT---CCGATCACTATACTTCTACCCAGTT
ACATAGCTATGGTCCCATGAACATGAATATGGCTGCT---CACCATGGGG
CAGGGGCCTTCTTTCGGTACATGAGACAACCGATAAAACAAGAGCTCATC
TGCAAATGGGTTGAACCGGAACAACTGACGAACCCGAAAAAGTCCTGCAA
CAAAACTTTTAGTACAATGCACGAACTAGTTACTCATCTCACAGTGGAAC
ACGTTGGGGGACCTGAACAGTCGAATCATATTTGTTTTTGGGAAGAGTGT
CCAAGGGAAGGAAAACCTTTTAAAGCCAAGTACAAACTTGTAAACCATAT
TAGAGTGCACACTGGCGAGAAACCGTTTCCATGTCCATTCCCTGGCTGTG
GGAAA
>Histiophryne cryptacanthus
AGTCTACTAATCCGCTCAGAACTAAGCCAGCCGGGCGCATTACTTGGAGA
TGACCAGATTTACAATGTCATCGTTACAGCACACGCCTTTGTAATGATCT
TCTTCATAGTTATGCCGATCATGATCGGGGGCTTTGGCAACTGACTCATC
CCCCTAATAATCGGCGCCCCAGACATAGCCTTCCCCCGAATAAACAACAT
AAGCTTCTGGCTTCTCCCCCCATCTTTCCTCTTACTTCTTGCATCCTCAG
GAGTCGAAGCAGGAGCAGGAACAGGCTGAACTGTTTACCCCCCTCTCGCT
GGCAACTTAGCCCATGCCGGAGCCTCCGTTGACCTCACCATCTTCTCCCT
TCACCTAGCAGGAGTCTCCTCTATCCTGGGAGCAATTAACTTTATTACCA
CCATCATCAACATAAAGCCCCCTGCCATCACACAATACCACACCCCCTTA
TTTGTTTGAGCCGTACTAGTAACCGCAGTCTTACTCCTCCTCTCCCTTCC
AGTTCTCGCTGCTGGAATTACAATGCTCCTCACCGATCGAAACCTGAATA
CCACCTTCTTTGACCCTGTCGGAGGAGGAGACCCCATCCTTTATCAACAC
CTCTTCTGATTTTTTGG---------------------------------
--------------------------------------------------
--------------------------------------------------
--------------------------------------------------
------------TTTCTAGAGAGAAACCTTCACCCATCCAACTGCCTTGG
GATGCTGTTGCTGTCAGATGCACACCAGTGCACCAAGCTATCCGAGCTGT
CCTGGGGCATGTGCCTCAGCAACTTTCCTGCTATTTGCAAGACTGATGAC
TTTCTCCAACTGCCAAAAGATATGGTGGTGCAGCTTTTGTCACACGAGGA
GTTAGAGACTGAAGATGAGAGACTTGTTTATGAAGCTGCCCTCAACTGGA
TCAACTACGACCTGGAAAAGAGGCAGGGTCACCTTCCAGAACTTTTGAGA
ACAGTCCGACTGGCTTTGTTACCTGCTATCTTTCTCATGGAGAACGTCTC
TACAGAAGAGCTGATCAATTCCCAACCCAAGAGCAAGGAATTGGTGGATG
AAGCCATCCGTTGTAAACTGAAGATCTTGCAGAATGATGGTGTTGTTAAC
AGTCCATGTGCTCGACCAAGAAAAACCAGCCATGCTCTTTTTCTTCTGGG
TGGGCAGACATTCATGTGTGACAAGTTGTACCTGGTGGACCAGAAAGCCA
AAGAGATCATCCCAAAAGCTGACATTCCCAGCCCCAGGAAGGAGTTTAGC
```

GCCTGCGCCATTGGCTGTAAGGTGTACATCACTGGAGGGA--GAGGCTC-
AGAGAACGGAGTGTCCAAAGATGTATGGGTCTATGACACGGTTCACGAAG
AATGGTCGAAGGCGGCGCCTATGCTCATTGCCAGGTTTGGCCATGGCTCT
GCAGANNNNNNNNNNNNNNNNNNNNNNNNNNNNNNNNNNNNNNNNNNNNNNNNNNNNNNNNNNNNNNNNN
NNNNNGGATGGATACATTATTGTATTCAGTCGTTCAACGACGAGGCTGAT
CCTGAACGAAGCCGAGCTAATCATGACATTGGCCCAGGAGTTTCAGATGA
GAGTGGTGACATTGTCCCTGGAGGAACAGTCTTTCTCTAGCATCATTCAG
ATGATCAGCAGAGCTGCCATGTTAGTCAGTATGCATGGAGCTCAACTTAT
CACCTCTCTCTTCCTCCCTAGAGGAGCTGTCGTGGTGGAGCTGTTTCCCT
TTGCCGTGAACCCAGAGCAGTACACCCCATATAAAACCCTTGCCTCTCTT
CCAGGCATGGACCTTCATTATATCTCTTGGAGGAACAATTATGAGGACAA
CACCATCACCTACCCAGACCGGCCCTGGGAACAAGGGGGTATTGCTCATT
TGGATAAAGGGGAGCAAGAAAGGATAAAGCTGAGTAAAGAAGTCCCCAGG
CATCTCTGCTGCCGAAATCCAGAGTGGCTCTTCCGGATCTACCAGGACAC
TATGGTGAACATCCCTTCCTTCCTGGAAGTCCTGAA---AGAGGGCATGA
AG---ACAAAACCCAATCTGAAGAA---GTCAAAGCTGGTTAGTGCGCTC
CATCCAGGCAGGGTCAGAGAACCTCAATGTCAGACCTCAGTTCAAACCAG
TAATGAGGCTAAGCTCACAGTCTCCTGGCAGATACCATGGAATCTGAAGT
ACCTGAAGGTCAGAGAGGTGAAATACGAGGTGTGGATCCAGAAAAAGGAC
GCCAACAAGGGGACCCTGGAGGATCAAATCATTCAAGCCAACCCTGCATT
GGAGGCTTTTGGCAACGCAAAAACAGTGAGGAATGACAACTCGTCCCGTT
TTGGGAAGTTCATCAGAATCCACTTTGGGACAAGCGGCAAACTGTCTTCT
GCTGACATTGAGACGTACCTGCTGGAAAAATCACGCGTCACTTTTCAGCT
CAAGGCTGAAAGAAATTATCACGTCTTCTACCAGATTTTGTCAAATCAGA
AGCCGGAGCTGCTGGACCTGCTGCTGGTCACCAACAATCCCTATGACTAC
TCCTACATCTCCCAAGGAGAGGTAACCGTCCCGTCCATCAACGACTCCGA
GGAGCTGATGGCCACAGACAGTGCTTTTGATGTGCTGGGCTTCACTCCAG
ATGAGAAGATGGGCGTCTACAAACTCACAGGAGCCATTATGCATTATGGC
AACATGAAGTTCAAACAAAAGCAGCGTGAGGAGCAGGCCGAACCGGACGG
GACGGAGGCCGCTGATAAATCGGCTTACCTAATGGGGGAGAACTCGGCGG
TGCTCATCAAAGGGTTGTGCCACCCCAGAGTCAAGGTAGGAAATGAATAC
GTCACCAAAGGCCAAAGTGTGGATCAAGTCTACTATCCCAACAAGGAGGC
CTTCAAGTGCGACGAGTGCGGGAAGCACTACAACACCAAGCTGGGATTCA
AGCGCCATGTGGCCATGCACTCTGCCACCGCGGGGGATCTCACCTGTAAA
GTGTGCATGCAGACCTACGAAAGCACACCTGTGCTGCTGGAGCACCTGAA
GAGCCACTCTGGGAAGTCGTCTGGCGGCACCAAGGAGAAGAAGCACCAGT
GTGACCACTGCGATCGGCGTTTCTACACCAGGAAGGATGTGAGGCGCCAC
ATGGTGGTCCACACGGGCAGGAAGGACTTCCTGTGCCAGTACTGCGCCCA
ACGCTTCGGCAGGAAAGACCACCTGACGCGCCACGTGAAGAAGAGCCACT
CACAGGAGCTGCTGAAAATCAAGACTGAGCCTCCGGATATGCTGGGTCTT
TTAGCTTCCGGGTCACCGCCATGCTCCGTGAAGGAGGAGCTCAGTCCGAT
GATGTGTGGTATGGGTCCCAACAAAGACCCCATCATGAGCAAGCCCTTTC
CCAGCGGCGCCCCTTTCCCGATGGGCATGTACAACCCTCATCAC------
CTCCAGGCCATGTCTAATTCAGGGGTGGGCCACTCG------CACCCGGC
CCTGATGCCCGGCTCCTTGTCTGCAGCTATGGGTATGGGCTGTCACATGG
AGTACCTCATCTACGCCTCTTTCTCCTTCATGGGATGTTTACAAATCAGT
GATGGGTCAAATATTGTGAACCTGCTGGCAAGTAACTCTCCGAGTGTTTC
GTATGCGCTGACCCAGCAAAAATACTTCAGTAACTACAGTCCGGTGATTG
GGTTTTATATTTATGAGCCCATTGAGTACTGGAACTCCACAGTGCAGGAG
CACCTGAGGACTCTGAGTCAAGGCTTCATCAAGATCTCCTGGATGGACAA
TTTCTTCCAGTACCTAAAGGTGGTGAATGTGAGTGCTTCAACCAAGAGTG
ACTTCATCAGCATTCTCAAGGGCTCCTTCCTGCGTAGCCCGGAGTACCAG
CACTTCACAGAGGACATCATTTTCTCAAAGA---ACCGTGAGACAG----

--ATGAGTACGACATCATCGCCTCACGGATGTACTTGGTGGCACGGACTA
CGGACAAGAAGCGTGAAGAAGTGATGGTGCTTCTGGAAAAACTTCGACCC
CTGATGCTGATCAACAGCATTAAGTTCATTGCCTTCAATCCTACATTTGT
GTTCATGGACCGCTACAGTTCCTCGGTCATCTCACCCATCCTGACCTCAG
GATTCAGCGTACTCACTATCCTCATCCTCACTTTCTTTCTAGTCATCAAT
CCGTTGGGTAATTTTTGGCTCATTCTCACTGTAACATCTGTGGAGCTGGG
CGTGCTGGGNNNNNNNGGCTTTCACCAGTTTGAATGGCAGCCAGCTCTCAAGAATGT
GTCTACATCTTGCAATGTTGGCATTATTAATGGTCTTTCTGGATGGGACT
CCTCAGTGGATGACTCCCCGGCTGACACCATCACTCGTCGGTTTCGCTAT
GATGTGGCACTGGTGTCAGCATTAAAGGATATGGAGGAGGACATCATAGA
GGGGCTGAGAGAGAATGGAATTGAAGACAGCACTAGCC------ACTTCA
ATGTCATGATCAAAGAGTCCTGTGATGGTATGGGTGATGTCAGCGAGAAG
CATGGTGGAGGACCGGCTGTCCCTGAGAAGGCTGTGCGTTTCTCTTTCAC
TGTCATGTCTGTCTCTTTCAGAGCAGACTGCAAGGAG-------------
-----------------GAAGAGGTCACCATCTTTACAGAATCAAAGCCA
AATTCAGAACTGTCCTGTAAGCCCCTTTGCCTGATGTTTGTGGACGAATC
GGACCACGAGACACTCACAGCCATCCTGCATCCTATAATTGCAGAGCGTA
ATGCGATGAAAGAGAGCAGATTAATCCTATCCATTGGTGGACTTCCTCGC
TCCTTCCGGTTTCATTTTAGGGGCACGGGATATGATGAGAAGATGGTGCG
TGAGATGGAGGGCCTTGAGGCCTCTGGGTCCACCTACATTTGCACCCTCT
GTGATGCCAGTCGTGCAGAGGCCTCTGAAAACATGGTGCTGCATTGTATC
ACCCGCAGTCATGAAGAGAACCTTGAGTGTTATGAAATTTGGAGAACCAA
TCCATTTTCTGAGTCTGTAGAAGAGTTGCGAGATAGAGTCAAAGGGGTCT
CTGCCAAGCCCTTCATGGAGACCCATCCCACAATGGATGCCTTACACTGT
GACATTGGCAATGCCACTGAGTTCTACAAAATATTCCAAGATGAGATTGG
GGAGGTGTACCAAAAGGT---CAAT---CCCAGCCGAGAGGAGAGGCGTA
GCTGGAGGGCAGCCCTAGATAAACAGCTGAGGAAGAAGATGAAGCTTAAA
CCAGTAATGAGGATGAATGGGAACTATGCCCGTAGGCTAATGACCCTGGA
GGCTGTGGAGGTGGTGTGTGAGCTGGTGCCCTCAGAGGAGAGGAGGGAGG
CCCTGAGGGAACTAATGAGACTCTACCTCCAGATGAAGCCAGTGTGGCGC
GCCACCTGCCCGGCCAAGGAGTGCCCTGACCAGCTGTGCCGATACAGCTT
TAACTCCCAGAGCTTTGCAGAGGTCCTCTCCTCTAAGTTCAAATATAGAT
ACAATGGAAAGATAACCAATTACCTGCACAAAACTCTGGCTCATGTGCCT
GAAATCATAGACAGAGATGGATCCATAGGGGCCTGGGCCAGCGAGGGGAA
CGAGTCAGCAAACAAATCCTACACCATCGAGATGGGCCCCTTGGGCCCCC
GATGGAAGGAGAACCCGCAGCCGTTCTCCTGCTCCATCGAAGACCCCACC
AAACAGACGAAGTTCAAGGGCATCAAGACGTACATTTCGTACCGTGTGAC
GCCGAGCCACACCGGGCGTCCCGTCTACAGGCGGTACAAACACTTCGACT
GGCTGTACAACCGCCTGCTGCACAAGTTCACCGTGATCTCCGTGCCGCAC
CTGCCCGAGAAGCAGGCCACGGGCCGATTCGAGGAGGACTTCATCGAGAA
GCGAAAGAGGCGGCTGATACTGTGGATGAACCACATGACCAGCCACCCAG
TCCTCTCCCAGTATGAAGGTTTCGAGCACTTTCTGATGTGCGCCGACGAC
AAGCAGTGGAAACTGGGAAAGAGACGGGCGGAGAAAGACGAGATGGTGGG
CGCTCATTTCATGCTGACCCTCCAGATCCCCAACGAGCACCAGGACCTAC
AGGACGTGGAGGAGCGGATCGACACCTTCAAGTCCTTTGCGAAAAAGATG
GATGACAGCGTGATGCAGCTCACGCACGTTGCGTCGGAGCTGGTGCGGAA
GCACCTGGGCGGATTCAGGAAGGAGTTCCAGAGGCTGGGAAACTCCTTCC
AGTCGATCAGCCAGGCGTTCATGCTGGACCCTCCCCACAGCTCGGAGACC
CTGAACAATGCCNNNNNNNNNNNNNNNNNNNNNNNNNNNNNNNNNNNNNNNNNNNNNNNNNNNNNNNNNNN
NNNNNNNNNNNNNNNNNNNNNNNNNNNNNNNNNNNNNNNNNNNNNNNNNNNNNNNNNNNNNNNGCGCCATA
CTATTTCCTGCTGGACCTGTGCGCCTGCGA
CATCCTGCGCTCCGCCATCTGCTTCCCCTTTGTCTTCACCTCGGTCAAAA
ATGGATCTGCCTGGACCTACGGAACGCTGACCTGCAAAGTGATCGCCTTC

CTGGGTGTGCTTTCTTGTTTCCACACGGCGTTTATGCTGTTCTGCGTCAG
CGTCACGCGCTACCTGGCCATCGCGCATCACCGTTTCTACACCAAGAGGT
TGACCTTCTGGACCTGCCTGGCCGTCATCTGCATGGTGTGGACGTTGTCG
GTGGCCATGGCGTTCCCTCCGGTGCTGGACGTAGGGACGTACTCGTTTAT
CCGCGAGGAGGACCAGTGCACCTTCCAGCACCGCTCGTTCAGGGCGAACG
ATTCGCTGGGCTTCATGCTCCTGCTGGCGCTCATCCTCCTGGCCACACAG
CTGGTTTACCTCAAGCTCATCTTCTTCGTTCACGACCGCCGAAAGATGAA
GCCTGTCCAGTTCGTGCCTGCTGTCAGCCAGAATTGGACCTTCCACGGGC
CAGGCGCCAGTGGGCAGGCAGCGGCCAACTGGCTGGCCGGGTTCGGTCGA
GGTCCAACCCCGCCTACCTTGCTGGGCATTCGGCAGAACAGTAACGCGGC
GGGCCGCAGGCGTCTGCTGGTGTTGGATGAGTTTAAAACGGAGAAGAGGA
TTAGTAGGATGTTCTACATCATGACGTTTTTCTTCCTGGCGCTGTGGGGG
CCCTACCTGGTCGCCTGCTACTGGCGGGTGTTTGCGAGGGGCCCCGTGGT
TCCTGGGGGCTACCTGACGGCGGCCGTGTGGATGAGCTTTGCCCAGGCTG
GGGTCAATCCTTTCATCTGCATCTTCTCCAACAGGGAGGCCAAATCTCGC
TTTCACCCTGGCATGGGGACTGGTCCTGGCACGGAGC---GCAGCGTCCC
ACTCGGCAACAGCTTGCTATCCCCGCAGCAAACCGAGGAGCCCACTGTTG
CCACCCCCCCGCAGCGATGGTTTGTCACCC---CTGCCAACAACCGACTG
GACTTTGCTGCCTCGGCATACGAAGCCGCC---------GATTTCGCCGG
TAACGCGGCCACCTTGCTGTCCTACGCAGCGGCCGGAGTAAAGGCTC---
---TTCCCCTGCCGACTGCAGGCTGCTCCAATCGGCCTCTTGGCTATTAC
GCAGACCCGTCAG---GCTGG---GGGGGACGTACGCCGCCGCAGTACTG
TGGCGTGAATAGCAAATCCAGTTCGGTCTTTTCTTGCTGGCCCTCTAACA
CTATCGGAGGCAGAGTGGGCA---CC---AATTACCTGG---------CT
GAGGA---GGGA---GACTC---TATCCCCACGGAGAGGTCACCG---AT
---CGGCGCGTCGGAGGAG---ACCAAACCCAAAGACATCAC---GTCAG
A---GTCGAATTGGATAGAG---ACGCCGTCCTCTATTAAATCAATCGAT
TCGAGTGATTCTGGAATCTTTG---AACAGGCCAAAAGGAGAAGAATTTC
ACCTTCTGCAACGCCA---------GCCTCAGAGACAGTGTCCCCGTTAA
NNNNNNNNCATCACTCAACAGGCGAAGTCACAGAGAGAGAAGTGGCGTTGGGGATAAA
TCCGTTCGCCGATGGGATGGGCGCCTTCAAAATAAACCACAGCTCTCACG
ATATTGGCTCCGG---ACAAACGGCGTTTTCCTCCCAGACG---CCCGGC
TAC---GCAGCGGCCGCCCTGGGA---CACCACCA------CCACCCGAC
TCACGTTGGCTCT---TACTCCACGGCTGCTTTCAACTCCACCAGGGACT
TTCTCTTCAGAAATAGAGGATTCGGGGATGCCACCGG------------A
GCGCAGCACAGTTTGTTCGCGTC---------CGGGAGTTT---C-----
-GCAGGGCCACATGGACACACAGATGCAGCGGGGCACCTGCTCTTCCCCG
GGCTCCACGAG---CAAGCGGCGACCCACGCGTCTTCCAACGTGGTCAAC
AGCCAGATGCGACTGGGCTTCTCGGGGGACATGTACGGACGGGCCGACCA
ATACGGCCACGTTACAAGCCCGCGGT---CCGACCACTATGCTTCGACCC
AGTTACACGGCTACGGCCCTATGAACATGAATATGGCCGCA---CACCAC
GGAGCCGGGGCCTTTTTTCGATATATGAGGCAGCCGATCAAACAAGAGCT
CATCTGCAAGTGGGTCGAGCCGGAGCAGCTGACAAATCCCAAAAAGTCTT
GCAACAAAACTTTTAGCACGATGCACGAGCTCGTGACCCATCTGACGGTG
GAGCACGTCGGTGGACCGGAGCAGACGAACCACGTCTGCGTCTGGGAAGA
CTGCTCCAGAGAAGGGAAGCCATTCAAAGCCAANNNNNNNNNNNNNNNNNNNNNNNNNNNNNNNNNN
NNNNNNNNNNNNNNNNNNNNNNNNNNNNNNNNNNNN
>Histiopterus typus
AGCCTACTCATTCGGGCAGAACTTAGCCAGCCCGGCGCCCTCTTGGGGGA
CGACCAGATTTATAATGTAATTGTTACAGCACATGCATTTGTAATAATTT
TCTTTATAGTAATACCAATTATGATCGGAGGATTTGGAAACTGACTTCTT
CCCCTAATAATCGGCGCCCCCGACATGGCATTTCCCCGAATAAATAACAT
GAGCTTTTGACTTCTCCCCCCATCATTCCTCCTCCTCCTTGCTTCCTCTG

GGGTTGAAGCTGGTGCAGGTACCGGGTGAACCGTCTACCCTCCCCTAGCT
GGTAACCTAGCCCACGCAGGAGCATCCGTTGACCTCACAATTTTTTCTCT
ACACTTAGCAGGCATCTCCTCAATCCTTGGAGCCATCAACTTCATTACAA
CCATCATTAACATAAAACCTCCCGCTATTTCCCAGTATCAGACCCCCCTC
TTTGTGTGAGCCGTATTAATTACTGCCGTCCTTCTTCTCCTCTCCCTTCC
GGTCCTCGCTGCTGGCATTACAATGCTTCTCACAGATCGAAACCTCAACA
CCACCTTCTTTGACCCCGCAGGAGGAGGAGACCCAATCCTTTACCAGCAC
CTG---

------------NNNNNNNNNNGAAACCTTCACCCATCTAACTGCCTTGGCATGCTGTTG
CTGTCTGACGCTCACCAGTGCACCAAGCTGTCAGAGCTCTCCTGGGGCAT
GTGCCTCAGCAACTTTCCAGCCATTTGCAAGACAGAGGACTTCCTCCAAC
TGCCCAAAGATATGGTGGTGCAGCTTTTGTCACATGAGGAGCTAGAGACA
GAAGATGAGAGACTGGTTTATGAAGCTGCGCTCAACTGGATCAACTATGA
CCTGGAAAAGAGGCACTGCCACCTTCCAGAGCTCCTGAGAACGGTCCGCC
TGGCCCTGCTGCCTGCCATCTTTCTCATGGAGAACGTTTCTACAGAAGAG
CTGATCAATGCCCAGGCCAAGAGCAAGGAGCTGGTGGATGAAGCTATCCG
CTGTAAGCTGAAGATCCTGCAGAATGATGGCGTCGTTAACAGTCCGTGTG
CTCGGCCAAGAAAAACCAGCCATGCCCTCTTTCTTCTGGGGGGGCAGACT
TTCATGTGTGACAAGTTGTACCTGGTGGACCAGAAAGCCAAAGAGATCAT
CCCCAAAGCTGACATTCCCAGCCCCAGGAAGGAGTTCAGCGCCTGCGCCA
TCGGCTGTAAGGTGTACATCACTGGTGGGA--GAGGCTC-AGAGAATGGT
GTGTCCAAAGATGTGTGGGTCTACGACACCGTCCACGAGGAATGGTCGAA
GGCGGCACCCATGCTCATTGCCAGGTTTGGCCATGGCTCTGCGGAGCTGA
AACACTGCCTGTACGNNNNNNNNNNNNNNNNNNNNNNNNNNNNNNNNNNNNNNNNNNNNNNNNGGATG
AATACATTGTTGTGTTTAGTCGTTCAACAA
CGAGGTTGATACTGAATGAAGTGGAGCTAATCATGGCGCTGGCCCAGGAG
TTCCAGATGAGAGTGGTCACGGTATCCCTGGAGGAACAGTCTTTCCCCAG
TATCGTCCAGGTGATCAGCGGTGCTTCCATGTTAGTCAGTATGCATGGAG
CTCAGCTTATCACCTCACTCTTCCTCCCTAGAGGAGCTGCTGTGGTGGAG
CTGTTCCCCTTTGCTGTGAACCCAGAGCAGTACACCCCGTATAAAACCCT
TGCCTCCCTTCCAGGCATGGACCTTCACTATGTATCCTGGAGGAACACTA
AGGAGGAGAACACCATCACCCACCCAGACAGACCCTGGGAACAAGGGGGC
ATTGCTCACTTGGAGAAGGAGGAGCAAGAGCGAATACTGGCGAGCAAAGA
TGTCCCCAGGCACCTGTGCTGCCGCAACCCAGAGTGGCTCTTCCGGATCT
ACCAGGACACTTTGGTGGACATCCCTTCCTTCCTGAAAGTCCTCAA---A
GAGGGCATGAAG---ACAAAACCCAGCTTGAAGAA---GTCAAAGCCGGC
CAGCACAGTCCATCCGGGCCGGGTCAGAGAACCCCAATGTCAGACCTCAG
TACAAACCACTAATGAGGCTAAACTCACAGTCTCCTGGCAGATCCCGTGG
AATCTGAAATACCTGAAGGTGAGAGAGGTCAAATACGAGGTG--------
-AAAAGAGACACCAGTAAGGGGACACTGGAGGATCAAATCATCCAGGCGA
ACCCTGCACTGGAGGCCTTCGGCAACGCCAAAACATTGAGAAATGACAAC
TCGTCTCGTTTTGGAAAATTCATCCGAATTCACTTCGGTACAAGCGGCAA
GCTGTCGTCTGCTGACATCGAGACGTACCTGCTGGAGAAGTCACGTGTCA
CCTTTCAGCTCAAGGCTGAGAGGAACTACCACATCTTCTACCAGATCCTG
TCCAATCAGAAGCCAGAGCTCCTGGACATGCTGCTGATCACCAACAACCC
GTACGACTACTCCTACATCTCCCAAGGAGAGGTAACGGTCGCCTCCATCA
ACGACTCAGAGGAGCTGATGGCCACCGACAGCGCCTTCGATGTGCTCGGC
TTCACTCCAGACGAGAAGATGGGCGTCTATAAACTGACCGGCGCCATCAT
GCACTACGGCAACATGAAGTTCAAACAGAAGCAGCGTGAGGAGCAGGCGG
AGCCGGACGGGACGGAGGCTGCTGATAAATCAGCTTACCTAATGGGGCTG

AACTCTGCTGACCTCATCAAAGGGCTGTGCCATCCCAGAGTCAAGGTAGG
AAATGAATACGTCACCAAAGGCCAAAGTGTGGATCAAGTCTACTACNNNNNNNNNNNNNNNNNNNNNN
NNNNNNNNNGGGA
AGCACTACAACACCAAGCTGGGATATAAGCGCCATGTGGCCATGCACTCT
GCCACGGCAGGGGATCTCACCTGTAAAGTGTGCATGCAGACCTACGAGAG
CACACCCGTGCTCTTGGAGCACCTCAAGAGCCACTCCGGGAAGTCTTCGG
GTGGCACCAAGGAGAAAAAACACCCGTGCGACCACTGTGACCGTCGTTTC
TACACGCGGAAGGATGTGAGACGGCACATGGTAGTCCACACGGGCCGAAA
GGACTTCCTGTGCCAGTACTGTGCCCAGCGCTTCGGCAGGAAGGACCATC
TGACACGCCACGTGAAGAAGAGCCACTCGCAGGAGCTGCTGAAGATCAAG
ACGGAGCCTCCTGATATGTTAGGTCTTTTAGCGTCGGGGTCACCACCCTG
CTCTGTGAAGGAGGAGCTCAGCCCCATGATGTGCGGCATGGGTCCCAACA
AAGACCCCATGATGGGCAAACCGTTCCCCAGTGGGGCCCCCTTCCCGATG
GGCATGTACAACCCCCACCAT------CTCCAGGCCATGTCTAATTCTGG
GGTGGGTCACCCA------CACCCGTCCCTGATGCCCAGTTCCTTGTCTG
CAGCTATGGGCATGGGCTGCCACANNNNNNNNNNNNNNNNNNNNNNNNNNNNNNNNNNNNNNNNNNNNN
NNNNNNNNNNNNNNNNNNNNNGTGAATCTGCTGGCTAGTAACTCTCC
GAGTGTTTCGTATGCTCTGACCCAGCAGAAATACTTCAGTAACTACAGTC
CCGTGATTGGGTTTTACATTTACGAGCCCATCGAGTACTGGAACTCAACG
GTGCAGGAGCACCTGAAGACTCTGAGTCATGGATTCAATAAGATCTCCTG
GATGGACAACTTTTTCCACTACCTGCGGGTGGTGAATGTGAGTGCGTCAA
CCAAGAGCGACTTCATCACCATCCTCAAGGGGTCCTTCCTGCGCAGCCCG
GAGTACCAGCACTTCACTGAGGACATCATATTCTCCAAGA---ACCGCGA
GACTG------ATGAGTACGACATTATCGCCTCACGGATGTACTTGGTGG
CGCGGACGACAGAGAAGAAGCGTGAAGAGGTGGTGGAGCTTCTGGAGAAG
CTTCGTCCGTTGATGCTGATCAACAGCATCAAGTTCATTGCCTTCAATCC
TACGTTTGTGTTCATGGACCGCTACAGCTCCTCTGTCATCTCGCCCATCC
TGACCTCAGGCTTCAGCGTACTCACAATCCTCATCCTCACTTTCTTCCTG
GTCATCAACCCCTTGGGAAACTTCTGNNNNNNNNNNNNNNNNNNNNNNNNNNNNNNNNNNNNNNNNNN
NNNNNNNNNNNNNNNNNNNNNNNNNNNNNNNNNNNNNNNNNNNNNNNNNNTCTTGCAATGTTGGCAT
TATTAAT
GGGCTCTCTGGATGGGCTTCCTCGGTGGATGACTGCCCAGCTGACACCAT
CACTCGTCGGTTTCGCTATGATGTGGCACTGGTGTCAGCAATAAAGGATC
TGGAGGAGGACATCATGGAGGGGCTGAGAGAGAGTGGGATGGAAGACAGT
ACGTGCACCTCGGGCTTCAGTGTCATGATCAAGGAATCTTGTGATGGCAT
GGGCGATGTCAGCGAGAAGCACGGTGGAGGACCAGTTGTTCCTGAGAAGG
CGGTACGTTTCTCTTTCACTGTTATGTCTGTCTCTGTCCTGGCAGACGAT
GAGGAA------------------------------GAAGAGGTTACCAT
CTTCACAGAGCCAAAGCCAAACTCAGAACTGTCCTGTAAGCCCCTTTGCC
TGACCTTTGTGGATGAGTCAGACCATGAGACACTCACAGCCGTCCTGGGG
CCTATAGTTGCAGAGCGTAATGCAATGAAAGAGAGCAGGCTCATCCTATC
CGTGGGTGGACTACCTCGCTCCTTCCGCTTTCACTTCAGAGGCACAGGAT
ACGATGAGAAGATGGTGCGTGAGATGGAGGGCCTCGAGGCCTCAGGGTCC
TCCTATATCTGCACTCTTTGTGACTCCAGTCGGGCAGAAGCCTCTCAAAA
CATGGTGCTACACTCGGTCACCCGCAGTCATGAAGAGAACCTAGAACGTT
ACGAAATATGGAGAACCAACCCCTTTTCTGAGTCTGTAGATGAGCTGCGA
GACAGAGTCAAAGGGGTCTCTGCCAAGCCCTTCATGGAGACCCATCCCAC
GCTGGATGCATTACACTGTGACATTGGCAATGCCACTGAGTTCTACAAAA
TCTTCCAGGACGAGATCGGGGAGGTGTACCAAAAGGT---CAAC---CCC
AGCCGGGAGGAACGGCGGAGCTGGAGGGCAGCCCTAGATAAACAACTGAG
GAAGAAGATAAAGCTTAAACCGGTAATGAGGATGAATGGGAACTTTGCCC
GCAGGCTAATGACCCAGGAGGCTGTGGAGGTAGTGTGCGAGCTGGTGCCC
TCAGAGGGAAGGAGGGAGGCCCTGAGGGAGCTTATGAGGCTCTACCTCCA

GATGAAGCCTGTGTGGCGCGCCACCTGCCCAGCCAAGGAGTGCCCCGACC
AGCTGTGCCGCTACAGCTTTAACTCCCAGCGCTTTGCCGACCTCCTGTCC
TCTACCTTCAAATATAGGTACAATGGAAAGATAACCAATTACCTGCACAA
GACCCTGGCCCATNNNNNNNNNNNNNNNNNNNNNNNNNNNNNNNNNNNNNNNNNNNNNNNNNNNNNNNN
NNNNNNNNNNNNNNNNTCGTACACCATCGACATGGCCCCTTGGGTCCCCGGT
GGAAGGAGAGCCCACAGCCTTTCTCCTGCTCCATTGAAGACCCCACAAAA
CAGACAAAGTTCAAGGGCATCAAGACGTACATTTCGTACCGGGTCACGCC
GAGCCACACAGGGCGTCCCGTCTACAGGCGCTACAAACACTTTGACTGGC
TGTACAACCGCTTGCTGCACAAGTTCACTGTGATCTCCGTGCCTCACCTG
CCTGAGAAGCAGGCCACGGGGCGATTTGAGGAAGACTTCATCGAGAAGCG
TAAGAGGCGACTGATACTGTGGATGAACCACATGACCAGTCACCCAGTCC
TCTCCCAGTATGAAGGCTTTGAGCACTTTCTGATGTGCGCTGATGACAAG
CAGTGGAAACTGGGCAAGAGACGGGCGGAGAAGGACGAGATGGTGGGTGC
CCATTTCATGCTGACCCTCCAGATCCCTAATGAGCACCAGGACCTTCAGG
ATGTAGAGGAGCGGGTCGACTCCTTCAAGGCCTTTGCTAAGAAAATGGAT
GACAGCGTGATGCAGCTCACGCACGTTGCCTCGGAGCTGGTGCGTAAGCA
CCTGGGTGGGTTCAGGAAGGAGTTCCAGCGGCTGGGAAATGCCTTCCAGT
CTATCAGCCAGGCCTTCATGCTGGACCCTCCCCACAGCTCAGAAACCTTC
AACAACGCCATCTCCCAT--------------------------------

-----NNNNNNNNNNNNNNNNNNNNNNNNNNNNNNNNNNNNNNNNNNNCGGAGC---
GCAGCGTCCCACTCGGCAACAGCTTGCTATCCCCGC
AGCAAACCGAGGAGCCCACTGTTGCCACCCCCCCGCAGCGATGGTTTGTC
ACCC---CTGCCAACAACCGACTGGACTTTGCTGCCTCGGCATACGACGC
CGCT---------GATTTCGCCGGTAACGCGGCCACCTTGCTGTCCTACG
CAGCGGCCGGAGTGAAGGCTC------TTCCCCTGCCGACTGCAGGCTGC
TCCAACCGGCCTCTTGGCTATTACGCAGACCCGTCAG---GCTGG---GG
AGGACGCACGCCGCCGCAGTACTGTGGTGTAAATAGCAAATCCAGCTCGG
TCTTTTCCTGCTGGCCCGCTAACTCTATCGGTGGCAGAGCAGGCA---CC
---AACTACCTGG---------CGGAGGA---GGGA---GACTC---CAT
CCCGACAGAGAGGTCACCG---AT---CGGCGGCTCGGAGGAG---ACCA
AACCCAAAGACCTGAC---ATCAGA---GTCGAGCTGGATAGAG---ACG
CCGTCCTCCATTAAGTCAATCGATTCAAGCGATTCTGGTATCTTTG---A
ACAGGCCAAAAGGAGAAGAATCTCACCTTCTGCCACGCCG---------G
TTTCAGAGACAGTGTCCCCNNNNNNNNNNNNNNNNNNNNNNNNNNNNNNNNNNNNNNNNNNNNNNNNNNN

NNNNNNNNNNNNNNNNGTTCGCGGATGGGAAGGGCGCCTTCAAAATA
AACCACAGCTCCCACGATATTGCCTCCGG---ACAAACGGCGTTTTCCTC
CCAGGCG---CCCGGCTAC---GCAGCAGCCGCCCTGGGA---CACCATC
A------CCACCCGACCCACGTTGGCTCT---TACTCCACGGCGGCTTTC
AACTCCACCAGGGACTTTCTCTTCAGAAATCGGGGTTTCGGGGACGCCAC
CGG------------GGCGCAGCACAGTTTGTTCGCCTC---------CG
GAAGTTT---C------GCAGGGCCACATGGACACTCAGATGCAGCGGGG
CACCTGCTCTTCCCAGGGCTCCACGAG---CAAGCGGCGAGCCATGCGTC
TTCCAACGTGGTCAACAGCCAAATGCGGCTGGGCTTCTCGGGGGACATGT
ACGGACGGGCCGACCAGTACGGCCACGTTACAAGCCCGCGGT---CCGAC
CACTACGCTTCGACCCAGCTGCACGGCTATGGCCCCATGAACATGAATAT
GGCCGCA---CACCACGGAGCAGGGGCCTTCTTTCGATACATGAGGCAGC
CGATCAAACAAGAGCTCATCTGCAAGTGGATCGAGCCGGAGCAGCTGACG
AATCCCAAAAAGTCGTGCAACAAAACTTTTAGCACGATGCACGAGCTTGT
GACCCATCTGACGGTGGAGCATGTGGGGGGACCGGAGCAGACCAACCACA
TCTGCTTCTGGGAGGANNNNNNNNNNNNNNNNNNNNNNNNNNNNNNNNNNNNNNNNNNNNNNNNNNNNNN
NNNNNNNNNNNNNNNNNNNNNNNNNNNNNNNNNNNNNNNNNNNNNNNNNNNNN
>Holtbyrnia latifrons
AGCCTCCTAATTCGAGCTGAATTAAGCCAGCCCGGGGCCCTTCTGGGCGA
CGACCAGATTTATAATGTTATCGTCACAGCACATGCCTTCGTAATGATCT
TCTTTATAGTAATACCAATCATGATTGGGGGCTTTGGAAACTGGCTGCTC
CCCCTAATACTGGGGGCCCCCGACATAGCATTTCCCCGAATAAATAACAT
GAGCTTCTGACTCCTCCCCCCCTCCCTCCTCCTCCTGCTGTCATCTTCCG
GGGTTGAAGCCGGAGCCGGAACAGGGTGAACCGTCTATCCCCCGCTTGCC
GGCAACCTTGCCCACGTCGGTGCCTCTGTAGACCTCGCTATTTTCTCCCT
TCACCTAGCGGGTGTTTCCTCAATCCTGGGGTCAATTAATTTTATTACCA
CAATTATCAACATGAAGCCCCCTGCCATCTCCCAGTACCAAACACCTTTA
TTTATCTGATCAGTCCTCGTCACAACAGTCCTCCTCCTCCTGTCACTCCC
AGTCCTGGCCGCAGGTATTACCATGCTCCTCACAGACCGAAACCTTAACA
CAACATTCTTTGACCCGGCTGGAGGAGGGGACCCCATCCTATACCAACAC
CTATTCTGATTCTTTGGCCACCCAGAAGTCTACATCTTAATCTTGCCCGG
ATTTGGCATCATCTCACACGTTGTTGCCTACTACGCCGGCAAAAAAGAGC
CATTTGGCTACATGGGCATGGTCTGAGCCATGATAGCCATTGGACTCCTG
GGCTTCATTGTATGAGCCCACCACATATTTACCGTGGGAATAGACGTAGA
CACCCGTGCCTN---------------------------------------

------------------------GGACGAGTACATCGTGGTTTTCAGCC
GCTCCGTCAACCGGCTCATCCTCAACGAGGCCGAGTTGATCCTGGCTCTG
GCGCAAGAGTTCCAGATGAGGGCCGTCACGGTGTCCCTGGAGGAGCAGTC

GTTCGCCAGCATCGTTCAGGCGATCAGCGGGGCGTCCATGCTGGTCAGCG
TCCATGGAGCCCAGCTGATCAGCTCCCTGTTCCTGCCGCGTGGCGCTGCC
GTGGTGGAGCTCTTCCCCTACGCCGTCAACCCGGAGCAGTACACTCCGTA
CAAAACCCTGGCTCTGTTACCAGGCATGGACCTCCAGTACGTGGCTTGGA
GGAACACCATGGAGCAGAACTCGGTGGCCTACCCCGAGCGCGCCTGGGAC
CAGGGTGGCATCGCCCACCTGGAGAAGGAGGAGCAGGAGCGCATCCTGGC
GAGCGACGAGGTGCCGCGGCACCTGTGCTGCCGCAACCCGGAGTGGCTCT
TCCGGATTTACCAGGACACCCAGGTGGACGTCCCCTCCTTGCTGGAGGTC
CTCCG---AGAAAATCTGAAG---GCCAAGCCTAACCTGAGAAA---GGC
CAAGGCAGCTAGTACAGTACACCCCGGTAGAGTCAGGGAGCCCAAGTGCC
AGACCTCGGTCCAGGCCACCAACGAGGCCAAGCTGACAGTGTCGTGGCAG
ATCCCCTGGAACCTCAAATACCTGAAGGTCCGGGAGGTCAAA--------
----------AAGAAGGATGCCAGCAAAGGGACCTTGGAGGATCAAATCA
TTCAAGCTAACCCAGCACTGGAGGCTTTTGGCAATGCCAAAACAGTGAGA
AATGACAACTCCTCACGCTTTGGGAAGTTCATTCGTATTCATTTTGGAAC
GAGCGGCAAACTCTCCTCTGCTGACATAGAAACTTACTTGCTTGAGAAAT
CCCGTGTGACCTTTCAGCTCAAATCGGAGAGGAACTACCACATCTTCTTC
CAGATATTGTCCAATGAAAAGCCAGAGCTGCTGGACATGCTGTTGATTAC
CAACAACCCTTATGATTATTGCTTCATCTCCCAAGGAGAAGTAACAGTTA
AATCTATCAATGACAGTGAGGAGTTGCTTGCCACTGACAGTGCCTTTGAT
GTGCTTGGCTTCACTCCAGAGGAGAAGATGGGGATCTATAAGTTGACAGG
TGCCATTATGCACTATGGCAACATGAAGTTCAAGCAGAAGCAGCGTGAGG
AGCAGGCAGAGCCTGATGGCACTGAGGCAGCTGACAAGTCAGCCTACCTG
ATGGGGCTTAACTCTGCTGATCTTGTGAAGGGACTCTGCCATCCCAGGGT
CAAAGTCGGCAATGAGTATGTAACAAAGGGACAAGGTGTAGATCAAGTCT
ATTACCCCAACAAGGAGGCCTTCAAGTGCGAGGAGTGCGGCAAGCACTAC
AACACCAAGCTGGGCTACAAGCGGCACGTGGCCATGCACTCGGCCACGGC
GGGCGACCTCACCTGCAAGGTGTGCCTGCAGAGCTACGAGAGCACGCCGG
CGCTGCTGGAGCACCTCAAGAGCCACTCGGGCAAGTCGTCGGGCGGCGCC
AAGGAGAAGAAGCACCCGTGCGACCACTGCGACCGCCGCTTCTACACGCG
CAAGGACGTGCGCCGCCACATGGTGGTGCACACGGGCCGCAAGGACTTCC
TGTGCCAGTACTGCGCGCAGCGCTTCGGCCGCAAGGACCACCTGACGCGC
CACGTCAAGAAGAGCCACTCGCAGGAGCTGCTGAAGATCAAGACGGAGCC
TCCGGACATGCTGGGCCTGCTGGGCTCGGGCTCGCCGCCCTGCGCCATCA
AGGAGGAGCTCAGCCCCATGATGTGCAGCATGGGCCCCGGCAAGGACCCC
ATGATGGCCAAGCCCTTCCCCAGCGGCACCCCCTTCCCCATGGGCATGTA
CAACCCCCACCAC------CTCCAGGCCATGTCCAACCCCGGAGTGGGCC
AC---------CACCACTCCCTGGTGCCCGGCTCCCTGTCGGCCGCCATG
GGGATGGGCTGCCACATGGAGTACCTGATTTACGCTTCCTTCTCGTTCAT
GGGATGTTTACAAATCAGCGACGGATCCAACATAGTCAACCTTTTGGCCA
GCGACTCGCCGAGCGTGTCGTATGCTCTGACCCAGCAGAAGTATTTCAGC
AACTACAGCCCAGTGATAGGGTTCTACATCTATGAGCCCATTGACTACTG
GAACGCCACTGTGCAGGAGCACCTCAAGACACTGGGCCAGGGGTTCAATA
CGATATCGTGGATCGATAATTACTTTCAGTATCTGAAGGTGACGAACGTC
AGCGCGTCGACCAAAAGCGACTTCATCGCCGTCCTCAAGACCTCGTTCCT
GAGGAGTCCCGAGTATCAGCACTTCACGGACGACATCATCTTCTCCAAAA
---CGGGGG------------ACGACTTCAACATCATCGCGTCCAGGATG
TACCTGGTGGCGCGGACCACGGAGAAGACCCGGGAGGAGGTGGTGGAGCT
GCTGGAGAGGCTCCGCCCTCTCTCGCTCATCAACAGCATCAAGTTCATCG
TGTTCAACCCCACCTTCGTGTTCATGGACCGCTACAGCTCCTCGGTGGTC
TCGCCCATCATGACGTCCGGGTTCAGCGTCCTGACCATCCTCGTGCTCAC
GTTCTTCCTCGTCGTCAACCCCCTGGGAAACTTCTGGTTGATACTGACCG
TCACCTCAGTGGAGCTGGGGGTCCTGGGCCTGATG---------------

```
--------------------------------------------------
--------------------------------------------------
--------------------------------------------------
--------------------------------------------------
--------------------------------------------------
--------------------------------------------------
--------------------------------------------------
--------------------------------------------------
--------------------------------------------------
--------------------------------------------------
--------------------------------------------------
--------------------------------------------------
--------------------------------------------------
--------------------------------------------------
--------------------------------------------------
--------------------------------------------------
--------------------------------------------------
--------------------------------------------------
--------------------------------------------------
--------------------------------------------------
--------------------------------------------------
--------------------------------------------------
--------------------------------------------------
--------------------------------------------------
--------------------------------------------------
--------------------------------------------------
--------------------------------------------------
------------------------------------------TCCTACAC
CATAGAGATGGGCCCAAGAGGGCCTCAGTGGAAGGAGAGTCCCCAGCCTT
TCGCTTGCTCTATCGAGGACCCCACTAAGCAAACCAAGTTCAAGGGGATT
AAGACTTACATATCGTATCGCGTGACTCCCAGCCACGTCGGCCGGCCTGT
GTACCGTCGCTACAAGCACTTCGACTGGCTGTATAACAGGTTGCTGCACA
AATTCACCGTCATCTCTGTGCCCCATCTGCCCGAGAAGCAGGCGACCGGG
CGTTTCGAGGAGGACTTTATCGAAAAGCGTAAGAGGCGGCTCATCCTCTG
GATGGACCACATGACCAGTCACCCCGTCCTCTCGCAGTACGAGGGCTTCG
AGCACTTCTTCATGTGCGGSGACGATAAACAGTGGAAGCTGGGCAAGCGG
AGGGCGGAGAAGGACGAGATGGTGGGAGCCCACTTCATGCTCACCTTCCA
GATCCCCAACGAGCACCAGGACCTGCAGGACGTGGAGGAGCGGGTGGACT
CCTTCAAGGCCTTCGCCAAGAAGATGGACGACAGCGTCATGCAGTTGACG
CACGTGGCCTCAGAGCTGGTGCGCAAACACCTGGGAGGGTTCCGCAAAGA
GTTCCAAAGGCTGGGGAACTGCTTCCAGTCCATCAGCCAGGCTTTCATGC
TGGACCCCCCCTACAGCTCCGACGCCCTGAATAACGCCATCTCCCAC---
--------------------------------------------------
--------------------------------------------------
--------------------------------------------------
--------------------------------------------------
--------------------------------------------------
--------------------------------------------------
--------------------------------------------------
--------------------------------------------------
--------------------------------------------------
--------------------------------------------------
--------------------------------------------------
```


----------------------------------GCCAAATCCCGCTTTC
ACCATGGCGTCGGCACGGGGCCTGGCACGGACC---GCAGCGTCCCACTC
---AACAGCTTGCTCTCCCCGCAACAAACCGATGAGACCGCAGTGG---C
CTCCCCGCAGCGATGGTTTGTCACCC---CTGCCAACAACCGACTGGACT
TTGCCGCTTCGGCATACGATGCCGCCGCTGCAGCTGATTTTGCCGGTAAC
GCGGCCACCCTTCTGTCGTACGCAGCTGCCGGAGTGAAAGCGC------T
CCCACTGCCCACTGCAGGCTGCTCCAACAGGGCCCTGGGCTATTACGCGG
AACCGCCAG---GGTGG---GGCACACGCACTCCACCGCAGTACTGT---
------AGTAAATCAAGCGCGGTTCTCTCATGCTGGCCCGCCAATTCCGT
TGGGTGCAGAACATCCA---CCTCCAATTACCTGG---TTGGCTTGGACG
A---CGGG---GACGC---TATCGCACCTGAGAGGTCACCT---CT---C
GGGGGGGCAGACGAA---GCCAAGCCAAAAGACCT------GTCGGA---
GTCAAGCTGGATAGAG---ACCCCGTCTTCAATTAAGTCAATCGACTCAA
GTGATTCTGGGATCTTTG---AGCAGGCCAAACGGAGGAGGATTTCGCCA
TCTGCTACACCG---------GTTTCAGAGACGTCGTCCCCATTAAAATC
AGAA---------ACAGGCGAAGTCACAGACAGAGAAGTGGCTTTAGGGA
TAAATCCATTCGCCGACGGGATGGGCGCTTTCAAAATCAACCACAGCTCC
CACGATATTGGCTCTGG---TCAAACGGCGTTTGCCTCGCAGGCG---CC
CGGCTAC---GCAGCCGCTGCCCTGGGA---CACCATCA------CCACC
CAACACATGTCAGCTCC---TACTCCACCGCGGCGTTCAACTCCACCCGG
GACTTTCTCTTTCGGAATCGGGGATTCGGAGACGCCTCTAG---------
---CGCGCAGCACAGTCTCTTCGCCTCAGC---TGCGGGAAGTTT---C-
-----GCTGGGCCACATGGACACACCGATGCCACGGGACACCTGCTCTTC
TCGGGACTGCACGAG---CAAGCGGCGACCCACGCGTCTTCGAACGCGGT
GAACAGTCAGATGCGCCTGGGCTTTTCGGGGGACATGTACGGCAGAGCCG
AGCAGTATGGTCACGTAACGAGCCCCCGGT---CCGAGCACTACGCTTCG
ACTCAGTTGCACGGCTACGGCCCCATGAACATGAATATGGCTGCC---CA
CCACGGGGCAGGGGCCTTCTTCCGTTACATGAGGCAGCCGATCAAACAAG
AGCTCATCTGCAAGTGGGTCGAACCAGAGCAGCTGTCGAATCCGAAAAAG
TCCTGCAACAAAACTTTCAGCACGATGCATGAGCTCGTGACCCACCTCAC
AGTGGAACATGTCGGGGGACCGGAACAGTCGAATCACATTTGCTTTTGGG
AAGAGTGTCCGCGAGAAGGGAAACCATTTAAAGCCAAGTACAAACTTGTA
AATCATATCAGAGTGCACACCGGA--------------------------

>Hoplostethus atlanticus
AGCTTACTCATCCGAGCTGAGCTTAGCCAACCTGGGGCGCTCTTAGGAGA
CGACCAGATTTACAATGTTATTGTTACAGCACATGCCTTTGTAATAATTT
TCTTTATAGTAATACCAATTATGATCGGTGGTTTTGGGAACTGACTTGTT
CCCTTAATGATTGGAGCTCCTGATATAGCATTTCCTCGAATAAATAATAT
GAGCTTTTGACTCCTCCCCCCTTCATTCCTTCTCCTCCTTTCCTCCTCCG
GAGTTGAAGCAGGGGCCGGAACAGGATGAACAGTTTACCCGCCCCTCGCA
GGAAACCTCGCCCACGCAGGGGCCTCCGTAGACCTAACCATCTTCTCCTT
ACATCTGGCAGGTGTTTCCTCAATTCTGGGGGCCATTAACTTCATTACAA
CCATTATTAATATGAAGCCTCCAGCCATTTCCCAGTATCAAACCCCCCTG
TTCGTATGATCCGTTTTAATTACAGCAGTCCTCCTCCTTTTATCCCTCCC

CGTCCTTGCAGCCGGCATCACCATGCTCCTTACAGACCGAAACCTAAATA
CAACCTTCTTTGATCCCGCAGGAGGAGGAGACCCCATTTTATACCAACAC
CTGTTCTGATTCTTTGGCCACCCC--------------------------

------------TTTCTAGAGAGAAACCTTCATCCATCTAACTGCCTGGG
CATGCTGTTGCTGTCTGATGCACACCAGTGTACCAAGCTATCAGAGCTCT
CCTGGGGCATGTGCCTCAGCAACTTTCCGGCTATTTGCAAGACAGAGGAC
TTCCTCCAGCTGCCCAAAGACATGGTGGTGCAGCTTCTATCCCATGAGGA
GCTGGAGACAGAAGATGAGAGACTGGTTTATGAAGCTGCCCTTAACTGGG
TCAACTATGACCTGGAAAGGAGGCACTGCCATCTGCCAGAGCTGCTGAGA
ACGGTTCGCCTGGCCCTGCTACCTGCCATCTTCCTCATGGAGAATGTCTC
TACAGAAGAGCTGATCAATGCCCAGGCCAAGAGCAAGGAGCTGGTGGATG
AGGCCATCCGCTGCAAGCTAAAGATCTTGCAGAATGACGGCGTGGTTAAC
AGCCCCTGTGCTCGGCCGAGAAAAACCAGCCATGCCCTTTTTCTGTTGGG
AGGTCAGACCTTCATGTGTGACAAGCTGTATCTGGTGGACCAGAAGGCAA
AAGAGATCATCCCCAAGGCTGACATCCCCAGCCCTAGGAAGGAGTTCAGT
GCCTGCGCCATCGGCTGTAAGGTCTACATCACAGGTGGCA--GAGGCTC-
AGAAAATGGTGTGTCTAAAGATGTATGGGTCTATGACACCGTCCAAGAGG
AATGGTCCAAGGCAGCGCCCATGCTCATCGCCAGATTTGGTCACGGCTCT
GCTGAGCTGAAACACTGCCTTTACGTAGTAGGAGGTCACACGGCAGCAAC
CGGCTGCCTCCCAGCCTCTCCCTCCGGATGAATACATTGTAGTGTTCAGC
CGTTCATCAACAAGGCTGATTCTGAACGAAGCGGAGGTAATCATGACGCT
GGCCCAGGAATTTCAGATGAGAGTGGTTACGGTCTCCCTAGAGGAACAAA
CTTTCCCCAGCATCGCCCAGGTGATCAGTGGGGCCTCCATGTTAGTCAGC
ATGCATGGAGCTCAGCTTGTCACCTCACTCTTCCTCCCCAGAGGAGCTGC
TGTGGTGGAGCTGTTCCCCTATGCTGTCAACCCAGAACAGTACACCCCAT
ATAAAACCCTCGCCTCCCTACCAGGCATGGACCTTCAATATGTTTCCTGG
AGGAACACTGTAGAAGAGAACACTGTCACCCACCCAGACAGATCCTGGGA
AGAAGGAGGCATCGCCCATTTGGAAAAGGACGAGCAAGAGCGAATACTGG
CCAGCAAGGATGTCCCCAGACACCTGTGCTGCCGCAACCCAGAGTGGCTC
TTCCGAATCTACCAGGACACTYTGGTGGACATCCCTTCATTCCTGGAGGT
CCTCAA---AGAGGGCCTGAAG---ACCAGGCCCAGYTTGAAGAA---GG
CCAAGCTGGCCAGCACGGTTCATCCAGGCCGGGTCAGAGAACCCCAGTGC
CACACCTCAGTCCAAGCCACCAACGAGGCTAAACTCACGGTTTCCTGGCA
GATCCCATGGAATCTGAAATACCTGAAGGTGAGAGAAGTGAAGTACGAGG
TGTGGATCCAGAAGAAGGATACCGACAAGGGGACACTGGGGGATCAAATC
ATCCAGGCAAACCCTGCGCTGGAGGCTTTTGGTAACGCCAAAACAGTGAG
GAATGACAACTCATCCCGTTTTGGAAAATTCATCCGAATTCACTTTGGAA
CCAGTGGCAAGCTGTCCTCTGCTGATATTGAAACTTACCTATTGGAGAAG
TCACGTGTCACCTTTCAGCTCAAGGCTGAGAGGAACTACCACATCTTCTT
CCAGATATTGTCCAATCAGAAGCCAGAGCTGTTGGACATGATGTTGATCA
CCAACAACCCATATGACTACTCCTACATCTCCCAAGGAGAGGTAACAGTA
GCATCCATCAATGACTCAGAGGAGCTGATGGCCACTGACAGCGCCTTTGA
CGTGCTTGGCTTTACTCAAGAGGAGAAGATGGGAGTATATAAGCTGATTG
GTGCCATTATGCGCTATGGCAACATGAAGTTTAAGCAGAAGCAGCGTGAG
GAGCAGGCTGAGCCTGACGGAACAGAGGCTGCCGATAAGTCAGCTTACCT
TAAGGTGGTGAATTATGCAGACCTCATCAAAGGGTANAGCCATCCCAGAGT
CAAGGTAGGAAATGAATATGTCACCAAAGGCCAAGGNNNNNNNNNNNNNNNNNNCCCAACAAGGAGGC
CTTCAAGTGCGAGGAGTGTGGCAAGCACTACAACACCAAGCTGGGATACA
AGCGCCATGTGGCCATGCACTCTGCCACAGCAGGGGACCTCACCTGTAAA
GTGTGCATGCAGAGCTATGAGAGCACGCCGGTGCTCCTGGAGCACCTTAA

GAGCCACTCGGGGAAGTCCTCGGGCGGCGCCAAGGAGAAGAAACACCCAT
GCGACCACTGCGACCGCCGTTTCTACACAAGGAAGGATGTGAGACGGCAC
ATGGTGGTCCACACGGGCCGAAAAGACTTCCTGTGCCAGTACTGCGCCCA
GCGCTTCGGCAGGAAGGACCACCTGACACGCCACGTAAAGAAGAGCCACT
CGCAGGAGCTGCTGAAGATCAAGACAGAGCCTCCAGATATGTTAGGTCTT
TTAGGTTCTGGCTCGCCGCCGTGCTCTGTCAAGGAGGAGCTTAGCCCTAT
GATGTGCAGCATGGGTCCCAACAAAGACCCCATGATGGGCAAACCCTTCC
CCAGTGGGACCCCCTTCCCCATGGGCATGTACAACCCCCCACCAC------
CTCCAGGCCATGTCCAATTCTGGGGTGGGCCACCCC------CACCCCTC
CCTGATGTCTGGCTCCCTGTCTGCAGCTATGGGCATGGGCTGTCACATGG
AATATCTCATCTATGCCTCTTTCTCATTCATGGGATGTTTACAAATCAGT
GATGGCTCAAACATTGTGAACTTGCTGGCTAGTAACTCTCCGAGCGTTTC
ATATGCTCTGACCCAGCAGAAATACTTCAGTAACTACAGTCCCGTGATTG
GATTTTACATTTACGAGCCCATTGAGTACTGGAACTCCACAGTGCAGGAG
CACCTTAAGACACTGAGTCATGGCTTCAACAAGATCTCCTGGATGGACAA
CTTCTTCCACTACCTGCGGGTGGTGAACGTGAGCGCGTCGACCAAGAATG
ACTTTATCAGCATCCTCAAGGGCTCTTTCCTGCACAGCCCGGAGTACCAG
CACTTCACTGAGGACATCATCTTCTCCAAGA---ACCGTGAGAGTG----
--ATGAGTATGACATTATTGCCTCGCGCATGTACCTGGTGGCGCGGACCA
CAGAGAAGAAGCGCGAGGAGGTGGTGGAGCTTCTGGAGAAACTGCGTCCA
CTGATGCTGATCAATAGCATCAAGTTCATCGCCTTCAACCCCACCTTTGT
TTTCATGGACCGCTACAGCTCCTCAGTCATCTCGCCCATCCTGACCTCAG
GCTTCAGCGTGCTCACCATCCTCATCCTCACTTTCTTCCTGGTCATCAAC
CCCTTGGGGAACTTCTGGCTCATCCTCACAGTTACTTCTGTGGAGCTGGG
TGTCTTGGGTTTNNNNGGCTATCATCTATTCGAATGGCAGCCGGCCCTCAAGAA
TGTGTCTGCATCCTGCCATGTGGGCATTATTAATGGGCTCTCTGGATTGG
CTGCCTTGGTGGATGACTCCCCGGCTGACACCATTACTCGACGGTTTCGC
TACGACGTGGCCCTGGTATCAGCCTTAAAGGATCTGGAGGAGCACATCAT
GGAGGGACTGAAAGAGTGTGGGCTGGAAGACAGTGCTTGCACCTCAGGCT
TCAGTGTTATGATCAAGGAATCTTGTGACGGCATGGGAGATGTCAGCGAG
AAGCACGGGGGAGGGCCGATGGTCCCTGAGAAGGCTGTACGCTTCTCTAT
TACTGTTATGTCTGTCTCTGTCCTGGCAGATGGAGAGGAA----------
--------------------GAGGCGGTTACCATCTTTACCGAGCCAAAG
CCCAACTCAGAACTGTCCTGTAAGCCCCTAAGCCTGATGTTTGTGGATGA
GTCAGACCATGAGACGCTCACTGCTGTCCTGGGACCTTTACTTGCAGAGC
GTAAAGCAATGAAGCAGAGTCAACTCATCCTACCTATGGGTGGCCTCCCT
CGCTCCTTCCGCTTCCACTTCAGAGCCACAGGATATGATGAGAAGATGGT
GCGTGACATGGAGGGCCTGGAGGCCTCAGGTTCCACCTATATCTGCACTC
TGTGTGATTCCGGTCGAGCAGAAGCCTCTCACAACATGATACTACACTCC
ATCACCCGCAGCCACGATGAGAACCTGGATCGTTATGAAATATGGAGGAC
CAATCCCTTTTCTGAGTCTGTTGATGAGCTGCGAGACCGGGTCAAAGGGG
TCTCTGCCAAGCCCTTTATGGAGACACAGCCCACTATTGATGCATTACAC
TGCGACATTGGTAATGCCACAGAGTTCTACAAAATCTTCCAGGATGAGAT
TGGGGAGGTGTACCAAGAAGT---CAAC---CCCACCCGGGAAGAACGGC
GGAGCTGGCGAGCAGCCCTAGACAAGCACCTGAGGAAGAAGATGAAGCTT
AAACCAGTGATGAGGATGAATGGGAACTACGCCCGGAAGCTAATGACCCA
GGAGGCTGCGGAGGTGGTGTGTGAGCTGATACCCTCAGAAGAGAGGAGAG
AGGCCCTGAGGGAGCTTATGGGGCTCTACATCCAGATGAAGCCTGTGTGG
CGCGCTACCTGCCCAGCCAAGGAATGCCCTGACGAGCTGTGCCGCTACAG
CTTTAACTCCCAGAACTTTGCCAATCTCCTCTCCACCACTTTCAAATATA
GGTACAATGGAAAGATCACCAATTATCTGCATAAGACCCTCGCCCATGTC
CCTGAAATCATCGAAAGAGATGGCTCTATAGGAGCCTGGGCCAGTGAGGG
GAATGAATCAGCAAACAANTCGTACACCATTGAGATGGCTCCAAAGGGGCC

CCAATGGAAGGAGAGTCCTCAGCCTTTCTCCTGCTCCATTGAAGACCCCA
CAAAACAGACCAAGTTCAAAGGCATCAAGACCTACATTTCGTACCGGGTC
ACACCGAGCCACACAGGGCGTCCCGTCTACAGACGCTACAAACACTTTGA
CTGGCTGTACAACCGCTTGCTGCACAAGTTCACTGTGATCTCGGTGCCCC
ACCTGCCTGAGAAGCAGGCCACGGGGCGCTTCGAGGAGGACTTCATCGAG
AAGCGTAAAAGACGACTGATTCTGTGGATGAACCACATGACCAGTCACCC
AGTCCTCTCCCAGTATGAGGGATTTGAGCACTTCCTTATGTGTGCTGACG
ACAAGCAATGGAAGCTGGGTAAGAGGCGGGCGGAGAAGGACGAGATGGTG
GGCGCCCACTTCATGTTGACCTTCCAGATTCCTAACGAGCATCAGGATCT
CCAGGATGTGGAGGAGCGGGTTGACTCCTTCAAGTCCTTTGCTAAGAAAA
TGGACGACAGCGTCATGCAGCTCACACATGTTGCCTCGGAGCTGGTGAGA
AAGCACCTGGGTGGATTCAGGAAGGAGTTCCAGCGGCTAGGAAATGCCTT
CCAGTCTATCAGCCAGGCGTTCATGCTGGAACCTCCCCACAGCTCTGATG
CCCTCAACAACGCCATCTCCCACNNNNNNNNCACGTT-CTCAAACTGACCTCTCTGGG
TTTCATCATTGGAGTCGGTGTGGTCGGGAACCTCCTGATCTCCATCCTGC
TGGTCAAAGACAAGAGCCTGCACCGCGCGCCCTACTACTTCCTGCTAGAC
CTGTGCGCCTCCGACATCCTGCGCTCTGCCATCTGCTTCCCCTTCGTCTT
CACCTCTGTCAAGAATGGATCCGCCTGGACCTATGGCACGCTCACCTGCA
AAGTGATCGCCTTCCTCGGCGTGCTCTCCTGTTTCCACACGGCGTTCATG
CTATTCTGTGTCAGCGTCACTCGCTACCTGGCTATTGCGCATCACCGCTT
CTACACCAAGAGGCTGACCTTTTGGACCTGTCTAGCTGTCATCTGCATGG
TGTGGACGTTGTCAGTAGCCATGGCTTTTCCCCCGGTGCTGGACGTAGGG
ACGTACTCTTTCATCCGGGAGGAGGACCAGTGCACATTCCAGCACCGTTC
TTTCAGGGCCAATGACTCACTGGGCTTCATGCTCCTGCTGGCACTCATCC
TCCTGGCCACACAGCTGGTTTACCTCAAGCTCATCTTCTTTGTCCACGAC
CGTCGGAAGATGAAGCCCGTCCAGTTTGTGCCTGCCGTCAGCCAGAACTG
GACCTTCCATGGGCCAGGCGCCAGTGGGCAGGCGGCTGCTAACTGGCTCG
CTGGATTTGGGAGAGGCCCCACCCCGCCTACCTTGCTAGGCATCCGGCAG
AACAGCAACGCGGCGGGCCGCAGGCGTCTGCTGGTGCTGGATGAGTTCAA
AACAGAGAAGAGGATTAGTAGGATGTTCTACATCATGACGTTTTTCTTCC
TGGCGTTGTGGGGGCCCTATCTGGTAGCCTGCTACTGGCGGGTATTTGCA
AGGGGCCCCGTAGTCCCCGGGGGCTACCTGACGGCAGCCGTGTGGATGAG
CTTTGCCCAGGCTGGGGTCAATCCATTCATCTGCATCTTCTCTAACAGGG
AGGCCAAATCTCGCTTTCACCCTGGCGTGGGGACTGGTCCTGGCACGGAG
C---GCAGCGTCCCACTCGGCAACAGCTTGCTATCCCCGCAGCAAACCGA
GGAGCCCACTGTTGCCACCCCCCCGCAGCGATGGTTTGTCACCC---CTG
CCAACAACCGACTGGACTTTGCTGCCTCGGCATACGACGCGGCC------
---GATTTCGCCGGTAACGCGGCCACCTTGCTGTCCTACGCAGCGGCCGG
AGTGAAGGCTC------TTCCTCTGCCGGCTGCAGGCTGCTCCAACCGGC
CTCTTGGCTATTACGCAGACCCGTCGG---GCTGG---GGCGGACGCACT
CCGCCGCAGTACTGTAGCGTAAACAGCAAACCAAGCTCGGTCCTTTCCTG
CTGGCCCTCTAACTCTGTCGGCGGCAGAGCAGGCG---CC---AACTATC
TGG---------CCGAGGA---CGGA---GACGC---CATCCCGACGGAG
AGATCCCCG---AT---CGGTGGCTCCGAGGAG---ACGAAACCCAAGGA
CCT------GTCGGA---GTCGAGCTGGATAGAG---ACGCCGTCCTCCA
TTAAGTCAATTGATTCAAGCGATTCTGGTATCTTCG---AACAGGCCAAA
AGGAGAAGAATCTCGCCCTCTGCCACGCCG---------GTTTCAGAAAC
TGTGTCCCCGTTAAAATCTNNNCATCACTCAACAGGCGAAGTCACAGAGAGAG
AAGTGGCGTTGGGGATAAATCCGTTCGCGGACGGGATGGGCGCCTTCAAA
ATCAACCACAGCTCCCACGATATCGGCTCCGG---ACAGACGGCGTTTTC
CTCCCAGGCG---CCCGGCTAC---GCAGCGGCCGCACTGGGA---CACC
ATCA------CCACCCGACCCACGTTGGCTCT---TACTCCACGGCGGCT
TTCAACTCCACCCGGGACTTTCTCTTTAGAAATCGGGGTTTCGGAGACGC

CACCGG------------GGCGCAGCACAGTTTGTTCGCCTC--------
-CGGAAGTTT---C------GCAGGGCCACATGGACACTCAGATGCCGCG
GGACACCTGCTCTTCCCAGGACTCCACGAG---CAAGCAGCGAGCCACGC
GTCTTCTAATGTGGTCAACAGCCAGATGCGATTGGGCTTCTCGGGGGACA
TGTACGGTCGGGCCGACCAGTATGGCCACGTTACAAGCCCAAGGT---CC
GACCACTATGCTTCGACCCAGTTGCACGGCTATGGCCCCATGAACATGAA
TATGGCCGCA---CACCACGGAGCAGGGGCCTTCTTTCGATACATGAGGC
AGCCGATCAAACAAGAGCTCATCTGCAAGTGGATCGAGCCGGAGCAACTG
ACGAATCCCAAAAAGTCGTGCAACAAAACTTTTAGTACGATGCATGAGCT
GGTGACCCATCTGACGGTGGAGCATGTGGGGGGACCAGAGCAGTCCAACC
ACATCTGCTTCTGGGAAGACTGCGCCAGAGAAGGGAAACCATTCAAAGCC
AAATACAAACTTGTAAATCATATCAGAGTACACACCGGAGAGAAACCCTT
TCCATGTCCGTTTCCCGGCTGTGGCAAA
>Howella zina

------------TTCCTGGAAGGAAACCTTCACCCGACTAACTGCCTCGG
CATGCTGTTGCTGTCTGACGCCCACCAGTGCACCAAGCTGTCAGAGCTCT
CCTGGGGCATGTGTCTCAGCAACTTCCCCGCTATTTGCAAGACGGAGGAC
TTCCTCCAACTGCCCAAAGATATGGTGGTGCAGCTTCTGTCACACGAGGA
GCTAGAGACAGAAGACGAGAGACTGGTTTATGAAGCTGCCCTTAACTGGA
TCAACTACGACCTGGAAAAGAGGCACTGCAACCTCCCAGAGCTCCTGAGA
ACGGTCCGTCTGGCCCTGCTGCCTGCCATCTTTCTCATGGAGAACGTTTC
TACAGAAGAGCTGATCAACGCCCAGGCCAAGAGCAAGGAGCTGGTGGACG
AAGCTATCCGCTGCAAGCTGAAGATCCTGCAGAACGATGGCGTCGTCAAC
AGCCCGTGTGCTCGGCCGAGAAAAACCAGCCATGCCCTCTTCCTTCTGGG
AGGGCAGACGTTCATGTGTGACAAGCTGTACCTGGTGGACCAGAAAGCCA
AAGAGATCATCCCCAAGGCTGACATACCCAGCCCAAGAAAGGAGTTCAGT
GCCTGCGCCATTGGCTGTAAGGTGTACATCACCGGTGGCA--GAGGCTC-
AGAGAACGGTGTGTCCAAAGATGTGTGGGTCTACGACACCGTCCACGAGG
AATGGTCGAAGGCAGCACCCATGCTCATCGCCAGGTTCGGCCACGGCTTT
GCGGAGCTGAAACACTGCCTCTACGNNNNNNNNNNNNNNNNNNNNNNNNNNNNNNNNNNNNNNNNNNNNN
NNNNNGGATGAATACATTGTTGTGTTCAGT
CGTTCGACAACGAGGCTGATACTGAATGAAGCGGAGCTAATCATGGTGCT
GGCCCAGGAGTTCCAGATGAGAGTGGTCACGGTATCCCTGGAGGAACAGT
CTTTCCCCAGTATCGTCCAGGTGATCAGCGGAGCTTCCATGTTAGTCAGT
ATGCACGGAGCTCAGCTCATCACCTCACTCTTCCTCCCCAGAGGAGCTGC
TGTGGTGGAGCTCTTCCCCTTTGCTGTGAACCCAGAGCAGTACACCCCGT
ATAAAACCCTTGCCTCGCTTCCAGGCATGGACCTTCACTATGTCTCCTGG

AGGAACACTAAGGAAGAGAACACTATCACCCACCCAGACAGACCCTGGGA
ACAAGGGGGCATTGCTCACTTGGAGAAGGAGGAGCAAGAGCGAATACTGG
TGAGCAAAGATGTCCCCAGGCACCTGTGCTGCCGCAACCCAGAGTGGCTC
TTCAGAATCTACCAGGACACTTTGGTGGACATCCCTTCCTTCCTGGAAGT
CCTCAA---AGAGGGCATGAAG---ACAAAGCCCAGTTTGAAGAA---GT
CAAAGCCGGCCAGCACAGTACACCCAGGCCGGGTCAGAGAACCCCAGTGT
CAGACCTCAGTACAAACCACTAATGAGGCTAAACTCACAGTCTCCTGGCA
GATCCCGTGGAACCTGAAATACCTGAAGGTGAGAGAGGTCAAATATGAAG
TG---------AAAAAAGACACCAGCAAGGGGACGCTGGAGGATCAAATC
ATCCAGGCGAACCCGGCACTTGAGGCCTTTGGCAATGCCAAAACTTTGAG
AAATGACAACTCGTCTCGTTTTGGAAAATTCATTCGAATTCACTTCGGTA
TGAGCGGCAAGTTGTCGTCCGCCGACATCGAGACGTACCTGCTGGAGAAG
TCACGTGTCACCTTTCAGCTCAAGGCTGAGAGGGACTACCATATCTTCTA
CCAGATCCTGTCCAATCAGAAGCCAGAGCTCCTGGACATGCTGCTGATCA
CCAACAACCCGTACGACTACTCCTACATCTCCCAAGGAGAGGTGACGGTT
GCCTCCATCAACGACTCGGAGGAGCTGATGGCCACCGACAGTGCCTTCGA
CGTGCTCGGCTTTACTCCAGACGAGAAGATGGGCGTCTATAAACTGACCG
GTGCCATCATGCACTACGGAAACATGAAGTTCAAACAGAAGCAGCGTGAG
GAGCAGGCTGAGTCCGACGGGACAGAGGCTGCTGATAAATCAGCTTACCT
GATGGGGCTGAACTCTGCTGACCTCATCAAAGGGCTGTGCCATCCCAGAG
TCAAGGTAGGAAATGAATATGTCACCAAAGGCCAAGGTGTGGATCAAGTC
TACTACNNNNNNNNNNNNNNNNNNNNNNNNNNNNNNNNGGGAAGCACTACAACACCAAGCTGGGATATA
AGCGCCATGTTGC
CATGCACTCGGCCACGGCAGGGGATCTCACCTGTAAAGTGTGCATGCAGA
CCTACGAGAGCACGCCTGTGCTCTTGGAGCACCTCAAGAGCCACTCCGGG
AAGTCTTCGGGTGGCACCAAGGAGAAAAAACACCCGTGCGATCACTGTGA
CCGTCGCTTCTACACACGGAAGGATGTGAGAAGGCACATGGTGGTCCACA
CGGGCCGAAAGGACTTCCTGTGCCAGTACTGTGCCCAGCGCTTCGGCAGG
AAGGACCATCTGACGCGCCACGTGAAGAAGAGCCACTCGCAGGAGCTGCT
GAAGATCAAGACGGAGCCTCCTGACATGTTAGGTCTTTTAGCGTCGGGGT
CACCACCCTGCTCTGTGAAGGAGGAGCTCAGCCCCATGATGTGCGGCATG
GGTCCCAACAAAGACCCCATGATGGGCAAACCGTTCCCCAGTGGGGCCCC
GTTCCCGATGGGCATGTACAACCCCCACCAT------CTCCAGGCCATGT
CTAATTCTGGGGTGGGTCACCCA------CACCCGTCCCTGATGCCCACT
TCCTTGTCTGCAGCTATGGGCATGGGCTGCCACATGGAANNNNNNNNNNNNNNNNNNNNNNNNNNNNNN
NNNNNNNNNNNNNNNNNNNNNNNNNNNNNNNCGTGAACCTGC
TGGCTAGTAACTCTCCGAGTGTTTCGTACGCTCTGACCCAGCAGAAATAC
TTCAGTAACTACAGTCCCGTAATTGGGTTTTACATTTACGAGCCCATAGA
GTACTGGAACGCAACGGTGCAGGAGCACCTGAAGACTCTGAGTCATGGCT
TCAACAAGATCTCCTGGATGGACAACTTTTTCCACTACCTGCGGGTGGTG
AATGTGAGTGCGTCAACCAAGAGCGACTTCATCACCATCCTCAAGGGCTC
CTTCTTGCGCAGCCCAGAGTACCAGCACTTCACCGAGGACATCATATTCT
CCAAGA---ACCGCGAGACTG------ATGAGTATGACATTATTGCCTCA
CGGATGTACTTGGTGGCACGGACGACAGAGAAGAAGCGTGAAGAGGTGGT
GGAGCTTCTGGAGAAACTTCGTCCGTTGATGCTGATCAACAGCATCAAGT
TCATTGCCTTCAATCCTACGTTTGTGTTCATGGACCGCTACAGTTCCTCT
GTCATCTCGCCCATCCTGACCTCAGGCTTCAGCGTACTCACAATCCTCAT
CCTCACTTTCTTTCTGGTCATCAACCCCTTGGAAAACTTCTGNNNNNNNNNNNNNNNNNNNNNNNNNNN
NNNNNNNNNNNNNNNNNNNNNNNNNNNNNNNNNNNNNNNNNNNNNNNNNNNNNNNNNNNNNNNNNNNNT
CTTGCAA
CGTCGGCATCATTAACGGGCTCTCCGGATGCTCTTCCTCGCTGGACGACT
CCCCTGCCGACACCGTCACTCGGCGGTTTCGCTACGATGTGGCTCTGGTG
TCGGCGATGAAGGACCTGGAGGAGGACATCATGGAGGGGCTGAGGGACAG

CGGCCTGGAAGACAGTGCGTGCACCTCAGGCTTCACCGTCATGATCAAGG
AGTCTTGCGACGGCATGGGCGACCTCAGCGAGAAGCACGGCGGAGGACCG
GCTGTTCCCGAGAAGGCCGTACGTTTCTCTTTCACCATCATGTCCGTCTC
CGTCGTGGCAGACGGCGAGGAG----------------------------
--GAAGCAGTCACCATCTTCACCGAGCCAAAGCCGAACTCAGAACTGTCC
TGTAAGCCCATCTGCCTGACGTTTGTGGACGAGTCAGACCACGAGACGCT
CACCGCCGTCCTGGGACCGTTAGTCGCAGAGCGCAACGCGATGAAAGACA
GCAGGCTCATCCTACCCGTGGGCGGACTCGCTCGCTCTTTCCGTTTTCAC
TTCAGAGGCACCGGTTACGACGAGAAGATGGTGCGCGAGATGGAGGGCCT
CGAGGCATCGGGGTCCTCCTACGTCTGCACTCTTTGTGACTCCAGCCGGG
CGGAAGCCGCTCAGAACATGGTGCTGCACTCCATCACCCGGAGTCATGAA
GAGAACCTGGAACGCTACGAAATCTGGAGAACGAACCCCTTTTCTGAGTC
TGTGGACGAGCTGCGAGACAGAGTCAAAGGAGTCTCCGCCAAGCCCTTCA
TGGAGATCCACCCCACCATGGACGCATTGCACTGCGACATCGGCAACGCC
ACAGAGTTCTACAAGATCTTTCAGGACGAGATCGGGGAGGTGTACCAAAG
GGC---CAAC---CCCAGCCGGGAGGAGCGGCGCAGCTGGAGAGCAGCCC
TGGACAAACAGCTGAGGAGGAAGATGAAGCTCAAACCGGTAATGAGGATG
AATGGGAACTACGCCCGCAAGCTGATGACCCAGGAGGCTGTGGAGGTGGT
GTGCGAGCTGGTGCCCTCAGAAGAGAGGAGGGAGGCCCTGAGGGAGCTCA
TGAGGCTCTACCTCCAGATGAAGCCCGTGTGGCGGGCCACCTGCCCGGCC
AAGGAATGCCCCGACCAGCTGTGCCGCTACAGCTTCAACTCCCAGCGCTT
CGCCGACCTCCTCGCCTCCACCTTCAAGTACAGGTACAACGGGAAGATAA
CCAACTACCTGCACAAGACCCTGGCCCACNNNNNNNNNNNNNNNNNNNNNNNNNNNNNNNNNNNNNNNNN
NNNNNNNNNNNNNNNNNNNNNNNNNNNNNNNTCGTACACCATCGAGATGTGC
CCCTTGGGGCCTGGGTGGAAGGAGAGTCCGCAGCCTTTCTCCTGCTCCGT
CGAAGACCCCACAAAACAGACAAAGTTCAAGGGCATCAAGACGTACATTT
CCTACCGGGTCACGCCGAGCCACACGGGGCAACCCGTCTACAGGCGCTAC
AAACACTTTGACTGGCTGTACAACCGCCTACTGCACAAGTTCACTGTGAT
CTCCGTGCCTCACCTGCCCGAGAAGCAGGCCACGGGGCGATTCGAGGAAG
ACTTCATCGAGAAGCGCAAGAGGCGACTGATACTGTGGATGAACCACATG
ACCAGTCACCCGGTCCTCTCCCAGTACGAAGGCTTCGAGCACTTTCTGAT
GTGCGCCGAYGACAAGCAGTGGAAACTGGGCAAGAGGCGGGCGGAGAAGG
ACGAGATGGTGGGCGCCCATTTCATGCTGACCCTCCAGATCCCGAACGAG
CACCAGGACCTTCAGGACGTAGAGGAGCGCATCGACACCTTCAAGGCCTT
CGCTAAGAAAATGGACGACAGCGTGATGCAGCTGACGCATGTTGCCTCGG
AGCTGGTGCGTAAACACCTGGGTGGGTTCAGGAAGGAGTTCCAGCGGCTG
GGAAACGCCTTCCAGTCCATCAGCCAGGCGTTCATGCTGGACCCTCCCCA
CAGCTCGGAAACCTTCAACAACGCCATCTCCCAC----------------

>Hygophum proximum
AGCCTCCTTATTCGCGCTGAACTAAGCCAACCCGGGGCTCTCCTCGGGGA
TGACCAAATCTACAATGTTATCGTAACAGCTCACGCCTTCGTAATAATTT
TCTTTATAGTCATACCAATTTTAATTGGAGGCTTCGGAAACTGGCTAATC
CCACTAATAATCGGAGCCCCTGACATAGCATTTCCCCGGATAAATAACAT
AAGCTTCTGACTCCTCCCTCCCTCCTTCCTGCTTCTTCTGGCATCTTCCG
GCGTTGAAGCCGGAGCCGGGACCGGCTGAACAGTCTACCCTCCCCTCGCC
GGCAATCTCGCCCACGCCGGGGCCTCAGTTGACTTAACCATCTTCTCACT
TCACCTAGCAGGTGTATCCTCTATTTTAGGTGCTATCAACTTTATTACAA
CTATTATTAACATAAAATCCCCTGCCATCTCCCAGTACCAAACCCCTCTT
TTCGTATGAGCAGTCCTAATCACAGCCGTACTTCTCCTCCTCTCACTGCC
AGTTCTGGCGGCCGGAATTACCATGCTTTTAACAGACCGAAATTTAAATA
CAACCTTCTTTGACCCCGCAGGCGGAGGAGACCCCATCCTCTACCAGCAC
TTANNNNNNNNNNNNNNNNNNNNNNNNNNNNNNNNNNNNNNNNNNNNNNNNNNNNNNNNNNNNNNNNNNN
NNNNNNNNNNNNNNNNNNNNNNNNNNNNNNNNNNNNNNNNNNNNNNNNNNNNNNNNNNNNNNNNNNNNNN

NNNNNNNNNNNNNNNNNNNNNNNNNNNNNNNNNNNNNNNNNNNNNNNNNNNNNNNNNNNNNNNNNNNNN
NN--

----------------GGAGGAGTACATYGTAGTATTCAGTCGCTCAACG
ACAAGACTCATTCTCAACGAAGCAGAACTGATTATGGCACTGTCCCAGGA
GTTTCAGATGAGGGTCGTCACTGTGTCCCTTGAAGAGCAACCTTTCCCCA
GTATAGTCCAGCTTATCAGTGGTGCCTCAATGTTGGTCAGTATGCATGGG
GCTCAGCTTGTCACCTCACTCTTCCTCCCCAGAGGAGCAGTGGTGGTTGA
GCTCTTTCCCTATGCTGTGAACCCAGAACAGTACACCCCTTATAAAACCC
TCGCCTCCTTACCGGGGATGGACCTGCAGTACGTTTCCTGGAGGAATACG
ATGGAGGAAAACACCATCACCCACCCAGATAGATCCTGGGACCAAGGAGG
CATCGCCCACCTGGAAAAGGATGAGCAAGAGCGAATCCTTTCCAGCAAGG
ATGTCCCGCGGCACCTGTGCTGCCACAACCCAGAATGGTTCTTCCGAATC
TACCAGGACACTCTGGTGGACATCCCTTCATTTCTGGAGGTTCTCAA---
AGATGGCCTGAAGATCACCAGACCAAGCCTAAAAAA---GGCCAAGCCAG
CCAGCACAGTACATCCAGGCCGGGTCAGGGAAGCACAGTGTCAAACCTCA
GTCCAAGCCACCAACGAAGCTAAACTCACGGTCTCCTGGCAGATCCCATG
GAACCTGAAATACCTGAAGGTGAGAGAGGTCAAATACGAAGTGTGGATCC
AGAAGAGGGACGCGAGCAAGGGGACCCTGGAGGATCAAATCATCCAGGCC
AACCCCGCTCTGGAGGCCTTTGGGAACGCCAAAACCATCCGCAACGACAA
CTCGTCCCGCTTCGGGAAGTTCATCCGCATCCACTTCGGGACGAGCGGCA
AGCTGGCGTCCGCCGACATCGAGACCTACCTGCTGGAGAAGTCGCGCATC
ACCTTCCAGCTCAAGGCCGAGCGGAACTACCACATCTTCTTCCAGATCCT
GTCCAACGAGAAGCCTGAGCTGCTGGAGATGCTGCTGATCACCAACAACC
CGTACGACTACAGCTACATCTCCCAGGGGGAGGTGACGGTGGCGTCCATC
AACGACAACGAGGAGCTGATCGCCACCGACAGCGCCTTCGACGTGCTGGA
ATTCACCCAGGAGGAGAAGATGTCCATCTACAAGCTGACCGGCGCCATCA
TGCACTACGGCAACATGAGGTTCAAGCAGAAGCAGCGCGAGGAGCAGGCC
GAGCCCGACGGCACCGAGGCAGCCGATAAGTCGGCGTACCTGATGGGGCT
GAACTCGGCCGACCTCATCAAAGGACTCTGCCACCCCCGAGTCAAGGTGG
GAAACGAGTATGTCACGAAAGGCCAGGGTGTAGACCAAGTCTACTAC---

-------------TATCTGATCTACGCCTCCTTCTCCTTCATGGGATGTT
TACAAATCAGTGATGGGTCAAACATTGTGAACCTGCTGGCAAGTAACTCT
CCGAGTGTGTCGTTTGCACTCACCCAGCAGAAGTACTTCAGTAACTACAG
CCCTGTTATTGGGTTCTACATCTATGAACCCATTGAGTACTGGAATGCCA
CGGTGCAGGAGCACCTGAAGACACTGAGTCACGGCTTCAACAAGATCTCC
TGGATCGACAACTTCTTCCACTATCTGCGGGTGGTAAATGTGAGCGCGTC
GACCAAGAATGACTTCATCACCATCCTAAAGGGCTCCTTCCTGCGCAGCC
CGGAGTACCAGCACTTCACCGAGGACATCATCTTCTCCAAGA---ACCGC
GAGAGCG------ATGAGTATGACATCATCGCCTCCCGCATGTACCTGGT
GGCACGCACCACCGAGAAGAAGCGCGAGGAGGTGGTGGAGCTGCTGGAGA
AGCTGCGTCCGCTGATGCTGATCAACAGCATCAAGTTCATCGCCTTCAAC
CCCACCTTCGTATTCATGGACCGCTACAGCTCCTCGGTCATATCGCCCAT
CCTTACCTCAGGCTTCAGCGTGCTCACCATCCTCATCCTCACATTCTTTC
TCGTCATCAACCCACTGGGAAACTTCTGGCTCATCCTGACGGNNNNNNNNNNNNNNNNNNNNNNNNNNNN
NNNNNNN--------

---T
CCTACACCATCGAGATGGGCCCCAAAGGGCCCCTGTGGAAGGAGAGCCCG
CAGCCCTTCACCTGTTCCATCGAGGACCCCACCAAACAGACCAAGTTCAA
GGGCATCAAGACGTACATCTCCTACCGGGTCACGCCGAGCCACACAGGTC
ATCCCGTGTACAGACGCTACAAGCACTTTGACTGGCTGTACAACCGCCTG
CTGCACAAGTTCACCGTGATCTCCGTCCCCCACCTGCCAGAGAAGCAGGC

GACGGGGCGCTTCGAGGAGGACTTCATAGAAAAGCGCAAGAGGCGGTTGA
TCCTCTGGATAAACCACATGACCAGTCACCCTGTCCTCTCCCAGTACGAG
GGGTTCGAACACTTCCTGATGTGCGGCGACGACAAGCAATGGAAGCTGGG
CAAGCGGCGAGCAGAGAAGGACGAAATGATCGGTGCCCACTTCATGCTGA
CCTTCCAGATCCCCAATGAGCACCAGGACCTGCAGGATGTGGAGGAGCGT
GTAGACTCCTTCAAGTCGTTTGCCAAGAAGATGGACGACAGTGTCATGCA
GCTGACACACGTGGCCTCGGAACTCGTGCGCAAGCACCTTGGTGGGTTTC
GCAAGGAGTTCCAGCGGCTGGGGAATGCCTTCCAGAACATCAGCCAGGCC
TTCATGCTTGACCCGCCACATAGCTCGGACGGCCTCAACAACGCCANNNNNNNN----

---------------------------------NNNNNNNNNNNNNNTCACCCTGGCGTGGGGA
GCGCTGCTGGCACGGAGC---GCAGCGTCCCACTCAGCAACAGCTTGCTC
TCTCCGCAACAAACCGAGGATCCCGCCGTCG---CCTCCCCGCAGCGATG
GTTTGTCACCC---CTGCCAACAACCGACTGGACTTCGCAGCCTCGGCAT
ACGACGCCGCT---------GATTTCGCCGGTAACGCGGCCACCTTGCTG
TCGTACGCCGCGGCCGGAGTGAAGGCTC------TCCCCCTGCCGACCGC
AGCTTGCTCCAACAGACCTCTGGGTTATTACGCAGACCCGTCGG---GCT
GG---GGCGCGCGCACGCCGCCCCAGTACTGT---------AGCAAGTCG
ACCTCGGTCCTTTCTTGTTGGCCCACGAATTCTGTAGGGAGCAGAACGGG
CA------CAAACTACCTAG---------TGGAGGA---CGGGTCGGACG
C---CTTCCCAACGGAGAGGTC---G---AT---TAATGGCACGGAGGAG
---GCGAAAACCAAAGACTT------GTCCGA---GTCTAGCTGGATAGA
G---ACGCCATCTTCGATCAAATCAATTGACTCAAGTGATTCTGGCATCT
TTG---AGCAAGCAAAGCGGAGACGAATCTCACCGTNNNNNNNNNNN---------
NNNNNNNNNNNNNNNNNNNNNNNNNNNNNNNNN-----

>Hypomesus pretiosus
AGCCTCCTTATCCGAGCCGAGCTTAGCCAACCTGGCGCTCTTCTGGGGGA
CGACCAGATTTATAATGTTATCGTCACTGCACACGCTTTTGTTATAATCT
TTTTTATGGTTATACCAATTATGATTGGAGGGTTTGGTAACTGGCTTATC
CCCCTTATGATCGGGGCCCCAGACATGGCATTCCCCCGTATGAACAACAT
GAGCTTCTGACTCCTCCCTCCCTCTTTCCTTCTTCTTTTGGCCTCCTCCG
GGGTCGAAGCAGGGGCCGGAACTGGTTGAACAGTTTACCCTCCGCTTGCG
GGGAATCTCGCCCATGCGGGAGCTTCCGTAGATTTGACCATTTTTTCTCT
CCATCTTGCAGGAATCTCCTCTATTTTAGGGGCAATTAACTTTATCACAA
CTATTATTAACATGAAACCCCCTGCCATTTCCCAGTACCAGACTCCTTTA
TTCGTTTGGGCCGTTCTAATTACAGCTGTCCTTCTTCTTCTTTCCCTTCC
AGTGCTAGCTGCTGGGATTACTATGCTTCTCACAGACCGAAACCTCAACA
CCACCTTCTTTGACCCTGCCGGGGGAGGGGACCCCATTCTGTACCAGCAT
CTATTCTGATTCTTCGGNNNNNNNNNNNNNNNNNNNNNNNNNNNNNNNNNNNNNNNNNNNNNNNNNNNNN
NNNNNNNNNNNNNNNNNNNNNNNNNNNNNNNNNNNNNNNNNNNNNNNNNNNNNNNNNNNNNNNNNNNNNN
NNNNNNNNNNNNNNNNNNNNNNNNNNNNNNNNNNNNNNNNNNNNNNNNNNNNNNNNNNNNNNNNNNNNNN
NN---------------------------------

------------------------------GGACCAGTACATCGTGGTGT
TCAGTCGTTCCCAAACCAGGCTGATCCTCAACGAGGCCGAGCTGATCCTG
GCGTTGGCGCAGGAGTTCCAGATGAGGGTGGTGACGGTCTCCATGGAGGA
CCAGACCTACCCCAGCATYGTGCGGGTTATMAGCGGCGCCTCCATGCTCG
TCAGCATGCACGGAGCTCAGCTCGTCACCTCGCTCTTCCTCCCCAGAGGG
GCGGCTGTGGTGGAGCTCTTCCCCTACGCGGTCAGCCCTGAGCAGTACAC
GCCTTACAAGACTTTGGCCACCTTGCCAGGCATGGACCTCCAGTATGTCG
CCTGGAGGAACACCATGGAGGAGAACTCCGTGGCTCACCCAGATAGACCC
TGGGATCAGGGGGGCATCGCTCACCTGGACAAGGACGAGCAGGAGCGCAT
CCTGGCCAGCAGAGAGGTGCCCAGACACCTTTGCTGCAGAAACCCAGAGT
GGCTGTTCCGCATCTATCAGGACACCCGGTTGGACATCCCCTCTCTGCTG
GACGTCCTCAG---GGAGAGCCTGAAG---ACCAGGCCCAACCTCAAGAA

---GACCCGGCCTGCCAGCACTGTTCATCCAGGCAGGGTGAGGGAGCCTC
AGTGCCAGACCTCAGTCCAGGCTRCCAACGAAGCCAAGCTCACAGTATCC
TGGCAGATCCCTTGGAACCTCAAGTACTTGAAGGTANNNNNNNNNNNNNNNNNNNNNNNNNNNNNNAAGA
GAGATCCCAG
CAAGGGAACCTTGGAGGATCAAATCATTCAGGCTAACCCTGCCTTGGAGG
CTTTTGGTAATGCCAAAACATTGAGGAATGACAACTCGTCACGATTTGGA
AAATTTATCCGGATTCACTTCGGAACCAGTGGCAAGCTGTCCTCAGCTGA
CATAGAGACTTATCTTTTGGAAAAGTCACGTGTCACCTTCCAACTCAAGT
CGGAAAGGAATTACCATATTTTCTTTCAGATCTTGTCCAATAAAAAGCCA
GAGCTGTTGGACATGCTTCTGATTACCAACAACCCATATGACTACTCGTA
CATCTCCCAAGGAGAGGTAACAGTAGCATCTATCAATGATGCTGATGAGT
TGATGGCCACTGACAGTGCCTTTGATATCCTTGGCTTTACTCAAGAGGAG
AAAATGGGGGTCTACAAGTTGACAGGAGCTATCATGCATTTTGGCAACAT
GAAGTTCAAGCAAAAGCAGCGTGAGGAGCAGGCAGAGCCTGACGGCACTG
AGGCGGCTGACAAGTCAGCTTACCTAATGGGGCTGAACTCTGCAGATCTT
GTGAAAGGACTCTGCCATCCCAGGGTCAAGGTTGGCAATGAGTATGTCAC
CAAAGGGCAGGGTGTAGATCAAGTCTACTACCCAAACAAGGAGGCCTTCC
GCTGCCAGGACTGTGGGAAGCACTACAACACCAAGCTGGGCTACAAGCGC
CATGTGGCCATGCACTCAGCCACAGCAGGGGATCTCACCTGCAAGGTGTG
CCTGCAGAGCTACGAGAGCACGCCGGCGCTGCTGGAGCACTTGAAGAGCC
ATTCGGGGAAGTCGTCGGGCGGTGCTAAGGAGAAGAAGCACCCCTGCGAC
CACTGCGACCGCCGCTTCTACACGCGCAAGGACGTACGCCGCCACATGGT
GGTGCACACGGGCCGCAAGGACTTCCTGTGCCAGTACTGCGCCCAGCGCT
TTGGCAGGAAGGACCACCTCACGAGACACGTGAAGAAGAGCCACTCCCAG
GAGCTGCTGAAGATCAAGACAGAGCCGCCAGACCTGCTGGGGCTGTTGGG
GTCTGGACC------------CATCAAGGAGGAGCTCAGCCCCATGATGT
GCAGCATGAGCTCCACCAAGGACCCCCTTATGGCCAAGCCCTTCCCCGGA
GGGACGGCCTTCCCCATGGGCATGTACAACCCCCACCAC------CTCCA
GCCCCTGCCCAGCCCCGGAGGGGGGCAC---------CACCCCTCCCTGA
TGCCCAGCTCCCTGTCTGCCGCTATGGGCATGGGCTGCCACATGGACTAT
CTGATCTACGCCTCCTTCTCCTTTATGGGGTGTCTGCAGATCAGCGACGG
GTCCAACGTGGTCAACCTGCTGGCCACTAACTCTCCCAGCGTCTCCTACG
CCATGACGCAGCAGAAATACTTCAGCAACTACAGCCCTGTGATCGGCTTC
TACATCTACGAGCCGATTGAGTACTGGAACTCCACGGTGCAGGAACACCT
GAGGACGCTGGGACAAGGCTTCAATAAGATCTCCTGGATCGATAACTACT
TCCACTACCTGAAGGTGGTGAACGTGAGCGCCTCCACCAAGAGCGACTTC
ATCTCCATCCTCAAAGGCTCATTCCTGCAGAGCCCGGAGTACCAGCACTT
CAYGGAGGACATCATCTTCTCCAAGA---ACGGCG------------AGG
AGTACGACATCATCGCGTCCAGGATGTACCTGGTGGCCAGGACCACGGAA
AAGACACGCGAGGAAGTGGTGGAGCTTCTGGAGAGACTCCGCCCCCTGTC
GCTCATCAACAGYATCAAGTTCATCGTGTTCAACCCCACCTTCGTCTTCA
TGGRTCGCTACATCTCCTCCGTCATCTCGCCCATCCTCACCTCTAGCTTC
AGCGTCCTCACCATCCTCATCCTCACCATCTTCCTGGTGGTCAGCCCCCT
GGGGAACTTCTGGCTGATCCTGACAGTGACGTCGGTGGAGCTAGGCGTGC
TAGGCCTGATG---------------------------------------

--GTCAGCGAGA
AGCACGGAGGGGGGCCGGCCATTCCTGAAAAGGCCGTCCGCTTCTCCTTC
ACCGTCATGTCCGTCTCCGTCCTGGCCGAGGGGGAAGAG-----------
-------------------GAAGCAGTCACCGTCTTCAGAGAGCAGAAGC

CCAACTCGGAGATGTCCTGCAAACCTCTCTGTCTGATGTTTGTGGACGAG
TCGGACCACGAGACCCTGACGGCCATCTTGGGGCCTGTGGTGGCCGAGAG
GAACGCCATGAAGAACAGCCGCCTCATCCTGGCCATGGGCGGCCTCCCTC
GCTCCTTCCGCTTCCACTTCAGGGGCACAGGCTACGACGAGAAGATGGTG
CGCGAGATGGAGGGCCTGGAGGCTTCAGGCTCCACCTACGTCTGCACCCT
CTGCGACGCCACCAGAGCTGAGGCCTCCCAGAACATGGTGCTCCACTCAG
TCACCCGCAGCCACGACGAGAACCTGGACCGCTACGAGCTGTGGAGGACC
AACCCTTACTCTGAATCGGCGGACGAGCTGCGAGACCGGGTGAAAGGGGT
CTCCGCCAAGCCATTCATGGAGACCCAGCCCACATTGGACGCGCTGCACT
GTGACATCGGCAACGCCACTGAGTTCTACAAGATCTTCCAGGACGAGATC
GGGGAGGTGCATTGCAGGCC---CAAC---CCGAGCAGGGAGGAGCGTCG
GAGCTGGAGGGCGGCTCTGGACAAGCAGCTC-------------------

------------------TCATACACCATTGAGATGGGCCCCAAAGGGCC
CCAGTGGAAAGAGAGCCCTCAGCCTTTCTCCTGTTCTGTGGAGGACCCTA
CCAAACAGACCAAGTTCAAGGGCATCAAGACCTATATATCTTACCGGGTC
ACCCCCAGCCATACAGGGCGACCCGTCTACCGCCGGTACAAGCACTTTGA
TTGGCTGTACAACCGTCTGCTGCATAAGTTCACCGTCATCTCTGTGCCTC
ACTTGCCAGAGAAACAGGCCACAGGGCGTTTTGAAGAGGACTTCATCGAG
AAGCGCAAAAGACGGTTGGTCATCTGGATGGACCACATGACCAGTCATCC
TGTCCTTTCACAGTACGAGGGCTTGGAACACTTCCTCATGTGTGCTGATG
ACAAGCAGTGGAAGCTGGGCAAGCGGCGGGCGGAGAAAGATGAGATGGTG
GGTGCCCACTTCATGCTGACCTTTCAGATTCCCAACGAGCACCAGGATCT
ACAGGACGTGGAGGAGCGCGTGGATACCTTCAAGTCCTTTGCTAGGAAAA
TGGATGAAAGCGTCATGCAGCTGACCCACGTCGCCTCAGAACTGGTTCGA
AAACACCTGGGTGGATTCAGGAAGGAGTTCCAGCGCCTGGGAAATGCGTT
CCAGTCCATCAGCCAGGCATTCATGCTGGACCCTCCCCATCGGTCGGATG
CCCTCAACAACGCCATCTCCCATNNNNNNNNNNNNNNNNNNNNNNGACCTCCTGGGCTTTCATCATCGA
GGT
CGGAGTGGTCGGCAATCTTCTGATCTCCATCCTACTGGTCAAAGACAAGA
GCCTACACCGAGCGCCCTACTACTTCTTACTGGACCTGTGCGCCTCTGAC
ATCCTGCGTTCCGCCATCTGCTTCCCCTTCGTGTTCACCTCCGTCAAGAA
TGGTTCCACCTGGACGTACGGAACCCTAACCTGCAAGGTGATCGCCTTCC
TGGGCGTGCTGTCCTGCTTTCACACGGCCTTTATGTTGTTCTGTGTCAGC
GTGACCCGCTACCTGGCCATCGCCCACCATCGCTTCTACACCAAGAGGCT
GACCTTCTGGACGTGCTTGGCCGTCATCTGCATGGTGTGGACGCTGTCAG
TGGCCATGGCCTTCCCCCCGGTGCTGGATGTGGGGACGTACTCCTTCATC
AGAGAGGAAGACCAGTGCACCTTCCAGCACCGCTCCTTCAGAGCCAACGA
TTCCCTGGGCTTTATGCTGCTGCTCGCCCTCATCCTTCTGGCCACGCAGC
TAGTCTACCTCAAGCTCATCTTTTTCGTCCACGACCGCCGGAAGATGAAA
CCGGTCCAGTTTGTGCCGGCGGTCAGCCAGAACTGGACCTTCCACGGGCC
GGGGGCCAGCGGCCAGGCGGCGGCTAACTGGCTGGCGGGCTTTGGGAGAG
GCCCCACTCCGCCCACCCTTCTAGGCATCAGGCAGAACAGCAACGCGGCG
GGCCGCAGACGACTCCTGGTGCTGGATGAGTTCAAGACAGAGAAGAGGAT
CAGCCGGATGTTCTACATCATGACCTTCTTCTTCCTGGCACTGTGGGGGC
CCTATCTGGTGGCCTGCTACTGGAGAGTGTTCGCCAGGGGCCCCGCTGTG
CCGGCGGGCTACCTGACTGCCGCCGTGTGGATGAGCTTCGCCCAGGCGGG

GGTCAACCCCTTCATCTGCATCTTCTCCAACAGGGAGGCCAAATCTCGCT
TTCACCCTGGCGTAGGGACTGGTCCTGGCACGGACC---GCAGCGTCCCA
CTTAGTAACAGCTTGCTATCCCCGCAACAAACCGAAGAGCCCACAGTTG-
--CTTCCCCACAGCGTTGGTTTGTCACCC---CTGCCAACAACCGACTGG
ACTTTGCCGCCTCGGCATACGATGCTGCCGCTGCTGCAGATTTTGCCGGC
AACGCGGCCACCTTGCTGTCCTACGCAGCGGCTGGAGTGAAGGCGC----
--TTCCCCTCTCCACGGCAGGTTGCTCCAACAGACCGCTCGGGTATTACG
CCGACCCGTCAG---GCTGG---GGCGCCCGTACGCCACCACAGTACTGT
---------AGCAAGTCGAGCTCCGTTCTCTCATGCTGGCCCACGAATAC
TGTTACCGGCAGAATGGGCC---CGTCCAGTTACTTGG---------CCG
AGGA---GGGA---GACGC---CATTCCCACAGAGCGGTCTCCA---AT-
--AGGGGCGTCAGACGAG---GCAAAACCAAAAGACTT------GTCCGA
---ATCCAGCTGGATAGAG---ACGCCGTCTTCAATAAAGTCAATTGATT
CCAGTGATTCTGGAATCTTTG---AGCAAGCAAAACGGAGAAGAATTTCT
CCGTCTGCCACACCA---------GTTTCGGAGACAGTGTCCCCGTTAAA
ATCCGAG---------ACAGGCGAAGTCACAGAAAGAGAAGTGGCTTTGG
GGATAAATCCGTTCGCAGACGGGATGGGTGCTTTCAAAATCAACCACAGT
TCGCATGATCTTGGCTCCGG---GCAAACTGCATTTTCCTCCCAAGCG--
-CCCGGCTAC---GCAGCCGCTGCCCTGGGA---CATCACCA------CC
ACCCGACTCATGTCAGTTCT---TACTCTACGGCGGCTTTCAATTCCACC
CGGGATTTTCTCTTCAGAAATCGAGGCTTCGGAGACGCTACCAG------
------CGCTCAGCACAGTCTCTTCGCCTCCGC---AGCGGGAAGTTT--
-T------GCAGCCCCACATGGACACTCAGATGCAGCGGGACACCTGCTC
TTCCCAGGACTTCACGAG---CAAGCCGCCASCCATGCTTCCTCAAATGT
TGTTAATAGTCAGATGCGATTGGGCTTTTCGGGGGACATGTACGGCAGAG
CCGACCAGTATGGCCACGTTACCAGCCCGCGGT---CCGACCACTATGCT
TCGACCCAGTTGCATGGCTATGGCCCTATGAACATGAATATGGCCGCG--
-CATCATGGAGCAGGGGCCTTCTTCCGTTACATGAGGCAGCCGATAAAAC
AAGAGCTGATCTGCAAGTGGATCGAACCGGAGCAACTAACGAACCCAAAA
AAGTCGTGCAACAAAACTTTAGCACAATGCACGAGCTCGTCACCCATCT
GACTGTGGAGCATGTGGGAGGACCGGAGCAGTCGAACCACATTTGCTTCT
GGGAAGAGTGTGCCCGAGAAGGAAAACCATTCAAAGCCAAATACAAACTT
GTGAACCACATCAGAGTGCACACCGGAGAGAAACCATTTCCATGTCCATT
TCCAGGTTGTGGCAAA
>Hypoptychus dybowski
AGCCTTCTTATTCGAGCTGAGCTAAGTCAGCCTGGTGCTCTTCTTGGCGA
CGACCAGATTTACAATGTTATCGTTACTGCCCACGCTTTCGTTATAATCT
TCTTTATAGTCATGCCAATCATGATCGGAGGCTTCGGCAACTGACTTATC
CCTTTAATAATCGGGGCCCCTGATATAGCATTTCCTCGAATAAACAACAT
AAGTTTTTGGCTTCTTCCCCCTTCTTTTCTTCTCCTTTTAGCTTCTTCTG
GGGTTGAGGCGGGAGCTGGAACAGGTTGAACTGTTTACCCGCCCCTTTCT
GGAAACCTTGCCCACGCAGGTGCTTCAGTTGACCTAACAATTTTTTCCCT
GCACCTAGCTGGGATTTCTTCTATTCTAGGTGCTATCAATTTCATTACCA
CAATTATTAACATAAAACCCCCAGCTATTTCCCAATACCAAACACCCCTG
TTCGTTTGGTCTGTGCTGATTACTGCAGTCCTCCTTCTCTTGTCCCTCCC
GGTTTTAGCAGCCGGAATCACCATGCTCTTAACAGACCGTAACCTAAATA
CAACCTTCTTTGACCCTGCCGGAGGCGGAGACCCCATCCTTTACCAACAC
TTATTCTGATTCTTCGGTCA------------------------------

-------------------------GGATGATTACGTCGCCGTGTTCAGT
CGCTCAGCAACAAGGCTGATACTGAATGAAGCTGAGCTAATCCTGGCGCT
GGCCCAGGAGTTCCAGATGAGAGTGGTCACGGTGTCCCTGGAGGAACAGT
CTTTCCCCGCTATCGTCCAGGTGATTAGCGGCGCCTCCGTGTTGGTCAGT
ATGCACGGCGCTCAGCTCATCACCTCGCTCTTCCTCCCCAGAGGAGCCAC
TGTAGTAGAGCTGTTCCCCTTTGCTGTGAACCCAGAACAGTACACCCCGT
ATAAAACCCTAGCCACCCTTCCAGGAATGGACCTTCACTATATCTCCTGG
AGGAACACTAAGGAGGAGAACACCATCACCCACCCAGACAGACCCTGGGA
GCAAGGGGGCATCGCTCACTTGGAAAAGGAGGAGCAAGAGCGCATACTGG
CGAGCGGAGATGTCCCCAGGCACCTGTGCTGCCGCAACCCCGAGTGGCTC
TTCCGGATCTACCAGGACACTTTGGTGGACATCTCCTACTTCCTGGACGC
CCTTAG---AGAGGGCATGAAG---ACAAAGCCCAGCGGGAAGAA---GT
CAAAGCCGGCCAGCACAGTCCACCCGGGCCGAGTCAGGGAGCCACAGTGT
CAGACCTCGGTACAAACCACCAACGAGGCTAAACTCACAGTTTCCTGGCA
GATCCCGTGGAATCTGAAATACCTGAAAGTGAGAGAGGTGAAGTACGAGG
TGTGGATCCAGAAACGAGACACTAGCAAGGGGACGCTGGAGGACCAGATC
ATCCAGGCCAACCCGGCACTGGAGGCTTTCGGCAATGCCAAAACTCTGAG
GAATGACAACTCGTCCCGTTTTGGGAAATTCATCCGAATTCACTTCGGTG
CGAGCGGGAAGCTGTCATCTGCCGACGTCGAGACTTACCTGCTTGAAAAG
TCCCGGTGCACCTTTCAGCTCAAGGCTGAGAGGAACTACCACATCTTCTA
CCAGATCCTGTCCAATCAGAAGCCAGAGCTGTTGGACATGCTGCTGATCA
CCAACAACCCTTACGACTACTCGTACATCTCCCAGGGAGAGGTGACGGTG
GCCTCCATCAACGACTCGGAGGAGCTGCTGGCCACCGACAGCGCCTTCGA
CGTGCTGGGCTTCACTCCGGAGGAGAAGACGGGCGTCTATAAGCTCACCG
GGGCCATCATGCACTATGGCAACATGAGGTTCAAACAGAAGCAGCGCGAG
GAGCAGGCTGAGCCGGATGGAACGGAGTCCGCTGATAAAACAGCCTACCT
GATGGGGCTGAACTCTGCCGACCTCATCAAGGGCTTGTGTCATCCCAGGG
TTAAGGTAGGAAATGAATACGTCACCAAAGGTCAAAGTGTGGACCAAGTG
TACTACCCAAACAAGGAAGCCTTCAAGTGTGAAGAGTGCGGGAAGCACTA
CAACACCAAGCTGGGATACAAGCGCCATGTGGCCATGCACTCTGCCACGT
CCGGGGACCTCACGTGCAAAGTGTGCATGCAGAGCTATGAGAGCACACCC
GTGCTGTTGGAGCACCTCAAGACCCATTCCGGGAAGTCCTCGGGCGGGGC
CAAGGAGAAGAAGCACCCGTGCGACCACTGTGACCGCCGCTTCTACACGC
GCAAGGACGTGAGACGGCACATGGTGGTCCACACGGGCCGGAAGGACTTC
CTCTGCCAGTACTGTGCCCAGCGCTTCGGCAGGAAGGACCACCTGACCCG
CCACGTGAAGAAGAGCCACTCGCAGGAGCTGCTGAGGATCAAGACGGAGC
CTTCGGATATGTTGGTGCTTTTGGCGTCCGGGTCCCCGCCCTGCTCCGTG
AAGGAGGAGCTCAGCCCCATGATGTGCGGCATGGGTCCCAACAAAGACCC
CATGATGAGCAAAGCGTTCCCCGGCGGGGCCCCTTTTCCGATGGCCATGT

ACAACCCCCACCAT------CTCCAGGCTATGTCTAATTCAGGGGCGGGT
CAGCAG------CACGCGTCCCTGATGCCCGGCCCCTTGTCTGCAGCTAT
GGGTATGGGCTGCCACATGGAATATCTCATCTACGCCTCTTTTTCATTCA
TGGGATGTTTACAGATCAGCGATGGATCAAACATAGTGAACCTGTTGGCA
AGTAACTCTCCGAGTGTTTCGTACGCTATGACCCAGCAGAAATACTTCAG
TAACTACAGTCCTGTGATTGGCTTTTACATTTATGAGCCCATCGAGTACT
GGAACTCCACGGTGCAGGAGCACCTGAAGACCCTGAGTCATGGTTTCAAC
AAGATCTCTTGGATGGACAACTTTTTCCACTACCTGCGCGTAGTAAATGT
GAGCGCGTCGACCAAGAGTGACTTCATCTCCATTCTCAAGGGGTCCTTCC
TTCGCAGCCCGGAGTACCAACATTTCACTGAGGACATCATATTTTCCTTG
A---ACCGCGAGACAG------AAGAGTACGACATTATCGCTTCGCGGAT
GTACCTTGTGGCACGGACGACAGAGAAGAAGCGGGAGGAGGTGGTGGAGC
TTCTGGAAAAGCTTCGTCCTTTGATGCTGATCAACAGCATTAAGTTTATT
GCCTTCAATCCTACGTTTGTGTTCATGGACCGCTACAGCTCTTCTGTCAT
CTCGCCCATCCTGACCTCAGGCTTCAGCGTACTCACAATCCTCATCCTCA
CTTTCTTCCTGGTCATCAACCCGTTGGGTAACTTCTGGCTCATCCTCACG
GTAACGTCTGTGGAGCTTGGCGTCTTGGGCTTGACANNNNNNNNNNNNNNNNNNNNNNNNNCAGCWCTCA
AGAAT
GTGTCTACATCTTGCAATGTTGGCATTATYAATGGGCTCTCTGGATGGGC
TTCCTCGGTGGATGACATCCCAGCTGACACCATCACTCGGCGGTTTCGGT
ACGATGTGGCCCTGGTGTCAGCATTAAAGGATCTGGAGGAGGACATCATG
GAGGGGCTCAGAGAGAGTGGCATGGAAGACAGTGCTTGCACCTCAGGCTT
CAGTGTCATGATCAAGGAATCCTGTGATGGCATGGGCGATGTCAGCGAGA
AGCACGGTGGAGGACCACCTGTTCCTGAGAAGGCTGTGCGTTTTTCTTTC
ACTGTTATGTCCGTCTCCGTGCTGGCAGACGAGGAGGTG-----------
-------------------GAAGAGGTTACCATCTTTACTGAGCCAAAGC
CAAACTCAGAACTGTCCTGTAAGCCCCTCAGCTTGATGTTCGTGGATGAG
TCAAACCATGAGACACTCACAGCTGTCCTGGGGCCAATAGTTGCAGAGCG
TAATGCAATGAAAGAAAGCAGGCTCATTCTCTCCGTGGGCGGACTACCTC
GCTCCTTCCGCTTTCACTTCAGAGGCACAGGATATGATGAGAAAATGGTG
CGCGAGATAGAGGGCCTGGAGGCCTCAGGGTCCACCTACGTCTGCACTCT
TTGTGACTCCACGCGGGCAGAGGCTTCTAAAAACATGGTGCTACACTCCA
TCACTCGCAGTCATGAAGAGAACCTAGATCGTTACGAAATATGGAGAACC
AACCCATTTTCTGAATCTGTAGATGAACTGCGGGACAGAGTCAAAGGGAT
CTCTGCCAAACCCTTCATGGAGACCCAGCCCACGCTGGATGCATTACACT
GTGACATTGGCAATGCCACTGAGTTCTACAAAATCTTCCAGGACGAGATC
GGGGAGGTATACCAAAAGGT---CAAC---CCTAGTCGGGAGGAACGGCG
GAGCTGGAGGGCAGCGCTGGATAAACAGCTCAGGACGAAGATGAAACTTA
AACCGGTGATGAGGATGAATGGGAACTACGCCCGCAGGCTAATGACTCAA
GATGTGGTAGAGGTGATTTGTGAGCTGGTGCCGTCAGAGGAGAGGAGAGA
GGCCCTGAGGGAGCTGATGAGGCTCTACATCCAAATGAAGCCTGTGTGGC
GCGCCACCTGCCCTGCCAAGGAGTGCCCCGACGAGCTGTGCCGCTACAGC
TTTAACTCCAAACGCTTTGCCGACCTTCTCTCCTCTACCTTCAAATATAG
GTATAATGGAAAGATAACCAACTACTTGCACAAGACGTTGGCCCATGTGC
CTGAAATCATAGAGAGANNNNNNNNNNNNNNNNNNNNNNNNNNNNNNNNNNNNNNNNNNNNNNNNNNNTC
GTACACCATCGAGATGGGGGCCCCGGGGCCC
CTGTGGAAGGAGAGCCCGCGGCCGTTCTCCTGCTCCATCGAGGACCCCAT
CAAACAGACAAAGTTCAAGGGCATCAAGACGTACATCTCGTACCGGGTGA
CGCCGAGCCACACGGCGCGCCCCGTCTACCGGCGCTACAAGCACTTCGAC
TGGCTGTACAACCGGCTGCTGCACAAGTTCACCGTGATCTCCGTGCCCCA
CCTGCCCGAGAAGCAGGCCACGGGGCGCTTCGAGGAGGACTTCATCGAGA
AGCGCAAGCGGCGGCTGATCCTGTGGATGAACCACATGACCAGCCACCCG
GTGCTCTCCCAGTACGAGGGCTTCGAGCACTTCCTGATGTGCGCCGACGA

CAAGCAGTGGAAGCTGGGCAAGAGGCGGGCGGAGAAGGACGAGATGGTGG
GCGCCCACTTCATGCTGACCCTGCAGATCCCCGGCGAGCACCAGGACCTG
CAGGACGTGGAGGAGCGCGTCGACACCTTCAAGGCCTTCGCCAAGAAGAT
GGACGACAGCGTGATGCAGCTGACGCACGTCGCCTCGGAGCTGGTGCGCA
AGCACCTGGGCGGCTTCAGGAAGGAGTTCCAGCGGCTGGGCAGTTCCTTC
CAGTCCGTCAGCCAGGCGTTCACGCTGGACCCGCCCAACAGCTCGGAGGG
CCTCAACAAAGCCATGTCGCACNCTCTCGCCACGTTCCTCAAACTGACCTC
TCTGGGTTTCATCATTGGAGTCGGTGTGGTCGGAAACCTCCTGATCTCCA
TCCTGCTCGTCAAAGACAAGAGTCTCCACCGAGCGCCCTACTATTTCCTG
CTGGACCTGTGCGCCTCTGACATCCTGCGCTCTGCCATCTGCTTCCCCTT
TGTCTTCACCTCGGTCAAAAATGGATCCGCCTGGACCTATGGCACGCTGA
CCTGCAAAGTGATCGCCTTCCTSGGTGTGCTTTCCTGTTTCCACACGGCG
TTTATGCTGTTCTGCGTCAGCGTCACGCGCTACCTGGCCATCGCACACCA
CCGCTTCTACACCAAGAGGCTGACCTTCTGGACCTGCCTAGCCGTCATCT
GCATGGTGTGGACGTTGTCGGTGGCTATGGCGTTCCCGCCGGTGCTCGAC
GTAGGGACGTACTCTTTCATCCGGGAGGAGGACCAGTGCACGTTCCAGCA
CCGTTCCTTCAGGGCGAATGATTCGCTGGGCTTCATGCTCCTGCTGGCGC
TCATCCTCCTGGCCACGCAGCTGGTCTACCTCAAGCTCATTTTCTTTGTC
CACGACCGTCGGAAGATGAAGCCCGTCCAGTTTGTGCCTGCTGTTAGCCA
GAACTGGACCTTCCACGGGCCAGGCGCCAGCGGGCAGGCGGCCGCCAACT
GGCTGGCCGGATTTGGTCGAGGCCCCACCCCGCCTACACTGCTGGGCATC
CGGCAGAACAGCAACGCAGCGGGCCGCAGGCGTCTACTGGTATTGGATGA
GTTCAAAACAGAGAAGAGGATTAGTAGGATGTTCTACATCATGACGTTTT
TCTTTCTGGCGCTTTGGGGGCCCTACCTGGTCGCCTGCTACTGGCGGGTG
TTTGCAAGGGGCCCCGTGGTCCCTGGGGGCTACCTGACGGCCGCCGTGTG
GATGAGCTTTGCCCAGGCTGGGGTCAATCCTTTCATCTGCNNNNNNNNNNNNNNNNNNNGCCAAATCTC
GCTTTCACCCTGGCGTGGGGAGCGCTCCTGGCACGGAGC---GCAGCGTC
CCACTCTGCAACAGCTTGCTCTCCCCGCAGCAAAGCGACGAGCCCCCTGC
TGCCACCCCCCCGCAGCGATGGTTTGTCACCC---CTGCCAACAACCGAC
TGGACTTTGCTGCCTCGGCATACGACGCCGCC---------GATTTCGCC
GGCAACGCGGCCACCTTGCTGTCCTACGCGGCGGCCGGAGTGAAGGCTC-
-----TTCCCCTGCCGGCGGCGGGCTGCTCCAACCGGCCTCTTGGYTATT
ACGCAGACCCGTCGG---GCTGG---GGAGGACGCACGCCGCCGCAGTAC
TGTGGCGTCAACAGCAAATCCAGCTCGGTGTTTTCCTGCTGGCCCGCGAA
CTCGATCGGTGGAAGAGCGGCCG---CC---GGCTACCTGG---------
CCGAGGA---GGGG---GACTC---CATCGCCACGGAGAGGTCGCCG---
AT---CGTCGGCTCGGACGAG---GCCAAAGCCAAAGACATGAC---GTC
CGA---GTCGAGCTGGATAGAG---ACGCCGTCCTCCATCAAGTCGATCG
ATTCGAGTGATTCTGGTATCTTTG---AACAGGCCAAAAGGAGGAGGATC
TCACCTTCTGCCACGCCG---------GTTTCAGAGACAGTGTCCCCGTT
GAAATCTGAGCATCACTCAACAGGCGAAGTCACGGAGAGAGAAGTGGCGT
TGGGGATCAATCCGTTCGCGGATGGGATGGGCGCCTTCAAAATAAACCAC
AGCTCCCACGACATCGGCTCCGG---ACAAACGGCGTTCTCCTCCCAGGC
A---CCCGGCTAC---GCAGCGGCCGCCCTGGGA---CACCATCA-----
-CCACCCGACCCACGTTGGCTCT---TACTCCACGGCGGCTTTCAACTCC
ACCAGGGACTTTCTGTTCAGAAATCGGGGTTTCGGGGACGCCGCCGG---
---------GGCGCAGCACAGTTTGTTCGCCTC---------AGGGAGTT
T---C------GCGGGGCCACACGGACACTCGGACGCGGCGGGCCACCTG
CTCTTCCCGGGACTCCACGAG---CAAGCGGCGAGCCATGCGTCCTCCAA
CGTGGTCAACGGCCAGATGCGCCTGGGCTTCTCGGGGGACATGTACGGGA
GGGCCGAGCAGTACGGCCACGTCACAAGCCCGCGGT---CCGACCACTAC
GCTTCGACCCAGCTGCACGGCTACGGCCCCATGAACATGAATATGGCCGC
G---CACCACGGAGCGGGGGCCTTCTTTCGATACATGAGGCAGCCGATCA

AGCAAGAGCTCATCTGCAAGTGGATCGAGCCGGAGCAGCTGACGAATCCT
AAAAAGTCGTGCAACAAAACTTTTAGCACGATGCACGAGCTCGTGACCCA
TCTGACGGTGGAGCATGTGGGGGGACCGGAGCAGACCAACCACATCTGCT
TCTGGGAGGAATGCGCCAGAGAAGGGAAGCCGTTCAAAGCCAAATACAAA
CTTGTAAATCATATCAGGGTACACACCGGAGAGAAGCCCTTCCCGTGTCC
GTTCCCCGGCTGNNNNNNN
>Icichthys lockingtoni

------------TTCCTAGAGAGGAACCTTCACCCGACTAATTGCCTTGG
CATGCTGTTGCTGTCTGACGCTCACCAGTGCACCAAGCTGTCAGAGCTCT
CTTGGGGCATGTGCCTCAGCAACTTCCCCACTATTTGCAAGACAGAGGAC
TTCCTCCAACTGCCCAAAGATATGGTGGTGCAGCTTTTGTCACATGAGGA
GCTGGAGACAGAAGATGAGAGACTGGTTTATGAAGCTGCCCTGAACTGGA
TCAACTATGACCTGGAAAGGAGGCACTGCCACCTTCCAGAGCTCCTGAGA
ACGGTCCGTCTTGCCCTGCTGCCCGCCATCTTTCTGATGGAGAATGTCTC
GACAGAAGAGCTGATCAACGCCCAGGCCAAGAGCAAGGAGCTGGTGGATG
AGGCTATCCGCTGTAAGCTGAAGATCCTKCAGAATGACGGCGTTGTTAAC
AGCCCGTGTGCTCGACCAAGAAAAACCAGCCATGCTCTCTTTCTCCTGGG
AGGGCAGACTTTCATGTGTGACAAGTTGTACCTGGTGGACCAGAAGGCCA
AAGAGATYATCCCGAAGGCGGACATTCCCAGCCCAAGGAAGGAGTTCAGC
GCCTGCGCCATCGGCTGTAAGGTGTACATCACAGGTGGGA--GAGGCTC-
AGAGAATGGTGTGTCCAAAGATGTATGGGTCTATGACACCGTCCAGGAGG
AATGGTCCAAAGCGGCGCCCATGCTCATCGCCAGGTTTGGTCATGGCTCT
GCAGAGCTGAAACACTGCCTGTAYGTGGTAGGAGGTCACACCGCAGCAAC
TGGCTGCCTCCCTGCTTCTCCATCCGGATGAATACATCGTTGTGTTCAGT
CGTTCAACAACAAGGCTGATACTGAACGAAGCCGAGCTAATCATGGTGCT
GGCCCAGGAGTTCCAGATGAGAGTGGTCACAGTTTCCCTAGAGGAACAGT
CTTTCCCCAGCATTGTCCAGGTGATCAGCGGTGCTTCCATGTTAGTCAGC
ATGCATGGAGCTCAGCTTATCACCTCACTCTTCCTCCCCAGAGGAGCTGC
TGTGGTGGAGCTGTTCCCCTTTGCTGTGAACCCAGAGCAGTACACCCCAT
ATAAAACCCTTGCCTCCCTTCCAGGCATGGACCTTCATTATGTTTCCTGG
AGGAACACTCAGGAGGAGAACACTGTCACCCATCCAGACAGACCCTGGGA
ACAAGGAGGCATCACTCACTTGGAGAAGGAGGAGCAGGAGCGAATTCTGA
CCAGCAAAGACGTCCCCAGGCACTTGTGCTGCCGCAATCCGGAGTGGCTC
TTCCGAATCTACCAGGACACTTTGGTAGACATCCCTTCCTTCCTGGAAGT
CCTCAA---GGAGGGAATGAAG---ACCAAGCCCAGCTTGAAGAA---GT
CCAAACCAGCCAGTACAGTCCATCCGGGCCGGGTAAGACAAGCCCAGTGT
CAGACCTCAGTACAAACCACCAATGAGGCTAAACTCACAGTCTCCTGGCA

GATCCCGTGGAATCTGAAATACCTGAAAGTAAGAGAGGTGAAGNNNNNNNNNNNNNNNNNNNAAAAGAG
ACACCAGCAAGGGAACTCTGGAGGATCAAATCATCCAGGCGAACCCGGCG
CTGGAGGCCTTCGGAAACGCCAAAACGCTGAGAAACGACAACTCGTCCCG
TTTTGGAAAATTCATCCGAATTCACTTCGGAAACAGCGGCAAGCTGTCGT
CCGCTGACATCGAGACGTNCNTGCTGGAGAAGTCTCGTGTCACCTTTCAGCT
CAAGGCTGAGAGGGANTACCACATCTTTTACCAGATCNTGTCCAATCAGAAG
CCAGAGCTGCTGGACATGCTGTTAATCACCAACAACCCGTACGACTACTC
CTACATCTCCCAAGGAGAGGTAACGGTCACCTCCATCAACGACTCGGAGG
AGCTGATGGCCACCGACAGTGCCTTCGATGTGCTCGGCTTCACTCCGGAG
GAGAAGATGGGCGTNTATAAACTGACCGGNGCCATCATGCACTACGGCAACA
TGAAGTTCAAGCAGAAGCAGCGTGAGGAGCAGGCTGAGTCTGACGGGACG
GAGGCTGCTGATAAATCAGCTTACTTGATGGGGCTGAACTCCGCTGACCT
CATCAAAGGGCTGTGCCATCCCAGAGTCAAGGTAGGAAACGAGTACGTCA
CCAAAGGCCAGAGTGTGGACCAAGTCTACTACCCCAACAAGGAGGCCTTC
AAGTGTGAGGAGTGTGGGAAGCACTACAACACCAAGCTGGGATATAAGCG
ACATGTGGCCATGCACTCTGCCACGGCAGGGGATCTCACCTGTAAAGTGT
GCATGCAGAGCTACGAGAGCACACCTGTTCTCCTGGAGCACCTCAAGAGC
CACTCGGGGAAGTCCTCGGGCGGAGCCAAGGAGAAAAAGCACCCGTGCGA
CCACTGCGACCGACGTTTCTACACGCGGAAGGATGTGAGACGGCACATGG
TGGTCCACACGGGCCGAAAGGACTTCNTGTGCCAGTACTGTGCCCAGCGCT
TTGGCAGGAAGGATCACCTGACCCGCCATGTGAAGAAGAGCCACTCGCAG
GAGCTGCTGAAGATCAAGACGGAGCCTCCTGATATGTTAGGTCTTTTAGC
TTCGGGGTCACCACCTTGCTCTGTGAAGGAGGAGCTTAGCCCCATGATGT
GCGGCATGGGTCCCAACAAAGACCCCATGATGGGCAAACCGTTCCCCAGT
GGTGCCCCTTTTCCTATGGGCATGTACAACCCCCACCAC------CTTCA
GGCCATGTCTAATTCTGGGGTGGGTCATCCA------CACCCGTCCCTGA
TGCCCAGCCCCCTGTCTGCAGCTATGGGCATGGGCTGTCACATGGAATAT
CTCATCTACGCCTCTTTTTCATTCATGGGATGTTTACAAATCAGTGATGG
ATCAAACATTGTGAACCTGCTGACTAGTAACTCTCCAAGTGTTTCGTACG
CTCTGACCCAGCAGAAATACTTCAGCAACTACAGTCCTGTGATTGGGTTT
TACATTTATGAGCCCATTGAGTACTGGAACTCCACGGTGCAGGAGCACCT
GAAGACTCTGAGTCACGGCTTCAACAAGATCTCCTGGATGGACAACTTTT
TCCACTACCTGCGGATGGTGAATGTGAGTGCGTCGACCAAGAACGACTTT
ATCACCATCCTCAAGGGCTCCTTCCTGCGCAGCCCAGAGTACCAGCACTT
CACTGAGGACATCATATTCTCCAAGA---ACCGTGAGACTG------ACG
AATACGACATTATCGCCTCACGCATGTATTTGGTGGCTCGGACCACAGAG
AAGAAGCGCGAGGAGGTGGTTGAGNTTNTGGAGAAGCTTCGCCCATTGATGC
TGATCAACAGCATCAAGTTCATTGCCTTCAATCCCACGTTTGTGTTCATG
GACCGCTACAGCTCCTCCGTCATCTCGCCCATCCTTACCTCAGGCTTCAG
CGTGCTCACCATCCTTATCCTCACTTTCTTCCTGGTCATCAACCCCTTGG
GGAACTTCTGGCTCATCCTCACGGTTACGTCCGTGGAGCTGGGCGTCTTG
GGTTTNNNNGGCTTTCACCAGTTTGAGTGGCAGCCAGCTCTCAAGAATGTGTCT
GCATCTTGCAATGTTGGCATTATTAATGGGCTCTCTGGGTGGACTTCCTC
GGTGGATGACTCCCCAGCTGACACCATCACTCGACGGTTTCGCTATGATG
TGGCACTAGTGTCAGCATTAAAGGATCTGGAGGAGGACATCATGGAAGGG
CTGAGAGGTAGCGGGATGGAAGACAGTGTTTGCACGTCAGGCTTCAGTGT
TATGATCAAGGAATCTTGTGATGGCATGGGTGATGTCAGCGAAAAGCACG
GTGGAGGACCAGTTGTTCCTGAGAAGGCTGTACGTTTCTCTATCACTATT
ATGTCTGTCTCTGTCCTGGCAGATGACGAGGAG-----------------
-------------GAGGCGGTTACTATCTTCACAGAGCCAAAGCCAAACT
CAGAACTGTCCTGTAAGCCCCTTTGCCTGATGTTTGTGGATGAATCAGAC
CATGAGACACTCACAGCTCTCCTGGGGCCTGTAGTTGCAGAGCGTAATGC
AATGAAAGAGAGCAGGCTCATCCTTTCCATTGGCGGCCTGCCTCGCTCCT

TCCGCTTCCACTTCAGAGGCACGGGATACGATGAGAAGATGGTGCGAGAG
ATGGAGGGCCTGGAGGCCTCAGGGTCCAGCTATGTCTGCACTCTGTGTGA
TTCCAGTCGGGCAGAAGCATCTGAAAACATGGTGCTACACTCCATCACCC
GCTGCCATGACGAGAACCTTGAACGTTACGAAATATGGAGAACCAACCCC
TTTTCTGAATCTGGAGAAGAGCTGCGAGATAGAGTCAAAGGGGTCTCTGC
CAAGCCCTTCATGGAGACCCAGCCCACGCTGGATGCATTACATTGTGACA
TTGGCAATGCCACTGAGTTCTACAAAATCTTCCAGGACGAAATTGGGGAG
GTGTACCAAAAGGT---CAAT---CCCACCCGGGAGGAACGGCGCAGCTG
GAGGGCAGCCCTAGATAAACAGCTGAGGAAGAAGTTGAAGCTTAGACCAG
TAATGAGGATGAATGGGAACTATGCCCGCAGGCTAATGACCCAGGAGTCT
GTGGAAGTGGTGTGTGAGCTGGTACCCTCAGAGGAGAGGAGGGAGGCCCT
GAGGGAGCTTATGAGGCTATACCTCCAGATGAAGCCTGTGTGGCGTGCCA
CCTGCCCAGCCAAGGAGTGCCCTGACCAGCTGTGCCGCTATAGCTTTAAC
TCCCAGAGCTTTGCCGACCTCCTCTCCTCTACCTTCAAATATAGGTACAA
TGGAAAGATAACCAATTACCTGCACAAGACCCTGGCCCATGTCCCTGAAA
TCATAGAGAGAGAGATGGATCCATCGGAGCCTGGGCCAGTGAGGGGAACGAG
TCGGCAAACAAANNNNNNNNNNNNNNNNNNNNNNNNNNNNNNNNNNNNNNNNNNNNNNNNNNNNNNNNN
NNNNNNNNNNNNNNNNNNNNNCCACAAAACAGACAAAGTTCAAAGGCATCAAGACGTAC
ATTTCATACCGGGTCACGCCGAGCCACACAGGGCATCCCGTCTACAGGCG
CTACAAACACTTTGACTGGCTGTACAACCGCCTGCTGCACAAGTTCACTG
TGATCTCCGTGCCTCACCTGCCTGAGAAGCAGGCCACGGGGCGATTTGAG
GAAGACTTTATTGAGAAGCGGAAGAGACGACTGATACTGTGGATGAACCA
CATGACCAGTCACCCTGTCCTCTCCCAGTATGAAGGCTTTGAGCACTTTC
TGATGTGCGCTGACGACAAGCAATGGAAGCTGGGCAAGAGACGAGCTGAG
AAGGAAGAGATGGTGGGCGCCCATTTCATGCTGACCCTCCAGATCCCTAA
CGAGCACCAGGACCTTCAGGATGTAGAGGAGAGAGTCGACACCTTCAAGG
CCTTTGCTAAGAAAATGGACGACAGTGTCATGCAGCTCACACATGTTGCC
TCGGAGCTGGTGCGTAAGCACCTAGGTGGATTCAGGAAGGAGTTCCAGCG
GCTGGGAAATGCCTTCCAGTCTATTAGTCAGGCATTCATGCTGGACCCTC
CCCATAGCTCAGACACCCTCAACAACGCCATCTCCCATNNNNNNNNNNNNTTCCTCAAGCTG
ACCTCTCTGGGTTTCATCATTGGAGTCGGTGTGGTTGGAAACCTCCTGAT
CTCCATCCTGCTGGTCAAAGACAAGAGCCTGCACCGAGCACCCTACTATT
TCCTGCTGGACCTGTGCGCCTCCGATATCCTCCGCTCTGCCATCTGCTTC
CCCTTTGTCTTCACCTCGGTCAAGAATGGATCTGCCTGGACCTATGGCAC
GCTGACCTGCAAAGTGATCGCCTTCCTGGGTGTGCTCTCCTGTTTCCACA
CGGCGTTCATGCTATTCTGCGTGAGCGTCACCCGCTACCTGGCCATCGCA
CATCACCGTTTCTACACCAAGAGGCTGACCTTCTGGACCTGTCTGGCTGT
CATCTGCATGGTGTGGACGTTGTCAGTAGCTATGGCGTTCCCGCCGGTGC
TAGACGTGGGGACGTACTCTTTTATCCGGGAGGAGGACCAGTGCACATTC
CAGCACCGCTCCTTCAGGGCGAATGATTCGCTGGGCTTCATGCTCCTGCT
GGCGCTCATTCTCCTGGCCACACAGCTGGTTTACCTCAAGCTCATCTTCT
TCGTCCACGACCGTCGAAAGATGAAGCCTGTCCAGTTCGTGCCTGCTGTC
AGCCAGAACTGGACCTTCCACGGGCCAGGCGCCAGCGGGCAGGCGGCGGC
CAACTGGCTGGCCGGATTTGGTAGAGGCCCCACCCCGCCTACTTTGCTGG
GCATCCGGCAGAACAGCAACGCAGCGGGCCGCAGGCGTCTACTGGTATTG
GATGAATTCAAAACAGAGAAGAGGATTAGTAGGATGTTCTACATCATGAC
GTTTTTCTTCCTGGCACTGTGGGGGCCCTATCTGGTAGCCTGCTACTGGC
GGGTGTTTGCAAGGGGCCCTGTAGTCCCTGGGGGCTACCTGACGGCAGCC
GTGTGGATGAGCTTTGCCCAAGCTGGGGTCAATCCTTTCATCTGCNNNNNNNNNNNNNNNNNNNGCCAA
ATCTCGCTTTCACCCTGGCGTGGGGACTGGTCCTGGCACGGAGC---GCA
GCGTCCCACTCGGCAACAGCTTGCTATCCCCGCAGCAAACCGAGGAGCCC
ACTGTTGCCACCCCCCCGCAGCGATGGTTTGTCACCC---CTGCCAACAA
CCGACTGGACTTTGCTGCCTCGGCATACGACGCCGCT---------GATT

TCGCCGGTAACGCGGCCACCTTGCTGTCCTACGCAGCGGCCGGAGTGAAA
GCTC------TTCCCCTGCCGACTGCAGGCTGCTCCAACCGGCCTCTTGG
CTATTACGCAGACCCGTCAG---GCTGG---GGAGGACGCACGCCGCCGC
AATACTGCGGCGTAAATAGCAAATCCAGCTCGGTCTTTTCCTGCTGGCCC
GCTAACTCTATCGGCGGCAGAGCAGGCA---CC---AACTACCTGG----
-----CCGAGGA---GGGA---GACTC---CATCCCAACGGAGAGGTCAC
CC---AT---C---GGCTCTGAGGAG---AGCAAACCCAAAGACATGAC-
--ATCAGA---GTCGAGCTGGATAGAG---ACGCCGTCCTCCATTAAGTC
AATTGATTCAAGCGATTCTGGTATCTTTG---AACAGGCCAAGAGGAGAA
GAATCTCACCTTCTGCCACCCCG---------GTTTCAGAGACAGTGTCC
CCGNNNNNNNNNNNNNNNNNNNNNNNNNNNGAAGTCACAGAGAGAGAAGTGGCGTTGGGATAAATCCGT
TTGTGGA
TGGGATGGGCGCCTTCAAAATAAACCACAGCTCCCACGATATTGGCTCCG
G---ACAAACGGGGTTTTCTTCCCAGGCA---CCCGGCTAC---GCAGCA
GCCGCCCTGGGA---CACCACCA------CCACCCGACCCACGTTGGCTC
T---TACTCCACGGCGGCTTTCAACTCCACCAGGGACTTTCTCTTCAGAA
ATCGGGGTTTCGGGGACGCCACCGG------------GGCGCAGCACAGT
TTGTTCGCCTC---------CGGAAGTTT---C------GCAGGGCCACA
TGGACACTCAGATGCAGCAGGGCACCTGCTCTTCCCAGGGCTCCACGAG-
--CAAGCGGCGAGCCATGCGTCTTCCAACGTGGTCAACAGCCAGATGCGG
CTGGGCTTCTCGGGGGACATGTACGGACGGGCCGACCAGTACGGCCACGT
TTCAAGCCCGCGGT---CCGACCACTATGCTTCGACCCAGTTGCACGGCT
ATGGCCCCATGAACATGAATATGGCCGCA---CACCACGGAGCAGGGGCC
TTCTTTCGATACATGAGGCAGCCGATCAAACAAGAGCTCATCTGCAAGTG
GGTCGAGCCGGAGCAGCTGACGAACCCCAAAAAGTCGTGCAACAAAACTT
TTAGCACGATGCACGAGTTAGTGACCCATCTGACGGTGGAGCATGTGGGG
GGACCAGAGCAGACCAACCACATCTGCTTCTGGGAAGAGTGCTGCAGAGA
AGGAAAGCCATTCAAAGCCAAATACAAACTTGTAAATCATATCAGAGTAC
ACACCGGANNNNNNNNNNNNNNNNNNNNNNNNNNNNNNNNNNNNNNN
>Ijimaia loppei

-------------------------GGACGAGTACATAGTGGTGTTCAGT
CGCTCCTCTACGAGGCTGATTCTGAACGAGCCAGAGCTAATCATGGTGTT
GGCGCAGGAGTTTCAGATGAGGGTGGTCACGGTGTCTCTGGAAGAGCAAT
CCTTCCCCAGTATCATCCAGGCGATCAGCGGCGCCTCCATGCTGGTCAGT
ATGCACGGAGCTCAGCTTGTTACCTCCCTCTTCCTCCCCAGAGGGGCTGC
CATTGTGGAGCTCTTCCCCTATGCTGTGAACCCAGAGCAGTATACTCCGT
ACAAAACCCTGGCCTCCCTCCCAGGCATGGATCTTCAATACGTTTCCTGG
AGAAACACAATCGAAGAGAACTCGGTTAGCCACCCCGATAGGTCCTGGGA
CCAAGGAGGGATTGCTCACCTAGACAAGGAAGAGCAGGAGAGAATTCTCG
CCAGCAAGGAGGTCCCCAGGCACCTGTGCTGCCGCAACCCCGAGTGGCTC
TTCCGAATCTACCAGGACACTCTGGTGGACATCCCCTCATTCCTCAAGAC
CCTCAA---GGAGGGCCTAAAG---ACCAAGCCCAGCTTGAAGAA---GT
CCAAGTCGGCCAGCACTGTTCACCCGGGCCGGGTCAGGGAAGCCCAGTGC
CAGACCTCGGTCCAGGACACCAACGAGGCTAAACTCACTGTCTCCTGGCA
GATCCCGTGGAATCTCAAGTACCTGAAGGTACGAGAGGTTAAGTACGAGG
TCTGGATCCAGAAGAAAGATTCAAGCAAAGGGACACTGGAGGATCAAATC
ATTCAGGCAAACCCTGCACTGGAGGCTTTTGGTAATGCCAAAACAGTGAG
GAATGATAACTCATCCCGCTTTGGGAAATTCATCCGAATTCACTTTGGAA
CCAGTGGCAAATTGTCCTCTGCCGACATTGAGACTTATCTCCTGGAGAAG
TCACGTGTTACCTTTCAGCTCAAGGCTGAGAGGAACTATCACATCTTCTT
CCAGATCCTGTCCAATCAAAAGCCAGAGCTGTTGGACATGATGTTGGTCA
CCAACAACCCATATGACTACTCCTTCATCTCCCAAGGAGAGGTAACAGTA
TCATCCATCAATGATGCAGAGGAGCTGATTGCCACTGACAGTGCCTTTGA
TGTCCTCGGCTTCACCCAGGAGGAGAAGCTGGGAGTTTACAAGTTGACAG
GTGCGATCATGCACTATGGCAACATGAAGTTCAAGCAAAAGCAGCGTGAA
GAGCAGGCTGAGTCTGATGGCACAGAGGCTGCTGATAAGTCAGCTTATCT
AATGGGGCTGAATTCTGCCGACCTCATTAAAGGGCTTTGCCATCCCAGAG
TCAAAGTAGGAAATGAGTACGTCACCAAAGGCCAAGGTGTAGATCAGGTT
TACTACCCCAACAAGGAGGCCTTCAAGTGTGAGGAGTGTGGTAAGCACTA
TAACACCAAGCTGGGATACAAGCGCCATGTGGCCATGCACTCGGCCACGG
CGGGCGACCTCACCTGTAAAGTGTGTATGCAGAGCTACGAGAGCACGCCG
GTCCTCCTGGAACACCTCAAGAGCCACTCTGGGAAATCTTCTGGTGGAGC
CAAGGAGAAAAAGCACCCATGTGATCACTGTGACCGTCGGTTCTACACCC
GGAAGGACGTGAGGCGGCACATGGTAGTCCACACGGGCCGAAAGGACTTC
CTGTGTCAATACTGCGCCCAGCGCTTTGGCCGGAAGGATCACCTGACGCG
ACACGTGAAGAAGAGCCACTCACAGGAGTTGCTGAAGATAAAGTCAGAGC
CTCCGGATATGCTGGGCCTCCTGGGCTCCGGCTCGCCGCCTTGTGCAGTT
AAGGAGGAGCTTAGCCCCATGATGTGCAGCGTGGGTCCCAACAAAGACCC
CATGATGGGGAAACCCTTCCCCAGCGGGACCCCTTTCCCCATGGGCATGT
ATAACCCGCACCAC------CTCCAGGCCATATCCAGCCCTGGGGTGGGC
CACCCC------CATCCCTCCCTGATGCCTAGTTCCCTGTCAGCGGCTAT
GGGCATGGGCTGTCATATGGAATATCTCATTTACGCTTCCTTCTCCTTCA
TGGGATGTTTACAAATCAGTGATGGGTCCAACATTGTGAATTTATTAGCT
AGTAATTCTCTGAGCGTCTCATATGCTCTGACCCAGCAAAAGTATTTCAG

```
TAACTATAGTCCCGTCATTGGGTTTTACATCTACGAGCCCATTGAGTACT
GGAACTCAACAGTGCAGGAGCACCTGAGGACACTGAGCGATGGTTTCAAC
AAGATCTCTTGGATGGACCACTTCTTCCACTACCTGCAGGTGGTGAATGT
GAGCGCGTCAACCAAGAGTGACTTTATCGGCATCCTCAAAGGCTCCTTCT
TGAGGAACCCGGAGTACCAGCACTTCATGGAGGACATCATCTTCTCCAAG
A---ACCGGGAGAGCG------ATGAGTACGACATCATCGCCTCACGCAT
GTACCTGGTGGCGCGCACCACGGAGAAGAGGCGCGAGGAGGTGGTTGAAC
TTCTGGAGAAGTTGCGACCGCTGATGCTCATCAACAGCATCAAGTTCATT
GCTTTCAACCCGACCTTTGTCTTCATGGACCGCTACAGCTCCTCCGTCAT
CTCACCCATCCTCACCTCGGGCTTCAGCGTCCTCACCATCCTCATCCTCA
CTTTCTTCCTGGTCATAAACCCGTTGGGGAACTTTTGGCTCATCCTGACG
GTCACATCCGTGGAGCTGGGAGTTTTGGGTTTAATG--------------
--------------------------------------------------
--------------------------------------------------
--------------------------------------------------
--------------------------------------------------
--------------------------------------------------
-------------------GCGAGAAGCACGGAGGAGGGCCGGCGATTCC
CGAGAAGGCGGTCCGCTTCTCCTTCGCCATTATGTCAGTCTCTGTCCGGG
TCGAAGGAACGGAA------------------------------GAGGCG
GTCACCGTCTTCAGGGAGCCGAAGCCCAACTCTGAATTGTCCTGTAAACC
CCTCTGTTTGATGTTTGTGGATGAGTCTGACCATGAGACCCTGACGGCCA
TTCTGGGGCCTTTGGTGGCCGAGCGGAATGCGATGAAGCAGAGCTGCCTG
ATCCTGTCATTGGGCGGCCTCCCGCGCTCTTTCTCCTTTGAGTTCCGAGG
CACGGGCTACGACGAGAAGATGGTGCGAGAGTTGGAGGGTATGGAGGCCT
CTGGTTCCACCTACGTCTGCACCCTGTGCGACTCCACCCGAGCGGAGGCC
TCTCGAAACATGGTGCTCCACTCCATCACCCGGAACCACGGCGAGAACCT
GGAGCGGTACGAGATGTGGAGGAGCAACCCTTTCTCGGAGTCGGCCGAGG
AGCTGCGGGAGCGGGTCAAGGGCATCTCTGCCAAACCCTTCCTGGAGACC
CAGCCCACTTTGGATGCATTGCACTGCGACATTGGCAACGCCACTGAGTT
TTACAAGATCTTCCAGGATGAAATCGGGGAGGTGTTCCGGGGGAC---CA
AC---CCCAGCAGAGAAGAGCGGAGGAGCTGGAGGACGGC----------
--------------------------------------------------
--------------------------G-----------------------
--------------------------------------------------
--------------------------------------------------
--------------------------------------------------
--------------------------------------------------
--------------------------------------------------
--------------------------------------------------
--------------------------------------------------
--------------------------------------------------
--------------------------------------------------
--------------------------------------------------
--------------------------------------------------
--------------------------------------------------
--------------------------------------------------
--------------------------------------------------
--------------------------------------------------
--------------------------------------------------
--------------------------------------------------
--------------------------------------------------
--------------------------------------------------
```

--CC
TCTTGCCACGTTCCTCAAACTGACCTCTCTGGGCTTCATAATTGGAGTCG
GCGTGGTAGGAAACCTGCTGATCTCTATCCTGCTGGTCAAAGACAAAAGC
CTGCACCGCGCACCCTACTACTTCCTGCTGGACCTGTGCGCCTCCGACAT
CCTGCGCTCCGCCATCTGCTACCCCTTCGTCTTCACCTCTGTCAAGAATG
GATCCGCCTGGACCTACGGCACGCTCACCTGCAAAGTGATTGCCTTCCTT
GGCGTGCTCTCCTGTTTCCACACGGCATTTATGCTGTTCTGCGTCAGCGT
CACCCGCTACCTGGCCATCGCCCACCACCGTTTCTACACCAAGAGGCTGA
CTTTCTGGACCTGCCTGGCCGTCATCTGCATGGTGTGGACGTTATCCGTG
GCCATGGCCTTCCCCCCAGTTCTCGATGTAGGGACTTATTCCTTTATCCG
GGAGGAGGACCAGTGTACATTCCAGCACCGCTCCTTCAGGGCTAACGACT
CCCTGGGCTTTATGCTCTTGCTGGCACTTATCCTGCTGGCTACGCAGCTG
GTTTACCTCAAGCTCATCTTCTTCGTCCACGACCGCAGGAAGATGAAGCC
TGTCCAGTTTGTGCCAGCTGTCAGCCAGAACTGGACCTTCCATGGGCCTG
GTGCCAGCGGGCAGGCAGCGGCCAACTGGCTGGCCGGATTCGGGAGAGGC
CCCACCCCGCCTACCTTGCTGGGCATCCGTCAGAACAGCAACGCGGCGGG
CCGCAGGCGTCTGCTGGTGCTGGATGAGTTTAAAACAGAGAAGAGGATAA
GTAGGATGTTCTACATCATGACGTTTTTTTTTCTGGTGCTGTGGGGGCCC
TATCTAGTGGCCTGCTACTGGCGGGTGTTCGCCAGGGGCCCCGTGGTGCC
CGGGGGCTACCTGACTGCAGCTGTGTGGATGAGCTTCGCCCAGGCGGGGG
TCAACCCCTTCATCTGCATCTTCTCCAACAGGGAG---------------

---------------------------CAGAGAGAGAGGTAGCTTTGGGG
ATAAATCCATTCGCAGACGGGATGGGCGCCTTTAAGATCAACCACACTTC
TCACGACATCGGCTCCGG---GCAGACGGCGTTTTCTTCCCAGGCG---C
CCGGGTACGCTGCCGCGGCCGCCCTGGGGCACCACCACCA------CCAC
CCGACCCATGTCAGCTCG---TACTCCACGGCGGCTTTCAACTCCACTCG
GGACTTTCTGTTCAGAAACCGGGGCTTCGGAGACGCCACCGG--------
----CGCGCAGCACAGCTTGTTCGCCTC---------GGGAGGTTT---T
------GCGGGGCCACATGGACACTCGGACGCCACGGGACACCTGCTCTT
CCCGGGGCTCCACGAG---CAAGCCGCGGGCCACGCGTCGTCCAATGTGG
TGAACAGCCAGATGCGCTTGGGTTTTTCAGGGGACGTGTACGGGAGGGCC
GAACAGTACGGCCACGTTACGAGCCCGAGGTCAGCAGAGCACTATGCCTC
GACCCAGTTGCACGGCTACGGCCCCATGAACATGAACATGGCCGCG---C
ACCACGGAGCGGGGGCCTTCTTCCGATACATGCGCCAGCCGATCAAGCAA
GAGCTCATCTGCAAGTGGATCGAACCGGAGCAGCTGACCAACCCCAAAAA
GTCGTGCAACAAAACTTTCAGCACCATGCACGAGCTGGTCACCCACCTGA
CGGTGGAGCATGTGGGGGGGCCGGAGCAGTCGAACCACATTTGCTTCTGG
GAGGACTGCTCCCGGGAAGGGAAACCGTTCAAAGCCAAATACAAACTTGT
GAAYCACATCAAG-------------------------------------

>Ipnops sp
AGCCTTCTCATTCGAGCTGAACTAAGCCAACCGGGGGCCCTTTTAGGTGA
CGACCAAATCTATAATGTCATTGTCACCGCACACGCTTTCGTAATAATCT
TCTTCATAGTCATGCCTATTATGATCGGCGGGTTCGGAAACTGGCTGGTC
CCTCTAATGATCGGAGCCCCTGATATAGCATTCCCCCGAATGAATAATAT
GAGCTTCTGACTTCTTCCCCCTTCTTTCCTCCTCCTACTAGCCTCCTCCG
GAGTAGAGGCTGGGGCCGGAACCGGCTGAACAGTCTACCCACCCCTGGCT
AGCAACCTCGCACATGCCGGAGCCTCAGTAGACCTAACCATTTTCTCATT
ACACTTAGCTGGTGTCTCCTCAATTCTAGGTGCTATTAACTTTATCACAA
CAATTATTAATATGAAACCCCCAGCCGTTTCTCAATACCAAACCCCTCTA
TTTGTATGGTCTGTCCTGGTTACAGCAGTTCTCCTCCTCCTTTCCCTACC
TGTCCTTGCAGCGGGAATTACCATGCTCCTCACAGATCGAAACTTGAACA
CTACCTTCTTTGACCCTGCAGGGGGAGGAGATCCCATCCTTTATCAACAC
TTATTCTGGTTCTTTGGGCACCCAGAAGTATACATTCTCATCCTTCCCGG
TTTTGGTATAATCTCACACATTGTTGCCTACTACTCCGGCAAAAAAGAAC
CCTTCGGCTACATGGGTATGGTCTGGGCCATGATGGCCATCGGCCTACTT
GGATTTATTGTGTGGGCTCACCACATGTTCACAGTAGGAATGGACGTAGA
CACTCGTGCCTATTCCTTGAGAGGAACCTGCACCCATCCAACTGCCTTGG
CATGCTGCTACTGTCAGATGCTCACCAGTGCACCAAGCTGTCAGAGCTGT
CCTGGGGTATGTGTCTCAGCAACTTCCCTGCCATCTGCAAGACGGAGGAC
TTCCTCCAGCTACCCAAAGACATGGTGGTGCAGCTTCTGTCCCATGAGGA
GCTGGAGACAGAAGATGAGAGACTGGTTTATGAGGCAGCCCTTAACTGGG
TCAACTATGACCTGGAGAGGAGACACTGCCACCTGTCAGAGCTGCTGAGA
ACCGTGCGCCTGGCCTTGTTACCTGCCATCTTCCTCATGGAGAACGTCTC
CACAGAGGAGCTGATCAATGCCCAAGTAAAGAGCAAGGAGTTAGTGGATG
AGGCTATCCGCTGCAAGCTGAAGATCCTGCAGAATGACGGTGTGGTCAAC
AGTCCCTGTGCCCGGCCCAGAAAGACCAGCCATGCCCTTTTCCTGCTGGG
AGGGCAGACCTTCATGTGTGACAAGCTGTACTTGGTGGACCAGAAGGCCA
AAGAGATCATCCCCAAGGCAGATATCCCCAGCCCCAGGAAGGAGTTCAGC
GCCTGTGCCATCGGCTGTAAGGTCTATGTCACAGGCGGGA--GAGGCTC-
AGAGAACGGCGTGTCTAAAGACGTGTGGGTCTATGACACGGTCCATGAGG
AGTGGTCTAAGGCAGCACCCATGCTCATTGCCAGGTTTGGCCATGGCTCT
GCTGAGCTCAAACACTGCCTCTATGTGGTTGGAGGACACACAGCAGCCAC
TGGCTGCCTCCCAGCTTCTCCGTCTGGATGAGTACATTGTAGTGTTCAGT
CGTTCTACAACAAGGCTGATTCTAAACGAAGCAGAGCTAATCCTGGCACT
GGCCCAGGAGTTTCAGATGAGGGTGGTTACAGTGTCTCTGGAGGAACAGT
CTTTCCCCAGCATCATCCAGGTGATCAGCGCGGCCTCCATGTTGGTTAGT
ATGCACGGAGCTCAGCTCGTCACCTCACTCTTCCTCCCTAGAGGAGCGGC
TGTAGTGGAGCTCTTCCCCTATGCTGTGAACCCAGAACAGTACACCCCAT
ATAAAACCCTAGCCTCCCTACCAGGCATGGACCTTCAATACGTTTCCTGG
AGGAACACTATTGAGGAGAACACTGTTRTCCACCCAGACAGACCTTGGGA
CCAAGGAGGCATTGCCCATTTGGAGAAAGACGAGCAGGAGAGAATCCTAG
CCAGCAAGGATGTCCCCAGGCACCTATGCTGCCGCAACCCAGAGTGGCTC
TTCAGGATCTACCAGGACACTCTGGTGGACATCCCTTCCTTCCTGGAAGT
CCTCAA---GGAGGGCCTGAAG---ACGAGGCCGAGCTTGAAGAA---GT
CCAAGCCGGCCAGCACGGTTCATCCGGGCCGGGTCCGAGAACCCCAGTGC
CAGACATCAGTCCAAGCCACCAACGAGGCTAAGCTCACGGTATCCTGGCA
GATCCCTTGGAACCTTAAGTACCTAACGGTGCGAGAGGTGAAGTATGATG
TATGGATCCGGAAAAAGGATTCAAGCAAGGGAACCCTGGAGGATCAAATC
ATTCAGGCAAACCCTGCACTGGAGGCTTTTGGTAATGCCAAAACAGCGAG
GAACGATAATTCCTCCCGTTTTGGAAAATTCATCCGAATTCACTTTGGAA
CCAGTGGTAAACTTTCCTCTGCGGACGTTGAGACCTACCTGCTGGAGAAG

TCACGGATCACCTTTCAGCTTAAGTCAGAGAGGAACTACCACATCTTCTT
CCAGATCTTGTCCAATCAAAAGCCAGAGCTGTTGGACATGCTGTTGATCA
CAAACAACCCTTATGACTACTCCTACATCTCCCAAGGAGAAGTAACCGTA
CCATCCATCAATGATTCAGAGGAGTTGATGGCCACTGACAGTGCCTTTGA
TGTGCTTGGCTTCACTCAAGAGGAGAAGATGGGAGTCTACAAGTTGACAG
GGGCCATTATGCACTATGGCAACATGAAGTTCAAGCAAAAGCAGCGTGAG
GAACAGGCTGAGCCTGATGGTACAGAGGCTGCTGATAAGTCAGCTTATCT
AATGGGGCTGAATTCAGCAGACCTCATCAAAGGTCTCTGCCATCCCAGAG
TCAAGGTAGGAAATGAGTATGTCACCAAAGGCCAGGGTGTAGATCAAGTC
TACTACCCCAACAAGGAGGCTTTCAAGTGTGAAGAGTGTGGCAAGCACTA
CAACACCAAGCTGGGATACAAGCGCCATGTGGCCATGCACTCTGCCACGG
CAGGGGACCTCACCTGCAAGGTCTGCATGCAGAGTTACGAGAGCACACCG
GTGCTCCTGGAACACCTCAAGAGCCACTCTGGGAAGTCCTCGGGTGGCGC
TAAGGAGAAGAAGCACCCGTGCGACCACTGCGACCGTCGCTTCTACACAC
GGAAGGATGTCAGGCGGCACATGGTTGTCCACACAGGCCGAAAGGACTTC
CTGTGCCAGTACTGTGCCCAGCGCTTCGGCAGGAAGGATCATCTGACACG
ACACGTGAAAAAGAGCCACTCGCAGGAGCTGCTGAAGATTAAGACGGAGC
CTCCAGATATGTTGGGTCTCCTGGGCTCTGGATCGCCGCCTTGCTCTGTC
AAGGAGGAGCTTAGCCCCATGATGTGCAACATGGGTCCCAATAAAGACCC
CATGATGGGCAAACCATTCCCCAGCGGGACCCCCTTCCCCATGGGCATGT
ACAACCCCCACCAC------CTCCGGGCCATTTCTAATCCAGGCGTGGGC
CACCCC------CACCCCTCCCTGATGCCCGGTTCCCTGTCTGCAGCTAT
GGGCATGAGCTGTCACATGGAATATCTCATCTACGCTTCCTTCTCCTTCA
TGGGATGTTTACAAATCAGCGACGGGTCGAACATCGTGAATTTGCTGGCC
ACTAACTCTCCAAGCGTCTCATACGCTATGACACAGCAGAAGTACTTCAG
TAACTACAGCCCAGTGATCGGGTTTTACATCTATGAGCCCATCGAGTACT
GGAACTCCACGGTGCAGGAGCACCTGAAGACGCTGAGCCACGGCTTCAAC
AAGATATCCTGGATGGACAATTTCTTCCACTACCTGCGAGTGGTGAACGT
GAGTGCGTCGACCAAGGCCGACTTCATCACCGTCCTCAAGGGCTCCTTCC
TGCGGAGCCCGGCTTACCAGCACTTCACAGAGGACATCATCTTCTCCAAG
A---GCCGGGAGAACA------ATGAGTACGAGATCATTGCCTCGCGCAT
GTACCTGGTGGCGCGCACCACGGAGAATAGGCGCGACGAGGTGGTGGAGC
TGCTGGAGAAGCTCCGGCCGCTGATGCTGATCAACAGCATCAAGTTCATC
GCCTTCAACCCCACGTTCGTCTTCATGGACCGCTACAGCTCCTCGGTCAT
TTCGCCCATCCTCACCTCGGGCTTCAGCGTGCTCACCATCCTCATCCTCA
CCTTCTTCCTGGTCATCAACCCGCTGGGGAATTTGTGGCTCATACTGACG
GTCACGTCCGTGGAGCTGGGCGTGCTGGGTCTGATGGGCTACCACACATT
CGAGTGGCAGCCAGCCCTCAAGAATGTGTCCCCATCCTGCCATGTGGGTA
TCATCAATGGGCTCTCTGGGTGGGCTTCCTCTGTGGATGATGCCCCTGCT
GAGACCATCACTCGTCGGTTTCGTTATGACGTGGCCCTGGTGTCAGCCCT
GAAGGACCTAGAGGAGGACATAATGGAAGGACTGAGGGAGCGTGGGCTGG
AAGACAGTGCTTGCACCTCAGGCTTCAGTGTTATGATCAAGGAATCCTGT
GATGGCATGGGAGATGTCAGCGAGAAGCATGGTGGAGGACCAGCGATGCC
TGAGAAGGCTGTGCGTTTCTCTATCACCATCATGTCCGTCTCTGTCCAGG
CCGATGGAGAGGAG------------------------------GAGGCG
GTTGTCATCTTCAGGGAGCCAAAGCCCAACTCTGAACTGTCCTGTAAGCC
CCTGTGCCTGATGTTTGTGGATGAGTCTGACCATGAGACAGTCACCGCTG
TCCTGGGGCCTTTGGTAGCAGAGAGGAATGCGATGAAGCAGAGCCGACTC
ATCCTTTCAGTGGGTGGCCTCCCCCGCTCTTTCCGCTTCCACTTCAGAGG
CACAGGCTATGATGAGAAGATGGTGCGAGAGATGGAAGGCCTGGAGGCCT
CAGGCTCCAGCTATATCTGCACTCTTTGTGACTCCACTCGGGCAGAAGCC
TCTCACAACATGGTGCTCCACTCTGTCACCCGCAGCCATGATGAGAACCT
GGAGCGCTATGAGATATGGAGGACCAACCCCTTCTCAGAGTCTGTCATGG

```
AACTACGAGACCGGGTCAAAGGGGTCTCTGCCAAACCATTTATGGAGACC
CATCCGACTCTTGATGCATTACACTGTGACATAGGTAATGCCACTGAATT
CTACAAAATCTTCCAGGATGAGATTGGGGAGGTGTTCCGGAAGGC---TA
AC---CTGAGCCGGGAGGAACGGCGCAGCTGGAGGGCAGCCCTCGATAAG
CAGCTGAGGAAGAAGTTGAAGTTGAGACCAGTGATGCGGATGAATGGGAA
CTATGCCCGAAGGCTGATGACCCAGGAGGCTGTGGAGGTGGTGTGTGAAC
TGGTGCCCACAGAAGAGAGGCGGGAGGCCCTAAGGGAGCTGATGGGGCTC
TATGTCCAGATGAAGCCTGTGTGGCGTGCTACCTGCCCAGCCAAGGAGTG
CCCTGATCAGCTGTGTGGCTACAGCTTTAACTCTCAGCGCTTTGCTGATC
TCCTCTCCTCCACCTTTAAATACAGGTATGATGGAAAGATCACCAACTAC
CTGCACAAGACCCTGGCCCATGTCCCTGAAATTATAGAGAGAGATGGCTC
CATTGGAGCCTGGGCCAGCGAGGGGAATGAGTCTGCAAACAAG-------
--------------------------------------------------
--------------------------------------------------
--------------------------------------------------
--------------------------------------------------
--------------------------------------------------
--------------------------------------------------
--------------------------------------------------
--------------------------------------------------
--------------------------------------------------
--------------------------------------------------
--------------------------------------------------
--------------------------------------------------
--------------------------------------------------
--------------------------------------------------
--------------------------------------------------
--------------------------------------------------
--------------------------------------------------
--------------------------------------------------
--------------------------------------------------
--------------------------------------------------
--------------------------------------------------
--------------------------------------------------
--------------------------------------------------
--------------------------------------------------
--------------------------------------------------
--------------------------------------------------
--------------------------------------------------
--------------------------------------------------
--------------------------------------------------
--------------------------------------------------
--------------------------------------------------
--------------------------------------------------
--------------------------------------------------
--------------------------------------------------
--------------------------------------------------
--------------------------------------------------
--------------------------------------------------
--------------------------------------------------
--------------------------------------------------
--------------------------------------------------
--------------------------------------------------
```


--------------ACAGGCGAAGTCACAGAAAGAGAAGTGGCTTTGGGG
ATAAATCCGTTCGCAGACGGGATGGGCGCCTTCAAAATAAACCACAGCTC
TCATGACATTGGCTCCGG---GCAAACGGCGTTTTCCTCCCAGGCG---C
CCGGCTAC---GCAGCGGCTGCCCTGGGA---CACCATCA------CCAC
CCGACCCACGTTAGCTCT---TATTCAACGGCAGCTTTCAATTCCACCCG
GGACTTTCTCTTCAGAAATCGGGGCTTCGGAGACGCCACCAG--------
----CGCGCAGCACAGTTTGTTCGCTTCCGC---CGCGGGAAGTTT---T
------GCAGGGCCACATGGACACTCAGATGCCGCTGGCCACCTGCTCTT
CCCTGGGCTTCATGAG---CAAGCCGCGAGCCATGCGTCATCTAACGTTG
TAAATAGCCAGATGCGACTGGGCTTTTCTGGGGACATGTACGGGCGGGCC
GACCAGTATGGCCACGTTACAAGCCCAAGGT---CCGACCACTATGCGTC
GACCCAACTGCATGGCTACGGCCCTATGAATATGAATATGGCCGCT---C
ACCACGGAGCAGGGGCCTTCTTTCGCTACATGAGGCAGCCGATTAAACAA
GAGCTAATATGCAAGTGGATCGAACCGGAGCAACTGACGAATCCCAAAAA
GTCGTGCAACAAAACTTTTAGCACGATGCATGAGCTCGTGACCCATCTGA
CGGTGGAGCATGTGGGGGGACCAGAGCAGTCGAACCACATTTGCTTCTGG
GAAGACTGTGCCCGAGAAGGGAAACCATTCAAAGCCAAATACAAACTTGT
AAATCATATCAGAGTTCACACCGGAGAAAAGCCGTTCCCGTGTCCATTCC
CCGGCT--------
>Kali normani
AGCCTTCTCATCCGAGCTGAACTCAGCCAACCAGGCGCCCTACTTGGGGA
TGACCAAATCTATAATGTTATCGTCACAGCCCACGCCTTCGTAATAATTT
TCTTTATAGTAATACCAATCATAATTGGAGGATTCGGGAACTGGTTAATC
CCACTTATGATCGGGGCCCCCGATATAGCCTTCCCTCGAATGAACAACAT
AAGCTTCTGACTTTTACCCCCTTCGTTCCTTCTCCTTCTCGCTTCTTCCG
GAGTTGAAGCCGGTGCCGGAACCGGGTGAACAGTCTACCCCCCTTTAGCC
GGTAATTTAGCCCATGCCGGAGCATCCGTTGATTTAACTATTTTTTCTCT
TCATCTGGCAGGTATCTCATCAATCCTGGGGGCCATCAATTTTATCACGA
CAATTATTAACATAAAACCTGCAGCTATCTCGCAGTACCAAACTCCCTTA
TTTGTTTGGGCCGTCTTAATTACAGCTGTTCTTCTTCTACTCTCTCTCCC
AGTTCTTGCCGCAGGCATTACAATACTTCTAACAGACCGAAACCTAAATA
CAACCTTTTTTGATCCGGCAGGAGGGGGAGACCCAATTCTTTATCAACAC
TTA---

------------NNNNNNNNNNNGAACCTTCACCCGACTAACTGCCTTGGCATGCTGTTGC
TGTCTGACGCCCACCAGTGCACCAAGCTGTCAGAGCTCTCATGGGGCATG
TGCCTCAGCAACTTCCCCGCTATTTGCAAGACAGAGGACTTCCTCCAACT
GCCCAAAGATATGGTAGTGCAGCTTTTGTCACATGAGGAGCTGGAGACAG
AAGATGAGAGACTGGTTTACGAAGCTGCCCTGAACTGGATCAACTATGAC
CTGGAAAGGAGGCACTGTCACCTTCCGGAGCTCCTGAGAACGGTCCGTCT
TGCCCTGCTGCCTGCCATCTTCCTAATGGAGAATGTCTCGACAGAAGAGC
TGATCAATGCCCAGGCCAAGAGCAAGGAGCTTGTGGATGAAGCTATCCGC
TGTAAGCTGAAAATCCTGCAGAACGACGGCGTCGTTAACAGCCCGTGTGC
TCGACCAAGAAAAACCAGCCATGCTCTCTTTCTCCTGGGAGGGCAGACTT

TCATGTGTGACAAGTTGTACCTGGTGGACCAGAAGGCCAAAGAGATCATC
CCCAAGGCGGACATTCCCAGCCCTCGGAAGGAGTTCAGCGCCTGTGCCAT
CGGCTGTAAGGTGTACATCACAGGTGGGA--GAGGCTC-AGAGAATGGCG
TGTCCAAAGATGTATGGGTCTATGACACCATCCATGAGGAGTGGTCCAAA
GCGGCGCCCATGCTCATCGCCAGGTTTGGTCATGGCTCTGCTAGCCTGAA
ACACTGCCNNNNNNNNNNNNNNNNNNNNNNNNNNNNNNNNNNNNNNNNNNNNNNNNNNNNNNNNGGATGA
ATACATTGTTGTCTTCAGTCGTTCAACAACAAGGCT
GATACTGAACGAAGCCGAGCTAATCATGGCGCTGGCCCAGGAGTTCCAGA
TGAGAGTGGTCACAGTTTCCCTAGAGGAACAGTCTTTCACCAGCATTGTC
GAGGTGATCAGCGGTGCTTCCATGTTAGTCAGCATGCATGGAGCTCAGCT
TATCACCTCACTCTTCCTCCCTAGAGGAGCTATTGTGGTGGAGCTGTTCC
CCTTTGCTGTGAACTCAGAGCAGTACACCCCATATAAAACCCTTGCCTCC
CTTCCAGGCATGGACCTTCATTATGTTTCCTGGAGGAACAGTCAGGAGGA
GAACACTGTCACCCATCCAGACAGACCCTGGGAACAAGGAGGCATCGCTC
ACTTGGAGAAGGAGGAGCAGGAGCGAATACTGACCAGCAAAGACGTCCCC
AGGCACCTGTGCTGCCGCAACCCGGAGTGGCTCTTCCGAATCTACCAGGA
CACTTTGGTTGACATTCCTTCCTTCTTGGAAGTCCTCAA---GGAGGGAA
TGAAG---AACAAGCCCAACTTGAAGAA---GTCCAAAACAGCCAGTACA
GTCCACCCAGGCCGGGTCAGAGAAGCCCAGTGTCAGACATCAGTACAAAC
CACCAATGAGGCTAAACTTACAGTCTCCTGGCAGATCCCGTGGAATCTGA
AATACCTGAAAGTAAGAGAGGTGAAGTACGAGGTA---------AAAAGA
GACGCCAGCAAAGGAACTCTGGAGGATCAAATCATCCAGGCGAACCCGGC
GCTGGAGGCCTTCGGAAATGCCAAAACGCTGAGAAACGACAACTCGTCCC
GTTTTGGAAAATTCATCCGAATTCACTTCGGAAACAGCGGCAAGCTGTCG
TCTGCTGACGTGGAGACGTACCTGTTGGAGAAGTCTCGGGTCACCTATCA
GCTCAAGGCTGAGAGGAACTACCACATCTTCTACCAGATCCTGTCCAATC
AGAAGCCAGAGCTGCTGGACATGCTGTTGATCACCAACAACCCGTACGAC
TACTCCTACATCTCCCAAGGAGAGGTAACAGTCGCCTCCATCAACGACTC
GGAGGAGCTGATGGCCACCGACAGCGCCTTCGATGTGCTCGGCTTCACTC
CGGAGGAGAAGATGGGCGTCTATAAACTGACCGGCGCCATCATGCACTAC
GGCAACATGAAGTTCAAGCAGAAGCAGCGTGAGGAGCAGGCGGAGCCTGA
CGGGACGGAGGCTGTTGATAAATCAGCTTACCTGATGGGGCTGAACTCTG
CTGACCTCATCAAAGGGCTGTGCCATCCCAGAGTCAAGGTAGGAAACGAG
TACGTCACCAAAGGCCAGAGTGTGGACCAAGTCTACTACCCCAACAAGGA
GGCCTTCAAGTGTGAGGAGTGTGGGAAGCACTACAACACCAAGCTGGGAT
ATAAGCGCCATGTGGCCATGCACTCTGCCACGGCGGGGGATCTCACCTGT
AAAGTGTGCATGCAGAGCTACGAGAGCACCCCTGTTCTCCTGGAGCACCT
CAAGAGCCACTCGGGGAAGTCCTCCGGCGGAGCCAAGGAGAAAAAGCACC
CGTGCGACCACTGCGACCGCCGCTTCTACACGCGGAAGGATGTGAGACGG
CACATGGTGGTCCACACAGGCCGAAAGGACTTCCTGTGCCAGTACTGTGC
CCAGCGCTTTGGCAGGAAGGATCACCTGACCCGACACGTGAAGAAGAGCC
ACTCGCAGGAGCTGCTGAAGATCAAGACGGAGCCTCCTGACATGTTAAGT
CTTTTAGCTTCAGGGTCACCACCGTGCTCTGTGAAGGAGGAGCTTAGCCC
CATGATGTGCGGCATGGGTCCCAACAAAGACCCCATGATGGGCAAACCAT
TCCCTAGTGGCGCCCCTTTCCCGATGGGCATGTACAACCCCCACCAC---
---CTTCAGGCCATGTCTAATTCTGGGGTGGGTCATGCA------CACCC
GTCCCTGATGCCCAGCCCCCTGTCTGCAGCTATGGGCATGGGCTGTCACA
TGGAATATCTAATCTATGCCTCTTTCTCATTCATGGGATGTTTACAAATT
AGTGATGGATCGAACATTGTGAACCTGCTGGCGAGTAACTCTCCGAGTGT
TTCGTACGCTCTGACCCAGCAGAAATACTTCAGCAACTACAGTCCCGTGA
TCGGGTTTTACATTTACGAGCCCATCGAGTACTGGAACTCCACGGTGCAG
GAGCACCTGAGAACTCTGAGTCACGGCTTCGAGAAGATCTCCTGGATGGA
CAACTTCTTCCACTACCTGCGGGTGGTGAACGTGAGTGCGTCGACCAAGA

GCGACTTCATCACCATCCTCATGGGCTCCTTCCTGCGCAGCCCGGAGTAC
CAGCACTTCACTGAGGACATCATATTTTCCAAGA---ACCGTGAGACGG-
-----ACGAATACGACATTATCGCCTCGCGCATGTATTTGATGGCACCGA
CCACCGAGAACAAGCGCGAGGAGGTGGTGGAGCTTCTGGAGAAGCTCCGC
CCCTTGATGCTGATTAACAGCATCAAGTTCATCGCCTTCAATCCCACGTT
TGTGTTCATGGACCGCTACAGCTCATCCGTCATCTCACCCATCCTGACCT
CAGGCTTCAGCGTGCTCACCATCCTCATCCTCACTTTCTTCCTGGTCATC
AACCCCCTGGGGAACTTCTGGCTCATCCTCACGGTTACATCCGTGGAGCT
GGGCGTCTTGGNNNNNNNNN---------------------------------------

------------------TCATACACCATCGAGATGGGTCCCCTGGGGCC
CCAATGGAAGGAGAGTCCACAGCCGTTCTCCTGCTCCGTTGAAGACCCCA
CAAAACAGACAAAGTTCAAAGGCATCAAGACGTACATTTCGTATCGGGTC
ACGCCGAGCCACACGGGGCATCCCGTCTACAGGCGCTACAAACACTTTGA
CTGGCTGTACAACCGCCTGCTGCACAAGTTCACTGTGATCTCCGTGCCTC
ACCTGCCCGAGAAGCAGGCCACGGGGCGATTTGAGGAAGACTTTATCGAG
AAGCGGAAGAGACGACTGATACTGTGGATGAACCACATGACCAGTCACCC
GGTCCTCTCCCAGTACGAAGGCTTCGAGCACTTTCTGATGTGTGCTGACG
ACAAGCAGTGGAAGCTGGGCAAGAGGCGAGCTGAGAAGGACGAGATGGTG
GGCGCCCATTTCATGCTGACCCTCCAGATCCCCAACGAGCACCAGGACCT
TCAGGACGTAGAGGAGAGAGTGGACACCTTCAAGGCCTTCGCTAAGAAAA
TGGACGACAGCGTTATGCAGCTCACACATGTTGCCTCGGAGCTGGTGCGT
AAGCACCTGGGTGGATTCAGGAAGGAGTTCCAGCGGCTGGGAAATGCCTT
CCAGTCTATTAGCCAGGCGTTCATGCTGGACCCTCCCCATAGCTCAGACA
CCGTCAANNNNNNNNNNNNNNNN---

--GCCAAA
TCTCGCTTTCACCCTGGCGTGGGGACTTGTCCTGGCACGGAAC---GCAG
CGTCCCACTCGGCAACAGCTTGCTATCCCCGCAGCAAGCCGAGGAGCCCA
CGGTGGCCACACCCCCGCAGCGATGGTTTGTCACCC---CTGCAAACAAC
CGACTGGACTTTGCCGCCTCGGCGTACGACGCCGCT---------GATTT
CGCCGGTAACGCGGCCACCTTGCTGTCCTACGCAGCGGCCGGAGTCAAGG
CTC------TTCCCCTGCCGACCGCAGGCTGCTCCAACCGGCCTCTTGGC
TATTACGCAGACCCGTCCG---GCTGG---GGAGGACGCACGCCGCCGCA
GTACTGTGGCGTCAATAGCAAACCCAGTTCGGTCTTTTCCTGCTGGCCCG
CTAACTCTATCGGCGGCAGAGCAGGCA---CC---AACTACCTGG-----
----CCGACGA---GGGA---GATCC---CATCCCCACAGAGAGGTCACC
C---AT---C---GGCTCTGAGGAG---ACCAAACCCAAAGACATAAC--
-ATCTGA---GTCGAGCTGGATAGAG---ACACCCTCCTCCATTAAGTCC
ATCGATTCAAGCGATTCTGGTATCTTTG---AGCAGGCCAAGAGGAGAAG
AATCTCACCTTCTGCCACACCG---------GTTTCAGAGACAGTGTCCC
AGTNNNNNNNNNNN---

>Kurtus gulliveri
NNNNNNNNNNNNNNNNNNNNNNNNNNNNNNNNAGGCGCACTACTGGGAGACGACCAAATCTATAATGTGA
TCGTCACAGCCC

ATGCTTTCGTAATAATCTTCTTTATAGTAATACCAATTATAATTGGAGGT
TTCGGGAACTGACTAGTCCCACTAATAATTGGAGCCCCAGACATAGCATT
TCCCCGAATAAATAACATAAGTTTCTGACTACTTCCCCCCTCTTTCCTAC
TCCTTCTTGCCTCTTCGGGTGTAGAAGCCGGTGCTGGCACTGGATGAACA
GTTTACCCCCCATTAGCCGGAAACCTAGCCCATGCAGGAGCATCCGTCGA
CCTCACAATCTTCTCCCTTCACTTGGCCGGCGTTTCCTCAATCCTAGGAG
CTATTAATTTTATTACTACAATTATTAATATAAAACCCCCTGCAGTATCT
CAATACCAAACTCCGCTATTTGTATGATCCGTTCTTATTACAGCCGTCCT
CCTTCTCTTATCCCTCCCAGTTCTTGCCGCCGGAATTACTATGCTACTCA
CAGATCGAAATTTAAATACTTCTTTCTTTGACCCAGCAGGAGGCGGCGAC
CCCATTCTATATCAACACCTCTTCTGATTCTTCGGCCACCCAGAAGTTTA
TATTCTAATCCTCCCCGGATTCGGTATAATCTCCCACATCGTAGCATACT
ATGCTGGGAAAAAAGAACCATTTGGGTACATAGGCATGGTTTGAGCCATG
ATAGCCATCGGCCTACTAGGCTTCATTGTATGAGCCCACCACATATTTAC
AGTTGGAATAGATGTTGATACACGAGCATANNNNNNNNNNNNNNNNNNNNNNNNNNNNNNNNNN
NGCTATTGCTGTCTGACGCCC
ACCAGTGTACCAAGCTGTCAGAGCTCTCCTGGGGCATGTGCCTCAGCAAC
TTCCCTGCTATTTGCAAGACAGAGGACTTCCTCCAACTGCCCAAAGATAT
GGTAGTGCAGCTTTTGTCACATGAGGAGCTAGAGACGGAAGATGAGAGAC
TGGTTTATGAAGCTGCTCTCAACTGGATCAACTATGACTTAGAAAGGAGG
CACTGCCACCTTCCAGAGCTCCTGAGAACAGTCCGCCTTGCCCTGCTGCC
TGCCATTTTTCTGATGGAGAATGTGTCAACAGAGGAGCTGATCAACGCCC
AGGCCAAGAGCAAGGAGTTGGTGGACGAGGCAATCCGCTGTAAACTGAAG
ATCCTGCAGAATGATGGTGTTGTAAACAGCCCATGTGCTCGACCAAGAAA
AACTAGCCATGCCCTCTTTCTTCTGGGAGGGCAGACTTTCATGTGTGACA
AGTTGTACCTGGTGGACCAGAAAGCCAAAGAGATCATTCCAAAGGCAGAC
ATCCCCAGCCCAAGAAAGGAGTTCAGTGCCTGCGCCATTGGCTGTAAGGT
GTATATAACAGGAGGAA--GAGGCTC-TGAGAATGGTGTGTCCAAAGATG
TGTGGGTCTATGACACAGTTCATGAGGAATGGTCCAAGGCAGCGCCTATG
CTCATCGCCAGATTTGGCCATGGCTCTGCAGAGCTCAAACACTGCCTCTA
TGTGGTTGGAGGTCACACCGCAGCGACTGGCTGCCTCCCGNNNNNNNNNNNNGGATGAATAC
ATTGTTGTCTTTAGTCGTTCAACAACAAGGCTGATACTCAATGAAGCAGA
GCTAATCATGGTACTGGCCCAGGAGTTCCAGAAGAGGGTGGTCACAGTAT
CTCTGGAGGAGCAGTCTTTCCCCAGTATCGTCCAGGTGATCAGCGGGGCA
TCTATGTTAGTCAGTATGCATGGAGCTCAGCTTATCACCTCACTCTTCCT
CCCCAGAGGAGCTGTTGTGGTTGAGCTGTACCCATTTGCTGTGAACCCAG
AACAGTACACCCCTTATAAAACCCTTGCATCCCTTCCAGGCATGGACCTT
CACTATATTTCATGGAGGAACACTAAGGAGGAGAACACTGTCACCCACCC
AGACAGGCCCTGGGAGCAAGGAGGCATCAGCCACTTGGAAAAAGAAGAGC
AAGAGCGTATACTGGCCAGCAAAGATGTCCCCAGGCACCTTTGCTGCCGC
AACCCGGAGTGGCTTTTCCGGATTTACCAGGACACTTTGGTGGACATACC
TTCATTCTTGGAAGTCCTCAA---AGAGGGCATGAAG---ACCAAGCCCA
TCTTAAAGAA---GTCCAAGCCGGCCAACGCGGTCCACCCAGGCCGGGTC
AGAGATCCTCAGTGTCAGACCTCAGTACAAACAACCAGTGAGGCTAAACT
CACGGTCTCCTGGCAGATCCCGTGGAATCTGAAATACCTGAAGGTAAGAG
AGGTCAAATACGAAGTGNNNNNNNNNAAAAAGGACACCAGTAAGGGGACACTGGAGGAT
CAAATCATCCAGGCAAATCCTGCACTGGAGGCCTTTGGCAACGCCAAAAC
AGTAAGAAATGACAACTCATCTCGTTTTGGAAAATTCATCCGAATTCATT
TCGGAAACAGTGGCAAGTTATCATCTGCCGACATTGAGACTTACCTACTG
GAGAAGTCACGCGTCACCTTTCAGCTTAAGGCTGAGAGGAACTACCACAT
CTTCTACCAGATTCTGTCCAATCAGAAGCCAGAGCTGCTGGACATGCTGC
TGATCACCAACAACCCGTATGACTATTCCTACATCTCCCAAGGAGAGGTA
ACAGTTGCCTCCATCAACGACTCAGAGGAACTGATGGCCACTGACAGTGC

CTTTGATGTGCTTGGCTTCACTCCAGAGGAGAAGATGGGTGTCTATAAAC
TGATCGGTGCCATCATGCACCATGGAAACATGAAGTTTAAACAAAAGCAG
CGTGAAGAGCAGGCTGAGCCCGACGGGACAGAGGCTGCTGATAAATCCGC
TTATCTTATGGGGCTGAACTCTGCTGACCTTATAAAAGGGCTGTGCCATC
CCAGGGTGAAGGTAGGAAATGAATATGTCACTAAAGGCCAGAGTGTGGAC
CAAGTCTACTANNNNNNNNNNNNNNNNNNNNNNNNNNNNNNNNNNNGGAAGCACTACAACACCAAGCTGG
GATATAAGCGCCATG
TGGCCATGCACTCTGCCACTGCAGGGGATCTCACTTGTAAAGTGTGCATG
CAGAGCTATGAGAGCACGCCTGTGCTCCTGGAGCACCTCAAGAGCCACTC
TGGGAAATCCTCAGGTGGTGCCAAGGAGAAAAAACACCCATGCGACCACT
GTGACCGCCGCTTCTACACAAGGAAGGATGTGAGGCGGCACATGGTGGTC
CACACAGGTAGAAAGGACTTTCTGTGTCAGTACTGTGCCCAACGCTTCGG
CAGGAAGGACCACCTGACACGTCATGTAAAGAAGAGCCACTCACAGGAGC
TGCTGAAGATCAAGACAGAACCCCCTGATATGTTAGGTTTGTTAGCTTCT
GGGTCACCACCTTGCTCTGTGAAGGAGGAGCTTAGTCCCATGATGTGTGG
CATGGGTCCCAATAAAGACCCTATAATGGGAAAACCTTTCCCCAGTGGAG
CCCCCTTTCCTATGAGCATGTACAACCCCCACCAT------CTCCAGGCC
ATGTCTAATTCTGGCGTGGGTCATCCA------CACCCTTCCCTAATGCC
CAGTTCTCTATCTGCAGCTATGGGCATGGGCTGCCANNNNNNNNNNNNNNNNNNNNNNNNNNNNNNNNNN
NNNNNNNNNNNNNNNNNNNNNNNNNNNNNNNNNNNNNTGAACCTGCTGGCT
AGTAACTCTCCAAGTGTTTCGTATGCTCTGACCCAACAGAAATACTTCAG
TAACTACAGTCCTGTGATTGGGTTTTACATTTATGAGCCTATTGAATACT
GGAACTCCACAGTGCAAGAACACCTAAAGACTCTGAGTCACGGATTCAAC
AAGATCTCCTGGATGGACAACTTTTTCCACTACTTGCGGGTGGTGAATGT
GAGTGCATCAACCAAAAGCGACTTCATTAGCATCCTCAAGGGCTCCTTCC
TGCGCAGCCCGGAGTACCAGCACTTCACCGAGGACATCATATTCTCCAAG
A---ATCGCGAGACTG------ACGAGTATGACATTATTGCCTCACGCAT
GTATTTGGTGGCACGCACCACGGAGAAGAAGCGTGAGGAGGTGGTGGAGC
TTTTAGAAAAGCTTCGCCCTTTGATGTTGATCAACAGCATCAAGTTCATT
GCCTTCAATCCCACGTTTGTGTTCATGGACCGCTACAGTTCCTCTGTCAT
CTCACCCATTCTAACCTCAGGCTTCAGTGTGCTCACCATTCTCATCCTCA
CTTTCTTCCTGGTCATCAACCCATTGGGAAACTTCTGNNNNNNNNNNNNNNNNNNNNNNNNNNNNNNNNN
NNNNNNNNNNNNNNNNGGCTTCCACCAGT
TTGAATGGCAGCCAGCTCTCCAGAATGTGTCTACATCTTGCAATGTTGGC
ATTATTAATGGGCTCTCTGGGTGGACATCTTCAGTGGATGACTCCCCAGC
TGACACAATCACGAGGCGCTTTCGCTACGATGTGGCGCTGGTGTCAGCAT
TAAAAGATCTGGAGGAGGACATTATGGACGGGCTGAGAGAGCGTGGGATG
GAAGATAGCACTTGCACCACAGACTTCAGAGTTATGATCAAGGAAGCCTG
TGACGGCATGGGTGATGTCAGTGAGAAGCATGGAGGAGGTCCTGCAGTTC
CTGAGAAGGCTGTTCGTTTCTCTTTCACTATTATGTCTGTGTCTGTCAAG
GCAGAGGAGGAGAAT------------------------------GCAGA
AGTTAGCATCTTCACGGAGCCAAAGCCAAATTCAGAACTGTCCTGTAAGC
CCCTTTGCTTGATGTTTGTGGATGAATCCGACCATGAGACACTCACAGCT
GTCTTGGGGCCGATAGTTGCAGAGCGTAATGCAATGAAACAGAGTAGGCT
CATCCTATCCATAGGTGGCTTACCCCGATCCTTCCGCTTTCACTTTAGAG
GCACAGGATATGATGAGAAGATGGTGCGTGAGCTGGAGGGCCTGGAGGCC
TCAGGATCCACATATGTTTGCACCCTGTGTGACTCCAGTCGAGCAGAGGC
CTCTCAAAATATGCTACTGCACTCCATCACCCGCAGTCATGAAGAGAACC
TAGAACGTTATGAAATTTGGAGAACGAACCCCTTTTCAGAATCTACAGAT
GAGCTGCGAGACAGAGTCAAAGGGGTCTCAGCTAAACCTTTTATGGAGAC
CCAACCCACACTGGATGCATTACACTGTGACATTGGCAATGCCACTGAGT
TCTACAAAATGTTCCAGGATGAGATTGGGGAAGTGTATCAAAAGGT---C
AAC---CCCAGCCGTGAGGAACGGCGCAGTTGGAGGGCAGCCTTAGATAA

ACAGCTGAGGAAGAAGATGAAGCTTAAACCTGTAATGAGGATGAATGGGA
ACTATGCCCGCAGGTTAATGACCCAGGAAGCTGTGGAGGTAGTGTGTGAG
CTAGTGCCCTCAGAGGAGAGGAGGGAGGCCCTGAGGGAGCTTATGAGGCT
CTACCTCCAGATGAAGCCTGTGTGGCGCGCCACCTGCCCAGCCAAGGAGT
GCCCCGACCAGCTGTGCCGCTACAGTTTTAACTCCCAGCGCTTTGCTGAC
CTCCTCTCCTCTACATTCAAATATAGGTACAATGGAAAGATAACCAATTA
CCTGCACAAGACCTTGGCTCATGTCCCTGAAATCATAGAGAGAGATGGAT
CCATAGGAGCCTGGGCCAGTGAGGGCAACGAGTCAGCAAACAAA------

NNNNNNNNNNNNNNNNNNNNNNNNNNNNNNNT
GGGTTTCATCATGGGAGTCGGTGTGGTTGGAAACCTCCTGATTTCCATCC
TGCTGGTCAAAGACAAGAGTCTGCACAGAGCGCCCTACTATTTCCTTCTG
GACCTGTGCGCCTCTGATATCCTGCGTTCTGCCATCTGCTTCCCCTTTGT
CTTCACCTCAGTTAAGAATGGATCTGCCTGGACCTATGGCACGCTGACCT
GCAAAGTGATTGCCTTCCTGGGGGTGCTCTCCTGTTTCCACACGGCATTC
ATGCTATTCTGTGTCAGTGTTACTCGCTACCTGGCAATAGCACATCATCG
TTTCTACACCAAGAGGCTAACCTTCTGGACCTGTCTGGCTGTCATCTGTA
TGGTTTGGACACTGTCAGTTGCTATGGCATTCCCGCCAGTACTAGACGTA
GGGACGTACTCTTTTATACGGGAGGAGGACCAGTGCACATTCCAGCACCG
TTCCTTCAGGGCAAATGATTCGCTGGGCTTCATGCTCCTGCTGGCACTTA
TCCTCCTGGCCACACAGCTGGTTTACCTCAAGCTCATTTTCTTCGTGCAC
GACCGTCGAAAGATGAAGCCAGTCCAGTTTGTGCCTGCAGTCAGCCAGAA
CTGGACCTTCCACGGGCCAGGCGCCAGCGGGCAGGCAGCAGCTAACTGGC
TGGCTGGATTTGGTCGAGGCCCCACCCCACCCACTTTGCTGGGCATCCGG
CAGAACAGCAATGCAGCGGGCCGCAGGCGTCTACTTGTATTGGATGAATT
CAAAACAGAGAAGAGGATTAGTAGGATGTTCTACATCATGACGTTTTTCT
TCCTGGCTCTGTGGGGGCCCTATCTGGTAGCCTGCTACTGGAGGGTGTTT
GCAAGGGGCCCTGTAGTCCCTGAGGGCTACCTGACAGCAGCCGTGTGGAT
GAGCTTTGNNNNNNNNNNNNNNNNNNNNNNNNNNNNNNNNNNNNNNNNNNNNNNNNNNNNNNNNNN
NNNNNNNNNNNNNNNNNNNNNNNNNNNNNNNNNC---
GCAGCGTCCCACTCGGCAACAGCTTGCTATCCCCGCAG
CAAACCGAGGAGCCCACTGTTGCCACCCCCCCGCAGCGATGGTTTGTCAC
CC---CTGCCAACAACCGACTGGACTTTCCTGCCTCGGCATACGACGCCG
CT---------GATTTCGCCGGTAACGCGGCCACCTTGCTGTCCTACGCA
GCGGCCGGAGTGAAAGCTC------TTCCCCTGCCGACTGCAGGCTGCTC
CAACCGGCCTCTTGGCTATTACGCAGACCCGTCAG---GCTGG---GGAG
GACGCACGCCGCCGCAGTATTGTGGCGTAAATAGCAAATCCAGCTCGGTC
TTTACTTGCTGGCCCGCTAACTCTATCGGCGGCAGAGCTGGCC---CC--
-AACTACCTGG---------CTGAGGA---GGGA---GACTC---AATCC
CGACAGAGAGGTCACCG---AT---TGGCGGCTCAGAAGAG---ACCAAA

CCCAAAGACAT------CTCAGA---ATCGAGCTGGATAGAA---ACGCC
GTCTTCTATTAAGTCCATTGATTCAAGTGATTCTGGTATCTATG---AAC
AGGCCAAACGGAGAAGAATCTCACCTTCTGCCACACCA---------GTT
TCAGAGACTGTGTCCCNNNNNNNNNNNNNNNNNNNNNNNNNNNNNNNNGTCACAGAGAGAGAAGTGGCGT
TGGGGATAAATC
CGTTCGCGGATGGGATGGGCGCCTTCAAAATAAACCACAGCTCCCACGAC
ATTGGCTCCGG---ACAGACGGCGTTTTCCTCCCAGGCG---CCCGGCTA
C---GCAGCAGCTGCCTTGGGA---CACCATCA------CCACCCGACCC
ACGTTGGCTCT---TACTCCACGGCGGCTTTCAACTCCACCAGGGACTTT
CTCTTCAGAAATCGGGGTTTCGGGGACGCCACCGG------------GGC
TCAGCACAGTTTGTTCGCCTC---------CGGAAGTTT---C------G
CAGGGCCACACGGACACTCAGATGCGGCGGGGCACCTGCTCTTCCCGGGG
CTCCACGAG---CAAGCGGCGAGCCATGCGTCTTCCAACGTGGTCAACGG
CCAGATGCGGTTGGGCTTCTCGGGGGACATGTACGGACGGGCCGACCAGT
ACGGCCACGTTACAAGCCCGCGGT---CCGACCACTACGCTTCGACCCAG
CTGCACGGCTATGGCCCCATGAACATGAATATGGCCGCA---CACCACGG
AGCAGGGGCCTTCTTTCGATACATGAGGCAGCCGATCAAGCAAGAGCTCA
TCTGCAAGTGGGTCGAGCCGGAGCAGTTGACAAATCCCAAAAAGTCGTGC
AACAAAACTTTTAGCACGATGCACGAGCTCGTGACCCATCTGACGGTGGA
GCATGTGGGGGGACCGGAACAGACGAACCACATCTGCTTCTGGGAGGATT
GCGCNNNNNNNNNNNNNNNNNNNNNNNNNNNNNNNNNNNNNNNNNNNNNNNNNNNNNNNNNNNNNNNNNN
NNNNNNNNNNNNNNNNNNNNNNNNNNNNNNNNNNNNN
>Labrisomus multiporosus

-------------------------GGACGAGTACATCGTGGTGTTCAGC
CGCTCCACCACGAGGCTCATCCTGAACGAGGCCGAGCTGATCATGGCACT
GGTGCAGGAGTTCCAGATGAAGGTGGTCACCGTGTCTCTGGAGGAGCAGT
CCTTCCCCAGCATCGTCCAGGCGGTCAGCGGCGCCTCCATGCTGGTCAGC
ATGCACGGGGCTCAGCTGATCACGTCGCTTTTCCTGCCCAGAGGCGCCGC
CGTGGTGGAGCTGTTCCCCTTTGCGGTGAACCCGGAGCAGTACACCCCGT
ATAAGACTCTGGCCTCGCTGCCCGGCATGGACGTCCACTACGTCTCCTGG
AGGAACACCATGGAGGAGAACACCGTCACCCACCCGGACCGGCCGTGGGA
GCAAGGCGGCATCGCTCACTTGGAAAAGGAGGAGCAGGAGCGGATCCTGG
CGAGCAAGGACGTCCCCAGACACCTGTGCTGCCGCAACCCGGAGTGGCTC
TACAGGATCTACCAGGATACGACGGTGGACGTCCCGTCTTTCCTGGAAGT
CCTCCG---AGAAGGCATGAAG---ACCAAGCCCAACCTGAAGAA---GG
CCAGAGCCGCCAGCATCGTCCACCCGGGCCGGGTCCGAGAGCCCCAGTGT
CAGACCTCGGTGCAGACCAGCAGCGAGGCCAAGCTGACGGTGTCCTGGCA
GATCCCGTGGAACCTCAAGTACCTGAAGGTCCGAGAGGTGAAGTACGAGG
TGTGGATCCAGAAGAAGGACACCAGCAAAGGGACCTTGGAGGATCAAATA
ATCCAGGCAAACCCGGCGCTTGAGGCCTTTGGCAACGCCAAGACGCTGAG
GAATGACAACTCCTCCAGGTTTGGAAAATTCATCCGGATTCACTTCGGCA
CCAGCGGCAAGCTGTCTTCAGCCGACATCGAGACGTACCTGTTGGAGAAG
TCCCGAGTCACCTTTCAGTTGAAGGCTGAGAGGAACTACCACATCTTCTA
CCAGATCCTGTCCAATCAAAAGCCAGAGCTGCTGGACATGCTCCTGATCA
CAAACAACCCTTATGACTACTCCTACATCTCACAGGGAGAAGTGACAGTC
GCCTCAATCAATGATGCGGAGGAGCTCATGGCGACTGACAGTGCCTTCGA
TGTGCTCGGCTTTACATCGGATGAGAAAATGGGTGTCTACAAGCTGACCG
GTGCCATCATGCACCACGGCAACATGAAGTTCAAACAGAAGCAACGCGAG
GAGCAGGCGGAGCCCGATGGGACCGAGGCTGCTGACAAGTCTGCCTACCT
GATGGGGCTGAACTCAGCTGACCTCATTAAGGGTCTGTGCCACCCCAGGG
TCAAAGTAGGAAACGAGTACGTGACAAAAGGACAAAGTGTGGACCAGGTG
TACTATCCAAACAAGGAGGCCTTCAAGTGCGAGGAGTGTGGGAAGCACTA
CAACACCAAGCTGGGATACAAGCGCCATGTGGCCATGCACTCCGCCACCG
CGGGGGATCTCACCTGCAAAGTGTGCATGCAGAGCTATGAGAGCACGCCT
GTCCTTTTGGAGCATCTCAAAAGCCACTCGGGAAAATCCTCCAGTGGCAC
CAAGGAGAAGAAACACCCATGTGACCACTGCGACCGCCGCTTCTACACGC
GGAAGGACGTGCGGCGGCACATGGTTGTCCACACTGGCAGAAAAGACTTC
CTGTGTCAGTACTGTGCCCAGCGCTTTGGCAGGAAGGACCATTTGACGCG
CCACGTGAAAAAGAGCCACTCACAGGAGCTTCTGAAGATCAAGACAGAGC
CCCCTGACATGCTCGGGCTTTTAGCCACGGGCTCGCCACCGTGCTCCGTC
AAGGAGGAGCTCAGCCCCATCATGTGTGGCATGGGGCCCAACAAAGACTC
GGTGATGGGCAAACCATTTCCCGGCGGGGCCCCGTTCCCCATGGGCATGT
ACAACCCCCACCAC------CTCCAGGCCATGTCCAATTCCGGGGTGGGT
CACCCT------CACCCTTCCCTGATGCCCGGCTCCTTGTCTGCAGCTAT
GGGCATGGGATGCCACATGGAANNNNNNNNNNNNNNNNNNNNNNNNNNNNNNNNNTGTTTACAGATCAGC
GACGGGTCGAATA
TCGTGAACCTGCTGGCGAGCAACTCCCCGAGCGTTTCGTACGCGCTCACG
CAGCAGAAGTACTTCAGTAACTACAGCCCGGTGATCGGGTTCTACATTTA
CGAGCCCATCGAGTACTGGAACTCCACGGTGCAGGAGCACCTGAAGACCC
TGAGTCACGGCTTCAACAAGATCTCCTGGATGGACAACTTTTTCCACTAC
CTGCGGGTGGTGAACGTGAGCGCGTCGACGAAGAGCGACTTCATCAGCAT
CCTCAAGGGCTCCTTCCTGCGGAGCCCGGAGTACCAGCACTTCACCGAGG
ACATCATATTCTCCAAGA---ACCGCGAGACCG------ACGAGTACGAC
ATCATCGCGTCGCGGATGTACCTGGTCGCGCGGACCACGGAGAAGAAGCG

CGAGGAGGTGGTGGAGCTCCTGGAGAAGCTCCGCCCCCTGATGCTGATCA
ACAGCATCAAGTTCATTGCCTTCAACCCCACCTTCGTGTTCATGGATCGC
TACAGCTCCTCCGTCATCTCGCCCATCCTGACCTCAGGCTTCAGCGTGCT
CACCATCCTCATCCTCACTTTCTTCCTGGTCATCAACCCCTTGGGAAACT
TCTGGNNNNNNNNNNNNNNNNNNNNNNNNNNNNNNNNNNNNNNNNNNNNNNGGTTTTCACCAGTTTGA
ATGGCAGCCAGCTCTCAAGAATGTGTCG
ACATCTTGCAACGTTGGCATTATTAATGGGCTCGCCGGATCAGCTGCCTC
GGTGGATGACGCCCCGGCTGACACCATCACTCGGCGCTTTCGCTATGATG
TGGCACTGGTGTCAGCACTAAAGGATCTGGAGGAGGACATCATGGAGGGG
CTGAGAGAGAATGGGATGGAAGACAGCGCTTGCACCTCAGGCTTTAGTGT
TATGATCAAGGAGTGCTGTGATGGCATGGGTGATGTCAGCGAGAAGCACG
GTGGAGGACCAGCTCTTCCCGAGAAGGCTGTACGTTTCTCTTTCACTGTT
ATGTCTGTCTCTGTCCTGGCAGGCGATAGGGAG-----------------
-------------GAGGAGGTTACCATTTTCACTGAGCCAAAGCCAAACT
CAGAACTGTCCTGTAAGCCCCTCTGCCTGATGTTTGTGGATGAGTCAGAC
CATGAGACACTCACAGCTGTCCTGGAGCCTATAGTTGCAGAGCGAAAGGC
CATGAAAGAGAGCAGGCTCATCCTGTCCATGGGCAACTTGCTCCGCTCTT
TTCGCTTCCACTTCAGGGGCACGGGATACGACGAGAAGATGGTGCGTGAG
ATGGAGGGACTGGAAGCCTCGGGGTCCACATACGTCTGCACTCTGTGTGA
CACCAGTCGTGCCGACGCTTCAGAGAACATGGTCCTGCACTCCATCACCC
GCAATCATGTGGAGAACCTAGACCGTTACGAAATATGGAGGACCAACCCT
TTTTCGGAGTCAGTGGAGGAGCTGCGGGAGAGAGTCAAAGGCGTCTCTGC
CAAGCCCTTTTTGGAGACTCAGCCCACACTCGATGCATTGCACTGTGACA
TTGGCAACGCCACTGAGTTCTACAAAATCTTCCAGGACGAGATTGGGGAA
GTTTACAAAAAGGT---CAAC---CCCAGCCGGGAGGAACGGCGCAGCTG
GCGGGCAGCCCTAGATAAACAGCTGAGGAAGAGGATGAAGCTTAAACCAG
TAATGAGGATGAATGGGAACTATGCCCGCAGGCTAATGACCCAAGAGGCT
GTGGAGGTGGTGTGTGAGCTGGTGCCCTCAGAGGAGAGGAGGGAGGCCCT
GAGGGAGCTTATGAGGCTCTATATTCAGATGAAGCCCGTGTGGCGTGCCA
CCTGCCCAGCCAAGGAGTGCCCTGACCAGCTGTGCCGCTACAGTTTTAAC
TCACAGAGCTTCGCCGACCTCCTCTCCTCTACCTTCAAATATAGGTACAA
TGGAAAGATAACCAATTATCTGCACAAGACCCTTGCTCACGTGCCCGAGA
TCATTGAGCGAGATGGATCTATCGGAGCCTGGGCCAGCGAGGGGAGCGAG
TCGGCTAACAAATCGTACAACATCGAGATGGGGGCCCTGGGCCCCCGGTG
GAACGACAACCCACAGCCCTTCTCCTGCTCCATCGAAGACCCCACCAAGC
AGACCAAGTTCAAAGGCATCAAGACCTACATTTCATACCGGGTCACGCCG
AGCCACACGGGACACCCCGTGTACAGGCGATACAAACACTTCGACTGGCT
CTACAACCGCCTGTTGCACAAGTTCACTGTGATCTCCGTGCCTCACCTGC
CCGAGAAGCAGGCCACGGGACGCTTCGAGGAGGACTTCATCGAGAAGCGC
AAGAGGCGGCTGATCCTGTGGATGAACCACATGACCAGCCACCCGGTCCT
CTCCCAGTACGAGGGCTTCGAGCACTTCCTCATGTGCGCCGACGACAAGC
AGTGGAAGCTGGGCAAGAGGCGGGCGGAGAAGGACGAGATGGTGGGCGCC
CACTTCATGCTGACCCTGCAGATCCCCACTGAGCACCAGGACCTGCAGGA
CGTCGAGGAGCGGGTGGACAACTTCAAGGCCTTCGCCAAGAAGATGGACG
ACAGCGTGTTGCAGCTCACGCACGTCGCCTCGGAGCTGGTGCGCAAGCAC
TTGGGTGGATTCAGGAAGGAGTTCCAGAGGCTGGGGAACGCGTTCCAGTC
CATCAGCCAGGCGTTCATGCTGGACCCGCCCCACAGGTCGGACACCCTCA
ACAACGCCATNNNNNNNNNNNNNNNNNNCGTTCCTCAAACTGACCTCTCTCGGTTTCATCATTGGCGT
CRGTGTGGTTGGAAACCTCCTGATCTCCATCCTGCTGGTCAAAGACAAGA
GCCTGCACCGAGCGCCCTACTATTTCCTGCTGGACCTGTGCGCCTCCGAT
ATCCTGCGATCTGCCATTTGCTTCCCCTTTGTCTTCACGTCGGTCAAAAA
TGGGTCTGCGTGGACCTACGGCACGTTGACCTGCAAGGTGATCGCCTTCC
TCGGGGTGCTCTCCTGTTTCCACACGGCGTTTATGCTCTTCTGTGTCAGC

GTGACCCGTTACCTGGCCATCGCACATCACCGTTTCTACACCAAGCGGCT
GACTTTCTGGACGTGCTTGGCTGTCATCTGCATGGTGTGGACGCTATCCG
TGGCTATGGCGTTCCCGCCGGTGCTGGACGTAGGGACGTACTCGTTCATC
CGGGAGGAGGACCAGTGCACGTTCCAGCACCGCTCCTTCAGGGCCAACGA
TTCGCTGGGCTTCATGCTCCTGCTGGCGCTCATCCTCCTCGCCACACAGC
TGGTTTACCTCAAGCTCATCTTCTTCGTCCACGACCGTCGAAAGATGAAG
CCCGTCCAGTTCGTGCCTGCCGTCAGCCAGAACTGGACCTTCCACGGGCC
GGGCGCCAGCGGGCAGGCGGCGGCCAACTGGCTGGCTGGATTCGGTCGAG
GCCCCACCCCGCCTACTTTGCTGGGTATCCGTCAGAACAGCAACGCAGCG
GGCCGCAGGCGTCTACTGGTATTAGATGAATTCAAAACAGAGAAGCGGAT
TAGTAGGATGTTCTACATCATGACGTTTTTCTTCCTGGCACTCTGGGGGC
CCTACCTGGTCGCCTGCTACTGGCGGGTGTTTGCGAGGGGCCCTGTAGTT
CCTGGGGGCTACCTGACAGCAGCCGTGTGGATGAGCTTCGCTCAGGCTGG
GGTCAATCCTTTCATCTGCATTTTCTCCAACAGGGAGGCCAAATCTCGCT
TTCACCCTGGCGTGGGGACTGGTCCTGGCACGGAGC---GCAGCGTCCCA
CTCGGCAACAGCTTGCTATCCCCGCAGCAAACCGAGGAGCCCACTGTTGC
CACCCCCCCGCAGCGATGGTTTGTCACCC---CTGCCAACAACCGACTGG
ACTTTGCTGCCTCGGCATACGACGCCGCG---------GATTTCGCCGGT
AACGCGGCCACCTTGCTGTCCTACGCAGCGGCCGGAGTGAAGGCTC----
--TTCCCCTGCCGACTGCAGGCTGCTCCAATCGGCCCCTTGGCTATTACG
CAGACCCGTCTG---GCTGG---GGAGGACGCACGCCGCCGCAGTACTGT
GGCGTAAATAGTAAATCCAGCTCGGTCTTTTCCTGCTGGCCCGCGAACTC
CATCGGCGGCCGAGCGGGGG---CC---AACTACCTGT---------CCG
AGGA---GGGC---GACTC---CATCACGACGGAGAGGTCGCCC---AT-
--CGGCGGCTCGGAGGAG---ACCAAACCCAAAGACATGAC---GTCCGA
---GTCGAGCTGGATAGAG---ACGCCGTCCTCCATTAAGTCAATCGATT
CCAGCGACTCTGGCATCTTTG---AGCAGGCCAAACGGAGGAGAATCTCC
CCCTCAGCCACGCCG---------GTTTCAGGGACAGTTTCCCCGTTAAA
ATCGGAGNNNNNNNNNNCAGGCGAAGTCACAGAGAGAGAAGTGGCGTTGGGGATAAATCC
GTTCGCAGATGGGATGGGCGCCTTCAAAATAAACCACAGCTCCCACGATA
TTGGCTCCGG---ACAGACGGCGTTTTCCTCCCAGGCG---CCCGGCTAC
---GCGGCAGCCGCACTGGGA---CACCACCA------CCACCCGACCCA
CGTTGGCTCT---TACTCCACGGCGGCTTTCAACTCCACCAGGGACTTTC
TCTTCAGAAATCGGGGTTTCGGGGATGCCACCGG------------GGCG
CAGCACAGTTTGTTCGCCTC---------CGGAAGTTT---C------GC
AGGGCCACATGGACACTCAGATGCAGCGGGGCACCTGCTCTTCCCGGGGC
TCCACGAG---CAAGCGGCGAGCCACGCGTCTTCGAACGTGGTCAACAGC
CAGATGCGGCTGGGCTTCTCGGGGGACATGTACGGACGGGCCGACCAGTA
CGGCCACGTTACGAGCCCGAGGT---CCGACCACTATGCTTCGACCCAGC
TGCACGGCTACGGCCCCATGAACATGAATATGGCCGCA---CACCACGGA
GCAGGGGCCTTCTTTCGATACATGAGGCAGCCCATCAAACAAGAGCTCAT
CTGCAAGTGGATCGAGCCGGAGCAGCTGACGAATCCCAAAAAGTCGTGCA
ACAAAACTTTCAGCACGATGCACGAGCTGGTGACCCATCTGACGGTGGAG
CATGTGGGGGGACCCGAGCAGACCAACCACATTTGCTTCTGGGAGGAATG
CGCCAGAGAAGGGAAACCGTTCAAAGCCAAATACAAACTTGTAAATCATA
TCAGAGTTCACACCGGAGAAAAGCCCTTCCCGTGTCCGTTCCCCGGCTGT
GGCAAA
>Lachnolaimus maximus
AGCCTTCTCATTCGAGCAGAACTAAGCCAACCAGGCGCCCTTCTAGGGGA
CGACCAGATTTATAACGTCATTGTTACGGCGCATGCATTTGTAATAATTT
TCTTTATAGTAATGCCAATTATGATTGGAGGGTTTGGAAACTGACTTATC
CCACTAATAATTGGAGCCCCTGACATGGCTTTCCCCCGAATAAACAACAT
AAGCTTTTGACTTCTCCCACCTTCATTTCTTCTCCTCTTGGCATCCTCAG

GGGTAGAAGCCGGGGCTGGAACCGGATGAACAGTTTACCCCCCGTTAGCA
GGCAATTTAGCCCATGCAGGGGCCTCTGTTGATCTAACCATCTTTTCTCT
GCATCTAGCAGGCATTTCTTCAATTCTTGGAGCAATTAATTTTATTACAA
CCATTATTAATATGAAACCCCCCGCCATCTCCCAATATCAGACCCCGCTG
TTTGTTTGGGCAGTTCTAATTACAGCAGTGCTCCTGCTCCTGTCCCTTCC
TGTCCTTGCTGCTGGGATTACAATGCTCTTAACAGACCGAAACCTAAACA
CCACCTTCTTCGACCCCGCAGGAGGGGGAGACCCAATTCTATATCAACAC
CTCTTC--

------------TTCTTGGAAAGAAACCTTCACCCGTCAAACTGCCTTGG
CATGCTGTTGTTGTCTGATGCCCACCAGTGCACCAAGCTGTCAGAGCTCT
CCTGGGGCATGTGCCTCAGCAACTTTCCCGCTATTTGCAAGACAGAGGAC
TTCCTCCAACTACGCAAAGATATGGTGGTGCAGCTCTTGTCACATGAGGA
GCTAGAGACAGAAGATGAGAGACTGGTTTATGAAGCTGCCCTCAACTGGA
TCAACTATGACTTGGAAAGGAGACACTGCCACCTTCCAGAGCTCCTGAGA
ACGGTTCGTCTGGCGCTGCTTCCAGCTATATTTCTCATGGAGAACGTATC
CACAGAGGAGCTGATCAACGCCCAGGCCAAGAGCAAGGAGCTGGTGGATG
AAGCTATCCGGTGTAAACTGAAGATCCTGCAGAATGATGGCGTTGTCAAC
AGCCCATGTGCCCGGCCAAGAAAAACCAGTCATGCCCTATTTCTTCTGGG
AGGGCAGACTTTCATGTGCGACAAGTTGTACCTGGTGGACCAGAAAGCCA
AAGAAATCATCCCAAAGGCTGACATACCCAGCCCTAGAAAAGAGTTCAGC
GCCTGCGCCATCGGCTGTAAGGTGTACATTACTGGTGGTA--GAGGCTC-
AGAGAATGGTGTGTCCAAAGATGTATGGGTCTACGACACCGTGCATGAGG
AATGGTCAAAGGCGGCGCCCATGCTCATTGCCAGGTTTGGCCATGGCTCT
GCAGAGCTGAAACACTGCCTCTATGNNNNNNNNNNNNNNNNNNNNNNNNNNNNNNNNNNNNNNNNNNNNNNN
NNNNNGGACGAATACATTGTTGTGTTTAGT
CGTTCAACAACGAGGCTGATACTGAATGAAGCAGAGTTAATCATGGTTCT
GGCCCAGGAGTTCCAGATGAGAGTTGTCACTGTATCCCTGGAGGAACAGT
CTTTCTCCAGTATTGTCCGGGTGATCAGCGGTGCTTCCATGTTAGTCAGT
ATGCATGGAGCTCAGCTTATCACCTCACTCTTCCTCCCCAGGCGTGCTAC
CGTTGTGGAGCTCTTTCCCTTTGCTGTGAACCCAGAGCAGTACACCCCCT
ATAAAACACTTGCCTCCCTTCCAGGAATGGACCTTCACTATATCTCTTGG
AGAAACAATAAGGAGGAGAACACCATCACCCACCCAGACAGACCCTGGGA
ACAAGGGGGCATCACTCACTTGGAGAAAGACGAGCAAGAGCGAATACTTT
CAAGCAAAGATGTCCCTAGGCACCTATGCTGCCGCAACCCTGAGTGGCTC
TTCCGGATCTACCAGGACACTTTGGTGGACATCCCTTCCTTCTTGGAAGT
CCTCAG---AGAGGGAATGAAA---ACAAAACCGAGCATGAAAAA---GT
CAAAGCCAGCCAGCACAGTCCACCCAGGCCGGGTCAGAGAACCCCAGTGT
CAGACCTCAGTACAAACCACCAATGAGGCTAAACTCACCGTGTCTTGGCA
GATCCCATGGAATTTAAAATACCTAAAGGTGAGAGAAGTAAAGTACNNNNNNNNNNNNNNNAAAA
AAGACACTAGCAAGGGGACACTGGAGGATCAAATCATCCAGGCGAACCCT
GCGCTGGAGGCCTTCGGCAATGCCAAAACTTTGAGAAATGACAACTCATC
ACGATTTGGAAAATTCATCCGAATTCACTTTGGTATGAGTGGCAAGCTGT
CATCTGCTGACATCGAGACGTACCTGCTGGAGAAGTCACGTGTCACCTTT
CAGCTCAAGGCTGAGAGGAACTATCACATCTTCTACCAAATCCTGTCCAA
TCAGAAGCCAGAGCTGCYGGACATGTTGCTGATCACCAACAACCCATACG
ACTACTCCTACATCTCCCAAGGAGAGGTTACAGTTGCCTCCATCAACGAC
TCTGAGGAGCTGATGGCTACCGACAGCGCCTTTGATGTGCTYGGCTTCAC
TGCAGAGGAGAAGATGGGCGTCTATAAATTGACCGGTGCCATCATGCACT
ATGGTAACATGAAGTTCAAACAGAAGCAGCGTGAGGAGCAGGCTGAGCCA
GATGGGACGGAGGCTGCCGATAAATCAGCTTACCTAATGGGGCTGAACTC

```
TGCTGACCTCATCAAAGGGCTGTGTCATCCCAGAGTCAAGGTGGGAAATG
AATATGTCACCAAAGGCCAAAGTGTGGACCAAGTCTACTATCCCAACAAG
GAGGCCTTCAAGTGTGAAGAGTGTGGGAAGCACTACAACACCAAGCTGGG
ATACAAGCGTCATGTGGCCATGCACTCCGCCACGGCGGGAGATCTCACCT
GTAAAGTGTGCATGCAGACGTATGAGAGCACGCCAGTGCTCCTGGAGCAC
CTGAAGAGCCACTCCGGGAAGTCCTCAGGAGGCACCAAGGAGAAGAAACA
TCCATGTGACCACTGTGACCGTCGCTTCTACACGCGGAAGGACGTGCGGC
GACACATGGTCGTCCACACCGGCCGCAAGGACTTCCTGTGTCAGTACTGC
GCCCAGCGCTTTGGCAGGAAGGACCACCTGACCCGTCACGTTAAGAAGAG
CCACTCGCAGGAGCTGCTGAAGATCAAGACAGAGCCTCCAGACATGCTGG
TGCTGTTAGCGTCAGGGTCGCCCCCCTGCCCGGTGAAAGAGGAGCTCAGC
CCCATGATGTGCGGCATGGGGCCCAACAAAGACCCCATGATGGGCAAACC
ATTCCCAAGCGGGCCCCCGTTTCCAATGGCATGTATAACCCCCACCAC-
-----CTCCAGACCATGTCTAACTCTGGGGTCGGTCACCCC------CAC
CCATCTCTGATGCCCCCCACCCTGTCTGCAGCAATGGGCATGGGCTGTCA
CATGGACTACCTCATATATGCCTCTTTCTCATTCATGGGATGTTTACAAA
TCAGCGACGGATCTAATATAGTGAACCTCCTGGCTAGTAACTCTCCGAGT
GTTGCCTATGCTATGACCCAGCAGAAATACTTCAGTAACTACAGTCCTGT
TATTGGGTTTTACATTTATGAGCCTATTGAGTATTGGAACTCCACAGTGC
AGGAGCACCTGAAGACTCTGAGTCATGGCTTCAACAAGATATCCTGGATG
GACAACTTCTTCCACTACCTGAGGGTGGTGAATGTGAGTGCATCAACCAA
GAGTGATTTTATTACTATACTTAGGAGCTCATTTCTGCGCAGTCCGGAGT
ACCAGCACTTCACTGAGGATATCATATTCTCCAAGA---ACCATGAGACT
G------ATGAGTATGACATTATCGCCTCAAGGATGTACTTGGTGGCACG
GACAACGGAAAAGAAGCGTGAGGAAGTGGTGGAGCTTCTGGAAAAGCTTC
GTCCGTTGATGCTGATCAACAGCATCAAGTTCATTGCCTTCAATCCTACA
TTTGTGTTCATGGACCGCTACAGCTCCTCTGTCATCTCACCCATCCTGAC
TTCAGGCTTTAGYGTACTCACAATCCTCATCCTTACTTTTTTCCTGGTCA
TCAATCCCTTGGGGAACTTCTGGCTAATTCTCACAGTAACCTCCGTAGAG
CTGGGCGTCTTAGGTTTGATG-----------------------------
--------------------------------------------------
--------------------------------------------------
--------------------------------------------------
--------------------------------------------------
--------------------------------------------------
--------------------------------------------------
--------------------------------------------------
--------------------------------------------------
--------------------------------------------------
--------------------------------------------------
--------------------------------------------------
--------------------------------------------------
--------------------------------------------------
--------------------------------------------------
--------------------------------------------------
--------------------------------------------------
--------------------------------------------------
--------------------------------------------------
--------------------------------------------------
--------------------------------------------------
--------------------------------------------------
--------------------------------------------------
--------------------------------------------------
```


----------------------------NNNNNNNNNNTTGAGATGGCTCCCTTGGGGCC
TGGGTGGAAGGAAAGTCCACAGCCTTTCTCCTGCTCCATCGAAGACCCAA
CAAAACAGACAAAGTTCAAGGGCATCAAGACGTACATTTCCTACCGGGTT
ACGCCTAGCCACACAGGGCGTCCCGTCTACAGACGTTACAAACACTTTGA
CTGGCTGTACAACCGCTTGCTGCACAAGTTCACTGTGATCTCAGTGCCTC
ACCTGCCTGAGAAGCAGGCCACGGGGCGATTTGAGGAAGACTTCATTGAG
AAGCGTAAGAGGCGACTGATACTCTGGATGAACCACATGACAAGTCACCC
GGTTCTCTCCCAGTATGAAGGCTTTGAGCACTTTCTGATGTGTGGTGACG
ACAAGCAGTGGAAACTGGGCAAGAGACGAGCGGAGAAGGACGAGATGGTT
GGTGCACACTTCATGCTGACCCTTCAGATCCCCAATGAACACCAGGACCT
TCAGGATGTAGAGGAGCGGATCGACTCCTTCAAGGCCTTTGCTAAGAAAA
TGGACGACAGTGTGATGCAGCTCACGCATGTGGCCTCTGAGCTGGTCCGT
AAGCACCTGGGCGGGTTCAGGAAGGAGTTCCAGCGGCTGGGAAACGCCTT
CCAGTCTATCAGCCAGGCATTCATGCTGGACCCTCCACACAGCTCAGAAA
CATTCAACAACGCCATTTCCCACNNNNNNNNNNNNNNNNNNAAACTGACCTCTCTGGGTTTTATTATT
GGAGTCGGCGTGGTTGGAAACCTCCTGATCTCCATCCTGCTGGTCAAAGA
CAAGAGCTTGCACCGAGCGCCCTATTATTTCCTGCTGGATTTGTGCGCTT
CTGACATCCTGCGCTCCGCCATCTGCTTCCCCTTCGTCTTCACCTCAGTC
AAGAATGGATCTGCCTGGACGTACGGCACGCTGACCTGCAAAGTCATTGC
CTTYCTGGGTGTGCTCTCCTGTTTCCACACGGCGTTTATGCTGTTCTGCG
TTAGCGTCACGCGCTACCTTGCCATTGCACATCACCGTTTCTACACAAAA
AGGCTGACCTTCTGGACCTGTTTGGCTGTTATCTGCATGGTGTGGACGTT
GTCGGTGGCAATGGCGTTTCCGCCGGTGCTGGACGTAGGGACGTACTCGT
TTATCCGCGAGGAGGACCAGTGCACGTTCCAGCACCGCTCCTTCAGGGCG
AACGATTCCTTGGGATTCATGCTACTGCTGGCGCTTATTCTCCTCGCCAC
ACAGCTGGTTTACCTCAAGCTCATCTTCTTCGTCCACGACCGCCGAAAAA
TGAAGCCCGTCCAGTTCGTGCCCGCCGTCAGCCAGAACTGGACCTTCCAC
GGGCCGGGCGCAAGCGGGCAGGCGGCGGCCAACTGGCTGGCTGGATTCGG
CCGAGGCCCGACTCCGCCTACTCTGCTGGGAATCCGGCAGAACAGCAACG
CAGCGGGCCGCAGGCGTCTTTTGGTGTTGGATGAGTTTAAAACGGAGAAG
AGGATTAGTAGGATGTTCTACATCATGACGTTTTTCTTCCTGGCGCTGTG
GGGGCCCTATCTGGTCGCCTGCTACTGGCGGGTGTTTGCTAGGGGCCCTG
TGGTCCCCGGGGGCTACCTGACGGCTGCCGTGTGGATGAGCTTCGCCCAG
GCTGGNNNNNNNNNNNNNNNNNNNNNNNNNNNNNNNNNNNNN----------------------------
----

-------------------------CATCACTCAACAGGCGAAGTCACAG

AGAGAGAAGTGGCGTTGGGGATAAATCCGTTTGCAGATGGGATGGGCGCC
TTCAAAATAAACCACAGCTCCCACGATATCGGCTCCGG---ACAAACGGC
GTTTTCCTCCCAGGCG---CCCGGCTAC---GCAGCAGCCGCCCTGGGA-
--CACCACCA------TCACCCGACCCACGTTGGCTCT---TACTCCACG
GCGGCGTTCAACTCCACCAGGGACTTTCTCTTCAGAAATCGGGGTTTCGG
GGATGCAGCCGG------------GGCGCAGCACAGTTTGTTCGCTTC--
-------CGGAAGTTT---C------GCAGGGCCACATGGACACTCAGAT
GCAGCGGGACACCTGCTCTTCCCGGGGCTCCACGAG---CAAGCTGCGAG
CCACGCGTCTTCCAACGTGGTCAACAGCCAGATGCGGCTGGGCTTCTCGG
GGGACATGTACGGAAGGGCCGACCAGTACGGCCACGTTACGAGCCCGCGG
T---CCGACCACTACGCCTCGACCCAGCTGCACGGCTACGGCCCCATGAA
CATGAATATGGCCGCA---CACCACGGAGCAGGGGCCTTCTTTCGGTACA
TGAGGCAGCCCATCAAACAAGAGCTCATCTGCAAGTGGATCGAGCCGGAG
CAGCTGACGAACCCCAAAAAGTCGTGCAACAAAACTTTTAGCACGATGCA
CGAGCTCGTGACCCATCTGACGGTGGAGCATGTGGGGGGACCGGAGCAGA
CGAACCACATCTGCTTCTGGGAGGACTGCGCCCGAGAGGGGAAGCCGTTC
AAAGCCAAATACAAACTTGTGAATCATATCAGAGTACACACCGGAGAGAA
GCCCTTTCCGTGCCCGTTCCCCGGCTGTGGCAAA
>Lactophrys triqueter
AGCCTACTCATCCGAGCAGAACTGAGCCAACCAGGCGCTCTCCTTGGGGA
TGATCAGATTTACAATGTTATTGTCACGGCACATGCATTCGTGATAATTT
TCTTTATAGTTATACCCATCATAATCGGAGGTTTTGGAAACTGACTAGTC
CCATTAATAATTGGGGCCCCCGACATAGCATTTCCCCGAATAAATAACAT
AAGCTTTTGACTTCTTCCCCCTTCCTTTCTTCTTCTCCTAGCTTCATCAG
GAGTTGAAGCAGGGGCAGGAACTGGTTGGACAGTTTACCCGCCCTTAGCA
GGAAACCTAGCACACGCGGGAGCATCCGTAGATTTAACTATTTTTTCACT
TCATCTAGCGGGAGTCTCCTCAATTCTAGGGGCTATTAATTTTATTACCA
CCATTATTAACATAAAACCCCCTGCTATCTCTCAATATCAAACCCCCTTA
TTTGTTTGGGCTGTCTTAATTACCGCCGTTCTTCTCCTCTTATCTCTACC
AGTTCTTGCTGCCGGCATCACAATGCTCCTAACAGACCGAAATCTCAACA
CCACCTTCTTTGACCCAGCAGGAGGCGGAGACCCAATCCTCTACCAACAC
CTATTCTGATTCTTCGGTCA------------------------------

------------TTTCTAGAGAGAAACCTTCACCCATCTAACTGCCTTGG
CATGCTATTACTGTCTGATGCCCACCAGTGCACCAAGCTGTCAGAGCTCT
CCTGGGGCATGTGCCTCAGTAACTTCCCCGCTATTTGCAAGACAGAGGAC
TTCCTCCAACTGCCCAAAGACATGGTGGTTCAGCTTTTGTCACACGAGGA
GCTAGAGACTGAAGATGAAAGACTGGTTTATGAAGCAGCGCTCAATTGGA
TCAACTATGACCTGGACAAGAGGCATGGCCACCTTCCAGAGCTCCTGAGA
ACGGTCCGCCTAGCCCTTCTGCCTGCCATCTTTCTCATGGAGAATGTTTC
TACAGAAGAGTTGATCAACGCCCAAGCGAAGAGCAAGGAGCTGGTGGATG
AAGCTATCCGCTGTAAGCTGAAGATCCTGCAGAATGATGGCGTTGTCAAC
AGCCCATGTGCTCGACCAAGAAAAACCAGCCATGCCCTCTTTCTTCTGGG
TGGGCAGACTTTCATGTGTGACAAGTTGTACCTGGTGGACCAGAAAGCCA
AAGAGATCATCCCCAAGGCTGACATTCCTAGCCCCAGGAAGGAGTTCAGC
GCCTGCGCCATTGGCTGTAAGGTGTATATCACTGGTGGGA--GAGGCTC-
AGAGAACGGTGTGTCCAAAGATGTGTGGGTCTACGACACTGTCCACGAGG
AATGGTCAAAGGCCGCACCCATGCTCATTGCTAGGTTTGGCCATGGCTCT
GCAGAGCTGAAACACTGCCTCTACGTGGTAGGAGGTCACACTGCAGCAAC
TGGTTGCCTCCCGGCCTCTCCGTCTGGATGATTATATTGTTGTATTTAGT
CGTTCAACAACAAGGCTCATACTGAATGAAGCAGAGCTAATTATGGCTCT

GGCCCAGGAGTTCCAGATGAGAGTGGTCACGGTATCTCTGGAGGAGCAGT
CCTTTCCCAGTATCGTCCAGCTGATCAGCAGTGCTACCATGTTAGTCAGT
ATGCATGGAGCTCAGCTTATCACCTCACTCTTCCTCCCCAGAGGAGCTGT
TGTGGTTGAGCTGTTCCCTTTTGCTGTGAACCCAGAACAATATACCCCAT
ATAAAACCCTTACCTCCCTTCCAGGCATGGACCTTCACTATATCTCCTGG
AGGAACGCTAAGGAGGAAAACACCATCACCCACCCAGACAGACCCTGGGA
TCAGGGGGGCATTGTTCACCTAGAGAAAGAGGAGCAAGTGCAAATACTGG
CGAGCAAAGATGTCCCCAGGCACCTGTGCTGCCGCAATCCANAGTGGCTTT
TCCTGATCTACCACGACACTTTGGTGGACATCCCTTCCTTTCTGGAAGTC
CTCAA---AGAGGGCTTGAAG---ACAAGGCCCAGTCTGAAGAA---GTC
AAAGCCAGCCAGCACACTTCATCCAGGCCGAGTCAGAGAACCTCAGTGTC
AGACCTCGGTACAAACCACCAATGAGGCTAAACTCACAGTATCCTGGCAG
ATTCCATGGAATCTGAAATACCTAATGGTCGGAGAGGTGAAATACGAGGT
GTGGATCCAGAGAAAAGACACCAGCAAGGGGACACTGGAGGATCAAATCA
TCCAGGCAAACCCGGCGCTGGAGGCCTTTGGCAACGCCAAAACACTGAGA
AATGACAACTCGTCTCGTTTTGGAAAATTTATCCGAATTCACTTTGGTAC
GAGTGGCAAGCTGTCATCTGCTGACATTGAGACATACCTACTGGAGAAGT
CACGGGTCACCTTCCAGCTCAAGGCTGAGAGAAATTACCACATCTTCTAC
CAGATCCTGTCCAATCAAAAGCCAGAGCTGCTTGACATGCTGCTGATCAC
CAACAACCCATACGACTACTCCTACATCTCCCAAGGAGAGGTATCAGTCG
CCTCCATCAACGACTCAGAAGAGTTGATGGCCACCGACAGTGCCTTCGAT
GTGCTTGGCTTCACTCCAGATGAGAAGATGGGTGTCTACAAACTGACGGG
AGCCATCATGCACTACGGCAACATGAAGTTCAAACAGAAACAGCGTGAGG
AGCAGGCTGAGCCCGATGGGACAGAGGCTGCTGATAAATCGGCTTACCTA
ATGGGGCTGAACTCTGCAGACCTCATCAAAGGGCTGTGCCACCCCAGAGT
CAAGGTAGGAAATGAATATGTGACCAAAGGCCAAAGTGTGGACCAAGTCT
ATTACCCCAACAAGGAAGCCTTCAAGTGTGAAGAATGTGGGAAGCACTAC
AATACCAAGCTCGGATATAAGCGCCATGTGGCCATGCACTCTGCCACGGC
AGGTGATCTCACCTGTAAAGTATGCATGCAGACCTATGAGAGCACACCTG
TGCTTCTGGAGCACCTCAAGAGCCATTCTGGGAAGTCCTCTGGGGGCACC
AAGGAGAAGAAACACCCTTGTGACCACTGTGACCGTCGTTTCTACACACG
AAAGGATGTGAGACGGCACATGGTGGTCCACACAGGACGAAAGGACTTTC
TATGCCAGTACTGCGCCCAGCGCTTTGGTAGGAAAGACCATCTGACACGC
CATGTAAAGAAGAGCCACTCGCAGGAGCTACTGAAAATCAAGACAGAGCC
GCCTGATATGTTGGGCCTTTTAGCTTCTGGGTCACCACCTTGCTCYGTGA
AGGAGGAGCTCAGCCCAATGATGTGTGGCATGGGTCCCAACAAAGATCCC
ATGATGGGCAAACCGTTCCCCAGCGGAGCCCCTTTTCCAATGGGCATGTA
CAACCCCCACCAC------CTCCAGGCCATGTCTACTTCTGGGGTGGGTC
ACCCA------CACCCGTCCCTAATGCCCNCTTCCTTGTCTGCAGCTATGA
GCATGGGCTGTCATATGGAANNNNNNNNNNNNGCATCTTTCTCATTCATGGGATGTTTACAA
ATCAGTGATGGATCAAATATTGTGAACTTGTTGGCCAGTAACTCTCCAAG
TGTTTCATATGCTTTGACCCAGCAAAAATACTTCAGTAACTACAGTCCTG
TGATTGGGTTTTATATTTATGAGCCCATTGAGTACTGGAACTCGACAGTG
CAGGAGCACCTGAAGACTCTTAGTCATGGCTTCAATAAGATCTCCTGGAT
GGACAACTTTTTCCACTACTTGAGGGTGGTGAATGTGAGTGCGTCAACCA
AGAGTGACTTCATCACCATCCTCAAGGGCTCCTTCTTGCGCAGCCCTGAG
TACCAGCACTTCACTGAGGACATCATATTCTCTAAGA---ACCGTGAGAC
TG------ACGAGTATGACATTATTGCCTCAAGGATGTACTTGGTGGCAC
GGACAACAGAGAAGAAGCGCGAAGAGGTGGTGGAGCTTCTGGAAAAGCTT
CGTCCACTGATGCTGATCAACAGCATCAAGTTCATTGCCTTCAATCCAAC
ATTTGTGTTCATGGACCGCTACAGCTCCTCTGTCATCTCGCCTATCCTGA
CCTCAGGATTCAGCGTACTCACAATCCTCATCCTCACTTTCTTCCTGGTA
ATCAACCCCTTGGGTAACTTCTGGCTCATCCTCACTGTAACGTCCGTAGA

GCTTGGGGTATTGGGTCTGATGGGCTTTCACCAGTTTGAATGGCAGCCAG
CTCTCAAGAATGTGTCTACATCTTGCAATGTAGGAATTATTAATGGGCTC
TCTGGATGGGTTTCTTCAGTGGATGACTCCCCAGCTGACACCATCACTCG
GCGGTTTCGCTATGATGTAGCACTGGTGTCAGCGTTAAAGGATCTGGAAG
AGGACATCCTGGAAGGGCTGAGAGAAAATGGAATGGAGGATAGAGCTTGC
ACCTCAGGCTTCAGTGTCATGATCAAAGAATCCTGTGATGGCATGGGCGA
TATCAGCGAGAAGCACGGTGGAGGACCAGTTGTTCCTGAGAAGGCTGTGC
GTTTTTCTTTCACTATTATGTCTGTCTCTGTCCTGGCAGACGGTGACGAA
-------------------------------GATGAAGTGAGAATCTACAC
AGAGCCAAAGCCAAACTCAGAGCTGTCGTGTAAGCCCCTTTCCCTGATGT
TTGTGGATGAATCAGACCATGAGACACTCACAGCCATTCTGTGGCCAATA
ATCGCAGAGCGTAACGCAATGAAAGAGAGCAGACTCATTCTATCCATCGG
TGGACTATCTCACTCCTTCCGGTTTCACTTCAGAGGCACAGGATATGATG
AGAAGATGGTGCGTGAGATGGAGGGCCTCGAGGCCTCTGGATCCACTTAC
ATCTGCACTCTTTGTGACTCTAGTCGCGCAGATGCCTCTCAAAACATGGT
GCTACACTCCATCACTCGCAGTCATGAGGAGAATCTAGAACGTTATGAAA
TATGGAGAACTAACCCCTTTTCTGAGTCTGTCGACGAGCTCCGAGACAGG
GTCAAAGGGGTCTCTGCCAAGCCCTTCATGGAAACCCATCCCACATTGGA
CGCACTACACTGTGACATAGGCAATGCCACTGAGTTTTACAAAATCTTCC
AGGATGAGATTGGGGAGGTGTACCTGAAGGT---CAAC---CCCAGCCRG
GAGGAGCGGCGCAGCTGGAGAGCAGCCCTTGATAAACAGCTGAGAAAGAA
GATGAAGCTCAAACCGGTAATGAGGATGAATGGGAACTATGCCCGCCGGC
TAATGACCCTGGAGGCTGTGGAGGTGGTGTGTGAGTTGGTGCCCTCAGAG
GAGAGAAGGGAGGCCCTGAGGGAACTTATGAGGCTCTACCTCCAGATGAA
GCCTGTGTGGCGCACCACCTGCCCTGCCAAGGAGTGCCCTGACCAGCTGT
GCCGCTACAGCTTTAACTCTCAGCGCTTTGCAGAGCTCCTCTCCTCTACC
TTCAAGTACAGGTACAATGGAAAGATAACCAATTACCTGCACAAGACCCT
GGCCCATGTTCCTGAAATCATTGAGAGAGATGGATCCATAGGAGCCTGGG
CCAGCGAGGGGAATGAGTCGGCAAATAAANNNNNNNNNNNNNNNNNNNNNNNNNNNNNNNNNNNNNNNNTGGAT
GGAAAGCCCACAGCCT
TTCTCCTGCTCAATTGAAGATCCTACAAAACAGACAAAGTTCAAGGGCAT
CAAGACGTACATTTCGTACCGGGTCACGCCGAGCCACACAGGACATCCTG
TCTACAGGCGCTACAAGCACTTTGACTGGCTGTACAACCGCTTACTGCAC
AAGTTCACTGTGATCTCCGTGCCTCACCTGCCTGAGAAGCAGGCCACGGG
GCGATTCGAGGAAGACTTCATTGAGAAGCGCAAGAGGCGACTGATACTGT
GGATGAACCACATGACCAGTCACCCAGTCCTCTCCCAGTATGAAGGCTTT
GAGCACTTTCTGATGTGCGCTGATGACAAGCAATGGAAATTGGGCAAGAG
GCGCGCAGAGAAGGATGAGATGGTGGGGGCGCATTTCATGCTGACCCTCC
AGATTCCGAGTGAGCACCAGGACCTCCAGGATGTAGAGGAGCGGATCGAC
TCCTTCAAGTCCTTTGCTAAGAAAATGGATGACAGTGTGATGCAGCTCAC
ACATGTTGCCTCAGAGCTGGTGCGTAAGCATCTGGGTGGGTTCAGGAAGG
AGTTCCAGCGGCTGGGAAATGCCTTCCAGTCTATCAGCCAGGCCTTCATG
CTAGACCCTCCCTACAGCTCAGAGACCCTAGACAACGCCATCTCCCATNNNNNNNNNNNNNNNNNNNNAT
TGACCTCTCTGGGTTTCATCATCGGAGTCGGCGTGGTTGGGAACCTGCTG
ATCTCCATCTTGCTGGTCAAAGACAAGAGTCTGCACCGAGCGCCCTACTA
TTTCCTGCTGGACCTGTGCGCCTCTGACATCCTTCGATCCGCCATCTGCT
TCCCCTTCGTCTTCACCTCGGTCAAGAATGGATCTGCCTGGACGTACGGC
ACGCTGACCTGCAAAGTGATTGCCTTCCTGGGTGTGCTCTCCTGTTTCCA
CACAGCGTTTATGCTGTTCTGTGTCAGTGTCACGCGCTATCTGGCCATCG
CTCATCACCGTTTCTACACTAAGAGGCTGACCTTCTGGACCTGCCTGGCT
GTCATCTGCATGGTGTGGACGTTATCAGTGGCGATGGCGTTCCCGCCTGT
GCTAGACGTAGGGACGTACTCTTTTATCCGGGAGGAGGATCAGTGCACGT
TCCAGCACCGCTCCTTCAGGGCGAATGATTCGCTGGGCTTCATGCTCCTG

```
TTGGCGCTCATCCTCCTGGCCACACAGCTGGTTTACCTCAAGCTCATCTT
TTTTGTCCATGACCGTCGAAAGATGAAGCCCGTCCAGTTCGTGCCTGCTG
TTAGCCAGAACTGGACCTTCCACGGGCCAGGTGCCAGCGGGCAGGCGGCC
GCCAACTGGCTGGCCGGATTCGGTCGAGGCCCCACTCCGCCTACTTTGCT
GGGCATCAGACAGAACAGCAATGCAGCGGGCCGCAGGCGTCTACTGGTAT
TGGATGAATTCAAAACGGAGAAGAGGATTAGTAGGATGTTCTACATCATG
ACATTTTTCTTCCTGGCACTGTGGGGGCCCTATCTGGTCGCCTGCTACTG
GCGAGTGTTTGCWAGGGGCCCTGTGGTCCCTGGGGGCTACCTGACAGCAG
CTGTGTGGATGAGCTTTGCCCAGGCTGGGGTCAATCCTTTCATCTGCATNNNNNNNNNNNNNNNNNNNNN
NNNNNNNNNNNNNNNNNNNNT
GGGGACTGGTCCTGGCACGGAGC---GCAGCGTCCCACTCGGCAACAGCT
TGCTATCCCCGCAGCAAACCGAGGAGACCACTGTTGCCACCCCCCCGCAG
CGTTGGTTTGTCACCC---CTGCCAACAACCGACTGGACTTTGCTGCCTC
GGCATACGACGCCGCT---------GATTTCGCCGGTAACGCGGCCACCT
TGCTGTCCTACGCTGCGGCCGGAGTGAAGGCTC------TTCCCCTGCCG
ACTGCAGGCTGCTCCAACCGGCCTCTTGGCTACTACGCAGACCCGTCAG-
--GCTGG---GGAGGACGCACGCCGCCGCAGTACTGTGGTGTGAATAGCA
AATCCAGCTCGGTCTTTTCCTGCTGGCCTTCTAACTCTATCGGAGGCAGA
GC------------------------------CGAGGA---AGGG--
-GACTC---CATCCCGACAGAGAGGTCATCG---AT---TGGCGGCTCCG
AGGAG---ACCAAACCTAAAGACATAAC---GTCAGA---GTCGAGCTGG
ATAGAG---ACGCCGTCCTCCATTAAGTCCATAGATTCGAGCGATTCTGG
AATCTTTG---AACAGGCCAAACGGAGAAGGATCTCACCTTCTGCCACGC
CA---------GTTTCAGAGACAGTGTCCCCGCTGAAGTCTGAGCATCAC
TCAACAGGCGAAGTCACAGAGAGAGAAGTGGCGTTGGGGATAAATCCGTT
CGCAGATGGGATGGGCGCCTTCAAAATAAACCACAGCTCCCACGATATTG
GCTCCGG---ACAAACGGCGTTTTCCTCGCAGGCG---CCTGGGTAC---
GCAGCAGCAGCCCTGGGA---CACCACCA------CCATCCGACCCACGT
TGGCTCT---TACTCTACGGCGGCTTTCAACTCCACCAGGGACTTTCTCT
TCAGAAATCGGGGATTCGGGGACGCCACCGG------------GGCTCAG
CACAGTTTGTTCGCCTC---------CGGGAGTTT---C------GCAGG
GCCACATGGACACTCAGATGCAGCGGGGCACCTGCTCTTCCCGGGGCTCC
ACGAG---CAAGCGGCGAGCCACGCGTCTACCAATGTGGTTAACAGCCAG
ATGCGGCTGGGCTTCTCGGGGGACATGTACGGACGGGCCGACCAGTACGG
CCACGTTACAAGTCCGCGTT---CCGACCCCTACGCCTCGACCCAGCTGC
ACGGCTACGGCCCCATGAACATGAATATGGCCGCG---CACCACGGAGCA
GGGGCCTTCTTTCGATACATGAGGCAGCCGATCAAGCAAGAGCTCATCTG
CAAGTGGATCGAGCCGGAGCAGCTGACGAATCCCAAAAAGTCATGCAACA
AAACTTTCAGCACGATGCACGAGCTCGTGACCCATCTGACGGTGGAGCAT
GTGGGGGGGCCAGAGCAGACCAACCACGTCTGCTTCTGGGAGGAATGTTC
CAGAGAAGGAAAGCCGTTCAAAGCCAAATACAAACTTGTGAATCATATCA
GAGTACACACCGGAGAAAAACCCTTCCCGTGTCCGNNNNNNNNNNNNNNNNNN
>Lampris guttatus
AGTCTTCTAATCCGAGCTGAGCTCAGCCAACCAGGAGCCCTCCTGGGAGA
CGACCAAATCTATAACGTAATCGTCACCGCCCATGCCTTCGTAATAATTT
TCTTTATAGTCATGCCTATCATAATTGGAGGCTTTGGGAACTGGCTGATT
CCCTTAATAATTGGCGCCCCTGATATGGCTTTTCCCCGAATGAACAACAT
AAGCTTCTGGCTTCTTCCCCCCTCTTTCCTACTCCTCCTAGCCTCCTCCG
GAGTGGAGGCCGGGGTAGGAACAGGCTGAACCGTATACCCCCCTCTAGCA
GGGAACCTAGCCCACGCAGGAGCCTCGGTTGACCTAGCCATTTTCTCCCT
CCACCTAGCAGGGGTTTCCTCTATTCTAGGAGCAATTAACTTTATTACGA
CAATTATTAATATGAAACCCCCGGCCATCTCTCAATACCAGACCCCTCTC
TTCGTATGGGCCACCCTGATTACGGCTGTTCTCCTTCTCCTCTCCCTCCC
```

TGTCTTAGCCGCCGGAATTACGATACTCCTAACAGATCGAAACCTTAATA
CTACATTCTTTGACCCCTCTGGAGGAGGAGACCCCATTCTATATCAACAT
TTGTTC--

-------------------------GGATGAATACATTGTTGTATTCAGC
CGTTCAACAACTAGACTGATTCTGAATGAACCTGAGCTGATCATGGCACT
GGCCCAGGAGTTTCAGATGAGAGTGATTACAGTGTCCCTGGAGGAACAAT
CTTTCCCAAGTATTGTTCAGGTTATCAGCGGGGCCTCCATGTTGGTGAGT
ATGCATGGAGCACAGCTTGTCACCTCACTCTTCCTTCCTCGGGGAGCTGC
CGTGGTGGAGCTGTTCCCCTACACTGTGAACCCAGAACAGTACACGCCTT
ATAAAACCCTCGCCTCCTTGCCAGGCATGGACCTTCAGTATGTTTCTTGG
AGGAACACAAAGGAAGAGAACACTGTCACTCACCCAGACAGACCCTGGGA
CCAAGGGGGAATTGCCCACTTGGAAAAGGATGAGCGAGAGCGTATACTGG
CCAGCAAGGAGGTCCCCAGACATCTATGCTGCCGCAACCCGGAATGGCTC
TTCCGAATCTACCAGGATACATTAGTGGATATCCCGTCATTTCTGGAGGT
CCTTAA---AGAGGGCATAAAG---GTCAGACCCAGCTTGAAGAA---GT
CCAAACCTACCAGTGCAGTTCACCCGGGCCGGGTCAGAGAACCCCAGTGC
CAGACTTCAGTCCAAGCCACCAATGAGGCAAAACTTACCGTCTCTTGGCA
GATCCCATGGAATCTGAAATACCTGAAGGTGAGAGAAGTGAAGTATGAGG
TTTGGATCCAG---------------------------------------

------CCCAACAAGGAGGCCTTCAAGTGTGAGGAGTGTGGCAAGCACTA
CAACACCAAGCTGGGATACAAGCGCCATGTGGCCATGCACTCTGCCACGG
CAGGGGACCTCACCTGCAAAGTGTGTATGCAGAGCTATGAGAGCACGCCA

GTGCTCCTGGAACACCTCAAGAGCCACTCGGGCAAGTCCTCGGGTGGTAC
CAAGGAGAAGAAGCACCCATGTGATCACTGCGACCGCCGCTTCTACACTC
GGAAGGATGTGAGGCGGCACATGGTGGTCCACACGGGCCGAAAGGACTTC
CTGTGCCAGTACTGTGCCCAGCGCTTTGGCAGGAAGGACCATCTGACGCG
CCACGTAAAGAAGAGTCACTCACAGGAGCTGCTGAAGATCAAGACAGAGC
CTCCGGACATGTTAGGTCTCCTAGGCTCCGGCTCGCCTCCTTGCTCTGTC
AAGGAGGAGCTTAGCCCCATGATGTGCAGCATGGGTCCCAACAAAGATCC
CATGATGGGAAAACCCTTCCCCAGTGGTACCCCTTTCCCCATGGGCATGT
ATAACCACCACCAC------CTCCAGGCCATGTCCAATTCGGGGGTGGGC
CATCCC------CACCCCTCCCTGATGCCCAACACCCTGTCTGCAGCAAT
GGGCATGGGCTGTCACATGGAATATCTTATCTACGCCTCCTTCTCCTTCA
TGGGATGTTTACAAATTAGTGATGGATCAAACATCGTGAATTTGCTGGCA
AGTAACTCCCCAAGTGTTTCGTATGCACTTACTCAGCAGAAGTACTTCAG
TAACTACAGTCCTGTGATTGGGTTCTACATTTACGAACCCATAGAGTACT
GGAACTCCACAGTGCAGGAGCACCTGAAGACACTGAGCCATGGCTTCAAC
AAGATCTCCTGGATGGACAACTTCTTCCACTACCTGAGGGTGGTAAATGT
GAGTGCATCGACCAAAACCGACTTCATCAACATCCTCAAGAGCTCCTTCC
TCCGCAGCCCCGAGTACCAGCACTTCACTGAGGACATAATCTTCTCCAAG
A---ACAGGGAGAGTG------ATGAATATGACATCATCGCCTCACGCAT
GTACCTTGTGGCGCGGACCACAGAGAAGAAGCGTGAGGAGGTAGTGGAGC
TTCTGGAAAAGCTGCGTCCACTGATGCTGATTAACAGCATCAAGTTCATT
GCCTTCAACCCCACCTTTGTGTTCATGGACCGGTACAGCTCCTCGGTTAT
TTCACCCATCCTAACCTCAGGCTTCAGTGTGCTTACCATCCTCATCCTCA
CTTTCTTCCTGGTCATCAATCCTCTAGGAAACTTCTGGCTCATCCTCACA
GTCACTTCTGTGGAGCTGGGCGTGTTGGGTTTGATGGGCTATCACCCATT
TGAGTGGCAGCCTGCCCTCAAGAATGTGTCTGCATCGTGCCAAGTGGGCA
TAATCAACGGGCTATCTGGGTGGGTTGCTTCTGTTGATGACTCTCCGGCT
GATACAATCACTCGCCGGTTTCGTTATGACGTGGCCCTGGTATCGGCCCT
CAAAGATCTGGAAGAGGACATTATTGAGGGACTGAGCAAGTGTGGGCTTG
AAGACAGTGCTTGCACCTCAGGCTTCAGTGTTATGATCAAGGAGTCTTGC
GATGGCATGGGTGATGTCAGCGAGAAGCACGGTGGAGGCCCTCCTGTCCC
AGAAAAAGCTGTGCGCTTTTCTTTCACCATTATGTCCATCTCTATATTGG
CTGATGGAGAGCAG------------------------------GAAGAG
GTAACTATCTTCAGGGAGCCAAAGCCAAATTCTGAACTGTCCTGTAAACC
TCTTTGCCTGATGTTTGTAGACGAGTCTGACCACGAAACCCTCACGGCTG
TCCTAGGGCCTGTGATTGCGGAACGAAACGCCATGAAGCAGAGCCAACTC
ATCCTATCTATGGGCGGGCTACCTCATTCTTTCCGCTTCCACTTTAGAGG
CACAGGCTATGATGAGAAGATGGTGCGTGAGATGGAGGGCCTCGAGGCCT
CAGGTTCCACTTATATCTGCACCCTGTGCGATTCCACTAGGGCGGAGGCC
TCTCACAACATGGTACTCCATTCTATCACACGCAGCCACAGTGAGAACCT
GGAGCGCTATGAGATATGGAGGACAAATCCCTTCTCTGAGTCTGCTGAGG
AGCTGCGAGACCGGGTCAAAGGAGTCTCTGCCAAGCCCTTTATGGAAACC
CAGCCTACTCTAGATGCATTACACTGTGACATCAGCAATGCCACAGAGTT
CTACAAAATCTTCCAGGATGAGATTGGAGAAGTGTTCCAAAGGCC---CA
AC---CCTACCCGGGAGGAAAGGCGAGGATGGAGGGCAGCCTTGGATAAG
CAGCTTAGGAGGAAGATGAAGCTTAAGCCTGTGATGAGGATGAATGGAAA
CTATGCCAGAAGGCTGATGACCAAGGAGGCTGTGGACGTGGTATGTGATT
TAGTGCCCACAGAGGAGAGGAGGGAGGCCCTGAGGGAGCTTATGGAGCTA
TACCTGCAGATGAAACCTGTATGGCGTGCTACCTGCCCCACCAATGAATG
CCCTGACCAGCTATGCCAGTACAGCTTCAACTCTCAGCGCTTTGCTGATC
TCCTTTCCACTGCCTTTAAATATAGATATAATGGAAAGATTACCAATTAC
CTGCACAAGACACTGGCCCATGTCCCTGAAATTATAGAGAGAGATGGCTC
CATCGGAGCCTGGGCCAGTGAAGGGAATGAGTCAGCAAACAAATCGTACA

CCATCGAGATGAGTCCCAAGGGGCCCCTGTGGAAGGAGAGCCCTCAGCCT
TTTTCCTGTTCGATTGAGGACCCCACAAAGCAGACCAAGTTCAAGGGCAT
CAAGACATACATATCGTACCGCGTCACGCCGAGCCACACAGGGCGACCTG
TTTATAGACGCTACAAACACTTCGACTGGCTGTACAACCGTCTGCTGCAT
AAGTTCACTGTGATCTCTGTGCCCCACCTGCCAGAGAAACAGGCTACAGG
GCGCTTTGAGGAGGACTTCATCGAGAAGCGAAAGCGTCGACTGATCCTGT
GGATGAACCATATGACCAGTCATCCAGTCCTTTCCCAGTATGAGGGCTTC
GAGCACTTCCTTATGTGTGCCGATGATAAGCAGTGGAAGCTGGGCAAGCG
TCGGGCGGAGAAGGACGAGATGGTGGGCGCACACTTCATGCTCACCTTCC
AGATCCCCAACGAGCACCAGGACCTTCAGGACGTCGAGGAGCGGGTCGAC
TCCTTCAAATCCTTCGCCAAGAAAATGGATGACAGTGTGATGCAGCTGAC
ACATGTGGCCTCGGAGCTTGTGCGGAAGCACCTGGGAGGGTTTCGGAAGG
AGTTCCAGCGGCTGGGGAATGCCTTCCAGTCCATCAGCCAGGCCTTCATG
CTGGATCCTCCCCACAGCTCAGAGGCCCTCAATAACGCCATCTCCCACCC
TCTTGCTACGTTCCTCAAACTGACCTCTCTGGGATTCATCATTGGAGTCG
GCGTGGTCGGGAACCTCCTGATCTCCATCCTGCTGGTCAAAGACAAGAGC
CTGCACCGCGCACCCTACTACTTCCTGCTGGACCTGTGTGCCTCCGACAT
CCTGCGCTCTGCCATCTGTTTCCCCTTTGTCTTCACCTCTGTCAAGAATG
GATCCACCTGGACCTATGGCACCCTTACCTGCAAAGTGATTGCCTTCCTG
GGTGTGCTCTCCTGTTTCCACACAGCATTCATGCTGTTCTGCGTCAGCGT
CACTCGCTACCTGGCCATTGCACATCACCGCTTCTATACGAAGAGGCTGA
CCTTCTGGACCTGTCTGGCCGTCATCTGCATGGTGTGGACGTTGTCAGTA
GCCATGGCCTTCCCCCCAGTGCTGGACGTAGGGACATACTACTTTATCCG
GGAGGAGGACCAGTGCACGTTCCAGCACCGCTCCTTCAGGGCCAACGACT
CGCTGGGTTTCATGCTCCTGCTGGCGCTCATCCTCCTAGCCACACAGCTG
GTTTACCTCAAGCTCATCTTCTTTGTTCATGACCGTCGGAAGATGAAGCC
CGTCCAGTTTGTGCCGGCTGTCAGCCAGAACTGGACCTTCCACGGACCAG
GCGCCAGCGGGCAGGCGGCAGCAAACTGGTTGGCTGGATTTGGGAGAGGC
CCCACCCCACCGACCTTGCTGGGCATCCGGCAGAATAGCAACGCGGCGGG
CCGCAGACGTCTGCTAGTGCTGGACGAGTTCAAAACAGAGAAGAGGATTA
GTAGAATGTTCTACATCATGACGTTTTTCTTTCTGGCTCTATGGGGGCCC
TATCTGGTTGCTTGCTACTGGCGGGTGTTTGCCAGGGGCCCCGTGGTCCC
TGCGGGCTACCTGACAGCAGCCGTGTGGATGAGCTTCGCCCAGGCTGGGG
TGAATCCCTTCATCTGCNNNNNNNNNNNNNNNNNNNNNNNNNNNNNNNNNNNNNNNNNNNNNNNNNNNN
NNNNNNNNNNNNNN---NACGCACGTCCCTCGGC-ACAGCTTGCTATC
CCCGCAGCAAACCGAGGAGCCCAGTGTTGCCACCCCCCCGCAGCGATGGT
TTGTCACCC---CTGCCAACAACCGACTGGACTTTGCTGCCTCGGCATAC
GACGCCGCT---------GATTTCGCCGGTAACGCGGCCACCTTGCTGTC
CTACGCAGCGGCCGGAGTGAAGGCTC------TTCCCCTGCCGACTGCAG
GCTGCTCCAACCGGCCTCTTGGCTATTACGCAGATCCGTCAG---GCTGG
---GGAGGACGCACGCCGCCGCAGTACTGTGGCGTAAATAGCAAATCCAG
CTCGGTGTTTTCCTGCTGGCCCGCTAACTCTATCAGCGGCAGAGCAGGGA
---CC---AACTACCTGG---------CCGAGGA---GGGA---GACTC-
--CATCCCGACGGAGAGGTCACCC---AT---C---AGCTCTGAGGAG--
-ACCAAACCAAAAGACATGAC---ATCAGA---GTCGAGCTGGATAGAG-
--ACGCCGTCCTCCATTAAGTCAATTGATTCAAGCGATTCTGGTATCTTT
G---AACAGGCCAAGAGGAGAAGGATCTCACCTTCTGCCACGCCG-----

GTTTCAGAGACAGTGTCCCCNNNNNNNNNNNNNNNNNNNNNNNNNNNNNNNNNNNNNNNNNNNNNNNNN
NNNNNNNNNNNNNNNNNNNNNNNNNNNNNNNNNNNNNNNNNNNNNNNNNNNNNNNNNNNNNNNNNNNNN
NNNNNN---NNNNNNGGCGTTTTCCTCCCAGGCG---T
CCGGCTACGCCGCCGCCGCCGCCCTGGGA---CACCACCA------CCAC
CCGACCCACGTCAGCTCC---TACTCCACGGCGGCTTTCAACTCCACGCG

GGACTTTCTCTTCAGGAATCGGGGTTTCGGCGACGCCACCAG--------
----CGCGCAGCACAGCTTGTTCGCGTCCGC---AGCCGGAAGTTTCGCG
------GCAGGGCCACATGGACACTCGGATGCCGCGGGACACCTGCTCTT
CCCCGGGCTGCACGAG---CAAGCGGCGAGCCATGCGTCCTCCAACGTGG
TCAACAGCCAGATGCGGCTGGGCTTTTCGGGAGATATGTACGGCAGGGCC
GACCAGTACGGCCACGTTACGAGCCCCAGAT---CCGACCACTACGCCTC
GACCCAGCTACACGGCTACGGCCCTATGAACATGAATATGGCCGCG---C
ACCACGGAGCAGGGGCCTTTTTCGATACATGAGGCAGCCCATCAAACAG
GAGCTCATCTGCAAGTGGATCGAGCCGGAGCAGTTGACGAACCCCAAAAA
GTCGTGCAACAAAACTTTTAGCACGATGCACGAGCTGGTGACCCATCTGA
CTGTGGAGCATGTGGGGGGACCCGAGCAGTCCAACCACATTTGCTTCTGG
GAAGACTGCTCGCGGGAGGGCAAACCGTTCAAAGCCAAATACAAACTTGT
GAATCATATCAGGGTACACACCGGAGAAAAGCCCTTCCCGTGTCCGTTCC
CCGGNNNNNNNNNN
>Lamprogrammus niger
AGTCTCTTAATTCGAGCTGAGCTGAGTCAACCGGGCGCCCTTCTCGGTGA
CGATCAAATTTATAATGTAATCGTCACAGCTCATGCTTTCGTAATAATTT
TCTTTATAGTAATACCAATTATAATTGGGGGCTTCGGCAACTGACTGATT
CCCCTAATGATTGGCGCCCCAGACATGGCGTTCCCCCGAATGAACAACAT
GAGCTTCTGGCTACTTCCCCCCTCATTCCTCCTCCTTCTAGCATCTTCAG
GAGTAGAAGCTGGCGCCGGTACCGGCTGAACAGTATACCCACCACTAGCA
GGTAACCTAGCACATGCCGGGGCCTCTGTAGACCTCACTATTTTCTCACT
ACATCTGGCGGGAGTTTCATCAATTCTCGGGGCCATCAATTTTATTACCA
CAATTATTAACATGAAACCCCCAGCCATCTCTCAATATCAAACTCCTCTC
TTCGTATGATCAGTATTAATTACCGCAGTATTACTACTCCTGTCCCTCCC
AGTCCTTGCCGCTGGTATTACAATACTTTTAACCGACCGTAACTTAAACA
CAACCTTCTTTGACCCCGCCGGCGGGGGTGACCCCATCCTCTACCAACAC
CTATTCTGATTCTTC-----------------------------------

------------TTTCTAGAGAGAAACCTTCACCCATCTAACTGCCTTGG
CATGCTCTTGCTGTCTGATGCCCATCAGTGTACCAAGCTATCAGAGCTCT
CCTGGGGCATGTGCCTCAGCAACTTTCCCGCCATTTGCAAGACAGAGGAC
TTCCTCCAGTTGCCCAAAGACATGGTGGTGCAGCTTTTGTCACATGAGGA
GCTGGAGACAGAAGATGAGAAACTGGTTTATGAAGCAGCCCTCAACTGGA
TCAACTATGACCTGGAAAGGAGGCACTGCCACCTTCCAGAGCTCCTGAGA
ACAGTCCGCCTGGCTCTGCTGCCTGCCATCTTCCTCATGGAGAATGTTTC
AACAGAAGAGCTGATCAATGCCCAGGCCAAGAGCAAGGAACTAGTGGATG
AGGCCATCCGCTGTAAGCTGAAGATCCTGCAGAACGACGGCGTAGTCAAC
AGCCCATGTGCCCGACCAAGAAAAACCAGCCATGCACTCTTTCTGTTGGG
AGGGCAGACTTTCATGTGTGACAAGCTGTACCTGGTGGACCAGAAAGCCA
AAGAGATCATCCCCAAAGCTGACATCCCCAGCCCCAGGAAGGAGTTTAGC
GCCTGTGCCATCGGCTGCAAGGTGTACATCACAGGTGGGA--GAGGTTC-
AGAGAATGGTGTGTCCAAAGATGTATGGGTGTACGATACTGTCCNNNNNNNNNNNNNNNNNNNNNNNNN
NNNNNNNNNNNNNNNNNNNNNNNNNNNNNNNNNNNNNNNNNNNNNNNNNNNNNNNNNNNNNNNNNNNNN
NNNNNNNNNNNNNNNNNNNNNNNNNNNNNNNNNNNNGGACAA
ATACATTGTTGTCTTTAGTCGTTCAACAACAAGGCTGATCTTGAATGAAG
CAGAGCTAATCATGGCACTGGCCCAGGAATTTCAGATGAGAGTTGTTACA
GTATCCCTGGAGGAACAGACTTTCCCCAGTATTGTCCAGATGCTCAGTGG
TGCTTCCATGTTGGTTAGCATGCATGGAGCCCAGCTCATAACCTCACTCT
TCCTCCCTAGAGGAGCTGCTGTGGTTGAGCTCTTCCCCTATGCTGTGAAC
CCAGAGCAGTACACGCCATATAAAACCCTTGCTTCCCTTCCAGGCATGGA

CTTGCACTATGTCTCCTGGAGGAATACCATGGAGGAGAACACAGTCACCC
ACCCAGACAGACCCTGGGAACAAGGAGGCATAGTTCATTTGGAGAAGGAG
GAACAGGAGCGAATACTGGCCAGCACAGATGTCCCCAGGCACCTGTGCTG
CCGCAATCCAGAGTGGCTCTTCAGGATCTACCAGGACACTTTGGTAGACA
TCCCTTCATTCCTGGAAGTCATCAA---AGAGGGCTTGAAG---ACCAAG
CCTAGTTTGAAGAA---GGCCAAGGCGGCCAGCACAGTTCACCCAGGCCG
GGTCAGAGAAGCCCAGTGTCAGACCTCAGTGCAAACCAGCAGTGAGGCTA
AACTCACAGTTTCCTGGCAGATCCCATGGAATCTGAAGTATCTGAAGGTG
AGAGAAGTGAAGTACGAGGTG---------AAGAAAGACACCAGCAAGGG
GACATTGGAAGATCAAATCATCCAGGCGAACCCAGCCCTGGAGGCCTTCG
GTAATGCCAAAACAGCGAGAAATGACAACTCATCCCGTTTTGGAAAATTC
ATCCGAATTCACTTTGGAAACAGCGGCAAGTTATCATCCGCTGACATTGA
GACATATTTGCTGGAGAAGTCCCGTGTCACCTTTCAGCTCAAGGCTGAGA
GGAACTACCACATCTTCTACCAGATCTTGTCCAATCAGAAGCCAGAGCTG
TTGGACATGCTGCTGATCACAAACAACCCGTATGACTACTCCTACATCTC
TCAAGGGGAGGTAACAGTTGCATCAATCAATGACGCAGAAGAGCTGATGG
CCACTGACAGTGCCTTTGACGTGCTCGGCTTCACTCAAGAGGAAAAGATG
GGTGTTTATAAGCTGATTGGTGCCATCATGCACTATGGCAACATGAAGTT
TAAGCAGAAGCAACGTGAGGAGCAGGCTGAACCTGACACGACAGAGGCTG
CTGATAAATCAGCTTACCTGATGGGCCTGAACTCTGCAGACCTCATTAAA
GGGTTGTGCCACCCCAGAGTTAAGGTAGGGAATGAGTATGTTACCAAAGG
CCAAAGTGTGGACCAAGTCTACTATCCCAACAAGGAGGCTTTCAAGTGTG
AGGAGTGTGGCAAGCACTACAACACCAAGCTGGGATATAAGCGACATGTG
GCCATGCACTCTGCCACAGCGGGGGATCTCACCTGCAAAGTGTGCATGCA
GAGCTACGAGAGCACGCCGGTGCTCCTGGAACACCTCAAGAGCCACTCTG
GGAAGTCCTCAGGTGGCACCAAGGAGAAAAAGCACCCGTGTGACCACTGT
GACCGTCGTTTCTACACGCGGAAGGATGTGAGACGGCACATGGTGGTCCA
CACGGGCAGAAAGGACTTTCTGTGCCAGTACTGTGCCCAGCGCTTTGGCA
GGAAGGACCACCTGACGCGTCACGTAAAGAAGAGCCACTCTCAGGAGCTG
CTGAAGATCAAGACGGAGCCCCCGGATATGTTAGGTCTGTTAGCTTCAGG
GTCACCACCTTGCTCTGTGAAGGAGGAGCTTAGCCCCATGATGTGCGGCA
TGGGTCCAAATAAAGACCCCATGATGGGAAAACCCTTCCCAAGTGGGGCC
CCTTTTCCGATGAGCATGTACAACCCCCACCAT------CTCCAGGCCAT
GTCCAATTCTGGGGTGGGCCACCCC------CACCCCTCCTTGATGCCTA
GTCCCCTGTCTGCAGCTATGGGTATGGGCTGTCATATGGAATATCTCATC
TATGCCTCTTTCTCATTCATGGGATGTTTACAAATCAGTGATGGATCAAA
CATTGTGAACCTGCTGGCCAGTAACTCTCCGAGTGTCTCCTACGCTCTGA
CCCAGCAGAAATACTTCAGCAACTACAGCCCCGTGATTGGGTTTTACATT
TACGAGCCCATTGAGTACTGGAATTCCACGGTGCAGGAGCATCTGAAGAC
TCTGGGTCACGGATTCAACAAAATCTCCTGGATGGACAACTTTTTCCACT
ACCTGCGTGTGGTGAATGTGAGCGCGTCCACCAAGGGCGATTTTATCTCC
ATCCTCAAGGGTTCCTTCTTGCGCAGCCCGGAGTACCAGCACTTCACTGA
GGACATCATCTTCTCTAAGA---ACCGTGAGACTG------ATGAGTATG
ACATTATTGCCTCACGAATGTATTTGGTGGCAAGGACCACCGAGAAGAAG
CGCGAGGAGGTGGTGGAGCTTTTGGAGAAGCTACGTCCGTTGATGCTGAT
CAACAGCATCAAATTCATTGCCTTCAACCCCACTTTTGTGTTCATGGACC
GCTACAGCTCCTCTGTAATCTCGCCCATCCTGACCTCAGGCTTTAGCGTT
CTCACTATCCTCATCCTCACTTTCTTCCTGGTCATCAATCCCTTAGTGTA
CTTCTGGCTCATCCTGACAGTTACATCCTGGGAGCTGGNNNNNNNNNNNNNNNN------------
----

---TCCTA
CACTATCGAGATGGGTCCCATGGGACCCCAGTGGAAGGAGAGCCCACAGC
CGTTTTCCTGTTCCATTGAAGACCCTACAAAACAGACAAAGTTCAAGGGT
ATCAAGACCTACATTTCATATCGGGTCACTCCAAGCCACACAGGGCACCC
TGTCTACAGACGCTACAAACACTTTGACTGGCTGTACAATCGCTTACTGC
ACAAGTTCACTGTGATCTCAGTACCCCACCTGCCTGAGAAGCAGGCTACT
GGGCGATTTGAGGAAGACTTCATCGAGAAGCGTAAAAGACGACTGATACT
GTGGATGAACCACATGACCAGTCACCCAGTCCTCTCCCAGTATGAAGGCT
TTGAACACTTTCTGATGTGTGCAGATGACAAACAATGGAAACTGGGTAAG
AGGCGGGCAGAAAAGGATGAAATGGTGGGTGCCCATTTCATGTTGACCCT
CCAGATTCCTAATGAACACCAGGACCTTCAGGATGTTGAGGAGCGAGTTG
ACACCTTCAAGGCCTTTGCTAAGAAAATGGATGATAGTGTCTTGCAGCTC
ACACATGTTGCCTCAGAGCTGGTGCGTAAGCACATTGGTGGATTCAGGAA
GGAGTTCCAGCGGCTTGGAAATGCATTCCAGTCCATCAGCCAGGCCTTCA
TGCTGGACCCTCCCCACAGCTCAGANNNNNNNNNNNNNNNNNNNNNNNNNNNNNNNNNNNCGTTTCTCAA
ACTGACCTCCCTGGG
CTTCATCATCGGCGTCGGCGTGGTCGGAAACCTCCTGATCTCCATCCTGC
TGGTTAAAGACAAGAGCCTGCACCGCGCGCCCTACTACTTCTTGCTGGAC
CTGTGCGCCTCTGACATCCTGCGTTCGGCTATCTGCTTCCCCTTTGTCTT
CACCTCTGTCAAGAATGGATCTGCCTGGACCTACGGCACGCTCACCTGCA
AAGTGATCGCCTTCCTGGGCGTGCTCTCCTGTTTCCACACAGCGTTCATG
CTGTTTTGTGTCAGCGTGACCCGCTATCTGGCCATCGCACATCACCGTTT
CTACACTAAGAGGCTGACCTTCTGGACCTGCCTGGCTGTCATCTGCATGG
TGTGGACGTTGTCGGTGGCCATGGCTTTCCCACCGGTGCTAGACGTGGGG
ACATACTCTTTCATCCGGGAAGAGGACCAGTGCACATTCCAGCACCGTTC
CTTCAGGGCGAATGACTCCCTGGGCTTCATGCTCCTGCTGGCACTCATCC
TCCTGGCCACACAGCTGGTTTACCTCAAGCTCATCTTCTTCGTCCATGAC
CGCCGGAAGATGAAGCCTGTCCAGTTCGTGCCTGCTGTCAGCCAGAACTG
GACCTTCCACGGGCCAGGTGCCAGCGGGCAGGCGGCAGCCAACTGGCTGG
CTGGATTTGGTAGAGGTCCCACCCCTCCTACTTTATTGGGCATTCGTCAG

AACAGCAACGCAGCAGGCCGCAGGCGTCTACTGGTTCTGGATGAATTCAA
AACAGAGAAGAGGATTAGTAGGATGTTCTACATCATGACGTTTTTCTTCC
TGTCTCTCTGGGGGCCGTATCTGGTTGCCTGCTACTGGCGGGTGTTTGCA
AGGGGCCCTGTAGTCCCCGGAGGCTACCTGACAGCAGCCGTGTGGATGAG
CTTTGCCCAGGCTGGGGTCAATCCTTTCATCTGCATCTTCTCCAACAGGG
AG--

----------------------NNNNNNNNNNNAGGCGAAGTCACGGAGAGAGAAGTGGCG
TTGGGGATAAATCCTTTCGCGGACGGATGGGCGCCTTCAAGATCAACCA
CAGCTCCCACGACATTGGCTCCGG---GCAGACGGCGTTTTCCTCCCAGG
CG---CCCGGCTAC---GCAGCGGCCGCCCTGGGA---CACCACCA----
--CCACCCGACCCACGTTGGCTCT---TACTCCACGGCGGCTTTCAACTC
CACCAGGGACTTTCTGTTCAGAAATCGGGGTTTCGGGGACGCGACCGG--
----------CGCGCAGCACAGCTTGTTCGCCTC---------CGGAAGT
TT---C------GCAGGGCCACATGGACACTCGGATGCGGCGGGGCACCT
GCTCTTCCCGGGGCTCCACGAG---CAAGCGGCGAGCCATGCGTCCTCCA
ACGTGGTCAACGGCCAGATGCGACTCGGCTTCTCGGGGGACATGTACGGC
CGGGCCGACCAGTACGGCCACGTTACGAGCCCCAGGT---CCGACCACTA
CGCGTCGACCCAGTTGCACGGCTACGGCCCCATGAACATGAATATGGCCG
CG---CACCACGGAGCGGGGGCCTTCTTTCGATACATGCGGCAGCCGATC
AAGCAAGAGCTCATCTGCAAGTGGATCGAACCGGAGCAGCTGACGAACCC
CAAAAAGTCGTGCAACAAAACTTTCAGCACGATGCATGAGCTTGTGACCC
ATCTGACGGTGGAGCATGTGGGGGGACCGGAGCAGACCAACCACATCTGC
TTCTGGGAGGACTGCTCCAGGGAAGGGAAGCCGTTCAAAGCCAAATACAA
ACTTGTGAATCACATCAGGGTGCACACCGGNNNNNNNNNNNNNNNNNNNNNNNNNNNNNNNNNNNNNNNN
>Larimichthys polyactis
AGTCTCATTATTCGAGCAGAGCTAAGCCAGCCCGGCTCGCTTCTCGGAGA
CGACCAGATTTTTAACGTAGTTGTTACGGCACATGCCTTCGTTATAATCT
TCTTTATAGTAATACCCGTAATAATCGGAGGGTTCGGAAACTGACTCGTG
CCTTTAATAATTGGCGCCCCCGACATAGCATTTCCCCGAATAAATAACAT
AAGCTTCTGACTTATCCCCCCTGCTTTCATTATGCTCGCAGCCTCATCAG
CGGTTGAAGCAGGGGCCGGAACAGGGTGAACAGTCTACCCCCCACTTGCT
GGAAATCTCGCACACGCAGGAGCTTCAGTCGACTTAGCCATTTTCGCTCT
GCACCTTGCGGGTGTCTCTTCAATCCTGGGGGCCATCAACTTCATCACAA
CAATTCTTAACATAAAACCCCCTGGCATAACCCAATACCAAACACCCCTG
TTTGTGTGATCCGTTCTGATTACAGCAGTCCTCCTCCTACTATCACTGCC
CGTCCTAGCTGCCGGCATCACAATGCTTTTAACAGACCGCAACCTCAACA
CAACCTTTTTTGACCCCTCAGGTGGAGGCGATCCCATCCTTTATCAACAC
CTATTCTGATTTTTGGTCACC-----------------------------


```
--------------------------------------------------
--------------------------------------------------
--------------------------------------------------
--------------------------------------------------
--------------------------------------------------
--------------------------------------------------
--------------------------------------------------
--------------------------------------------------
--------------------------------------------------
--------------------------------------------------
--------------------------------------------------
--------------------------------------------------
--------------------------------------------------
--------------------------------------------------
--------------------------------------------------
--------------------------------------------------
--------------------------------------------------
--------------------------------------------------
--------------------------------------------------
--------------------------------------------------
--------------------------------------------------
--------------------------------------------------
--------------------------------------------------
--------------------------------------------------
--------------------------------------------------
--------------------------------------------------
--------------------------------------------------
--------------------------------------------------
--------------------------------------------------
--------------------------------------------------
--------------------------------------------------
--------------------------------------------------
--------------------------------------------------
--------------------------------------------------
--------------------------------------------------
--------------------------------------------------
--------------------------------------------------
--------------------------------------------------
--------------------------------------------------
--------------------------------------------------
--------------------------------------------------
--------------------------------------------------
--------------------------------------------------
--------------------------------------------------
--------------------------------------------------
--------------------------------------------------
--------------------------------------------------
--------------------------------------------------
--------------------------------------------------
--------------------------------------------------
--------------------------------------------------
--------------------------------------------------
--------------------------------------------------
--------------------------------------------------
--------------------------------------------------
--------------------------------------------------
```

```
--------------------------------------------------------
--------------------------------------------------------
--------------------------------------------------------
--------------------------------------------------------
--------------------------------------------------------
--------------------------------------------------------
--------------------------------------------------------
--------------------------------------------------------
--------------------------------------------------------
--------------------------------------------------------
--------------------------------------------------------
--------------------------------------------------------
--------------------------------------------------------
--------------------------------------------------------
--------------------------------------------------------
--------------------------------------------------------
--------------------------------------------------------
--------------------------------------------------------
--------------------------------------------------------
--------------------------------------------------------
--------------------------------------------------------
--------------------------------------------------------
--------------------------------------------------------
--------------------------------------------------------
--------------------------------------------------------
--------------------------------------------------------
--------------------------------------------------------
--------------------------------------------------------
--------------------------------------------------------
--------------------------------------------------------
--------------------------------------------------------
--------------------------------------------------------
--------------------------------------------------------
--------------------------------------------------------
--------------------------------------------------------
--------------------------------------------------------
--------------------------------------------------------
--------------------------------------------------------
--------------------------------------------------------
--------------------------------------------------------
--------------------------------------------------------
--------------------------------------------------------
--------------------------------------------------------
--------------------------------------------------------
--------------------------------------------------------
--------------------------------------------------------
--------------------------------------------------------
--------------------------------------------------------
--------------------------------------------------------
--------------------------------------------------------
--------------------------------------------------------
--------------------------------------------------------
--------------------------------------------------------
--------------------------------------------------------
--------------------------------------------------------
--------------------------------------------------------
```

```
---------------------------------------------------
---------------------------------------------------
---------------------------------------------------
---------------------------------------------------
---------------------------------------------------
---------------------------------------------------
---------------------------------------------------
---------------------------------------------------
---------------------------------------------------
---------------------------------------------------
---------------------------------------------------
---------------------------------------------------
---------------------------------------------------
---------------------------------------------------
---------------------------------------------------
---------------------------------------------------
---------------------------------------------------
---------------------------------------------------
---------------------------------------------------
---------------------------------------------------
---------------------------------------------------
---------------------------------------------------
---------------------------------------------------
---------------------------------------------------
---------------------------------------------------
---------------------------------------------------
---------------------------------------------------
---------------------------------------------------
---------------------------------------------------
---------------------------------------------------
---------------------------------------------------
---------------------------------------------------
---------------------------------------------------
---------------------------------------------------
---------------------------------------------------
---------------------------------------------------
---------------------------------------------------
---------------------------------------------------
---------------------------------------------------
---------------------------------------------------
---------------------------------------------------
---------------------------------------------------
---------------------------------------------------
---------------------------------------------------
---------------------------------------------------
---------------------------------------------------
---------------------------------------------------
---------------------------------------------------
---------------------------------------------------
---------------------------------------------------
---------------------------------------------------
---------------------------------------------------
---------------------------------------------------
---------------------------------------------------
---------------------------------------------------
---------------------------------------------------
```


>Lates niloticus
AGCCTACTCATCCGAGCTGAACTTAGCCAACCTGGCGCACTCCTAGGAGA
CGACCAGATCTATAACGTAATCGTTACCGCCCACGCTTTCGTAATAATTT
TCTTTATAGTAATACCAATTATGATCGGAGGCTTTGGAAACTGACTTATC
CCACTAATAATTGGGGCCCCAGACATAGCATTCCCTCGAATAAATAACAT
GAGTTTCTGACTCCTTCCACCATCTTTCCTGCTCCTCCTAGCTTCTTCTG
GAGTAGAAGCCGGAGCTGGAACCGGTTGAACTGTGTATCCACCCCTCGCT
GGGAACCTTGCCCATGCTGGAGCATCCGTCGACCTGACCATCTTCTCACT
TCACTTAGCAGGAATTTCCTCAATTCTCGGGGCCATCAACTTTATTACTA
CCATCTTCAATATGAAGCCGGCTGCCGTATCTATGTACCAAATTCCTCTA
TTCGTCTGAGCAGTGTTAATCACAGCTGTCCTACTCCTTCTTTCCCTCCC
AGTCTTAGCTGCGGGTATCACAATACTTCTCACAGACCGAAATCTAAATA
CAGCATTCTTTGACCCCGCCGGAGGAGGAGACCCCATTCTCTATCAACAC
CTA---

-------------------------GGACGATTACATTGTTGTGTTCAGT
CGTTCAACAACGAGGCTTATACTTAATGAAGCAGAGCTAATCATGGCATT
GGCCCAGGAGTTCCACATGAGAGTGGTCACAGTATCCCTGGAGGAACAGT
CTTTTCCCAGTATCATCCAGGTGATCAGCGGTGCTTCCATATTAGTCAGC
ATGCATGGAGCTCAGCTTATCACCTCACTCTTCCTCCCCAGAGGAGCTGT
TGTGGTGGAACTGTTTCCCTTTGCTGTGAATCCGGAGCAGTACACTCCTT
ATAAAACCCTTGCCTCCCTCCCAGGCATGGACCTTCACTATATCTCCTGG
AGAAATACTAAGGAGGAAAACACCATCACCCACCCAGACAGACCCTGGGA
ACAAGGAGGTATTGCTCACTTGGAGAAGGAGGAGCGAGAGAGAATACTGG
CCAGTAAGGAGGTCCCCAGGCACCTGTGCTGCCGCAACCCAGAGTGGCTC
TTCCGGATCTATCAGGACACCTTAGTTGACATCCCCTCCTTCCTAGAAAT

CCTCAA---AGAGGGCATGAAG---ACCAAGCCCAGCTTGAAGAA---GT
CAAAGCCTGCCAGCACAGTTCACCCAGGCCGAGTCAGAGAACCCCAGTGT
CAGACCTCAGTACAAACCACTAATGAGGCAAAACTCACAGTCTCCTGGCA
GATCCCATGGAATCTGAAATACTTGAAGGTAAGAGAAGTCAAATACGAAG
TGTGGATCCAGAAAAAAGATGCCAGCAAGGGGACACTGGAGGATCAAATC
ATCCAGGCAAACCCTGCACTGGAGGCCTTCGGCAACGCCAAAACGTTGAG
AAACGACAATTCGTCACGTTTTGGAAAATTTATTCGCATTCACTTTGGTA
CGAGTGGCAAACTGTCATCTGCTGACATCGAGACGTACCTGCTGGAGAAG
TCACGCGTCACTTTTCAGCTCAAGGCTGAGAGGAACTACCACATCTTCTA
CCAGATCCTGTCCAATCAGAAGCCAGAGCTGCTGGACATGCTGCTGATCA
CCAACAACCCGTACGACTACTCCTACATCTCCCAAGGAGAGGTAACAGTT
GCTTCCATCAATGACTCGGAGGAGCTGATGGCCACTGACAGCGCCTTCGA
TGTGCTTGGCTTCACTCCAGAAGAGAAGATGGCGGTCTATAAACTGACCG
GTGCCATCATGCACTACGGCAACATGAAGTTCAAACAGAAGCAGCGTGAG
GAGCAGGCTGAACCTGACGGGACGGAGGCTGCTGATAAATCAGCTTACCT
AATGGGGCTGAACTCCGCTGACCTCATCAAAGGCCTTTGCCACCCCAGAG
TCAAAGTGGGAAATGAATACGTCACCAAAGGCCAAAGTGTGGACCAAGTC
TACTANNNNNNNNNNNNNNNNNNNNNNNNNNNNNNNNNNNGTAAGCACTACAACACCAAGCTGGGATACA
AGCGTCATGTGGCCA
TGCACTCTGCCACAGCAGGGGATCTCACCTGTAAAGTGTGCATGCAGAGC
TACGAGAGTACACCTGTTCTTCTGGAGCACCTCAAAAGCCACTCTGGGAA
GTCTTCCGGTGGTGCCAAGGAGAAGAAACACCCGTGTGACCACTGTGACC
GTCGTTTCTACACACGGAAGGATGTCAGACGGCACATGGTGGTCCACACA
GGCCGAAAGGACTTCCTGTGCCAGTACTGTGCCCAGCGCTTCGGCAGGAA
GGACCACCTGACACGCCACGTGAAGAAGAGCCACTCGCAGGAGCTGCTGA
AGATCAAGACGGAGCCTCCGGATATGTTAGGCCTTTTAGCTTCTGGGTCA
CCACCTTGCTCTGTGAAGGAAGAGCTTAGCCCCATGATGTGCGGCATGGG
TCCCAACAAAGACCCCATGATGGGCAAAACGTTCCCCAGTGGGGCCCCTT
TTCCAATGGGCATGTACAACCCCCACCAT------CTTCAGGCAATGTCT
AATACTGGGGTGGGTCACCCA------CACCCATCCCTAATGCCCAGTTC
CTTATCTGCAGCTATGGGCATGGGCTGCCANNNNNNNNNNNNNNNNNNNNNNNNNNNNNNNNNNNNNNNN
NNNNNNNNNNNNNNNNNNNNNNNNNNNNNCGTGAATCTGCTGGCTAGCA
ACTCTCCAAGTGTTTCATATGCTCTGACCCAGCAGAAATATTTCAGTAAC
TACAGTCCTGTGATCGGGTTTTACATTTATGAGCCCATTGAATACTGGAA
CTCTACAGTTCAGGAGCACCTGAAGACCCTGAGTCATGGCTTCAACAAGA
TCTCCTGGATGGACAACTTCTTCCACTATCTGCGAGTGGTGAATGTGAGT
GCATCAACCAAGAGTGACTTCATCACCATCCTTAAAGGCTCCTTCCTGCG
CAGCCCAGAGTACCAGCACTTCACTGAGGACATCATTTTCTCCAAGA---
ACAGTGATACTG------ACGAGTATGATATTATCGCCTCACGGATGTAC
TTGGTGGCCCGGACGACGGAGAAGAAGCGAGAGGAGGTGGTGGAGCTTCT
GGAAAAGCTTCGTCCATTGATGCTAATCAACAGCATTAAGTTCATTGCCT
TCAATCCCACATTTGTGTTCATGGACCGCTACAGCTCCTCTGTCATCTCG
CCCATCCTGACCTCAGGCTTCAGTGTACTCACTATCCTCATCCTCACTTT
CTTCCTGGTCATCAACCCATTGGAAAACTTCTGNNNNNNNNNNNNNNNNNNNNNNNNNNNNNNNNNNNNN
NNNNNNNNNNNNGGTTTTCACCAGTTTGA
GTGGCAGCCAGCTCTCAAGAATGTTTCCACGTCTTGCAACGTTGGCATTA
TTAATGGGCTCTCTGGATGGGCTGCCTCGCTGGATGACTCCCCAGCCGAC
ACCATCACTCGGCGGTTTCGCTATGATGTGGCACTGGTGTCAGCATTAAA
AGATCTGGAGGAGGACATCATGGAGGGGCTGAGAGAGAGTGGGATGGAAG
ACAGTGCTTGCACCTCAGGCTTTAGTGTCATGATCAAGGAATGTTGTGAC
GGCATGGGTGATGTCAGTGAAAAGCACGGTGGAGGACCAGCCGTTCCTGA
GAAGGCTGTACGCTTCTCTTTCACCGTTATGTCTGTCTCTGTCCTAGCAG
ACGATGACAGG------------------------------CAGGAGGTT

ACCATCTTCACCGAGCTGAAGCCAAACTCAGAGCTGTCCTGTAAGCCCCT
TTGCCTGATGTTTGTGGATGAGTCAGACCATGAGACACTCACAGCCGTTC
TGGGGCCTATAATAGCAGAGCGTAACGCAATGAAAGAGAGCAGGCTCATC
CTACCCATAGGAGGCCTGCCTCGCTCCTTCCGCTTCCACTTCAGAGGCAC
GGGATACGACGAGAAGATGGTGAGAGAGATGGAGGGCCTGGAGGCCTCGG
GGTCCACATATATCTGTACTCTGTGTGACTCCAGTCGGGCGGAGGCCTCT
CACAACATGGTGCTGCACTCCATCACCCGCTCTCACGAAGAGAACCTGGA
GCGTTACGAAATATGGAGAACCAACCCCTTCTCTGAGTCTGTGGAGGAGC
TGCGAGACAGAGTCAAAGGGGTCTCTGCCAAGCCCTTCATGGAGACCCAG
CCCACACTCGATGCATTACACTGTGACATTGGCAATGCCACCGAGTTCTA
CAAAATTTTCCAGGATGAGATCGGGGAGGTGTACCAAAAGGT---CAAC-
--CCCAGCCGGGAGGAGCGGCGCAGCTGGAGGGCAGCCCTAGATAAACAG
CTGAGGAAGAAGATGAAGCTTAAACCCATAATGAGGATGAATGGGAACTA
CGCCCGCAGGCTAATGACCCTGGAGGCTGTGGAGGTGGTGTGCGAGCTGG
TGCCCTCGGAGGAGAGGAGGGAGGCCCTGAGGGAGCTCATGAGGCTCTAC
CTCCAGATGAGGCCTGTGTGGCGTGCCACCTGCCCAGCCAAGGAATGCCC
CGACCAGCTGTGCCGCTACAGCTTCAACTCCCAGCGCTTTGCCGACCTCC
TGTCCTCTACCTTCAAATATAGGTACAACGGAAAGATAACCAATTACCTT
CACAAGACCCTGGCCCATGTGCCCGAAATCATAGAGAGGGATGGATCCAT
CGGAGCCTGGGCCAGCGAGGNNNNNNNNNNNNNNNNNNNNTCATACACCATCGAGATGGGCCCCCTTGGG
CCCCGGTGGAAGGAGAACCCACAGCCTTTCTCCTGCTCCATTGAAGACCC
CACAAAACAGACAAAGTTCAAGGGCATCAAGACCTACATTTCGTACCGGG
TCACGCCGAGCCACACAGGGCGTCCCGTCTACAGACGTTACAAACACTTT
GACTGGCTGTACAACCGCTTACTGCACAAGTTCACTGTGATCTCCGTGCC
TCACCTGCCTGAGAAGCAGGCTACGGGGCGATTTGAGGAAGACTTCATCG
AGAAGCGCAAGAGACGACTGATACTGTGGATGAACCACATGACCAGTCAC
CCAGTCCTCTCCCAGTATGAAGGCTTTGAGCACTTCCTGATGTGTGCTGA
CGACAAGCAGTGGAAACTGGGAAAGAGACGGGCGGAGAAGGACGAGATGG
TGGGTGCTCATTTCATGCTGACCCTCCAGATCCCTAACGAGCACCAGGAC
CTTCAGGATGTAGAGGAGCGGGTCGACTCCTTCAAGGCCTTTGCTAAAAA
AATGGACGACAGCGTGATGCAGCTCACACATGTTACTTCAGAGCTGGTGC
GTAAACACCTGGGTGGGTTCAGGAAGGAGTTCCAGCGGCTGGGAAATGCC
TTCCAGTCTATCAGCCAGGCATTCATGCTGGACCCTCCCCACAGCTCAGA
GGCCGTCAACANNNNNNNNNNNNNN---------------------------------------
----

NNNNNNNNNNNNNNNNNNNNNNNNNNNNNNNNNNNNNNNNNNNNNNNNN--
-NNAGCGTCCCACTCGGCAACAGCTTGCTATCCCCGCAGCAAACCGAGGAGC
CCACTGTTGCCACCCCCCCGCAGCGATGGTTTGTCACCC---CTGCCAAC
AACCGACTGGACTTTGCTGCCTCGGCATACGACGCTGCT---------GA
TTTCGCCGGTAACGCGGCCACCTTGCTGTCCTACGCAGCGGCCGGAGTGA
AGGCTC------TTCCCCTGCCGACTGCAGGCTGCTCCAACCGGCCTCTT
GGCTATTACGCAGACCCGTCAG---GCTGG---GGAGGACGCACGCCGCC
GCAGTACTGTGGTGTAAATAGCAAACCCAGCTCGGTCTTTTCCTGCTGGC
CCGCTAACTCTATCGGGGGCAGAGCGGGCA---CC---AACTACCTGG--
-------CCGAGGA---GGGC---GACAC---CATTCCGACGGAGAGATC
ACCG---AT---CGGTGGCTCGGAGGAG---ACCAAACCCAAAGACATGG
C---ATCAGA---GTCGAGCTGGATAGAG---ACGCCGTCCTCCATTAAG
TCAATAGATTCGAGCGATTCTGGGATCTTTG---AACAGGCCAAAAGGAG
AAGAATCTCACCGTCTGCCACGCCG---------GTTTCAGAGACTGTGT
CCCNNNNNNNNNNNNNN---

>Leiognathus equulus
AGTCTTCTTATTCGGGCAGAGCTAAGCCAGCCAGGTGCTCTGCTCGGAGA
CGACCACATTTATAATGTTATTGTCACCGCACATGCATTCGTAATAATTT
TTTTTATGGTCATACCTATTATGATTGGAGGTTTCGGTAACTGGCTAATC
CCACTAATAATTGGAGCCCCTGACATAGCATTCCCACGAATGAACAACAT
AAGTTTCTGACTTCTACCTCCCTCATTCTTACTTCTTTTGGCATCCTCAG
GCATTGAAGCTGGAGCAGGAACTGGGTGAACAGTCTACCCCCCTCTTGCA
GGCAACCTTGCCCACGCAGGCGCTTCCGTAGACCTGACTATCTTCTCCCT
CCACTTAGCCGGAATCTCGTCAATCTTAGGGGCCATTAACTTCATTACAA
CAATTATTAATATAAAACCCCCAGCCATCTCACAATTCCAAACGCCCCTG
TTTGTCTGAGCAGTGCTAATCACAGCAGTTCTCCTTCTCCTTTCTCTCCC
AGTTCTTGCAGCGGGTATCACAATGCTTCTAACTGACCGTAACCTTAATA
CTACGTTCTTTGACCCTGCAGGAGGTGGAGACCCAATTTTATACC-----

------------TTCCTTGAGAGAAACCTTCACCCGTCCAACTGCCTCGG
CATGCTGCTGCTGTCTGATGCCCACCAGTGCACCAAGTTGTCCGAGCTCT
CCTGGGGCATGTGCCTCAGCAACTTTCCTGCTATCTGCAAGACGGAGGAC

TTCCTCCAACTGCCCAAAGATATGGTGGTTCAGCTTTTGTCACATGAGGA
GTTAGAGACAGAAGATGAGAGACTGGTTTACGAAGCTGCTCTCAACTGGA
TCAACTACGACCTGGAAAAGAGGCACTGCCACCTTCCAGAGCTCCTGAGA
ACAGTGCGTTTGGCCCTGCTGCCTGCCATCTTTCTCATGGAGAATGTGTC
CACAGAAGAGCTCATCAACACCCAGGCCAAGAGCAAGGAGCTGGTAGATG
AAGCCATCCGCTGCAAGCTGAAGATCCTGCAGAATGACGGCGTGGTCAAC
AGCCCGTGTGCCAGACCGAGAAAAACTAGCCATGCCCTCTTTCTTCTGGG
AGGTCAGACTTTCATGTGCGACAAGTTGTACCTGGTGGACCAGAAAGCCA
AAGAGATCATTCCCAAGGCTGATATTCCCAGCCCTAGGAAGGAGTTCAGC
GCCTGCGCAATTGGTTGCAAGGTATACATTACTGGTGGGA--GAGGGTC-
TGAGAATGGCGTGTCCAAAGATGTGTGGGTCTATGACACCGTCCATGAAG
AATGGTCAAAGGCGGCACCCATGCTCATTGCTCGGTTTGGCCATGGATCC
GCAGAGCTGAAACACTGCCTGTACGTCGTGAGAGGTCACACAGCTGCAAC
CGGTTGCCTTCCGGCATCTCCATCCGGAAGAGTACATTGTTGTGTTCAGT
CGCTCAACAACGAGACTGATATTGAATGAAGCAGAGCTAATCATGGCACT
GGCCCAGGAATTCCAGATGAGAGTGGTCACAGTATCCCTGGAGGACCAGT
CTTTCCCCAGTATTGTCCAGGTGATCAGTGGTGCTACTATGCTGGTCAGT
ATGCACGGAGCTCAACTTATCACCTCGCTCTTCCTTCCCAGAGGAGCTGC
TGTGGTGGAGCTGTTCCCCTTTGCTGTGAACCCAGAGCAGTACACCCCGT
ACAAAACCCTTGCCTCCCTACCAGGTATGGACCTTCATTATGTCTCCTGG
AGGAATACCAAGGAGGAGAACACTATTACCCATCCAGACAGACCCTGGGA
GCAAGGGGGCATTGTGCACCTGGAGAAGGAGGAGCAGGAGAGAATCGTGA
GGAGCAAAGACGTCCCCAGGCATCTGTGCTGCCGCAACCCAGAGTGGCTC
TTTCGGATCTATCAGGACACTTTGGTGGACATCCCTTCCTTCCTGGAGGT
CCTGAA---GGAGGGCATGAAA---ACAAAGCCCATCTTAAAGAA---AT
CAAAGCCAGTCACCACAGTCCACCCAGGCCGGGTTAGAGAACCCCAGTGT
CAGACCTCTGTACAAGCTGCTAATGAGGCCAAACTCACAGTCTCCTGGCA
GATCCCATGGAATCTGAAGTACCTGAAGGTCAGGGAGGTGAAGTATGAGG
TGTGGATCCAGAAAAAAGACACCAGCAAAGGGACCTTGGAGGACCAGATC
ATCCAGGCCAATCCTGCTCTGGAGGCCTTTGGCAACGCCAAGACACTGAG
AAATGACAACTCATCACGTTTTGGAAAATTCATCCGGATTCACTTTGGTA
CCAGTGGCAAACTTTCATCTGCTGACATTGAGACGTACCTGCTGGAGAAG
TCACGAGTCACCTTTCAGCTCAAGGCTGAGAGGAACTACCACATCTTCTA
CCAGATCCTGTCCAATCAGAAGCCAGAGCTGCTGGACATGCTGCTGATCA
CCAACAACCCCTACGACTACTCCTACATCTCCCAAGGAGAAGTAACGGTC
GCCTCCATTAACGACTCGGAGGAGCTGATGGCCACAGACAGTGCCTTCGA
TGTGCTCGGCTTCACTGCAGAGGAGAAGATGGGCGTCTACAAGCTGACGG
GTGCCATCATGCACTATGGCAACATGAAGTTCAAGCAGAAGCAGCGTGAG
GAGCAGGCTGAGCCAGACGGCACTGAGGCTGCTGATAAATCCGCGTACCT
AATGGGGCTGAACTCTGCCGACCTCATCAAAGGGCTGTGCCACCCCCGAG
TCAAGGTAGGAAATGAATACGTCACCAAAGGTCAGAGTGTGGACCAAGTC
TACTACCCCAACAAGGAGGCCTTCAAGTGCGAGGAGTGTGGCAAGCACTA
CAACACCAAGCTGGGATACAAGCGTCATGTGGCCATGCACTCTGCCACGG
CCGGCGACCTCACCTGCAAGGTGTGCATGCAGACCTACGAGAGCACGCCC
GTGCTCCTGGAGCACCTCAAGAGCCACTCCGGGAAGTCCTCGGGCGGCAC
CAAGGAGAAGAAACACCCGTGCGACCACTGCGACCGTCGCTTCTACACGC
GGAAGGATGTGAGACGGCACATGGTGGTCCACACGGGCCGAAAGGACTTC
CTGTGCCAGTACTGTGCCCAGCGCTTCGGCAGAAAGGACCATCTGACGCG
GCACGTGAAGAAGAGCCACTCGCAGGAGCTGCTGAAGATCAAGACGGAGC
CTCCCGACATGCTGGGCCTGCTGGCGTCCGGGTCGCCGCCGTGCTCCGTG
AAGGAGGAGCTCAGCCCCATGATGTGCGGCATGACTGCCAACAAAGACCC
CATGATGGGCAAACCCTTCCCTGGGGGGGCCCCGTTTCAGATGGGCATGT
ACAATCCCCACCAC------CTCCAGGCCATGTCGAATTCCGGGGTGGGT

CACCCG------CACCCGTCCCTGATGCCCGGCCCCCTGTCTGCAGCCAT
GGGCATGGGCTGCCACATGGAANNNNNNNNNNNNNNNNNNNNNNNNNNATGGGATGTTTACAAATTAGT
GATGGAT
CAAACATTGTGAACCTGCTGGCTAGTAACTCTCCGAGTGTTTCATATGCT
CTGACCCAGCAAAAATACTTTAGTAACTACAGTCCTGTGATTGGCTTTTA
TATTTATGAGCCCATTGAGTACTGGAACTCCACAGTGCAGGAGCACCTGA
AGACCTTGAGTCACGGCTTCAACAAGATCTCCTGGATGGACAACTTTTTC
CATTACCTGCGGGTGGTGAATGTGAGTGCATCAACCAAAAGCGACTTCAT
CACCATCCTCAAGGGCTCGTTCCTGCGCAGCCCAGAGTACCAGCACTTCA
CTGAGGACATCATATTTTCTAAGA---ACCGCGAGACAG------ACGAG
TACGAAATCATCGCCTCTCGGATGTACCTGGTGGCACGGACGACAGAGAA
GAAGCGTGAAGAGGTGGTGGAGCTCCTGGAGAAGCTTCGCCCGTTGATGC
TGATCAACAGCATCAAATTCATTGCCTTTAATCCCACCTTTGTGTTTATG
GATCGCTACAGCTCATCCGTCATCTCGCCCATCCTGACCTCAGGATTCAG
TGTGCTCACAATCCTCATCCTCACTTTCTTCCTGGTCATCAACCCCTTGG
CAAACTTCTGGNNNNNNNNNNNNNNNNNNNNNNNNNNNNNNNNNNNNNNNNNNNNNNNNNNNNNNNN
NNNNNNNNNNCAGCCAGCTCTCAAGAATGTATCTACATCTTGCAATGTT
GGCATTATTAACGGGCTCTCTGGATGGACCTCCTCAGTGGATGATGCCCC
AGCTGACACCATAACGCGACGTTTTCGCTACGATGTGGCTCTTGTGTCAG
CACTAAAGGACCTGGAGGAGGACATCATGGAAGGGCTGAGGGAGAATGAG
ATGGAGGACAGTGCTTGCACTTCAGGCTTCACTGTCATGATCAAGGAGTC
CTGCGATGGCATGGGTGACGTCAGTGAGAAGCACGGAGGAGGACCAGCTG
TCCCAGAGAAGGCTGTGCGTTTCTCTTTCACTATCATGTCTATTTCAGTG
ACGACTGAGG---AGGAG------------------------------AA
AGAAGTCAGCATCTTCACAGAGATGAAGCCAAACTCAGAGCTGTCCTGTA
AGCCCCTCAGCCTCACATTTGTGGATGAGTCAGACCATGAGACACTGACA
GCCATACTGTGGCCTATTGTTGCAGAGCGTAATGCAATGAAAGAGAGCAG
GCTCATCCTGTCCATTGGTGGACTGCCTCGCTCCTTCCGCTTTCATTTCA
GAGGCACGGGATATGATGAGAAGATGGTACGTGAGATGGAGGGCCTCGAA
GCCTCAGGGTCCACTTACGTCTGCACTCTTTGTGACTCAGGTCGAGCGGA
GGCCTCTGAAAACATGGTGCTTCACACCATAACTCGGAATCACGAAGAGA
ACCTAGAGCGATATGAGATATGGAGAAGCAACCCCTTTTCTGAGTCAGCA
GATGAGCTTCGAGACAGAGTCAAAGGGGTCTCCGCCAAACCCTTCATGGA
GACCCATCCCACGATGGATGCATTACACTGCGACATAGGCAATGCCACAG
AGTTCTACAAAATCTTTCAGGATGAGATCGGGGAGGTGTTTGAAAAGCC-
--CAAC---CCCAGCCGAGAGGAGCGCCGCAGCTGGAGGGCAGCCCTGGA
TAAACAGCTGAGGATGAAGCTGAAGCTCAAACCGGTGATGAGGATGAATG
GGAACTATGCACGCCGGCTAATGACGACAGAGGCTGTGGAAGCAGTGTGT
GAGCTGGTGCCCTCAGAGGCAAGGAGGGAGGCCCTGAGGGAGCTGATGGA
GCTCTACCTCCAGATGAAGCCCGTGTGGCGTTCCAGCTGCCCAGCCAGGG
AGTGCCCCGACCAGCTGTGCCGTTACAGCTTCAACTCCCAGCGCTTTGCT
GACCTCCTGTCTTCCACCTTCAAATATAGGTACAAAGGAAAGATACCCAA
TTACCTGCACAAGACCCTGGCCCATGTGCCTGAAATCATAGAGAGAGATG
GACCCATAGGAGCTTGGGCCAGTGANNNNNNNNNNNNNNNNNNNNNTCGTACACCATCGAGATGGGTCC
CT
TGGGTCCCAGGTGGAAAGATAGCCCTCAGCCTTTCTCCTGCTCCATTGAA
GACCCAACGAAACAGACAAAGTTCAAAGGCATCAAGACGTACATTTCGTA
CCGGGTGACGCCGAGCCACACGGGGCACCCAGTCTACAGACGCTACAAAC
ACTTTGACTGGCTGTACAACCGCCTGCTGCACAAGTTCACTGTGATCTCC
GTGCCTCACCTGCCTGAGAAGCAGGCCACGGGGCGATTCGAGGAAGACTT
CATCGAGAAGCGCAAGAGGCGACTGATACTGTGGATGAACCACATGACCA
GCCACCCGGTCCTCTCCCAGTATGAAGGCTTCGAGCACTTTCTGATGTGC
GCTGATGACAAGCAGTGGAAACTGGGCAAGAGACGGGCGGAGAAGGACGA

AATGGTCGGCGCCCATTTCATGCTGACTCTGCAGATCCCCAACGAGCACC
AAGACCTTCAGGATGTGGAGGAGCGGATCGACTCCTTCAAGTCCTTTGCC
AAGAAGATGGACGACAGCGTGATGCAACTCACGCACGTGGCCTCGGAGCT
GGTTCGTAAGCACCTGGGTGGCTTCAGGAAGGAGTTTCAGCGACTGGGAA
ACGCCTTTCAGTCCATCAGCCAGGCGTTCATGCTGGACCCCCCACACTGC
TCAGAGGCCCTCAACAANNNNNNNNNNNNN---------------------------------
----

------------------------NNNNNNNNNNNNNNNNNNNNCACAGAGAGAGAAGTGGCGTTGGGGA
TAAACCCCTTCGCGGACGGGATGGGCGCCTTCAAGATCAACCACAGCTCC
CACGACCTGGGCTCCGG---GCAGACGGCGTTTTCCTCCCAGGCA---CC
GGGCTACGCAGCCGCGGCCGCGCTGGGG---CACCACCA------CCACC
CGACGCACGTCGGCTCC---TACTCAACA------TTCAACTCCACCAGG
GACTTTCTCTTCAGGAACCGGGGTTTCGGGGATGCAACTGG---------
---GGCGCAGCACAGTTTGTTCGCCTC---------TGGAAGTTT---C-
-----CCCGGCGCACATGGACACACGGAGGCTGCAGGACACCTGCTCTTC
CCGGGGCTCCATGAG---CAGGCGGCGGGACACGCATCCACCAACG----
-----GGCAGATGCGACTCGGCTTCTCCGGTGACATGTACGGACGTGCAG
ACCAGTACGGCCACGTTACCAGCCCCAGGT---CCGAGCACTACGCCTCC
ACCCAGCTGCACGGCTACGGCCCCGTCAACATGAATATGGCTGCA---CA
CCACGGTGCAGGGGCCTTTTTTCGGTACATGCGGCAGCCCATCAAACAAG
AGCTCATCTGCAAGTGGATCGAGCCGGAGCAGCTGACGAACCCCAAAAAG

TCGTGCAACAAAACTTTCAGCACCATGCACGAGCTGGTGACCCACTTGAC
GGTGGAACACGTGGGGGGACCCGAGCAGNNNNNNNNNNNNNNNNNNNNNNNNNNNNNNNNNNNNNNNNNN
NNNNNNNNNNNNNNNNNNNNNNNNNNNNNNNNNNNNNNNNNNNNNNNNNNNNNNNNNNNNNNNNNNNNNN
NNNNNNNNNNNNNNNNNNNNNNN
>Lepidogalaxias salamandroides
NNNNNNTTAATCCGCTCAGAATTAAGCCAACCAGGCTCACTTCTAGGGGACGACCA
AATTTACAATGTAATCGTAACAGCCCATGCTTTCGTAATAATCTTTTTTA
TAGTCATACCTATTATAATTGGAGGTTTCGGTAATTGATTAATTCCTCTG
ATAATTGGAGCCCCTGATATAGCGTTCCCACGAATAAACAACATAAGTTT
TTGACTTCTACCTCCCTCTTTTATACTTCTTTTAGCCTCCTCTGCAATTG
AGGCAGGGGCAGGCACAGGATGAACTGTTTACCCCCCATTAGCAGGCAAT
CTTGCTCATGCAGGAGCTTCAGTAGACTTCACAATTTTTTCTCTGCACTT
GGCAGGTATTTCTTCAATCCTAGGGGCAATTAACTTTATTACTACCATCT
TAAACATAAAACCCCCAGCTTTGACTCAATACCAACTCCCACTGTTTGTT
TGATCTGTCTTCATTACAGCCGTACTCTTGTTACTCTCCCTCCCAGTCCT
AGCAGCGGGGATTACAATACTTCTCACTGACCGAAACCTTAATACAACCT
TCTTTGACCCCGCAGGGGGCGGAGACCCTATTCTCTACCAACACTTATTC
TGATTTTTCGGGCACCCCGAAGTCTACATTTTAATTCTTCCAGGGTTTGG
CATAATTTCCCACATCGTTACCTACTATTCCGGCAAAAAAGAACCCTTCG
GCTACATAGGCATAGTATGAGCAATAATAGCAATTGGGTTATTAGGGTTT
ATTGTATGAGCCCATCATATGTTTACTGTGGGTATAGATGTAGACACTCG
AGCNNN---

--AAGAAGGACCCAAGCAAAGGAACACTGGAGGATCAAATCATCCAGGCT

AACCCTGCCCTGGAGGCTTTTGGCAATGCCAAAACATTGCGAAATGACAA
TTCTTCGCGGTTTGGAAAATTCATCCGCATTCATTTTGGAACAAGTGGCA
AATTGTCTTCTGCTGACATAGAGACTTATCTGCTGGAAAAATCACGTGTC
ACCTTTCAACTTAAAGCGGAAAGGAACTATCACATTTTTTTTCAGATATT
GTCCAATCAAAAGCCAGAGTTGCTGGACATGCTGTTAGTTACCAACAATC
CATATGATTATTCTTACATCTCCCAAGGAGAGGTAACAGTTCTATCCATC
AATGATTCTGAAGAACTTATGGCCACTGATAGKGCATTTGATGTGCTTGG
ATTTTCTTCAGAGGAAAAGAGTGGGGTCTATAAATTGACAGGTGCCATCA
TGCACTATGGCAACATGAGGTTCAAGCAAAAGCAGCGTGAGGAGCAAGCA
GAACCTGATGGCACAGAAGCTGCTGACAAGGCTGCCTACCTAATGGGACT
GAACTCAGCAGATCTCTTGAAAGGACTTTGTCACCCAAGAGTCAAGGTTG
GAAATGAGTATGTAACCAAAGGGCAAAGTGTAGATCAAGTATACTAC---

-------------TACCTGATCTACGCCTCCTTCTCCTTCATGGGCTGCC
TGCAGATCAGCGACGGCTCCAACATCGTCAACCTGCTGGCCAGTAATTCG
CCGAGCGTGTCCTACGCGCTCGCACAGCAGAAGTACTTCAGCAACTACAG
CCCCGTGATCGGCTTTTACATCTACGAGCCCATCGAGTACTGGAACTCCA
CCGTGCAGGAGCACCTCAGGACGCTGGGTCAGGGCTTCAACAAAATCTCC
TGGATGGACAACTTCTTCCACTACCTGCGGGCGGTGAACGCCAGCGCCTC
CACCAAGGCCGACTTCATCGCCACGCTCAAGGTCTCCTTCCTGCGTAGCC
CGGAGTACCAGCACTTCACGGAGGACATCATCTTCTCCATGA---ACGGC
G------------AGGAGTACGACATCATCGCGTCCAGGATGTACCTGGT
GGCGAGGACCACCGAGAAGACGCGGGAGGAGGTGGTGGAGCTCCTGGAGA
GGCTGAGGCCCCTGTCGCTCATCAACAGCATCAAGTTCATCGTCTTCAAC
CCCACCTTCGTCTTCATGGACCGATACAGCTCGTCCGTGGTGTCGCCCAT
CCTGACGTCGGCCTTCAGCGTGCTCACCATCCTCATCCTCACCTTCTTCC
TGGTCATCAACCCGCTGGGGAACTTCTGGCTGATACTGACCATCACCTCC
GTGGAGCTCGGCGTCCTGGGCCTGATG-----------------------

--GC
CGTGCGCTTCTCCTTCACACTCATGTCTATCTCCGTCCGGGCCGAAGGTC
AGGAG------------------------------CAGCCAGTCACGGTC
TTCAGGGAGGCCAAGCCCAACTCAGAACTCTCCTGCAAGCCTATGTGCCT
GATGTTCGTGGACGAGTCGGACCACGAGACGCTCACCGCCATCCTGGGGC
CCGTGGTTGCCGAGCGGACGCCATGAAGGAGAGCAGACTCATCCTCTCG
CTGGGTGGCCTCCAGCGCTCTTTCCGCTTCCTGTTCCGGGGCACGGGCTA
TGATGAGAAGATGGTGCGGGAGATGGAGGGGCTGGAGGCCTCGGGCTCCT

CCTATGTCTGCACGCTGTGCGACTCCACCCGCGCTGAGGCCTCCCAGAAC
ATGGTGCTCCACTCCATCACCCGCAGCCACGAGGAGAACCGCGAGCGCTA
CGAGCTGTGGAGGACCAACCCCTTCGCCGAGTCGGCCGACGAGCTTCGCG
AGCGCGTCAGGGGCGTGTCCGCCAAGCCCTTCCTGGAGACCACGCCCACG
CTGGATGCGCTCCACTGTGACATCGGCAACGCCACTGAGTTCTACAAAAT
CTTCCAGGATGAGATTGGCGAGATGCACGGCAGCGC---CAAC---CCCA
GCCGGGAGGAACGGAGGAGCTGGCGCGCCGCCCTGGACAAGCAGCTGAGG
AGGAAGATGAAGCTGAAGCCCGTGATGAGGATGAACGGGAACTACGCCCG
CCGGCTGATGACCCAGGAGGCCGTGGAGGCGGTGTGCGAGCTGGTGCCAT
CGGAGGAGAGGAGGGAGGCGCTGAAGGAGCTCGTGGGGCTCTACGTCCAG
ATGAAG--

NNNNNNNNNNNNNNNNNNNNNNNNNNNNNNNNNNNNNNNNNNNNNNNNNNNNNNNNNNNNNNNAATCTCC
TGAT
CTCTATCCTGCTCGTCAAAGACAAGAGCTTGCACCGGGTGCCCTACTACT
TCCTGCTGGACCTGTGCGCGTCGGACGCGCTGCGCTCCGCCATCTGCTTC
ACCTTTGTGTTCGCGTCGGTGAGAGATGGCTCTGCCTGGTCGTACAGCAT
GATGACATGCAAAGTGATGGCCTTCCTGGGGGTGCTATTGTGCTTTCACA
CCGCCTTCATGCTGTTCTGCGTGAGCGTGTCCCGGTACCTCGCCATAGCG
CACCACCGCTTCTACACCAAGAGGCTGACGTTGTGGACGTGCCTCATTGT
CATCTGCATGGTGTGGACGCTGTCCGTGGCCATGGCCTTCCCCCCGGTGC
TGGACGTGGGGACCTACTCGTTCATCAGGGAGGAGGACCAGTGCACCTTC
CAGCACCGCTCCTTCAGGGCCAACGACTCGCTGGGTTTCATGCTGCTCCT
CGCCCTCATCCTCCTCGCCACGCAGCTCGTCTACCTCAAGCTAATCTTCT
TCGTTCATAACCGCAGGAAGATGAAACCCGTCCAGTTTGTGCCCGCCGTT
AGTCAGAACTGGACCTTCCACGGTCCGGGAGCAAGTGGACAAGCCGCAGC
TAACTGGCTGGTGGGGTTCGGGAGAGGCCCAACTCCGCCTACTCTTCTGG
GTATCAGGCATAGCAACAGCAGATCGGACCAAAGGCGACTACTGGTTTTA
GACGAATTTAAGACTGAGAAGAGGATGAGCAGGATGTACTACATCATGAC
CTTCTTCTTTGTCGTATTGTGGGCCCCTATCTGGTCGCATGTTACTGGA
GGGTGTTCGCTAGAGGTCAAGTGGTCCCAGGGGCATACCTGACTACTGCT
GTCTGGATNNNNNNNNNNNNNNNNNNNNNNNNNNNNNNNNNNNNNNNNNNNNNNNNNNN-------
----

-------------------------------------CAGGGGAAAGTCA
CAGAGAGAGAAGTGGCTTTGGGGATAAATCCGTTCGCTGATGGGATGGGC
GCTTTTAAAATCAACCACAGCTCTCATGATCTTGGCTCCGG---GCAAAC
GGCGTTTTCCTCCCAGGCA---CCGGGCTAC---GCAGCCGCTGCCCTGG
GA---CACCATCA------TCACCCTACCCATGTCAGCTCC---TACTCC
ACCGCGGCTTTCAATTCTACCCGGGACTTTCTCTTCCGAAATCGGGGGTT
CGGAGACGCCACAAG------------CGCGCAGCACAGTCTCTTTGCCT
CAGC---TGCGGGAAGTTT---T------GCAGGGCCACATGGACACTCA
GATGCTACGGGACACCTGCTATTCCCAGGACTTCACGAG---CAAGCTGC
GACCCACGCGTCGTCGAACGTGGTAAACAGTCAGATGCGCTTGGGCTTTT
CGGGAGACATGTACGGGAGGGCTGACCAGTACGGCCATGTTACGAGCCCC
AGGT---CTGATCACTACGCTTCGACTCAGTTGCACGGCTATGGCCCCAT
GAACATGAATATGGCCGCT---CATCACGGAGCAGGGGCCTTCTTCCGTT
ACATGAGACAGCCGATAAAGCAAGAGCTTATCTGTAAGTGGGTCGAACCG
GAGCAACTGTCGAATCCAAAAAAGTCTTGCAACAAAACTTTCAGCACAAT
GCACGAGCTCGTGACCCACCTAACGGTGGAACACGTAGGGGGACCAGAAC
AGTCGAATCATATTTGTTTCTGGGAGGAGTG-------------------

>Lepidogobius lepidus

-----------AAGAAGGATCAGAACAAAGGCACGTTAGAGGACCAGATC
ATCCAGGCCAACCCGGCCCTCGAGGCCTTTGGAAACGCCAAAACCGCCAG
AAACGACAACTCGTCTCGCTTTGGGAAGTTCATCAGGATCCACTTTGGCA
CGAGCGGAAAGCTTTCCTCCGCCGACGTGGAGACCTACCTCCTGGAGAAG
TCCAGAGTCACTTTTCAGCTGAAAGCTGAGAGGAACTACCACATCTTCTA
CCAGATAATGTCCAACCAGAAGCCCGAGCTCCTGGAGCAGCTGCTGATCA
CCACAAACCCGTATGATTACTCCTACACGTCTCAGGGCGAGGTCACCGTG
GCCTCCATCAACGACGCTGACGAGCTGATGGCCACAGACAGCGCCTTCGA
CGTGCTCGGCTTTACTGCCGAAGAAAAGGCCACTGTCTACAAACTCACCG
GAGCCATCATGCACTATGGAAACATGAGGTTCAAGCAGAAGCAGCGTGAG
GAGCAGGCGGAGCCTGATGGCACTGAGTCGGCTGATAAGTCTGCCTTCCT
CATGGGGCTGAACTCGGCCGACCTCATCAAAGGCCTGTGCCACCCAAGAG
TCAAAGTGGGAAATGAGTATGTGACCAAAGGTCAGAGCGTGGACCAGGTT
TACTATCCCAACAAGGAGGCCTTCAAGTGCGACGAGTGTGGGAAGCACTA
CAACACCAAGCTGGGCTACAAGCGCCACGTGGCCATGCACTCCGCCACCT
CCGGAGACCTGACGTGTAAAGTGTGTCTGCAGAGCTACGAGAGCACGCCG
GTGCTGCTGGAGCACCTGAAGAGCCACTCAGGGAAGTCCTCCGGAGGAGC
CAAGGAGAAGAAGCACCCGTGTGACCACTGCGACCGCCGCTTTTACACGC
GTAAGGACGTGCGGCGCCACATGGTGGTGCACACGGGCAGAAAGGACTTC
CTGTGTCAGTACTGCGCCCAACGCTTCGGCCGCAAAGACCACCTGACCAG
ACACGTGAAGAAGAGCCACTCGCAGGAGCTGCTGAAGATCAAGAGCGAGC
CGCCCGACATGCTGGGTCTGCTGGCCTCAGTTTCGCCCCCGTGTGTCGTG
AAGGAGGAGCTCAGCCCCATGATGTGTGCCATGGGCCCCAACAAAGAGCC
TCTGATGGGGAAGCCCTTCCCCGGGGCCGGGCCCTTCTCCATGGGCATGT
ACAACCCACACCTCNNNNNNNNNNNNNNNNNNNNNNNNNNNNNNNNNNNNNNNNNNNNNNNNNNNNNNNN
NNNNNNNNNNNNNNNNNNNNNNNNNNNNNNNNNNNNNNNNNNNNNN------
TATCTCATCTATGCCTCTTTTTCATTCATG
GGATGTTTACAAATTAGTGATGGATCCAACATTGTAAACTTGCTTGCTAG
TAACTCTCCGAGTGTTTCATATGCATTGACCCAGCAGAAGTACTTCAGTA

ACTACAGTCCAGTGATTGGATTTTACATTTATGAACCAATAGAATACTGG
AACTCTACTGTGCAAGAGCACCTCAAGACTCTGAGTCATGGATTCAATAA
AATATCTTGGATGGACAACTTTTTCCATTACTTACGGGTGGTGAACGTGA
GCGCGTCCACCAAAAGTGACTTCATCAGTATCCTCAAGGGCTCGTTCCTA
CGCAGCCCGGAGTACCAGCACTTCACTGAGGACATCATCTTCTCCAAGA-
--ACCGTGAGACGG------ATGAGTATGACATCATTGCCTCGCGTATGT
ACTTAGTGGCACGGACCACAGAGAAGAAGCGTGAGGAGGTGGTGGAGCTT
CTGGAGAAACTTCGGCCACTGATGTTGATTAACAGCATCAAGTTCATTGC
CTTCAATCCAACTTTTGTCTTCATGGACCGTTATAGCTCTTCTGTCATCT
CGCCCATTCTGACCTCAGGCTTCAGTGTGCTCACCATCCTCATCCTCACC
TTCTTCCTGGTCATCAACCCTCTGGGGAACTTCTGGCTCATCCTGACGGT
CACCTCCGTGGAGCTGGGGGTGTTGGGTTTGATGNNNNNNNNNNNNNNNNNNNNNNNNNNNNNNNNNNNN
NNNNNNNNNNNNNNNTGCAGCGTGGGCATTA
TTAATGGTCTCTCGGGATGGACGTCGTCGTTGGATGATGCCCCCGCTGAC
ACAATCACGCGTCGCTTTCGTTATGATGTGGCGTTGGTGTCTGCGYTCAA
AGATCTCGAGGAGGACATTATGGAGGGACTGAGAGGCAGTGAACTGGATG
ACAGCGAGTGCACCTCAGGCTTCAGTGTCATGATCAAGGAATCGTGCGAT
GGCATGGGTGATGTCAGCGAAAAGCATGGAGGAGGACCTGCTGTTCCAGA
GAAAGCTGTTCGTTTCTCTTTCACTATAATGTCAGTGACTTTGGTGAGCG
ATGACAAGGCG------------------------------GGGGAGGTG
ACCATCTTCACTGAATCCAAACCAAACTCAGAGCTGTCCTGTAAGCCCCT
CTGCCTCACGTTTGTTGATGAATCCGACCACGAGACCCTCACTGCTGTTT
TGGCGCCTGTAGTGGCAGAACGCAAAGCAATGAAAGAAAGCAGACTCATT
CTATCCATCGGCGGACTCCCACGGTCGTTCCGTTTCCACTTCAGGGGGAC
AGGTTATGATGAGAAGATGGTGCGTGAGATGGAGGGGCTCGAGGCGTCAG
GATCCACCTATGTCTGCACCCTTTGTGACACCACTCGCTCTGAAGCCTCT
AATAATATGGTTCTGCACTCCATCACACGCAGCCACGAGGAAAACTTAGA
GCGCTATGAAATCTGGAGGAAAAATCCTTACTCTGAGTCCGTGGACGAGC
TCCGAGATAGGGTTAAAGGTGTTTCCGCAAAACCCTTCATGGAGACCCAA
CCGACTCTTGACGCGTTGCACTGCGACATCGGCAACGCCACGGAGTTCTA
CAAAATTTTCCAGGATGAGATCGGGGAGGTGTATGCGAAGGT---TAAC-
--CCCAGTCGGGAAGAACGACGCAGCTGGAGAACGGCCCTGGATAAACAA
CTGAGGAAGAAACTCAGACTCAAACCTGTAATGAGGATGAATGGAAACTA
TGCCCGCAAGCTGATGACCCAGGAGGCTGCAGAGGCCGTTTGCGAGCTGG
TGCCCACAGAGGAGCGAAGGGAGGCCTTGAGGGAGCTCATGAGATTGTAT
ATCCAGATGAAGCCCGTCTGGAGAGCCACCTGCCCCGCAAAGGAGTGCCC
CGACCAGTTGTGCCGATACAGCTTCAACTCCCAGAGAATTGCCGACCTCC
TCTCCTCCACCTTCAAATATAGGTATAATGGGAAAATAACCAATTACCTG
CACAAGACCCTGGCCCACGTCCCAGAAATNNNNNNNNNNNNNNNNNNNNNNNNNNNNNNNNNNNNNNNNN
NNNNNNNNNNNNNNNNNNNN---------------------

----------------------------------NNNNNTTGCACGTTCCTTAAA
TTGACCTCCCTGGGTTTCATCATTGGCGTCGGCGTGGTCGGAAACCTCCT
GATCTCCATCCTGTTGGTCAAAGACAAGAGCTTGCACCGAGCGCCGTACT
ACTTCCTGCTGGACCTGTGCGCGTCGGACATCCTGCGCTCCGCTATCTGC
TTCCCCTTTGTTTTCACCTCGGTCAAGAATGGGTCGGCCTGGACGTACGG
CACGCTGACCTGCAAAGTCATCGCCTTCCTGGGGGTGCTGTCCTGTTTCC
ACACGGCGTTCATGTTGTTCTGCGTGAGCGTTACGCGGTACCTGGCCATC
GCGCATCACCGCTTCTACACCAAGCGGTTGACGTTCTGGACGTGCTTGGC
GGTCATCTGTATGGTGTGGACATTGTCGGTGGCGATGGCGTTCCCCCCGG
TGCTGGACGTAGGGACGTACTCGTTTATCCGTGAGGAGGACCAGTGCACG
TTCCAGCACCGCTCCTTCAGGGCCAACGACTCGCTGGGCTTTATGCTGCT
GCTGGCGCTCATCCTCCTGGCCACACAGCTGGTTTACCTCAAGCTCATTT
TCTTTGTTCACGACCGCCGGAAAATGAAACCGGTCCAGTTTGTGCCTGCT
GTCAGCCAGAACTGGACCTTTCACGGGCCTGGAGCTAGCGGTCAGGCCGC
AGCTAACTGGTTAGCGGGGTTTGGTCGTGGGCCCACCCCGCCCACTCTGC
TGGGCATCCGGCAGAACAGCAACGCAGCAGGCCGACGCCGCCTGCTGGTG
CTGGACGAGTTCAAAACGGAGAAGAGGATTAGTAGGATGTTCTACATCAT
GACTTTTTTCTTCCTGGCTCTGTGGGGGCCCTATCTGGTGGCCTGTTACT
GGAGGGTGTTTGCCAGGGGCCCAGTGGTCCCTGGGGGCTACCTGACCGCA
GCCGTGTGGATGAGCTTCGCCCAGGCCGGGGTCAACCCCTTCATCTGCAT
CTTCTCCAACAGGGAG----------------------------------

NNNNNNNNNNNNNNNNNNNNNNNNNNNNNNNNNNNNNNNNNNNNNNNNNNNNNNNNNNNNNNNNNNNNNN
NNNNNNNNNNNNNNNNNCACAGCTCCCACGA
CTTGGGCTCAGG---ACAGACAGCGTTCTCGTCCCAGGCG---CCCAGTT
AC---------GCCGCGTTAGGA---CACCATCA------CCACGCGACC
CATGTTGGCTCC---TACTCCACA------TTTAACTCCACGCGGGACTT
TCTGTTCAGAAATCGGGGGTTTGGAGACGCTGCAGG------------GG
CACAGCACAGTTTATTCGCTTC---------TGGAAGTTT---T------
GCCGGGCCACATGGACACTCTGAAGCGGCGGGGCACCTGCTCTTCCCCGG
GCTACACGAG---CAAGCGT---------CTTCGTCCAACGTGGTCAATA
GCCAGATGCGCTTGGGCTTCTCAGGGGATGTGTACGGAAGGGCCGAGCAG
TACGGCCATGTCACCAGCCCACGGT---CCGACCATTACGCATCCACGCA
ACTGCACGGCTATGGCCCCATGAACATGAATATGGCCGCGGCGCACCACG
GAGCCGGGGCCTTCTTCAGATATATGCGGCAGCCCATCAAACAGGAGCTC
ATCTGCAAATGGATAGAACCCGAGCAGCTGAGTAACCCCAAGAAGGCTTG
CAACAAAACTTTCAGCACCATGCACGAGCTCGTCACACATTTAACGGTGG
AGCACGTGGGGGACCAGAGCAAACGAACCACGTCTGCTTCTGGGAGGAG
TGCGCCCGAGAGGGGAAACCTTTCAAAGCCAAATACAAACTGGTCAATCA
CATCCGAGTGCACACAGGGGAGAAGCCCTTCCCGTGTCNNNNNNNNNNNNNNNNNNNN

```
>Lepidophanes guentheri
AGCATGCTCATCCGAGCTGAACTAAGCCAACCCGGAGCCCTTCTGGGTGA
CGACCAAATTTACAACGTAATCGTGACAGCTCACGCCTTCGTTATGATTT
TCTTTATGGTAATGCCTCTCATGATTGGAGGGTTCGGAAACTGACTCATC
CCCCTTATGATCGGAGCCCCCGACATGGCCTTCCCCCGAATGAACAACAT
GAGTTTCTGACTTCTCCCACCATCTTTCCTCCTTCTGCTAGCCTCGTCTG
GCATCGAAGCAGGGGCAGGAACAGGCTGAACAGTCTACCCTCCACTCGCA
GGCAATCTTGCCCACGCGGGGGCCTCAGTAGACCTAACAATCTTCTCGCT
ACATCTAGCAGGTATTTCTTCAATTCTCGGGGCTATCAACTTTATTACAA
CTATCATCAACATGAAACCACCAGCAACTACTCAATTCCAAACACCCCTG
TTTGTTTGGGCAGTTCTAATTACAGCCGTTCTCCTGCTCCTTTCACTCCC
GGTCCTGGCTGCAGGAATTACTATGCTCCTGACAGACCGAAACTTAAACA
CCACCTTCTTCGACCCCGCAGGAGGGGGAGACCCTATTCTATATCAACAC
CTCTTCTGATTCTTCGGACACCCGGAAGTCTACATTCTGATTCTCCCTGG
CTTTGGCATGATTTCGCACATCGTCACATACTACGCAGGTAAAAAAGAAC
CTTTCGGCTACATGGGCATGGTTTGAGCCATGATGACCATCGGAATCCTA
GGCTTTATCGTATGAGCCCACCACATGTTTACTGTCGGAATGGACGTAGA
CACACGTGCCTA--------------------------------------
--------------------------------------------------
--------------------------------------------------
--------------------------------------------------
--------------------------------------------------
--------------------------------------------------
--------------------------------------------------
--------------------------------------------------
--------------------------------------------------
--------------------------------------------------
--------------------------------------------------
--------------------------------------------------
--------------------------------------------------
--------------------------------------------------
--------------------------------------------------
--------------------------------------------------
--------------------------------------------------
--------------------------------------------------
--------------------------------------------------
--------------------------------------------------
--------------------------------------------------
--------------------------------------------------
--------------------------------------------------
--------------------------------------------------
--------------------------------------------------
--------------------------------------------------
--------------------------------------------------
--------------------------------------------------
--------------------------------------------------
--------------------------------------------------
--------------------------------------------------
--------------------------------------------------
--------------------------------------------------
--------------------------------------------------
--------------------------------------------------
--------------------------------------------------
```


------CCCAACAAGGAGGCCTTCAAGTGCGAGGAGTGTGGCAAGCACTA
CAATACCAAGCTGGGATACAAGCGCCATGTGGCCATGCACTCCGCCACGG
CGGGGGACCTCACCTGCAAAGTGTGCATGCAGAGCTACGAGAGCACGCCC
GTGCTCCTGGAGCACCTCAAGAGCCACTCGGGGAAGTCGTCGGGAGGCGC
CAAGGAGAAGAAGCACCCGTGCGACCACTGCGACCGCCGCTTCTACACGC
GGAAGGACGTCCGGCGGCACATGGTGGTGCACACGGGCCGGAAGGACTTC
CTGTGCCAGTACTGCGCGCAGCGCTTCGGCCGGAAGGACCACCTGACGCG
CCACGTGAAGAAGAGCCACTCGCAGGAGCTGCTAAAGATCAAGACGGAGC
CTCCAGACATGCTGGGCCTGCTGGGCTCCGGCTCGCCGCCCTGCTCCATC
AAGGAAGAGCTCAGCCCCATGATGTGCAGCATGGGTCCCAACAAGGACCC
CATGATGGGCAAGCCCTTCCCCAGCGGGACGCCTTTCCCCATGGGCATGT
ACAACCCCCACCAC------CTCCAGGCCATGTCCAGTCCAGGGGTGGGG
CACCCG------CACCCCTCCCTGATGCCCAGCTCCCTGTCAGCGGCCAT
GGGCATGGGCTGCCACATGGAGTACCTGATCTACGCTTCCTTCTCCTTCA
TGGGATGTTTACAAATCAGCGATGGGTCAAACATCGCCAACCTGCTGGCG
AGTAACTCTCCGAGCGTGTCTTTCGCACTCACCCAGCAGAAGTACTTCAG
TAACTACAGCCCCGTTATTGGGTTCTACATTTACGAACCCATCGAGTACT
GGAACGCCACGGTGCAGGAGCACCTGAAGACACTGAGCCACGGCTTCAAC
AAGATCTCCTGGATCGACAACTTCTTCCACTACCTGCGGGTGGTGAACGT
GAGCGCGTCGACCAAGAACGACTTCATCACCATCCTAAAGGGCTCCTTCC
TGCGCAGCCCGGAGTACCAGCACTTCACCGAGGACATCATCTTCTCCAAG
A---ACCGCGAGAGCG------ACGAGTACGACATCATCGCCTCGCGCAT
GTACCTGGTGGCGCGCACCACCGAGAAGAAGCGCGAGGAGGTGGTGGAGC
TGCTGGAGAAGCTGCGTCCGCTGATGCTGATCAACAGCATCAAGTTCATC
GCCTTCAACCCCACCTTCGTCTTCATGGACCGATACAGCTCCTCGGTGCT
GTCGCCGATCCTTACCTCAGGCTTCAGCGTGCTCACCATCCTCATCCTCA
CCTTCTTTCTYGTCATCAACCCGCTGGGGAACTTCTGGCTCATCCTGACG
GTCACGTCCGTGGAGCTGGGCGTGCTGGGTCTGATGNNNNNNNNNNNNNNNNNNNNNNNNNNNNNCCCTCA
AGAGCGTG
TCCCCCTCTTGCCACGTGGGCATCATCAACGGGCTCTCTGGCTGGGCCGA
CTTGGTGGATGACTCACCAGCGGACACCATCAGCCGGAGGTTTCGCTACG
ACGTGGCCCTTGTGTCTGCCTTGAAGGATCTGGAGGAGGAGGTAATGGAG
GGACTGAAAGAGTGCGGGCTAGACGACAGCACTTGCACCTCAGGCTTCAG
CGTAATGATCAAAGAGTCCTGCGACGGCATGGGAGATGTCAGTGAGAAGC
ACGGCGGGGGGCCCGCGATCCCCGAGAAGGCGGTCCGTTTCTCCTTCACC
ATTATGTCCATCTCCGTCAAGCCAGATGGGAATGCG--------------
----------------GAGGCGGTCACCATCTTCACAGAGCCAAAGCCCA
ACTCCGAGCTGTCCTGCAAGCCCATGTGCCTGATGTTCGTGGACGAGTCG
GACCACGAGATGCTCACTGCCGTCCTGGGGCCCGTGGTCGCAGAGCGGTC
GGCAATGATGGAAAGCCGCCTCATCCTGTCCATGGGTGGCCTCCCCCGCT
CGTTCCSSTTYTACTTCAGAGGCACCGGSTACGATGAGAAGATGGTGCGC
GAGATGGAGGGCCTTGAGGCATCAGGCTCTACCTATGTCTGCACCCTGTG
SGACTCCACCCGGGCRGAGGCCTCCCACAACATGGTGCTCCACTCCATCA
CCCGCAGCCACAGCGAGAACCTGGAGCGCTACGAGATATGGCGGAGCAAC
CCCTTTTCGGAGTCTGTCGAGGAGCTGCGAGATCGGGTGAAGGGGGTCTC

CGCCAAGCYCTTCATGGAAACCCAGCCTACTCTGGACGCGCTGCACTGTG
ACATTGGCAATGCCACCGAGTTCTACAAAATCTTCCAGGATGAGATCGGG
GAGGTGTTCCAGAAGGC---CAAT---CCCAGCCGCGAGGAAAGGCGACG
CTGGAGGGCTGCCCTCGACAAGCAGCTGAGGAAAAAACTCAAGCTCAAGC
CAGTGATGCGGATGAACGGGAACTACGCCCGGAGGCTGATGTCGGAGGAG
ACCGTGGAGGTGGTGTGTGAACTGGTGCCCTCGGAGGAACGGCGGGAGGC
TCTGAGGGAGCTAATGGGGCTTTACCTCCAGATGAAACCTGTGTGGCGCG
CCAGCAACCCAGCCAAGGAATGCCCCGACCAACTGTGCCGCTACAGTTTC
AACTCCCAGCGCTTCGCTGACCTCCTCTCCACCACCTTCAAATACAGGTA
TGACGGAAAGATTACCAATTACCTGCACAAGACCCTGGCCCACGTCCCTG
AAATCATAGAAAGAGATGGCTCCATTGGAGCTTGGGCCAGTGAGGGGAAC
GAGTCAGC-------TCGTACACCATCGAGATGGGCCCCAAGGGGCCCCT
GTGGAAGGAGAGCCCTCAGCCCTTCTCCTGTTCCATCGAGGACCCCACGA
AACAGACCAAGTTCAAGGGCATCAAGACGTACATCTCCTACCGGGTCACG
CCGAGCCACACCGGTCACCCCGTGTACAGACGCTACAAGCACTTTGACTG
GTTGTACAACCGCTTGCTGCACAAGTTCACCGTGATCTCTGTCCCCCACC
TGCCGGAGAAGCAGGCGACCGGGCGCTTTGAGGAGGACTTCATAGAAAAG
CGCAAGAGGCGGCTGATCCTCTGGATAAACCACATGACCAGTCACCCTGT
CCTCTCCCAGTATGAGGGCTTTGAACACTTTCTGATGTGTGGCGACGACA
AGCAATGGAAGCTGGGCAAGCGGCGAGCGGAGAAGGATGAAATGGTCGGC
GCCCACTTCATGCTGACCTTCCAGATCCCCAACGAGCACCAGGACCTCCA
GGACGTAGAGGAGCGCGTCGACTCCTTCAAGGCGTTTGCCAAGAAGATGG
ACGACAGTGTCATGCAGCTGACACACGTGGCCTCGGAACTCGTGCGCAAG
CACCTGGGTGGGTTCCGCAAGGAGTTCCAGCGGCTGGGGAACGCCTTCCA
GAACATTAGCCAGGCCTTCATGCTTGACCCGCCGCATAGCTCGGACGGCC
TCAACAACGCCATCTCACAC------------------------------

NNNNNNNNNNNNNNNNNNNNNNNGGTGGCGTTGGGGA
TCAACCCGTTCGCGGACGGCATGGGCGCCTTCAAAATAAACCACGGCTCC
CACGACATCGGCTCCGG---GCAGACGGCGTTCTCCTCGCAGGCG---CC
CGGCTAC---GCGGCGGCCGCCCTGGGG---CACCACCA------CCACC
CGACCCACGTCAGCTCG---TACTCCACGGCAGCCTTCAACTCCACCAGG
GACTTTCTGTTCAGAAACAGGGGCTTCGGAGACGCCACCAG---------
---CGCCCAGCACAGTTTGTTCGCCTCCGC------GGGAAGTTT---C-
-----GCGGGGCCACATGGACACTCGGATGCCGCGGGGCACCTGCTCTTC
CCCGGGCTGCACGAA---CAAGCCGCRAGCCACGCGTCCTCGAACGTCGT
CAACAGCCAGATGCGACTGGGCTTTTCGGGGGACATGTACGGACGGGCCG
ACCAGTACGGCCACGTCACGAGCCCGCGGT---CCGACCACTACGCGTCG
ACCCAGTTGCACGGCTACGGCCCTATGAACATGAATATGGCCGCG---CA
CCACGGAGCCGGGGCCTTCTTTCGGTACATGCGGCAGCCGATCAAGCAAG
AGCTCATCTGCAAGTGGATCGAGCCGGAGCAACTGTCGAACCCCAAGAAG
GCGTGCAACAAAACTTTCAGCACGATGCACGAGCTCGTCACCCATCTGAC
GGTGGAGCATGTGGGGGGGCCGGAGCAGGCCAACCACATATGCGTCTGGG
AGGACTGCGCTCGCGAGGGGAAGCCGTTCAAAGCCAAATACAAACTTGTG
AATCATATCAGAGTACACACTGGAGGAAAACCCTTTCCGTGTCCCTTTNNNNNNNN--

>Lepomis macrochirus
AGCCTACTCATTCGAGCAGAGCTCAGCCAACCAGGCGCTCTCCTGGGCGA
CGACCAAATTTATAACGTAATTGTGACAGCACATGCATTCGTAATAATTT
TCTTTATAGTAATACCAATTATGATTGGTGGCTTTGGCAACTGACTTGTC
CCATTAATAATTGGAGCCCCCGATATAGCATTCCCCCGAATAAATAACAT
AAGCTTTTGACTTCTCCCCCCCTCTTTCCTTCTTCTTCTCGCCTCCTCCG
GGGTTGAAGCCGGGGCTGGCACAGGATGAACCGTTTACCCCCCTCTCGCT
GGTAACCTAGCCCATGCAGGAGCATCAGTCGACCTTACTATCTTCTCCCT
GCATCTCGCAGGGGTCTCTTCAATCCTGGGAGCTATTAATTTTATTACCA
CAATTATTAACATGAAGCCCCCTGCTATTTCCCAGTACCAGACCCCTTTA
TTTGTCTGATCAGTCCTAATTACTGCCGTCTTACTCCTGCTTTCCCTTCC
AGTCCTTGCTGCAGGCATCACAATACTACTTACAGACCGTAATCTAAACA
CCACTTTCTTTGACCCAGCGGGGGGCGGGGACCCAATCCTCTATCAGCAC
CTG---


```
--------------------------------------------------------
--------------------------------------------------------
--------------------------------------------------------
--------------------------------------------------------
--------------------------------------------------------
--------------------------------------------------------
----------------------------GGATGAATACATTGTTGTGTTTAGT
CGTTCAACGACGAGGCTGATTCTAAATGAAGCAGAGCTAATCATGGCGCT
GGCCCAGGAGTTCCAGATGAGAGTTGTCACGGTATCACTGGAGGAACAGT
CCTTCCCCGGCATCGTGCAGGTGATCAGCGGTGCTTCCATGTTAGTCAGT
ATGCATGGAGCTCAGCTTATCACCTCCCTCTTCCTCCCCAGAGGAGCTGC
TGTGGTGGAGCTCTACCCTTTTGCTGTTAACCCAGAGCAATACACCCCGT
ATAAAACCCTTGCGTCCCTTCCAGGAATGGACCTTCACTATATCTCCTGG
AGGAACACTAAGGAGGAGAACACCATCACCCACCCAGACAGACCCTGGGA
ACAAGGGGGCATTGCTCACTTGGAGAAGGAGGAGCAAGAGCGTATACTGG
CTAGCAAGGACGTCCCCAGGCACTTGTGCTGCCGCAACCCAGAGTGGCTC
TTCCGGATCTACCAGGACACTTTGGTCGACATCCCTTCCTTCCTGGAAGT
CCTCAA---AGAGGGCATGAAG---ACAAAGCCCAGCCTGAAAAA---GT
CAAAGCCGGCCAGCACAGTCCATCCAGGCCGGGTCAGAGAACCCCAGTGT
CAGACCTCAGTACAAAACACTAACGAGGCTAAACTCACAGTCTCTTGGCA
GATCCCATGGAATCTGAAATACCTGAAGGTGCGAGAGGTGAAGTACNNNNNNNNNNNNNNNNAAAA
GAGACACCAGCAAAGGGACCCTGGAGGATCAAATCATCCAGGCAAACCCG
GCGCTGGAGGCCTTCGGCAACGCCAAAACGCTGAGAAACGACAACTCGTC
TCGTTTTGGAAAATTCATAAGAATTCACTTCGGTACGAGCGGCAAGCTGG
CATCTGCCGACATCGAGACGTACCTGCTGGAGAAGTCACGTGTCACCTTT
CAGCTCAAGGCTGAGAGAAACTACCACATATTCTACCAGATCCTGTCCAA
TCAGAAGCCAGAGCTGCTGGACATGTTGCTCATCACCAACAACCCGTACG
ACTACTCCTACATCTCCCAAGGAGAGGTGACAGTCGCCTCCATCAACGAC
TCCGAGGAGCTGATGGCCACCGACAGCGCCTTCGATGTACTCGGCTTCAC
TGCAGAGGAGAAGATGGGCGTCTATAAACTGACGGGCGCCATCATGCACT
ACGGCAACATGAAGTTCAAACAGAAGCAGCGTGAGGAGCAGGCGGAGCCG
GACGGGACGGAGGCTGCTGATAAGTCAGCTTACCTAATGGGGCTGAACTC
TGCTGACCTCATCAAAGGGCTGTGCCATCCCAGAGTCAAGGTAGGAAACG
AGTACGTCACCAAAGGACAAAGTGTGGACCAAGTCAACTACCCTAACAAG
GAGGCCTTCAAGTGTGAGGAGTGTGGGAAGCACTACAACACCAAGCTGGG
ATATAAGCGCCATGTGGCCATGCACTCTGCCACAGCAGGGGATCTCACCT
GTAAAGTTTGCATGCAGACCTACGAGAGCACACCCATACTCCTGGAGCAC
CTCAAGAGCCACTCTGGGAAGTCTTCTGGTGGCGCCAAGGAGAAAAAACA
CCCGTGCGACCACTGTGACCGGCGTTTCTATACACGAAAGGATGTGAGAC
GGCACATGGTGGTCCACACAGGCCGAAAGGACTTCCTGTGCCAGTACTGT
GCCCAGCGTTTTGGCAGGAAGGACCATCTGACACGCCATGTGAAGAAGAG
CCACTCACAGGAGCTGCTGAAGATCAAGACAGAGCCTCCTGATATGTTAG
GTCTTTTAGCCTCAGGGTCACCGCCTTGCTCTGTGAAGGAGGAGCTCAGC
CCCATGATGTGTGGAATGGGTCCCAATAAAGACCCAATGATGGGCAAACC
ATTCCCCAGTGGGGCCCCTTTTCCGATGGGCATGTACAACCCTCACCAT-
-----CTCCAGGCCATGTCTAATTCTGGGGTGGGTCACCCA------CAC
CCGTCCCTGATGCCCAGTTCCTTGTCTGCAGCTATGGGCATGGGCTGTCA
CATGGAA-------------------------------------------
--------------------------------------------------------
--------------------------------------------------------
--------------------------------------------------------
--------------------------------------------------------
--------------------------------------------------------
```


---------------------GGCTTTCACCAATTTGAATGGCAGCCAGC
TCTTAAGAATGTGTCTATATCGTGCAATGTTGGCATTATTAATGGGCTCT
CAGGATGGGCTTCCTCGGTGGACGACGCTCCAGCTGACACTATCACTCGG
CGGTTTCGCTATGATGTGGCACTGGTGTCAGCATTAAAGGATCTGGAGGA
GGACATCATGGAGGGGCTGAGAGAGAGTGGGATGGAAGACAGTGCTTGCA
CTTCAGGCTTCAATGTCATGATCAAGGAATCTTGTGATGGCATGGGTGAT
GTCAGCGAGAAGCACGGTGGAGGACCAGTTGTTCCTGAGAAGGCTGTACG
TTACTCTTTCACTGTTATGCGTGTCTCTGTCCTGGCAGACGATGAGAAA-
-----------------------------GAAGAGGTAACCATCTTCACC
GAGCCAAAGCCAAACTCAGAACTGTCCTGTAAGCCCCTTTGCCTGACTTT
TGTAGATGAGTCCGACCATGAGACACTTACAGCTGTCCTGAGCCCTATAG
TTGCTGAACGTAACGCAATGAAAGAGAGCAGGCTCATCCTATCCATGGGC
GGACTACCTCGCTCCTTCCGCTTCCACTTCAGAGCCACTGGATATGATGA
GAAGATGGTGCGTGAGATGGAGGGCCTCGAGGCCTCAGGGTCCACCTATA
TCTGCACTCTTTGTGACTCCAGTCGGGCAGAGGCCTCTCAAAACATGGTG
CTACACTCTGTCACCCGCAGTCATGAAGAGAACCTTGAACGCTATGAAAT
ATGGAGAACCAACCCCTTTTCTGAGTCTGCAGATGAGCTGCGAGACAGAG
TCAAAGGTGTTTCTGCCAAGCCCTTCATGGAGACCCAGCCCACACTGGAT
GCATTACACTGTGACATCGGAAATGCCACTGAGTTTTACAAAATCTTCCA
GGACGAGATCGGGGAGGTGTACAAAAAGGC---CAAC---CCCAGCCGGG
AGGAACGGCGCAGCTGGAGGGCAGCCCTAGATAAACAGCTGAGGAAGAAG
ATGAAGCTTAAACCGGTGATGAGGATGAATGGGAACTATGCCCGCAGGCT
AATGACCCAGGAGGCTGTGGAGGTGGTGTGTGAGCTAGTGCCCTCAGAGG
AGAGGAGAGAGGCCCTGAGGGAGCTTATGAGGCTTTACCTCCAGATGAAG
CCTGTGTGGCGTGCCACCTGCCCAGCCAAAGAGTGCCCTGACCAGCTGTG
CCGCTACAGCTTTAACTCCCAGAGTTTTGCCGACCTCCTCTCCTCTACCT
TCAAATACAGGTACAATGGAAAGATAACCAATTACTTGCACAAGACCCTG
GCCCATGTGCCTGAAATCATAGAGAGAGATGGATCCATAGGAGCCTGGGC
TAGCGAGGGGAATGAGTCAGCAAACAAA----------------------

--CATCACTCAA
CAGGTGAAGTCACAGAGAGAGAAGTGGCGTTGGGGATAAATCCGTTCGCA
GATGGGATGGGCGCCTTCAAAATAAACCACAGCTCCCACGATATTGGCTC
CGG---ACAAACGGCGTTTTCCTCCCAGGCG---CCCGGCTAC---GCGG
CAGCCGCTCTGGGA---CACCATCA------CCACCCGACCCACGTTGGG
TCT---TACTCCACGGCGGCTTTCAACTCCACCAGGGACTTTCTCTTCCG
AAATCGGGGCTTTGGGGACGCCACCGG------------GGCGCAACACA
GTTTGTTCGCCTC---------CGGAAGTTT---C------GCAGGGCCA
CATGGACATTCGGATGCAGCGGGGCACCTGCTCTTCCCAGGGCTCCACGA
G---CAAGCTGCGAGCCATGCGTCTTCCAACGTGGTCAACAGCCAGATGC
GGCTGGGCTTCTCGGGGGACATGTACGGACGCGCCGACCAGTATGGCCAC
GTTACAAGCCCGCGGT---CCGACCACTACGCTTCGACCCAGCTGCACGG
CTATGGCCCCATGAACATGAATATGGCCGCA---CACCACGGAGCAGGGG
CCTTCTTTCGATACATGAGGCAGCCCATCAAACAAGAGCTCATCTGCAAG
TGGGTCGAGCCGGAGCAGTTGACGAATCCCAAAAAGGCGTGCAACAAAAC
TTTTAGCACGATGCACGAGCTTGTGACCCATCTGACGGTGGAGCATGTGG
GGGGACCGGAGCAGACCAACCACATCTGCTTCTGGGAGGAATGCGCCAGA
GAAGGAAAGCCATTCAAAGCCAAATACAAACTTGTAAATCATATCAGAGT
ACACACCGGAGAAAAGCCCTTTCCGTGTCCGTTCCCCGGCTGTGGCAAA
>Lestidiops jayakari
AGTCTTCTTATCCGAGCGGAACTAAGTCAACCCGGGGCCCTCTTGGGTGA
CGACCAGATTTACAACGTAATCGTCACAGCTCATGCCTTCGTAATAATTT

TCTTCATGGTCATGCCCATCATAATCGGCGGCTTCGGGAACTGGCTTATC
CCACTGATAGTCGGAGCCCCTGATATGGCTTTCCCTCGAATAAACAACAT
AAGCTTCTGACTGCTCCCTCCGTCCTTCCTGCTCCTTCTGTCATCCTCCG
CCGTAGAGGCGGGCGCAGGGACCGGGTGAACGGTTTATCCCCCCCTTGCC
AGCAATCTGGCGCACGCGGGTGCCTCTGTCGACCTGACCATCTTCTCCCT
TCACCTGGCCGGCATTTCGTCCATCCTCGGAGCCATCAACTTCATCACGA
CCATCATTAACATAAAACCCCCTGCCATCACCCAGTATCAAACGCCCCTC
TTTGTTTGAGCCGTTCTTATTACTGCTGTGCTTCTCCTTCTCTCCCTGCC
TGTCTTAGCAGCAGGCATTACAATGCTTCTTACAGACCGAAACCTAAACA
CGACCTTCTTTGACCCCGCTGGGGGCGGCGACCCCATCCTATATCAGCAC
CTCTTCTGGTTCTTCGGCCACCCGGAAGTCTACATTCTCATCTTACCCGG
CTTCGGAATAATCTCCCACATCGTTGCCTACTATTCAGGGAAAAAAGAAC
CTTTCGGGTATATAGGCATAGTCTGAGCAATAATAGCAATCGGCCTCCTC
GGGTTTATTGTCTGAGCCCACCTCATGTTCACGGTCGGGATAGACGTGGA
CCCACGAGCATATTCCTCGAGAGGAACCTGCACCCATCCAACTGCCTTGG
CATGCTGCTACTGTCAGATGCCCACCAGTGCACCAAGCTGTCAGAGCTGT
CCTGGGGTATGTGTCTCAGCAACTTCCCTGCCATCTGCAAGACGGAAGAC
TTCCTCCAGCTGCCCAAAGACATGGTGGTGCAGCTCCTGTCCCATGAAGA
GCTGGAGACGGAAGATGAAAGACTGGTTTATGAGGCTGCCCTTAACTGGG
TCAACTATGACCTGGAGAGGAGACACTGCCACCTGCCCGAGCTGTTGAGA
ACCGTACGCCTGGCCTTGCTTCCCGCCATCTTCCTCATGGAGAACGTCTC
CACAGAGGAGCTGATCAATGCCCAGGCAAAGAGCAAGGAGTTGGTGGACG
AGGCCATCCGTTGCAAGCTGAAGATCCTGCAGAATGACGGTGTTGTTAAC
AGTCCCTGTGCCCGGCCCAGAAAGACCAGCCACGCCCTTTTCCTACTGGG
AGGGCAGACTTTCATGTGTGACAAGCTGTATTTGGTGGACCAGAAGGCCA
AAGAGATCATCCCCAAGGCGGACATCCCCAGCCCCAGGAAGGAGTTCAGC
GCCTGTGCCATTGGCTGTAAGGTGTACGTCACAGGCGGGA--GAGGCTC-
GGAGAACGGTGTATCTAAAGATGTGTGGGTCTATGACACCGTCCACGAGG
AGTGGTCGAAGGCAGCACCCATGCTCATTGCCAGGTTTGGCCATGGCTCT
GCTGAGCTCAAACACTGCCTCTACGTGGTTGGAGGACACACAGCAGCCAC
TGGCTGCCTCCCAGCCTCTCCGTCTGGATGAGTACATCGTAGTGTTCAGT
CGTTCCACAACGAGGCTGATTCTGAACGAAGCGGAGCTGATCCTGGCGCT
GGCCCAAGAGTTTCAGATGAGGGTGGTCACTGTGTCCCTGGAGGAACAGT
CTTTCCCCAGCATCGTACAGGTCATCAGTCGGGCCTCCATGTTGGTTAGT
ATGCACGGAGCTCAGCTCGTCACCTCCCTCTTCCTGCCTAGAGGAGCCGC
CGTGGTAGAGCTTTTCCCCTACGCTGTGAACCCGGAACAGTACACCCCAT
ACAAAACCCTAGCCTCCCTACCAGGCATGGACCTTCAATACGTTTCCTGG
AGGAACACTATTGAGGAGAACACCGTCACCCATTCAGACAGACCTTGGGA
CCAAGGAGGCATCACCCATTTGGAGAAGGAAGAGCAGGAGAGAATCCTAG
CCGGCAAGGATGTCCCCAGGCACCTATGCTGCCGCAACCCGGAGTGGCTC
TTCAGGATCTACCAGGACACTCTGGTAGACATCCCGTCCCTCCTGGAAGT
CCTCAA---GGAAGGCCTGAAG---ATGAGGCCAGGCTTGAAGAA---GT
CCAGGCCGGCCAGCACGGTTCACCCGGGCCGGGTCCGAGAACCCCAGTGC
CAGACTTCGGTTCAGGCCACCAACGAGGCTAAACTCACAGTGTCCTGGCA
GATCCCGTGGAACCTCAAGTACCTGAAGGTGCGAGAGGTGAAGTACGAGG
TATGGATCCAGAAAAGGGACACAAGCAAGGGAACCCTGGAGGATCAAATC
ATTCAGGCAAACCCTGCACTGGAGGCTTTTGGTAATGCCAAAACAATCAG
GAATGATAATTCCTCCCGTTTTGGAAAATTCATCCGAATCCACTTTGGAA
ACAGTGGTAAACTGTCCTCTGCGGACATTGAGACCTACCTGCTGGAGAAG
TCACGGTGCACCTTTCAGCTTAAGGCAGAGAGGAACTACCACATCTTCTT
CCAGATCTTGTCCGAGCAAAAGCCAGAGCTTCTGGACATGCTGTTGATCA
CCAACAACCCCTATGACTACAGCTACATCTCCCAAGGAGAAGTAACTGTA
GCATCCATCAATGATTCCGAAGAGCTGATGGCCACTGACAGTGCCTTTGA

TGTGCTTGGCTTCACGCAAGAGGAGAAGATGGGAGTCTATAAGTTGATAG
GGGCCATTATGCACTATGGCAACATGAGGTTCAAGCAAAAGCAGCGAGAG
GAACAGGCTGAGCCTGACGGTACAGAGGCTGCTGATAAGTCAGCTTATCT
AATGGGGCTGAATTCAGCAGACCTAATCAAGGGACTCTGCCATCCCAGAG
TCAAGGTTGGGAATGAGTATGTCACCAAAGGCCAGGGTGTAGATCAAGTC
TACTACCCCAACAAGGAGGCCTTCAAGTGCGAGGAGTGCGGCAAGCACTA
CAACACCAAGCTGGGATACAAGCGTCATGTGGCCATGCACTCGGCCACGG
CGGGGGACCTCACCTGCAAGGTGTGCATGCAGAGCTACGAGAGCACGCCG
GTGCTGCTGGAGCACCTCAAGAGCCACTCGGGGAAGTCGTCGGGTGGCGC
CAAGGAGAAGAAGCACCCGTGCGACCACTGCGACCGCCGCTTCTACACGC
GCAAGGACGTGCGGCGGCACATGGTCGTCCACACGGGCCGCAAGGACTTC
CTGTGCCAGTACTGCGCCCAGCGCTTCGGCAGGAAGGACCACCTGACGCG
GCACGTGAAAAAGAGCCACTCGCAGGAGCTGCTGAAGATCAAGACGGAGC
CTCCGGACATGCTGGGCCTCCTGGGCACCGGCTCGCCGCCTTGCTCCGTC
AAGGAGGAGCTGAGCCCCATGATGTGCAGCATGGGTCCCAACAAAGACCC
CATGATGGGCAAGCCCTTCCCCAGCGGGACGCCCTTCCCCATGGGCATGT
ACAACCCCCACCAC------CTGCAGGCCATGTCTAACCCCGGGGTGGGC
CACCCG------CACCCCTCCCTCATGCCCGGCTCCCTGTCGGCGGCGAT
GGGCATGGGCTGCCACATGGAGTATCTCATCTATGCTTCCTTCTCCTTCA
TGGGATGTTTACAAATCAGCGACGGGTCGAACGTAGTGAATTTGCTGGCG
AGTAACTCTCCGAGCGTCTCGTACGCTCTGACGCAGCAGAAGTACTTCAG
TAACTATAGCCCCGTGATYGGGTTTTACATCTACGAGCCCATCGAGTACT
GGAACTCCACGGTGCAGGAGCACCTGAAGACGCTGAGCCACGGCTTCAAT
AAGATCTCTTGGGTGGACAATTTCATCCACTACCTGCGGGCGGTGAACCT
GAGCGCGTCGACCAAGGCCGACTTYGTTGCCGTCCTCAAGGGCTCCTTCC
TGCGGAGCCCGGTGTACCAGCACTTCACAGAGGACATCATCTTCTCCAAG
A---GCCACGAGAACA------ACGAGTACGACATCATCGCCTCGCGCAT
GTACCTGGTGGCGCGCACCACGGAGAAGAGGCGCGAGGATGTGGTGGAGC
TGCTGGAGAAGCTCCGGCCGCTGATGTTGATCAACAGCATCAAGTTCATT
GCCTTCAACCCCACGTTCGTCTTCATGGACCGCTACAGCTCCTCGGTCAT
CTCGCCCATCCTCACCTCGGGCTTCAGCGTGCTCACCATCCTCATCCTCA
CCTTCTTCCTGGTCATCAACCCGCTGGGGAACCTGTGGCTCATCCTGACG
GTCACGTCCGTGGAGCTGGGCGTGCTGGGCCTGATGGGCTACCACACATT
CGAGTGGCAGCCGGCCCTGAAGAACGTGTCCCCATCCTGCCACGTGGGGA
TCATCAACGGGCTTTCCGGGTGGGCCACCTCGGTGGACGACGTCCCTGCT
GACACCATCACCCGCCGGTTTCGCTACGATGTGGCGCTCGTATCGGCCCT
GAAGGACCTAGAGGAGGACATCGTGGAAGGACTGAGGGAGTGTGGGCTGG
AAGACAGCACTTGTACCTCAGGCTTCAGCGTTATGATCAAGGAATCCTGC
GATGGCATGGGAGACGTCAGCGAGAAGCACGGCGGGGGGCCACCGGTGCC
CGAGAAGGCCGTGCGTTTCTCCTTCACCGTCATGTCCGTCTCCGTCCAGG
CTGACGGAGAAGAG------------------------------GAGGAG
GCTGTCGTCTTCAGGGAGCCAAAGCCCAACTCCGAGCTGTCCTGTAAGCC
CCTGTGCCTGATGTTTGTGGACGAGTCTGACCACGAGACGCTCACCGCCG
TCCTGGGGCCTTTGATCGCGGAGAGGAACGCGATGAAGGAAAGCCGMCTC
ATCCTCGCGATGGGAGGCCTCCCGCGCTCCTTCCGTTTCCACTTCAGAGG
CACGGGCTACGATGAGAAGATGGTGCGAGAGATGGAAGGCCTGGAGGCCT
CGGGCTCCACCTATATCTGCACTCTTTGTGACTCCACTCGGGCAGAAGCC
GCTCACAACATGGTGCTCCACTCCGTCACCCGCAGCCACGAGGAGAACCT
GGAGCGCTACGAGATATGGAGGACCAACCCCTTCTCGGAGTCCGCCGTGG
AGCTGCGAGATCGGGTCAAAGGGGTCTCCGCCAAACCGTTCATCGAGACC
CATCCGACTATGGACGCGTTGCATTGTGACATAGGTAATGCCACTGAATT
CTACAAGATCTTCCAGGATGAGATCGGGGAGGTGTTCCAGAAGGC---CA
AC---CCAAGCCGGGAGGAGCGGCGCAGCTGGCGGGCAGCCCTCGATAAA

CAGCTGAGGAAGAAGATGAAGCTGAAGCCGGTGATGCGGATGAATGGGAA
CTATGCCCGAAAGCTGATGACCGAGGAGGCCATAGAGGCGGTGTGTGAAC
TGGTGCCCTCGGAAGAGAGACGGGAGGCCCTGAGGGAGCTGATGAGGCTC
TATATCCAGATGAAGCCGGTGTGGCGTGCTACCTGCCCGGCCAAGGAGTG
CCCCGACCAGCTGTGCCGCTACAGCTTTAACTCTCAGCGCTTCGCCGATC
TCCTCTCCTCCACCTTCAAATACAGGTACGATGGGAAGATCACCAACTAC
CTGCACAAGACCCTGGCCCACGTTCCTGAGATCATAGAGAGAGACGGCGC
CATCGGAGCCTGGGCCAGCGAGGGGAATGAGTCTGCGAACAAA-------

>Lestrolepis japonica
AGCCTGCTAATCCGAGCAGAACTAAGCCAACCGGGGGCCCTATTGGGCGA
CGACCAGATTTATAATGTCATCGTTACAGCCCACGCTTTCGTGATAATCT
TCTTCATAGTCATGCCCGTTATAATTGGGGGCTTCGGAAACTGACTAATC
CCACTAATAATCGGAGCCCCTGATATGGCTTTCCCCCGAATAAATAACAT
GAGCTTCTGACTTCTTCCCCCCTCTTTTCTCCTACTTCTTGCCTCCTCTG
CCGTCGAAGCTGGGGCCGGCACCGGATGAACAGTTTACCCCCCTCTTGCG
AGCAACTTGGCTCATGCAGGTGCTTCTGTGGACCTGACTATTTTTTCCCT
TCACTTAGCCGGGATTTCATCGATCCTAGGAGCCATTAATTTTATTACCA
CAATCATTAACATAAAACCTCCAGCTATCACGCAATATCAAACCCCTCTA
TTCGTGTGAGCCGTTTTAATCACCGCTGTCCTCCTCCTCTCCCTCCC
TGTTCTAGCCGCAGGGATCACAATACTACTCACAGACCGGAACTTAAACA
CAACTTTCTTCGACCCGGCAGGAGGCGGCGACCCAATTCTGTACCAACAC
CTGTTTTGATTCTTTGGCCACCCAGAGGTATATATTTTGATCTTACCCGG
ATTTGGGATAATCTCTCACATTGTGGCATACTACTCAGGAAAAAAAGAAC
CTTTTGGGTATATGGGTATAGTATGGGCAATAATAGCAATTGGCCTCCTG
GGGTTCATCGTATGAGCCCACCACATATTTACAGTGGGGATAGACGTAGA
CACACGAGCATATTCCTCGAGAGGAACCTGCACCCATCCAACTGCCTTGG
CATGCTGCTACTGTCAGATGCCCACCAGTGCACCAAGCTGTCAGAGCTGT
CCTGGGGTATGTGCCTCAGCAACTTCCCTGCCATCTGCAAGACGGAAGAC
TTCCTCCAGCTGCCCAAAGACATGGTGGTCCAGCTTCTGTCCCATGAAGA
GCTGGAGACTGAAGATGAGAGACTGGTTTATGAGGCTGCCCTTAACTGGG
TCAACTATGACCTGGAGAGGAGACACTGCCACCTGCCAGAGCTGTTGAGA
ACCGTACGCCTGGCCTTGCTTCCCGCCATTTTCCTCATGGAGAACGTCTC
CACGGAGGAGCTGATCAATGCCCAGGCAAAGAGCAAGGAGTTGGTGGATG
AGGCCATCCGTTGCAAGCTGAAGATCCTGCAGAATGACGGTGTGGTCAAC
AGTCCCTGTGCCCGGCCCAGAAAGACCAGCCACGCCCTTTTCCTGCTGGG
AGGGCAGACCTTCATGTGTGACAAGCTGTACTTGGTGGACCAGAAGGCCA
AAGAGATCATCCCCAAGGCGGACATCCCCAGCCCCAGGAAGGAGTTCAGC
GCCTGTGCCATTGGCTGTAAGGTGTACGTCACAGGCGGGA--GAGGCTC-
AGAGAACGGTGTATCTAAAGATGTGTGGGTCTATGACACCGTCCACGAGG

AGTGGTCCAAGGCAGCACCCATGCTCATTGCCAGGTTTGGCCATGGCTCT
GCCGAGCTCAAACACTGCCTCTATGTGGTTGGAGGGCACACAGCAGCCAC
TGGCTGCCTCCCAGCCTCTCCGTCT-------------------------

-----------AAAAGGGATACAAGCAAGGGAACCTTGGAGGATCAAATC
ATTCAGGCAAACCCTGCACTAGAGGCTTTTGGGAATGCCAAAACATTGAG
GAACGATAATTCCTCCCGTTTTGGAAAATTCATCCGAATCCACTTTGGAA
CCAGTGGTAAACTGTCCTCTGCAGACATTGAGACCTACCTGCTGGAGAAG
TCACGGTGCACCTTTCAGCTTAAGGCAGAGAGGAACTACCACATCTTCTT
CCAGATCTTGTCCAATCAAAAGCCAGAGCTGCTGGACATGCTGTTGATCA
CCAACAACCCCTATGACTACAGCTACATCTCCCAAGGAGAAGTGACCGTA
GCATCCATCAATGATTCCGAAGAGCTGATGGCCACTGACAGTGCCTTTGA
TGTGCTTGGCTTCACGCAAGAGGAGAAGATGGGAGTCTACAAGTTGATAG
GGGCCATTATGCACTATGGCAACATGAGGTTCAAGCAAAAGCAGCGAGAG
GAACAGGCTGAGCCTGATGGTACAGAGGCTGCTGATAAGTCAGCTTATCT
AATGGGGCTGAATTCAGCAGACCTAATCAAGGGACTCTGCCATCCTAGAG
TCAAGGTTGGGAATGAATATGTCACCAAAGGCCAGGGTGTAGATCAAGTC
TACTACCCCAACAAGGAGGCCTTCAAGTGCGAAGAGTGTGGCAAGCACTA
CAACACCAAGCTGGGATACAAGCGCCATGTGGCCATGCACTCGGCCACAG
CGGGGGACCTCACCTGCAAAGTGTGCATGCAGAGCTACGAGAGCACCCCG
GTGCTCCTGGAGCACCTCAAGAGCCACTCGGGGAAGTCCTCGGGGGGCGC
CAAGGAGAAGAAGCACCCGTGCGACCACTGCGACCGCCGCTTCTACACGC
GGAAGGACGTCCGGCGGCACATGGTCGTCCACACGGGCCGGAAGGACTTC
CTGTGCCAGTACTGTGCCCAGCGCTTCGGCAGGAAGGATCACCTGACGCG
GCACGTGAAAAAGAGCCACTCGCAGGAGCTGCTGAAGATTAAGACGGAGC
CTCCGGATATGCTGGGTCTCCTGGGCTCCGGGTCGCCGCCTTGCTCCGTC
AAGGAGGAGCTGAGCCCCATGATGTGCAGCATGGGTCCCAATAAAGACCC
CATGATGGGCAAACCCTTTCCCAGCGGGACCCCCTTCCCCATGGGCATGT
ACAACCCCCACCAC------CTCCAGGCCATGTCTAATCCCGGGGTGGGA
CACCCC------CATCCCTCCCTTATGCCCGGTTCCCTGTCAGCAGCTAT
GGGCATGGGCTGTCATATGGAA----------------------------

------------------------------------GGCTACCACACATT
TGAGTGGCAGCCAGCCCTGAAGAACGTGTCCCCGTCCTGCCATGTGGGCA
TCATCAATGGGCTTTCCGGCTGGGCCACCTCGGTGGATGACGTCCCCGCT
GACACCATCACCCGCCGGTTTCGCTATGATGTGGCGCTTGTATCAGCCCT
GAAGGACCTGGAGGAGGACATCATGGAAGGGCTGAGGGAGTGCGGGCTGG
AGGACAGCGCTTGCACGTCAGACTTCAGTGTTATGATCAAGGAATCCTGT
GACGGCATGGGGGATGTCAGCGAGAAGCACGGCGGGGGGCCACCGGTGCC
CGAGAAGGCCGTGCGTTTCTCTTTCACCGTCATGTCCGTCTCCGTCCAGG
CTGACGGAGAAGAG------------------------------GAAGAG
GTCGTCGTCTTCAGGGAGCCAAAGCCCAACTCTGAGCTGTCCTGTAAGCC
CCTGTGCCTGATGTTTGTGGACGAGTCTGACCACGAGACGCTCACCGCCG
TCCTGGGGCCTTTGATTGCGGAGAGGAGTGCGATGAAGGAAAGCCGACTC
ATCCTCCCGGTGGGAGGCCTCCCCCGCTCCTTCCGCTTCCACTTCAGAGG
CACGGGCTACGACGAGAAGATGGTGCGAGAGATGGAGGGCCTGGAGGCCT
CGGGCTCCACCTATATCTGCACTCTTTGCGACTCCACTCGGGCAGAAGCC
TCTCACAACATGGTGCTCCACTCCGTCACTCGCAGCCACGAGGAGAACCT
GGAGCGCTACGAGATATGGAGGACCAACCCCTTCTCGGAGTCTGCCACGG
AGCTGCGAGATCGGGTCAAAGGGGTCTCTGCCAAACCGTTTGTCGAGACC
CATCCGACCATGGATGCGTTACACTGTGATATAGGTAATGCCACCGAATT
CTACAAAATCTTCCAAGATGAGATTGGGGAGGTGTTCCAGAAGGC---CA
AC---CCGAGCCGGGAGGAGCGGCGCAGCTGGAGGGCAGCCCTCGATAAG
CAGCTGAGGAAGAAGATGAAGCTGAAACCGGTGATGCGGATGAATGGAAA
CTATGCCCGAAAGCTGATGACCGAGGAGGCCACAGAGGCGGTGTGTGAAC
TGGTGCCCTCAGAAGAGAGACGGGAGGCCCTGAGGGAGCTGATGAGGCTC
TATATCCAGATGAAGCCGGTGTGGCGTGCTACCTGCCCAGCCAAGGAGTG
CCCTGACCAGCTGTGCCGCTACAGCTTTAACTCTCAGCGCTTTGCTGATC
TCCTCTCCTCCACCTTCAAATACAGATATGATGGGAAGATCACCAACTAC
CTGCACAAGACCCTGGCCCATGTCCCTGAAATCATAGAGAGAGATGGCGC
CATCGGAGCCTGGGCCAGCGAGGGGAATGAGTCTGCAAACAAA-------

-----------------------AAACGGCGTTCTCCTCGCAGGCG---C
CCGGCTACGCGGGGGCCGCCGCCCTCGGA---CACCACCA------CCAC
CCGACGCACGTGAGCTCC---TACTCCACGGCGGCGTTCAACTCCACGCG
GGACTTCCTCTTCCGCAACCGGGGCTTCGGCGACGCTACGAGCGCCGCCG
CCGCCGCGCAACACARCCTCTTCGCCTCGGC------GGGGAGCTTCGCC
------GCGGGGCCCCACGGCCACGGCGATGCCGCGGGACACCTGCTCTT
CCCGGGGCTCCACGAG---CAGACGG---GCCACGCGTCGTCCAACG---
------GGCAGATGCGGCTCGGTTTCACCGGGGACATGTACGGCCGCGCC
GACCAGTACGGCCACGTCGCGAGCCCGCGGT---CCGACCATTACGCGTC
GACCCAKYTGCACGGCTACGGCCCCATGAACATGAACATGGCCGCG---C
ACCACGGCGCGGGGGCCTTCTTTCGGTACATGCGGCAGCCGATCAAGCAG
GAGCTCATCTGCAAGTGGATCGAGCCGGAGCAGCTGTCGAACCCGAAGAA
GTCGTGCAACAAAACTTTCAGCACCATGCACGAGCTCGTGACCCACCTGA
CGGTGGAGCACGTGGGGGGGCCCGAGCAGTCGAACCACATATGCTTCTGG
GAGGACTG--

>Leuroglossus stilbius
AGCCTCTTAATCCGAGCTGAACTAAGCCAACCGGGAGCCCTTATAGGGGA
CGACCAAATTTACAACGTTATTGTGACAGCACATGCTTTCGTCATAATCT
TTTTTATAGTGATGCCAGTTATAATCGGCGGCTTTGGAAACTGACTAATC
CCTCTTATGATCGGAGCCCCCGACATAGCCTTCCCTCGAATAAATAATAT
AAGCTTCTGGCTTCTTCCTCCCTCATTCCTTCTCCTCCTAGCCTCTTCCG

GCGTAGAAGCCGGAGCAGGGACAGGATGAACAGTTTACCCTCCTCTAGCA
AGCAACCTTGCCCATGCAGGAGCTTCCGTAGACCTAACTATTTTCTCCCT
TCACCTCGCTGGGATTTCCTCAATTCTTGGGGCAATCAACTTCATCACAA
CCATTATCAACATGAAACCCCCAGCTATCTCCCAGTACCAAACACCCCTT
TTTATCTGGGCTGTCCTTATTACAGCTGTGCTCCTTCTCCTCTCCCTTCC
CGTCCTAGCCGCAGGAATTACAATACTACTTACAGACCGAAACCTAAATA
CCACCTTCTTTGACCCCGCTGGAGGGGGAGACCCCATTCTTTATCAACAT
CTCTTCTGATTCTTCGGCCACCCCGAAGTCTACATTTTGATTCTTCCAGG
CTTCGGCATGATCTCCCACGTCGTCGCCTACTACGCGGGCAAAAAAGAAC
CCTTCGGCTACATGGGCATGGTCTGAGCAATGATGGCTATTGGACTTCTA
GGCTTCATCGTTTGAGCCCACCACATGTTTACAGTCGGAATGGACGTTGA
CACTCGTGCCTATTCTATGAGAGAAATCTGCACCCGTCTAACTGCTTGGG
CATGCTGCTGCTGTCMGACGCCCACACCTGCACCAAGCTCTMCGAGCTYT
CCTGGGGCATGTGWCTCAGCAACTTCCCTGCCATCTGCAAGACGGAGGAC
TTCCTGCAGCTGCCCAAGGACATGGTGGTGCAGCTGCTGTCCCACGAGGA
GCTGGAGACGGAGGACRARAGGCTGGTGTACGAGGCGGCTCTCAGCTGGG
TCAACTACGACCTGGAGAGGAGGCACTGCCAGCTGCCCGAGATGCTGAGG
ACAGTCCGCCTAGCCCTGCTGCCCGCCATCTTCCTCATGGAGAACGTATC
GACGGAAGAGCTGATCAACGCCCAGGTCAAGAGCAAGGAGCTGGTGGACG
AGGCTATCCGCTGCAAGCTGAGAATCCTGCAGAACGAAGGCGTGGTCAAC
AGCCTGTGCGCCCGGCCCAGGAAGACCAGCCACGCCCTGTTCCTGCTCGG
TGGCCAGACCTTCATGTGTGACAAGCTGTACCTGGTGGACCAGAAGGCCA
AGGAGATCATCCCCAAGGCAGACATCCCAAGCCCCAGAAAGGAGTTCAGY
GCCTGTGCCATCGGCTGCAAGGTCTACGTCACCGGGGGAA--GAGGCTC-
GGAGAACGGGGTGTCCAAAGACGTGTGGGTYTAMGACACCATCCAGGAGG
AGTGGTCCAAGGCGGCGCCCATGCTCATCGCCAGGTTCGGCCACGGCTCT
GCAGAGCTGAAACACTGCCTCTACGTSGTGGGGGGGCACACAGCTGCCAC
GGGC---------------------GGAAGAGTACATAGTGGTGTTCAGC
CGTTCCATTAACAGGCTGATTCTTAACGAGGCAGAGCTCATTCTGGCCCT
AGCACAGGAGTTCCAGATGAAGGTTGTGACTGTGTCTCTGGAGGAGCAAT
CCTTTGCTGCTATAGTGAAGGTGGTCAGTGAGGCCACCATGTTAGTCAGT
ATGCACGGAGCCCAACTGGTGTCCTCTCTTTTCCTGCCAAGAGGGGCCGC
AGTGGTTGAGCTTTTCCCCTATGCAGTGAACCCAGAGCAGTACGCTCCCT
ACAAAACCTTAGCCTCACTCCCTGGCATGGACTTGCAGTATGTGGCCTGG
AGGAACACGATAGAGGAGAACTCGCTGGCCTACCCCGAGAGGCCCTGGGA
GCAGGGGGGCATCACCCACTTGGAGAAGGACGAGCAAGATCGCATCCTGG
CCAGCAAAGAGGTACCGAGGCACCTGTGCTGTCGCAACCCAGAGTGGCTC
TACCGTATCTACCAGGACACCATAGTGGACATCCCGTCTTTACTGGACAC
CCTCAG---AGAGACCCTGAAA---ACCAGGCCCAACCTGAAGAA---GG
CCAAGCCTGCCAGCACGGTTCACCCGGGCCGGGTGAGAGAGCCCCAGTGC
CAGACCTCAGTCCAGGCCACCAACGAGGCCAAGCTCACAGTGTCCTGGCA
GATCCCCTGGAACCTGAAGTACCTGAAGGTCCGGGAGGGAAATA------
-----------AAGAGGGATGCAAGCAAGGGAACCCTAGAGGATCAAATC
ATCCAGGCTAACCCTGCACTCGAGGCTTTTGGTAACGCCAAAACATTGAG
AAATGATAATTCATCCCGTTTTGGCAAATTCATCCGTATTCATTTCGGAC
AAAGTGGCAAATTGTCCTCTGCAGATATAGAGACGTATCTGCTTGAGAAG
TCACGTTGTACCTTTCAGCTGAAATCAGAGAGGAACTACCATATCTTCTT
TCAGATCTTGTCCAATGAAAAGCCAGAGCTGTTGGACATGCTGTTGATCA
CCAACAATCCATATGACTACGCCTACATCTCCCAAGGAGAGGTGACAGTA
GCATCCATCAACGATTCTGAGGAGCTGATAGCCACTGACAGTGCTTTTGA
TGTGCTGGGCTTTACTGCAGAGGAGAAAATGTCGGTCTACAAACTGACAG
GTGCCATAATGCACTATGGCAACATGAGGTTCAAGAACAAGCAGCGGGAG
GAGCAGGCAGAGCCTGACGGTACAGAGGCTGCTGACAAGTCAGCTTATCT

GATGGGACTGAACTCTGCAGATCTCATTAAAGGACTCTGCCATCCAAGAG
TCAAGGTTGGCAATGAGTATGTCACCAAAGGTCAGAGTGTCGATCAAGTC
TACTACCCCAACAAGGAGGCCTTCAAGTGCGAGGAGTGTGGCAAGCACTA
CAATACCAAGCTGGGATACAAGCGCCATGTGGCCATGCACTCAGCCACGG
CGGGGGACCTCACCTGCAAAGTGTGCATGCAGAGCTACGAGAGCACGCCC
GTGCTCTTGGAGCACCTCAAGAGCCACACGGGGAAGTCGTCAGGAGGCGC
CAAGGAGAAGAAGCATCCATGCGACCACTGCGATCGCCGCTTCTACACAC
GGAAGGACGTCCGGCGGCACATGGTGGTGCACACGGGCCGGAAGGACTTC
CTGTGCCAGTACTGCGCGCAGCGCTTCGGCCGCAAGGACCACCTGACGCG
CCATGTGAAGAAGAGCCACTCACAGGAGCTGCTAAAGATCAAGACGGAGC
CTCCAGACATGCTGGGCCTGCTGGGCTCTGGCTCGCCGCCCTGCTCCATC
AAGGAGGAGCTCAGCCCCATGATGTGCAGCATGGGTCCCAACAAGGACCC
CATGATGGGCAAACCCTTCCCCAGCGGGACCCCTTTCCCCATGGGCATGT
ACAACCCCCACCAC------CTCCAGGCCATGTCCAGTCCAGGGGTGGGG
CACCCC------CACCCCTCCCTGATGCCCAGCTCTTTGTCAGCGGCCAT
GGGCATGGGCTGCCACATGGAGTACCTGATCTACGCTTCCTTCTCCTTCA
TGGGATGTTTACAAATCAGCGATGGGTCAAACATCGTGAACCTGATGGCG
AGTAACTCTCCGAGCGTGTCTTTCGCGCTCACCCAGCAGAAGTACTTCAG
TAACTACAGCCCCGTTATTGGGTTCTACATTTACGAACCCATCGAGTACT
GGAACGCCACGGTGCAGGAGCACCTGAAGACGCTCAGTCACGGCTTCAAC
AAGATCTCCTGGATCGACAACTTCTTCCACTACCTGCGGGTGGTGAACGT
GAGCGCGTCGACCAAGAGCGACTTCATCAGCATCCTGAAGGGCTCCTTCC
TGCGCAGCCCGGAGTACCAGCACTTCAACGAGGACATCATCTTCTCCAAG
A---ATCGCGAGAGCG------ACGAGTACGACATCATCGCCTCGCGCAT
GTACCTGGTGGCGCGCACCACCGAGAAGAAGCGCGAGGAGGTGGTGGAGC
TGCTGGAGAAGCTGCGTCCGCTGATGCTGATCAACAGCATCAAGTTCATC
GCCTTCAACCCCACCTTCGTCTTCATGGACCGCTACAGCTCCTCGGTCAT
CTCGCCCATCCTTACCTCAGGCTTCAGCGTGCTCACCATCCTCATCCTCA
CGTTCTTTCTCGTCATCAACCCGCTGGGGAACTTCTGGCTCATCCTGACG
GTCACGTCCGTGGAGCTGGGCGTGCTGGGTCTGATGGGCTACCACCCCTT
CGAGTGGCAGCCGGCCCTCAAGAATGTGACCACGTCATGCCATGTGGGCA
TCATGAATGGGCTCGCAGGATGGACAGCCTCAGTGGATGATGCCCCTGCR
GATACCATAACCCGGCGGTACCGCTATGACGTGGCCCTGGTGTCGGCCCT
GAAGGATCTGGAAGAGGACATCATGGATGGGCTACGGGAGCGAGGGCTGG
ACGACAGCGCCTGCACCTCTGGCTTCAATGTGATGATCAAGGAGTCCTGC
GATGGCATGGGGGACGTCAGTGAGAAACATGGTGGTGGGCCGCCGGTCCC
CGAGAAGGCTGTGCGCTTCTCCATTACCGTCATGTCTGTCTCTGTCCAGG
CTGAAGGAGAGGAG------------------------------GAGGCG
GTCACCATCTTCCGGGAGCCCAAGCCCAACTCTGAACTGTCCTGTAAACC
ACTGTGCTTGATGCTTGTCGATGAATCGGACCACGAGACACTCACGGCAG
TCCTAGGGCCCTTGGTGGCCGAGCGGAATGCTATGAAGCACAGCCGGCTC
ATTCTGTCCCTGGGTGGGCTCCCTCGCTCCATCCGCTTCCACTTCAGAGG
AACGGGTTACGATGAGAAGATGGTGCGTGAGATGGAGGGCCTGGAGGCCT
CTGGCTCCACCTATATCTGCACCTTGTGTGACTCCACCAGGGCAGAGGCC
TCCCACAACATGGTGCTCCACTCCATCACCCGCAGCCACGATGAAAACCT
GGAGCGCTACGAGTTGTGGAGGACCAACCCCTTCTCGGAGTCCGCAGAGG
AGCTGCGGGACCGGGTCAAAGGTGTCTCTGCCAAGCCCTTTATGGAGACC
CAGCCCACACTGGATGCCCTGCACTGCGACATCGGCAATGCCACTGAGTT
CTACAAGATCTTCCAGGACGAGATCGGGGAGGTGTACCGCAGCCC---CA
AC---CCCAGGCGAGAAGAGAGACGGAGCTGGAGGGCCACTCTGGACAAG
CAGCTGAGGAAGAAGATGAAGTTGAGGCCGGTGATGCGAATGAACGGGAA
CTATGCGCGTCGGCTGATGACCCGGCAGGCGGTAGAGGTGGTGTGTGAGC
TGGTGCCGTCAGAAGAGAGGCGAGAGGCCCTGAGGGAGCTGATGGGGCTC

TACCTTCAAATGAAGCCAGTGTGGCGCTCTTCCTGCCCAGCCACTGAGTG
CCCAGACCAGATGTGTCGCTATAGCTTCAACTCCCAGCGCTTCGCAGAGC
TCCTCTCCAGCACCTTCAAGTACAGGTA----------------------

---TCCTACA
CCATCGAGATGGGCACCAAAGGGCCGCAGTGGAAAGAGAGTCCCCAGCCG
TTCTCCTGCTCCATCGAGGACCCCACCAAGCAGACCAAGTTTAAAGGCAT
CAAGACGTACATTTCGTACCGGGTCACCCCGAGCCACATGGGGCGGCCTG
TGTACCGGCGCTACAAGCACTTTGACTGGCTGTACAATCGCCTGCTGCAC
AAGTTCACCGTCATCTCCGTGCCTCACCTGCCGGAGAAGCAGGCCACAGG
GCGCTTCGAGGATGACTTCGTCGAGAAGCGCAAGAGGCGGCTGGTTCTCT
GGATCAACCACATGACCAGCCACCCGGTCCTGTCTCAGTACGAAGGCTTC
GAGCACTTCCTCATGTGCGCCGACGACAAGCAGTGGAAGCTGGGGAAGCG
GCGGGCGGAGAAGGACGAGATGGTGGGCGCCCACTTCATGCTGACGTTCC
AGATTCCCACGGAGCACCAGGACCTGCAGGACGTGGAGGAACGCGTGGAC
AACTTCAAGTCCTTCGCCAAGAAGATGGACGACAGCGTGTTGCAGCTGAC
GCACGTGGCGTCGGAACTGGTGCGGAAGCACGTCGGAGGGTTCAGGAAGG
AGTTTCAGCGGCTGGGGAACGCCTTCCAGTCGGTCAGCCAGGCCTTCATG
CTGGATCCTCCGCACAGCTCGGACGCCCTCAACAACGCCATCTCTCAT--

-----------------------------------GCCAAATCTCGCTTT
CACCCTGGCGTAGGACTGGTCCTGGCACAGACC---GCAGCGTCCCACT
TAGTAACAGCTTGCTGTCCCCGCAACAAACCGAAGAGCACACAGTTG---
CTTCCCCGCAGCGATGGTTTGTCACCC---CTGCCAACAACCGACTGGAC
TTCGCCGCCTCGGCATACGACGCCGCCGCGGCTGCTGATTTCGCCGGTAA
CGCGGCCACCTTGCTGTCTTACGCAGCTGCTGGAGTAAAGGCCC------
TTCCCCTGCCCACTGCTGGCTGCTCCAACAGACCGTTGGGTTATTATACC
GACCCATCCG---GCTGG---GGCACGCGCACACCACCTCAGTACTGC--
-------AGTAAATCCAGCTCGGTGCTTTCATGCTGGCCCACAAATGCGC
TTGGAAGCCGAACAGGCA---CGTCCAATTACCTGC---------CGGAA
GA---CGGG---GACCC---CATCCCCACGGAGAGGTCTCCG---AT---
CGGCGTGCCAGAGGAG---ACCAAACCAAAAGACTT------GTCCGA--
-GTCCAGCTGGATAGAG---ACGTCGTCTTCAATAAAGTCGATAGATTCA
AGTGATTCTGGGATCTTTG---AGCAGGCCAAACGGAGAAGAATTTCACC
GTCTGCCACACCG---------GTNTCAGAGACAGTTTCCCCGCTGAAATC

AGAGCATCACTCAACAGGCGAAGTCACAGACAGAGAAGTGGCTTTGGGGA
TAAATCCGTTCGCCGACGGGATGGGAGCGTTCAAAATCAACCACAGCTCT
CATGATCTCGGCTCCGG---GCAAACGGCGTTTTCCTCCCAAGCT---CC
GGGCTAC---GCGGCCGCCGCCTTGGGA---CACCACCA------CCACC
CGACCCATGTCAGCTCC---TACTCCACCGCCGCGTTCAACTCCACCCGG
GACTTTCTTTTTCGAAATCGGGGCTTCGGAGACGCGACCAG---------
---CGCCCAACACAGCCTGTTCGCCTCCGC---AGCGGGAAGTTT---C-
-----GCAGGGCCCCACGGACACTCCGATGCGGCGGGACACCTGCTCTTC
CCCGGACTGCACGAG---CAAGCCGCGAGCCACGCGTCCTCCAACGTTGT
AAACAGTCAGATGCGCTTGGGCTTTTCGGGGGACATGTACGGGCGGGCCG
ACCAGTACGGCCATGTGACGAGCCCGCGGT---CCGACCACTATGCGTCG
ACCCAGTTGCACGGTTATGGCCCCATGAACATGAATATGGCCGCA---CA
CCACGGAGCAGGGGCCTTCTTCCGTTACATGAGGCAGCCGATCAAACAAG
AGCTCATCTGCAAGTGGGTCGAGCCGGAGCAGTTGTCGAACCCGAAAAAG
TCGTGCAACAAAACTTTCAGCACGATGCATGAGCTGGTGACCCACCTGAC
CGTGGAACATGTGGGGGGACCGGAGCAGTCGAACCATATTTGCTTTTGGG
AAGAGTGCTGCCGAGAAGGAAAACCCTTCAAAGCTAAATACAAACTTGTA
AATCACATCAGAGTACACACCGGAGAGAAACCGTTCCCGTGTCCATTCCC
CGGCTGTGGCAAA
>Liparis mucosus
AGCTTATTAATTCGGGCCGAACTAAGCCAACCCGGAGCCCTTTTAGGCGA
CGACCAAATCTACAACGTAATTGTTACAGCACATGCTTTCGTAATAATTT
TCTTTATGGTAATACCAATTATAATTGGAGGTTTTGGGAATTGACTCATC
CCCTTAATGATCGGGGCCCCCGATATAGCCTTTCCTCGAATAAATAACAT
AAGTTTTTGACTTCTCCCTCCCTCGTTCTTGCTCCTCCTTGCCTCTTCTG
GTGTAGAAGCGGGGGCCGGAACCGGATGAACTGTTTACCCCCCTCTTGCT
GGTAACTTGGCCCACGCTGGAGCTTCTGTAGACCTAACAATTTTCTCACT
GCATTTAGCAGGAATTTCTTCAATTCTTGGGGCAATTAATTTTATTACAA
CCATCATCAATATAAAACCCCCTGCTATATCTCAGTACCAAACTCCCCTT
TTCGTTTGATCTGTGCTCATTACAGCCGTACTTCTCCTCCTCTCTCTCCC
TGTTCTTGCTGCCGGCATCACCATGCTTCTGACAGACCGAAATCTTAACA
CAACCTTCTTTGACCCTGCAGGGGGAGGAGACCCAATTCTTTATCAACAC
CTCTTCTGATTCTTCGG---------------------------------

-------------------------GGACGGCTACGTTGTCGTGTTCAGC

CGCTCCGAGACGCGGCTGATCCTGAATGAGGCCGAGCTAATMATGGCACT
GGCCCAGGAGTTCCAGATGAGAGTGGTCACGGTGTCCCTGGAGGAGCAGT
CCTTCCCCAGCATCGTCCAGGTGATCAGCGGTGCGTCCGTGTTGGTCAGC
ATGCACGGCGCCCAGCTCATCACCTCGCTCTTCCTCCCCAGAGGAGCGAC
GGTGGTGGAGCTGTTCCCCTTTGCCGTGAACCCAGAGCAGTACACCCCGT
ATAAAACCCTTGCCACCCTTCCTGGCATGGACCTTCACTATATCTCCTGG
AGGAACTCTCGGGAGGAGAACACCATCACCCACCCTGACCGAGCCTGGGA
GCAAGGGGGCATCGCTCACTTGGACAAGGAGGAGCAAGAGCGCATTCTGG
CGAGCAAAGATGTGCCCAGGCACCTGTGCTGCCGCAACCCGGAGTGGCTG
TTCCGGATCTACCAGGACACGTTGGTGGACATCCCTTCCTTCCTGGWAAC
CCTGAA---AGAGGGCATGAAG---ACAAAGCCCAGCGTGAAGAA---GT
CAAAGCCAGCCAGCACAGTCCACCCGGGCCGGGTCCGGGAGCCCCGGTGT
CAGACCTCGGTCCAAACCACCAACGAAGCCCGGCTCACGGTCTCCTGGCA
GATCCCGTGGAATCTGAAGTACTTGAAGGTGCGAGAGGTGAAGTACGAGG
TGTGGATCCAGAAGAGAGACCCGAGCAAGGGGACGCTGGAGGACCAGATC
ATCCAGGCCAACCCGGCGCTGGAGGCCTTCGGCAACGCCAAAACGCTGAG
GAACGACAACTCGTCTCGTTTTGGAAAGTTCATCCGCATTCACTTCGGGA
TGAGCGGGAAGCTGTCGTCGGCCGACATCGAGACCTACCTGCTGGAGAAG
TCCCGCTGCACCTTCCAGCTCAAGGCTGAGAGGAACTACCACATCTTCTA
CCAGATCCTGTCCAATCAGAAGCCAGAGCTGCTGGACATGCTGCTGATCA
CCAACAACCCGTACGACTACTCCTACATCTCCCAAGGGGAGGTCACCGTC
GCCTCCATCAACGACGCGGAGGAGCTGCTGGCCACCGACTGCGCCTTCGA
CGTGCTGGGCTTCACCGCAGAGGAGAAGATGGGCATCTATAAGCTCACCG
GCGCCATCATGCACTTCGGCAACATGAARTTCAAACAGAAGCAGCGCGAG
GAGCAGGCGGAGCCCGACGGCACGGAGGCGGCCGATAAGACGGCGTACCT
CATGGGCCTGAACTCCGCCGACATCATCAAGGGGCTGTGCCACCCCAGGG
TCAAGGTGGGGAACGAGTACGTCACCAAAGGCCAGAGTGTGGACCAGGTG
TACTAC--

----------------------TATCTCATCTACGCCTCTTTCTCATTCA
TGGGATGTTTACAGATCAGTGATGGATCAAATATAGTGAACCTGCTGGCT
AGTAACTCCCCGAGTGTGTCTTACGCTCTGACGCAGCAGAAGTACTTCAG
CAACTACAGTCCCGTGATTGGGTTCTACATTTATGAGCCCATCGAGTACT
GGAACTCCACGGTGCAGGAGCATCTGAAGACTCTGAGTCATGGCTTCAAC
AAGATCTCTTGGATGGACAACTTTTTCCACTACCTGCGTGTGGTGAACGT
GAGCGCATCAACCAAGAGCGATTTCATCACTATCCTCAAGGGCTCCTTCC
TTCGCAGCCCGGAGTACCAGCACTTCACTGAGGACATCATATTCTCCAAG
A---ACCGTGAGACTG------ACGAGTACGACATCATCGCCTCGCGGAT
ATACCTGGTGGCACGAACAACAGAGAAAAAACGTGAGGAGGTGGTGGAGC
TTCTAGAAAAGCTTCGGCCCTTAATGCTGATCAACAGCATTAAGTTCATC
GCCTACAATCCTACGTTTGTGTTCATGGACCGCTACAGCTCCTCTGTCAT
CTCGCCCATCCTGACCTCAGGGTTCAGCGTACTCACAATCCTTATCCTCA

```
CTTTCTTCCTGGTCATCAACCCGTTGGGGAACTTTTGGCTCATCCTCACG
GTAACCTCCGTAGAGCTGGGCGTCCTGGGTTTGATGGGCTTTCACCAGTT
TGAATGGCAGCCAGCTCTCAAGAATGTGTCTACATCTTGCAGTGTTGGCA
TTATTAATGGGCTCTCTGGATGGGCTTCCTCGGTGGATGATTTCCCAGCT
GACACCATCACTAGACGGTTCCGCTACGATGTGGCTCTGGTGTCAGCGTT
GAAGGATCTGGAGGAGGACATCTTGGAGGGGCTGAGAGAGAGTGGGATGG
AAGACAGTGCTTGCACCTCGTGCTTCAGTGTCAAGATCAAGGAATGCTGT
GATGGTATGGGCGATGTCAACGAGAAGCACGGTGGAGGACCAGCTGTTCC
TGAGAAGGTTGTGCGTTTCTCTTTCACTGTTATGTCGGTCTCTGTCCTGG
CAGACGATGAGGAG------------------------------GACGAC
GTTACCATCTTCACTGAGCCAAAGCCAAACTCAGAACTGTCCTGTAAGCC
CCTGTGCCTGATGTTTGTGGATGAGTCAGACCATGAGACACTCACAGCCG
TCCTGGGGCCTGTAGTTGCAGAGCGTAATGCAATGAAAGACAGCAACCTC
ATCCTGTCCGTGGGCGGACTTCCTCGGTGCTTCCGCTTTCAATTCAGAGG
CACAGGATATGATGAGAAGATGGTGCGCCAGGTCGAGGGCATGGAGGCCT
CAGGGTCCACCTACGTCTGCACACTTTGTGACTCCACCCGGGCACAGGCC
TCTCAAAACATGGTGCAACACTCCATCACTCGCAGTCATGAAGAGAACCT
CGATCGTTACGAAATATGGAGAACCAACCCCTTTTCTGAGTCTGTCGATG
AGCTGCGCGAAAGGGTTAAAGGATCTCTGCCAAGCCCTTCCTGGAGACC
CAGCCGACGATGGATGCATTACATTGTGACATCGGCAATGCCACTGAGTT
CTACAAAATCTTCCAGGACGAGATCGGAGAGGTATACCAAAAGGT---CA
AC---CCCAGCCGGGTGGAACGGCGCAGCTGGAGGGCAGCGCTGGATAAA
CAGCTTAGGAAGAAAATGAAGCTTAAACCAGTGGTGAGGATAAATGGGAA
CTATGCCCGCAGGCTTATGACTCAAGAGGCTGTGGAGGTGATCTGTGAGC
TGGTGCCCTCAGAGGAGAGGAGGGAGGCTCTGAGGGAGCTGATGAGGATC
TACATCCAGATGAAGCCGGTGTGGCGCGCCACCTGCCCTGCCAAGGAGTG
TCCCGACCAGCTGTGCCGCTACAACTTTAATGCCCAGCGCTTTGCCGACC
TTCTCTCCTCCACCTTCAAATACAGGTACAATGGAAAGATAACCAATTAC
CTGCACAAGACCCTGGCCCATGTGCCTGAAATCATAGAGAGAGATGGATC
TATCGGAGCCTGGGCCAGCGAAGGGAACGAGTCTGCAAACAAG-------
--------------------------------------------------
--------------------------------------------------
--------------------------------------------------
--------------------------------------------------
--------------------------------------------------
--------------------------------------------------
--------------------------------------------------
--------------------------------------------------
--------------------------------------------------
--------------------------------------------------
--------------------------------------------------
--------------------------------------------------
--------------------------------------------------
--------------------------------------------------
--------------------------------------------------
--------------------------------------------------
--------------------------------------------------
--------------------------------------------------
--------------------------------------------------
--------------------------------------------------
--------------------------------------------------
--------------------------------------------------
--------------------------------------------------
```


-----------------------------------NNNNNNNNNNNCTTTCACCCTGGCGT
GGGGACTGGTCCTGGCACGGAGC---GCAGCGTCCCACTCGGCCACAGCT
TGCTGTCCCCGCAGCAAACCGACGAGCCCAGTGTTGCCACCCCCCCGCAG
MGATGGTTTGTCACCC---CTGCCAACAACCGACTGGACTTTGCTGCCTC
GGCATACGACGCCGCT---------GATTTCGCCGGTAACGCGGCCACCT
TGCTGTCCTACGCAGCGGCCGGAGTGAAGGCTC------TTCCCCTGCCG
ACTGCAGGCTGCTCCAACCGGGCGCTTGGCTATTACGCAGACCCGTCAG-
--GCTGG---GGAGGACGCACACCGCCGCAGTACTGCGGCGTAAACAACA
AATCCAGCTCGGTCTTTTCCTGCTGGCCCGCTAACTCTATCGGGGGCCGA
GCAGGTA---CC---AACTACCTGY---------CCGAGGA---AGGA--
-GACTC---CATCACGACAGAGAGGTCGCCG---AT---CGGCGGCTCGG
ACGAG---ACCAAACCCAAAGACATGAC---ATCAGA---GTCGAGCTGG
ATAGAG---ACGCCGTCCTCCATTAAGTCCATTGATTCGAGCGATTCTGG
TATCTTTG---AACAGGCCAAACGGAGAAGAATCTCACCTTCTGCCACGC
CG---------GTTTCAGAGACAGTGTCCCCGTTAAAGTCNNNNCATCACTCAA
CAGGCGAAGTCACAGAGAGAGAAGTAGCGTTGGGGATAAATCCGTTCGCG
GATGGGATGGGCGCCTTCAAAATAAACCACAGCTCCCACGATATCGGCTC
CGG---ACAAACGGCGTTTTCCTCCCAGGCG---CCCGGCTAC---GCGG
CAGCCGCCCTAGGA---CACCATCA------CCACCCGAGCCACGTTGGC
TCT---TACTCCACGGCGGCTTTCAACTCCACGAGGGACTTTCTCTTCAG
AAATCGGGGCTTCGGGGACGCCACCGG------------GGCGCAGCACA
GTTTGTTCGCCTC---------CGGAAGTTT---C------GCTGGGCCA
CATGGACACTCAGATGCAGCGGGGCACCTGCTCTTCCCGGGGCTCCACGA
G---CAAGCGGCGAGCCATGCGTCTTCCAACGTGGTCAACAGCCAGATGC
GGCTGGGCTTCTCGGGGGACATGTACGGACGGCCGACCAGTACGGCCAC
GTTACGAGCCCGCGGT---CCGACCACTACGCTTCGACCCAGCTGCACGG
CTATGGCCCCATGAACATGAATATGGCCGCA---CATCACGGAGCAGGGG
CCTTCTTTCGATACATGAGGCAGCCGATCAAGCAAGAGCTCATCTGCAAG
TGGATCGAGCCGGAGCAGCTGACGAATCCCAAAAAGTCGTGCAACAAAAC
TTTTAGCACGATGCACGAGCTTGTGACCCATCTGACGGTGGAGCATGTGG
GGGGACCGGAGCAGACCAACCACATCTGCTTCTGGGAGGAATGCGCCAGA
GAGGGAAAGCCATTCAAAGCCAAATACAAACTTGTAAATCATATTAGAGT
ACACACCGGAGAAAAGNNNNNNNNNNNNNNNNNNNNNNNNNNNNNNNNN
>Lophius americanus
AGCTTGCTCATTCGGGCTGAACTGAGCCAACCCGGCGCCCTCTTAGGGGA
TGACCAAATCTACAACGTTATTGTTACCGCTCACGCCTTTGTAATAATTT
TCTTTATGGTTATACCAATTATGATTGGAGGGTTTGGCAACTGACTTATC
CCCTTAATGATTGGGGCCCCGGACATAGCCTTCCCCCGAATAAACAACAT
AAGCTTCTGGCTACTTCCCCCTTCTTTCCTCCTGCTACTTGCATCTTCCG
GGGTTGAAGCCGGAGCAGGCACTGGATGAACCGTCTACCCCCCGTTGGCA
GGGAACCTTGCACATGCAGGAGCCTCTGTTGACCTAACTATTTTTTCCCT
CCACCTAGCAGGAATTTCTTCAATCCTAGGAGCAATCAACTTCATCACAA
CAATTATTAACATAAAACCCCCCACAATCTCCCAGTACCAGACACCTTTA

TTCGTTTGAGCTGTTTTAATCACAGCAGTTCTTTTACTCCTATCCCTACC
CGTGCTTGCCGCAGGCATTACCATGCTCTTAACAGACCGAAACTTAAACA
CCACCTTCTTCGACCCCACAGGAGGAGGGGACCCTATCCTATACCAACAC
TTGTTC--

------------TTCCTTGAGACAAACCTCCACCCGACAAATTGCCTTGG
CATGCTATTGTTGTCTGATGCCCACCAGTGCACCAAGTTGTCAGAGCTCT
CATGGGGTATGTGCCTCAGCAACTTTCCCACTATTTGCAAGACAGAGGAC
TTCCTCCAACTGCCGAAAGATATGGTGGTGCAGCTTTTGTCACATGAAGA
ACTAGAGACCGAAGATGAGAGACTTGTTTATGATGCTGCCCTGAATTGGA
TCAACTATGATCTGGAAAAGCGGCATAGCCACCTTCCAGAGCTCCTGAAA
ACAGTCCGTCTGGCTCTGCTACCCGCCATCTTTCTTATGGAGAACGTCTC
TACAGAAGAGCTGATAAACGCCCAGGCTAAGAGCAAAGAGTTAGTGGATG
AAGCTATTCGCTGCAAGCTGAAGATCCTGCAGAATGATGGTGTTGTTAAC
AGCCTGTGTGCTCGACCCAGAAAAACAANCCATGCGCTTTTTCTTCTGGGT
GGACAGACTTTTATGTGTGACAAGTTGTATCTGGTGGACCAAAAAGCCAA
AGAGATCATCCCCAAAGCTGACATTCCCAGCCCAAGGAAGGAGTTCAGTG
CCTGTGCCATCGGCTGTAAAGTGTACATCACTGGTGGGA--GAGGCTC-T
GAGAATGGCGTGTCCAAAGATGTATGGGTCTACGACACGGTCCATGAGGA
ATGGTCAAAGGCGGCACCCATGTTAATCGCAAGGTTTGGCCATGGCTCTG
CCGAGCTCAAGCACTGCCTTTATGTGGTAGGGGGTCACACTGCAGCAACG
GGCTGCCTCNNNNNNNNNNNNNNNAGATGAATGCATAGTTGTGTTCAGTCGCTCAACAACAAGAT
TGATACTGAATGAAGCTGAACTAATCATGGCGCTTGCCCAGGAGTTCGAG
ATGAGAGTGGTCACAGTGTCCCTGGAGGAGCAGTCCTTCTCCAGTATCAT
CCAGGTCATCAGCAGGGCCTCCATGTTGGTTAGTATGCATGGAGCTCAAC
TCATAACCTCGCTCTTCCTCCCTAGAGGAGCAGTTGTGGTCGAGCTGTTC
CCCTTTGCTGTGAACCCAGAGCAGTACACCCCGTATAAAACCCTTGCCTC
CTTACCGGGTATGGACCTTCATTATATCTCCTGGAAGAACACGAAGGAGG
AAAACACCATAACCCATCCAGACCGATCGTGGGAACAAGGTGGTATCGAT
CATTTGGAGAAGGAGGAACAAGAGCGAATAAAGATGAGCAGAGATGTCCC
CAGACATCTGTGCTGCCGCAACCCAGAGTGGCTTTTTCGGATCTACCAGG
ACACTTTGGTGGACATCCCTTCCTTCCTGGAAGTCCTCAA---AGAGGGC
ATGAAG---ACAAAGCCCAACTCGAAGAA---GTCAAAGCCGGCCAGTAC
ACTCCACCCGGGCAGAGTCAGACAACCCCAGTGTCAGACCTCAGTACAAA
CCAGTAATGAGGCTAAACTCACGGTGTCCTGGCAGATCCCGTGGAATCTG
AAATACCTGAAAGTCAGAGAAGTGAAGTACGAGGTGTGGATCCAGAAGAA
AGACGCCAGTAAGGGAACCCTGGAGGATCAAATCATCCAAGCTAACCCTG
CGCTGGAAGCCTTCGGTAATGCCAAAACACTAAGAAACGACAACTCCTCC
CGTTTTGGGAAGTTCATCCGAATACACTTTGGTCCAAGCGGCAAGCTGTC
ATCTGCTGACATCGAGACATACCTGCTGGAGAAGTCCCGAGTCACCTTTC
AGCTCAAGGCTGAGAGAAATTACCACATCTTCTACCAGATCCTGTCCAAT
CAGAAGCCAGAGCTGCTGGACATGCTCCTGATCACCAACAACCCATACGA
CTACTCCTACATCTCTCAAGGAGAGGTAACCGTTTCCTCAATCAACGACT
CTGAGGAGCTGATATCCACGGACAGCGCCTTCGACGTGCTTGGCTTCACA
GCAGATGAGAAGATGGGTGTCTACAAACTAACGGGGGCCATCATGCACTA
TGGAAATATGAAGTTCAAGCAGAAGCAACGCGAGGAGCAGGCTGAGCCCG
ACGGCACGGAGGCCGCTGACAAAACAGCTTACCTAATGGGGCTGAACTCT
GCTGACCTGATCAAGGGGCTGTGCCATCCCAGAGTCAAGGTGGGAAATGA
GTACGTAACCAAAGGCCAAAGTGTCGACCAAGTGTACTACCCCAACAAGG
AGGCCTTCAAATGTGAGGAGTGTGGGAAGCACTACAACACCAAGCTGGGA
TACAAGCGGCATGTTGCCATGCATTCCGCCACGGCGGGGGATCTCACCTG

TAAAGTGTGCATGCAGACCTACGAGAGCACGCCGGTGCTGCTGGAGCACC
TCAAGAGCCACTCTGGGAAGTCCTCAGGTGGCACCAAGGAGAAGAAACAC
CAGTGTGACCACTGTGATCGGCGTTTCTACACAAGGAAGGATGTGAGGCG
CCACATGGTGGTGCACACCGGCAGAAAGGACTTCCTCTGCCAGTACTGTG
CCCAACGCTTCGGCAGGAAGGACCATCTGACGCGGCACGTGAAAAAGAGC
CACTCACAGGAGCTGCTGAAGATCAAGACGGAGCCTCCTGACATGTTGGG
TCTTTTAGCTTCTGGGTCACCGCCGTGCTCGGTGAAGGAGGAGCTCAGCC
CCATGATGTGTGGCATGGGTCCCAACAAAGATCCCATGATGAGTAAACCA
TTTCCCAGCGGGGCCCCCTTCCCGATGGGAATGTATAACCATCACCAT--
----CTCCAGGCCATGTCGAATTCTGGGGTGGGCCACCCC------CACC
CATCCCTCATGCCCAGTTCCTTGTCTGCAGCTATGGGCATGGGCTGTCAC
ATGGAATACCTCATSTACGCCTCCTTCTCATTCATGGGATGTTTGCAGAT
CAGTGATGGATCCAACATTGTGAACCTGCTGGCCAGTAACTCTCCCAGCG
TCTCATATGCTCTGACTCAGCAGAAGTACTTTAGTAATTACAGTCCTGTG
ATCGGGTTCTACATTTACGAGCCCATTGAGTACTGGAACTCCACGGTGCA
GGAACACCTGAAGACTCTGAGTCATGGCTTCAACAAGATATCCTGGATGG
ACAACTTTTTCCACTACCTGCGGGTTGTTAATGTGAGCGCATCCACCAAG
AGCGACTTCATCTCCATCCTCAAGGGTTCCTTCCTGCGTAGCCCGGAGTA
CCAACACTTCACCGACGACATCATCTTCTCAAAGA---ACCGGGATACTG
------ATGAGTACGACATTATTGCCTCAAGGATGTACCTGGTGGCACGG
ACTACAGAGAAGAAGCGTGAGGAGGTGGTGGAGCTGCTGGAGAAGCTCCG
TCCACTGATGCTGATCAACAGCATCAAGTTCATTGCCTTCAATCCCACAT
TTGTGTTCATGGACCGCTACAGCTCCTCCGTCATCTCACCCATCCTGACC
TCAGGATTCAGCGTACTCACAATCCTCATCCTCACCTTCTTCCTAGTCAT
CAACCCTTTGGGTAACTTCTGGCTCATCCTCACTGTGACGTCCGTGGAGC
TGGGCGTCTTGGGTTTGATGNNNNNNNNNNNNNNNNNNNNNNNNNNNNNNNNNNNNNNNNNNNNNNNNNN
NNNNNNNNNNNNNNNGATTAACGGGCTTTCTGGAAGGGGTTCCTC
TCTGGATGACTCTCCAGCTAACACCATCACCCGGCGGTTTCGCTATGATG
CACCACTGGTATCAGCATTAAAAGATCTGGAGGAGGACATCATGGAAGGA
CTGAGAGAGAATGGAATTGAATACAGCACTTGCAACTCAGGCTTCAGAGT
CATGATCAAAGAATCTTGCGATGGCATGGGCGACATCAGCGAGAAGCATG
GTGGAGGACCAGCTGTCCCTGATAAAGCTGTGCGTTTCTCTTTCACTGTG
ATGTCTGTCTCTCTCCGGACAGGGAATGAGGAA-----------------
-------------GAGGAGGTAGCAATCTTCACAGAGGCAAAGCCAAACT
CAGAACTGTCCTGTAAGCCCCTTTGCCTAATGTTTGTGGATGAGTCAGAC
CATGAGACACTAACAGCCATCTTGCACCCTATAATTGCAGAGCGTAAGGC
GATGAAAGAGTCCAGACTCATCTTGAGCATGGGTGGACTACTTCGCTCTT
TCCAATTTCACTTCATAGGTACGGGGTATGATGAGAAGATGGTACGAGAG
GTTGAGGGCCTAGAGGCGTCTGGGTCCACCTACGTTTGCACTCTCTGTGA
CTCCAGCCGTGCAGAGGCCTGTAAGAACATGGTGCTACATTCCATCACCC
GCAGTCATGAAGAGAACTTAGAACGTTATGAAATGTGGAGAACCAACCCC
TTCTCTGAGTCTGTTGATGAGCTGCGAGACAGAGTCAAAGGGGTCTCTGC
CAAGCCCTTCATGGAAACCCATCCCACCCTCGATGCATTACATTGTGACA
TARGAAATGCCACCGAGTTCTACAAAATATTCCAGGATGAGATTGRGGAG
GTATACCAAAAGGG---AAAT---CCAAGCCGTGAGGAGCGGCGCAGCTG
GAGGGCAGCCCTAGATAAACAGCTGAGGAAGAAGATGAAACTCAAACCGG
TAATGAGGATGAATGGGAACTTTGCCCGCAAGCTAATGACCAAGGAGGCT
GTGGAGGTGGTGTGCGAGCTGGTGCCCTTAGAGGAGAGGAGGGAGGCCCT
GAGGGAGCTTATGAGCCTCTACCTCCAGATGAAGCATGTGTGGCGTGCCA
CCTTCCCAGTCAAGGAGTGCCCTGACCAGCTGTGCCGCTACAGCTTTAAC
TCCCATCGCTTTGCTGAACTCCTCTCCTCGACCTTCAAATATAGATACAA
TGGAAAGANNNNNNNNNNNNNNNNNNNNNNNNNNNNNNNNNNNNNNNNNNNNNNNNNNNNNNNNNNNNNN
NNNNNNNNNNNNNNNNNNNNNNNNNNNNNNNNNNNNNNNNNNTCTTACACCATCGAGATGGGTCCCTTAG

GGCCCCTGTGGAAG
GAGAGCCCGCAGCCTTTCTCCTGCTCCATTGAAGACCCCACAAAACAGAC
TAAGTTCAAGGGCATCAAGACCTACATTTCGTACCGGGTCACGCCGAGCC
ACACCGGCCATCCCGTCTACAGACGCTACAAACACTTTGACTGGCTGTAC
AACCGCCTACTGCACAAGTTCACCGTGATCTCGGTGCCTCACCTGCCGGA
GAAGCAGGCCACGGGGCGTTTTGAGGAGGACTTCATTGAAAAGCGCAAGA
GGCGACTGATACTGTGGATGAACCACATGACCAGTCACCCGGTCCTCTCC
CAGTACGAAGGTTTGGAGCACTTTCTCATGTGCGGCGACGACAAGCAGTG
GAAGCTGGGCAAGAGACGAGCGGAGAAGGATGAGATGGTGGGCGCCCATT
TCATGCTGACCCTCCAGATCCCTAGCGAGCACCAGGACCTTCAGGATGTC
GAGGAGCGGATCGACTCCTTCAAGTCCTTTGCTAAAAAAATGGATGACAG
CGTGATGCAGCTCACGCACGTTGCTTCGGAGCTGGTGCGGAAACACCTGG
GCGGGTTCAGGAAGGAGTTCCAGAGGCTGGGGAACGCCTTCCAGTCTATC
AGCCAGGCGTTCATGCTGGACCCTCCCCACAGTTCCGASACCTTCAACAA
CGCCATCTCCCACNNNNNNNNNNNNNNNNCTCAAACTGACCTCTCTGGGTTTCATCATTGGAGTCG
GCGTGGTCGGAAACCTCCTGATCTCCATCCTGCTGGTTAAAGACAAAAGC
CTGCACCGAGCACCCTACTATTTCCTGCTGGACCTGTGCGCCTCCGACAT
CCTGCGCTCCGCCATCTGCTTCCCCTTCGTCTTCACCTCGGTCAAGAATG
GATCCGCTTGGACGTACGGCACGCTGACCTGTAAGGTGATCGCCTTCCTG
GGTGTGCTCTCCTGTTTCCACACGGCGTTTATGCTATTCTGCGTCAGCGT
CACGCGCTACCTGGCCATCGCACATCACCGCTTCTACACCAAGAGGCTGA
CCTTCTGGACCTGCCTAGCTGTCATCTGCATGGTGTGGACGTTGTCGGTG
GCTATGGCGTTCCCGCCAGTGCTAGACGTAGGGACGTACTCTTTCATACG
GGAGGAGGACCAGTGCACCTTCCAGCACCGCTCTTTCAGGGCCAACGATT
CGCTAGGCTTCATGCTCCTGCTGGCGCTCATCCTCCTGGCCACGCAGCTG
GTTTACCTCAAGCTCATCTTCTTCGTCCACGACCGCAGGAAGATGAAGCC
TGTCCAGTTTGTGCCTGCCGTCAGCCAGAACTGGACCTTCCACGGACCGG
GGGCCAGCGGCCAGGCGGCGGCCAACTGGCTGGCCGGTTTCGGTCGAGGC
CCCACCCCGCCTACCTTGCTAGGAATCCGTCAGAACAGCAACGCAGCGGG
CCGCAGGCGTCTACTGGTGTTGGATGAGTTCAAAACAGAGAAGAGGATTA
GTAGGATGTTCTACATCATGACGTTTTTCTTCCTGGCCCTCTGGGGGCCC
TATCTGGTCGCCTGCTACTGGCGGGTGTTTGCCAGGGGCCCTGTGGTCCC
GGGAGGCTACCTGACGGCCGCCGTGTGGATGAGCTTCGCCCAGGCTGGGG
TCAATCCGTTCATCTGCATCTTCTCTAACNNNNNNGCCAAATCTCGCTTTCACCCT
GGCATGGGGACTGGTCCTGGCACGGAGC---GCAGCGTCCCACTCTGCAA
CAGCTTGCTATCCCCGCAGCAAAGCGAGGAGCCCACTGTTGCCACCCCCC
CGCAGCGATGGTTTGTCACCC---CTGCCAACAACCGACTGGACTTTGCA
GCCTCAACCTACGACGCCGCC---------GATTTCGCCGGTAACGCCGC
CACCTTGCTGTCTTACGCTGCGGCAGGAGTGAAGGCTC------TCCCGC
TGCCGACCGCAGGCTGCTCCAACCGGCCTCTCGGTTATTACGCAGACCCG
TCGG---GATGG---GGAGGACGCACGCCGCCGCAGTACTGCGGCATGAA
CAGCAAATCCAGCTCGGTGTTTTCCTGCTGGCCCCCTAACTCTATCGGAA
GCCGAGCTGCAA---GC---AACTACCTGG---------CCGAGGA---G
GGA---GAGTC---CATCCCCGCAGAGAGGTCACCG---AT---CGGCGG
CTCAGAGGAG---ACCAAACCCAAAGACTTAAC---GTCAGA---GTCGA
GCTGGATAGAG---ACGCCCTCCTCCATCAAATCCATCGATTCCAGCGAT
TCTGGGATCTTTG---AACAGGCCAAAAGGAGAAGGATCTCACCTTCCGC
CACGCCG---------GTGTCAGAGACAGTGTCCCCGTTAAAATCGGAGC
ATCACTCAACAGGCGAAGTCACAGAGCGAGAAGTGGCGCTGGGGATAAAT
CCGTTCGCGGATGGATGGGCGCCTTCAAAATAAACCACAGCTCCCACGA
TATTGGCTCCGG---ACAGACGGCGTTTTCCTCCCAGGCT---CCCGGCT
AC---GCAGCAGCCGCCTTGGGG---CACCATCA------CCACCCGACC
CACGTTGGCTCT---TACTCCACCGCTGCTTTCAACTCCACCAGGGACTT

TCTCTTCAGAAATCGAGGATTCGGGGATGCCGGCGG------------CG
CGCAGCACAGTTTGTTCGCCTC---------CGGAAGTTT---C------
GCAGGGCCACATGGACACTCAGATGCAGCGGGGCACCTGCTCTTCCCGGG
GCTCCACGAG---CAAGCGGCGACCCACGCGTCTTCCAATGTGGTCAACA
GCCAGATGCGGCTGGGTTTCTCGGGGGACATGTACGGACGGGCTGACCAG
TATGGCCACGTTACGAGCCCTCGCT---CCGACCACTATGCCTCGACCCA
GCTGCATGGCTACGGCCCTATGAACATGAATATGGCCGCA---CACCACG
GTGCAGGGGCCTTCTTCCGATACATGAGGCAGCCCATCAAACAAGAGCTC
ATCTGCAAGTGGATCGAGCCGGAGCAGCTGTCGAACCCCAAAAAGTCGTG
CAACAAAACTTTTAGCACTATGCACGAGCTGGTGACCCATCTGACGGTGG
AGCATGTTGGGGGACCGGAGCAGACCAACCACATCTGCTTCTGGGAGGAT
TGCGCCAGAGAGGGGAAGCCATTCCAAGCCAAGTACAAACTTGTAAATCA
TATCAGAGTTCACACCGGAGAGAAGCCCTTTCCGTGTCNNNNNNNNNNNNNNNNNN
>Lota lota
AGCCTGCTCATTCGAGCAGAGCTAAGTCAACCCGGCGCACTCCTTGGTGA
CGATCAGATTTATAATGTAATCGTCACAGCACACGCTTTCGTAATAATTT
TCTTTATAGTAATACCACTAATAATTGGAGGCTTCGGAAACTGACTAGTC
CCCCTAATGATCGGCGCCCCCGATATAGCCTTTCCTCGTATGAACAACAT
GAGCTTCTGACTTCTTCCTCCATCATTCTTGCTCCTCCTAGCATCCTCTG
GGGTAGAAGCCGGAGCCGGAACAGGTTGAACTGTATACCCTCCTCTAGCA
GGCAATCTTGCTCATGCTGGGGCTTCTGTTGACCTTACTATTTTCTCCCT
GCATTTAGCAGGGGTCTCATCAATTCTTGGAGCAATTAATTTTATTACCA
CCATCATCAACATGAAACCCCCAGCAATCTCACAATATCAAACACCGCTA
TTTGTCTGAGCAGTCCTAATTACAGCCGTCCTACTACTCCTGTCTCTCCC
CGTTTTAGCCGCTGGTATTACGATACTGCTGACTGATCGAAATCTTAATA
CTTCCTTCTTTGACCCCGCTGGAGGAGGGGACCCAATTCTGTATCAGCAC
TTA---

------------TTCCTGGAGAGGAACCTCCACCCGACCAACTGCCTGGG
CATGCTGCTGCTGTCCGACGCCCACCAGTGCGTCAAGCTCTCGGAGCTGT
CCTGGAGCATGTGCCTGAGCAACTTCCCTGCCATATGCAAGACGGAGGAT
TTTCTCCAACTGCCCAAAGACATGGTTGTGCAGCTCTTGTCCCACGAGGA
GCTGGAGACGGAGGACGAGAAGCTTGTCTACGAGGCCGCGCTGAACTGGG
TCACCTATGACCTGCGAGGGAGGCACCCCCACTTGCCGGAGCTGCTGAAA
ACGGTGCGTCTGGCCCTTCTGCCCGCCGTCTTCCTCATGGAAAATGTCTC
CACCGAAGAGCTGATCAACAGCCAGGCCAAGAGCAAGGAGCTGGTGGACG
AGGCCATCTGCTGCAAGCTGAAGATCCTGCAGAACGACGGCGTGGTCAAC
AGCCCGTGTGCCCGTCCCAGGAAGACCAGCCACGCRCTCTTCCTGCTGGG
CGGCCAGACCTTCATGTGCGACAAGCTGTACCTGGTGGACCAGAAAGCCA
AAGAGATCATCCCCAAGGCCGACATCCCCAGCCCCAGGAAGGAGTTCAGC
GCCTGCGCCATCGGCTGCAGGGTGTACATCACGGGCGGGC--GGGGCTC-
GGAGAACGGCGTGTCCAAGGACGTGTGGGTGTACGACACGGTGCAGGAGG
AGTGGTCCAAGGCGGCGCCCATGCTCATCGCCCGCTTCGGCCACGGCTCC
GCGGAGCTGAAGCACTGCCTGTACGTGGTGGGCGGGCACACGGCCGCCAC
GGGCTGCCTCCCGGCGTCNCCCTCCGGAGGATTACATTGTGGTCTTCTCTC
GTTCCACAACGAGGTTGATCCTAAATGAAGCAGAGCTGATCATGGCACTG
GCCCAGGAGTTTCAGATGAGAGTCTTTACGGTGTCCCTGGAGGAGCAGTC
TTTCGCCAGCATAGTGCAGGTGATCAGCGGAGCCTCCATGCTGTTCAGTA
TGCATGGCGCTCAGCTCGTCACGTCATTGTTTCTGCCCAGAGGGGCAGCT
GTGGTGGAGCTGTTCCCCTATGCGGTTAACCCAGAGCAGTACACCCCGTA
TAAAACCCTAGCCTCCCTGCCGGGCATGGACCTCCAGTATGTCTCCTGGA

GGAACTCGATGGAGGAGAACACTGTCACTCATCCGGACCGGCCCTGGGAC
CAAGGGGGGATCGTGCACTTGGAGAAGGAGGAACAAGAGCGCATACAGGC
CAGCAAGGACGTCCCCAGGCACCTGTGCTGTCGTAACCCAGAGTGGCTAT
TCAGAATCTACCAGGACACTTTGGTGGACATCCCTTCACTCCTAGAGGTG
CTGAA---GGAGGGCCTCAAA---ACCAGGCCCAACGTGAAAAA---GAA
CAAGGCGGCCAGCACAGTTCACCCAGGGCGTGTCCGAGAACCCCAATGCC
AAACCTCGGTCCAGGCCACCAACGAGGCCAAACTCACAGTCTCCTGGCAG
ATCCCATGGAACCTGAAATTCCTGAAGGTGAGAGAGGTGAAGTATGAGGT
GTGGATACAGAAGAAGGATGCCAGCAAAGGAACTCTGGAGGATCAAATTA
TCCAGGCCAACCCTGCCCTGGAGGCCTTCGGTAACGCCAAAACCCTGAGA
AATGACAACTCCTCCCGTTTTGGAAAGTTCATCCGGATTCACTTTGGGAA
CAGCGGCAAGCTGAGCTCTGCGGACATCGAGACCTACCTGCTTGAAAAGT
CCAGAGTCACTTTCCAGCTGAAGTCTGAGAGGAACTATCACATATTCTTC
CAGATCTTGTCCAACCAGAAACCGGAGCTGCTGGACATGCTGCTGATTAC
CAACAATCCATACGATTACTCGTACATCTCCCAAGGAGAGGTAACCGTCG
CCTCCATCAACGATTCTGAGGAGCTCATTGCCACTGATAGTGCATTTGAC
GTCCTCGGCTTCACCCTGGAAGAAAAGATGGGGGTGTACAAGCTGACTGG
TGCCATCATGCACTATGGCAACATGAGATTCAAGCAGAAACAGCGTGAAG
AGCAGGCAGAGTCAGACGGGACTGAAGCAGCTGATAAATCAGCATACCTC
ATGGGTCTGAACTCGGCGGACCTCATCAAAGGCCTCTGCCACCCCAGGGT
GAAGGTAGGAAATGAATATGTCACCAAAGGTCAAGGGGTGGACCAGGTCT
ACTAC---

---------------------TACCTCATCTACGCGTCCTTCTCCTTCAT
GGGATGCCTACAGATCAGCGACGGCTCCAACGTGGTCAACCTGCTGGCCA
GCAACTCGCCCAGTGTGTCGTACGCGCTGACGCAGCAGAAGTACTTCAGC
AACTACAGCCCCGTCATTGGCTTCTACATCTACGAGCCCATCGAGTACTG
GAACGCCACGGTGCAGGAGCACCTGAAGACGCTGAGCAACGGCTTCAACA
AGATCTCCTGGATGGACAACTTCTACCAGTACCTGCGCGTGGCCAACGTG
AGCGCGTCCACCAAGAGCGACTTCATCGGCGTCCTGAAGGGCTCCTTCCT
GCGGAGCCCGGAGTACCAGCACTTCACGGAGGACATCATCTTCTCCAAGA
---ACCGCGAGAGCG------ACGAGTACGACATCATCGCCTCGCGCATG
TACCTGGTGGCCAGGACCGCGGAGAAGAAGCGCGAGGAGGTGGTGGAGCT
GCTGGAGAAGCTGCGTCCGCTGATGCTCATCAACAGCATCAAGTTCATCG
CTTTCAACCCCACCTTCGTGTTCATGGACCGCTACAGCTCCTCCGTCATC
TCCCCCGTCCTCACCTCGGGCTTCAGCGTGCTCACCGTCCTCGTCCTCAC
CTTCTTCCTGGTGGTCAACCCGCTGGGGAACCTGTGGCTGATCCTGACCG
TGACCTCGGTGGAGCTTGGGCGTCTGGGCCTCATGNNNNNNNNNNNNNNNNNNNNNNNNNNNNNNNNNNN
NNNNNNNNNNNNNNNNNNNNNNNNNNNNNNNNNNNNNNNNNNNNNNNNNNNNNNNNNNNNNNNNNNNNNN
NNNNNNNNNNNNNNNNNNNNNNNNNNNNNNNNNNNNNNNNNNNNNNNNNNNNNNNNNNNNNNNNNNNNNN
NNNNNNNNNNNNNNNNNNNNCTGAGGGAGCGCGGG
CTGGGAGACGGCGCCTGCGCCTCGGCCTTCTCCGTCATGGTCAAGGAGTC

CTGCGACGGCATGGGGGACGTGAGCGAGAAGCACGGCGGGGGCCCGGCCG
TCCCGGAGAAAGCGGTGCGTTTCTCGTTCACCGTCATGTCCGTCTCCTTG
CACGCAGCCGGCGGCGAG------------------------------GA
GGAGGTGATCATCTTCCAGGAGCCGAAGCCCAACTCTGAACTGTCCTGTA
AGCCCCTGTGCCTCATGTTTGTGGACGAGTCCGACCACGAGACGCTCACC
GCCGTCCTGGGGCCCGTGGCGGCCGAGCGCAGCGCCATGAAGCGCAGCCG
GCTCGTCCTCGCCATCGGCGGGCTTCCCCGCGTCTTCAGCTTCCGCTTCA
GAGGCTCGGGCTACGACGAGAAGATGGTGCGCGACGTGGAGGGCCTGGAG
GCCTCCGGCTCCAGCTACGTCTGCACGCTTTGTGACTCCACCCGCGCCGA
GGCCGCCGACAACATGGTGCTCCACTCCGTGACCCGCAGCCACCAGGAGA
ACCTGGAGCGCTATGAGACCTGGAGGACCAACCCCTTCTCGGAGTCTGCG
GAGGAACTGAGGGACAGGGTGAAGGGGGTCTCCGCCAAGCCCTTCCTGGA
GACCCACCCCACGCTGGACGCGCTGCACTGCGACATCGGCAACGCCACCG
AGTTCTACAAGATCTTCCAGGATGAAATCGGCGAGGTGTTCCAGAGGGC-
--CAGC---CCGACCAGGGAGGAGCGACGGGGCTGGAGGGCGGCCCTGGA
CAAGCAGNNNNNNNNNNNNNNNNNNNNNNNNNNNNNNNNNNNNNNNNNNNNNNNNNNNNNNNNNNN
NNNNNNNNNNNNNNNNNNNNNNNNNNNNNNNNNNNNNNNNNNNNNNNNNNNNNNNNNNNNNNNNNNNNNN
NNNNNNNNNNNNNNNNNNNNNNNNNNNNNNNNNNNNNNNNNNNNNNNNNNNNNNNNNNNNNNNNNNNNNN
NNNNNNNNNNNNNNNNNNNNNNNNNNNNNNNNNNNNNNNNNNNNNNNNNNNNNNNNNNNNNNNNNNNNNN
NNNNNNNNNNNNNNNNNNNNNNNNNNNNNNNNNNNNNNNNNNNNNNNNNNNNNNNNNNNNNNNNNNNNNN
NNNNNNNNNNNNNNNNNNNNNNNNNNNNNNNNNNNNNNNNNNNN---
TCGTACACCATCGAGATGGGTCCCAAGGGCCCCCAGTGGA
AGGAGAGCCCCCAGCCCTTCTCCTGCTCCATTGAAGACCCCACCAAACAG
ACGAAGTTCAARGGCATCAAGACSTACATTTCATACCGGGTSACACCCAG
CCACACSGGCGGGGCCGTGTACAGGCGCTACAAACACTTTGACTGGCTGT
ACAACCGCYTRCTGCACAAGTTCACCGTGATCTCCGTGCCYCACCTGCCC
GAGAAGCAGGCCACGGGCCGMTTYGAGGAAGACTTCATCGACAAGCGCAA
GAGGCGRCTCATCCTGTGGATGAACCACATGACCAGYCACCCAGTSCTGT
CCCAGTACGAGGGCTTCGAGCACTTYCTCATGTGYGCGGAYGACAAGCAG
TGGAARCTGGGCAAGAGGCGGGCGGAGAAGGACGAGATGGTGGGTGCCCA
CTTCATGCTCACCTTCCAGATCCCCAACGAGCACCAGGACCTGCAGGAYG
TGGAGGAGCGCGTGGACTCCTTCAAGTCCTTCACCAAGAAGATGGATGAC
AGCGTCATGCAGCTGACCCACGGYGCCTCCGAGATGGTGCGGAGGCACCT
GGGAGAGTTCCGGAAGGAGTTCCACCGGCTCGGGAGCGCCTTCCAGTCCC
TGTCGCAGGCGTTCATGCTGGAACCCCCGCACAGCTCGGAGCCGCTCAAC
AACGCCATCTCGCAC-----------------------------------

--GCCAAATCTCGCTTTCACCCTGGCGTGGGGACTGGTCCTGGCACGGAG
C---GCAGCGTCCCACTCGGCAACAGCTTGCTGTCCCCGCAGCAAACCGA
CGAGCCCACTGTTGCCACCCCCCCGCAGCGATGGTTTGTCACCC---CTG
CCAACAACCGACTGGACTTTGCTGCCTCGGCATACGACGCCGCT------
---GATTTCGCCGGTAACGCGGCCACCTTGCTGTCCTACGCAGCGGCCGG
AGTGAAGGCTC------TTCCCCTGCCGACTGCAGGCTGCTCCAACCGGG
CTCTTGGCTATTACGCCGACCCGTCGG---GCTGG---GGAGGACGCACA
CCGCCACAATACTGTGGCAGTAACAGCAAATCCAGCTCTGTATTTTCCTG
CTGGCCTGCTAACTCTCATCGGGGCAGAGCGGGCA---CC---AACTACC
TGG---------CTGAGGA---GGGA---GACTC---CATCCCGACAGAG
AGGTCCCCG---AT---CGGCGGCTCGGAGGAG---ACCAAACCCAAAGA
CATGAC---ATCTGA---GTCCAACTGGATAGAG---ACGCCGTCCTCCA
TTAAATCCATTGATTCGAGCGACTCTGGTATCTTTG---AACAGGTCAAA
AGGAGAAGAATCTCACCTTCTGCCACGCCG---------GTTTCAGAGAC
AGTGTCCCCGTTAAAATCTGAGCATCACTCAACAGGCGAAGTCACAGAAA
GAGAAGTCGCGTTGGGGATAAATCCGTTCGCAGACGGAATGGGCGCCTTC
AAAATAAACCACAGTTCTCACGACATTGGCTCCGG---ACAAACTGCGTT
TTCATCCCAGGCG---CCCGGCTAC---GCAGCGGCTGCACTCGGA---C
ACCACCA------TCACCCCACCCATGTTAGCTCT---TACTCCACCGCC
GCCTTCAACTCTACACGGGACTTTCTTTTCAGAAATCGGGGCTTCGGAGA
CGCCACCAG------------CGCGCAGCACAGTTTGTTCGCCTCCGC--
-CGCGGGAAGTTT---T------GCAGGGCCACATGGACACTCCGATGCC
ACGGGACACCTGCTCTTCCCCGGGCTCCACGAG---CAAGCGGCGAGCCA
TGCGTCTTCTAATGTCGTCAACAGCCAGATGCGACTGGGCTTTTCCGGAG
ACATGTACGGCCGGGCCGACCAGTATGGTCACGTTACGAGCCCACGGT--
-CCGACCACTACGCGTCGACCCAGTTGCACGGCTATGGCCCTATGAACAT
GAATATGGCCGCG---CACCACGGAGCCGGGGCCTTCTTTCGCTACATGA
GGCAGCCGATCAAGCAAGAGCTGATCTGCAAGTGGATCGAGCCGGAGCAA
CTGTCAAATCCCAAAAAGTCGTGCAACAAAACGTTCAGCACGATGCACGA
GCTGGTGACCCATCTGACGGTGGAACATGTGGGGGGACCCGAGCAGTCGA
ACCACATTTGCTTCTGGGAAGAATGTTCACGAGAAGGAAAACCGTTCAAA
GCTAAATACAAACTTGTGAATCATATTAGAGTACACACCGGAGAAAAGCC
GTTCCCGTGTCCATTCCCCGGCTGTGGCAAA
>Lucigadus nigromarginatus

---TCT
CCTGGAGCATGTGCCTGAGCAACTTCCCTGCCATTTGCAAGACAGAGGAC
TTCCTCCAACTGCCCAAAGACATGGTCGTGCAGCTTTTGTCCCACTAGGA
GCTGGAGACGGAAGACGAGAAACTGGTTTACGAGGCCGTGCTGAACTGGG
TCACCTACGACCTGCCGGGGAGGCACGCCCACTTGCCAGAGCTCCTGAAA
ACGGTGCGCCTGGCTCTCCTGCCCGCCATTTTCCTCATGGAGAACGTCTC
CATGGAGGAGCTGATCAATGCGCAGACCAAGAGCAAGGAGCTGGTGGACG
AGGCCATCTGCTGCAAGCTGAAGATCCTGCATAACGACGGCGTGGTCGAC
AGCCCTTTGGCCCGTCCCAGGAAGACCAGCCACGCGCTCTTCCTGCTTGG
CGGGCAGACCTTCATGTGCGACTATTTTTTTCTGGTGGACCAGAAAGCCA
AAGAGATCATCCCCAAGGCCGACATCCCCAGCCCCCGCAAGGAGTTCAGC
GCCTGCGCCATCGGCTGCAAGGTGTACATCACCGGGCGGCCGGGGGCTCG
GGAGAACGGCGTGTCCAAGGACGTGTGGGTGTACGACACCGTGCAAGAGG
AGTGGTTCAGGGCGGCGCCCATGCTCATCGCCCGCTTCGGCCACGGCTCC
AC--
-------------------------GGAGGATTACATTGTGGTCTTCTCT
CGCTCCACAACGAGGTTGATCCTAAATGAAGCGGAGCTGATCATGGCGCT
GGCCCAGGAGTTTCAGATGCGAGTCTTGACGGTGTCCCTGGAGGAGCAGT
CTTTTGCCAGCATCGTGCAGGTGATCAGCGGAGCAACCATGCTGTTCAGT
ATGCACGGCGCTCAGCTCGTCACGGCGTTGTTCCTGCCCAGAGGTGCAGC
CGTGGTGGAGCTGTTCCCCTATGCCGTGAACCCGGAGCAGTACACCCCGT
ATAAAACCCTCGCCTCCCTGCCGGGAATGGACCTCCAGTACATCTCCTGG
AGGAACACAATGGAGGAGAACACCGTCACTCACCCGGACCGACCCTGGGA
CCAAGGGGGTGTCGTGCACTTGGAGAAGGAGGAACAAGAGCGCATACTAG
CCAGCAAGGACGTCCCCAGGCACCTATGCTGCCGTAACCCAGAGTGGCTT
TTCAGAATCTACCAGGACACTTTGGTGGACATCCCTTCACTCCTCGAGGT
GCTCAA---GGAGGGCCTCAAA---ACCAGGCCCAACTTGAAAAA---GA
GCAAGGCRGCCAGCACAGTTCACCCGGGGAGGGTCCGAGAACCCCAGTGC
CAGACCTCGGTCCAAGCCACCAATGAGGCCAAACTCACAGTGTCCTGGCA
GATCCCGTGGAACCTGAAGTTCCTGAAGGTGAGAGAGGTGAAGTACGA--
-----------AAGAAGGATGCCAGCAAAGGAACCCTGGAGGATCAAATC
ATCCAGGCCAACCCTGCCCTGGAGGCCTTCGGTAATGCCAAAACCCTGAG
AAATGACAACTCCTCCCGTTTTGGAAAGTTCATCCGGATTCACTTTGGGA
ACAGTGGCAAGCTTAGCTCAGCGGACATTGAGACCTACCTGCTAGAGAAG
TCCAGAGTCACTTTCCAGCTCAAGTCAGAGAGGAACTATCACATATTCTT
CCAGATCTTGTCCAATCAGAAGCCAGAGCTGCTGGACATGCTGCTGATAA
CCAACAACCCATACGATTACTCCTACATCTCTCAAGGAGAAGTAACTGTG
GCCTCCATCAATGACTCTGAGGAGCTCATGGCCACTGACAGTGCATTTGA
CGTCCTTGGCTTCACCCTGGAAGAGAAGATGGGAGTGTACAAGCTCACGG
GTGCCATCATGCACTATGGCAACATGAGGTTCAAGCAGAAGCAGCGTGAG
GAGCAGGCAGAGTCAGACGGGACTGAAGCTGCTGATAAATCAGCGTACCT
CATGGGTTTGAACTCTGCAGACCTCATCAAAGGCCTCTGCCACCCCAGGG
TGAAGGTAGGAAATGAATATGTCACCAAAGGTCAAGGGGTGGACCAAGTC
TACTAC--


```
--------------------------------------------------
--------------------------------------------------
--------------------------------------------------
----------------------TACCTTATCTATGCCTCCTTCTCCATTA
TGGGCWGTTTACAGATCAGCGACGGCTCCNACGTGGTCAACYTGCTGGCCA
GTAACTCCCCGAGCGTATCCTATGCTCTGACSCAGCAGAAGTACTTCAGT
AACTACAGTCCTGTGATCGGCTTCTACATCTACGAGCCCATCGAGTACTG
GAACTCCACCGTGCAGGAGCATCTGAAGACCCTGAGTCACGGTTTCAACA
AGATCTCCTGGATGGACAACTTTTACCAKTACCTGCGGGTGGCCAACGTC
AGCGCGTCCACCAAGAACGATTTCATTCACATCCTGAAGGGTTCCTTCTT
GCGGAGCCCGGAGTATCAGCACTTCACAGAGGACATCATCTTCTCCAAGA
---ACCGTGAGAGCG------ACGAGTATGACATCATTGCCTCACGCATG
TACCTGGTGGCCCGGACCACGGAGAAGAAGCGAGAGGAGGTGGTGGAGCT
KCTGGAGAAGCTGCGWCCAYTGATGCTCATCAACAGCATCAAGTTCATCG
CCTTCAATCCCACCTTCGTGTTCATGGACCGGTACAGCTCCTCTGTCATC
TCGCCCATCCTCACCTCCGGCTTTAGCGTGCTCACCATCCTCATCCTCAC
CTTCTTCYTGGTYATCAACCCTCTGGGGAACCTCTGGTTGATCTTGACCG
TGACCTCGGTGGAGCTGGGCGTCCTGGGTCTGATG---------------
--------------------------------------------------
--------------------------------------------------
--------------------------------------------------
--------------------------------------------------
--------------------------------------------------
--------------------------------------------------
--------------------------------------------------
--------------------------------------------------
--------------------------------------------------
--------------------------------------------------
--------------------------------------------------
--------------------------------------------------
--------------------------------------------------
--------------TCTGCACACTGTGAGATTCCACTCGGGCAGAGGCCT
CCCACAACATGGTGCTACACTCCGTCACCCGCAGCCACGAGGAGAACCTA
GAGCGCTACGAGACATGGAGGACCAACCCCTTTTCAGAGTCCACGGACAA
GCTGAGAGACAGGGTGAAGGGGGTCTCCGCCAAACCCTTCATGGAGACCC
ATCCCACAATGGATGCGTTGCACTGTGACATAGGCAACGCCACAGAGTTC
TACAAAATCTTCCAGGATGAAATTGGGGAGGTGTTCCAGAAGAC---CAA
C---CCCACCCGGGACGAGCGGCGGAGCTGGAGGACGGCCCTGGACAAGC
AGCTGAGGAAGACGCTCAAGCTCAAGCCGGTGATGAGGATAAACGGCAAT
TACGCCCGGAAGCTGATGACCATGGAGGCCGCAGAGGCCGTGTGCGAGCT
GGTGCCGTCGGAGGACAGTCGGGAGGCCATCAGGCAGCTCA---------
--------------------------------------------------
--------------------------------------------------
--------------------------------------------------
--------------------------------------------------
------------------------------------------CGTAACAC
GGTGGAGATCGGCCCCAAGGGCCCCCAGTGGCAGGAGAGTCCCCGGCCCT
TCTCCTGCTCCATCGAGGACCCCACCAAACAGACTAAGTTCAAGGGCATC
AAGACCTACATCTCGTACCGCGTGACGCCCAGCCACACCGGCATGGCCGT
GTACCGCCGCTACAAACACTTTGACTGGCTGTACAACCGCCTGCTGCACA
AGTTCACCGTGATCTCCGTGCCCCACCTGCCCGAGAAGCAGGCCACGGGC
CGCTTCGAGGAGGACTTCATCGACAAGCGCAAGCGGCGGCTGGTCCTGTG
GATGAACCACATGACCAGCCACCCGGTGCTTTCGCAGTACGAGGGCTTCG
```

AGCACTTCCTCATGTGCGCCGACGACAAGCAGTGGAAGCTGGGCAAGCGG
CGGGCGGAGAAGGACGAGATGGTGGGCGCCCACTTCATGCTCACCTTCCA
GATCCCCAACGAGCACCAGGACCTGCAGGATGTGGAGGAGCGCGTGGACT
CCTTCAAGGCCTTCACCAAGAAGATGGACGACAGCGTCATGCAGCTGACG
CACGGCGCCTCGGAGATGGTGCGCCGGCACCTGGGAGAGTTCCGCAAGGA
GTTCCACCGGCTGGGCGGTGCCTTCCAGTCGCTGTCGCAGGCCTTCATGC
TGGAGCCGCCGCACAGCTCGGAGCCACTCAACAACGCCATCTCGCAT---

----------------------------------GCCAAATCTCGCTTTC
ACCCTGGCATGGGGACTGGTCCTGGCACGGAGC---GCAGCGTCCCACTC
GGCAACAGCTTGCTATCCCCGCAGCAAAGCGAGGAGCCCACTGTTGCCAC
CCCCCCGCAGCGATGGTTTGTCACCC---CTGCCAACAACCGACTGGACT
TTGCAGCCTCGGCTTACGACGCCGCC---------GATTTCGCCGGTAAC
GCCGCCACCTTGCTGTCTTACGCGGCGGCAGGAGTGAAGGCTC------T
CCCCCTGCCGACTGCAGGCTGCTCCAACCGGCCTCTCGGCTATTACGCAG
ACCCGTCGG---GCTGG---GGAGGACGCACGCCGCCGCAGTATTGCGGG
GTGAACAGCAAATCCAGCTCGGTGTTTTCCTGCTGGCCCCCTAACTCTAT
CGGAAGCCGGGCTGCCA---CC---AACTACCTGG---------CCGAGG
A---GGGA---GACTC---GATCCCCGCAGAGAGGTCACCG---AT---C
GGCGGCTCCGAGGAG---ACCAAACCCAAAGACTTAAC---GTCAGA---
GTCGGGCTGGATAGAG---ACGCCGTCCTCCATCAAATCTATTGATTCCA
GCGATTCTGGGATCTTTG---AACAGGCCAAAAGGAGACGGATCTCACCT
TCTGCCACACCA---------GTGTCAGAGACAGTGTCTCCGTTAAAATC
TGAG--TCACCCAACAGGGGAGGTCACGGACAGAGAAGTCGCGTTGGGGA
TCAACCCGCTCGCGGACGGGATGGGCGCCTTCAAAATCAACCACAGCTCC
CACGACATCGGCTCCGG---TCAAACGGCGTTTTCCTCCCAGGCG---CC
CGGCTACGCGGCGGCGGCCGCCCTCGGA---CACCACCACCATCCCCACC
CCACGCACGTGAGCTCT---TACTCCACCGCCGCCTTCAACTCCACACGG
GACTTTCTTTTCAGAARTCGGGGTTTCGGAGACGCCACCGG---------
---CGCGCAGCACAGCTTGTTCGCCTCCGC---CGCGGAAGTTT---C-
-----GCGGGGCCACACGGACACTCCGAGGCCACGGGGCACCTGCTCTTC
CCGGGGCTCCACGAG---CAGGCGGCCAGCCACGCGTCCTCCAATGTGGT
CAACGGCCAGATGCGACTGGGCTTTTCCGGAGACATGTACGGAAGGGCCG
ACCAGTACGGGCACGTAACGAGCCCGAGGT---CCGACCACTACGCGTCG
ACCCAGTTGCACGGCTACGGCCCCATGAACATGAATATGGCCGCGGCTCA

CCACGGGGCCGGGGCCTTCTTTCGGTACATGCGGCAGCCGATCAAACAGG
AGCTGATCTGCAAGTGGATCGAGCCGGAGCAGCTGTCCAATCCCAAAAAG
TCGTGCAACAAAACTTTCAGCACGATGCACGAGCTGGTGACCCACCTGAC
GGTGGAGCACGTGGGGGGGCCCGAGCAGTCGAACCACATCTGCTTCTGGG
AAGAGTGTTCACGGGAAGGGAAGCCGTTCAAGGCTAAATACAAACTTGTG
AACCACATCAGGGTTCACACCGGA-AGAAACCGTTCCCATGTCC------

>Luvarus imperialis
AGTCTGCTCATCCGGGCAGAGCTCAGCCAACCAGGCGCCCTCCTCGGGGA
CGACCAAATTTACAATGTTATCGTTACGGCGCACGCATTCGTTATGATTT
TCTTTATAGTAATGCCAATTATGATTGGAGGGTTCGGAAACTGACTGATC
CCCCTCATGATCGGAGCCCCCGACATAGCATTCCCCCGAATAAACAACAT
GAGCTTTTGACTCCTCCCTCCCTCTTTCCTTCTCCTTCTTGCCTCCTCTG
GAGTAGAAGCTGGTGCCGGCACCGGATGAACTGTCTACCCCCCATTAGCT
GGAAACCTCGCACATGCGGGAGCATCCGTAGACCTGACCATTTTCTCCCT
GCACCTAGCAGGTATTTCCTCAATCCTGGGGGCCATTAACTTCATTACAA
CTATCATCAACATGAAACCTCCCGCTATTTCCCAATACCAAACCCCTCTT
TTCGTATGGGCTGTTCTAATTACTGCTGTCCTGCTCCTCCTCTCACTTCC
AGTCCTTGCTGCTGGAATCACAATGCTCTTAACAGATCGAAACCTCAACA
CCACCTTCTTCGACCCCGCGGGCGGAGGGGACCCCATTCTTTACCAACAC
CTGTTCTGATTCTTCGGCCA------------------------------

------------NNNCTAGAGAGAAACCTTCACCCATCTAACTGCCTTGGCAT
GCTGTTGCTGTCTGATGCCCACCAGTGTATCAAGCTGTCAGAGCTCTCCT
GGGGCATGTGCCTCAGCAACTTTCCCGCTATTTGCAAGACAGAGGACTTC
CTCCAGCTGCCCAAAGATATGGTGGTGCAGCTTTTGTCACATGAGGAGCT
AGAAACAGAAGAYGAGAGACTGGTTTATGAAGCTGCCCTCAATTGGATCA
ACTATGACCTGGAAAAGAGACACTGCCACCTACCAGAGCTCCTGAGAACA
GTCCGTCTGGCCCTGCTGCCTGCCATCTTTCTCATGGAGAACGTTTCTAC
AGAAGAGCTGATCAACGCCCAGGCCAAGAGCAAGGAGCTGGTGGATGAAG
CTATCCGCTGTAAGCTGAAGATCCTGCAGAATGATGGCGTTGTTAACAGC
CCATGTGCTCGACCAAGAAAAACCAGCCATGCTCTCTTTCTTCTGGGAGG
GCAGACTTTCATGTGTGACAAGTTGTACCTGGTGGACCAGAAAGCCAAAG
AGATCATCCCCAAGGCTGACATTCCCAGCCCCAGGAAGGAGTTCAGCGCC
TGTGCCATTGGCTGTAAGGTGTACATCACTGGTGGAA--GAGGCTC-AGA
GAATGGCGTGTCCAAAGATGTATGGGTCTACGACACCGTCCACGAGGAAT
GGTCGAAGGCGGCACCCATGCTCATTGCCAGGTTTGGCCATGGCTCTGCG
GAGCTGAAACACTGCCTCTACGTGGTAGGAGGTCACACAGCTGCAACTGG
TTGCCTCCCGGCCTCTCCGTCTGGATGAATACATTGTTGTGTTTAGTCGT
TCAACAACGAGGCTGATACTGAATGAAGCAGAGTTAATCATGGTGCTGGC
CCAGGAGTTCCAGATGAGAGTGGTCACAGTATCCCTGGAGGAACAGTCTT
TCCCCAGTATCGTCCAGGTGATCAGCGGTGCTACCATGTTAGTCAGTATG
CATGGAGCTCAGCTTATTACCTCACTCTTCCTCCCCAGAGGAGCTGTTGT
GGTGGAGCTGTTCCCCTTTGCTGTGAACCCAGAGCAGTACACTCCATATA
AAACCCTTGCCTCCCTTCCAGGCATGGACCTTCACTATGTCTCCTGGAGG
AACACCAAGGAGGAGAACACCATCACCCACCCAGACAGACACTGGGAACA
GGGGGGCATCGCTCACTTGGAGAAGGAGGAGCAAGAGCGAATACTGGCAA
GCAAAGATGTCCCCAGGCACCTGTGCTGCCGCAACCCAGAATGGCTTTTT
CGGATCTACCAGGACACTTTGGTGGACATCCCCTCCTTTCTGGAAGTCCT
CAA---AGAGGGCATGAAG---ACAAAGCCCAGCTTGAAGAA---GTCAA
AGTTGGCCAGCACTGTCCACCCAGGCCGTGTCAGAGAACCCCAGTGTCAG

ACCTCAGTGCAAACCACAAATGAGGCTAAACTCACAGTCTCCTGGCAGAT
CCCGTGGAATCTGAAATACCTANNNNNNNNNNNNNNNNNNNNNNNNNNNNNNNNNNNNNNAAAAAAGACASC
AGCAAGGGGACGCTGG
AGGATCAGATCATCCAGGCGAACCCTGCGCTGGAGGCCTTCGGCAACGCC
AAGACGCTGAGGAACGACAACTCGTCCCGGTTTGGAAAATTCATCCGAAT
TCACTTTGGCACCAGCGGCAAGCTGTCATCCGCCGACATCGAGACGTACC
TGCTGGAGAAGTCACGCGTCACCTTCCAGCTCAAGGCTGAGAGGAATTAC
CACATCTTCTACCAGATCCTGTCCAATCAGAAGCCAGAGCTGCTGGACAT
GCTGCTGATCACCAACAACCCATACGACTACTCCTACATCTCCCAAGGAG
AGGTATCGGTCGCCTCCATCAATGACTCGGAGGAGCTGATGGCCACGGAC
AGCGCCTTCGACGTGCTCGGCTTCACCTCAGACGAGAAGATGGGTGTCTA
CAAGCTGACGGGCGCCATCATGCACTACGGCAACATGAAGTTCAAACAGA
AGCAGCGTGAGGAGCAGGCCGAGCCGGATGGGACGGAGGCTGCCGATAAA
TCGGCTTACCTGATGGGGCTGAACTCAGCCGACCTCATCAAGGGGCTGTG
CCATCCCAGGGTCAAGGTAGGAAATGAATATGTCACCAAAGGCCAAAGTG
TGGACCAGGTCTACTACCCCAACAAGGAGGCCTTCAAATGTGAGGAGTGT
GGGAAGCACTACAACACCAAGCTGGGATATAAGCGCCATGTGGCCATGCA
CTCTGCCACGGCAGGGGATCTCACCTGTAAAGTGTGCATGCAGACCTACG
AGAGCACGCCTGTGCTCTTGGAGCACCTAAAGAGCCACTCTGGGAAGTCT
TCAGGTGGCACCAAGGAGAAAAAACACCCGTGCGACCACTGTGACCGTCG
TTTCTACACACGGAAGGATGTGAGACGGCACATGGTGGTCCACACAGGCC
GAAAGGACTTCCTGTGCCAGTATTGTGCCCAACGTTTTGGCAGGAAGGAC
CACCTGACACGCCATGTGAAGAAGAGCCACTCGCAGGAGCTGCTGAAGAT
CAAGACGGAGCCTCCTGATATGTTAGGTCTTTTAGCTTCCGGGTCACCAC
CTTGCTCTGTGAAAGAAGAGCTCAGCCCCATGATGTGCGGCATGGGTCCC
AACAAAGACCCCATGATGGGCAAACCATTCCCCAGTGGGGCCCCTTTTCC
AATGGGCATGTACAACCCCCACCAT------CTCCAGGCCATGTCTAATT
CTGGGGTGGGTCACCCA------CACCCGTCCCTGATGCCCAGTTCCTTG
TCTGCAGCTATGGGCATGGGCTGTCACATGGATNNNNNNNNNNNNNNNNNNNNNNNNNNNNNNNATGTT
TACAGATCAGTG
ATGGATCAAATATCGTGAACCTGCTGGCTAGTAACTCTCCAAGTGTTTCA
TATGCTCTTACCCAGCAAAAATACTTCAGTAACTATAGTCCTGTGATTGG
GTTTTACATTTATGAGCCCATCGAGTACTGGAACTCCACGGTGCAGGAGC
ACCTGAAGACTCTGAGTCATGGCTTCAACAAGATCTCCTGGATGGACAAC
TTTTTCCACTACCTGCGGGTAGTGAATGTGAGTGCGTCAACCAAGAGTGA
CTTCATTACCATCCTCAAGGGTTCCTTCTTGCGCAGCCCAGAGTACCAGC
ACTTCACCGAGGACATCATATTCTCCAAGA---ACCGTGAGACTG-----
-ATGAGTATGACATTATCGCCTCACGGATGTATTTGGTGGCGCGGACGAC
AGAGAAGAAGCGCGAAGAGGTGGTGGAGCTTCTGGAAAAGCTTCGTCCGT
TGATGCTGATCAACAGCATCAAGTTCATTGCCTTCAATCCTACATTTGTG
TTCATGGACCGCTACAGCTCCTCTGTCATCTCACCCATCCTGACCTCAGG
ATTCAGCGTACTCACAATCCTCATCCTCACNNNNNNNNNNNNNNNNNNNNNNNNNNNNNNNNNNNNNNNN
NNNNNNNNNNNNNNNNNNNNNNNNNNNNNNNNNNNNNNNNNNNNNNNNNNNNNNNNNNNNNNNNNNNNNN
NNNNNNNNNNNNNNNNNNNNNNNNNNNNNNNNNNNNNNNNNNNNNNNNNNNNNNNNNNNNNNNNNNNNNN
NNNNNNNNNNNNNNNNNNNNNNNNNNNNNNNNNNNNNNNNNNNNNNNNNATGTGGCACTGGTGTCAGCA
TTAAGGGATCTGGAGGAGGATATCATGGAGGGGCTGAGAGAGAGTGGGAT
GGAAGACAGTGCTTGCACCTCAGGCTTTAGTGTCATGATCAAGGAATCTT
GTGATGGCATGGGCGATGTCAGCGAGAAGCATGGTGGAGGACCAGTTGTT
CCTGAGAAGGTTGTACGTTTCTCTTTCACTATCATGTCTATCTCTGTCCT
GGCAGATGATGAGGAG------------------------------GAAG
AGGTTACCATCTTCACAGAGCCAAAGCCAAACTCAGAACTGTCCTGTAAG
CCCCTTTGCCTGACGTTTGTTGATGAGTCAGATCATGAGACACTCACAGC
CATCCTGTCGCCTGTAATTGCAGAGCGTAATGCAATAAAAGAGAGCAGGC

TCATCTTATCCATCAGTGGACTACCTTGCGCCTTCCGCTTTCACTTCAGA
GGCACAGGATATGATGAGAAGATGGTGCGTGTGATGGAGGGCCTTGAGGC
CTCAGGGTCCACCTATGTCTGCACTCTTTGTGACTCCAGTCGGGCACAGG
CCTCTCAAAACATGGTGTTACACTCCATCACCCGCAGTCATGAAGAGAAC
CTAGAACGCTATGAAATATGGAGAACCAACCCCTTTTCTGAGTCTGTAGA
TGAGCTGCGAGACAGAGTCAAAGGGGTCTCTGCCAAGCCCTTTATGGAGA
CCCATCCCACACTGGATGCATTGCACTGTGACATAGGCAATGCCACTGAG
TTCTACAAAATCTTCCAGGATGAGATCGGGGAGGTGTACCAAAAGGT---
CAAC---CCCAGCCGGGAGGAAAGGCGCAGCTGGAGGGCAGCCCTAGATA
AACAGCTGAGGAACAAGTTGAAGCTTAAACCGGTAATGAGGATGAATGGG
AACTATGCCCGCCGGCTAATGACCCTGGAGACTGTAGAGGTGGTGTGTGA
GCTGGTGCCCTCAGAGGGGAGGAGAGAGGCCCTGAGGGAGCTCATAAGGC
TCTACCTCCAGATGAAGCCTGTGTGGCGNNNNNNNNNNNNNNNNNNNNNNNNNNNNNNNNNNNNNNNNN
NNNNNNNNNNNNNNNNNNNNNNNNNNNNNNNNNNNNNNNNNNNNNNNNNNNNNNNNNNNNNNNNNNNNN
NNNNNNNNNNNNNNNNNNNNNNNNNNNNNNNNNNNNNNNNNNNNNNNNNNNNNNNNNNNNNNNNNNNNN
NNNNNNNNNNNNNNNNNNNNNNNNNNNNNNNNNNTCGTACACCATCGAGATGGATC
CCTTGGGGCCCCGGTGGAAGGAGAGCCCACAGCCTTTCTGCTGCTCCATT
GAAGACCCCACAAAACAGACAAAGTTCAAGGGCATCAAGACGTACATTTC
GTACCGGGTCACTCCGAGCCACACAGGGCATCCTGTCTACAGGCGCTACA
AACACTTTGACTGGCTATACAACCGCTTACTGCACAAGTTCACTGTGATC
TCCGTGCCTCACCTGCCTGAGAAGCAGGCCACGGGGAGATTTGAGGAAGA
CTTCATTGAGAAGCGCAAGAGGCGACTGATACTGTGGATGAACCACATGA
CCAGTCACCCAGTCCTCTCCCAGTATGAAGGCTTCGAGCACTTTCTGATG
TGTGCTGATGACAAGCAGTGGAAACTGGGCAAGAGACGGGCAGAGAAGGA
TGAGATGGTGGGTGCCCATTTTATGCTGACCCTACAGATCCCTAACGAGC
ACCAGGACCTTCAGTATGTGGAGGAGCGGATCGACTCCTTCAAGGCCTTT
GCTAAGAAAATGGATGACAGCGTGATGCAGCTCACGCATGTTGCCTCGGA
GCTCGTGCGTAAGCACTTGGGTGGGTTCAGGAAGGAGTTCCAGCGACTGG
GAAATGCCTTCCAGTCNNNNNNNNNNNNNNNNNNNNNNNNNNNNNNNNNNNNNNNNNNNNNNNNNN
NNNNNNNNNNNNNNNNNNNNNNNNNNNNNNNNNNNNNNNNNNNTGGGTTTCATCATTGGAGTTGGCGTG
GTTGGAAA
CCTTCTGATCTCCATCCTGCTTGTCAAAGACAAGAGCCTGCACCGAGCGC
CCTACTATTTCCTGCTGGACCTGTGCGCCTCTGATATCTTACGCTCCGCC
ATCTGCTTCCCCTTTGTCTTCACCTCGGTCAAGAATGGATCTGCCTGGAC
GTATGGCACGCTGACCTGCAAAGTGATCGCCTTCCTGGGTGTGCTCTCCT
GTTTCCACACGGCGTTTATGCTGTTCTGTGTCAGTGTCACGCGCTACCTG
GCCATCGCACATCACCGTTTCTACACCAAGAGGCTGACCTTCTGGACCTG
TCTGGCTGTCATCTGCATGGTGTGGACGTTGTCAGTGGCTATGGCGTTCC
CGCCGGTGCTAGACGTAGGGACGTACTCTTTTATCCGAGAGGAGGACCAG
TGCACGTTCCAGCACCGTTCCTTCAGGGCGAATGATTCGCTGGGCTTCAT
GCTCCTGCTGGCGCTCATCCTCCTGGCCACACAGCTGGTTTACCTCAAGC
TCATCTTCTTCGTTCACGACCGTCGAAAGATGAAACCTGTCCAGTTCGTG
CCTGCTGTCAGCCAGAACTGGACCTTCCACGGGCCTGGCGCCAGCGGGCA
GGCGGCGGCCAACTGGCTGGCCGGATTCGGTCGAGGCCCCACCCCGCCAA
CTTTGCTGGGCATCCGGCAGAACAGCAACGCAGCAGGCCGCAGGCGTCTA
CTGGTATTGGATGAATTCAAAACAGAGAAGAGGATTAGTAGGATGTTCTA
CATCATGACGTTTTTCTTCCTGGCACTGTGGGGGCCCTATTTGGTCGCCT
GCTACTGGCGAGTGTTCGCAAGGGGCCCCGTGGTCCCTGGGGGCTACCTG
ACGNNNNNNNNNNNNNNNNNNNNNNNNNNNNNNNNNNNNNNNNNNNNNNNNNNNNNNNNNNNNNNNNNN
NNNNNNNNNNNNNNTTTCACCCTGGCATGGGGACTGGTTCTGGCACGGAGC---GCAGGGT
TCCACTTGGCAACAGCTTGCTATCCCCGCAGCAAACCGAGGAGCCCACTG
TTGCCCCCCCCCCGCAGCGATGGTTTGTCACCC---CTGCCAACAACCGA
CTGGACTTTGCTGCCTCGGCATACGACGCTGCT---------GATTTCGC

CGGTAACGCGGCCACCTTGCTGTCCTACGCAGCGGCCGGAGTGAAGGCAC
------TTCCCCTGCCGACTGCAGGCTGCTCCAACCGGCCTCTTGGCTAT
TACGCAGACCCATCAG---GCTGG---GGAGGACGTACGCCGCCGCAGTA
CTGTGGCGTAAATAGCAAATCCAGTTCGGTCTTTTCCTGCTGGCCCACTA
ACTCTATCGGTGGCAGAGCGGGCA---CC---AACTACCTGG--------
-CTGAGGA---GGGA---GACTC---CATACCGACAGAGAGGTCACCG--
-AT---AGGCGGCTCAGAGGAG---ACCAAACCCAAAGAAATGAC---AC
CAGA---GTCGAACTGGATAGAG---ACGCCGTCCTCCATTAAGTCCATC
GATTCAAGCGATTCTGGTATCTTTG---AACAGGCCAAAAGGAGAAGGCT
CTCACNNNNNNNNNNNNNN---------
NNNNNNNNNNNNNNNNNNNNNNNNNNNNNNNCATCACTCAACAGGCGAAGTCACAGAGAGAGAAGTG
GCRTTGGGGATAAATCCGTTCGCGGATGGAATGGGCGCCTTCAAAATAAA
CCACAGTTCCCACGATATTGGCTCCGG---ACAAACGGCGTTTTCCTCCC
AGGCG---CCCGGCTAC---GCAGCAGCCGCCCTGGGA---CACCATCA-
-----CCACCCGACCCACGTTGGCTCT---TACTCCACGGCGGCTTTCAA
CTCCACCAGGGACTTTCTCTTCAGAAATCGGGGATTCGGGGACGCCACCA
G------------CGCGCAGCACAGTTTGTTCGCCTC---------CGGA
AGTTT---C------GCAGGGCCACATGGACACTCAGATGCAGCGGGGCA
CCTGCTCTTCCCGGGGCTCCACGAG---CAAGCTGCGAGCCATGCGTCTT
CCAACGTGGTCAACAGCCAGATGCGGCTGGGCTTCTCGGGGGACATGTAC
GGACGGGCCGACCAGTACGGCCACGTTACGAGCCCGCGGT---CCGACCA
CTATGCCTCGACCCAGCTGCACGGCTACGGCCCCATGAACATGAATATGG
CCGCC---CACCACGGAGCAGGGGCCTTCTTTCGATACATGAGGCAGCCG
ATCAAACAAGAGCTCATCTGCAAGTGGATCGAGCCGGAGCAGCTGACAAA
TCCCAAAAAGTCGTGCAACAAAACTTTTAGCACGATGCACGAGCTCGTGA
CCCATCTGACGGTGGAGCATGTGGGGGGACCGGAGCAGACCAACCACGTC
TGCTTCTGGGAGGATTGCGCCAGAGAAGGAAAGCCATTCAAAGCCAAATA
CAAACTTGTAAATCATATCAGAGTACACACCGGAGAAAAGCCCTTCCCGT
GTCCGTTCCCCGGCTGTGGCAAA
>Maccullochella peelii
AGTTTACTCATCCGAGCAGAACTAAACCAACCAGGCCCATTGCTTGGAGA
TGACCAACTTTACAATGTAATCGTTACAGCCCATGCATTCGTAATAATCT
TTTTTATAGTAATACCAGTCATAATTGGTGGATTTGGAAACTGACTAGTT
CCTTTAATAATTGGCGCCCCAGATATGGCTTTCCCCCGGATAAATAACAT
AAGCTTCTGGCTACTCCCCCCATCCCTCCTCCTCTTACTTGCCTCCTCAG
GAGTAGAAGCCGGAGCCGGTACAGGCTGAACTGTATACCCACCCTTATCA
GGAAATTTAGCTCATGCCGGAGCATCTGTTGATTTAACTATTTTCTCCCT
TCATTTAGCAGGGATCTCTTCAATCCTTGGAGCCATTAATTTTATCACAA
CTATCATTAATATAAAACCTCCTGCCATCTCACAATATCAAACCCCATTA
TTTGTATGATCAGTACTAATCACTGCCGTCCTCCTACTACTATCACTCCC
CGTTCTTGCTGCCGGGATTACAATGCTTCTCACAGATCGAAATCTTAACA
CTACCTTCTTCGACCCAGCAGGTGGAGGTGACCCAATTCTTTACCAACAC
CTATTC--

------------TTCCTAGAGAGAAACCTTCACCCATCTAACTGCCTTGG
CATGCTGTTGCTGTCTGACGCCCACCAGTGCACCAAGCTATCAGAGCTCT
CCTGGGGCATGTGCCTGAGCAACTTTCCCGCTATTTGCAAGACAGAGGAC
TTCCTCCAACTGCCCAAAGATATGGTGGTGCAGCTTTTGTCACATGAGGA
GCTAGAGACAGAAGATGAGAAACTGGTTTATGAAGCTGCCCTTAACTGGA
TCAACTATGACCTGGAAAAGAGGCACTGCAACCTTCCAGAGCTCCTGAGA
ACGGTCCGTCTGGCCCTGCTGCCTGCCATCTTTCTCATGGAGAACGTATC

TACAGAAGAGCTGATCAACGCCCAAGCCAAGAGCAAGGAACTGGTGGATG
AAGCTATCCGGTGTAAGCTGAAGATCCTGCAGAACGATGGCGTTGTTAAC
AGCCCGTGTGCTCGACCAAGAAAAACCAGCCATGCCCTCTTTCTTCTGGG
AGGACAGACTTTCATGTGTGACAAGTTGTATTTGGTGGACCAGAAAGCCA
AAGAGATCATCCCCAAGGCTGACATTCCCAGCCCCAGGAAAGAGTTCAGC
GCCTGCGCCATCGGCTGTAAAGTGTACATCACTGGTGGGA--GAGGCTC-
AGAGAATGGTGTGTCCAAAGATGTATGGGTCTATGACACCGTCCATGAGG
AATGGTCGAAGGCGGCACCCATGCTCATTGCCAGGTTTGGCCATGGCTCT
GCTGAGCTGAAACACTGCCTCTACGTGGTAGGAGGTCACACCGCAGCGAC
TGGCTGCCTCCCGGCCTCTCCGTCTGGATGAATACATTGTTGTGTTTAGT
CGTTCATCAACGAGGCTGATACTGAATGAAGCAGAGCTAATCATGGCGCT
GGCCCAGGAGTTCCAGATGAGAGTGGTCACGGTATCCCTAGAGGAACAGT
CCTTCCCCAGTATCGTTCAGGTGATCAGCGGTGCTTCCATGTTAGTCAGT
ATGCATGGAGCTCAGCTTATCACCTCACTCTTCCTCCCCAGAGGAGCTGC
TGTGGTGGAGCTGTTCCCTTTTGCTGTTAACCCAGAGCAGTACACCCCAT
ATAAAACCCTTGCCTCGCTTCCAGGCATGGACCTTCACTATGTCTCCTGG
AGGAACACTAAGGAGGAGAACACCATCACCCACCCAGACAGACCCTGGGA
ACAAGGGGGCATTGTTCACTTGGAGAAGGAGGAGCAAGAGCGAATACTGG
CAAGCAAAGATGTCCCCAGGCACTTGTGCTGCCGCAACCCAGAGTGGCTC
TTCCGGATCTACCAGGACACTTTGGTGGACATCCCTTCCTTCCTGGAAGT
GCTCAA---AGAGGGCATGAAG---ACAAAGCCCAGCCTGAAGAA---GT
CAAAGCCGGCCAGCACAGTCCACCCGGGTCGGGTCAGAGAACCCCAGTGT
CAGACCTCAGTACAAACCACTAATGAGGCTAAACTCACAGTCTCCTGGCA
GATCCCGTGGAATCTGAAATACCTAAAGGTGAGAGAGGTGAAGTACGAAG
TGTGGATCCAGAAAAAAGACACCAGCAAAGGGACACTGGAGGATCAAATC
ATCCAGGCAAACCCTGCCCTGGAGGCCTTCGGTAACGCCAAAACGCTGAG
AAACGACAACTCGTCTCGTTTTGGTAAATTCATCCGAATTCACTTCGGTA
CGAGCGGTAAGCTGTCATCTGCTGACATTGAGACGTACCTGCTGGAGAAG
TCACGTGTCACCTTTCAGCTCAAGGCTGAGAGGAACTACCACATATTCTA
CCAGATCCTGTCCAATCAGAAGCCAGAGCTGCTGGACCTGCTGCTGATCA
CCAACAACCCGTACGACTACTCCTACATCTCCCAAGGAGAGGTAACAGTC
GCCTCCATTAATGACTCGGAGGAGCTGATGGCCACCGACAGTGCCTTTGA
TGTGCTTGGCTTCACTCCAGAAGAGAAGATGGGTGTCTATAAACTGACTG
GCGCCATCATGCACTATGGCAACATGAAGTTCAAACAGAAGCAGCGGGAG
GAGCAGGCTGAGCCGGATGGGACGGAGGCTGCTGATAAATCAGCTTACCT
AATGGGGCTGAACTCTGCTGACCTCATCAAAGGGCTGTGCCATCCCAGAG
TCAAGGTAGGAAATGAATACGTCACCAAAGGACAAAATGTGGACCAAGTC
TACTACCCCAACAAGGAGGCCTTCAAGTGTGAGGAGTGTGGGAAGCACTA
CAACACCAAGCTGGGATATAAGCGCCATGTGGCCATGCACTCTGCCACAG
CAGGGGATCTCACCTGTAAAGTGTGCATGCAGACCTTCGAGAGCACACCC
GTACTCTTGGAGCACCTCAAGAGCCACTCCGGGAAGTCTTCGGGTGGCGC
CAAGGAGAAAAAACACCCGTGCGACCACTGTGACCGTTGTTTCTACACAC
GGAAGGATGTGAGACGGCACATGGTGGTCCACACGGGCCGAAAGGACTTC
CTGTGCCAGTACTGTGCCCAGCGCTTCGGCAGGAAGGACCATCTGACACG
CCACGTGAAGAAGAGCCACTCACAGGAGCTGCTGAAGATCAAGACGGAGC
CTCCTGATATGTTAGGTCTTTTAGCTTCGGGGTCACCGCCTTGCTCTGTG
AAGGAGGAGCTCAGCCCCATGATGTGTGGAATGGGTCCCAACAAAGACCC
CATGATGGGCAAACCATTCCCCAGTGGTGCCCCTTTTCCGATGGGCATGT
ACAACCCCCACCAT------CTCCAGGCGATGTCTAATTCTGGGGTGGGT
CACCCA------CACCCGTCCCTGATGCCCAGTTCCTTGTCTGCAGCTAT
GGGCATGAGCTGTCACATGGAATATCTCATCTACGCCTCTTTCTCATTTA
TGGGATGTTTACAAATCAGTGATGGATCAAATATCGTGAACCTGCTGGCT
AGTAACTCTCCGAGTGTTTCGTATGCTCTGACCCAGCAGAAATACTTCAG

TAACTACAGCCCCGTGATTGGGTTTTACATTTACGAGCCCATCGAGTACT
GGAACTCCACGGTGCAGGAGCACCTGAAGACTCTGAGTCATGGCTTCAAC
AAGATCTCCTGGATGGACAACTTTTTCCACTACCTGCGGGTGGTGAATGT
GAGTACGTCAACCAAGAGCGACTTCATCACCATCCTCAAGGGCTCCTTCC
TGCGCAGCCCGGAGTACCAGCACTTCACTGAGGACATCATATTCTCCAAG
A---ACCGTGAGACTG------ATGAGTACGACATTATCGCCTCACGGAT
GTACTTGGTGGCACGGACGACAGAGAAGAAGCGCGAAGAGGTGGTGGAGC
TTCTGGAAAAGCTTCGTCCGTTGATGCTAATCAACAGCATCAAGTTCATT
GCCTTCAATCCTACATTTGTGTTCATGGACCGCTACAGTTCCTCTGTCAT
CTCGCCCATCCTGACCTCAGGCTTCAGCGTACTCACAATCCTCATCCTCA
CTTTCTTCCTGGTCATCAACCCCTTGGGGAACTTCTGGCTCATCCTCACR
GTAACGTCCGTGGAGCTGGGCGTCTTGGGTTTGATGNNNNNNNNNNNNNNNNNNNNGCAGCCCGCTCTCA
AGAATGTGTCTACATCTTACAATGTTGGCATTATTAATGGGCTCTCTGGA
TGGGCTTCCTCGGTGGATGACGCTCCAGCTGACACCATCACTCGGCGGTT
TCGCTATGATGTGGCACTGGTGTCAGCATTAAAGGATCTGGAGGAGGACA
TCATGGAGGGGCTGAGAGAGAGTGGGATGGAAGACAGTGCTTGCACCTCA
GGCTTCAGTGTCATGATCAAGGAATCTTGTGATGGCATGGGCGATGTCAG
CGAGAAGCATGGTGGAGGACCAGTTGTTCCCGAGAAGGCTGTACGTTTCT
CTTTCACTGTTATGTCTGTCTCTGTCCTGGCAGACGATGAGGAG------
------------------------GAAGAGGTTACCATCTTCACTGAGCC
AAAGCCAAACTCAGAACTGTCCTGTAAGCCCCTTTGCCTGACATTTGTGG
ATGAGTCAGACCATGAGACACTCACAGCTGTCCTGAGCCCTATAGTTACA
GAGCGTAATGCAATGAAAGACAGCAGGCTCATCCTATCCATGGGTGGACT
ACCTCGTTCCTTCCGCTTCCACTTCAGAGGCACAGGATACGATGAGAAGA
TGGTGCGTGAGATGGAGGGCCTCGAGGCCTCAGGCTCCACCTATATCTGC
ACTCTTTGCGACTCCAGTCGGGCAGAGGCCTCTCAAAACATGGTGCTACA
CTCTGTCACCCGCAGTCATGAAGAGAACTTGGAACGTTACGAGATATGGA
GAACCAACCCCTTTTCTGAGTCTGTAGATGAGCTGCGAGACAGAGTCAAA
GGTGTCTCTGCCAAGCCCTTCATGGAGACCCAGCCCACACTGGATGCATT
ACACTGTGACATTGGAAATGCCACTGAGTTCTACAAAATCTTCCAGGACG
AGATCGGGGAGGTGTACAAAAAGGT---CAAC---CCCAGCCGGGAGGAA
CGGCGCAGCTGGAGGGCAGCCCTAGATAAACAGCTGAGGAAGAAGATGAA
GCTTAAACCGGTGATGAGGATGAATGGGAACTATGCCCGCAGGCTAATGA
CCCAGGAGGCTGTGGAGGTGGTGTGTGAGCTGGTGCCCTCAGAGGAGAGG
AGGGAGGCGCTGAGGGAGCTTATAAGGCTCTACCTCCAGATGAAGCCTGT
GTGGCGCGCCACCTGCCCAGCGAAGGAGTGCCCCGACCAGCTGTGCCGCT
ACAGCTTTAACTCCCAGAGCTTTGCCGACCTCCTCTCCTCTACCTTCAAA
TATAGGTACAATGGAAAGATAACCAATTACCTGCACAAGACCCTGGCCCA
TGTGCCTGAAATCATAGAGAGAGATGGATCCATAGGAGCCTGGNNNNNNNNNNNNNNNNNNNNNNNNNNN
NNNTCGTACA
CCATCGATATGGGTCCTTTGGGGCCCCGGTGGAAGGAGAGCCCACAGCCT
TTCTCCTGCTCCATTGAAGACCCCACAAAACAGACAAAGTTCAAGGGCAT
CAAGACGTACATTTCGTACCGGGTCACGCCGAGCCACACAGCTCATCCTG
TCTACAGGCGCTACAAACACTTTGACTGGCTGTACAACCGCTTACTGCAC
AAGTTCACTGTGATCTCCGTGCCTCACCTGCCTGAGAAGCAGGCCACGGG
GCGATTTGAGGAAGACTTCATCGAGAAGCGAAAGAGACGACTGATACTGT
GGATGAACCACATGACCAGTCACCCAGTCCTCTCCCAATATGAAGGCTTT
GAGCACTTCCTGATGTGTGCTGATGACAAGCAGTGGAAACTGGGCAAGAG
ACGGGCAGAGAAGGACGAGATGGTGGGTGCCCATTTCATGCTGACCCTCC
AGATCCCTAACGAGCACCAGGACCTTCAGGATGTAGAGGAGCGGATTGAC
ACCTTCAAGGCATTTGCTAAGAAAATGGATGATAGCGTGATGCAGCTCAC
ACATGTTGCCTCAGAGCTGGTGCGTAAGCACCTGGGTGGGTTCAGGAAGG
AGTTCCAGCGGCTGGGAAATGCCTTCCAGTCGATCAGCCAGGCGTTCATG

CTGGACCCTCCCCACAGCTCAGAGACCTTCAACAACGCCGTCTCCCATNNNNNNNNNNNNTT
CCTCAAACTGACCTCTCTGGGTTTCATCATTGGAGTCGGTGTGGTTGGAA
ACCTCCTGATCTCCATCCTGCTGGTCAAAGACAAGAGCCTGCACCGAGCG
CCCTACTACTTCCTGCTGGACCTGTGCGCCTCTGATATCCTGCGCTCTGC
CATCTGCTTCCCCTTTGTCTTCACCTCGGTCAAGAATGGATCTGCCTGGA
CCTATGGCACGCTGACCTGCAAAGTGATTGCCTTCCTGGGTGTGCTGTCC
TGTTTCCACACGGCGTTTATGCTGTTCTGTGTCAGTGTCACACGCTACCT
GGCCATCGCACATCACCGTTTCTACACCAAGAGGCTGACCTTCTGGACCT
GTCTAGCTGTCATCTGCATGGTGTGGACATTGTCAGTGGCTATGGCGTTC
CCGCCGGTGCTAGACGTAGGGACGTACTCTTTTATCCGGGAGGAGGACCA
GTGCACATTCCAGCACCGTTCCTTCAGGGCGAATGATTCGCTGGGCTTCA
TGCTCCTGCTGGCACTCATCCTCCTGGCCACACAGCTGGTTTACCTCAAG
CTCATCTTCTTCGTCCACGACCGTCGAAAGATGAAGCCTGTCCAGTTTGT
GCCTGCTGTCAGCCAGAACTGGACCTTCCACGGGCCCGGCGCCAGCGGGC
AGGCGGCGGCCAACTGGTTGGCCGGATTTGGTCGAGGCCCCACCCCGCCT
ACTTTGCTGGGCATCCGGCAGAACAGCAACGCAGCGGGCCGCAGGCGTCT
ACTGGTATTGGATGAATTCAAAACAGAGAAGAGGATTAGTAGGATGTTCT
ACATCATGACGTTTTTCTTCCTGGCGCTGTGGGGGCCCTATCTGGTCCCC
TGCTACTGGCGGGTGTTTGCAAGGGGCCCTGTGGTCCCTGGGGGCTACCT
GACGGCAGCCGTGTGGATGAGCTTTGCCCAGGCTGGGGTCAATCCTTTCA
TCTNNNNNNNNNNNNNNNNNNNNGCCAAATCTCGCTTTCACCCTGGCATGGGGACTGGTCCTGGCACGGA
GC---GCAGCGTCCCACTCGGCAACAGCTTGCTATCCCCGCAGCAAACCG
AGGAGCCCACTGTTGCCACCCCCCCGCAGCGATGGTTTGTCACCC---CT
GCCAACAACCGACTGGACTTTGCTGCCTCGGCATACGACGCCGCT-----
----GATTTCGCCGGTAACGCGGCCACCTTGCTGTCCTACGCAGCGGCCG
GAGTGAAAGCTC------TTCCCCTGCCGACTGCAGGCTGCTCCAACCGG
CCTCTTGGCTATTACGCAGACCCGTCAG---GCTGG---GGAGGACGCAC
GCCGCCGCAGTACTGTGGCGTAAATAGCAAATCCAGCTCGGTCTTTTCCT
GCTGGCCCGCTAACTCTATCGGTGGAAGAGCGGGCA---CC---AACTAC
CTGG---------CTGAGGA---GGGA---GACTC---CATCCCGACAGA
GAGGTCACCG---AT---CGGCGGCTCGGATGAG---ACCAAACCCAAAG
ACATGAC---ATCAGA---GTCGAGCTGGATAGAG---ACGCCGTCCTCC
ATTAAGTCCATTGATTCAAGCGATTCTGGTATCTTTG---AACAGGCCAA
AAGGAGAAGAATCTCACCTTCTGCCACGCCG---------GTTTCAGAGA
CAGTGTCCCCTTTAAAATCTGAGCATCACTCAACAGGCGAAGTCACAGAG
AGAGAAGTGGCGTTGGGGATAAATCCGTTCGCGGATGGGATGGGCGCCTT
CAAAATAAACCACAGCTCCCACGATATTGGCTCCGG---ACAAACGGCGT
TTTCCTCCCAGGCG---CCCGGCTAC---GCAGCAGCAGCCCTGGGA---
CACCATCA------CCACCCAACCCATGTTGGCTCT---TACTCCACGGC
GGCTTTCAACTCCACCAGGGACTTTCTCTTCAGAAATCGGGGTTTTGGGG
ACGCCACCGG------------GGCGCAGCACAGTTTGTTCGCCTC----
-----CGGAAGTTT---C------GCAGGGCCACATGGACACTCAGATGC
AGCGGGGCACCTGCTCTTCCCAGGGCTCCACGAG---CAAGCGGCGAGCC
ATGCGTCTTCCAACGTGGTCAACAGTCAGATGCGGCTGGGCTTCTCGGGG
GACATGTACGGACGGGCCGACCAGTACGGCCACGTTACAAGCCCGCGGT-
--CCGACCACTATGCTTCGACCCAGCTGCACGGCTATGGCCCCATGAACA
TGAATATGGCCGCA---CACCATGGTGCAGGGGCCTTCTTTCGATACATG
AGGCAGCCGATCAAACAAGAGCTCATCTGCAAGTGGGTCGAGCCGGAGCA
GCTGACGAATCCCAAAAAGGCGTGCAACAAAACTTTTAGCACGATGCACG
AGCTTGTGACCCATCTGACGGTGGAGCATGTGGGGGGACCGGAGCAGACC
WACCACATCTGCTTCTGGGAGGAATGCGCCAGAGAAGGAAAGCCATTCAA
AGCCAAATACAAACTTGTAAATCATATCAGAGTACACACCGGAGAAAAGC
CCTTTCCGTGTCNNNNNNNNNNNNNNNNNNN

```
>Macropinna microstoma
AGCCTGCTAATCCGGGCTGAATTAAGCCAACCCGGGGCCCTCCTAGGGGA
CGACCAAATCTACAATGTTATCGTGACAGCACATGCTTTCGTTATAATCT
TCTTTATAGTAATACCTGTCATAATTGGCGGCTTTGGGAACTGGTTAATC
CCCTTGATAATCGGAGCCCCGGATATAGCATTCCCCCGCATAAATAACAT
AAGCTTCTGACTTCTCCCCCCCTCCTTCCTTCTTCTCCTGGCCTCTTCCG
GGGTTGAAGCCGGAGCTGGCACAGGCTGAACAGTATACCCCCCCTTAGCA
GGAAACCTTGCCCATGCAGGAGCCTCCGTAGACCTAACTATTTTTTCCCT
CCACCTCGCCGGTATTTCCTCAATTCTTGGCTCAATTAACTTCATTACAA
CTATCATCAATATAAAACCCCCGGCCATCTCCCAATACCAAACCCCTCTT
TTCGTCTGAGCCCTACTTATTACTACAGTCCTTCTGCTCCTCTCACTCCC
TGTCCTAGCTGCAGGAATCACTATACTGCTTACGGACCGAAATTTAAATA
CTACTTTCTTTGACCCCGCAGGAGGGGGTGACCCAATCCTCTACCAACAT
CTANNNNNNNNNNNNNNNNNNNNNNNNNNNNNNNNNNNNNNNNNNNNNNNNNNNNNNNNNNNNNNNNNNN
NNNNNNNNNNNNNNNNNNNNNNNNNNNNNNNNNNNNNNNNNNNNNNNNNNNNNNNNNNNNNNNNNNNNNN
NNNNNNNNNNNNNNNNNNNNNNNNNNNNNNNNNNNNNNNNNNNNNNNNNNNNNNNNNNNNNNNNNNNNNN
NN-----------------------------------------------
--------------------------------------------------
--------------------------------------------------
--------------------------------------------------
--------------------------------------------------
--------------------------------------------------
--------------------------------------------------
--------------------------------------------------
--------------------------------------------------
--------------------------------------------------
--------------------------------------------------
--------------------------------------------------
--------------------------------------------------
--------------------------------------------------
--------------------------------------------------
--------------------------------------------------
----------------GGAGGAGTACATAGTGGTGTTCAGCCGTTCAATT
AGCAGGCTGATTCTTAACGAAGCAGAGCTGATTCTGGCCCTAGCACAGGA
GTTCCAGATGAAGGTTGTAACTGTGTCTCTGGAGGAGCAGTCCTTTGCCA
GTATAGTGAAGATGGTCAGTGAGGCCTCCATGTTAGTCAGTATGCACGGA
GCCCAACTGGTGTCCTCTCTCTTCCTGCCCAGGGGGGCCGCAGTGGTCGA
GCTCTTCCCCTATGCAGTGAACCCAGAGCAGTACGCTCCCTACAAAACCT
TGGCCTCACTCCCTGGCATGGACCTGCAGTATGTGGCCTGGAGGAACATG
ATAGAGGAGAACTCAGTGGCCTACCCTGAGAGGCCTTGGGAGCAGGGAGG
CATCGCCCATTTGGAGAAGGACGAGCAAGAGCGCATCTCGGCCAGCAAAG
AGGTACCAAGGCACCTGTGCTGCCGCAACCCAGAGTGGCTCTACCGTATC
TACCAAGACACCATAGTGGACATCCCGTCTTTACTGGAGACACTCAG---
AGAGACCCTGAAA---ACCCGGCCAAACCTGAAGAA---GGCCAAGCCTG
CCAGCACGGTCCACCCGGGCCGAGTGAGGGAGCCCCAGTGCCAGACCTCG
GTCCAGGCCACCAACGAGGCCAAGCTCACCGTGTCCTGGCAGATCCCCTG
GAACCTGAAGTACCTGAAGGTCAGGGAGGTTAAATACGAAGTGTGGATAC
AGAAGAGGGATCCAAGCAAGGGTACCCTAGAGGATCAAATCATCCAGGCT
AACCCTGCACTGGAGGCTTTCGGTAATGCCAAAACATTGAGAAATGATAA
TTCATCACGTTTTGGCAAATTCATCCGTATTCATTTCGGACAAAGTGGCA
AATTGTCCTCTGCTGACATAGAGACTTATCTGCTTGAGAAGTCACGTTGC
ACCTTTCAGCTGAAATCGGAGAGGAACTACCATATCTTCTTCCAGATCTT
GTCCAATCAAAAGCCAGAACTGTTGGACATGCTGTTGATCACCAACAATC
```

CATATGACTACGCCTACATCTCCCAAGGAGAGGTGACAGTAGCATCCATC
AATGATTCTGAGGAGCTGATTGCCACTGACAGTGCCTTTGATGTGCTGGG
CTTTACTGCAGAGGAGAAAATGGGGATCTACAAGTTGACAGGTGCCATAA
TGCACTATGGCAACATGAAGTTCAAGAACAAGCAGCGGGAGGAGCAGGCA
GAGTCTGACGGTACAGAGGCTGCTGACAAGTCAGCTTATCTAATGGGACT
GAACTCTGCAGATCTTATTAAAGGACTGTGCCATCCAAGAGTCAAGGTTG
GCAATGAGTATGTCACCAAAGGTCAGAGTGTAGATCAAGTCTACTAC---

---------------------------GGCTACCACCCCTTCGAGTGGCA
GCCGGCCCTCAAGAACGTGGCCACATCATGCCAGGTAGGCATCATCGATG
GGCTCTCAGGGTGGACGGGCTCAGTGGATGATGCCCCTGCGGATACCACA
ACCCGGCGGTTCCGCTATGACGTGGCCCTGGTGTCGGCCCTGAAGGACCT
GGAGGAGGACATCATGGAGGGGCTGAGGGAGCGAGGGCTGGAGGACAGCG
CCTGCACCTCTGGCTTCAGTGTGATGATCAAGGAGTCCTGCGATGGCATG
GGGGACGTCAGTGAGAAGCACGGCGGAGGACCGCCCATCCCTGAGAAGGC
TGTGCGCTTCTCCATTACCATCATGTCTGTCTCTGTCCAGGCTGAAGGAG
AGGAG------------------------------GAGGCAGTCACTATC
TTCCGGGAGCCCAAGCCGAACTCTGAACTGTCCTGTAAGCCACTGTGCCT
GATGCTTGTGGACGAGTCGGACCACGAGACACTCACGGCAGTCCTAGGGC
CTGTGGTGGCCGAGCGGAACTCCATGAAGCACAGCCGGCTCATTCTGTCC
CTGGGTGGGCTCCCTCGCTCCTTCCGCTTCCACTTCAGAGGAACGGGTTA
TGATGAGAAGATGGTGCGTGAGATGGAGGGCCTGGAGGCCTCTGGCTCCA
CCTACATCTGCACCTTGTGTGACTCCACCAGGGCAGAGGCCTCCCACAAC
ATGGTGCTCCACTCCATCACCCGCAGCCACGACGAGAACCTGGAGCGCTA
TGAGTTGTGGAGGACCAATCCCTTCTCAGAGTCCGCAGAGGAGCTGCGGG
ACCGGGTCAAAGGTGTCTCTGCCAAGCCCTTCATGGAGACCCAGCCCACA
CTGGATGCCCTGCACTGCGATATCGGCAACGCCACTGAGTTCTACAAGAT

CTTCCAGGATGAGATCGGGGAGGTGTACCGCAGCCC---CAAC---CCCA
GACGGGAGGAGCGGCGGAGCTGGAGAGCCGCTCTGGACAAGCAGCTGAGG
AAGAAGATGAAGTTGAGGCCGGTGATGCGTATGAACGGGAACTACGCGCG
GCGGCTGATGACCCGGGAGGCAATAGAGGTGGTGTGTGAGCTGGTGCCGT
CAGAGGAGAGGCGAGAGGCCCTGAGGGAGCTGATGGGGCTCTACCTCCAG
ATGAAGCCAGTGTGGCGTTCCTCCTGCCCAGTCAAGGAGTGCCCAGACCA
GCTGTGTCGCTACAGCTTCAACTCCCAGCGCTTTGCAGAGCTCCTCTCCA
CCACCTTCAAGTACAGGTATTGT---------------------------

----------------------------------TCGTACACCATCGAGA
TGGGCTCCAAAGGTCCTCAATGGAAAGAGAGTCCCCAGCCTTTCTCTTGC
TCCATCGAAGACCCCACCAAGCAGACAAAGTTTAAAGGCATCAAGACCTA
CATATCGTACCGGGTAACCCCCAGCCACATGGGGCGGCCTGTGTACCGGC
GCTACAAGCACTTTGACTGGCTGTACAATCGCCTGCTGCATAAGTTTACG
GTCATATCTGTGCCCCACCTGCCCGAGAAACAGGCCACGGGGCGCTTCGA
GGAGGATTTCATTGAGAAGCGTAAAAGGCGACTGGTCCTCTGGATAAACC
ACATGACCAGCCACCCAGTCCTGTCTCAGTACGAAGGCTTCGAGCACTTC
CTGATGTGTGCCGATGACAAGCAGTGGAAGCTGGGTAAGCGGCGGGCAGA
GAAGGACGAGATGGTGGGCGCCCATTTCATGCTCACCTTCCAGATCCCCG
CGGAGCACCAAGACCTTCAGGATGTGGAGGAGCGCGTGGACAACTTCAAG
TCCTTCGCCAAGAAAATGGATGACAGTGTCATGCAGCTGACGCACGTGGC
GTCGGAACTGGTGCGGAAGCATCTCGGAGGATTCCGGAAGGAGTTTCAGC
GGCTGGGGAACGCATTCCAGTCCGTCAGTCAGGCATTCATGCTGGACCCT
CCTCACAGCTCAGACGCCCTCAACAATGCCANNNNNNNN-------------------

------------------GCCAAATCTCGCTTTCACCCTGGCGTAGGGAC
TGGTCCTGGCACAGACC---GCAGCGTCCCACTTAGTAACAGCTTGCTAT
CCCCGCAACAAACCGAAGAGCACACAGTTG---CTTCCCCGCAGCGATGG
TTTGTCACCC---CTGCCAATAACCGACTGGACTTTGCCGCCTCGGCATA
CGATGCCGCCGCGGCTGCTGATTTTGCCGGTAACGCGGCCACCTTGCTGT
CCTACGCAGCGGCTGGAGTAAAAGCAC------TTCCCCTGCCCACTGCT
GGGTGTTCAAACAGACCTTTGGGTTATTATGCCGATCCATCCG---GCTG
G---GGCACCCGCACACCACCTCAGTACTGT---------AGTAAATCCA
GCTCAGTGCTCTCGTGCTGGCCCACAAATGCTGTTGGAAGCCTTACAGGC
A---CGTCCAATTACCTGG---------CGGAGGA---TGGG---GACAC

---CATCCCTACGGAGAGGTCTCCA---AT---CGGAGTGCCAGAGGAG-
--ACAAAACCAAAAGACTT------GTCCGA---ATCCAGCTGGATAGAG
---ACGCCGTCTTCAATAAAGTCGATCGATTCAAGTGATTCTGGAATCTT
TG---AGCAGGCCAAACGGAGAAGAATTTCACCGTCTGCCACACCG----
-----GTTACGGAGACAGTNNNNNNNNNNNNNNNNNNN---------ACAGGCGAAGTCACAGACAGAG
AAGTGGCTTTGGGGATAAATCCGTTCGCAGACGGGATGGGCGCTTTTAAA
ATCAACCACAGCTCTCATGATCTTGGCTCCGG---GCAAACGGCGTTTTC
CTCCCAAGCG---CCCGGCTAC---GCAGCCGCTGCCTTGGGA---CATC
ATCA------CCATCCGACCCATGTCAGCTCC---TACTCCACCGCCGCT
TTCAATTCCACCCGGGACTTTCTTTTTCGAAATCGGGGCTTCGGAGACGC
GACCAG------------CGCCCAACACAGTCTGTTCGCCTCCGC---AG
CGGGAAGTTT---T------GCAGGGCCACATGGACACTCAGATGCCGCG
GGACACCTGCTCTTCCCAGGACTTCACGAG---CAAGCCGCGAGCCATGC
GTCCTCCAATGTTGTAAACAGTCAGATGCGATTGGGTTTTTCGGGGGACA
TGTACGGGCGGGCTGACCAATATGGCCATGTTACGAGCCCACGGT---CC
GACCACTACGCTTCGACCCAGTTGCATGGCTATGGCCCCATGAACATGAA
TATGGCCGCA---CACCACGGAGCAGGGGCCTTCTTCCGTTACATGAGGC
AGCCGATCAAACAAGAGCTCATCTGCAAGTGGGTCGAGCCGGAGCAGTTG
TCGAACCCGAAAAAGTCGTGCAACAAAACTTTCAGCACGATGCACGAGCT
CGTGACCCACCTGACAGTGGAACATGTGGGGGGACCAGAGCAGTCAAACC
ATATTTGCTTTTGGGAAGAGTGTTGCCGAGAAGGAAAACCATTCAAAGCT
AAATACAAACTTGTAAATCATATCAGAGTACACACCGGAGAAAAACCGTT
CCCATGTCCATTCCCCGGCTGTGGCAAA
>Macroramphosus scolopax
AGCTTACTTATCCGAGCAGAACTAAGCCAACCCGGCGCCCTTCTTGGGGA
TGACCAAATTTATAACGTAATTGTTACGGCCCACGCCTTTGTAATGATTT
TCTTTATAGTAATACCAATCATGATCGGGGGCTTTGGCAACTGACTTATT
CCCCTAATGATCGGAGCACCCGACATGGCATTCCCTCGAATGAATAACAT
GAGCTTTTGACTACTCCCTCCCTCATTCCTTCTGCTCCTAGCCTCTTCTG
GAGTTGAAGCCGGTGCGGGCACCGGCTGAACAGTCTACCCCCCACTATCA
GGAAACCTCGCTCACGCTGGGGCCTCCGTTGACTTAACCATTTTCTCCCT
CCACTTAGCCGGTATTTCATCTATTCTAGGGGCCATTAATTTCATCACAA
CTATTATTAACATGAAACCTCCCGCAATCTCGCAATACCAAACACCGCTC
TTTGTATGAGCAGTACTAATTACAGCTGTTCTTCTCCTCCTCTCCCTCCC
CGTCCTGGCCGCCGGCATCACAATGCTTCTCACGGACCGAAATTTAAATA
CCACCTTCTTCGACCCCGCAGGTGGAGGTGACCCCATCCTCTACCAACAC
CTA---

------------NNNNNNNNGAGAAACCTTCACCCGTCTAACTGCCTCGGCATGCTGC
TGCTCTCCGACGCCCACCAGTGCACCAAGCTGTCGGAGCTCTCCTGGGGC
ATGTGTCTCAGCAACTTCCCCGCTATTTGCAAGACAGAGGACTTCCTCCA
ACTCCCCAAAGATATGGTGGTTCAGCTTTTGTCCCACGAGGAGCTGGAGA
CAGAAGATGAGCGGCTTGTTTATGAGGCTGCCCTGAACTGGATCAACTAT
GACCTGGACAGGAGGCACTGCCACCTCCCTGAGCTCCTCAGAACCGTCCG
GCTGGCGCTGCTGCCCGCCATCTTTCTAATGGAGAATGTCTCGACGGAAG
AGCTGATCAACGCCCAGGCCAAGAGCAAGGAGCTGGTGGACGAGGCCATC
CGCTGCAAGCTGAGGATCCTGCAGAATGACGGCGTTGTGAACAGCCAGTG
TGCTCGACCCAGAAAGACCAGCCACGCCCTCTTTCTTCTGGGGGGGCAGA
CGTTCATGTGTGACAAGTTGTACCTGGTGGACCAGAAGGCCAAAGAGATC
ATCCCGAAGGCGGACATCCCCAGCCCCAGGAAGGAGTTCAGCGCCTGTGC
CATCGGCTGTAAGGTGTACATCACAGGCGGAC--GAGGCTC-GGAGAACG

GTGTGTCCAAAGACGTGTGGGTCTACGACACCGTCCATGAGGAGTGGTCC
AAAGCGGCGCCCATGCTGATTGCCAGATTCGGTCACGGCTCGGCGGAGCT
GAAACACTGCCTATACGTGGTCGGNNNNNNNNNNNNNNNNNNNNNNNNNNNNNNNNNNNNNNNNNNN---
----

----------AAAAAGGACGCCAGTAAGGGAACACTGGAGGATCAAATCA
TCCAGGCCAATCCTGCACTGGAGGCCTTCGGCAACGCCAAAACACTGAGA
AATGACAACTCGTCTCGTTTTGGAAAATTCATCCGAATTCACTTTGGAAA
CAGTGGAAAGCTGTCATCTGCTGACATTGAGACTTACCTGCTGGAGAAGT
CACGCGTCACCTACCAGCTCAAGGCTGAGAGGAACTACCACATCTTCTAC
CAGATCCTGTCCAAACAGAAGCCGGAACTGCTGGACATGCTGCTAATTAC
CAACAACCCGTACGACTACTCCTACATCTCCCAGGGAGAAGTAACAGTCG
CCTCCATCAACGACTCAGAGGAGCTGATGGCCACCGACAGCGCCTTCGAT
GTGCTCGGCTTCACTCCAGAGGAGAAGATGGCCGTCTACAAACTAACCGG
CGCCATCATGCACTACGGCAACATGAAGTTCAAGCAGAAGCAGCGTGAGG
AGCAGGCGGAGCCCGACGGGACAGAGGCTGCCGATAAGTCTGCTTACCTG
ATGGGACTCAACTCGGCTGACCTCATCAAAGGGCTCTGCCACCCCAGAGT
CAAGGTAGGAAATGAGTACGTCACCAAAGGCCAGAGTGTGGATCAAGTCT
ACTACNNNNNNNNNNNNNNNNNNNNNNNNNNNNNNNNNNGGAAGCACTACAACACCAAGCTGGGATACAA
GCGCCATGTGGCCA
TGCACTCTGCCACTGCAGGGGATCTCACCTGTAAAGTGTGCATGCAGAGC
TACGAGAGCACGCCGATTCTCTTGGAGCACCTCAAGAGCCACTCGGGGAA
GTCCTCGGGCGGCGCGAAGGAAAAAAAACACCCGTGTGACCACTGTGATC
GTCGTTTCTACACACGGAAGGATGTGAGGCGGCACATGGTGGTCCACACT
GGCCGAAAAGACTTCTTGTGCCAGTACTGTGCCCAGCGCTTTGGCAGGAA
GGATCACCTGACTCGCCACGTGAAGAAAAGCCACTCGCAGGAGCTGCTGA
AGATCAAGACGGAGCCCCCTGACATGTTGGGTCTGTTAGCTTCTGGGTCG
CCTCCTTGCAATGTGAAGGAGGAGCTCAGCCCGATGATGTGCGGCATGGG
TCCCAACAAAGACCCCATGATGGGAAAACCGTTCCCCAGTGGGGCCTCTT
TTCCGATGGGCATGTACAACCCCCACCAC------CTTCAGGCCATGTCC
AATCCTGGGGTTGGTCATCCA------CACCCATCCCTGATGCCCAGTCC
CCTGTCTGCAGCTATGGGCATGGGCTGCCACATGNNNNNNNNNNNNNNNNNNNNNNNNNNNNNNNNNNNN
NNNNNNNNNNNNNNNNNNNNNNNNNNNNNNGTAAACCTTCTGGCCA
GCAACTCTCCGAGTGTTTCCTACGCTCTGACCCAGCAGAAATACTTCAGC
AACTACAGTCCTGTGATCGGGTTTTACATTTACGAGCCCATCGAGTACTG
GAACTCCACGGTGCAGGAGCACCTTAAGACTCTGAGTCACGGCTTCAATA
AGATCTCCTGGATGGACAACTTCTTCCACTACCTGCGAGTGGTGAATGTG
AGCGCGTCGACCAAGAGTGACTTCATCACCATCCTCAAGGGTTCCTTCCT
GCGGAGCCCCGAGTACCAGCACTTCACCGAGGACATCATATTCTCTAAGA
---ACCGTGAGACGG------ACGAATACGACATTATTGCCTCACGCATG

TATCTGGTGGCACGGACGACAGAGAAGAAGCGGGAGGAGGTGGTGGAGCT
TCTGGAGAAGCTTCGCCCGTTGATGCTGATCAACAGCATCAAGTTCATTG
CCTTCAATCCCACCTTTGTGTTCATGGACCGCTACAGCTCCTCCGTTATC
TCCCCCATCCTGACCTCAGGCTTCAGCGTGCTCACCATCCTTATTCTCAC
TTTCTTCCTGGTCATCAACCCCTTGGAAAACTTCTGNNNNNNNNNNNNNNNNNNNNNNNNNNNNNNNNNN
NNNNNNNNNNNNNNNNNNNNNNNNNNNNNNNNNNNNNNNNNNNNNNNNNNNNNNNNNNNNNNNTCTTGGA
CAGTTGG
CATTGTTAATGGGCTCTCCGGATGGACGTCCTCGCTGGATGACTCACCGG
CTGACACCATCACTCGGCGATTCCGCTATGATGTTGCTCTGGTGTCAGCG
CTGAAGGATCTGGAGGAGGACATCTTGGAGGGGCTGAGGGAGAGCGGGCT
GGAAGACAGCGCTTGCACCACAGGCTTCAGCGTCATGATCAAAGAGTCTT
GCGACGGCATGGGTGATGTCAGCGAGAAGCACGGCGGAGGACCGCTCGTT
CCCGAGAAGGCTGTACGCTTCTCTTTCACTGTTATGTCTATTTCTGTCCT
GGCGGAGGAAAAGCAG------------------------------GAGG
AAGTTACCGTCTTCACTGAGCCGAAGCCGAACTCGGAGCTGTCCTGCAAG
CCCCTGTGCCTCACGTTCGTGGATGAATCCGACCATGAGACACTCACGGC
CGTACTGGGGCCGATAGTTGCGGAACGTAGTGCGATGAAGGAGAGCAGAC
TCATCCTGTCGATCGGAGGGATGCTCCGCTCCTTCCGCTTCCACTTCAGA
GGCACCGGATACGATGAGAAGATGGTGCGGGAAATGGAGGGCCTGGAAGC
CTCGGGGTCCACCTACATCTGCACTCTGTGCGACGCCAGTCGGGCAGACG
CGTCTGAAAACATGGTGCTGCACTCCGTCACGCGCAGCCATGGAGAGAAC
CTGGAACGTTACGAGATATGGAGGACCAACCCGTTCTCCGAGTCTGCAGA
CGAGCTGCGGGACMGAGTGAAAGGCGTCTCCGCCAAGCCCTTCTTGGAGA
CCCGCTCCACGATGGATGCTTTACACTGCGACATCGGCAACGCCACCGAG
TTCTACAAAATCTTCCAGGATGAAATAGGGGAGGTGTACAAAAAGGT---
CAAC---CCCAGCCGGGAGGAGCGGCGCAGCTGGAGGGCCGCCCTAGATA
AAGAGCTGAGGAGGAAGATGAAGCTTAAACCCATAATGAGGATGAATGGA
AACTACGCTCGAAAGCTAATGACCCTGGAAGCCGTGGAGGTGGTGTGTGA
TCTGGTGCCCTCGGAGGAGCGGAGGGAGGCCCTGAGGGAGCTCATGAGGC
TCTACCTCCAGATGAAGCCCGTGTGGCGCGCCACCTGCCCGGCCAAGGAG
TGCCCCGACCAGCTGTGCCGCTACAGCTTCAACTCCCAGCGCTTCGCCGA
CGTCCTCTCCTCCACCTTCAAATACAGGTACAACGGGAAGATAACCAATT
ACCTGCACAAGACCCTGGCCCANNNNNNNNNNNNNNNNNNNNNNNNNNNNNNNNNNNNNNNNNNNNNNNNN
NNNNNNNNNNNNNNNNNNNNNNNNNTCATACACCATTGACACCGGCCCCCTMG
GGCCCCAGTGGAAGGAGAGCCCACAGCCTTTCTCCTGCTCTATTGAAGAC
CCCACAAAGCAGACAAAGTTCAAAGGCATCAAGACGTACATTTCGTACAG
AGTCACTCCGAGCCACACAGGTCTCCCTGTATATAGGCGCTACAAACACT
TTGACTGGCTGTACAACCGCCTGCTGCACAAGTTCACTGTGATCTCCGTG
CCTCACCTGCCTGAGAAGCAGGCCACGGGGCGATTTGAGGAAGACTTTAT
TGAGAAACGTAAGAGACGACTGATACTGTGGATGAACCACATGACCAGTC
ACCCRGTCCTCTCCCAATATGAAGGCTTTGAGCACTTTCTGATGTGTGCT
GACGACAAGCAGTGGAAGCTGGGCAAGAGACGAGCCGAGAAGGAAGAGAT
GGTGGGCGCCCAGTTCATGCTGACCCTCCAAATCCCGAAAGAGCACCAGG
ACCTTCAGGATGTAGAGGAGAGGGTTGACAGCTTTAAGTCCTTCGTCAAG
AAAATGGACGACAGCGTCTTGCAGCTCACTCATGTTGCTTCAGAGCTGGT
GCGTAAGCACCTGGGTGGATTTAGGAAGGAGTTCCAGCGACTGGGAAATT
CTTTCCAGTCTATCAGCCAGTCATTTATGCTGGACCCACCCCATAGCTCA
GAGGCCCTCAACAANNNNNNNNNNNNNNNNNNNNNNNNNNNNNNNNNNNNNNNNNNNNNNNNNNTTATCA
TTGGAGTCGGTGTGGTTGGGAACCTCTTGA
TCTCCATCCTGCTGGTCAAAGACAAGAGCCTGCACCGAGCGCCCTATTAT
TTCCTGCTGGACCTGTGCGCCTCCGATATCCTGCGCTCAGCCATCTGCTT
CCCCTTTGTCTTCACGTCAGTCAAGAATGGCTCTGCCTGGACCTACGGCA
CGTTAACCTGCAAAGTGATCGCCTTCCTGGGTGTGCTGTCCTGTTTCCAC

ACCGCGTTCATGCTATTCTGCGTCAGCGTCACGCGCTACCTGGCCATCGC
ACATCACCGTTTCTACACAAAGCGTCTGACCTTCTGGACCTGTCTGGCTG
TCATCTGCATGGTGTGGACGTTGTCGGTGGCGATGGCGTTCCCGCCGGTG
TTAGACGTAGGGACGTATTCTTTCATCCAGGAGGAGGACCAGTGCACATT
CCAGCACCGCTCCTTCAGGGCAAACGATTCCCTTGGCTTCATGCTCCTTC
TGGCGCTCATCCTCCTGGCCACACAGCTGGTTTACCTCAAGCTCATCTTC
TTTGTCCACGACCGCCGAAAGATGAAGCCTGTCCAGTTCGTGCCTGCTGT
CAGCCAGAACTGGACTTTCCACGGGCCTGGCGCCAGCGGGCAGGCCGCGG
CAAACTGGCTAGCTGGATTTGGTAGAGGCCCCACCCCGCCTACTTTGCTG
GGCATCCGGCAGAACAGCAACGCAGCGGGCCGCAGGCGTCTACTGGTGTT
GGATGAGTTCAAAACAGAGAAGAGGATTAGTAGGATGTTCTACATCATGA
CGTTTTTCTTCCTGGCGCTGTGGGGGCCCTATCTGGTAGCCTGCTACTGG
CGGGTGTTCGCAAGGGGCCCTGTAGTCCCTGCAGGCTACCTTACGGCAGC
CGTGTGGATGAGCTTNNNNNNNNNNNNNNNNNNNNNNNNNNNNNNNNNNNNNNNNNNNNNNNNN------
----

NNNNNNNNNNNNNNNNNNNNNNNNNNNNNNNNNNNNNNNNNNNNNNNNNNNNNGTTCGCGGATGGGAT
GGGCGCCTTCAAAATCAACCACAGCTCCCACGATATTGGGTCCGG---AC
AGACGGCGTTTTCCTCCCAGGCA---CCCGGCTAC---GCGGCAGCCGCC
CTAGGACACCACCACCA------CCACCCGACCCACGTTGGCTCT---TA
CTCCACGGCGGCTTTCAACTCCACCAGGGACTTTCTCTTCAGAAATCGGG
GGTTCGGGGACGCCACCGG------------GGCGCAGCACAGTTTGTTC
GCCTC---------CGGAAGTTT---C------GCAGGGCCACATGGACA
CTCAGATGCAGCGGGGCACCTGCTCTTCCCAGGGCTCCACGAG---CAAG
CGGCGAGCCATGCGTCTTCCAACGTGGTCAACAGCCAGATGCGTCTGGGC
TTTTCCGGGGACATGTACGGACGGGCCGACCAGTACGGCCACGTTACGAG
CCCACGAT---CCGA---CTATGCTTCGTCCCAGTTGCACGGCTATGGCC
CCATGAACATGAATATGGCCGCG---CACCACGGAGCAGGGGCCTTCTTT
CGATACATGCGGCAGCCGATCAAGCAAGAGCTCATCTGCAAGTGGGTCGA
GCCGGAGCAGCTGACGAATCCCAAAAAGTCGTGCAGCAAAACTTTTAGCA
CAATGCACGAGCTCGTGACCCATCTGACAGTGGAGCATGTGGGGGGACCA
GAACAGTCCAACCACATCTGCTTCTGGGAGGANNNNNNNNNNNNNNNNNNNNNNNNNNNNNNNNNNNNNN
NNNNNNNNNNNNNNNNNNNNNNNNNNNNNNNNNNNNNNNNNNNNNNNNNNNNNNNNNNNNNNNNNNNNNN
N
>Macrourus sp
AGCCTTCTCATTCGAGCCGAGCTCAGTCAGCCCGGAGCACTTTTAGGTGA
TGATCAAATTTATAATGTTATCGTCACAGCCCATGCATTTGTAATAATCT
TTTTCATAGTTATACCTCTAATAATCGGAGGTTTCGGAAACTGGTTAATC
CCCCTAATAATTGGGGCTCCTGATATAGCTTTCCCTCGAATAAATAATAT
AAGCTTCTGACTTCTCCCCCCTTCTTTTCTTCTTCTAGCATCCTCTG

GCGTTGAGGCTGGAGCGGGGACCGGATGAACTGTCTACCCTCCCCTAGCA
GGCAACTTAGCTCACGCAGGGGCATCCGTGGATTTAACTATCTTTTCTCT
TCACTTAGCCGGAATTTCATCAATTTTAGGGGCAATTAATTTTATTACTA
CTATTATTAATATAAAACCACCCGCTATTACGCAGTACCAAACACCTTTA
TTTGTCTGAGCCGTTTTAATCACAGCGGTACTTCTTTTACTATCACTTCC
TGTACTAGCAGCCGGAATTACTATACTTCTCACAGATCGAAACCTTAATA
CTTCTTTCTTTGATCCTGCCGGTGGAGGTGACCCCATTCTTTATCAACAT
CTA---

-------------------------AGAGGATTACATTGTGGTCTTCTCT
CGTTCCACAACAAGGTTGATCCTAAACGAAGCAGAGCTGATCATGGCGCT
TGCCCAGGAGTTTCAGATGAGAGTCTTGACAGTATCCCTGGAGGAACAGT
CTTTTGCCAGCATCGTGCAGGTGATCAGCGGAGCCTCCATGCTGTTCAGT
ATGCACGGCGCGCAGCTCATCACATCGCTGTTCCTGCCCCGAGGTGCAGC
CGTGGTGGAGCTGTTCCCCTACGCCGTGAACCCAGAGCAGTACACCCCGT
ACAAAACCCTGGCCTCCCTGCCGGGCATGGACCTCCAGTACGTCTCCTGG
AGGAACACGATGGAGGAGAACACCGTCACTCACCCGGACCGGCCCTGGGA
CCAAGGGGGCGTGGTGCATTTGGAGAAGGAGGAACAAGAGCGCATACTAG
CCAGCAAGGACGTCCCCAGGCACCTGTGCTGCCGTAACCCAGAGTGGCTT
TTCAGAATCTACCAGGACACTTTAGTGGACATCCCTTCACTCCTCGAGGC
GCTCAA---GGATGGCCTCAGA---ACCAAGCCCAACTTGAAAAA---GA
GCAAGGCGGCCAGTACAGTTCACCCGGGCCGAGTCCGAGAACCCCAGTGC
AAGACCTCAGTCCAAGCTACCAACGAGGCCAAACTCACAGTGTCCTGGCA
GATCCCGTGGAACCTGAAGTTCCTGAAGGTGAGAGAGGTGAAGTACGAGNNN-
--------AAGAAGGATGCCAGCAAAGGAACCCTGGAGGATCAAATCATC
CAGGCTAACCCTGCCCTGGAGGCCTTTGGTAATGCCAAAACCCTGAGAAA
TGACAACTCCTCACGTTTTGGAAAGTTTATCCGGATTCACTTTGGGAACA
GTGGCAAGCTGAGCTCTGCAGACATTGAGACCTACCTCCTAGAAAAGTCC
AGGGTCACCTTCCAGCTCAAGTCAGAGAGGAACTATCACATATTTTTCCA
GATCTTGTCCAATCAGAAGCCAGAGCTGCTGGACATGCTGCTGATTACCA
ACAACCCGTACGATTACTCCTACATCTCCCAAGGAGAGGTAACCGTCGCC
TCCATCAACGACTCTGAGGAGCTCATGGACACTGACAGTGCGTTTGACGT
CCTTGGCTTCACCCTGGAAGAGAAGATGGGGGTGTACAAGCTGACTGGTG
CCATCATGCACTACGGCAACATGAGGTTCAAACAGAAGCAGCGTGAGGAG
CAGGCAGAGTCCGACGGGACTGAAGCAGCTGATAAATCAGCGTACCTCAT

```
GGGCTTGAACTCTGCAGACCTCATCAAATGCCTCTGCCACCCCAGGGTGA
AGGTAGGGAATGAATATGTCACCAAAGGTCAAGGGGTGGACCAAGTCTAC
TAC-----------------------------------------------
--------------------------------------------------
--------------------------------------------------
--------------------------------------------------
--------------------------------------------------
--------------------------------------------------
--------------------------------------------------
--------------------------------------------------
--------------------------------------------------
--------------------------------------------------
--------------------------------------------------
--------------------------------------------------
--------------------------------------------------
-------------------TACCTCATCTACGCTTCCTTCTCCTTCATGG
GATGTTTACAGATCAGCGACGGCTCCAACGTGGTCAACCTGCTGGCCAGT
AACTCTCCCAGTGTGTCCTACGCTCTGACACAGCAGAAGTACTTCAGTAA
CTACAGTCCCGTCATTGGCTTCTATATCTACGAGCCCATCGAGTACTGGA
ACTCCACGGTGCAGGAGCACCTGAAGACGCTGAGCCACGGCTTCAACAAG
ATCTCCTGGATGGACAACTTCTACCAGTACCTGCGCGTGGCCAACGTCAG
CGCGGCCACCAAGAACGAGTTCATCCACATCCTGAAGGGCTCCTTCCTGC
GCAGCCCGGAGTACCAGCACTTCACCGAGGACATCATCTTTTCCAAGA--
-ACCGCGAGAGCA------ACGAGTATGACATCATCGCCTCGCGCATGTA
CCTGGTGGCCCGCACCACGGAGAAGAAGCGCGAGGAGGTGGTGGAGCTGC
TGGAGAAGCTGCGGCCGCTGATGCTCATCAACAGCATCAAGTTCATCGCC
TTCAACCCCACCTTCGTGTTCATGGACCGCTACAGCTCCTCCGTCATCTC
GCCCATCCTCACCTCCGGCTTCAGCGTGCTCACCATCCTCATCCTCACCT
TCTTCCTGGTCATCAACCCCCTGGGGAACCTGTGGTTGATCCTGACCGTG
ACCTCGGTGGAGCTGGGCGTCCTGGGCCTGATG-----------------
--------------------------------------------------
--------------------------------------------------
--------------------------------------------------
--------------------------------------------------
--------------------------------------------------
--------------------------------------------------
--------------------------------------------------
--------------------------------------------------
--------------------------------------------------
--------------------------------------------------
--------------------------------------------------
--------------------------------------------------
--------------------------------------------------
--------------------------------------------------
--------------------------------------------------
--------------------------------------------------
--------------------------------------------------
--------------------------------------------------
--------------------------------------------------
--------------------------------------------------
--------------------------------------------------
--------------------------------------------------
--------------------------------------------------
```


--TCGTACACGG
TGGAGATCGGCCCCAAGGGCCCCCAGTGGAAGGAGAGCCCCCAGCCCTTC
TCCTGCTCCATCGAGGACCCCACCAAACAGACTAAGTTCAAGGGCATCAA
GACGTACATCTCGTACCGCGTGACGCCCAGCCACACCGGCATGGCCGTGT
ACCGGCGCTACAAACACTTTGACTGGCTGTACAACAGGCTGCTGCACAAG
TTCACCGTCATCTCCGTGCCCCACCTGCCTGAGAAGCAGGCCACGGGCCG
ATTCGAGGAGGACTTCATCGACAAACGCAAACGGCGGCTTGTCCTGTGGA
TGAACCACATGACCAGCCACCCGGTGCTGTCGCAGTACGAGGGCTTCGAG
CACTTCCTCATGTGCGCCGACGACAAGCAGTGGAAGCTGGGCAAGCGACG
GGCGGAGAAGGACGAGATGGTGGGCGCCCACTTCATGCTCACCTTCCAGA
TCCCCAACGAGCACCAGGACCTGCAGGATGTGGAGGAGCGAGTGGACTCC
TTCAAGGCGTTCACCAAGAAGATGGACGACAGCGTCATGCAGCTGACGCA
CGGCGCCTCCGAGATGGTGCGCCGGCACCTGGGGGAGTTCCGGAAGGAGT
TCCACCGGCTGGGCGGCGCCTTCCAGTCGCTGTCGCTGGCCTTCATGCTG
GAGCCGCCGCAAAGCTCTGAGCCTCTCAACAACGCCATCTCGCAC-----

>Malakichthys elegans

------------NNNNNNNNNAGAAACCTTCACCCATCTAACTGCCTTGGCATGCTGTT
GCTGTCTGATGCCCACCAGTGCACCAAGCTGTCAGAGCTCTCCTGGGGCA
TGTGCCTCAGCAACTTTCCCGCTATTTGCAAGACAGAGGACTTCCTCCAA
CTGCCCAAAGATATGGTGGTGCAGCTTTTGTCACATGAGGAGCTAGAGAC
AGAAGATGAGAGACTGGTTTATGAAGCTGCCCTTAACTGGATCAACTATG
ACCTGGAAAAGAGGCACTGCCACCTTCCAGAGCTCCTGAGAACGGTCCGT
CTGGCCCTGCTGCCTGCCATCTTTCTCATGGAGAACGTTTCTACAGAAGA
GCTGATCAATGCCCAGGCCAAGAGCAAGGAGCTGGTGGATGAAGCTATCC
GCTGTAAGCTGAAGATCCTGCAGAATGACGGCGTCGTTAACAGTCCGTGT
GCTCGACCAAGAAAAACCAGCCATGCCCTCTTTCTTCTGGGAGGGCAGAC
TTTCATGTGTGACAAGTTGTACCTGGTGGACCAGAAAGCCAAAGAGATCA
TCCCCAAAGCTGACATTCCCAGCCCCAGGAAGGAGTTCAGCGCCTGCGCC
ATCGGCTGTAAGGTGTACATCACTGGTGGGA--GAGGCTC-AGAGAATGG
TGTGTCCAAAGATGTGTGGGTCTATGACACCGTCCACGAGGAATGGTCGA
AGGCGGCACCCATGCTCATTGCCAGATTTGGCCATGGCTCTGCGGAGCTG
AAACACTGCCTCTACGTGGTNNNNNNNNNNNNNNNNNNNNNNNNNNNNNNNNNNNNNNNNNNNNNNGGAT
GAATACATTGTTGTGTTTAGTCGTTC

AACAACGAGGCTGATACTGAATGAAGCGGAGCTAATCATGGCGCTGGCCC
AGGAGTTCCAGATGAGAGTGGTCACGGTATCCCTGGAGGAACAGTCTTTC
CCCAGTATCGTCCAGGTGATCAGCGGTGCTTCCATGTTAGTCAGTATGCA
TGGAGCTCAGCTTATCACCTCACTCTTCCTCCCCAGAGGAGCTGCTGTGG
TGGAGCTGTTCCCCTTTGCCGTGAATCCAGAGCAGTACACCCCGTATAAA
ACCCTTGCCTCCCTTCCAGGCATGGACCTTCACTATGTCTCCTGGAGGAA
CACTAAGGAGGAGAACACCATCACCCACCCAGACAGACCCTGGGAACAAG
GGGGCATTGCTCACTTGGAGAAGGAGGAGCAAGAGCGAATACTGGCGAGC
AAAGATGTCCCCAGGCACCTGTGCTGCCGCAACCCAGAGTGGCTCTTCCG
GATCTACCAGGACACTTTGGTGGACATCCCTTCCTTCCTGGAAGTCCTCA
A---AGAGGGCATGAAG---ACAAAGCCCAGCTTGAAGAA---GTCAAAG
CCGGCCAGCACAGTCCATCCGGGCCGGGTCAGAGAACCCCAATGTCAGAC
CTCAGTACAAACCACTAATGAGGCTAAACTCACAGTCTCCTGGCAGATCC
CGTGGAATCTGAAATACCTGAAGGTGAGAGAGGTCAAATATGAGGTG---
------AAAAAAGACACCAGCAAGGGGACACTGGAGGATCAAATCATCCA
GGCGAACCCTGCGCTGGAGGCCTTCGGCAACGCCAAAACATTGAGAAACG
ACAACTCGTCTCGTTTTGGAAAATTCATCCGAATTCACTTCGGTACGAGC
GGCAAGCTGTCGTCTGCTGACATCGAGACGTACCTGCTGGAGAAGTCACG
TGTCTCCTTTCAGCTCAAGGCTGAGAGGAACTACCATATCTTCTACCAGA
TCCTGTCCAATCAGAAGCCAGAGCTGCTGGACATGCTGCTGATCACCAAC
AACCCATACGACTACTCCTACATCTCCCAAGGAGAGGTAACGGTCGCCTC
CATCAACGACTCGGAGGAGCTGATGGCCACCGACAGCGCCTTCGATGTGC
TCGGCTTCACTCCAGACGAGAAGATGGGCGTCTATAAACTGACCGGCGCC
ATCATGCACTACGGCAACATGAAGTTCAAACAGAAGCAGCGTGAGGAGCA
GGCGGAGCCGGACGGGACGGAGGCTGCTGACAAATCAGCTTACCTAATGG
GGCTGAACTCTGCTGACCTCATTAAAGGGCTGTGCCATCCCAGAGTCAAG
GTAGGAAATGAATACGTCACCAAAGGCCAAAGTGTGGATCAAGTCTACTA
CNNNNNNNNNNNNNNNNNNNNNNNNNNNNNNNNNNGGAAGCACTACAACACCAAGCTGGGATACAAGCGC
CATGTGGCCATGCA
CTCTGCCACGGCAGGGGATCTCACCTGCAAAGTGTGCATGCAGACCTACG
AGAGCACGCCCGTGCTCTTGGAGCACCTCAAGAGCCACTCCGGGAAGTCT
TCGGGTGGCACCAAGGAGAAAAAACACCCATGCGACCACTGTGACCGCCG
TTTCTACACGCGGAAGGATGTGAGACGGCACATGGTGGTCCACACGGGCC
GAAAGGACTTCCTGTGCCAGTACTGTGCCCAGCGCTTCGGCAGGAAGGAT
CATCTGACACGCCACGTGAAGAAGAGCCACTCGCAGGAGCTGCTGAAGAT
CAAGACGGAGCCTCCTGATATGTTAGGTCTTTTAGCGTCGGGGTCACCAC
CCTGCTCTGTGAAGGAGGAGCTCAGCCCCATGATGTGCGGCATTGGTCCC
AATAAAGACCCCATGATGGGCAAACCGTTCCCCAGTGCGGCCCCTTTCCC
GATGGGCATGTACAACCCCCACCAT------CTCCAGGCCATGTCTAATT
CTGGGGTGGGTCACCCA------CACCCGTCCCTGATGCCCAGTTCCTTG
TCTGCAGCTATGGGCATGGGCTGTCACATGNNNNNNNNNNNNNNNNNNNNNNNNNNNNNNNNNNNNNNNN
NNNNNNNNNNNNNNNNNNNNNNNNNNGTGAACCTGCTGGCTAGTAA
CTCTCCGAGTGTTTCGTACGCTCTGACCCAGCAGAAATACTTCAGTAACT
ACAGTCCCGTGATTGGGTTTTACATTTACGAGCCCATCGAGTACTGGAAC
TCAACGGTGCAGGAGCACCTGAAGACTCTGAGTCATGGCTTCAACAAGAT
CTCCTGGATGGACAACTTTTTCCACTACCTGCGGGTGGTGAATGTGAGTG
CGTCAACCAAGGGCGACTTCATCACCATCCTGAAGGGCTCCTTCCTGCGC
AGCCCGGAGTACCAGCACTTCACTGAGGACATCATATTCTCCAAGA---A
CCGCGAGACTG------ATGAGTACGACATTATCGCCTCACGGATGTACT
TGGTGGCACGGACGACAGAGAAGAAGCGCGAAGAGGTGGTGGAGCTTCTG
GAGAAGCTTCGTCCGTTGATGCTGATCAACAGCATCAAGTTCATTGCCTT
CAACCCTACGTTTGTGTTCATGGACCGCTACAGCTCCTCTGTCATCTCGC
CCATCCTGACCTCAGGCTTCAGCGTACTCACAATCCTCATCCTCACTTTC

TTCCTGGTCATCAACCCCTTGGGAAACTTCTGNNNNNNNNNNNNNNNNNNNNNNNNNNNNNNNNNNNNNN
NNNNNNNNNNNNNNNNNNNNNNNNNNNNNNNNNNNNNNNNNNNNNNNNNNNNNNNNNNNTCTTGCAATGT
TGGCATT
ATTAATGGGCTCTCTGGATGTGCTTCCTCGGTGGATGACGCCCCAGCTGA
CACCATCACTCGGCGGTTTCGCTATGATGTGGCACTGGTGTCAGCATTAA
AGGATCTGGAGGAGGACATCATGGAGGGGCTGAGAGAGAGTGGGATGGAA
GACAGTGCTTGCACCTCAGGCTTCAGTGTCATGATCAAGGAGTCTTGTGA
TGGCATGGGCGATGTCAGCGAGAAGCACGGTGGAGGACCACTTGTTCCTG
AGAAGGCTGTACGTTTCTCTTTCACTGTTATGTCTGTCTCTGTCCTGGCA
GACGAGGAGGAG------------------------------GAAGAGGT
TACCATCTTCACCGAGCCAAAGCCAAACTCAGAACTGTCCTGTAAGCCCC
TTTGCCTGACGTTTGTGGACGAGTCAGACCATGAGACACTCACAGCCGTC
CTGGGGCCTATAGTTGCAGAACGCAAAGGGATGAAAGAGAGCAGGCTCAT
CCTATCCATGGGTGGACTACCACGCTCCTTCCGCTTTCACTTCAGAGGCA
CGGGATACGATGAGAAGATGGTGCGTGAGATGGAGGGCCTCGAGGCCTCA
GGGTCCTCCTATGTCTGCACTCTTTGTGACTCCAGTCGGGCAGACGCCTC
TCAAAGCATGGTGCTACACTCCGTCACCCGCAGTCATGATGAGAACCTAG
AACGTTACGAAATATGGAGAACCAACCCCTTTTCTGAGTCTGTAGATGAG
CTGCGAGACAGAGTCAAAGGGGTCTCTGCCAAGCCCTTCATGGAGACCCA
TGCCACGCTGGATGCATTACACTGTGACATTGGCAATGCCGCGGAGTTCT
ACAAAATCTTCCAGGACGAGATCGGGGAGGTGTACCAAAAGGT---CAAC
---CCCAGCCGGGAGGAACGGCGCAGCTGGAGGGCAGCCCTAGATAAACA
GCTGAGGAAGAAGATAAAGCTTAAACCGGTAATGAGGATGAATGGGAACT
ATGCCCGCAAGCTAATGACCCAGGAGGCTGTGGAGGTGGTGTGTGAGCTG
GTGCCCTCAGAAGAGAGGAGGGAGGCCCTGAGGGAGCTTATGAGGCTCTA
CCTCCAGATGAAGCCTGTGTGGCGCGCCACCTGCCCAGCCAAGGAGTGCC
CCGACCAGCTGTGCCGCTACAGCTTTAACTCCCAGCGCTTTGCCGACCTC
CTCTCCTCTACCTTCAAATATAGGTACAACGGAAAGATAACCAATTACCT
GCACAAGACCCTGGCCCATNNNNNNNNNNNNNNNNNNNNNNNNNNNNNNNNNNNNNNNNNNNNNNNNNNN
NNNNNNNNNNNNNNNNNNNNNNTCGTACACCATCGACATGGCCCCTTGGGTC
CCCGGTGGAAGGAGAGCCCACAGCCTTTCTCCTGCTCCATTGAAGACCCC
ACAAAACAGACAAAGTTCAAGGGCATCAAGACGTACATTTCGTACCGGGT
CACGCCGAGCCACACAGGGCGTCCCGTCTACCGGCGCTACAAACACTTTG
ACTGGCTGTACAACCGCTTACTGCACAAGTTCACTGTGATCTCCGTGCCT
CACCTGCCTGAGAAGCAGGCCACGGGGCGATTTGAGGAAGACTTCATCGA
GAAGCGCAAGAGGCGACTGATACTGTGGATGAACCACATGACCAGTCACC
CGGTCCTCTCCCAGTATGAAGGCTTTGAGCACTTTCTGATGTGCGCTGAT
GACAAGCAGTGGAAACTGGGCAAGAGACGGGCGGAGAAGGACGAGATGGT
GGGCGCCCATTTCATGCTGACCCTCCAGATCCCCAACGAGCACCAGGACC
TTCAGGATGTAGAGGAGCGGGTCGACTCCTTCAAGTCCTTTGCTAAGAAA
ATGGATGACAGCGTGATGCAGCTCACACACGTCGCCTCGGAGCTGGTGCG
TAAGCACCTGGGTGGGTTCAGGAAGGAGTTCCAGCGGCTGGGAAATGCCT
TCCAGTCTATCAGCCAGGCCTTCATGCTGGACCCTCCCCACAGCTCAGAA
ACCTTCAACAACGCCATCTCCCAT--------------------------

-----------NNNNNNNNNNNNNNNNNNNNNNNNNNNNNNNNNNNNNNNNNNNCGGAGC---
GCAGCGTCCCACTCGGCAACAGCTTGCTAT
CCCCGCAGCAAACCGAGGAGCCCACTGTTGCCACCCCCCCGCAGCGATGG
TTTGTCACCC---CTGCCAACAACCGACTGGACTTTGCTGCCTCGGCATA
CGACGCCGCT---------GATTTCGCCGGTAACGCGGCCACCTTGCTGT
CCTACGCAGCGGCCGGAGTGAAGGCTC------TTCCCCTGCCGACTGCA
GGCTGCTCCAACCGGCCTCTTGGCTATTACGCAGACCCGTCAG---GCTG
G---GGAGGACGCACGCCGCCTCAGTACTGTGGCGTAAATAGCAAATCCA
GCTCGGTCTTTTCCTGCTGGCCCGCTAACTCTATCGGTGGCAGAGCAGGC
A---CC---AACTACCTGG---------CGGAGGA---GGGA---GACTC
---CATCCCGACGGAGAGGTCACCG---AT---CGGCGGCTCGGAGGAG-
--GCCAAACCCAAAGACCTGAC---ATCAGA---GTCGAGCTGGATAGAG
---ACGCCGTCCTCCATTAAGTCCATTGATTCGAGCGATTCTGGTATCTT
TG---AACAGGCCAAAAGGAGAAGAATCTCACCTTCTGCCACGCCG----

GTTTCAGAGACAGTGTCCCCCTTAAAGTCNNNNNNNNNNNNNNNNNNNNNNNNNNNNNNNNNNNNNNNNN
NNNNNNNNNNNNNNNNGTTCGCGGATGGGATG
GGCGCCTTCAAAATAAACCACAGCTCCCACGATATTGGCTCCGG---ACA
AACGGCGTTTTCCTCCCAGGCG---CCCGGCTAC---GCAGCGGCCGCCC
TGGGA---CACCACCA------CCACCCGACCCACGTTGGCTCT---TAC
TCCACGGCGGCTTTCAACTCCACCAGGGACTTTCTCTTCAGAAATCGGGG
CTTCGGGGACGCCACCGG------------GGCGCAGCACAGTTTGTTCG
CCTC---------CGGAAGTTT---C------GCAGGGCCACATGGACAC
TCAGATGCAGCGGGGCACCTGCTCTTCCCGGGGCTCCACGAG---CAAGC
GGCGAGCCATGCGTCTTCCAACGTGGTCAACAGCCAGATGCGGCTGGGCT
TCTCGGGGGACATGTACGGACGGGCCGACCAGTACGGCCACGTTACGAGC
CCGCGGT---CCGACCACTACGCTTCGACCCAGCTGCACGGCTATGGCCC
CATGAACATGAATATGGCCGCG---CACCACGGAGCAGGGGCCTTCTTTC
GATACATGAGGCAGCCGATCAAGCAAGAGCTCATCTGCAAGTGGATCGAG
CCGGAGCAGCTGACGAATCCCAAAAAGTCGTGCAACAAAACTTTTAGCAC
GATGCACGAGCTCGTGACCCATCTGACGGTGGAGCATGTGGGGGGACCGG
AGCAGACCAACCACATCTGCTTCTGGAGGANNNNNNNNNNNNNNNNNNNNNNNNNNNNNNNNNNNNNNNN
NNNNNNNNNNNNNNNNNNNNNNNNNNNNNNNNNNNNNNNNNNNNNNNNNNNNNNNNNNNNNNNNNNNNNN
>Mallotus villosus
AGCCTCCTTATCCGAGCCGAGCTAAGCCAACCTGGCGCTCTTCTGGGGGA
CGACCAGATTTATAATGTTATCGTCACCGCACACGCTTTTGTTATAATCT
TTTTTATAGTAATACCCATCATGATCGGAGGGTTCGGCAACTGGCTAATC
CCTCTTATGATCGGGGCCCCAGACATGGCGTTCCCACGCATAAATAACAT
GAGTTTCTGACTTTTACCTCCCTCTTTCCTTCTCCTCTTAGCCTCCTCCG
GGGTAGAAGCAGGAGCCGGTACCGGCTGAACAGTTTACCCGCCACTTGCT
GGCAATCTCGCTCACGCGGGGGCTTCCGTAGATTTAACCATCTTTTCCCT
CCACCTTGCGGGTATCTCCTCTATTCTAGGGGCCATTAATTTTATTACAA
CTATTATTAACATGAAACCTCCTGCTATTTCTCAGTACCAGACTCCTTTA

TTTGTTTGAGCTGTGCTAATTACAGCCGTTCTTCTTCTACTATCCCTTCC
TGTCTTAGCCGCTGGAATTACAATGCTTCTGACAGACCGAAATCTAAACA
CCACTTTCTTTGACCCTGCTGGGGGAGGAGATCCTATTCTATATCAGCAT
TTANNNNNNNNNNNNNNNNNNNNNNNNNNNNNNNNNNNNNNNNNNNNNNNNNNNNNNNNNNNNNNNNNNN
NNNNNNNNNNNNNNNNNNNNNNNNNNNNNNNNNNNNNNNNNNNNNNNNNNNNNNNNNNNNNNNNNNNNNN
NNNNNNNNNNNNNNNNNNNNNNNNNNNNNNNNNNNNNNNNNNNNNNNNNNNNNNNNNNNNNNNNNNNNNN
NN---

----------------GGACCAGTACATCGTGGTGTTCAGTCGTTCCCAA
ACCAGGCTGATCCTCAACGAGGCCGAGCTGATCCTGGCGTTGGCGCAGGA
GTTCCAGATGAGGGTGGTGACGGTSTCCATGGAGGACCAGACCTACGCCA
GCATCGTGCAGGTCGTCAGCGGAGCCTCCATGCTCGTCAGCATGCACGGA
GCTCAGCTCGTCACCTCGCTCTTCCTCCCCAGAGGGGCGGCCGTGGTGGA
ACTCTTCCCCTACGCGGTCAGCCCTGAGCAGTACACCCCTTACAAGACCT
TGGCCACCTTGCCAGGCATGGACCTCCAGTATGTGGCCTGGAGGAACACC
ATGGAGGAGAACTCCGTGGCCCACCCAGAGAGACCCTGGGATCAGGGGGG
AATCACTCACCTGGACAAAGACGAGCAGGAGCGCATCCTGGCCAGCAGAG
ATGTGCCCAGACACCTCTGCTGCAGAAACCCCGAGTGGCTGTTCCGCATC
TACCAGGACACCCGGTTGGACATCCCCTCTCTGCTGGACGTCCTCAG---
GGAGAGCCTGAAG---ACCAGGCCCAGCCTCAAGAA---GACCCKGTCTG
CCAGCACGGTTCATCCAGGCAGGGTGAGGGAGCCTCAGTGCCAGACCTCA
GTCCAGGCTACCAACGAAGCCAAGCTCACAGTATCSTGGCAGATCCCTTG
GAACCTCAAGTACTTGAAGGTNNNNNNNNNNNNNNNNNNNNNNNNNNNNNNNAAGAGAGATCCCAGCAAG
GGAACCTTGGA
GGATCAAATCATCCAGGCTAACCCTGCCTTGGAGGCTTTTGGTAATGCCA
AAACATTGAGGAATGACAACTCGTCACGCTTTGGAAAATTCATCAGGATT
CACTTCGGAACCAATGGCAAGCTGTCCTCAGCTGACATAGAGACTTATCT
TCTGGAAAAGTCACGTGTCACCTTCCAACTCAAGTCAGAAAGGAATTACC
ATATCTTCTTTCAGATCTTGTCTGAAAAAAAGCCAGAGCTGTTGGACATG
CTTCTGATCACCAACAACCCATATGACTACTCGTACATCTCCCAAGGAGA
GGTAACAGTAGCATCTATCAATGATGCTGATGAGTTGATGGCCACCGACA
GTGCCTTTGATATCCTTGGCTTTACTCAAGAGGAGAAAAACGGGGTCTAC
AAGTTGACTGGAGCTATCATGCATTTTGGCAACATGAAGTTCAAGCAGAA
GCAGCGTGAGGAGCAGGCAGAGGCTGATGGCACTGAGGCGGCTGACAAGT
CAGCTTACCTAATGGGGCTGAACTCTGCAGATCTTGTGAAAGGACTCTGC
CATCCCAGGGTCAAGGTTGGCAATGAGTATGTCACCAAAGGGCAGGGCGT
AGATCAAGTCTACTAC----------------------------------

--GGCT
ATCATCCCTTCRAATGGCAGCCGGCCCTCCGGAACGTTTCCCCGGCCTGC
CAGGTGGGCATCATCGACGGCCTGTCAGGATGGACGGCTTCGGTGGACGA
CTCCCCAGCGGACACCATCTCGCGGAGGTTCCGCTACGACGTGGCTCTGG
TGTCTGCTCTGAAGGACCTAGAGGAGGACATCGTGGAGGGCTTGAGGGAG
CACGGCCTGGAAGACAGTTCCTGCACCTCTGGCTTCACCGTGACCATCAA
GGAGTCCTGTGACGGCATGGGGGACGTCAGCGAGAAGCACGGCGGGGGGC
CGGCCGTTCCCGAAAAGGCCGTCCGCTTCTCCTTCACCGTCATGTCCGTC
TCCGTCCTGGCCGAGGGGGAGGAG--------------------------
----GAAGCGGTCACCGTCTTCAGAGAGCCCAAGCCCAATTCCGAGATGT
CCTGCAAACCTCTCTGTCTGATGTTTGTGGACGAGTCGGACCACGAGACC
CTGACGGCCATCTTGGGGCCTGTGGTGGCTGAGAGGAAGGCCATGAAGAA
CAGCCGCCTCATCCTGCCCATGGGCGGCCTCCCTCGCTCCTTCCGCTTCC
ACTTCAGGGGCACAGGCTATGACGAAAAGATGGTGCGCGAGATGGAGGGC
CTGGAGGCTTCAGGCTCCACCTACGTCTGCACCCTCTGCGACGCCACCAG
AGCTGAGGCCTCCCGAAACATGGTGCTGCACTCAGTCACCCGCAGCCACG
ACGAGAACCTGGACCGTTACGAGTTGTGGAGGACCAACCCGTACGGCGAA
TCGGCGGACGAGCTGCGAGACCGGGTGAAAGGGGTCTCCGCCAAGCCCTT
CATGGAGACCCAGCCCACAATGGACGCGCTGCACTGTGACATCGGCAACG
CCACCGAGTTCTACAAGATCTTCCAGGACGAGATCGGGGAGGTGCATTGC
AGGCC---CAAC---CCGAGCAGGGAGGAGCGTCGGAGCTGGAGGGCGGC
TCTGGACAAGCAGCTCAGGAAGAAGATGAAGCTGAAGCCCGTGATGAGGA
TGAACGGCAACTTCGCCCGGCGACTGATGACGGCGGAGGCGGTGGAGGCA
GTGTGCGAGCTGGTGCCGTCGCAGCAGCGGAGCGAAGCCCTCAGGGAGCT
GATGAACCTCTACATCCAGATGAAGCCCGTCTGGCGCGCCACGTGCCCGG
CCAAAGAGTGCCCCGACCAGCTGTGTCGCTACAGCTTCAACTCCCAGCGC
TTCGCTGATCTCCTCTCTTCCACCTTCAAGTACAGGTACGACGGGAAGAT
CACTAACTACCTCCTCAAGACTCTGGCGCACGTTCCTGAAATCATAGAGA
GGGACGGCTCCATCGGGGCCTGGGCCAGTGAGGGGAACGAGTCGGGAAAC

```
AAA-----------------------------------------------
--------------------------------------------------
--------------------------------------------------
--------------------------------------------------
--------------------------------------------------
--------------------------------------------------
--------------------------------------------------
--------------------------------------------------
--------------------------------------------------
--------------------------------------------------
--------------------------------------------------
--------------------------------------------------
--------------------------------------------------
--------------------------------------------------
--------------------------------------------------
--------------------------------------------------
--------------------------------------------------
--------------------------------------------------
--------------------------------------------------
--------------------------------------------------
--------------------------------------------------
--------------------------------------------------
--------------------------------------------------
--------------------------------------------------
--------------------------------------------------
--------------------------------------------------
--------------------------------------------------
--------------------------------------------------
--------------------------------------------------
--------------------------------------------------
--------------------------------------------------
--------------------------------------------------
--------------------------------------------------
--------------------------------------------------
--------------------------------------------------
--------------------------------------------------
--------------------------------------------------
--------------------------------------------------
--------------------------------------------------
--------------------------------------------------
--------------------------------------------------
--------------------------------------------------
--------------------------------------------------
--------------------------------------------------
--------------------------------------------------
--------------------------------------------------
--------------------------------------------------
--------------------------------------------------
---------------------------GGCGAAGTCACAGAAAGAGAAGT
GGCTTTGGGGATAAATCCGTTCGCAGACGGGATGGGTGCTTTCAAAATCA
ACCACAGTTCGCATGATCTTGGCTCCGG---GCAAACTGCATTTTCCTCC
CAAGCG---CCCGGCTAC---GCAGCCGCTGCCCTGGGA---CATCACCA
------CCACCCGACTCATGTCAGCTCT---TACTCTACGGCGGCTTTCA
```

ATTCCACCCGGGATTTTCTCTTCAGAAATCGAGGCTTCGGAGACGCTACC
AG------------CGCTCAGCATAGTCTCTTCGCCTCCGC---AGCGGG
AAGTTT---T------GCAGCCCCACATGGACACTCAGATGCAGCGGGAC
ACCTGCTCTTCCCAGGACTTCACGAA---CAAGCCGCGAGCCATGCTTCC
TCAAATGTTGTTAATAGTCAGATGCGATTGGGCTTTTCGGGGGACATGTA
CGGCAGAGCCGACCAGTATGGCCACGTTACCAGCCCGCGGT---CCGACC
ACTATGCTTCGACCCAGTTGCATGGCTATGGCCCTATGAACATGAATATG
GCCGCG---CATCATGGAGCAGGGGCCTTCTTCCGTTACATGAGACAGCC
GATAAAACAAGAGCTGATCTGCAAGTGGATCGAACCGGAGCAACTAACGA
ACCCGAAAAAGTCGTGCAACAAAACTTTAGCACAATGCACGAGCTCGTC
ACCCATCTGACGGTGGAGCATGTGGGAGGACCGGAGCAGTCGAACCACAT
TTGCTTCTGGGAAGAGTGTGCCCGAGAAGGAAAACCATTCAAAGCCAAAT
ACAAACTTGTGAACCACATCAGAGTGCACACCGGAGAG------------

>Margrethia obtusirostra
AGCCTGCTTATCCGAGCAGAGTTAAACCAACCCGGTGCCCTTCTAGGCGA
CGACCAAATTTACAACGTTATTGTTACGGCACATGCCTTCGTAATAATTT
TCTTTATAGTGATACCAATTATGATTGGTGGCTTCGGAAATTGACTAATT
CCCCTTATGATCGGAGCCCCTGACATAGCCTTCCCTCGAATAAACAACAT
AAGTTTCTGACTTCTCCCTCCCTCCTTTCTTCTACTATTAGCCTCTGCAG
GAGTTGAAGCCGGTGCCGGAACAGGATGAACAGTCTACCCGCCCCTTGCC
GGTAATTTAGCCCACGCAGGGGCATCTGTTGATCTTACCATCTTCTCCCT
TCACCTTGCAGGTGTCTCTTCAATCCTCGGAGCAATTAACTTTATCACAA
CAATTATTAACATGAAGCCCCCAGCCATCTCCCAATACCAAACACCTCTC
TTCGTGTGAGCTGTTCTCATTACTGCTGTCCTCCTTCTCTTGTCCCTCCC
AGTACTGGCTGCCGGGATCACTATACTCCTCACAGATCGAAACCTAAACA
CAACTTTCTTCGACCCTGCAGGAGGAGGAGACCCCATCCTCTACCAGCAC
CTCTTCTGGTTCTTCGGACACCCCGAAGTATACATTTTGATTCTTCCAGG
CTTTGGCATGATCTCCCACATTGTTGCCTACTACGCAGGGAAAAAAGAAC
CCTTCGGGTACATGGGCATGGTCTGAGCCATGATGGCCATCGGACTCCTG
GGCTTTATTGTCTGAGCCCACCACATGTTCACAGTCGGTATAGATGTAGA
CACCCGAGCCTNTTCCTGGAGAGGAACCTGCACCCCACCAACTGCCTGGGC
ATGCTGCTGCTGTCCGACGCCCACCAGTGCACCAAGCTGTCCGAGCTGTC
CTGGGGCATGTGCCTCAGCAACTTCCCCACCATCTGCAAGACGGAGGACT
TCCTCCAGCTCCCCAAGGACATGGTGGTGCAGCTGCTGTCCCACGAGGAG
CTGGAGACGGAGGACGAGAGGCTGGTCTACGAGGCCGCCCTCAACTGGGT
CAACTACGACCTGGAGAGGCGCCACTCCCACCTGGCCGAGCTGCTGCGGA
CAGTGCGCCTGGCGCTTCTGCCCGCCATCTTCCTCATGGAGAATGTGTCC
ACGGAGGAGCTGATCAACGCCCAGGTCAAGAGCAAGGAGCTGGTGGACGA
GGCCATCCGTTGCAAGCTGAAGATNNNNNNNNNNNNNNNNNNNNNNNNNNNNNNNNNNNNNNNNCCCCCGC
AAGACCAGCCACGCCCTCT
TCCTGCTGGGCGGGCAGACCTTCATGTGCGACAAGCTGTACCTGGTGGAC
CAGAAGGCCAAGGAGATCATCCCCAAGGCGGACATCCCCAGCCCCAGGAA
GGAGTTCAGCGCCTGCGCCATCGGCTGCAAGGTCTACGTCACCGGGGGGA
--GGGGCTC-GGAGAACGGCGTGTCCAAGGACGTGTGGGTGTACGACACT
GTCCAGGAGGAGTGGTCGAAGGCGGCGCCCATGCTCATCGCCCGGTTCGG
CCATGGCTCAGCCGAGCTGAAACACTGCCTCTACGTGGTGGGAGGGCACA
CCGCAGCCACGGGCTGCCTGCCGGCCTCCCCCTCG---------------

---------------------AAGAGGGATCCCAGCAAGGGAACCTTGGA
GGATCAAATCATCCAGGCTAACCCTGCCCTGGAGGCTTTCGGTAATGCTA
AAACACTGAGAAATGACAACTCATCACGCTTTGGCAAATTCATCCGGATT
CACTTTGGAACCAGTGGCAAGTTGTCCTCTGCGGACATAGAGACTTACCT
TCTGGAAAAGTCACGTGTCACCTTTCAGCTCAAGGCAGAGAGGAACTATC
ATATCTTCTTCCAGATCTTGTCCAATCAAAAGCCAGAGCTGTTGGACATG
CTTTTAATCACCAACAATCCATWTGACTACTCCTACATCTCCCAAGGAGA
GGTAACAGTAGCATCCATCAATGATTCTGAGGAGTTGATGGCCACTGACA
GCGCATTCGATGTGCTTGGCTTTACACCAGAGGAGAAAATGGGAGTCTAC
AAGTTGACAGGTGCAATCATGCATTACGGCAACATGAAGTTCAAGCAAAA
GCAGCGAGAGGAGCAGGCAGAGCCTGACGGCACTGAGGCTGCTGACAAGT
CAGCCTTCCTAATGGGGCTGAACTCTGCAGATCTAGTGAAAGGACTCTGC
CATCCCAGGGTTAAGGTCGGCAATGAGTATGTCACTAAAGGGCAGGGTGT
AGACCAAGTCTATTAC----------------------------------

--------------------------------TATCTGATCTACGCCTCC
TTCTCCTTCATGGGATGTTTACAAATCAGTGACGGTTCCAACGTGGTCAA
CCTGTTGGCCAGCAACTCGCCGAGCGTGTCCTACGCGCTGACCCAGCAGA
AGTACTTCAGCAACTACAGCCCCGTCATCGGGTTCTACATCTACGAGCCC
ATCGAGTACTGGAACTCCACGGTGCAGGAGCACCTGAGGACGCTGGGCCA
TGGCTTCAACAAGATCTCCTGGATGGACAACTACTTCCACTACCTGAAGG
TGGTGAACGTGACCGCCTCCACCAAGAGCCACTTCATCGCCATCCTCAAG
GGCTCCTTCCTGAGGAGCCCGGAGTACCAGCACTTCACAGAGGACATCAT
CTTCTCGAAGA---ACGGCG------------ACGAGTACGACATCATCG
CGTCCCGGATGTACCTGGTGGCGCGCACCACGGAGAAGACGCGGGAGGAG
GTGGTGGAGCTGCTGGAGAGGCTGCGGCCGCTCTCCCTCATCAACAGCAT
CAAGTTCATCGTCTTCAACCCCACCTTCGTCTTCATGGACCGCTACAGCT
CGTCGGTGGTCTCGCCCATCCTCACCTCGGGCTTCAGTGTCCTCACCATC
CTCATCCTCACCTTCTTCCTGGTCATCAACCCGCTGGGGAACTTCTGGTT
GATCCTGACGGTGACCTCGGTGGAGCTGGGCGTGCTGGGCCTCATG----

--------------AAGGCTGTGCGGTTCTCCTTCACCGTCATGTCTGTC
TCCGTCCAGGCCGAGGGGGAGGAT--------------------------
----GAGGCCATCACCATCTTCAGGGAGTCCAAGCCAAACTCTGAGCTGT
CCTGCAAGCCCCTGTGTCTGATGTTCGTGGATGAGTCTGATCACGAGACG
CTGACGGGCATCCTTGGGCCCGTGGTGGCTGAGAGGAACGCCATGAAGAA
CAGCCGCCTCATCCTGTCTCTGGGAGGCCTCCTCAGATCCTTCCGCTTCC
ACTTCAGGGGTACAGGCTATGATGAGAAGATGGTGCGTGACCTGGAGGGC
CTGGAGGCCTCAGGCTCCACATACATCTGCACCCTCTGCGACTCCACCCG
GGCGGAGGCCGCCCACAACATGGTGCTTCACTCCATCACCCGCAGCCACG
ACGAGAACGCGGAGCGCTACGAGATCTGGAGGACCAATCCCTACTCCGAG
TCCGCAGACGAGCTTCGGGATCGGGTGAAAGGTGTCTCCGCCAAGCCCTT
CATGGAGACCCAGCCCACCCTGGACGCTCTACACTGTGACATCGGTAACG
CCACCGAGTTCTACAAGATCTTCCAGGAYGAGATCGGCGAGGTGCACCGC
AGGCC---CAAC---CCCAGCCGGGAGGAGCGCCGGAGCTGGCGGGCCGC
TCTGGACAAGCAGCTGCGGAGGAAGATGAAGCTGAGGCCGGTGATGAGGA
TGAACGGGAACTACGCCCGGCGGCTGATGACCAGGGAGGCGGCAGAGGTG
GTGTGTGAGCTGGTCCCCTCGGAACAGCGGCGCGAGGCCCTCAGGGAGCT
GATGGGGCTCTACCTCCAGATGAAG-------------------------

---TCTTACACAATCGAAATGGGCCCCAAAGGGCCTCAGTGGAAGGAGAG
CCCTCAGCCTTTCTCTTGTTCTGTTGAGGACCCTACCAAACAGACCAAGT
TCAAAGGCATCAAGACCTACATATCTTACCGGGTCACCCCCAGCCACACC
GGGCGAYCCGTATACCGTCGGTACAAGCACTTTGATTGGCTATACAACCG
CCTGMTGCATAAGTTCACTGTCATCTCTGTGCCTCACCTGCCGGAGAAGC
AGGCCACAGGGCGTTTCGAGGAGGACTTCATCGAAAAGCGCAAAAGGCGG
CTGATCCTCTGGATGGACCACATGACCAGTCACCCAGTCCTCTCCCAGTA
TGAGGGCTTTGAGCACTTCCTCATGTGCGCTGATGACAAACAGTGGAAGC
TGGGCAAGCGGAGGGCGGAGAAGGATGAGATGGTGGGTGCCCACTTCATG
CTGACCTTTCAGATTCCCAACGAGCACCAGGACCTGCAGGACGTGGAGGA
GCGAGTGGACTCCTTCAAGTCTTTTGCCAAGAAAATGGACGATAGTGTCA
TGCAGCTGACACATGTGGCCTCAGAACTGGTGCGGAAACATCTAGGGGGT
TTCAGGAAGGAGTTCCAGCGTCTGGGGAACGCTTTCCAATCCATTAGTCA
GGCATTCATGCTGGACCCTCCTCACAGCTCTGATGCTCTCAACAACGCCA
TCTCGCAC--

---GCCAA
ATCTCGTTTTCACCCTGGCGTAGGGACTGGTCCTGGCACGGACC---GCA
GCGTCCCACTTAGTAACAGCTTGCTATCCCCGCAACAAACCGAAGAGCCC
ACGGTTG---CTTCCCCACAGCGATGGTTTGTCACCC---CTGCCAACAA
CCGACTGGACTTTGCCGCCTCGGCATACGATGCTGCCGCTGCTGCAGATT
TTGCCGGCAACGCAGCCACCTTGCTGTCCTACGCAGCGGCTGGAGTGAAG
GCCC------TTCCCCTGTCCACGGCAGGTTGCTCAAACAGACCTCTCGG
TTATTATGCCGACCCATCAG---GTTGG---GGCGCCCGCACGCCACCAC
AATACTGT---------AGCAAGTCCAGCTCAGTTCTCTCTTGCTGGCCC
ACAAATACTGTTGGAGGCAGAACAGGCA------CCAATTACCTGG----
-----CTGAGGA---TGGG---GA------------CACAGAGAGGTCTC
CA---AT---AGGCGCGTCCGACGAA---ACAAAACCAAAAGACTT----
--GTCCGA---GTCCAGCTGGATAGAG---ACGCCGTCTTCAATAAAGTC
AATTGATTCAAGTGATTCTGGAATCTTTG---AGCAAGCGAAACGGAGAA
GAATTTCTCCTTCTGTCACACCA---------GTTTCGGAGGCCGTGTCC
CCGTTGAAATCAGAG-----------------------------------

>Megalops atlanticus
AGCTTACTAATCCGGGCTGAGCTAAGCCAACCCGGGGCACTACTTGGTGA
TGACCAAATTTATAATGTCATCGTCACGGCACACGCCTTCGTAATAATCT
TCTTTATAGTAATGCCTATTTTAATTGGTGGGTTTGGAAACTGACTAGTC
CCTCTTATGATCGGGGCCCCAGACATAGCATTCCCCCGTATAAACAATAT
AAGCTTCTGGCTTCTCCCGCCGTCATTCCTTCTTCTGTTAGCTTCCTCGG
GAGTCGAAGCGGGGGCAGGTACTGGATGAACAGTCTACCCTCCTCTTGCT
GGTAACCTAGCCCACGCAGGTGCATCTGTAGACCTTACCATCTTTTCTCT
CCACCTGGCAGGTGTCTCCTCAATCCTAGGCGCTATCAACTTTATTACTA
CAATTATCAACATAAAACCGCCCGCCATGTCACAATACCAAACACCACTA
TTCGTTTGATCTGTCTTAGTTACCGCAGTACTTCTCCTGCTATCTCTGCC
GGTATTAGCTGCAGGTATTACTATACTCCTCACAGATCGCAATCTAAACA
CAACCTTCTTTGACCCTGCAGGAGGAGGAGACCCAATCCTATACCAACAC
CTATTTTGATTCTTTGGACACCCAGAAGTTTATATCCTCATCCTGCCAGG

ATTTGGAATAATTTCCCATATCGTAGCCTACTACGCCGGTAAAAAAGAAC
CCTTTGGATACATGGGCATGGTTTGAGCAATAATAGCCATTGGTCTACTA
GGATTCATTGTATGAGCACACCACATATTCACAGTAGGGATGGATGTGGA
CACTCGCGNNNN--

--ACTACAAC
ACCAAGCTGGGCTACAAGCGCCACGTGGCCATGCACGCGGCCACCAGCGG
CGACCTCACCTGCAAGGTGTGCCTGCAGAGCTACGAGAGCACGCCCGTGC
TGCTGGAGCACCTCAAGAGCCATTCGGGGAAGTCCTCGGGCGGGGCCAAG
GAGAAGAAGCACCCGTGCGACCACTGCGACCGCCGCTTCTACACGCGCAA
GGACGTGCGCCGCCACATGGTGGTGCACACCGGCCGCAAGGACTTCCTGT

GCCAATACTGCGCCCAGCGCTTCGGCCGCAAGGACCACCTGACCCGGCAC
GTGAAGAAGAGCCACTCGCAGGAGCTGCTGAAGATCAAGACGGAGCCGCC
GGACATGCTGGGCCTGCTGGGGTCC-GCTCTCCGCCCTGCCCCGTCAAGG
AGGAGCTCAGCCCGATGATGTG---CATGGGTCCCTCCAAGGACTCCCTA
ATGGGGAAGCCTTTCCCCGGCG------CTTTCCCCATGGGCATGTACAA
CCCCCAC---------TTGCAGGCCATGTCCAACTCCGGGGTGCCC----
--------CACCACTCCCTGGTGTCTGGGTCACTGTCCGCGGC-ATGGGA
ATGGGCTGCCMCA-----TACCTGATTTACGCGTCATTTTCGTTCATGGG
ATGTTTACAAATCAGCGACGGATCGAACATAGTCAACCTTTTAGCCAGCA
ACTCTCCCAGCGTCTCCTATGCTCTCACCCAGCAAAAATACTTCAGCAAC
TACAGTCCTGTGATTGGGTTCTACATCTACGAGCCCATAGAGTACTGGAA
TTCCACTGTTCAGGAACACCTGAAGACACTGGGCCACGGATTCAATAAGA
TTTCTTGGATCGACAACTACTTTCAGTATCTGAGGGTGGTGAATGTCAGC
GCATCGACCAAAAGCGAATTCATATCTATCCTCCAGACTTCGTTCTTGAG
GAGCCCAGAGTACCAGCACTTCAAGGACGACATAATTTTTTACAAAA---
TGAGGG------------ACGAAACGGAGATCATCGCGTCCAGGATGTAC
CTGGTGGCCAGAACCACAGAAAAAACGCGTGAGGAGGTGGTGGAGTTACT
GGAGAGACTGAGACCTCTGTCTCTTATCAACAGCATAAAGTTCATTGTCT
TCAACCCCACCTTTGTGTTCATGGATCGATACAGCTCCTCCGTCATTTCC
CCCATCCTGACCTCTGGATTCAGCGTCCTGATCGTTCTGATCCTGACGTT
TTTTCTGGTCATCAACCCCCTGGGAAACTTCTGGTTGATTCTGACAGTCA
CCTCAGTGGAGCTGGGCGTTCTTGGCCTCATGGGGTTCCACCCCTTCGAG
TGGCAGCCGGCACTCAAGAATGTGTCCAGTGCCACTGAGGTGGGCATCAT
TGATGGGTTGTCGGGGTGGATGGCCACTGTGGATGACTCACCTGCAGACA
CCATTGCCCGGAGGTTCCGCTACGATGTGGCTCTGGTAGCTGCATTGAAG
GATCTTGAGGAAGACATAATGGAAGGCCTGAGAGAGAATGGCTTGGAGGA
CTGTGCCTGCACAGGCGGGTTCTCTGTGGTGATCAAGGAGTCTTGTGATG
GGATGGGGGATGTCAGTGAAAAGCACGGGGGTGGGCCAACGGTCCCCGAA
AAAGCAGTGCGGTTCTCCTTTACGGTAATGTCAGTCTCCGTCCAGCTGGA
AGGCAGGGGT------------------------------GAGCAGATCA
CAGTGTTCCGGGAGCCAAAGCCTAACTCCGAGCTGAGCTGTAAGCCACTG
TGCCTGATGTTTGTCGATGAGTCGGACCATGAAACACTGACCGCCGTGCT
GGGGCCTGTGGTGGCGGAGAGGAATGCTATGAAGGAGAGCCGGCTCATCC
TGTCCATTGGAGGGCTCCTGCGCTCCTTCCGCTTCCACTTCCGAGGCACG
GGCTATGATGAGAAGATGGTGAGGGAGATGGAGGGCCTGGAAGCTTCAGG
ATCCACCTACATCTGTACCCTGTGTGACTCTACAAGGGCTGAGGCCTCCC
AGAACATGGTGCTGCACTCCATCACCAGGAGCCATGACGAGAACCTGGAG
CGCTATGAGATCTGGAGAACAAATCCCTACTCGGAGTCTGCTGAGGAGCT
GCGTGACCGGGTCAAAGGTGTCTCAGCCAAGCCCTTCATGGAGACCCAGC
CTACCATGGATGCGCTTCACTGTGACATTGGCAATGCCACTGAGTTCTAC
AAGATCTTCCAGGATGAGATTGGTGAGGT--------------CCAC--
-CCCAGCCGCGAGGAGAGACGATGCTGGAGGTCTGCCCTTGACAAGCAGC
TCAGAAAGAAGCTGAAGTTGAAGCCAGTGATGCGAATGAACGGGAACTAC
GCCCGGCGCCTGATGACCCGAGAGGCAGTGGATGCAGTATGTGAGCTGGT
GCCCTCCGATGAGCGTAGGCAAGCCTTGAAGGAGCTGATGGAGCTCTACC
TGCAGATGAAGCCCGTCTGGCGCTCTACATGTCCTGTGCAGGAATGTCCA
GACCAGCTCTGCCGCTATAGCTTCAACTCCCAGCGCTTTGCCGAGCTCCT
CTCCTCCACCTTCAAGCACCGCTATGATGGCAAGATCACCAACTACCTCC
ACAAGACCCTAGCCCACGTGCCTGAGATCATCGAGAGGGATGGCTCTATT
GGGGCATGGGCCAGTGAAGGGAACGAGTCTGGGAACAAGTCCTATGCTAT
TGAGATGGGCCCACTGGGGCCTCAGTGGAAGGAGAGCCCCCAGCCTTTCT
CTTGCTCCATCGAGGACCCCACSAAGCAGACAAAGTTCAAGGGCATAAAG
ACCTACATCTCATACCGAGTGACACCTAGCCACACTGGCCGGCCAGTGTA

CCGCCGCTACAAGCACTTTGACTGGCTCTACAACCGGCTGCTGCACAAGT
TCACCATCATCTCCGTGCCCCACCTGCCCGAGAAGCAGGCAACCGGGCGC
TTTGAGGAGGACTTCATCGAGAAGCGCAAGAGGCGGCTCATCCTCTGGAT
GGACCACATGACGAGCCACCCGGTGCTGTCGCAGTACGAGGGCTTTGAGC
ACTTCCTCATGTGCACTGACACCAAGCAGTGGAAGCTGGGTAAGCGGCGG
GCCGAGAAGGATGAGATGGTGGGTGCCCACTTCCTGCTCACTGTCCAGAT
GCCCAATGAACACCAGGACCTGCAGGACATGGAGGAGCGGGTGGACTCCT
TTAAGGCCTTCACCAGGAARATGGACGACAGCGTCCTGCAGCTGACGCAT
GTGGCCTCTGAGCTGGTACGCAAACACCTGGGCGGCTTCCGCAAGGAGTT
CCAGAGGCTGGGGAACGCCTTCCAGTCCATCAGCCAGGCCTTCACCATGG
ACCCGCCCTACTGCTCTGATGCCCTTAACAACGCAATATCCCAC------

-------------------------------GCCAAATCCCGCTTCCACC
CTGGCGCCGGGACCGGTCCTGGCACGGACC---GCAGCGTCCCCCTTAGC
AACAGCTTGCTATCCCCGCAACAAGCCGAGGAGACCGCAGCAG---CCTC
CCCGCAGCGATGGTTTGTCACCC---CTGCCAACAATCGACTGGACTTTG
CCGCCTCGGCATACGATGCCGCCGCCGCTGCTGATTTCGCCGGTAACGCG
GCCACCCTGCTGTCTTACGCAGCTGCTGGAGTCAAGGCAC------TTCC
ACTGCCCACGGCGGGGTGCTCAGGTAGACCCCTGGGTTATTACGCCGATC
CCTCGG---GGTGG---GGCACTCGCACGCCGCCCCAGTATTGC------
---AGCAAATCCGGCTCGGTTCTATCCTGCTGGCCCTCAAATTCTGCTGG
GAGCAGGACTGGCG---CTTCAACGTATCTGG---------TGGACGA--
-GCCG---GACAC---CATCGCGACGGAGCGGTCTCCG---CT---GGGT
GGGTCCGAAGAC---GCCAAACCAAAAGACCT------GTCGGA---ATC
GAGCTGGATAGAG---ACACCTTCCTCAATAAAGTCAATCGATTCGAGCG
ATTCTGGAATTTTTG---AGCAGGCGAAACGGAGGCGGATATCGCCGTCT
GCCACGCCG---------GTTTCGGAAACCTCGTCCCCGTNNNNNNNNNNN---------A
CCGGCGAAGTCACAGACAGAGAAGTGGCTTTGGGGATAAATCCCTTTGCT
GATGGGATGGGCGCTTTTAAAATCAACCACAGCACCCACGACCTGGGCTC
TGG---ACAAACAGCGTTTTCCTCCCAAGCG---CCAGGCTAT------G
CCGCTGCCCTGGGA---CACCACCA------CCACCCCACACATGTCAGT
TCC---TATTCCACGGCGGCGTTCAATTCCACCCGGGACTTTCTGTTTCG
AAATCGTGGCTTCGGAGACGCCACGAG------------CGCTCAGCACA
GTCTTTTCGCCTCCGC---AGCGGGAAGTTT---T------GCAGGGCCA
CATGGACACTCGGATGCCACTGGGCACCTGCTTTTCCCGGGACTTCACGA

G---CAAGCGGCCACTCACGCGTCTTCCAATGTAGTAAACAGTCAGATGC
GTCTGGGCTTTTCGGGGGACATGTACGGCAGAGCCGATCAGTACGGCCAT
GTAACCAGCCCCAGGT---CTGATCATTACGCCTCGACCCAGCTGCACGG
CTATGGCCCCATGAACATGAACATGGCTGCG---CACCATGGGGCAGGGG
CCTTCTTTCGGTACATGAGGCAACCGATAAAGCAGGAACTTATCTGCAAA
TGGATCGAGCCGGAGCAATTGACGAACCCCAAAAAGTCCTGCAACAAAAC
TTTCAGTACCATGCACGAGCTGGTTACGCACCTCACAGTGGAGCACGTTG
GTGGACCAGAACAATCGAATCATATCTGCTTTTGGGAAGAGTGTCCCCGG
GAAGGAAAACCGTTTAAAGCAAAGTACAAACTTGTAAATCACATCAGAGT
ACACACCGGCGAGAAACCCTTCCCTG-----------------------
>Meiacanthus grammistes

------------TTCTTGGAAAGAAACCTTCACCCGTCCAACTGTCTCGG
TATGCTGCTTCTGTCTGATGCCCATCAGTGTACCAAGCTGTCTGAGCTCT
CCTGGGGAATGTGCCTCAGCAACTTCCCTGCGATCTGCAAGACAGARGAC
TTCCTCCAACTGCCCAAAGATATGGTGGTGCAGCTCTTGTCACATGAGGA
GCTGGAGACAGAAGATGAGAGACTGGTTTATGAAGCYGCCCTTAACTGGA
TCAACTACGACCTGGATAGGAGACACTGTCATCTTCCAGAGCTCTTGGGA
ACGGTGCGACTCGCCCTGCTGCCTGCCATCTTTTTAATGGAAAATGTCTC
AACAGAGGAGCTCATCAATGCCCAGGCCAAGAGCAAAGAGCTGGTGGATG
AGGCCATCCGCTGTAAGCTGAAGATCCTGCAAAACGATGGAGTTGTAAAC
AGCCCTTGTGCCCGTCCAAGAAAAACCAGCCACGCCCTGTTTCTTCTTGG
CGGGCAGACTTTCATGTGCGACAAGTTGTACTTGGTTGACCAGAAGGCCA
AAGAGATCATCCCGAAAGCTGACATCCCCAGCCCAAGGAAGGAATTCAGT
GCTTGTGCTATTGGCTGTAAGGTGTACATCACAGGTGGAC--GAGGCTC-
GGAGAATGGCGTGTCCAAAGATGTATGGGTCTATGACACGGTCCATGAGG
AATGGTCAAAAGCCGCGCCCATGCTCATCGCCCGGTTCGGCCATGGATCT
GCAGAGCTTAAGCACTGTCTCTACGTTGTAGGAGGTCACACTGCTGCGAC
TGGTTGCCTCCCGGCTTCTCCGTCAGGACGAGTACGTCGTGGTGTTCAGC
CGCTCCGCCACGAGACTGATTCTAAACGAGGCCGAACTGATCATGGCGCT
GGTCCAGGAGTTCCAGATGAAGGTGGTCACGGTGTCGTTAGAAGAACAGT
CTTTCCCCAGCATCGTCCAGGTGATCAGCGGCGCCTCCGCGCTAGTCAGC
ATGCACGGCGCCCAGCTCATCACCTCGCTCTTCCTCCCCAGGGGAGCTGC
TGTGGTGGAGCTCTTCCCCTTCGCGGTGAACCCCGAACAATACACCCCGT
ACAAAACCCTGGCCTCCTTGCCAGGCATGGACCTCCACTACGTCTCCTGG
AGGAACACCATGGAGGAGAACACCGTCACCCACCCAGACAGGTCCTGGGA
ACAAGGAGGCATCGCACACTTGGAGAGAGCGGAGCAGGAGCGCATCCTGG
CGAGCAAGGACGTCCCCAGACACCTCTGCTGCCGAAACCCGGAGTGGCTC

TTCAGGATCTACCAGGACACCGCCGTAGACATCGCGTCCTTCCTGGAAGC
CGTGCG---GGAGGGCATGAAG---TCCAAACCCGGCTCCAAGAA---AT
CCAAGGCGTCCGGCGTCGTCCACCCCGGCCGTGTCAGGGAGCCCCAGTGT
CAGACCTCGGTCCAGACCAGCAACGAGGCCAAGCTCACCGTCTCCTGGCA
GATCCCGTGGAATCTCAAGTATCTGAAAGTGCGAGAGGTGAAGTACGAGG
TGTGGATCNNNAAGAAAGACACCAGCAAAGGTACCCTGGAGGACCAGATCATC
CAGGCCAATCCAGCTTTGGAAGCCTTTGGTAATGCCAAAACAGTGAGGAA
CGACAACTCATCCCGGTTTGGAAAATTCATCCGGATTCACTTTGGCACAA
GTGGAAAGCTGTCGTCCGCCGACATTGAAACATACCTGCTGGAAAAGTCA
CGGGTGACGTTTCAGCTGAAGGCTGAGAGAGACTACCACATCTTTTATCA
AATTCTGTCCAATCAGAAGCCAGAGCTGCTGGACATGCTCCTGATCACCA
ACAACCCCTACGACTACTCCTACATCTCTCAGGGTGAGGTGACGGTCACC
TCCATCAACGATTCCGAGGAGCTGATGGCAACCGACAGCGCCTTTGACGT
GCTGGGCTTCACACCAGACGAGAAGATGGGCGTGTACAAGCTGACCGGCG
CCATCATGCATTACGGCAACATGAAGTTCAAACAGAAGCAACGCGAGGAG
CAAGCTGAGCCCGATGGCACTGAAGCAGCCGACAAGTCRGCCTATCTGAT
GGGGCTGAACTCGGCCGACCTCATCAAGGGATTGTGCCATCCAAGGGTCA
AGGTTGGAAATGAGTACGTCACCAAAGGTCAAAGTGTGGACCAAGTCTAC
NNNCCTAACAAGGAAGCCTTCAAGTGTGAGGAGTGTGGGAAGCACTACAACAC
CAAGCTGGGATACAAGCGCCATGTGGCCATGCACTCTGCCACGGCGGGGG
ATCTGACCTGTAAAGTGTGTATGCAGAGCTACGAGAGCACGCCCGTTCTC
CTGGAGCACCTCAAGAGCCACTCGGGAAAGTCTTCGGGTGGTGCCAAGGA
GAAGAAGCACCCGTGCGACCACTGCGACCGTCGTTTCTACACGCGGAAAG
ACGTGAGGCGGCACATGGTGGTCCACACCGGTCGAAAGGACTTCCTGTGC
CAGTACTGCGCACAGCGCTTCGGCAGGAAAGACCATCTGACGCGCCACGT
GAAGAAGAGCCACTCGCAGGAGCTGCTGAAGATCAAGACGGAGCCTCCGG
ATATGCTAGGTCTTTTGGCCACGGGGTCGCCGCCATGCTCGGTGAAGGAA
GAGCTCANCCCCATGATGTGCGGCATGGGGCCCAACAAAGATCCCATGATG
GGCAAACCATTTCCCAGTGGAGCCCCTTTCCCGATGGGCATGTACAACCC
CCATCAC------CTTCAGGCCATGTCAAATTCTGGGGTGGGCCACCCG-
-----CACCCGCCCCTGATGCCAGGCCCTTTGTCTGCAGCTATGGGCATG
GGATGCCACATGGAGTATCTCATCTACGCGTCTTTCTCCTTCATGGGATG
TTTACAAATTAGTGATGGATCGAACATCGTGAACTTGCTGGCTAGTAACT
CTCCCAGTGTTTCCTATGCTCTGACCCAGCAGAAATACTTCAGTAACTAC
AGTCCCGTGATTGGGTTTTATATTTACGAGCCAATCGAGTATTGGAACTC
CACCGTGCAGGAGCACCTGAAAACTCTGAGTCACGGCTTCAACAAAATCT
CCTGGATGGACAATTTTTTCCACTACCTGCGGGTGGTGAACGTGAGCGCC
TCAACGAAGAACGACTTCATCACCATCCTGAAGGGTTCGTTCCTCCGGAG
CCCGGAGTACCAGCACTTCACCGAGGACATCATATTCTCAAAGA---ACC
CAGAGACCG------ATGAGTACGACATCATCGCGTCGAGGATGTACTTG
GTCGCGCGGACCATGGAGAAGAAGCGCGAGGAGGTGGTGGAGCTTTTGGA
AAAGCTCCGCCCTTTGATGCTCATCAACAGCATCAAATTCATTGCCTTCA
ATCCCACGTTTGTGTTTATGGACCGCTACAGCTCCTCCGTCATCTCACCT
ATCCTGACCTCAGGCTTCAGCGTACTCACCATCCTCATCCTCACTTTCTT
CCTTGTCATCAACCCACTGGGGAACTTCTGGCTCATCCTTACAGTTACGT
CCGTGGAGTTGGGCGTCTTGGNNNNNNNNGGTTTTCACCAGTTTGAGTGGCAGCCAGC
CCTCAAGAATGTGTCGACAACTTGCTGTGTTGGCATTATTAATGGGCTCG
CTGGATCCGCGTTCTCGGTGGATGATGCCCCGGCCGACACCATCACTCGC
CGCTTTCGCTATGATGTGGCACTAGTGTCAGCATTAAAGGATCTAGAGGA
GGACATCATGGAGGGATTGAGAGAGAATGGGATGGAAGACAGTGCTTGCA
CCTCAGGCTTTAGTGTTGTGATCAAGGAGTGCTGTGATGGCATGGGTGAT
GTCAATGAGAAGCATGGTGGTGGACCAATTGTTGCYGAGAAGGCTGTGCG
TTTCTCTTTCACTGTGATGTCTGTCTCAGTCCTGCCAGATGATGAGGAG-

------------------------------GGAGAGGTTTCCATTTTCACT
GAGCCAAAGCCCAACTCAGAACTATCCTGTAAGCCCCTCTGTCTTATGTT
TGTGGATGAGTCAGACCATGAGACACTCACAGCTGTCCTGGGGCCCATAG
TCGCAGAGCGCAATGCAATGAAAGAAAGCAGACTCATCCTGTCCATGGGA
GGCTTGCTCCGTTCATTTCGCTTCCACTTTAGGGGCACCGGGTATGATGA
GAAGATGGTGCGTGAGATGGAAGGACTGGAAGCCTCCGGGTCTTCATATG
TCTGTACACTGTGTGACGCCAGTCGTGCAGAGGCCTCTCACAACATGGTG
CTGCACTCAATCACACGTAATCATGAAGAGAACTTAGAACGTTATGAAAT
ATGGAGAACCAATCCCTTCTCTGAGTCTGTAGATGAGCTACGAGAAAGAG
TAAAAGGAGTGTCTGCCAAACCCTTTATGGAGACTCACCCCATGCTCGAC
GCACTGCACTGCGACATTGGCAATGCCACTGAATTCTACAAAATCTTCCA
GGACGAGATTGGAGAAGTTTACAAAAAGGT---CAGT---CCCAGCCGGG
AGGAACGCCGCAGCTGGCGGGCAGCCCTCGATAAACAGCTGAGGAAGAGG
ATGAAGCTTAAACCGGTAATGAGGATGAATGGGAACTATGCCCGCAGGCT
AATGACCCAGGAGGCTGTGGAGGTGGTGTGTGAGCTTGTGCCCTCAGAGG
AGAGGAGGGAGGCCCTGAGGGAGCTTATGAGGCTGTATATACAGATGAAG
CCTGTGTGGCGTGCCACCTGCCCAGCAAAGGAGTGCCCTGACCAGCTGTG
CCGCTACAGCTTCAACTCCCAGCGCTTTGCTGACCTCCTCTCCTCTACCT
TTAAATATAGGTACAATGGAAAGATAACCAATTATCTGCACAAGACCCTT
GCTCATGTTCCTGAGATCATTGAGAGAGATGGATCTATTGGAGCCTGGGC
CAGCGAGGGAAACGAGTCAGCTAACAAATCGTACACCATTGAGATGGGGC
TTCTGGGACCCCGGTGGAAAGACAACCCACAGCCTTTCTCCTGCTCCATT
GAAGACCCCACCAAACAGACCAAGTTTAAAGGTATCAAAACCTACATATC
GTACCGGGTCACCCCGAGCCACACGGCGCACCCCGTCTACAGACGCTACA
AACACTTTGACTGGCTGTACAACCGCTTACTGCACAAGTTCACTGTGATC
TCCGTGCCCCACCTGCCCGAGAAGCAGGCCACGGGCCGATTCGAGGAGGA
CTTCATTGAGAAGCGCAAGAGGCGACTGATCCTGTGGATGAACCACATGA
CCAGTCACCCGGTCCTTTCCCAGTACGAGGGCTTCGAGCACTTTCTCATG
TGCGCCGATGATAAACAGTGGAAACTGGGCAAGAGGCGGGCGGAGAAGGA
CGAGATGGTGGGTGCCCACTTCATGCTGACCCTCCAAATCCCCAACGAGC
ACCAGGACCTTCAAGACGTCGAGGAGAGGGTGGACAACTTCAAGGCCTTC
GCTAAGAAAATGGACGACAGCGTGCTGCAGCTCACTCACGTTGCCTCGGA
GTTGGTGCGCAAGCACTTGGGTGGATTCAGGAAAGAGTTTCAACGGCTGG
GTAATGCTTTCCAGTCCATCAGCCAGGCGTTCATGCTGGACCCTCCCCAT
AGGTCAGACACCCTCAACAACGCCATCTCCCATNNNNNNNNNNNNNNNNNNNNNNNNNNNNNNNNNN
NNNNNNTTGGCGTCGGTGTGGTT
GGAAACCTCCTGATCTCCATCCTGCTGGTAAAAGACAAGAGCCTGCACCG
AGCACCCTACTATTTCCTGCTAGACCTGTGCGCCTCTGACATCCTGCGCT
CGGCCATTTGCTTCCCCTTTGTCTTCACGTCGGTCAAGAATGGATCTGCC
TGGACCTACGGCACGTTGACCTGCAAAGTGATTGCCTTCCTCGGGGTGCT
CTCCTGTTTCCACACGGCGTTCATGCTTTTCTGTGTCAGCGTGACCCGGT
ACCTGGCCATCGCTCACCACCGATTCTACACAAAAAGGCTAACTTTCTGG
ACGTGCTTGGCCGTCATCTGCATGGTGTGGACTTTATCCGTGGCTATGGC
GTTCCCACCGGTGCTAGACGTGGGGACGTACTCTTTTATCCGCGAGGAGG
ACCAGTGCACGTTTCAGCACCGTTCCTTCAGGGCGAATGATTCGTTGGGC
TTCATGCTCCTGCTGGCACTCATTCTCCTCGCCACACAGCTGGTTTACCT
CAAGCTCATCTTCTTCGTCCACGACCGCCGAAAAATGAAGCCCGTCCAGT
TTGTGCCTGCCGTCAGCCAGAACTGGACCTTCCATGGGCCGGGCGCCAGC
GGGCAGGCGGCGGCTAATTGGCTGGCCGGATTTGGTCGAGGCCCCACCCC
GCCTACTTTGCTGGGAATCCGGCAAAACAGCAACGCGTCGGGCCGCAGGC
GCCTACTGGTATTAGATGAATTCAAAACGGAGAAGAGGATTAGTAGGATG
TTCTACATCATGACGTTTTTCTTCCTGGCACTGTGGGGGCCCTACCTGGT
TGCCTGCTACTGGCGGGTGTTTGCAAGGGGCCCCGTAGTTCCTGGGGGCT

ACCTGACAGCAGCCGTGTGGATGAGCTTTGCTCAGGCTGGGGTCAATCCT
TTCATCTGCATTTTCNNNNNNNNNNNNGCCAAATCTCGCTTTCACCCTGGCGTGGGGACTGG
TCCTGGCACGGAGC---GCAGCGTCCCACTCGGCAACAGCTTGCTATCCC
CGCAGCAAAGCGAGGAGCCCACTGTTGCCACCCCCCCGCAGCGATGGTTT
GTCACCC---CTGCCAACAACCGACTGGACTTTGCTGCCTCGGCATACGA
CGCCGCT---------GATTTTGCCGGTAACGCGGCCACCTTGCTGTCCT
ACGCAGCGGCCGGAGTGAAAGCTC------TTCCCCTGTCGACTGCAGGC
TGCTCCAATCGGCCCCTTGGCTACTACACAGACCCGTCTG---GCTGG--
-GGGGGACGCACGCCGCCGCAGTACTGCGGCGTAAACGCCAAATCCGGCT
CGGTGTTTTCCTGCTGGCCCACTAACTCCATCGGTAGCAGGGCCGGGG--
-CC---AACTACCTGT---------CCGAGGA---GGGA---GACTC---
CATCACCACGGAGAGGTCGCCC---AT---CGGCGCCTCGGAGGAG---G
CCAAACCCAAAGACATGAC---GTCCGA---GTCGAACTGGATAGAA---
ACGCCGTCCTCCATCAAGTCCATCGATTCCAGCGACTCTGGCATCTTCG-
--AGCAGGCCAAGAGAAGGAGAATCTCACCTTCAGCCACGCCG-------
--GTCTCAGAGACAGTGTCCCCCTCAAANNNNNNNNNNNCTCAACAGGCGAAGTCACAGAG
AGAGAAGTGGCGTTGGGGATAAATCCGTTCGCGGATGGGATGGGCGCCTT
CAAAATCAACCACAGCTCCCACGACATTGGCTCCGG---ACAGACGGCGT
TCTCCTCCCAGGCG---CCCGGCTAC---GCGGCGGCCGCGCTGGGA---
CACCATCA------CCACCCGACCCACGTTGGCTCT---TACTCCACGGC
GGCTTTCAACTCCACCAGGGACTTTCTCTTCAGAAATCGGGGTTTCGGGG
ATGCCGCCGG------------GGCGCAGCACAGTTTGTTCGCCTC----
-----CGGGAGTTT---T------GCAGGGCCACATGGACACTCGGATGC
GGCGGGGCACCTGCTATTCCCGGGGCTGCACGAG---CAGGCGGCGAGCC
ACGCGTCTTCGAACGTCGTCAACAGCCAGATGCGGCTGGGTTTCTCGGGG
GACATGTACGGACGGGCCGACCAGTACGGCCACGTCACGAGTCCGAGGT-
--CCGATCACTACGCCTCGACCCAGCTGCACGGCTACGGCCCCATGAATA
TGAACATGGCCGCG---CACCACGGAGCAGGGGCCTTCTTCCGTTACATG
AGGCAGCCCATCAAGCAAGAACTCATCTGCAAGTGGATCGAACCGGAGCA
GCTGACGAACCCCAAAAAGTCGTGTAACAAAACTTTTAGCACCATGCACG
AGCTGGTGACCCACCTGACGGTGGAGCATGTGGGGGGACCCGAGCAGACC
AACCACATTTGCTTCTGGGAGGAATGCTCCAGAGAAGGAAAGCCATTCAA
AGCCAAATACAAACTTGTAAATCATATCAGAGTTCACACCGGAGAAAAGC
CCTTTCCGTGTCCGTTCCCCGGCTGTGGCAAA
>Melamphaes polylepis

------------TTTCTAGAGAGGAACCTCTACCCATCCAACTGCCTTGG
CATGCTGTTGCTGTCCGATGCTCACCAGTGTACCAAGCTGTCAGAGCTCT

CCTGGGGCATGTGCCTCAGCAACTTTCCTGCTATTTGCAAGAAGGAAGAC
TTCCTCCAACTGCCCAAAGACATGGTGGTGCAGCTCCTGTCCCACGAGGA
GCTGGAGACAGAAGACGAGAGACTGGTTTATGAAGCTGCCCTCAACTGGG
TCAACTACGACCTGGAAAGGAGGCACTGCCAACTGCCAGAGCTGCTGAGA
ACGGTCCGGCTGGCCCTGCTGCCCGCCATATTCCTCATGGAGAACGTCTC
CACAGAAGAGCTGATCAACGCCCAGGCCAAGAGCAAGGAGCTGGTCGATG
AAGCCATCCGCTGCAAGCTGAAGATCTTGCAGAACGACGGTGTGGTGAAC
AGCCCCTGTGCTCGACCGAGAAAAACCAGCCACGCCCTCTTTCTGCTGGG
TGGGCAGACCTTCATGTGTGACAAGCTGTACCTGGTGGACCAGAAGGCCA
AAGAGATCATCCCCAAGGCAGACATCCCCAGCCCCAGGAAGGAGTTCAGC
GCCTGCGCCATCGGCTGTAAGGTCTACATCACAGGTGGGA--GGGGCTC-
AGAGAATGGTGTGTCCAAAGATGTGTGGGTCTACGACACTGTCCACGAGG
AATGGTCCAAGGCAGCACCCATGCTCATCGCCAGGTTCGGTCATGGCTCT
GCCGAGCTGAAACACTGCCTCTATGTGGTCGGAGGTCACACAGCTGCCAC
TGGCTGCCTCCCAGCCTCCCCCTCCGGAAGAATACATCATCATGTTCAGT
CGTTCAGTGACGAGGCTGATCCTGAATGAAGCAGAGCTAATCATGGCGCT
GGCACAGGAGTTTCAGATGAGAGTGGTTACGGTGTCCCTAGAGGAACAAA
CTGTTGCCAGCATTGTCCAGTTGATCAGCGGGGCTTCCATGTTAGTCAGC
ATGCATGGAGCTCAGCTAGTCACCTCACTCTTCCTCCCCAGAGGAGCTGC
TGTGGTGGAGTTGTTCCCCTATGCTGTGAATCCAGAACAGTACACCCCAT
ACAAAACGCTTGCCTCCCTACCAGGCATGGACCTTCAATACGTGGCCTGG
AGGAACACTATGGAGGAGAACACCATCACCCACCCAGGCCGACCCTGGGA
ACAAGGCGGCATCGCTCATTTGGAAAAGGACGAGCAAGAGCGAATACTGG
CCAGCAGGGATGTTCCCAGGCACCTGTGCTGCCGCAACCCGGAGTGGCTC
TTCCGAATCTACCAAGACACTTTGGTGGACATCCCTTCATTGCTGGACGT
CCTCAA---AGAGGGCCTGAGG---GCCAGGCCCAGCTTAAAGAA---GG
CCAAGCCGGCCAGCACCGTTCACCCGGGCCGGGTCAGAGAACCCCAGTGC
CAAACCTCAGTCCAAGCCACCAACGAGGCAAAACTCACAGTTTCCTGGCA
GATCCCGTGGAACCTGAAGTACCTAAAGGTGAGAGAAGTGAAGTATGAGG
TGTGGATCCAGAAGAGGGATACCAGCAAGGGAACACTGGAAGATCAAATC
ATCCAGGCAAACCCTGCCCTGGAGTCCTTTGGTAACGCCAAAACACTGAG
GAATGACAACTCATCCCGTTTTGGAAAATTCATCCGAATTCACTTTGGAA
CCAGTGGCAAGCTGTCGTCTGCTGACGTTGAGACTTACCTACTGGAGAAG
TCACGTGTCACCTTTCAGCTCAAGGCTGAGAGGAACTATCACATCTTCTT
CCAGATCTTGTCCAATCAGAAGCCAGAGCTTCTGGACATGCTGCTGATTA
CCAACAACCCATACGACTACTCTTTCATCTCCCAAGGAGAGGTAACAGTA
CACTCCATCAACGATTCAGAGGAACTGATAGCCACTGATAGCGCCTTTGA
TGTGCTTGGCTTCAGTTCAGATGAGAAGATGGGAATCTACAAGCTGGTCG
GTGCCATTATGCACTATGGCAACATGAAGTTCAAGCAGAAGCAGCGTGAG
GAGCAAGCTGAGCCTGACGGAACAGAGGCTGCTGACAAGGCAGCTTACCT
GATGGGGCTGAACTCTGCAGATCTCATCAAAGGACTGTGCCATCCCAGAG
TCAAGGTAGGAAATGAATATGTCACCAAAGGCCAGGGTGTAGAACAAGTC
TACTACCCCAACAAGGAGGCCTTCAAGTGCGAGGAGTGCGGCAAGCACTA
CAACACCAAGCTGGGATACAAGCGCCATGTGGCCATGCACTCGGCCACGG
CGGGGGACCTCACCTGCAAAGTGTGCATGCAGAGCTATGAGAGCACGCCC
GTGTTGCTGGAGCACCTCAAGAGCCACTCGGGGAAGTCCTCAGGGGGCGC
CAAGGAGAAGAAGCACCCGTGTGACCACTGTGACCGCCGCTTCTACACCC
GCAAGGATGTGAGACGGCACATGGTGGTCCACACGGGCCGAAAGGACTTC
CTGTGCCAGTACTGCGCCCAGCGCTTTGGCAGGAAGGACCACCTGACGCG
GCACGTCAAGAAGAGTCACTCGCAGGAGCTGCTGAAGATCAAGACGGAGC
CTCCGGATATGTTAGGGCTTTTAGGTTCCGGTTCCCCGCCTTGCTCTGTC
AAGGAGGAGCTCAGCCCTATGATGTGCAGCATGGGTCCCAACAAAGACCC
TATGATGGGCAAGCCCTTCCCCAGCGGTACCCCCTTCCCGATGGGCATGT

ACAACCCTCACCAC------CTCCAGGCCATGTCCAATCCTGGGGTGGGT
CACCCG------CACCCCTCCCTGATGCCCGGTCCCCTGTCTGCAGCTAT
GGGCATGGGCTGCCACATGGAGTATCTCATCTACGCCTCRTTTTCATTCA
TGGGATGTTTACAAATCAGCGATGGGTCAAACATTGTGAACTTGCTGGCT
AGTAACTCTCCAAGCGTTTCGTTTGCTCTGACCCAGCAGAAATACTTCAG
TAACTACAGCCCCGTGATTGGGTTTTACATTTACGAGCCCATTGAGTACT
GGAACTCCACGGTGCAGGAGCATCTGAAGACACTGAGTCATGGTTTCAAC
AAGATCTCCTGGATGGACAACTTCTTCCACTACCTGCGGGTGGTGAACGT
GAGTGCGTCGACCAAGAGCGACTTTATCACCATCCTCAAGGGTTCCTTCC
TGCGCAGCCCGGAGTACCAGCACTTCACCGAGGACATCATCTTCTCCAAG
A---ACCGCGAGAGYG------ACGAGTATGACATTATTGCCTCGCGCAT
GTACCTGGTGGCGCGGACCACGGAGAAAAGGCGCGAGGAGGTGGTGGAGC
TTCTGGAGAAGCTGCGTCCGCTGATGCTGATCAACAGCATCAAGTTCATC
GCCTTCAACCCCACCTTTGTTTTATGGACCGCTACAGCTCCTCTGTCAT
CTCGCCCATCCTGACCTCAGGCTTCAGCGTGCTCACCATCCTCATCCTCA
CATTCTTCCTGGTCATCAACCCCTTGGGGAACTTCTGGCTCATCTTGACG
GTTACTTCTGTGGAACTGGGCGTCTTGGGTTTAATGNNNNNNNNNNNNNNNNNAGTGGCAGCCGGCC
CTCAAAAATGTGTCTGCATCGTGCCATGTCGGCATTATTAACGGGCTCTC
TGGATGGGCTGCCTCAGTGGACGACTCCCCAGCTGACACCATCACTCGGC
GATTCCGCTATGATGCGGCCCTGGTGTCGGCCTTAAAGGATCTGGAGGAG
GATATCATGGAGGGACTGCGAGAGTGTGGGCTGGAAGACAGTGCTTGCAC
CACGGGCTTCACTGTTTTGATCAAGGAATCGTGTGACGGCATGGGAGATG
TCAGTGAAAAGCACGGTGGCGGGCCTATGGTCCCCGAGAAGGCTGTACGT
TTCTCTTTTACTGTCATGTCTGTCTCTGTCTTGGCAGAAGGAGAGGAG--
----------------------------GAGGAGGTTACCATCTTCAGGG
AGCAAAAGCCGAACTCAGAACTGTCCTGTAAGCCCCTATGTCTGATGTTT
GTGGATGAGGCAGACCACGAGACACTCACTGCCGTCCTGGGGCCCGTCGT
TGCAGAGCGAAACGCTATGAAGCAGAGTCGACTCATCCTATCTGTGGGTG
GCCTGCCTCGCTCATTTCGCTTCGAGTTCAGAGGCACGGGATACGATGAG
AAGATGGTGCGTGATTTGGAGGGCCTGGAGGCCTCAAGCTCCACCTACAT
CTGCACTCTGTGTGACTCCACACGGGCAGAGGCCTCCCACAACATGGTGC
TGCACTCCATCACCCGCAGTCATGACGTAAACCTGGAGCGTTATGAAATA
TGGAGGACCAATCCGTTTTTCGAGTCCGCCGACGAGCTGCGAGACCGGGT
CAAAGGTGTCTCCGCCAAGCCCTTCATGGAGACCCATCCCACTCTGGATG
CATTGCACTGTGACATNGGTAATGCTACAGAGTTCTACAAAATCGTCCAGG
ATGAAAACGGGGAGTGCTACCAAAAGGC---CAAC---CCCAGCCGGGAG
CAGCGGCGGAGCTGGAGGGCAGCTCTAGACAAGCAACTGAGGAAGAAGAT
GAAGCTTAAACCGGTGATGAGGATGATTGGGAACTATGCCCGGAAGCTAA
TGACGAAGGAGGCCGTGGAGGTGGTGTGTGAGCTGGTGCCCTCAAAGAAG
AGGAAGCAGGCCCTGAGGGAGCTTATGGGGCTCTCTCTGCAAATGAGGCC
TGGTGTGGCTGCCACCTGCCCAGCAAAAGGATGCCNNNNNNNNNNNNNNNNNNNNNNNNNNNNNNNNNN
NNNNNNNNNNNNNNNNNNNNNNNNNNNNNNNNNNNNNNNNNNNNNNNNNNNNNNNNNNNNNNNNNNNNNN
NNNNNNNNNNNNNNNNNNNNNNNNNNNNNNNNNNNNNNNNNNNNNNNNNNNNNNNNNNNNNNNNNNNNNN
NNNNNNNNNNNNNNNNNTCGTACACCATTGAG
ATGAGCCCCAAGGGGCCCCAGTGGAAGGAGAGTCCGCAGCCCTTCTCCTG
CTCTGTTGAAGACCCCACAAAGCAGACCAAGTTCAAGGGCATCAAGACCT
ACATCTCGTACCGGGTCACGCCGAGCCACACGGGACGTCCCGTCTACAGG
CGCTACAAACACTTTGATTGGCTGTACAGCCGCCTGCTGCACAAGTTCAC
TGTGATCTCCGTGCCCCACCTGCCGGAGAAGCAGGCCACGGGCCGCTTCG
AGGACGACTTCATCGAGAAGCGCAAGAGAAGGCTGATCCTGTGGATGAAC
CACATGACCAGCCATCCGGTCCTCTCCCAGTACGAGGGCTTCGAGCACTT
CCTGATGTGTGCCGACGAGAAGCAGTGGAAGCTGGGTAAGAGGCGGGCGG
AGAAGGACGAGATGGTGGGCGCCCACTTCATGCTGACCTTCCAGATCCCT

AACGAGCACCAGGACCTCCAGGACGTGGAGGAGCGAGTCGACACCTTCAA
GTCCTTCGCCAAGAAAATGGACGACAGCGTGATGCAGCTCACGCACGTGG
CCTCGGAGCTGGTGCGCAAACACCTGGGGGGCTTCAGGAAGGAGTTCCAG
CGGCTGGGAAACGCCTTCCAGTCCATCAGCCAGGCCTTCATGCTGGACCC
TCCCCACAGCTCCGATTCTCTCAACAACGCCATCTCCCAC----------

---------------------------GCCAAATCTCGCTTTCACCCTGG
CGTGGGGACTGGTCCTGGCACGGAGC---GCAGCGTCCCACTCGGCAACA
GCTTGCTGTCCCCGCAGCAAACCGACGAGCCCACTGTTGCCACCCCCCCG
CAGCGATGGTTTGTCACCC---CTGCCAACAACCGACTGGACTTTGCTGC
CTCGGCATACGACGCCGCT---------GATTTCGCCGGTAACGCGGCCA
CCTTGCTGTCCTACGCAGCGGCCGGAGTGAAAGCTC------TTCCCCTG
CCGACTGCAGGCTGCTCCAACCGGGCTCTTGGCTATTACGCCGACCCGTC
AG---GCTGG---GGAGGACGCACACCGCCACAATACTGTGGCGTAAACA
GCAAATCCAGCTCGGTATTTTCCTGCTGGCCTGCTAACTCTATCGGGGGC
AGAGCGGGCA---CC---AACTACCTGG---------CTGAGGA---GGG
A---GACTC---CATCCCGACAGAGAGGTCACCG---AT---CGGCGGCT
CAGAGGAG---ACCAAACCCAAAGACATGAC---ATCTGA---GTCCAGC
TGGATAGAG---ACTCCGTCCTCCATTAAATCCATTGATTCGAGCGACTC
TGGTATCTTTG---AACAGGCCAAAAGGAGAAGAATCTCACCTTCTGCCA
CGCCG---------
GTTTCAGAGACAGTGTCCCCGTTAAAATCTNNNNNNNNNNNNNNNNNNNNNNNNNNNNNN
NNNNNNNNNNNNNNNNNNNNNNNNNNNNNNNNNNNNNNNNNNNNNNNNNNNNNNNNNNNN
NNNNNN---NNNGAC
GGCGTTCTCCTCGCAGGCT---CCCGGCTAT---GCAGCGGCCGCCCTGG
GA---CACCATCA------CCACCCGACCCACGTCGGCTCTGCCTACTCC
ACGGCGGCTTTCAACTCCACGCGGGACTTTCTCTTCCGAAATCGGGGCTT
CGGAGACGCCGCCGG------------GGCGCAGCACAGCTTGTTCGCCT
C---------CGGGAGTTT---C------GCAGGGCCACATGGACACTCT
GATGCGGCGGGGCACCTGCTCTTCCCGGGGCTCCACGAG---CAAGCAGC
GAGCCACGCGTCTTCCAACGTGGTCAACAGCCAGATGCGACTGGGCTTCT
CGGGGGACATGTACGGTCGGGCAGATCAGTACGGCCACGTTACAAGCCCG
AGGT---CCGACCACTATGCTTCGACCCAGTTGCACGGCTATGGTCCCAT
GAACATGAATATGGCCGCA---CACCATGGAGCAGGGGCCTTCTTTCGAT
ACATGAGGCAGCCGATCAAGCAAGAGCTCATCTGCAAGTGGATCGAGCCG

```
GAGCAGCTCTCGAACCCGAAAAAGTCGTGCAACAAAACTTTTAGCACGAT
GCACGAGCTGGTGACCCATCTGACGGTGGAGCATGTGGGGGGGCCAGAGC
AGTCCAACCACATCTGCTTCTGGGAAGACTGCGCCAGAGAAGGGAAGCCG
TTCAAAGCCAAATACAAACTTGTGAATCATATCAGGGTACACACCGGAGA
AAAGCCCTTCCCGTGTCNNNNNNNNNNNNNNNNNNN
>Melanonus zugmayeri
AGTCTTCTCATTCGAGCAGAACTAAGCCAACCCGGTGCCCTTCTGGGCGA
TGATCAAATTTATAACGTGATCGTCACGGCACATGCATTCGTAATAATTT
TCTTTATAGTAATACCCCTAATAATTGGAGGGTTCGGAAACTGACTCGTC
CCCCTAATGATTGGAGCCCCAGACATAGCCTTCCCCCGTATAAATAACAT
AAGCTTCTGACTTCTCCCCCCATCATTTTTACTTCTCTTAGCATCTTCCG
GAGTAGAAGCCGGGGCAGGAACAGGATGAACTGTGTATCCCCCACTAGCA
GGAAACCTGGCCCACGCCGGAGCCTCTGTTGATTTAACTATTTTCTCCCT
TCATCTAGCAGGTGTCTCATCAATTCTTGGGGCCATTAATTTCATTACCA
CTATTATTAATATGAAACCCCCCGCAATTTCACAATATCAGACCCCCCTA
TTCGTCTGATCAGTCTTAGTTACAGCCGTACTTCTACTATTATCACTACC
CGTACTAGCAGCTGGAATTACAATACTTTTAACTGACCGAAATTTAAATA
CTTCCTTCTTTGACCCCGCCGGAGGAGGAGATCCAATTCTATACCAACAC
TTA-----------------------------------------------
--------------------------------------------------
--------------------------------------------------
--------------------------------------------------
--------------------GAGGAACCTTCACCCCACCAACTGCCTTGG
CATGCTGCTGCTGTCCGACGCCCACCAGTGCACCAAGCTTTCCGAGCTCT
CTTGGAGCATGTGCCTGAGCAACTTCCCTACCATTTGCAAGACAGAGGAT
TTTCTCCAACTGCCCAAAGACATGGTTGTGCAACTTTTATCCCAYGAGGA
GCTGGAGACGGAAGACGAGAAACTTGTTTACGAGGCCGCGCTTAACTGGG
TCACCTATGACCTGCAAGGGAGGCACCCCCACTTGCCAGAACTGCTGAAA
ACAGTGCGCCTGGCCCTTCTGCCCGCCATCTTCCTCATGGAAAATGTCTC
CACGGAAGAACTGATCAACGCCCAGACCAAGAGCAAGGAGCTGGTGGACG
AGGCCATCTGCTGCAAGCTGAAGATCTTGCAAAACGACGGCGTGGTCAAC
AGCCCGTGTGCCCGTCCAAGGAAGACCAGCCATGCACTTTTTCTGCTGGG
CGGGCAGACCTTCATGTGTGACAAGCTATACCTGGTGGACCAGAAAGCCA
AAGAGATCATCCCCAAGGCCGACATACCCAGCCCCAGGAAGGAGTTCAGC
GCTTGTGCCATCGGCTGCAAGGTGTACATCACGGGCGGAC--GAGGCTC-
GGAGAACGGCGTGTCCAAAGACGTGTGGGTGTACGACACGGTACAGGAGG
AGTGGTCCAAGGCGGCGCCCATGCTCATCGCCCGCTTCGGCCACGGCTCC
ACGGAGCTGAAACACTGCTTGTACGTGGTGGGCGGACACACGGCCGCCAC
CGGCTGCCTCCCG-------------------------------------
--------------------------------------------------
--------------------------------------------------
--------------------------------------------------
--------------------------------------------------
--------------------------------------------------
--------------------------------------------------
--------------------------------------------------
--------------------------------------------------
--------------------------------------------------
--------------------------------------------------
--------------------------------------------------
--------------------------------------------------
--------------------------------------------------
--------------------------------------------------
```


----------------------CTTCTCCGTCATGATAAAAGAGTCCTGC
GACGGCATGGGAGACGTGAGCGAGAAGCATGGCGGAGGGCCGGCGGTCCC
GGAGAAAGCTGTGCGTTTCTCTTTCACCGTTATGTCTGTCTCTTTGCACA
TGGCAGATGGAGAGGAGGAGGAG---------------------GAGGCA
GTGCTGATCTTCCAGGAGCCAAAGCCCAACTCTGAACTGTCCTGTAAACC
CTTGTGCCTTATGTTTGTAGATGAGTCAGACCATGAGACGCTCACTGCCG
TCCTGGGGCCCGTGGCAGCCGAGCGCAGTGCAATGAAGTGCAGCCGGCTC
ATCCTTTCCATCGGCGGGCTTCCGCGGTTCTTTAGCTTCCACTTCAGAGG

```
CTCGGGCTACGATGAGAAGATGGTGCGGGACGTGGAGGGCTTGGAGGCCT
CCGGCTCCACTTACGTCTGCACGCTTTGTGACTCCACCCGTGCCGAGGCC
TCCCACAACATGTTGCTTCACTCTGTCACCCGCAGCCACGCGCAGAACCT
GGAGCGCTACGAGGTCTGGAGGACCAACCCCTTCTCGGAGTCCGTGGACG
AGCTGAGAGACAGAGTGAAGGGGGTCTCCTCCAAGCCCTTCCTGGAGACC
CAGCCGACGCTGGACGCGCTGCACTGTGACATCGGCAACGCCACAGAGTT
CTACAAAATCTTCCAGGACGAGATCGGCGAGGTGTTCCAGAAAAC---CA
AC---CCCACCCGGGAAGAGCGGCGGAGCTGGAGGGCG------------
--------------------------------------------------
--------------------------------------------------
--------------------------------------------------
--------------------------------------------------
--------------------------------------------------
--------------------------------------------------
--------------------------------------------------
--------------------------------------------------
--------------------------------------------------
--------------------------------------------------
--------------------------------------------------
--------------------------------------------------
--------------------------------------------------
--------------------------------------------------
--------------------------------------------------
--------------------------------------------------
--------------------------------------------------
--------------------------------------------------
--------------------------------------------------
--------------------------------------------------
--------------------------------------------------
--------------------------------------------------
--------------------------------------------------
--------------------------------------------------
--------------------------------------------------
--------------------------------------------------
--------------------------------------------------
--------------------------------------------------
--------------------------------------------------
--------------------------------------------------
--------------------------------------------------
--------------------------------------------------
--------------------------------------------------
--------------------------------------------------
--------------------------------------------------
--------------------------------------------------
--------------------------------------------------
--------------------------------------------------
--------------------------------------------------
--------------------------------------------------
--------------------------------------------------
--------------------------------------------------
--------------------------------------------------
--------------------------------------------------
--------------------------------------------------
```


>Mene maculata
AGTCTACTCATCCGAGCAGAACTTAACCAACCTGGCACTCTCCTGGGAGA
CGACCAAATCTATAATGTAATTGTTACGGCACACGCCTTTGTAATAATTT
TCTTTATAGTAATACCAATTATGATTGGAGGCTTCGGAAACTGACTGATC
CCCCTAATAGTTGGAGCCCCCGACATAGCATTCCCTCGAATAAACAACAT
GAGCTTCTGACTTCTCCCTCCCTCGTTCCTTCTCCTACTGGCCTCCTCAG
GAGTAGAAGCCGGTGCCGGAACGGGGTGAACCGTATACCCGCCTCTTGCC
GGGAATTTAGCCCACGCCGGAGCATCTGTTGACCTCACAATTTTCTCACT
TCACTTGGCCGGGGTCTCTTCAATTCTTGGGGCAATTAATTTTATTACTA
CGATTATCAACATGAAACCACCTACTGTCTCAATGTACCAAATTCCTTTA
TTTGTTTGAGCAGTCCTAATTACAGCCGTCCTTCTCCTCCTTTCCCTCCC
GGTCCTAGCTGCCGGAATTACAATGCTGTTAACAGACCGAAACCTGAACA
CCGCTTTCTTTGACCCTACTGGAGGAGGCGACCCTATTCTCTACCAACAC
CTA---

------------NNNNNNNNNNGAAACCTTCACCCGTCTAACTGTCTCGGCATGCTGTTG
CTGTCTGACGCCCACCAGTGTACCAAGCTGTCAGAGCTTTCCTGGGGCAT
GTGTCTCAGCAACTTCCCTGCTATTTGCAAAACAGAAGACTTCCTCCAAC
TGCCCAAAGATATGGTGGTACAGCTTTTGTCACATGAGGAGCTGGAGACA
GAAGATGAGAGACTGGTTTATGAAGCTGCCCTTAACTGGATCAACTATGA
TCTGGAAAGAAGGCACTGCCACCTTCCAGAGCTTCTGAGAACAGTGCGCC

TCGCCCTGCTGCCGGCCATCTTTCTGATGGAGAACGTCTCAACAGAAGAG
CTGATCAACGCCCAGGCCAAGAGCAAGGAGCTGGTTGACGAAGCTATCCG
CTGTAAGCTGAAAATCCTGCAGAATGACGGCGTTGTTAACAGCCCGTGTG
CTCGGCCAAGAAAAACCAGCCATGCTCTCTTTCTGCTGGGTGGGCAGACT
TTCATGTGTGACAAGTTGTACCTGGTGGATCAAAAGGCCAAAGAGATCAT
CCCCAAGGCTGACATCCCCAGCCCCAGGAAAGAGTTCAGCGCCTGTGCCA
TCGGCTGTAAGGTGTACATCACTGGTGGGA--GAGGCTC-AGAGAACGGT
GTGTCTAAAGATGTATGGGTCTACGACACCGTCCACGAGGAATGGTCCAA
AGCAGCTCCCATGCTCATCGCCAGGTTTGGCCATGGCTCTGCAGAGCTGA
AACACTGCCTCTACGNNNNNNNNNNNNNNNNNNNNNNNNNNNNNNNNNNNNNNNNNNNNNNNNNNNGGATG
ATTACATCGTCGTGTTCAGTCGTTCAACAA
CGAGGCTGATACTTAACGAAGCGGAGCTGATAATGGTGCTGGCCCAGGAG
TTCCAGATGAGGGTGGTCACAGTATCCCTGGAGGAACAGTCTTTCCCCAG
TATCATCCAGGTGATCAGCGGGGCTTCCATGTTAGTCAGCATGCATGGAG
CTCAGCTCATCACCTCACTCTTCCTTCCCAGAGGAGCTGTTGTGGTGGAG
CTGTTCCCCTTTGCTGTGAACCCTGAGCAGTACACCCCATATAAAACACT
GACCTCTCTCCCGGGCATGGACCTTCACTATATCTCCTGGAGGAACACTA
AGGAGGAAAACACTATCACCCACCCAGACAGAGCCTGGGAACAAGGAGGC
ATTGCTCACTTGGAGAAGGAAGAGCGGGAGCGAATACTGGCCAGTAAGGA
CGTACCCAGGCACCTGTGCTGCCGCAACCCAGAGTGGCTCTTCAGGATCT
ACCAGGACACTTTGGTGGACATTCCTTCCTTCCTGGAAGTCCTCAA---G
GAGGGTATGAAG---ACCAAGCCCAGCTTTAAGAA---GTCAAAGCCAGC
GAGCATAGTCCACCCAGGCCGAGTCAGAGAACCTCAGTGTCAGACCTCAG
TACAAACCTCTAATGAGGCTAAGCTCACAGTTTCCTGGCAGATCCCGTGG
AATCTGAAATACCTGAAAGTAAGAGAGGTCAAATACGAAGTGNNNNNNNNNAAAAAAGA
CACCAGCAAGGGGACACTAGAGGATCAAATCATCCAGGCGAACCCTGCGC
TGGAGGCATTTGGCAACGCCAAAACACTAAGAAACGACAACTCATCTCGT
TTTGGAAAATTCATTCGAATTCACTTTGGTACAAGTGGCAAACTGTCATC
TGCTGACATAGAGACATACCTGCTGGAGAAGTCACGTGTCACCTTTCAGC
TCAAGGCTGAGAGAAACTACCACATCTTCTACCAAATCCTGTCCAATCAG
AAGCCAGAGCTGCTGGACATGCTGCTGATCACCAACAACCCATACGACTA
CTCCTACATCTCCCAAGGAGAGGTAACAGTCGCCTCAATCAACGACTCAG
AGGAGCTGATGGCCACTGACAGTGCCTTTGATGTGCTCGGGTTCACTCCA
GAGGAGAAGATGGGTGTCTACAAACTGACCGGTGCCATCATGCACTATGG
CAACATGAAGTTCAAACAGAAGCAGCGTGAGGAGCAGGCTGAACCTGATG
GGACGGAGGCTGCTGATAAATCAGCTTATCTGATGGGACTAAACTCTGCT
GACCTCATCAAAGGCCTGTGCCACCCCAGAGTCAAGGTAGGAAATGAGTA
TGTCACCAAAGGCCAAAGTGTTGATCAAGTCTACTACNNNNNNNNNNNNNNNNNNNNNNNNNNNNNNNNN
NGGAAGCACTACAA
CACCAAGCTGGGATACAAGCGCCATGTGGCGATGCACTCGGCCACGGCGG
GCGATCTCACCTGCAAAGTGTGCATGCAGAGCTACGAGAGCACGCCCGTT
CTCCTGGAGCACCTCAAGAGCCACTCCGGGAAGTCGTCGGGCGGCGCCAA
GGAGAAGAAACACCCGTGCGACCACTGCGACCGCCGTTTCTACACGCGGA
AGGACGTGAGACGGCACATGGTGGTCCACACGGGCCGGAAGGACTTCCTG
TGCCAGTACTGCGCCCAACGCTTTGGCAGGAAGGACCACCTGACGCGCCA
CGTGAAGAAGAGCCACTCGCAGGAGCTGCTGAAGATCAAGACGGAGCCTC
CGGACATGTTGGGTCTTTTGGCTTCGGGGTCGCCGCCTTGTTCTGTGAAG
GAGGAGCTCAGCCCCATGATGTGCAGCATGGGTCCCAACAAAGACCCCAT
GATGGGCAAACCGTTCCCCAGCGGGGCCCCTTTTCCCATGGGCATGTACA
ACCCCCACCAT------CTCCAGGCCATGTCCAACACCGGGGTGGGTCAC
CCC------CACCCGTCCCTGATGCCCGGGTCCCTGTCTGCAGCTATGGG
CATGGGCAGTCANNNNNNN--------------------------------------

NNNNNNNNNNNNNNNNNNNNNNNNNNNNNNNNNNNNNNNNNNNNNNNNTCTTGCAGTGTTGGCATAATTA
AT
GGGCTCTCTGGATGGGCTTCCTCAGTGGATGACGCCCCTGCTGACACAAT
CACTCGCCGGTTTCGCTATGATGTGGCGCTGGTGTCGGCATTAAAGGATC
TGGAGGAGGACATCATGGAGGGGCTGAGAGAGACCGGAATGGAAGACAGC
GCTTGCACCACAGGCTTTAGCGTCATGATCAAGGAATGCTGCGATGGCAT
GGGTGACGTCAGCGAGAAGCACGGTGGAGGACCAGCTGTTCCTGAGAAGG
CTGTACGCTTCTCTTTCACAATTATGTCTATTTCTATCCTGGCAGAGGGT
GAGGAG------------------------------AAGGAGGTTGCCAT
CTTCACAGAGCCAAAGCCTAACTCAGAACTGTCCTGTAAGCCCCTTTGCC
TGATGTTCGTTGATGAGTCAGACCATGAGACGCTCACAGCCGTCCTGGGG
CCCATAGCTGCAGAGCGTGGAGCCATGAAAGAGAGCAGGCTCATCCTGTC
CGTGGGCGGCCTGCCTCGATCCTTCCGCTTTCATTTCAGAGGTACAGGAT
ACGATGAGAAGATGGTGCGAGAGATGGAAGGCCTGGAGTCCTCAGGGTCC
ACATATGTCTGCACTCTGTGTGACTCCAGTCGGGCAGAGGCCTCTCAAAA
CATGGTGCTTCACTCCATCACCCGCAGTCATGAAGAGAACCTCGAACGTT
ACGAAATATGGAGAACCAACCCCTTCTCTGAGTCTGTAGACGAGCTGCGA
GATAGAGTCAAAGGGGTCTCTGCTAAGCCCTTCTTGGAAACCCAGCCCAT
GTTAGATGCATTACACTGTGACATTGGCAATGCCACTGAGTTCTATAAAA
TCTTCCAGGACGAGATTGGGGAGGTGTACCAAAAGGC---CAAC---CCC
AGCCGCGAGGAACGGCGCAGCTGGAGGGCAGTCCTAGATAAACAGCTGAG
GAAGAAGATGAAGCTTAAACCAGTGATGAGGATGAATGGAAACTATGCCC
GCAAGCTAATGACCCTGGAAGCTGTGGAGGTGGTGTGCGAGCTGGTGCCC
TCAGAGGAGAGGAGGGAGGCCCTGAGGGAGCTCATGAGGCTCTACCTCCA
GATGAGGCCCGTGTGGCGCGCCACCTGCCCAGCCAAGGAATGCCCCGACC
AGCTGTGCCGCTACAGCTTTAACTCCCAGCGCTTTGCCGACCTCCTCTCC
TCTACCTTCAAATACAGGTACAATGGAAAGATAACCAATTACCTTCACAA
GACCCTGGCACATGTACCTGAACNNNNNNNNNNNNNNNNNNNNNNNNNNNNNNNNNNNNNNNNNNNNNNN
NNNNNNNNNNNNNNNGCATACACCATCGAGATGGGGACCGTG
GGGCCCCGGTGGAAGGAGAACCCACAGCCCTTCTCCTGCTCCATTGAAGA
CCCCACAAAACAGACAAAGTTCAAGGGCATCAAGACCTACATTTCATACC
GGGTCACGCCGAGCCACACGGGGCGTCCCGTCTACAGGCGTTACAAACAC
TTTGACTGGCTGTACAACCGCTTGCTGCACAAGTTCACTGTGATCTCCGT
GCCTCACCTGCCTGAGAAGCAGGCGACGGGGCGATTTGAGGAAGACTTCA
TCGAGAAGCGAAAGAGACGGCTGATACTGTGGATGAACCACATGACCAGT
CACCCAGTCCTCTCCCAGTACGAAGGCTTTGAACACTTTCTGATGTGCGC
TGACGACAAGCAGTGGAAACTGGGAAAGAGGCGGGCGGAGAAGGATGAGA
TGGTGGGCGCCCATTTCATGCTGACCCTCCAGATCCCCAACGAGCACCAG
GACCTTCAGGACGTGGAGGAGCGGGTGGACTCCTTCAAATCCTTCGCWAA
AAAAATGGATGACAGCGTGATGCAGCTCACGCATGTCGCCTCAGAGCTGG

TGCGTAAACACCTGGGTGGGTTCAGGAAGGAGTTCCAGCGGCTGGGAAAC
GCCTTCCAGTCCATCAGCCAGGCGTTCATGCTTGACCCTCCCCACAGCTC
AAACGCCCTCAACAACGCCATCTCCCATNNNNNNNNNNNNNNNNNNNNNNNNNNNNNNNNNGGTTTCATC
ATTGGAGTCGGCG
TGGTCGGAAACCTCCTGATCTCCATCCTGCTGGTCAAAGACAAGAGCCTG
CACCGAGCGCCCTACTATTTCTTGCTGGACCTGTGCGCCTCTGATATCCT
GCGCTCAGCCATCTGCTTCCCCTTTGTCTTCACCTCGGTCAAGAATGGAT
CTGCCTGGACCTATGGCACGCTGACCTGCAAAGTGATCGCCTTCCTGGGT
GTGCTCTCCTGTTTCCACACGGCGTTCATGCTATTCTGTGTSAGCGTCAC
TCGCTACCTGGCCATYGCACATCACCGTTTCTACACCAAGAGGCTGACCT
TCTGGACCTGTCTGGCTGTCATCTGTATGGTGTGGACGTTGTCGGTGGCT
ATGGCGTTCCCGCCCGTGCTAGACGTAGGGACGTACTCTTTCATCCGGGA
GGAGGAYCAGTGCACATTCCAGCACCGTTCCTTCAGGGCGAATGATTCAC
TGGGCTTCATGCTCCTGCTRGCTCTCATCCTCCTGGCCACACAGCTGGTT
TACCTCAAGCTCATCTTTTTCGTCCACGACCGTCGAAAGATGAAGCCAGT
CCAGTTCGTGCCTGCCGTCAGCCAGAACTGGACCTTCCACGGRCCAGGCG
CCAGTGGGCAGGCGGCAGCCAACTGGCTGGCTGGATTTGGTCGAGGCCCC
ACCCCGCCTACTTTGCTGGGCATCCGGCAGAACAGCAACGCAGCGGGCCG
CAGGCGTCTACTGGTGTTGGATGAATTCAAAACAGAGAAGAGGATTAGTA
GGATGTTCTACATCATGACGTTTTTCTTCCTGGCACTGTGGGGGCCCTAT
CTGGTCGCCTGCTACTGGCGGGTGTTTGCAAGGGGCCCCGTAGTCCCTGG
GGGCTACCTGACGGCAGCTGTGTGGATGAGCTTTGCNNNNNNNNNNNNNNNNNNNNNNNNNNNNNNNNNN
NNNNNNNNNNNNNNNNNNNNNNNNNNNNNNNNNNNNNNNNNNNNNNNNNNNCGGAGC---GCAGC
GTCCCACTCGGCAACAGCTTGCTATCCCCGCAGCAAACCGAGGAGCCCAC
TGTTGCCACCCCCCCGCAGCGATGGTTTGTCACCC---CTGCCAACAATC
GACTGGACTTTGCTGCCTCGGCATACGACGCCGCT---------GATTTC
GCCGGTAACGCAGCCACCTTGCTGTCCTACGCAGCGGCCGGAGTGAAGGC
TC------TTCCCCTGCCGGCGGCAGGCTGCTCCAACCGGCCTCTTGGCT
ATTACGCAGACCCGTCGG---GCTGG---GGAGGACGCACGCCACCGCAG
TACTGTGGCGTAAATAGCAAATCCAGCTCGGTCTTTTCCTGCTGGCCCGC
CAACTCTATCGGTGGCAGAGCAGGCA---CC---AACTACCTGA------
---CCGAGGA---GGTA---GACTC---CATCCCCACGGAGAGATCACCC
---AT---CGGCGGCTCGGAGGAG---ACCAAACCCAAAGACATGAC---
ATCTGA---GTCGAGCTGGATAGAG---ACGCCGTCCTCCATTAAGTCCA
TCGACTCGAGTGATTCTGGGATCTTTG---AACAGGCCAAGAGGAGGAGA
ATCTCACCTTCTGCCACGCCG---------GTTTCAGAGACAGTGTCCCC
ATTANNNNNNNNNNNNNNNNNNNNNNNNNNNNNNNNNNNNNNNNNNNNNNNNNNNNNNNNNNNNNNNNGTTC
GCGGATGGGATGGGCGCCTTCAAAATAAACCACAGCTCCCAC
GATATTGGCTCCGG---GCAGACGGCGTTTTCCTCTCAGGCG---CCCGG
CTAC---GCGGCAGCCGCCCTGGGA---CACCATCA------CCACCCGA
CCCACGTTGGCTCT---TACTCCACGGCGGCTTTCAACTCCACCAGGGAC
TTTCTCTTCAGAAATCGGGGTTTCGGGGACGCCACCGG------------
GGCGCAGCACAGTTTGTTCGCCTC---------TGGAAGTTT---C----
--GCAGGGCCACATGGACACTCAGATGCAGCGGGGCACCTGCTCTTCCCA
GGGCTCCACGAG---CAAGCGGCGAGCCATGCCTCTTCCAACGTGGTCAA
CAGCCAGATGCGGCTGGGCTTCTCGGGGGACATGTACGGACGCGCCGACC
AGTATGGCCACGTTACAAGCCCACGGT---CCGACCACTATGCTTCCACC
CAGCTGCACGGCTATGGCCCCATGAACATGAATATGGCCGCG---CACCA
CGGAGCAGGGGCCTTCTTTCGATACATGAGGCAGCCGATCAAACAAGAGC
TCATCTGCAAGTGGATCGAGCCGGAGCAGCTGACAAATCCCAAAAAGTCG
TGCAACAAAACTTTTAGCACGATGCACGAGCTTGTGACCCATCTGACGGT
GGAGCATGTGGGGGGACCGGAGCAGACCAACCACGTCTGCTTCTGGGAGG
ANNNNNNNNNNNNNNNNNNNNNNNNNNNNNNNNNNNNNNNNNNNNNNNNNNNNNNNNNNNNNNNNNNNNN

NNNNNNNNNNNNNNNNNNNNNNNNNNNNNNNNNNNNNNNN
>Menticirrhus littoralis

------------TTCCTAGAGCGAAACCTTCACCCTTCTAACTGCCTTGG
CATGTTGTTGCTGTCTGACGCCCACCAGTGCACCAAGCTGTCAGAGCTTT
CCTGGGGCATGTGCCTCAGCAACTTCCCCGCTATTTGCAAGACAGAGGAC
TTCCTCCAACTGCCCAAAGATATGGTGGTGCAGCTTTTGTCGCATGAGGA
GCTAGAGACAGAAGATGAGAGACTGGTGTATGAAGCTGCCCTCAACTGGA
TCAACTATGACTTGGAAAAGAGACACTGTCACCTGCCAGAGCTCCTGAGA
ACGGTCCGTCTGGCCTTGCTGCCTGCCATCTTTCTCATGGAGAACGTATC
TACGGAAGAGCTGATCAATGCCCAGGCCAAGAGCAAGGAGCTGGTGGACG
AAGCTATCCGCTGTAAGCTGAAGATCCTGCAGAATGATGGTGTTGTAAAC
AGCCCGTGTGCTCGACCAAGAAAAACCAGCCACGCGCTCTTTCTTCTGGG
AGGGCAGACTTTCATGTGCGACAAGTTGTACCTGGTGGACCAGAAAGCCA
AAGAGATCATCCCCAAAGCTGACATTCCCAGCCCCAGGAAGGAGTTCAGC
GCCTGCGCCATCGGCTGTAAAGTGTACATCACTGGTGGAA--GAGGCTC-
AGAGAACGGTGTGTCCAAAGACGTGTGGGTCTATGACACTGTCCACGAGG
AATGGTCGAAGGCGGCACCCATGCTCATCGCCAGGTTCGGCCATGGCTCT
GCAGAGCTGAAACACTGCCTCTACGTGGTAGGAGGTCACACCGCAGCAAC
TGGCTGCCTCCCGGCCTCCCCGTCTGGATGAATACATTGTTGTGTTTAGT
CGTTCAACAACAAGGTTGGTACTGAATGAAGCAGAGTTAATCATGGCACT
GGCCCAGGAGTTCCAGATGAGAGTGGTCACAGTATCACTGGAGGAACAGT
CTTTCCCCAGTATTGTCCAGGTGATCAGCAGTGCTACCATGTTAGTCAGT
ATGCATGGAGCTCAGTTGATCACCTCACTGTTCCTTCCCAGAGGAGCTGT
CGTGGTGGAGCTGTTCCCCTTTGCTGTGAACCCAGAACAGTACACCCCAT
ATAAAACCCTTGCCTCCCTTCCAGGCATGGACCTCCATTATATCTCCTGG
AGGAACACGAAAGAGGAGAACACCATCACCCACCCAGACAGACCCTGGGA
ACAAGGGGGCATTGCTCACTTGGAGAAGGAGGAGCAAGAGCGAATATTGT
CGAGCAAAGATGTCCCAAGGCACCTGTGCTGCCGCAACCCTGAGTGGCTC
TTTCGGATCTACCAGGACACCTTGGTGGACATCCCTTCTTTTCTGGAAGT
CCTGAA---AGAGGGCATGAAG---ACAAAGCCCAGCTTTAAGAA---GT
CAAAGCCGGCCAGTACAGTCCATCCAGGCCGGGTCAGAGAACCCCATTGT
CAGACCTCAGTACAAACCACTAATGAAGCTAAACTCACAGTCTCCTGGCA
GATCCCATGGAATCTGAAATACCTGAAGGTCAGAGAGGTAAAATACGAGG
TGTGGATCCAGAAGAAAGACACCAGCAAGGGAACACTGGAGGATCAAATC
ATCCAGGCCAACCCGGCGCTGGAGGCGTTTGGCAACGCCAAAACACTGAG
AAATGACAACTCGTCTCGTTTTGGAAAATTCATCCGAATTCACTTCGGTA
CGAGTGGAAAGCTGTCATCTGCTGACATTGAGACGTACCTGCTGGAGAAG

TCACGTGTCACCTTTCAGCTCAAAGCTGAGAGGAACTACCACATCTTCTA
CCAGATCCTGTCCAATCAGAAGCCAGAACTGCTGGACATGCTGCTGATCA
CCAACAACCCGTACGACTACTCCTACATCTCCCAAGGAGAGGTGACGGTC
GCCTCCATCAACGACTCAGAGGAGCTGATGGCCACCGATAGCGCCTTCGA
TGTGCTTGGCTTCACTCCAGATGAGAAGCTGGGCGTCTATAAATTGACGG
GCGCCATCATGCACTACGGCAACATGAAGTTCAAACAGAAGCAGCGTGAG
GAGCAGGCTGAGCCAGACAGCACGGAGGCTGCAGATAAATCTGCTTATCT
AATGGGGCTGAACTCTGCTGACCTCATCAAAGGGCTGTGCCATCCCAGAG
TAAAGGTAGGAAATGAATATGTGACCAAAGGCCAAAGTGTGGACCAGGTC
TACTATCCCAACAAGGAGGCCTTCAAGTGTGAAGAGTGTGGGAAGCATTA
CAACACCAAGCTGGGATATAAGCGCCATGTAGCCATGCACTCTGCAACGG
CAGGGGATCTCACCTGCAAAGTGTGCATGCAGACCTACGAGAGCACACCT
GTGCTCTTGGAGCACCTCAAGAGCCACTCTGGGAAGTCTTCAGGTGGCAC
CAAGGAGAAAAAACACCCATGTGACCACTGTGACCGTCGCTTCTACACAC
GAAAGGATGTGAGACGGCACATGGTGGTCCACACGGGCCGAAAAGACTTC
CTGTGCCAGTACTGTGCCCAACGCTTCGGCAGGAAGGACCATCTGACACG
CCATGTGAAGAAGAGCCACTCACAGGAGCTGCTAAAGATCAAGACAGAGC
CTCCTGATATGTTAGGTCTTTTAGCTTCCGGGTCACCACCCTGCTCTGTG
AAGGAGGAGCTCAGCCCCATGATGTGCGGTATGGGTCCCAACAAAGACCC
CATGATGGGAAAACCATTTCCCAGTGGGGCCCCTTTTCCAATGGGCATGT
ACAACCCCCACCAT------CTCCAGGCCATGTCCAATTCTGGGGTGAGT
CACCCA------CACCCTTCCCTGATGCCCAGTTCCTTATCTGCAGCTAT
GGGCATAGGCTGTCACATGGAATATCTCATCTACGCATCTTTTTCATTCA
TGGGATGTTTACAAATTAGTGATGGATCAAATATTGTGAATCTGCTGGCT
AGTAACTCTCCGAGTGTTTCCTATGCTCTGACCCAGCAAAAATACTTCAG
TAACTACAGTCCCGTGATTGGGTTTTACATTTATGAGCCTATTGAGTACT
GGAACTCAACGGTGCAGGAGCACCTGAAGACTCTGAGTCATGGCTTCAAC
AAGATCTCCTGGATGGACAACTTTTTCCACTACTTACGGGTAGTGAATGT
GAGTGCGTCAACCAAGGGTGACTTCATCACCATCCTCAAGGGCTCCTTCC
TGCGCAGCCCGGAGTACCAGCACTTCACTGAGGACATCATATTCTCCAAG
A---ACCGTGAGACTG------ATGAGTATGACATTATCGCCTCACGGAT
GTACTTGGTGGCACGGACGACAGAGAAAAAGCGAGAAGAGGTGGTGGAGC
TTCTTGAAAAGCTTCGTCCATTGATGCTAATCAACAGCATCAAATTCATT
GCCTTCAATCCTACGTTTGTGTTCATGGACCGCTACAGCTCTTCTGTCAT
CTCGCCCATCCTGACCTCAGGATTCAGCGTACTCACAATCCTCATCCTCA
CTTTCTTCTTGGTCATCAACCCCTTGGGTAACTTCTGGCTCATTCTCACT
GTAACGTCCGTGGAGCTGGGCGTCTTGGGTTTGATGGGCTTTCACCAGTT
TGAATGGCAGCCAGCTCTCAAGAATGTGTCTACATCTTGCAATGTTGGCA
TTATTAATGGGCTCGCTGGATGGGTTTCCTCAGTGGATGATTCCCCAGCT
GACACCATCACTCGGCGGTTTCGCTATGATGTGGCACTTGTGTCGGCATT
AAAGGACCTGGAGGAGGACATCATGGAGGGGCTGAAAGAGAGTGGGATGG
AAGACAGTGCTTGCACCTCAGGCTTCAGTGTCATGATCAAAGAATCTTGT
GATGGCATGGGCGATGTCAGTGAGAAGCATGGTGGAGGACCAGTTGTTCC
CGAGAAGGCTGTACGTTTCTCTTTCACTATTATGTCTGTCTCTGTCCTAG
CAGACGAGAAGGAG------------------------------GAAGAG
GTTACCATCTTCACGGAGCCAAAGCCAAACTCAGAACTCTCCTGTAAGCC
CCTTTGCCTGACATTTGTAGATGAGTCAGACCATGAGACACTCACAGCCA
TCCTGTGGCCTATTGTTGCAGAGCGTAACGCAATGAAAGAAAGTAGGCTT
ATCCTACCCATAGGTGGACTACCTCGCTCCTTCCGCTTTCACTTCAGAGG
CACGGGTTATGATGAGAAGATGGTGCGTGAGATGGAGGGCCTCGAAGCCT
CAGGGTCCACCTACATCTGCACTCTTTGTGACTCCAGTCGGGCAGAGGCC
TCTCAAAACATGGTGTTACACTCTGTCACCCGCAGTCATGAAGAGAACTT
AGACCGTTACGAAATATGGCGAACCAACCCCTTTTCTGAGTCTGTAGAGG

AGCTGCGAGAAAGAGTCAAAGGGGTTTCTGCCAAGCCTTTCATGGAAACA
CACCCCACACTGGATGCATTACACTGTGACATAGGCAATGCCACTGAGTT
CTACAAAATCTTCCAGGATGAGATCGGGGAGGTGTATGAAAAGGT---CA
AC---CCCAGTCGGGAGGAGCGGCGCAGCTGGAGGGCAGCCCTAGATAAA
CAGCTGAGAAACAAGATGAAGCTTAAACCGGTAATGAGGATGAATGGGAA
CTATGCCCGCAAGCTAATGACCATGGAGGCTGTGGAGGTAGTGTGTGAGC
TGGTGCCCTCAGAGGAGAGGAGGGAGGCCCTGAGGGAGCTTATGAGGCTC
TACTTGCAGATGAAGCCTGTGTGGCGTGCCACCTGCCCAGCCAAGGAGTG
CCCTGACCAGCTGTGCCGCTACAGTTTTAACTCCCAGCGCTTTGCCGACC
TCCTCTCCTCTGCCTTTAAATATAGGTACAATGGAAAGATAACCAATTAC
CTGCACAAGACCCTGGCCCATGTGCCAGAAATCATAGAGAGAGATGGATC
TATAGGAGCCTGGGCCAGCGAGGGGAATGAGTCTGCAAACAAATCATACA
CCATCGAGATGGGTCCCATGGGGCCCAGGTGGAAGGAGAGCCCACAACCT
TTCTCCTGCTCCATCGAAGACCCAACCAAACAGACAAAGTTCAAGGGCAT
CAAGACGTACATATCGTACCGCGTCACACCGAGCCACACAGAGCATCCTG
TCTACAGGCGCTACAAACACTTTGACTGGCTGTACAACCGCTTACTGCAC
AAGTTCACTGTGATCTCTGTGCCTCACCTGCCTGAGAAGCAGGCCACAGG
GCGATTTGAGGAAGACTTCATCGAGAAGCGCAAAAGACGACTTATACTGT
GGATGAACCACATGACCAGTCACCCTGTCCTCTCCCAGTATGAAGGCTTC
GAGCACTTTCTGATGTGTGCTGATGACAAACAGTGGAAACTGGGCAAGAG
ACGGGCGGAGAAGGACGAGATGGTGGGCGCCCATTTCATGCTGACCCTGC
AGATCCCTAATGAACACCAGGACCTTCAGGATGTAGAGGAGCGGATCGAC
TCCTTCAAGTCCTTTGCTAAGAAGATGGATGACAGCGTGATGCAGCTCAC
ACATGTTGCCTCAGAGCTGGTGCGTAAGCACCTGGGTGGTTTCAGGAAGG
AGTTCCAGCGACTGGGGAATGCCTTCCAGTCTGTAAGCCAGGCATTCATG
CTGGACCCACCCCACAGCTCAGAGACCTTCAACAACGCCATCTCCCAT--
-------ACGTTCCTCAAACTGACCTCTCTGGGTTTCATCATTGGAGTCG
GTGTGGTTGGAAACCTCCTGATCTCCATCCTGCTGGTCAAAGACAAGAGC
CTGCACCGAGCGCCCTACTATTTCCTGCTGGACCTGTGCGCCTCCGATAT
CCTGCGCTCAGCCATCTGCTTCCCCTTTGTCTTCACCTCCGTCAAGAATG
GATCTGCCTGGACCTATGGCACGCTGACCTGCAAAGTGATCGCCTTTCTC
GGTGTGCTCTCCTGTTTCCACACAGCGTTTATGCTGTTCTGTGTCAGTGT
CACACGCTACCTGGCCATCGCGCATCATCGTTTCTACACCAAGAGGCTGA
CCTTCTGGACCTGTCTAGCTGTCATCTGCATGGTGTGGACGTTGTCAGTG
GCTATGGCATTCCCGCCGGTGCTGGATGTAGGGACGTACTCTTTTATCCG
GGAGGAGGACCAGTGCACATTCCAGCACCGTTCCTTCAGGGCGAATGATT
CGCTGGGCTTCATGCTCCTGCTGGCGCTCATCCTCCTGGCCACACAGCTG
GTTTACCTCAAGCTCATCTTCTTTGTCCACGACCGTCGAAAGATGAAGCC
TGTCCAGTTCGTGCCTGCTGTCAGCCAGAACTGGACCTTTCACGGGCCAG
GCGCCAGCGGGCAGGCGGCGGCCAACTGGCTGGCTGGATTTGGTCGAGGC
CCCACCCCGCCTACTTTGCTGGGCATCCGGCAGAACAGCAACGCAGCAGG
CCGCAGGCGTCTACTGGTGTTGGATGAATTCAAAACAGAGAAGAGGATTA
GTAGGATGTTCTACATCATGACGTTTTTCTTCCTGGCACTGTGGGGGCCA
TATCTGGTCGCCTGCTACTGGCGGGTGTTTGCAAGGGGCCCCGTGGTCCC
CGGGGGCTACCTGACGGCAGCCGTGTGGATGAGCTTTGCCCAGGCTGGGG
TCAATCCTTTCATCTGCATC---------------GCCAAATCTCGCTTT
CACCCTGGCATGGGGACTGGTCCTGGCACGGAGC---GCAGCGTCCCACT
CGGCAACAGCTTGCTATCCCCGCAGCAAACCGAGGAGCCCACTGTTGCCA
CCCCCCCGCAGCGATGGTTTGTCACCC---CTGCCAACAACCGACTGGAC
TTTGCTGCCTCGGCATACGACGCCGCT---------GATTTCGCCGGTAA
CGCGGCCACCTTGCTGTCCTACGCAGCGGCCGGAGTGAAGGCTC------
TTCCCCTGCCGACTGCAGGCTGCTCCAACCGGCCTCTTGGCTATTACGCA
GACCCGTCAG---GCTGG---GGAGGACGCACGCCGCCGCAGTACTGTGG

TGTAAATAGCAAATCCAGCTCGGTCTTTTCCTGCTGGCCCACTAACTCTA
TCGGTGGCAGAGCTAGTA---CC---AACTACCTGG---------CCGAG
GA---GGGA---GACTC---CATCCCCACAGAGAGGTCACCG---AT---
CGGCGGCACGGAGGAG---ACCAAACCCAAAGACATGAC---ATCAGA--
-GTCGAACTGGATAGAG---ACGCCGTCCTCCATTAAGTCCATTGATTCA
AGCGATTCTGGTATCTTTG---AACAGGCCAAGAGGAGAAGGATCTCACC
CTCTGCCACGCCG---------GTTTCAGAGACAGTGTCCCCGTTAAAAT
CTGAGCATCACTCAACAGGCGAAGTCACAGAGAGAGAAGTGGCGTTGGGG
ATAAATCCGTTCGCCGATGGGATGGGCGCCTTCAAAATAAACCACAGCTC
CCATGATATTGGCTCCGG---ACAAACGGCGTTTTCCTCCCAGGCG---C
CCGGCTAC---GCGGCAGCCGCCCTGGGG---CACCATCA------CCAC
CCGACCCACGTTGGCTCT---TACTCCACGGCGGCTTTCAACTCCACCAG
GGACTTTCTCTTCAGAAATCGGGGATTCGGGGACGCCACCGG--------
----CGCGCAGCACAGTTTGTTCGCCTC---------CGGAAGTTT---C
------GCAGGGCCACATGGACACTCAGATGCAGCGGGGCACCTGCTCTT
CCCGGGGCTCCACGAG---CAAGCGGCGAGCCATGCGTCTTCCAATGTGG
TCAACAGCCAGATGCGGCTGGGCTTCTCGGGGGACATGTACGGACGGGCC
GACCAGTACGGCCACGTTACGAGCCCGCGGT---CCGACCACTATGCTTC
GACCCAGCTGCACGGCTACGGCCCCATGAACATGAATATGGCCGCA---C
ATCATGGAGCAGGGGCCTTCTTTCGATACATGAGGCAGCCGATCAAACAA
GAGCTCATCTGCAAGTGGGTCGAGCCGGAGCAGCTGACGAATCCCAAAAA
GTCGTGCAACAAAACTTTTAGCACGATGCACGAGCTCGTGACCCATCTGA
CGGTGGAGCATGTGGGGGGACCGGAGCAGACGAACCACATCTGCTTCTGG
GAGGAATGCGCCAGAGAAGGAAAGCCATTCAAAGCCAAATACAAACTTGT
AAATCATATCAGAGTACACACCGGAGAAAAGCCGTTTCCATGTCCGTTCC
CGGGCTGTGGCAAA
>Merluccius merluccius
AGCCTACTCATCCGAGCAGAACTTAGTCAACCAGGCGCACTCCTGGGCGA
CGATCAAATTTATAACGTGATCGTCACGGCACACGCCTTCGTAATAATCT
TCTTTATAGTAATACCACTAATAATTGGAGGCTTCGGAAACTGACTCGTC
CCCCTAATGATCGGAGCCCCCGACATGGCCTTCCCCCGAATAAACAACAT
GAGCTTCTGACTCCTCCCTCCATCGTTCCTGCTCCTCCTAGCATCTTCCG
GGGTGGAAGCCGGGGCCGGGACAGGCTGGACGGTCTATCCCCCTCTTGCA
AGTAATCTTGCCCACGCTGGCGCTTCCGTTGACCTCACCATTTTCTCCCT
CCACCTAGCAGGCGTTTCATCAATTCTGGGAGCAATTAATTTTATTACTA
CCATCATCAACATAAAACCCCCTGCCATTTCACAATACCAAACACCCCTC
TTTGTTTGATCCGTCCTTATTACAGCCGTACTCCTCCTGCTGTCCCTGCC
CGTCCTAGCCGCCGGCATCACAATACTACTAACCGACCGAAACCTCAACA
CCTCTTTCTTTGACCCCGCCGGAGGAGGAGACCCAATCCTGTACCAACAC
CTGTTC--

------------------------------------GGCTTCCACGCCTT
CGAATGGCAGCCCGCCCTGAAGAACGTCTCGGCCTCCTGCCACGTGGGCA
TCATCGACGGGCTCTCGGGCTGGGCGGCCTCCGTGGACGACGTCCAGGCG
GACACCATCACCCGCCGCTTCCGCTACGACGTGGCGCTGGCGTCGGCCCT
GATGGACCTGGAAGAGGAGATCATGGACGGGCTGAGGGAGCGTGGGCTGG
AAGACAGCGATTGCACTTCAGGCTTCTCTGTCATGATCAAAGAGTCCTGT
GATGGCATGGGAGATGTGAGCGAGAAGCATGGCGGCGGGCCACCGGTCCC
GGAGAAAGCTGTGCGTTTCTCTTTCACCATTATGTCCATCTCTTTGCACA
TGGAAGATGGAAAGAAGGAGGAGGAGGAGGCGGCGGCGCGGGAGGAGGCC
GTGATGATCTTCCAGGAGCCAAAGCCCAACTCTGAACTGTCCTGTAAACC
CCTCTGCCTTATGTTTGTAGATGAGTCAGACCACGAGACGCTCACTGCTG
TCCTGGGGCCTGTGGCGGCCGAGCGCAGCGCGATGAAGCGCAGCCGACTC
ATCCTCTCCATCGGTGGGCTCCCGCGGTTCTTCAGCTTCCACTTCAGAGG
CTCGGGCTATGATGAGAAGATGGTGCGGGACGTGGAGGGCTTGGAGGCCT
CCGGCTCCACTTACGTTTGCACGCTTTGCGATTCCACCCGCGCCGAGGCC
TCCCACAACATGGTGCTCCACTCGATCACCCGCAGCCACGAGGAGAACCT
GGAGCGCTACGAGATCTGGAGGACCAACCCCTTCTCAGAGTCCGTGGACG
CGCTGAGAGACAGGGTGAAGGGGGTCTCCGCCAAACCTTTCCTGGAGACC
CAGCCGACGCTGGACGCCCTACACTGCGACATCGGCAACGCCACAGAGTT
CTACAAGATCTTCCAGGATGAAATCGGCGAGGTCTTCCAGAAGAC---CA
AC---CCCAGCCGAGCGGAGCGGCGAGGCTGGAGGGCGGCTCTGGACAAG
CAGCTGAGGAAGACGATGAAGCTCAAACCGGTGATGAGGATGAACGGCAA
CTATGCCCGGAAGCTGATGAGCCTGGAGGCCGTGGAGGCGGTGTGCGAGT
TGGTGCCATCTGAGGAGAGACGGACCGCCCTGAAGCAACTCATGGAGATC
TACCTGGAGATGAAGCCGGTGTGGCGCGCCACAAGTCCAGCGAAGGAGTG
CCCCGACCAGCTGTGTCGCTACACCTTCAACTCCCAGCGCTTCGCCGATC
TCCTCTCCTCCACGTTCAAATACAGGTACAACGGAAAGATCACCAATTAC
CTGCACAAAACCCTGGCGCACGTGCCGGAGATCATAGAGAGAGACGGCTC
TATCGGCGCGTGGGCGAGCGAGGGGAACGAGTCTGCAAACAAA-------

>Mirorictus taningi
NNNNNNNNNNNNNNNNNNNNNNNNNNNNNNNNNNNNNNNNNNNNNNNNNNNNNNNNNNNNNNNNNNNNNN

```
NNNNNNNNNNNNNNNNNNNNNNNNNNNNNNNNNNNNNNNNNNNNNNNNNNNNNNNNNNNNNNNNNNNNNN
NNNNNNNNNNNCCCTAATACTGGGAGCCCCCGACATAGCATTCCCCCGAATAAATAACATG
AGCTTCTGGCTACTCCCTCCCTCCCTCCTCCTCCTCTTATCGTCTTCCGG
GGTTGAAGCCGGGGTCGGAACAGGATGAACCGTTTATCCCCCACTAGCCG
GCAACCTGGCCCACGTAGGTGCCTCTGTAGACCTCGCTATTTTCTCCCTC
CACCTGGCGGGCGTTTCCTCAATCCTGGGGTCAATTAATTTTATTACCAC
AATTATTAACATGAAGCCCCCCGCCATCTCCCAGTACCAAACACCATTAT
TTATCTGATCGGTCCTCGTTACAACAGTCCTCCTCCTCCTCTCACTCCCG
GTCCTGGCCGCAGGTATTACCATGCTCCTCACAGACCGAAATCTTAACAC
AACATTCTTTGACCCGGCCGGAGGAGGAGACCCCATCCTTTACCAACACC
TCTTCTGATTTTTTGGCCACCCAGAGGTCTACATCTTGATCTTGCCCGGG
TTTGGTATCATCTCACACGTTGTTGCCTACTACGCCGGCAAAAAAGAGCC
ATTTGGCTACATGGGCATGGTCTGAGCCATAATAGCCATTG---------
--------------------------------------------------
--------------------------------------------------
--------------------------------------------------
--------------------------------------------------
--------------------------------------------------
--------------------------------------------------
--------------------------------------------------
--------------------------------------------------
--------------------------------------------------
--------------------------------------------------
--------------------------------------------------
--------------------------------------------------
--------------------------------------------------
--------------------------------------------------
--------------------------------------------------
--------------------------------------------------
--------------------------------------------------
-------------------------GGACGAGTACATCGTGGTTTTCAGCC
GCTCCGTCAACCGGCTCATCCTCAACGAGGCCGAGTTGATCCTGGCTCTG
GCGCAAGAGTTCCAGATGAGGGCCGTCACGGTGTCCCTGGAGGAGCAGTC
GTTCGCCAGCATCGTTCAGGCGATCAGCGGGGCGTCCATGCTGGTCAGCG
TCCATGGAGCCCAGCTGATCAGCTCCCTGTTCCTGCCGCGTGGCGCTGCC
GTGGTGGAGCTCTTCCCCTACGCCGTCAACCCGGAGCAGTACACTCCGTA
CAAAACCCTGGCCCTGTTACCAGGCATGGACCTCCAGTACGTGGCTTGGA
GGAACACCATGGAGCAGAACTCGGTGGCTTACCCCGAGCGCGCCTGGGAC
CAGGGTGGCATCGCCCACCTGGAGAAGGAGGAGCAGGAGCGCATCCTGGC
GAGCGACGACGTGCCGCGGCACCTGTGCTGCCGCAACCCAGAGTGGCTCT
TCCGGATTTACCAGGACACCCAGGTGGACGTCCCCTCCTTGCTGGAGGTC
CTCCG---AGAAAATCTGAAG---GCCAAGCCTAACCTGAGAAA---GGC
CAAGGCAGCTAGTACAGTACACCCCGGTAGAGTCAGGGAGCCCAAGTGCC
AGACCTCGGTCCAGGCCACCAACGAGGCCAAGCTGACAGTGTCGTGGCAG
ATCCCCTGGAACCTCAAATACCTGAAGGTCAGGGAGGTCAAA--------
----------AAGAAGGATGCCAGCAAAGGGACCTTGGAGGATCAAATCA
TTCAAGCTAACCCAGCACTGGAGGCTTTTGGCAATGCCAAAACAGTGAGA
AATGACAACTCCTCACGCTTTGGGAAGTTCATTCGTATTCATTTTGGAAC
GAGCGGCAAACTCTCCTCTGCTGACATAGAAACTTACTTGCTTGAGAAAT
CCCGTGTGACCTTTCAGCTCAAATCGGAGAGGAACTACCACATCTTCTTC
CAGATATTGTCCAATGAAAAGCCAGAGCTGCTGGACATGCTGTTGATTAC
CAACAACCCTTATGATTATTGCTTCATCTCCCAAGGAGAAGTAACAGTTA
AATCTATCAATGACAGTGAGGAGTTGCTTGCCACTGACAGTGCCTTTGAT
```

GTGCTTGGCTTCACTCCAGAGGAGAAGATGGGGATCTATAAGTTGACAGG
TGCCATTATGCACTATGGCAACATGAAGTTCAAGCAGAAGCAGCGTGAGG
AGCAGGCAGAGCCTGATGGCACTGAGGCAGCTGACAAGTCAGCCTACCTG
ATGGGGCTTAACTCTGCTGATCTTGTGAAGGGACTCTGCCATCCCAGGGT
CAAAGTCGGCAATGAGTATGTAACAAAGGGACAAGGTGTAGATCAAGTCT
ATTACCCCAACAAGGAGGCCTTCAAGTGCGAGGAGTGCGGAAAGCACTAC
AACACCAAGCTGGGCTACAAGCGGCACGTGGCCATGCACTCGGCCACGGC
GGGCGACCTCACCTGCAAGGTGTGCCTGCAGAGCTACGAGAGCACGCCGG
CGCTGCTGGAGCACCTCAAGAGCCACTCGGGCAAGTCGTCGGGCGGCGCC
AAGGAGAAGAAGCACCCGTGCGACCACTGCGACCGCCGCTTCTACACGCG
CAAGGACGTGCGCCGCCACATGGTGGTGCACACGGGCCGCAAGGACTTCC
TGTGCCAGTACTGCGCGCAGCGCTTCGGCCGCAAGGACCACCTGACGCGC
CACGTCAAGAAGAGCCACTCGCAGGAGCTGCTGAAGATCAAGACGGAACC
TCCGGACATGCTGGGCCTGCTGGGCTCGGGCTCGCCGCCCTGCGCCATCA
AGGAGGAGCTCAGCCCCATGATGTGCAGCATGGGCCCGGCAAGGACCCC
ATGATGGCCAAGCCCTTCCCCAGCGGCACCCCCTTCCCCATGGGCATGTA
CAACCCCCACCAC------CTCCAGGCCATGTCCAACCCCGGAGTGGGCC
AC---------CACCACTCCCTGGTGCCCGGCTCCCTGTCGGCCGCCATG
GGGATGGGCTGCCACATGGAG-----------------------------

----------------------TTCAGTGTGACGATCAAGGAGTCCTGCG
ACGGCATGGGGGACATCAGGGAGAAACACGGCGGTGGGCCGCCCGTCCCA
GAGAAGGCCGTGCGCTTCTCCATCACCGTCATGTCTGTCTCCGTCCAGGC
CGAAGGAGAGGAG------------------------------GAGGCGG
TCACCATCTTCCGGGAGTCCAAGCCCAACTCTGAACTGACCTGTAAGCCA
CTGTGCTTGATGCTGGTGGACGAGTCGGACCACGAGACCCTCACGGCAGT
CCTGGGGCCTCTGGTGGCCGAGAGGAACGCCATGAAGCACAGCCGGCTCA
TCCTGTCCCTGGGCGGGCTCCCTCGCTCCATCCGCTTCCACTTCAGAGGA
ACGGGTTATGACGAGAAGATGGTGCGGGACATGGAGGGCCTGGAAGGCTC
CGGCTCCACCTATATCTGCACCTTGTGCGACTCCACCAGGGCAGAGGCCT
CCCACAACATGGTGCTCCACTCCATCACCCGCAGCCACGACGAAAACCTG
GAGCGCTACGAGCTGTGGAGGACCAACCCCTTCTCGGAGTCCGCAGAGGA
GCTGCGAGACCGGGTCAAAGGTGTCTCGGCTAAGCCCTTCATGGAGACAC
AGCCCACACTGGACGCCCTGCACTGCGACATTGGCAATGCTACGGAGTTC
TACAAGATCTTCCAGGATGAGATCGGGGAGGTGTACCGCAGCCC---CAA
C---CCCAGGCGAGAGGAGCGACGGGGCTGGAGGGCCGCTCTGGACAAGC

AGCTGAGGAAGGAGATGAAGTTGAGGCCGGTGATGCGAATGAACGGGAAC
TACGCGCGGCGGCTGATGACCCGGCAGGCGGTGGAG--------------

--------------------------------GAGAAGTGGCTTTGGGGA
TAAATCCGTTCGCCGACGGGATGGGCGCTTTCAAAATCAACCACAGCTCC
CACGATATTGGCTCTGG---TCAAACGGCGTTTGCCTCGCAGGCG---CC
CGGCTAC---GCAGCCGCTGCCCTGGGA---CACCATCA------CCACC
CAACCCATGTCAGCTCC---TACTCCACCGCGGCGTTCAACTCCACCCGG
GACTTTCTCTTTCGGAATCGGGGATTCGGAGACGCCACTAG---------
---CGCGCAGCACAGTCTCTTCGCCTCAGC---TGCGGGAAGTTT---C-
-----GCTGGGCCACATGGACACACCGATGCCACGGGACACCTGCTCTTC
TCGGGACTGCACGAG---CAAGCGGCGACCCACGCGTCTTCGAACGTGGT
GAACAGTCAGATGCGCCTGGGCTTTTCGGGGGACATGTACGGCAGAGCCG
AGCAGTATGGTCACGTAACGAGCCCCCGGT---CCGAGCACTACGCTTCG
ACTCAGTTGCACGGCTACGGCCCCATGAACATGAATATGGCTGCC---CA
CCACGGGGCAGGGGCCTTCTTCCGTTACATGAGGCAGCCGATCAAACAAG
AGCTCATCTGCAAGTGGGTCGAACCAGAGCAGCTGTCGAATCCGAAAAAG
TCCTGCAACAAAACTTTCAGCACGATGCATGAGCTCGTGACCCACCTCAC
AGTGGAACATGTCGGGGGACCGGAACAGTCGAATCACATTTGCTTTTGGG
AAGAGTGTCCGCGAGAAGGGAAACCATTTAAAGCCA--------------

>Mola mola
AGCCTACTCATTCGAGCGGAACTAAGTCAACCTGGTGCTCTTCTTGGAGA
CGACCAAATTTACAATGTCATCGTCACAGCACATGCATTTGTAATAATTT
TCTTTATAGTAATACCAATTATGATTGGGGGCTTCGGAAACTGACTGGTC
CCTCTTATGATCGGGGCCCCCGATATGGCCTTCCCCCGAATGAACAATAT
AAGCTTTTGACTCTTGCCCCCATCTTTTCTTCTACTTCTTGCCTCCTCAG
GCGTCGAAGCAGGTGCCGGAACAGGATGAACTGTATACCCCCCTTTAGCC
GGAAACTTAGCCCATGCAGGCGCCTCTGTTGATTTAACAATCTTTTCCCT
TCACCTAGCCGGCATCTCCTCAATTCTAGGGGCCATTAATTTTATCACAA
CGATCATTAACATGAAACCGCCTGCGATTTCACAATACCAGACCCCCCTG
TTTGTATGGGCGGTCCTCATTACAGCAGTACTTCTTCTCCTCTCGCTTCC
AGTCCTTGCAGCTGGGATTACGATGCTTCTTACAGATCGAAACCTTAACA
CCACTTTCTTCGACCCGGCAGGCGGAGGAGATCCAATTCTATATCAACAT
CTCTTCT---

------------TTTCTAGAGAGAAACCTTCACCCATCTAACTGCCTTGG
CATGCTGCTGCTATCCGACGCCCACCAGTGCACCAAGCTGTCAGAGCTCT
CCTGGGGCATGTGCCTCAGCAACTTCCCCGCTATTTGTAAGACAGAGGAC
TTCCTCCAACTGCCCAAAGATATGGTGGTGCAGCTTTTGTCCCATGAGGA
GCTAGAGACTGAAGATGAGAGACTGGTGTATGAAGCTGCCCTCAATTGGA
TCAACTATGACCTAGAAAAGAGGCACTGCCACCTTCCAGAGCTCCTGAGA
ACGGTCCGTCTGGCCCTTCTGCCCGCCATTTTCCTCATGGAAAACGTCTC
TACAGAAGAGCTGATCAACGCCCAGGCCAAGAGCAAAGAGCTGGTGGACG
AGGCTATCCGCTGCAAGCTGAAGATCCTGCAGAATGATGGTGTGGTGAAC
AACCCATGTGCGCGTCCCAGAAAAACCAGCCATGCCCTCTTTCTTCTTGG
CGGGCAGACTTTCATGTGTGACAAGTTGTACCTGGTGGACCAGAAAGCCA
AAGAAATCATCCCCAAGGCTGACATCCCCAGCCCCAGGAAGGAGTTCAGT
GCCTGTGCCATTGGCTGCAAGGTGTACATCACAGGTGGGA--GAGGCTC-
AGAGAATGGTGTGTCCAAAGACGTGTGGGTCTACGACACCGTCCACGAGG

AATGGTCAAAGGCGGCGCCCATGCTTATCGCCCGGTTTGGCCATGGCTCT
GCGGAGCTGAAACACTGCCTCTACGTGGTCGGAGGCCACACTGCAGCAAC
CGGTTGCCTCCCAGCCTCTCCTTCTGGACAACTACATTGTTGTGTTTAGT
CGTTCCACCACAAGGCTGATACTGAATGAAGCAGAGCTAATTATGGCGCT
GGCCCAGGAGTTCCAGATGAGAGTGGTCACAGTATCTCTGGAGGAACATT
CTTTCCCCAGTATTGTCCAAGTGATAAGCAGTGCTACTATGTTAATCAGT
ATGCATGGAGCTCAGCTTATTACCTCACTGTTCCTCCCTCGAGGAGCTGT
TGTGGTTGAGCTATTCCCCTTTGCTGTGAACCCGGAGCAATATACTCCAT
ATAAAACCCTTGCCTTCCTTCCAGGCATGGACCTACACTATATCTCATGG
AGGAACAATAAAGAACAGAACACCATCACCCACCCACAGAGACCCTGGGA
ACAAGGGGGCATCATACACTTAGAGAAGGAGGAACAAAAGCGAATACTTA
CTAGCAAAGATGTTCCCAGACACCTGTGCTGCCGCAACCCAGAGTGGCTA
TTCCGGATCTACCAGGACACTTTGGTGGATATCCCTTCCTTCCTGGACAT
CCTCAA---AGAAGGCTTAAAG---ACGAAGCCTAGTTTGAAGAA---GT
TAAAACCAGCAAGCGCACTCCACCCTGGCCGGGTCAGAGAACCTCAGTGT
CAGACTTCAGTACAAACCAGTAATGAGGCTAGACTCACAGTCTCCTGGCA
GATTCCTTGGAATCTCAAGTACCTGAAGGTCAGAGAAGTGAAATATGAAG
TGTGGATCCAGAAGAAGGACACAAGCAAGGGGACGCTGGAGGATCAAATC
ATCCAGGCGAACCCTGCACTGGAAGCCTTCGGTAATGCCAAAACATTGAG
AAATGACAACTCGTCTCGTTTTGGAAAATTCATCCGAATTCACTTTGGTA
CGAGTGGCAAGCTGTCGTCCGCTGACATCGAGACGTACCTGCTGGAGAAG
TCACGTGTCACCTTTCAGCTCAAGGCTGAAAGAAATTACCACATCTTCTA
CCAGATACTGTCCAATCACAAGCCAGAGCTACTGGACATGTTGCTGATCA
CCAACAACCCGTACGACTACTCCTACATCTCCCAGGGAGAAGTTACGGTG
GCCTCCATCAATGACTCAGAGGAGCTGATGGCCACCGACAGCGCCTTTGA
CGTACTTGGCTTCACTTCAGAGGAGAAGATGGGCGTATACAAGCTGACGG
GCGCTATCATGCACTACGGCAACATGAAGTTCAAACAGAAGCAGCGAGAG
GAACAGGCTGAGCCGGATGGGACGGAGGCGGCGGATAAATCAGCTTACCT
CATGGGGCTGAACTCTGCTGATCTCATCAAAGGGCTGTGCCACCCCAGAG
TCAAGGTAGGAAACGAATATGTCACCAAAGGCCAAAGTGTGGACCAAGTC
TACTACCCCAACAAGGAGGCTTTCAAGTGTGAGGAGTGTGGGAAGCACTA
CAACACCAAGCTGGGATATAAGCGCCATGTGGCCATGCACTCTGCAACTG
CAGGCGATCTCACATGTAAAGTGTGCATGCAGACCTACGAGAGCACACCG
GTGCTCTTGGAGCACCTAAAGAGCCACTCTGGGAAGTCCTCAGGTGGCAC
CAAGGAGAAAAAACACCCCTGTGACCATTGTGATCGTCGCTTCTACACAC
GGAAGGACGTGAGACGGCACATGGTGGTCCACACAGGCCGAAAGGACTTC
CTGTGTCAGTACTGTGCCCAACGCTTTGGCAGGAAGGACCATTTGACACG
CCATGTGAAGAAGAGCCACTCACAGGAGCTGCTGAAAATTAAGACGGAGC
CTCCTGATATGTTAGGTCTTTTAGCTTCTGGGTCACCACCCTGCTCTGTG
AAGGAGGAGCTCAGCCCCATGATGTGTGGCATGGGTCCCAACAAGGACCC
CATGATGGGGAAACCTTTCCCCAGTGGAGCCCCATTTTCAATGGGCATGT
ATAACCCCCACCAT------CTCCAGGCCATGTCTAATCCTGGGGTGAGT
CACCCA------CACCCTTCCCTGATGCCCAGTTCCTTGTCTGCAGCTAT
GGGCATGAGTTGTCTCCTGGAANNNNNNNNNNNNNNNNNNNNATTTTCATGGGGAGTTTACAGATCAGT
G
ATGGATCAAACATTGTGAACCTGTTGGCTAGTAACTCTCCAAGTGTTTCA
TATGCTTTGACCCAGCAAAAATACTTCAGTAACTACAGTCCTGTGATCGG
GTTTTACATTTATGAGCCCATTGAGTATTGGAACTCAACGGTGCAGGAGC
ACCTGAAGACTCTGAGTAATGGCTTCAATAAGATCTCCTGGATGGACAAC
TTTTTCCACTACCTGAGAGTAGTGAATGTGAGTGCATCAACCAAGAAGGA
CTTCATTAACATCCTTAAGGGCTCCTTCCTGCGCAGCCCGGAGTACCAGC
ACTTCACTGAGGACATCATATTCTCTAAGA---ATCGTGAGACTG-----
-ATGAATACGACATTATTGCTTCAAGGATGTACTTGGTGGCACGTATAAC

AGAAAAGAAGCGTGAAGAGGTGGTGGAGCTTCTGGAAAAGCTTCGTCCAT
TAATGTTGATCAACAGCATCAAGTTCATTGCCTTCAATCCTACATTTGTG
TTCATGGACCGCTACAGTTCGTCCGTCATCTCACCCATCCTGACCTCAGG
ATTCAGCGTACTCACAATCCTCATCCTCACTTTTTTCTTGGTCATCAACC
CCTTNNNNNNNNNNNNNNNNNNNNNNNNNNNNNNNNNNNNNNNNNNNNNNNNNNNNNNNNNNNNNNNNNN
NNNNNNNNNNNNNNNNNNNNNNNNNNNNNNNNNNNNNNNNNNNNNNNNNNNNNNNNNNNNNNNNNNNNNN
NNNNNNNNNNNNNNNNNNNNNNNNNNNNNNNNNNNNNNNNNNNNNNNNNNNNNNNNNNNNNNNNNNNNNN
NNNNNNNNNNNNNNNNNNNNNNNNNNNNNNNNNNNNNNNNNNNNNNNNNNNNNNNNNNNNNNNNNNNNNN
NNNNNNNNNNNNNNNNNNNNNNNNNNNNNNNNNNNNNNNNNNNNNNNNNNNNNNNNNNNNNNNNNNCAGC
GAGAAGCACGGCGGTGGACCGGTTGTTCCTGAGAAGGCAGTA
CGTTTCTCTTTCACCGTTATGTCCGTGTCTGTGCGGGCAGATGGCAAGGA
G------------------------------GACGAAATTACCATCTACA
GGGAGCCAAAACCAAACTCAGAGCTGTCCTGTAAGCCCCTTTGCCTGATG
TTTGTGGATGAATCAGACCACGAGATGCTCACAGCCATCCTGTGGCCCAT
AATTGCAGAGCGTAATGCAATGAAAGAGAGCAGACTCATCATATCCATTG
GTGGACTGCCTCGCTCCTTCCGCTTTCACTTCCGAGGCACAGGCTATGAT
GAGAAGATGGTGCGTGAGATGGAGGGCCTAGAGGCCTCTGGATCCACCTA
CATCTGCACACTTTGTGATTCCGGTCGTGCAGAGGCCTCTCAAAACATGG
TCCTACACGCCATCACACGCAGTCACGAAGAGAACTTAGAGCGCTACGAA
ATATGGAGAACCAACCCTTTTTCTGAGTCTGTAGAGGTGCTTAGAGACAG
AGTCAAAGGGGTGTCTGCCAAGCCTTTCATGGAAACCCACCCAACGCTGG
ATGCACTCCACTGCGACATAGGCAACGCCACTGAGTTTTACAAAATTTTC
CAGGACGAGATTGGGGAAGTCTACCAAAAGGT---CAAC---CCCAGCCG
GGAGGAGCGGCGCAGCTGGAGAGCAGCCCTTGACAAACAGCTGAGGAAAA
AGATGAAGCTCAGACCCGTGATGAGGATGNNNNNNNNNNNNNNNNNNNNNNNNNNNNNNNNNNNNNNNNN
NNNNNNNNNNNNNNNNNNNNNNNNNNNNNNNNNNNNNNNNNNNNNNNNNNNNNNNNNNNNNNNNNNNNNN
NNNNNNNNNNNNNNNNNNNNNNNNNNNNNNNNNNNNNNNNNNNNNNNNNNNNNNNNNNNNNNNNNNNNNN
NNNNNNNNNNNNNNNNNNNNNNNNNNNNNNNNNNNNNNNNNNNNNNNNNNNNNNNNNNNNNNNNNNNNNN
NNNNNNNNNNNNNNNNNNNNNNNNNNNNNNNNNNNNNNNNNNNNNNNNNNNNNNNNNNNNNNNNNNNNNN
NNNNNNNNNNNNNNNNNNNNNNNNNNNNNNTCTTACACCATCGAGATGGGT
TCCTTGGGGCCCAGATGGAAGGAGAGTCCCCAGCCTTTCTCGTGCTCCAT
CGAAGACCCCACAAAACAGACAAAGTTTAAGGGCATCAAGACGTACATTT
CGTACCGGGTCACGCCGAGCCATACGGGGCACCCTGTCTACAGGCGTTAC
AAACACTTTGACTGGCTGTACAACCGCTTGCTGCACAAGTTCACTGTGAT
CTCCGTGCCCCACCTGCCTGAGAAGCAGGCCACAGGGCGATTTGAGGAGG
ACTTCATCGAAAAGCGCAAGAGGCGACTGATACTTTGGATGAACCACATG
ACCAGTCACCCAGTCCTCTCCCAGTATGAAGGCTTTGAGCACTTTCTGAT
GTGTGCCGACGACAAGCAGTGGAAGCTGGGCAAGAGGCGCGCAGAGAAAG
ATGAGATGGTGGGCGCGCATTTCATGCTGACCCTTCAGATTCCCAACGAG
CACCAAGACCTTCAGGATGTAGAGGAGCGGATTGACTCCTTCAAGTCCTT
TGCCAAGAAAATGGACGACAGCGTGATGCAGCTCACGCATGTCACCTCAG
AGCTGGTGCGAAAGCACCTGGGTGGGTTCAGGAAGGAGTTCCAGCGGCTG
GGAAATGCCTTCCAGTCCATCAGCCAGGCATTCATGCTGGACCCTCCCCA
CAGCTCAGAGGCCTTAAACAACGCCATCTCCCATNNNNNNNNCGCCGTTCTCAAACTG
ACCTCTCTGGGTTTCATCATTGGAGTCGGTGTGGTTGGAAACCTACTGAT
CTCCATCCTGCTGGTCAAAGACAAAAGCCTGCACCGAGCGCCCTACTATT
TCCTACTGGACCTGTGCGCCTCTGACATACTTCGGTCCGCCATCTGCTTC
CCCTTTGTCTTTACCTCGGTCAAGAATGGATCTGCCTGGACGTATAGTAC
GCTGACCTGCAAAGTGATAGCCTTCCTGGGTGTGCTCTCCTGTTTCCACA
CGGCGTTCATGCTGTTCTGCGTCAGTGTCACGCGCTATCTGGCCATCGCA
CATCACCGTTTCTACACCAAGAGGCTGACCTTCTGGACCTGTCTAGCTGT
CATCTGCATGGTGTGGACGTTATCAGTGGCCATGGCCTTCCCACCAGTGC
TCGACGTAGGGACGTACTCTTTTATCCATCAGGAGGACCAATGCACGTTC

CAGCACCGCTCCTTCAGGGTGAACGATTCGCTGGGCTTTATGCTCCTGCT
GGCGCTCATCCTCCTGGCCACACAGCTGGTTTACCTCAAGCTCATCTTTT
TCGTCCACGACCGTCGAAAGATGAAGCCCGTCCAGTTCGTGCCTGCTGTT
AGCCAGAACTGGACCTTCCACGGGCCCGGTGCCAGTGGGCAGGCGGCCGC
CAACTGGTTGGCCGGATTCGGGCGAGGCCCCACCCCACCTACTCTGCTGG
GCATCCGGCAGAACAGCAATGCAGCGGGCCGCAGGCGTCTACTGGTACTG
GATGAATTCAAAACAGAGAAGAGGATTAGTAGGATGTTCTACATCATGAC
CTTTTTCTTCCTGGCACTGTGGGGGCCTTATCTGGTGGCCTGCTACTGGC
GGGTGTTTGCGAGGGGCCCCGCGGTCCCTGGGGGCTACCTGACGGCAGCC
GTGTGGATGAGCTTTGCCCAGGCTGGGGTCAATCCTTTCATCNNNNNNNNNNNNNNNNNNNNNGCCAAAT
C
TCGCTTTCACCCTGGCATGGGCACTGGTGCTGGCACCGAGC---GCAGCG
TCCCACTCGGCAACAGCTTGCTGTCCCCGCAGCAAACCGAGGAGCCCACT
GTTGCCACCCCCCCGCAGCGATGGTTTGTCACCC---CTGCCAACAACCG
ACTGGACTTTGCTGCCTCGGCATACGACGCCGCC---------GATTTCG
CCGGTAACGCAGCCACCTTGCTGTCCTACGCAGCGGCCGGAGTGAAGGCT
C------TCCCCCTGCCGACCGCAGGCTGCTCCAACCGGCCTCTTGGCTA
TTACGCAGACCCGTCGG---GCTGG---GGAGGACGCACGCCGCCGCAGT
ACTGTGGCGTGAACAGCAAACCCAACTCGGTCTTTTCCTGCTGGCCCTCT
AACTCGATCGGGGGCAGGGCGGGCA---CC---AACTACCTGG-------
--CCGAGGA---GGGG---GAGCC---CATTCCGACCGAGAGGTCACCG-
--AT---CGTTGGCTCCGAGGAG---ACCAAAGCCAAGGATATAACGGGG
TCGGA---GTCGAACTGGATAGAG---ACGCCCCCCTCCATAAAGTCCAT
CGATTCGAGCGACTCTGGAATCTTTG---AACAGGCCAAACGGAGGCGGA
TCTCACCGTCCGCCACGCCG---------GTCTCAGAGACGGTTCCCCGC
TCNNNNNNNNNNNNNNNNNNNNNNNNNNNNNNNNNNNNNNNNNNNNNNNNNNNNNNNNNNNNNNNNNNNN
NNNNNNNNNNNNNNNNNNNNNNNNNNNNNCACAGCTCCCACGATATTGGCTCCGG---
ACAGACTGCGTTTTCCTCT
CAGGCG---CCCGGGTACGCAGCCGCAGCAGCCCTTGGA---CACCATCA
------CCACCCGACCCACGTTGGCTCT---TACTCCACGGCGGCTTTCA
ACTCCACCAGGGACTTTCTCTTCAGAAATCGGGGATTCGGGGACGCCACC
AG------------TGCGCAGCACAGTTTGTTCGCGTC---------CGG
AAGTTT---C------GCAGGGCCACATGGACACTCCGATGCAGCAGGAC
ACCTGCTCTTCCCCGGGCTCCACGAG---CAGGCGGCAAGCCACGCGCCT
TCCAATGTGGTCAACAGCCAGATGCGGCTGGGCTTCTCGGGGGACATGTA
TGGACGAGCCGAGCAGTACGGCCACGTTACGAGTCCCCGGT---CTGACC
ACTACGCGTCGACCCAGCTGCACGGCTACGGCCCCATGAACATGAATATG
GCAGCG---CACCACGGAGCGGGGGCCTTCTTCCGATACATGCGGCAGCC
GATCAAACAAGAGCTTATCTGCAAGTGGATCGAGCCGGAGCAGCTGACAA
ACCCCAAAAAGTCGTGCAACAAAACTTTTAGCACGATGCACGAGCTCGTG
ACTCATCTGACGGTGGAGCATGTAGGGGGACCGGAGCAGACCAACCATGT
GTGCTTCTGGGAGGACTGCTCCAGAGAAGGAAAGCCATTCAAAGCCAAAT
ACAAACTTGTGAATCATATCAGAGTACACACCGGAGAAAAGCCCTTCCCG
TGTCCGTNNNNNNNNNNNNNNNNN
>Monocentris japonica
AGCCTCCTCATCCGGGCTGAACTTAGTCAGCCAGGGGCTTTACTGGGAGA
CGACCAGATTTATAATGTTATTGTTACAGCACATGCTTTTGTAATAATTT
TCTTTATAGTAATACCAATTATAATTGGTGGCTTTGGGAACTGACTTGTA
CCTCTAATAATCGGAGCCCCCGACATGGCATTTCCTCGCATAAATAATAT
GAGCTTCTGACTTCTTCCCCCATCATTCCTCCTTCTCCTTGCCTCTTCTG
GGGTTGAAGCGGGGGCCGGTACAGGCTGAACAGTCTACCCGCCTCTCGCA
GGAAACCTTGCCCACGCAGGTGCCTCCGTGGACCTAACCATTTTCTCCCT
GCATTTGGCCGGTATTTCCTCTATTTTAGGGGCTATCAACTTTATTACAA

CTATCATTAATATAAAACCTCCCGCCATTTCCCAATACCAGACCCCTCTG
TTCGTATGGGCCGTTTTAATTACAGCAGTCCTTCTCCTCCTATCCCTCCC
CGTACTTGCAGCCGGCATCACCATGCTCCTAACAGATCGTAACCTAAACA
CAACCTTCTTTGATCCTGCAGGAGGAGGAGACCCTATCCTGTATCAACAC
CTGTTT--

------------TTTCTAGAGAGAAACCTTCATCCATCTAACTGCCTGGG
CATGCTGTTGCTGTTTGATGCACACCAGTGTACCAAGCTATCAGAGCTCT
CCTGGGGCATGTGCCTCAGCAACTTTCCGGCTATTTGCAAGACAGAGGAC
TTCCTCCAGCTGCCCAAAGACATGGTGGTGCAGCTTCTATCCCATGAGGA
GCTGGAGACAGAAGATGAGAGACTGGTTTATGAAGCTGCCCTYAACTGGG
TCAACTATGACTTGGAAAGGAGGCACTGCCACCTGCCAGAGCTGCTGAGA
ACGGTTCGCCTGGCCCTGCTACCTGCCATCTTCCTCATGGAGAATGTCTC
TACAGAAGAGCTGATCAATGCCCAGGCCAAGAGCAAGGAGCTGGTGGATG
AGGCCATCCGGTGCAAGCTGAAGATCTTGCAGAATGACGGTGTGGTTAAC
AGCCCCTGTGCTCGGCCGAGAAAAACCAGCCATGCCCTTTTCCTCTTGGG
AGGGCAGACCTTCATGTGTGACAAGCTGTATTTGGTGGATCAGAAGGCAA
AAGAGATCATCCCCAAGGCTGACATCCCCAGCCCTAGGAAGGAGTTCAGT
GCCTGCGCCATCGGCTGTAAAGTCTACATCACAGGTGGCA--GAGGCTC-
AGAAAATGGTGTGTCTAAAGATGTGTGGGTCTATGACACTGTCCATGAGG
AATGGTCCAAGGCAGCACCCATGCTCATCGCTAGATTTGGTCATGGCTCT
GCTGAGCTGAAACACTGCCTTTACNNNNNNNNNNNNNNNNNNNNNNNNNNNNNNNNNNNNNNNNNNNNNN
NNNNNGGATGAATACATTGTGGTGTTCAGCC
GTTCAACAACAAGGCTGATTCTGAACGAAGCAGAGCTAATCATGACACTG
GCCCAGGAATTTCAGATGAGAGTGGTTACAGTCTCCCTAGAGGAACAAAC
TTTCCCCAGCATCGCCCAGGTGATCAGTGGGGCCTCCATGTTAGTCAGTA
TGCATGGAGCTCAGCTTGTCACCTCACTCTTCCTCCCCAGAGGAGCTGCT
GTGGTGGAGCTGTTCCCCTATGCTGTCAACCCAGAACAGTACACCCCATA
TAAAACCCTCGCCTCCCTACCAGGCATGGATCTTCAATATGTTTCCTGGA
GGAACACTATAGAGGAGAACACTGTCACCCACCCAGACAGACCGTGGGAA
CAAGGAGGCATCACCCATTTGGAAAAGGACGAACAAGAGCGAATACTGGC
CAGCAATGATGTCCCCAGACACCTGTGCTGCCGCAACCCAGAGTGGCTCT
TCCGAATCTACCAGGACACTTTGGTGGACATCCCTTCATTCCTGGAGGTC
CTCAA---AGAGGGCCTGAAG---ACCAGGCCCAGCTTGAAGAA---GAC
CAAGCTGGCCAGCACGGTTCACCCAGGCCGGGTCAGAGAACCCCAGTGCC
ACACCTCAGTCCAAGCCACCAACGAGGCTAAACTCACGGTTTCCTGGCAG
ATCCCATGGAATCTGAAATACCTGAAGGTGAGAGAAGTGAAGTACGAGGT
GTGGATCCAGAAGAAGGATACCAGCAAGGGAACACTGGAGGATCAAATCA
TCCAGGCAAACCCTGCACTGGAGGCTTTTGGTAATGCCAAAACAGTGAGG
AATGACAATTCATCCCGTTTTGGAAAATTCATCCGAATTCACTTTGGAAC
CAGTGGCAAACTGTCCTCTGCTGATATTGAAACTTACCTATTGGAGAAGT
CACGTGTCACCTTTCAGCTCAAGTCTGAGAGGAACTACCACATCTTCTTC
CAGATATTGTCCAATCAGAAGCCAGAGCTGTTGGACATGATGTTGATCAC
TAACAACCCATATGACTACTCCTACATCTCCCAAGGAGAGGTAACAGTAG
CATCCATCAACGACTCAGAGGAGCTGATGGCCACTGACAGCGCCTTTGAC
GTGCTTGGCTTCACTCAAGAGGAGAAGATGGGAGTATATAAGCTGATTGG
TGCCATTATGCACTATGGCAACATGAAGTTTAAGCAGAAGCAGCGTGAGG
AGCAGGCTGAGCCTGACGGAACAGAGGCTGCTGATAAGTCAGCATACCTT
ATGGGGCTGAACTCTGCAGACCTCATCAAAGGGCTATGCCATCCCAGAGT
CAAGGTAGGAAATGAATATGTCACCAAAGGCCAAGGTGTAGACCAAGTCT
ACTACCCAAACAAGGAGGCCTTCAAGTGCGAGGAGTGTGGCAAGCACTAC

AACACCAAGCTGGGATACAAACGCCATGTGGCCATGCACTCTGCCACAGC
AGGGGACCTCACCTGTAAAGTGTGCATGCAGAGCTATGAGAGCACGCCGG
TGCTCCTGGAGCACCTTAAGAGCCACTCGGGGAAGTCCTCAGGTGGCGCC
AAGGAGAAAAAACACCCATGCGACCACTGCGACCGCCGTTTTTACACACG
GAAGGATGTGAGACGGCACATGGTGGTCCACACGGGCCGAAAGGACTTCC
TGTGCCAGTACTGTGCCCAGCGCTTTGGCAGGAAGGACCACCTGACACGC
CATGTAAAGAAGAGCCACTCACAAGAGCTGCTGAAGATCAAAACGGAGCC
TCCAGATATGTTAGGTCTTTTAGGTTCTGGCTCACCGCCATGCTCTGTCA
AGGAGGAGCTTAGCCCTATGATGTGCAGCATGGGTCCCAACAAAGACCCC
ATGATGGGCAAACCCTTCCCCAGCGGGACCCCATTCCCCATGGGTATGTA
CAACCCCCACCAC------CTCCAGGCCATGTCCAATTCTGGGGTGGGTC
ACCCC------CACCCCTCCCTGATGCCTAGCTCCCTGTCTGCAGCTATG
GGCATGGGCTGTCACATGGAATATCTCATCTATGCCTCTTTCTCATTCAT
GGGTTGTTTACAAATCAGTGATGGCTCAAACATTGTGAACTTGCTGGCTA
GTAACTCTCCGAGCGTTTCATATGCTCTGACCCAGCAGAAATACTTCAGT
AACTACAGTCCTGTGATTGGATTTTACATTTATGAGCCCATTGAGTACTG
GAACTCCACAGTGCAGGAGCACCTGAAGACACTGAGTCATGGCTTCAACA
AGATCTCCTGGATGGACAACTTCTTCCACTACCTGCGGGTGGTGAACGTG
AGCGCGTCGACCAAGAATGACTTTATCAGCATCCTCAAGGGCTCTTTCCT
GCGCAGCCCGGAGTACCAGCACTTCACTGAGGACATCATTTTCTCCAAGA
---ACCGTGAGAGTG------ATGAGTATGACATTATTGCCTCGCGCATG
TACCTAGTGGCGCGGACCACAGAGAAGAAGCGCGAGGAGGTGGTGGAGCT
TCTGGAGAAACTGCGTCCACTGATGCTGATCAATAGCATCAAGTTCATCG
CCTTCAACCCCACCTTTGTTTTCATGGACCGCTACAGCTCTTCAGTCATC
TCGCCCATCCTGACCTCAGGCTTCAGCGTGCTCACCATCCTCATCCTAAC
TTTCTTCCTGGTCATCAACCCCTTGGGGAACTTCTGGCTCATCCTCACAG
TTACTTCTGTGGAGCTGGGTGTCTTGGGTTTAATGGGCTATCATCTATTT
GAATGGCAGCCGGCCCTCAAGAATGTGTCTGCATCCTGCCATGTGGGCAT
TATTAATGGGCTCTCTGGAGGGACTGCCTTGGTGGATGACTCCCCACCTG
ACACCATTACTCGACGGTTTCGCTACGATGTGGCCCTGGTATCAGCCTTA
AAGGATCTGGAGGAGCACATCATGGAGGGACTGAGAGAGTGTGGGCTGGA
AGACAATGCTTGCACCTCAGGCTTCAGTGTTATGATCAAGGAATCTTGTG
ACGGCATGGGAGATGTCAGTGAGAAGCACGGGGGAGGGCCAGTGGTCCCC
GAGAAGGCTGTACGCTTCTCTATTACTGTTATGTCTGTCTCTGTCCTGGC
AGATGGAGAGGAA------------------------------GAGGCGG
TTACCATCTTTAGGGAGCCAAAGCCCAACTCAGAACTGTCCTGTAAGCCC
CTAAGCCTGATGTTTGTGGATGAGTCAGACCATGAGACAGTCACTGCTGT
CCTGGGACCTTTAATTGCAGAGCGTAATGCAATGAAGCAGAGTCAACTCA
TCCTATCTATGGGTGGCCTCCCTCGCTCCTTTCGCTTCCACTTCAGAGCC
ACAGGATATGATGAGAAGATGGTGCGTGAGATGGAGGGCCTGGAGGCCTC
AGGTTCCACCTATATCTGCACTCTGTGTGATTCCAGTCGAGCAGAAGCCT
CTCACAACATGGTTCTACATTCCATCACCCGCAACCACGATGAGAACCTG
GAGCGTTATGAAATATGGAGGACCAATCCCTTTTCTGAGTCTGTTGAGGA
GCTGCGAGACCGGGTCAAAGGGATCTCTGCCAAGCCCTTTATGGAGACCC
AGCCCACTATGGATGCATTACACTGTGACATTGGTAATGCCACAGAGTTC
TACAAAATCTTCCAGGATGAGATTGGGGAAGTGTATCAGAAGGC---CAA
C---CCCACCCGAGAGGAACGGCGGAGCTGGCGAGCAGCCCTAGACAANCA
NCTGAGGAAGAAGATGAAGCTTAANACAGTGATGAGGATGAATGGGAATTAT
GCCCGGAGGCTAATGACCAAGGAGGCTGTGGAAGTGGTGTGTGAGCTGGT
ACCTTCAGAAGAGAGGCGAGAGGTCCTGAGGGAGCTTATGGGGCTCTACA
TCCAGATGAGCCTGGTGTGGCGCGCCTACCTGCCAGCCAAGGAATGCCCT
GATGAGCTATGTCCCTACACGTTTCACTCCCAGACATTTGCCGATCTCCT
CTCCANTACCTTCAATAATGGAACAAAGGGAAAGATCACCAATTTCCTGCA

CAAGACATTCGCACATGTCCTTGAAACTATTGTAAGANNNNNNNNNNNNNNNNNNNNNNNNNNNNNN
NNNNNNNNNNNNNNNNNNNTCGTACACCATTG
AGATGGCTCCTAAGGGGCCCCAATGGAAGGAGAGTCCTCAGCCTTTCTCC
TGCTCCATTGAAGACCCCACAAAACAGACCAAGTTCAAAGGCATCAAGAC
CTACATTTCGTACCGGGTCACGCCGAGCCACACAGGGCGTCCCGTCTACA
GACGCTACAAACACTTTGACTGGCTGTACAACCGCTTACTGCACAAGTTC
ACTGTGATCTCGGTGCCCCACCTGCCTGAGAAGCAGGCCACGGGGCGCTT
CGAGGAGGACTTCATTGAGAAGCGTAAAAGACGACTGATTCTGTGGATGA
ACCACATGACTAGTCACCCAGTCCTCTCCCAGTATGAGGGGTTTGAGCAC
TTCCTTATGTGTGCTGATGACAAGCAATGGAAGCTGGGTAAGAGGCGGGC
GGAGAAGGACGAGATGGTGGGCGCCCACTTCATGCTGACCTTCCAGATTC
CTAACGAGCATCAGGATCTCCAGGATGTGGAGGAGCGGGTTGACTCCTTC
AAGTCCTTTGCTAAGAAAATGGATGACAGCGTCATGCAGCTCACACATGT
TGCCTCGGAGCTGGTGCGTAAGCACCTGGGTGGATTCAGGAAGGAGTTCC
AGCGGCTAGGAAATGCCTTCCAGTCTATCAGCCAGGCATTCATGCTGGAA
CCTCCCCACAGCTCTGATGCCCTCAACAACGCCATCTCCCACCCTCTCGC
CACGTTCCTCAAACTGACCTCTCTGGGTTTCATCATTGGAGTCGGTGTGG
TCGGGAACCTCCTGATCTCCATCCTGCTGGTCAAAGACAAGAGCCTGCAC
CGCGCACCCTACTACTTCCTGCTAGACCTGTGCGCCTCTGACATCCTGCG
CTCTGCCATCTGCTTCCCCTTCGTCTTCACCTCTGTCAAGAATGGATCCG
CCTGGACCTATGGCACGCTCACCTGCAAAGTGATCGCCTTCCTGGGTGTG
CTCTCCTGTTTCCACACGGCGTTCATGCTATTCTGTGTCAGTGTCACTCG
CTACCTGGCTATTGCGCATCACCGCTTCTACACCAAGAGGCTGACCTTCT
GGACCTGTCTAGCTGTCATCTGCATGGTGTGGACGTTGTCAGTAGCCATG
GCTTTTCCCCCAGTGCTGGACGTAGGGACGTACTCCTTCATCCGAGAGGA
GGACCAGTGCACATTCCAGCACCGTTCTTTCAGGGCCAATGACTCACTGG
GCTTCATGCTCCTGCTGGCACTCATCCTCCTGGCCACACAGCTGGTTTAC
CTCAAGCTCATCTTCTTTGTCCACGACCGTCGGAAGATGAAGCCCGTGCA
GTTCGTGCCTGCCGTCAGCCAGAACTGGACCTTCCATGGGCCAGGCGCCA
GTGGGCAGGCGGCAGCTAACTGGCTTGCTGGATTTGGGAGAGGCCCCACC
CCGCCTACCTTGCTGGGCATCCGGCAGAACAGCAACGCGGCGGGCCGCAG
GCGTCTGCTGGTGCTGGATGAGTTCAAAACAGAGAAGAGGATTAGTAGGA
TGTTCTACATCATGACGTTTTTCTTCCTGGCGTTGTGGGGGCCCTATCTG
GTAGCCTGCTACTGGCGGGTGTTTGCAAGGGGCCCCGTAGTCCCCGGGGG
CTACCTGACGGCAGCCGTGTGGATGAGCTTTGCCCAGGCTGGGGTCAATC
CCTTCATCTGCATNNNNNNNNNNNNNNNNNGCCAAATCTCGCTTTCACCCTGGCGTGGGGACTGGTC
CTGGCACGGAGC---GCAGCGTCCCACTCGGCAACAGCTTGCTATCCCCG
CAGCAAACCGAGGAGCCCACTGTTGCCACCCCCCCGCAGCGATGGTTTGT
CACCC---CTGCCAACAACCGACTGGACTTTGCTGCCTCGGCATACGACG
CGGCC---------GATTTCGCCGGTAACGCGGCCACCTTGCTGTCCTAC
GCAGCGGCCGGAGTGAAGGCTC------TTCCTCTGCCGGCTGCAGGCTG
CTCCAACCGGCCTCTTGGCTATTACGCAGACCCGTCGG---GCTGG---G
GCGGACGCACGCCGCCGCAGTACTGTAGCGTAAACAGCAAACCAAGCTCG
GTCCTTTCCTGCTGGCCCTCTAACTCTGTCGGCGGCAGAGCAGGCG---C
C---AACTACCTGG---------CCGAGGA---CGGA---GACGC---CA
TCCCGACGGAGAGATCCCCG---AT---CGGTGGCTCCGAGGAG---ACG
AAACCCAAGGACCT------GTCGGA---GTCGAGCTGGATAGAG---AC
GCCGTCCTCCATTAAGTCAATTGATTCAAGCGATTCTGGTATCTTCG---
AACAGGCCAAAAGAAGAAGAATCTCTCCCTCTGCCACGCCG---------
GTTTCAGAAACTGTGTCCCCGTTAAAATCTNNNNNNNNNTCAACAGGCGAAGTCACAGA
GAGAGAAGTGGCGTTGGGGATAAATCCGTTCGCGGACGGGATGGGCGCCT
TCAAAATCAACCACAGCTCCCACGATATTGGCTCCGG---ACAGACGGCG
TTTTCCTCCCAGGCG---CCCGGCTAC---GCAGCGGCCGCCCTGGGA--

```
-CACCATCA------CCACCCGACCCACGTTGGCTCT---TACTCCACGG
CGGCTTTCAACTCCACCCGGGACTTTCTCTTCAGAAATCGGGGTTTCGGA
GACGCCACCGG------------GGCGCAGCACAGTTTGTTCGCCTC---
------CGGAAGTTT---C------GCAGGGCCACATGGACACTCAGATG
CCGCGGGACACCTGCTCTTCCCAGGACTCCACGAG---CAAGCAGCGAGC
CACGCGTCTTCTAACGTGGTCAACAGCCAGATGCGATTGGGCTTCTCGGG
GGACATGTATGGTCGGGCCGACCAGTATGGCCACGTTACAAGCCCAAGGT
---CCGACCACTATGCTTCGACCCAGTTGCACGGCTATGGCCCCATGAAC
ATGAATATGGCCGCA---CACCACGGAGCAGGGGCCTTCTTTAGATACAT
GAGGCAGCCGATCAAACAAGAGCTCATCTGCAAGTGGATCGAGCCGGAGC
AACTGACGAATCCCAAAAAGTCGTGCAACAAAACTTTTAGTACGATGCAT
GAGCTGGTGACCCATCTGACGGTGGAGCATGTGGGGGGACCAGAGCAGTC
CAACCACATCTGCTTCTGGGAAGACTGCGCCAGAGAAGGGAAACCATTCA
AAGCCAAATACAAACTTGTAAATCATATCAGAGTACACACCGGAGAGAAA
CCCTTTCCATGTCCGTTCCCGGGCTGTGGNNNN
>Monopterus albus
AGCCTATTAATTCGAGCCGAACTCTGCCAGCCCGGCTCCCTCCTAGGGGA
TGACCAAATCTACAATGTTATTGTAACAGCGCATGCATTTATTATAATCT
TCTTTATAGTAATACCTATCATAATCGGGGGCTTTGGAAACTGGTTAGTC
CCACTAATAATCGGGGCCCCAGACATAGCCTTTCCCCGAATAAACAACAT
AAGCTTTTGACTCCTCCCCCCCTCTTTTCTATTACTCTTAGCCTCCGCCG
GCATTGAAGCCGGGGCGGGAACAGGTTGAACTGTCTACCCCCCTCTCGCT
GGAAACCTTGCTCACGCAGGCGCCTCTGTTGACCTTACAATCTTCTCCTT
ACACCTAGCAGGTGTTTCTTCAATTCTTGGGGCCATCAACTTTATCACAA
CCATCATCAACATAAAACCTCCCGCAATTTCTCAGTATCAAACCCCCCTA
TTCGTATGATCTGTCATAATCACAGCCATTCTTCTCCTTCTATCGCTCCC
TGTGCTAGCAGCCGGAATTACAATATTACTAACGGACCGAAACCTTAACA
CAACTTTCTTTGACCCTGCTGGAGGGGGGGACCCCATCCTGTACCAACAC
CTATTCTGATTCTTCGGCCACCCTGAAGTATATATCTTGATTCTTCCAGG
CTTTGGAATAATCTCTCATATCGTTACCTTTTATGCTGGTAAAAAAGAAC
CTTTCGGCTATATAGGGATAGTGTGAGCAATAATAGCCATTGGCCTACTA
GGCTTTATTGTTTGAGCTCATCATATATTCACAGTCGGGATAGACGTTGA
CACACGAG----TTCCTGGAGAGAAACCTTCACCCATCTAATTGTCTTGG
CATGTTGCTGCTGTCCGACGCCCACCAGTGTACCAAGCTGTCAGAGCTCT
CCTGGGGCATGTGCCTCAGCAACTTCCCTGCTATTTGCAAGAMTGACGAC
TTCCTCCAACTGCCCAAAGATATGGTGGTGCAGCTTTTGTCACATGAGGA
GCTTGAGACAGAAGATGAGAAACTGGTTTATGAAGCTGCCCTTAACTGGA
TCAACTATGACTTGGAAAGGAGGCACTGCCACCTTCCAGAGCTCCTGCGA
ACAGTCCGCCTGGCTCTACTGCCTGCCATCTTTCTGATGGAGAATGTCTC
GACAGAAGAGCTGATCAATGCCCAGGCCAAGAGCAAGGAGCTGGTGGATG
AAGCTATCCGCTGTAAGCTGAAGATCCTGCAGAATGATGGTGTTGTTAAC
AGCCCATGTGCTCGACCAAGAAAAACCAGTCATGCCCTTTTTCTTCTGGG
AGGGCAAACCTTCATGTGTGACAAGTTGTACCTGGTRGAYCAGAAGGCCA
AAGAGATCATCCCCAAGGCTGATATCCCCAGCCCCAGGAAGGAATTTAGC
GCCTGCGCCATCGGGTGTAAGGTGTACATCACTGGAGGGA--GAGGCTC-
AGAGAACGGTGTGTCCAAAGATGTGTGGGTCTATGACACCATCCATGAGG
AATGGTCCAAAGCGGCTCCCATGCTYATCGCCAGGTTTGGCCAYGGCTCT
ACAGAGCTGAAACACTGCCTCTATGTGGTAGGAGGTCACACTGCAGCAAC
TGGCTGCCTCCCGGCTTCTCCATCAGGATGATTATATTGTTGTGTTTACT
CGTTCAACAACAAGGCTGATAATTAATGAAGCAGAGCTAATCATGGCACT
GGCACAGGAGTTCCAGATGAAAGTAGTCACAGTATCCCTGGAGGAACAGT
CTTTCAATAGTATCGTCCAGGTGATCAGTGGTGCTTCCATGTTAGTAAGC
ATGCATGGAGCTCAACTAATCACCTCACTTTTCCTCCCCAGAGGAGCTAC
```

TGTGGTGGAGCTGTTCCCCTTTGCTGTAAACCCAGAACAGTACACCCCAT
ATAAAACCCTTGCATCTCTTCCAGGCATGGATCTTCACTATATCTCTTGG
AGGAACACTAAGGAAGAGAACACCATCACCCACCCAGACAGAACCTGGGA
ACAAGGAGGCATTGCTCACTTGGAGAAGGAAGAGCAAGAGCGAATACTGG
CCAGTAAGGATGTCCCCAGGCACCTGTGCTGTCGTAACCCAGAGTGGCTC
TTCCGGATCTACCAGGACACATTGGTGGACATTCCTTCTTTCTTGGAAAT
TCTCAA---AGAGGGCATGAAG---ACCAAGCCCAGCTTTAAGAA---GT
CAAAGTTAGCCAGCACAGTCCACCCAGGCCGGGTTAGAGAACCCCAGTGT
CAGACCTCTGTACAAACTACTAATGAGGCTAAACTCACAGTCTCCTGGCA
GATCCCATGGAATCTGAAATACCTGAAGGTAAGAGAGGTGAAGTATGAAG
TGTGGATCCAGAAAAAAGACAGCAGCAAGGGGACGCTGGAGGATCAGATC
ATCCAGGCAAACCCTGCACTGGAGGCCTTTGGCAACGCTAAAACAGTAAG
AAATGACAACTCCTCTCGTTTTGGAAAATTCATACGAATTCATTTTGGTA
CAAGTGGAAAACTGGCATCTGCTGACATTGAGACGTACCTGCTGGAGAAG
TCACGTGTCACCTTTCAGCTCAAGGCTGAGAGAAGCTACCATGTCTTCTA
CCAGATCCTGTCCAATCAGAAGCCAGAGCTGTTGGACATGCTACTGGTCA
CCAACAACCCATACGACTACGCCTACATCTCCCAAGGAGAGGTAACAGTT
GCTTCTATCAATGACTCAGAGGAGCTGATGGCCACTGACAATGCCTTTGA
TGTGCTTGGCTTCACTCCAGAGGAGAAGATGGGCATCTATAAATTGACTG
GTGCCATCATGCACTATGGCAACATGAAGTTCAAACAGAAGCAGCGTGAG
GAGCAGGCTGAACCTGACGGGACAGAGGCGGCTGATAAGACAGCTTTCTT
AATGGGGCTAAACTCTGCTGACCTCATCAAAGGCCTGTGCCATCCCAGAG
TCAAGGTAGGAAATGAGTACGTCACCAAGGGCCAAGGCGTGGACCAAGTC
TACTATCCCAACAAGGAGGCCTTCAAGTGTGAGGAGTGTGGGAAGCACTA
CAACACCAAGCTAGGATATAAGCGCCATGTGGCCATGCACTCTGCCACTG
CAGGGGATCTCACCTGTAAAGTGTGCATGCAGAGCTATGAGAGTACACCT
GTTCTCCTGGAGCATCTCAAGAGCCACTCCGGGAAGTCTTCAAGTGGCAC
CAAGGAAAAGAAACATCCATGCGACCACTGTGACCGCCGTTTCTACACGC
GGAAGGATGTGAGGCGGCACATGGTGGTACACACAGGTCGAAAGGACTTC
CTGTGCCAGTACTGTGCCCAGCGCTTCGGTAGGAAGGACCACCTGACACG
CCACGTGAAGAAAAGCCACTCCCAGGAGCTGCTGAAGATCAAGACAGAGC
CTCCGGACATGTTAGGTCTTTTAGCTTCAGGGTCACCACCTTGTTCTGTC
AAGGAGGAGCTCAGCCCCATGATGTGCAGCATGGGTCCCAACAAAGACCC
CATGATGGGCAAACCATTCCCCAGTGGGGCGCCTTTTCCAATGGGCATGT
ACAACCCCCACCAT------CTTCAGGCCATGTCTAATAGTGGGGTGGGT
CACCCA------CACCCATCCCTGATGCCCACTTCCTTGTCTGCAGCTAT
GGGGATGGGCTGTCACATGGAANNNNNNNNNNNNNNNNNNNNNNNNNNNNGGGGTGTTTACAGATCAGT
GATGGATCA
AACATTGTGAACCTACTGGCTAGTAACTCTCCAAGTGTTTCATATGCACT
GACCCAACAGAAATATTTCAGTAACTACAGTCCTGTGATTGGATTTTACA
TTTACGAGCCCATTGAGTACTGGAACTCGACGGTGCAGGAGCACCTGAAG
TCTCTGAGTCATGGCTTCAACAAGATCTCCTGGATGGACAACTTTTTCCA
CTACCTGAAGGTAGTAAATGTGAGTGCATCAACCAAGAGCGATTTTATCA
ACATCCTCAAGGGCTCTTTCCTCCGCAGCCCAGAATACCAACACTTCACT
GAGGACATCATTTTCTCTAAGA---ACCGTGAGACTG------ACGAGTA
TGATATTATTGCCTCACGGATGTACCTGGTGGCACGGACAACAGAGAAAA
AGCGCGAGGAGGTGGTGGAGCTTTTGGAAAAGCTTCGTCCGTTGATGCTG
ATCAACAGCATCAAGTTCATTGCCTTCAATCCAACATTTGTGTTCATGGA
CCGCTACAGCTCCTCTGTCATTTCACCCATCCTGACCTCAGGCTTCAGCG
TACTCACTATTCTCATCCTCACTTTCTTCCTGATCATCAACCCCCCTGGGA
AACTTCNNNNNNNNNNNNNNNNNNNNNNNNNNNNNNNNNNNNNNNNNNNNNNNNGGCTTCCACCAGT
TTGAATGGCAGCCAGCTCTCAAGAATGTGTC
TCCATCCTGCAATGTCAGCATTATTAATGGGCTCTCTGGATGGGCTTCCT

CAGTGGATGACTCTGCAGCTGATACCATCGGTCGGCGTTTTCGCTATGAT
GTGGCACTGGTGTCAGCATTAAAGGATCTGGAGGAGGACATCATGGAGGG
GCTGAGAGAGAGTGGGATGGAAGACAGTGCTTGCACCTCAGGCTTTAGTG
TCATGATCAAGGAATGTTGTGATGGCATGGGTGACGTCAGCGAGAAGCAC
GGCGGGGGACCGATGGTTCCTGAGAAAGCTGTACGTTTCTCTTTCACTGT
TATGTCTGTCTCTGTCCTGGCGGATGATAGGGAG----------------
--------------AAGGAGGTTATCATTTTCACTGAGCCAAAGCCAAAC
TCAGAACTATCCTGTAAGCCCCTATGCCTGATGTTTGCAGATGAGTCAGA
CCATGAGACACTCACAGCTGTCCTGGGGCCTATAGTTGCAGAGCGTAATG
CAATGAAAGAGAGCCGACTCATTCTATCTATTGGCGGACTTGCCTCGCTC
CTCCGCTTCCACTTCAGAGGCACAGGATATGATGAGAAGATGGTGCGAGA
GATTGGAGGGCTGGAGGCCTCTGGGTCCACATACGTCTGCACTCTTTGTG
ACTCAAGTCGAACAGAGGCCTCTCAAAACATGGTTCTACACTGCATCACC
CGCAGTCATGAAGAGAACCTAGAACGTTATGAAATATGGAGAACCAACCC
CTTCTCTGAATCTGTGGATGAGCTGCGAGACAGAGTCAAAGGGGTCTCGG
CAAAGCCCTTCATGGAGACCCAGCCCACGCTAGACGCATTGCACTGTGAC
ATTGGCAATGCCACTGAGTTCTATAAAATCTTCCAAGATGAGATTGGCGA
GGTGTATCAAAAGGT---CAAT---CCCAGCAGGGAGGAACGGCGCAGCT
GGAGGGCAGCCCTAGATAAACAACTGAGGAAGAAGATGAAGCTCAAGCCG
GTAATGAGGATGAATGGGAACTATGCCCGCAGGCTAATGACCCTAGAGGC
TGTGGAGGTGGTGTGTGAGCTGGTGCCCTCAGAGGAGAGAAGGGAGGCCC
TAAGGGAGCTTATGCGGCTTTACCTCCAAATGAAGCCTGTGTGGCGTGCC
ACCTGCCCGGCCAAGGAGTGCCCCGACCAGCTGTGTCGCTACAGCTTTAA
CTCCCAGCGCTTTGCCGACATCCTCTCCTCTACTTTCAAATATAGATACA
ATGGAAAAATAACCAATTACCTGCACAAGACTCTGGCCCATGTGCCTGAA
ATAATAGAGAGAGATGGATCCATAGGAGCCTGGGCCAGTGAGGGTAATGA
GTCTGCAAACAAATCATACACCATCGAGATGGGTTCCGTAGGGCCTCAGT
GGAAGGAGAGCCCGCAGCCTTTCTCCTGCTCCATTGAAGACCCCACAAAA
CAGACAAAGTTCAAGGGCATCAAGACATACATTTCATACCGAGTCACACC
GAGCCACACAGGGCGTCCCGTTTACAGGCGTTACAAACACTTTGACTGGC
TGTACAACCGCTTACTGCACAAGTTCACTGTGATCTCTGTGCCTCACCTG
CCTGAGAAGCAGGCGACGGGGCGATTTGAGGAAGATTTCATCGAGAAGCG
TAAGAGGCGATTGATACTATGGATGAACCACATGACCAGCCATCCAGTCC
TCTCCCAGTATGAAGGCTTTGAGCACTTTCTGATGTGTGCTGATGACAAA
CAGTGGAAACTGGGCAAGAGAAGGGCAGAGAAGGACGAGATGGTGGGTGC
TCATTTCATGCTGACCCTCCAGTTCCCTAAAGAGCACCAGGACCTTCAGG
ATGTAGAGGAGCGGGTCGACACCTTCAAGGCCTTTGCTAAGAAAATGGAT
GACAGTGTGATGCAGCTCATGCATGTTGCCTCTGAGCTGGTGCGTAAACA
CCTGGGTGGGTTCAGGAAGGAGTTCCAGCGGCTGGGAAATGCCTTCCAGT
CTATCAGCCAGGCCTTCATGCTGGACCCTCCCCACAGCTCAGAAGCCCTC
AACAGCGCCATCTCCCATNNNNNNNNCTACGTTCCTCAAACTGACCTCTCTGGGTTTC
ATCATTGGAGTTGGTGTGGTAGGAAACCTCCTGATCTCCATCCTCCTGGT
CAAAGACAAGAGTCTGCACCGAGCACCCTACTATTTCCTGCTAGACCTGT
GTGCTTCAGATATCCTGCGATCTGCGATCTGCTTCCCCTTTGTCTTCACA
TCAGTCAAGAATGGATCTGCCTGGACCTATGGCACGCTGACCTGCAAAGT
GATAGCCTTCCTGGGTGTGCTCTCCTGTTTCCATACAGCATTCATGTTAT
TCTGTGTCAGTGTCACCCGCTACCTGGCCATCGCACATCACCGTTTCTAC
ACCAAGAGGCTGACCTTCTGGACCTGTCTGGCTGTCATCTGCATGGTATG
GACATTGTCAGTGGCTATGGCATTTCCACCAGTGTTAGACGTAGGGACAT
ACTCTTTTATCCGGGAGGAGGACCAGTGCACATTCCAGCACCGTTCCTTC
AGGGCAAATGATTCTCTGGGCTTCATGCTCCTGCTGGCGCTCATTCTCTT
GGCTACACAGCTGGTTTACCTCAAGCTCATCTTCTTTGTTCATGACCGCC
GAAAGATGAAGCCTGTCCAGTTCGTGCCTGCTGTCAGCCAGAACTGGACC

TTCCACGGACCAGGTGCCAGTGGTCAGGCGGCAGCCAACTGGCTGGCAGG
ATTTGGTCGAGGCCCCACCCCGCCTACTTTGTTAGGCATTCGGCAAAACA
GCAATGCAGCGGGTCGCAGGCGTCTATTGGTATTGGATGAATTCAAAACA
GAGAAGAGGATTAGTAGGATGTTCTACATCATGACATTTTTCTTCCTGGC
GCTGTGGGGGCCCTATCTAGTCGCCTGTTACTGGCGGGTGTTTGCACGGG
GCCCAGTAGTCCCTGGGGGCTACCTAACAGCAGCTGTTTGGATGAGCTTT
GCCCAGGCTGGGGTCAATCCTTTTATCTGCNNNNNNNNNNNNNNNNNNNGCCAAATCTCGCTTTCACCC
TGGCGTGGGGACTGGTCCTGGCACGGAGC---GCAGCGTCCCACTCGGCA
ACAGCTTGCTATCCCCGCAGCAAACCGAGGAGCCCACTGTTGCCACCCCC
CCGCAGCGATGGTTTGTCACCC---CTGCCAACAACCGACTGGACTTTGC
TGCCTCGGCATACGACGCCGCT---------GATTTCGCCGGTAACGCAG
CCACCTTACTGTCCTACGCAGCGGCCGGAGTGAAGGCTC------TTCCC
CTGCCGACTGCAGGCTGCTCCAACCGGCCTCTTGGCTATTACGCAGACCC
ATCRG---GCTGG---GGAGGACGCACGCCGCCGCAATACTGCGGCGTAA
ATAGCAAATCTAGCTCGGTCTTTTCTTGCTGGCCCGCCAACTCAATCGGC
AGCAGAGCGGCCA---CC---AACTACCTGG---------CCGAGGA---
GGGT---GACTC---CATCACGACMGAGAGGTCACCG---AT---CGGCG
TCTCGGAGGAG---ACCAAACCCAAAGACATGAC---ATCAGA---GTCG
AGCTGGATAGAA---ACGCCCTCCTCCATTAAATCAATCGATTCAAGCGA
TTCCGGTATTTTTG---AACAGGCCAAAAGGAGAAGAATCTCACCTTCTG
CCACGCCG---------GTTTCAGAGACAGTGTCCCCGTTAAAATCTGAG
CATCACTCAACAGGAGAAGTCACAGAGAGAGAAGTGGCGCTGGAGATAAA
TCCGTTAGCGGATGGGATGGGCGCCTTTAAAATAAACCACAGCTCCCATG
ATATTGGCTCCGG---GCAGACGGCGTTTTCCTCCCAGGCG---CCGGGC
TAC---GCAGCAGCCGCCCTGGGA---CACCATCA------CCACCCGAC
CCACGTTGGCTCT---TACTCCACGGCAGCTTTCAACTCCACCAGGGACT
TCCTCTTCAGAAATCGGGGTTTCGGAGATGCCACCGG------------G
GCGCAGCACAGTTTGTTCGCCTC---------CGGAAGTTT---C-----
-GCAGGGCCACATGGACACTCAGATGCAGCGGGGCACCTGCTCTTCCCAG
GGCTCCACGAG---CAAGCGGCGAGCCATGCGTCTTCCAACGTCGTCAAC
AGCCAGATGCGACTGGGCTTCTCTGGGGACATGTACGGACGGGCCGACCA
GTACGGCCACGTTACAAGCCCACGGT---CCGACCACTATGCTTCGACCC
AGCTGCACGGCTATGGCCCCATGAACATGAATATGGCCGCA---CACCAC
GGAGCAGGGGCCTTCTTTCGATACATGAGGCAGCCGATCAAACAAGAGCT
CATCTGCAAGTGGATCGAACCGGAGCAGCTGACAAATCCCAAAAAGTCGT
GCAACAAAACTTTTAGCACCATGCACGAGCTTGTGACCCATCTGACGGTG
GAGCATGTGGGGGGACCAGAGCAGACCAACCACATCTGCTTCTGGGAGGA
CTGCTCCAGAGAAGGGAAGCCATTCAAAGCCAAATACAAACTTGTGAATC
ATATCAGAGTACACACCGGCGAAAAGCCGTTTCCGTGTCCGTTCCCCGGC
TGTGGCAAA
>Morone chrysops
AGTCTTCTTATTCGAGCAGAGCTAAGCCAACCGGGCGCCCTCCTTGGGGA
TGACCAAATCTACAACGTAATCGTTACCGCACATGCATTTGTAATGATCT
TTTTTATAGTAATACCAATCATGATCGGAGGATTCGGAAACTGACTTATT
CCACTAATAATTGGAGCACCTGATATAGCATTCCCCCGAATGAACAACAT
AAGTTTTTGACTACTTCCCCCATCCTTCCTTCTCCTCTTGGCTTCTTCAG
GCGTCGAAGCCGGGGCTGGGACTGGCTGAACCGTCTATCCCCCCCTTGCA
AGCAACCTTGCACACGCAGGTGCATCTGTAGACTTAACAATTTTTTCTCT
CCACCTGGCTGGGATCTCCTCAATTCTGGGGGCCATTAATTTCATCACAA
CTATTATTAACATGAAGCCTCCCGCTATTTCCCAGTACCAAACCCCCTTA
TTTGTCTGAGCTGTTCTAATTACAGCCGTCCTCTTACTTCTCTCTCTGCC
AGTCCTCGCAGCCGGAATTACAATACTACTTACAGACCGAAATCTAAACA
CCACCTTCTTTGACCCTGCCGGGGGAGGGGACCCCATCCTCTACCAACAC

CTC---

-------------TTCTTAGAGAGAAACCTTCACCCATCTAACTGTCTTGG
CATGCTGTTGCTGTCTGATGCCCACCAGTGTACCAAGCTGTCAGAGCTTT
CCTGGGGCATGTGCCTCAGCAACTTTCCTGCTATTTGCAAGACAGAGGAC
TTCCTCCAACTGCCCAAAGATATGGTAGTGCAGCTCTTGTCACATGAGGA
GCTAGAGACAGAAGATGAGAGACTGGTTTATGAAGCTGCCCTCAACTGGA
TCAACTATGACCTAGAAAAGAGGCACTGTCACCTTCCAGAGCTCCTGAGA
ACAGTCCGTCTGGCCCTACTGCCTGCCATCTTTCTCATGGAGAACGTTTC
TACAGAAGAGTTGATCAACGCCCAGGCCAAGAGCAAGGAGCTAGTGGATG
AAGCTATCCGCTGTAAGCTGAAGATACTACAGAATGATGGCGTTGTTAAC
AGCCCCTGTGCTCGACCAAGAAAAACCAGCCATGCCCTTTTTCTTCTGGG
TGGGCAGACTTTCATGTGTGACAAGTTGTACCTAGTGGATCAGAAAGCCA
AAGAGATAATCCCCAAGGCAGACATTCCCAGCCCCAGGAAGGAGTTCAGC
GCCTGCGCCATCGGCTGTAAGGTGTACATCACCGGTGGAA--GAGGCTC-
AGAGAATGGTGTTTCCAAAGATGTATGGGTCTATGACACCGTCCATGAGG
AATGGTCGAAGGCAGCACCCATGCTCATTGCCAGGTTTGGCCACGGCTCT
GCGGAGCTGAAGCACTGCCTGTATGTAGTAGGAGGTCACACCGCAGCAAC
TGGCTGCCTCCCGGCCTCTCCGTCCGGATGAATACATTGTCGTGTTTAGT
CGTTCAACAACAAGGCTGATACTGAATGAAGCAGAGCTGATCATGGCGCT
GGCCCAGGAGTTCCAGATGAGAGTGGTCACAGTATCCTTGGAGGAGCAGT
CTTTCCCCAGTATCGTCCAGGTGATAAGCGGTGCCACCATGTTAGTCAGC
ATGCATGGAGCTCAGCTTATCACCTCACTCTTCCTCCCCAGGGGAGCTGC
TGTGGTAGAACTATTCCCCTTTGCAGTGAACCCAGAGCAGTACACCCCAT
ATAAAACCCTTGCCTCCCTTCCAGGCATGGACCTTCACTATGTCTCCTGG
AGGAACACTAGGGAGGAGAACACCATCACCCATCCAGACAGACCCTGGGA
ACAAGGGGGCATCGTTCACTTGGAGAAGGAGGAACAAGAGCGAATACTGG
CGAGCAAAGATGTCCCCAGGCACCTGTGCTGCCGCAACCCAGAGTGGCTC
TTCCGGATCTACCAGGACACTTTGGTGGACATCCCTTCTTTCCTGGAGGT
CCTCAA---AGAGGGCATGAAG---ACAAAGCCCAGCTTGAAGAA---AT
CAAAGCCAGCCAGCACAGTCCACCCGGGCCGGGTCAGAGAACCCCACTGT
CACACCTCAGTACAAACCACTAATGAGGCTAAACTCACGGTCTCCTGGCA
GATCCCCTGGAATCTGAAATACCTGAAGGTGAGAGAGGTGAAGTACGANNNNNNNNNNNNNNAA
AAAAGACACCAGCAAGGGAACACTGGAGGATCAAATCATCCAGGCGAACC
CTGCGTTGGAGGCCTTCGGCAACGCCAAAACGTTGAGAAACGACAACTCG
TCTCGTTTTGGAAAATTCATCCGGATTCACTTCGGTACGAGTGGCAAGCT
GTCGTCTGCTGACATAGAGACGTACCTGCTGGAGAAGTCACGTGTCACCT
TTCAGCTCAAGGCTGAGAGGAACTACCACATCTTCTACCAGATCCTGTCC
AATCAGAAGCCAGAGCTGCTGGACATGCTGCTTATCACCAACAACCCGTA
CGACTACTCCTACATCTCCCAAGGAGAGGTAACGGTTGCCTCCATCAACG
ACTCAGAGGAGTTGATGGCCACTGACAGTGCCTTCGATGTGCTCGGCTTC
ACTCCAGATGAGAAGATGGGTGTCTATAAACTGACGGGCGCCATAATGCA
CTACGGCAATATGAAGTTCAAACAGAAGCAGCGTGAGGAGCAGGCAGAGC
CCGATGGGACAGAGGCTGCGGATAAAACAGCCTACCTAATGGGGCTGAAC
TCTGCTGACCTCATCAAAGGGTTGTGCCATCCCAGAGTCAAGGTAGGAAA
TGAATATGTCACCAAAGGCCAAAGTGTGGACCAAGTCTACTACCCCAACA
AGGAGGCCTTCAAGTGTGAGGAGTGTGGGAAGCACTACAACACCAAGCTG
GGATATAAGCGCCATGTGGCCATGCACTCTGCCACAGCAGGGGATCTCAC
CTGTAAAGTATGCATGCAGACGTACGAGAGCACGCCTGTGCTGTTGGAGC
ACCTCAAGAGCCACTCCGGAAAGTCTTCAGGAGGCACCAAGGAGAAGAAA
CACCCGTGCGACCACTGTGACCGCCGTTTCTACACACGGAAGGATGTGAG

ACGGCACATGGTGGTGCACACAGGCCGAAAGGACTTCCTGTGCCAGTACT
GTGCCCAGCGCTTCGGCAGGAAGGATCATCTGACACGCCATGTGAAGAAG
AGCCACTCGCAGGAGCTGCTGAAGATCAAGACGGAGCCTCCTGATATGTT
AGGTCTTTTAGCTTCCGGGTCACCACCTTGCTCTGTGAAGGAGGAGCTCA
GCCCCATGATGTGCAGCATGGGTCCCAACAAAGACCCCATGATGGGCAAA
CCGTTCCCTAGTGGGGCCCCTTTTCCGATGGGCATGTACAACCCCCACCA
T------CTCCAGGCCATGTCTAATTCTGGGGTGGGTCACCCA------C
ACCCATCCCTGATGCCCACTTCCTTGTCTGCAGCTATGGGCATGGGCTGT
CACATGGAATATCTCATCTATGCCTCATTCTCATTCATGGGATGTTTACA
AATCAGTGATGGATCAAATATTGTGAACCTGCTGGCTAGTAACTCTCCAA
GTGTTTCATACGCTCTGACCCAGCAGAAATACTTCAGTAACTATAGTCCC
GTGATTGGGTTTTACATTTACGAGCCCATTGAATACTGGAACTCCACGGT
GCAGGAGCACCTGAAGACTNTGAGTCATGGCTTCAACAAGATCTCCTGGAT
GGACAACTTTTTCCACTACCTGCGGGTGGTGAATGTGAGTGCGTCAACAA
AGAATGACTTCATCACCATCCTCAAGGGCTCCTTCCTGCGTAGCCCGGAG
TACCAGCACTTCACTGAGGACATCATATTCTCCAAGA---ACCGTGAGAC
TG------ATGAGTACGATATTATTGCCTCACGGATGTACTTGGTGGCAC
GGACGACAGAGAAGAAGCGCGAAGAAGTGGTGGAGCTTCTGGAAAAGCTT
CGACCGTTGATGCTGATCAACAGCATCAAGTTCATTGCCTTCAACCCTAC
GTTTGTGTTCTTGGACCGCTACAGCTCCTCTGTCATCTCGCCCATCCTGA
CCTCAGGATTCAGCGTACTCACAATCCTCATCCTCACTTTCTTCCTGGTC
ATCAACCCCTTGGGGAACTTCTGGCTCATCCTTACTGTAACGTCCGTGGA
GCTGGGCGTCTTGGGTTTGANNGGCTTTCACCAGTTTGAATGGCAGCCAGCT
CTCAAGAATGTGTCTACATCTTGCAATGTTGGCATTATTAATGGGCTCTC
TGGATGGGCTTCCTCAGTGGATGAGTCCCCAGCTGACACCATCACTCGGC
GGTTTCGCTATGATGTGGCACTGGTGTCAGCATTAAAGGATCTGGAGGAG
GACATCATGGAGGGGCTGAGAGAGAGTGGGATGGAAGACAGCGCTTGCAC
CTCAGGCTTCAGTGTCATGATCAAGGAATCTTGTGATGGCATGGGAGATG
TCAGCGAGAAGCATGGTGGAGGACCAGTTGTTCCTGAAAAGGCTGTACGT
TTCTCTTTCACTGTTATGTCTGTCTCTGTCCTGGCAGACGATGAGGAG--
----------------------------GAAGAGGTTACCATCTTCACGG
AGCCAAAGCCAAACTCAGAACTGTCCTGTAAGCCCCTTTGCCTGATGTTT
GTGGATGAGTCAGACCATGAGACACTCACATCCGTCCTGTGGCCTATAGT
TGCAGAGCGTAATTCCATGAAAGAGAGTAGGCTCATCCTATCTATGGGTG
GACTACCTCGCTCCTTCCGCTTCCACTTCAGAGGCACGGGATATGATGAA
AAGATGGTGCGTGAGATGGAGGGCCTTGAAGCATCAGGGTCCACCCATAT
CTGCACTCTTTGTGACTCCAGTCGGGCAGAGGCATCTCAAAACATGGTTC
TACACTCCATCACCCGCAATCATGAAGAGAACCTAGAACGTTATGAAATT
TGGAGAACCAACCCCTTTTCTGAGTCTGTGGATGAGCTGCGAGACAGAGT
CAAAGGGGTCTCTGCCAAGCCCTTCATGGAGACTCATCCCACGCTGGATG
CATTACACTGTGACATAGGCAATGCCACTGAGTTCTACAAAATCTTCCAA
GATGAGATCGGGGAGGTGTTCAAAAAGGT---CAAC---CCCACCCGGGA
GGAACGGCGCAGCTGGAGGGCAGCCCTAGATAAACAACTGAGGAAGAAGA
TGAAGCTTAAACCGGTAATGAGGATGAATGGGAACTATGCCCGCAGGCTA
ATGACCCTGGAGGCTGTGGAGGTGGTGTGTGAGCTGGTGCCCTCAGAGGA
GAGGAGGGAGGCCCTGAGGGAGCTTATAAGGCTCTACCTACAGATGAAGC
CTGTGTGGCGTGCCACCTGCCCAGCCAAGGAGTGCCCTGACCAGCTGTGC
CGCTACAGCTTTAACTCCCAGCGCTTTGCCGACCTCCTCTCCTCTACTTT
CAAATATAGGTACAATGGAAAGATAACCAATTACCTGCACAAGACCCTGG
CCCATGTGCCTGAAATCATAGAGAGAGAYGGATCCATAGGAGCATGGGCC
AGCGAGGGGAACGAGTCAGCAAACAAANNNNNNNNNNNNNNNNTGGGTCCCATGGGGCCCCTGTGG
AAGGATAGCCCACAGCCTTTCTCCTGCTCCATTGAAGACCCCACTAAGCA
GACAAAGTTCAAGGGCATCAAGACGTACATTTCGTACCGGGTGACGCCGA

GCCACACAGGGCGTCCTGTCTACAGGCGCTACAAACACTTTGACTGGCTG
TACAACCGCTTACTGCACAAGTTCACTGTGATCTCCGTGCCTCACTTGCC
CGAGAAGCAGGCCACGGGGCGATTTGAGGAAGATTTCATTGAGAAGCGTA
AGAGGCGACTGATACTGTGGATGAACCACATGACCAGTCACCCAGTCCTC
TCCCAGTATGAAGGCTTCGAGCACTTTCTGATGTGTGCTGATGACAAACA
GTGGAAACTGGGCAAGAGACGGGCTGAGAAGGACGAAATGGTGGGTGCCC
ATTTCATGCTGACCCTCCAGATCCCTAATGAGCACCAGGACCTTCAGGAT
GTAGAGGAGAGGATCGACTCCTTCAAATCCTTTGCTAAGAAAATGGATGA
CAGCGTGATGCAACTCACACATGTTGCCTCGGAGCTGGTTCGTAAGCACC
TGGGAGGGTTCAGAAAGGAGTTCCAGCGGCTGGGAAATGCCTTCCAGTCT
ATCAGCCAGGCGTTCGCGCTGGACCCTCCACACAGCTCAGAGACCTTCAA
CAACGCCATCTCCCATNNNNNNNNNNNGTTCCTCAAACTGACCTCTCTGGGTTTCATCATT
GGAGTCGGTGTGGTTGGAAACCTCCTGATCTCCATCCTGCTGGTCAAAGA
CAAGAGCCTGCACCGAGCGCCCTACTATTTCCTGCTGGACCTGTGCGCCT
CTGATATCCTGCGCTCCGCCATCTGCTTCCCCTTTGTCTTCACCTCGGTC
AAGAATGGATCTGCATGGACCTATGGCACGCTAACCTGCAAAGTGATCGC
CTTCCTGGGTGTGCTCTCCTGTTTCCACACGGCGTTTATGCTATTCTGTG
TCAGTGTCACACGTTACCTGGCCATCGCACACCACCGTTTCTACACCAAG
AGGCTGACCTTCTGGACCTGTCTAGCTGTCATCTGCATGGTGTGGACATT
GTCAGTGGCTATGGCTTTCCCACCGGTGCTAGACGTAGGGACGTACTCTT
TTATCCGGGAGGAGGACCAGTGCACGTTCCAGCACCGTTCCTTCAGGGCG
AATGATTCACTGGGCTTCATGCTCCTGCTGGCTCTCATCCTCCTGGCCAC
ACAGCTGGTTTACCTCAAGCTCATCTTTTTCGTCCACGATCGTCGAAAGA
TGAAGCCTGTCCAGTTCGTGCCTGCTGTCAGCCAGAACTGGACCTTCCAC
GGGCCAGGCGCCAGTGGGCAGGCGGCGGCCAACTGGCTGGCTGGATTCGG
CCGAGGACCCACCCCACCTACCTTGCTCGGCATCCGGCAGAACAGCAACG
CAGCAGGCCGCAGGCGTCTACTGGTATTGGATGAATTCAAAACAGAGAAG
AGGATTAGTAGGATGTTCTACATCATGACGTTTTTCTTCCTGGCACTGTG
GGGGCCCTATCTGGTCGCCTGCTACTGGCGGGTGTTTGCAAGGGGCCCTG
TGGTCCCTGGGGGCTACCTGACGGCAGCCGTGTGGATGAGCTTTGCCCAA
GCTGGGGTCAATCCTTTCATCTGCATCTTCTCCNNNNNNNNNGCCAAATCTCGCTTTCA
CCCTGGCATGGGGACTGGTCCTGGCACGGAGC---GCAGCGTCCCACTCG
GCAACAGCTTGCTATCCCCGCAGCAAAACGAGGAGTCCACTGTTGCCACC
CCCCCGCAGCGATGGTTTGTCACCC---CTGCCAACAACCGACTGGACTT
TGCTGCCTCGGCATACGACGCTGCT---------GATTTCGCCGGTAACG
CGGCCACCTTGCTGTCCTACGCAGCGGCCGGAGTGAAGGCTC------TT
CCCCTGCCGACTGCAGGCTGCTCCAACCGGCCTCTTGGCTATTACGCAGA
CCCGTCGG---GCTGG---GGAGGACGCACGCCGCCGCAGTACTGTGGTG
TAAATAGCAAATCCAGCTCGGTCTTTTCCTGCTGGCCCGCTAACTCTCTC
GGTGGCAGAACGGGCA---CC---AACTACCTGG---------CTGAGGA
---GGTA---GACTC---CATCCCGACAGAGAGGTCACCG---AT---CG
GCGGCTCGGAGGAG---GCCAAACCCAAAGACATGAC---ATCAGA---G
TCGAACTGGATAGAG---ACGCCGTCCTCCATTAAGTCAATTGATTCAAG
CGATTCTGGTATCTTTG---AACAGGCCAAGAGGAGAAGAATCTCACCTT
CTGCCACGCCG---------GTTTCAGAGACAGTGTCCCCATTAAANNNNNNNCATC
ACTCAACAGGCGAAGTCACAGAGAGAGAAGTGGCGTTGGGGATAAATCCG
TTCGCTGATGGGATGGGCGCCTTCAAAATAAACCACAGCTCCCATGATAT
TGGCTCCGG---ACAAACGGCGTTTTCCTCCCAGGCG---CCCGGCTAC-
--GCAGCAGCCGCCCTGGGA---CACCATCA------CCACCCGACCCAC
GTTGGCTCT---TACTCCACGGCGGCTTTCAACTCCACCAGGGACTTTCT
CTTCAGAAATCGGGGTTTCGGGGACGCCACCGG------------GGCGC
AGCACAGTTTGTTCGCCTC---------CGGAAGTTT---C------GCA
GGGCCACATGGACACTCAGATGCAGCGGGGCACCTGCTCTTCCCGGGGCT

CCACGAG---CAAGCGGCGAGCCATGCGTCTTCCAACGTGGTCAACAGCC
AGATGCGGCTGGGCTTCTCGGGGGACATGTACGGACGGGCCGACCAGTAC
GGCCACGTTACGAGCCCGCGGT---CCGACCACTACGCATCGACCCAGCT
GCACGGCTACGGCCCCATGAACATGAATATGGCCGCA---CACCACGGAG
CCGGGGCCTTCTTTCGATACATGAGGCAGCCGATCAAGCAAGAGCTCATC
TGTAAGTGGGTCGAGCCGGAGCAGCTGACCAATCCCAAAAAGTCGTGCAA
CAAAACTTTTAGCACGATGCACGAGCTCGTGACCCATCTGACGGTGGAGC
ATGTGGGGGGACCGGAGCAGACCAACCACATCTGCTTCTGGGAGGAATGC
GCCAGAGAAGGAAAGCCATTCAAAGCCAAATACAAACTTGTAAATCATAT
CAGAGTACACACCGGAGAAAAGCCCTTTCCGTGTCCGTTCCCCGGCTGTG
GCAAA
>Muraenolepis microps

-------------------------GGAGGATTACATTGTGGTCTTCTCT
CGTTCCACAACCAGGTTGATCCTAAATGAAGCAGAGCTGATCATGGCGCT
GGCCCAGGAGTTTCAGATGAGAGTCTTAACGGTGTCCCTGGAGGAGCAGT
CGTTTGCCAGCATAGTGCAGGTGATCAGCGGCGCCTCCATGCTGTTCAGT
ATGCACGGTGCTCAGCTTGTCACGTCACTGTTTCTGCCTCGAGGTGCAGC
TGTGGTGGAGCTGTTCCCCTATGCCGTTAACCCAGAGCAGTACACCCCGT
ATAAAACCCTAGCCTCCCTGCCGGGCATGGACCTCCAATATGTGTCCTGG
AGGAATACCATGGAGGAGAACACGGTCCCTCATCCGGACCGGCCCTGGGA
CCAAGGGGGCATCGTGCACTTGGAGAAGGAGGAACAAGAGCGCATACAAA

CCAGCAAGGATGTCCCCAGGCACCTGTGCTGTCGTAACCCAGAGTGGCTT
TACAGAATCTACCAGGACACTTTGGTGGACATCCCGTCACTCCTGGAGGT
GCTCAA---GGAGGGCCTCAAA---ACCAGGCCCAACTTGAAAAG---GA
GCAAGGCGGCCAGCACAGTTCACCCGGGACGGGTCAGAGAACCCCAGTGC
CGAACCTCGGTCCAAGCCACCAACGAGGCCAAACTCACCGTCTCCTGGCA
GATCCCGTGGAACCTGAAGTTCCTGAAGGTGCGAGAGGTGAAGTATGAGG
TG---------AAGAAGGACCCCAGCAAAGGAACTCTGGAGGATCAAATC
ATCCAGGCCAACCCTGCCCTGGAGGCCTTTGGTAACGCCAAAACCCTGAG
AAATGACAACTCCTCCCGTTTTGGAAAGTTCATCCGGATTCACTTTGGGA
ACAGCGGCAAGCTGAGCTCTGCGGACATCGAGACCTACCTGCTGGAAAAG
TCCAGGGTCACTTTCCAGCTCAAGTCAGAGAGGAACTATCACATATTCTT
CCAGATCTTGTCCAAGCAGAAGCCGGAGCTGCTGGACATGATGCTGATAA
CCAACAACCCGTATGATTACTCCTACATCTCCCAAGGAGAGGTAACCGTA
GCCTCCATCAATGACTCTGAGGAGCTCATGGCCACCGACAGTGCATTTGA
TGTCCTTGGCTTCACCCTGGAAGAGAAGATGGGGGTGTACAAGCTGACTG
GTGCCATCATGCACTTCGGAAACATGAGGTTCAAGCAGAAGCAGCGCGAG
GAGCAGGCTGAGTCAGACGGGACTGAAGCAGCTGACAAATCAGCGTACCT
CATGGGTTTGAACTCTGCAGACCTCATCAAAGGGCTCTGCCACCCCAGGG
TGAAGGTAGGAAATGAATATGTTACCAAAGGTCAAGGGGTTGACCAAGTC
TACTAC--

----------------------TACCTCATCTACGCCTCCTTCTCCTTCA
TGGGGTGCCTGCAGATCAGCGACGGCTCCAACGTGGTCAACCTGCTGGCA
AGCAACTCGCCGAGCGTGTCGTACGCGCTGACGCAGCAGAAGTACTTCAG
CAACTACAGCCCCGTCATCGGCTTCTACATCTACGAGCCCATCGAGTACT
GGAACGCCACGGTGCAGGAGCACCTGAAGACCCTCAGCCACGGCTTCAAC
AAGATCTCCTGGATGGACAACTTCTACCACTACCTGCGCGTGGCCAACGT
CAGCGCGTCCACCAAGAACGATTTCATCCAGATCCTGAAGGGCTCCTTCC
TGCGCAGCCCGGAGTACCAGCACTTCACCGAGGACATCATCTTCTCCCGG
A---ACCGTGAGAACA------ACGAGTACGACATCATCGCCTCGCGCAT
GTACCTGGTGGCCCGCACCACCGAGAAGAAGCGCGAGGAGGTGGTGGAGC
TGCTGGAGAAGCTGCGGCCGCTCATGCTCATCAACAGCATCAAGTTCATC
GCCTTCAACCCCACCTTCGTGTTCATGGACCGCTACAGCTCCTCCGTCAT
CTCGCCCATCCTCACCTCGGGCTTCAGCGTGCTCACCGTCCTCATCCTCA
CCTTCTTCCTGGTCATCAACCCCCTGGGGAACCTGTGGCTCATCCTGACG
GTGACCTCGGTGGAGCTGGGCGTGCTGGGCCTGATGNNNNNNNNNNNNNNNNNNNNNNNNNNNNNNNNNN
NNNNNNNNNNNNNNNNNNNNNNNNNNNNNNNNNNNNNNNNNNNNNNNNNNNNNNNNNNNNNNNNNNNNNN
NNNNNNNNNNNNNNNNNNNNNNNNNNNNNNNNNNNNNNNNNNNNNNNNNNNNNNNNNNNNNNNNNNNNNN
NNNNNNNNNNNNNNNNNNNNNCTGAGAGAGCGGGG
GCTGGAAGACAGCGCCTGTTCTTCAGGCTTCTCCGTCATGATAAAAGAGT
CCTGCGATGGCATGGGAGATGTGAGTGAGAAGCACGGAGGGGGGCCTGCG
GTCCCGGAGAAAGTTGTACGGTTCTCTTTCGCCGTTATGTCTGTCTCTTT

GTGCGGAGCAGACGGAGAGGAG---------------------------G
AGGCTGTGATGATCTTCCAGGAGCCAAAGCCCAACTCTGAACTGTCCTGC
AAACCCCTGTGCCTCATGTTTGTCGACGAGGCAGACCACGAAACGCTCAC
AGCCATCCTGGGGCCCGTGGCGGCCGAGCGCAGTGCGATGAAACACAGCC
GGCTCGTTCTTTCCATCGGCGGGCTTCCCCGGTTCTTCAGCTTCCAGTTT
AGAGGCACAGGCTACGACGAGAAGATGGTGAGGGACGTGGAGGGCTTGGA
GGCCTCTGGCTCCACTTACGTCTGCACGCTTTGCGATTCCACCCGCGCTG
AAGCCTCCGACAACATGGTGCTTCACTCTGTGACCCGCAGCCACGAGGAG
AACCTGGACCGCTACGAGATCTGGAGGACCAACCCCTTCTCCGAGTCTGC
AGATGAATTAAGAGACCGAGTAAAGGGGGTCTCCGCCAAGCCCTTTCTGG
AGACTCAGATGACGCTGGACGCGCTACATTGTGACATCAGCAACGCCACA
GAGTTCTACAAAATCTTCCAGGATGAAATTGGTGAGGTGTTCCAGAAGAC
---CAAC---CCCACACGGGAAGAGCGGCGAAGCTGGAGGGCAGCCCTGG
ACAAGCAGCTGAGGAAGACGATGAAGCTCAAACCGGTGATGAGGATGAAC
GGCAATTACGCCCGAAAGCTGATGACCGTGGAGGCTGTGGATGCCGTGTG
CGAACTGGTTCCGTCCGAGGAGCGGCGGGCGGCCCTGAAGCAGCTCATGG
CGATCTACCTGGAGATGAAGCCAGTGTGGCGCGCCACAAGTCCAGCCAAG
GAGTGCCCTGACCAGCTGTGCCGCTACAGCTTCAACTCCCAGCGCTTTGC
CGATCTCCTCTCATCCACGTTCAAATACAGATACAATGGAAAGATCACCA
ATTACCTGCACAAAACCCTGGCACACGTGCCTGAGATCATAGAGAGAGAC
GGCTCTATCGGCGCTTGGGCCAGCGAGGAAAACGAGTCNNNNNNN---TCCTACACC
ATCGAGATCGGCCCCAAGGGCCCCCAGTGGAAGGAGAGCCCTCAGCCCTT
CTCCTGCTCCATCGAGGACCCCACCAAACAGACTAAGTTCAAGGGCATCA
AGACCTACATCTCGTACCGGGTGACGCCCAGCCACATCGACGCGGCCGTG
TACAGGCGCTACAAGCACTTTGACTGGCTGTACAACCGCCTGCTGCACAA
GTTCACCGTGATCTCCGTGCCCCACCTGCCCGAGAAGCAGGCCACGGGCC
GCTTCGAGGAGGACTTCATCGACAAGCGCAAGCGGCGGCTCATCCTGTGG
ATGAACCACATGACCAGCCACCCGGTGCTGTCACAGTACGAGGGCTTCGA
GCACTTCCTCATGTGCGCCGACGACAAGCAGTGGAAGCTGGGCAAGAGGC
GGGCAGAGAAGGACGAGATGGTGGGCGCCCACTTCATGCTCACCTTCCAG
ATCCCCAACGAGCACCAGGACCTGCAGGACGTGGAGGAGCGCGTGGACTC
CTTCAAGTGCTTCACCAAGAAGATGGACGACAGCGTCATGCAGCTGACGC
ATGGCGCCTCGGAGATGGTGCGCCGGCACCTGGGGGAGTTCCGGAAGGAG
TTCCACCGGCTCGGCAGTGCCTTCCAGTGCCTGTCGCAGGCCTTCATGCT
GGAGCCGCCGCACAGCTCGGAGCCCCTGAACAACGCCATCTCGCAC----

>Myctophum punctatum
AGCCTCCTTATCCGAGCCGAACTCAGCCAACCCGGCGCCCTCCTCGGAGA
CGATCAAATTTACAACGTAATTGTTACGGCTCACGCCTTTGTAATAATCT
TCTTTATAGTCATGCCCATCCTAATTGGAGGATTCGGAAACTGACTAATC
CCCCTGATGATTGGGGCCCCCGACATGGCATTCCCCCGAATGAATAATAT
GAGTTTCTGACTTCTCCCTCCTTCCTTCCTCCTGCTCCTAGCCTCCTCCG
GCGTAGAAGCGGGGGCAGGGACCGGCTGAACTGTATACCCACCCCTCGCG
GGAAACCTCGCCCACGCTGGAGCCTCAGTCGACTTAACAATTTTTTCGCT
CCACCTAGCAGGCGTGTCTTCAATTCTAGGCGCTATTAACTTTATTACAA
CTATCATTAACATAAAATCCCCTGCTATCTCACAGTACCAGACGCCCCTC
TTTGTCTGAGCCGTCCTAATCACCGCCGTACTCCTCCTTCTATCCCTCCC
TGTTCTAGCTGCTGGCATTACTATGCTCTTAACGGATCGAAACCTAAACA
CCACTTTCTTCGACCCAGCAGGGGGAGGAGACCCCATCCTTTACCAACAC
CTATTTTGATTCTTCGGGCACCCTGAAGTCTATATTCTTATCCTCCCCGG
GTTCGGAATAATTTCCCACATCATCGCCTACTACTCAGGTAAGAAAGAAC
CCTTCGGCTATATAGGCATGGTCTGAGCAATAATAGCTATCGGTTTCCTT
GGGTTTATTGTATGAGCCCACCACATATTTACTGTCGGAATAGACGTAGA
TACACGAGCATA--------------------------------------

-------------------------GGATGAGTACATCGTAGTGTTCAGT
CGCTCAACAACAAGACTCATTCTCAATGAAGCAGAACTGATTATGGCACT
GTCCCAGGAGTTTCAGATGAGGGTGGTCACTGTGTCCCTTGAAGAGCAAT
CTTTCCCCAGTATAGTCCAGCTTATCAGTGGTGCCTCAATGTTGGTCAGT
ATGCATGGGGCTCAGCTTGTCACCTCACTCTTCCTCCCCAGAGGAGCTGT
GGTGGTGGAGCTTTTCCCCTATGCTGTGAACCCAGAACAGTACACCCCTT
ATAAAACCCTCACCTCCTTACCGGGGATGGACCTGCAGTATGTTTCCTGG
AGGAATACGATGGAGGAAAACACCATCACCCACCCAGATAGATCCTGGGA
CCAAGGAGGCATCGCCCACCTCGAAAAGGATGAGCAAGAGCGAATCCTTG
CCAGCAAGGATGTCCCACGGCACCTGTGCTGCCACAACCCAGAATGGTTC
TTCCGAATCTATCAGGACACTCTGGTGGACATCCCTTCATTTCTGGAGGT
TCTCAA---AGATGGCCTGAAGATCACCAGACCAAACCTAAAAAA---GG
CCAAGCCAGCCAGCACAGTACATCCAGGCCGGGTCAGGGAAGCACAGTGT
CAAACCTCAGTCCAAGCCACCAACGAAGCTAAACTCACGGTCTCCTGGCA
GATCCCATGGAACCTGAAATACCTGAAGGTGAGGGAGGTGAAGTACGAGG
TGTGGATCCGGAAGAGGGACACGAGCAAGGGAACCCTGGAGGATCAAATC
ATCCAGGCCAACCCTGCACTGGAGGCCTTCGGGAACGCCAAGACCATTCG
CAACGACAACTCGTCGCGCTTTGGCAAGTTCATCCGCATCCACTTTGGGA
CGAGCGGCAAGCTGGCGTCCGCCGACGTTGAGACCTACCTGCTGGAGAAG
TCACGCATCACCTTCCAGCTCAAGGCCGAGCGAAACTACCACATCTTCTT
CCAGATCCTGTCCCAAGAGAAGCCGGAGCTGCTGGAGATGCTGCTGATCA
CCAACAACCCGTACGACTACAGCTACATCTCCCAGGGCGAGGTGACGGTG
TCGTCCATCAACGACAACGAGGAGCTGATCGCCACGGACAGCGCCTTCGA
CGTGCTCGAGTTCACCCCGGAGGAGAAGGCGTCCATCTACAAGCTGACGG
GCGCCATCATGCACTACGGCAACATGAGGTTCAAGCAGAAGCAGCGTGAG
GAGCAGGCCGAGCCCGACGGCACCGAGGCGGCCGACAAGGCGGCGTACCT
GATGGGGCTGAACTCGGCCGACCTCATCAAGGGACTCTGCCATCCCCGGG
TCAAGGTGGGTAATGAGTACGTCACAAAGGGCCAGGGTGTGGACCAAGTC
TACTACCCCAACAAGGAGGCCTTCAAGTGTGAGGAGTGTGGCAAGCACTA
CAATACCAAGCTGGGATACAAGCGCCATGTGGCCATGCACTCAGCCACAG
CGGGGGACCTCACCTGCAAGGTGTGCATGCAGAGCTATGAGAGCACGCCC
GTGCTCCTGGAACACCTCAAGAGCCACTCGGGGAAGTCGTCGGGAGGCGC
CAAGGAGAAGAAGCATCCGTGCGACCACTGCGACCGCCGCTTCTACACGC
GGAAGGACGTCCGGCGGCACATGGTGGTGCACACGGGACGGAAGGACTTC
CTGTGCCAGTACTGCGCACAGCGCTTCGGCCGGAAGGACCACCTGACGCG
CCACGTGAAGAAGAGCCACTCACAGGAGCTGCTAAAGATCAAGACGGAGC
CTCCGGACATGCTGGGCCTGCTGGGCTCTGGCTCGCCCCCCTGCTCCATC
AAGGAGGAGCTCAGCCCCATGATGTGCAGCATGGGTCCCAACAAGGACCC

CATGATGGGCAAACCCTTCCCCAGCGGGACCCCGTTCCCCATGGGCATGT
ACAACCCACACCAT------CTCCAGGCCATGTCCAGTCCAGGGGTGGGG
CACCCC------CACCCCTCCCTGATGCCCAGCTCCCTGTC---AGCCAT
GGGCATGGGCTGCCACATGGACTATCTGATCTACGCTTCCTTCTCCTTCA
TGGGATGTTTACAAATCAGCGATGGGTCAAACATCGTGAACCTGCTGGCA
AGTAACTCTCCGAGTGTGTCTTTTGCGCTCACCCAGCAGAAGTACTTCAG
TAACTACAGCCCTGTTATTGGGTTCTACATTTATGAACCCATTGAGTACT
GGAACGCCACGGTGCAGGAGCACCTGAAGACACTGAGTCACGGCTTCAAC
AAGATCTCCTGGATCGACAACTTTTTCCACTACCTGCGGGTGGTAAATGT
GAGTGCGTCGACCAAGAACGACTTCATCACCATCCTGAAGGGCTCCTTCC
TACGCAGCCCGGAGTACCAGCACTTCACTGAGGACATCATCTTCTCCAAG
A---ACCGCGAGAGCG------ACGAGTACGACATCATCGCCTCTCGCAT
GTACCTGGTGGCGCGCACCACAGAGAAGAAACGCGAGGAGGTGGTGGAGC
TGTTGGAGAAGCTGCGTCCGCTGATGCTGATCAACAGCATCAAATTCATC
GCCTTCAACCCCACCTTCGTCTTCATGGACCGCTACAGCTCCTCGGTCAT
ATCGCCCATCCTTACCTCAGGCTTCAGCGTGCTCACCATTCTCATCCTCA
CGTTCTTTCTTGTCATCAACCCACTGGGGAACTTCTGGCTCATCCTGACG
GTCACGTCTGTGGAGCTGGGCGTGTTGGGTCTGATG--------------

-------------------GCAA-----------------------TCCC
TGAGAAAGCAGTCCGTTTCTCTTTCACCATTATGTCCATCTCTGTGATGC
CGGATGAGAAAGCA------------------------------GAGGCG
GTTACCATCTTCACAGAGCCTAAGCCCAACTCCGAGCTATCCTGTAGGCC
CCTCTGCCTGATGTTTGTGGATGAGTCGGACCACGAGATGCTCACTGCCG
TCTTGGGGCCCGTCGTCGCAGAGCGGACTGCAATGACAGAAAGCCGACTT
ATCCTGTCCATGGGTGGCCTCCCCCGCTCATTCCGCTTCTACTTCAGAGG
CACAGGCTATGATGAGAAGATGGTACGCGAGATGGAGGGCCTGGAGGCTT
CAGGCTCTACCTATGTCTGCACTCTGTGTGACTCCACCCGGGCAGAGGCC
TCTCACAACATGGTGCTCCATTCTATCACCCGCAGCCACAGCGAGAACCT
GGAGCGCTACGAAATATGGCGGAGCAACCCCTTTTCGGAGTCTGTTGATG
AGCTGCGAGATCGGGTGAAGGGGGTCTCCGCCAAGCCCTTCATGGAAACC
CAGCCCACTCTGGATGCATTGCACTGCGACATTGGTAACGCCACTGAGTT
CTACAAAATCTTCCAGGATGAGATCGGAGAGGTGTTCCGGAAGGC---CA
AC---CCCAGCCGCGAGGAAAGGCGCCGCTGGAGGGCTGCCCTTGACAAG
CAGTTGAGGAAAAAACTCAAGCTCAAGCCGGTGATGCGGATGAATGGGAA
CTATGCTCGGAGGCTGATGTCCGAGGAGACTGTGGACGTGGTGTGTGAAC
TGGTGCCCTCAGAGGAAAGGCGGGAGGCTCTGAGGGAGCTAATGGGGC--

---TCCTACA
CCATCGAGATGGGCCCCAAAGGGCCCCTGTGGAAGGAGAGCCCGCAGCCC
TTCACCTGTTCCATCGAGGACCCCACTAAACAGACCAAGTTCAAGGGCAT
CAAGACGTACATCTCCTACCGGGTCACGCCAAGCCACACGGGTCACCCTG
TGTACAGACGCTACAAGCACTTTGACTGGCTGTACAATCGCCTGCTGCAC
AAGTTCACCGTGATCTCCGTCCCCCACCTGCCAGAGAAGCAGGCGACTGG
GCGCTTCGAGGAGGACTTCATAGAAAAGCGCAAGAGGCGGTTGATCCTCT
GGATAAACCACATGACCAGTCACCCTGTCCTCTCCCAGTATGAGGGGTTC

GAACACTTCCTGATGTGTGGCGACGACAAGCAATGGAAGCTGGGCAAGCG
GCGAGCAGAGAAGGACGAAATGATCGGTGCCCACTTCATGCTGACCTTCC
AGATCCCCAACGAGCACCAGGATCTGCAGGATGTGGAGGAGCGCGTAGAC
TCCTTCAAGGCGTTTGCCAAGAAGATGGACGACAGTATCATGCAGCTGAC
ACACGTGGCCTCGGAACTCGTGCGCAAGCACCTTGGTGGGTTCCGCAAGG
AGTTCCAGCGGCTGGGGAATGCCTTCCAGAACATCAGCCAGGGCTTCATG
CTTGACCCACCACATAGCTCAGACAGCCTCAACAACGCCATCTCACAC--

-----------------------------------GCCAAGTCCCGCTTT
CACCCTGGCGTGGGGAGCGCTGCTGGCACGGAGC---GCAGCGTCCCACT
CAGCAACAGCTTGCTCTCTCCGCAACAAACCGAGGATCCCGCCGTCG---
CCTCCCCGCAGCGATGGTTTGTCACCC---CTGCCAACAACCGACTGGAC
TTCGCAGCCTCGGCATACGACGCCGCT---------GATTTCGCCGGTAA
CGCGGCCACCTTGCTGTCGTACGCCGCGGCCGGAGTGAAGGCTC------
TCCCACTGCCGACCGCAGCGTGCTCCAACAGACCCCTGGGTTATTACGCG
GACCCGTCGG---GCTGG---GGCGCGCGCACGCCTCCCCAGTACTGT--
-------AGCAAGTCGACCTCGGTCCTTTCTTGCTGGCCCACGAATTCTG
TAGGGAGCAGAACGGGCA------CAAACTACCTAG---------TGGAG
GA---CGGATCGGACGC---CATCCCAACGGAGAGGTCCCCG---AT---
TAATGGCTCGGAGGAG---GCGAAAGCCAAAGACTT------GTCCGA--
-GWCTAGCTGGATAGAG---ACGCCATCTTCGATCAAATCAATTGACTCA
AGTGATTCTGGCATCTTTG---AGCAAGCAAAGCGGAGACGAATTTCACC
GTCTGCCACACCA---------GTAACAGAAACGGTGTCTCCGTTGAGAT
CTGAA---


```
--------------------------------------------------
--------------------------------------------------
--------------------------------------------------
--------------------------------------------------
--------------------------------------------------
--------------------------------------------------
--------------
>Nanobrachium lineatum
-------------------------GCCAACCCGGAGCCCTTCTGGGCGA
CGACCAAATTTATAATGTGATCGTGACAGCCCACGCTTTTGTAATGATCT
TCTTTATAGTAATGCCTATCATGATTGGAGGATTCGGAAACTGACTAATC
CCCCTAATGATCGGAGCCCCAGATATGGCATTCCCCCGTATAAATAATAT
GAGCTTCTGGCTCCTACCACCATCTTTCCTCCTTCTCCTAGCTTCATCAG
GCGTTGAAGCAGGGGCCGGTACTGGCTGAACAGTATATCCGCCGCTAGCA
GGCAACCTTGCCCACGCTGGGGCCTCTGTTGACCTAACAATCTTCTCACT
TCATCTAGCTGGTGTCTCATCAATTCTTGGTGCTATCAACTTTATCACAA
CGATCATCAACATGAAGCCCCCAGCAATTACTCAATACCAAACCCCTCTA
TTTGTCTGAGCCGTAATGATTACAGCCGTACTCCTTCTCCTTTCACTCCC
AGTCCTAGCCGCTGGAATTACTATGCTTCTAACAGACCGAAACTTAAATA
CCACCTTCTTTGACCCCGCTGGCGGGGGAGACCCCATCCTCTACCAACAC
CTATTCTGATTCTTCGGGCACCCCGAAGTCTATATTCTTATTCTTCCCGG
CTTCGGCATGATCTCGCACATTGTTGCTTACTACTCAGGTAAGAAAGAAC
CTTTCGGGTACATGGGCATGGTCTGAGCAATAATGGCCATTGGGTTCCTC
GGCTTTATCGTCTGAGCTCATCACATGTTCACCGTTGGAATAGACGTCGA
CGCACGAGCGTA--------------------------------------
--------------------------------------------------
--------------------------------------------------
--------------------------------------------------
--------------------------------------------------
--------------------------------------------------
--------------------------------------------------
--------------------------------------------------
--------------------------------------------------
--------------------------------------------------
--------------------------------------------------
--------------------------------------------------
--------------------------------------------------
--------------------------------------------------
--------------------------------------------------
--------------------------------------------------
-------------------------GGATGAGTACATTGTTGTGTTCAGT
CGCTCAACGACGAGACTCATTCTCAACGAAGCAGAACTTATTATGGCACT
GTCCCAGGAGTTTCAGATGAGGGTGGTCACTGTGTCCCTTGAAGAGCAAT
CTTTCCCCAGTATAGTCCAGCTTATCAGTGGTGCCTCCATGTTGGTCAGT
ATGCATGGGGCTCAGCTTGTCACCTCACTCTTCCTCCCCAGAGGAGCTGT
GGTGGTGGAGCTCTTTCCCTATGCCGTGAACCCAGAACAGTACACCCCTT
ATAAAACCCTCGCCTCCTTACCAGGGATGGACCTGCAGTATGTTTCCTGG
AGGAATACGATGGAGGAAAACACCATCACCCACCCAGATAGACCTTGGGA
CCAAGGTGGCATCGCCCACCTGGAAAAAGACGAGCAAGAGCGAATCCTTG
CCAGCAAGGATGTCCCGCGGCACCTGTGCTGCCACAACCCAGAATGGTTC
TTCAGAATCTACCAGGACACTCTGGTGGACATCCCTTCATTCCTGGAAGT
TCTCAA---AGATGGCCTGAAG---AGCAGACCAAGCTTAAAAAA---GG
CCAAGCCAGCGAGCACAGTACACCCAGGACGGGTCAGGGAAGCACAATGT
```

```
CAAACCTCGGTCCAAGCCACCAACGAAGCTAAACTCACAGTCTCCTGGCA
GATCCCATGGAACCTGAAG-------------------------------
--------------------------------------------------
--------------------------------------------------
--------------------------------------------------
--------------------------------------------------
--------------------------------------------------
--------------------------------------------------
--------------------------------------------------
--------------------------------------------------
--------------------------------------------------
--------------------------------------------------
--------------------------------------------------
--------------------------------------------------
--------------------------------------------------
--------------------------------------------------
--------------------------------------------------
--------------------------------------------------
--------------------------------------------------
--------------------------------------------------
--------------------------------------------------
--------------------------------------------------
--------------------------------------------------
--------------------------------------------------
--------------------------------------------------
--------------------------------------------------
--------------------------------------------------
--------------------------------------------------
----------------------TACCTGATCTACGCCTCCTTCTCCTTCA
TGGGATGTTTACAAATCAGCGACGGGTCGAACATCGTCAACCTGATGGCG
AGCAACTCTCCGAGCGTGGCTTTCGCGCTCACCCAGCAGAAGTACTTCAG
TAATTACAGTCCCGTTATCGGGTTCTACATTTATGAACCCATCGAGTACT
GGAATGCCACGGTGCAGGAGCACCTGAAGACACTGAGTCATGGCTTCAAC
AAGATCTCCTGGATCGACAACTTCTTTCACTACCTGCGGGTGGTCAATGT
GAGCGTGTCGACCAAGAACGACTTCATCACCATCCTGAAGGGCTCCTTCC
TGCGCAGCCCGGAGTACCAGCACTTCAACGAGGACATCATCTTCTCCAAG
A---ACCGCGAGAGTG------ACGAGTACGACATCATCGCCTCGCGCAT
GTACCTGGTGGCGCGCACCACCGAGAAGAAGCGCGAGGAGGTGGTGGAGC
TGCTGGAGAAGCTGCGTCCGCTGATGCTGATCAACAGCATCAAGTTCATC
GCCTTCAACCCCACCTTCGTCTTCATGGACCGCTACAGCTCCTCGGTCAT
ATCGCCCATCCTTACCTCAGGCTTCAGCGTGCTCACCATCCTCATCCTCA
CGTTCTTTCTCGTCATCAACCCGCTGGGGAACTTCTGGCTCATCCTGACG
GTCACGTCCGTGGAGCTGGGCGTGCTGGGTCTGATGGGCTACCACCCCTT
TGAGTGGCCTCCGGCCCTCCCGAGCGTGTCCCCCTCTNGCCGCGTGGAAT
CATCGTCGGGCTTTCTGGCTGGGCCGACTCGGTGGATGACTCGCCGGCGG
ACACCATAAGCCGGAGGTTTCGCTACGATGTGGCCCTTGTGTCTGCTTTG
AAGGACCTGGAGGAGGAAGTGATGGAGGGACTGAAAGAGTGCGGGCTAGA
TGACAGTGCATGCACTTCNGGCTTCAGCGTAATGATCAAAGAGTCCTGCGA
TGGCATGGGAGACGTCAGCGAGAAGCATGGCGGGGGGCCTGCGATCCCCG
AGAAAGCGGTCCGCTTCTCTTTCACGATTATGTCCATCTCTGTGACGCCG
GACGGGAAAGCG------------------------------GAGGCGGT
CACCATCTTCACGGAGCCGAAGCCCAACTCCGAGCTGTCCTGTAAGCCCA
TGTGCCTGATGTTTGTGGACGAGTCGGACCACGAGATGCTCACCGCCGTC
```

TTGGGGCCCGTGGTCGCAGAGCGGACGGCGATGACGGAAAGCCGACTCAT
CCTGTCCATGGGCGGCCTCCCCCGCTCGTTCCGCTTCTACTTCAGAGGCA
CAGGCTACGACGAGAAGATGGTGCGCGAGATGGAGGGCCTGGAGGCGTCA
GGCTCTACCTATGTCTGCACTCTGTGCGACTCCACCCGGGCGGAGGCCTC
CCACAACATGGTGCTCCATTCCATCACCCGCAGCCACAGCGAGAACCTGG
AGCGCTACGAGATATGGCGGAGCAACCCCTTCTCGGAGTCGGTCGAGGAG
CTGCGGGATCGGGTGAAGGGGGTCTCCGCCAAGCCCTTCATGGAAACCCA
GCCCACTCTGGATGCGTTGCACTGTGACATCGGTAACGCCACGGAGTTCT
ACAAAATCTTCCAGGATGAGATCGGGGAGGTGTTCCAGAAGGC---CAAC
---CCCAGCCGCGAGGAACGGAGACGCTGGAGGGCTGCCCTCGACAAGCA
GCTGAGGAAAAAACTCAAGCTCAAGCCGGTGATGCGGATGAACGGGAATT
ACGCCCGGAGGCTGATGTCCGAGGAGACCGTGGACGTGGTGTGCGAACTG
GTGCCCTCGGAGGAAAGACGGGAGGCTCTGAGGGAGCTCATGGGGCTTTA
CCTCCAGATGAAACCTGTGTGGCGCGCCAGCAACCCGGCCAAAGAATGCC
CTGACCAACTGTGCCGCTACAGTTTCAACTCCCAGCGCTTTGCCGACCTC
CTCTCCACCACCTTCAAATACAGGTATGACGGCAAGATTACCAATTACCT
GCACAAGACCCTGGCCCACGTCCCTGAAATCATAGAAAGAGATGGCTCTA
TTGGAGCGTGGGCCAGCGAGGGGAACGAGTCGGCGAACAAATCGTACACC
ATCGAGATGGGCCCCAAGGGGCCCCTGTGGAAGGAGAGCCCTCAGCCCTT
CTCCTGTTCCATTGAGGACCCCACTAAACAGACCAAGTTCAAGGGCATCA
AGACTTACATCTCCTACCGGGTCACACCGAGCCACACGGGTCACCCCGTG
TACAGACGCTACAAGCACTTTGACTGGCTGTACAACCGCTTGCTGCACAA
GTTCACCGTGATCTCCGTCCCCCACCTGCCGGAGAAGCAGGCGACCGGGC
GCTTTGAGGAGGACTTTATAGAAAAGCGCAAGAGGCGGCTGATCCTCTGG
ATAAACCACATGACCAGCCACCCTGTCCTCTCCCAGTATGAGGGCTTTGA
ACACTTCCTGATGTGTGGCGACGACAAGCAATGGAAGCTGGGCAAGCGGC
GAGCGGAGAAGGATGAAATGGTCGGTGCACACTTCATGCTGACCTTCCAG
ATCCCCAACGAGCACCAGGACCTCCAGGACGTGGAGGAGCGCGTTGACTC
CTTCAAGGCGTTTGCCAAGAAGATGGACGACAGTGTCATGCAGCTGACAC
ACGTAGCCTCGGAACTCGTGCGCAAGCACCTCGGTGGGTTCCGCAAGGAG
TTCCAGCGGCTGGGGAACGCCTTCCAGAACATCAGCCAGGCCTTCATGCT
CGACCCACCGCATAGCTCGGACGGCCTCAACAACGCCATCTCACAC----

------------TCAGGCGAAGTCACGGAGAGAGAAGTGGCGTTGGGGAT
CAACCCGTTCGCGGACGGGATGGGCGCCTTCAAGATCAACCACGGCTCCC
ACGACATCGGCTCCGG---ACAGACGGCGTTCTCCTCGCAGGCG---CCC
GGCTAC---GCGGCGGCCGCCCTGGGG---CACCACCA------CCACCC
GACCCACGTCAGCTCG---TACTCCACGGCAGCCTTCAACTCCACCCGGG
ACTTTCTGTTCAGAAATCGGGGCTTCGGAGACGCCACCAG---------C
GCCGCCCAGCACAGTTTGTTCGCCTCCGC---CGCGGGGAGTTT---C--
----GCGGGGCC-CATGGACACTCGGATGCCGCGGGGCACCTGCTCTTCC
CCGGGCTCCACGAA---CAAGCCGCGAGCCACGCGTCCTCCAACGTTGTC
AACGGCCAGATGCGGCTGGCTTTTTCGGGGGACATGTACGGCCGGGCCGA
CCAGTATGGCCACGTCACGAGCCCGCGGT---CCGACCACTACGCGTCGA
CCCAGTTGCACGGCTACGGCCCCATGAACATGAACATGGCCGCG---CAC
CACGGGGCCGGGGCCTTCTTTCGGTACATGCGGCAGCCGATCAAGCAGGA
GCTCATCTGCAAGTGGATCGAGCCGGAGCAGCTGTCGAACCCCAAGAAGG
CGTGCAACAAAACTTTCAGCACGATGCACGAGCTCGTCACCCATCTGACG
GTGGAGCATGTGGGGGGGCCCGAGCAGGCGAACCACATTTGCGTCTGGGA
GGACTGTGCTCGGGAGGGCAAGCCGTTCAAAGCCAAATATAAACTTGTGA
ATCATATCAGAGTACACACTGGAGAAAAACCCTTTCCGTGTCCCTTCCCC
GNNN--------
>Nansenia ardesiaca
AGCCTTTTAATCCGGGCCGAGCTAAGCCAACCGGGAGCCCTCCTGGGGGA
CGACCAAATTTATAATGTTATCGTAACAGCACATGCTTTCGTTATAATCT
TTTTTATAGTGATGCCAATCATGATCGGCGGCTTTGGAAACTGATTAATT
CCCCTAATAATCGGGGCCCCTGACATAGCCTTTCCCCGAATAAATAACAT
GAGCTTCTGACTTCTGCCCCCCTCTTTCCTTCTTCTCCTAGCTTCTTCCG
GGGTTGAAGCCGGAGCCGGGACAGGCTGAACAGTATACCCTCCTTTAGCA
GGAAACCTTGCTCACGCAGGGGCCTCCGTAGACCTAACCATCTTCTCCCT
CCATCTCGCGGGTATCTCCTCAATCCTCGGGGCCATTAACTTTATTACAA
CGATCATTAACATGAAACCCCCAACCATCTCCCAATACCAGACCCCCATA
TTCGTCTGAGCAGTACTTATTACAGCAGTCCTTCTGCTCCTCTCTCTTCC
CGTCCTAGCTGCCGGTATTACCATGCTACTGACAGACCGAAACTTAAATA
CCACTTTCTTTGACCCCGCAGGGGGAGGGGACCCCATCCTCTACCAGCAC
CTGTTCTGGTTCTTTGGCCACCCTGAAGTCTATATTTTAATTCTTCCTGG
CTTCGGTATAATCTCCCACATTGTTGCTTACTATTCCGGTAAAAAAGAAC
CTTTCGGCTACATAGGCATGGTTTGAGCAATGATGGCTATCGGGCTTCTA
GGCTTTATTGTCTGAGCCCACCACATGTTTACCGTCGGAATGGACGTAGA
CACTCGTGNNNN--

---------------------GGAGGAGTACATCGTGGTGTTCAGCCGTT
CCATTAACAGGCTGATTCTGAACGAGGCAGAGCTCATTCTGGCCCTAGCA
CAGGAGTTTCAGATGAAGGCTGTAACTGTGTCTCTGGAGGAGCAGTCCTT
TGCCAGTATTGTGAAGCTGCTCAGTGAGGCCTCTATGTTAGTCAGTATGC
ACGGAGCCCAACTGGTGTCCTCCCTCTTCCTTCCCAGGGGAGCCGCAGTG
GTCGAGCTCTTCCCCTATGCAGTGAATCCAGAGCAGTACGCTCCCTACAA
AACCTTGGCCTCACTCCCTGGCATGGACCTGCAGTATGTGGCCTGGAGGA
ACATGATAGAGGAGAACTCGGTGGCCTACCCCGAGAGGCCCTGGGAGCAG
GGAGGCATCGCCCATTTGGAGAAGGACGAGCAAGAGCGCATCCTGGCCAG
CAAAGAGGTACCAAGGCACCTGTGCTGCCGCAACCCAGAGTGGCTCTACC
GTATCTACCAAGACACCATAGTGGACATCCCTTCTTTACTGGAGACCCTC
AG---AGAGACCCTGAAA---ACCAGGCCAAACCTGAAGAA---GGCCAA
GCCTGCCAGCACGGTCCACCCAGGCCGAGTGAGAGAGCCCCAGTGCCAGA
CCTCGGTCCAGGCCACCAACGAGGCCAAGCTCACCGTGTCCTGGCAGATC
CCCTGGAACCTGAAATACCTGAAGGTCAGGGAGGTCAAATACGAAGTGNNNNNNNNNAA
GAGGGATCCAAGCAAGGGTACCCTAGAGGATCAAATCATCCAGGCTAACC
CTGCACTGGAGGCTTTCGGTAATGCCAAAACATTGAGAAATGATAATTCA
TCACGTTTTGGCAAATTCATTCGTATTCATTTCGGACAAAGTGGCAAATT
GTCCTCTGCTGACATAGAGACGTATCTGCTTGAGAAGTCACGTTGCACCT
TTCAGCTGAAATCGGAGAGAAACTACCATATCTTCTTCCAGATCTTGTCC
AATCAAAAGCCAGAGCTGTTGGACATGCTGTTGATCACCAACAATCCATA
TGACTACGCCTACATCTCCCAAGGAGAGGTGACAGTAGCATCCATCAATG
ATTCTGAGGAGTTGATTGCCACTGACAGTGCTTTTGATGTGCTGGGCTTT
ACTGCAGAGGAGAAAATGGCGGTCTACAAATTGACAGGTGCCATAATGCA
CTATGGCAACATGAAGTTCAAGAACAAGCAACGGGAGGAGCAGGCAGAGC
CTGACGGTACAGAGGCTGCTGACAAGTCAGCTTATCTAATGGGACTGAAC
TCTGCAGATCTTATTAAAGGATTGTGCCATCCGAGAGTCAAGGTTGGCAA
TGAGTATGTCACCAAAGGTCAGAGTGTCGATCAAGTCTACTAC-------

------------------------------TCGTACACCATCGAGATGGG
CTCCAAAGGTCCTCAATGGAAAGAGAGTCCCCAGCCTTTCTCTTGCTCCA
TCGAAGACCCCACCAAGCAGACAAAGTTTAAAGGCATCAAGACCTACATA
TCATACCGGGTAACCCCCAGCCACATGGGGCGGCCCGTGTACCGGCGCTA
CAAGCACTTTGACTGGCTGTACAATCGCCTGCTGCACAAGTTTACGGTCA
TATCGGTGCCCCACCTTCCTGAGAAACAGGCCACGGGGCGTTTCGAGGAG
GATTTCATTGAGAAGCGAAAAAGGCGATTGGTCCTCTGGATAAACCACAT
GACCAGCCACCCGGTCCTGTCCCAGTACGAAGGCTTCGAGCACTTCCTAA
TGTGTGCCGATGACAAGCAGTGGAAGCTGGGTAAACGGCGAGCGGAGAAG
GACGAGATGGTGGGCGCCCATTTCATGCTCACCTTCCAGATCCCCACGGA
GCACCAAGACCTTCAGGATGTGGAGGAGCGTGTGGACAACTTCAAGTCCT

TCGCCAAGAAAATGGATGACAGTGTCATGCAGCTGACGCACGTGGCATCA
GAACTGGTGCGGAAGCACCTCGGAGGATTCCGGAAGGAGTTTCAGCGGCT
GGGGAACGCATTCCAGTCCGTCAGTCAGGCATTCATGCTGGACCCTCCTC
ACAGCTCAGAGACCCTCAACAACGCCANNNNNNNN-----------------------

--------------GCCAAATCTCGCTTTCACCCTGGCGTAGGGACTGGT
CCTGGCACAGACC---GCAGCGTCCCACTTAGTAACAGCTTGCTATCCCC
GCAACAAACCGAAGAGCACACAGTTC---CTTCCCCGCAGCGATGGTTTG
TCACCC---CTGCCAATAACCGACTGGACTTTGCCGCCTCGGCATACGAC
GCCGCCGCGGCTGCTGATTTTGCCGGTAACGCGGCCACCTTGCTGTCCTA
CGCAGCGGCTGGAGTAAAAGCAC------TTCCCCTGCCCACTGCTGGGT
GTTCAAACAGACCTTTGGGTTATTATGCCGACCCATCCG---GCTGG---
GGCACCCGCACACCTCCTCAGTACTGT---------AGTAAATCCAGCTC
AGTGCTTTCGTGCTGGCCCACAAATGCTGTTGGAAGCAGAACAGGCA---
CATCCAATTACCTGA---------CGGAAGA---TGGG---GACAC---C
ATCCCCACGGAGAGGTCTCCA---AT---CGTAGTGCCAGAGGAG---AC
AAAACCAAAAGACTT------GTCCGA---ATCCAGCTGGATAGAG---A
CGTCATCTTCAATAAAGTCGATAGATTCAAGTGATTCTGGGATCTTTG--
-AGCAGGCCAAACGGAGAAGAATTTCGCCGTATTCCACAACG--------
-GTCTCGGAGACAGNNNNNNNNNNNNNNNNNNNN---------ACAGGCGAGGTCACAGACAGAGAAGTG
GCTTTGGGGATAAATCCGTTCGCRGACGGGATGGGCGCTTTCAAAATCAA
CCACAGCTCTCATGATCTGGGCTCCGG---GCAAACGGCGTTTTCCTCCC
AGGCT---CCCGGTTAC---GCAGCGGCCGCCTTGGGG---CACCATCA-
-----CCACCCGACCCATGTCAGCTCY---TACTCCACCGCCGCTTTCAA
TTCCACCCGGGACTTTCTTTTTCGAAATCGGGGCTTCGGAGACGCGACCA
G------------CGCGCAACACAGCCTGTTCGCCTCCGC---AGCGGGG
AGTTT---T------GCAGGGCCACATGGACACTCGGATGCCGCGGGACA
CCTGCTCTTCCCTGGACTCCACGAG---CAAGCCGCGAGCCACGCGTCCT
CCAATGTTGTGAACAGTCAGATGCGATTGGGTTTTTCGGGGGACATGTAC
GGGCGGGCTGACCAATATGGCCATGTTACGAGCCCACGGT---CCGACCA
CTACGCCTCGACCCAGTTGCACGGCTATGGTCCCATGAACATGAATATGG
CCGCA---CACCACGGAGCAGGGGCCTTCTTCCGTTACATGAGGCAGCCG
ATCAAACAAGAGCTSATCTGCAAGTGGGTCGAGCCGGAGCAGTTGTCGAA
CCCGAAAAAGTCGTGCAACAAAACTTTCAGCACGATGCACGAGCTCGTGA
CCCACCTGACAGTGGAACATGTGGGGGGACCAGAGCAGTCGAACCATATT

TGCTTTTGGGAAGAGTGTTGCCGAGAAGGGAAACCGTTCAAAGCTAAATA
CAAACTTGTAAATCATATCAGAGTACACACCGGAGAGAAACCGTTCCCGT
GTCCGTTCCCCGGCTGTGGCAAA
>Naso lituratus
AGTCTACTTATTCGGGCAGAACTAAGCCAACCAGGCGCCCTCCTCGGAGA
TGACCAAATCTATAATGTAATTGTTACAGCACATGCTTTCGTAATAATTT
TCTTTATAGTAATGCCAATTATAATTGGAGGGTTTGGAAACTGACTAATC
CCACTAATGATCGGGGCCCCGGATATGGCATTTCCCCGAATGAACAACAT
GAGCTTCTGACTACTCCCTCCTTCTTTCCTTCTCCTTCTTGCATCATCTG
GGGTTGAAGCTGGGGCCGGAACCGGATGAACAGTCTACCCCCCTCTAGCT
GGTAACCTAGCACACGCAGGGGCTTCCGTTGATCTAACTATCTTCTCCCT
TCATCTGGCAGGGATTTCCTCAATTCTAGGGGCAATTAATTTTATCACAA
CTATCATTAACATGAAACCTCCTGCTATTTCTCAGTACCAAACCCCTCTA
TTCGTCTGAGCTGTATTAATCACGGCAGTACTGCTCCTTCTTTCTCTTCC
AGTCCTTGCTGCTGGCATCACAATACTCCTCACCGACCGAAACCTGAACA
CAACCTTCTTCGACCCTGCAGGCGGAGGAGATCCGATTCTTTACCAACAC
CTC---

------------NNNCTAGAGAGAAACCTTCACCCATCTAACTGTCTTGGCAT
GCTGTTGCTGTCTGACGCCCACCAGTGCACCAAGCTGTCAGAGCTCTCCT
GGGGCATGTGCTTGAGCAACTTCCCTGCTATTTGCAAGACAGAGGACTTC
CTCCAGCTGCCCAAAGATATGGTAGTGCAGCTTTTGTCACATGAAGAGCT
AGAAACAGAAGATGAGAGACTGGTTTATGAAGCTGCCCTCAACTGGATCA
ACTATGACTTGGAAAAGAGGCACTGCCACCTTCCAGAGCTCCTGAGAACA
GTTCGTCTGGCCCTCCTGCCTGCCATCTTTCTCATGGAGAATGTTTCTAC
AGAGGAGCTGATCAACGCCCAGGCCAAGAGCAAAGAGCTAGTGGATGAAG
CCATCCGCTGTAAGCTGAAGATCCTGCAGAATGATGGTGTCGTTAACAGC
CCGTGTGCTCGACCAAGAAAAACCAGCCATGCCCTCTTTCTTCTAGGAGG
GCAGACTTTTATGTGTGACAAGTTGTACCTGGTGGACCAGAAAGCCAAAG
AGATCATCCCCAAGGCCGATATTCCCAGTCCCAGGAAGGAGTTCAGCGCC
TGTGCCATCGGCTGTAAGGTGTACATCACCGGTGGAA--GAGGCTC-AGA
GAATGGTGTGTCCAAAGATGTATGGGTCTACGACACTGTCCACGARGAAT
GGTCGAAGGCAGCACCTATGCTCATTGCCAGGTTCGGCCATGGCTCTGCA
GAGCTGAAGCACTGCCTCTATGTGGTAGGAGGTCACACGGCTGCCACTGG
CTGCCTCCCGGCCTCTCCGTCTGGATGAATACATAGTTGTGTTTAGTCGT
TCCACAACAAGACTGATTCTGAATGAAGCGGAGGTAATCATGGCTCTGGC
CCAGGAATTCCAGATTAGAGTGGTCACGGTATCTTTGGAGGAACAGTCTT
TCCCCAGTATTGTCCAGGTAATCAGCAGTGCTACCATGTTAGTCAGCATG
CATGGAGCTCAGCTAATTACCTCACTCTTTCTTCCCAGAGGAGCTGCTGT
AGTGGAACTGTTCCCTTTTGCTGTTAACCCAGAGCAGTACACCCCATATA
AAACCCTAGCGTCCCTTCCAGGCATGGACCTTCACTATATCTCCTGGAGG
AACACTAAGGAAGAGAACACCATCACCCACCCAGACAGACCTTGGGAACA
AGGGGGCATCAGTCACTTGGAAAAGAAGGAGCAAGAGAGAATACTGGCAA
GCAAAGATGTCCCCAGGCACCTGTGCTGCCGCAACCCAGAGTGGCTCTTC
CGTATCTACCAGGACACTTTAGTAGACATCCCTTCCTTCCTGGAAGTCCT
CAA---AGAGGGCATGAAA---ACAAAGCCCAGCTTGAAGAA---GTCAA
AGCCAGCCAGCACTGTTCACCCAGGTCGAGTTAGAGAGCCCCAGTGTCAG
ACCTCAGTACAAACCACTAATGAGGCTAAACTCACGGTCTCCTGGCAGAT
CCCATGGAATCTGAAATACCTNNNNNNNNNNNNNNNNNNNNNNNNNNNNNNNNNNNNNAAAAAGGACACA
AACAAGGGGACACTGGA
GGATCAAATCATCCAGGCAAACCCTGCGTTAGAGGCCTTCGGCAACGCCA

AAACGGTAAGAAATGACAACTCATCTCGGTTTGGAAAATTCATCCGAATT
CACTTTGGTACGAGTGGCAAGCTTTCGTCTGCTGATATTGAGACGTACCT
GCTGGAGAAGTCACGTGTAACCTTTCAGCTCAAGGCTGAGAGGAATTACC
ACATCTTCTACCAGATCCTTTCCAATCAGAAGCCAGAGCTGCTCGACATG
CTGCTGATCACTAACAATCCATATGACTATTCTTACATCTCCCAAGGAGA
GGTAACTGTCGCATCCATCAATGACTCAGAGGAGCTGATGGCCACAGACA
GCGCCTTCGATGTGCTTGGCTTTACCACTGAGGAGAAGATGGGAGTCTAC
AAACTGACAGGCGCCATCATGCACTACGGTAATATGAAGTTTAAACAGAA
ACAGCGTGAGGAGCAGGCTGAGCCTGATGGCACCGAGGCTGCTGATAAAT
CAGCTTACCTAATGGGGCTGAACTCTGCTGACCTCATCAAAGGGCTGTGC
CATCCCAGAGTCAAGGTAGGAAATGAGTACGTCACCAAGGGCCAAAGTGT
GGACCAAGTCAACTATCCCAACAAGGAGGCCTTCAAGTGTGAGGAGTGTG
GAAAGCATTACAACACAAAGCTAGGATATAAGCGCCATGTGGCCATGCAC
TCTGCCACGGCAGGAGATCTCACCTGTAAAGTGTGCATGCAGTCGTACGA
GAGCACACCAGTGCTCTTGGAGCACCTCAAGAGCCACTCTGGGAAGTCTT
CAGGTGGCACCAAGGAGAAAAAACATCCTTGCGACCACTGTGACCGTCGT
TTCTATACACGCAAGGATGTGAGAAGGCACATGGTCGTCCACACGGGCCG
AAAGGACTTCCTATGCCAGTACTGTGCCCAACGCTTCGGTAGGAAGGACC
ATCTGACACGCCATGTGAAGAAGAGTCACTCGCAAGAGCTGCTGAAGATC
AAGACGGAGCCTCCTGATATGTTAGGCCTTCTAGCCTCCGGGTCACCACC
TTGTTCTGTGAAGGAGGAGCTCAGCCCCATGGTGTGCGGCATGGGTTCCA
ACAAAGACCCCATGATGGGCAAACCTTTCCCCAGTGGGGCCCCTTTTCCG
ATGGGCATGTACAACCCCCACCAT------CTCCAGGCCATGTCTAATTC
TGGGGTGGGTCACCCA------CACCCGTCCCTGATGCCCAGTTCCTTGT
CTGCAGCTATGAGCATGGGCTGTCACATGGAANNNNNNNNNNNNNNNNNNNNNNNNNNNNNNNNATGTTT
ACAGATCAGTGA
TGGATCAAATATCGTGAACCTGCTGGCTAGTAACTCTCCGAGTGTTTCGT
ACGCTCTGACCCAGCAAAAATACTTTAGTAACTACAGTCCTGTGATTGGG
TTTTACATTTATGAGCCTATTGAGTACTGGAACTCCACGGTGCAAGAGCA
CCTGAAAACTCTGAGTCATGGCTTCAACAAGATCTCCTGGATGGACAACT
TTTTCCACTACCTGCGGGTAGTGAATGTAAGTGCATCAACTAAGAGCGAC
TTCATCACCATCCTGAAAGGCTCCTTCCTGCGCAGCCCGGAGTACCAGCA
CTTCACTGAGGACATCATATTCTCAAAGA---GCCGTGAGACTG------
ATGAGTACGACATTATTGCCTCACGGATGTACTTGGTGGCACGGACGACA
GAGAAGAAGCGCGAAGAGGTAGTGGAGCTTCTGGAAAAGCTTCGTCCATT
GATGCTGATCAACAGCATCAAGTTCATTGCCTTCAATCCGACATTTGTGT
TCATGGACCGCTACAGCTCCTCTGTCATCTCGCCCATCCTGACCTCAGGA
TTTAGCGTACTCACAATCCTCATCCTCACNNNNNNNNNNNNNNNNNNNNNNNNNNNNNNNNNNNNNNNNN
NNNNNNNNNNNNNNNNNNNNNNNNNNNNNNNNNNNNNNNNNNNNGGCTTTCACCAGTTTGAATGG
CAGCCAGCTCTCAAGAATGTGTCTACATCTTGCAATGTTGGCATTATTAA
TGGGCTCTCTGGAAGGGCTTCCTCGGTGGATGACTCCCCAGCTGACACCA
TCACTCGGCGGTTTCGCTATGATGTGGCACTTGTGTCAGCATTAAAGGAT
ATGGAGGAGGACATCATGGAGGGGCTGAGAGAGAAAGAGATGGAAGACAG
CATTTGCACCTCAGGCTTCAGTGTCATGATCAAAGAATCCTGTGATGGCA
TGGGCGATGTCAGCGAGAAGCATGGCGGAGGACCAGTTGTTCCTGAGAAG
GCTGTGCGTTTCTCTTTCACGATTATGTCTATTTCTGTCTTGGCAGATGG
TGAAGAG------------------------------GAAGAGGTTACCA
TCTTCACTGAGCCAAAGCCAAACTCAGAACTGTCCTGTAGGCCCCTTTGC
CTGACATTTGTGGATGAGTCAGACCATGAGACACTCACAGCCATCCTGTC
GCCTTTAGTTGCAGAGCGTGACGCGATGAAACACAGCAGGTTGATCTTAT
CCATCGGTGGACTATCTCGCGCCTTCCGCTTTCACTTCAGGGGCACGGGA
TATGACGAGAAGATGGTGCGTGAGATGGAGGGCCTCGAGGCCTCGGGGTC
AACCTATGTCTGTACACTTTGTGACGCCGAACGGGCAGAGGCCTCCCAAA

ACATGGTGCTACACTCCATTACCCGCAATCATGAAGAGAACCTTGAACGT
TACGAAATATGGCGAACCAACCCCTTTTCTGAGTCTGTAGATGAGCTGCG
AGACAGAGTCAAAGGGGTCTCTGCCAAGCCCTTTATGGAGACCCATCCCA
CGCTGGATGCATTGCACTGTGACATTGGCAATGCCACTGAGTTCTACAAA
ATCTTCCAGGACGAGATCGGGGAGGTGTACCAAAGGGT---CAAC---CC
CAGCCGAGAAGAACGGCGCAGCTGGAGGGCTGCCCTAGATAAACAGCTGA
GGAAGAAGTTGAAGCTTAAACCTGTAATGAGGATGAATGGGAACTATGCC
CGCCGGCTAATGACTCTTGAGGCTGTAGAGGTGGTGTGTGAGCTGGTGCC
CTCAGAGGGGAGGAGGGAGGCCCTGAGGGAGCTCATGAGGCTTTATCTCC
AGATGAAGCCTGTGTGGCGTGCCACCTGCCCCGCCAAGGAATGCCCTGAC
CAGCTGTGCCGCTACAGCTTTAACTCCCAGCGCTTTGCTGACCTCCTCTC
CTCTGCGTTCAAATATAGGTACAACGGAAAGATACCTAATTACCTCCACA
AGACCCTGGCTCATGTCCCTGAAATCATAGAAAGAGATGGATCCATAGGC
GCCTGGGCCAGCGAAGGGAACGAGTCAGCAAATAAGTCATACACCATCGA
GATGGGTCCCTTGGGACCCCGGTGGAAGGAGAGCCCACAGCCTTTCACCT
GCTCCGTTGAAGACCCCACAAAGCAGACAAAGTTCAAGGGCATCAAGACG
TACATTTCGTACCGGGTCACGCCAAGCCACACAACGCGTCCCGTCTACAG
GCGCTACAAACACTTTGACTGGCTGTACAACCGCTTGCTGCACAAGTTCA
CTGTGATCTCCGTGCCCCACCTGCCTGAGAAGCAGGCCACGGGGCGATTT
GAGGAAGACTTCATCGAGAAGCGCAAGAGGCGACTGATCCTGTGGATGAA
CCACATGACCAGTCACCCAGTCCTCTCCCAGTATGAAGGGTTCGAGCACT
TTCTGATGTGTGCTGATGACAAGCAGTGGAAACTTGGCAAGAGGCGAGCG
GAGAAGGACGAGATGGTGGGCGCCCATTTCATGCTGACCCTCCAGATCCC
TAACGAGCACCAGGACCTCCAGGATGTAGAGGAGCGGATCGATTCCTTCA
AGGCCTTCGCTAAGAAAATGGATGACAGTGTGATGCAGCTCACACATGTT
GCCTCGGAGCTGGTGCGTAAGCACCTGGGTGGGTTCAGAAAGGAGTTCCA
ACGACTGGGAAATGCCTTCCAGTCTATCAGCCAGGCCTTCATGCTGGACC
CTCCCCACAGCTCAGAGGCCCTCAACAAGNNNNNNNNNNNN---------------------
----

----------------NNNNNNNNNNNNTTTCACCCTGTCATGGGGACTGGTCCTGGCACGG
AGC---GCAGCGTCCCACTCGGCAACAGCTTGCTATCCCCGCAGCAAACC
GAGGAGCCCACTGTTGCCACCCCCCCGCAGAGATGGTTTGTCACCC---C
TGCCAACAACCGACTGGACTTTGCTGCCTCGGCATACGACGCCGCT----
-----GATTTCGCCGGTAACGCGGCCACCTTGCTGTCCTACGCAGCGGCC
GGAGTGAAGGCTC------TTCCCCTGCCGTCTGCAGGCTGCTCCAACCG

GCCTCTTGGCTATTACGCAGACCCGTCAG---GCTGG---GGAGGACGCA
CGCCGCCGCAGTACTGTGGCGTAAATAGCAAATCCAGCTCGGTGTTTTCC
TGCTGGCCCACTAACTCTATCGGTAGCAGAGCGAGCA---CC---AACTA
CCTGA---------CCGAGGA---GGGA---GACCC---CATCCAGCCAG
AGAGGTCACCG---AT---CGGCGGCTCGGAGGAG---GCCAAACCCAAA
GACATGAC---CTCAGA---GTCGAACTGGATAGAG---ACGCCGTCCTC
CATTAAGTCGATCGATTCGAGCGATTCTGGTATCTTTG---AACAGGCCA
AAAGGAAGACGATCACCGNNNNNNNNNNNNNN---------
NNNNNNNNNNNNNNNNNNNNNNNNNNNNNNNNNCATCACTCAACAGGCGAAGTCAC
AGAGAGAGAAGTGGCGTTGGGGATAAATCCGTTCGCGGATGGGATGGGCG
CCTTCAAAATAAACCACAGCTCCCACGATATTGGTTCCGG---ACAAACG
GCGTTTTCATCCCAGGCG---CCCGGCTAC---GCAGCAGCTGCCCTGGG
A---CACCATCA------CCACCCGACCCACGTTGGCTCT---TACTCCA
CGGCGGCTTTCAACTCCACCAGGGACTTTCTCTTCAGAAATCGGGGATTC
GGGGACGCCACCAG------------CGCGCAGCACAGTTTGTTCGCCTC
---------CGGAAGTTT---C------GCAGGGCCACATGGACACTCAG
ATGCAGCGGGGCACCTGCTCTTCCCGGGGCTCCACGAG---CAAGCCGCG
AGCCATGCGTCTTCCAACGTGGTCAACAGCCAGATGCGGCTGGGCTTCTC
GGGGGACATGTACGGACGGGCCGACCAGTATGGCCACGTTACAAGCCCGC
GGT---CCGACCACTATGCCTCGACCCAGCTGCACGGCTATGGCCCCATG
AACATGAATATGGCCGCA---CACCACGGAGCAGGGGCCTTCTTTCGATA
CATGAGGCAGCCGATMAAACAAGAGCTCATCTGCAAGTGGATCGAGCCGG
AGCAGCTGACGAATCCCAAAAAGTCGTGCAACAAAACTTTTAGCACGATG
CACGAGCTCGTGACCCATCTGACGGTGGAGCATGTGGGGGGACCGGAGCA
GACAAACCACATCTGTTTCTGGGAGGATTGCGCCAGAGAAGGAAAGCCAT
TCAAAGCCAAATACAAACTTGTAAATCATATCAGAGTACACACCGGAGAA
AAACCCTTTCCGTGTCCGTTCCCCGGCTGTGGCAAA
>Neochanna burrowsius

NNNNNNNNNNNNNNNNNNNNNNNNNNNNNNNNNNNNNNGGCTCATCCTCAACGAGGCGGAGGT
GATCCTGGCGCTGGCCCAGGAGTTCCAGATGAGGGTGGTGACGGTGTCCC
TGGAGGAGCACTCCTTCCCCGCCATCGTCCAGGTCCTCAGCCGGGCCTCC
ATGCTGGTGAGCATGCACGGAGCCCAGCTCATCACCTCCCTCTTCCTCCC
TCGCGGGGCGGTGGTGGTGGAGCTCTTCCCCTACGCCGTCAACCCCGAGC
AGTACACGCCCTACAAGACCCTGGCCTCTCTGCCCGGCATGGACCTGCAC
TACGCAGCCTGGAGGAACACCATGGAGGAGAACACGGTGGCCCACCCGGA
CAGACCCTGGGACCAGGGGGGCATCGCTCACCTGGACGCGGAGGAGCAGG
AGCGCATCCTAGCCAGCCGGGACGTCCCACGACACCTGTGCTGCCGGAAC
CCGGAGTGGCTGTACCGGATCTACCAGGACACCAGGGTGGACGTTCCCTC
TCTCCTGGAGCTCCTGCG---GGAGAACTTGAAG---ACCCGGCCCAGCC
TGAGGAA---GGCCCGGCCCGTCAGCACCGTCCACCCGGGGCGGGTCCGC
CAGGCCCAGTGTCTGACGTCCGTCCAGGCCGCCAACGAGGCCAAGCTGAC
GGTGTCGTGGCAGATCCCCTGGAACTTAAAGTACCTGAAGGTCAGGGAGG
TGAAGTACGAGNNNNNNNNNNNNAAGAGGGATCCAAGCAAGGGGACCCTGGAGGATCAAATC
ATTCAGGCAAATCCTGCACTGGAGGCTTTTGGTAATGCCAAAACGCTAAG
AAACGACAACTCATCCCGCTTTGGTAAATTCATCCGAATTCACTTTGGAA
CTACTGGAAAATTATCCTCTGCTGACATAGAGACTTACCTTCTTGAGAAA
TCGCGGGTTACCTTTCAGCTAAAGTCAGAGAGAAACTACCATATTTTCTT
CCAGATCTTGTCCAATCAAAAGCCAGAGCTGTTGGACATGCTGTTAATCA
CCAACAATCCCTATGACTACTCCTACATCTCCCAGGGAGAGGTGACAGTA
CATTCAATTAATGATTCCGAAGAGCTAATGGCAACTGACAGTGCCTTTGA
TGTGCTGGGCTTTACTCAGGAGGAAAAAATGGGTGTCTACAAGTTGACAG
GTGCCATCATGCACTACGGCAACATGAGGTTCAAGCAAAAGCAGCGTGAA
GAGCAGGCCGAGCCAGATGGTACAGAGGCTGCTGACAAATCAGCTTACCT
GATGGGTCTCAATTCTGCAGACCTTATTAAAGGGCTTTGTCATCCCAGAG
TCAAAGTAGGCAATGAGTATGTTACCAAAGGTCAAGGTGTAGATCAAGTC
TACTAC--

------------------------------------GGCTACTACGACTT
CGAGTGGCAACCGGCCCTGAGGAACGTGTCCCGGGACTGCCACGTGGGCA
TCATCGACGGCCTCTCGGGCTGGGCGGCGTCGGTGGACGACTCGCCGGCG
GACACCATCACCCGCCGCTTCCGCTACGATGTGGCCCTGGCCTCCGCCCT
GAAGGACCTGGAGGAGGACATCCTGGCGGGCCTGAGGGAGCGTGGCCTGG
AGGAGGGCGCCGGCGCAACCCACTTCAGCGTCATGGTCAAGGAGTCCTGC
GATGGCATGGGTGACGTGAGCGAGAAGCACGGCGGCGGGCCGGCCGTCCC
AGAGAAGGCGGTGCGCTTCTCCATCACCATCATGTCCGTCTCCGTCCAGG
CGGAGGGGCAGGGG------------------------------GAGGCC
GTGACGGTGTTCAGGGAGACCAAGCCCAACTCGGAGCTCTCCTGCAGGCC
CCTGAGTCTGATGTTCGTGGACGAGTCGGACCACGAGACGCTCACAGCCA
TCCTCGGCCCGGTGGTGGGCGAGCGGGACGCCATGAAGCGGAGCCGGCTC
CTCCTGACCCTGGGCGGCCTCCAGCGCTCCTTCCGCTTTCACTTCCGGGG
CAGCGGCTACGACGAGAAGATGGTGCGCGAGATGGAGGGCCTGGAGGCCT
CGGGCTCCACCTACATCTGCACCCTGTGTGACTCCACTCGGGCGGAGGCT
TCGTCCAACATGGTGCTCCACTCCATCACCCGCAGCCACGACGAGAACCT
GGAGCGTTACGAGGTGTGGAGGACCAACCCGTTCTCCGAGTCGGCCGACG
AGCTGCGAGACCGGGTGAAGGGGGTCTCCGCCAAGCCCTTCATGGAGACC
CAGCCGACCCTGGACGCGCTGCACTGCGACATCGGCAACGCCACCGAGTT
CTACAAGATGTTCCAGGATGAGATCGGGGAGGTGTTCCGGAAGCC---CA
AC---CCCAGTCGGGAGGAGAGGCGCAGCTGGCGGGCAGCCCTGGACAAA
CAACTCAGGAAGAAGCTGAAGCTGAAGCCGGTCATGCGCATGAACGGGAA
CTACGCCCGGCGCCTGATGACCCAGGAGGCGGTGGAGGTGGTGTGTGAGC
TGGTGCCCTCGGAGGAGAGGCGACTAGCCCTCAGGGAGCTCATGGGCCTG
TACATCCAGATGAAGCCGGTGTGGCGCGCCACATGCCCGGCCAAGGAGTG
CCCTGACCAGCTGTGCCGCTACAGCTTCAACTCGCAGCGCTTCGCCGAGC
TCCTCTCCACCACCTTCAAGTACAGGTACGAAGGCAAGATCACCAACTAC
CTCCACAAAACGCTGGCCCATGTGCCCGAGATCATAGAGAGGGACGGGTC
CATTGGAGCGTGGGCCAGCGAGGGGAACGAGTCGGGCAACAAGNNNNNNNNNNNNNNNNNNNNNNNNNNN
NNNCCCCAGT
GGAAGGCCAGCCCTCAGCCGTTCACCTGCTCCATCGAGGACCCCACCAAA
CAGACCAAGTTCAAAGGCATCAAGACCTACATATCGTACCGCGTGACCCC
CAGCGACACGGGCAGGCCGGTGTACCGGCGCTACAAACACTTCGACTGGC
TCTACAACCGCCTGCTCCACAAGTTCACCGTGATCTCCGTGCCCAACCTG
CCCGAGAAGCAGGCCACGGGGCGCTTCGAGGAGGACTTCATCGAGAAGAG
GAAGAGGCGCCTCATCCTGTGGATCAACCACATGACCAGCCACCCGGTGC
TGTCGCAGTACGAGGGCTTCGAACACTTCCTGATGTGCGCCGACGACAAG
CAGTGGAAGCTGGGTAAGAGGCGCGCCGAGAAGGACGAGATGATCGGGGC
GCATTTCATGCTGACCTTCCAGATCCCCAACGAGCACCAGGACCTGCAGG
ACGTGGAGGAGAGAGTGGACACCTTTAAGAACTTCGCCAAGAAGATGGAC
GACAGCGTTGTGCAGGTGAANNNNNNNNNNNNNNNNNNNNNNNNNNNNNNNNNNNNNNNNNNNNNNNNNN
NNNNNNNNNNNNNNNNNNNNNNNNNNNNNNNNNNNNNNNNNNNNNNNNNNNNNNNNNNNNNNNNNNNNNN
NNNNNNNNNNNNNNNNNNNNNNNNNNNN------------------------------

-------GCCAAATCTCGATTTCACCCTGGCGTAGGGACTGGTCCTGGCA
CAGACC---GCAGCGTCCCACTTAGCAACAGCTTGCTATCTCCGCAACAA
ACCGAGGAGCCCACAGTTG---CTTCCCCACAGCGTTGGTTTGTCACCC-
--CTGCCAATAACCGACTGGACTTTGCCGCCTCGGCATACGATGCCGCTG
CTGCGGCTGATTTTGCCGGTAACGCAGCCACCTTGCTATCCTACGCAGCG
GCTGGAGTAAAGGCAC------TACCCCTGCCCACAGCAGGTTGCTCAAA
TAGGCCACTTGGTTATTATGCCGATCCATCAG---GGTGG---GGCGCCC
GGACACCACCCCAATACTGT---------AGTAAATCCAGTTCAGTACTC
TCTTGCTGGCCTAGTAATTCTGTTGGCGGCAGAACAACTA---------A
CTACCTGG---------TGGATGA---TGTA---GACGC---CCTTCCTA
CAGAGAGATCGCCC---AT---TGGTGGCTCCGAGGAG---ACAAAACCT
AAAGACTT------GTCAGA---ATCAAGCTGGATAGAA---ACGCCATC
TTCAATAAAGTCAATTGATTCAAGTGATTCTGGAATCTTTG---AGCAAG
CCAAACGGAGAAGAATTTCACCGTCTGCCACTCCG---------GTTTCA
GAGACTGTGTCCCCGTTGAAATCTGAA-----------------------

>Neonesthes capensis
AGCCTGCTAATCCGGGCAGAGCTAAGTCAACCCGGTGCCCTTCTAGGCGA

CGACCAAATCTATAATGTTATCGTAACAGCACATGCTTTCGTAATAATTT
TCTTTATAGTAATACCCCTTATGATCGGGGGCTTTGGAAACTGACTGGTC
CCCCTTATGATTGGGGCTCCTGATATGGCCTTCCCTCGAATAAATAACAT
AAGCTTCTGACTCCTCCCTCCCTCCTTCCTCCTACTTCTGGCTTCCTCAG
GGGTTGAAGCCGGGGCCGGAACAGGATGAACTGTGTACCCTCCTCTCGCC
GGTAACCTAGCACATGCGGGAGCCTCCGTTGACCTAACAATCTTCTCACT
TCACCTGGCGGGGATCTCTTCAATTCTGGGGGCAATCAACTTCATCACCA
CTATCATCAACATAAAGCCCCCGGCCATCTCCCAATACCAAACACCCCTC
TTCGTCTGATCCGTCCTTGTTACTGCTGTTCTCCTCCTACTATCCCTACC
AGTCCTAGCCGCCGGAATCACTATGCTTTTAACAGACCGAAACCTTAACA
CAACCTTCTTTGACCCGGCAGGAGGAGGAGACCCTATTCTTTACCAACAC
CTCTTCTGGTTCTTTGGTCACCCAGAAGTCTACATCTTGATTCTCCCCGG
CTTCGGAATGATCTCTCATATTGTGGCCTACTACTCAGGAAAGAAAGAGC
CCTTTGGCTACATGGGAATAGTCTGAGCTATGATGGCTATCGGACTCCTG
GGGTTTATTGTCTGAGCCCACCACATGTTCACAGTTGGCATGGACGTAGA
TACCCGCGCCTNNNNNNNNNNNNNNNNNNNNNNNNNNNNNNNNNNNNNNNNNNNNNNNNNNCTCTCCGATG
CCCATCAGTGTACCAAGCTGTCCGAGCTG
TCCTGGGGCATGTGCCTCAGCAACTTTCCCACCATTTGCAAGACTGAGGA
CTTCCTACAGTTGCCYAAAGACATGGCGATCCAGCTGCTGTCTCACGAGG
AGCTGGAGACGGAGGACGAGAGGCTGGTCTATGAGGCCGCCCTCAACTGG
GTCAACTACGATCTGGAGAGGCGGCACTGTCACTTGCCAGAGCTGCTAANA
ACCGTTCGTCTGGCCTTGCTGCCCGCCATCTTCCTCATGGAGAACGTCTC
CATGGAGGAGCTGATCAACGCCCAGACCAAGAGCAAGGAGCTGGTGGATG
AGGCCATCCGCTGCAAGCTGAGGATCCTGCAGAATGAGGGTGTGGTCAAC
AGCCCGCTGGCCAGGCCAAGGAAGACCAGCCACTCCCTCTTCTTGCTGGG
TGGGCAGACCTTCATGTGTGACAAACTCTACCTGGTGGACCAGAAGGCCA
AGGAGATCATCCCCAAGGCGGACATCCCCAGCCCCAGGAAGGAGTTCAGC
GCCTGCGCCATCGGCTGCAAGGTCTACATCACCGGAGGCA--GGGGCTC-
TGAGAACGGGGTGTCCAAAGATGTTTGGGTGTACGATACCTCCCACGAGG
AGTGGTCGAAGGCGGCGCCCATGCTCATCGCTCGGTTCGGCCATGGCTCT
GCACAGCTGAAACACTGCCTCTACGTGGTAGGAGGACACACTGCCGCCAC
AGGCTGCCTCCCCTGCTNNNNNNNNNGGACGAGTACATCGTGGTGTTCAGTCGTTCCGT
CACCAGGTTGATCCTCAACGAAGCAGAGTTGATCTTGGCGTTGGCGCAGG
AGTTTCACATGAGGGTGGTGACGGTGTCTCTAGAGGACCAAACCCTCTCC
AGCATTGTCCAGGTGATCAGCGGCGCCGCCATGCTAGTAAGTATGCACGG
AGCACAGCTGGTCACCTCCCTCTTCCTCCCCAGAGGAGCAGCCGTGGTGG
AACTCTTCCCCTACGCTGTCAACCCTGAACAGTACACCCCTTACAAGACC
TTGGCCACCTTGCCCGGCATGGACCTTCAGTACGCAGCCTGGAGGAACAC
GGTGGAGGAGAACTCTGTGGCCTACCCAGGTAGACCCTGGGACCAAGGAG
GCATCGCCCATCTGGACAAGGATGAGCAGGAGCGCATCCTGACCAGCAGC
GATGTTCCCAGGCATCTGTGCTGCCGAAACCCGGAGTGGCTCTTCCGCAT
TTACCAGGACACCAGAGTCGATATCCCATCTCTGCTGGAGGTGTTGAG--
-GCAGAGCCTGAAG---ACCAGGCCCAGTTTGAAGAA---GACCAGGCCT
GCCAGCACCGTCCATCCAGGCCGGGTCAGGGAGCCCCATTGCCAAACCTC
GGTCCAGGCGACTAATGAAGCCAAGCTTACAGTGTCCTGGCAAATCCCCT
GGAACCTGAAANNNNNNNNNNNNNNNNNNNNNNNNNNNNNNNNNNNNNNNNNNNNAAGAGGGATSCCAGCAA
GGGAACCTTGGAGGATCAAATC
ATCCAAGCTAACCCTGCACTGGAGGCTTTCGGCAATGCCAAAACCTTGAG
AAATGACAACTCGTCACGCTTTGGTAAATTCATCCGGATTCACTTTGGAA
CCWCWGGCAAGTTGTCCTCTGCAGACATAGAGACTTACCTTCTGGAAAAG
TCACGAGTCACCTTTCAGCTCAAGTCAGAGAGGAACTATCATATCTTCTT
CCAGATCTTGTCCAATCAAAAGCCAGAGCTGTTGGACATGCTTTTAATCA
CCAACAATCCGTATGACTACTCCTTCATCTCCCAAGGAGAGGTAACAGTA

GCATCCATCAATGATTCTGAGGAGTTGATAGCCACTGACAGTGCATTCGA
TGTGCTTGGCTTTACTCAAGAGGAGAAAATGGGAGTGTACAAGTTGACAG
GTGCAATCATGCATTACGGCAACATGAAGTTCAAGCAAAAGCAGCGAGAG
GAGCAGGCGGAGCCTGATGGCACGGAGGCTGCTGACAAGTCAGCTTACCT
AATGGGGCTGAACTCTGCAGATCTAGTGAAGGGACTCTGCCATCCCAGGG
TTAAGGTTGGCAATGAGTTTGTCACTAAAGGGCAGAGTGTAGACCAAGTC
TATTACATTAACAAGGAGGCTTTCAAGTGCGAGGAGTGTGGCAAGCACTA
CAACACCAAGCTGGGCTACAAGCGCCATGTGGCCATGCACTCGGCCACGG
CGGGCGACCTCACTTGCAAGGTGTGCCTGCAGAGCTACGAGAGCACACCC
GCCCTGCTGGAGCACCTGAAGAGCCACTCSGGGAAGTCGTCGGGCGGGGC
CAAGGAGAAGAAGCACCCGTGCGACCACTGCGACCGCCGTTTCTACACAC
GCAAGGACGTCAGGCGCCACATGGTGGTGCACACCGGCCGCAAGGACTTC
CTGTGCCAGTACTGCGCCCAGCGCTTTGGCAGGAAGGACCATCTGACACG
GCACGTGAAGAAGAGCCACTCGCAGGAGCTGCTGAAGATCAAACCGGAGC
CTCCRGATATGTTAGGTCTTCTAGGTTCTGGCTCGCCACCTTGCTCAGTG
AAGGAGGAGCTTAGCCCTATGATGTGCAGCATGGGTCCCAACAAAGACCC
CATGATGGGCAAACCCTTCCCCAGTGGACCCCCTTCCCCATGGGCATGT
ACAACCCCCATCAC------CTCCAGGCCATGTCCAATTCTGGGGTGGGT
CACCCC------CACCCCTCCCTGATGCCTACCCCCCTGTCTGCAGCTAT
GGGGATGGGCTG----------TACCTGATCTACGCCTCCTTCTCCTTCA
TGGGATGTTTACAAATCAGCGACGGATCCAACGTTGTCAACCTGTTAGCC
AGCAACTCCCCCAGTGTATCGTACGCTCTGACCCAGCAGAAGTACTTCAG
TAACTACAGCCCTGTGATCGGTTTCTACATCTACGAACCCATCGAGTACT
GGAACTCCACGGTGCAGGAGCACCTGAGAACTCTGGGCCAAGGCTTCAAC
AAGATCTCCTGGATGGACAACTACTTCCACTACCTGAAGGTGGTGAATGT
GACCGCCTCCACCAAGAGCGACTTCATCGCCATCCTCAAAGGCTCCTTCC
TGAAGAGCCCGGAGTACCAGCACTTCACTGAGGACATCATCTTCTCAAAG
A---AAGGCG------------AGGAGTATGACATCATTGCGTCCAGGAT
GTACCTGGTAGCACGGACTACGGAGAAGACGCGGGAGGAGGTGGTGGAGC
TGCTGGAGAGGCTCCGGCCCCTCTCCCTCATCAACAGCATCAAGTTCATC
GTCTTCAACCCCACCTTCGTCTTCATGGACCGTTACAGCTCTTCGGTCGT
ATCGCCCATTCTCACCTCTGGCTTCAGTGTGCTCACCATCCTCATCCTCA
CCTTCTTCCTGGTCATCAACCCCCTGGGGAACTTTTGGTTGATCCTGACG
GTGACCTCTGTGGAGCTGGGTGTTCTGGGCCTGATG--------------

----ARGGCCGTGCGCTTCTCCTTCACTGTCATGTCCATCTCCGTCCAGG
CCGAGGGCGCGGAC------------------------------GAGGCC
GTCACTGTCTTCCGGGAGCCCAAGCCCAACTCCGAGCTGTCCTGCAAGCC
CCTGTGCCTGATGTTCGTGGACGAGTCCGACCACGAGACGCTGACGGCCA
TCCTGGGGCCCGTGGTGGCCGAGAGGGACGCCATGAAGAGCAGCCGCCTC
ATCCTGTCGATGGGGGGCCTCCCTCGCTCTTTCCGCTTCCACTTCAGGGG
TACGGGCTACGACGAGAAGATGGTACGCGAGATGGAGGGGCTGGAGGCCT
CGGGCTCCATGTACATCTGCACCCTCTGCGACTCCACCCGGGCGGAGGCC
TCCCACAACATGGTGCTCCACTCCATCACCCGTAGCCACGAGGAGAACGT
GGAGCGCTACGAGATCTGGAGGACCAACCCCTACTCGGAGAGCGCGGACC
AGCTGCGCGACCGGGTGAAGGGCGTCTCGGCCAAGCCCTTCATGGAGACC
CAGCCCACCCTGGACGCCCTGCACTGCGACATCGGCAACGCCACCGAGTT
CTACAAGATGTTCCAGGACGAGATCGGGGAGGTGCACCGGAGGCC---CG

GG---CCCAGCCGGGAGGAGCGGCGCGGCTGGCGCGCAGCGCTGGACCAG
CAGCTGAGGAGGAAGATGAAGCTGAGGCCGGTGATGAGGATGAACGGGAA
CTACGCCCGCCGGCTGATGACCGTGGAGGCGGTGGAGGCGGTGTGCGAGC
TGGTCCCCTCGGAGCCGCGGCGGGAGGCCCTCCGGGAGCTGATGGCGCTC
TACCTCCAGATGAAGCCCGTGTGGCGC-----------------------

---TCCTACA
CAATCGAAATGGGCCCCAAAGGGCCTCAGTGGAAGGAGAGCCCTCAGCCG
TTCTCTTGTTCTGTTGAGGACCCTACCAAACAGACCAAGTTCAAAGGCAT
CAAGACCTACATATCTTACCGGGTCACCCCAAGCCACACTGGGCGACCTG
TATACCGTCGGTACAAGCATTTTGATTGGCTATACAACCGCCTGCTGCAT
AAGTTCACTGTCATCTCTGTGCCTCACCTGCCGGAGAAGCAGGCCACAGG
GCGTTTCGAGGAGGACTTCATCGAAAAGCGCAAAAGGAGGCTGATCCTCT
GGATGGACCACATGACCAGTCACCCAGTCCTCTCCCAATATGAGGGCTTT
GAGCACTTCCTCATGTGCGCTGATGACAAACAGTGGAAGCTGGGCAAGCG
GCGAGCGGAGAAGGATGAAATGGTGGGCGCCCACTTCATGCTGACCTTTC
AGATTCCCAAAGAGCACCAGGACCTGCAGGATGTGGAGGAGAGAGTGGAC
TCCTTCAAGTCTTTTGCTAAGAAAATGGATGATAGTGTCATGCAGCTGAC
TCACGTGACCTCAGAACTGGTGCGGAAACATCTAGGGGGTTTCAGAAAGG
AATTCCAGCGTCTGGGGAACGCTTTCCAGTCCATTAGTCAGGCATTCACG
CTGGACCCTCCTCACTGCTCTGAAGCCCTCAACAACGCCATCTCACAC--

-----------------------GTCACAGAGAGAGAAGTGGCTTTGGGG
ATAAATCCGTTCGCAGACGGGATGGGCGCTTTCAAAATCAACCACAGCTC
GCATGATCTCGGTTCAGG---CCAAACGGCGTTTTCCTCCCAAGCA---C
CCGGCTAC---GCAGCTGCTGCCCTCGGC---CACCACCA------CCAC
CCGACACACGTCAGCTCC---TACTCCACCGCGGCTTTCAACTCAACCCG
GGACTTTCTCTTCAGAAATCGGGGCTTCGGAGACGCCACCAG--------
----TGCGCAACACAGTCTCTTCGCATCCGC---AGCGGGAAGTTT---T
------GCAGGACCACATGGACACTCAGATGCCGCGGGGCACCTGCTCTT
CCCAGGACTTCACGAG---CAAGCCGCGAGCCACGCGTCCTCAAATGTTG
TTAACAGTCAAATGCGGTTGGGCTTTTCGGGGGACATGTACGGGCGGGCC
GACCAGTATGGCCACGTTAACAGCCCGCGGT---CCGACCACTACGCTTC
GACCCAGTTGCATGGCTATGGCCCTATGAACATGAATATGGCCGCA---C
ATCACGGAGCAGGGGCCTTCTTCCGTTATATGAGGCAGCCGATAAAACAA
GAGCTGATCTGCAAGTGGATCGAGCCAGAGCAACTAACGAATCCGAAAAA
GTCGTGCAACAAAACTTTTAGCACGATGCACGAGCTGGTGACCCATTTGA
CGGTGGAGCATGTGGGAGGACCGGAGCAGTCGAACCATATTTGCTTCTGG
GAAGAGTGTGCCCGAGAAGGAAAACCATTCAAAGCCAAATACAAACTTGT
GAACCACATCAGAG------------------------------------

>Neosalanx jordani
AGCCTCCTCATTCGGGCCGAACTTAGCCAGCCCGGCGCTCTTCTCGGAGA
CGACCAAATCTATAACGTCATCGTTACTGCACACGCCTTCGTAATAATCT
TCTTCATAGTAATACCCATCCTGATTGGCGGGTTTGGAAACTGACTCGTC
CCCCTCATGATTGGGGCCCCGGACATGGCATTCCCCCGAATAAACAACAT
AAGCTTCTGACTCCTACCTCCTTCTTTCCTGCTGCTCCTAGCCTCCTCTG
GGGTTGAAGCTGGGGCTGGGACCGGATGAACCGTATACCCCCCTCTTGCT
GGCAATCTCGCCCATGCAGGAGCCTCAGTAGACCTGACGATCTTCTCCCT
GCACCTTGCTGGGATCTCCTCCATCCTGGGGGCAATTAACTTCATTACAA
CCATTATTAACATAAAACCCCCTGCCACCTCTCAGTACCAGACACCCCTC
TTCGTCTGATCTGTTCTGATTACTGCAGTCCTACTGCTACTCTCTCTCCC
AGTCTTAGCTGCTGGCATCACCATGCTCCTGACAGACCGAAACCTAAACA
CCACCTTCTTCGACCCCGCAGGAGGAGGGGATCCTATCTTGTACCAACAC
CTGTTCTGATTCTTTNNNNNNNNNNNNNNNNNNNNNNNNNNNNNNNNNNNNNNNNNNNNNNNNNNNNNNN
NNNNNNNNNNNNNNNNNNNNNNNNNNNNNNNNNNNNNNNNNNNNNNNNNNNNNNNNNNNNNNNNNNNNNN
NNNNNNNNNNNNNNNNNNNNNNNNNNNNNNNNNNNNNNNNNNNNNNNNNNNNNNNNNNNNNNNNNNNNNN
NNNNNNNNNNNNNNNNNNNNNNNNNNNNNNNNNNNNNNNNNNNNNNNNNNNNNNNNNNNNNNNNNNNNNN
NNNNNNNNNNNNNNNNNNNNNNNNNNNNNNNNNNNNNNNNNNNNNNNNNNNATCTGCAAGACGGAGGAC
TTCCTCCAGCTCCCCAA
GGACATGGTGGTTCAGCTGCTGTCCCACGAGGAGCTGGAGACGGAGGACG
AGAGGCTGGTCTACGAGGCTGCCCTCAACTGGGTGAACTACGACCTGGAG
AGACGCCACTGCAGCCTGCCGGAGCTGCTGCACACAGTGCGCCTGGCGCT
GCTGCCCGCCATCTTCCTCATGGAGAACGTATCCACGGAGGAGCTGATCA
ACGCCCAGGCAAAGAGCAAGGAGCTGGTGGACGAGGCCATCCGCTGCAAG
CTGAAGATCCTGCAGAATGATGGGGTGGTCAACAGCCCTTGCGCCCGCCC
CCGCAAGACGAGCCACGCCCTGTTCCTGTTGGGCGGCCAGACCTTCATGT
GCGACAAGCTCTACCTGGTGGACCAGAAGGCCAAGGAGATCATCCCCAAG
GCGGACATTCCCAGTCCTAGGAAGGAGTTCAGTGCCTGCGCCATCGGCTG
CAAGGTCTACGTGACAGGGGGGA--GGGGCTC-GGAGAACGGCGTGTCCA
AAGATGTGTGGGTGTACGACACNNNNNNNNNNNNNNNNNNNNNNNNNNNNNNNNNNNNNNNNNNNNNNNN

NNNNNNNNNNNNNNNNNNNNNNNNNNNNNNNNNNNNNNNNNNNNNNNNNNNNNNNNNNNNNNNNNNNNNN
NNNNNNNNNNNNNNNNNNNNNNNNNNNNNNNNNNNNGTTTAGTCGCTCCCAAACCAGGTTGATC
CTCAACGAGGCTGAGCTGATCCTGGCTTTGGCGCAGGAGTTTCAGATGAG
GGTGGTGACGGTTTCCATGGAAGAGCAGACCTACTCCAGCATCGTGCAGG
TTATCAGCAGAGCCTCCATGCTGGTTAGCATGCATGGAGCTCAGCTCATC
ACCTCGCTCTTCCTCCCCAGAGGGGCGGCTGTGGTGGAACTCTTCCCCTA
TGCAGTCAACCCTGAGCAGTACACCCCTTACAAGACATTGGCCACATTGC
CAGGTATGGACCTCCAGTATGCTGCCTGGAGGAACACCATGGAGGAGAAC
TCGGTGACCCACCCAGATAGACACTGGGATCAGGGGGGCATCGCTCACCT
GGACATGGATGAGCAGGAGCGCATCCTGGCCAGCAGAGATGTGCCCAGAC
ACCTTTGCTGCAGAAACCCAGAGTGGCTGTTCCGCATCTACCAGGACACC
AGAGTGGATGTCCCCTCTCTGCTGGAAGTCCTCAG---GGAGAGCCTGAA
G---ACTAGGCCCACCCTCAAGAA---GACCCGGCCTGCCAGCACTGTTC
ATCCAGGCAGGGTGAGGGAGCCACAGTGCCANNNNNNNNNNNNNNNNNNNNNNNNNNNNNNNNNNNNNNN
NNNNNNNNNNNNNNNNNNNNNNNNNNNNNNNNNNNNNNNNNNNNNNNNNNNNNNNNNNNNNNNNNNNNNN

----GAGACTCTGACAGCCATCTTAGGGCCTGTAGTGGCCGAGAGGAATG
CCATGAAGAACAGCCGACTCATCCTCTCCATGGGCGGCCTCTCTCGCTGT
TTCCGCTTCCATTTCAGGGGCACGGGCTATGATGAGAAGATGGTGCGTGA
GATGGAGGGCCTGGAGGCATCAGGCTCCACCTATGTCTGCACCCTCTGCG
ATGCCACCAGAGCCGAGGCCTCCCAAAACATGGTGCTCCACTCCGTCACC
CGCAGCCATGAAGAGAACCTGGACCGCTACGAGATGTGGAGGACCAATCC
GTACTCGGAATCAGCCGAGGAGCTGCGAGACCGGGTGAAAGGGGTCTCCG
CCAAGCCTTTCATGGAGACCCGGCCCACGCTGGATGCGCTGCACTGCGAC
ATCGGCAATGCCACCGAGTTCTACAAGATCTTTCAGGATGAGATTGGAGA
GGTGCATCGCAGGCC---CAAC---CCGAGCAGGGAAGAGCGTCGGAGCT
GGAGGGCAGCTCTGGATAAACAGCTCAGGAAGAAGCTGAAGCTGAAG---

-------------TCGTACACCATTGAGATGGGCCCAAAAGGCCCTCAGT
GGAAAGAGAGCCCTCAGCCTTTTTCCTGTTCTGTGGAGGATCCCACCAAA
CAGACCAAATTCAAAGGCATCAAAACCTATATATCTTACCGGGTCACTCC
CAGCCATACAGGACGACCTGTCTACCGCCGGTACAAGCACTTTGATTGGC
TGTACAACCGCCTGCTGCATAAGTTCACCGTCATCTCCGTGCCCCACCTG
CCAGAGAAGCAGGCTACGGGACGCTTCGAGGAGGACTTCATCGAGAAGCG
GAAGAGACGTTTGGTCATCTGGATGGATCACATGACCAGCCATCCTGTCC
TATCACAGTACGAGGGCTTGGAACACTTCCTCATGTGTGCCGATGACAAA
CAGTGGAAGCTGGGCAAGAGGCGGGCGGAGAAAGATGAGATGGTGGGAGC
TCACTTCATGCTGACCTTTCAGATTCCTAACGAGCATCAGGACCTCCAGG
ATGTGGAGGAGAGGGTGGACACCTTCAAGTCTTTTGCCAGGAAGATGGAT
GAAAGCGTCATGCAGCTGACCCACGTGGCCTCAGAACTGGTGAGAAAACA
CCTCGGCGGCTTNNNNNNNNNNNNNNNNNNNNNNNNNNNNNNNNNNNNNNNNNNNNNNNNNNNNNNNNNN
NNNNNNNNNNNNNNNNNNNNNNNNNNNNNNNNNNNNNNNNNNNNNNNN----------------------
----

--ACGGCATTTTC
CTCCCAAGCT---CCCGGCTAC---GCAGCCGCTGCCCTGGGA---CATC
ACCA------CCATCCGACTCATGTCAGTTCT---TACTCTACTGCGGCT
TTCAATTCCACCCGGATTTTCTCTTCAGAAATCGAGGCTTCGGAGACGC
CACCAG------------CGCTCAGCATAGTCTATTCGCCTCTGC---AG
CGGGAAGTTT---T------GCAGCCCCACATGGACACTCAGATGCAGCG
GGACACCTGCTCTTCCCAGGACTTCACGAA---CAAGCAGCGAGCCATGC
TTCCTCAAATGTTGTTAATAGTCAGATGCGATTGGGCTTTTCGGGGGACA
TGTACGGCAGAGCCGACCAGTACGGCCACGTTACCAGCCCGCGGT---CC
GACCACTATGCATCGACCCAGTTGCATGGCTATGGCCCTATGAACATGAA
TATGGCCGCG---CATCATGGAGCAGGGGCCTTCTTCCGTTACATGAGGC
AGCCGATAAAACAAGAGTTGATTTGCAAATGGATCGAACCGGAACAACTA
ACGAATCCAAAAAAGTCGTGCAACAAAACTTTTAGCACAATGCACGAGCT
CGTCACTCATTTGACAGTGGAGCATGTAGGGGGACCGGAACAGTCAAACC
ACATTTGCTTCT--------------------------------------

>Neoscopelus macrolepidotus
AGCCTCTTTATTCGGGCCGAGCTCAGTCAACCGGGAGCCCTCGTGGGGGA
CGACCAGATCTATAATGTAATTGTAACAGCCCATGCCTTTGTAATAATTT
TCTTTATGGTAATGCCTATTATGATTGGAGGGTTTGGAAACTGACTAATC
CCCCTTATAATTGGAGCCCCCGACATAGCATTCCCCCGTATAAATAATAT
AAGCTTCTGACTTCTCCCCCCCTCTTTTCTCCTCCTTTTAGCATCATCTG
GTGTAGAAGCCGGAGCCGGGACCGGTTGAACAGTATACCCCCCCCTTGCA
GGTAACCTCGCCCATGCGGGAGCCTCTGTTGACCTAACAATTTTTTCACT

GCATCTAGCAGGTATCTCTTCAATTTTGGGGGCAATCAACTTCATTACAA
CCATTATTAATATAAAACCCCCTGCCATCTCCCAATACCAGACCCCCTTA
TTTGTATGGGCAGTCCTTATTACGGCCGTTCTTCTCCTCCTCTCCCTACC
CGTCCTAGCCGCTGGTATTACTATACTTCTAACAGACCGAAACCTAAACA
CCACCTTTTTCGACCCAGCAGGCGGGGGAGACCCCATCCTGTATCAACAT
CTCTTCTGATTTTTCGGGCACCCTGAAGTATATATTCTTATTCTTCCGGG
CTTTGGCATAATCTCTCATATTGTTGCGTACTATTCAGGTAAAAAAGAAC
CCTTTGGATATATGGGTATAGTTTGAGCCATGATGGCAATTGGACTTCTC
GGATTCATCGTGTGGGCCCACCATATGTTTACAGTAGGAATGGATGTAGA
TACACGAGCATATTTCTAGAGAGGAACCTTCACCCATCCAACTGCTTGGG
CATGCTGTTGCTGTCAGATGCTCACCAATGTACTAAGTTATCAGAGCTTT
CTTGGGGCATGTGCCTCAGCAACTTCCCTGCCATCTGCAAGACAGAGGAC
TTCCTTCAACTTCCAAAAGACATGGTGGTGCAGCTCTTGTCCCATGAAGA
ACTGGAAACGGAAGATGAGAGACTGGTTTACGAAGCTGCCCTTAACTGGG
TGAACTATGACCTGGAAAGGAGGCACTGTCATCTGCCAGAGCTGCTGAGA
ACAGTCCGTCTGGCTCTGCTTCCTGCCATCTTCCTCATGGAGAATGTTTC
CACGGAGGAGTTGATCAATGCCCAGGCCAAAAGTAAAGAGCTTGTGGATG
AGGCCATCCGCTGCAAACTGAAGATCTTACAGAACGATGGTGTGGTTAAC
AGCCTTTGTGCCCGGCCCAGGAAGACCAGCCATGCTCTCTTCCTGCTAGG
AGGGCAAACCTTCATGTGTGACAAGTTGTACCTGGTGGACCAGAAGGCCA
AAGAGATAATCCCCAAGGCAGACATCCCCAGCCCCAGGAAGGAGTTCAGC
GCCTGTGCCATCGGTTGTAAGGTTTACATCACCGGCGGGA--GGGGCTC-
AGAGAATGGTGTGTCTAAAGATGTGTGGGTCTACGACACCGTCCATGAGG
AGTGGTCTAAGGCAGCACCCATGCTTATTGCTAGGTTTGGCCATGGCTCA
GCTGAGTTGAAACACTGCCTCTACGTGGTGGGAGGTCACACTGCAGCTAC
TGGATGTCTTCCAGCCTCTCCTTCT-------------------------

-----------AAGAAGGATACCAGTAAGGGAACCTTGGAGGATCAAATC
ATCCAGGCCAACCCTGCGCTGGAGGCCTTTGGGAACGCCAAAACAGCCAG
GAATGACAACTCATCCCGTTTTGGAAAATTCATCCGGATCCACTTCGGCA
CAAGCGGTAAGCTATCATCTGCTGACATCGAGACCTACCTGCTGGAGAAG
TCACGTGTCACCTTTCAGCTCAAGGCCGAGAGGAACTATCACATCTTCTT
CCAGATTTTGTCCAATCAGAAGCCGGAGCTGTTGGACATGCTGCTCATCA
CTAACAACCCATACGACTACTCCTACATCTCCCAAGGAGAGGTGACAGTA
GCCTCCATCAACGACTCAGAGGAGCTGATGGCCACGGACAGCGCCTTCGA
TGTGCTCGGCTTCACTCAGGAGGAGAAGATGGGAGTCTATAAGCTGACTG
GTGCCATTATGCACTATGGCAACATGAAGTTTAAGCAGAAGCAGCGTGAG
GAGCAGGCAGAGCCTGACGGCACTGAGGCTGCTGATAAGTCAGCTTACCT
AATGGGGCTGAACTCTGCTGACCTCATCAAAGGACTTTGCCATCCCAGAG
TCAAGGTAGGAAATGAGTATGTCACCAAAGGCCAGGGTGTAGATCAAGTC

TACTACCCCAACAAGGAGGCCTTCAAGTGTGATGAGTGTGGGAAGCACTA
CAACACCAAGCTGGGATACAAGCGCCATGTGGCCATGCACTCCGCTACCG
CCGGGGATTTAACCTGCAAGGTGTGCATGCAGAGTTACGAGAGCACGCCG
GTTCTCTTGGAGCACCTCAAGAGCCACTCGGGAAAGTCTTCGGGTGGTGC
CAAAGAGAAAAAGCACCCTTGTGACCACTGCGACCGACGTTTCTACACTC
GGAAGGATGTGAGACGGCATATGGTGGTCCATACAGGCCGCAAGGACTTT
CTCTGCCAGTACTGTGCCCAGCGGTTTGGCAGGAAGGACCACCTGACGCG
GCATGTGAAGAAGAGCCACTCGCAGGAGCTCCTAAAGATCAAGACAGAGC
CTCCAGATATGTTGGGCTTGCTAGCGTCAGGGTCCCCGCCATGCCCTGTC
AAGGAAGAGCTCAGCCCCATGATGTGTGGCATGGGGCCCAACAAAGACCC
GATGATGGGTAAGTCCTTCCCCAGCGGTGCACCATTTCCCATGAGCATGT
ACAACCCCCACCAT------CTCCAGGCCATGTCCAATTCTGGGGTGGCT
CACCCG------CACCCCTCCCTGATGCCCAGTTCCCTGTCTGCAGCTAT
GGGCATGGGCTGCCACATGGAATATCTCATCTACGCTTCCTTCTCCTTCA
TGGGATGTTTACAAATCAGCGACGGGTCGAACATCGTGAACCTGCTGGCG
AGTAACTCTCCGAGTGTGTCGTACGCGCTCACCCAGCAGAAGTACTTCAG
CAACTACAGCCCTGTGATTGGGTTCTACATTTACGAACCCATCGAGTACT
GGAACGCCACGGTGCAGGAGCACCTGAAGACACTGAGTCACGGCTTCAAT
AAGATCTCCTGGATGGACAACTTCTTCCACTACCTGCGGGTGGTGAATGT
GAGCGCGTCAACCAAGAACGACTTCATCACCATCCTCAAGGGCTCCTTCT
TACGCAGCCCGGAGTACCAGCACTTCACCGAGGACATCATCTTCTCCAAG
A---ACCGCGAGAGCA------ACGAGTATGACATCATCGCCTCGCGCAT
GTACCTGGTGGCGCGCACCACCGAGAAGAAGCGCGAGGAGGTGGTGGAGC
TGCTGGAGAAGCTGCGTCCGCTGATGCTGATCAACAGCATCAAGTTCATC
GCCTTCAACCCCACCTTCGTGTTCATGGACCGCTATAGCTCCTCCGTCAT
ATCGCCCATCCTTACCTCGGGCTTCAGCGTGCTCACCATCCTCATCCTCA
CGTTCTTTCTCGTCATCAACCCGCTGGGGAATTTCTGGCTCATCCTGACG
GTCACGTCCGTGGAGCTGGGCGTGTTGGGTCTGATGNNNNNNNNNNNNNNNNNNNNNNNNNNNNNNNNNN
NNNNNNNNNNNNNNNNNNNNNNNNNNNNNNNNNNNNNNNNNNNNNNNNNNNNNNNNNNNNNNNNNNNNNN
NNNNNNGATGGACACCATCG
GCGGGAGGTACCGCTACCACCTGGCGCTGGCGTCCGCATTGAAGGACCTG
GAGGAGGACGTGATGGAGGGACTGAGAGAGCGAGGGCTGGAGGACAACAC
CGGCACATCGGGCTTCAGCGTGATGATCAAAGAGTCCTGCGACGGCATGG
GAGACGTGAGCGAGAAGCACGGCGGGGGCCCCCCGTCCCCGAGAAGGCG
GTGCGTTTCTCTTTCACCATCATGTCCGTCTCCGTGCGGCCGGACGGGCG
AGCG------------------------------GAGCCGGTCACTATCT
TCACGGAACCGAAGCCCAACTCGGAGCTGTCCTGTAAGCCCCTGTGCCTG
ATGTTCGTGGACGAGTCGGACCACGAGATGCTCACCGCCGTCCTGGGGCC
CGTGGTCGCCGAGCGGAAGGCGATGACGGAGAGCCGGCTCATCCTGTCTA
TGGGCGGCCTCCCCCGCTCCTTCCGCTTCCACTTCCGAGGCACCGGCTAC
GACGAGAAGATGGTGCGCGAGATGGAGGGCCTGGAGGCGTCGGGCTCCAC
CTACGTCTGCACCCTGTGTGACTCCACCCGGGCGGAGGCCTCTCACAACA
TGGTGCTCCACTCCGTCACCCGCAGCCACAGCGAGAACCTGGAGCGCTAC
GAGATCTGGCGGAGCAACCCCTTCTCCGAGTCCGTCGAGGAGCTGCGAGA
TCGGGTGAAGGGGGTCTCCGCCAAGCCCTTCATGGAGACCCAGCCCACTC
TGGACGCGCTGCACTGCGACATCGGCAACGCCACCGAGTTCTACAAAATT
TTCCAGGACGAGATCGGGGAGGTGTTCCAGAGGGC---CAAC---CCCAC
CCGCGAGGAGAGGCGGAGCTGGAGGGCCGCCCTCGACAAGCAGCTGAGGA
AGAAATTGAAGCTCAAGCCTGTGATGCGGATGAACGGGAACTATGCCCGG
AGGCTGATGTCCGAGGAGACCGTGGACGTGGTGTGCGAACTGGTGCCCTC
GGAGGAAAGGCGGGAGGCCCTGAGGGAGCTAATGGGGCTTTACCTCCAGA
TGAAACCCGTGTGGCGTGCCACCCACCCAGCCAAGGAGTGCCCTGACCAG
CTGTGCCGCTACAGTTTCAACTCCCAGCGCTTTGCGGACCTCCTCTCCAC

CACCTTCAAATATAGGTACGACGGAAAGATCACCAATTACCTGCACAAGA
CCCTGGCCCACGTCCCTGAAATCATAGAGAGAGACGGCTCCATCGGAGCA
TGGGCCAGCGAGGGGAACGAGTCAGCCAACAAATCATACACCATTGAGAT
GGGCCCCAAAGGGCCCCTGTGGCAGGAGAGCCCTCAGCCCTTCTCCTGTA
ACATCGAGGACCCCACCAAACAGACCAAGTTCAAGGGCATCAAGACGTAC
ATCTCCTACCGGGTCACGCCGAGCCACACGGGTCGACCCGTGTACAGACG
CTACAAACACTTTGACTGGCTGTACAACCGCTTGCTGCACAAGTTCACCG
TGATCTCCGTCCCCCACCTGCCGGAGAAGCAGGCGACCGGGCGCTTCGAG
GAGGACTTCATAGAAAAGCGCAAGAGACGACTGATCCTCTGGATGAACCA
CATGACCAGTCACCCTGTCCTCTCCCAGTACGAGGGGTTCGAACACTTCC
TGATGTGTGCCGACGACAAGCAATGGAAGCTGGGCAAGCGGCGGGCAGAG
AAGGACGAGATGGTGGGTGCCCACTTCATGCTGACCTTCCAGATCCCCAA
CGAGCACCAGGACCTTCAGGATGTGGAGGAGCGCGTCGACTCCTTCAAGG
CGTTTGCCAAGAAGATGGACGACAGTGTCATGCAGCTGACACACGTGGCC
TCAGAGCTCGTGCGCAAGCACCTTGGGGGGTTCCGCAAGGAGTTCCAGCG
GCTGGGGAACGCCTTCCAGAACATCAGCCAGGCCTTCATGCTGGACCCAC
CACATAGCTCGGACGCCCTCAACAACGCCATCTCCCACCCTCTCGCCACG
TTCCTCAAACTGACCTCTCTGGGTTTTATCATCGGCGTCGGCGTGGTCGG
AAACCTCCTGATCTCCATCCTGCTGGTCAAAGACAAGAGCCTGCACCGGG
CGCCCTACTACTTCCTGCTGGACCTGTGCGCCTCCGACATCCTGCGCTCC
GCCATCTGCTTCCCCTTCGTCTTCACCTCGGTCAAGAATGGATCCGCCTG
GACGTACGGCACGCTGACCTGCAAAGTGATCGCCTTCCTGGGGGTGCTCT
CCTGTTTCCACACGGCGTTTATGCTGTTCTGCGTGAGCGTCACGCGATAC
TTGGCCATCGCGCACCACCGTTTCTACACCAAGAGAGTGACCTTTTGGAC
CTGTCTGGCCGTCATCTGCATGGTGTGGACGTTGTCGGTTGCGATGGCGT
TCCCGCCGGTGCTAGACGTAGGGACGTACTCTTTCATACGCGAGGAGGAC
CAGTGCACGTTCCAGCACCGCTCCTTCAGGGCGAACGATTCGCTGGGCTT
TATGCTCCTGCTGGCTCTCATCCTCCTGGCCACACAGCTGGTTTACCTCA
AGCTCATCTTCTTCGTCCACGACCGCCGGAAGATGAAGCCCGTCCAGTTC
GTGCCTGCCGTCAGCCAGAACTGGACTTTCCACGGGCCAGGCGCCAGCGG
GCAGGCGGCGGCTAACTGGCTGGCCGGGTTCGGTCGAGGCCCCACCCCGC
CTACCTTGCTGGGCATCCGGCAGAACAGCAACGCGGCGGGCCGCAGGCGT
CTACTGGTGTTGGATGAATTCAAAACGGAGAAGAGGATTAGTAGGATGTT
CTACATCATGACGTTTTTCTTCCTGGCGCTGTGGGGGCCCTATCTGGTCG
CCTGCTACTGGCGGGTGTTTGCGAGGGGCCCCGTGGTCCCCGGGGGTTAC
CTGACGGCGGCCGTGTGGATGAGCTTTGCCCAGGCCGGGGTCAATCCTTT
CATCTGCATCTTCTCCAACAGGGAGGCCAAGTCCCGCTTCCACCCGGGCG
TCGGGAACGCTCCTGGCACGGAGC---GCAGCGTCTCACTCAGCAACAGC
TTGCTGTCTCCGCAACAAACCGAAGAACCCACTGTCG---CTCCCCCACA
GCGCTGGTTTGTCACCC---CCGCCAACAACCGACTGGACTTTGCAGCCT
CGGCATACGATGCCGCT---------GATTTCGCCGGTAACGCAGCCACC
TTGCTGTCCTACGCAGCGGCTGGAGTGAAGGCTC------TTCCTCTGCC
GACCGCAGGTTGCTCCAACAGACCTCTGGGGTATTACGCAGACCCGTCCG
---GCTGG---GGAACACGCACACCGCCTCAGTACTGT---------AGC
AAATCAGCCTCGGTCCTCTCCTGCTGGCCCACGAATTCTGTTGGAGGCAG
AACGGGCA------CTAACTACCTGG---------CGGAGGA---TGGA-
--GACGC---CATCCCCACGGAGAGATCCCCA---AT---GAACGGCTCT
GAGGAG---ACCAAAGCCAAAGACTT------GTCTGA---GTCGAGCTG
GATAGAG---ACGCCATCTTCGATAAAGTCCATTGATTCAAGTGATTCTG
GAATCTTTG---AACAAGCAAAGCGGAGAAGGATTTCACCGTCTGCTACA
CCA---------GTTACAGACACTGTGTCCCCGTTAAAATCTGAG-----

>Neoscopelus microchir
AGCCTCCTCATTCGAGCCGAACTCAGTCAACCAGGAGCCCTCTTGGGGGA
TGACCAGATTTATAATGTAATTGTAACAGCCCATGCCTTTGTAATAATTT
TCTTTATGGTAATGCCTATTATAATTGGGGGGTTCGGAAACTGACTAATT
CCCCTTATAATTGGGGCCCCTGACATGGCATTCCCTCGAATAAATAATAT
GAGCTTCTGACTCCTGCCCCCCTCTTTCCTCCTACTCTTAGCATCCTCAG
GCGTAGAAGCCGGGGCCGGAACCGGCTGGACAGTCTACCCCCCGCTTGCA
GGCAACCTCGCCCATGCAGGAGCCTCTGTTGACCTAACAATCTTCTCCCT
TCATCTAGCAGGTATTTCTTCAATTTTAGGAGCAATCAACTTCATTACAA
CCATTATTAATATAAAACCCCCCGCCATCTCCCAGTACCAAACCCCCCTG
TTTGTGTGAGCAGTACTTATTACAGCCGTCCTTCTCCTTCTCTCGCTCCC
CGTCTTAGCTGCTGGAATTACCATACTCTTAACAGACCGAAACCTCAACA
CCACCTTTTTCGACCCAGCAGGCGGGGGAGACCCCATCTTATACCAACAC
CTTTTCTGGTTTTTTGGACACCCTGAAGTATATATTCTTATTCTTCCAGG
CTTTGGGATGATTTCTCATATTGTTGCATACTATTCAGGTAAAAAAGAAC
CCTTTGGGTATATGGGCATGGTCTGAGCCATAATGGCTATTGGACTTCTC
GGCTTCATCGTATGGGCCCACCACATGTTTACAGTTGGGATGGACGTAGA
TACACGAGCATATTTCTAGAGAGGAACCTTCACCCATCCAACTGCTTGGG
CATGCTGTTGCTGTCAGATGCTCACCAATGTACTAAGTTATCAGAGCTTT
CTTGGGGCATGTGCCTCAGCAACTTCCCTGCCATCTGCAAGACAGAGGAC
TTCCTTCAACTTCCAAAAGACATGGTGGTGCAGCTCTTGTCCCATGAAGA
ACTGGAAACAGAAGATGAGAGACTGGTTTACGAAGCTGCCCTTAACTGGG
TGAACTATGACCTGGAAAGGAGGCACTGTCATCTGCCAGAGCTGCTGAGA
ACAGTCCGTCTGGCTCTGCTTCCTGCCATCTTCCTCATGGAGAATGTTTC
CACGGAGGAGTTGATCAATGCCCAGGCCAAAAGTAAAGAGCTTGTGGATG
AGGCCATCCGCTGCAAACTGAAGATCTTACAGAACGATGGTGTGGTTAAC
AGCCTTTGTGCCCGGCCCAGGAAGACCAGCCATGCTCTCTTCCTGCTAGG
AGGGCAAACCTTCATGTGTGACAAGTTGTACCTGGTGGACCAGAAGGCCA
AAGAGATAATCCCCAAGGCAGACATCCCCAGCCCCAGGAAGGAGTTCAGC
GCCTGTGCCATCGGTTGTAAGGTTTACATCACCGGTGGGA--GGGGCTC-
AGAGAATGGTGTGTCTAAAGATGTGTGGGTCTACGACACCGTCCATGAGG
AGTGGTCTAAGGCAGCACCCATGCTTATTGCTAGGTTTGGCCATGGCTCA
GCCGAGTTGAAACACTGCCTCTACGTGGTGGGAGGTCACACTGCAGCTAC
NNNNNNNNNNNNNNNNNNNNNNNNNGGACGAGTACATTGTGGTGTTCAGTCGTTCGACGACGAGACTCAT
TCTCA
ACGAAGCGGAACTGATTATGGCGCTGTACCAGGAGTTTCAGATGAGGGTG

GTCACCGTGTCCCTTGAAGAGCAATCTTTCCCCAGTATAGTCCAGCTTAT
CAGTGGTGCCTCCATGTTGGTCAGTATGCATGGGGCTCAGCTTGTCACCT
CACTCTTCCTCCCCAGAGGAGCTGTGGTGGTGGAGCTCTTTCCCTATGCC
GTGAACCCGGAACAGTACACCCCATATAAAACCCTCGCCTCCCTACCAGG
GATGGACCTGCAGTATGTTTCCTGGAGGAATTTGAGGGAGGAAAACACTG
TCACCCACCCAGATAGACCCTGGGACCAAGGAGGCATCGCCCACCTGGAA
AAAGACGAGCAAGAGCGAATCCTCACTAGCAAGGATGTCCCGCGGCACCT
GTGCTGCCACAACCCAGAATGGTTTTTCCGAATCTACCAGGACACTCTGG
TGGACATCCCTTCATTCCTGGAAGTTCTCAA---AGAGGGCCTGAAG---
ACCAGACCTAGCTTAAAAAA---GGCCAAGCCAGCCAGCACAGTACATCC
AGGCCGGGTCAGGGAAGCACAGTGTCAAACCTCGGTCCAAGCCACCAACG
AAGCTAAACTCACAGTCTCCTGGCAGATCCCGTGGAACCTGAAATACCTG
AAGGTGAGAGAGGTGAAGTACGANNNNNNNNNNNNNAAGAAGGACACCAGTAAGGGAACCTTG
GAGGATCAAATCATCCAGGCCAACCCTGCGCTGGAGGCCTTTGGGAACGC
CAAAACAGCCAGGAATGACAACTCATCCCGTTTTGGAAAATTCATCCGGA
TCCACTTCGGGACAAGCGGTAAGCTATCATCTGCTGACATCGAGACCTAC
CTGCTGGAGAAGTCACGTGTCACCTTTCAGCTCAAGGCCGAGAGGAACTA
CCACATCTTCTTCCAGATTTTGTCCAATCAGAAGCCGGAGCTGTTGGACA
TGCTGCTCATCACTAACAACCCATACGACTACTCCTACATCTCCCAAGGA
GAGGTGACAGTAGCCTCCATCAACGACTCTGAGGAGCTGATGGCCACGGA
CAGCGCCTTCGATGTGCTCGGCTTCACTCAGGAGGAGAAGATGGGAGTCT
ATAAGCTGACTGGTGCCATTATGCACTACGGCAACATGAAGTTTAAGCAG
AAGCAGCGTGAGGAGCAGGCAGAGCCTGACGGCACTGAGGCTGCTGATAA
GTCAGCTTATCTAATGGGGCTGAACTCTGCTGACCTCATCAAATGTCTTT
GCCATCCCAGAGTCAAGGTAGGAAATGAGTATGTCACCAAAGGCCAGGGT
GTAGATCAAGTCTACTACCCCAACAAGGAGGCCTTCAAGTGCGAGGAGTG
TGGCAAGCACTACAACACCAAGCTGGGCTACAAGCGCCATGTGGCCATGC
ACTCCGCCACGGCGGGGGACCTCACTTGCAAAGTGTGCATGCAGAGCTAC
GAGAGCACGCCCGTGCTCCTGGAGCACCTCAAGAGCCACTCGGGGAAGTC
CTCGGGAGGCGCCAAGGAGAAGAAGCACCCGTGCGACCACTGCGACCGCC
GCTTCTACACGCGGAAGGACGTCAGGCGGCACATGGTGGTGCACACGGGC
CGGAAGGACTTCCTGTGCCAGTACTGCGCGCAGCGCTTCGGCCGGAAGGA
CCACCTGACGCGCCACGTGAAGAAGAGCCACTCGCAGGAGCTGCTGAAGA
TCAAGACGGAGCCTCCGGACATGCTGGGTCTGCTGGGGTCCGGCTCGCCG
CCCTGCTCCATCAAGGAGGAGCTCAGTCCCATGATGTGCAGCATGGGTCC
CAACAAGGACCCCATGATGGGCAAGCCCTTCCCCAGCGGGACCCCTTTCC
CCATGGGCATGTACAACCCCCACCAC------CTCCAGGCCATGTCCAGT
CCCGGGGTGGGACACCCC------CACCCCTCCCTGATGCCCAGCTCCCT
GTCAGCGGCCATGGGCATGGGCTGCCACATGGAGTATCTCATCTACGCTT
CCTTCTCCTTCATGGGATGTTTACAAATCAGCGACGGGTCGAACATCGTG
AACCTGCTGGCGAGTAACTCTCCGAGCGTGTCGTATGCGCTCACCCAGCA
GAAGTACTTCAGCAACTACAGCCCTGTGATTGGGTTCTACATTTACGAAC
CCATCGAGTACTGGAACGCCACGGTGCAGGAGCACCTGAAGACACTGAGT
CACGGCTTCAACAAGATCTCCTGGATGGACAACTTCTTCCACTACCTGCG
GGTGGTGAATGTGAGCGCGTCGACCAAGAATGACTTCATCACCATCCTCA
AGGGCTCCTTCTTACGCAGCCCGGAGTACCAGCACTTCACCGAGGACATC
ATCTTCTCCAAGA---ACCGCGAGAGCA------ACGAGTACGACATCAT
CGCCTCGCGCATGTACCTGGTGGCGCGCACCACCGAGAAGAAGCGCGAGG
AGGTGGTGGAGCTGCTGGAGAAGCTGCGTCCGCTGATGCTGATCAACAGC
ATCAAGTTCATCGCCTTCAACCCCACCTTCGTGTTCATGGACCGCTATAG
CTCCTCCGTCATATCGCCCATCCTTACCTCGGGCTTCAGCGTGCTCACCA
TCCTCATCCTCACTTTCTTTCTCGTCATCAACCCGCTGGGGAATTTCTGG
CTCATCCTGACGGTCACGTCCGTGGAGCTGGGCGTGTTGGGTCTGNNN-----

--TCATACACCATTGAGATGGGCCCCAAAGGGCCCCAGTGGCAGGAGAGC
CCTCAGCCCTTCTCCTGTAACATTGAGGACCCCACCAAACAGACCAAGTT
CAAGGGCATCAAGACGTACATCTCCTACCGGGTCACGCCGAGCCACACGG
GTCGACCCGTGTACAGACGCTACAAACACTTTGACTGGCTGTACAACCGC
TTGCTGCACAAGTTCACCGTGATCTCCGTCCCCCACCTGCCGGAGAAGCA
GGCGACCGGGCGCTTCGAGGAGGACTTCATAGAAAAGCGCAAGAGGCGAC
TGATCCTCTGGATGAACCACATGACCAGTCACCCTGTCCTCTCCCAATAC
GAGGGGTTTGAACACTTCCTGATGTGTGCCGACGACAAGCAATGGAAGCT
GGGCAAGCGGCGGGCAGAGAAGGACGAGATGGTCGGTGCCCACTTCATGC
TGACCTTCCAGATCCCCAACGAGCACCAGGACCTTCAGGATGTGGAGGAG
CGTGTCGACTCCTTCAAGGCGTTTGCCAAGAAGATGGACGACAGTGTCAT
GCAGCTGACACACGTGGCCTCAGAGCTTGTGCGCAAGCACCTTGGGGGGT
TCCGCAAGGAGTTCCAGCGGCTGGGGAACGCCTTCCAGAACATCAGCCAG
GCCTTCATGCTGGACCCACCACATAGCTCGGACGCCCTCAACAACGCCNNNNNNNNN--

-----------------------------------GCCAAGTCCCGCTTC
CACCCTGGCGTCGGGGCCGCTGCTGGCACGGAGC---GCAGCGTCCCACT
CAGCAACAGCTTGCTGTCTCCGCAACAAACCGAAGAACCCACTGTCG---
CCTCCCCACAGCGCTGGTTTGTCACCC---CCGCCAACAACCGACTGGAC
TTTGCAGCCTCGGCATACGATGCCGCT---------GATTTCGCCGGTAA
CGCAGCCACCTTGCTGTCCTACGCAGCGGCTGGAGTGAAGGCTC------
TTCCTCTGCCGACCGCAGGTTGCTCCAACAGACCTCTGGGGTATTACGCA
GACCCGTCCG---GCTGG---GGAACACGCACACCGCCGCAGTACTGT--
-------AGCAAATCAGCCTCGGTCCTCTCCTGCTGGCCCACGAATTCTG
TTGGAGGCAGAACAGGCA------CTAACTACCTGG---------CGGAG
GA---TGGA---GACGC---CATCCCAACGGAGAGATCCCCA---AT---
GAACGGCTCTGAGGAG---ACCAAAGCCAAAGACTT------GTCTGA--
-GTCGAGCTGGATAGAG---ACGCCATCTTCGATAAAGTCCATTGATTCC
AGTGATTCTGGAATCTTTG---AACAAGCAAAGCGGAGAAGGATTTCACC
GTCTGCTACACCA---------GTTACAGACACTGTGTCCCCGNNNNNNNNNNNN-------
--ACAGGCGAAGTCACAGAGAGAGAAGTGGCGTTGGGGATAAACCCGTTC
GCGGACGGGATGGGCGCCTTCAAAATCAACCACAGCTCTCACGACATCGG
CTCCGG---ACAGACGGCGTTCTCCTCCCAGGCG---CCCGGCTAC---G
CGGCGGCTGCTCTGGGA---CACCACCA------CCACCCGACCCACGTC
AGCTCC---TATTCCACAGCAGCTTTCAACTCCACCAGGGACTTTCTCTT
CAGAAATCGGGGCTTCGGAGACGCTACCAG------------CGCCCAGC
ACAGTTTGTTCGCGTCCGC---CGCGGGAAGTTT---T------GCAGGG
CCCCACGGACACTCAGACGCCGCTGGACACCTGCTCTTCCCCGGGCTGCA
CGAA---CAAGCCGCCAGCCACGCGTCCTCTAATGTTGTCAACAGCCAGA
TGCGACTGGGCTTTTCGGGGGACATGTACGGACGGGCCGACCAGTACGGC
CACGTTACAAGCCCGAGGT---CCGACCACTATGCATCGACCCAGTTGCA
CGGCTATGGCCCTATGAACATGAATATGGCCGCA---CACCACGGAGCAG
GGGCCTTCTTTCGATACATGAGGCAGCCGATCAAACAAGAGCTCATCTGC
AAGTGGATCGAACCGGAGCAACTAACGAATCCCAAAAAGTCGTGCAACAA
AACTTTCAGCACGATGCATGAGCTCGTCACCCATCTGACGGTGGAACATG
TGGGGGGGCCAGAACAGTCGAACCACATTTGCTTCTGGGAGGACTGCTCT
CGAGAAGGGAAACCATTCAAAGCCAAATACAAACTAGTGAATCATATCAG
AGTTCACACCGGAGAAAAGCCCTTTCCATGCCCCTTCCCCGGCTGTGGCA
AA
>Nezumia bairdii

-------------------------GGAAGATTACATTGTGGTCTTCTCT
CGCTCCACAACAAGGTTGATCCTAAATGAAGCGGAGCTGATCATGGCGCT
GGCCCAGGAGTTTCAGATGCGAGTCTTGACGGTGTCCCTGGAGGAGCAGT
CTTTTGCCAGCATCGTGCAGGTGATCAGCGGAGCAGCCATGCTGTTCAGT
ATGCACGGTGCTCAGCTCATCACAGCGCTGTTCCTGCCCAGAGGTGCAGC
CGTGGTGGAGCTGTTCCCCTACGCCGTGAACCCGGAGCAGTACACCCCAT
ATAAAACCCTTGCCTCCCTGCCGGGCATGGACCTCCAGTACGTCTCCTGG
AGGAACACAATGGAGGAGAACACCGTCACTCACCCGGACCGGCCCTGGGA
CCAAGGGGGCGTTGTGCACTTGGAGAAGGAGGAACAAGAGCGCATACTAG
CCAGCAAGGACGTCCCCAGGCACCTGTGCTGCCGTAACCCAGAGTGGCTT
TTCAGAATCTACCAGGACACTTTGGTGGACATCCCTTCACTCCTCGAGGT
GCTCAA---GGAGGGCCTCAGA---ACCAGGCCCAACTTGAAAAA---GA
GCAAGGCGGCCAGCACGGTTCACCCGGGGCGGGTCCGAGAACCCCAGTGC
CAGACCTCGGTCCAAGCCACCAACGAGGCCAAACTCACAGTGTCCTGGCA
GATCCCGTGGAACCTGAAGTTCCTGAAGGTGAGAGAGGTGAAGTACGAAG
TG--

------CCCAACAAAGAGGCCTTCAGGTGCGAGGAGTGCGGCAAGCACTA
CAACACCAAGCTGGGCTACAAACGCCATGTGGCCATGCACTCGGCCACGG
CGGGCGACCTGACCTGCAAGGTGTGCCTGCAGAGCTACGAGAGCACGCCG

GCGCTGCTGGAGCACCTCAAGAGCCACTCGGGCAAGTCGTCGTCGGCGCG
CAAGGAGAAGAAGCACCCGTGCGACCACTGCGACCGCCGCTTCTACACGC
GCAAGGACGTGCGCCGCCACATGGTGGTGCACACGGGCCGCAAGGACTTC
CTGTGCCAGTACTGCGCCCAGCGCTTTGGCCGCAAGGACCACCTGACGCG
GCACGTGAAGAAGAGTCACTCGCAGGAGCTGCTGAAGATCAAGACGGAGC
CGGCGGACATGCTGGGGCTGCTGAGCTCCGGGTCGCCGCCCTGCTCCATC
AAGGAGGAGCTCAGCCCCATGATGTGCAGCATGGGTCCCAGTAAGGACCC
CATGATGGGCAAGCCCTTCCCCAGCGGCACACCCTTCCCCATGGGCATGT
ACAACCCCCACCAC------CTCCAGGCCATGTCCAACTCCGGGGTGGGC
CACCCT------CACCCTTCCCTGATGCCCACCTCGCTGTCTGCGGCCAT
GGGCATGGGCTGCCACATAGAGTACCTCATCTACGCCTCCTTCTCCTTCA
TGGGCTGCTTACAGATCAGCGATGGCTCCAACGTCGTCAACCTGCTGGCC
AGCAACTCCCCGAGCGTGTCCTACGCGCTGACGCAGCAGAAGTACTTCAG
CAACTACAGTCCCGTCATCGGCTTCTACATCTACGAGCCCATCGAGTACT
GGAACTCCACGGTGCAGGAGCACCTGAAGACCCTGAGCCATGGCTTCAAC
AAGATCTCCTGGATGGACAACTTCTACCACTACCTGCGCGTGGCCAACGT
CAGCGCGTCCACCAAGAACGACTTCATCCACATCCTGAAGGGCTCCTTCT
TGCGGAGCCCGGAGTATCAGCACTTCACAGAGGACATCATCTTCTCCAAG
A---ACCGCGAGAGCG------ACGAGTACGACATCATCGCCTCCCGCAT
GTACCTGGTGGCCCGGACCACGGAGAAGAAACGCGAGGAGGTGGTGGAGC
TGCTGGAGAAGCTTCGGCCGCTGATGCTCATCAACAGCATCAAGTTCATC
GCCTTCAACCCCACCTTCGTGTTCATGGACCGCTACAGCTCCTCCGTCAT
CTCACCCATCCTCACCTCCGGCTTCAGCGTGCTCATCATCCTCATCCTCA
CCTTCTTCCTGGTCATCAACCCCCTGGGGAACCTCTGGTTGATCCTGACC
GTGACCTCGGTGGAGCTGGGCGTCCTGGGACTGATG--------------

---TCGTACA

CGGTGGAGATCGGCCCCAAGGGCCCCCAGTGGCAGGAGAGCCCCCAGCCC
TTCTCCTGCTCCATCGAGGACCCCACCAAACAGACTAAGTTCAAGGGCAT
CAAGACCTACATCTCGTACCGCGTGACGCCCAGCCACACCGGCATGGCCG
TGTACCGCCGCTACAAACACTTTGACTGGCTGTATAACCGTCTGCTGCAC
AAGTTCACCGTGATCTCCGTGCCCCACCTGCCCGAGAAGCAAGCCACGGG
CCGCTTTGAGGAGGACTTCATCGACAAGCGCAAGCGGCGGCTGGTCCTGT
GGATGAACCACATGACCAGCCACCCGGTGCTGTCGCAGTACGAGGGCTTC
GAGCACTTCCTCATGTGCGCCGACGACAAGCAGTGGAAGCTGGGAAAGCG
GCGGGCGGAGAAGGACGAGATGGTGGGCGCCCATTTCATGCTCACCTTCC
AGATCCCCAACGAGCACCAGGACCTGCAGGACGTGGAGGAACGTGTGGAC
TCCTTCAAGGCCTTCACCAAGAAGATGGACGACAGCGTCATGCAGCTGAC
GCATGGCGCCTCGGAGATGGTGCGCCGGCACCTGGGAGAGTTTCGGAAGG
AGTTCCACCGGCTGGGAGGTGCCTTCCAGTCGCTGTCGCAGGCCTTCATG
CTGGAGCCGCCGCACAGCTCAGAGCCACTCAACAACGCCATCTCGCAC--

>Normichthys yahganorum

-------------------------GGACGAGTACATCGTGGTTTTCAGC
CGCTCCGTCAACCGGCTCATCCTCAACGAGGCCGAGTTGATCCTGGCTCT
GGCGCAAGAGTTCCAGATGAGGGCCGTCACGGTGTCCCTGGAGGAGCAGT
CGTTCGCCAGCATCGTTCAGGCGATCAGCGGGGCGTCCATGCTGGTCAGC
GTCCATGGAGCCCAGCTGGTCAGCTCCCTGTTCCTGCCGCGTGGCGCTGC
CGTGGTGGAGCTCTTCCCCTACGCCGTCAACCCGGAGCAGTACACTCCAT

ACAAAACCCTGGCCCTGTTACCAGGCATGGACCTCCAGTACGTGGCTTGG
AGGAACACCATGGAGCAGAACTCGGTGGCTTACCCCGAGCGCGCCTGGGA
CCAGGGTGGCATCGCCCACCTGGAGAAGGAGGAGCAGGAGCGCATCCTGG
CGAGCGACGACGTGCCGCGGCACCTGTGCTGCCGCAACCCAGAGTGGCTC
TTCCGGATTTACCAGGACACCCAGGTGGACGTCCCCTCCTTGCTGGAGGT
CCTCCG---AGAAAATCTGAAG---GCCAAGCCTAACCTGAGAAA---GG
CCAAGGCAGCTAGTACAGTACACCCCGGTAGAGTCAGGGAGCCCAAGTGC
CAGACCTCGGTCCAGGCCACCAACGAGGCCAAGCTGACAGTGTCGTGGCA
GATCCCCTGGAACCTCAAATACC---------------------------
-----------AAGAAGGATGCCAGCAAAGGGACCTTGGAGGATCAAATC
ATTCAAGCTAACCCAGCACTGGAGGCTTTTGGCAATGCCAAAACAGTGAG
AAATGACAACTCCTCACGCTTTGGGAAGTTCATTCGTATTCATTTTGGAA
CGAGCGGCAAACTCTCCTCTGCTGACATAGAAACTTACTTGCTTGAGAAA
TCCCGTGTGACCTTTCAGCTCAAATCGGAGAGGAACTACCACATCTTCTT
CCAGATATTGTCCAATGAAAAGCCAGAGCTGCTGGACATGCTGTTGATTA
CCAACAACCCTTATGATTATTGCTTCATCTCCCAAGGAGAAGTAACAGTT
AAATCTATCAATGACAGTGAGGAGTTGCTTGCCACTGACAGTGCCTTTGA
TGTGCTTGGCTTCACTCCAGAGGAGAAGATGGGGATCTATAAGTTGACAG
GTGCCATTATGCACTATGGCAACATGAGGTTCAAGCAGAAGCAGCGTGAG
GAGCAGGCAGAGCCTGATGGCACTGAGGCAGCTGACAAGTCAGCCTACCT
GATGGGGCTTAACTCTGCTGATCTTGTGAAGGGACTCTGCCATCCCAGGG
TCAAAGTCGGCAATGAGTATGTAACAAAGGGACAAGGTGTAGATCAAGTC
TATTACCCCAACAAGGAGGCCTTCAAGTGCGAGGAGTGCGGCAAGCACTA
CAACACCAAGCTGGGCTACAAGCGGCACGTGGCCATGCACTCGGCCACGG
CGGGCGACCTCACCTGCAAGGTGTGCCTGCAGAGCTACGAGAGCACGCCG
GCGCTGCTGGAGCACCTCAAGAGCCACTCGGGCAAGTCGTCGGGCGGCGC
CAAGGAGAAGAAGCACCCGTGCGACCACTGCGACCGCCGCTTCTACACGC
GCAAGGACGTGCGCCGCCACATGGTGGTGCACACGGGCCGCAAGGACTTC
CTGTGCCAGTACTGCGCGCAGCGCTTCGGCCGCAAGGACCACCTGACGCG
CCACGTCAAGAAGAGCCACTCGCAGGAGCTGCTGAAGATCAAGACGGAGC
CTCCGGACATGCTGGGCCTGCTGGGCTCGGGCTCGCCGCCCTGCGCCATC
AAGGAGGAGCTCAGCCCCATGATGTGCAGCATGGGCCCCGGCAAGGACCC
CATGATGGCCAAGCCCTTCCCCAGTGGCACCCCCTTCCCCATGGGCATGT
ACAACCCCCACCAC------CTCCAGGCCATGTCCAACCCCGGAGTGGGC
CAC---------CACCACTCCCTGGTGCCCGGCTCCCTGTCGGCCGCCAT
GGGGATGGGCTGCCACATGGAGTACCTGATTTACGCTTCCTTCTCGTTCA
TGGGATGTTTACAAATCAGCGACGGATCCAACATAGTTAACCTTTTGGCC
AGCGACTCGCCGAGCGTGTTGTACGCTCTGACCCAGCAGAAGTATTTCAG
CAACTACAGCCCAGTGATAGGGTTCTACATCTATGAGCCCATTGACTACT
GGAACGCCACTGTGCAGGAGCACCTCAAGACACTGGGCCAGGGGTTCAAT
ACGATATCGTGGATCGATAATTACTTTCAGTATCTGAAGGTGACGAACGT
CAGCGCGTCGACCAAAAACGACTTCATCGCCGTCCTCAAGACCTCGTTCC
TGAGGAGCCCCGAGTATCAGCACTTCACGGACGACATCATCTTTTCCAAA
A---CGGGGG------------ACGACTTCAACATCATCGCGTCCAGGAT
GTACCTGGTGGCACGGACCACAGAGAAGACCCGGGAGGAGGTGGTGGAGC
TGCTGGAGAGGCTCCGGCCGCTCTCGCTCATCAACAGCATCAAGTTCATC
GTGTTCAACCCCACCTTCGTGTTCATGGACCGCTACAGCTCCTCGGTCGT
CTCGCCCATCATGACGTCCGGCTTCAGCGTCCTGACCATCCTCGTGCTCA
CGTTCTTCCTCGTCGTCAACCCCCTGGGAAACTTCTGGTTGATACTGACC
GTCACCTCCGTGGAGCTGGGAGTCCTGGGCCTGATG--------------


```
--------------------------------------------------
--------------------------------------------------
--------------------------------------------------
--------------------------------------------------
--------------------------------------------------
--------------------------------------------------
--------------------------------------------------
--------------------------------------------------
--------------------------------------------------
--------------------------------------------------
--------------------------------------------------
--------------------------------------------------
--------------------------------------------------
--------------------------------------------------
--------------------------------------------------
--------------------------------------------------
--------------------------------------------------
--------------------------------------------------
--------------------------------------------------
--------------------------------------------------
--------------------------------------------------
--------------------------------------------------
--------------------------------------------------
--------------------------------------------------
-------------------------------------------TCCTACA
CCATAGAGATGGGCCCAAGAGGGCCTCAGTGGAAGGAGAGCCCCCAGCCT
TTCGCTTGCTCCATCGAGGACCCCACTAAGCAAACCAAGTTCAAGGGGAT
TAAGACTTACATATCGTACCGCGTGACTCCCAGCCACGTCGGCCGGCCTG
TGTACCGTCGCTACAAGCACTTTGACTGGCTGTATAACAGGTTGCTGCAC
AAATTCACCGTCATCTCTGTGCCCCATCTGCCCGAGAAGCAGGCGACCGG
GCGTTTTGAGGAGGACTTTATTGAAAAGCGTAAGAGGCGGCTCATCCTCT
GGATGGACCACATGACCAGTCACCCTGTCCTCTCGCAGTACGAGGGCTTC
GAGCACTTCTTCATGTGCGGTGATGATAAACAGTGGAAGCTGGGCAAGCG
GAGGGCGGAGAAGGACGAGATGGTGGGAGCCCACTTCATGCTCACCTTCC
AGATCCCCAACGAGCACCAGGACCTGCAGGACGTGGAGGAGCGGGTGGAC
TCCTTCAAGGCCTTCGCCAAGAAGATGGACGACAGCGTCATGCAGTTAAC
GCACGTGGCCTCCGAGCTGGTGCGCAAACACCTGGGAGGGTTCCGCAAAG
AGTTCCAAAGGCTGGGGAACTCCTTCCAGTCCATCAGCCAGGCGTTCATG
CTGGACCCCCCCTACAGCTCTGACGCCCTGAATAATGCCATCTCCCAC--
--------------------------------------------------
--------------------------------------------------
--------------------------------------------------
--------------------------------------------------
--------------------------------------------------
--------------------------------------------------
--------------------------------------------------
--------------------------------------------------
--------------------------------------------------
--------------------------------------------------
--------------------------------------------------
--------------------------------------------------
--------------------------------------------------
--------------------------------------------------
```


-----TGGCGTCGGCACGGGGCCTGGCACGGACC---GCAGCGTCCCACT
C---AACAGCTTGCTATCCCCGCAACAAACCGATGAGACCGCAGTGG---
CCTCCCCGCAGCGATGGTTTGTCACCC---CTGCCAACAACCGACTGGAC
TTTGCCGCTTCGGCATACGATGCCGCCGCTGCAGCTGATTTTGCCGGTAA
CGCGGCCACCCTTCTGTCGTACGCAGCTGCCGGAGTGAAAGCGC------
TCCCACTGCCCACTGCAGGCTGCTCCAACAGGGCTCTGGGCTATTACGCG
GAGCCGCCAG---GGTGG---GGCACACGCACTCCACCGCAGTACTGT--
-------AGTAAATCAAGCGCGGTTCTCTCATGCTGGCCCGCCAATTCCG
TTGGGTGCAGAACATCCA---CCTCCAATTACCTGG---TTGGCTTGGAC
GA---CGGG---GACGC---TATCGCACCTGAGAGGTCACCT---CT---
CGGGGGGGCAGACGAA---GCCAAGCCAAAAGACCT------GTCGGA--
-GTCAAGCTGGATAGAG---ACATCGTCTTCAATTAAGTCAATCGACTCA
AGTGATTCTGGGATCTTTG---AGCAGGCCAAACGGAGGAGGATTTCGCC
ATCTGCTACACCG---------GTTTCAGAGACGTCGTCCCCATTAAAAT
CAGAA-----------------AGTCACAGACAGAGAAGTGGCTATGGGG
ATAAATCASTTCGCCGACGGGATGGGCGCTTTCAAAATCAACCACAGCTC
CCACGATATTGGCTCTGG---TCAAACGGCGTTTGCCTCGCAGGCG---C
CCGGCTAC---GCAGCCGCTGCCCTGGGA---CACCATCA------CCAC
CCAACCCATGTCAGCTCC---TACTCCACCGCGGCGTTCAACTCCACCCG
GGACTTTCTCTTTCGGAATCGGGGATTCGGAGACGCCACTAG--------
----CGCGCAGCACAGTCTCTTCGCCTCAGC---TGCGGGAAGTTT---C
------GCTGGGCCACATGGACACACCGATGCCACGGGACACCTGCTCTT
CTCGGGACTGCACGAG---CAAGCGGCGACCCACGCGTCTTCGAACGTGG
TGAACAGTCAGATGCGCCTGGGCTTTTCGGGGGACATGTACGGCAGAGCC
GAGCAATATGGTCACGTAACGAGCCCCCGGT---CCGAGCACTACGCTTC
GACTCAGTTGCACGGCTACGGCCCCATGAACATGAATATGGCTGCC---C
ACCACGGGGCAGGGGCCTTCTTCCGTTACATGAGGCAGCCGATCAAACAA
GAGCTCATCTGCAAGTGGGTCGAACCAGAGCAGCTGTCGAATCCGAAAAA
GTCCTGCAACAAAACTTTCAGCACGATGCATGAGCTCGTGACCCACCTCA
CAGTGGAACATGTCGGGGGACCGGAACAGTCGAATCACATTTGCTTTTGG
GAAGAGTGTCCGCGAGAAGGGAAACCATTTAAAGCCAAGTACAAACTTGT
AAATCATATCAGAGTACACACCGGAGAGAAGCCGTTTCCATGTCCATTCC
CCGCTGTGGCAAGT
>Ogcocephalus nasutus

-------------------------GGATGAATTCATTGTTGTGTTTCGT
CGTTCAACAACGAGACTGATCCTGAATGAAGCAGAGCTTATCATGACGCT
GGCCCAGGAGTTTCAGATGAGGGTGGTCACAGTAAACCTGGAGGAACAGT
CATTCCCCAGTATTATTCAGGTGATTAGCACTGCCACCATCTTAGTCAGT
ATGCATGGAGCTCAACTCATAACCTCACTCTTCCTCCCAAGAGGAGCTGT
TGTGGTGGAGCTGTTCCCCTTTGCTGTGAACCCAGAGCAGTACACTCCAT
ATAAAACCCTTGCCTCCCTTCCAGGCATGGACCTTCATTACATCTCCTGG
AGAAACACTATGGAGGAGAACACAGTCACTCACCCAGGCAGGCCCTGGGA
ACAAGGGGGTATCGCTCATTTGGATAAAGAGGAGCAGGAACGAATACTGG
TGAGTAAGGATGTGCCGAGGCATCTCTGCTGCCGAAACCCCGAGTGGCTC
TTCCGGATCTACCAGGATACTTTTGTGGACATCACCTCTTTCCTGGAAGT
CCTTAA---AGAGGGCATGAAT---GCAAATCTCAACTTGAAGAA---GT
CAAAACCGGCCAGCACACTCCACCCGGGCAGGGTCAGAGAACCCCAATGT
CAGACTTCAGTTCAAACCAGCAATGAGGCTAAACTCACAGTCTCCTGGCA
GATACCATGGAATCTGAAATACCTGAAGGTCAGAGAGGTGAAATACGAAG
TGTGGATCCGGAAGAAAGATGCCAACAAGGGGACCCTGGAGGATCAAATC
ATACAAGCGAACCCTGCGCTGGAGGCCTTCGGGAACGCCAAAACGCTCCG
AAACGACAATTCGTCCCGTTTTGGAAAGTTCATTCGAATTCACTTTGGTA
CCAGCGGAAAGCTGTCGTCTGCTGACATCGAGACGTATCTGCTGGAGAAG
TCCCGAGTCACCTTTCAGCTCAAGGCTGAGAGAAATTACCACATATTCTA
CCAGATCCTGTCGAACCAGAAGCCAGAGCTGCTGGACATGCTGCTGATCA
CCAACAATCCCTACGACTACTCCTACATCTCCCAGGGAGAGGTGACCGTC
GCCTCCATCAACGACTCAGAGGAGCTGATGGCCACAGATAGCGCCTTCGA
CGTGCTCGGCTTCACTCCGGACGAGAAGACGGGCGTCTATAAACTCACGG
GCGCCATCATGCACTACGGCAACATGAAATTCAAACAGAAGCAACGCGAG
GAGCAGGCYGAGCCGGACGGGACGGAGGCTGCCGATAAATCCGCTTACCT
AATGGGGCTGAACTCTGCTGACCTCATCAAAGGGCTGTGCCACCCCAGAG
TCAAGGTAGGGAATGAATATGTCACCAAAGGCCAAAGTGTGGATCAAGTC
TACTACCCCAACAAGGAGGCCTTCAAATGCGATGAGTGTGGGAAGCACTA
CAACACCAAGCTGGGATACAAGCGCCATGTGGCCATGCACTCTGCCACAG
CGGGGGATCTCACCTGCAAGGTGTGCATGCAGACCTACGGCAGCACACCG
GTGCTCCTGGAGCACCTGAAGAGCCACTCGGGGAAGTCCTCGGGTGGCAC
CAAGGAGAAGAAACACCAGTGCGACCACTGTGATCGGCGTTTCTACACAA
GGAAGGACGTGAGACGCCACATGGTGGTGCACACGGGCCGGAAGGACTTC

CTGTGCCAGTACTGTGCTCAGCGCTTCGGCAGGAAGGACCACCTGACGCG
CCACGTGAAGAAGAGCCACTCGCAGGAGCTGCTGAAGATCAAGACGGAGC
CTCCAGACATGTTGGGCCTCCTGGCTTCAGGGTCGCCACCGTGCTCCGTG
AAGGAGGAGCTTAGCCCCATGATGTGCGGCATGGGGCCCAACAAAGACCC
CATGATGGGCAAAGCCTTCCCCGGCGGGGCCCCTTTTCCAGTGGGCGTGT
ACAACCCTCACCAC------CTGCAGGCCATGCCTCACCCTGGGGCGGCT
CACTCG------CACCCGTCCCTGATGCCAGGCTCCCTGTCTGCGGCGAT
GGGCATGGGCTGTCCCATGGACNNNNNNNNNNNNNNNNNNNNNNNNNNNNNNNNNTGTTTACAGATCAGT
GACGGGTCAAATA
TCGTGAACCTGCTGGCGAGTAACTCTCCAAGTGTCTCATATGCTCTGACC
CAGCAAAAATACTTCAGTAACTACAGTCCGGTCATTGGGTTTTACATTTA
TGAGCCCATCGAGTACTGGAACGCTACAGTGCAGGAGCACCTGAAGACTT
TGAGTCATGGCTTCAACAAGATCTCCTGGATGGACAACTTTTTCCACTAC
CTGCGGGTGGTGAATGTGAGTGCATCAACCAAGAGCGACTTCATCACCAT
CCTCAAGGGCTCCTTCCTGCGCAGCCCGGAGTACCAGCACTTCACTGAGG
ACATCATTTTTTCAAAGA---ACCGTGAGACAG------ACGAGTACGAC
ATCATTGCCTCGCGGATGTACTTGGTGGCGCGGACTACAGAGAAGAAACG
TGAAGAAGTGGTAGAGCTTCTGGAAAAACTTCGCCCATTGATGCTGATCA
ACAGCATCAAGTTCATTGCCTTCAATCCTACGTTTGTGTTCATGGACCGC
TACAGCTCCTCGGTCATCTCGCCCATCCTGACCTCAGGATTCAGCGTACT
CACAATCCTCATCCTCACTTTGTTCCTGGTCATCAACCCCTTGGNNNNNNNNNNNNNNNNNNNNNNNNNN
NNNNNNNNNNNNNNNNNNNNNNNNNNNNNNNNN------

-TCTTACACCATCGAGATGGGTCCCGTGGGGCCCCGATGGAARGAGAGCC
CGCAGCCGTTCTCCTGCTCCGTTGAAGACCCCACGAAACAGACAAAGTTC
AAGGGCATCAAGACGTACATTTCATACAGGGTGACGCCGAGCCACACCGG

GCGACCCGTCTACAGACGCTACAAACACTTTGACTGGCTGTACAACCGCT
TACTGCACAAGTTCACTGTGATCTCTGTGCCTCATCTGCCCGAGAAGCAG
GCCACGGGGCGCTTTGAGGATGACTTCATCGAGAAGCGCAAGAGGCGACT
GATACTGTGGATGAACCACATGACCAGTCACCCAGTCCTCTCCCAGTATG
AGGGCTTCGAGCACTTCCTGATGTGYGCTGACGACAAGCAGTGGAAACTG
GGAAAGAGACGGGCAGAGAAAGACGAGATGGTGGGAGCCCATTTCATGCT
GACCCTCCAGATCCCTAACGAGCACCAGGACCTTCAGGATGTGGAGGAGC
GGATCGACTCCTTCAAGTCCTTCGCTAAGAAGATGGACGACAGCGTGATG
CAGCTCACGCATGTTGCCTCGGAGCTGGTGCGTAAGCACCTGGGCGGGTT
CAGGAAGGAGTTCCAGAGGCTGGGAAATGCCTTCCAGTCTATCAGCCAGG
CGTTCATGCTGGACCCTCCCCAGAGCTCGGAGACCTTAAACAACGCCATC
TCCCAC--

>Omosudis lowei
AGTCTCCTCATCCGAGCAGAACTTAGCCAACCTGGGGCCCTCCTAGGAGA
CGACCAAATCTACAATGTAATTGTTACTGCTCACGCCTTCGTAATAATTT
TCTTTATAGTAATGCCCATTATGATCGGCGGGTTCGGAAACTGACTTATC
CCCTTGATGATCGGAGCCCCTGACATAGCCTTCCCCCGAATAAACAACAT
GAGCTTCTGGTTACTCCCTCCCTCTTTCCTACTTCTCCTCTCTTCCTCCG
CCGTAGAGGCAGGGGCCGGAACGGGATGAACCGTCTACCCTCCCCTCGCC
AGCAACTTGGCACACGCCGGAGCTTCCGTAGACCTGACCATTTTTTCTCT
TCACCTAGCAGGAATTTCCTCTATTCTAGGTGCCATCAACTTTATTACAA
CGATTATTAACATAAAACCCCCCGCCATTACCCAGTATCAAACCCCCTTA
TTTGTTTGAGCAGTCCTAATTACCGCTGTCCTCCTCCTCCTCTCCCTTCC
TGTTTTAGCGGCAGGCATTACAATGCTGCTCACAGACCGAAACTTAAACA
CAACTTTCTTTGACCCCGCAGGTGGAGGTGATCCCATTCTCTACCAACAC
CTGTTCTGATTCTTTGGCCACCCAGAAGTCTATATTTTAATTCTCCCCGG
CTTTGGAATAATTTCCCACATTGTTGCCTACTATTCAGGTAAAAAAGAAC
CCTTCGGCTACATGGGCATGGTGTGAGCAATGATAGCAATCGGGCTCCTC
GGCTTTATTGTCTGAGCCCATCTCATGTTTACAGTCGGAATGGACG-GGA
CACGCGTGCCTATTCCTTGAGAGGAACCTCCACCCATCCAACTGCCTTGG
CATGCTGCTACTGTCAGATGCCCACCAGTGCACCAAGCTGTCAGAGCTGT
CCTGGGGTATGTGCCTCAGCAACTTCCCTGCCATCTGCAAGACGGAGGAC
TTCCTCCAGCTGCCCAAAGACATGGTGGTGCAGCTCCTGTCCCATGAAGA
GCTGGAGACGGAAGATGAGAGACTGGTTTATGAGGCTGCCCTTAACTGGG
TCAACTATGACCTGGAGAGGAGACACTGCCAGCTGCCAGAGCTGCTGAAA
ACCGTACGTCTGGCCTTGCTTCCCGCCATCTTCCTCATGGAGAATGTCTC
CACGGAGGAGCTGATCAATGCCCAGTTGAAGAGCAAGGAGTTGGTGGACG
AGGCCATCCGCTGCAAGCTAAAGATCCTGCAGAATGACGGTGTGGTCAAC
AGTCCCTGTGCCCGGCCCAGAAAGACCAGTCATGCCCTTTTCCTGCTGGG
AGGGCAGACCTTCATGTGTGACAAGCTGTACTTGGTGGACCAGAAGGCCA
AAGAGATCATCCCCAAGGCGGACATCCCCAGCCCCAGGAAGGAGTTCAGC
GCCTGTGCCATTGGCTGTAAGGTGTACGTCACAGGTGGGA--GAGGCTC-
AGAGAACGGCGTATCTAAAGACGTGTGGGTCTATGACACTGTCCATGAGG
AGTGGTCCAAGGCAGCACCCATGCTCATTGCCAGATTTGGCCATGGCTCC
GCCGAGCTCAAACACTGCCTCTATGTGGTTGGAGGACACACGGCAGCCAC
TGGCTGCCTCCCAGCCTCTCCGTCTGGATGAGTACATTGTAGTGTTCAGT
CGTTCCACAACCAGGCTGATTCTGAACGAAGCAGAGCTGATACTGGCACT
GGCCCAGGAGTTTCAGATGAGGGTGGTCACCGTGTCCCTGGAGGAACAGT
CCTTCCCCAGCATCGTACAGGTCATCAGCGGTGCCTCCATGCTGGTCAGT
ATGCACGGTGCTCAGCTCGTCGCCTCGCTCTTCCTCCCCAGGGGAGCCGC
CGTGGTAGAGCTCTTCCCCTATGCCGTGAACCCAGAACAGTACACCCCRT
ACAAAACCCTAGCCTCCCTGCCGGGCATGGACTTTCAGTACGTTCCCTGG
AGGAACACGATCGAGGAGAACACCGTCTCCCACCCGGACAGACCCTGGGA
CCAAGGAGGCATCGCCCATTTGGAGAAAGAAGAGCAGGAGAGAATCCTAG

CCAGCAAGGATGTCCCCAGGCACCTGTGCTGYCGCAAYCCGGAGTGGCTC
TTCAGGATCTACCAGGACACTCTGGTAGACATCCCCTCCTTCCTGGACGT
CCTCAG---GGAGGGCCTGAAG---ACGAGGCCGAGCTTGAAGAA---GT
CCAAGCCGGCCAGCACGGTTCACCCCGGCCGGGTTCGAGAACCCCAGTGC
CAGACTTCGGTCCAAGCCGCCAACGAGGCTAAACTTACGGTGTCCTGGCA
GATCCCGTGGAACCTTAAGTACCTGAAGGTGCGAGAGGTGAAGTACGA-G
TATGGATCCGGAAGAAGGACACAAGCAAGGGAACCCTGGAGGATCAAATC
ATTCAGGCAAACCCCGCGCTGGAGGCTTTCGGTAATGCCAAAACACTGAG
GAACGATAATTCCTCCCGTTTTGGAAAATTCATCCGAATCCACTTTGGAA
CCAGTGGTAAGCTGTCTTCTGCGGACATCGAGACCTACCTGTTGGAGAAG
TCACGGGTCACCTTTCAGCTGAAGGCAGAGAGGAACTACCACGTCTTCTT
CCAGATCTTGTCCAATCAAAAGCCAGAGCTGCTGGACATGCTGTTGATCA
CCAACAACCCCTATGACTACTGCTACATCTCCCAAGGAGAAGTCACCGTA
GCGTCCATCAATGATTCAGAAGAGCTGATGGCCACCGACAGTGCCTTCGA
TGTGCTTGGCTTCACGCAAGAGGAGAAGATGGGAGTCTACAAGTTGATAG
GGGCCATTATGCACTTTGGCAACATGAGGTTCAAGCAAAAGCAGCGCGAG
GAACAGGCMGAGTCTGATGGCACAGAGGCTGCTGATAAGGCAGCTTATCT
AATGGGGCTGAATACAGCAGACCTAATCAAGGGGCTCTGCCATCCCAGAG
TCAAGGTAGGGAATGAGTACGTCACCAAAGGCCAGGGTGTAGATCAAGTC
TACTACCCCAACAAGGAGGCCTTAAAGTGTGAAGAGTGTGGCAAGCACTA
CAACACCAAGGTGGGATACGAGGTGGTGGTAGAGGAACAGTGGTTCCATA
GGCGGGACCTCACCTGCGAAGTGTAAATGCAGAGTTACGAGAGCACCCCG
GTGCTCCTGGAGCACCTCAAGAGCCACTCGGGGAAGTCCTCGGGCGGCGC
CAAGGAGAAGAAGCACCCGTGCGACCACTGCGACCGTCGCTTCTACACGC
GGAAGGATGTCAGGCGGCACATGGTCGTCCACACGGGCCGGAAGGACTTC
CTGTGCCAGTACTGTGCCCAGCGCTTTGGCAGGAAGGACCATCTGACGCG
GCACGTGAAAAAGAGCCACTCGCAGGAGCTGCTGAAGATCAAGACGGAGC
CTCCGGACATGCTGGGCCTGCTGGGCTCCGGCTCGCCTCCTTGCTCTGTC
AAGGAGGAGCTGAGTCCCATGATGTGCAGCATGGGTCCCAATAAAGACCC
CATGATGGGCAAACCCTTCCCCAGCGGGACCCCCTTCCCCATGGGCATGT
ACAACCCCCACCAC------CTCCAGGCCATGTCTAATCCCGGGGTGGGC
CACCCC------CACCCCTCCCTTATGCCCAGTTCCCTGTCTGCAGCTAT
GGGCATGGGCTGTCATATGGAA----------------------------

-------------------------------------GGCTACCACACATT
CGAGTGGCAGCCAGCCCTAAAAAATGTGTCCCCATCCTGCCACGTGGGTA
TCATCAATGGGCTTTCCGGGTGGGCAGCCTCGGTGGATGATGTCCTTGCT
GACACCATCACCCGTCGGTTTCGCTATGACGTGGCGCTTGTATCGGCCCT
GAAGGACTTAGAGGAGGACATCATGGAAGGACTGAGGGTGCATGGGCTGG
AAGACAGCGCTTGCACTTCAGGCTTCAGTGTTATGATCAAGGAATCCTGT
GATGGCATGGGAGATGTCAGCGAGAAGCACGGCGGGGGGCCAGCTGTGCC

TGAGAAGGCTGTGCGTTTCTCTTTCACCGTCATGTCTGTCTCTGTCCAGG
CCGACGGAGAGGAG------------------------------GAGGCG
GTTGTTGTCTTCAGGGAGCCAAAACCCAACTCTGAACTGTCCTGTAAGCC
CATGTGCCTGATGTTTGTGGACGAGTCTGACCACGAGACACTCACTGCTG
TCCTTGGGCCTTTGATAGCAGAGAGGAATGCAATGAAGCAAAGCCGACTC
ATCCTCCCAATGGGAGACCTCCCCCGCTCCTTCCGCTTCCACTTCAGAGG
CACGGGCTACGATGAGAAGATGGTGCGAGAGATTGAAGGCCTGGAGGCCT
CAGGCTCCACCTATATCTGCACTCTTTGCGACTCCACTCGGGCAGAAGCC
TCTCACAACATGGTGCTCCACTCTGTCACTCGCAGCCACGACGAGAACCT
GGAGCGCTACGAGATATGGAGGACCAACCCCTTCTCGGAGTCTGCCATGG
AGCTGCGAGATCGGGTCAAAGGCGTCTCTGCCAAACCCTTTATCGAGACC
CATCCGACTATGGATGCATTACATTGTGACATAGGTAATGCCACCGAATT
CTACAAAATCTTCCAGGATGAGATCGGGGAGGTGTTCCAGAAGGT---CA
AC---CCTAGCCGGGAGGAGCGGCGCAGCTGGAGGGCAGCCCTTGATAAA
CAGCTGAGAAAGAAGTTGAAGCTGAAACCAGTGATGCGGATGAACGGGAA
CTTCGCCCGGAGGCTGATGACCGAGGAGGCTGCGGAGGTGGTGTGTGAAC
TGGTGCCCACGGAAGAGAGACGGGAGGCCCTGAGGGAGCTGATGGGGCTC
TATGTCCAGATGAAGCCGGTGTGGCGCGCTACCTGCCCGGCCAAGGAGTG
CCCCGACCAGCTGTGCCGCTACAGCTTTAACTCTCAGCGCTTTGCCGATC
TCCTCTCGTCCACCTTCAAGTACAGGTACGACGGGAAGATCACCAACTAC
CTGCACAAGACTCTGGCCCATGTTCCCGAGATCATAGAGAGAGATGGCTC
CATCGGAGCCTGGGCCAGCGAGGGGAACGAGTCTGCGAACAAA-------

--------------ACAGGCGAAGTCACAGAAAGAGAAGTGGCTTTGGGG
ATAAATCCGTTCGCGGACGGCATGGGCGCCTTCAAAATAAACCACAGCTC
CCACGATATTGGCTCCGG---GCAAACGGCGTTTTCCTCCCAGGCG---C
CCGGCTAC---GCGGCGGCCGCCCTGGGA---CACCATCA------CCAC
CCGACCCACGTCAGCTCC---TATTCCACGGCAGCCTTCAATTCCACGCG
GGACTTTCTCTTCAGAAATCGGGGCTTCGGAGACGCCACCAG--------
----CGCGCAGCACAGTCTGTTCGCCTCCGC---CGCGGGAAGTTT---T
------GCAGGGCCACACGGACACTCAGATGCCGCGGGCCACCTGCTCTT
CCCCGGGCTTCACGAG---CAAGCCGCGAGCCACGCGTCGTCTAACGTCG
TGAACAGCCAGATGCGACTGGGCTTTTCCGGGGACATGTACGGCCGGGCC
GACCAGTATGGCCACGTTACAAGCCCGAGGT---CCGACCACTACGCTTC
GACCCAGCTGCACGGCTATGGCCCTATGAACATGAATATGGCCGCT---C
ACCACGGAGCGGGGGCCTTCTTTCGGTACATGCGGCAGCCGATCAAACAA
GAGCTGATCTGCAAGTGGATCGAACCGGAGCAACTGTCGAATCCCAAGAA
GTCGTGCAACAAAACTTTCAGCACGATGCATGAGCTCGTGACCCACCTGA
CGGTGGAGCATGTGGGAGGACCCGAGCAGTCGAACCACATTTGCTTCTGG
GAAGATTGTGCCAGGGAAGGGAAACCGTTCAAAGCCAAATACAAACTCGT
AAATCATATCAGAGTTCACACCGGAGAAAAACCGTTCCCGTGTCCGTTCC
CCGGCT--------
>Onuxodon parvibrachium
AGTCTACTTATCCGGGCAGAACTAAGCCAGCCTGGTGCTCTCCTCGGTGA
TGACCAAATTTATAATGTAATCGTCACAGCACACGCATTCGTAATAATCT
TCTTTATAGTAATACCGATCATGATCGGGGGCTTCGGGAATTGACTAATC
CCGCTAATAATTGGTGCCCCAGACATGGCCTTTCCTCGAATGAATAACAT
AAGCTTCTGATTACTTCCACCCTCTTTTCTTCTTCTACTGGCGTCATCAG
GCGTAGAAGCCGGGGCCGGAACCGGATGAACTGTCTACCCGCCCTTATCT
GGCAACTTAGCCCACGCAGGGGCTTCTGTAGACCTAACAATTTTTTCTCT
GCACTTAGCAGGCATTTCCTCCATCCTCGGGGCAATTAACTTTATCACCA
CCATCATCAACATGAAACCCCCTGCTATTTCTCAGTACCAGACACCCCTG
TTCGTATGGGCAGTACTAATTACTGCAGTCCTTCTTCTCCTTTCTCTCCC
AGTCCTAGCGGCTGGTATCACCATGCTCCTAACTGATCGAAACCTCAACA
CCACCTTTTTTGACCCAGCTGGGGGTGGGGATCCAATTCTATACCAACAC
CTA---

------------TTTCTAGAGAGAAACCTGCACCCGTCTAACTGCCTTGG
CATGCTTTTGCTGTCCGATGCCCATCAGTGTACCAAGCTGTCAGAGCTGT
CCTGGGGCATGTGTCTCAGCAACTTCCCTGCTATTTGCAAGACAGAGGAC
TTCCTGCAATTGCCCAAAGACATGGTGGTGCAGCTTCTGTCACACGAGGA
GCTGGAGACAGAAGATGAGAGACTGGTTTATGAAGCTGCCCTTAACTGGA
TCAACTATGACCTGGAAAGGAGGCACTGCCACCTCCCAGAGCTCCTGAGA
ACAGTTCGTCTGGCTCTGCTGCCTGCCATCTTTCTCATGGAGAATGTCTC
AACAGAAGAGCTGATCAATGCCCAGGCCAAGAGCAAGGAGCTTGTGGATG
AGGCCATACGCTGTAAACTGAAGATCCTGCAGAATGATGGCGTAATCAAC
AGCCCATGTGCCCGTCCAAGAAAAACCAGCCATGCACTCTTTCTGTTGGG
AGGGCAGACATTCATGTGTGACAAGCTGTACCTGGTGGACCAGAAAGCCA
AAGAGATCATCCCCAAAGCTGACATCCCCAGCCCCAGGAAGGAGTTTAGT
GCCTGTGCCATTGGCTGCAAGGTTTACATCACAGGTGGGA--GAGGTTC-
AGAGAACGGTGTGTCCAAAGATGTATGGGTCTACGATACCGTCCATGAGG
AATGGTCCAAGGCAGCACCCATGCTTATTGCCAGGTTTGGTCACGGCTCT
GCTGAGCTGAAACACTGCCTGTATGTGGTGGGAGGCCACACGGCAGCGAC
TGGCTGCCTCCCGGCCTCTCNNNNNGGACAAATACATTGTTGTCTTCAGTCGTTC
AGCAACAAGGCTAATCTTGAATGAAGCAGAGCTAATCATCGCACTGGCCC
AGGAATTTCGAATGAAAGTTGTTACAATTTCACTGGAGGAACAAACTTTT
CCCAATATTGTCCAGGTGATTAGTGGTGCTTCCATGTTGGTTAGCATGCA
TGGAGCCCAGCTCATAACCTCACTCTTCCTACCTAGAGGAGCTGCTGTAG
TTGAACTCTTCCCCTATGCTGTGAATCCAGAGCAGTACACACCATATAAA
ACCCTGGCTTCTCTTCCAGGCATGGACCTACACTATGTCTCTTGGAGAAA
CATCATGGAGGAAAACACAGTAACCCACCCAGACAGATCCCCGGAACAAG
GGGGCATAGTTCACTTTGAGAAGGATGAGCAGGAGCGCATACTGGCTAGC
AAAGATGTTCCCAGGCACCTGTGCTGCCGCAATCCAGAGTGGCTCTTTCG
GATCTACCAGGACACTTTGGTTGATATCCCTTCATTCCTGGAAGTCATCA
A---AGAAGGCTTGAAG---ACCAAGCCTAGTTGGAAGAA---AGCCAAG
GTGGCCAGCGCAGTTCACCCTGGTCGGGTCAGAGATGCCCAGTGTCAGAC
CTCAGTGCAAACCAGCAGTGAGGCTAAACTCACAGTTTCTTGGCAGATCC
CATGGAATCTAAAGTACCTTAAGGTGAGAGAAGTGAAGTACGAGGTG---
------AAGAAAGACACCAGCAAGGGGACACTGGAAGATCAAATCATCCA
GGCGAACCCAGCCCTGGAGGCTTTTGGGAATGCCAAAACAGCAAGAAATG
ACAACTCATCCCGTTTCGGAAAATTCATTCGAATTCACTTTGGAAACAGT
GGCAAGTTGTCATCTGCTGACATTGAGACATATCTGCTGGAAAAATCACG
TGTCACCTTTCAACTCAAGGCTGAGAGGAACTATCACATCTTCTACCAGA
TATTATCAAATCATAAACCAGACCTGCTGGACATGCTGCTGATCACAAAT
AACCCATATGACTACTCCTACATTTCTCAAGGGGAGGTAACTGTCGCATC
AATCAATGATGCAGAAGAACTGATAGCCACAGACAGTGCTTTTGATGTTC
TCGGCTTCACTCCAGAGGAAAAGATGGGTGTTTATAAGCTGACTGGTGCC
ATCATGCACTATGGCAACCTCAAGTTCAAGCAGAAGCAACGTGAGGAGCA
GGCCGAACCCGACAGCACGGAGGCTGCTGATAAATCGGCTTACCTAATGG
GTCTGAACTCTGCAGACCTCATTAAAGGGCTATGCCATCCCAGAGTTAAA
GTGGGGAATGAGTATGTTACCAAAGGCCAAAGTGTGGACCAAGTCTACTA
TCCCAACAAGGAGGCCTTCAAGTGCGAGGAGTGTGGCAAGCACTACAACA
CCAAGCTGGGATACAAGCGACATGTGGCCATGCACTCTGCCACAGCAGGG
GATCTCACGTGCAAGGTGTGCATGCAGAGCTACGAGAGCACGCCCGTGCT
CCTGGAACACCTCAAGAGCCACTCTGGGAAGTCCTCGGGTGGCACCAAGG
AGAAAAAGCACCCGTGCGACCACTGCGACCGCCGCTTCTACACGCGGAAG
GATGTGAGGCGGCACATGGTGGTCCACACGGGCCGCAAGGACTTCCTGTG
CCAGTACTGTGCCCAGCGCTTTGGCAGGAAGGACCACCTGACGCGCCACG
TGAAGAAGAGCCACTCCCAGGAGCTGCTGAAGATCAAGACGGAGCCGCCG
GATATGTTGGGTCTTTTAGCTTCGGGGTCGCCCCCGTGCTCTGTGAAGGA

GGAGCTTAGCCCCATGATGTGCGGCATGGGTCCAAATAAAGACCCCATGA
TGGGAAAACCCTTCCCGAGTGGGGCCCCTTTTCCGATGGGCATGTACAAC
CCCCACCAT------CTCCAGGCCATGTCCAATTCTGGGGTGGGCCACCC
C------CACGCCTCCTTGATGCCCAGTCCCCTGTCTGCAGCTATGGGCA
TGGGCTGCCACATGGAGTATCTCATCTACGCCTCCTTCTCCTTCATGGGA
TGTTTACAAATCAGCGACGGCTCCAACATCGTCAACCTGCTGGCCAGCAA
CTCCCCCAGCGTGTCCTACGCGCTGACTCAGCAGAAGTACTTCAGCAACT
ACAGCCCCGTGATCGGTTTTTACATCTACGAGCCCATCGAGTACTGGAAC
TCCACCGTGCAGGAGCACCTGAAGACTCTGAGTCACGGGTTCAACAAGAT
CTCCTGGATGGACAACTTCTTCCACTACCTGCGGGTGGTGAACGTGAGCG
CCTCCACCAAGAGCGACTTCATCTCCATCCTCAAGGGTTCCTTCCTGCGC
AGCCCGGAGTACCAGCACTTCACCGAGGACATCATCTTCTCCAAGA---A
CCCCGAGACGG------ACGAGTACGACATCATCGCCTCGCGGATGTACC
TGGTGGCCCGGACCACGGAGAAGAAGCGCGAGGAGGTGGTGGAGCTGCTG
GAGAAGCTGCGGCCGCTGATGCTGATCAACAGCATCAAGTTCATCGCCTT
CAACCCCACCTTCGTCTTCATGGACCGCTACAGCTCGTCGGTGATCTCGC
CCATCCTCACGTCGGGCTTCAGCGTGCTCACCATCCTCGTCCTGACTTTC
TTCCTGGTCATCAACCCCCTGGGGAACTTCTGGCTCATCCTGACGGTCAC
CTCCGTGGAGCTGG------------------------------------

--------------------------------------TCCTACACTATT
GAGATGGGCACCGTGGGACCCCTGTGGAAAGAGAGCCCACAGCCGTTCTC
TTGCTCCATTGAAGACCCCACAAAACAGACAAAGTTCAAGGCATCAAGA
CCTACATTTCCTACAGGGTCACTCCGAGCCACACGGGGCACCCCGTCTAC
AGACGCTACAAACACTTTGACTGGCTGTACAACCGCCTACTGCACAAGTT
CACTGTGATCTCAGTTCCACACTTGCCCGAGAAGCAGGCTACCGGGCGAT
TCGAGGAAGATTTCATCGAGAAGCGTAAAAGGCGGCTGATACTGTGGATG

AACCACATGACGAGCCACCCGGTCCTCTCCCAGTATGAAGGCTTCGAGCA
CTTTCTCATGTGTGCAGACGACAAACAGTGGAAACTGGGGAAGAGGCGGG
CGGAGAAGGACGAAATGGTGGGGGCCCATTTCATGTTGACCCTCCAGATT
CCCAATGAACACCAGGACCTTCAGGACGTAGAGGAGAGAGTTGACACCTT
TAAGGCCTTTGCCAAGAAAATGGACGATAGTGTCTTGCAGCTGACACACG
TTGCCTCAGAGCTGGTGCGGAAGCACATCGGTGGGTTCAGGAAGGAGTTC
CAGCGGCTTGGAAATGCATTCCAGTCCATCAGCCAGGCCTTCATGCTGGA
CCCTCCCCACAGCTCCGNNNNNNNNNNNNNNNNNNNNNNNNNN---------------------------
------

------------------------NNNNNNNNNACAGGGGAAGTCACAGAGAGAGAAGT
GGCGTTGGGGATAAATCCTTTCGCGGACGGGATGGGCGCCTTCAAAATAA
ACCACAGCTCCCACGACATCGGCTCCGG---GCAGACGGCGTTCTCCTCC
CAGGCG---CCCGGCTAC---GCAGCGGCCGCCCTGGGA---CACCACCA
------CCACCCGACCCACGTTGGCTCT---TACTCCACGGCGGCTTTCA
ACTCCACCAGGGACTTTCTGTTCAGAAATCGGGGTTTCGGGGACGCTGCC
GG------------CGCTCAGCACAGCTTGTTCGCCTC---------CGG
AAGTTT---C------GCAGGGCCACATGGACACTCGGATGCGGCGGGGC
ACCTGCTCTTCCCGGGGCTCCACGAG---CAGGCAGCCAGCCATGCGTCT
TCCAACGTGGTCAACGGCCAGATGCGTCTGGGCTTCTCGGGGGACATGTA
CGGCCGGGCCGACCAGTACGGCCACGTTACGAGCCCCAGGT---CCGACC

ACTACGCGTCGACCCAGTTGCACGGCTACGGCCCCATGAACATGAATATG
GCCGCG---CACCACGGAGCGGGGGCCTTCTTTCGATACATGCGGCAGCC
GATCAAGCAAGAGCTCATCTGCAAGTGGATCGAGCCGGAGCAGCTGACGA
ACCCCAAAAAGTCGTGCAACAAAACTTTCAGCACGATGCATGAGCTTGTG
ACCCATCTGACGGTGGAGCATGTGGGGGGACCGGAGCAGACCAACCACAT
CTGCTTCTGGGAGGACTGCCCCNNNNNNNNNNNNNNNNNNNNNNNNNNNNNNNNNNNNNNNNNNNNNNNN
NNNNNNNNNNNNNNNNNNNNNNNNNNNNNNNNNNNNNNNNNNNNNNNNNNNN
>Ophiblennius atlanticus
AGCTTATTAATTCGAGCAGAATTAAGCCAGCCAGGAGCTCTCTTAGGAGA
TGACCAAATTTACAATGTAATCGTTACAGCCCATGCCTTCGTTATAATTT
TCTTTATAGTAATACCAATTATGATTGGAGGCTTTGGAAACTGACTTATT
CCTCTTATGATCGGGGCCCCTGATATAGCTTTCCCCCGAATAAACAATAT
GAGCTTTTGACTTCTTCCTCCTTCCTTCCTTCTTCTTCTTGCCTCTTCAG
GTGTTGAAGCGGGAGCCGGGACAGGGTGGACTGTTTACCCCCCACTTTCG
GGAAACTTAGCTCATGCAGGTGCATCTGTTGACCTTACCATCTTCTCCCT
TCACCTGGCAGGGATCTCGTCAATTTTAGGTGCAATTAATTTTATTACAA
CTATTATTAACATGAAACCTCCAGCCATTTCTCAGTACCAAACACCCCTG
TTTGTCTGAGCCGTTCTAATTACAGCTGTCCTTCTTCTTCTATCCCTTCC
AGTGTTAGCTGCGGGCATCACTATGCTCTTAACTGACCGAAACCTAAACA
CAACCTTTTTTGATCCGGCTGGCGGGGGAGACCCAATTCTCTACCAACAT
CTT---

------------TTCCTGGAGAGGAACCTTCACCCATCCAACTGTCTCGG
TATGCTGCTTCTGTCCGATGCCCATCAGTGTACCAAGCTGTCGGAGCTCT
CCTGGGGCATGTGCCTCAGCAACTTCCCCGCGATTTGCAAAACAGAGGAC
TTCCTCCAACTGCCCAAAGATATGGTGGTGCAGCTTTTATCACATGAGGA
ACTAGAGACAGAAGATGAAAGACTGGTTTATGAAGCTGCCCTCAACTGGA
TCAACTATGACCTGGAAAGGAGGCACTGTCATCTTCCAGAGCTCTTGAGA
ACAGTCCGCCTCGCATTGCTGCCTGCCATCTTTTTAATGGAAAATGTCTC
AACAGAAGAACTCATCAATGCCCAAGCCAAGAGCAAAGAGTTGGTGGACG
AAGCCATCCGCTGTAAGCTGAAGATCCTGCAGAATGACGGGGTCGTAAAC
AGTCCTTGTGCTCGACCAAGAAAAACCAGCCATGCCCTCTTTCTCCTGGG
TGGGCAGACTTTCATGTGTGACAAGTTGTACCTGGTTGACCAGAAGGCCA
AAGAGATCATCCCGAAAGCTGATATCCCCAGCCCAAGGAAGGAGTTCAGC
GCTTGCGCCATTGGCTGCAAGGTGTACATCACAGGCGGGC--GAGGCTC-
TGAGAATGGAGTGTCCAAAGATGTATGGGTCTATGACACCGTCCACGAGG
AATGGTCAAAGGCTGCGCCGATGCTCATCGCCAGGTTCGGCCATGGATCT
GCAGAGCTGAAGCACTGCCTCTATGTTGTAGGAGGTCACACAGCCGCAAC
CGGTTGCCTCCCAGCTNNNNNNNNNNAGATGAATATATTGTTGTGTTTAGTCGTTCGACA
ACCAGGCTGATTTTAAATGAAGCTGAATTGATAATGGCGTTGGTTCAGGA
GTTTCAGATGAAAGTCGTCACTGTGTCTTTAGAGGAACAGTCTTTTTCCA
GCATCGTCCAGGTGATCAGTCGTGCTTCCATGCTGGTCAGCATGCACGGA
GCTCAGCTGATCACCTCACTGTTCCTGCCCAGAGGAGCTGCTGTGGTGGA
GCTGTTTCCCTTTGCAGTGAACCCAGAACAGTACACCCCATATAAAACTC
TGGCCTCCCTCCCTGGCATGGACATTCACTANGTTTCCTGGAGGAACACGA
TGGAGGAGAACACTGTCACCCATCCAGACAGACCCTGGGAACAAGGAGGC
ATTGCACATTTAGAGAAGGAGGAGCANGAGCGTATCCTGGCAAGCAAGGAT
GTCCCCAGACACCTGTGCTGCCGCAACCCGGAGTGGCTCTTCAGGATCTA
CCAGGACACCAGGGTGGACATCAGTTCCTTCTTGGAGGTCCTGCG---GG
AAGGCATGAAG---TCCAAGCCCAACCTGAAGAA---GTCCAAGGCAGTC
AGCATCGTTCACCCGGGCCGGGTCCGAGAGCCCCAGTGTCAGACATCGGT

ACAGACAAGCAGCGAGGCCAAACTCACGGTTTCCTGGCAGATCCCGTGGA
ATCTGAAGTACCTCAAAGTCCGAGAGGTAAAGNNNNNNNNNNNNNNNNNNAAGAAAGACACCAGCAAA
GGGACCCTGGAGGACCAGATTATCCAGGCCAACCCAGCGCTGGAAGCCTT
CGGTAATGCCAAAACATTACGAAATGACAACTCGTCCCGGTTTGGAAAAT
TCATCCGGATTCACTTTGGCNNGAGTGGGAAGCTCTCGTCTGCTGACATCGA
GACATACCTGTTGGAGAAGTCGCGAGTCACTTTTCAGCTGAAGGCTGAGA
GAAACTACCATATCTTCTACCAGATCCTTTCCAATCAGAAGCCAGAGCTG
CTGGACATGCTGCTGATCACCAACAACCCGTATGACTACTCCTACATCTC
TCAGGGGGAGGTGACGGTTGCCTCCATTAACGACGCAGAGGAGTTGATGG
CAACCGATAGCGCCTTTGACGTGCTGGGCTTTACGCCAGACGAGAAGATG
GGCGTCTACAAGCTGACCGGCGCCATCATGCACTACGGCAACATGAAGTT
CAAACAGAAACAACGCGAGGAACAAGCTGAACCTGACGGCACCGAAGCAG
CCGACAAATCTGCCTACCTGATGGGGTTGAACTCAGCCGACCTCATCAAG
GGACTGTGCCATCCCCGGGTCAAAGTGGGCAACGAGTACGTGACAAAAGG
CCAAGGCGTGGACCAGGTGTACTACCCAAACAAGGAAGCCTTCAAGTGTG
AGGAGTGTGGGAAGCACTACAACACCAAGCTGGGATACAAGCGCCATGTG
GCCATGCACTCTGCCACCGCAGGGGATCTAACCTGCAAAGTGTGCATGCA
GAGCTACGAGAGCACGCCTGTTCTCCTGGAGCATCTCAAGAGCCACTCGG
GGAAGTCTTCAGGCGGCGCCAAGGAGAAGAAACACCCATGCGACCACTGT
GATCGTCGTTTTTACACACGGAAAGACGTGCGACGGCACATGGTGGTCCA
CACTGGTCGAAAGGACTTCCTGTGTCAGTACTGCGCGCAGCGCTTTGGCA
GGAAAGACCATCTGACACGCCATGTGAAGAAGAGCCACTCGCAGGAGCTG
CTGAAGATCAAGACTGAGCCTCCGGACATGCTCGGTCTTTTAGCTACGGG
GTCCCCGCCATGCTCCGTGAAAGAGGAGCTCAGCCCCATGATGTGTGGCA
TGGGCCCCAATAAAGATCCTATGATGGGCAAACCATTTCCCAGTGGAGCG
CCTTTCCCGATGGGCATGTACAACCCCCATCAT------CTCCAGGCCAT
GTCGAATTCCGGGGTGGGTCACCCA------CACCCTCCCCTGATGCCTG
GCCCTTTGTCTGCAGCTATGGGCATGGGATGCCACATGGAGNATCTGATTT
ACGCGTCTTTCTCGTTCATGGGATGTTTACAAATTAGCGATGGGTCGAAT
ATCGTGAACTTGCTGGCGAGTAACTCTCCCAGCGTTTCCTACGCGTTGAC
CCAGCAGAAATACTTCAGTAACTACAGTCCCGTGATTGGGTTTTATATTT
ACGAGCCCATCGAATACTGGAACTCCACGGTGCAGGAGCACCTGAAGACT
CTGAGTCACGGCTTCAACAAAATCTCCTGGATGGACAATTTTTTCCACTA
CCTGCGGGTGGTGAACGTGAGCGCCTCGACGAAGAGCGACTTCATCAGCA
TCCTGAAGGGTTCTTTCCTTCGGAGCCCGGAGTACCAGCATTTCACCGAG
GACATCATATTCTCTAAGA---ACCGCGAGACTG------ATGAATACGA
GATCATCGCATCCAGGATGTACTTGGTAGCACGGACCACAGAGAAGAAGC
GNGAGGAGGTGGTGGAGCTTTTAGAAAAGCTCCGCCCCCTGATGCTGATCA
ACAGCATCAAATTCATTGCCTTCAACCCCACCTTTGTGTTTATGGACCGA
TACAGCTCCTCCGTCATCTCACCCATCCTGACCTCAGGCTTCAGCGTACT
CACCATCCTCATCCTCACCTTCTTCCTCGTCATCAACCCCTTGGGAAACT
TCTGGCTCATCCTCACGGTTACGTCCGTGGAGCTGGGCGTCTTGGGTTTA
NNNGGTTTTCACCAGTTTGAATGGCAGCCAGCTCTCAAGAATGTGTCGACCAC
TTGCAGTGTAGGCATTATTAATGGGCTCGCCGGTTGCGCTTTCTCGGTGG
ATGATGCCCCAGCTGACACCATCACTCGGCGCTTTCGCTATGATGTGGCG
CTGGTGTCAGCGTTAAAGGATCTGGAGGAGGACATCATGGAGGGATTGAG
AGAGAATGGGATGGAAGACAGTGCTTGCACCTCAGGCTTTCGTGTTATGA
TCAAGGAATGCTGCGATGGCATGGGCGATGTCAGCGAGAAGCACGGCGGA
GGACCAGCTGTTCCTGAGAAGGCTGTACGTTTCTCTTTCACCGTTATGTC
TGTCTCGGTCCTGCCAGACGAGGAGGAG----------------------
--------GAGGAGGTTTCCATTTTCACTGAGCCAAAGCCAAACTCAGAA
CTGTCCTGTAAGCCTCTCTGCCTGATGTTTGTGGATGAGTCAGACCATGA
GACACTCACAGCTGTCCTGGGGCCGATCATCGCAGAGCGGAACGCAATGA

AAGAGAGCAGACTCATCCTGTCCATGGGAGGCTTACTTCGCTCTTTTCGC
TTCCACTTCAGGGGCACGGGGTATGATGAGAAGATGGTGCGTGAGATGGA
GGGACTGGAAGCCTCGGGGTCCTCTTATGTCTGCACGCTGTGTGACGCCA
CTCGGACAGAGGCGTCTCACAACATGGTGCTGCACTCAATCACACGTAAT
CACGAAGAGAACTTAGAACGTTACGAAATATGGAGAACGAATCCCTTTTC
TGAGTCTGTCGATGAGCTTCGAGACAGAGTCAAAGGGGTGTCTGCCAAAC
CCTTCATGGAGACTCATCCCACTCTCGACGCGTTGCATTGCGACATCGGC
AACGCCACCGAATTCTACAAAATCTTCCAGGACGAGATTGGGGAAGTTTA
CCAAAAGGT---CAAC---CCCAGCCGGGAGGAGCGGCGCAGCTGGAGGG
CAGCCCTAGATAAACAGCTGAGGAAGAAGATGAAGCTTAAACCGGTAATG
AGGATGAATGGGAACTATGCCCGCAGGCTAATGACCCAGGAGGCTGTGGA
GGTGGTGTGTGAGCTGGTGCCCTCAGAGGAGAGGAGGGAGGCCCTGAGGG
AACTTATGAGGCTGTATATACAGATGAAGCCTGTGTGGCGTGCCACCTGC
CCAGCAAAGGAGTGCCCCGACCAGCTGTGCCGCTACAGCTTCAACTCACA
GCGCTTTGCTGACCTCCTCTCCTCTACCTTCAAATATAGGTACAGCGGAA
AGATAACCAACTATCTGCACAAGACCCTTGCTCACGTTCCTGAGATCATT
GAGAGAGATGGATCTATTGGAGCCTGGGCCAGCGAGGGTAACGAGTCAGC
TAACAAGTCATACACTATTGAGATGGGCACCCTAGGGCCCCAGTGGAAGG
ACAACCCACAGCCTTTCAACTGCTCCATTGAAGATCCCACTAAACAGACC
AAGTTTAAAGGGATCAAAACCTACATTTCATACCGGGTCACCCCGAGTCA
CACAGCGCACCCGGTCTACAGACGCTACAAACACTTTGACTGGCTGTACA
ACCGCCTACTGCACAAGTTCACTGTGATCTCTGTGCCTCACCTGCCTGAA
AAGCAGGCCACGGGCCGATTTGAGGAGGATTTTATCGAGAAGCGCAAGAG
GCGACTGATCTTGTGGATGAACCACATGACCAGCCACCCTGTCCTGTCGC
AATACGAAGGCTTCGAGCACTTTCTCATGTGTGCTGACGACAAACAGTGG
AAGCTGGGTAAGAGGCGGGCGGAGAAGGACGAGATGGTAGGCGCCCATTT
CATGCTGACTCTCCAAATCCCCAACGAGCACCAGGACCTTCAAGATGTCG
AGGAGCGGGTTGACAACTTCAAGGCCTTTGCTAAAAAAATGGACGACAGC
GTGCTTCAGCTCACTCATGTTGCCTCCGAGTTGGTGCGCAAGCATTTGGG
TGGATTCAGGAAAGAGTTTCAGCGACTGGGTAATGCTTTCCAGTCCATCA
GCCAGGCTTTCATGCTGGACCCTCCCCATAGGTCAGACACCCTCAACAAC
GCCATCTCCCATNNNNNNNNNNNNNNNNNNNNNNNNNNNNNNNTGGGTTTCATCATTGGAGTCGGTGTGG
TGGGAAACCTC
CTGATCTCCATCCTGCTGGTTAAAGACAAGAGCCTGCATCGAGCGCCCTA
CTATTTCCTGTTGGACCTGTGCGCCTCTGATATCCTGCGCTCTGCCATTT
GCTTCCCCTTTGTCTTCACCTCAGTCAAGAATGGATCTGCGTGGACCTAC
GGCACGCTGACCTGCAAAGTGATTGCCTTCCTGGGTGTGCTCTCCTGTTT
YCACACGGCGTTCATGCTGTTCTGTGTCAGCGTCACCCGATACCTGGCCA
TCGCACATCACCGTTTCTACACCAAGAGGCTGACGTTCTGGACGTGCTTG
GCTGTCATCTGCATGGTGTGGACACTGTCGGTGGCTATGGCGTTCCCACC
GGTGCTGGACGTGGGCACCTACTCTTTTATCCGGGAGGAGGACCAGTGCA
CATTCCAGCACCGTTCCTTCAGGGCGAATGATTCACTGGGYTTCATGCTC
CTAYTGGCGCTCATCCTTCTTGCCACGCAGCTAGTTTACCTCAAGCTCAT
CTTCTTCGTCCACGACCGTCGAAAGATGAAGCCTGTCCAGTTCGTGCCTG
CTGTCAGTCAGAACTGGACTTTCCATGGGCCTGGTGCTAGCGGGCAGGCG
GCGGCCAACTGGCTGGCTGGATTCGGTCGAGGTCCGACCCCGCCTACKTT
GCTGGGCATCCGACAGAACAGCAACGCAGCAGGCCGCAGGCGTCTTCTGG
TRCTAGATGAATTCAAAACMGAGAAGAGGATTAGTAGGATGTTCTACATC
ATGACGTTTTTGTTCCTGGCGCTGTGGGGACCCTATCTGGTGGCCTGCTA
CTGGCGGGTGTTTGCACGGGGCCCTGTAGTCCCTGGAGGCTACCTGACAG
CAGCTGTGTGGATGAGCTTTGCNNNNNNNNNNNNNNNNNNNNNNNNNNNNNNNNNNNNNNNNNNNNNGC
CAAATCTCGCTTTCACCCTGGCGTGG
GGACTGGTCCTGGCACGGAGC---GCAGCGTCCCACTCGGCAACAGCTTG

CTATCCCCGCAGCAAAGCGAGGAGCCCACTGTTGCCACCCCCCCGCAGCG
ATGGTTTGTCACCC---CTGCCAACAACCGACTGGACTTTGCTGCCTCGG
CATACGACGCCGCT---------GATTTTGCCGGTAACGCGGCCACCTTG
CTGTCCTACGCAGCGGCCGGAGTGAAAGCTC------TTCCCCTGCCGGC
TGCAGGCTGCTCCAATCGGCCCCTTGGCTATTACGCAGACCCGTCTG---
GCTGG---GGAGGACGCACGCCGCCGCAGTACTGCGGCGTCAATACGAAA
TCCGGCTCGGTCTTTTCCTGCTGGCCCGCCAACTCCATCGGTAGCAGAGC
NGGCG---CC---AACTACCTGT---------CCGAGGA---GGGC---GA
CTC---CATCACGACGGAGAGGTCGCCC---AT---CGGCGCGTCGGAGG
AG---ACCAAACCCAAAGACATGAC---GTCCGA---GTCGAACTGGATA
GAG---ACGCCGTCCTCTATCAAGTCTATTGATTCCAGCGACTCTGGCAT
CTTTG---AGCAGGCCAAACGGAGGAGAATCTCACCGTCGGCCACACCG-
--------GTTTCAGAGACGGTGTCCCCGCTAAAATNNNNNNNNNNTTCAACAGAGGAAG
TCACAGAGAGAGAAGTGGCGTTGGGAATAAATCCGTTTGTGGATGGGATG
GGCGCCTTCAAAATAAACCACAGCTCCCACGATATCGGCTCCGG---ACA
GACGGCGTTTTCCTCCCAGGCT---CCCGGCTAC---GCGGCRGCTGCSC
TGGGA---CACCATCA------TCACCCGACCCACGTTGGCTCT---TAC
TCCACGGCGGCTTTCAACTCCACCAGGGACTTTCTCTTCAGAAATCGGGG
TTTCGGAGATGCCACCGG------------GGCGCAGCACAGTTTGTTCG
CCTC---------CGGAAGTTT---T------GCAGGGCCACACGGACAC
TCAGATGCAGCGGGGCACCTGCTCTTCCCGGGGCTGCACGAG---CAAGC
GGCGAGCCACGCGTCTTCGAACGTGGTCAACAGCCAGATGCGGCTGGGCT
TCTCGGGGGACATGTACGGACGGGCCGACCAGTACGGCCACGTTACGAGC
CCGAGGT---CCGACCACTAYGCCTCCACCCAGCTGCACGGCTACGGCCC
CATGAATATGAACATGGCCGCG---CATCACGGAGCAGGGGCCTTCTTYC
GATACATGAGGCAGCCGATCAAACAAGAGCTCATCTGCAAGTGGATCGAG
CCGGAGCAGCTGACGAACCCCAAAAAGTCGTGCAACAAAACTTTTAGCAC
CATGCACGAGCTGGTGACCCACCTGACGGTGGAGCATGTGGGGGGACCGG
AGCAGACCAACCACATYTGCTTCTGGGAGGAATGCTCCAGAGAAGGAAAG
CCATTCAAAGCCAAATACAAACTTGTAAATCATATCACAGTTCACACCGG
AGAAAAGCCCTTTCCGTGNNNNNNNNNNNNNNNNNNNNNN
>Opisthoproctus grimaldii

-----------------GGAGAGAAACCTGCATCCGTCCAACTGCTTGGG
CATGCTGCTGCTGTCAGATGCCCACACGTGCACCAAGCTCTCAGAGCTCT
CCTGGGGCATGTGTCTCAGCAACTTCCCTGCAATCTGCAAGACAGAGGAC
TTCCTGCAGCTGCCCAAGGACATGGTGGTTCAGCTCCTGTCCCATGAGGA
GCTGGAGACTGAGGACGAAAGGCTGGTCTACGAGGCCGCTCTCAACTGGG

TCAACTACGACCTGGAGAGGAGGCACTGCCACCTGCCGGAGATGCTGAGG
ACCGTCCGTCTGGCCCTGCTGCCCGCCATCTTCCTCATGGAGAATGTGTC
CACGGAGGAGCTGATCAACGCCCAGGTCAAGAGCAAGGAGCTGGTGGATG
AGGCCGTCCGCTGCAAGCTGAAGATCCTGCAGAACGACGGCGTGGTCAAC
AGTCTATGCGCCCGGCCCAGGAAGACCAGCCATGCCCTCTTCCTGCTGGG
CGGCCAGACCTTCATGTGTGACAAGCTGTACCTGGTGGACCAGAAGGCCA
AGGAGATTATCCCCAAGGCAGACATCCCAAGCCCCAGGAAGGAGTTCAGT
GCCTGTGCCATCGGATGTAAGGTCTATGTCACAGGGGGGA--GGGGCTC-
TGAGAACGGCGTGTCCAAAGACGTGTGGGTCTATGACACCATCCAGGAGG
AGTGGTCCAAGGCAGCACCCATGCTCATCGCCCGGTTTGGCCATGGCTCA
GCAGAGCTGAAACACTGCCTCTACGTGGTCGGAGGTCACACAGCTGCCAC
AGGCTGCCTGCCCGCCTCCCCATCT-------------------------

-----------AAGAGGGATCCAAGCAAGGGTACCCTAGAGGATCAAATC
ATTCAAGCTAACCCTGCACTGGAGGCTTTCGGTAATGCCAAAACACTGAG
AAATGATAATTCATCACGTTTTGGCAAATTCATCCGTATTCATTTCGGAC
AAAGTGGCAAATTGTCCTCTGCTGACATAGAGACTTATCTGCTTGAGAAG
TCACGTTGCACCTTTCAGCTGAAATCGGAGAGGAACTACCATATCTTCTT
CCAGATCTTGTCCAATCAAAAGCCAGAGCTGTTGGACATGCTGTTGATCA
CCAACAATCCATATGACTATGCCTACATCTCCCAAGGAGAGGTGACTGTA
GCATCCATCAATGATTCTGAGGAGCTGATTGCCACTGACAGTGCCTTTGA
TGTGCTGGGCTTTACTGCAGAGGAGAAAATGGGGATCTACAAGTTGACAG
GTGCCATAATGCACTATGGCAACATGAAGTTCAAGAACAAGCAGCGGGAG
GAGCAGGCAGAGCCTGACGGTACAGAGGCTGCTGACAAATCAGCTTATCT
AATGGGACTGAACTCTGCAGATCTTATTAAAGGACTGTGCCATCCAAGAG
TCAAGGTTGGCAATGAGTATGTCACCAAAGGTCAGAGTGTAGATCAAGTC
TACTAC--

---CGGCCCT
GAAGGACCTGGAGGAGGACATCATGGAGGGGCTGAGGGAGCGGGGGCTGG
AGGACAGCGCCTGCACCTCCGGCTTCAGCGTGATGATCAAGGAGTCCTGC
GACGGCATGGGGGACATCAGCGAGAAGCACGGCGGAGGGCCGCCCATCCC
CGAGAAGGCCGTGCGCTTCTCCATTACCATCATGTCTGTCTCCGTCCAGG
CTGACGGAGAGGAG------------------------------GAGGCG
GTCACCGTCTTCCGGGAGCCCAAGCCGAACTCGGAACTGTCCTGCAAGCC
GCTGTGCCTGATGCTTGTGGACGAGTCGGACCACGAGACACTCACGGCGG
TCCTAGGGCCTGTGGTGGCCGAGCGGAACGCCATGAAGCAAAGCCGGCTC
CTTCTGTCCCTGGGCGGGCTCCCCCGCTCCTTCCGCTTCCACTTCAGAGG
GACGGGTTATGACGAGAAGATGGTGCGTGAGATGGAGGGCCTGGAGGCCT
CTAGCTCCACCTACATCTGCACCTTGTGTGACTCCACCAGGGCAGAGGCC
TCCCGCAACATGGTGCTCCACTCCGTCACCCGCAGCCACGACGAGAACCT
GGAGCGCTACGAGTTGTGGAGGACCAACCCCTTCTCGGAGTCAGCAGAGG
ACCTGCGGGACCG-------------------------------------

-----------------------------------GCCAAATCTCGCTTT
CACCCTGGCGTAGGGACTGGTCCTGGCACAGACC---GCAGCGTCCCACT
TAGTAACAGCTTGCTATCCCCGCAACAAACCGAAGAGCACACAGTTG---
CTTCCCCGCAGCGATGGTTTGTCACCC---CTGCCAATAACCGACTGGAC
TTTGCCGCCTCGGCATACGATGCCGCCGCGGCTGCTGATTTTGCCGGTAA
CGCGGCCACCTTGCTGTCCTACGCAGCGGCTGGAGTAAAAGCAC------
TTCCCCTGCCCACGGCTGGGTGTTCAAACAGACCTTTGGGTTATTATGCC
GATCCATCCG---GCTGG---GGCACCCGCACACCACCTCAGTACTGT--
-------AGTAAATCCAGCTCAGTGCTCTCGTGCTGGCCAACAAATGCTG
TTGGAAGCATTACGGGCA---CGTCCAATTACCTGG---------CGGAG
GA---TGGG---GACAC---CATCCCTACGGAAAGGTCTCCA---AT---
AAGAGTGCCAGAGGAG---CCAAAACCAAAAGACTT------GTCCGA--
-ATCCAGCTGGATAGAG---ACGCCGTCTTCAATAAAGTCGATAGATTCA
AGTGATTCTGGAATCTTTG---AGCAGGCCAAACGGAGAAGAATTTCACC
GTCTGTCACACCG---------GTTTCGGAGACAGTGTCCCCGCTGAAAT
CAGAGCATCACTCAACAGGCGAAGTCACAGACAGAGAAGTGGCTTTGGGG
ATAAATCCGTTCGCAGACGGGATGGGCGCTTTTAAAATCAACCACAGCTC
TCATGATCTTGGCTCCGG---GCAAACGGCGTTTTCCTCCCAAGCG---C
CCGGCTAC---GCAGCCGCTGCCTTGGGA---CATCATCA------CCAT
CCGACCCATGTCAGCTCC---TACTCCACCGCTGCTTTCAATTCCACCCG
GGACTTTCTTTTTCGAAATCGGGGTTTCGGAGACGCGACCAG--------
----CGCTCAACACAGTCTGTTCGCCTCGGC---AGCGGGAAGTTT---T
------GCAGGGCCACATGGACACTCAGATGCCGCGGGACACCTGCTCTT
CCCAGGACTTCACGAG---CAAGCCGCGAGCCATGCGTCCTCCAATGTTG
TAAACAGTCAGATGCGATTGGGTTTTTCGGGGGACATGTACGGGCGGGCT
GACCAGTATGGCCATGTTACGAGCCCACGGT---CCGACCACTACGCTTC
GACCCAGTTGCATGGCTATGGCCCCATGAACATGAATATGGCCGCA---C
ACCACGGAGCAGGGGCCTTCTTCCGTTACATGAGGCAGCCGATCAAACAA
GAGCTCATCTGCAAGTGGGTCGAGCCGGAGCAGTTGTCGAACCCGAAAAA
GTCGTGCAACAAAACTTTCAGCACGATGCACGAGCTCGTGACCCACCTGA
CAGTGGAACATGTGGGGGGACCAGAGCAGTCAAACCATATTTGCTTTTGG

GAAGAGTGTTGCCGAGAAGGAAAACCATTCAAAGCTAAATACAAACTTGT
AAATCATATCAGAGTACACACYGGAGAAAAACCGTTCCCATGTCCATTCC
CCGGCTGTGGCAAA
>Opsanus pardus

NNNNNNNNNNNNNNNNNNNNNCATTCTAACTGCCTTGGCATGCTTTTGCTATTTGATGC
TCACCAGTGTACCAAGCTATCAGAGCTGTCCTGGGGTATGTGCCTCAGCA
ACTTCCCTTCTATTTGCAAGACAGAGGACTTCCTTCAGTTACCCAAAGAT
ATGGTGGTGCAGCTTTTGTCACATGAGGAGCTGGAAACAGAAGATGAGAG
ACTGGTTTATGAAGCTGCCCTGAACTGGATCAACTATGACCTAGAGAGGA
GACACTGCCACCTTCCAGAGCTTCTGAGAACAGTCCGTCTAGCGCTGTTG
CCTGCCATCTTTCTGATGGAGAATGTCTCGACGGAAGAGCTGATCAATGC
CCAGGCCAAAAGCAAGGAGCTGGTGGATGAAGCGATCCGTTGTAAGCTGA
AGATCCTGCAAAATGATGGCGTTGTTAACAGCCCGTGTGCCCGGCCGAGA
AAAACCAGCCATGCTCTGTTTCTTCTGGGCGGGCAGACCTTTATGTGTGA
CAAGTTGTACTTGGTGGATCAGAAAGCCAAAGAAATCATCCCAAAGGCTG
ACATCCCCAGTCCAAGGAAGGAGTTCAGTGCCTGCGCTATTGGCTGTAAG
GTGTATATCACTGGCGGGA--GAGGCTC-AGAGAATGGTGTATCCAAAGA
TGTGTGGGTCTACGACACTGTCCACGAGGAATGGTCCAAGGCAGCGCCCA
TGCTCATTGCCCGGTTTGGCCATGGCTCTGCAGAGTTAAAACACTGCCTC
TATGTAGTAGGAGGGCATACTGCAGCAACTGGATGCCTCCCAGCTTCTCC
ATCCGGACGAATACGTTGTTGTGTTTAGTCGCTCAACAACCAGACTGATA
CTGAACGAAGCCGAGCTCATCATGGCTCTGGCTCAGGAGTTTCAGATGAG
AGTGGTGACGGTGTCCCTGGAGGAACAGTCTTTTCCCAGCATTGTCCAGG
TGATCAGCGGTGCTTCCACGTTGGTTAGCATGCACGGAGCTCAGCTCATT
ACGTCCCTCTTCCTTCCCAGAGGAGCTGCTGTGGTTGAGTTGTTTCCCTT
TGCTGTCAACCCGGAGCAGTACACCCCATATAAAACCCTGGCCTCTCTTC
CAGGCATGGACCTCCACTACATCTCCTGGAGGAACACCATGGAGGAGAAC
ACAGTCACCCACCCGGAGAGACCCTGGGAGCAGGGAGGCATTGCTCACCT
GGCGAAAGAGGAGCAAGAGCGAATACTGGCCAGCAAAGACGTCCCGAGGC
ACCTGTGCTGCCGCAACGCAGAGTGGCTCTTCCGAATCTACCAGGACACG
CTGGTAGACGTTCCTTCTTTGGTGGAAGCCCTCAG---AGAAGGCTTAAA
G---ACGAAGCCCAATTTGAAGAA---GGCGAAGACGGCCAGCACGGTCC
ATCCGGGCCGGGTCAGGGAAGCCCAGTGTCAGACGTCTGTGCAGACCACC
AGTGAGGCCAAACTCACAGTGTCCTGGCAGATCCCGTGGAACCTGAAGTA
CCTGAAGGTGAGAGAAGTTAAGTACGAGGTGTGGATCCAGAAAAAAGACA
CCAGCAAGGGGACGCTGGAAGATCAAATCATTCAGGCGAATCCTGCACTT

GAGGCTTTTGGCAACGCCAAAACGGCAAGAAACGATAACTCTTCTCGTTT
TGGAAAATTCATCAGAATTCACTTTGGAACCAGTGGAAAGCTCTCGTCTG
CAGACATCGAGACGTACCTGCTCGAGAAGTCACGCGTCACCTTCCAGCTC
AAGGCTGAGAGGAACTACCACATCTTCTACCAAATCCTGTCCAATCAGAA
GCCAGAGCTGCTGGACCTGCTGCTGATCACCAACAACCCATACGACTACT
CCTACATCTCCCAAGGAGAGGTAACGGTGGCATCCATTAACGATCCGGAG
GAGCTGATGGCTACAGACAATGCCTTTGATGTGCTGGGCTTCACTCCAGA
GGAGAAGACGGCTGTCTACAAGCTGACCGGCGCCATCATGCACTACGGGA
ACATGAGGTTTAAGCAGAAGCAACGTGAGGAGCAGGCTGAACCTGATGGG
ACCGAGGCGGCCGATAAGTCGGCATACTTAATGGGGCTGAACCCCGCTGA
CCTCATCAAAGGCCTGTGCCATCCCCGGGTCAAGGTAGGAAACGAGTACG
TCACCAAAGGGCAGAGTGTGGACCAAGTCTACTACCCTAACAAGGAGGCA
TTCAAGTGTGAAGAGTGTGGGAAGCACTACAATACCAAGCTGGGATATAA
GCGCCATGTGGCCATGCACTCAGCCACAGCAGGAGATCTCACCTGCAAAG
TGTGTATGCAGAGCTATGAGAGCACACCAGTGCTCCTAGAGCATCTGAAG
AGCCACTCGGGGAAATCCTCAGGTGGCGCCAAGGAGAAAAAACACCCGTG
TGATCACTGTGATCGCCGTTTTTACACGCGAAAGGATGTGAGACGACACA
TGGTGGTACACACGGGCCGAAAGGACTTCCTGTGCCAGTACTGTGCTCAG
CGCTTTGGAAGGAAGGACCATCTGACACGTCACGTAAAAAAAAGCCACTC
ACAGGAGCTGCTGAAGATCAAGACAGAGCCACCTGATATGTTAGGTCTTT
TAGCTTCGGGCTCACCACCTTGCTCAGTGAAAGAGGAGCTTAGCCCCATG
ATGTGTGGCATGGGTGCCAACAAAGACCCCATGATGGGCAAACCGTTTCC
CAGTGGTGCACCTTTTCCCATGAGCATGTACAACCCTCATCAT------C
TCCAGGCCATGTCTAATTCTGGGGTTGGTCATCCA------CACCCCTCT
TTGATGCCCAGTTCGCTGTCTGCAGCTATGNNNNNNNNNNNNNNNNNNNNTACCTCATCTACGCGTCTT
T
TTCTTTCATGGGATGTTTACAGATCAGTGATGGGTCCAACATCGTAAACC
TGTTAGCTAGCAACTCTCCGAGCGTGTCGTACGCTCTGACACAGCAGAAG
TACTTCAGCAACTACAGTCCCGTGATTGGATTTTACATTTATGAACCCAT
TGAGTACTGGAACTCCACGGTGCAGGAGCACCTGAAGACTCTGAGCCACG
GCTTCAACAAGATCTCCTGGATGGACAACTTCTTCCACTACCTGCGGGTG
GTGAATGTGAGCACGTCGACCAAGAGCGACTTCATCGCGATCCTCAGAGG
CTCGTTCTTGCGCAGTCCAGCATACCAGCATTTCACAGAGGACATCATAT
TCTCTAAGA---ACCGGGAGACTG------ATGAGTATGACATCATTGCC
TCACGCATGTACTTGGTGGCACGGACCACGGAGAAGAAACGTGAGGAGGT
GGTGGAGCTTCTGGAGAAGCTTCGCCCATTGATGCTCATCAACAGCATCA
AGTTCATTGCCTTCAATCCCACTTTTGTGTTCATGGACCGCTACAGTTCT
TCTGTCATCTCGCCCATCCTGACCTCAGGCTTCAGTGTGCTCACCATCCT
CATTCTCACCTTCTTCCTGGTCATCAACCCATTAGGGAACTTCTGGCTCA
TTCTGACGGTTACGTCCGTGGAGCTTGGCGTCTTGGGTTTGATGNNNNNNNNNNNNNNNNNNNNNNNNN
NNNNNNNNNNNNNNNNNNNNNNNNNNNNNATGTAG
GCATTATTAATGGGCTTTCTGGACGAGCATCCTCCGTGGACGACATCCCA
GTCGACACCATCACTCGCCGTTTTAGATATGATGTGGCGCTGGTATCAGC
ATTAAAAGACCTGGAGGAGCATATCATGGAGGGGCTGACAGAGTGTGGAA
TGGAAGACAGCGCTTGCACCTCAGGCTTCAGCGTCATGATCAAAGAATCT
TGTGACGGCATGGGTGACGTCAGCGAGAAACATGGTGGGGGACCTGCGGT
TCCTGAGAAGGCGGTACGCTTCTCATTCACTGTTATGTCTATCTCCCTCA
TGGCAGACGGCGAGGAA-------------------------------AAG
TCGGTTACCATCTATACTGAGCCAAAGCCAAACTCAGAACTGTCCTGTAA
GCCCCTTTGCCTGATGTTTGTTGACGAGTCAGACCATGAAACCCTCACAG
CTGTCCTGGGGCCAATTGTTGCTGAGCGTGAAGCGATGAAAGACAGCAGA
CTCATTCTGTCAATGGGTGGCTTGCCTCGCTCCTTCCGCTTCTACTTCAG
AGGCACGGGATATGATGAGAAGATGGTACGTGAACTGGAGGGCCTAGAGG

CCTCAGGTTCCAGCTATATCTGCACGCTGTGTGACTCCAGTCGGGCAGAG
GCCTCTCACAATATGGTGCTGCATGCCATTACCCGCAGCCATGAAGAGAA
CCTGGAGCGTTACGAAATATGGAGAACTAATCCCTTTTCAGAGTCTGTCG
ATGAGCTGCGGGACCGTGTCAAAGGGGTCTCTGCCAAACCCTTTATGGAG
ACCCATGCAACGCTGGATGCATTACACTGTGACATTGGCAATGCCACTGA
GTTCTACAAAATCTTCCAAGATGAGATCGGGGAAGTGTACAAAAAAGC--
-CAAC---CCCACCCGTGAGGAACGGCGTAGCTGGAGAGCAGCCCTAGAT
AAACAGTTGAGGAAGAAAATCAAGCTGAAACCAATAATGAGGATGAATGG
GAACTATGCCCGGAAGCTAATGACCATGGAGGCGATGGAGGCGGTGTGTG
AGTTGGTACCCACAGAGGAGAGGAGGGAAGGCCTGAGGGAGCTCATGAGT
CTCTACATTCAGATGAAGCCTGTCTGGCGTGCCTCCTGTCCAGCCAAAGA
ATGCCCTGACCAGCTGTGCCGCTACAGCTTTAACTCCCAGCGCTTTGCTG
ACCTCCTGGCCTCCACATTCAAATATCGGTACAATGGAAAGATCACCAAC
TACCTCCACAAGACGCTGGCTCATGTCCCTGAAATTGTTGAACGAAATGG
GTCTATCGGAGCCTGGGCCANNNNNNNNNNNNNNNNNNNNNNNNNNTCGTACACTATTGAGATGGGTCCC
TTGGGG
CCGAGATGGAAGGAGAATCCACATCCTTTCTCTTGTTCCATTGAAGATCC
AACAAAACAGACAAAGTTTAAAGGCATCAAAACGTACATTTCGTACCGGG
TCACGCCAAGCCACACAGGGCGTCCTGTCTACAGGCGCTACAAGCACTTT
GACTGGCTATACAACCGGTTACTGCATAAGTTCACTGTGATCTCCGTGCC
TCACCTTCCCGAGAAACAGGCCACTGGGCGATTTGAGGAAGACTTCATCG
AGAAGCGTAAGAGACGATTGATACTGTGGATGAACCACATGACTACTCAC
CCGGTTCTCTCGCAGTATGAAGGCTTTGAGCACTTTCTCATGTGTGCTGA
TGACAAGCAATGGAAACTGGGCAAGAGACGGGCAGAGAAGGATGAGATGG
TGGGTGCCCATTTCATGCTGACCCTCCAGATTCCTATTGAGCACCAAGAC
CTTCAGGATGTAGAGGAGCGAGTTGATTCCTTCAAGACATTTGCTAAGAA
AATGGACGATAGTGTCATGCAGCTCACACTTGTTACCTCGGAGCTGGTGC
GCAAGCACCTTGGTGGATTCAGGAAGGAGTTCCAACGGCTAGGAAATGCC
TTTCAGTCTATCAGTCAGGCGTTCATGCTGGACCCTCCCCATTCTTCAGA
GGCTCTCAATCATNNNNNNNNNNNNNNNNNNNNNNNCCGTTCTCAAACTGACCTCCCTGGGTTTCATCATC
GG
CGTCGGCGTGGTCGGAAACCTCCTGATTTCCATCCTGCTGGTCAAAGACA
AGAGCCTGCACCGGGCGCCCTACTACTTCCTGCTGGACCTGTGCGCTTCC
GACATCCTCCGCTCGGCCATCTGCTTCCCCTTCGTCTTCACCTCGGTCAA
GAATGGGTCCGCCTGGAGCTACGGCACGCTCACCTGCAAAGTGATCGCCT
TCCTGGGCGTGCTCTCCTGCTTCCACACGGCGTTCATGCTCTTCTGCGTC
AGCGTCACCCGCTACCTGGCCATCGCGCACCACCGTTTCTACACCAAGCG
GTTGACCTTCTGGACGTGCCTGGCCGTCATCTGCATGGTGTGGACGTTGT
CGGTGGCCATGGCGTTCCCGCCGGTGCTGGATGTGGGGACGTACTCTTTT
ATCCGCGAGGAGGACCAGTGCACGTTCCAGCACCGCTCCTTCCGGGCGAA
CGATTCCCTCGGCTTCATGCTCCTGYTGGCGCTCATCCTCCTGGCCACAC
AGCTGGTTTACCTCAAGCTTATCTTCTTCGTCCACGACCGTCGAAAGATG
AAGCCCGTCCAGTTTGTGCCTGCCGTCAGCCAGAACTGGACTTTCCASGG
GCCGGGCGCAAGCGGCCAAGCGGCGGCCAACTGGCTAGCTGGATTCGGTA
GAGGCCCCACCCCGCCTACTCTGCTAGGCATCCGGCAGAACAGCAACGCG
GCGGGCCGCAGGCGTCTACTGGTGTTGGATGAGTTTAAAACGGAGAAGAG
GATTAGTAGGATGTTCTACATCATGACGTTTTTCTTCCTGGCTCTGTGGG
GGCCCTACCTTGTCGCCTGCTACTGGCGGGTTTTTGCAAGGGGCCCCGTG
GTCCCCGGAGGGTATCTGACGGCGGCCGTGTGGATGAGCTTTGCCCAAGC
CGGGGTCAACCCTTTCATCNNNNNNNNNNNNNNNNNNNNNNGCCAAATCTCGCTTTCACTCTGGCGTGGGG
A
CTGGCCCTGGCACGGAGC---GCAGCGTCCCACTCGGCAACAGCTTGCTG
TCCCCGCACCAAACCGAGGAGCCCACTGTTGCCTCCCCCCCGCAGCGATG

GTTTGTCACCC---CTGCCAACAACCGACTGGACTTCGCGGCCTCAGCAT
ACGATGCGGCT---------GATTTCGCCGGTAACGCGGCCACCTTGCTG
TCCTACGCAGCGGCTGGAGTGAAGGCTC------TTCCCATGTCGGCGGC
GGGCTGCTCCAACCGGCCTCTTGGCTATTACGCAGACCCGTCCG---GCT
GG---GGAGGACGCACGCCGCCGCAGTACTGCGGCGTCAATAACAAATCC
AGCTCGGTGTTCCCCTGCTGGCCCGCCAACTCCATCGGCGGCAGAACGAA
CG---CC---AACTACCTGA---------GCGAGGA---GGGA---GACT
C---CATCCCCACGGAGAGGTCACCG---AT---CGGCGTCTCCGAGGAG
---ACCAAACCCAAAGACGT------GCCAGA---GTCGAACTGGATAGA
GACCACGCCCTCCTCCATTAAGTCCATGGACTCCAGCGATTCTGGGATCT
TTG---AACAAGCCAAGAGGAGACGGATCTCACCGTCGGCCACGCCC---
------GTGTCGGAGACCGTGTCCCCGCTCAAGTCTGAG-----------

>Opsariichthys uncirostris
AGCCTCCTTATTCGAGCCGAACTAAGCCAACCCGGGTCACTTCTAGGCGA
CGATCAAATTTATAATGTTATTGTTACTGCCCACGCCTTTGTTATAATTT
TCTTTATAGTAATACCAATTCTTATTGGAGGGTTTGGAAACTGACTTGTG
CCGCTAATGATTGGGGCACCTGACATGGCGTTTCCTCGAATAAATAACAT
AAGCTTTTGACTTCTCCCTCCCTCTTTCCTCCTGTTATTAGCCTCTTCTG
GTGTTGAGGCCGGGGCTGGGACAGGATGAACAGTGTACCCGCCACTTGCA
GGCAATCTCGCCCATGCGGGCGCATCTGTAGACCTAACAATTTTCTCACT
CCACCTAGCAGGGGTTTCATCAATTTTAGGAGCAATTAATTTTATTACTA
CAACTATTAATATGAAACCCCCAGCCATCTCTCAATATCAAACACCCCTG
TTTGTTTGAGCCGTACTTGTGACGGCTGTGCTTCTTCTTCTATCCCTACC
CGTATTGGCTGCCGGGATTACGATGCTCCTTACAGACCGAAATCTTAACA
CCACATTCTTCGACCCTGCGGGAGGAGGAGACCCAATTCTATATCAACAC
CTATTCTGATTCTTCGGTCACCCAGAAGTCTACATTCTTATTCTACCAGG
ATTCGGAATTATCTCCCACGTCGTAGCCTATTACTCCGGGAAAAAAGAGC
CCTTCGGTTATATGGGAATAGTTTGAGCTATAATGGCCATTGGCCTTTTA
GGATTTATTGTGTGAGCCCATCATATGTTTACTGTCGGAATAGACGTAGA
CACTCGTGCTTATTTCTGGAGAAGAACCTCCACCCGTCCAACTGCCTGGG
CATGCTGCTGCTCTCGGACGCTCACCAGTGCACCCAGCTGTTCCAGCTGT
CCTGGAGCATGTGTCTCAGTAACTTCCCTGCTATCTGCAAGACTGAGGAG
TTTCTCCAGCTGCCCAAGGACACGCTGGTCCAACTGCTGGCGCACGAAGA
GCTCGAAACCGAGGACGAGCGTCTGGTCTATGAGTCTGCGCTCAACTGGG
TAAACTACGACCTTGAGAGGAGGCACTGTCACCTTCCGGAGCTGCTCCGT

ACTGTGCGTCTGGCTCTCCTTCCTGCCATCTTCCTTATGGAGAATGTCTC
TACGGAGGAGCTCATCATCGCAGAGGCTAAAAGCAAAGAGCTGGTAGATG
AGGCCATCCGCTGCAAACTGCGCATCTTGCAAAACGACGGCGTCGTCAAC
AGTCCTTGCGCCCGGCCCCGCAAGACCAGTCATGCCCTTTTCTTGCTGGG
CGGCCCCACGTTTATGTGCGACAAGCTGTACCTGGTGGACCAGAAGGCCA
AAGAGATCATTCCAAAAGCAGACATCCCCAGCCCAAGAAAGGAGTTCAGC
GCCTGTGCCATTGGCTGCAAAGTGTACGTGACGGGTGGCC--GGGGCTC-
AGAGAACGGAGTGTCTAAGGATGTTTGGGTGTACGATACATTACACGAGG
AATGGTCCAAAGCGGCTCCGATGCTGATAGCACGTTTCGGTCATGGTTCC
GCTGAGATGCGCCACTGCCTGTATGTCGTAGGAGGTCACACAGCTGCCAC
NNNNNNNNNNNNNNNNNNNNNNNNCGATGACTACATTGTGGTTTTTAAACGTACCACTAACAGGCTCAT
CCTTA
ATGAGGCAGAACTTCTTCTGGCTTTAGCCCAAGAGTTTCAAATGAGGACT
GTCACTGTGTCTTTGGAGGAGCAGTCGTTCGATAGCGTCATCCAAATGAT
CAGTGGGGCGACTATGTTGGTCAGCATGCACGGAGCCCAGATGGTCACCT
CCATGTTCTTGCCCCGTGGTGCCGCTGTGGTTGAACTCTTCCCTTATGCT
GTAAACCCAGAGCAGTACACTCCCTATAAAACCTTGGCCTCTTTGCCCGG
CATGGACCTTCAGTATGTCGCATGGAGAAATACCAATGAAATGAACACCG
TTACATATCCCGATCGGCCCTGGGATCAGGGTGGCATCGTCCACTTGGAC
AAAGAGGAACAAGAACGTATCCTCGCCAGCAAGGAGGTGCCGAGACATCT
TTGTTGTCGTAATCCAGAGTGGCTTTTTCGCATTTACCAGGACACTATTG
TGGACATTGCCTCTTTTCTGGACGTACTTAG---AGAAGCTCTAAAA---
---AGGCCCAATCTCAAAAA---GGCAAAGGTGGCTAGCACTGTACATCC
TGGTCGAGTCAGAGAGCCCAAGTGTCAGACGTCGGTACAGGCCACCAATG
AAGCAAAGCTCTCAGTGTCATGGCAGATTCCTTGGAATCTGAAGTACTTG
AAGGTGAAAGAGGTGAAATATGAGGTGTGGATCCAGAAGAAAGACTCTAA
CAAGGGAACCCTGGAGGATCAGATTATTCAGGCCAATCCTGCACTCGAGG
CTTTTGGCAATGCTAAAACGCTCCGAAACGACAACTCCTCCCGCTTTGGG
AAATTCATCCGCATTCATTTTGCAACAAGCGGTAAACTCGCTTCAGCGGA
TATCGAAACATACCTGCTGGAGAAGTCTCGGGTGACGTTTCAGCTGAAGT
CGGAGAGGAACTACCATATTTTCTTCCAAATACTGTCCAATGAAAAGCCT
GAACTGCTGGACATGCTGTTGATAACTAATAATCCATATGACTACTCTTA
CATCTCCCAAGGAGAAGTAACCGTAGCTTCCATCAATGACAACGATGAGC
TGATTGCGACCGACAAGGCATTTGATGTACTCGGCTTCACGTCCGAAGAA
AAGATGGGAGTCTACAAGCTGACAGGTGCTATCATGCACTATGGCAATAT
GAAATTCAAACAGAAACAGCGTGAGGAGCAGGCAGAGCCTGACGGTACTG
AAGATGCAGATAAGGCGGCATATCTAATGGGGCTGAACTCTGCTGACCTG
CTCAAAGGACTCTGCCATCCAAGGGTCAAGGTAGGCAATGAGTATGTCANNNNNNNNNNNNNNNNNNNN
NNNNNNNNNNNNNNNA
CAAGGAGGCCTTTAAGTGCGAGGAGTGTGGCAAACACTATAACACAAAAC
TGGGCTACAAGCGCCACATGGCTATGCACTCAGCCACAGCCGGTGATCTT
ACCTGTAAGGTGTGCTTGCAAAGCTACGAGAGCACGCCTGCACTCTTGGA
GCACCTCAAGAGCCACTCAGGCAAGTCTTCAGGTGGAGCCAAGGAGAAGA
AACACCCCTGTGACCACTGTGATCGTCGCTTTTACACACGAAAGGATGTT
CGCCGACACATGGTGGTACACACGGGACGCAAAGACTTCCTGTGCCAGTA
CTGTGCCCAACGTTTTGGGCGGAAGGACCATCTGACGCGACACGTCAAGA
AGAGTCACTCGCAGGAGCTGCTGAAGATCAAGACGGAACCTCCGGATATG
TTGGGTTTACTTGGGTCTGGTTCTCCACCATGTGCTGTGAAAGAAGAAAT
AAGCCCTATGATGTGTAGCATGGGACCTACCAAGGACTCGATGATGGCAA
AGCCTTTCCACGGTGCCACCCCTTTTCCCATGGGCATGTACAACCCTCAT
CAT------CTCCAGGCCATGTCCAATTCGGGTGTGGGCCAC--------
-CATCACTCATTGGTTCCTGGGACTTTATCCACAGCTATGGGCATGNNNNNNNNNNNNNNNTACC
TGATTTACGCCTCCTTTTCATTCATGGGATGTTTACAAATCAGTGATGGC

TCCAACATAATTAATCTGCTAGCTAGTAATTCACCCAGTGTGTCGTTCGC
CCTGACTCAACAGAAATACTTCAGCAACTACAGTCCTGTGATTGGGTTTT
ACATCTACGAACCCATCGAGTACTGGAACTCCACAGTGCAGGAGCACCTC
AAGACATTGGGCCAGGGCTTTAATAAGATTTCATGGATCGATAATTACTT
CCACTATCTGAGGGTCGTTAATGTCAGCGCATCAACCAAAAGTGATTTCA
TTAACATCCTCAAGACTTCTTTTCTGAGGAGCCCAGAATATCAACACTTT
GTGGACGACATCATTTTCTCTAAAA---ATGGAG------------ATGA
GTACGACATCATCGCGTCCAAGATGTACTTGGTGGCCAGGACGACCGAGA
AGACCAGGGAGGAGGTCGTGGAGTTGCTGGAGAGACTTCGACCTCTCTCC
CTTATAAACAGCATCAAGTTCATCGTTTTCAACCCAACGTTCGTGTTCAT
GGACCGCTACAGTTCCTCCATCATCTCCCCCATCCTGACGTCGGGTTTCA
GCGTGCTCACTATTCTCATCCTCACCTTCTTCCTGGTCATTAACCCACTG
GGGAACTTCTGGTTGATCTTGACCGTCACCTCGGTGGAACTGGGCGTCTT
GGGTCTCANNNNNNNNNNNNNNNNNNNNNNNNNNNNNNNNNNNNNNNNNNNNNNNNNNNNNNNNNNNN
NGCATCATTGACGGCCTCTCTGGTTGGACAACCTTTGTTGAGG
ATGTCCCTGCAGAGACCATCGCCAGACGATTCCGCTATGATGTGGCACTG
GTTTCTGCATTAAAAGACCTGGAGGAGGACATAATGGAAGGACTAAGAGA
GAGGGGGCTGGATGACAGCATGTGCACCTCGGGCTTTACTGTGGTGGTGA
AAGAATCATGTGATGGTATGGGAGATGTCAGTGAGAAGCATGGAACTGGT
CCAGCGGTTCCTGAGAAGGCAGTGAGGTTTTCCTTCACAATCATGTCCAT
CTCCATCCGAGTTGAGGGTGAGGAC-------------------------
-----GATGGCATCACCATCTTCCAAGAACAAAAGCCAAACTCTGAGCTC
TCTTGTAGACCTTTGTGCCTCATGTTTGTGGATGAGTCCGACCACGAGAC
CCTGACAGCTATCTTGGGACCTGTGGTAGCAGAGCGAAAAGCCATGATGG
AAAGTCGGCTCATCATATCTGTTGGTGGTCTGCTACGCTCATTTAGGTTT
TTCTTCCGGGGCACAGGCTATGATGAGAAGATGGTTCGTGAAATGGAAGG
GCTAGAAGCATCAGGCTCCACTTATATATGCACACTCTGCGACTCAACCC
GAGCTGAAGCCTCTCAAAACATGGTCCTACATTCCATCACACGTAGCCAC
GATGAAAATCTTGAACGCTATGAGATCTGGAGGACAAATCCTTTCTCAGA
GTCCGCAGATGAACTCCGTGACCGGGTCAAAGGTGTTTCCGCCAAGCCTT
TCATGGAGACCCAGCCTACTCTGGATGCCCTGCACTGCGACATAGGCAAT
GCGACTGAATTCTACAAGATCTTTCAGGATGAGATCGGCGAAGTCTACCA
GAGGAG---CAAT---CCGAGTCGAGAGGAACGCCGTCGTTGGCGGTCAA
CCCTGGACAAGCAGCTACGAACTAAAATGAAACTGAAGCCTGTGATGAGG
ATGAATGGAAACTATGCCCGTCGGCTGATGACACGTGAGGCTGTGGAGGT
GGTCTGTGAGCTGGTCCCTTCCGAGGAACGACGTGAGGCACTACGGAGAC
TGATGGAACTCTACCTCCAGATGAAGCCGGTGTGGCGTTCCACCTGTCCC
TCCCGAGACTGCCCTGACCAGCTCTGCCGATATAGCTACAACTCTCAGCA
ATTTGCTGACATCCTTTCCACCACATTTAAGTACCGCTATGATGGAAAAA
TTACCAACTACCTCCACAAGACTCTGGCTCATGTTCCTGAGATTGTTGAG
AGAGACGGGTCTATTGGAGCATGGGCCAGTGAAGGGAATGAGTCTGGAAA
CAAGTCGTACTCCATTGAAATGGGCCCCCGGGGGCCGCAGTGGAAGGAGA
GCCCTCAACCCTTCTCCTGTTCCATTGAAGACCCCACCAAACAGACGAAG
TTCAAAGGTATTAAGACGTACATCTCGTACCGCGTGACCCCCAGCCATAC
TAGCAGGCCAGTTTACCGCCGCTACAAGCACTTTGACTGGCTGTACAACA
GACTACTGCACAAATTCACAGTGATTTCTGTGCCGCACCTCCCCGAGAAA
CAGGCCACTGGACGGTTCGAGGAAGACTTCATCGAGAAGAGGAAGAGGAG
GCTCATTTTATGGATGGATCACATGACCAGCCACCCTGTGCTGTCCCAGT
ATGAGGGTTTCGAACACTTCCTCATGTGCGGAGACGACAAACAGTGGAAA
CTCGGGAAGAGACGGGCAGAGAAGGACGAGATGGTTGGGGCTCATTTCAT
GCTCACCTTCCAGATTCCCAACGAGCACCAGGACCTTCAGGACGTGGAGG
AGCGTATTGACTCCTTCAAGGCGTTCGCCAAAAAGATGGACGACAGTGTG
ATGCAGTTGACGCACGTCGCGTCGGAACTAGTCCGCAAACATCTTGGAGG

ATTCCGGAGGGAGTTCCAGAGGCTGGGGAACGGCTTCCAGTCCATCAGCC
AGTCATTCCTGCTGGACCCTCCTTACAGCTCGGACGCCCTGAGCAACGCC
ATCTCGCAC---

--GCCA
AGTCCCGCTTTCACCCTGGCGTCGGAGGCGCTCCTGGCACGGACC---GC
AGCGTCCCACTTAGTAACAGCTTGCTCTCCCCGCAACAAACCGAGGAGAC
CACGGTGG---CCTCCCCGCAGCGATGGTTTGTCACCC---CTGCCAACA
ATCGACTGGACTTTGCCGCCTCGGCATACGATGCTGCCGCGGCTGCTGAT
TTCGCCGGTAACGCGGCCACCCTTCTGTCGTACGCAGCTGCCGGAGTTAA
AGCGC------TGCCCCTGCCCGGGGCGGATGCTCCAACAGACCTCTGG
GGTATTACGGCGAGCCGCCGG---GGTGG---GGCACGCGCACGCCACCG
CAATATTGC---------AGCAAGTCCAGCTCCGTCCTCTCGTGCTGGCC
GTCCAACTCCGTAGGGAGCAGAACGACCT---CGTCCAGTTACCTGGTCC
CTGGCCTCGAGGA---CGGA---GACGC---CATCGCGCCCGAGAGGTCA
CCG---CT---AGGCGGAGCGGATGAG---TCCAAACCCAAAGACCT---
---GTCTGA---ATCCAGCTGGATAGAA---ACCCCGTCCTCAATTAAGT
CGATCGATTCGAGCGATTCTGGGATTTTTG---AGCAAGCCAAGCGGAGG
AGGATTTCGCCATCTGCCACGCCG---------GTTTCAGAGACATCGTC
TCCATTAAAATCTGAA---------NNNNNNNNGTTACAGACAGAGAAGTGGCTTTGG
GTATCAATCCGTTCGCCGACGGCATGGGCGCTTTTAAAATCAACCACAGC
TCCCACGATCTTGGCTCTGG---GCAAACAGCGTTTTCCTCGCAAGCG--
-CCCGGCTACGCCGCCGCCGCTGCCCTGGGA---CACCATCA------TC
ACCCCACTCATGTGAGCTCG---TACTCCACCGCCGCCTTCAACTCCACT
CGGGACTTTCTGTTTCGCAATCGGGGCTTCGGAGACGCCACGAG------
------CGCGCAGCACAGCCTCTTCGCCTCCGC---CGCTGGAAGTTT--
-C------GCCGGGCCACATGGACACTCTGACGCCGCTGGGCACCTGCTC
TTCCCGGGACTGCACGAG---CAAGCGGCCACGCACGCGTCCTCCAACGT
GGTGAACAGTCAGATGCGCCTGGGCTTTTCCGGGGACATGTACGGCAGGG
CCGAGCAATACGGTCACGTCGCGAGTCCGAGGT---CCGAGCACTACGCC
TCGACTCAGTTGCACGGCTACGGCCCCATGAACATGAACATGGCTGCT--
-CATCACGGGGCAGGGGCCTTCTTTCGGTACATGCGACAGCCCATAAAGC
AAGAGCTCATCTGCAAATGGGTCGAACCCGAGCAGTTGACGAATCCGAAA
AAGTCCTGCAACAAAACTTTCAGCACCATGCACGAGCTTGTGACCCACCT
GACGGTGGAGCACGTTGGGGGACCGGAGCAGTCGAATCACATTTGCTTTT
GGGAAGAGTGTTCCCGGGAAGGGAAACCGTTTAAAGCAAAGTATAAACTC

GTGAATCATATCCGAGTGCACACCGGAGAGAAACCGTTTCCGTGTCCGTT
CCCCGGCTGTGGAAAA
>Oryzias latipes
AGCCTGCTAATTCGGGCTGAATTAAGTCAGCCCGGGTCTCTATTAGGTGA
CGACCAAATCTATAATGTAATTGTAACCGCGCATGCCTTCGTAATAATTT
TCTTTATAGTAATACCAATCATGATTGGGGGCTTTGGCAACTGATTAATT
CCTCTAATGATCGGGGCCCCTGATATGGCCTTTCCTCGAATAAACAACAT
GAGCTTCTGACTACTTCCCCCCTCCTTCCTTCTATTGTTAGCTTCCTCTG
GCGTGGAGGCCGGTGCGGGGACAGGATGGACCGTGTACCCGCCTCTATCA
GGCAACCTAGCGCATGCGGGAGCATCCGTGGATTTAACCATCTTTTCCCT
CCATCTGGCAGGTATCTCCTCTATCTTAGGGGCTATTAATTTTATTACAA
CAATTATTAATATAAAACCCCCGGCCATCTCTCAGTATCAGACCCCTTTA
TTCGTGTGAGCTGTACTAATTACCGCAGTATTACTTCTACTCTCCCTCCC
TGTGCTCGCTGCAGGTATCACTATGCTCCTAACGGACCGAAACCTAAATA
CAACATTCTTTGACCCTGCAGGGGGAGGGGACCCTATTCTCTACCAGCAC
CTGTTCTGATTTTTTGGTCACCCCGAAGTTTATATTTTAATTCTCCCCGG
CTTCGGAATAATTTCACATATTGTGGCCTATTATTCGGGTAAAAAAGAAC
CTTTCGGGTACATGGGTATGGTCTGAGCCATAATGGCAATTGGGCTTCTA
GGGTTTATTGTATGAGCCCACCACATATTCACCGTAGGGATAGATGTGGA
TACTCGGG----TTCCTTGAGAGAAACCTTCATCCATCCAACTGCCTTGG
CATGCTGCTGCTGTCTGACGCCCACCAGTGCACCAAGCTGTCAGAGCTCT
CCTGGGGCATGTGTCTTAGCAACTTCCCTGCTATTTGCAAGACAGAGGAT
TTCCTCCAACTGCCCAAAGACATGGTGGTGCAGCTGTTGTCCCATGAGGA
ACTAGAGACAGAAGATGAGAGACTGGTTTATGAAGCTGCCCTCAACTGGA
TCAACTACGATCTGGAGAGGAGGCACTGCCATCTTCCAGAACTCCTCAGA
ACAGTCCGCCTTGCTCTGCTTCCTGCCATCTTTTTAATGGAGAATGTGTC
AACAGAGGAGCTGATCAACGCTCAGGCAAAGAGTAAGGAACTGGTAGATG
AGGCTATACGCTGTAAGCTTAAGATCCTGCAGAATGACGGCGTTGTCAAC
AGCCCATGTGCTCGACCAAGAAAAACCAGCCATGCCCTCTTCCTTCTGGG
TGGGCAGACCTTCATGTGTGACAAGTTGTACTTGGTTGATCAGAAAGCCA
AAGAGATCATTCCCAAAGCTGACATTCCCAGTCCTAGGAAAGAATTCAGT
GCATGTGCCATTGGTTGTAAAGTCTACATTACAGGTGGAA--GGGGATC-
AGAGAACGGAGTGTCCAAAGATGTCTGGGTCTATGATACTGTCCACGAGG
AATGGTCAAAGGCTGCCCCCATGCTAATCGCCCGGTTGGGACATGGGTCC
TCTGAGCTGAAACACTGTCTCTATGTCGTTGGAGGACACACTGCAGCTAC
TGGTTGCTGGCCGGCTTCTCCATCTTGATCAATACGTCGTCATCTTTAGC
CGTTCAACGACTAGGTTAATCCTGAATGAAGCTGAGGTGGTCATGGCCAT
AGTCCAGGAGTTACAGATGAGGGTGGTCACAGTTTCTCTAGAGGAACAAT
CTTTTCCCAGCATCGTCCAGGTGATCAGCGGTGCCTCCATGCTTGTCAGT
ATGCATGGAGCTCAGCTCATTACCTCACTGTTCCTTCCCAGAGGTGCTGT
TGTTGTTGAATTGTTCCCATTTGCTGTCAACCCTGAGCATTACACCCCAT
ATAAAACCCTGGCGTCCCTTCCCGGCATGGACCTTCACTACATTTCCTGG
AGGAATACTAAAGAAGAAAACACTGTCACCTATCCAGAAAGACCCTGGGA
GCAAGGAGGAATAGTTCACCTGGAAAAAGAGGATCAGAGGCGAATTTTGA
AAAGCAAGGAAGTCCCGCGACACTTGTGCTGCCGGAACCCAGAGTGGCTC
TTCCGCATCTACCAAGACACTTTGGTGGACATACCTTCTTTCATCGAGCT
TCTCAA---AGAGGCTTCGAAG---ACCAAGCTAAGCCTGAGAAA---AG
ACAAAGCGTCCAGTACGGTCCACCCTGGTCGCGTCAGAGAAGCCCAGTGT
CAAACTTCAGTTCAAACGACCAAGGAAGCCAAGCTCTCCGTCTCCTGGCA
GATCCCATGGAATCTAAAATACCTGAAGGTTAGAGACGTGAAGTACGAAG
TGTGGATCCAGAAAAAAGATAGCAGCAAGGGTACATTGGAGGATCAAATC
ATTCAGGCAAACCCTGCACTTGAGGCCTTTGGGAATGCCAAAACGGCGAG
AAATGACAACTCTTCTCGTTTTGGTAAATTCATCCGTATTCATTTTGGCA

CAAGTGGCAAACTGTCATCTGCCGACATTGAAACATACCTGCTGGAGAAG
TCCCGTGTCACCTTTCAGCTCAAGGCTGAAAGGAATTATCACATCTTCTA
CCAGATCCTGTCCAATCAAAAGCCAGAGCTGCTGGACCTGCTGCTCATAA
CAAACAACCCATACGACTATTCCTACATCTCCCAAGGAGAGGTAACTGTT
GCCTCCATCAATGACTCGGAGGAGCTGATGGCCACTGACAGTGCCTTTGA
TGTCCTTGGCTTCACTCAAGAGGAGAAAATGGGTGTCTACAAGTTGACCG
GTGCCATAATGCACTACGGCAACATGAAGTTCAAACAGAAGCAGCGGGAG
GAGCAGGCTGAACCTGACGGCACAGAGGCTGCTGATAAATCAGCTTACCT
AATGGGGCTGAACTCTGCAGACCTCATTAAAGGACTCTGTCATCCCAGAG
TTAAGGTTGGAAATGAATACGTCACCAAAGGCCAAGGTGTAGATCAAGTC
TACTACCCCAACAAGGAGGCCTTCAAGTGTGACGAGTGCGGAAAGCACTA
CAACACCAAGCTGGGATACAAACGCCACGTGGCCATGCACTCTGCAACTG
CGGGGGACCTGACCTGTAAAGTGTGCATGCAGAGCTACGAGAGCACCCCC
GTTCTCCTGGAGCACCTCAAGAGCCACTCGGGGAAGTCCTCGGGTGGTGC
CAAGGAGAAGAAGCATCCATGTGACCACTGTGACAGACGTTTCTACACGC
GGAAGGACGTGCGGCGTCACATGGTGGTCCACACGGGCCGAAAGGACTTC
CTGTGCCAGTACTGTGCCCAACGCTTCGGCAGGAAGGACCATCTGACGCG
CCACGTGAAGAAGAGCCACTCTCAGGAGCTGCTGAAGATTCAAACAGAAC
CTCCGGATATGCTGGGCCTCCTGGCGTCGGGGTCTCCGCCGTGCTCTGTG
AAGGAGGAGCTCAGTCCCATGATGTGTGGCATGGCGCCCAACAAAGACCC
CATGATGGGCAAGTCGTTCCCGAGCGGGGCATCTTTTCCGATGAGCATGT
ACAACCCCCACCAT------TTGCAGGCCATGTCTAAACCCGGGGTGGGT
CATCCA------CACCCGTCCCTGATGCCCAACTCCTTGTCTGCAGCTAT
GGGCATGGGCTGTTCCATGGAGTATCTCATCTATGCCTCTTTCTCCTTCA
TGGGATGTTTACAAATCAGTGATGGATCGAACATTGTGAACCTGCTGGCT
AGTAACTCACCAAGCGTTTCCTACGCTTTAACCCAACAGAAATACTTCAG
TAACTATAGTCCTGTTATTGGATTCTACATTTACGAACCCATAGAGTACT
GGAACTCCACTGTGCAGGAGCACCTAAAGACTTTGAGCCATGGATTCAAC
AAGATCTCTTGGATGGACAACTTTTTCCACTACCTTCGGGTGGTGAACGT
GAGCGCATCAACCAAGGGCGACTTTATCTCCATCCTGAAGGGCTCTTTCT
TGCGCAGCCCGGAGTACCAGCATTTCACGGAGGACATAATATTCTCTAGG
A---ACCGCGAGACTA------ACGAATACGACATTATTGCCTCACGAAT
GTACTTGGTAGCACGGACGACTGAAAAGAAGCGAGAGGAGGTGGTGGAGC
TTTTGGAGAAGCTCCGCCCATTGATGCTAATCAACAGCATCAAGTTCATT
GCCTTCAACCCCACATTTGTTTTCATGGACCGCTACAGCTCATCCGTCAT
CTCCCCTATCTTGACCTCAGGCTTCAGTGTACTTACCATTCTAATACTCA
CATTCTTCCTGGTTATCAATCCCCTTGGAAATTTCTGGCTCATTCTCACT
GTTACTTCTGTTGAGCTGGGTGTCTTGGGATTGATGGGCTTTCACCTTTT
TGAATGGCAGCCAGCCCTCAAAAATGTGTCTACATCTTGCAATGTTGGCA
TTATTAATGGACTCTCCGGGTGGACGTCATCTGTTGATGATATCCCGGCT
GAGACCATCACTCGGCGCTTTCGCTATGATGTGGCACTGGTGGCAGCATT
GAAGGATCTGGAGGAGGACATCATGGATGGGCTGAGAGAGAGTGGGATGG
AAGACAGCACTTGCACCTCAGGCTTCAGCGTTTTGATCAAAGAATGCTGT
GATGGCATGGGTGATGTCAGTGAGAAACACGGTGGGGGACCAGCTGTTCC
TGAGAAAGCTGTCCGCTTCTCTTTCACCATCATGTCTATATCTGTCCAAG
TAGAAGACGGAGAC------------------------------AAAGAA
GTTACCATTTTCACAGAGCCAAAGCCAAACTCAGAGCTGTCCTGCAAGCC
CCTTTGCTTAATGTTTGTGGATGAGTCAGACCACGAGACTCTCACAGCCA
TCCTGGGGCCGATAGTGGCAGAGAGAGAGATTCAATGAAACACAGCAGGCTC
ATATTGTCCATTGGAGGCCTGCAACGCTCCTTTCGCTTCCACTTTAGGGG
CACGGGTTACGATGAGAAAATGGTGCGTGAAATGGAGGGCCTGGAGGCCT
CGGGGTCAACTTATGTTTGCACACTGTGTGACTCCAGCCGAGCAGAGGCT
GCTCAGAACATGGTCTTACACTCCGTCACCCGCAGTCACGACGAGAATCT

TGAACGCTATGAAATATGGAGAACNAACCCCTATTCTGATTCTGTGGATGA
GCTGCGGGACAGAGTCAAAGGCGTGTCTGCCAAGCCGTTCTTGGAGACGC
AGCCCACGCTGGACGCATTACACTGCGACATCGGCAATGCCACAGAATTT
TACAAAATCTTCCAGGATGAAATCGGAGAAGTGTACAAAAAGGT---CAA
C---CCCAGTCGGGAGGAACGGCGCAGTTGGAGAGCAGCCTTAGATAAAC
AGCTGAGGAAAAACATGAAGCTCAAGCCAATAATGAGGATGAATGGAAAT
TACGCCCGCAAGCTAATGACCATGGAGGCTGTGGAGGTGGTGTGTGAGCT
GGTGCCATCCGAGGAGAGGAGAGAGGCTCTTAGGGAGCTGATGAAGCTCT
ACCTTCAGATGAAGCCAGTGTGGCGGGCCACATGCCCAGCCAAGGAGTGC
CCTGACCAGCTGTGTCGCTACAGTTTCAACTCCCAGCGTTTTGCTGACCT
CCTCTCCTCTACCTTTAAATACAGGTACAATGGAAAGATAACAAATTACC
TGCACAAGACCCTGGCTCATGTGCCTGAAATCATAGAGAGAGATGGATCC
ATAGGGGCCTGGGCCAGTGAGGGAAACGAGTCAGCAAACAAATCTTACAC
TATTGAAATGGGTCCTTTGGGACCCAAGTGGAAGGAAAACCCCAACCCTT
TCATCTGCTCAATTGAAGATCCCACCAAACAGACAAAGTTCAAGGGCATC
AAGACCTACATATCATACAGGGTCACACCAAGTCACACCGGGCGTCCCGT
CTACCGGCGCTACAAGCACTTCGACTGGTTGTACAACCGACTGCTAAACA
AGTTCACTGTGATCTCAGTCCCTCATTTGCCTGAGAAGCAAGCAACGGGG
CGCTTTGAAGAGGACTTCATCGAGAAGCGCAAGAGGCGCCTGATACTGTG
GATGAACCACATGACCAGTCACCCCGTTCTCTCCCAGTACGAAGGCTTTG
AGCACTTCCTCATGTGTGCCGACGATAAACAGTGGAAACTGGGCAAGAGG
CGGGCTGAGAAGGATGAAATGGTGGGCGCCCATTTCATGCTGACTCTTCA
GATCCCCAATGAGCACCAGGACCTTCAGGATGTGGAGGAGCGGGTTGACA
ACTTTAAGACCTTCGCCAAAAAAATGGATGACAGCGTGATGCAGCTCACA
AATGTGGCCTCCGAGCTGGTGAGAAAACATCTGGGTGGATTCCGGAAGGA
GTTCCAGCGACTGGGGAACGCCTTTCAGTCCATCAGCCAGGCGTTTATGC
TGGACCCCCCCCATCGCTCGGACGGCCTCAACAATGCCATCTCCCACCCT
CTCGCCACGTTCCTCAAACTGACCTCTCTGGGTTTCATCATCGGCGTCGG
CGTGGTAGGAAACCTACTGATCTCCATCCTGCTGGTCAAAGACAAGAGCT
TGCACCGAGCGCCCTACTATTTCCTGCTGGACCTGTGCGCCTCCGACATC
CTGCGCTCGGCTATCTGCTTCCCGTTCGTCTTTACCTCGGTGAAAAATGG
ATCGGCGTGGACGTACGGCACGCTGACCTGCAAGGTGATCGCTTTTCTGG
GCGTGCTCTCCTGTTTCCACACCGCCTTCATGCTGTTCTGCGTCAGCGTC
ACCCGCTACCTGGCCATCGCGCATCACCGCTTCTACACCAAAAGGCTCAC
CTTCTGGACGTGCTTGGCTGTCATCTGCATGGTGTGGACGCTGTCGGTGG
CGATGGCATTCCCGCCGGTCCTGGACGTGGGGACGTACTCCTTCATCCGG
GAGGAGGACCAGTGCACGTTTCAGCACCGCTCCTTCCGGGCCAACGATTC
TTTGGGCTTCATGCTCTTGCTGGCGCTCATCCTCCTAGCCACACAGCTGG
TTTACCTCAAACTCATCTTCTTCGTCCACGACCGGCGAAAGATGAAGCCC
GTCCAGTTTGTGCCCGCCGTCAGCCAGAACTGGACCTTCCACGGTCCGGG
CGCTAGTGGGCAGGCAGCGGCCAACTGGCTGGCTGGATTCGGTCGGGGCC
CTACCCCGCCAACCTTGCTGGGCATTCGGCAGAACAGCAACGCAGCGGGC
CGCCGGCGTCTACTCGTACTGGATGAGTTCAAAACAGAGAAGAGGATTAG
TAGGATGTTCTACATCATGACCTTTTTTTTCTTGGCGCTTTGGGGGCCCT
ACTTGGTGGCCTGCTACTGGCGGGTGTTTGCCAGGGGGCCCGTGGTTCCC
GGGGGTTACCTAACAGCAGCCGTGTGGATGAGCTTTGCCCAGGCGGGGGT
CAACCCTTTCATCTGCATCTTCTCCAATAGGGAGGCCAAATCTCGCTTTC
ACCCTGGCGTGGGGACTGCTCCTGGCACGGAGC---GCAACGTTCCACTC
GGCAACAGCTTGCTGTCTCCTCAGCAAGCCGAGGAGCCCACTGTTGCCAC
CCCCCCGCAGCGATGGTTTGTCACCC---CTGCCAACAACCGACTGGACT
TTGCAGCCTCGGCATACGACGCCGCT---------GATTTCGCCGGTAAC
GCGGCCACCTTGCTGTCCTACGCAGCGGCCGGAGTGAAGGCTC------T
CCCCCTGCCGACTGCGAGCTGCTCCAACCGGCCTCTTGGCTATTACGCAG

ACCCGTCTG---GCTGG---GGAGGACGCACGCCGCCGCACTACTGCGGC
GTCAACAGCAAAGCCAGCTCGGTCTTTTCCTGCTGGCCCGCGAACACCAT
CGGGGGCCGCTCAGGCA---CC---AGCTACCTGC---------AGGAGG
A---GGGG---GACTC---CATCAGCACGGAGAGGTCACCG---AT---C
GGTGGCGCGGAGGAG---AGCAAAGCCAAAGACATCAC---ATCTGA---
GTCTAGCTGGATAGAG---ACGCCCTCCTCCATCAAATCCATCGATTCCA
GCGATTCTGGGATCTTTG---AACAGGCCAAACGGAGGAGAATCTCCCCC
ACTGCCACGCCG---------GTTTCAGAGACTGTGTCCCCGTTAAAGTC
TGAGCATCACTCAACAGGCGAAGTCACGGAGAGAGAAGTGGCGCTGGGGA
TAAACCCATTCGCGGATGGGATGGGCGCCTTCAAAATAAACCACAGCTCC
CACGACATCGGCTCCGG---ACAGACTGCGTTTTCCTCCCAGGCG---CC
CGGCTAC---GCGGCAGCCGCTTTGGGA---CACCATCA------CCATC
CGACCCACGTTGGCTCT---TACTCCACGGCGGCTTTCAACTCCACCAGG
GACTTTCTTTTCAGAAATCGGGGTTTCGGGGATGCCACCGG---------
---AGCGCAGCACAGCCTGTTTGCCTC---------CGGAAGTTT---C-
-----GCAGGGCCACATGGACACTCCGATGCAGCGGGGCACCTGCTCTTC
CCCGGGCTGCACGAG---CAAGCGGCGAGCCACGCGTCTTCCAACGTGGT
CAACAGCCAGATGCGGCTGGGCTTCTCGGGGGACATGTACGGGCGGGCTG
ATCAGTACGGCCACGTTACGAGCCCGAGAT---CCGACCACTATGCTTCG
ACTCAGCTGCACGGCTACGGCCCCATGAACATGAATATGGCCGCA---CA
CCACGGAGCAGGGGCCTTCTTCCGATACATGAGGCAGCCGATCAAACAAG
AGCTCATCTGTAAGTGGATCGAACCGGAGCAGCTGACAAACCCCAAAAAG
TCTTGCAACAAAACTTTCAGCACGATGCACGAGCTCGTCACCCATCTGAC
CGTGGAACATGTGGGGGGACCGGAGCAGACGAACCACGTTTGCTACTGGG
AGGACTGCTCCAGAGAAGGGAAGCCGTTCAAAGCCAAATACAAACTTGTA
AATCACATCCGAGTACACACCGGAGAAAAGCCCTTTCCGTGTCCGTTCCC
CGGCTGTGGCAAA
>Osmerus mordax
AGCCTCCTTATCCGAGCCGAGCTAAGCCAACCTGGTGCCCTCCTGGGGGA
CGACCAGATTTATAATGTTATCGTCACTGCGCACGCTTTTGTTATAATCT
TTTTTATAGTAATACCAATCATGATTGGAGGTTTCGGTAACTGGCTCATC
CCCCTTATGATTGGGGCCCCAGACATGGCCTTCCCCCGTATAAACAACAT
AAGTTTCTGACTTTTACCTCCCTCCTTTCTACTCCTCTTAGCTTCTTCTG
GAGTTGAAGCAGGGGCTGGGACCGGCTGAACAGTCTATCCCCCACTTGCT
GGTAATCTAGCCCATGCTGGAGCTTCGGTAGATCTAACAATTTTTTCTCT
TCACCTTGCGGGAATCTCCTCTATTCTAGGGGCAATCAATTTTATTACAA
CTATTATTAATATGAAGCCTCCAGCCATCTCCCAGTACCAGACCCCCTTA
TTCGTCTGAGCCGTTCTGATCACGGCCGTCCTTCTTCTCCTTTCCCTCCC
AGTACTAGCTGCTGGGATTACCATGCTTCTGACAGACCGAAACCTGAACA
CTACTTTCNNNNNNNNNNNNNNNNNNNNNNNNNNNNNNNNNNNNNNNNNNNNNNNNNNNNNNNNNNNNNNNNNN
NNNNNNNNNNNNNNNNNNNNNNNNNNNNNNNNNNNNNNNNNNNNNNNNNNNNNNNNNNNNNNNNNNNNNNNNNNNNN
NNNNNNNNNNNNNNNNNNNNNNNNNNNNNNNNNNNNNNNNNNNNNNNNNNNNNNNNNNNNNNNNNNNNNNNNNNNNN
NNNNNNNNNNNNNNNNNNNNNNNNNNNNNNNNNNNNNNNNNNNNNNNNNNNNNNNNNNNTTCCTGGAGAGGAACCTG
CACCCGTCCAACTGCCTGGGCATG
CTGCTGCTCTCCGACGCCCACCAGTGCACCAAGCTGTCGGAGCTGTCCTG
GGGCATGTGCCTGAGCAACTTCCCGGCCATTTGTAAGACGGAGGACTTCC
TCCAGCTCCCCAAGGACATGGTGGTCCAGCTGCTGTCCCACGAGGAGCTG
GAGACGGAGGACGAGAGGCTGGTGTACGAGGCGGCCCTCAACTGGGTGAA
CTACGACCTGGAGAGGCGCCACTGCGGCCTGCCGGAGCTGCTGCGCACGG
TCCGCCTGGCGCTGCTGCCCGCCATCTTCCTCATGGAGAACGTCTCCACG
GAGGAGCTGATCAACGCCCAGGTGAAGAGCAAGGAGCTGGTGGACGAGGC
CATCCGCTGCAAGCTGAAGATCCTGCAGAACGACGGCGTGGTCAACAGCC
CCTGCGCCCGCCCCCGCAAGACGAGCCACGCCCTCTTCCTGTTGGGCGGA

CAGACCTTCATGTGCGACAAGCTCTACCTGGTGGACCAGAAGGCCAAGGA
GATCATCCCCAAGGCGGACATCCCCAGCCCCAGGAAGGAGTTCAGTGCCT
GTGCCATCGGCTGCAAGGTCTACGTCACGGGGGGGC--GGGGCTC-GGAG
AACGGCGTGTCCAAAGATGTGTGGGTGTACGACACCGTGCAGGAGGAGTG
GTCCAAGGCGGCACCGATGCTCATCGCCCGGTTTGGCCACGGCTCAGCCG
AGCTGAAACACTGCCTCTACGTGGTGGGAGGGCATACCGCCGCCACGGGC
TGCCTGCCGGCCTCGCCCTCCGGACCAGTACATTGTGGTGTTCAGTCGTT
CCCAAACCAGGCTGATCCTCAACGAGGCCGAGCTGATCCTGGCGTTGGCG
CAGGAGTTCCAGATGAGGGTGGTGACGGTCTCCATGGAGGACCAGACCTA
CTCCAGCATCGTGCAGTTGATCAGCGGAGCCTCCATGCTCGTCAGCATGC
ACGGAGCTCAGCTCGTCACCTCGCTCTTCCTCCCCAGAGGGGCGGCTGTG
GTTGAGCTCTTCCCCTACGCGGTCAGCCCCGAGCAGTACACCCCTTACAA
GACCTTGGCCACCTTGCCAGGCATGGACCTCCAGTATGTCGCCTGGAGGA
ACACCATGGAGGAGAACTCCGTGGCCCACCCAGATAGACCCTGGGATCAG
GGGGGCATCGCTCACCTGGACAAGGATGAGCAGGAGCGCATCCTGGCCAG
CAGAGAGGTGCCCAGACACCTTTGCTGCCGAAACCCCGAGTGGCTGTTCC
GCATCTACCAGGACACCAGGTTGGACATCCCCTCTCTGCTGGACGTCCTC
AG---GGAGAGCCTGAAG---ACCAGGCCCAACCTCAAGAA---GACCCG
GCCTGCCAGCACTGTTCATCCAGGCAGGGTGAGGGAGCCTCAGTGCCAGA
CCTCAGTCCAGGCTACCAACGAAGCCAAGCTCACAGTATCCTGGCAGATC
CCTTGGAACCTCAAGTACTTGAAGGTNNNNNNNNNNNNNNNNNNNNNNNNNNNNNNAAGAGAGATCCCA
GCAAGGGAACC
TTGGAGGATCAAATCATCCAGGCTAACCCTGCCTTGGAGGCTTTTGGTAA
TGCCAAAACTTTGAGRAATGACAACTCGTCACGCTTCGGAAAATTCATCC
GGATTCACTTCGGAACCAGTGGCAAGCTGTCCTCAGCTGACATAGAGACT
TATCTTCTGGAAAAGTCACGTGTCACCTTCCAACTCAAGTCAGAAAGGAA
TTACCATATCTTCTTTCAGATCTTGTCCAATAAAAAGCCAGAGCTGTTGG
ACATGCTTCTGATTACCAACAACCCATATGACTACTCGTACATCTCCCAA
GGAGAGGTAACAGTAGCATCTATCAATGATGCTGATGAATTGATGGCCAC
TGACAGTGCCTTTGATATCCTTGGCTTTACTCAAGAGGAGAAAATGGGGG
TCTACAAGTTGACAGGAGCTATCATGCATTATGGCAACATGAAGTTCAAG
CAAAAGCAGCGTGAGGAGCAGGCAGAGCCTGACGGCACTGAGGCGGCTGA
CAAGTCAGCTTACCTAATGGGGCTGAACTCTGCAGATCTTGTGAAAGGAC
TCTGCCATCCCAGGGTAAAGGTTGGCAATGAGTATGTCACCAAAGGGCAG
GGTGTAGATCAAGTCTACTATCCAAACAAAGAGGCCTTCCGCTGCCAGGA
CTGTGGGAAGCACTACAACACCAAGCTGGGCTACAAACGCCATGTAGCCA
TGCACTCGGCCACAGCAGGGGACCTCACCTGCAAGGTGTGCCTGCAGAGC
TACGAGAGCACGCCGGCGCTGCTGGAGCACCTGAAGAGCCATTCGGGGAA
GTCGTCGGGCGGCGCCAAGGAGAAGAAGCACCCCTGCGACCACTGCGACC
GCCGCTTCTACACGCGCAAGGACGTCCGCCGCCACATGGTGGTGCACACG
GGCCGCAAGGACTTCCTGTGCCAGTACTGCGCCCAGCGCTTTGGCAGGAA
GGACCACCTCACGAGACACGTGAAGAAGAGCCACTCCCAGGAGCTGCTGA
AGATCAAGACGGAGCCGCCAGACCTGCTGGGGCTGCTGGGGTCTGGACC-
-----------CATCAAGGAGGAGCTCAGCCCCATGATGTGCAGCATGAG
CTCCACCAAGGACCCCCTCATGGCCAAGCCCTTCCCCGGTGGGACGGCCT
TCCCCATGGGCATGTACAACCCCCACCAC------CTCCAGCCCCTGCCC
AGCCCTGGAGGGGGGCAC---------CACCCCTCCCTGATGCCCAGCTC
CCTGTCTGCCGCTATGGGCATGGGCTGCCACATGGAC-------------

--GC
TTTGGTGTCTGCCCTGAAGGACCTGGAGGAGGACATCGTGGAGGGCTTGA
GGGAGCACGGCCTGGAAGACAGTTCCTGCACCTCCGGCTTCACCGTGACC
ATCAAGGAGTCCTGTGATGGCATGGGGGACGTCAGTGAGAAGCACGGAGG
GGGGCCGGCCATTCCTGAAAAGGCCGTCCGCTTCTCCTTCACCGTCATGT
CCGTGTCCGTCCTGGCCGAGGGGGAGGAG---------------------
---------GAAGCGGTCACCGTCTTCAGAGAGCAGAAGCCCAACTCGGA
GATGTCCTGYAAACCTCTCTGCCTGATGTTTGTGGACGAGTCGGACCACG
AGACCCTGACGGCCATCTTGGGGCCTGTGGTGGCCGAGAGGAACGCCATG
AAGAACAGCCGCCTCATCCTGGCCATGGGCGGCCTCCCTCGCTCCTTCCG
CTTCCACTTCAGGGGCACGGGCTATGATGAGAAGATGGTGCGTGAGATGG
AGGGCCTGGAGGCCTCAGGCTCCACCTACGTCTGCACCCTCTGCGACGCC
ACCAGAGCTGAGGCCTCCCAAAACATGGTGCTCCACTCAGTCACCCGCAG
CCACGACGAGAACCTGGACCGCTACGAGATGTGGAGGAGAAACCCCTACT
CTGAATCGGCGGACGAGCTGCGAGACCGGGTGAAAGGGGTCTCCGCCAAG
CCCTTCATGGAGACCCAGCCCACATTGGACGCGCTGCACTGTGACATCGG
CAACGCCACCGAGTTCTACAAGATCTTCCAGGACGAGATCGGGGAGGTGC
ATTGCAGGCC---CAAC---CCGAGCAGGGAGGAGCGTCGGAGCTGGAGG
GCGGCTCTGGACAAGCAGCTCAGGAAGAAGATGAAGCTGAAGCCCGTGAT
GAGGATGAACGGAAACTTTGCCCGGCGACTGATGACGGCGGAGGCGGTGG
AGGTGGTGTGCGAGCTGGTGCCGTCGCAGCAGCGGAGCGAAGCCCTCAGG
GAGCTGATGAACCTCTACCTCCAGATGAAGCCCGTCTGGCGCGCCACGTG
CCCAGCCAAAGAGTGCCCCGACCAGCTGTGTCGCTACAGCTTCAACTCCC
AGCGCTTTGCTGATCTCCTCTCTTCCACCTTCAAGTACAGGTACGACGGG
AAGATCACCAACTACCTCCACAAGACTCTGGCCCACGTTCCTGAAATCAT
AGAGAGAGATGGCTCCATCGGGGCCTGGGCCAGTGAGGGGAACGAGTCGG
GAAACAAATCATACACCATTGAGATGGGCCCAAAAGGGCCCCAGTGGAAA
GAGAGCCCTCAGCCTTTCTCCTGTTCTGTGGAGGACCCTACCAAACAGAC
CAAGTTCAAAGGCATCAAGACCTATATATCTTACCGGGTCACCCCCAGCC
ATACAGGGCGACCTGTCTACCGCCGGTACAAGCACTTCGATTGGCTATAC
AACCGTCTGCTGCATAAGTTCACCGTCATCTCTGTGCCTCACCTGCCGGA
AAAACAGGCCACAGGACGTTTTGAGGAGGACTTCATCGAGAAGCGCAAAA
GACGGTTGGTCATCTGGATGGACCACATGACCAGTCATCCTATCCTTTCA
CAGTACGAGGGCTTGGAACACTTCCTCATGTGTGCTGATGACAAGCAGTG
GAAGCTGGGAAAGCGGCGGGCGGAGAAAGATGAGATGGTGGGTGCCCACT
TCATGCTGACCTTTCAGATACCCAACGAGCACCAGGACCTGCAGGATGTG
GAGGAGCGAGTGGACACCTTCAAGTCCTTTGCTAGGAAGATGGATGAAAG
CGTCATGCAGCTGACCCACGTCGCCTCAGAACTGGTTCGAAAACACCTTG
GTGGCTTCAGGAAAGAGTTTCAGCGCCTGGGAAATGCGTTCCAGTCCATC
AGCCAGGCGTTCGTGCTTGACCCTCCTCACAGCTCGGATGCCCTCAACAA
TGCCATTTCTCACNNNNNNNNNNNNNNNNNNNNNCTGACCTCCTTGGGTTTCATCATCGGCGTTGGAGTG
G

TCGGCAATCTTCTGATCTCCATCCTACTGGTCAAAGACAAGAGCCTGCAC
CGAGCGCCCTACTACTTCTTGCTGGACCTGTGCGCCTCCGACATCCTGCG
TTCCGCCATCTGCTTCCCCTTCGTGTTCACCTCCGTCAAGAATGGTTCCG
CCTGGACGTACGGAACCCTAACCTGNNNGNNGATCGCCTTCCTGGGGGTCCTGTC
CTGCTTTCACACGGCCTTCATGTTGTTCTGCGTCAGCGTGACCCGCTACC
TGGCCATCGCCCACCATCGCTTCTACACCAAGAGGCTGACCTTCTGGACG
TGCTTGGCCGTCATCTGCATGGTGTGGACGCTGTCAGTGGCCATGGCCTT
TCCCCCAGTACTGGATGTAGGGACGTACTCCTTCATCAGAGAGGAAGACC
AGTGCACCTTCCAGCACCGCTCCTTCAGAGCCAACGACTCCCTGGGCTTC
ATGCTGCTGCTCGCCCTCATCCTGCTGGCCACGCAGCTGGTCTACCTCAA
GCTCNTCTTTTTCGTCCACGACCGCCGGAAGATGAAGCCGGTCCAGTTCGT
GCCGGCGGTCAGCCAGAACTGGACCTTCCACGGGCCGGGGGCCAGCGGCC
AGGCGGCGGCTAACTGGCTGGCGGGCTTCGGGAGAGGCCCCACCCCGCCC
ATCCTTCTGGGCATCAGGCAGAACAGCAACGCGGCGGGCCGCAGGCGACT
CCTGGTGCTGGACGAGTTCAAGACGGAGAAGAGGATCAGCCGGATGTTTT
ACATCATGACCTTCTTCTTCCTGGCACTGTGGGGGCCCTATCTGGTGGCC
TGCTATTGGANAGTGTTCNNNNNNNNNNNNNNNNNNNNNNNNNNNNNNNNNNNNNNNNNNNNNNNNNNNNN
NNNNNNNNNNNNNNNNNNNNNNNNNNNNNNNNNNNNNNNNNNNNNNNNNNNNGCCAAATCTCGCTTTCA
CCCTGGCGTAGGGACT
GGTGCTGGCACGGACC---GCAGCGTCCCACTTAGTAACAGCTTGCTATC
CCCGCAACAAACCGAAGAGCCCACAGTTG---CTTCCCCACAGCGTTGGT
TTGTCACCC---CTGCCAACAACCGACTGGACTTTGCCGCCTCGGCATAC
GATGCTGCCGCTGCTGCAGATTTTGCCGGCAACGCGGCCACCTTGCTGTC
CTACGCAGCGGCTGGAGTGAAGGCGC------TTCCCCTCTCCACGGCAG
GTTGCTCCAACAGACCGCTCGGGTATTACGCCGACCCGTCAG---GCTGG
---GGTGCCCGTACGCCACCACAGTACTGT---------AGCAAGTCGAG
CTCCGTTCTCTCATGCTGGCCCACGAATACTGTTACCGGCAGAACGGGCC
---CGTCCAGTTACCTGG---------CCGAGGA---GGGA---GACGC-
--AATTCCCACAGAGAGGTCTCCA---AT---AGGGGCGTCAGACGAG--
-GCAAAACCAAAAGACTT------GTCCGA---ATCCAGCTGGATAGAA-
--ACGCCGTCTTCAATAAAGTCAATTGATTCCAGTGATTCTGGAATCTTT
G---AGCAAGCAAAACGGAGAAGAATTTCTCCGTCTGCCACACCA-----
----GTTTCGGAGACAGTGTCCCCGTTAAAATCCGAA---------ACAG
GCGAAGTCACAGAAAGAGAAGTGGCTTTGGGGATAAATCCGTTCGCAGAC
GGGATGGGTGCTTTCAAAATCAACCACAGTTCGCATGATCTTGGCTCCGG
---GCAAACTGCATTTTCCTCCCAAGCG---CCCGGCTAC---GCAGCCG
CTGCACTGGGA---CATCACCA------CCACCCGACTCATGTCAGTTCT
---TACTCTACGGCGGCTTTCAATTCCACCCGGGATTTTCTCTTCAGAAA
TCGAGGCTTCGGAGACGCTACCAG------------CGCTCAGCATAGTC
TCTTCGCCTCCGC---AGCGGGAAGTTT---T------GCAGCCCCACAT
GGACACTCAGATGCAGCGGGACACCTGCTCTTCCCAGGACTTCACGAA--
-CAAGCCGCGAGCCATGCTTCCTCAAATGTTGTTAATAGTCAGATGCGAT
TGGGCTTTTCGGGGGACATGTACGGCAGGGCCGACCAGTATGGCCATGTT
ACAAGCCCGCGGT---CCGACCACTATGCTTCGACCCAGTTGCATGGCTA
TGGCCCTATGAACATGAATATGGCCGCG---CATCATGGAGCAGGGGCCT
TCTTCCGTTACATGAGGCAGCCGATAAAACAAGAGCTGATCTGCAAGTGG
ATCGAACCAGAGCAACTAACGAACCCGAAAAAGTCGTGCAACAAAACTTT
TAGCACAATGCACGAGCTCGTCACCCATCTGACGGTGGAGCATGTGGGAG
GACCGGAGCAGTCGAACCACATTTGCTTCTGGGAAGAGTGTGCCCGAGAA
GGAAAACCATTCAAAGCCAAATACAAACTTGTGAACCACATCAGAGTGCA
CACCGGAGAGAAACCATTTCCATGTCCATTTCCCGGTTGTGGCAAA
>Ostracoberyx dorygenys

------------NNNNNNNNNNGAAACCTTCACCCATCTAACTGCCTTGGCATGCTGTTG
CTGTCTGACGCCCACCAGTGCACCAAGCTGTCAGAGCTCTCCTGGGGCAT
GTGCCTCAGCAACTTTCCTGCTATTTGCAAGACAGAGGACTTCCTCCAAC
TGCCCAAAGATATGGTGGTGCAGCTTTTGTCACATGAGGAGCTAGAGACA
GAAGATGAGAGACTGGTTTACGAAGCTGCCCTTAACTGGATCAACTATGA
CCTGGAAAAGAGGCACTGCAACCTTCCAGAGCTCCTGAGAACGGTCCGTC
TGGCCCTGCTGCCTGCAATCTTTCTCATGGAGAACGTTTCTACAGAAGAG
CTGATCAACGCCCAGGCCAAAAGCAAGGAGCTGGTGGATGAAGCTATCCG
CTGTAAGCTGAAGATCCTGCAGAATGATGGCGTCGTTAACAGCCCATGTG
CTCGACCAAGAAAAACCAGCCATGCCCTCTTTCTTCTGGGAGGGCAGACT
TTCATGTGTGACAAGTTGTACCTGGTGGACCAGAAAGCCAAAGAGATCAT
CCCCAAAGCTGACATTCCCAGCCCCAGGAAGGAGTTCAGCGCCTGCGCCA
TCGGCTGTAAGGTGTACATCACTGGTGGGA--GAGGCTC-AGAGAATGGT
GTGTCCAAAGATGTGTGGGTCTACGACACCATCCACGAGGAATGGTCGAA
GGCGGCACCCATGCTCATTGCCAGGTTTGGCCATGGCTCTGCGGAGCTGA
AACACTGCCTCTACGTGGTAGGAGNNNNNNNNNNNNNNNNNNNNNNNNNNNNNNNNNNNNNNNNNGGATG
AATACATTGTTGTGTTCAGTC
GTTCAACAACGAGGCTGATACTGAATGAAGCGGAGCTAATCATGGTGCTG
GCCCAGGAGTTCCAGATGAGAGTGGTCACGGTATCCCTGGAGGAACAGTC
TTTCCCCAGTATCGTCCAGGTGATCAGCGGTGCTTCCATGTTAGTCAGTA
TGCATGGAGCTCAGCTTATCACCTCACTCTTCCTCCCCAGAGGAGCTGCT
GTGGTGGAGCTGTTCCCCTTTGCTGTGAACCCAGAGCAGTACACCCCGTA
TAAAACCCTTGCCTCCCTTCCAGGCATGGACCTTCACTATGTCTCCTGGA
GGAACACTAAGGAGGAGAACACCATCACCCACCCAGACAGACCCTGGGAA
CAAGGGGGCATTGCTCACTTGGAGAAGGAGGAGCAAGAGCGAATATTGGC
GAGCAAAGATGTTCCCAGGCACCTGTGCTGCCGCAACCCAGAGTGGCTCT
TCCGGATCTACCAGGACACTTTGGTGGACATCCCTTCCTTCCTGGAAGTT
CTCAA---AGAGGGCATGAAG---ACAAAGCCCAGCTTGAAGAA---GTC
AAAGCCGGCCAGCACAGTCCACCCAGGCCGGGTCAGAGAACCCCAGTGTC
AGACCTCAGTACAAACCACTAATGAGGCTAAACTCACAGTCTCCTGGCAG
ATCCCATGGAATCTGAAATACCTGAAGGTGAGAGAGGTCAAATACGAAGT
G---------AAAAAAGACACCAGTAAGGGGACACTGGAGGATCAAATCA
TCCAGGCGAACCCTGCGCTGGAGGCCTTCGGCAATGCCAAAACATTGAGA
AACGACAACTCGTCTCGTTTTGGAAAATTCATCCGAATTCACTTCGGTAC
GAGCGGCAAGCTGTCGTCTGCTGACATCGAGACGTACCTGCTGGAGAAGT
CACGTGTCACCTTTCAGCTCAAGGCTGAGAGGAACTACCACATCTTCTAC
CAGATCCTGTCCAATCAGAAGCCAGAGCTCCTGGACATGCTGCTGATCAC
CAATAACCCGTACGACTACTCCTACATCTCCCAAGGAGAGGTAACGGTTG

CTTCCATCAATGACTCAGAGGAGCTGATGGCCACCGACAGCGCCTTCGAT
GTGCTCGGCTTCACTCCAGACGAGAAGATGGGCGTCTATAAACTGACTGG
TGCCATCATGCACTACGGCAACATGAAGTTCAAACAGAAGCAGCGTGAGG
AGCAGGCAGAGCCGGACGGGACGGAGGCTGCTGATAAAACAGCTTACCTA
ATGGGGCTGAACTCTGCTGACCTCATCAAAGGGCTGTGCCATCCCAGAGT
CAAGGTAGGAAATGAATACGTCACCAAAGGCCAAAGTGTGGATCAAGTCT
ACTACNNNNNNNNNNNNNNNNNNNNNNNNNNNNNNNNNNGGAAGCACTACAACACCAAGCTGGGATATAA
GCGCCATGTGGCCA
TGCACTCTGCCACAGCAGGGGATCTCACCTGTAAAGTGTGCATGCAGACC
TACGAGAGCACACCCGTGCTCTTGGAGCACCTCAAGAGCCACTCCGGGAA
GTCTTCGGGTGGCACCAAGGAGAAAAAACACCCATGCGATCACTGTGACC
GTCGTTTCTACACACGGAAGGATGTGAGACGGCACATGGTGGTCCACACA
GGCCGAAAGGACTTCCTGTGCCAGTACTGTGCCCAGCGCTTCGGCAGGAA
GGACCATCTGACGCGCCACGTGAAGAAGAGCCACTCGCAGGAGCTGCTGA
AGATCAAGACGGAGCCTCCTGATATGTTAGGTCTTTTAGCATCGGGGTCA
CCACCCTGCTCTGTGAAGGAGGAGCTCAGCCCCATGATGTGCGGCATGGG
TCCCAACAAAGACCCCATGATGGGCAAACCGTTCCCCAGTGGGGCCCCTT
TCCCGATGGGCATGTACAACCCCCACCAT------CTCCAGGCCATGTCT
AATTCTGGGGTGGGTCACCCA------CACCCGTCCCTGATGCCCAGTTC
TTTGTCTGCAGCTATGGGCATGGGCTGTNNNNNNNNNNNNNNNNNNNNNNNNNNNNNNNNNNNNNNNNNNNN
NNNNNNNNNNNNNNNNNNNNNNNNNNNNNNGTGAACCTGCTGGCTAGTAACT
CTCCGAGTGTTTCGTATGCTCTGACCCAGCAGAAATACTTCAGTAACTAC
AGTCCCGTAATTGGGTTTTACATTTACGAGCCCATAGAGTACTGGAACTC
AACGGTGCAGGAGCACCTGAAGACTCTGAGTCATGGCTTCAACAAGATCT
CCTGGATGGACAACTTTTTCCACTACCTGCGGGTGGTGAATGTGAGTGCG
TCAACCAAGAGTGACTTCATCACCATCCTCAAGGGCTCCTTCCTGCGCAG
CCCGGAGTACCAGCACTTCACTGAGGACATCATATTCTCCAAGA---ACC
GCGAGACTG------ATGAGTACGACATTATCGCCTCACGGATGTACTTG
GTGGCACGGACGACAGAGAAGAAGCGTGAAGAGGTGGTGGAGCTTCTGGA
GAAGCTTCGTCCGTTGATGCTGATCAACAGCATCAAGTTCATTGCCTTCA
ATCCTACGTTTGTGTTCATGGACCGCTACAGCTCCTCTGTCATCTCGCCC
ATCTTGACCTCAGGCTTCAGCGTACTCACAATCCTCATCCTCACTTTCTT
CCTGGTCATCAACCCCTTGGAAAACTTCTGNNNNNNNNNNNNNNNNNNNNNNNNNNNNNNNNNNNNNNNN
NNNNNNNNNNNNNNNNNNNNNNNNNNNNNNNNNNNNNNNNNNNNNNNNNNNNNNNNNTCTTGCAATGTTG
GCATTAT
TAATGGGCTCTCTGGATGGACTTCCTCGGTGGATGACTCCCCATCTGACA
CCATCACTCGGCGGTTTCGCTATGATGTGGCATTGGTGTCAGCAATAAAG
GATCTGGAGGAGGACATCATGGAGGGGCTGAGAGAGAGTGGGATGGAAGA
CAGTGCATGCACCTCAGGCTTCAGTGTCATGATCAAGGAATCTTGTGATG
GCATGGGCGATGTCAGCGAGAAGCACGGTGGAGGACCAGCTGTTCCTGAG
AAGGCTGTACGTTTCTCTTTCACTGTTATGTCTGTCTCTGTCCTGGCAGA
CGATGAGGAG------------------------------GAGGAGGTTA
CCATCTTCACCGAGCCAAAGCCAAACTCAGAACTGTCCTGTAAGCCCCTT
TTCCTGACGTTTGTGGATGAGTCAGACCATGAGACACTCACAGCCATCCT
GGGGCCTATTGTTGCAGAGCGTAATGCAATGAAAGAGAGCAGGCTCATCC
TATCCATGGGTGGACTACCTTGCTCCTTCCGCTTTCACTTCAGAGGCTCG
GGATACGATGAGAAGATGGTGCGTGAGATGGAGGGCCTCGAGGCCTCAGG
GTCCTCCTATATCTGCACTCTTTGTGACTCCAGTCGGGCAGAAGCCTCTC
AAAACATGGTGCTACACTCCGTCACCCGCAGTCATGAAGAGAACCTAGAA
CGTTATGAAATATGGAGAACCAACCCCTTTTCTGAGTCTGTAGATGAGCT
GCGAGACAGAGTCAAAGGGGTCTCTGCCAAGCCCTTCATGGAGACCCATC
CCACGCTGGATGCATTACACTGTGACATTGGCAATGCCACTGAGTTCTAC
AAAATCTTCCAGGACGAGATCGGGGAGGTGTACCAAAAGGT---CAAC--

-CCCAGCCGGGAGGAACGGCGCAGCTGGAGGGCAGCCCTAGATAAACAGC
TGAGGAATAAGGTAAAGCTTAAACCGGTAATGAGGATGAATGGGAACTAT
GCCCGCAGGCTAATGACCCAGGAGGCTGTGGAGGTGGTGTGTGAGCTGGT
GCCCTCAGAAGGGAGGAGGGAGGCCCTGAGGGAGCTTATGAGGCTCTACC
TCCAGATGAAGCCTGTGTGGCGCGCCACCTGCCCAGCCAAGGAGTGCCCC
GACCAGCTGTGCCGCTACAGCTTTAACTCCCAGCGCTTTGCCGACCTCCT
CTCCTCTACCTTCAAATATAGATACAATGGAAAGATAACCAATTACCTGC
ACAAGACCCTGGCCCANNNNNNNNNNNNNNNNNNNNNNNNNNNNNNNNNNNNNNNNNNNNNNNNNNNNNN
NNNNNNNNNNNNNNNNNNNTCGTACACCATCGAGATGTGCCCCTTGGGGCCCC
GGTGGAAGGAGAGCCCACAGCCTTTCTCCTGCTCCATTGAAGACCCCACA
AAACAGACAAAGTTCAAGGGCATCAAGACGTACATTTCGTACCGGGTCAC
GCCGAGCCACACAGGGCATCCCGTCTACAGGCGCTACAAACACTTTGACT
GGCTGTACAACCGCTTACTGCACAAGTTCACTGTGATCTCTGTGCCTCAC
CTGCCTGAGAAGCAGGCCACGGGGCGATTTGAGGAAGACTTCATTGAGAA
GCGTAAGAGGCGACTGATACTGTGGATGAACCACATGACCAGTCACCCAG
TCCTCTCCCAGTATGAAGGCTTTGAGCACTTTCTGATGTGTGCTGATGAC
AAGCAGTGGAAACTGGGCAAGAGACGGGCGGAGAAGGACGAGATGGTGGG
TGCCCATTTCATGCTGACCCTCCAGATCCCTAACGAGAACCAGGACCTTC
AGGATGTAGAGGAGCGGGTCGACTCCTTCAAGGCCTTTGCTAAGAAAATG
GATGACAGCGTGATGCAGCTCACACATGTTGCCTCAGAGCTGGTGCGTAA
GCACCTGGGTGGGTTCAGGAAGGAGTTCCAGCGGCTGGGAAATGCCTTCC
AGTCTATCAGCCAGGCGTTCATGCTGGACCCTCCCCACAGCTCAGACAAC
TTCAACAACGCCATCTCCCAT-----------------------------

--------NNNNNNNNNNNNNNNNNNNNNNNNNNNNNNNNNNNNNNNNNNNNNNAGC---
GCAGCGTCCCACTCGGCAACAGCTTGCTATCCCCGC
AGCAAACCGAGGAGCCCACTGTTGCCACCCCCCCGCAGCGATGGTTTGTC
ACCC---CTGCCAACAACCGACTGGACTTTGCTGCCTCGGCATACGACGC
CGCT---------GATTTCGCCGGTAACGCGGCCACCTTGCTGTCCTACG
CAGCGGCCGGAGTGAAGGCTC------TTCCCCTGCCGACTGCAGGCTGC
TCCAACCGGCCTCTTGGCTATTACGCAGACCCGTCAG---GCTGG---GG
AGGACGCACGCCGCCGCAGTACTGTGGTGTAAATAGCAAATCCAGCTCGG
TCTTTTCCTGCTGGCCCGCTAACTCTATCGGTGGCAGAGCAGGCA---CC
---AACTACCTGG---------TGGAGGA---GGGA---GACTC---CAT
CCCGACGGAGAGGTCACCG---AT---CGGCGGCTCGGAGGAG---ACCA

```
AACCCAAAGACCTGAC---ATCAGA---GTCGAGCTGGATAGAG---ACG
CCGTCCTCCATTAAGTCCATTGATTCAAGCGATTCTGGTATCTTTG---A
ACAGGCCAAAAGGAGAAGAATCTCACCTTCTGCCACGCCG---------G
TTTCAGAGACAGTGTCCCCNNNNNNNNNNNNNNNNNNNNNNNNNNNNNNNNNNNNNNNNNNNNNNNNNNN
NNNNNNNNNNNNNNNNGTTCGCGGATGGGAGGGCGCCTTCAAAATA
AACCACAGCTCCCACGATATTGGCTCCGG---ACAAACGGCGTTTTCCTC
CCAGGCG---CCCGGCTAC---GCAGCAGCCGCCCTGGGA---CACCATC
A------CCACCCGACCCACGTTGGCTCT---TACTCCACGGCGGCGTTC
AACTCCACCAGGGACTTTCTCTTCAGAAATCGGGGTTTCGGGGACGCCAC
CGG------------GGCTCAGCACAGTTTGTTCGCCTC---------CG
GAAGTTT---C------GCAGGGCCACATGGACACTCAGATGCAGCGGGG
CACCTGCTCTTCCCAGGGCTCCACGAG---CAAGCGGCGAGCCACGCGTC
TTCCAATGTGGTCAACAGCCAGATGCGGCTGGGCTTCTCGGGGGACATGT
ACGGACGGGCCGACCAGTACGGCCACGTTACAAGTCCGCGGT---CCGAC
CACTATGCTTCGACCCAGCTGCACGGCTATGGCCCCATGAACATGAATAT
GGCCGCA---CACCACGGAGCAGGGGCCTTCTTTCGATACATGAGGCAGC
CGATCAAACAAGAGCTCATCTGCAAGTGGATCGAGCCGGAGCAGCTGACG
AATCCCAAAAAGTCGTGCAACAAAACTTTTAGCACGATGCACGAGCTTGT
GACCCATCTGACGGTGGAGCATGTGGGGGGACCGGAGCAGACGAACCACA
TCTGCTTCTGGGAGGANNNNNNNNNNNNNNNNNNNNNNNNNNNNNNNNNNNNNNNNNNNNNNNNNNNNNN
NNNNNNNNNNNNNNNNNNNNNNNNNNNNNNNNNNNNNNNNNNNNNNNNNNNNNNN
>Pachypanchax playfairii
--------------------------------------------------
--------------------------------------------------
--------------------------------------------------
--------------------------------------------------
--------------------------------------------------
--------------------------------------------------
--------------------------------------------------
--------------------------------------------------
--------------------------------------------------
--------------------------------------------------
--------------------------------------------------
--------------------------------------------------
--------------------------------------------------
--------------------------------------------------
--------------------------------------------------
--------------------------------------------------
------------NNNNNNNNNNNNAACCTTCACCCGTCCAACTGTCTTGGCATGCTGCTGCT
GTCTGACGCCCACCAGTGTACAAAGTTATCAGAGCTCTCTTGGGGCATGT
GTCTMAGCAATTTTCCTGCCATTTGCAAGACAGAGGACTTTCTCCAACTG
CCCAAAGATATGGTAGTGCAGCTGTTGTCACACGAGGAGCTTGAGACAGA
AGATGAGAGATTGGTTTATGAAGCTGCCCTTAACTGGATTAACTATGACC
TAGAAAGGAGGCATTGCCATCTCCCAGAGCTCCTGAGRACAGTTCGTCTC
GCCCTGCTGCCTGCCATCTTTCTAATGGAGAATGTTTCAACAGAAGAGCT
GATAAATGCCCAAGAAAAGAGCAAGGAGCTGGTGGATGAAGCTATTCGCT
GTAAGTTGAAGATCCTCCAGAATGACGGCGTCGTCAACAGTCCATGTGCT
CGGCCGAGAAAAACCAGCCATGCCCTTTTCCTTCTTGGAGGTCAGACCTT
CATGTGTGATAAGTTATACTTGGTGGACCAAAAGGCCAAAGAGATAATCC
CAAAAGCTGACATTCCCAGTCCCAGGAAGGAGTTTAGTGCCTGCGCCATT
GGCTGCAAGGTGTACATTACTGGTGGGA--GGGGATC-AGAGAACGGTGT
GTCCAAAGATGTGTGGGTCTACGACACTGTGCATGAAGAATGGTCGAAGG
CTGCTCCCATGCTCATTGCCCGGTTTGGTCATGGCTCTGCAGAACTGAAA
```

CACTGTCTGTATGTTGTTGGAGGACACACTGCAGCAACCGGCTGCCTCCC
AGCNNNNNNNNNNAGATGAATACATTGTAGTTTTCAGCCGTTCAACGACAAGGCTAATAC
TGAATGAAGCCGAGCTAATCATGGCACTGGCTCAAGAGTTCCAGAAGAGA
GTAATCACAGTATCTTTGGAGGAACAGTCTTTTTTTAATATCATCCAGGT
GATCCGTGGTGCTTCCATGTTAGTCAGCATGCATGGAGCTCAGCTTATCA
CCTCACTCTTCCTCCCCAGAGGTGCTGTTGTTGTAGAGCTGTTTCCCTTT
GCTGTGAATCCTGAGCAGTACACGCCATACAAAACCCTTGCCTCCCTTCC
AGGCATGGATCTTCACTATGTCTCTTGGAGGAACATGAAAGAAGAGAATA
CTGTAACCCATCCAAATAGACCCTGGGAACAAGGCGGAATATCCCATTTG
GAAAAAGACGAGCAGGAGCGAATCCTGGCGAGTAAGGACGTCCCAAGGCA
CTTGTGTTGCCGTAACCCAGAGTGGCTCTTCCGAATTTACCAGGACACTT
TGGTAGACATCCCATCCTTTCTCAGTGTGCTCAG---AGATGCAATGAAG
---GCCAAACCCAACTTGAAGAA---GGCCAAGATAGCGAGCACAGTCCA
TCCTGGCCGGGTCAGAGAAGCCCGTTGTCAGACTTCAGTTCAAACGTCTA
ATGAAGCAAAGCTCACAGTCTCTTGGCAGATCCCATGGAATCTGAAATAC
CTGAAAGTAAGAGAANNNNNNNNNNNNNNNNNNNNNNNN-------------------------------
----

NNNNNNNNNNNNNNNNNNNNNNNNNNNNNNNNNTGTTTACAGATCAGTGATGGGTCA
AATATAGTGAACCTGTTGGCTAGTAACTCACCAAGTGTTTCTTATGCTTT
GACCCAACAGAAGTACTTCAGTAATTATAGTCCTGTCATTGGGTTTTATA
TTTACGAACCCATTGAGTATTGGAACTCCACTGTGCAGGAACACCTGAAG
ACACTAAGTCATGGCTTCAACAAGATCTCCTGGATGGACAACTTCTTCTA
CTACCTGCGGATGGTGAATGTGAGCGCATCAACTAAGAGCGACTTCATCA
ATATTTTAAAAGGTTCTTTCCTGCGCAGCCCAGAGTACCAGCACTTCACA
GAGGACATCATCTTCTCCAAAA---ACCGTGAGACAG------ACGAATA
TGACATCATTGCCTCACGGATGTATTTAGTAGCACGGACCACAGAGAAGA
AGCGGGAAGAGGTGGTGGAGCTTTTGGAAAAGCTTCGTCCATTGATGCTG

ATTAACAGCATTAAATTCATTGCCTTCAATCCAACGTTTGTTTTTATGGA
CCGTTACAGCTCCTCTGTCATCTCACCCACCCTGACCTCAGGTTTCAGTG
TACTCACTATTCTCATTCTAACTTTCTTCCTGGTCATCAACCCCTTGGANNNNNNNNNNNNNNNNNNNNN
NNNNNNNNNNNNNNNNNNNNNNNNNNNNNNNNNNNNNNNNNNNNNNNNNNNNNNNNNNNNNNNNNNNNNN
NNNNNNNNNT
CTGCATCTTACAGTGTTGGCATTATTAATGGTCTTTCTGGATGGGCTTCC
TCGGTGGATGACTCCCCAGCTGACACCATATCTCGACGCTTCCGCTATGA
TGTGGCACTGGCTGCAGCACTGAAAGATCTGGAGGAGGACATCATGGAGG
GGCTTAGAGAGACTGGGATGGAAGACAGCGCTTGCACCCTGGGCTTTAGT
GTCATGATCAAGGAATGCTGTGACGGCATGGGTGATGTCAGCGAAAAACA
TGGAGGAGGACCAGCTATTCCTGAAAAGGCTGTTCGCTTCTCCTTCACTG
TTATGTCTGTCTCTGTCCAGTCTGAAGACGATGGT---------------
---------------CAAGATGTCACCATATTCACTGAGCCGAAACCAAA
CTCAGAACTGTCCTGTAAGCCCCTTTGCCTTATGTTTGTGGATGAGTCAG
ATCATGAGATGCTCACGGCTGTCCTGGGGCCCATAGTTGCAGAGCGTAAT
GCCATGAAGGAAAGCAGGCTCATTCTGTCCATGGGCGGCATGCCACGCTC
CTTTCGCTTCCACTTCAGAGGCACGGGGTACGATGAAAAAATGGTGCGAG
AGATGGAGGGATTGGAGGCTTCGGGGTCCACATATATCTGCACTTTATGT
GATTCCAGTCGCGCAGAAGCTGCTCAAAACATGGTGTTACACTCCGTCAC
CCGCTGCCACGAGGAGAACTTGGAACGTTATGAAATATGGAGAACCAACC
CTTATTCAGAGTCAGCAGATGAGCTGAGAGACAGAGTCAAAGGAGTCTCT
GCAAAGCCCTTCATGGAAACCCAGCCCACTCTTGATGCATTACACTGTGA
TATTGGTAATGCCACAGAATTTTACAAAATCTTCCAAGATGAGATCGGAG
AAGTGTATAAAAAGGT---CAAC---CCCAGCCGGGAGGAACGACGCAGC
TGGAGGGCAGCTTTAGATAAACAGCTGAGGAAGAAGATGAAGCTTAAACC
AGTAATGAGAATGAATGGAAACTACGCCCGCAGGTTAATGACCATGGAGG
CTATGGATGTGGTGTGTGAGCTGGTGCCCTCAGAGGAAAGGAGAGAAGCC
CTGAGGGAGCTTATGAGGCTCTACCTCCAGATGAAGCCTGTGTGGCGCGC
CACCTGCCCAGCTAAGGAATGCCCCGACCAGCTGTGCCGCTACAGCTTTA
ACTCCCAGCGCTTTGCTGACCTGCTCTCCTCCACTTTCAAATATAGATAC
AATGGTAAGATAACTAATTACCTGCACAAGACCCTGGCTCATGTGCCTGA
AATCATAGAAAGAGATGGATCCATCGGCGCCTGGGCCAGCGAAGGAAACG
AGTCNNNNNNNNNNNTCATATTCCATTGAGATGGGTCCCTTGGGGCCACGGTGGAAGGACA
ACCCACAGCCTTTCACTTGCTCCATTGAAGACCCTACCAAGCAGACCAAG
TTCAAGGGTATCAAGACCTACATATCATACCGGGTCACACCGAGTCACAC
TGGGCTTCCTGTTTACAGACGTTACAAACACTTTGACTGGCTTTACAACC
GTTTACTGCACAAGTTCACTGTGATTTCCGTTCCTCACCTCCCAGAGAAG
CAGGCCACAGGCCGATTTGAGGAAGACTTCATCGAGAAGCGTAAAAGACG
ATTAATACTGTGGATGAACCACATGACAAGTCACCCAGTCCTCTCCCAGT
ATGAAGGTTTTGAGCATTTTCTGATGTGTACTGATGATAAGCAGTGGAAA
CTGGGTAAGAGGCGGGCAGAGAAGGATGAGATGGTAGGGGCCCATTTCAT
GCTGACCCTCCAAATCCCCACTGAGCACCAGGACCTTCAGGATGTTGAGG
AAAGGGTTGACAATTTCAAGACTTTTGCCAAGAAAATGGATGACAGTGTG
ATGCAGCTCACGCACGTTGCTTCAGAGCTGGTTCGCAAACACCTAGGTGG
ATTCAGGAAGGAATTCCAACGTCTAGGAAATGCCTTCCAGTCCATCAGCC
AAGCATTCATGCTGGATCCACCCTACAAGTCAGATACACTCAACAACGCC
ATNNNNNNNNNNNNNNNNNNNNNNNNNNAACTGACCTCTCTGGGTTTTATCATTGGAGTTGGTGTGGTAG
GGAACC
TCCTGATCTCCATCCTGTTGGTCAAAGACAAGAGTCTGCACCGGGCACCC
TACTATTTTCTGCTGGACCTGTGCGCCTCTGATATCCTGCGCTCAGCCAT
CTGTTTCCCCTTTGTCTTCACCTCAGTAAAGAATGGATCTGCCTGGACTT
ATGGCACGCTAACCTGCAAAGTGATTGCCTTCCTGGGTGTGCTGTCTTGT
TTCCACACGGCATTCATGCTATTCTGCGTCAGTGTCACCCGCTACCTGGC

CATAGCACACCACCGCTTCTACACCAAGAGGCTGACTTTCTGGACGTGCT
TAGCTGTCATCTGCATGGTGTGGACATTGTCAGTGGCTATGGCGTTTCCT
CCGGTGCTAGATGTTGGAACGTACTCCTTTATCCGGGAGGAGGATCAGTG
TACATTTCAGCACCGGTCCTTCAGGGCGAATGATTCACTGGGCTTCATGC
TCCTGCTGGCTCTCATCCTCCTGGCCACGCAGCTGGTTTACCTCAAACTC
ATTTTCTTTGTCCATGACCGCCGAAAGATGAAGCCTGTTCAGTTTGTGCC
TGCTGTCAGCCAGAACTGGACCTTCCACGGGCCAGGCGCCAGTGGGCAGG
CTGCGGCCAACTGGCTGGCTGGATTTGGTCGTGGCCCCACCCCGCCTACC
TTGCTGGGTATCCGACAGAACAGCAATGCTGCAGGCCGCAGGCGTCTACT
AGTACTGGACGAATTCAAAACAGAGAAGAGGATTAGCAGGATGTTCTACA
TAATGACGTTTTTCTTCCTGGCATTATGGGGGCCCTACCTGGTCGCCTGC
TACTGGCGGGTGTTTGCAAGGGGCCCCGTGGTCCCTGGAGGCTACCTGAC
AGCAGCCGTGTGGATGAGCTTTGCCCAGGCTGGGGTCAATCCCTTCATCT
GNNNNNNNNNNNNNNNNNNNGCCAAATCTCGCTTTCACCCTGGCGTGGGGACTGGTCCTGGCACGGAGC
---GCAGCGTCCCACTCGGCAACAGCTTGCTATCCCCGCAGCAAACCGAG
GAGCCCACTGTTGCCACCCCCCCGCAGCGATGGTTTGTCACCC---CTGC
CAACAACCGACTGGACTTTGCAGCCTCGGCATACGACGCCGCC-------
--GATTTTGCCGGTAACGCGGCCACCTTGCTGTCCTACGCAGCGGCCGGA
GTGAAGGCTC------TCCCCCTGCCGACAGCGGGTTGCTCCAACCGGCC
TCTTGGCTATTACGCAGACCCGTCTG---GCTGG---GGAGGACGCACGC
CGCCTCAGTACTGTGGCGTAAACAGTAAATCCAGCTCGGTCTTTTCCTGC
TGGCCCGCTAACTCCCTCAGCGGCAGGGCGGCCA---CC---AACTACCT
GT---------CGGAGGA---GGGA---GACTC---CATCGCGACGGAGA
GGTCACCG---AT---CGGTGGCTCGGAGGAG---ACCAAACCCAAAGAC
ATCAC---GTCTGA---GTCGAGCTGGATTGAG---ACGCCGTCGTCAAT
TAAGTCGATTGATTCGAGTGATTCTGGGATCTTTG---AACAGGCCAAAC
GGAGAAGAATCTCACCCTCTGCTACACCG---------GTCTCAGAATCG
GTGTCCCCGTTAAAATCTGAGNNNNNNNNNNNNCAGAGAAGTCGCAGAGAGAGAAGTGGCGC
TGGGGATAAACCCGTTCGCGGATGGGATGGGCGCCTTCAAAATAAACCAC
AGCTCCCACGACATTGGCTCCGG---ACAGACGGCGTTTTCCTCTCAGGC
T---CCCGGCTAC---GCGGCAGCCGCTTTGGGA---CACCATCA-----
-CCACCCGACCCACGTCGGCTCT---TACTCCACGGCGGCTTTCAACTCC
ACCAGGGACTTTCTCTTTAGAAATCGGGGTTTCGGAGATGCTACTGG---
---------GGCGCAGCACAGTCTGTTTGCCTC---------CGGAAGTT
T---C------GCAGGGCCACATGGACACTCAGATGCAGCGGGGCACCTG
CTCTTTCCGGGGCTTCACGAG---CAAGCGGCGAGCCACGCGTCTTCCAA
CGTGGTCAACAGCCAGATGCGACTGGGCTTCTCGGGGGACATGTACGGAC
GGGCCGACCAGTACGGCCACGTTACGAGCCCACGAT---CCGACCACTAC
GCCTCCACTCAGCTGCACGGCTATGGCCCCATGAACATGAATATGGCCGC
G---CACCACGGAGCAGGGGCCTTCTTCCGATACATGAGGCAGCCGATCA
AACAAGAGCTTATCTGCAAGTGGATCGAACCGGAGCAGCTGACGAATCCC
AAAAAGTCGTGCAACAAAACTTTCAGCACGATGCACGAGCTTGTGACCCA
TCTGACGGTGGAGCATGTGGGGGGACCCGAGCAGACCAACCACATCTGCT
TCTGGGAGGACTGCTCCAGAGAAGGGAAGCCGTTCAAAGCCAAATACAAA
CTTGTAAATCATATCAGAGTTCACACCGGAGAAAAACCCTTTCCGTGTCC
GTTCCNNNNNNNNNNNNNNN
>Paragalaxias mesotes

------------------------------------GGCTACCACGACTT
TGAGTGGCAGCCGGCCCTGAGGAACGTGTCCCCGGACTGCCACGTGGGCA
TCATCGACGGCCTCTCGGGCTGGTCGGCGTCGGTGGACGACTCACCGGCG
GACACCATCACCCGCCGCTTCCGCTACGACGTGGCCCTGGCGTCCGCCCT
GAAGGACCTGGAGGAGGACATCCTGGCGGGCCTGAGGGAGCGCGGCCTGG
AGGACGGCGCCGGCAACTCCCACTTCAGCGTCATGGTCAAGGAGTCCTGC
GACGGCATGGGCGACGTGAGCGAGAAGCACGGCGGCGGGCCGGCCGTCCC
GGAGAAGGCGGTGCGCTTCTCCTTCACCGTCATGTCCGTCTCCGTCCAGG
CGGAGGGGCGCGAG------------------------------GAGGCG
GTGACGGTGTTCAGGGAGGCCAAGCCCAAYTCGGAGCTCTCCTGCAAGCC
CCTGAGTCTGATGTTCGTGGACGAGTCGGACCACGAGACGCTCACTGCCA
TCCTGGGGCCGGTGGTGGGCGAGCGGGACGCCATGAAGCGGAGCYGCCTC
CTCCTGACCCTGGGGGGCCTCCAGCGGTCCTTCCGCTTTCACTTCCGGGG
CAGCGGCTACGACGAGAAGATGGTGCGCGAAATGGAGGGCCTGGAGGCCT
CGGGCTCCACCTACATCTGCACCCTGTGCGACTCCACCCGGGCGGAGGCC
TCGTCCAACATGGTGCTCCACTCCATCACCCGCAGCCACGAGGAGAACCT
GGAGCGCTACGAGTTGTGGAGGACCAACCCGTACGCTGAGTCGGCCAACG
AGCTGCGAGACCGGGTGAAGGGGGTCTCCGCCAAGCCCTTCCTGGAGACC
CAGCCCACCCTGGACGCGCTGCACTGCGACATCGGTAACGCCACCGAGTT
CTACAAGATGTTCCAGGACGAGATCGGGGAGGTGTTCCGGAAGCC---CA
AC---CCCAGTCGGGAGGAGAGGCGCAGCTGGCGGGCGGCCCTGGACAAA
CAACTCAGGAAGAAGCTGAAGCTGAAGCCAGTCATGCGCATGAACGGCAA
CTACGCCCGGCGCCTGATGACCCAGGAGGCGGTGGAGGTGGTGTGCGAGC
TGGTGCCCTCGGAGGAGAGGCGGCTGGCCCTCAGGGAGCTCATGRGCCTG
TACATCCAGATGAAGCCGGTGTGGCGCACCTCTTGCCCGGCCAAGGAKTG

```
CCCTGACCAGCTGTGCCGCTACAGCTTCAACTCGCAGCGCTTCGCCGAGC
TCCTCTCCACCACCTTCAAGTACAGGTATAACGGCAAGATCACCAACTAC
CTCCACAAAACGCTGGCCCATGTGCCCGAGATCATAGAGAGGGATGGGTC
CATTGGAGCGTGGGCCAGCGAGGGGAATGAGTCGGGCAACAAG-------
--------------------------------------------------
--------------------------------------------------
--------------------------------------------------
--------------------------------------------------
--------------------------------------------------
--------------------------------------------------
--------------------------------------------------
--------------------------------------------------
--------------------------------------------------
--------------------------------------------------
--------------------------------------------------
--------------------------------------------------
--------------------------------------------------
--------------------------------------------------
--------------------------------------------------
--------------------------------------------------
--------------------------------------------------
--------------------------------------------------
--------------------------------------------------
--------------------------------------------------
--------------------------------------------------
--------------------------------------------------
--------------------------------------------------
--------------------------------------------------
--------------------------------------------------
--------------------------------------------------
--------------------------------------------------
--------------------------------------------------
--------------------------------------------------
--------------------------------------------------
--------------------------------------------------
--------------------------------------------------
--------------------------------------------------
--------------------------------------------------
--------------------------------------------------
--------------------------------------------------
--------------------------------------------------
--------------------------------------------------
--------------------------------------------------
--------------------------------------------------
--------------------------------------------------
--------------------------------------------------
--------------------------------------------------
--------------------------------------------------
--------------------------------------------------
--------------------------------------------------
--------------------------------------------------
--------------------------------------------------
--------------------------------------------------
```


>Paraliparis meganchus

-------------------------GGACGAYTACGTTGTGGTGTTCAGT
CGCTCCGAGACGAGGCTGATACTGAATGAAGCCGAGCTAATCATGGCACT

```
GGCCCAGGAGTTCCAGATGAGAGTGGTCACGGTGTCCCTTGAGGAGCAGT
CTTTCCCCAGTATCGTCCAGGTGATCAGCGGTGCGTCCGTGTTGGTCAGT
ATGCATGGTGCTCAGCTCATCACCTCACTCTTCCTCCCCAGAGGAGCTGC
TGTGGTGGAGCTGTTCCCCTTTGCCGTGAACCCAGAGCAGTACACCCCGT
ATAAAACCCTTGCCACCCTTCCAGGCATGGACCTTCACTATATCTCCTGG
AGGAACACTCKGGAGGAGAACACCATCACCCACCCTGACAGAGCCTGGGA
GCAAGGGGGCATCGCTCACTTGGAGAAGGAGGAGCAAGAGCGTATACTGG
CGAGCAAAGATGTCCCCAGGCACCTGTGCTGTCGCAACCCGGAGTGGCTC
TTCCGGATCTACCAGGACACTTTGGTGGACATCCCTTCCTTCCTGGAAAM
CCTGAA---AGAGGGCATGAAG---ACRAAGCCCAGCGTGAAGAA---GT
CAAAGCCAGCCAGCACAGTCCACCCGGGCCGGGTCCGAGAACCCCAGTGT
CAGACCTCGGTACAAACCACCAACGAGGCCAAACTCACAGTCTCCTGGCA
GATCCCGTGGAATCTGAAGTACTTGAAGGTGCGAGAGGTGAAGTACGAGG
TGTGGATCCAGAAGAGAGACCCGAGCAAGGGGACGCTGGAGGACCAGATC
GTCCAGGCCAACCCGGCGCTGGAGGCTTTCGGCAACGCCAAAACGCTGAG
AAACGACAACTCGTCTCGTTTTGGAAAATTCATCCGCATTCACTTCGGTA
TGAGCGGGAAGCTGTCGTCCGCCGACATCGAGACCTACCTGCTGGAGAAG
TCCCGGTGCACCTTCCAGCTCAAGGCTGAGAGGAACTACCACATCTTCTA
CCAGATCCTGTCCAATCAGAAGCCAGAGCTGCTGGACATGCTGCTGATCA
CCAACAACCCGTACGACTACTCCTACATCTCCCAAGGGGAGGTCACCGTC
GCCTCCATCAACGACTCGGAGGAGCTGMTGGCCACCGACTGCGCCTTCGA
CGTGCTGGGCTTCACCGCAGAGGAGAAGATGGGCATCTATAAACTCACCG
GCGCCATCATGCACTTCGGCAACATGAAGTTCAAACAGAAGCAGCGCGAG
GAGCAGGCGGAGTCCGACGGCACGGAGGCGGCCGATAAGACGGCGTACCT
CATGGGCCTGAACTCCGCCGACATCATCAAGGGGCTGTGCCACCCCAGAG
TCAAGGTGGGGAATGAGTACGTCACCAAAGGCCAGAGTGTGGACCAGGTC
TACTAC--------------------------------------------
--------------------------------------------------
--------------------------------------------------
--------------------------------------------------
--------------------------------------------------
--------------------------------------------------
--------------------------------------------------
--------------------------------------------------
--------------------------------------------------
--------------------------------------------------
--------------------------------------------------
--------------------------------------------------
--------------------------------------------------
----------------------TATCTCATCTACGCCTCTTTCTCATTCA
TGGGATGTTTACAGATCAGTGATGGATCAAATATAGTGAACCTGCTGGCT
AGTAACTCCCCGAGTGTGTCTTACGCTCTGACGCAGCAGAAGTACTTCAC
CAACTACAGTCCCGTGATTGGGTTCTACATTTACGAGCCCATCGAGTACT
GGAACTCCACGGTGCAGGGGCATCTGAAGACCCTGAGTCATGGCTTCAAC
AAGATCTCTTGGATGGACAACTTTTTCCACTACCTGCGTGTGGTGAACGT
GAGCGCATCAACCAAGGGCGATTTCATCACTATCCTCAAGGGCTCCTTCC
TTCGCAGCCCGGAGTACCAGCACTTCACAGAGGACATAATATTCTCCAAG
A---ACCGCGAGACTG------ACGAGTACGACATCATCGCCTCGCGGAT
ATACCTGGTGGCACGAACGACGGAGAGAAAACGCGAGGAGGTGGTGGAGC
TTCTAGAAAAGCTTCGTCCCTTAATGCTGATCAACAGCATCAAGTTCATC
GCCTACAATCCCACGTTTGTGTTCATGGACCGCTACAGCTCCTCTGTCAT
CTCGCCCATCCTGACCTCAGGGTTCAGCGTACTCACAATCCTCATCCTCA
CTTTCTTCCTGGTCATCAACCCGTTGGGGAACTTTTGGCTCATCCTCACG
```

GTGACCTCCGTAGAGCTCGGCGTCTTGGGTTTGATGNNNNNNNNNNNNNNNNNNNNNNNNNNNNNNNN
NNNNNNNGTCTACATCTTGCA
GTGTTGGCATTATCAATGGGCTCTCTGGATGGGCTTCCTCGGTGGATGAT
TTCCCAGCTGACACCATCACTAGACGGTTCCGCTACGATGTGGCTCTGGT
GTCAGCGTTGAAGGATCTGGAGGAGGACATCTTGGAGGGGCTGAGAGAGA
GTGGGATGGAAGACAGTGCTTGCACCTCATGCTTCAGTGTCAAGATCAAG
GAATCCTGTGATGGCATGGGCGATGTCAACGAGAAGCACGGTGGAGGACC
AGCTGTTCCTGAGAAGGTTGTGCGTTTTTCTTTCACTGTTATGTCGGTCT
CTGTCCTGGCAGACGATGAGGAG---------------------------
---GAAGACGTTACCATCTTCACTGAGCCAAAGCCAAACTCAGAACTGTC
CTGTAAGCCCCTGTGCCTGATGTTTGTGGATGAGTCAGACCATGAGACAC
TCACAGCTGTCCTGGGGCCTGTGGTTGCCGAGCGTAATGCAATGAAAGAG
AGCAACCTCATCCTGTCCGTGGGCGGACTTCCTCGTTCCTTCCGCTTTCA
CTTCAGAGGCACAGGATATGATGAGAAGATGGTGCGCGAGGTCGAGGGCA
TGGAGGCCTCAGGGTCCACCTACGTCTGCACTCTTTGTGACTCCACTCGG
GCACAGGCCTCTCAAAACATGGTGCAACACTCCATCACTCGCAGTCATGA
AGAGAACCTCGATCGTTACGAAATATGGAGAACCAACCCCTTTTCTGAGT
CTGTAGATGAGCTGCGCGACAGGGTTAAAGGGATCTCTGCCAAGCCCTTC
ATGGAGACCCACCCAACGATGGATGCATTACATTGTGACATCGGCAATGC
CACTGAGTTCTACAAAATCTTCCAGGACGAGATCGGAGAGGTGTACCAAA
AGTT---CAAA---CCCAGCCGGGTGGAACGGCGCAGCTGGAGAGCAGCC
CTGGATAAACAGCTTAGGAAGAAAATGAAGCTTAGACCAGTGATGAGGAT
GAATGGGAACTATGCCCGCAGGCTTATGACCCAAGAGGCTGTGGAGGTGA
TCTGTGAGCTAGTGCCCTCAGAGGAGAGGAGGGAGGCTCTGAGGGAGCTG
ATGAGGATCTACATCCAGATGAAGCCGGTGTGGCGCGCCACCTGCCCTGC
TAAGGAGTGTCCCGACCAGCTGTGCCGCTACAACTTTAACTCCCAGCGCT
TTGCCGAACTTCTCTCCTCCACCTTCAAATACAGGTACAATGGAAAGATA
ACCAATTACCTGCACAAGACCCTGGCCCATGTGCCTGAAATCATAGAGAG
AGATGGATCCATCGGAGCCTGGGCCAGCGAAGGAAACGAGTCNNNNNNNNNNTCCTACAC
CGTGCAGATGGGACCCGCGGGGCCCCGGTGGCAGGAGAGCCCGCAGCCGT
TCTCCTGCTCCGTCGAGGACCCCACGAAACAGACCAAGTTCAAGGGCATC
AAGACCTACATCTCGTACCGGGTCACGCCGAGCCACACGGGGCGTCCCGT
CTACCGGCGCTACAAACACTTCGACTGGCTGTACAACCGCCTGCTGCACA
AGTTCACCGTGATCTCGGTGCCCCACCTGCCCGAGAAGCAGGCCACGGGG
CGCTTCGAGGAGGACTTCATCGAGAAGCGCAAGCGGCGGCTGGTCCTGTG
GATGAACCACATGACCAGCCACCCGGTCCTCTCCCAGTACGAGGGCTTCG
AGCACTTCCTGACGTGCGCCGACGACAAGCAGTGGAAGCTGGGCAAGAGG
CGGGCGGAGAAGGAGGAGATGGTGGGCGCCCACTTCATGCTGACCCTGCA
GATCCCCAACGAGCACCAGGACCTTCAGGACGTGGAGGAGCGGATCGACA
CCTTCAAGGCCTTCGCCAAGAAAATGGACGACAGCGTGATGCAGCTCRCG
CACGTCGCCTCGGAGCTGGTGCGTAAGCACCTGGGCGGCTTCCGGAAGGA
GTTCCAGCGGCTGGGGAATTCGTTCCAGTCCGTGAGCCTGGCGTTCACGC
TGGACCCTCCCCACGGCTCGGAGGCCCTCAACAAAGCCGTCTCCCAC---

----------------------------------GCCAAATCTCGCTTTC
ACCCTGGCGTGGGGACTGGTCCTGGCACGGAGC---GCAGCGTCCCACTC
GGCCACAGCTTGCTGTCCCCGCAGCAAACCGACGAGCCCAGTGTTGCCAC
CCCCCCGCAGCGATGGTTTGTCACCC---CTGCCAACAACCGACTGGACT
TTGCTGCCTCGGCATACGACGCCGCT---------GATTTCGCCGGTAAC
GCGGCCACCTTGCTGTCCTACGCAGCGGCCGGAGTGAAGGCTC------T
TCCCCTGCCGACTGCAGGCTGCTCCAACCGGGCTCTTGGCTATTACGCAG
ACCCGTCAG---GCTGG---GGAGGACGCACACCGCCGCAGTACTGCGGC
GTAAACAACAAATCCAGCTCGGTCTTTTCCTGCTGGCCCGCTAACTCTAT
CGGGGGCCGAGCAGGTA---CC---AACTACCTGG---------CCGAGG
A---AGGA---GACTC---CATCACGACAGAGAGGTCGCCG---AT---C
GGCGGCTCGGACGAG---ACCAAACCCAAAGACATGAC---ATCAGA---
GTCGAGCTGGATAGAG---ACACCGTCCTCCATTAAGTCCATTGATTCGA
GCGATTCTGGTATCTTTG---AACAGGCCAAACGGAGAAGAATCTCACCT
TCTGCCACGCCG---------GTTTCAGAGACAGTGWCCCCGTTAAAATC
TGAGNNNNNNNNNNNNNNNNNNNNNNNNNNNNNNNNNNNNNNNNNNNNNNNNNNNNNNNNNNNNNNNNNNNN
NNNNNNNNNTTCAAAATAAACCACAGCTCCCACGATATCGGCTCCGG---ACAAA
CGGCGTTTTCCTCCCAGGCG---CCTGGCTAC---GCGGCAGCCGCCCTA
GGA---CACCATCA------CCACCCGAGCCACGTTGGCTCT---TACTC
CACGGCGGCTTTCAACTCCACGAGGGACTTTCTCTTCAGAAATCGGGGGT
TCGGGGACGCCACCGG------------GGCACAGCATAGTTTGTTCGCC
TC---------CGGAAGTTT---C------GCTGGGCCACATGGACACTC
AGATGCAGCGGGGCACCTGCTCTTCCCAGGGCTCCATGAG---CAAGCGG
CGAGCCATGCGTCTTCCAACGTGGTCAACAGCCAGATGCGACTGGGCTTC
TCGGGGGACATGTACGGACGGGCCGACCAGTACGGCCACGTTACGAGCCC
GCGGT---CCGACCACTACGCTTCGACCCAGCTGCACGGCTATGGCCCCA
TGAACATGAATATGGCCGCA---CACCACGGAGCAGGGGCCTTCTTTCGA
TACATGAGGCAGCCGATCAAGCAAGAGCTCATCTGCAAGTGGATCGAGCC
GGAGCAGCTGACGAATCCTAAAAAGTCGTGCAACAAAACTTTTAGCACGA
TGCACGAGCTTGTGACCCATCTGACGGTGGAGCATGTGGGGGGACCGGAG
CAGACCAACCACATCTGCTTCTGGGAGGAATGTGCCAGAGAGGGAAAGCC
ATTCAAAGCCAANNNNNNNNNNNNNNNNNNNNNNNNNNNNNNNNNNNNNNNNNNNNNNNNNNNNNNNNNNNN
NNNNNNNNNNNNNNNNNN
>Parapercis clathrata
AGCCTTCTTATTCGAGCAGAACTAAGTCAACCAGGTGCTCTTCTAGGAGA
CGACCAAATTTATAATGTTATCGTTACAGCCCATGCCTTCGTAATAATTT
TCTTTATAGTAATGCCTATTATAATCGGGGGCTTCGGTAACTGATTAGTC
CCTCTGATAATTGGAGCTCCGGACATAGCATTCCCCCGTATAAACAATAT
AAGCTTTTGACTTCTCCCTCCTTCCCTCCTTCTTCTCTTAGCCTCTTCCG
CAGTAGAGGCCGGAGCCGGAACTGGATGAACCGTGTATCCCCCTCTAGCC
GGAAACCTAGCTCACGCAGGGGCATCAGTAGACTTAACCATTTTCTCCCT
GCATTTAGCTGGCATTTCATCTATTTTAGGGGCCATCAACTTCATCACAA
CTATTCTTAATATGAAACCTCCAGCAGTCACCCAATATCAGACCCCTCTC
TTTGTCTGAGCCGTTCTAATCACTGCTGTCCTCCTCCTCCTCTCCCTCCC

CGTCCTAGCCGCAGGAATTACAATGCTCTTAACAGATCGAAATCTAAATA
CAACTTTCTTCGATCCAGCAGGAGGAGGAGACCCCATCCTTTATCAACAC
CTA---

-------------------------GGATGAATACATAGTTGTGTTTAGT
CGTTCAACAACGAGACTGATATTGAATGAAGCAGAGCTAATCATGGCACT
GGCACAGGAGTTCCAGATGAGAGTGGTCACGGTATCCCTGGAGGACCAGT
CCTTCCCCAGTATCATCCAGGTGATCAGCAGTGCTTCTATGCTAGTCAGT
ATGCATGGAGCTCAGCTTATCACCTCACTCTTCCTCCCCAGAGGAGCCAC
TGTAGTGGAGCTGTTCCCCTTTGCTGTGAACCCAGAGCAGTACACCCCAT
ACAAAACCCTTGCATCTCTCCCAGGGATGGACCTTCACTACATCTCCTGG
AGGAACACTATGGTGGAGAACACCATCACCCACCCTGACAGACCCTGGGA
ACAAGGAGGCATCGTTCACTTGGACAAGGAGGAGCAAGAGCGAATACTGG
CGAGCCAAGATGTCCCTCGGCACCTGTGCTGCCGAAACCCAGAATGGCTC
TTCCGGATCTACCAGGACACTCTAGTGGACATCCCTTCCTTCTTGGACAC
CCTCAA---GGAGGGCATGAAG---ACAAAGCCGGGCTTTAAGAA---GT
CAAAGCTGGCTAGCACTGTCCATCCAGGTCGGGTCAGAGACCCCCAGTGT
CAGACCTCAGTACAAACCACTAATGAGGCTAAGCTCACTGTGTCATGGCA
GATCCCATGGAATCTGAAATACCTGAAGGTGAGAGAGGTGAAGNNNNNNNNNNNNNNNNNNAAAAAAG
ATGCCAGCAAAGGGACTCTGGAGGACCAGATCATCCAGGCCAACCCGGCA
CTGGAGGCCTTTGGAAACGCCAAAACGTTGAGAAATGACAACTCGTCTCG
TTTTGGAAAATTCATTCGGATTCACTTTGGTATGAGTGGAAAGTTGTCAT
CCGCCGACATTGAGACATACCTGCTGGAGAAGTCACGCGTCACCTTCCAG
CTCAAGGCAGAGAGGAACTACCACATCTTCTACCAGATCCTGTCCAATCA
GAAGCCCGAGCTGCTGGACCTGCTGCTGATCACCAATAACCCATACGACT
ACTCCTACATCTCCCAGGGCGAGGTGACTGTCGCCTCTATCAACGACTCG
GAGGAGCTGATGGCCACTGACAGCGCCTTTGATGTCCTTGGCTTCACTGC
GGAGGAGAAGACCGCTGTCTACAAGCTGACGGGCGCCATCATGCACTATG
GTAACATGAAGTTCAAACAGAAGCAGCGTGAGGAGCAGGCGGAGCCGGAT
GGCACCGAGGCTGCTGATAAGTCTGCATACCTGATGGGCCTGAACTCTGC
AGACCTCATCAAAGGCCTCTGCCACCCCAGGGTCAAGGTGGGAAATGAGT
ATGTCACCAAAGGCCAAAGCGTGGACCAGGTGTACTACCCAAACAAGGAG
GCCTTCAAGTGTGAGGAGTGTGGGAAGCACTACAACACCAAGCTGGGATA
CAAGCGCCACGTGGCCATGCACTCTGCCACTGCAGGAGATCTCACCTGTA
AAGTGTGCATGCAGACCTACGAGAGCACCCCTGTGCTCTTGGAGCATCTC

AAGAGTCACTCTGGAAAGTCTTCAGGTGGCACCAAGGAGAAAAAACACCC
GTGCGATCACTGTGACCGCCGTTTCTATACGCGAAAGGATGTGAGACGGC
ACATGGTGGTTCACACGGGCCGAAAGGACTTTCTGTGCCAGTACTGTGCC
CAGCGCTTTGGCAGGAAGGACCATCTGACACGTCATGTGAAGAAGAGCCA
CTCACAGGAGCTCCTGAAGATTAAGACAGAGCCTCCTGATATGTTAGGTC
TTTTAGCTTCGGGGTCACCGCCGTGCTCTGTGAAGGAGGAGCTCAGCCCC
ATGATGTGCAGCATGGGTCCCAACAAAGATCCCATGATGGGCAAACCCTT
CTCCAGTGGGCCTCCTTTTCCAATGGGCATGTACAACCCCCACCAT----
--CTTCAGGCCATGTCTAATTCTGGGGTGGGTCACCCG------CACCCG
TCCCTGATGCCTAGTTCCTTATCTGCAGCTATGGGCATGGGCTGTCACAT
GGAATATCTCATATACGCCTCTTTCTCGTTCATGGGATGTTTACAAATTA
GTGATGGATCGAATATCGTGAACCTGCTGGCGAGTAACTCTCCAAGCGTT
TCGTACGCTATGACCCAGCAGAAGTACTTCAGTAACTACAGTCCCGTGAT
TGGGTTTTACATTTACGAGCCCATCGAGTACTGGAACTCGACTGTGCAGG
AGCACCTGAAGACTNTGAGTCATGGGTTCAACAAGATCTCCTGGATGGACA
ACTTTTTCCACTACCTGCGGGTGGTGAATGTGAGTGCGTCAACAAAGAGC
GACTTCATCACCATTCTCAAGGGCTCCTTCCTGCGCAGCCCGGAGTACCA
GCACTTCACAGAGGACATCATATTCTCCAAGA---ACAGGGAGACCG---
---ATGAGTACGACATTATCGCCTCACGGATGTACTTGGTCGCACGAACG
ACAGAGAAGAAGCGGGAAGAGGTGGTGGAGCTTCTGGAGAAGCTTCGTCC
GCTGATGCTGATCAACAGCATCAAATTCATTGCCTTCAACCCCACGTTTG
TGTTCATGGACCGCTACAGCTCCTCCGTCATCTCGCCCATCCTGACCTCA
GGCTTCAGCGTGCTCACAATCCTCATCCTCACTTTCTTCCTGGTCATCAA
CCCCTTGGGGAACTTCTGGCTCATCCTCACAGTAACGTCCGTGGAGCTGG
GTGTNNNNNNNNNNNNNGGCTTTCACCAGTTTGAATGGCAGCCCGCTCTCAAGACTGTGTCTA
CATCTTGCAACATTGGCATTATTAATGGGCTCTCTGGATGGGCTTCGTCG
GTGGATGACGCCCCGGCTGACACCATCACTCGGCGCTTTCGCTATGATGT
GGCACTGGTGTCAGCTTTAAAGGATCTGGAGGAGGACATCATGGAGGGGC
TGAGAGAGCGTGGGCTGGAAGACAGCGCTTGCACCTCAGGCTTCAGTGTC
ATGATCAAAGAGTCCTGTGATGGCATGGGCGATGTCAGCGAGAAGCACGG
CGGAGGACCAGTTGTTCCCGAGAAGGCTGTACGTTTCTCTTTCACTGTTA
TGTCTATCTCTGTCCGGGCAGACGAGGAGGAG------------------
------------GAAGAGTTTACCATCTTCACTGAGCTGAAGCCAAACTC
GGAGCTGTCCTGCAAGCCCCTCTGCCTGATGTTCGTGGATGAGTCAGACC
ACGAGACGCTCACTGCTGTCCTGGGGCCTATAGTGGCGGAGCGTAAGGCG
ATGCAACAGAGCCGGCTCATCCTTTCCATCGGTGGACTGCCTCGCTTCTT
CCGCTTCCATTTCAGAGGAACAGGGTACGACGAGAAGATGGTGCGTGAGA
TGGAGGGCCTCGAGGCCTCGGGATCCGCCTACATCTGCACCCTTTGTGAC
TCCAGTCGCGCAGAGGCTTCTGAGAACATGGTGCTGCACTCGGTCACACG
CAGTCACGAGGAGAACTTAGAGCGTTATGAAATATGGAGAAGCAACCCCT
TCTCTGAGTCTGCGGAAGAGCTGCGAGACAGAGTCAAAGGGGTCTCTGCT
AAGCCGTTCATGGAGACCCAGCCAACGCTCGATGCATTACACTGTGACAT
TGGCAATGCCACCGAGTTCTACAAAATCTTCCAGGACGAGATTGGGGAGG
TGTACAAAAAGGT---CAAC---CCCACACGGGAGGAGCGGCGCAGCTGG
AGGGCAGCCCTAGATAAACAGCTGAGGAAGAAAATGAAGCTTAAACCAGT
AATGAGGATGAATGGGAACTTTGCCCGCAGGCTAATGACCCTGGAAACTG
TGGAGGTGGTGTGTGAGCTGGTGCCCTCAGAGGAGAGGAGGGAGGCCCTG
AGGGAGCTCATGAGGCTCTACCTCCAGATGAAGCCCGTGTGGCGAGCCAC
CTGCCCAGTCAAGGAATGCCCCGACCAGCTGTGCCGCTACAGCTTTAACT
CCCAGCACTTCGCCGACCTCCTCTCCTCTACCTTCAAATACAGGTACAAC
GGGAAGATAACCAATTACCTGCACAAGACCCTGGCCCACGTGCCTGAGAT
CATCGAGAGAGACGGGTCCATCGGAGCCTGGGCCAGCGAGGGAGACGAGT
CAGCCAACAAATCGTACACCATCGACATGGGTCCTTTGGGTCCCCTGTGG

AAGGAGAGCCCGCAGCCTTTCACCTGCTCCATCGAAGACCCAACAAAACA
GACAAAGTTCAAGGGCATCAAGACGTACATTTCCTACCGGGTCACGCCAA
GCCACACGTCGCGTCCCGTCTACAGGCGCTACAAACACTTTGACTGGCTG
TACAACCGGCTGCTGCACAAGTTCACTGTGATCTCCGTGCCTCACCTGCC
CGAGAAGCAGGCCACGGGGCGATTCGAGGAAGACTTCATCGAGAAGCGTA
AGAGACGACTGATACTGTGGATGAACCACATGACCAGTCACCCAGTCCTC
TCCCAGTACGAAGGCTTTGAGCACTTTCTGATGTGCGCTGACGACAAACA
GTGGAAACTGGGCAAGAGGCGGGCGGAGAAGGACGAGATGGTGGGTGCCC
ATTTCATGTTGACCCTCCAGATCCCCAACGAGCACCAGGACCTTCAGGAT
GTAGAGGAGCGGGTCGACGCCTTCAAGAACTTTGCTAAGAAAATGGACGA
CAGCGTGATGCAGCTCACGCATGTCGCCTCAGAGCTGGTTCGGAAACACC
TGGGCGGCTTCAGGAAGGAGTTCCAGCGGCTGGGGAACGCCTTCCAGTCC
ATCAGCCAGGCGTTCATGCTGGACCCTCCCCACAGCTCAGACACCTTCAA
CAACGCCATCTCCCAC----------------------------------

---GCCAAATCTCGCTTTCACCCTGGCGTGGGGACTGGTCCTGGCACGGA
GC---GCAGCGTCCCACTCGGCAACAGCTTGCTATCCCCGCAGCAAAGCG
AGGAGCCCACTGTTGCCACCCCCCCGCAGCGATGGTTTGTCACCC---CT
GCCAACAACCGACTGGACTTTGCTGCCTCGGCATACGACGCCGCT-----
----GATTTCGCCGGTAACGCGGCCACCTTGCTGTCCTACGCGGCGGCCG
GAGTGAAGGCTC------TCCCCCTGCCGACTGCGGCTGCTCCAACCGG
CCTCTTGGCTATTACGCAGACCCGTCGG---GCTGG---GGAGGACGCAC
GCCGCCGCAGTACTGTGGCGTTAACAGCAAGCCCAGCTCGGTCTTTTCCT
GCTGGCCCGCGAACTCTATTGGCGGAAGGGCCGGAG---CC---AACTAC
CTGG---------CCGAGGA---AGGG---GACTC---AATCCCGCCAGA
GAGGTCACCG---AT---CGGGGGCTCGGAGGAG---GCCAAACCCAAGG
ACATGAC---GTCGGA---GTCGAGCTGGATAGAG---ACGCCGTCCTCC
ATCAAGTCCATCGACTCGAGCGACTCTGGGATCTTTG---AGCAGGCCAA
AAGGAGGAGGATCTCACCTACGGCCACGCCG---------GTCTCAGAGA
CAGTGTCCCCGTTAAAATNNNNNCATCACTCAACAGGCGAAGTCACAGAGAGAGA
AGTGGCGTTGGGGATAAATCCGTTCGCGGATGGGATGGGCGCCTTCAAAA
TAAACCACAGCTCCCACGATATTGGTTCCGG---ACAGACGGCGTTTTCC
TCCCAGGCG---CCCGGCTAT---GCAGCAGCCGCCCTGGGA---CACCA
CCA------CCACCCGACTCACGTTGGTTCT---TACTCCACGGCGGCTT
TCAACTCCACCAGGGACTTTCTCTTCAGAAATCGGGGATTCGGGGACGCC

```
ACCGG------------GGCGCAGCACAGTTTGTTCGCCTC---------
CGGAAGTTT---C------GCAGGGCCACATGGACACTCAGATGCGGCGG
GGCACCTGCTCTTCCCGGGGCTCCACGAG---CAAGCGGCGAGCCATGCG
TCCTCCAACGTGGTCAACAGCCAGATGCGGCTGGGCTTCTCGGGGGACAT
GTACGGACGGGCCGACCAGTACGGCCACGTTACGAGCCCGCGCT---CCG
ACCACTACGCCTCGACCCAGCTCCACGGCTACGGCCCCATGAACATGAAT
ATGGCCGCG---CACCACGGAGCAGGGGCCTTCTTTCGATACATGAGGCA
GCCGATCAAACAAGAGCTCATCTGCAAGTGGATCGAGCCGGAGCAGCTGA
CGAATCCCAAAAAGTCGTGCAACAAAACTTTTAGCACGATGCACGAGCTC
GTGACCCATCTGACGGTGGAGCATGTGGGGGACCGGAGCAGACCAACCA
CATCTGCTTCTGGGAGGAATGCGCCCGAGAAGGGAAGCCGTTCAAAGCCA
AATACAAACTTGTAAATCATATCAGAGTACACACCGGAGAAAAGCCCTTC
CCCTGTCCGTTCCCCGGCTGTGGCAAA
>Parapriacanthus ransonneti
AGCCTGCTGATTCGTGCAGAATTAAGTCAGCCAGGTTCCCTCTTAGGTGA
CGACCAGATTTATAATGTAATCGTCACAGCGCACGCATTCGTGATAATTT
TCTTTATAGTAATACCAATCATGATCGGTGGTTTCGGAAATTGACTCATT
CCTCTGATGATCGGAGCCCCTGACATAGCCTTCCCTCGAATGAATAACAT
AAGCTTTTGACTTCTTCCCCCTTCCTTCCTTCTTCTCTTAGCCTCATCAG
GTGTTGAAGCCGGAGCAGGTACTGGGTGAACAGTCTATCCCCCATTAGCA
GGTAACCTCGCACATGCGGGAGCTTCTGTAGACTTAACTATTTTCTCACT
CCACCTAGCTGGTGTTTCATCTATTCTTGGTGCAATTAACTTCATCACCA
CAATTATTAATATGAAACCTCCTGCTATGACTCAGTACCAACTCCCCTTA
TTTGTGTGAGCTGTTCTTATCACTGCTGTACTCCTGCTCCTTTCACTCCC
AGTCCTAGCTGCTGGCATTACAATACTCCTAACAGACCGAAACCTAAACA
```
[truncated: 519,463 more chars]
